# Supplementary material for: Passive acoustic monitoring of baleen whale seasonal presence across the New York Bight
Source: PLoS One. 2025 Feb 13;20(2):e0314857. doi: 10.1371/journal.pone.0314857 (PMC11825016; doi:10.1371/journal.pone.0314857)
Supplement: S1 Table — (PDF) [file pone.0314857.s001.pdf]

Supporting Information

1. Deployment Information

Table S1. Sensor deployment information for each site and survey year.

Survey Year	Deployment Name	Site	Latitude	Longitude	Depth (m)	Analysis Start Date	Analysis End Date	Total Analysis Days
**1**	M-D1	1M	40.347982	-71.224167	90	16-Oct-17	18-Mar-18	154
**1**	M-D2	1M	40.348321	-71.225644	90	12-Apr-18	14-Jul-18	94
**1**	M-D3	1M	40.34884	-71.22556	88	20-Jul-18	15-Oct-18	88
**2**	M-D3	1M	40.34884	71.22556	88	16-Oct-18	23-Dec-18	69
**2**	M-D4	1M	40.34938	71.22603	88	13-Jan-19	10-Apr-19	88
**2**	M-D5	1M	40.34756	71.22794	88.9	12-Apr-19	3-Aug-19	114
**2**	M-D6	1M	40.34755	71.22902	86.5	5-Aug-19	15-Oct-19	72
**3**	M-D6	1M	40.347553	-71.22902	86.5	16-Oct-19	24-Oct-19	9
**3**	M-D7	1M	40.347891	-71.22981	85.5	26-Oct-19	22-Jan-20	89
**3**	M-D8	1M	40.347786	-71.230618	87.8	24-Jan-20	27-Jun-20	156
**3**	M-D9	1M	40.347852	-71.231932	88.7	10-Jul-20	15-Oct-20	98
**1**	M-D1	2M	40.34219	-71.606067	84	16-Oct-17	17-Mar-18	153
**1**	M-D2	2M	40.342242	-71.607933	84	No Data	No Data	No Data
**1**	M-D3	2M	40.34263	-71.60848	84	No Data	No Data	No Data
**2**	M-D4	2M	40.34231	71.6076	83	14-Jan-19	10-Apr-19	87
**2**	M-D5	2M	40.34307	71.60787	84.6	12-Apr-19	3-Aug-19	114
**2**	M-D6	2M	40.34304	71.6085	82.6	5-Aug-19	15-Oct-19	72
**3**	M-D6	2M	40.343041	-71.608501	82.6	16-Oct-19	24-Oct-19	9
**3**	M-D7	2M	40.342957	-71.609415	81.3	26-Oct-19	22-Jan-20	89
**3**	M-D8	2M	40.342794	-71.610601	83.3	24-Jan-20	25-Jun-20	154
**3**	M-D9	2M	40.342827	-71.610971	82.5	9-Jul-20	9-Jul-20	1
**1**	M-D1	3M	40.333845	-71.999408	65	16-Oct-17	18-Mar-18	154
**1**	M-D2	3M	40.333843	-72.000828	65	No Data	No Data	No Data
**1**	M-D3	3M	40.33393	-72.0013	64	20-Jul-18	15-Oct-18	88
**2**	M-D3	3M	40.33393	72.0013	64	16-Oct-18	22-Dec-18	68
**2**	M-D4	3M	40.33381	72.00063	65	14-Jan-19	10-Apr-19	87
**2**	M-D5	3M	40.33433	72.00121	65.3	12-Apr-19	3-Aug-19	114
**2**	M-D6	3M	40.33471	72.00192	65.3	5-Aug-19	15-Oct-19	72
**3**	M-D6	3M	40.334706	-72.001923	65.3	16-Oct-19	23-Oct-19	8
**3**	M-D7	3M	40.334684	-72.002578	63.8	No Data	No Data	No Data
**3**	M-D8	3M	40.334158	-72.000763	64.8	23-Jan-20	28-Jun-20	158
**3**	M-D9	3M	40.334159	-72.002521	64.1	9-Jul-20	15-Oct-20	99

Survey Year	Deployment Name	Site	Latitude	Longitude	Depth (m)	Analysis Start Date	Analysis End Date	Total Analysis Days
**1**	M-D1	4M	40.327372	-72.408	54	16-Oct-17	17-Dec-17	63
**1**	M-D2	4M	40.327631	-72.408339	54	No Data	No Data	No Data
**1**	M-D3	4M	40.32781	-72.40787	53	20-Jul-18	15-Sep-18	58
**2**	M-D3	4M	40.32781	72.40787	53	No Data	No Data	No Data
**2**	M-D4	4M	40.32791	72.40718	54	14-Jan-19	10-Apr-19	87
**2**	M-D5	4M	40.32778	72.40862	54.1	11-Apr-19	3-Aug-19	115
**2**	M-D6	4M	40.32823	72.40906	54.1	5-Aug-19	15-Oct-19	72
**3**	M-D6	4M	40.32823	-72.409055	54.1	16-Oct-19	23-Oct-19	8
**3**	M-D7	4M	40.328341	-72.409856	53.3	25-Oct-19	21-Jan-20	89
**3**	M-D8	4M	40.328529	-72.410882	53.7	23-Jan-20	13-May-20	112
**3**	M-D9	4M	40.328664	-72.411243	52.9	9-Jul-20	5-Oct-20	89
**1**	M-D1	5M	40.319978	-72.781935	50	16-Oct-17	23-Feb-18	131
**1**	M-D2	5M	40.319907	-72.782415	50	12-Apr-18	14-Apr-18	3
**1**	M-D3	5M	40.31991	-72.7825	50	No Data	No Data	No Data
**2**	M-D4	5M	40.31928	72.78249	50.4	14-Jan-19	10-Apr-19	87
**2**	M-D5	5M	40.31922	72.78351	50.7	11-Apr-19	2-Aug-19	114
**2**	M-D6	5M	40.31896	72.7844	50.5	4-Aug-19	15-Oct-19	73
**3**	M-D6	5M	40.318961	-72.784396	50.5	16-Oct-19	23-Oct-19	8
**3**	M-D7	5M	40.319107	-72.784496	50.2	25-Oct-19	21-Jan-20	89
**3**	M-D8	5M	40.319013	-72.785293	50.4	23-Jan-20	20-Jun-20	150
**3**	M-D9	5M	40.318051	-72.786949	49.5	No Data	No Data	No Data
**1**	M-D1	6M	40.284352	-73.152112	40	16-Oct-17	18-Mar-18	154
**1**	M-D2	6M	40.284681	-73.153109	40	12-Apr-18	14-Jul-18	94
**1**	M-D3	6M	40.28409	-73.1523	40	20-Jul-18	30-Sep-18	73
**1**	M-D3	6M	40.28409	73.1523	40	No Data	No Data	No Data
**2**	M-D4	6M	40.28386	73.15194	40	14-Jan-19	9-Apr-19	86
**2**	M-D5	6M	40.284	73.15348	39.8	No Data	No Data	No Data
**2**	M-D6	6M	40.28438	73.15464	39	4-Aug-19	15-Oct-19	73
**3**	M-D6	6M	40.284382	-73.15464	39	16-Oct-19	23-Oct-19	8
**3**	M-D7	6M	40.284842	-73.155409	39.5	25-Oct-19	21-Jan-20	89
**3**	M-D8	6M	40.285197	-73.156822	39.7	23-Jan-20	20-Jun-20	150
**3**	M-D9	6M	40.285505	-73.158954	38.9	No Data	No Data	No Data
**1**	M-D1	7M	40.347333	-73.484445	30	16-Oct-17	18-Mar-18	154
**1**	M-D2	7M	40.34779	-73.486217	30	12-Apr-18	26-Jun-18	76
**1**	M-D3	7M	40.34806	-73.48566	30	20-Jul-18	15-Oct-18	88
**2**	M-D3	7M	40.34806	73.48566	30	16-Oct-18	23-Dec-18	69
**2**	M-D4	7M	40.34806	73.48566	36	14-Jan-19	9-Apr-19	86
**2**	M-D5	7M	40.34836	73.48706	30.2	11-Apr-19	2-Aug-19	114

Survey Year	Deployment Name	Site	Latitude	Longitude	Depth (m)	Analysis Start Date	Analysis End Date	Total Analysis Days
**2**	M-D6	7M	40.34891	73.48807	27.6	4-Aug-19	15-Oct-19	73
**3**	M-D6	7M	40.348906	-73.488074	27.6	16-Oct-19	23-Oct-19	8
**3**	M-D7	7M	40.349921	-73.488591	27.6	25-Oct-19	21-Jan-20	89
**3**	M-D8	7M	40.350122	-73.488815	28.2	No Data	No Data	No Data
**3**	M-D9	7M	40.35043	-73.489172	28.2	No Data	No Data	No Data
**1**	A-D1	8A	40.41723	-73.766032	28	16-Oct-17	14-Jul-18	272
**1**	A-D2	8A	40.41739	-73.76308	28	No Data	No Data	No Data
**2**	A-D2	8A	40.41739	73.76308	28	No Data	No Data	No Data
**2**	A-D3	8A	40.41367	73.76214	32	14-Mar-19	15-Oct-19	216
**2**	A-D3	8A	40.41367	-73.76214	32	16-Oct-19	19-Oct-19	4
**3**	A-D4	8A	40.414579	-73.758602	30	No Data	No Data	No Data
**3**	A-D5	8A	40.41515	-73.75763	26	No Data	No Data	No Data
**1**	A-D1	9A	40.20305	-73.628742	38	16-Oct-17	14-Jul-18	272
**1**	A-D2	9A	40.20318	-73.62847	38	16-Jul-18	15-Oct-18	92
**2**	A-D2	9A	40.20318	73.62847	38	16-Oct-18	13-Mar-19	149
**2**	A-D3	9A	40.19968	73.62833	37	14-Mar-19	15-Oct-19	216
**3**	A-D3	9A	40.19968	-73.62833	37	16-Oct-19	19-Oct-19	4
**3**	A-D4	9A	40.20154	-73.629489	36	No Data	No Data	No Data
**3**	A-D5	9A	40.203054	-73.630477	36.5	No Data	No Data	No Data
**1**	M-D1	10M	40.032333	-73.499988	49	16-Oct-17	23-Feb-18	131
**1**	M-D2	10M	40.031789	-73.500126	49	12-Apr-18	14-Jul-18	94
**1**	M-D3	10M	40.03231	-73.49923	49	No Data	No Data	No Data
**2**	M-D4	10M	40.03189	73.49901	48	19-Jan-19	24-Apr-19	96
**2**	M-D5	10M	40.03275	73.5	48	26-Apr-19	1-Aug-19	98
**2**	M-D6	10M	40.03372	73.49926	47.4	3-Aug-19	15-Oct-19	74
**3**	M-D6	10M	40.03372	-73.49926	47.4	16-Oct-19	19-Oct-19	4
**3**	M-D7	10M	40.034733	-73.499356	46	21-Oct-19	20-Jan-20	92
**3**	M-D8	10M	40.035185	-73.49968	47.7	22-Jan-20	19-Jun-20	150
**3**	M-D9	10M	40.035483	-73.499426	46.7	21-Jun-20	15-Oct-20	117
**1**	A-D1	11A	39.88428	-73.293273	49	16-Oct-17	14-Jul-18	272
**1**	A-D2	11A	39.88345	-73.29187	49	16-Jul-18	15-Oct-18	92
**2**	A-D2	11A	39.88345	-73.29187	49	16-Oct-18	13-Mar-19	149
**2**	A-D3	11A	39.88142	-73.28865	49	15-Mar-19	20-Jan-20	302
**3**	A-D4	11A	39.888638	-73.292945	50	No Data	No Data	No Data
**3**	A-D5	11A	39.88974	-73.294121	54	No Data	No Data	No Data
**1**	M-D1	12M	39.734522	-73.106817	51	16-Oct-17	12-Mar-18	148
**1**	M-D2	12M	39.73377	-73.105267	51	12-Apr-18	14-Jul-18	94
**1**	M-D3	12M	39.73353	-73.10452	51	16-Jul-18	7-Aug-18	23

Survey Year	Deployment Name	Site	Latitude	Longitude	Depth (m)	Analysis Start Date	Analysis End Date	Total Analysis Days
**1**	M-D3	12M	39.73353	73.10452	51	No Data	No Data	No Data
**2**	M-D4	12M	39.73312	73.10454	50	19-Jan-19	24-Apr-19	96
**2**	M-D5	12M	39.73422	73.10571	51	26-Apr-19	1-Aug-19	98
**2**	M-D6	12M	39.73487	73.10667	48.9	3-Aug-19	15-Oct-19	74
**3**	M-D6	12M	39.73487	-73.10667	48.9	16-Oct-19	19-Oct-19	4
**3**	M-D7	12M	39.735634	-73.106751	50	21-Oct-19	21-Jan-20	93
**3**	M-D8	12M	39.7361	-73.106361	50.1	23-Jan-20	19-Jun-20	149
**3**	M-D9	12M	39.73635	-73.136457	49.5	21-Jun-20	15-Oct-20	117
**1**	A-D1	13A	39.591833	-72.923667	63	16-Oct-17	14-Jul-18	272
**1**	A-D2	13A	39.59182	-72.92208	63	16-Jul-18	15-Oct-18	92
**2**	A-D2	13A	39.59182	72.92208	63	16-Oct-18	13-Mar-19	149
**2**	A-D3	13A	39.58882	72.92199	63	15-Mar-19	15-Oct-19	215
**3**	A-D3	13A	39.58882	-72.921994	63	16-Oct-19	19-Oct-19	4
**3**	A-D4	13A	39.590675	-72.923145	61	No Data	No Data	No Data
**3**	A-D5	13A	39.590111	-72.922941	62.4	No Data	No Data	No Data
**1**	M-D1	14M	39.438865	-72.72855	80	No Data	No Data	No Data
**1**	M-D2	14M	39.438872	-72.729047	80	12-Apr-18	14-Jul-18	94
**1**	M-D3	14M	39.43877	-72.72832	80	20-Jul-18	15-Oct-18	88
**2**	M-D3	14M	39.43877	72.72832	80	16-Oct-18	19-Dec-18	65
**2**	M-D4	14M	39.43941	72.72879	81	18-Jan-19	24-Apr-19	97
**2**	M-D5	14M	39.44002	72.72978	81	26-Apr-19	1-Aug-19	98
**2**	M-D6	14M	39.44064	72.73072	79.6	No Data	No Data	No Data
**3**	M-D6	14M	39.44064	-72.73072	79.6	No Data	No Data	No Data
**3**	M-D7	14M	39.441003	-72.730863	79	21-Oct-19	21-Jan-20	93
**3**	M-D8	14M	39.440391	-72.730293	80.1	23-Jan-20	19-Jun-20	149
**3**	M-D9	14M	39.441048	-72.730799	79.7	21-Jun-20	15-Oct-20	117

2. Detector Performance

2.1. Sei Whale

Using the full 20th-day groundtruthed dataset, the sei whale detector had a TPR of 0.33 on a detector-to-signal basis (558 TP and 1,122 FN), and a TPR of 0.33 on a daily scale (7 days with at least one TP and 12 days with FN). The low TPR may be caused by several factors: 1) it is possible that signals with a low signal-to-noise ratio (SNR) were not detected as well as signals with a higher SNR, and 2) we observed many distorted sei whale downsweeps (Figure S1) which could be due to signal propagation and reflection of the signal off the seafloor and water surface [1-3], for which the detector was not prepared to detect.

To test the influence of SNR on an event being detected (TP) or missed (FN) by the detector, a random set of 100 TP and 100 FN events were selected from the groundtruthed dataset. Event boxes were adjusted around the signal to exclude non-target signals from the SNR measurement and to ensure that at least 25% of the time-frequency bins in the event box contained background noise. We calculated the signal-to-noise ratio in which *noise* is the 25th percentile time-frequency bin within the event box, and the *signal* is the 100th percentile bin [4]. Event boxes that could not be resized to exclude noise from signal were removed from this analysis, leaving 191 usable sei whale downsweeps. A one-way ANOVA showed a statistically significant difference ($F_{1,189}$ = 6.48, $p = .0117$) in SNR between TP (n = 24, mean SNR = 9.28 dB ± 0.37 SE) and FN (n = 167, mean SNR dB = 8.27 ± 0.14 SE), confirming that lower SNR events were more likely to be missed by the detector.

To investigate the influence that the distorted sei whale downsweep signal may have on the detector, we marked all groundtruthed sei whale events as a) signals that were a single band (n = 433) or b) a signal in which the downsweep appears to be split into multiple bands (n = 666). Events for which distortion of the signal was difficult to determine were excluded from this analysis. When distorted downsweeps were excluded, the detector yielded a TPR of 0.51, suggesting that the distorted sei whale signals negatively influenced the detector performance. A Fisher's exact test showed that the probability of a TP detection is significantly higher for signals that were not distorted than for signals that are ($p < .0001$).

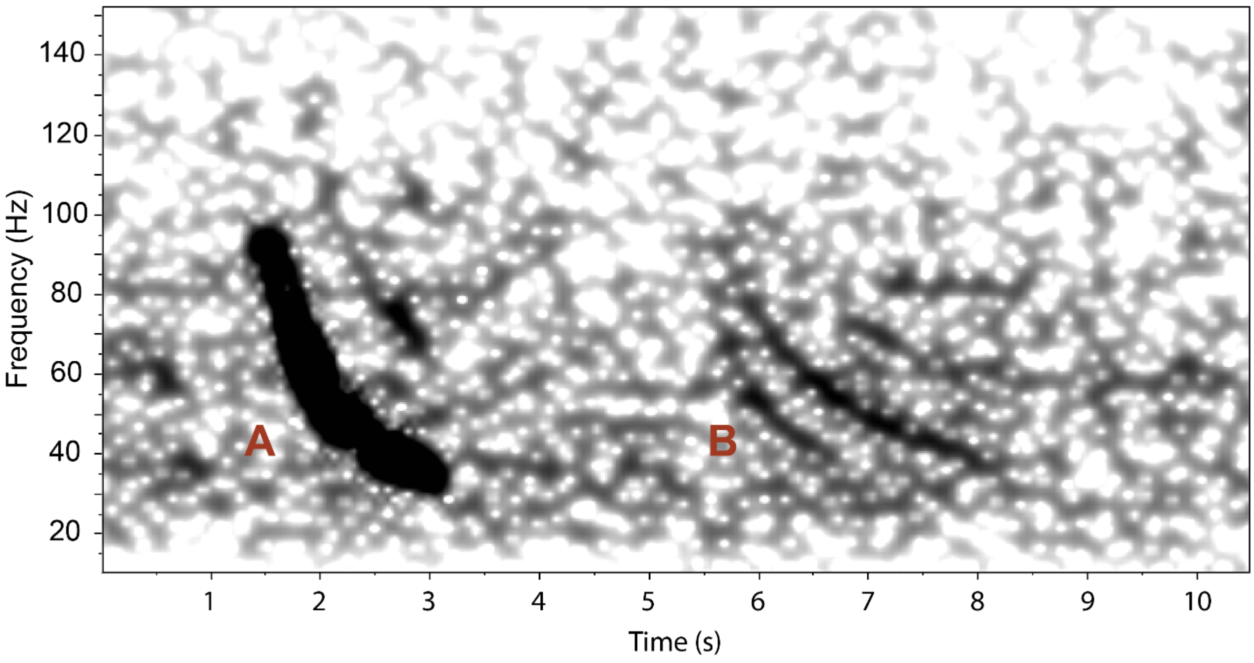

S1 Fig. Spectrogram of sei whale downsweeps exhibiting frequency dispersion: A) a sei whale downsweep similar to the templates used for the template detector, B) a multi-path (distorted) sei whale downsweep, which was often not detected by the template detector. Spectrogram was created with a window size = 2048, DFT = 4096, with frequency and time bins of 1.22 Hz and 0.0614 s, respectively.

In NY Bight, we observed many instances of mode dispersion in sei whale downsweeps (60% of the groundtruthed sei whale events), which negatively affected the performance of the template detector. As such, it is possible that sei whale daily presence is underestimated in these data. Because mode dispersion is a function of signal propagation in shallow water [1], it is possible that signals produced by sei whales further from the recording site were more likely to experience mode dispersion by the time they were received by the recording unit [1, 5]. Therefore, sei whales that vocalized further from the recording unit were more often missed by the detector, which used non-dispersed sei whale downseeps as templates. In the future, the frequent occurrence of mode dispersion in sei whale downsweeps in New York Bight could present an opportunity for sei whale detection range estimation work [1, 5] and acoustics-based density estimation sampling [6]. The frequent observations of mode dispersed downsweeps is also an important consideration when implementing automated acoustic detection and classification for sei whales in the NY Bight [5]. Signal-to-noise ratio also affected the recall of the detection, where faint downsweeps were detected less. A single sei whale downsweep could occasionally be detected across multiple sensors, where the first arrival typically had a higher SNR than subsequent arrivals of the signal at nearby sites. When this occurs, it is possible that the detector would find the higher SNR signal and miss the subsequent, fainter arrivals of the signal at other sites. However, in such a situation, sei whales would be marked as present during that day. Thus, it is possible that fewer days were falsely missed by the detector than what the detector performance metrics suggest.

3. Species Presence

Table S2. Daily presence for each focal whale species by month across all sites, and the corresponding percentage of days in which each species was detected.

Month-Year	Total Days Recorded	Right		Humpback		Fin		Sei		Blue	
		Days Detected	% Days Detected	Days Detected	% Days Detected	Days Detected	% Days Detected	Days Detected	% Days Detected	Days Detected	% Days Detected
**17-Oct**	16	2	13	3	19	16	100	8	50	0	0
**17-Nov**	30	25	83	11	37	30	100	9	30	3	10
**17-Dec**	31	31	100	28	90	31	100	7	23	7	23
**18-Jan**	31	24	77	26	84	31	100	10	32	8	26
**18-Feb**	28	11	39	28	100	28	100	5	18	8	29
**18-Mar**	31	22	71	25	81	31	100	24	77	0	0
**18-Apr**	30	19	63	25	83	30	100	30	100	0	0
**18-May**	31	14	45	29	94	29	94	27	87	0	0
**18-Jun**	30	7	23	29	97	30	100	7	23	0	0
**18-Jul**	30	3	10	28	93	30	100	6	20	0	0
**18-Aug**	31	4	13	31	100	31	100	2	6	0	0
**18-Sep**	30	3	10	29	97	30	100	0	0	0	0
**18-Oct**	31	4	13	29	94	31	100	3	10	0	0
**18-Nov**	30	19	63	27	90	30	100	5	17	0	0
**18-Dec**	31	28	90	24	77	31	100	1	3	0	0
**19-Jan**	31	24	77	26	84	31	100	0	0	6	19
**19-Feb**	28	16	57	22	79	28	100	1	4	1	4
**19-Mar**	31	24	77	30	97	31	100	27	87	0	0
**19-Apr**	30	28	93	27	90	30	100	30	100	0	0
**19-May**	31	25	81	31	100	31	100	31	100	0	0

Table S2 (continued). Daily presence for each focal whale species by month across all sites, and the corresponding percentage of days in which each species was detected.

Month-Year	Total Days Recorded	Right		Humpback		Fin		Sei		Blue	
		# Days Detected	% Days Detected	# Days Detected	% Days Detected	# Days Detected	% Days Detected	# Days Detected	% Days Detected	# Days Detected	% Days Detected
**19-Jun**	30	23	77	30	100	30	100	10	33	0	0
**19-Jul**	31	2	6	31	100	31	100	0	0	0	0
**19-Aug**	31	7	23	25	81	31	100	14	45	0	0
**19-Sep**	30	2	7	29	97	30	100	4	13	0	0
**19-Oct**	31	3	10	23	74	31	100	0	0	0	0
**19-Nov**	30	12	40	26	87	30	100	3	10	0	0
**19-Dec**	31	19	61	31	100	31	100	2	6	0	0
**20-Jan**	31	14	45	18	58	28	90	4	13	6	19
**20-Feb**	29	0	0	15	52	29	100	25	86	11	38
**20-Mar**	31	0	0	10	32	31	100	26	84	0	0
**20-Apr**	30	16	53	5	17	30	100	26	87	0	0
**20-May**	31	7	23	21	68	31	100	30	97	0	0
**20-Jun**	30	1	3	5	17	28	93	16	53	0	0
**20-Jul**	31	9	29	14	45	31	100	0	0	1	3
**20-Aug**	31	5	16	31	100	31	100	0	0	0	0
**20-Sep**	30	5	17	28	93	30	100	0	0	0	0
**20-Oct**	15	0	0	15	100	15	100	0	0	0	0

Table S3. Summary of daily detections for each baleen whale species Total Days refers to the number of days that were sampled during the 3-year survey.

Site	Total Days	Right		Humpback		Fin		Sei		Blue	
		Days presence	% Days	Days presence	% Days	Days presence	% Days	Days presence	% Days	Days presence	% Days
**1M**	1031	85	8	244	24	833	81	193	19	42	4
**2M**	679	77	11	140	21	599	88	176	26	36	5
**3M**	848	52	6	297	35	773	91	134	16	20	2
**4M**	693	44	6	277	40	610	88	107	15	10	1
**5M**	655	56	9	172	26	403	62	34	5	0	0
**6M**	727	69	9	251	35	404	56	33	5	0	0
**7M**	757	56	7	197	26	266	35	4	1	0	0
**8A**	492	15	3	43	9	125	25	5	1	0	0
**9A**	733	99	14	188	26	344	47	35	5	0	0
**10M**	856	76	9	213	24	585	68	27	3	0	0
**11A**	814	121	15	300	37	726	89	49	6	0	0
**12M**	896	76	8	280	31	715	80	93	10	5	1
**13A**	732	81	11	386	53	689	94	113	15	1	0
**14M**	801	57	7	194	24	588	73	182	23	17	2
**Total**	10714	964	9	3182	30	7660	71	1185	11	131	1

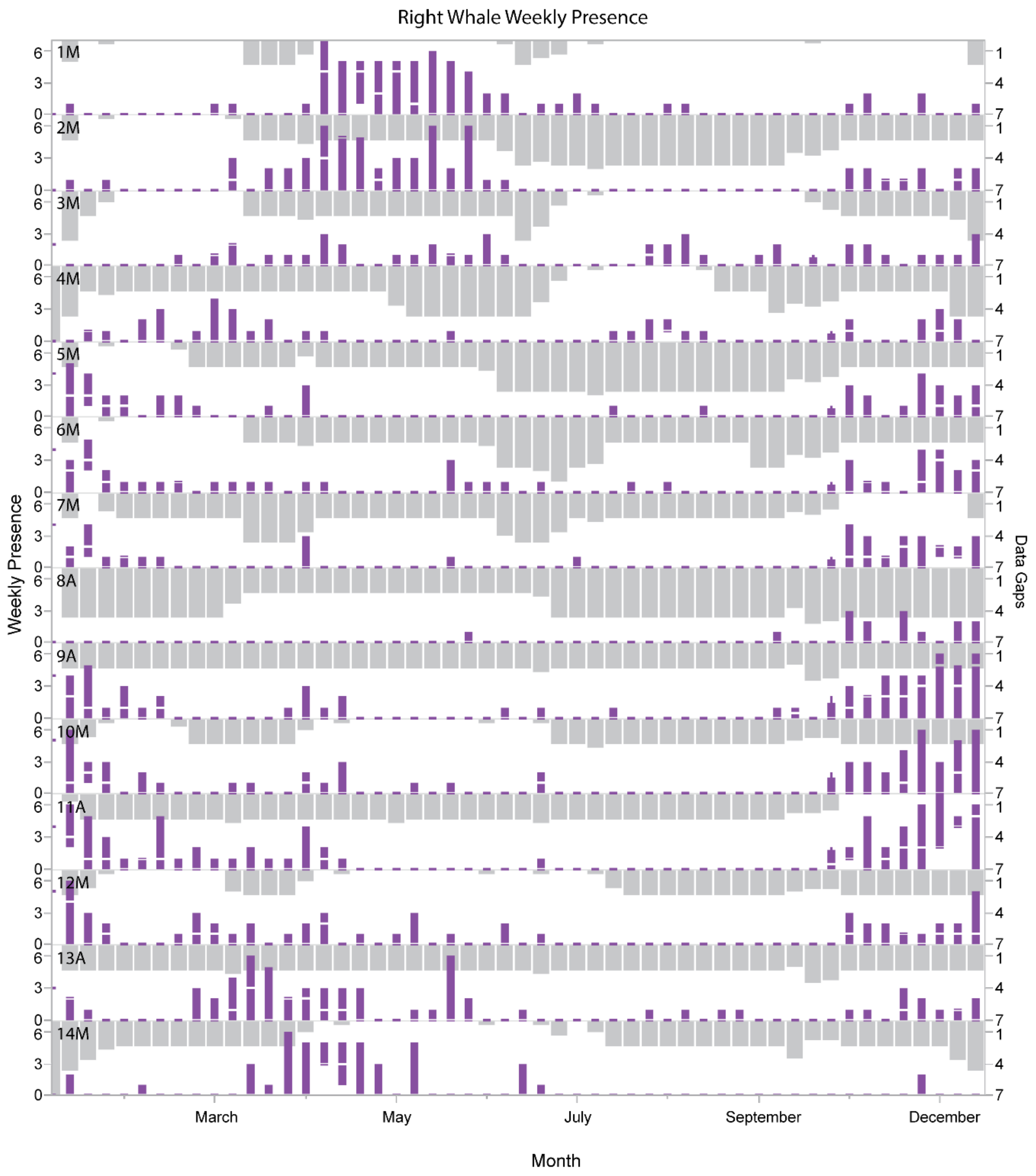

S2 Fig. Quantile boxplots of weekly acoustic presence of North Atlantic right whales per site in New York Bight between October 2017 and October 2020, shown as proportion of recorded days per week with confirmed right whale upcall detections across all sensors (purple). Grey bars indicate the mean number of days per week without data, along the inverted secondary x-axis.

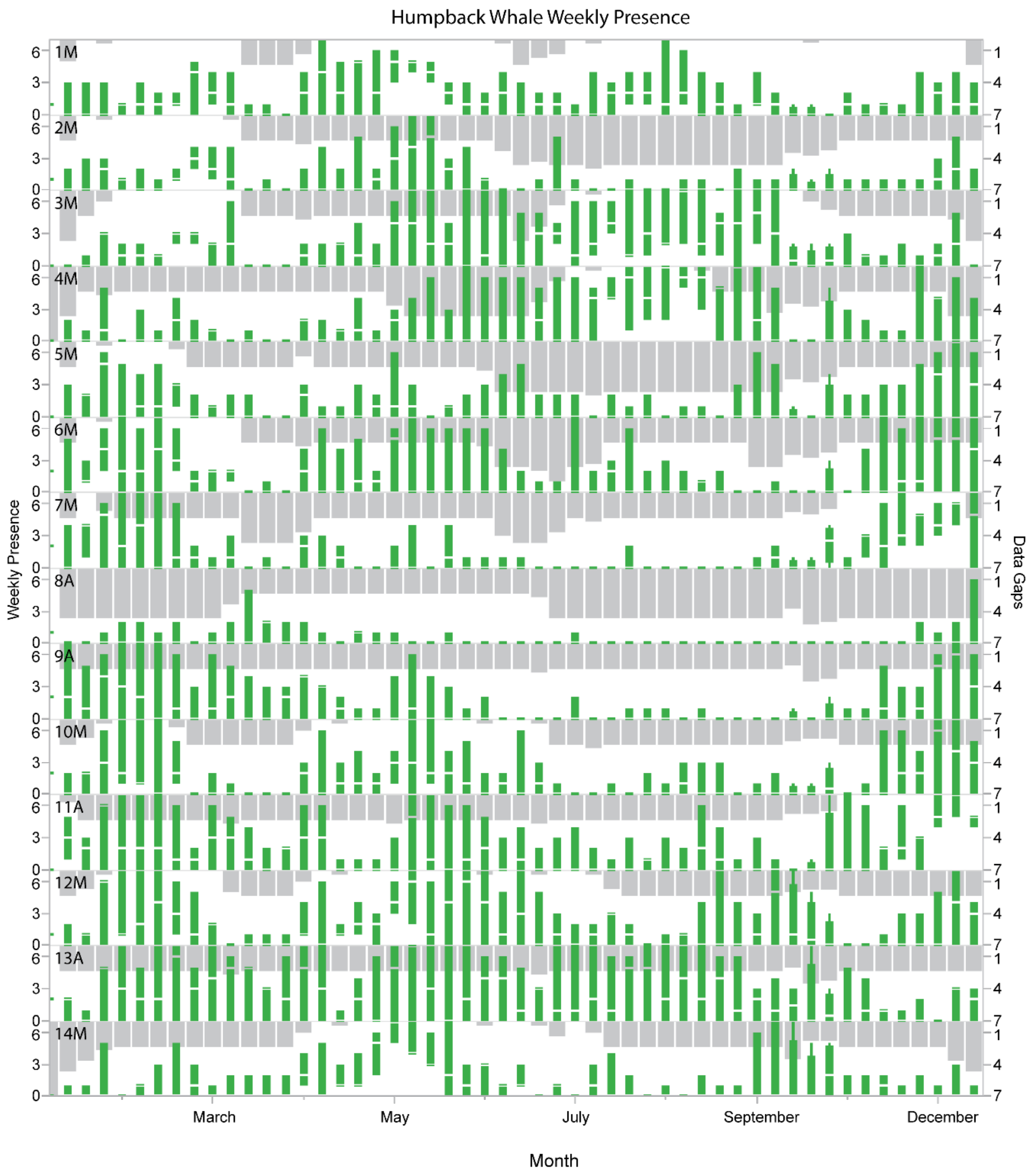

S3 Fig. Quantile boxplots of weekly acoustic presence of humpback whales per site in New York Bight between October 2017 and October 2020, shown as proportion of recorded days per week with confirmed humpback whale song and non-song detections across all sensors (green). Grey bars indicate the mean number of days per week without data, along the inverted secondary x-axis.

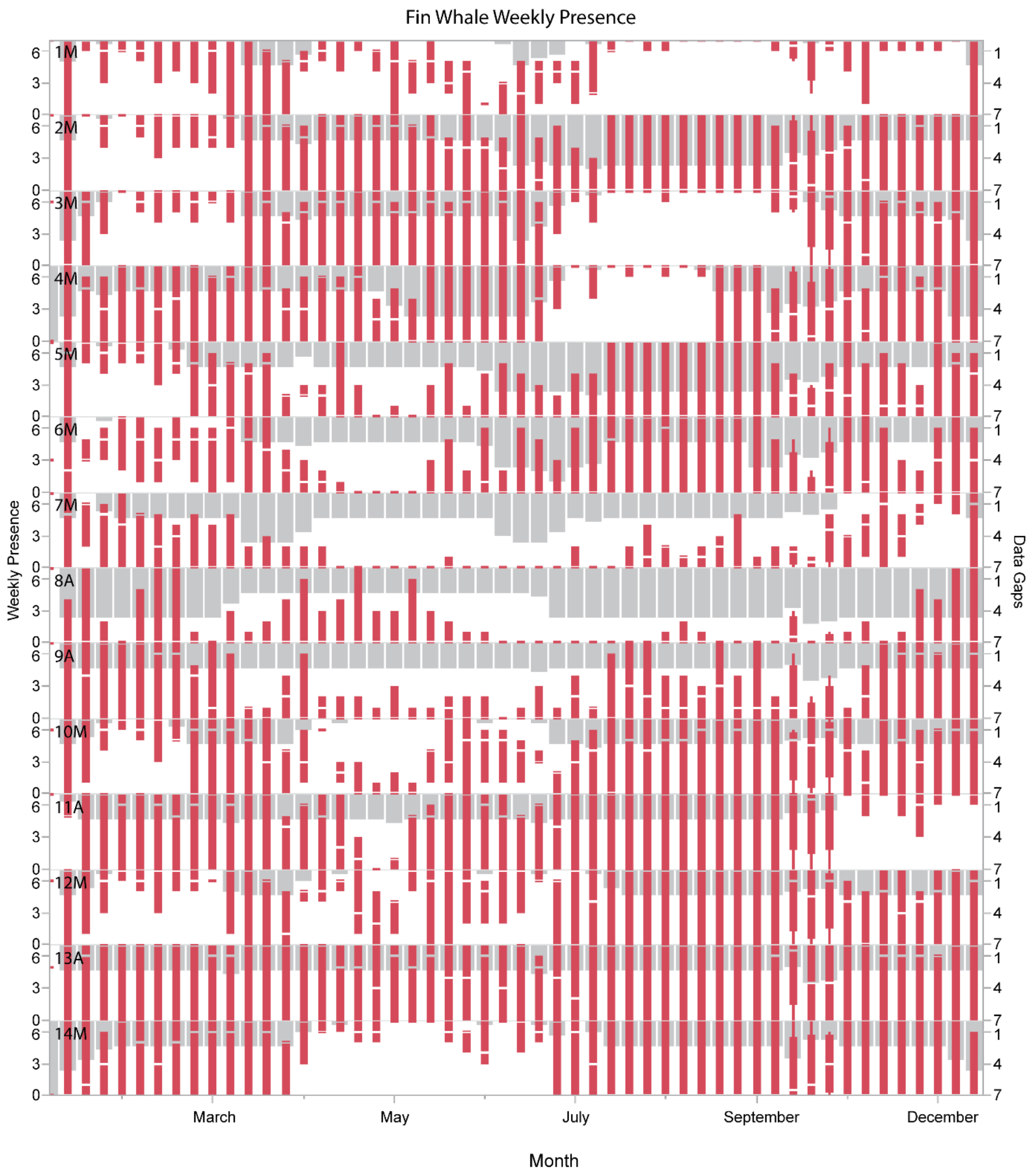

S4 Fig. Quantile boxplots of weekly acoustic presence of fin whales per site in New York Bight between October 2017 and October 2020, shown as proportion of recorded days per week with confirmed fin whale 20-Hz pulse detections across all sensors (red). Grey bars indicate the mean number of days per week without data, along the inverted secondary x-axis.

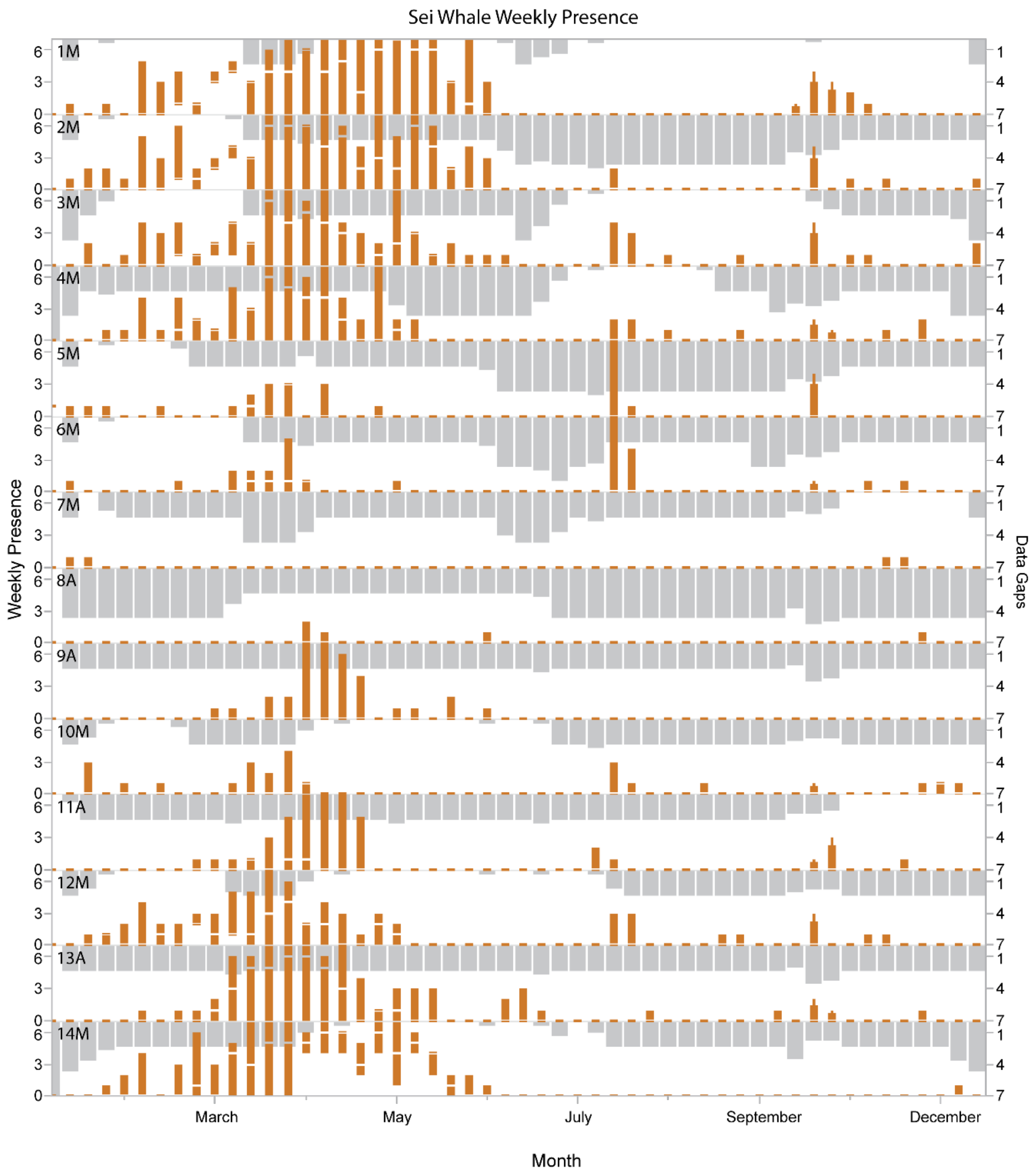

S5 Fig. Quantile boxplots of weekly acoustic presence of sei whales per site in New York Bight between October 2017 and October 2020, shown as proportion of recorded days per week with confirmed sei whale downsweep detections across all sensors (orange). Grey bars indicate the mean number of days per week without data, along the inverted secondary x-axis.

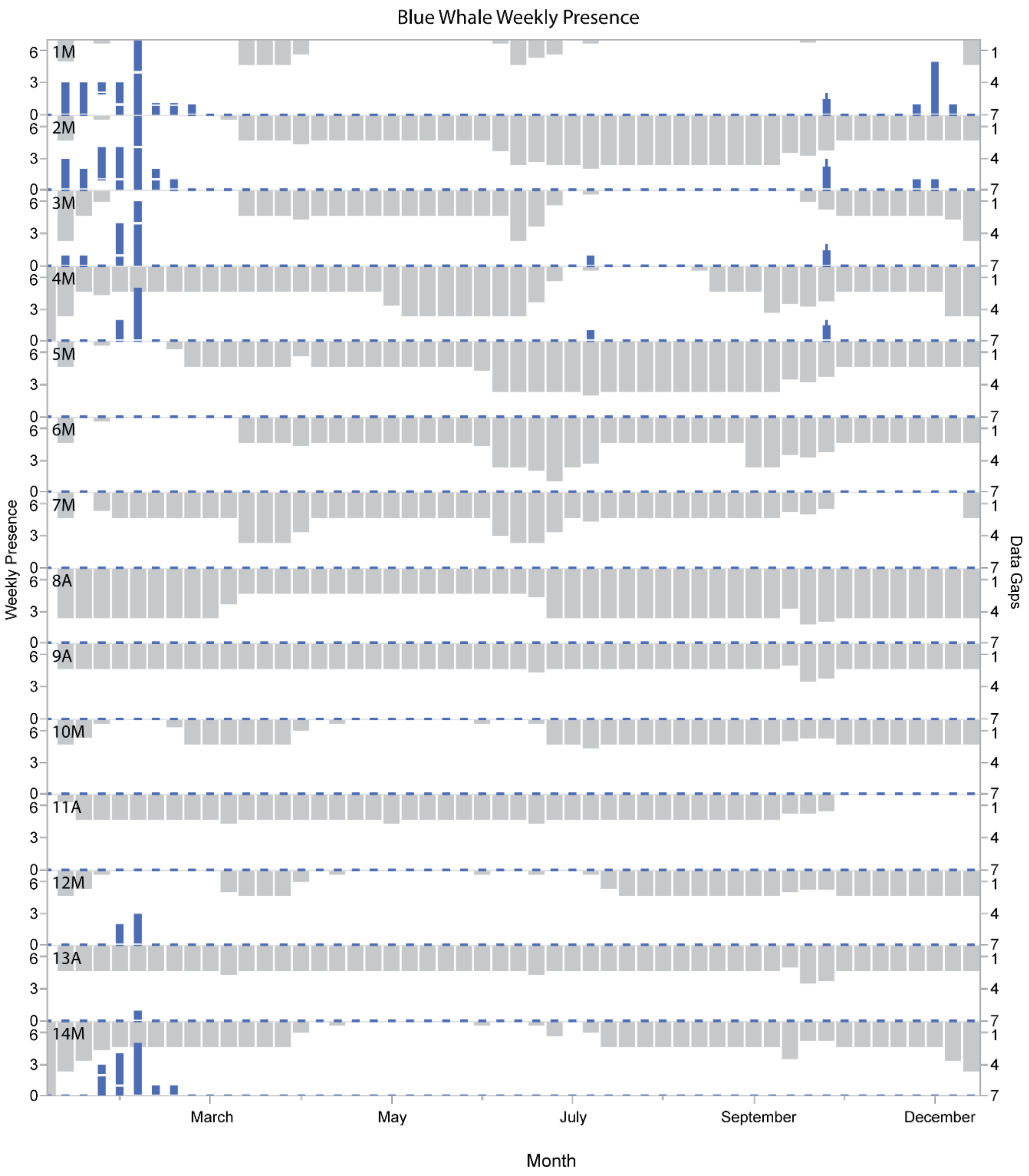

S6 Fig. Quantile boxplots of weekly acoustic presence of blue whales per site in New York Bight between October 2017 and October 2020, shown as proportion of recorded days per week with confirmed blue whale song detections across all sensors (blue). Grey bars indicate the mean number of days per week without data, along the inverted secondary x-axis.

4. Ambient Noise

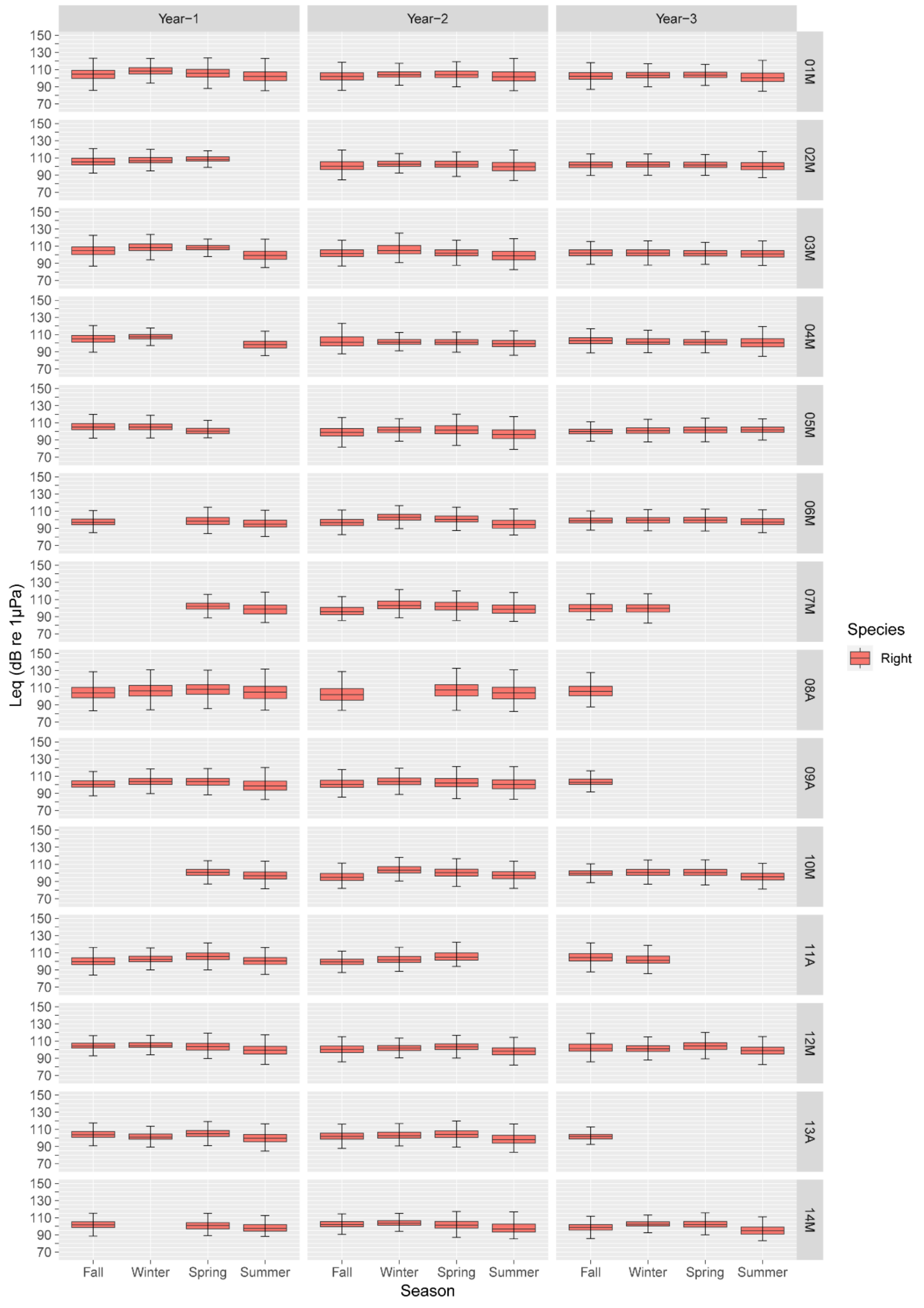

S7 Fig. Boxplots of noise L_{eq} (dB re 1μPa) measurements for the right whale frequency band (70.8 – 224 Hz), survey year (Year 1 = October 2017 – 2018, Year 2 = October 2018 – 2019, Year 3 = October 2019 – 2020), season and site.

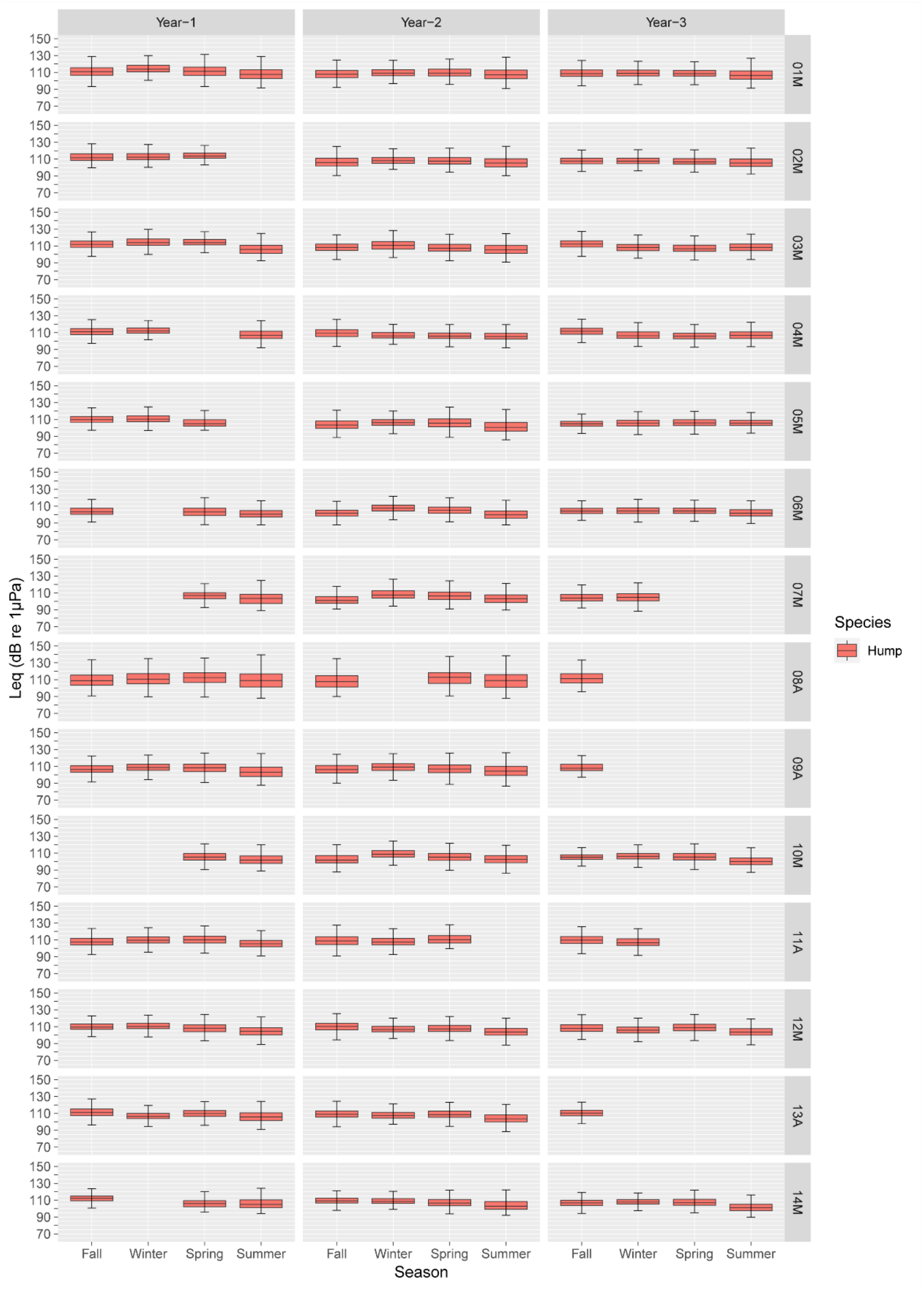

S8 Fig. Boxplots of noise L_{eq} (dB re 1µPa) measurements for the humpback whale frequency band (17.8 – 708 Hz), survey year (Year 1 = October 2017 – 2018, Year 2 = October 2018 – 2019, Year 3 = October 2019 – 2020), season and site.

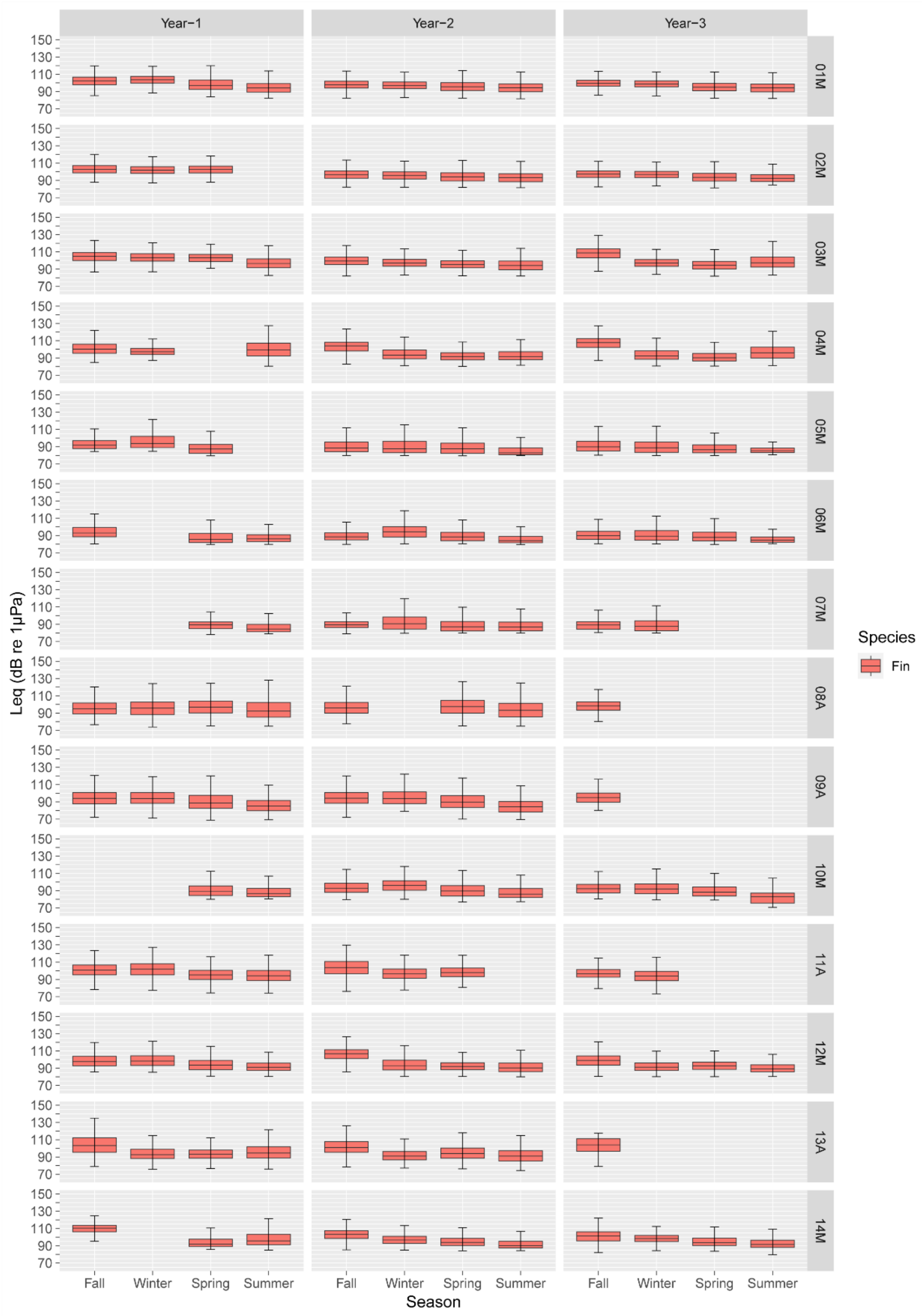

S9 Fig. Boxplots of noise L_{eq} (dB re 1μPa) measurements for the fin whale frequency band (17.8 – 28.2 Hz), survey year (Year 1 = October 2017 – 2018, Year 2 = October 2018 – 2019, Year 3 = October 2019 – 2020), season and site.

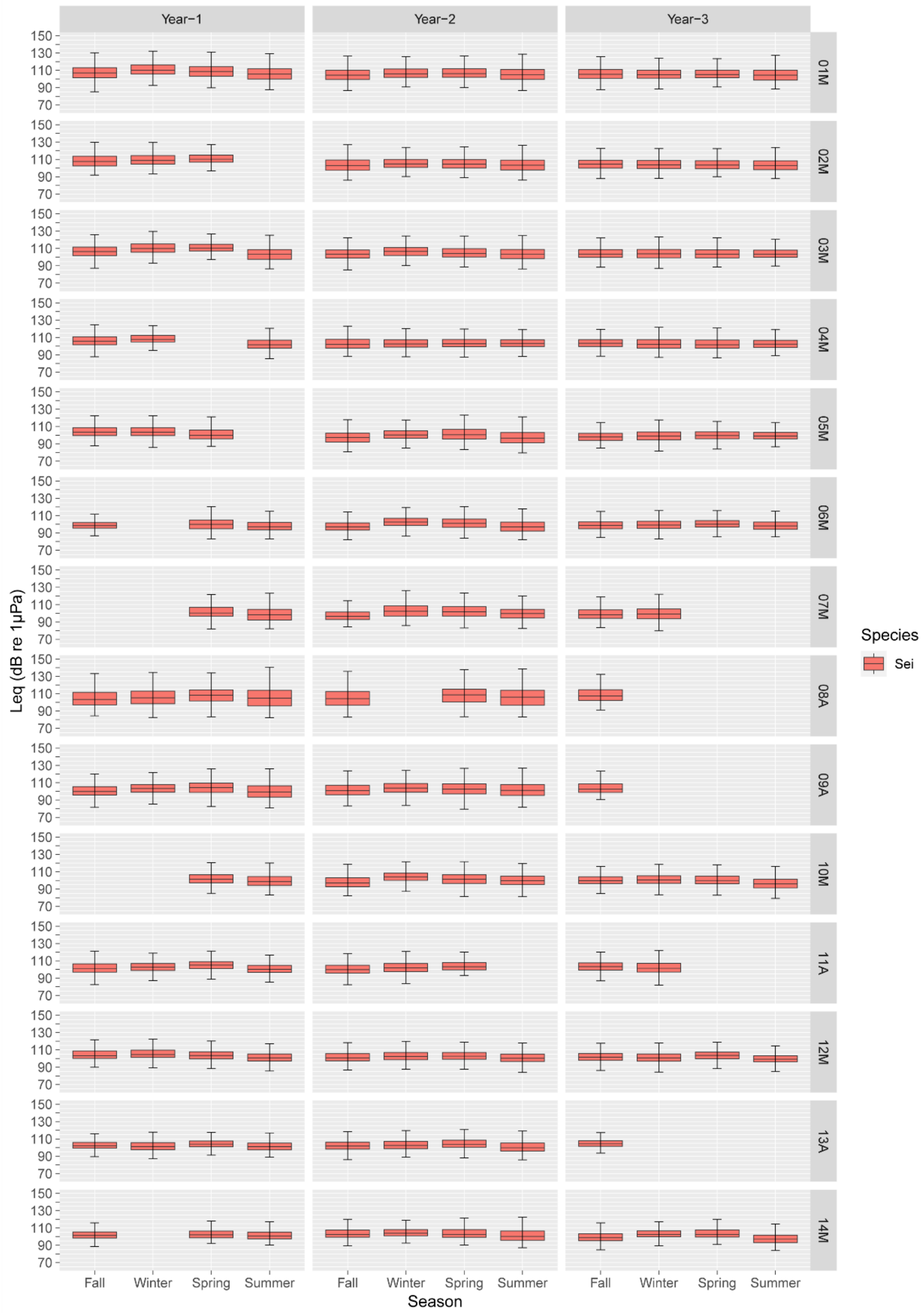

S10 Fig. Boxplots of noise L_{eq} (dB re 1μPa) measurements for the sei whale frequency band (44.7 – 112 Hz), survey year (Year 1 = October 2017 – 2018, Year 2 = October 2018 – 2019, Year 3 = October 2019 – 2020), season and site.

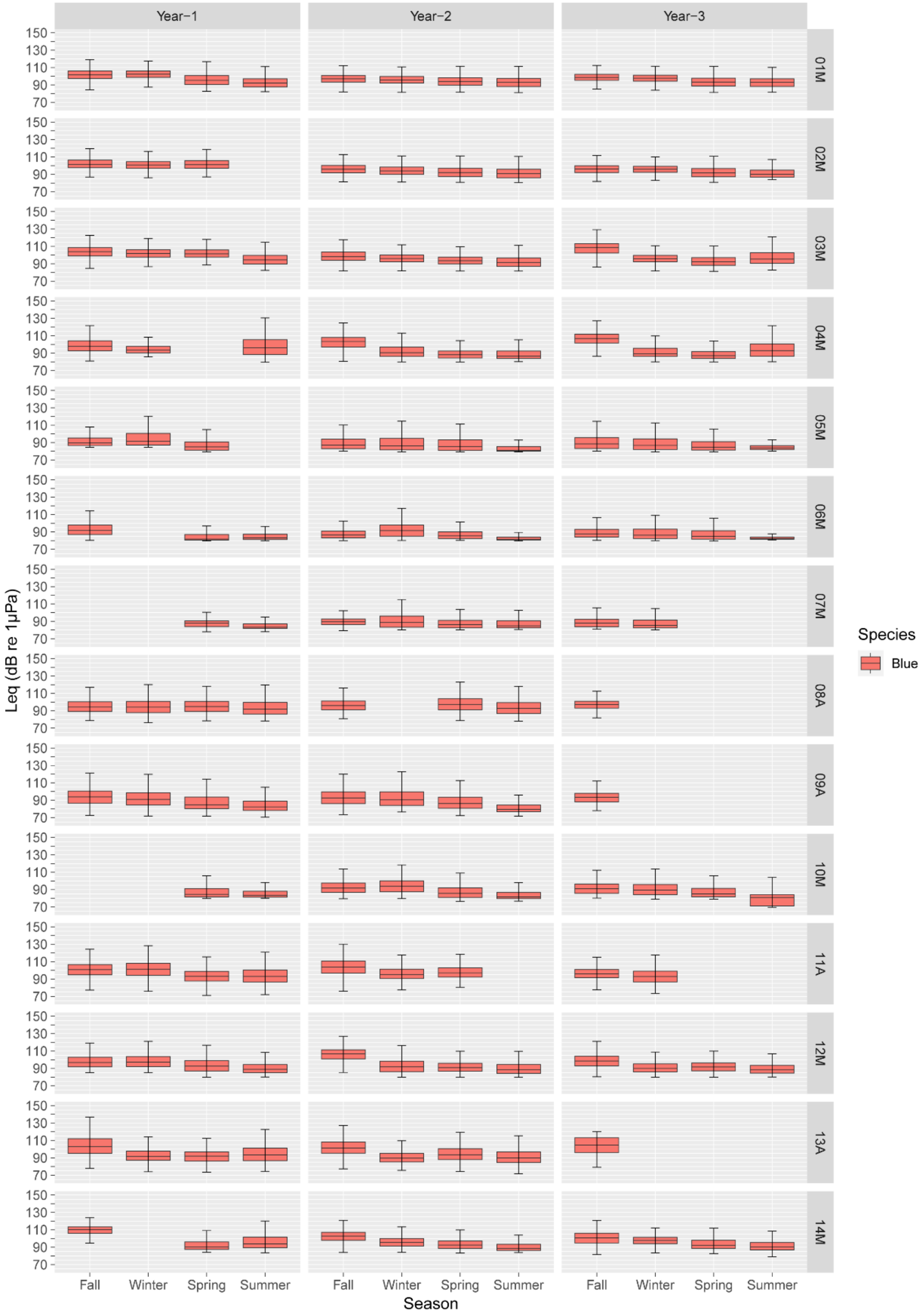

S11 Fig. Boxplots of noise L_{eq} (dB re 1μPa) measurements for the blue whale frequency band (14.1 – 22.4 Hz), survey year (Year 1 = October 2017 – 2018, Year 2 = October 2018 – 2019, Year 3 = October 2019 – 2020), season and site.

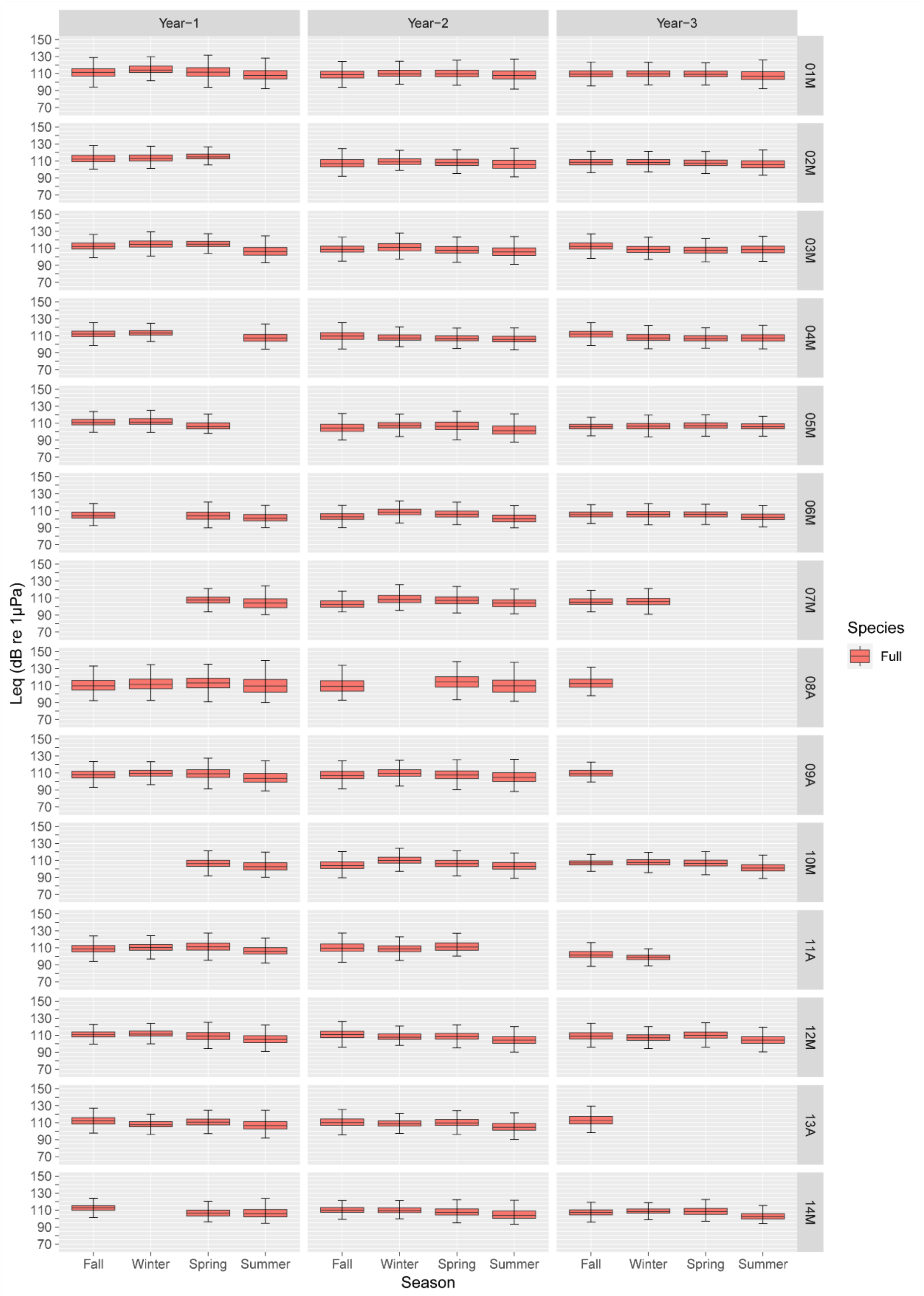

S12 Fig. Boxplots of noise L_{eq} (dB re 1μPa) measurements for the full frequency band (8.9 – 2239.6 Hz), survey year (Year 1 = October 2017 – 2018, Year 2 = October 2018 – 2019, Year 3 = October 2019 – 2020), season and site.

5. Detection Range Estimation

Table S5. Mean and standard error (Std Error) detection range estimates for each species and site averaged across Year 1, Year 2, and Year 3 for the lowest (5th percentile), median (50th percentile), and highest (95th percentile) noise conditions between 16 October 2017 and 15 October 2020 in the NY Bight. Blank cells indicate that the estimated detection range exceeded 500 km. Blank cells in the standard error column indicate that there was only one value used for the estimate.

Site	Species	5th Percentile		50th percentile		95th Percentile	
		Mean (km)	Std Error	Mean (km)	Std Error	Mean (km)	Std Error
**1M**	Blue	312.34	6.99	131.56	15.13	31.08	6.12
**2M**	Blue	323.01	56.94	140.31	35.02	36.91	9.00
**3M**	Blue	301.35	43.37	127.45	25.37	19.71	5.45
**4M**	Blue	333.85		209.86	37.13	24.52	4.94
**5M**	Blue	354.64		321.63	61.22	44.79	12.77
**6M**	Blue			268.29	77.72	80.18	11.49
**7M**	Blue			361.08	16.34	101.80	16.13
**8A**	Blue	425.03		170.66	6.64	29.13	3.53
**9A**	Blue			314.34	10.57	32.59	2.45
**10M**	Blue			287.44	70.28	52.77	20.59
**11A**	Blue	410.24	49.88	141.70	0.87	16.71	3.36
**12M**	Blue	448.09	19.39	149.21	9.99	27.98	5.16
**13A**	Blue			147.62	3.28	16.27	4.00
**14M**	Blue	359.19	15.00	123.81	5.51	22.30	5.10
**1M**	Fin	284.83	0.90	109.58	13.75	22.62	3.96
**2M**	Fin	273.10	47.03	119.03	28.88	28.96	6.89
**3M**	Fin	258.35	39.83	100.89	19.21	16.21	4.07
**4M**	Fin	317.36	62.87	160.86	31.39	18.65	2.66
**5M**	Fin	343.78		267.28	40.88	33.90	9.50
**6M**	Fin			211.52	43.87	66.17	14.27
**7M**	Fin			308.12	34.68	75.22	9.10
**8A**	Fin			160.17	1.19	18.88	0.88
**9A**	Fin			221.04	0.51	28.57	1.50
**10M**	Fin	485.50		214.33	48.68	41.84	15.67
**11A**	Fin	347.18	18.86	122.98	4.81	14.55	2.65
**12M**	Fin	384.76	22.49	152.17	11.19	23.83	4.11
**13A**	Fin	429.32	27.49	137.81	7.67	15.83	4.32
**14M**	Fin	327.21	30.10	110.20	4.49	20.07	4.37
**1M**	Humpback	6.27	0.17	2.39	0.04	1.89	0.04
**2M**	Humpback	5.86	1.60	2.42	0.07	1.94	0.07
**3M**	Humpback	7.30	0.70	2.46	0.07	1.96	0.05

Site	Species	5th Percentile		50th percentile		95th Percentile	
		Mean (km)	Std Error	Mean (km)	Std Error	Mean (km)	Std Error
**4M**	Humpback	9.72	0.74	3.60	0.56	2.08	0.04
**5M**	Humpback	12.49	4.16	3.95	0.70	2.08	0.07
**6M**	Humpback	14.17	1.19	4.60	0.22	2.27	0.04
**7M**	Humpback	12.86	0.76	3.58	0.29	2.08	0.02
**8A**	Humpback	9.92	1.36	2.37	0.01	1.77	0.00
**9A**	Humpback	11.70	0.18	2.90	0.03	2.00	0.02
**10M**	Humpback	14.36	1.34	3.90	0.74	2.13	0.08
**11A**	Humpback	7.97	0.22	3.32	0.04	2.11	0.01
**12M**	Humpback	9.29	0.34	3.55	0.24	2.11	0.04
**13A**	Humpback	8.21	0.70	2.63	0.15	2.04	0.01
**14M**	Humpback	8.78	0.74	2.61	0.12	2.05	0.02
**1M**	Right	18.24	0.34	4.91	0.54	1.94	0.26
**2M**	Right	16.20	4.38	5.45	1.02	1.98	0.26
**3M**	Right	20.76	1.74	5.77	0.97	1.99	0.27
**4M**	Right	23.16	0.92	7.25	0.87	2.05	0.28
**5M**	Right	25.02	8.31	7.05	1.36	2.03	0.27
**6M**	Right	34.32	2.74	11.76	1.65	3.09	0.46
**7M**	Right	35.68	0.82	9.28	0.90	2.12	0.25
**8A**	Right	27.05	3.56	4.55	0.38	1.71	0.36
**9A**	Right	30.63	0.55	7.25	0.03	1.89	0.43
**10M**	Right	35.27	4.68	8.60	1.66	2.28	0.11
**11A**	Right	22.17	1.12	7.00	0.43	1.96	0.38
**12M**	Right	23.81	1.85	6.83	0.64	2.14	0.18
**13A**	Right	20.59	2.28	6.20	0.23	1.89	0.43
**14M**	Right	25.76	2.73	7.58	0.60	2.09	0.30
**1M**	Sei	19.61	0.68	7.93	2.19	6.01	2.00
**2M**	Sei	20.03	4.27	8.00	2.35	6.21	2.10
**3M**	Sei	21.67	1.11	8.27	2.13	6.28	2.10
**4M**	Sei	27.31	3.65	9.12	1.63	6.48	2.15
**5M**	Sei	47.95	11.40	11.93	2.30	6.73	2.27
**6M**	Sei	44.45	2.78	16.29	2.05	7.57	1.79
**7M**	Sei	65.76	2.51	17.51	0.32	6.82	2.22
**8A**	Sei	47.67	2.45	8.73	1.61	4.94	2.84
**9A**	Sei	50.69	0.98	10.61	0.36	5.41	3.09
**10M**	Sei	51.92	7.16	11.30	2.65	6.86	2.30
**11A**	Sei	33.77	2.47	9.68	1.18	5.58	3.22
**12M**	Sei	28.59	2.37	9.78	1.06	6.66	2.21
**13A**	Sei	24.97	1.47	9.34	1.33	5.48	3.11
**14M**	Sei	25.73	3.95	10.02	0.65	6.46	2.07

Supporting Information References

1. Newhall AE, Lin Y-T, Lynch JF, Baumgartner MF, Gawarkiewicz GG. Long distance passive localization of vocalizing sei whales using an acoustic normal mode approach. Journal of the Acoustical Society of America. 2012;131:1814-25. doi: 10.1121/1.3666015.

2. Munger LM, Wiggins SM, Hildebrand JA. North Pacific right whale up-call source levels and propagation distance on the southeastern Bering Sea shelf. Journal of the Acoustical Society of America. 2011;129(6):4047-54. doi: 10.1121/1.3557060

3. Wiggins SM, McDonald MA, Munger LM, Moore SE, Hildebrand JA. Waveguide propagation allows range estimates for North Pacific right whales in the Bering Sea. Canadian Acoustics. 2004;32(2):146-54.

4. Mellinger DK, Bradbury JW. Acoustic measurements of marine mammal sounds in noisy environments. Proceedings of the Second International Conference on Underwater Acoustic Measurements: Technologies and Results. 2007:273-80.

5. Bonnel J, Thode AM, Blackwell SB, Kim K, Macrander AM. Range estimation of bowhead whale (*Balaena mysticetus*) calls in the Arctic using a single hydrophone. Journal of the Acoustical Society of America. 2014;136(1):145-55. doi: 10.1121/1.4883358.

6. Marques TA, Thomas L, Martin SW, Mellinger DK, Ward JA, Moretti DJ, et al. Estimating animal population density using passive acoustics. Biological Reviews. 2013;88(2):287-309. doi: 10.1111/brv.12001.
