# Supplementary material for: Passive acoustic monitoring of baleen whale seasonal presence across the New York Bight
Source: PLoS One. 2025 Feb 13;20(2):e0314857. doi: 10.1371/journal.pone.0314857 (PMC11825016; doi:10.1371/journal.pone.0314857)
Supplement: S2 Table — (PDF) [file pone.0314857.s002.pdf]

3. Species Presence

Table S2. Daily presence for each focal whale species by month across all sites, and the corresponding percentage of days in which each species was detected.

Month-Year	Total Days Recorded	Right		Humpback		Fin		Sei		Blue	
		Days Detected	% Days Detected	Days Detected	% Days Detected	Days Detected	% Days Detected	Days Detected	% Days Detected	Days Detected	% Days Detected
**17-Oct**	16	2	13	3	19	16	100	8	50	0	0
**17-Nov**	30	25	83	11	37	30	100	9	30	3	10
**17-Dec**	31	31	100	28	90	31	100	7	23	7	23
**18-Jan**	31	24	77	26	84	31	100	10	32	8	26
**18-Feb**	28	11	39	28	100	28	100	5	18	8	29
**18-Mar**	31	22	71	25	81	31	100	24	77	0	0
**18-Apr**	30	19	63	25	83	30	100	30	100	0	0
**18-May**	31	14	45	29	94	29	94	27	87	0	0
**18-Jun**	30	7	23	29	97	30	100	7	23	0	0
**18-Jul**	30	3	10	28	93	30	100	6	20	0	0
**18-Aug**	31	4	13	31	100	31	100	2	6	0	0
**18-Sep**	30	3	10	29	97	30	100	0	0	0	0
**18-Oct**	31	4	13	29	94	31	100	3	10	0	0
**18-Nov**	30	19	63	27	90	30	100	5	17	0	0
**18-Dec**	31	28	90	24	77	31	100	1	3	0	0
**19-Jan**	31	24	77	26	84	31	100	0	0	6	19
**19-Feb**	28	16	57	22	79	28	100	1	4	1	4
**19-Mar**	31	24	77	30	97	31	100	27	87	0	0
**19-Apr**	30	28	93	27	90	30	100	30	100	0	0
**19-May**	31	25	81	31	100	31	100	31	100	0	0

Table S2 (continued). Daily presence for each focal whale species by month across all sites, and the corresponding percentage of days in which each species was detected.

Month-Year	Total Days Recorded	Right		Humpback		Fin		Sei		Blue	
		# Days Detected	% Days Detected	# Days Detected	% Days Detected	# Days Detected	% Days Detected	# Days Detected	% Days Detected	# Days Detected	% Days Detected
**19-Jun**	30	23	77	30	100	30	100	10	33	0	0
**19-Jul**	31	2	6	31	100	31	100	0	0	0	0
**19-Aug**	31	7	23	25	81	31	100	14	45	0	0
**19-Sep**	30	2	7	29	97	30	100	4	13	0	0
**19-Oct**	31	3	10	23	74	31	100	0	0	0	0
**19-Nov**	30	12	40	26	87	30	100	3	10	0	0
**19-Dec**	31	19	61	31	100	31	100	2	6	0	0
**20-Jan**	31	14	45	18	58	28	90	4	13	6	19
**20-Feb**	29	0	0	15	52	29	100	25	86	11	38
**20-Mar**	31	0	0	10	32	31	100	26	84	0	0
**20-Apr**	30	16	53	5	17	30	100	26	87	0	0
**20-May**	31	7	23	21	68	31	100	30	97	0	0
**20-Jun**	30	1	3	5	17	28	93	16	53	0	0
**20-Jul**	31	9	29	14	45	31	100	0	0	1	3
**20-Aug**	31	5	16	31	100	31	100	0	0	0	0
**20-Sep**	30	5	17	28	93	30	100	0	0	0	0
**20-Oct**	15	0	0	15	100	15	100	0	0	0	0
