# Supplementary material for: Passive acoustic monitoring of baleen whale seasonal presence across the New York Bight
Source: PLoS One. 2025 Feb 13;20(2):e0314857. doi: 10.1371/journal.pone.0314857 (PMC11825016; doi:10.1371/journal.pone.0314857)
Supplement: S3 Table — (PDF) [file pone.0314857.s003.pdf]

Table S3. Summary of daily detections for each baleen whale species Total Days refers to the number of days that were sampled during the 3-year survey.

Site	Total Days	Right		Humpback		Fin		Sei		Blue	
		Days presence	% Days	Days presence	% Days	Days presence	% Days	Days presence	% Days	Days presence	% Days
**1M**	1031	85	8	244	24	833	81	193	19	42	4
**2M**	679	77	11	140	21	599	88	176	26	36	5
**3M**	848	52	6	297	35	773	91	134	16	20	2
**4M**	693	44	6	277	40	610	88	107	15	10	1
**5M**	655	56	9	172	26	403	62	34	5	0	0
**6M**	727	69	9	251	35	404	56	33	5	0	0
**7M**	757	56	7	197	26	266	35	4	1	0	0
**8A**	492	15	3	43	9	125	25	5	1	0	0
**9A**	733	99	14	188	26	344	47	35	5	0	0
**10M**	856	76	9	213	24	585	68	27	3	0	0
**11A**	814	121	15	300	37	726	89	49	6	0	0
**12M**	896	76	8	280	31	715	80	93	10	5	1
**13A**	732	81	11	386	53	689	94	113	15	1	0
**14M**	801	57	7	194	24	588	73	182	23	17	2
**Total**	10714	964	9	3182	30	7660	71	1185	11	131	1
