# Supplementary material for: Passive acoustic monitoring of baleen whale seasonal presence across the New York Bight
Source: PLoS One. 2025 Feb 13;20(2):e0314857. doi: 10.1371/journal.pone.0314857 (PMC11825016; doi:10.1371/journal.pone.0314857)
Supplement: S4 Table — Blank cells indicate that the estimated detection range exceeded 500 km. Blank cells in the standard error column indicate that there was only one value used for the estimate. (PDF) [file pone.0314857.s004.pdf]

4. Detection Range Estimation

Table S4. Mean and standard error (Std Error) detection range estimates for each species and site averaged across Year 1, Year 2, and Year 3 for the lowest (5th percentile), median (50th percentile), and highest (95th percentile) noise conditions between 16 October 2017 and 15 October 2020 in the NY Bight. Blank cells indicate that the estimated detection range exceeded 500 km. Blank cells in the standard error column indicate that there was only one value used for the estimate.

Site	Species	5th Percentile		50th percentile		95th Percentile	
		Mean (km)	Std Error	Mean (km)	Std Error	Mean (km)	Std Error
**1M**	Blue	312.34	6.99	131.56	15.13	31.08	6.12
**2M**	Blue	323.01	56.94	140.31	35.02	36.91	9.00
**3M**	Blue	301.35	43.37	127.45	25.37	19.71	5.45
**4M**	Blue	333.85		209.86	37.13	24.52	4.94
**5M**	Blue	354.64		321.63	61.22	44.79	12.77
**6M**	Blue			268.29	77.72	80.18	11.49
**7M**	Blue			361.08	16.34	101.80	16.13
**8A**	Blue	425.03		170.66	6.64	29.13	3.53
**9A**	Blue			314.34	10.57	32.59	2.45
**10M**	Blue			287.44	70.28	52.77	20.59
**11A**	Blue	410.24	49.88	141.70	0.87	16.71	3.36
**12M**	Blue	448.09	19.39	149.21	9.99	27.98	5.16
**13A**	Blue			147.62	3.28	16.27	4.00
**14M**	Blue	359.19	15.00	123.81	5.51	22.30	5.10
**1M**	Fin	284.83	0.90	109.58	13.75	22.62	3.96
**2M**	Fin	273.10	47.03	119.03	28.88	28.96	6.89
**3M**	Fin	258.35	39.83	100.89	19.21	16.21	4.07
**4M**	Fin	317.36	62.87	160.86	31.39	18.65	2.66
**5M**	Fin	343.78		267.28	40.88	33.90	9.50
**6M**	Fin			211.52	43.87	66.17	14.27
**7M**	Fin			308.12	34.68	75.22	9.10
**8A**	Fin			160.17	1.19	18.88	0.88
**9A**	Fin			221.04	0.51	28.57	1.50
**10M**	Fin	485.50		214.33	48.68	41.84	15.67
**11A**	Fin	347.18	18.86	122.98	4.81	14.55	2.65
**12M**	Fin	384.76	22.49	152.17	11.19	23.83	4.11
**13A**	Fin	429.32	27.49	137.81	7.67	15.83	4.32
**14M**	Fin	327.21	30.10	110.20	4.49	20.07	4.37
**1M**	Humpback	6.27	0.17	2.39	0.04	1.89	0.04
**2M**	Humpback	5.86	1.60	2.42	0.07	1.94	0.07
**3M**	Humpback	7.30	0.70	2.46	0.07	1.96	0.05

Site	Species	5th Percentile		50th percentile		95th Percentile	
		Mean (km)	Std Error	Mean (km)	Std Error	Mean (km)	Std Error
**4M**	Humpback	9.72	0.74	3.60	0.56	2.08	0.04
**5M**	Humpback	12.49	4.16	3.95	0.70	2.08	0.07
**6M**	Humpback	14.17	1.19	4.60	0.22	2.27	0.04
**7M**	Humpback	12.86	0.76	3.58	0.29	2.08	0.02
**8A**	Humpback	9.92	1.36	2.37	0.01	1.77	0.00
**9A**	Humpback	11.70	0.18	2.90	0.03	2.00	0.02
**10M**	Humpback	14.36	1.34	3.90	0.74	2.13	0.08
**11A**	Humpback	7.97	0.22	3.32	0.04	2.11	0.01
**12M**	Humpback	9.29	0.34	3.55	0.24	2.11	0.04
**13A**	Humpback	8.21	0.70	2.63	0.15	2.04	0.01
**14M**	Humpback	8.78	0.74	2.61	0.12	2.05	0.02
**1M**	Right	18.24	0.34	4.91	0.54	1.94	0.26
**2M**	Right	16.20	4.38	5.45	1.02	1.98	0.26
**3M**	Right	20.76	1.74	5.77	0.97	1.99	0.27
**4M**	Right	23.16	0.92	7.25	0.87	2.05	0.28
**5M**	Right	25.02	8.31	7.05	1.36	2.03	0.27
**6M**	Right	34.32	2.74	11.76	1.65	3.09	0.46
**7M**	Right	35.68	0.82	9.28	0.90	2.12	0.25
**8A**	Right	27.05	3.56	4.55	0.38	1.71	0.36
**9A**	Right	30.63	0.55	7.25	0.03	1.89	0.43
**10M**	Right	35.27	4.68	8.60	1.66	2.28	0.11
**11A**	Right	22.17	1.12	7.00	0.43	1.96	0.38
**12M**	Right	23.81	1.85	6.83	0.64	2.14	0.18
**13A**	Right	20.59	2.28	6.20	0.23	1.89	0.43
**14M**	Right	25.76	2.73	7.58	0.60	2.09	0.30
**1M**	Sei	19.61	0.68	7.93	2.19	6.01	2.00
**2M**	Sei	20.03	4.27	8.00	2.35	6.21	2.10
**3M**	Sei	21.67	1.11	8.27	2.13	6.28	2.10
**4M**	Sei	27.31	3.65	9.12	1.63	6.48	2.15
**5M**	Sei	47.95	11.40	11.93	2.30	6.73	2.27
**6M**	Sei	44.45	2.78	16.29	2.05	7.57	1.79
**7M**	Sei	65.76	2.51	17.51	0.32	6.82	2.22
**8A**	Sei	47.67	2.45	8.73	1.61	4.94	2.84
**9A**	Sei	50.69	0.98	10.61	0.36	5.41	3.09
**10M**	Sei	51.92	7.16	11.30	2.65	6.86	2.30
**11A**	Sei	33.77	2.47	9.68	1.18	5.58	3.22
**12M**	Sei	28.59	2.37	9.78	1.06	6.66	2.21
**13A**	Sei	24.97	1.47	9.34	1.33	5.48	3.11
**14M**	Sei	25.73	3.95	10.02	0.65	6.46	2.07
