# Supplementary figures and images for: Passive acoustic monitoring of baleen whale seasonal presence across the New York Bight
Source: PLoS One. 2025 Feb 13;20(2):e0314857. doi: 10.1371/journal.pone.0314857 (PMC11825016; doi:10.1371/journal.pone.0314857)

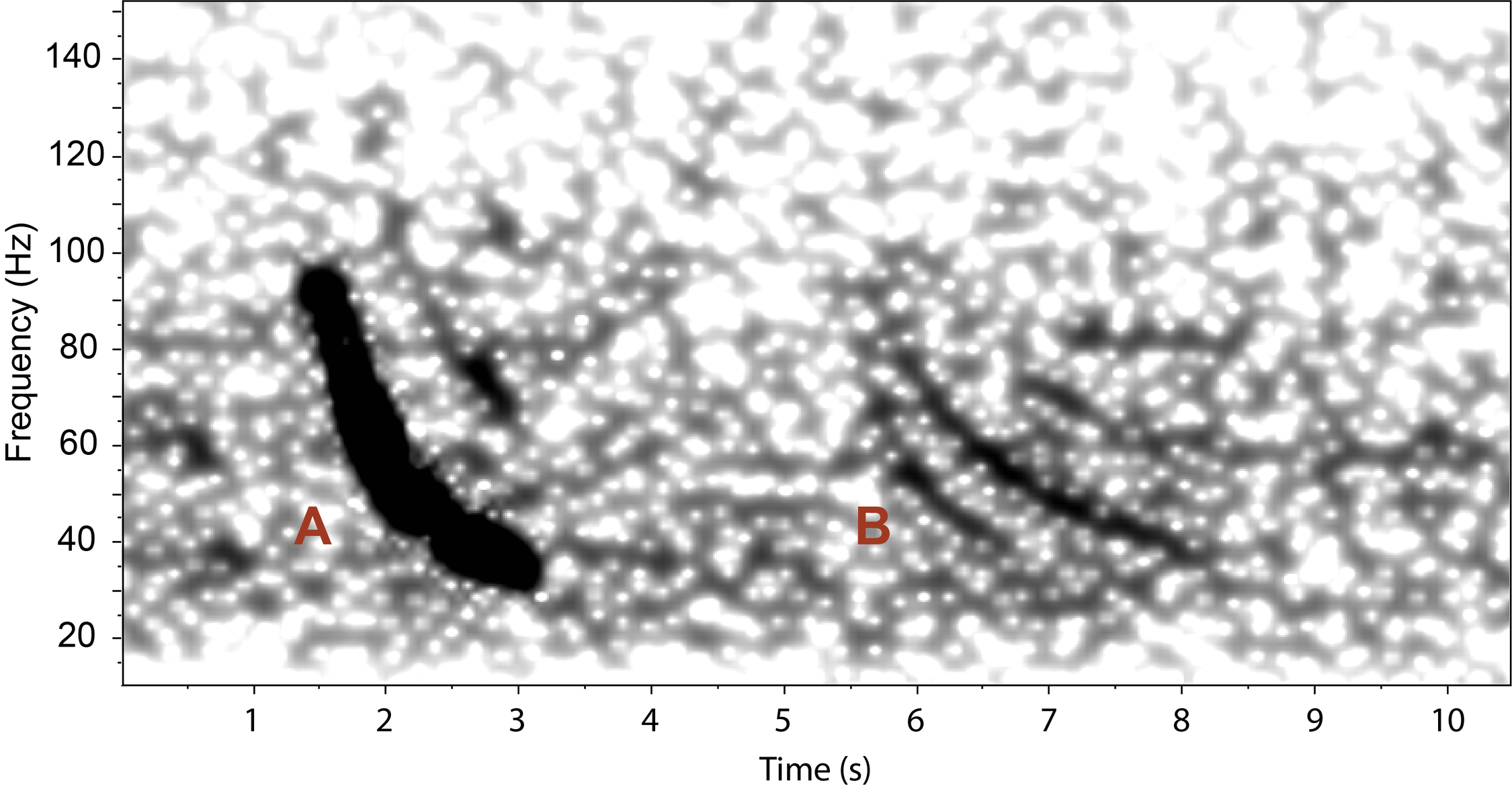

Supplement: S1 Fig — Spectrogram of sei whale downsweeps exhibiting frequency dispersion: A) a sei whale downsweep similar to the templates used for the template detector, B) a multi-path (distorted) sei whale downsweep, which was often not detected by the template detector. Spectrogram was created with a window size = 2048, DFT = 4096, with frequency and time bins of 1.22 Hz and 0.0614 s, respectively. (TIF) [file pone.0314857.s006.tif]

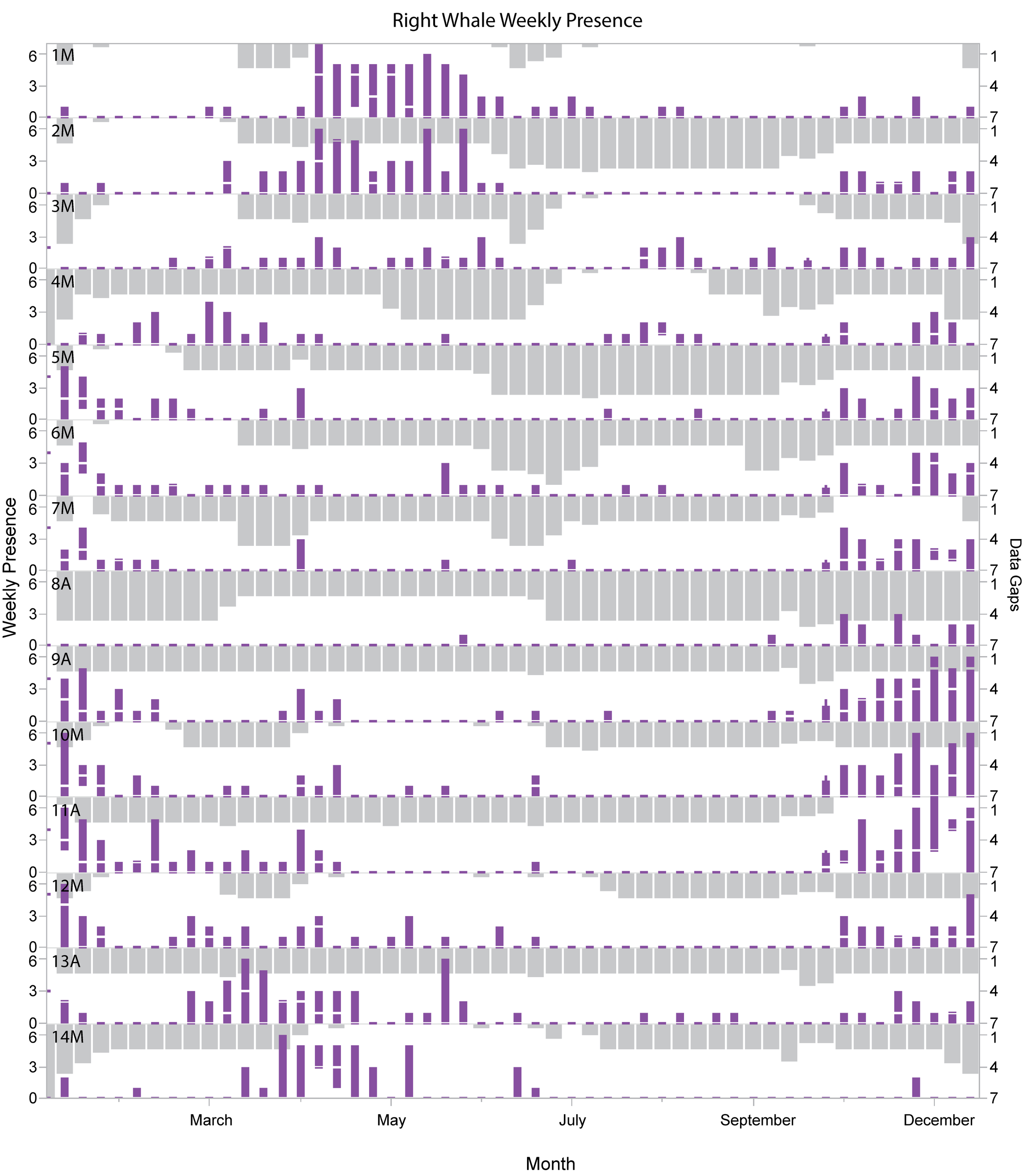

Supplement: S2 Fig — Grey bars indicate the mean number of days per week without data, along the inverted secondary x-axis. (TIF) [file pone.0314857.s007.tif]

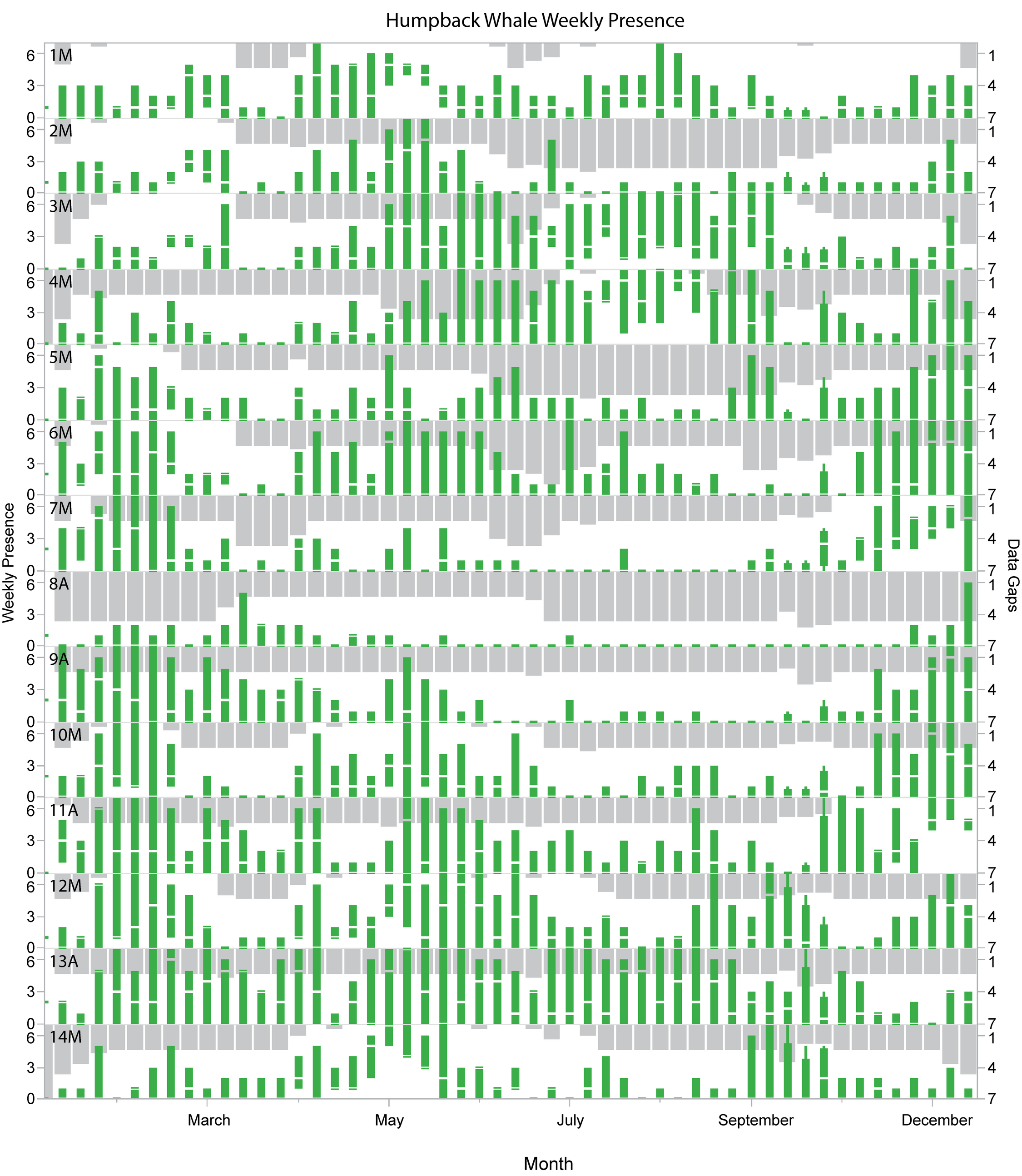

Supplement: S3 Fig — Grey bars indicate the mean number of days per week without data, along the inverted secondary x-axis. (TIF) [file pone.0314857.s008.tif]

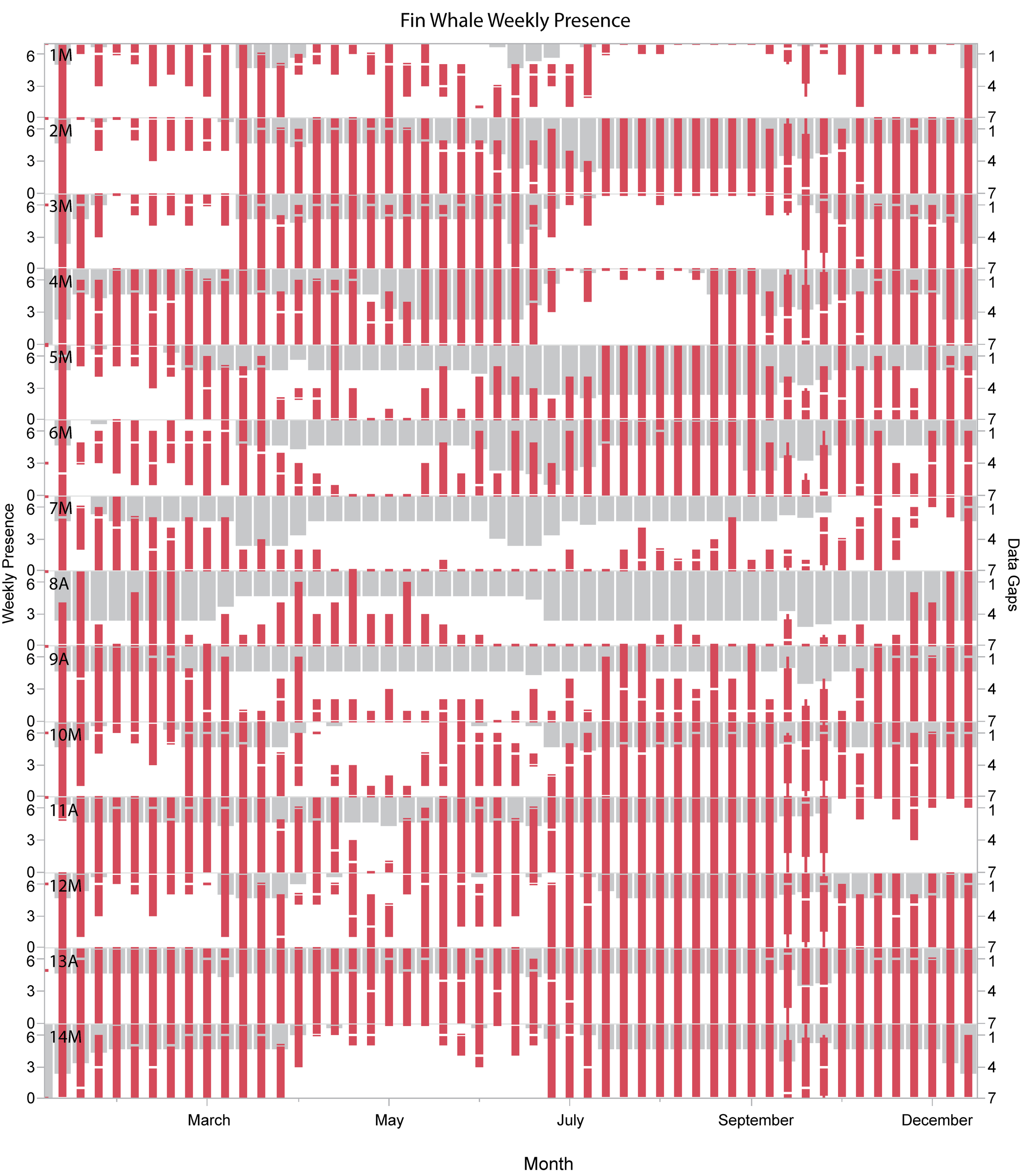

Supplement: S4 Fig — Grey bars indicate the mean number of days per week without data, along the inverted secondary x-axis. (TIF) [file pone.0314857.s009.tif]

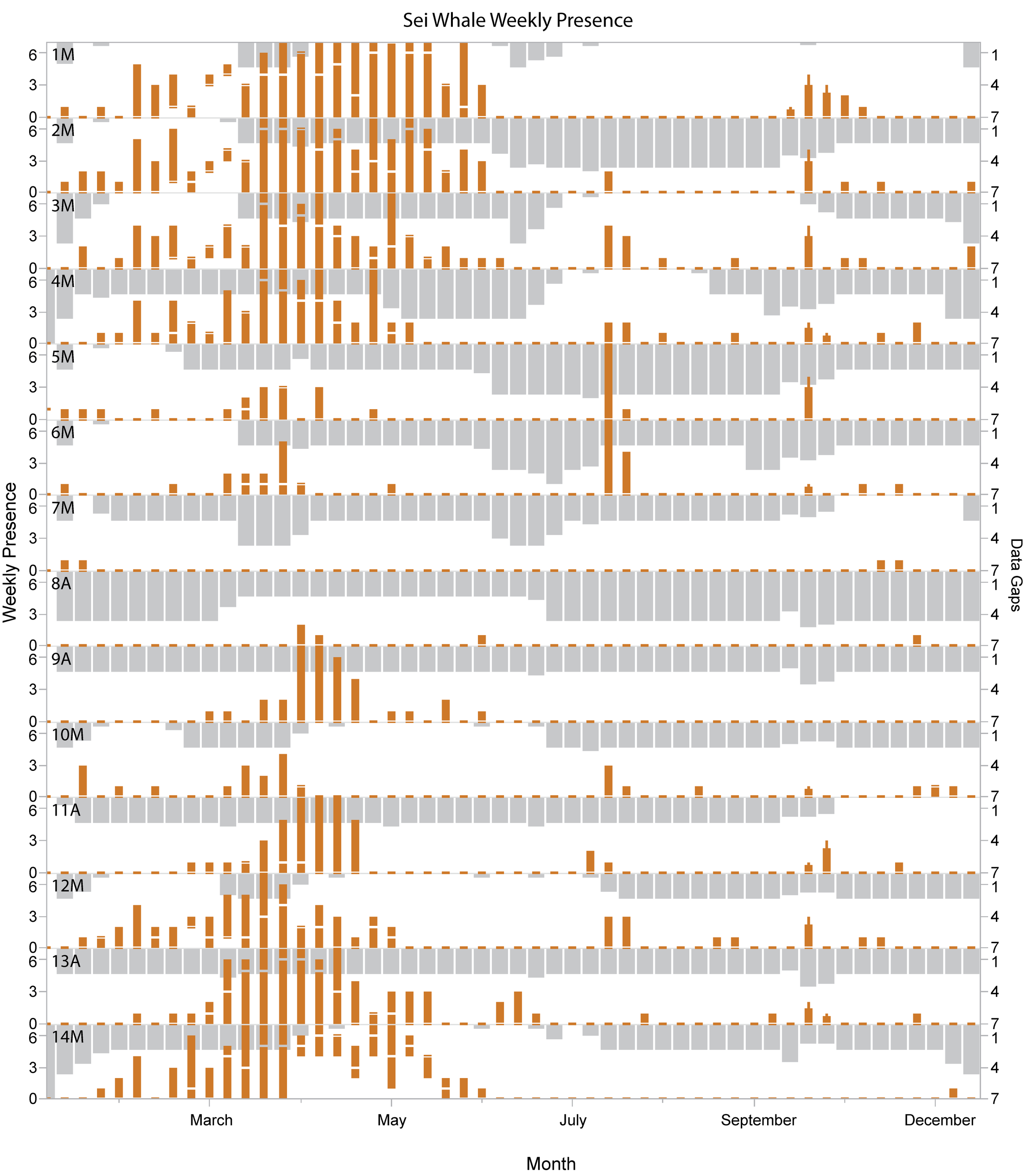

Supplement: S5 Fig — Grey bars indicate the mean number of days per week without data, along the inverted secondary x-axis. (TIF) [file pone.0314857.s010.tif]

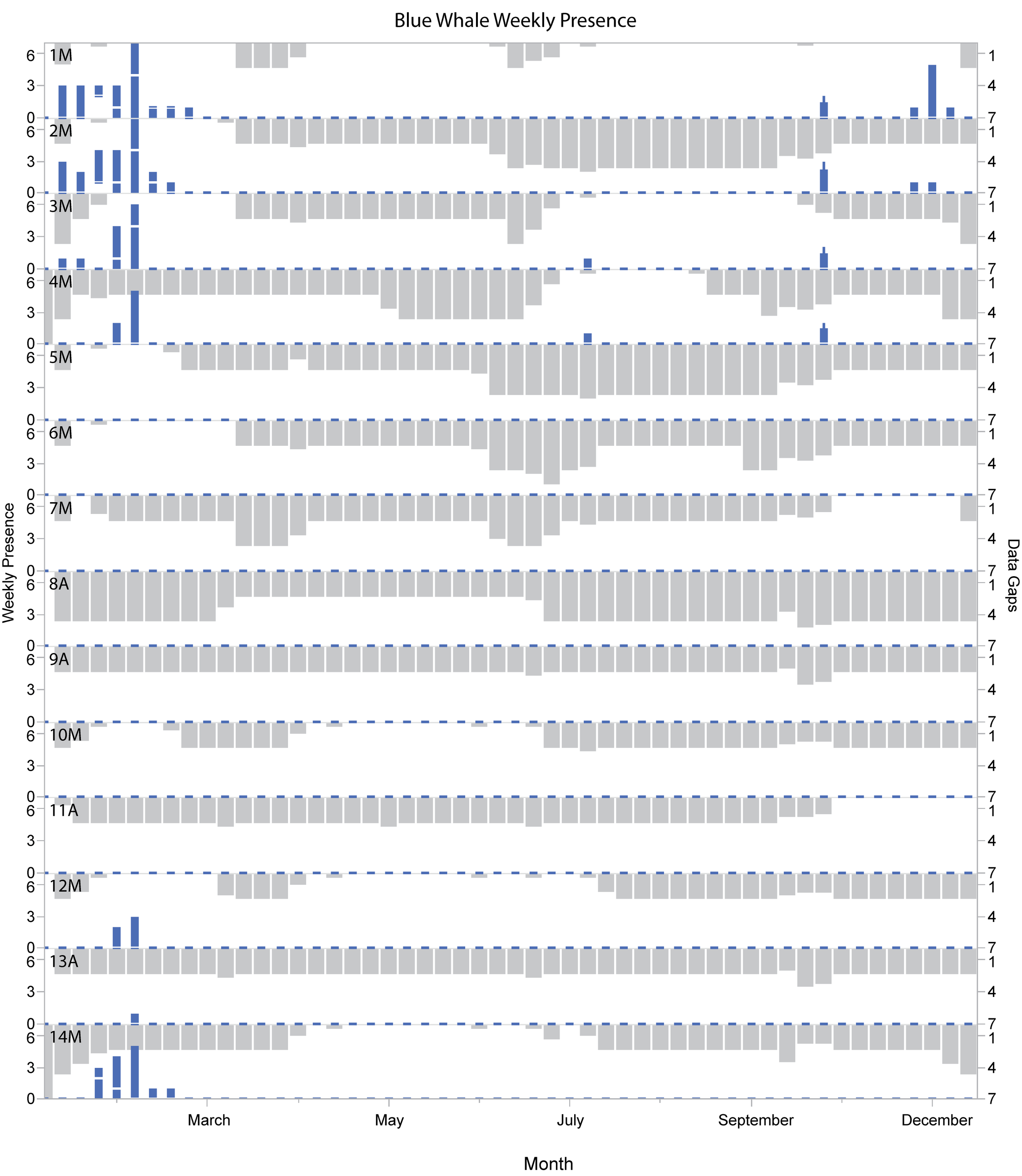

Supplement: S6 Fig — Grey bars indicate the mean number of days per week without data, along the inverted secondary x-axis. (TIF) [file pone.0314857.s011.tif]

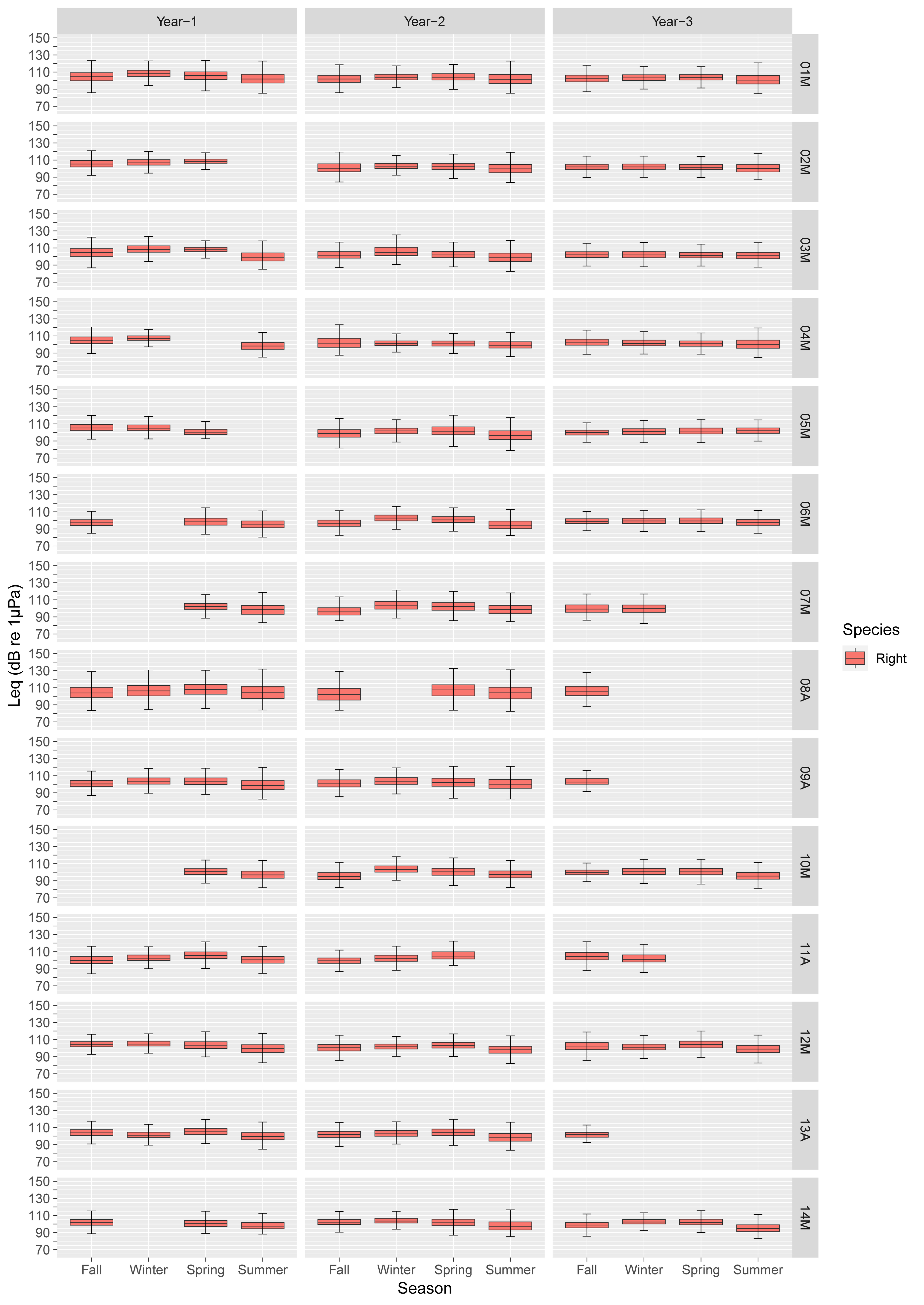

Supplement: S7 Fig — (TIF) [file pone.0314857.s012.tif]

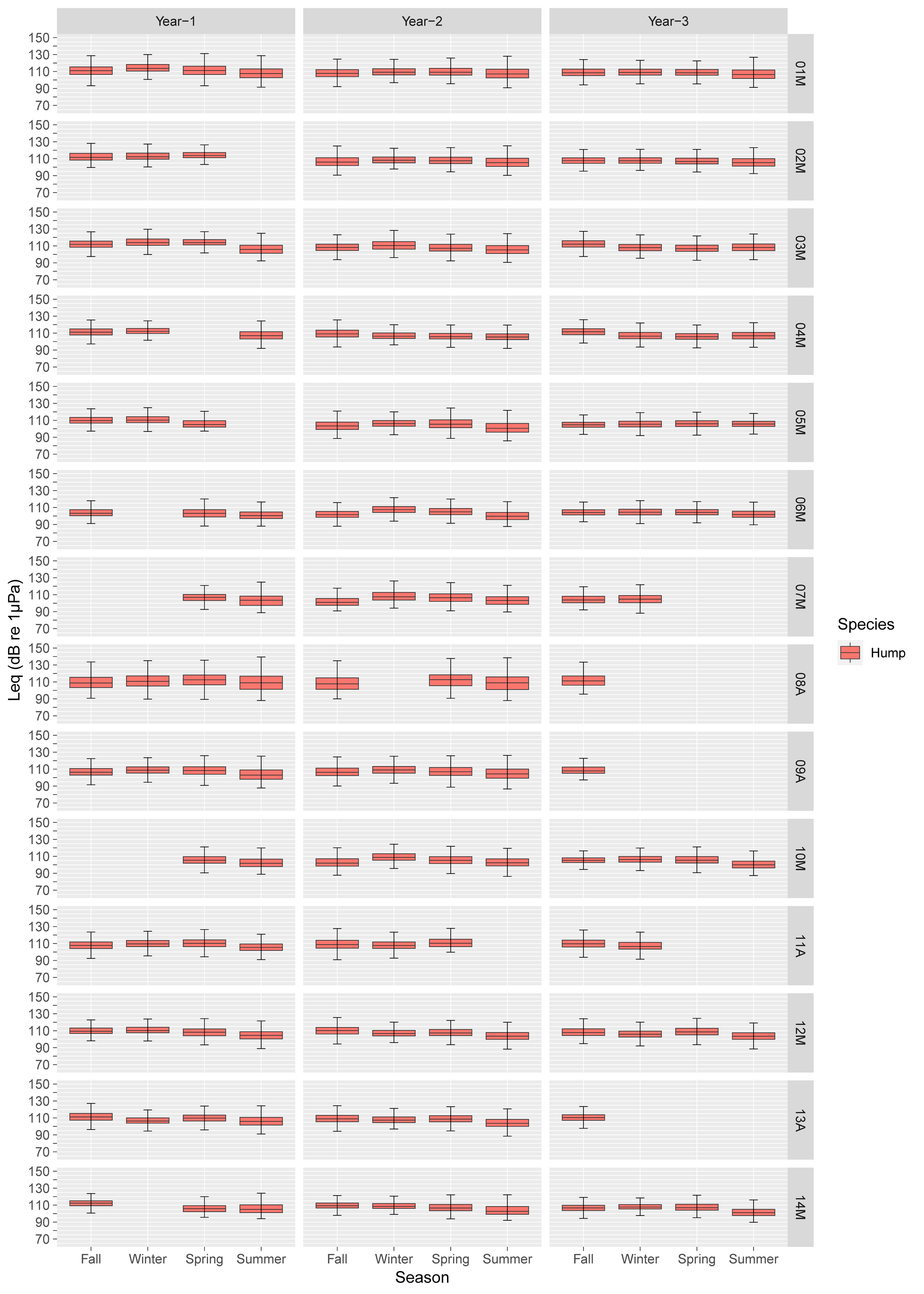

Supplement: S8 Fig — (TIF) [file pone.0314857.s013.tif]

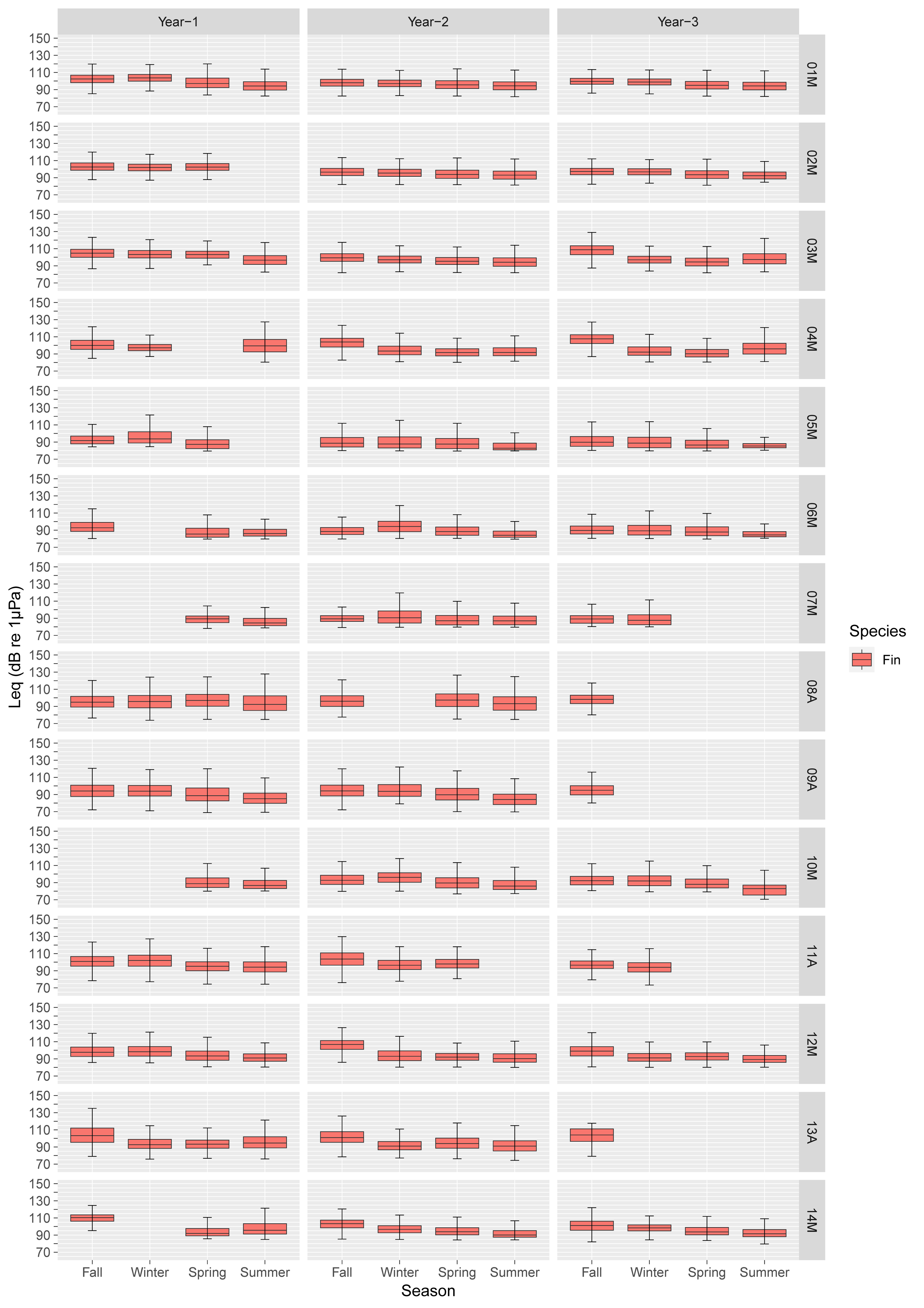

Supplement: S9 Fig — (TIF) [file pone.0314857.s014.tif]

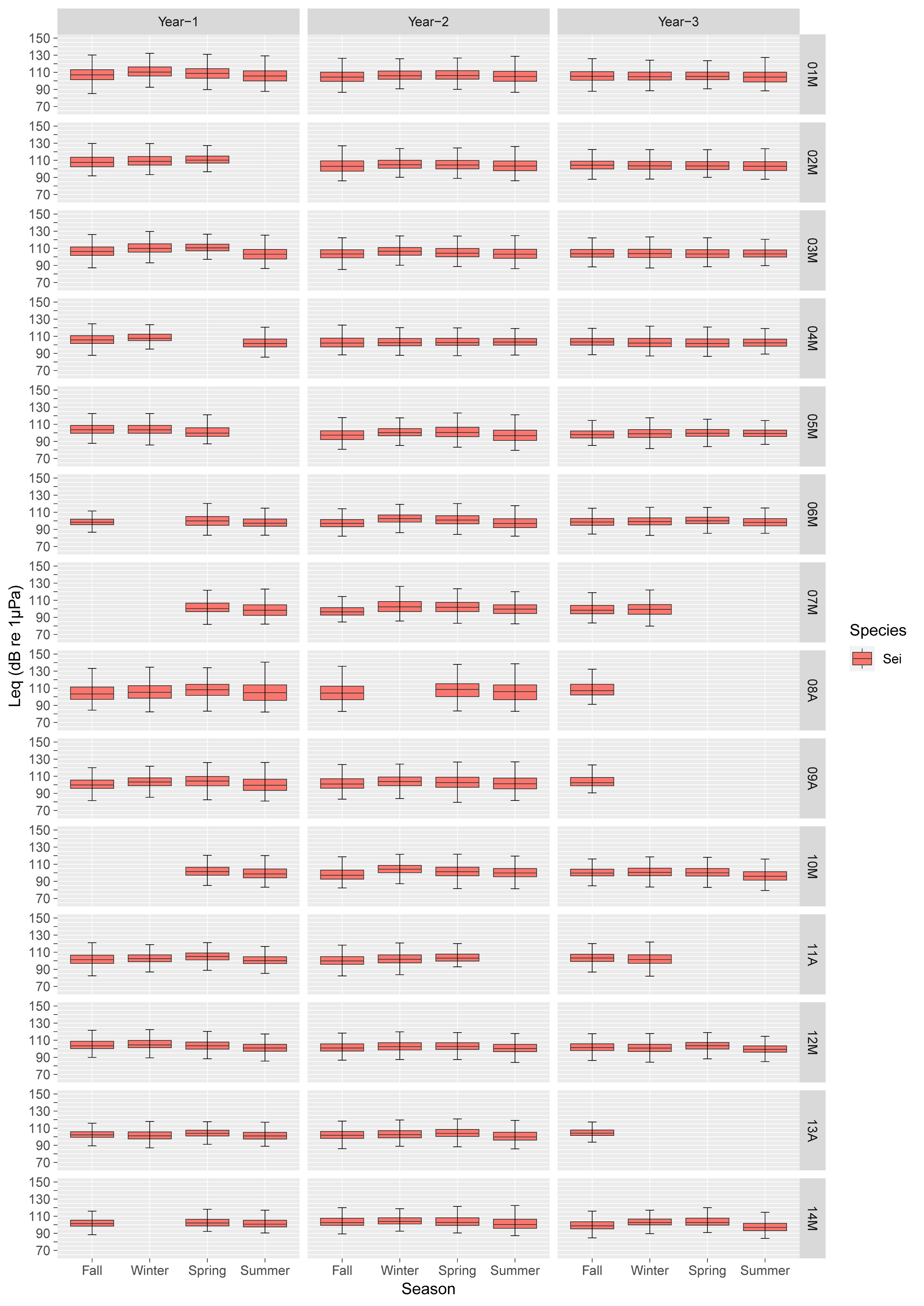

Supplement: S10 Fig — (TIF) [file pone.0314857.s015.tif]

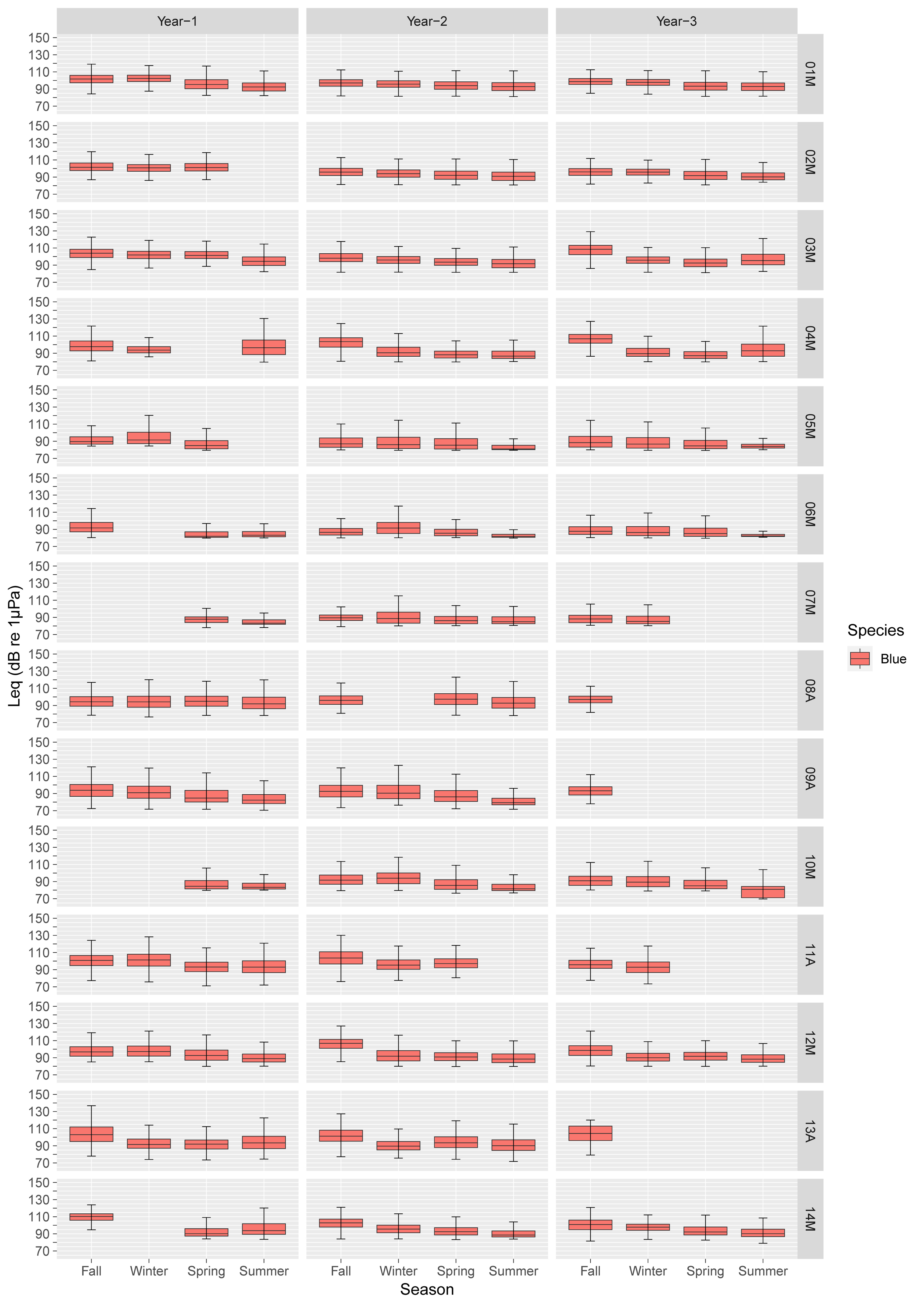

Supplement: S11 Fig — (TIF) [file pone.0314857.s016.tif]

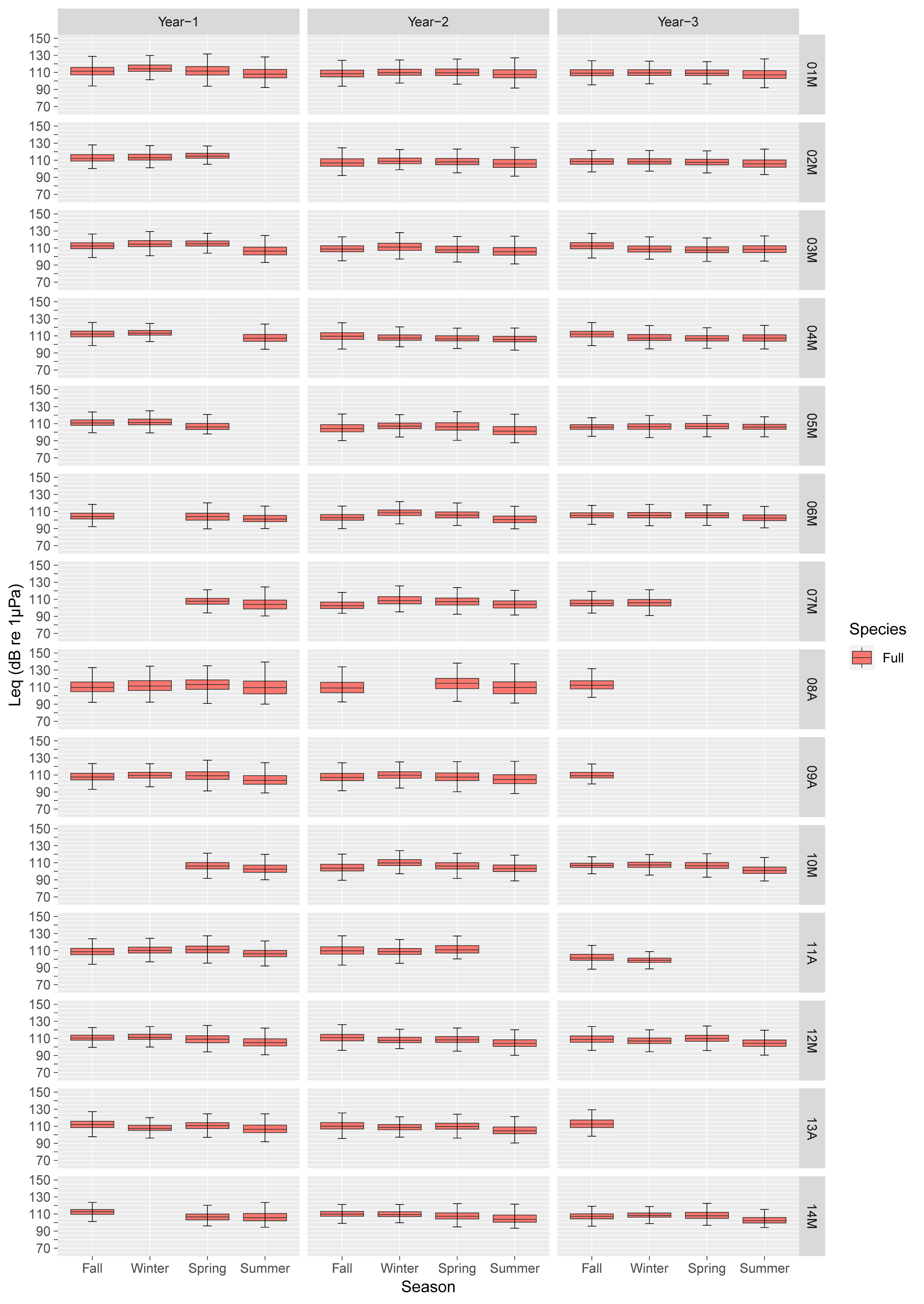

Supplement: S12 Fig — (TIF) [file pone.0314857.s017.tif]
