# Supplementary material for: Passive acoustic monitoring of baleen whale seasonal presence across the New York Bight
Source: PLoS One. 2025 Feb 13;20(2):e0314857. doi: 10.1371/journal.pone.0314857 (PMC11825016; doi:10.1371/journal.pone.0314857)
Supplement: S5 Table — Presence = 1 denotes a detection of the target species on that day and site, Presence = 0 denotes no detection of the target species, and a blank cell denotes there are no data for that date and site. Data Gap indicates dates with sound recording for a site (0) and dates without sound recording for the site (1).e. (PDF) [file pone.0314857.s005.pdf]

S5 Table. Daily acoustic detection of North Atlantic right whale (RI), humpback whale (HB), fin whale (FP), sei whale (SE), and blue whale (BL) signals by site in the New York Bight between October 2017 and October 2020. Presence = 1 denotes a detection of the target species on that day and site, Presence = 0 denotes no detection of the target species, and a blank cell denotes there are no data for that date and site. Data Gap indicates dates with sound recording for a site (0) and dates without sound recording for the site (1).

Date	Species	Site	Month	Year	Survey Year	Presence	Data Gap	Season
10/16/2017	BL	1M	10	2017	Year-1	0	0	Fall
10/16/2017	BL	2M	10	2017	Year-1	0	0	Fall
10/16/2017	BL	3M	10	2017	Year-1	0	0	Fall
10/16/2017	BL	4M	10	2017	Year-1	0	0	Fall
10/16/2017	BL	5M	10	2017	Year-1	0	0	Fall
10/16/2017	BL	6M	10	2017	Year-1	0	0	Fall
10/16/2017	BL	7M	10	2017	Year-1	0	0	Fall
10/16/2017	BL	10M	10	2017	Year-1	0	0	Fall
10/16/2017	BL	12M	10	2017	Year-1	0	0	Fall
10/16/2017	BL	14M	10	2017	Year-1		1	Fall
10/16/2017	BL	8A	10	2017	Year-1	0	0	Fall
10/16/2017	BL	9A	10	2017	Year-1	0	0	Fall
10/16/2017	BL	11A	10	2017	Year-1	0	0	Fall
10/16/2017	BL	13A	10	2017	Year-1	0	0	Fall
10/17/2017	BL	1M	10	2017	Year-1	0	0	Fall
10/17/2017	BL	2M	10	2017	Year-1	0	0	Fall
10/17/2017	BL	3M	10	2017	Year-1	0	0	Fall
10/17/2017	BL	4M	10	2017	Year-1	0	0	Fall
10/17/2017	BL	5M	10	2017	Year-1	0	0	Fall
10/17/2017	BL	6M	10	2017	Year-1	0	0	Fall
10/17/2017	BL	7M	10	2017	Year-1	0	0	Fall
10/17/2017	BL	10M	10	2017	Year-1	0	0	Fall
10/17/2017	BL	12M	10	2017	Year-1	0	0	Fall
10/17/2017	BL	14M	10	2017	Year-1		1	Fall
10/17/2017	BL	8A	10	2017	Year-1	0	0	Fall
10/17/2017	BL	9A	10	2017	Year-1	0	0	Fall
10/17/2017	BL	11A	10	2017	Year-1	0	0	Fall
10/17/2017	BL	13A	10	2017	Year-1	0	0	Fall
10/18/2017	BL	1M	10	2017	Year-1	0	0	Fall
10/18/2017	BL	2M	10	2017	Year-1	0	0	Fall
10/18/2017	BL	3M	10	2017	Year-1	0	0	Fall
10/18/2017	BL	4M	10	2017	Year-1	0	0	Fall
10/18/2017	BL	5M	10	2017	Year-1	0	0	Fall
10/18/2017	BL	6M	10	2017	Year-1	0	0	Fall
10/18/2017	BL	7M	10	2017	Year-1	0	0	Fall
10/18/2017	BL	10M	10	2017	Year-1	0	0	Fall
10/18/2017	BL	12M	10	2017	Year-1	0	0	Fall

Date	Species	Site	Month	Year	Survey Year	Presence	Data Gap	Season
10/18/2017	BL	14M	10	2017	Year-1		1	Fall
10/18/2017	BL	8A	10	2017	Year-1	0	0	Fall
10/18/2017	BL	9A	10	2017	Year-1	0	0	Fall
10/18/2017	BL	11A	10	2017	Year-1	0	0	Fall
10/18/2017	BL	13A	10	2017	Year-1	0	0	Fall
10/19/2017	BL	1M	10	2017	Year-1	0	0	Fall
10/19/2017	BL	2M	10	2017	Year-1	0	0	Fall
10/19/2017	BL	3M	10	2017	Year-1	0	0	Fall
10/19/2017	BL	4M	10	2017	Year-1	0	0	Fall
10/19/2017	BL	5M	10	2017	Year-1	0	0	Fall
10/19/2017	BL	6M	10	2017	Year-1	0	0	Fall
10/19/2017	BL	7M	10	2017	Year-1	0	0	Fall
10/19/2017	BL	10M	10	2017	Year-1	0	0	Fall
10/19/2017	BL	12M	10	2017	Year-1	0	0	Fall
10/19/2017	BL	14M	10	2017	Year-1		1	Fall
10/19/2017	BL	8A	10	2017	Year-1	0	0	Fall
10/19/2017	BL	9A	10	2017	Year-1	0	0	Fall
10/19/2017	BL	11A	10	2017	Year-1	0	0	Fall
10/19/2017	BL	13A	10	2017	Year-1	0	0	Fall
10/20/2017	BL	1M	10	2017	Year-1	0	0	Fall
10/20/2017	BL	2M	10	2017	Year-1	0	0	Fall
10/20/2017	BL	3M	10	2017	Year-1	0	0	Fall
10/20/2017	BL	4M	10	2017	Year-1	0	0	Fall
10/20/2017	BL	5M	10	2017	Year-1	0	0	Fall
10/20/2017	BL	6M	10	2017	Year-1	0	0	Fall
10/20/2017	BL	7M	10	2017	Year-1	0	0	Fall
10/20/2017	BL	10M	10	2017	Year-1	0	0	Fall
10/20/2017	BL	12M	10	2017	Year-1	0	0	Fall
10/20/2017	BL	14M	10	2017	Year-1		1	Fall
10/20/2017	BL	8A	10	2017	Year-1	0	0	Fall
10/20/2017	BL	9A	10	2017	Year-1	0	0	Fall
10/20/2017	BL	11A	10	2017	Year-1	0	0	Fall
10/20/2017	BL	13A	10	2017	Year-1	0	0	Fall
10/21/2017	BL	1M	10	2017	Year-1	0	0	Fall
10/21/2017	BL	2M	10	2017	Year-1	0	0	Fall
10/21/2017	BL	3M	10	2017	Year-1	0	0	Fall
10/21/2017	BL	4M	10	2017	Year-1	0	0	Fall
10/21/2017	BL	5M	10	2017	Year-1	0	0	Fall
10/21/2017	BL	6M	10	2017	Year-1	0	0	Fall
10/21/2017	BL	7M	10	2017	Year-1	0	0	Fall
10/21/2017	BL	10M	10	2017	Year-1	0	0	Fall
10/21/2017	BL	12M	10	2017	Year-1	0	0	Fall
10/21/2017	BL	14M	10	2017	Year-1		1	Fall
10/21/2017	BL	8A	10	2017	Year-1	0	0	Fall

Date	Species	Site	Month	Year	Survey Year	Presence	Data Gap	Season
10/21/2017	BL	9A	10	2017	Year-1	0	0	Fall
10/21/2017	BL	11A	10	2017	Year-1	0	0	Fall
10/21/2017	BL	13A	10	2017	Year-1	0	0	Fall
10/22/2017	BL	1M	10	2017	Year-1	0	0	Fall
10/22/2017	BL	2M	10	2017	Year-1	0	0	Fall
10/22/2017	BL	3M	10	2017	Year-1	0	0	Fall
10/22/2017	BL	4M	10	2017	Year-1	0	0	Fall
10/22/2017	BL	5M	10	2017	Year-1	0	0	Fall
10/22/2017	BL	6M	10	2017	Year-1	0	0	Fall
10/22/2017	BL	7M	10	2017	Year-1	0	0	Fall
10/22/2017	BL	10M	10	2017	Year-1	0	0	Fall
10/22/2017	BL	12M	10	2017	Year-1	0	0	Fall
10/22/2017	BL	14M	10	2017	Year-1		1	Fall
10/22/2017	BL	8A	10	2017	Year-1	0	0	Fall
10/22/2017	BL	9A	10	2017	Year-1	0	0	Fall
10/22/2017	BL	11A	10	2017	Year-1	0	0	Fall
10/22/2017	BL	13A	10	2017	Year-1	0	0	Fall
10/23/2017	BL	1M	10	2017	Year-1	0	0	Fall
10/23/2017	BL	2M	10	2017	Year-1	0	0	Fall
10/23/2017	BL	3M	10	2017	Year-1	0	0	Fall
10/23/2017	BL	4M	10	2017	Year-1	0	0	Fall
10/23/2017	BL	5M	10	2017	Year-1	0	0	Fall
10/23/2017	BL	6M	10	2017	Year-1	0	0	Fall
10/23/2017	BL	7M	10	2017	Year-1	0	0	Fall
10/23/2017	BL	10M	10	2017	Year-1	0	0	Fall
10/23/2017	BL	12M	10	2017	Year-1	0	0	Fall
10/23/2017	BL	14M	10	2017	Year-1		1	Fall
10/23/2017	BL	8A	10	2017	Year-1	0	0	Fall
10/23/2017	BL	9A	10	2017	Year-1	0	0	Fall
10/23/2017	BL	11A	10	2017	Year-1	0	0	Fall
10/23/2017	BL	13A	10	2017	Year-1	0	0	Fall
10/24/2017	BL	1M	10	2017	Year-1	0	0	Fall
10/24/2017	BL	2M	10	2017	Year-1	0	0	Fall
10/24/2017	BL	3M	10	2017	Year-1	0	0	Fall
10/24/2017	BL	4M	10	2017	Year-1	0	0	Fall
10/24/2017	BL	5M	10	2017	Year-1	0	0	Fall
10/24/2017	BL	6M	10	2017	Year-1	0	0	Fall
10/24/2017	BL	7M	10	2017	Year-1	0	0	Fall
10/24/2017	BL	10M	10	2017	Year-1	0	0	Fall
10/24/2017	BL	12M	10	2017	Year-1	0	0	Fall
10/24/2017	BL	14M	10	2017	Year-1		1	Fall
10/24/2017	BL	8A	10	2017	Year-1	0	0	Fall
10/24/2017	BL	9A	10	2017	Year-1	0	0	Fall
10/24/2017	BL	11A	10	2017	Year-1	0	0	Fall

Date	Species	Site	Month	Year	Survey Year	Presence	Data Gap	Season
10/24/2017	BL	13A	10	2017	Year-1	0	0	Fall
10/25/2017	BL	1M	10	2017	Year-1	0	0	Fall
10/25/2017	BL	2M	10	2017	Year-1	0	0	Fall
10/25/2017	BL	3M	10	2017	Year-1	0	0	Fall
10/25/2017	BL	4M	10	2017	Year-1	0	0	Fall
10/25/2017	BL	5M	10	2017	Year-1	0	0	Fall
10/25/2017	BL	6M	10	2017	Year-1	0	0	Fall
10/25/2017	BL	7M	10	2017	Year-1	0	0	Fall
10/25/2017	BL	10M	10	2017	Year-1	0	0	Fall
10/25/2017	BL	12M	10	2017	Year-1	0	0	Fall
10/25/2017	BL	14M	10	2017	Year-1		1	Fall
10/25/2017	BL	8A	10	2017	Year-1	0	0	Fall
10/25/2017	BL	9A	10	2017	Year-1	0	0	Fall
10/25/2017	BL	11A	10	2017	Year-1	0	0	Fall
10/25/2017	BL	13A	10	2017	Year-1	0	0	Fall
10/26/2017	BL	1M	10	2017	Year-1	0	0	Fall
10/26/2017	BL	2M	10	2017	Year-1	0	0	Fall
10/26/2017	BL	3M	10	2017	Year-1	0	0	Fall
10/26/2017	BL	4M	10	2017	Year-1	0	0	Fall
10/26/2017	BL	5M	10	2017	Year-1	0	0	Fall
10/26/2017	BL	6M	10	2017	Year-1	0	0	Fall
10/26/2017	BL	7M	10	2017	Year-1	0	0	Fall
10/26/2017	BL	10M	10	2017	Year-1	0	0	Fall
10/26/2017	BL	12M	10	2017	Year-1	0	0	Fall
10/26/2017	BL	14M	10	2017	Year-1		1	Fall
10/26/2017	BL	8A	10	2017	Year-1	0	0	Fall
10/26/2017	BL	9A	10	2017	Year-1	0	0	Fall
10/26/2017	BL	11A	10	2017	Year-1	0	0	Fall
10/26/2017	BL	13A	10	2017	Year-1	0	0	Fall
10/27/2017	BL	1M	10	2017	Year-1	0	0	Fall
10/27/2017	BL	2M	10	2017	Year-1	0	0	Fall
10/27/2017	BL	3M	10	2017	Year-1	0	0	Fall
10/27/2017	BL	4M	10	2017	Year-1	0	0	Fall
10/27/2017	BL	5M	10	2017	Year-1	0	0	Fall
10/27/2017	BL	6M	10	2017	Year-1	0	0	Fall
10/27/2017	BL	7M	10	2017	Year-1	0	0	Fall
10/27/2017	BL	10M	10	2017	Year-1	0	0	Fall
10/27/2017	BL	12M	10	2017	Year-1	0	0	Fall
10/27/2017	BL	14M	10	2017	Year-1		1	Fall
10/27/2017	BL	8A	10	2017	Year-1	0	0	Fall
10/27/2017	BL	9A	10	2017	Year-1	0	0	Fall
10/27/2017	BL	11A	10	2017	Year-1	0	0	Fall
10/27/2017	BL	13A	10	2017	Year-1	0	0	Fall
10/28/2017	BL	1M	10	2017	Year-1	0	0	Fall

Date	Species	Site	Month	Year	Survey Year	Presence	Data Gap	Season
10/28/2017	BL	2M	10	2017	Year-1	0	0	Fall
10/28/2017	BL	3M	10	2017	Year-1	0	0	Fall
10/28/2017	BL	4M	10	2017	Year-1	0	0	Fall
10/28/2017	BL	5M	10	2017	Year-1	0	0	Fall
10/28/2017	BL	6M	10	2017	Year-1	0	0	Fall
10/28/2017	BL	7M	10	2017	Year-1	0	0	Fall
10/28/2017	BL	10M	10	2017	Year-1	0	0	Fall
10/28/2017	BL	12M	10	2017	Year-1	0	0	Fall
10/28/2017	BL	14M	10	2017	Year-1		1	Fall
10/28/2017	BL	8A	10	2017	Year-1	0	0	Fall
10/28/2017	BL	9A	10	2017	Year-1	0	0	Fall
10/28/2017	BL	11A	10	2017	Year-1	0	0	Fall
10/28/2017	BL	13A	10	2017	Year-1	0	0	Fall
10/29/2017	BL	1M	10	2017	Year-1	0	0	Fall
10/29/2017	BL	2M	10	2017	Year-1	0	0	Fall
10/29/2017	BL	3M	10	2017	Year-1	0	0	Fall
10/29/2017	BL	4M	10	2017	Year-1	0	0	Fall
10/29/2017	BL	5M	10	2017	Year-1	0	0	Fall
10/29/2017	BL	6M	10	2017	Year-1	0	0	Fall
10/29/2017	BL	7M	10	2017	Year-1	0	0	Fall
10/29/2017	BL	10M	10	2017	Year-1	0	0	Fall
10/29/2017	BL	12M	10	2017	Year-1	0	0	Fall
10/29/2017	BL	14M	10	2017	Year-1		1	Fall
10/29/2017	BL	8A	10	2017	Year-1	0	0	Fall
10/29/2017	BL	9A	10	2017	Year-1	0	0	Fall
10/29/2017	BL	11A	10	2017	Year-1	0	0	Fall
10/29/2017	BL	13A	10	2017	Year-1	0	0	Fall
10/30/2017	BL	1M	10	2017	Year-1	0	0	Fall
10/30/2017	BL	2M	10	2017	Year-1	0	0	Fall
10/30/2017	BL	3M	10	2017	Year-1	0	0	Fall
10/30/2017	BL	4M	10	2017	Year-1	0	0	Fall
10/30/2017	BL	5M	10	2017	Year-1	0	0	Fall
10/30/2017	BL	6M	10	2017	Year-1	0	0	Fall
10/30/2017	BL	7M	10	2017	Year-1	0	0	Fall
10/30/2017	BL	10M	10	2017	Year-1	0	0	Fall
10/30/2017	BL	12M	10	2017	Year-1	0	0	Fall
10/30/2017	BL	14M	10	2017	Year-1		1	Fall
10/30/2017	BL	8A	10	2017	Year-1	0	0	Fall
10/30/2017	BL	9A	10	2017	Year-1	0	0	Fall
10/30/2017	BL	11A	10	2017	Year-1	0	0	Fall
10/30/2017	BL	13A	10	2017	Year-1	0	0	Fall
10/31/2017	BL	1M	10	2017	Year-1	0	0	Fall
10/31/2017	BL	2M	10	2017	Year-1	0	0	Fall
10/31/2017	BL	3M	10	2017	Year-1	0	0	Fall

Date	Species	Site	Month	Year	Survey Year	Presence	Data Gap	Season
10/31/2017	BL	4M	10	2017	Year-1	0	0	Fall
10/31/2017	BL	5M	10	2017	Year-1	0	0	Fall
10/31/2017	BL	6M	10	2017	Year-1	0	0	Fall
10/31/2017	BL	7M	10	2017	Year-1	0	0	Fall
10/31/2017	BL	10M	10	2017	Year-1	0	0	Fall
10/31/2017	BL	12M	10	2017	Year-1	0	0	Fall
10/31/2017	BL	14M	10	2017	Year-1		1	Fall
10/31/2017	BL	8A	10	2017	Year-1	0	0	Fall
10/31/2017	BL	9A	10	2017	Year-1	0	0	Fall
10/31/2017	BL	11A	10	2017	Year-1	0	0	Fall
10/31/2017	BL	13A	10	2017	Year-1	0	0	Fall
11/1/2017	BL	1M	11	2017	Year-1	1	0	Fall
11/1/2017	BL	2M	11	2017	Year-1	1	0	Fall
11/1/2017	BL	3M	11	2017	Year-1	0	0	Fall
11/1/2017	BL	4M	11	2017	Year-1	0	0	Fall
11/1/2017	BL	5M	11	2017	Year-1	0	0	Fall
11/1/2017	BL	6M	11	2017	Year-1	0	0	Fall
11/1/2017	BL	7M	11	2017	Year-1	0	0	Fall
11/1/2017	BL	10M	11	2017	Year-1	0	0	Fall
11/1/2017	BL	12M	11	2017	Year-1	0	0	Fall
11/1/2017	BL	14M	11	2017	Year-1		1	Fall
11/1/2017	BL	8A	11	2017	Year-1	0	0	Fall
11/1/2017	BL	9A	11	2017	Year-1	0	0	Fall
11/1/2017	BL	11A	11	2017	Year-1	0	0	Fall
11/1/2017	BL	13A	11	2017	Year-1	0	0	Fall
11/2/2017	BL	1M	11	2017	Year-1	1	0	Fall
11/2/2017	BL	2M	11	2017	Year-1	1	0	Fall
11/2/2017	BL	3M	11	2017	Year-1	1	0	Fall
11/2/2017	BL	4M	11	2017	Year-1	1	0	Fall
11/2/2017	BL	5M	11	2017	Year-1	0	0	Fall
11/2/2017	BL	6M	11	2017	Year-1	0	0	Fall
11/2/2017	BL	7M	11	2017	Year-1	0	0	Fall
11/2/2017	BL	10M	11	2017	Year-1	0	0	Fall
11/2/2017	BL	12M	11	2017	Year-1	0	0	Fall
11/2/2017	BL	14M	11	2017	Year-1		1	Fall
11/2/2017	BL	8A	11	2017	Year-1	0	0	Fall
11/2/2017	BL	9A	11	2017	Year-1	0	0	Fall
11/2/2017	BL	11A	11	2017	Year-1	0	0	Fall
11/2/2017	BL	13A	11	2017	Year-1	0	0	Fall
11/3/2017	BL	1M	11	2017	Year-1	0	0	Fall
11/3/2017	BL	2M	11	2017	Year-1	1	0	Fall
11/3/2017	BL	3M	11	2017	Year-1	1	0	Fall
11/3/2017	BL	4M	11	2017	Year-1	1	0	Fall
11/3/2017	BL	5M	11	2017	Year-1	0	0	Fall

Date	Species	Site	Month	Year	Survey Year	Presence	Data Gap	Season
11/3/2017	BL	6M	11	2017	Year-1	0	0	Fall
11/3/2017	BL	7M	11	2017	Year-1	0	0	Fall
11/3/2017	BL	10M	11	2017	Year-1	0	0	Fall
11/3/2017	BL	12M	11	2017	Year-1	0	0	Fall
11/3/2017	BL	14M	11	2017	Year-1		1	Fall
11/3/2017	BL	8A	11	2017	Year-1	0	0	Fall
11/3/2017	BL	9A	11	2017	Year-1	0	0	Fall
11/3/2017	BL	11A	11	2017	Year-1	0	0	Fall
11/3/2017	BL	13A	11	2017	Year-1	0	0	Fall
11/4/2017	BL	1M	11	2017	Year-1	0	0	Fall
11/4/2017	BL	2M	11	2017	Year-1	0	0	Fall
11/4/2017	BL	3M	11	2017	Year-1	0	0	Fall
11/4/2017	BL	4M	11	2017	Year-1	0	0	Fall
11/4/2017	BL	5M	11	2017	Year-1	0	0	Fall
11/4/2017	BL	6M	11	2017	Year-1	0	0	Fall
11/4/2017	BL	7M	11	2017	Year-1	0	0	Fall
11/4/2017	BL	10M	11	2017	Year-1	0	0	Fall
11/4/2017	BL	12M	11	2017	Year-1	0	0	Fall
11/4/2017	BL	14M	11	2017	Year-1		1	Fall
11/4/2017	BL	8A	11	2017	Year-1	0	0	Fall
11/4/2017	BL	9A	11	2017	Year-1	0	0	Fall
11/4/2017	BL	11A	11	2017	Year-1	0	0	Fall
11/4/2017	BL	13A	11	2017	Year-1	0	0	Fall
11/5/2017	BL	1M	11	2017	Year-1	0	0	Fall
11/5/2017	BL	2M	11	2017	Year-1	0	0	Fall
11/5/2017	BL	3M	11	2017	Year-1	0	0	Fall
11/5/2017	BL	4M	11	2017	Year-1	0	0	Fall
11/5/2017	BL	5M	11	2017	Year-1	0	0	Fall
11/5/2017	BL	6M	11	2017	Year-1	0	0	Fall
11/5/2017	BL	7M	11	2017	Year-1	0	0	Fall
11/5/2017	BL	10M	11	2017	Year-1	0	0	Fall
11/5/2017	BL	12M	11	2017	Year-1	0	0	Fall
11/5/2017	BL	14M	11	2017	Year-1		1	Fall
11/5/2017	BL	8A	11	2017	Year-1	0	0	Fall
11/5/2017	BL	9A	11	2017	Year-1	0	0	Fall
11/5/2017	BL	11A	11	2017	Year-1	0	0	Fall
11/5/2017	BL	13A	11	2017	Year-1	0	0	Fall
11/6/2017	BL	1M	11	2017	Year-1	0	0	Fall
11/6/2017	BL	2M	11	2017	Year-1	0	0	Fall
11/6/2017	BL	3M	11	2017	Year-1	0	0	Fall
11/6/2017	BL	4M	11	2017	Year-1	0	0	Fall
11/6/2017	BL	5M	11	2017	Year-1	0	0	Fall
11/6/2017	BL	6M	11	2017	Year-1	0	0	Fall
11/6/2017	BL	7M	11	2017	Year-1	0	0	Fall

Date	Species	Site	Month	Year	Survey Year	Presence	Data Gap	Season
11/6/2017	BL	10M	11	2017	Year-1	0	0	Fall
11/6/2017	BL	12M	11	2017	Year-1	0	0	Fall
11/6/2017	BL	14M	11	2017	Year-1		1	Fall
11/6/2017	BL	8A	11	2017	Year-1	0	0	Fall
11/6/2017	BL	9A	11	2017	Year-1	0	0	Fall
11/6/2017	BL	11A	11	2017	Year-1	0	0	Fall
11/6/2017	BL	13A	11	2017	Year-1	0	0	Fall
11/7/2017	BL	1M	11	2017	Year-1	0	0	Fall
11/7/2017	BL	2M	11	2017	Year-1	0	0	Fall
11/7/2017	BL	3M	11	2017	Year-1	0	0	Fall
11/7/2017	BL	4M	11	2017	Year-1	0	0	Fall
11/7/2017	BL	5M	11	2017	Year-1	0	0	Fall
11/7/2017	BL	6M	11	2017	Year-1	0	0	Fall
11/7/2017	BL	7M	11	2017	Year-1	0	0	Fall
11/7/2017	BL	10M	11	2017	Year-1	0	0	Fall
11/7/2017	BL	12M	11	2017	Year-1	0	0	Fall
11/7/2017	BL	14M	11	2017	Year-1		1	Fall
11/7/2017	BL	8A	11	2017	Year-1	0	0	Fall
11/7/2017	BL	9A	11	2017	Year-1	0	0	Fall
11/7/2017	BL	11A	11	2017	Year-1	0	0	Fall
11/7/2017	BL	13A	11	2017	Year-1	0	0	Fall
11/8/2017	BL	1M	11	2017	Year-1	0	0	Fall
11/8/2017	BL	2M	11	2017	Year-1	0	0	Fall
11/8/2017	BL	3M	11	2017	Year-1	0	0	Fall
11/8/2017	BL	4M	11	2017	Year-1	0	0	Fall
11/8/2017	BL	5M	11	2017	Year-1	0	0	Fall
11/8/2017	BL	6M	11	2017	Year-1	0	0	Fall
11/8/2017	BL	7M	11	2017	Year-1	0	0	Fall
11/8/2017	BL	10M	11	2017	Year-1	0	0	Fall
11/8/2017	BL	12M	11	2017	Year-1	0	0	Fall
11/8/2017	BL	14M	11	2017	Year-1		1	Fall
11/8/2017	BL	8A	11	2017	Year-1	0	0	Fall
11/8/2017	BL	9A	11	2017	Year-1	0	0	Fall
11/8/2017	BL	11A	11	2017	Year-1	0	0	Fall
11/8/2017	BL	13A	11	2017	Year-1	0	0	Fall
11/9/2017	BL	1M	11	2017	Year-1	0	0	Fall
11/9/2017	BL	2M	11	2017	Year-1	0	0	Fall
11/9/2017	BL	3M	11	2017	Year-1	0	0	Fall
11/9/2017	BL	4M	11	2017	Year-1	0	0	Fall
11/9/2017	BL	5M	11	2017	Year-1	0	0	Fall
11/9/2017	BL	6M	11	2017	Year-1	0	0	Fall
11/9/2017	BL	7M	11	2017	Year-1	0	0	Fall
11/9/2017	BL	10M	11	2017	Year-1	0	0	Fall
11/9/2017	BL	12M	11	2017	Year-1	0	0	Fall

Date	Species	Site	Month	Year	Survey Year	Presence	Data Gap	Season
11/9/2017	BL	14M	11	2017	Year-1		1	Fall
11/9/2017	BL	8A	11	2017	Year-1	0	0	Fall
11/9/2017	BL	9A	11	2017	Year-1	0	0	Fall
11/9/2017	BL	11A	11	2017	Year-1	0	0	Fall
11/9/2017	BL	13A	11	2017	Year-1	0	0	Fall
11/10/2017	BL	1M	11	2017	Year-1	0	0	Fall
11/10/2017	BL	2M	11	2017	Year-1	0	0	Fall
11/10/2017	BL	3M	11	2017	Year-1	0	0	Fall
11/10/2017	BL	4M	11	2017	Year-1	0	0	Fall
11/10/2017	BL	5M	11	2017	Year-1	0	0	Fall
11/10/2017	BL	6M	11	2017	Year-1	0	0	Fall
11/10/2017	BL	7M	11	2017	Year-1	0	0	Fall
11/10/2017	BL	10M	11	2017	Year-1	0	0	Fall
11/10/2017	BL	12M	11	2017	Year-1	0	0	Fall
11/10/2017	BL	14M	11	2017	Year-1		1	Fall
11/10/2017	BL	8A	11	2017	Year-1	0	0	Fall
11/10/2017	BL	9A	11	2017	Year-1	0	0	Fall
11/10/2017	BL	11A	11	2017	Year-1	0	0	Fall
11/10/2017	BL	13A	11	2017	Year-1	0	0	Fall
11/11/2017	BL	1M	11	2017	Year-1	0	0	Fall
11/11/2017	BL	2M	11	2017	Year-1	0	0	Fall
11/11/2017	BL	3M	11	2017	Year-1	0	0	Fall
11/11/2017	BL	4M	11	2017	Year-1	0	0	Fall
11/11/2017	BL	5M	11	2017	Year-1	0	0	Fall
11/11/2017	BL	6M	11	2017	Year-1	0	0	Fall
11/11/2017	BL	7M	11	2017	Year-1	0	0	Fall
11/11/2017	BL	10M	11	2017	Year-1	0	0	Fall
11/11/2017	BL	12M	11	2017	Year-1	0	0	Fall
11/11/2017	BL	14M	11	2017	Year-1		1	Fall
11/11/2017	BL	8A	11	2017	Year-1	0	0	Fall
11/11/2017	BL	9A	11	2017	Year-1	0	0	Fall
11/11/2017	BL	11A	11	2017	Year-1	0	0	Fall
11/11/2017	BL	13A	11	2017	Year-1	0	0	Fall
11/12/2017	BL	1M	11	2017	Year-1	0	0	Fall
11/12/2017	BL	2M	11	2017	Year-1	0	0	Fall
11/12/2017	BL	3M	11	2017	Year-1	0	0	Fall
11/12/2017	BL	4M	11	2017	Year-1	0	0	Fall
11/12/2017	BL	5M	11	2017	Year-1	0	0	Fall
11/12/2017	BL	6M	11	2017	Year-1	0	0	Fall
11/12/2017	BL	7M	11	2017	Year-1	0	0	Fall
11/12/2017	BL	10M	11	2017	Year-1	0	0	Fall
11/12/2017	BL	12M	11	2017	Year-1	0	0	Fall
11/12/2017	BL	14M	11	2017	Year-1		1	Fall
11/12/2017	BL	8A	11	2017	Year-1	0	0	Fall

Date	Species	Site	Month	Year	Survey Year	Presence	Data Gap	Season
11/12/2017	BL	9A	11	2017	Year-1	0	0	Fall
11/12/2017	BL	11A	11	2017	Year-1	0	0	Fall
11/12/2017	BL	13A	11	2017	Year-1	0	0	Fall
11/13/2017	BL	1M	11	2017	Year-1	0	0	Fall
11/13/2017	BL	2M	11	2017	Year-1	0	0	Fall
11/13/2017	BL	3M	11	2017	Year-1	0	0	Fall
11/13/2017	BL	4M	11	2017	Year-1	0	0	Fall
11/13/2017	BL	5M	11	2017	Year-1	0	0	Fall
11/13/2017	BL	6M	11	2017	Year-1	0	0	Fall
11/13/2017	BL	7M	11	2017	Year-1	0	0	Fall
11/13/2017	BL	10M	11	2017	Year-1	0	0	Fall
11/13/2017	BL	12M	11	2017	Year-1	0	0	Fall
11/13/2017	BL	14M	11	2017	Year-1		1	Fall
11/13/2017	BL	8A	11	2017	Year-1	0	0	Fall
11/13/2017	BL	9A	11	2017	Year-1	0	0	Fall
11/13/2017	BL	11A	11	2017	Year-1	0	0	Fall
11/13/2017	BL	13A	11	2017	Year-1	0	0	Fall
11/14/2017	BL	1M	11	2017	Year-1	0	0	Fall
11/14/2017	BL	2M	11	2017	Year-1	0	0	Fall
11/14/2017	BL	3M	11	2017	Year-1	0	0	Fall
11/14/2017	BL	4M	11	2017	Year-1	0	0	Fall
11/14/2017	BL	5M	11	2017	Year-1	0	0	Fall
11/14/2017	BL	6M	11	2017	Year-1	0	0	Fall
11/14/2017	BL	7M	11	2017	Year-1	0	0	Fall
11/14/2017	BL	10M	11	2017	Year-1	0	0	Fall
11/14/2017	BL	12M	11	2017	Year-1	0	0	Fall
11/14/2017	BL	14M	11	2017	Year-1		1	Fall
11/14/2017	BL	8A	11	2017	Year-1	0	0	Fall
11/14/2017	BL	9A	11	2017	Year-1	0	0	Fall
11/14/2017	BL	11A	11	2017	Year-1	0	0	Fall
11/14/2017	BL	13A	11	2017	Year-1	0	0	Fall
11/15/2017	BL	1M	11	2017	Year-1	0	0	Fall
11/15/2017	BL	2M	11	2017	Year-1	0	0	Fall
11/15/2017	BL	3M	11	2017	Year-1	0	0	Fall
11/15/2017	BL	4M	11	2017	Year-1	0	0	Fall
11/15/2017	BL	5M	11	2017	Year-1	0	0	Fall
11/15/2017	BL	6M	11	2017	Year-1	0	0	Fall
11/15/2017	BL	7M	11	2017	Year-1	0	0	Fall
11/15/2017	BL	10M	11	2017	Year-1	0	0	Fall
11/15/2017	BL	12M	11	2017	Year-1	0	0	Fall
11/15/2017	BL	14M	11	2017	Year-1		1	Fall
11/15/2017	BL	8A	11	2017	Year-1	0	0	Fall
11/15/2017	BL	9A	11	2017	Year-1	0	0	Fall
11/15/2017	BL	11A	11	2017	Year-1	0	0	Fall

Date	Species	Site	Month	Year	Survey Year	Presence	Data Gap	Season
11/15/2017	BL	13A	11	2017	Year-1	0	0	Fall
11/16/2017	BL	1M	11	2017	Year-1	0	0	Fall
11/16/2017	BL	2M	11	2017	Year-1	0	0	Fall
11/16/2017	BL	3M	11	2017	Year-1	0	0	Fall
11/16/2017	BL	4M	11	2017	Year-1	0	0	Fall
11/16/2017	BL	5M	11	2017	Year-1	0	0	Fall
11/16/2017	BL	6M	11	2017	Year-1	0	0	Fall
11/16/2017	BL	7M	11	2017	Year-1	0	0	Fall
11/16/2017	BL	10M	11	2017	Year-1	0	0	Fall
11/16/2017	BL	12M	11	2017	Year-1	0	0	Fall
11/16/2017	BL	14M	11	2017	Year-1		1	Fall
11/16/2017	BL	8A	11	2017	Year-1	0	0	Fall
11/16/2017	BL	9A	11	2017	Year-1	0	0	Fall
11/16/2017	BL	11A	11	2017	Year-1	0	0	Fall
11/16/2017	BL	13A	11	2017	Year-1	0	0	Fall
11/17/2017	BL	1M	11	2017	Year-1	0	0	Fall
11/17/2017	BL	2M	11	2017	Year-1	0	0	Fall
11/17/2017	BL	3M	11	2017	Year-1	0	0	Fall
11/17/2017	BL	4M	11	2017	Year-1	0	0	Fall
11/17/2017	BL	5M	11	2017	Year-1	0	0	Fall
11/17/2017	BL	6M	11	2017	Year-1	0	0	Fall
11/17/2017	BL	7M	11	2017	Year-1	0	0	Fall
11/17/2017	BL	10M	11	2017	Year-1	0	0	Fall
11/17/2017	BL	12M	11	2017	Year-1	0	0	Fall
11/17/2017	BL	14M	11	2017	Year-1		1	Fall
11/17/2017	BL	8A	11	2017	Year-1	0	0	Fall
11/17/2017	BL	9A	11	2017	Year-1	0	0	Fall
11/17/2017	BL	11A	11	2017	Year-1	0	0	Fall
11/17/2017	BL	13A	11	2017	Year-1	0	0	Fall
11/18/2017	BL	1M	11	2017	Year-1	0	0	Fall
11/18/2017	BL	2M	11	2017	Year-1	0	0	Fall
11/18/2017	BL	3M	11	2017	Year-1	0	0	Fall
11/18/2017	BL	4M	11	2017	Year-1	0	0	Fall
11/18/2017	BL	5M	11	2017	Year-1	0	0	Fall
11/18/2017	BL	6M	11	2017	Year-1	0	0	Fall
11/18/2017	BL	7M	11	2017	Year-1	0	0	Fall
11/18/2017	BL	10M	11	2017	Year-1	0	0	Fall
11/18/2017	BL	12M	11	2017	Year-1	0	0	Fall
11/18/2017	BL	14M	11	2017	Year-1		1	Fall
11/18/2017	BL	8A	11	2017	Year-1	0	0	Fall
11/18/2017	BL	9A	11	2017	Year-1	0	0	Fall
11/18/2017	BL	11A	11	2017	Year-1	0	0	Fall
11/18/2017	BL	13A	11	2017	Year-1	0	0	Fall
11/19/2017	BL	1M	11	2017	Year-1	0	0	Fall

Date	Species	Site	Month	Year	Survey Year	Presence	Data Gap	Season
11/19/2017	BL	2M	11	2017	Year-1	0	0	Fall
11/19/2017	BL	3M	11	2017	Year-1	0	0	Fall
11/19/2017	BL	4M	11	2017	Year-1	0	0	Fall
11/19/2017	BL	5M	11	2017	Year-1	0	0	Fall
11/19/2017	BL	6M	11	2017	Year-1	0	0	Fall
11/19/2017	BL	7M	11	2017	Year-1	0	0	Fall
11/19/2017	BL	10M	11	2017	Year-1	0	0	Fall
11/19/2017	BL	12M	11	2017	Year-1	0	0	Fall
11/19/2017	BL	14M	11	2017	Year-1		1	Fall
11/19/2017	BL	8A	11	2017	Year-1	0	0	Fall
11/19/2017	BL	9A	11	2017	Year-1	0	0	Fall
11/19/2017	BL	11A	11	2017	Year-1	0	0	Fall
11/19/2017	BL	13A	11	2017	Year-1	0	0	Fall
11/20/2017	BL	1M	11	2017	Year-1	0	0	Fall
11/20/2017	BL	2M	11	2017	Year-1	0	0	Fall
11/20/2017	BL	3M	11	2017	Year-1	0	0	Fall
11/20/2017	BL	4M	11	2017	Year-1	0	0	Fall
11/20/2017	BL	5M	11	2017	Year-1	0	0	Fall
11/20/2017	BL	6M	11	2017	Year-1	0	0	Fall
11/20/2017	BL	7M	11	2017	Year-1	0	0	Fall
11/20/2017	BL	10M	11	2017	Year-1	0	0	Fall
11/20/2017	BL	12M	11	2017	Year-1	0	0	Fall
11/20/2017	BL	14M	11	2017	Year-1		1	Fall
11/20/2017	BL	8A	11	2017	Year-1	0	0	Fall
11/20/2017	BL	9A	11	2017	Year-1	0	0	Fall
11/20/2017	BL	11A	11	2017	Year-1	0	0	Fall
11/20/2017	BL	13A	11	2017	Year-1	0	0	Fall
11/21/2017	BL	1M	11	2017	Year-1	0	0	Fall
11/21/2017	BL	2M	11	2017	Year-1	0	0	Fall
11/21/2017	BL	3M	11	2017	Year-1	0	0	Fall
11/21/2017	BL	4M	11	2017	Year-1	0	0	Fall
11/21/2017	BL	5M	11	2017	Year-1	0	0	Fall
11/21/2017	BL	6M	11	2017	Year-1	0	0	Fall
11/21/2017	BL	7M	11	2017	Year-1	0	0	Fall
11/21/2017	BL	10M	11	2017	Year-1	0	0	Fall
11/21/2017	BL	12M	11	2017	Year-1	0	0	Fall
11/21/2017	BL	14M	11	2017	Year-1		1	Fall
11/21/2017	BL	8A	11	2017	Year-1	0	0	Fall
11/21/2017	BL	9A	11	2017	Year-1	0	0	Fall
11/21/2017	BL	11A	11	2017	Year-1	0	0	Fall
11/21/2017	BL	13A	11	2017	Year-1	0	0	Fall
11/22/2017	BL	1M	11	2017	Year-1	0	0	Fall
11/22/2017	BL	2M	11	2017	Year-1	0	0	Fall
11/22/2017	BL	3M	11	2017	Year-1	0	0	Fall

Date	Species	Site	Month	Year	Survey Year	Presence	Data Gap	Season
11/22/2017	BL	4M	11	2017	Year-1	0	0	Fall
11/22/2017	BL	5M	11	2017	Year-1	0	0	Fall
11/22/2017	BL	6M	11	2017	Year-1	0	0	Fall
11/22/2017	BL	7M	11	2017	Year-1	0	0	Fall
11/22/2017	BL	10M	11	2017	Year-1	0	0	Fall
11/22/2017	BL	12M	11	2017	Year-1	0	0	Fall
11/22/2017	BL	14M	11	2017	Year-1		1	Fall
11/22/2017	BL	8A	11	2017	Year-1	0	0	Fall
11/22/2017	BL	9A	11	2017	Year-1	0	0	Fall
11/22/2017	BL	11A	11	2017	Year-1	0	0	Fall
11/22/2017	BL	13A	11	2017	Year-1	0	0	Fall
11/23/2017	BL	1M	11	2017	Year-1	0	0	Fall
11/23/2017	BL	2M	11	2017	Year-1	0	0	Fall
11/23/2017	BL	3M	11	2017	Year-1	0	0	Fall
11/23/2017	BL	4M	11	2017	Year-1	0	0	Fall
11/23/2017	BL	5M	11	2017	Year-1	0	0	Fall
11/23/2017	BL	6M	11	2017	Year-1	0	0	Fall
11/23/2017	BL	7M	11	2017	Year-1	0	0	Fall
11/23/2017	BL	10M	11	2017	Year-1	0	0	Fall
11/23/2017	BL	12M	11	2017	Year-1	0	0	Fall
11/23/2017	BL	14M	11	2017	Year-1		1	Fall
11/23/2017	BL	8A	11	2017	Year-1	0	0	Fall
11/23/2017	BL	9A	11	2017	Year-1	0	0	Fall
11/23/2017	BL	11A	11	2017	Year-1	0	0	Fall
11/23/2017	BL	13A	11	2017	Year-1	0	0	Fall
11/24/2017	BL	1M	11	2017	Year-1	0	0	Fall
11/24/2017	BL	2M	11	2017	Year-1	0	0	Fall
11/24/2017	BL	3M	11	2017	Year-1	0	0	Fall
11/24/2017	BL	4M	11	2017	Year-1	0	0	Fall
11/24/2017	BL	5M	11	2017	Year-1	0	0	Fall
11/24/2017	BL	6M	11	2017	Year-1	0	0	Fall
11/24/2017	BL	7M	11	2017	Year-1	0	0	Fall
11/24/2017	BL	10M	11	2017	Year-1	0	0	Fall
11/24/2017	BL	12M	11	2017	Year-1	0	0	Fall
11/24/2017	BL	14M	11	2017	Year-1		1	Fall
11/24/2017	BL	8A	11	2017	Year-1	0	0	Fall
11/24/2017	BL	9A	11	2017	Year-1	0	0	Fall
11/24/2017	BL	11A	11	2017	Year-1	0	0	Fall
11/24/2017	BL	13A	11	2017	Year-1	0	0	Fall
11/25/2017	BL	1M	11	2017	Year-1	0	0	Fall
11/25/2017	BL	2M	11	2017	Year-1	0	0	Fall
11/25/2017	BL	3M	11	2017	Year-1	0	0	Fall
11/25/2017	BL	4M	11	2017	Year-1	0	0	Fall
11/25/2017	BL	5M	11	2017	Year-1	0	0	Fall

Date	Species	Site	Month	Year	Survey Year	Presence	Data Gap	Season
11/25/2017	BL	6M	11	2017	Year-1	0	0	Fall
11/25/2017	BL	7M	11	2017	Year-1	0	0	Fall
11/25/2017	BL	10M	11	2017	Year-1	0	0	Fall
11/25/2017	BL	12M	11	2017	Year-1	0	0	Fall
11/25/2017	BL	14M	11	2017	Year-1		1	Fall
11/25/2017	BL	8A	11	2017	Year-1	0	0	Fall
11/25/2017	BL	9A	11	2017	Year-1	0	0	Fall
11/25/2017	BL	11A	11	2017	Year-1	0	0	Fall
11/25/2017	BL	13A	11	2017	Year-1	0	0	Fall
11/26/2017	BL	1M	11	2017	Year-1	0	0	Fall
11/26/2017	BL	2M	11	2017	Year-1	0	0	Fall
11/26/2017	BL	3M	11	2017	Year-1	0	0	Fall
11/26/2017	BL	4M	11	2017	Year-1	0	0	Fall
11/26/2017	BL	5M	11	2017	Year-1	0	0	Fall
11/26/2017	BL	6M	11	2017	Year-1	0	0	Fall
11/26/2017	BL	7M	11	2017	Year-1	0	0	Fall
11/26/2017	BL	10M	11	2017	Year-1	0	0	Fall
11/26/2017	BL	12M	11	2017	Year-1	0	0	Fall
11/26/2017	BL	14M	11	2017	Year-1		1	Fall
11/26/2017	BL	8A	11	2017	Year-1	0	0	Fall
11/26/2017	BL	9A	11	2017	Year-1	0	0	Fall
11/26/2017	BL	11A	11	2017	Year-1	0	0	Fall
11/26/2017	BL	13A	11	2017	Year-1	0	0	Fall
11/27/2017	BL	1M	11	2017	Year-1	0	0	Fall
11/27/2017	BL	2M	11	2017	Year-1	0	0	Fall
11/27/2017	BL	3M	11	2017	Year-1	0	0	Fall
11/27/2017	BL	4M	11	2017	Year-1	0	0	Fall
11/27/2017	BL	5M	11	2017	Year-1	0	0	Fall
11/27/2017	BL	6M	11	2017	Year-1	0	0	Fall
11/27/2017	BL	7M	11	2017	Year-1	0	0	Fall
11/27/2017	BL	10M	11	2017	Year-1	0	0	Fall
11/27/2017	BL	12M	11	2017	Year-1	0	0	Fall
11/27/2017	BL	14M	11	2017	Year-1		1	Fall
11/27/2017	BL	8A	11	2017	Year-1	0	0	Fall
11/27/2017	BL	9A	11	2017	Year-1	0	0	Fall
11/27/2017	BL	11A	11	2017	Year-1	0	0	Fall
11/27/2017	BL	13A	11	2017	Year-1	0	0	Fall
11/28/2017	BL	1M	11	2017	Year-1	0	0	Fall
11/28/2017	BL	2M	11	2017	Year-1	0	0	Fall
11/28/2017	BL	3M	11	2017	Year-1	0	0	Fall
11/28/2017	BL	4M	11	2017	Year-1	0	0	Fall
11/28/2017	BL	5M	11	2017	Year-1	0	0	Fall
11/28/2017	BL	6M	11	2017	Year-1	0	0	Fall
11/28/2017	BL	7M	11	2017	Year-1	0	0	Fall

Date	Species	Site	Month	Year	Survey Year	Presence	Data Gap	Season
11/28/2017	BL	10M	11	2017	Year-1	0	0	Fall
11/28/2017	BL	12M	11	2017	Year-1	0	0	Fall
11/28/2017	BL	14M	11	2017	Year-1		1	Fall
11/28/2017	BL	8A	11	2017	Year-1	0	0	Fall
11/28/2017	BL	9A	11	2017	Year-1	0	0	Fall
11/28/2017	BL	11A	11	2017	Year-1	0	0	Fall
11/28/2017	BL	13A	11	2017	Year-1	0	0	Fall
11/29/2017	BL	1M	11	2017	Year-1	0	0	Fall
11/29/2017	BL	2M	11	2017	Year-1	0	0	Fall
11/29/2017	BL	3M	11	2017	Year-1	0	0	Fall
11/29/2017	BL	4M	11	2017	Year-1	0	0	Fall
11/29/2017	BL	5M	11	2017	Year-1	0	0	Fall
11/29/2017	BL	6M	11	2017	Year-1	0	0	Fall
11/29/2017	BL	7M	11	2017	Year-1	0	0	Fall
11/29/2017	BL	10M	11	2017	Year-1	0	0	Fall
11/29/2017	BL	12M	11	2017	Year-1	0	0	Fall
11/29/2017	BL	14M	11	2017	Year-1		1	Fall
11/29/2017	BL	8A	11	2017	Year-1	0	0	Fall
11/29/2017	BL	9A	11	2017	Year-1	0	0	Fall
11/29/2017	BL	11A	11	2017	Year-1	0	0	Fall
11/29/2017	BL	13A	11	2017	Year-1	0	0	Fall
11/30/2017	BL	1M	11	2017	Year-1	0	0	Fall
11/30/2017	BL	2M	11	2017	Year-1	0	0	Fall
11/30/2017	BL	3M	11	2017	Year-1	0	0	Fall
11/30/2017	BL	4M	11	2017	Year-1	0	0	Fall
11/30/2017	BL	5M	11	2017	Year-1	0	0	Fall
11/30/2017	BL	6M	11	2017	Year-1	0	0	Fall
11/30/2017	BL	7M	11	2017	Year-1	0	0	Fall
11/30/2017	BL	10M	11	2017	Year-1	0	0	Fall
11/30/2017	BL	12M	11	2017	Year-1	0	0	Fall
11/30/2017	BL	14M	11	2017	Year-1		1	Fall
11/30/2017	BL	8A	11	2017	Year-1	0	0	Fall
11/30/2017	BL	9A	11	2017	Year-1	0	0	Fall
11/30/2017	BL	11A	11	2017	Year-1	0	0	Fall
11/30/2017	BL	13A	11	2017	Year-1	0	0	Fall
12/1/2017	BL	1M	12	2017	Year-1	0	0	Winter
12/1/2017	BL	2M	12	2017	Year-1	0	0	Winter
12/1/2017	BL	3M	12	2017	Year-1	0	0	Winter
12/1/2017	BL	4M	12	2017	Year-1	0	0	Winter
12/1/2017	BL	5M	12	2017	Year-1	0	0	Winter
12/1/2017	BL	6M	12	2017	Year-1	0	0	Winter
12/1/2017	BL	7M	12	2017	Year-1	0	0	Winter
12/1/2017	BL	10M	12	2017	Year-1	0	0	Winter
12/1/2017	BL	12M	12	2017	Year-1	0	0	Winter

Date	Species	Site	Month	Year	Survey Year	Presence	Data Gap	Season
12/1/2017	BL	14M	12	2017	Year-1		1	Winter
12/1/2017	BL	8A	12	2017	Year-1	0	0	Winter
12/1/2017	BL	9A	12	2017	Year-1	0	0	Winter
12/1/2017	BL	11A	12	2017	Year-1	0	0	Winter
12/1/2017	BL	13A	12	2017	Year-1	0	0	Winter
12/2/2017	BL	1M	12	2017	Year-1	0	0	Winter
12/2/2017	BL	2M	12	2017	Year-1	0	0	Winter
12/2/2017	BL	3M	12	2017	Year-1	0	0	Winter
12/2/2017	BL	4M	12	2017	Year-1	0	0	Winter
12/2/2017	BL	5M	12	2017	Year-1	0	0	Winter
12/2/2017	BL	6M	12	2017	Year-1	0	0	Winter
12/2/2017	BL	7M	12	2017	Year-1	0	0	Winter
12/2/2017	BL	10M	12	2017	Year-1	0	0	Winter
12/2/2017	BL	12M	12	2017	Year-1	0	0	Winter
12/2/2017	BL	14M	12	2017	Year-1		1	Winter
12/2/2017	BL	8A	12	2017	Year-1	0	0	Winter
12/2/2017	BL	9A	12	2017	Year-1	0	0	Winter
12/2/2017	BL	11A	12	2017	Year-1	0	0	Winter
12/2/2017	BL	13A	12	2017	Year-1	0	0	Winter
12/3/2017	BL	1M	12	2017	Year-1	0	0	Winter
12/3/2017	BL	2M	12	2017	Year-1	0	0	Winter
12/3/2017	BL	3M	12	2017	Year-1	0	0	Winter
12/3/2017	BL	4M	12	2017	Year-1	0	0	Winter
12/3/2017	BL	5M	12	2017	Year-1	0	0	Winter
12/3/2017	BL	6M	12	2017	Year-1	0	0	Winter
12/3/2017	BL	7M	12	2017	Year-1	0	0	Winter
12/3/2017	BL	10M	12	2017	Year-1	0	0	Winter
12/3/2017	BL	12M	12	2017	Year-1	0	0	Winter
12/3/2017	BL	14M	12	2017	Year-1		1	Winter
12/3/2017	BL	8A	12	2017	Year-1	0	0	Winter
12/3/2017	BL	9A	12	2017	Year-1	0	0	Winter
12/3/2017	BL	11A	12	2017	Year-1	0	0	Winter
12/3/2017	BL	13A	12	2017	Year-1	0	0	Winter
12/4/2017	BL	1M	12	2017	Year-1	0	0	Winter
12/4/2017	BL	2M	12	2017	Year-1	0	0	Winter
12/4/2017	BL	3M	12	2017	Year-1	0	0	Winter
12/4/2017	BL	4M	12	2017	Year-1	0	0	Winter
12/4/2017	BL	5M	12	2017	Year-1	0	0	Winter
12/4/2017	BL	6M	12	2017	Year-1	0	0	Winter
12/4/2017	BL	7M	12	2017	Year-1	0	0	Winter
12/4/2017	BL	10M	12	2017	Year-1	0	0	Winter
12/4/2017	BL	12M	12	2017	Year-1	0	0	Winter
12/4/2017	BL	14M	12	2017	Year-1		1	Winter
12/4/2017	BL	8A	12	2017	Year-1	0	0	Winter

Date	Species	Site	Month	Year	Survey Year	Presence	Data Gap	Season
12/4/2017	BL	9A	12	2017	Year-1	0	0	Winter
12/4/2017	BL	11A	12	2017	Year-1	0	0	Winter
12/4/2017	BL	13A	12	2017	Year-1	0	0	Winter
12/5/2017	BL	1M	12	2017	Year-1	0	0	Winter
12/5/2017	BL	2M	12	2017	Year-1	0	0	Winter
12/5/2017	BL	3M	12	2017	Year-1	0	0	Winter
12/5/2017	BL	4M	12	2017	Year-1	0	0	Winter
12/5/2017	BL	5M	12	2017	Year-1	0	0	Winter
12/5/2017	BL	6M	12	2017	Year-1	0	0	Winter
12/5/2017	BL	7M	12	2017	Year-1	0	0	Winter
12/5/2017	BL	10M	12	2017	Year-1	0	0	Winter
12/5/2017	BL	12M	12	2017	Year-1	0	0	Winter
12/5/2017	BL	14M	12	2017	Year-1		1	Winter
12/5/2017	BL	8A	12	2017	Year-1	0	0	Winter
12/5/2017	BL	9A	12	2017	Year-1	0	0	Winter
12/5/2017	BL	11A	12	2017	Year-1	0	0	Winter
12/5/2017	BL	13A	12	2017	Year-1	0	0	Winter
12/6/2017	BL	1M	12	2017	Year-1	0	0	Winter
12/6/2017	BL	2M	12	2017	Year-1	0	0	Winter
12/6/2017	BL	3M	12	2017	Year-1	0	0	Winter
12/6/2017	BL	4M	12	2017	Year-1	0	0	Winter
12/6/2017	BL	5M	12	2017	Year-1	0	0	Winter
12/6/2017	BL	6M	12	2017	Year-1	0	0	Winter
12/6/2017	BL	7M	12	2017	Year-1	0	0	Winter
12/6/2017	BL	10M	12	2017	Year-1	0	0	Winter
12/6/2017	BL	12M	12	2017	Year-1	0	0	Winter
12/6/2017	BL	14M	12	2017	Year-1		1	Winter
12/6/2017	BL	8A	12	2017	Year-1	0	0	Winter
12/6/2017	BL	9A	12	2017	Year-1	0	0	Winter
12/6/2017	BL	11A	12	2017	Year-1	0	0	Winter
12/6/2017	BL	13A	12	2017	Year-1	0	0	Winter
12/7/2017	BL	1M	12	2017	Year-1	0	0	Winter
12/7/2017	BL	2M	12	2017	Year-1	0	0	Winter
12/7/2017	BL	3M	12	2017	Year-1	0	0	Winter
12/7/2017	BL	4M	12	2017	Year-1	0	0	Winter
12/7/2017	BL	5M	12	2017	Year-1	0	0	Winter
12/7/2017	BL	6M	12	2017	Year-1	0	0	Winter
12/7/2017	BL	7M	12	2017	Year-1	0	0	Winter
12/7/2017	BL	10M	12	2017	Year-1	0	0	Winter
12/7/2017	BL	12M	12	2017	Year-1	0	0	Winter
12/7/2017	BL	14M	12	2017	Year-1		1	Winter
12/7/2017	BL	8A	12	2017	Year-1	0	0	Winter
12/7/2017	BL	9A	12	2017	Year-1	0	0	Winter
12/7/2017	BL	11A	12	2017	Year-1	0	0	Winter

Date	Species	Site	Month	Year	Survey Year	Presence	Data Gap	Season
12/7/2017	BL	13A	12	2017	Year-1	0	0	Winter
12/8/2017	BL	1M	12	2017	Year-1	0	0	Winter
12/8/2017	BL	2M	12	2017	Year-1	0	0	Winter
12/8/2017	BL	3M	12	2017	Year-1	0	0	Winter
12/8/2017	BL	4M	12	2017	Year-1	0	0	Winter
12/8/2017	BL	5M	12	2017	Year-1	0	0	Winter
12/8/2017	BL	6M	12	2017	Year-1	0	0	Winter
12/8/2017	BL	7M	12	2017	Year-1	0	0	Winter
12/8/2017	BL	10M	12	2017	Year-1	0	0	Winter
12/8/2017	BL	12M	12	2017	Year-1	0	0	Winter
12/8/2017	BL	14M	12	2017	Year-1		1	Winter
12/8/2017	BL	8A	12	2017	Year-1	0	0	Winter
12/8/2017	BL	9A	12	2017	Year-1	0	0	Winter
12/8/2017	BL	11A	12	2017	Year-1	0	0	Winter
12/8/2017	BL	13A	12	2017	Year-1	0	0	Winter
12/9/2017	BL	1M	12	2017	Year-1	0	0	Winter
12/9/2017	BL	2M	12	2017	Year-1	0	0	Winter
12/9/2017	BL	3M	12	2017	Year-1	0	0	Winter
12/9/2017	BL	4M	12	2017	Year-1	0	0	Winter
12/9/2017	BL	5M	12	2017	Year-1	0	0	Winter
12/9/2017	BL	6M	12	2017	Year-1	0	0	Winter
12/9/2017	BL	7M	12	2017	Year-1	0	0	Winter
12/9/2017	BL	10M	12	2017	Year-1	0	0	Winter
12/9/2017	BL	12M	12	2017	Year-1	0	0	Winter
12/9/2017	BL	14M	12	2017	Year-1		1	Winter
12/9/2017	BL	8A	12	2017	Year-1	0	0	Winter
12/9/2017	BL	9A	12	2017	Year-1	0	0	Winter
12/9/2017	BL	11A	12	2017	Year-1	0	0	Winter
12/9/2017	BL	13A	12	2017	Year-1	0	0	Winter
12/10/2017	BL	1M	12	2017	Year-1	1	0	Winter
12/10/2017	BL	2M	12	2017	Year-1	1	0	Winter
12/10/2017	BL	3M	12	2017	Year-1	0	0	Winter
12/10/2017	BL	4M	12	2017	Year-1	0	0	Winter
12/10/2017	BL	5M	12	2017	Year-1	0	0	Winter
12/10/2017	BL	6M	12	2017	Year-1	0	0	Winter
12/10/2017	BL	7M	12	2017	Year-1	0	0	Winter
12/10/2017	BL	10M	12	2017	Year-1	0	0	Winter
12/10/2017	BL	12M	12	2017	Year-1	0	0	Winter
12/10/2017	BL	14M	12	2017	Year-1		1	Winter
12/10/2017	BL	8A	12	2017	Year-1	0	0	Winter
12/10/2017	BL	9A	12	2017	Year-1	0	0	Winter
12/10/2017	BL	11A	12	2017	Year-1	0	0	Winter
12/10/2017	BL	13A	12	2017	Year-1	0	0	Winter
12/11/2017	BL	1M	12	2017	Year-1	0	0	Winter

Date	Species	Site	Month	Year	Survey Year	Presence	Data Gap	Season
12/11/2017	BL	2M	12	2017	Year-1	0	0	Winter
12/11/2017	BL	3M	12	2017	Year-1	0	0	Winter
12/11/2017	BL	4M	12	2017	Year-1	0	0	Winter
12/11/2017	BL	5M	12	2017	Year-1	0	0	Winter
12/11/2017	BL	6M	12	2017	Year-1	0	0	Winter
12/11/2017	BL	7M	12	2017	Year-1	0	0	Winter
12/11/2017	BL	10M	12	2017	Year-1	0	0	Winter
12/11/2017	BL	12M	12	2017	Year-1	0	0	Winter
12/11/2017	BL	14M	12	2017	Year-1		1	Winter
12/11/2017	BL	8A	12	2017	Year-1	0	0	Winter
12/11/2017	BL	9A	12	2017	Year-1	0	0	Winter
12/11/2017	BL	11A	12	2017	Year-1	0	0	Winter
12/11/2017	BL	13A	12	2017	Year-1	0	0	Winter
12/12/2017	BL	1M	12	2017	Year-1	1	0	Winter
12/12/2017	BL	2M	12	2017	Year-1	0	0	Winter
12/12/2017	BL	3M	12	2017	Year-1	0	0	Winter
12/12/2017	BL	4M	12	2017	Year-1	0	0	Winter
12/12/2017	BL	5M	12	2017	Year-1	0	0	Winter
12/12/2017	BL	6M	12	2017	Year-1	0	0	Winter
12/12/2017	BL	7M	12	2017	Year-1	0	0	Winter
12/12/2017	BL	10M	12	2017	Year-1	0	0	Winter
12/12/2017	BL	12M	12	2017	Year-1	0	0	Winter
12/12/2017	BL	14M	12	2017	Year-1		1	Winter
12/12/2017	BL	8A	12	2017	Year-1	0	0	Winter
12/12/2017	BL	9A	12	2017	Year-1	0	0	Winter
12/12/2017	BL	11A	12	2017	Year-1	0	0	Winter
12/12/2017	BL	13A	12	2017	Year-1	0	0	Winter
12/13/2017	BL	1M	12	2017	Year-1	1	0	Winter
12/13/2017	BL	2M	12	2017	Year-1	0	0	Winter
12/13/2017	BL	3M	12	2017	Year-1	0	0	Winter
12/13/2017	BL	4M	12	2017	Year-1	0	0	Winter
12/13/2017	BL	5M	12	2017	Year-1	0	0	Winter
12/13/2017	BL	6M	12	2017	Year-1	0	0	Winter
12/13/2017	BL	7M	12	2017	Year-1	0	0	Winter
12/13/2017	BL	10M	12	2017	Year-1	0	0	Winter
12/13/2017	BL	12M	12	2017	Year-1	0	0	Winter
12/13/2017	BL	14M	12	2017	Year-1		1	Winter
12/13/2017	BL	8A	12	2017	Year-1	0	0	Winter
12/13/2017	BL	9A	12	2017	Year-1	0	0	Winter
12/13/2017	BL	11A	12	2017	Year-1	0	0	Winter
12/13/2017	BL	13A	12	2017	Year-1	0	0	Winter
12/14/2017	BL	1M	12	2017	Year-1	0	0	Winter
12/14/2017	BL	2M	12	2017	Year-1	0	0	Winter
12/14/2017	BL	3M	12	2017	Year-1	0	0	Winter

Date	Species	Site	Month	Year	Survey Year	Presence	Data Gap	Season
12/14/2017	BL	4M	12	2017	Year-1	0	0	Winter
12/14/2017	BL	5M	12	2017	Year-1	0	0	Winter
12/14/2017	BL	6M	12	2017	Year-1	0	0	Winter
12/14/2017	BL	7M	12	2017	Year-1	0	0	Winter
12/14/2017	BL	10M	12	2017	Year-1	0	0	Winter
12/14/2017	BL	12M	12	2017	Year-1	0	0	Winter
12/14/2017	BL	14M	12	2017	Year-1		1	Winter
12/14/2017	BL	8A	12	2017	Year-1	0	0	Winter
12/14/2017	BL	9A	12	2017	Year-1	0	0	Winter
12/14/2017	BL	11A	12	2017	Year-1	0	0	Winter
12/14/2017	BL	13A	12	2017	Year-1	0	0	Winter
12/15/2017	BL	1M	12	2017	Year-1	1	0	Winter
12/15/2017	BL	2M	12	2017	Year-1	0	0	Winter
12/15/2017	BL	3M	12	2017	Year-1	0	0	Winter
12/15/2017	BL	4M	12	2017	Year-1	0	0	Winter
12/15/2017	BL	5M	12	2017	Year-1	0	0	Winter
12/15/2017	BL	6M	12	2017	Year-1	0	0	Winter
12/15/2017	BL	7M	12	2017	Year-1	0	0	Winter
12/15/2017	BL	10M	12	2017	Year-1	0	0	Winter
12/15/2017	BL	12M	12	2017	Year-1	0	0	Winter
12/15/2017	BL	14M	12	2017	Year-1		1	Winter
12/15/2017	BL	8A	12	2017	Year-1	0	0	Winter
12/15/2017	BL	9A	12	2017	Year-1	0	0	Winter
12/15/2017	BL	11A	12	2017	Year-1	0	0	Winter
12/15/2017	BL	13A	12	2017	Year-1	0	0	Winter
12/16/2017	BL	1M	12	2017	Year-1	1	0	Winter
12/16/2017	BL	2M	12	2017	Year-1	0	0	Winter
12/16/2017	BL	3M	12	2017	Year-1	0	0	Winter
12/16/2017	BL	4M	12	2017	Year-1	0	0	Winter
12/16/2017	BL	5M	12	2017	Year-1	0	0	Winter
12/16/2017	BL	6M	12	2017	Year-1	0	0	Winter
12/16/2017	BL	7M	12	2017	Year-1	0	0	Winter
12/16/2017	BL	10M	12	2017	Year-1	0	0	Winter
12/16/2017	BL	12M	12	2017	Year-1	0	0	Winter
12/16/2017	BL	14M	12	2017	Year-1		1	Winter
12/16/2017	BL	8A	12	2017	Year-1	0	0	Winter
12/16/2017	BL	9A	12	2017	Year-1	0	0	Winter
12/16/2017	BL	11A	12	2017	Year-1	0	0	Winter
12/16/2017	BL	13A	12	2017	Year-1	0	0	Winter
12/17/2017	BL	1M	12	2017	Year-1	1	0	Winter
12/17/2017	BL	2M	12	2017	Year-1	1	0	Winter
12/17/2017	BL	3M	12	2017	Year-1	0	0	Winter
12/17/2017	BL	4M	12	2017	Year-1	0	0	Winter
12/17/2017	BL	5M	12	2017	Year-1	0	0	Winter

Date	Species	Site	Month	Year	Survey Year	Presence	Data Gap	Season
12/17/2017	BL	6M	12	2017	Year-1	0	0	Winter
12/17/2017	BL	7M	12	2017	Year-1	0	0	Winter
12/17/2017	BL	10M	12	2017	Year-1	0	0	Winter
12/17/2017	BL	12M	12	2017	Year-1	0	0	Winter
12/17/2017	BL	14M	12	2017	Year-1		1	Winter
12/17/2017	BL	8A	12	2017	Year-1	0	0	Winter
12/17/2017	BL	9A	12	2017	Year-1	0	0	Winter
12/17/2017	BL	11A	12	2017	Year-1	0	0	Winter
12/17/2017	BL	13A	12	2017	Year-1	0	0	Winter
12/18/2017	BL	1M	12	2017	Year-1	1	0	Winter
12/18/2017	BL	2M	12	2017	Year-1	0	0	Winter
12/18/2017	BL	3M	12	2017	Year-1	0	0	Winter
12/18/2017	BL	4M	12	2017	Year-1		1	Winter
12/18/2017	BL	5M	12	2017	Year-1	0	0	Winter
12/18/2017	BL	6M	12	2017	Year-1	0	0	Winter
12/18/2017	BL	7M	12	2017	Year-1	0	0	Winter
12/18/2017	BL	10M	12	2017	Year-1	0	0	Winter
12/18/2017	BL	12M	12	2017	Year-1	0	0	Winter
12/18/2017	BL	14M	12	2017	Year-1		1	Winter
12/18/2017	BL	8A	12	2017	Year-1	0	0	Winter
12/18/2017	BL	9A	12	2017	Year-1	0	0	Winter
12/18/2017	BL	11A	12	2017	Year-1	0	0	Winter
12/18/2017	BL	13A	12	2017	Year-1	0	0	Winter
12/19/2017	BL	1M	12	2017	Year-1	0	0	Winter
12/19/2017	BL	2M	12	2017	Year-1	0	0	Winter
12/19/2017	BL	3M	12	2017	Year-1	0	0	Winter
12/19/2017	BL	4M	12	2017	Year-1		1	Winter
12/19/2017	BL	5M	12	2017	Year-1	0	0	Winter
12/19/2017	BL	6M	12	2017	Year-1	0	0	Winter
12/19/2017	BL	7M	12	2017	Year-1	0	0	Winter
12/19/2017	BL	10M	12	2017	Year-1	0	0	Winter
12/19/2017	BL	12M	12	2017	Year-1	0	0	Winter
12/19/2017	BL	14M	12	2017	Year-1		1	Winter
12/19/2017	BL	8A	12	2017	Year-1	0	0	Winter
12/19/2017	BL	9A	12	2017	Year-1	0	0	Winter
12/19/2017	BL	11A	12	2017	Year-1	0	0	Winter
12/19/2017	BL	13A	12	2017	Year-1	0	0	Winter
12/20/2017	BL	1M	12	2017	Year-1	0	0	Winter
12/20/2017	BL	2M	12	2017	Year-1	0	0	Winter
12/20/2017	BL	3M	12	2017	Year-1	0	0	Winter
12/20/2017	BL	4M	12	2017	Year-1		1	Winter
12/20/2017	BL	5M	12	2017	Year-1	0	0	Winter
12/20/2017	BL	6M	12	2017	Year-1	0	0	Winter
12/20/2017	BL	7M	12	2017	Year-1	0	0	Winter

Date	Species	Site	Month	Year	Survey Year	Presence	Data Gap	Season
12/20/2017	BL	10M	12	2017	Year-1	0	0	Winter
12/20/2017	BL	12M	12	2017	Year-1	0	0	Winter
12/20/2017	BL	14M	12	2017	Year-1		1	Winter
12/20/2017	BL	8A	12	2017	Year-1	0	0	Winter
12/20/2017	BL	9A	12	2017	Year-1	0	0	Winter
12/20/2017	BL	11A	12	2017	Year-1	0	0	Winter
12/20/2017	BL	13A	12	2017	Year-1	0	0	Winter
12/21/2017	BL	1M	12	2017	Year-1	0	0	Winter
12/21/2017	BL	2M	12	2017	Year-1	0	0	Winter
12/21/2017	BL	3M	12	2017	Year-1	0	0	Winter
12/21/2017	BL	4M	12	2017	Year-1		1	Winter
12/21/2017	BL	5M	12	2017	Year-1	0	0	Winter
12/21/2017	BL	6M	12	2017	Year-1	0	0	Winter
12/21/2017	BL	7M	12	2017	Year-1	0	0	Winter
12/21/2017	BL	10M	12	2017	Year-1	0	0	Winter
12/21/2017	BL	12M	12	2017	Year-1	0	0	Winter
12/21/2017	BL	14M	12	2017	Year-1		1	Winter
12/21/2017	BL	8A	12	2017	Year-1	0	0	Winter
12/21/2017	BL	9A	12	2017	Year-1	0	0	Winter
12/21/2017	BL	11A	12	2017	Year-1	0	0	Winter
12/21/2017	BL	13A	12	2017	Year-1	0	0	Winter
12/22/2017	BL	1M	12	2017	Year-1	0	0	Winter
12/22/2017	BL	2M	12	2017	Year-1	0	0	Winter
12/22/2017	BL	3M	12	2017	Year-1	0	0	Winter
12/22/2017	BL	4M	12	2017	Year-1		1	Winter
12/22/2017	BL	5M	12	2017	Year-1	0	0	Winter
12/22/2017	BL	6M	12	2017	Year-1	0	0	Winter
12/22/2017	BL	7M	12	2017	Year-1	0	0	Winter
12/22/2017	BL	10M	12	2017	Year-1	0	0	Winter
12/22/2017	BL	12M	12	2017	Year-1	0	0	Winter
12/22/2017	BL	14M	12	2017	Year-1		1	Winter
12/22/2017	BL	8A	12	2017	Year-1	0	0	Winter
12/22/2017	BL	9A	12	2017	Year-1	0	0	Winter
12/22/2017	BL	11A	12	2017	Year-1	0	0	Winter
12/22/2017	BL	13A	12	2017	Year-1	0	0	Winter
12/23/2017	BL	1M	12	2017	Year-1	0	0	Winter
12/23/2017	BL	2M	12	2017	Year-1	0	0	Winter
12/23/2017	BL	3M	12	2017	Year-1	0	0	Winter
12/23/2017	BL	4M	12	2017	Year-1		1	Winter
12/23/2017	BL	5M	12	2017	Year-1	0	0	Winter
12/23/2017	BL	6M	12	2017	Year-1	0	0	Winter
12/23/2017	BL	7M	12	2017	Year-1	0	0	Winter
12/23/2017	BL	10M	12	2017	Year-1	0	0	Winter
12/23/2017	BL	12M	12	2017	Year-1	0	0	Winter

Date	Species	Site	Month	Year	Survey Year	Presence	Data Gap	Season
12/23/2017	BL	14M	12	2017	Year-1		1	Winter
12/23/2017	BL	8A	12	2017	Year-1	0	0	Winter
12/23/2017	BL	9A	12	2017	Year-1	0	0	Winter
12/23/2017	BL	11A	12	2017	Year-1	0	0	Winter
12/23/2017	BL	13A	12	2017	Year-1	0	0	Winter
12/24/2017	BL	1M	12	2017	Year-1	0	0	Winter
12/24/2017	BL	2M	12	2017	Year-1	0	0	Winter
12/24/2017	BL	3M	12	2017	Year-1	0	0	Winter
12/24/2017	BL	4M	12	2017	Year-1		1	Winter
12/24/2017	BL	5M	12	2017	Year-1	0	0	Winter
12/24/2017	BL	6M	12	2017	Year-1	0	0	Winter
12/24/2017	BL	7M	12	2017	Year-1	0	0	Winter
12/24/2017	BL	10M	12	2017	Year-1	0	0	Winter
12/24/2017	BL	12M	12	2017	Year-1	0	0	Winter
12/24/2017	BL	14M	12	2017	Year-1		1	Winter
12/24/2017	BL	8A	12	2017	Year-1	0	0	Winter
12/24/2017	BL	9A	12	2017	Year-1	0	0	Winter
12/24/2017	BL	11A	12	2017	Year-1	0	0	Winter
12/24/2017	BL	13A	12	2017	Year-1	0	0	Winter
12/25/2017	BL	1M	12	2017	Year-1	0	0	Winter
12/25/2017	BL	2M	12	2017	Year-1	0	0	Winter
12/25/2017	BL	3M	12	2017	Year-1	0	0	Winter
12/25/2017	BL	4M	12	2017	Year-1		1	Winter
12/25/2017	BL	5M	12	2017	Year-1	0	0	Winter
12/25/2017	BL	6M	12	2017	Year-1	0	0	Winter
12/25/2017	BL	7M	12	2017	Year-1	0	0	Winter
12/25/2017	BL	10M	12	2017	Year-1	0	0	Winter
12/25/2017	BL	12M	12	2017	Year-1	0	0	Winter
12/25/2017	BL	14M	12	2017	Year-1		1	Winter
12/25/2017	BL	8A	12	2017	Year-1	0	0	Winter
12/25/2017	BL	9A	12	2017	Year-1	0	0	Winter
12/25/2017	BL	11A	12	2017	Year-1	0	0	Winter
12/25/2017	BL	13A	12	2017	Year-1	0	0	Winter
12/26/2017	BL	1M	12	2017	Year-1	0	0	Winter
12/26/2017	BL	2M	12	2017	Year-1	0	0	Winter
12/26/2017	BL	3M	12	2017	Year-1	0	0	Winter
12/26/2017	BL	4M	12	2017	Year-1		1	Winter
12/26/2017	BL	5M	12	2017	Year-1	0	0	Winter
12/26/2017	BL	6M	12	2017	Year-1	0	0	Winter
12/26/2017	BL	7M	12	2017	Year-1	0	0	Winter
12/26/2017	BL	10M	12	2017	Year-1	0	0	Winter
12/26/2017	BL	12M	12	2017	Year-1	0	0	Winter
12/26/2017	BL	14M	12	2017	Year-1		1	Winter
12/26/2017	BL	8A	12	2017	Year-1	0	0	Winter

Date	Species	Site	Month	Year	Survey Year	Presence	Data Gap	Season
12/26/2017	BL	9A	12	2017	Year-1	0	0	Winter
12/26/2017	BL	11A	12	2017	Year-1	0	0	Winter
12/26/2017	BL	13A	12	2017	Year-1	0	0	Winter
12/27/2017	BL	1M	12	2017	Year-1	0	0	Winter
12/27/2017	BL	2M	12	2017	Year-1	0	0	Winter
12/27/2017	BL	3M	12	2017	Year-1	0	0	Winter
12/27/2017	BL	4M	12	2017	Year-1		1	Winter
12/27/2017	BL	5M	12	2017	Year-1	0	0	Winter
12/27/2017	BL	6M	12	2017	Year-1	0	0	Winter
12/27/2017	BL	7M	12	2017	Year-1	0	0	Winter
12/27/2017	BL	10M	12	2017	Year-1	0	0	Winter
12/27/2017	BL	12M	12	2017	Year-1	0	0	Winter
12/27/2017	BL	14M	12	2017	Year-1		1	Winter
12/27/2017	BL	8A	12	2017	Year-1	0	0	Winter
12/27/2017	BL	9A	12	2017	Year-1	0	0	Winter
12/27/2017	BL	11A	12	2017	Year-1	0	0	Winter
12/27/2017	BL	13A	12	2017	Year-1	0	0	Winter
12/28/2017	BL	1M	12	2017	Year-1	0	0	Winter
12/28/2017	BL	2M	12	2017	Year-1	0	0	Winter
12/28/2017	BL	3M	12	2017	Year-1	0	0	Winter
12/28/2017	BL	4M	12	2017	Year-1		1	Winter
12/28/2017	BL	5M	12	2017	Year-1	0	0	Winter
12/28/2017	BL	6M	12	2017	Year-1	0	0	Winter
12/28/2017	BL	7M	12	2017	Year-1	0	0	Winter
12/28/2017	BL	10M	12	2017	Year-1	0	0	Winter
12/28/2017	BL	12M	12	2017	Year-1	0	0	Winter
12/28/2017	BL	14M	12	2017	Year-1		1	Winter
12/28/2017	BL	8A	12	2017	Year-1	0	0	Winter
12/28/2017	BL	9A	12	2017	Year-1	0	0	Winter
12/28/2017	BL	11A	12	2017	Year-1	0	0	Winter
12/28/2017	BL	13A	12	2017	Year-1	0	0	Winter
12/29/2017	BL	1M	12	2017	Year-1	0	0	Winter
12/29/2017	BL	2M	12	2017	Year-1	0	0	Winter
12/29/2017	BL	3M	12	2017	Year-1	0	0	Winter
12/29/2017	BL	4M	12	2017	Year-1		1	Winter
12/29/2017	BL	5M	12	2017	Year-1	0	0	Winter
12/29/2017	BL	6M	12	2017	Year-1	0	0	Winter
12/29/2017	BL	7M	12	2017	Year-1	0	0	Winter
12/29/2017	BL	10M	12	2017	Year-1	0	0	Winter
12/29/2017	BL	12M	12	2017	Year-1	0	0	Winter
12/29/2017	BL	14M	12	2017	Year-1		1	Winter
12/29/2017	BL	8A	12	2017	Year-1	0	0	Winter
12/29/2017	BL	9A	12	2017	Year-1	0	0	Winter
12/29/2017	BL	11A	12	2017	Year-1	0	0	Winter

Date	Species	Site	Month	Year	Survey Year	Presence	Data Gap	Season
12/29/2017	BL	13A	12	2017	Year-1	0	0	Winter
12/30/2017	BL	1M	12	2017	Year-1	0	0	Winter
12/30/2017	BL	2M	12	2017	Year-1	0	0	Winter
12/30/2017	BL	3M	12	2017	Year-1	0	0	Winter
12/30/2017	BL	4M	12	2017	Year-1		1	Winter
12/30/2017	BL	5M	12	2017	Year-1	0	0	Winter
12/30/2017	BL	6M	12	2017	Year-1	0	0	Winter
12/30/2017	BL	7M	12	2017	Year-1	0	0	Winter
12/30/2017	BL	10M	12	2017	Year-1	0	0	Winter
12/30/2017	BL	12M	12	2017	Year-1	0	0	Winter
12/30/2017	BL	14M	12	2017	Year-1		1	Winter
12/30/2017	BL	8A	12	2017	Year-1	0	0	Winter
12/30/2017	BL	9A	12	2017	Year-1	0	0	Winter
12/30/2017	BL	11A	12	2017	Year-1	0	0	Winter
12/30/2017	BL	13A	12	2017	Year-1	0	0	Winter
12/31/2017	BL	1M	12	2017	Year-1	0	0	Winter
12/31/2017	BL	2M	12	2017	Year-1	0	0	Winter
12/31/2017	BL	3M	12	2017	Year-1	0	0	Winter
12/31/2017	BL	4M	12	2017	Year-1		1	Winter
12/31/2017	BL	5M	12	2017	Year-1	0	0	Winter
12/31/2017	BL	6M	12	2017	Year-1	0	0	Winter
12/31/2017	BL	7M	12	2017	Year-1	0	0	Winter
12/31/2017	BL	10M	12	2017	Year-1	0	0	Winter
12/31/2017	BL	12M	12	2017	Year-1	0	0	Winter
12/31/2017	BL	14M	12	2017	Year-1		1	Winter
12/31/2017	BL	8A	12	2017	Year-1	0	0	Winter
12/31/2017	BL	9A	12	2017	Year-1	0	0	Winter
12/31/2017	BL	11A	12	2017	Year-1	0	0	Winter
12/31/2017	BL	13A	12	2017	Year-1	0	0	Winter
1/1/2018	BL	1M	1	2018	Year-1	0	0	Winter
1/1/2018	BL	2M	1	2018	Year-1	0	0	Winter
1/1/2018	BL	3M	1	2018	Year-1	0	0	Winter
1/1/2018	BL	4M	1	2018	Year-1		1	Winter
1/1/2018	BL	5M	1	2018	Year-1	0	0	Winter
1/1/2018	BL	6M	1	2018	Year-1	0	0	Winter
1/1/2018	BL	7M	1	2018	Year-1	0	0	Winter
1/1/2018	BL	10M	1	2018	Year-1	0	0	Winter
1/1/2018	BL	12M	1	2018	Year-1	0	0	Winter
1/1/2018	BL	14M	1	2018	Year-1		1	Winter
1/1/2018	BL	8A	1	2018	Year-1	0	0	Winter
1/1/2018	BL	9A	1	2018	Year-1	0	0	Winter
1/1/2018	BL	11A	1	2018	Year-1	0	0	Winter
1/1/2018	BL	13A	1	2018	Year-1	0	0	Winter
1/2/2018	BL	1M	1	2018	Year-1	0	0	Winter

Date	Species	Site	Month	Year	Survey Year	Presence	Data Gap	Season
1/2/2018	BL	2M	1	2018	Year-1	0	0	Winter
1/2/2018	BL	3M	1	2018	Year-1	0	0	Winter
1/2/2018	BL	4M	1	2018	Year-1		1	Winter
1/2/2018	BL	5M	1	2018	Year-1	0	0	Winter
1/2/2018	BL	6M	1	2018	Year-1	0	0	Winter
1/2/2018	BL	7M	1	2018	Year-1	0	0	Winter
1/2/2018	BL	10M	1	2018	Year-1	0	0	Winter
1/2/2018	BL	12M	1	2018	Year-1	0	0	Winter
1/2/2018	BL	14M	1	2018	Year-1		1	Winter
1/2/2018	BL	8A	1	2018	Year-1	0	0	Winter
1/2/2018	BL	9A	1	2018	Year-1	0	0	Winter
1/2/2018	BL	11A	1	2018	Year-1	0	0	Winter
1/2/2018	BL	13A	1	2018	Year-1	0	0	Winter
1/3/2018	BL	1M	1	2018	Year-1	0	0	Winter
1/3/2018	BL	2M	1	2018	Year-1	0	0	Winter
1/3/2018	BL	3M	1	2018	Year-1	0	0	Winter
1/3/2018	BL	4M	1	2018	Year-1		1	Winter
1/3/2018	BL	5M	1	2018	Year-1	0	0	Winter
1/3/2018	BL	6M	1	2018	Year-1	0	0	Winter
1/3/2018	BL	7M	1	2018	Year-1	0	0	Winter
1/3/2018	BL	10M	1	2018	Year-1	0	0	Winter
1/3/2018	BL	12M	1	2018	Year-1	0	0	Winter
1/3/2018	BL	14M	1	2018	Year-1		1	Winter
1/3/2018	BL	8A	1	2018	Year-1	0	0	Winter
1/3/2018	BL	9A	1	2018	Year-1	0	0	Winter
1/3/2018	BL	11A	1	2018	Year-1	0	0	Winter
1/3/2018	BL	13A	1	2018	Year-1	0	0	Winter
1/4/2018	BL	1M	1	2018	Year-1	0	0	Winter
1/4/2018	BL	2M	1	2018	Year-1	0	0	Winter
1/4/2018	BL	3M	1	2018	Year-1	0	0	Winter
1/4/2018	BL	4M	1	2018	Year-1		1	Winter
1/4/2018	BL	5M	1	2018	Year-1	0	0	Winter
1/4/2018	BL	6M	1	2018	Year-1	0	0	Winter
1/4/2018	BL	7M	1	2018	Year-1	0	0	Winter
1/4/2018	BL	10M	1	2018	Year-1	0	0	Winter
1/4/2018	BL	12M	1	2018	Year-1	0	0	Winter
1/4/2018	BL	14M	1	2018	Year-1		1	Winter
1/4/2018	BL	8A	1	2018	Year-1	0	0	Winter
1/4/2018	BL	9A	1	2018	Year-1	0	0	Winter
1/4/2018	BL	11A	1	2018	Year-1	0	0	Winter
1/4/2018	BL	13A	1	2018	Year-1	0	0	Winter
1/5/2018	BL	1M	1	2018	Year-1	0	0	Winter
1/5/2018	BL	2M	1	2018	Year-1	0	0	Winter
1/5/2018	BL	3M	1	2018	Year-1	0	0	Winter

Date	Species	Site	Month	Year	Survey Year	Presence	Data Gap	Season
1/5/2018	BL	4M	1	2018	Year-1		1	Winter
1/5/2018	BL	5M	1	2018	Year-1	0	0	Winter
1/5/2018	BL	6M	1	2018	Year-1	0	0	Winter
1/5/2018	BL	7M	1	2018	Year-1	0	0	Winter
1/5/2018	BL	10M	1	2018	Year-1	0	0	Winter
1/5/2018	BL	12M	1	2018	Year-1	0	0	Winter
1/5/2018	BL	14M	1	2018	Year-1		1	Winter
1/5/2018	BL	8A	1	2018	Year-1	0	0	Winter
1/5/2018	BL	9A	1	2018	Year-1	0	0	Winter
1/5/2018	BL	11A	1	2018	Year-1	0	0	Winter
1/5/2018	BL	13A	1	2018	Year-1	0	0	Winter
1/6/2018	BL	1M	1	2018	Year-1	0	0	Winter
1/6/2018	BL	2M	1	2018	Year-1	0	0	Winter
1/6/2018	BL	3M	1	2018	Year-1	0	0	Winter
1/6/2018	BL	4M	1	2018	Year-1		1	Winter
1/6/2018	BL	5M	1	2018	Year-1	0	0	Winter
1/6/2018	BL	6M	1	2018	Year-1	0	0	Winter
1/6/2018	BL	7M	1	2018	Year-1	0	0	Winter
1/6/2018	BL	10M	1	2018	Year-1	0	0	Winter
1/6/2018	BL	12M	1	2018	Year-1	0	0	Winter
1/6/2018	BL	14M	1	2018	Year-1		1	Winter
1/6/2018	BL	8A	1	2018	Year-1	0	0	Winter
1/6/2018	BL	9A	1	2018	Year-1	0	0	Winter
1/6/2018	BL	11A	1	2018	Year-1	0	0	Winter
1/6/2018	BL	13A	1	2018	Year-1	0	0	Winter
1/7/2018	BL	1M	1	2018	Year-1	0	0	Winter
1/7/2018	BL	2M	1	2018	Year-1	0	0	Winter
1/7/2018	BL	3M	1	2018	Year-1	0	0	Winter
1/7/2018	BL	4M	1	2018	Year-1		1	Winter
1/7/2018	BL	5M	1	2018	Year-1	0	0	Winter
1/7/2018	BL	6M	1	2018	Year-1	0	0	Winter
1/7/2018	BL	7M	1	2018	Year-1	0	0	Winter
1/7/2018	BL	10M	1	2018	Year-1	0	0	Winter
1/7/2018	BL	12M	1	2018	Year-1	0	0	Winter
1/7/2018	BL	14M	1	2018	Year-1		1	Winter
1/7/2018	BL	8A	1	2018	Year-1	0	0	Winter
1/7/2018	BL	9A	1	2018	Year-1	0	0	Winter
1/7/2018	BL	11A	1	2018	Year-1	0	0	Winter
1/7/2018	BL	13A	1	2018	Year-1	0	0	Winter
1/8/2018	BL	1M	1	2018	Year-1	0	0	Winter
1/8/2018	BL	2M	1	2018	Year-1	0	0	Winter
1/8/2018	BL	3M	1	2018	Year-1	0	0	Winter
1/8/2018	BL	4M	1	2018	Year-1		1	Winter
1/8/2018	BL	5M	1	2018	Year-1	0	0	Winter

Date	Species	Site	Month	Year	Survey Year	Presence	Data Gap	Season
1/8/2018	BL	6M	1	2018	Year-1	0	0	Winter
1/8/2018	BL	7M	1	2018	Year-1	0	0	Winter
1/8/2018	BL	10M	1	2018	Year-1	0	0	Winter
1/8/2018	BL	12M	1	2018	Year-1	0	0	Winter
1/8/2018	BL	14M	1	2018	Year-1		1	Winter
1/8/2018	BL	8A	1	2018	Year-1	0	0	Winter
1/8/2018	BL	9A	1	2018	Year-1	0	0	Winter
1/8/2018	BL	11A	1	2018	Year-1	0	0	Winter
1/8/2018	BL	13A	1	2018	Year-1	0	0	Winter
1/9/2018	BL	1M	1	2018	Year-1	0	0	Winter
1/9/2018	BL	2M	1	2018	Year-1	0	0	Winter
1/9/2018	BL	3M	1	2018	Year-1	0	0	Winter
1/9/2018	BL	4M	1	2018	Year-1		1	Winter
1/9/2018	BL	5M	1	2018	Year-1	0	0	Winter
1/9/2018	BL	6M	1	2018	Year-1	0	0	Winter
1/9/2018	BL	7M	1	2018	Year-1	0	0	Winter
1/9/2018	BL	10M	1	2018	Year-1	0	0	Winter
1/9/2018	BL	12M	1	2018	Year-1	0	0	Winter
1/9/2018	BL	14M	1	2018	Year-1		1	Winter
1/9/2018	BL	8A	1	2018	Year-1	0	0	Winter
1/9/2018	BL	9A	1	2018	Year-1	0	0	Winter
1/9/2018	BL	11A	1	2018	Year-1	0	0	Winter
1/9/2018	BL	13A	1	2018	Year-1	0	0	Winter
1/10/2018	BL	1M	1	2018	Year-1	0	0	Winter
1/10/2018	BL	2M	1	2018	Year-1	0	0	Winter
1/10/2018	BL	3M	1	2018	Year-1	0	0	Winter
1/10/2018	BL	4M	1	2018	Year-1		1	Winter
1/10/2018	BL	5M	1	2018	Year-1	0	0	Winter
1/10/2018	BL	6M	1	2018	Year-1	0	0	Winter
1/10/2018	BL	7M	1	2018	Year-1	0	0	Winter
1/10/2018	BL	10M	1	2018	Year-1	0	0	Winter
1/10/2018	BL	12M	1	2018	Year-1	0	0	Winter
1/10/2018	BL	14M	1	2018	Year-1		1	Winter
1/10/2018	BL	8A	1	2018	Year-1	0	0	Winter
1/10/2018	BL	9A	1	2018	Year-1	0	0	Winter
1/10/2018	BL	11A	1	2018	Year-1	0	0	Winter
1/10/2018	BL	13A	1	2018	Year-1	0	0	Winter
1/11/2018	BL	1M	1	2018	Year-1	0	0	Winter
1/11/2018	BL	2M	1	2018	Year-1	0	0	Winter
1/11/2018	BL	3M	1	2018	Year-1	0	0	Winter
1/11/2018	BL	4M	1	2018	Year-1		1	Winter
1/11/2018	BL	5M	1	2018	Year-1	0	0	Winter
1/11/2018	BL	6M	1	2018	Year-1	0	0	Winter
1/11/2018	BL	7M	1	2018	Year-1	0	0	Winter

Date	Species	Site	Month	Year	Survey Year	Presence	Data Gap	Season
1/11/2018	BL	10M	1	2018	Year-1	0	0	Winter
1/11/2018	BL	12M	1	2018	Year-1	0	0	Winter
1/11/2018	BL	14M	1	2018	Year-1		1	Winter
1/11/2018	BL	8A	1	2018	Year-1	0	0	Winter
1/11/2018	BL	9A	1	2018	Year-1	0	0	Winter
1/11/2018	BL	11A	1	2018	Year-1	0	0	Winter
1/11/2018	BL	13A	1	2018	Year-1	0	0	Winter
1/12/2018	BL	1M	1	2018	Year-1	1	0	Winter
1/12/2018	BL	2M	1	2018	Year-1	1	0	Winter
1/12/2018	BL	3M	1	2018	Year-1	0	0	Winter
1/12/2018	BL	4M	1	2018	Year-1		1	Winter
1/12/2018	BL	5M	1	2018	Year-1	0	0	Winter
1/12/2018	BL	6M	1	2018	Year-1	0	0	Winter
1/12/2018	BL	7M	1	2018	Year-1	0	0	Winter
1/12/2018	BL	10M	1	2018	Year-1	0	0	Winter
1/12/2018	BL	12M	1	2018	Year-1	0	0	Winter
1/12/2018	BL	14M	1	2018	Year-1		1	Winter
1/12/2018	BL	8A	1	2018	Year-1	0	0	Winter
1/12/2018	BL	9A	1	2018	Year-1	0	0	Winter
1/12/2018	BL	11A	1	2018	Year-1	0	0	Winter
1/12/2018	BL	13A	1	2018	Year-1	0	0	Winter
1/13/2018	BL	1M	1	2018	Year-1	1	0	Winter
1/13/2018	BL	2M	1	2018	Year-1	1	0	Winter
1/13/2018	BL	3M	1	2018	Year-1	1	0	Winter
1/13/2018	BL	4M	1	2018	Year-1		1	Winter
1/13/2018	BL	5M	1	2018	Year-1	0	0	Winter
1/13/2018	BL	6M	1	2018	Year-1	0	0	Winter
1/13/2018	BL	7M	1	2018	Year-1	0	0	Winter
1/13/2018	BL	10M	1	2018	Year-1	0	0	Winter
1/13/2018	BL	12M	1	2018	Year-1	0	0	Winter
1/13/2018	BL	14M	1	2018	Year-1		1	Winter
1/13/2018	BL	8A	1	2018	Year-1	0	0	Winter
1/13/2018	BL	9A	1	2018	Year-1	0	0	Winter
1/13/2018	BL	11A	1	2018	Year-1	0	0	Winter
1/13/2018	BL	13A	1	2018	Year-1	0	0	Winter
1/14/2018	BL	1M	1	2018	Year-1	1	0	Winter
1/14/2018	BL	2M	1	2018	Year-1	1	0	Winter
1/14/2018	BL	3M	1	2018	Year-1	0	0	Winter
1/14/2018	BL	4M	1	2018	Year-1		1	Winter
1/14/2018	BL	5M	1	2018	Year-1	0	0	Winter
1/14/2018	BL	6M	1	2018	Year-1	0	0	Winter
1/14/2018	BL	7M	1	2018	Year-1	0	0	Winter
1/14/2018	BL	10M	1	2018	Year-1	0	0	Winter
1/14/2018	BL	12M	1	2018	Year-1	0	0	Winter

Date	Species	Site	Month	Year	Survey Year	Presence	Data Gap	Season
1/14/2018	BL	14M	1	2018	Year-1		1	Winter
1/14/2018	BL	8A	1	2018	Year-1	0	0	Winter
1/14/2018	BL	9A	1	2018	Year-1	0	0	Winter
1/14/2018	BL	11A	1	2018	Year-1	0	0	Winter
1/14/2018	BL	13A	1	2018	Year-1	0	0	Winter
1/15/2018	BL	1M	1	2018	Year-1	0	0	Winter
1/15/2018	BL	2M	1	2018	Year-1	0	0	Winter
1/15/2018	BL	3M	1	2018	Year-1	0	0	Winter
1/15/2018	BL	4M	1	2018	Year-1		1	Winter
1/15/2018	BL	5M	1	2018	Year-1	0	0	Winter
1/15/2018	BL	6M	1	2018	Year-1	0	0	Winter
1/15/2018	BL	7M	1	2018	Year-1	0	0	Winter
1/15/2018	BL	10M	1	2018	Year-1	0	0	Winter
1/15/2018	BL	12M	1	2018	Year-1	0	0	Winter
1/15/2018	BL	14M	1	2018	Year-1		1	Winter
1/15/2018	BL	8A	1	2018	Year-1	0	0	Winter
1/15/2018	BL	9A	1	2018	Year-1	0	0	Winter
1/15/2018	BL	11A	1	2018	Year-1	0	0	Winter
1/15/2018	BL	13A	1	2018	Year-1	0	0	Winter
1/16/2018	BL	1M	1	2018	Year-1	0	0	Winter
1/16/2018	BL	2M	1	2018	Year-1	0	0	Winter
1/16/2018	BL	3M	1	2018	Year-1	0	0	Winter
1/16/2018	BL	4M	1	2018	Year-1		1	Winter
1/16/2018	BL	5M	1	2018	Year-1	0	0	Winter
1/16/2018	BL	6M	1	2018	Year-1	0	0	Winter
1/16/2018	BL	7M	1	2018	Year-1	0	0	Winter
1/16/2018	BL	10M	1	2018	Year-1	0	0	Winter
1/16/2018	BL	12M	1	2018	Year-1	0	0	Winter
1/16/2018	BL	14M	1	2018	Year-1		1	Winter
1/16/2018	BL	8A	1	2018	Year-1	0	0	Winter
1/16/2018	BL	9A	1	2018	Year-1	0	0	Winter
1/16/2018	BL	11A	1	2018	Year-1	0	0	Winter
1/16/2018	BL	13A	1	2018	Year-1	0	0	Winter
1/17/2018	BL	1M	1	2018	Year-1	1	0	Winter
1/17/2018	BL	2M	1	2018	Year-1	1	0	Winter
1/17/2018	BL	3M	1	2018	Year-1	1	0	Winter
1/17/2018	BL	4M	1	2018	Year-1		1	Winter
1/17/2018	BL	5M	1	2018	Year-1	0	0	Winter
1/17/2018	BL	6M	1	2018	Year-1	0	0	Winter
1/17/2018	BL	7M	1	2018	Year-1	0	0	Winter
1/17/2018	BL	10M	1	2018	Year-1	0	0	Winter
1/17/2018	BL	12M	1	2018	Year-1	0	0	Winter
1/17/2018	BL	14M	1	2018	Year-1		1	Winter
1/17/2018	BL	8A	1	2018	Year-1	0	0	Winter

Date	Species	Site	Month	Year	Survey Year	Presence	Data Gap	Season
1/17/2018	BL	9A	1	2018	Year-1	0	0	Winter
1/17/2018	BL	11A	1	2018	Year-1	0	0	Winter
1/17/2018	BL	13A	1	2018	Year-1	0	0	Winter
1/18/2018	BL	1M	1	2018	Year-1	1	0	Winter
1/18/2018	BL	2M	1	2018	Year-1	1	0	Winter
1/18/2018	BL	3M	1	2018	Year-1	0	0	Winter
1/18/2018	BL	4M	1	2018	Year-1		1	Winter
1/18/2018	BL	5M	1	2018	Year-1	0	0	Winter
1/18/2018	BL	6M	1	2018	Year-1	0	0	Winter
1/18/2018	BL	7M	1	2018	Year-1	0	0	Winter
1/18/2018	BL	10M	1	2018	Year-1	0	0	Winter
1/18/2018	BL	12M	1	2018	Year-1	0	0	Winter
1/18/2018	BL	14M	1	2018	Year-1		1	Winter
1/18/2018	BL	8A	1	2018	Year-1	0	0	Winter
1/18/2018	BL	9A	1	2018	Year-1	0	0	Winter
1/18/2018	BL	11A	1	2018	Year-1	0	0	Winter
1/18/2018	BL	13A	1	2018	Year-1	0	0	Winter
1/19/2018	BL	1M	1	2018	Year-1	0	0	Winter
1/19/2018	BL	2M	1	2018	Year-1	0	0	Winter
1/19/2018	BL	3M	1	2018	Year-1	0	0	Winter
1/19/2018	BL	4M	1	2018	Year-1		1	Winter
1/19/2018	BL	5M	1	2018	Year-1	0	0	Winter
1/19/2018	BL	6M	1	2018	Year-1	0	0	Winter
1/19/2018	BL	7M	1	2018	Year-1	0	0	Winter
1/19/2018	BL	10M	1	2018	Year-1	0	0	Winter
1/19/2018	BL	12M	1	2018	Year-1	0	0	Winter
1/19/2018	BL	14M	1	2018	Year-1		1	Winter
1/19/2018	BL	8A	1	2018	Year-1	0	0	Winter
1/19/2018	BL	9A	1	2018	Year-1	0	0	Winter
1/19/2018	BL	11A	1	2018	Year-1	0	0	Winter
1/19/2018	BL	13A	1	2018	Year-1	0	0	Winter
1/20/2018	BL	1M	1	2018	Year-1	1	0	Winter
1/20/2018	BL	2M	1	2018	Year-1	0	0	Winter
1/20/2018	BL	3M	1	2018	Year-1	0	0	Winter
1/20/2018	BL	4M	1	2018	Year-1		1	Winter
1/20/2018	BL	5M	1	2018	Year-1	0	0	Winter
1/20/2018	BL	6M	1	2018	Year-1	0	0	Winter
1/20/2018	BL	7M	1	2018	Year-1	0	0	Winter
1/20/2018	BL	10M	1	2018	Year-1	0	0	Winter
1/20/2018	BL	12M	1	2018	Year-1	0	0	Winter
1/20/2018	BL	14M	1	2018	Year-1		1	Winter
1/20/2018	BL	8A	1	2018	Year-1	0	0	Winter
1/20/2018	BL	9A	1	2018	Year-1	0	0	Winter
1/20/2018	BL	11A	1	2018	Year-1	0	0	Winter

Date	Species	Site	Month	Year	Survey Year	Presence	Data Gap	Season
1/20/2018	BL	13A	1	2018	Year-1	0	0	Winter
1/21/2018	BL	1M	1	2018	Year-1	0	0	Winter
1/21/2018	BL	2M	1	2018	Year-1	0	0	Winter
1/21/2018	BL	3M	1	2018	Year-1	0	0	Winter
1/21/2018	BL	4M	1	2018	Year-1		1	Winter
1/21/2018	BL	5M	1	2018	Year-1	0	0	Winter
1/21/2018	BL	6M	1	2018	Year-1	0	0	Winter
1/21/2018	BL	7M	1	2018	Year-1	0	0	Winter
1/21/2018	BL	10M	1	2018	Year-1	0	0	Winter
1/21/2018	BL	12M	1	2018	Year-1	0	0	Winter
1/21/2018	BL	14M	1	2018	Year-1		1	Winter
1/21/2018	BL	8A	1	2018	Year-1	0	0	Winter
1/21/2018	BL	9A	1	2018	Year-1	0	0	Winter
1/21/2018	BL	11A	1	2018	Year-1	0	0	Winter
1/21/2018	BL	13A	1	2018	Year-1	0	0	Winter
1/22/2018	BL	1M	1	2018	Year-1	0	0	Winter
1/22/2018	BL	2M	1	2018	Year-1	0	0	Winter
1/22/2018	BL	3M	1	2018	Year-1	0	0	Winter
1/22/2018	BL	4M	1	2018	Year-1		1	Winter
1/22/2018	BL	5M	1	2018	Year-1	0	0	Winter
1/22/2018	BL	6M	1	2018	Year-1	0	0	Winter
1/22/2018	BL	7M	1	2018	Year-1	0	0	Winter
1/22/2018	BL	10M	1	2018	Year-1	0	0	Winter
1/22/2018	BL	12M	1	2018	Year-1	0	0	Winter
1/22/2018	BL	14M	1	2018	Year-1		1	Winter
1/22/2018	BL	8A	1	2018	Year-1	0	0	Winter
1/22/2018	BL	9A	1	2018	Year-1	0	0	Winter
1/22/2018	BL	11A	1	2018	Year-1	0	0	Winter
1/22/2018	BL	13A	1	2018	Year-1	0	0	Winter
1/23/2018	BL	1M	1	2018	Year-1	1	0	Winter
1/23/2018	BL	2M	1	2018	Year-1	1	0	Winter
1/23/2018	BL	3M	1	2018	Year-1	0	0	Winter
1/23/2018	BL	4M	1	2018	Year-1		1	Winter
1/23/2018	BL	5M	1	2018	Year-1	0	0	Winter
1/23/2018	BL	6M	1	2018	Year-1	0	0	Winter
1/23/2018	BL	7M	1	2018	Year-1	0	0	Winter
1/23/2018	BL	10M	1	2018	Year-1	0	0	Winter
1/23/2018	BL	12M	1	2018	Year-1	0	0	Winter
1/23/2018	BL	14M	1	2018	Year-1		1	Winter
1/23/2018	BL	8A	1	2018	Year-1	0	0	Winter
1/23/2018	BL	9A	1	2018	Year-1	0	0	Winter
1/23/2018	BL	11A	1	2018	Year-1	0	0	Winter
1/23/2018	BL	13A	1	2018	Year-1	0	0	Winter
1/24/2018	BL	1M	1	2018	Year-1	0	0	Winter

Date	Species	Site	Month	Year	Survey Year	Presence	Data Gap	Season
1/24/2018	BL	2M	1	2018	Year-1	0	0	Winter
1/24/2018	BL	3M	1	2018	Year-1	0	0	Winter
1/24/2018	BL	4M	1	2018	Year-1		1	Winter
1/24/2018	BL	5M	1	2018	Year-1	0	0	Winter
1/24/2018	BL	6M	1	2018	Year-1	0	0	Winter
1/24/2018	BL	7M	1	2018	Year-1	0	0	Winter
1/24/2018	BL	10M	1	2018	Year-1	0	0	Winter
1/24/2018	BL	12M	1	2018	Year-1	0	0	Winter
1/24/2018	BL	14M	1	2018	Year-1		1	Winter
1/24/2018	BL	8A	1	2018	Year-1	0	0	Winter
1/24/2018	BL	9A	1	2018	Year-1	0	0	Winter
1/24/2018	BL	11A	1	2018	Year-1	0	0	Winter
1/24/2018	BL	13A	1	2018	Year-1	0	0	Winter
1/25/2018	BL	1M	1	2018	Year-1	0	0	Winter
1/25/2018	BL	2M	1	2018	Year-1	0	0	Winter
1/25/2018	BL	3M	1	2018	Year-1	0	0	Winter
1/25/2018	BL	4M	1	2018	Year-1		1	Winter
1/25/2018	BL	5M	1	2018	Year-1	0	0	Winter
1/25/2018	BL	6M	1	2018	Year-1	0	0	Winter
1/25/2018	BL	7M	1	2018	Year-1	0	0	Winter
1/25/2018	BL	10M	1	2018	Year-1	0	0	Winter
1/25/2018	BL	12M	1	2018	Year-1	0	0	Winter
1/25/2018	BL	14M	1	2018	Year-1		1	Winter
1/25/2018	BL	8A	1	2018	Year-1	0	0	Winter
1/25/2018	BL	9A	1	2018	Year-1	0	0	Winter
1/25/2018	BL	11A	1	2018	Year-1	0	0	Winter
1/25/2018	BL	13A	1	2018	Year-1	0	0	Winter
1/26/2018	BL	1M	1	2018	Year-1	0	0	Winter
1/26/2018	BL	2M	1	2018	Year-1	0	0	Winter
1/26/2018	BL	3M	1	2018	Year-1	0	0	Winter
1/26/2018	BL	4M	1	2018	Year-1		1	Winter
1/26/2018	BL	5M	1	2018	Year-1	0	0	Winter
1/26/2018	BL	6M	1	2018	Year-1	0	0	Winter
1/26/2018	BL	7M	1	2018	Year-1	0	0	Winter
1/26/2018	BL	10M	1	2018	Year-1	0	0	Winter
1/26/2018	BL	12M	1	2018	Year-1	0	0	Winter
1/26/2018	BL	14M	1	2018	Year-1		1	Winter
1/26/2018	BL	8A	1	2018	Year-1	0	0	Winter
1/26/2018	BL	9A	1	2018	Year-1	0	0	Winter
1/26/2018	BL	11A	1	2018	Year-1	0	0	Winter
1/26/2018	BL	13A	1	2018	Year-1	0	0	Winter
1/27/2018	BL	1M	1	2018	Year-1	0	0	Winter
1/27/2018	BL	2M	1	2018	Year-1	0	0	Winter
1/27/2018	BL	3M	1	2018	Year-1	0	0	Winter

Date	Species	Site	Month	Year	Survey Year	Presence	Data Gap	Season
1/27/2018	BL	4M	1	2018	Year-1		1	Winter
1/27/2018	BL	5M	1	2018	Year-1	0	0	Winter
1/27/2018	BL	6M	1	2018	Year-1	0	0	Winter
1/27/2018	BL	7M	1	2018	Year-1	0	0	Winter
1/27/2018	BL	10M	1	2018	Year-1	0	0	Winter
1/27/2018	BL	12M	1	2018	Year-1	0	0	Winter
1/27/2018	BL	14M	1	2018	Year-1		1	Winter
1/27/2018	BL	8A	1	2018	Year-1	0	0	Winter
1/27/2018	BL	9A	1	2018	Year-1	0	0	Winter
1/27/2018	BL	11A	1	2018	Year-1	0	0	Winter
1/27/2018	BL	13A	1	2018	Year-1	0	0	Winter
1/28/2018	BL	1M	1	2018	Year-1	1	0	Winter
1/28/2018	BL	2M	1	2018	Year-1	0	0	Winter
1/28/2018	BL	3M	1	2018	Year-1	0	0	Winter
1/28/2018	BL	4M	1	2018	Year-1		1	Winter
1/28/2018	BL	5M	1	2018	Year-1	0	0	Winter
1/28/2018	BL	6M	1	2018	Year-1	0	0	Winter
1/28/2018	BL	7M	1	2018	Year-1	0	0	Winter
1/28/2018	BL	10M	1	2018	Year-1	0	0	Winter
1/28/2018	BL	12M	1	2018	Year-1	0	0	Winter
1/28/2018	BL	14M	1	2018	Year-1		1	Winter
1/28/2018	BL	8A	1	2018	Year-1	0	0	Winter
1/28/2018	BL	9A	1	2018	Year-1	0	0	Winter
1/28/2018	BL	11A	1	2018	Year-1	0	0	Winter
1/28/2018	BL	13A	1	2018	Year-1	0	0	Winter
1/29/2018	BL	1M	1	2018	Year-1	0	0	Winter
1/29/2018	BL	2M	1	2018	Year-1	0	0	Winter
1/29/2018	BL	3M	1	2018	Year-1	0	0	Winter
1/29/2018	BL	4M	1	2018	Year-1		1	Winter
1/29/2018	BL	5M	1	2018	Year-1	0	0	Winter
1/29/2018	BL	6M	1	2018	Year-1	0	0	Winter
1/29/2018	BL	7M	1	2018	Year-1	0	0	Winter
1/29/2018	BL	10M	1	2018	Year-1	0	0	Winter
1/29/2018	BL	12M	1	2018	Year-1	0	0	Winter
1/29/2018	BL	14M	1	2018	Year-1		1	Winter
1/29/2018	BL	8A	1	2018	Year-1	0	0	Winter
1/29/2018	BL	9A	1	2018	Year-1	0	0	Winter
1/29/2018	BL	11A	1	2018	Year-1	0	0	Winter
1/29/2018	BL	13A	1	2018	Year-1	0	0	Winter
1/30/2018	BL	1M	1	2018	Year-1	0	0	Winter
1/30/2018	BL	2M	1	2018	Year-1	0	0	Winter
1/30/2018	BL	3M	1	2018	Year-1	0	0	Winter
1/30/2018	BL	4M	1	2018	Year-1		1	Winter
1/30/2018	BL	5M	1	2018	Year-1	0	0	Winter

Date	Species	Site	Month	Year	Survey Year	Presence	Data Gap	Season
1/30/2018	BL	6M	1	2018	Year-1	0	0	Winter
1/30/2018	BL	7M	1	2018	Year-1	0	0	Winter
1/30/2018	BL	10M	1	2018	Year-1	0	0	Winter
1/30/2018	BL	12M	1	2018	Year-1	0	0	Winter
1/30/2018	BL	14M	1	2018	Year-1		1	Winter
1/30/2018	BL	8A	1	2018	Year-1	0	0	Winter
1/30/2018	BL	9A	1	2018	Year-1	0	0	Winter
1/30/2018	BL	11A	1	2018	Year-1	0	0	Winter
1/30/2018	BL	13A	1	2018	Year-1	0	0	Winter
1/31/2018	BL	1M	1	2018	Year-1	0	0	Winter
1/31/2018	BL	2M	1	2018	Year-1	0	0	Winter
1/31/2018	BL	3M	1	2018	Year-1	0	0	Winter
1/31/2018	BL	4M	1	2018	Year-1		1	Winter
1/31/2018	BL	5M	1	2018	Year-1	0	0	Winter
1/31/2018	BL	6M	1	2018	Year-1	0	0	Winter
1/31/2018	BL	7M	1	2018	Year-1	0	0	Winter
1/31/2018	BL	10M	1	2018	Year-1	0	0	Winter
1/31/2018	BL	12M	1	2018	Year-1	0	0	Winter
1/31/2018	BL	14M	1	2018	Year-1		1	Winter
1/31/2018	BL	8A	1	2018	Year-1	0	0	Winter
1/31/2018	BL	9A	1	2018	Year-1	0	0	Winter
1/31/2018	BL	11A	1	2018	Year-1	0	0	Winter
1/31/2018	BL	13A	1	2018	Year-1	0	0	Winter
2/1/2018	BL	1M	2	2018	Year-1	0	0	Winter
2/1/2018	BL	2M	2	2018	Year-1	0	0	Winter
2/1/2018	BL	3M	2	2018	Year-1	0	0	Winter
2/1/2018	BL	4M	2	2018	Year-1		1	Winter
2/1/2018	BL	5M	2	2018	Year-1	0	0	Winter
2/1/2018	BL	6M	2	2018	Year-1	0	0	Winter
2/1/2018	BL	7M	2	2018	Year-1	0	0	Winter
2/1/2018	BL	10M	2	2018	Year-1	0	0	Winter
2/1/2018	BL	12M	2	2018	Year-1	0	0	Winter
2/1/2018	BL	14M	2	2018	Year-1		1	Winter
2/1/2018	BL	8A	2	2018	Year-1	0	0	Winter
2/1/2018	BL	9A	2	2018	Year-1	0	0	Winter
2/1/2018	BL	11A	2	2018	Year-1	0	0	Winter
2/1/2018	BL	13A	2	2018	Year-1	0	0	Winter
2/2/2018	BL	1M	2	2018	Year-1	0	0	Winter
2/2/2018	BL	2M	2	2018	Year-1	0	0	Winter
2/2/2018	BL	3M	2	2018	Year-1	0	0	Winter
2/2/2018	BL	4M	2	2018	Year-1		1	Winter
2/2/2018	BL	5M	2	2018	Year-1	0	0	Winter
2/2/2018	BL	6M	2	2018	Year-1	0	0	Winter
2/2/2018	BL	7M	2	2018	Year-1	0	0	Winter

Date	Species	Site	Month	Year	Survey Year	Presence	Data Gap	Season
2/2/2018	BL	10M	2	2018	Year-1	0	0	Winter
2/2/2018	BL	12M	2	2018	Year-1	0	0	Winter
2/2/2018	BL	14M	2	2018	Year-1		1	Winter
2/2/2018	BL	8A	2	2018	Year-1	0	0	Winter
2/2/2018	BL	9A	2	2018	Year-1	0	0	Winter
2/2/2018	BL	11A	2	2018	Year-1	0	0	Winter
2/2/2018	BL	13A	2	2018	Year-1	0	0	Winter
2/3/2018	BL	1M	2	2018	Year-1	0	0	Winter
2/3/2018	BL	2M	2	2018	Year-1	0	0	Winter
2/3/2018	BL	3M	2	2018	Year-1	0	0	Winter
2/3/2018	BL	4M	2	2018	Year-1		1	Winter
2/3/2018	BL	5M	2	2018	Year-1	0	0	Winter
2/3/2018	BL	6M	2	2018	Year-1	0	0	Winter
2/3/2018	BL	7M	2	2018	Year-1	0	0	Winter
2/3/2018	BL	10M	2	2018	Year-1	0	0	Winter
2/3/2018	BL	12M	2	2018	Year-1	0	0	Winter
2/3/2018	BL	14M	2	2018	Year-1		1	Winter
2/3/2018	BL	8A	2	2018	Year-1	0	0	Winter
2/3/2018	BL	9A	2	2018	Year-1	0	0	Winter
2/3/2018	BL	11A	2	2018	Year-1	0	0	Winter
2/3/2018	BL	13A	2	2018	Year-1	0	0	Winter
2/4/2018	BL	1M	2	2018	Year-1	0	0	Winter
2/4/2018	BL	2M	2	2018	Year-1	0	0	Winter
2/4/2018	BL	3M	2	2018	Year-1	0	0	Winter
2/4/2018	BL	4M	2	2018	Year-1		1	Winter
2/4/2018	BL	5M	2	2018	Year-1	0	0	Winter
2/4/2018	BL	6M	2	2018	Year-1	0	0	Winter
2/4/2018	BL	7M	2	2018	Year-1	0	0	Winter
2/4/2018	BL	10M	2	2018	Year-1	0	0	Winter
2/4/2018	BL	12M	2	2018	Year-1	0	0	Winter
2/4/2018	BL	14M	2	2018	Year-1		1	Winter
2/4/2018	BL	8A	2	2018	Year-1	0	0	Winter
2/4/2018	BL	9A	2	2018	Year-1	0	0	Winter
2/4/2018	BL	11A	2	2018	Year-1	0	0	Winter
2/4/2018	BL	13A	2	2018	Year-1	0	0	Winter
2/5/2018	BL	1M	2	2018	Year-1	0	0	Winter
2/5/2018	BL	2M	2	2018	Year-1	0	0	Winter
2/5/2018	BL	3M	2	2018	Year-1	0	0	Winter
2/5/2018	BL	4M	2	2018	Year-1		1	Winter
2/5/2018	BL	5M	2	2018	Year-1	0	0	Winter
2/5/2018	BL	6M	2	2018	Year-1	0	0	Winter
2/5/2018	BL	7M	2	2018	Year-1	0	0	Winter
2/5/2018	BL	10M	2	2018	Year-1	0	0	Winter
2/5/2018	BL	12M	2	2018	Year-1	0	0	Winter

Date	Species	Site	Month	Year	Survey Year	Presence	Data Gap	Season
2/5/2018	BL	14M	2	2018	Year-1		1	Winter
2/5/2018	BL	8A	2	2018	Year-1	0	0	Winter
2/5/2018	BL	9A	2	2018	Year-1	0	0	Winter
2/5/2018	BL	11A	2	2018	Year-1	0	0	Winter
2/5/2018	BL	13A	2	2018	Year-1	0	0	Winter
2/6/2018	BL	1M	2	2018	Year-1	0	0	Winter
2/6/2018	BL	2M	2	2018	Year-1	0	0	Winter
2/6/2018	BL	3M	2	2018	Year-1	0	0	Winter
2/6/2018	BL	4M	2	2018	Year-1		1	Winter
2/6/2018	BL	5M	2	2018	Year-1	0	0	Winter
2/6/2018	BL	6M	2	2018	Year-1	0	0	Winter
2/6/2018	BL	7M	2	2018	Year-1	0	0	Winter
2/6/2018	BL	10M	2	2018	Year-1	0	0	Winter
2/6/2018	BL	12M	2	2018	Year-1	0	0	Winter
2/6/2018	BL	14M	2	2018	Year-1		1	Winter
2/6/2018	BL	8A	2	2018	Year-1	0	0	Winter
2/6/2018	BL	9A	2	2018	Year-1	0	0	Winter
2/6/2018	BL	11A	2	2018	Year-1	0	0	Winter
2/6/2018	BL	13A	2	2018	Year-1	0	0	Winter
2/7/2018	BL	1M	2	2018	Year-1	0	0	Winter
2/7/2018	BL	2M	2	2018	Year-1	0	0	Winter
2/7/2018	BL	3M	2	2018	Year-1	0	0	Winter
2/7/2018	BL	4M	2	2018	Year-1		1	Winter
2/7/2018	BL	5M	2	2018	Year-1	0	0	Winter
2/7/2018	BL	6M	2	2018	Year-1	0	0	Winter
2/7/2018	BL	7M	2	2018	Year-1	0	0	Winter
2/7/2018	BL	10M	2	2018	Year-1	0	0	Winter
2/7/2018	BL	12M	2	2018	Year-1	0	0	Winter
2/7/2018	BL	14M	2	2018	Year-1		1	Winter
2/7/2018	BL	8A	2	2018	Year-1	0	0	Winter
2/7/2018	BL	9A	2	2018	Year-1	0	0	Winter
2/7/2018	BL	11A	2	2018	Year-1	0	0	Winter
2/7/2018	BL	13A	2	2018	Year-1	0	0	Winter
2/8/2018	BL	1M	2	2018	Year-1	1	0	Winter
2/8/2018	BL	2M	2	2018	Year-1	1	0	Winter
2/8/2018	BL	3M	2	2018	Year-1	1	0	Winter
2/8/2018	BL	4M	2	2018	Year-1		1	Winter
2/8/2018	BL	5M	2	2018	Year-1	0	0	Winter
2/8/2018	BL	6M	2	2018	Year-1	0	0	Winter
2/8/2018	BL	7M	2	2018	Year-1	0	0	Winter
2/8/2018	BL	10M	2	2018	Year-1	0	0	Winter
2/8/2018	BL	12M	2	2018	Year-1	0	0	Winter
2/8/2018	BL	14M	2	2018	Year-1		1	Winter
2/8/2018	BL	8A	2	2018	Year-1	0	0	Winter

Date	Species	Site	Month	Year	Survey Year	Presence	Data Gap	Season
2/8/2018	BL	9A	2	2018	Year-1	0	0	Winter
2/8/2018	BL	11A	2	2018	Year-1	0	0	Winter
2/8/2018	BL	13A	2	2018	Year-1	0	0	Winter
2/9/2018	BL	1M	2	2018	Year-1	1	0	Winter
2/9/2018	BL	2M	2	2018	Year-1	1	0	Winter
2/9/2018	BL	3M	2	2018	Year-1	1	0	Winter
2/9/2018	BL	4M	2	2018	Year-1		1	Winter
2/9/2018	BL	5M	2	2018	Year-1	0	0	Winter
2/9/2018	BL	6M	2	2018	Year-1	0	0	Winter
2/9/2018	BL	7M	2	2018	Year-1	0	0	Winter
2/9/2018	BL	10M	2	2018	Year-1	0	0	Winter
2/9/2018	BL	12M	2	2018	Year-1	0	0	Winter
2/9/2018	BL	14M	2	2018	Year-1		1	Winter
2/9/2018	BL	8A	2	2018	Year-1	0	0	Winter
2/9/2018	BL	9A	2	2018	Year-1	0	0	Winter
2/9/2018	BL	11A	2	2018	Year-1	0	0	Winter
2/9/2018	BL	13A	2	2018	Year-1	1	0	Winter
2/10/2018	BL	1M	2	2018	Year-1	1	0	Winter
2/10/2018	BL	2M	2	2018	Year-1	1	0	Winter
2/10/2018	BL	3M	2	2018	Year-1	1	0	Winter
2/10/2018	BL	4M	2	2018	Year-1		1	Winter
2/10/2018	BL	5M	2	2018	Year-1	0	0	Winter
2/10/2018	BL	6M	2	2018	Year-1	0	0	Winter
2/10/2018	BL	7M	2	2018	Year-1	0	0	Winter
2/10/2018	BL	10M	2	2018	Year-1	0	0	Winter
2/10/2018	BL	12M	2	2018	Year-1	0	0	Winter
2/10/2018	BL	14M	2	2018	Year-1		1	Winter
2/10/2018	BL	8A	2	2018	Year-1	0	0	Winter
2/10/2018	BL	9A	2	2018	Year-1	0	0	Winter
2/10/2018	BL	11A	2	2018	Year-1	0	0	Winter
2/10/2018	BL	13A	2	2018	Year-1	0	0	Winter
2/11/2018	BL	1M	2	2018	Year-1	1	0	Winter
2/11/2018	BL	2M	2	2018	Year-1	1	0	Winter
2/11/2018	BL	3M	2	2018	Year-1	1	0	Winter
2/11/2018	BL	4M	2	2018	Year-1		1	Winter
2/11/2018	BL	5M	2	2018	Year-1	0	0	Winter
2/11/2018	BL	6M	2	2018	Year-1	0	0	Winter
2/11/2018	BL	7M	2	2018	Year-1	0	0	Winter
2/11/2018	BL	10M	2	2018	Year-1	0	0	Winter
2/11/2018	BL	12M	2	2018	Year-1	0	0	Winter
2/11/2018	BL	14M	2	2018	Year-1		1	Winter
2/11/2018	BL	8A	2	2018	Year-1	0	0	Winter
2/11/2018	BL	9A	2	2018	Year-1	0	0	Winter
2/11/2018	BL	11A	2	2018	Year-1	0	0	Winter

Date	Species	Site	Month	Year	Survey Year	Presence	Data Gap	Season
2/11/2018	BL	13A	2	2018	Year-1	0	0	Winter
2/12/2018	BL	1M	2	2018	Year-1	1	0	Winter
2/12/2018	BL	2M	2	2018	Year-1	1	0	Winter
2/12/2018	BL	3M	2	2018	Year-1	0	0	Winter
2/12/2018	BL	4M	2	2018	Year-1		1	Winter
2/12/2018	BL	5M	2	2018	Year-1	0	0	Winter
2/12/2018	BL	6M	2	2018	Year-1	0	0	Winter
2/12/2018	BL	7M	2	2018	Year-1	0	0	Winter
2/12/2018	BL	10M	2	2018	Year-1	0	0	Winter
2/12/2018	BL	12M	2	2018	Year-1	0	0	Winter
2/12/2018	BL	14M	2	2018	Year-1		1	Winter
2/12/2018	BL	8A	2	2018	Year-1	0	0	Winter
2/12/2018	BL	9A	2	2018	Year-1	0	0	Winter
2/12/2018	BL	11A	2	2018	Year-1	0	0	Winter
2/12/2018	BL	13A	2	2018	Year-1	0	0	Winter
2/13/2018	BL	1M	2	2018	Year-1	0	0	Winter
2/13/2018	BL	2M	2	2018	Year-1	0	0	Winter
2/13/2018	BL	3M	2	2018	Year-1	0	0	Winter
2/13/2018	BL	4M	2	2018	Year-1		1	Winter
2/13/2018	BL	5M	2	2018	Year-1	0	0	Winter
2/13/2018	BL	6M	2	2018	Year-1	0	0	Winter
2/13/2018	BL	7M	2	2018	Year-1	0	0	Winter
2/13/2018	BL	10M	2	2018	Year-1	0	0	Winter
2/13/2018	BL	12M	2	2018	Year-1	0	0	Winter
2/13/2018	BL	14M	2	2018	Year-1		1	Winter
2/13/2018	BL	8A	2	2018	Year-1	0	0	Winter
2/13/2018	BL	9A	2	2018	Year-1	0	0	Winter
2/13/2018	BL	11A	2	2018	Year-1	0	0	Winter
2/13/2018	BL	13A	2	2018	Year-1	0	0	Winter
2/14/2018	BL	1M	2	2018	Year-1	0	0	Winter
2/14/2018	BL	2M	2	2018	Year-1	0	0	Winter
2/14/2018	BL	3M	2	2018	Year-1	0	0	Winter
2/14/2018	BL	4M	2	2018	Year-1		1	Winter
2/14/2018	BL	5M	2	2018	Year-1	0	0	Winter
2/14/2018	BL	6M	2	2018	Year-1	0	0	Winter
2/14/2018	BL	7M	2	2018	Year-1	0	0	Winter
2/14/2018	BL	10M	2	2018	Year-1	0	0	Winter
2/14/2018	BL	12M	2	2018	Year-1	0	0	Winter
2/14/2018	BL	14M	2	2018	Year-1		1	Winter
2/14/2018	BL	8A	2	2018	Year-1	0	0	Winter
2/14/2018	BL	9A	2	2018	Year-1	0	0	Winter
2/14/2018	BL	11A	2	2018	Year-1	0	0	Winter
2/14/2018	BL	13A	2	2018	Year-1	0	0	Winter
2/15/2018	BL	1M	2	2018	Year-1	0	0	Winter

Date	Species	Site	Month	Year	Survey Year	Presence	Data Gap	Season
2/15/2018	BL	2M	2	2018	Year-1	1	0	Winter
2/15/2018	BL	3M	2	2018	Year-1	0	0	Winter
2/15/2018	BL	4M	2	2018	Year-1		1	Winter
2/15/2018	BL	5M	2	2018	Year-1	0	0	Winter
2/15/2018	BL	6M	2	2018	Year-1	0	0	Winter
2/15/2018	BL	7M	2	2018	Year-1	0	0	Winter
2/15/2018	BL	10M	2	2018	Year-1	0	0	Winter
2/15/2018	BL	12M	2	2018	Year-1	0	0	Winter
2/15/2018	BL	14M	2	2018	Year-1		1	Winter
2/15/2018	BL	8A	2	2018	Year-1	0	0	Winter
2/15/2018	BL	9A	2	2018	Year-1	0	0	Winter
2/15/2018	BL	11A	2	2018	Year-1	0	0	Winter
2/15/2018	BL	13A	2	2018	Year-1	0	0	Winter
2/16/2018	BL	1M	2	2018	Year-1	0	0	Winter
2/16/2018	BL	2M	2	2018	Year-1	0	0	Winter
2/16/2018	BL	3M	2	2018	Year-1	0	0	Winter
2/16/2018	BL	4M	2	2018	Year-1		1	Winter
2/16/2018	BL	5M	2	2018	Year-1	0	0	Winter
2/16/2018	BL	6M	2	2018	Year-1	0	0	Winter
2/16/2018	BL	7M	2	2018	Year-1	0	0	Winter
2/16/2018	BL	10M	2	2018	Year-1	0	0	Winter
2/16/2018	BL	12M	2	2018	Year-1	0	0	Winter
2/16/2018	BL	14M	2	2018	Year-1		1	Winter
2/16/2018	BL	8A	2	2018	Year-1	0	0	Winter
2/16/2018	BL	9A	2	2018	Year-1	0	0	Winter
2/16/2018	BL	11A	2	2018	Year-1	0	0	Winter
2/16/2018	BL	13A	2	2018	Year-1	0	0	Winter
2/17/2018	BL	1M	2	2018	Year-1	0	0	Winter
2/17/2018	BL	2M	2	2018	Year-1	0	0	Winter
2/17/2018	BL	3M	2	2018	Year-1	0	0	Winter
2/17/2018	BL	4M	2	2018	Year-1		1	Winter
2/17/2018	BL	5M	2	2018	Year-1	0	0	Winter
2/17/2018	BL	6M	2	2018	Year-1	0	0	Winter
2/17/2018	BL	7M	2	2018	Year-1	0	0	Winter
2/17/2018	BL	10M	2	2018	Year-1	0	0	Winter
2/17/2018	BL	12M	2	2018	Year-1	0	0	Winter
2/17/2018	BL	14M	2	2018	Year-1		1	Winter
2/17/2018	BL	8A	2	2018	Year-1	0	0	Winter
2/17/2018	BL	9A	2	2018	Year-1	0	0	Winter
2/17/2018	BL	11A	2	2018	Year-1	0	0	Winter
2/17/2018	BL	13A	2	2018	Year-1	0	0	Winter
2/18/2018	BL	1M	2	2018	Year-1	0	0	Winter
2/18/2018	BL	2M	2	2018	Year-1	0	0	Winter
2/18/2018	BL	3M	2	2018	Year-1	0	0	Winter

Date	Species	Site	Month	Year	Survey Year	Presence	Data Gap	Season
2/18/2018	BL	4M	2	2018	Year-1		1	Winter
2/18/2018	BL	5M	2	2018	Year-1	0	0	Winter
2/18/2018	BL	6M	2	2018	Year-1	0	0	Winter
2/18/2018	BL	7M	2	2018	Year-1	0	0	Winter
2/18/2018	BL	10M	2	2018	Year-1	0	0	Winter
2/18/2018	BL	12M	2	2018	Year-1	0	0	Winter
2/18/2018	BL	14M	2	2018	Year-1		1	Winter
2/18/2018	BL	8A	2	2018	Year-1	0	0	Winter
2/18/2018	BL	9A	2	2018	Year-1	0	0	Winter
2/18/2018	BL	11A	2	2018	Year-1	0	0	Winter
2/18/2018	BL	13A	2	2018	Year-1	0	0	Winter
2/19/2018	BL	1M	2	2018	Year-1	0	0	Winter
2/19/2018	BL	2M	2	2018	Year-1	0	0	Winter
2/19/2018	BL	3M	2	2018	Year-1	0	0	Winter
2/19/2018	BL	4M	2	2018	Year-1		1	Winter
2/19/2018	BL	5M	2	2018	Year-1	0	0	Winter
2/19/2018	BL	6M	2	2018	Year-1	0	0	Winter
2/19/2018	BL	7M	2	2018	Year-1	0	0	Winter
2/19/2018	BL	10M	2	2018	Year-1	0	0	Winter
2/19/2018	BL	12M	2	2018	Year-1	0	0	Winter
2/19/2018	BL	14M	2	2018	Year-1		1	Winter
2/19/2018	BL	8A	2	2018	Year-1	0	0	Winter
2/19/2018	BL	9A	2	2018	Year-1	0	0	Winter
2/19/2018	BL	11A	2	2018	Year-1	0	0	Winter
2/19/2018	BL	13A	2	2018	Year-1	0	0	Winter
2/20/2018	BL	1M	2	2018	Year-1	0	0	Winter
2/20/2018	BL	2M	2	2018	Year-1	0	0	Winter
2/20/2018	BL	3M	2	2018	Year-1	0	0	Winter
2/20/2018	BL	4M	2	2018	Year-1		1	Winter
2/20/2018	BL	5M	2	2018	Year-1	0	0	Winter
2/20/2018	BL	6M	2	2018	Year-1	0	0	Winter
2/20/2018	BL	7M	2	2018	Year-1	0	0	Winter
2/20/2018	BL	10M	2	2018	Year-1	0	0	Winter
2/20/2018	BL	12M	2	2018	Year-1	0	0	Winter
2/20/2018	BL	14M	2	2018	Year-1		1	Winter
2/20/2018	BL	8A	2	2018	Year-1	0	0	Winter
2/20/2018	BL	9A	2	2018	Year-1	0	0	Winter
2/20/2018	BL	11A	2	2018	Year-1	0	0	Winter
2/20/2018	BL	13A	2	2018	Year-1	0	0	Winter
2/21/2018	BL	1M	2	2018	Year-1	0	0	Winter
2/21/2018	BL	2M	2	2018	Year-1	0	0	Winter
2/21/2018	BL	3M	2	2018	Year-1	0	0	Winter
2/21/2018	BL	4M	2	2018	Year-1		1	Winter
2/21/2018	BL	5M	2	2018	Year-1	0	0	Winter

Date	Species	Site	Month	Year	Survey Year	Presence	Data Gap	Season
2/21/2018	BL	6M	2	2018	Year-1	0	0	Winter
2/21/2018	BL	7M	2	2018	Year-1	0	0	Winter
2/21/2018	BL	10M	2	2018	Year-1	0	0	Winter
2/21/2018	BL	12M	2	2018	Year-1	0	0	Winter
2/21/2018	BL	14M	2	2018	Year-1		1	Winter
2/21/2018	BL	8A	2	2018	Year-1	0	0	Winter
2/21/2018	BL	9A	2	2018	Year-1	0	0	Winter
2/21/2018	BL	11A	2	2018	Year-1	0	0	Winter
2/21/2018	BL	13A	2	2018	Year-1	0	0	Winter
2/22/2018	BL	1M	2	2018	Year-1	0	0	Winter
2/22/2018	BL	2M	2	2018	Year-1	0	0	Winter
2/22/2018	BL	3M	2	2018	Year-1	0	0	Winter
2/22/2018	BL	4M	2	2018	Year-1		1	Winter
2/22/2018	BL	5M	2	2018	Year-1	0	0	Winter
2/22/2018	BL	6M	2	2018	Year-1	0	0	Winter
2/22/2018	BL	7M	2	2018	Year-1	0	0	Winter
2/22/2018	BL	10M	2	2018	Year-1	0	0	Winter
2/22/2018	BL	12M	2	2018	Year-1	0	0	Winter
2/22/2018	BL	14M	2	2018	Year-1		1	Winter
2/22/2018	BL	8A	2	2018	Year-1	0	0	Winter
2/22/2018	BL	9A	2	2018	Year-1	0	0	Winter
2/22/2018	BL	11A	2	2018	Year-1	0	0	Winter
2/22/2018	BL	13A	2	2018	Year-1	0	0	Winter
2/23/2018	BL	1M	2	2018	Year-1	1	0	Winter
2/23/2018	BL	2M	2	2018	Year-1	0	0	Winter
2/23/2018	BL	3M	2	2018	Year-1	0	0	Winter
2/23/2018	BL	4M	2	2018	Year-1		1	Winter
2/23/2018	BL	5M	2	2018	Year-1	0	0	Winter
2/23/2018	BL	6M	2	2018	Year-1	0	0	Winter
2/23/2018	BL	7M	2	2018	Year-1	0	0	Winter
2/23/2018	BL	10M	2	2018	Year-1	0	0	Winter
2/23/2018	BL	12M	2	2018	Year-1	0	0	Winter
2/23/2018	BL	14M	2	2018	Year-1		1	Winter
2/23/2018	BL	8A	2	2018	Year-1	0	0	Winter
2/23/2018	BL	9A	2	2018	Year-1	0	0	Winter
2/23/2018	BL	11A	2	2018	Year-1	0	0	Winter
2/23/2018	BL	13A	2	2018	Year-1	0	0	Winter
2/24/2018	BL	1M	2	2018	Year-1	0	0	Winter
2/24/2018	BL	2M	2	2018	Year-1	0	0	Winter
2/24/2018	BL	3M	2	2018	Year-1	0	0	Winter
2/24/2018	BL	4M	2	2018	Year-1		1	Winter
2/24/2018	BL	5M	2	2018	Year-1		1	Winter
2/24/2018	BL	6M	2	2018	Year-1	0	0	Winter
2/24/2018	BL	7M	2	2018	Year-1	0	0	Winter

Date	Species	Site	Month	Year	Survey Year	Presence	Data Gap	Season
2/24/2018	BL	10M	2	2018	Year-1		1	Winter
2/24/2018	BL	12M	2	2018	Year-1	0	0	Winter
2/24/2018	BL	14M	2	2018	Year-1		1	Winter
2/24/2018	BL	8A	2	2018	Year-1	0	0	Winter
2/24/2018	BL	9A	2	2018	Year-1	0	0	Winter
2/24/2018	BL	11A	2	2018	Year-1	0	0	Winter
2/24/2018	BL	13A	2	2018	Year-1	0	0	Winter
2/25/2018	BL	1M	2	2018	Year-1	0	0	Winter
2/25/2018	BL	2M	2	2018	Year-1	0	0	Winter
2/25/2018	BL	3M	2	2018	Year-1	0	0	Winter
2/25/2018	BL	4M	2	2018	Year-1		1	Winter
2/25/2018	BL	5M	2	2018	Year-1		1	Winter
2/25/2018	BL	6M	2	2018	Year-1	0	0	Winter
2/25/2018	BL	7M	2	2018	Year-1	0	0	Winter
2/25/2018	BL	10M	2	2018	Year-1		1	Winter
2/25/2018	BL	12M	2	2018	Year-1	0	0	Winter
2/25/2018	BL	14M	2	2018	Year-1		1	Winter
2/25/2018	BL	8A	2	2018	Year-1	0	0	Winter
2/25/2018	BL	9A	2	2018	Year-1	0	0	Winter
2/25/2018	BL	11A	2	2018	Year-1	0	0	Winter
2/25/2018	BL	13A	2	2018	Year-1	0	0	Winter
2/26/2018	BL	1M	2	2018	Year-1	0	0	Winter
2/26/2018	BL	2M	2	2018	Year-1	0	0	Winter
2/26/2018	BL	3M	2	2018	Year-1	0	0	Winter
2/26/2018	BL	4M	2	2018	Year-1		1	Winter
2/26/2018	BL	5M	2	2018	Year-1		1	Winter
2/26/2018	BL	6M	2	2018	Year-1	0	0	Winter
2/26/2018	BL	7M	2	2018	Year-1	0	0	Winter
2/26/2018	BL	10M	2	2018	Year-1		1	Winter
2/26/2018	BL	12M	2	2018	Year-1	0	0	Winter
2/26/2018	BL	14M	2	2018	Year-1		1	Winter
2/26/2018	BL	8A	2	2018	Year-1	0	0	Winter
2/26/2018	BL	9A	2	2018	Year-1	0	0	Winter
2/26/2018	BL	11A	2	2018	Year-1	0	0	Winter
2/26/2018	BL	13A	2	2018	Year-1	0	0	Winter
2/27/2018	BL	1M	2	2018	Year-1	1	0	Winter
2/27/2018	BL	2M	2	2018	Year-1	0	0	Winter
2/27/2018	BL	3M	2	2018	Year-1	0	0	Winter
2/27/2018	BL	4M	2	2018	Year-1		1	Winter
2/27/2018	BL	5M	2	2018	Year-1		1	Winter
2/27/2018	BL	6M	2	2018	Year-1	0	0	Winter
2/27/2018	BL	7M	2	2018	Year-1	0	0	Winter
2/27/2018	BL	10M	2	2018	Year-1		1	Winter
2/27/2018	BL	12M	2	2018	Year-1	0	0	Winter

Date	Species	Site	Month	Year	Survey Year	Presence	Data Gap	Season
2/27/2018	BL	14M	2	2018	Year-1		1	Winter
2/27/2018	BL	8A	2	2018	Year-1	0	0	Winter
2/27/2018	BL	9A	2	2018	Year-1	0	0	Winter
2/27/2018	BL	11A	2	2018	Year-1	0	0	Winter
2/27/2018	BL	13A	2	2018	Year-1	0	0	Winter
2/28/2018	BL	1M	2	2018	Year-1	0	0	Winter
2/28/2018	BL	2M	2	2018	Year-1	0	0	Winter
2/28/2018	BL	3M	2	2018	Year-1	0	0	Winter
2/28/2018	BL	4M	2	2018	Year-1		1	Winter
2/28/2018	BL	5M	2	2018	Year-1		1	Winter
2/28/2018	BL	6M	2	2018	Year-1	0	0	Winter
2/28/2018	BL	7M	2	2018	Year-1	0	0	Winter
2/28/2018	BL	10M	2	2018	Year-1		1	Winter
2/28/2018	BL	12M	2	2018	Year-1	0	0	Winter
2/28/2018	BL	14M	2	2018	Year-1		1	Winter
2/28/2018	BL	8A	2	2018	Year-1	0	0	Winter
2/28/2018	BL	9A	2	2018	Year-1	0	0	Winter
2/28/2018	BL	11A	2	2018	Year-1	0	0	Winter
2/28/2018	BL	13A	2	2018	Year-1	0	0	Winter
3/1/2018	BL	1M	3	2018	Year-1	0	0	Spring
3/1/2018	BL	2M	3	2018	Year-1	0	0	Spring
3/1/2018	BL	3M	3	2018	Year-1	0	0	Spring
3/1/2018	BL	4M	3	2018	Year-1		1	Spring
3/1/2018	BL	5M	3	2018	Year-1		1	Spring
3/1/2018	BL	6M	3	2018	Year-1	0	0	Spring
3/1/2018	BL	7M	3	2018	Year-1	0	0	Spring
3/1/2018	BL	10M	3	2018	Year-1		1	Spring
3/1/2018	BL	12M	3	2018	Year-1	0	0	Spring
3/1/2018	BL	14M	3	2018	Year-1		1	Spring
3/1/2018	BL	8A	3	2018	Year-1	0	0	Spring
3/1/2018	BL	9A	3	2018	Year-1	0	0	Spring
3/1/2018	BL	11A	3	2018	Year-1	0	0	Spring
3/1/2018	BL	13A	3	2018	Year-1	0	0	Spring
3/2/2018	BL	1M	3	2018	Year-1	0	0	Spring
3/2/2018	BL	2M	3	2018	Year-1	0	0	Spring
3/2/2018	BL	3M	3	2018	Year-1	0	0	Spring
3/2/2018	BL	4M	3	2018	Year-1		1	Spring
3/2/2018	BL	5M	3	2018	Year-1		1	Spring
3/2/2018	BL	6M	3	2018	Year-1	0	0	Spring
3/2/2018	BL	7M	3	2018	Year-1	0	0	Spring
3/2/2018	BL	10M	3	2018	Year-1		1	Spring
3/2/2018	BL	12M	3	2018	Year-1	0	0	Spring
3/2/2018	BL	14M	3	2018	Year-1		1	Spring
3/2/2018	BL	8A	3	2018	Year-1	0	0	Spring

Date	Species	Site	Month	Year	Survey Year	Presence	Data Gap	Season
3/2/2018	BL	9A	3	2018	Year-1	0	0	Spring
3/2/2018	BL	11A	3	2018	Year-1	0	0	Spring
3/2/2018	BL	13A	3	2018	Year-1	0	0	Spring
3/3/2018	BL	1M	3	2018	Year-1	0	0	Spring
3/3/2018	BL	2M	3	2018	Year-1	0	0	Spring
3/3/2018	BL	3M	3	2018	Year-1	0	0	Spring
3/3/2018	BL	4M	3	2018	Year-1		1	Spring
3/3/2018	BL	5M	3	2018	Year-1		1	Spring
3/3/2018	BL	6M	3	2018	Year-1	0	0	Spring
3/3/2018	BL	7M	3	2018	Year-1	0	0	Spring
3/3/2018	BL	10M	3	2018	Year-1		1	Spring
3/3/2018	BL	12M	3	2018	Year-1	0	0	Spring
3/3/2018	BL	14M	3	2018	Year-1		1	Spring
3/3/2018	BL	8A	3	2018	Year-1	0	0	Spring
3/3/2018	BL	9A	3	2018	Year-1	0	0	Spring
3/3/2018	BL	11A	3	2018	Year-1	0	0	Spring
3/3/2018	BL	13A	3	2018	Year-1	0	0	Spring
3/4/2018	BL	1M	3	2018	Year-1	0	0	Spring
3/4/2018	BL	2M	3	2018	Year-1	0	0	Spring
3/4/2018	BL	3M	3	2018	Year-1	0	0	Spring
3/4/2018	BL	4M	3	2018	Year-1		1	Spring
3/4/2018	BL	5M	3	2018	Year-1		1	Spring
3/4/2018	BL	6M	3	2018	Year-1	0	0	Spring
3/4/2018	BL	7M	3	2018	Year-1	0	0	Spring
3/4/2018	BL	10M	3	2018	Year-1		1	Spring
3/4/2018	BL	12M	3	2018	Year-1	0	0	Spring
3/4/2018	BL	14M	3	2018	Year-1		1	Spring
3/4/2018	BL	8A	3	2018	Year-1	0	0	Spring
3/4/2018	BL	9A	3	2018	Year-1	0	0	Spring
3/4/2018	BL	11A	3	2018	Year-1	0	0	Spring
3/4/2018	BL	13A	3	2018	Year-1	0	0	Spring
3/5/2018	BL	1M	3	2018	Year-1	0	0	Spring
3/5/2018	BL	2M	3	2018	Year-1	0	0	Spring
3/5/2018	BL	3M	3	2018	Year-1	0	0	Spring
3/5/2018	BL	4M	3	2018	Year-1		1	Spring
3/5/2018	BL	5M	3	2018	Year-1		1	Spring
3/5/2018	BL	6M	3	2018	Year-1	0	0	Spring
3/5/2018	BL	7M	3	2018	Year-1	0	0	Spring
3/5/2018	BL	10M	3	2018	Year-1		1	Spring
3/5/2018	BL	12M	3	2018	Year-1	0	0	Spring
3/5/2018	BL	14M	3	2018	Year-1		1	Spring
3/5/2018	BL	8A	3	2018	Year-1	0	0	Spring
3/5/2018	BL	9A	3	2018	Year-1	0	0	Spring
3/5/2018	BL	11A	3	2018	Year-1	0	0	Spring

Date	Species	Site	Month	Year	Survey Year	Presence	Data Gap	Season
3/5/2018	BL	13A	3	2018	Year-1	0	0	Spring
3/6/2018	BL	1M	3	2018	Year-1	0	0	Spring
3/6/2018	BL	2M	3	2018	Year-1	0	0	Spring
3/6/2018	BL	3M	3	2018	Year-1	0	0	Spring
3/6/2018	BL	4M	3	2018	Year-1		1	Spring
3/6/2018	BL	5M	3	2018	Year-1		1	Spring
3/6/2018	BL	6M	3	2018	Year-1	0	0	Spring
3/6/2018	BL	7M	3	2018	Year-1	0	0	Spring
3/6/2018	BL	10M	3	2018	Year-1		1	Spring
3/6/2018	BL	12M	3	2018	Year-1	0	0	Spring
3/6/2018	BL	14M	3	2018	Year-1		1	Spring
3/6/2018	BL	8A	3	2018	Year-1	0	0	Spring
3/6/2018	BL	9A	3	2018	Year-1	0	0	Spring
3/6/2018	BL	11A	3	2018	Year-1	0	0	Spring
3/6/2018	BL	13A	3	2018	Year-1	0	0	Spring
3/7/2018	BL	1M	3	2018	Year-1	0	0	Spring
3/7/2018	BL	2M	3	2018	Year-1	0	0	Spring
3/7/2018	BL	3M	3	2018	Year-1	0	0	Spring
3/7/2018	BL	4M	3	2018	Year-1		1	Spring
3/7/2018	BL	5M	3	2018	Year-1		1	Spring
3/7/2018	BL	6M	3	2018	Year-1	0	0	Spring
3/7/2018	BL	7M	3	2018	Year-1	0	0	Spring
3/7/2018	BL	10M	3	2018	Year-1		1	Spring
3/7/2018	BL	12M	3	2018	Year-1	0	0	Spring
3/7/2018	BL	14M	3	2018	Year-1		1	Spring
3/7/2018	BL	8A	3	2018	Year-1	0	0	Spring
3/7/2018	BL	9A	3	2018	Year-1	0	0	Spring
3/7/2018	BL	11A	3	2018	Year-1	0	0	Spring
3/7/2018	BL	13A	3	2018	Year-1	0	0	Spring
3/8/2018	BL	1M	3	2018	Year-1	0	0	Spring
3/8/2018	BL	2M	3	2018	Year-1	0	0	Spring
3/8/2018	BL	3M	3	2018	Year-1	0	0	Spring
3/8/2018	BL	4M	3	2018	Year-1		1	Spring
3/8/2018	BL	5M	3	2018	Year-1		1	Spring
3/8/2018	BL	6M	3	2018	Year-1	0	0	Spring
3/8/2018	BL	7M	3	2018	Year-1	0	0	Spring
3/8/2018	BL	10M	3	2018	Year-1		1	Spring
3/8/2018	BL	12M	3	2018	Year-1	0	0	Spring
3/8/2018	BL	14M	3	2018	Year-1		1	Spring
3/8/2018	BL	8A	3	2018	Year-1	0	0	Spring
3/8/2018	BL	9A	3	2018	Year-1	0	0	Spring
3/8/2018	BL	11A	3	2018	Year-1	0	0	Spring
3/8/2018	BL	13A	3	2018	Year-1	0	0	Spring
3/9/2018	BL	1M	3	2018	Year-1	0	0	Spring

Date	Species	Site	Month	Year	Survey Year	Presence	Data Gap	Season
3/9/2018	BL	2M	3	2018	Year-1	0	0	Spring
3/9/2018	BL	3M	3	2018	Year-1	0	0	Spring
3/9/2018	BL	4M	3	2018	Year-1		1	Spring
3/9/2018	BL	5M	3	2018	Year-1		1	Spring
3/9/2018	BL	6M	3	2018	Year-1	0	0	Spring
3/9/2018	BL	7M	3	2018	Year-1	0	0	Spring
3/9/2018	BL	10M	3	2018	Year-1		1	Spring
3/9/2018	BL	12M	3	2018	Year-1	0	0	Spring
3/9/2018	BL	14M	3	2018	Year-1		1	Spring
3/9/2018	BL	8A	3	2018	Year-1	0	0	Spring
3/9/2018	BL	9A	3	2018	Year-1	0	0	Spring
3/9/2018	BL	11A	3	2018	Year-1	0	0	Spring
3/9/2018	BL	13A	3	2018	Year-1	0	0	Spring
3/10/2018	BL	1M	3	2018	Year-1	0	0	Spring
3/10/2018	BL	2M	3	2018	Year-1	0	0	Spring
3/10/2018	BL	3M	3	2018	Year-1	0	0	Spring
3/10/2018	BL	4M	3	2018	Year-1		1	Spring
3/10/2018	BL	5M	3	2018	Year-1		1	Spring
3/10/2018	BL	6M	3	2018	Year-1	0	0	Spring
3/10/2018	BL	7M	3	2018	Year-1	0	0	Spring
3/10/2018	BL	10M	3	2018	Year-1		1	Spring
3/10/2018	BL	12M	3	2018	Year-1	0	0	Spring
3/10/2018	BL	14M	3	2018	Year-1		1	Spring
3/10/2018	BL	8A	3	2018	Year-1	0	0	Spring
3/10/2018	BL	9A	3	2018	Year-1	0	0	Spring
3/10/2018	BL	11A	3	2018	Year-1	0	0	Spring
3/10/2018	BL	13A	3	2018	Year-1	0	0	Spring
3/11/2018	BL	1M	3	2018	Year-1	0	0	Spring
3/11/2018	BL	2M	3	2018	Year-1	0	0	Spring
3/11/2018	BL	3M	3	2018	Year-1	0	0	Spring
3/11/2018	BL	4M	3	2018	Year-1		1	Spring
3/11/2018	BL	5M	3	2018	Year-1		1	Spring
3/11/2018	BL	6M	3	2018	Year-1	0	0	Spring
3/11/2018	BL	7M	3	2018	Year-1	0	0	Spring
3/11/2018	BL	10M	3	2018	Year-1		1	Spring
3/11/2018	BL	12M	3	2018	Year-1	0	0	Spring
3/11/2018	BL	14M	3	2018	Year-1		1	Spring
3/11/2018	BL	8A	3	2018	Year-1	0	0	Spring
3/11/2018	BL	9A	3	2018	Year-1	0	0	Spring
3/11/2018	BL	11A	3	2018	Year-1	0	0	Spring
3/11/2018	BL	13A	3	2018	Year-1	0	0	Spring
3/12/2018	BL	1M	3	2018	Year-1	0	0	Spring
3/12/2018	BL	2M	3	2018	Year-1	0	0	Spring
3/12/2018	BL	3M	3	2018	Year-1	0	0	Spring

Date	Species	Site	Month	Year	Survey Year	Presence	Data Gap	Season
3/12/2018	BL	4M	3	2018	Year-1		1	Spring
3/12/2018	BL	5M	3	2018	Year-1		1	Spring
3/12/2018	BL	6M	3	2018	Year-1	0	0	Spring
3/12/2018	BL	7M	3	2018	Year-1	0	0	Spring
3/12/2018	BL	10M	3	2018	Year-1		1	Spring
3/12/2018	BL	12M	3	2018	Year-1	0	0	Spring
3/12/2018	BL	14M	3	2018	Year-1		1	Spring
3/12/2018	BL	8A	3	2018	Year-1	0	0	Spring
3/12/2018	BL	9A	3	2018	Year-1	0	0	Spring
3/12/2018	BL	11A	3	2018	Year-1	0	0	Spring
3/12/2018	BL	13A	3	2018	Year-1	0	0	Spring
3/13/2018	BL	1M	3	2018	Year-1	0	0	Spring
3/13/2018	BL	2M	3	2018	Year-1	0	0	Spring
3/13/2018	BL	3M	3	2018	Year-1	0	0	Spring
3/13/2018	BL	4M	3	2018	Year-1		1	Spring
3/13/2018	BL	5M	3	2018	Year-1		1	Spring
3/13/2018	BL	6M	3	2018	Year-1	0	0	Spring
3/13/2018	BL	7M	3	2018	Year-1	0	0	Spring
3/13/2018	BL	10M	3	2018	Year-1		1	Spring
3/13/2018	BL	12M	3	2018	Year-1		1	Spring
3/13/2018	BL	14M	3	2018	Year-1		1	Spring
3/13/2018	BL	8A	3	2018	Year-1	0	0	Spring
3/13/2018	BL	9A	3	2018	Year-1	0	0	Spring
3/13/2018	BL	11A	3	2018	Year-1	0	0	Spring
3/13/2018	BL	13A	3	2018	Year-1	0	0	Spring
3/14/2018	BL	1M	3	2018	Year-1	0	0	Spring
3/14/2018	BL	2M	3	2018	Year-1	0	0	Spring
3/14/2018	BL	3M	3	2018	Year-1	0	0	Spring
3/14/2018	BL	4M	3	2018	Year-1		1	Spring
3/14/2018	BL	5M	3	2018	Year-1		1	Spring
3/14/2018	BL	6M	3	2018	Year-1	0	0	Spring
3/14/2018	BL	7M	3	2018	Year-1	0	0	Spring
3/14/2018	BL	10M	3	2018	Year-1		1	Spring
3/14/2018	BL	12M	3	2018	Year-1		1	Spring
3/14/2018	BL	14M	3	2018	Year-1		1	Spring
3/14/2018	BL	8A	3	2018	Year-1	0	0	Spring
3/14/2018	BL	9A	3	2018	Year-1	0	0	Spring
3/14/2018	BL	11A	3	2018	Year-1	0	0	Spring
3/14/2018	BL	13A	3	2018	Year-1	0	0	Spring
3/15/2018	BL	1M	3	2018	Year-1	0	0	Spring
3/15/2018	BL	2M	3	2018	Year-1	0	0	Spring
3/15/2018	BL	3M	3	2018	Year-1	0	0	Spring
3/15/2018	BL	4M	3	2018	Year-1		1	Spring
3/15/2018	BL	5M	3	2018	Year-1		1	Spring

Date	Species	Site	Month	Year	Survey Year	Presence	Data Gap	Season
3/15/2018	BL	6M	3	2018	Year-1	0	0	Spring
3/15/2018	BL	7M	3	2018	Year-1	0	0	Spring
3/15/2018	BL	10M	3	2018	Year-1		1	Spring
3/15/2018	BL	12M	3	2018	Year-1		1	Spring
3/15/2018	BL	14M	3	2018	Year-1		1	Spring
3/15/2018	BL	8A	3	2018	Year-1	0	0	Spring
3/15/2018	BL	9A	3	2018	Year-1	0	0	Spring
3/15/2018	BL	11A	3	2018	Year-1	0	0	Spring
3/15/2018	BL	13A	3	2018	Year-1	0	0	Spring
3/16/2018	BL	1M	3	2018	Year-1	0	0	Spring
3/16/2018	BL	2M	3	2018	Year-1	0	0	Spring
3/16/2018	BL	3M	3	2018	Year-1	0	0	Spring
3/16/2018	BL	4M	3	2018	Year-1		1	Spring
3/16/2018	BL	5M	3	2018	Year-1		1	Spring
3/16/2018	BL	6M	3	2018	Year-1	0	0	Spring
3/16/2018	BL	7M	3	2018	Year-1	0	0	Spring
3/16/2018	BL	10M	3	2018	Year-1		1	Spring
3/16/2018	BL	12M	3	2018	Year-1		1	Spring
3/16/2018	BL	14M	3	2018	Year-1		1	Spring
3/16/2018	BL	8A	3	2018	Year-1	0	0	Spring
3/16/2018	BL	9A	3	2018	Year-1	0	0	Spring
3/16/2018	BL	11A	3	2018	Year-1	0	0	Spring
3/16/2018	BL	13A	3	2018	Year-1	0	0	Spring
3/17/2018	BL	1M	3	2018	Year-1	0	0	Spring
3/17/2018	BL	2M	3	2018	Year-1	0	0	Spring
3/17/2018	BL	3M	3	2018	Year-1	0	0	Spring
3/17/2018	BL	4M	3	2018	Year-1		1	Spring
3/17/2018	BL	5M	3	2018	Year-1		1	Spring
3/17/2018	BL	6M	3	2018	Year-1	0	0	Spring
3/17/2018	BL	7M	3	2018	Year-1	0	0	Spring
3/17/2018	BL	10M	3	2018	Year-1		1	Spring
3/17/2018	BL	12M	3	2018	Year-1		1	Spring
3/17/2018	BL	14M	3	2018	Year-1		1	Spring
3/17/2018	BL	8A	3	2018	Year-1	0	0	Spring
3/17/2018	BL	9A	3	2018	Year-1	0	0	Spring
3/17/2018	BL	11A	3	2018	Year-1	0	0	Spring
3/17/2018	BL	13A	3	2018	Year-1	0	0	Spring
3/18/2018	BL	1M	3	2018	Year-1	0	0	Spring
3/18/2018	BL	2M	3	2018	Year-1		1	Spring
3/18/2018	BL	3M	3	2018	Year-1	0	0	Spring
3/18/2018	BL	4M	3	2018	Year-1		1	Spring
3/18/2018	BL	5M	3	2018	Year-1		1	Spring
3/18/2018	BL	6M	3	2018	Year-1	0	0	Spring
3/18/2018	BL	7M	3	2018	Year-1	0	0	Spring

Date	Species	Site	Month	Year	Survey Year	Presence	Data Gap	Season
3/18/2018	BL	10M	3	2018	Year-1		1	Spring
3/18/2018	BL	12M	3	2018	Year-1		1	Spring
3/18/2018	BL	14M	3	2018	Year-1		1	Spring
3/18/2018	BL	8A	3	2018	Year-1	0	0	Spring
3/18/2018	BL	9A	3	2018	Year-1	0	0	Spring
3/18/2018	BL	11A	3	2018	Year-1	0	0	Spring
3/18/2018	BL	13A	3	2018	Year-1	0	0	Spring
3/19/2018	BL	1M	3	2018	Year-1		1	Spring
3/19/2018	BL	2M	3	2018	Year-1		1	Spring
3/19/2018	BL	3M	3	2018	Year-1		1	Spring
3/19/2018	BL	4M	3	2018	Year-1		1	Spring
3/19/2018	BL	5M	3	2018	Year-1		1	Spring
3/19/2018	BL	6M	3	2018	Year-1		1	Spring
3/19/2018	BL	7M	3	2018	Year-1		1	Spring
3/19/2018	BL	10M	3	2018	Year-1		1	Spring
3/19/2018	BL	12M	3	2018	Year-1		1	Spring
3/19/2018	BL	14M	3	2018	Year-1		1	Spring
3/19/2018	BL	8A	3	2018	Year-1	0	0	Spring
3/19/2018	BL	9A	3	2018	Year-1	0	0	Spring
3/19/2018	BL	11A	3	2018	Year-1	0	0	Spring
3/19/2018	BL	13A	3	2018	Year-1	0	0	Spring
3/20/2018	BL	1M	3	2018	Year-1		1	Spring
3/20/2018	BL	2M	3	2018	Year-1		1	Spring
3/20/2018	BL	3M	3	2018	Year-1		1	Spring
3/20/2018	BL	4M	3	2018	Year-1		1	Spring
3/20/2018	BL	5M	3	2018	Year-1		1	Spring
3/20/2018	BL	6M	3	2018	Year-1		1	Spring
3/20/2018	BL	7M	3	2018	Year-1		1	Spring
3/20/2018	BL	10M	3	2018	Year-1		1	Spring
3/20/2018	BL	12M	3	2018	Year-1		1	Spring
3/20/2018	BL	14M	3	2018	Year-1		1	Spring
3/20/2018	BL	8A	3	2018	Year-1	0	0	Spring
3/20/2018	BL	9A	3	2018	Year-1	0	0	Spring
3/20/2018	BL	11A	3	2018	Year-1	0	0	Spring
3/20/2018	BL	13A	3	2018	Year-1	0	0	Spring
3/21/2018	BL	1M	3	2018	Year-1		1	Spring
3/21/2018	BL	2M	3	2018	Year-1		1	Spring
3/21/2018	BL	3M	3	2018	Year-1		1	Spring
3/21/2018	BL	4M	3	2018	Year-1		1	Spring
3/21/2018	BL	5M	3	2018	Year-1		1	Spring
3/21/2018	BL	6M	3	2018	Year-1		1	Spring
3/21/2018	BL	7M	3	2018	Year-1		1	Spring
3/21/2018	BL	10M	3	2018	Year-1		1	Spring
3/21/2018	BL	12M	3	2018	Year-1		1	Spring

Date	Species	Site	Month	Year	Survey Year	Presence	Data Gap	Season
3/21/2018	BL	14M	3	2018	Year-1		1	Spring
3/21/2018	BL	8A	3	2018	Year-1	0	0	Spring
3/21/2018	BL	9A	3	2018	Year-1	0	0	Spring
3/21/2018	BL	11A	3	2018	Year-1	0	0	Spring
3/21/2018	BL	13A	3	2018	Year-1	0	0	Spring
3/22/2018	BL	1M	3	2018	Year-1		1	Spring
3/22/2018	BL	2M	3	2018	Year-1		1	Spring
3/22/2018	BL	3M	3	2018	Year-1		1	Spring
3/22/2018	BL	4M	3	2018	Year-1		1	Spring
3/22/2018	BL	5M	3	2018	Year-1		1	Spring
3/22/2018	BL	6M	3	2018	Year-1		1	Spring
3/22/2018	BL	7M	3	2018	Year-1		1	Spring
3/22/2018	BL	10M	3	2018	Year-1		1	Spring
3/22/2018	BL	12M	3	2018	Year-1		1	Spring
3/22/2018	BL	14M	3	2018	Year-1		1	Spring
3/22/2018	BL	8A	3	2018	Year-1	0	0	Spring
3/22/2018	BL	9A	3	2018	Year-1	0	0	Spring
3/22/2018	BL	11A	3	2018	Year-1	0	0	Spring
3/22/2018	BL	13A	3	2018	Year-1	0	0	Spring
3/23/2018	BL	1M	3	2018	Year-1		1	Spring
3/23/2018	BL	2M	3	2018	Year-1		1	Spring
3/23/2018	BL	3M	3	2018	Year-1		1	Spring
3/23/2018	BL	4M	3	2018	Year-1		1	Spring
3/23/2018	BL	5M	3	2018	Year-1		1	Spring
3/23/2018	BL	6M	3	2018	Year-1		1	Spring
3/23/2018	BL	7M	3	2018	Year-1		1	Spring
3/23/2018	BL	10M	3	2018	Year-1		1	Spring
3/23/2018	BL	12M	3	2018	Year-1		1	Spring
3/23/2018	BL	14M	3	2018	Year-1		1	Spring
3/23/2018	BL	8A	3	2018	Year-1	0	0	Spring
3/23/2018	BL	9A	3	2018	Year-1	0	0	Spring
3/23/2018	BL	11A	3	2018	Year-1	0	0	Spring
3/23/2018	BL	13A	3	2018	Year-1	0	0	Spring
3/24/2018	BL	1M	3	2018	Year-1		1	Spring
3/24/2018	BL	2M	3	2018	Year-1		1	Spring
3/24/2018	BL	3M	3	2018	Year-1		1	Spring
3/24/2018	BL	4M	3	2018	Year-1		1	Spring
3/24/2018	BL	5M	3	2018	Year-1		1	Spring
3/24/2018	BL	6M	3	2018	Year-1		1	Spring
3/24/2018	BL	7M	3	2018	Year-1		1	Spring
3/24/2018	BL	10M	3	2018	Year-1		1	Spring
3/24/2018	BL	12M	3	2018	Year-1		1	Spring
3/24/2018	BL	14M	3	2018	Year-1		1	Spring
3/24/2018	BL	8A	3	2018	Year-1	0	0	Spring

Date	Species	Site	Month	Year	Survey Year	Presence	Data Gap	Season
3/24/2018	BL	9A	3	2018	Year-1	0	0	Spring
3/24/2018	BL	11A	3	2018	Year-1	0	0	Spring
3/24/2018	BL	13A	3	2018	Year-1	0	0	Spring
3/25/2018	BL	1M	3	2018	Year-1		1	Spring
3/25/2018	BL	2M	3	2018	Year-1		1	Spring
3/25/2018	BL	3M	3	2018	Year-1		1	Spring
3/25/2018	BL	4M	3	2018	Year-1		1	Spring
3/25/2018	BL	5M	3	2018	Year-1		1	Spring
3/25/2018	BL	6M	3	2018	Year-1		1	Spring
3/25/2018	BL	7M	3	2018	Year-1		1	Spring
3/25/2018	BL	10M	3	2018	Year-1		1	Spring
3/25/2018	BL	12M	3	2018	Year-1		1	Spring
3/25/2018	BL	14M	3	2018	Year-1		1	Spring
3/25/2018	BL	8A	3	2018	Year-1	0	0	Spring
3/25/2018	BL	9A	3	2018	Year-1	0	0	Spring
3/25/2018	BL	11A	3	2018	Year-1	0	0	Spring
3/25/2018	BL	13A	3	2018	Year-1	0	0	Spring
3/26/2018	BL	1M	3	2018	Year-1		1	Spring
3/26/2018	BL	2M	3	2018	Year-1		1	Spring
3/26/2018	BL	3M	3	2018	Year-1		1	Spring
3/26/2018	BL	4M	3	2018	Year-1		1	Spring
3/26/2018	BL	5M	3	2018	Year-1		1	Spring
3/26/2018	BL	6M	3	2018	Year-1		1	Spring
3/26/2018	BL	7M	3	2018	Year-1		1	Spring
3/26/2018	BL	10M	3	2018	Year-1		1	Spring
3/26/2018	BL	12M	3	2018	Year-1		1	Spring
3/26/2018	BL	14M	3	2018	Year-1		1	Spring
3/26/2018	BL	8A	3	2018	Year-1	0	0	Spring
3/26/2018	BL	9A	3	2018	Year-1	0	0	Spring
3/26/2018	BL	11A	3	2018	Year-1	0	0	Spring
3/26/2018	BL	13A	3	2018	Year-1	0	0	Spring
3/27/2018	BL	1M	3	2018	Year-1		1	Spring
3/27/2018	BL	2M	3	2018	Year-1		1	Spring
3/27/2018	BL	3M	3	2018	Year-1		1	Spring
3/27/2018	BL	4M	3	2018	Year-1		1	Spring
3/27/2018	BL	5M	3	2018	Year-1		1	Spring
3/27/2018	BL	6M	3	2018	Year-1		1	Spring
3/27/2018	BL	7M	3	2018	Year-1		1	Spring
3/27/2018	BL	10M	3	2018	Year-1		1	Spring
3/27/2018	BL	12M	3	2018	Year-1		1	Spring
3/27/2018	BL	14M	3	2018	Year-1		1	Spring
3/27/2018	BL	8A	3	2018	Year-1	0	0	Spring
3/27/2018	BL	9A	3	2018	Year-1	0	0	Spring
3/27/2018	BL	11A	3	2018	Year-1	0	0	Spring

Date	Species	Site	Month	Year	Survey Year	Presence	Data Gap	Season
3/27/2018	BL	13A	3	2018	Year-1	0	0	Spring
3/28/2018	BL	1M	3	2018	Year-1		1	Spring
3/28/2018	BL	2M	3	2018	Year-1		1	Spring
3/28/2018	BL	3M	3	2018	Year-1		1	Spring
3/28/2018	BL	4M	3	2018	Year-1		1	Spring
3/28/2018	BL	5M	3	2018	Year-1		1	Spring
3/28/2018	BL	6M	3	2018	Year-1		1	Spring
3/28/2018	BL	7M	3	2018	Year-1		1	Spring
3/28/2018	BL	10M	3	2018	Year-1		1	Spring
3/28/2018	BL	12M	3	2018	Year-1		1	Spring
3/28/2018	BL	14M	3	2018	Year-1		1	Spring
3/28/2018	BL	8A	3	2018	Year-1	0	0	Spring
3/28/2018	BL	9A	3	2018	Year-1	0	0	Spring
3/28/2018	BL	11A	3	2018	Year-1	0	0	Spring
3/28/2018	BL	13A	3	2018	Year-1	0	0	Spring
3/29/2018	BL	1M	3	2018	Year-1		1	Spring
3/29/2018	BL	2M	3	2018	Year-1		1	Spring
3/29/2018	BL	3M	3	2018	Year-1		1	Spring
3/29/2018	BL	4M	3	2018	Year-1		1	Spring
3/29/2018	BL	5M	3	2018	Year-1		1	Spring
3/29/2018	BL	6M	3	2018	Year-1		1	Spring
3/29/2018	BL	7M	3	2018	Year-1		1	Spring
3/29/2018	BL	10M	3	2018	Year-1		1	Spring
3/29/2018	BL	12M	3	2018	Year-1		1	Spring
3/29/2018	BL	14M	3	2018	Year-1		1	Spring
3/29/2018	BL	8A	3	2018	Year-1	0	0	Spring
3/29/2018	BL	9A	3	2018	Year-1	0	0	Spring
3/29/2018	BL	11A	3	2018	Year-1	0	0	Spring
3/29/2018	BL	13A	3	2018	Year-1	0	0	Spring
3/30/2018	BL	1M	3	2018	Year-1		1	Spring
3/30/2018	BL	2M	3	2018	Year-1		1	Spring
3/30/2018	BL	3M	3	2018	Year-1		1	Spring
3/30/2018	BL	4M	3	2018	Year-1		1	Spring
3/30/2018	BL	5M	3	2018	Year-1		1	Spring
3/30/2018	BL	6M	3	2018	Year-1		1	Spring
3/30/2018	BL	7M	3	2018	Year-1		1	Spring
3/30/2018	BL	10M	3	2018	Year-1		1	Spring
3/30/2018	BL	12M	3	2018	Year-1		1	Spring
3/30/2018	BL	14M	3	2018	Year-1		1	Spring
3/30/2018	BL	8A	3	2018	Year-1	0	0	Spring
3/30/2018	BL	9A	3	2018	Year-1	0	0	Spring
3/30/2018	BL	11A	3	2018	Year-1	0	0	Spring
3/30/2018	BL	13A	3	2018	Year-1	0	0	Spring
3/31/2018	BL	1M	3	2018	Year-1		1	Spring

Date	Species	Site	Month	Year	Survey Year	Presence	Data Gap	Season
3/31/2018	BL	2M	3	2018	Year-1		1	Spring
3/31/2018	BL	3M	3	2018	Year-1		1	Spring
3/31/2018	BL	4M	3	2018	Year-1		1	Spring
3/31/2018	BL	5M	3	2018	Year-1		1	Spring
3/31/2018	BL	6M	3	2018	Year-1		1	Spring
3/31/2018	BL	7M	3	2018	Year-1		1	Spring
3/31/2018	BL	10M	3	2018	Year-1		1	Spring
3/31/2018	BL	12M	3	2018	Year-1		1	Spring
3/31/2018	BL	14M	3	2018	Year-1		1	Spring
3/31/2018	BL	8A	3	2018	Year-1	0	0	Spring
3/31/2018	BL	9A	3	2018	Year-1	0	0	Spring
3/31/2018	BL	11A	3	2018	Year-1	0	0	Spring
3/31/2018	BL	13A	3	2018	Year-1	0	0	Spring
4/1/2018	BL	1M	4	2018	Year-1		1	Spring
4/1/2018	BL	2M	4	2018	Year-1		1	Spring
4/1/2018	BL	3M	4	2018	Year-1		1	Spring
4/1/2018	BL	4M	4	2018	Year-1		1	Spring
4/1/2018	BL	5M	4	2018	Year-1		1	Spring
4/1/2018	BL	6M	4	2018	Year-1		1	Spring
4/1/2018	BL	7M	4	2018	Year-1		1	Spring
4/1/2018	BL	10M	4	2018	Year-1		1	Spring
4/1/2018	BL	12M	4	2018	Year-1		1	Spring
4/1/2018	BL	14M	4	2018	Year-1		1	Spring
4/1/2018	BL	8A	4	2018	Year-1	0	0	Spring
4/1/2018	BL	9A	4	2018	Year-1	0	0	Spring
4/1/2018	BL	11A	4	2018	Year-1	0	0	Spring
4/1/2018	BL	13A	4	2018	Year-1	0	0	Spring
4/2/2018	BL	1M	4	2018	Year-1		1	Spring
4/2/2018	BL	2M	4	2018	Year-1		1	Spring
4/2/2018	BL	3M	4	2018	Year-1		1	Spring
4/2/2018	BL	4M	4	2018	Year-1		1	Spring
4/2/2018	BL	5M	4	2018	Year-1		1	Spring
4/2/2018	BL	6M	4	2018	Year-1		1	Spring
4/2/2018	BL	7M	4	2018	Year-1		1	Spring
4/2/2018	BL	10M	4	2018	Year-1		1	Spring
4/2/2018	BL	12M	4	2018	Year-1		1	Spring
4/2/2018	BL	14M	4	2018	Year-1		1	Spring
4/2/2018	BL	8A	4	2018	Year-1	0	0	Spring
4/2/2018	BL	9A	4	2018	Year-1	0	0	Spring
4/2/2018	BL	11A	4	2018	Year-1	0	0	Spring
4/2/2018	BL	13A	4	2018	Year-1	0	0	Spring
4/3/2018	BL	1M	4	2018	Year-1		1	Spring
4/3/2018	BL	2M	4	2018	Year-1		1	Spring
4/3/2018	BL	3M	4	2018	Year-1		1	Spring

Date	Species	Site	Month	Year	Survey Year	Presence	Data Gap	Season
4/3/2018	BL	4M	4	2018	Year-1		1	Spring
4/3/2018	BL	5M	4	2018	Year-1		1	Spring
4/3/2018	BL	6M	4	2018	Year-1		1	Spring
4/3/2018	BL	7M	4	2018	Year-1		1	Spring
4/3/2018	BL	10M	4	2018	Year-1		1	Spring
4/3/2018	BL	12M	4	2018	Year-1		1	Spring
4/3/2018	BL	14M	4	2018	Year-1		1	Spring
4/3/2018	BL	8A	4	2018	Year-1	0	0	Spring
4/3/2018	BL	9A	4	2018	Year-1	0	0	Spring
4/3/2018	BL	11A	4	2018	Year-1	0	0	Spring
4/3/2018	BL	13A	4	2018	Year-1	0	0	Spring
4/4/2018	BL	1M	4	2018	Year-1		1	Spring
4/4/2018	BL	2M	4	2018	Year-1		1	Spring
4/4/2018	BL	3M	4	2018	Year-1		1	Spring
4/4/2018	BL	4M	4	2018	Year-1		1	Spring
4/4/2018	BL	5M	4	2018	Year-1		1	Spring
4/4/2018	BL	6M	4	2018	Year-1		1	Spring
4/4/2018	BL	7M	4	2018	Year-1		1	Spring
4/4/2018	BL	10M	4	2018	Year-1		1	Spring
4/4/2018	BL	12M	4	2018	Year-1		1	Spring
4/4/2018	BL	14M	4	2018	Year-1		1	Spring
4/4/2018	BL	8A	4	2018	Year-1	0	0	Spring
4/4/2018	BL	9A	4	2018	Year-1	0	0	Spring
4/4/2018	BL	11A	4	2018	Year-1	0	0	Spring
4/4/2018	BL	13A	4	2018	Year-1	0	0	Spring
4/5/2018	BL	1M	4	2018	Year-1		1	Spring
4/5/2018	BL	2M	4	2018	Year-1		1	Spring
4/5/2018	BL	3M	4	2018	Year-1		1	Spring
4/5/2018	BL	4M	4	2018	Year-1		1	Spring
4/5/2018	BL	5M	4	2018	Year-1		1	Spring
4/5/2018	BL	6M	4	2018	Year-1		1	Spring
4/5/2018	BL	7M	4	2018	Year-1		1	Spring
4/5/2018	BL	10M	4	2018	Year-1		1	Spring
4/5/2018	BL	12M	4	2018	Year-1		1	Spring
4/5/2018	BL	14M	4	2018	Year-1		1	Spring
4/5/2018	BL	8A	4	2018	Year-1	0	0	Spring
4/5/2018	BL	9A	4	2018	Year-1	0	0	Spring
4/5/2018	BL	11A	4	2018	Year-1	0	0	Spring
4/5/2018	BL	13A	4	2018	Year-1	0	0	Spring
4/6/2018	BL	1M	4	2018	Year-1		1	Spring
4/6/2018	BL	2M	4	2018	Year-1		1	Spring
4/6/2018	BL	3M	4	2018	Year-1		1	Spring
4/6/2018	BL	4M	4	2018	Year-1		1	Spring
4/6/2018	BL	5M	4	2018	Year-1		1	Spring

Date	Species	Site	Month	Year	Survey Year	Presence	Data Gap	Season
4/6/2018	BL	6M	4	2018	Year-1		1	Spring
4/6/2018	BL	7M	4	2018	Year-1		1	Spring
4/6/2018	BL	10M	4	2018	Year-1		1	Spring
4/6/2018	BL	12M	4	2018	Year-1		1	Spring
4/6/2018	BL	14M	4	2018	Year-1		1	Spring
4/6/2018	BL	8A	4	2018	Year-1	0	0	Spring
4/6/2018	BL	9A	4	2018	Year-1	0	0	Spring
4/6/2018	BL	11A	4	2018	Year-1	0	0	Spring
4/6/2018	BL	13A	4	2018	Year-1	0	0	Spring
4/7/2018	BL	1M	4	2018	Year-1		1	Spring
4/7/2018	BL	2M	4	2018	Year-1		1	Spring
4/7/2018	BL	3M	4	2018	Year-1		1	Spring
4/7/2018	BL	4M	4	2018	Year-1		1	Spring
4/7/2018	BL	5M	4	2018	Year-1		1	Spring
4/7/2018	BL	6M	4	2018	Year-1		1	Spring
4/7/2018	BL	7M	4	2018	Year-1		1	Spring
4/7/2018	BL	10M	4	2018	Year-1		1	Spring
4/7/2018	BL	12M	4	2018	Year-1		1	Spring
4/7/2018	BL	14M	4	2018	Year-1		1	Spring
4/7/2018	BL	8A	4	2018	Year-1	0	0	Spring
4/7/2018	BL	9A	4	2018	Year-1	0	0	Spring
4/7/2018	BL	11A	4	2018	Year-1	0	0	Spring
4/7/2018	BL	13A	4	2018	Year-1	0	0	Spring
4/8/2018	BL	1M	4	2018	Year-1		1	Spring
4/8/2018	BL	2M	4	2018	Year-1		1	Spring
4/8/2018	BL	3M	4	2018	Year-1		1	Spring
4/8/2018	BL	4M	4	2018	Year-1		1	Spring
4/8/2018	BL	5M	4	2018	Year-1		1	Spring
4/8/2018	BL	6M	4	2018	Year-1		1	Spring
4/8/2018	BL	7M	4	2018	Year-1		1	Spring
4/8/2018	BL	10M	4	2018	Year-1		1	Spring
4/8/2018	BL	12M	4	2018	Year-1		1	Spring
4/8/2018	BL	14M	4	2018	Year-1		1	Spring
4/8/2018	BL	8A	4	2018	Year-1	0	0	Spring
4/8/2018	BL	9A	4	2018	Year-1	0	0	Spring
4/8/2018	BL	11A	4	2018	Year-1	0	0	Spring
4/8/2018	BL	13A	4	2018	Year-1	0	0	Spring
4/9/2018	BL	1M	4	2018	Year-1		1	Spring
4/9/2018	BL	2M	4	2018	Year-1		1	Spring
4/9/2018	BL	3M	4	2018	Year-1		1	Spring
4/9/2018	BL	4M	4	2018	Year-1		1	Spring
4/9/2018	BL	5M	4	2018	Year-1		1	Spring
4/9/2018	BL	6M	4	2018	Year-1		1	Spring
4/9/2018	BL	7M	4	2018	Year-1		1	Spring

Date	Species	Site	Month	Year	Survey Year	Presence	Data Gap	Season
4/9/2018	BL	10M	4	2018	Year-1		1	Spring
4/9/2018	BL	12M	4	2018	Year-1		1	Spring
4/9/2018	BL	14M	4	2018	Year-1		1	Spring
4/9/2018	BL	8A	4	2018	Year-1	0	0	Spring
4/9/2018	BL	9A	4	2018	Year-1	0	0	Spring
4/9/2018	BL	11A	4	2018	Year-1	0	0	Spring
4/9/2018	BL	13A	4	2018	Year-1	0	0	Spring
4/10/2018	BL	1M	4	2018	Year-1		1	Spring
4/10/2018	BL	2M	4	2018	Year-1		1	Spring
4/10/2018	BL	3M	4	2018	Year-1		1	Spring
4/10/2018	BL	4M	4	2018	Year-1		1	Spring
4/10/2018	BL	5M	4	2018	Year-1		1	Spring
4/10/2018	BL	6M	4	2018	Year-1		1	Spring
4/10/2018	BL	7M	4	2018	Year-1		1	Spring
4/10/2018	BL	10M	4	2018	Year-1		1	Spring
4/10/2018	BL	12M	4	2018	Year-1		1	Spring
4/10/2018	BL	14M	4	2018	Year-1		1	Spring
4/10/2018	BL	8A	4	2018	Year-1	0	0	Spring
4/10/2018	BL	9A	4	2018	Year-1	0	0	Spring
4/10/2018	BL	11A	4	2018	Year-1	0	0	Spring
4/10/2018	BL	13A	4	2018	Year-1	0	0	Spring
4/11/2018	BL	1M	4	2018	Year-1		1	Spring
4/11/2018	BL	2M	4	2018	Year-1		1	Spring
4/11/2018	BL	3M	4	2018	Year-1		1	Spring
4/11/2018	BL	4M	4	2018	Year-1		1	Spring
4/11/2018	BL	5M	4	2018	Year-1		1	Spring
4/11/2018	BL	6M	4	2018	Year-1		1	Spring
4/11/2018	BL	7M	4	2018	Year-1		1	Spring
4/11/2018	BL	10M	4	2018	Year-1		1	Spring
4/11/2018	BL	12M	4	2018	Year-1		1	Spring
4/11/2018	BL	14M	4	2018	Year-1		1	Spring
4/11/2018	BL	8A	4	2018	Year-1	0	0	Spring
4/11/2018	BL	9A	4	2018	Year-1	0	0	Spring
4/11/2018	BL	11A	4	2018	Year-1	0	0	Spring
4/11/2018	BL	13A	4	2018	Year-1	0	0	Spring
4/12/2018	BL	1M	4	2018	Year-1	0	0	Spring
4/12/2018	BL	2M	4	2018	Year-1		1	Spring
4/12/2018	BL	3M	4	2018	Year-1		1	Spring
4/12/2018	BL	4M	4	2018	Year-1		1	Spring
4/12/2018	BL	5M	4	2018	Year-1	0	0	Spring
4/12/2018	BL	6M	4	2018	Year-1	0	0	Spring
4/12/2018	BL	7M	4	2018	Year-1	0	0	Spring
4/12/2018	BL	10M	4	2018	Year-1	0	0	Spring
4/12/2018	BL	12M	4	2018	Year-1	0	0	Spring

Date	Species	Site	Month	Year	Survey Year	Presence	Data Gap	Season
4/12/2018	BL	14M	4	2018	Year-1	0	0	Spring
4/12/2018	BL	8A	4	2018	Year-1	0	0	Spring
4/12/2018	BL	9A	4	2018	Year-1	0	0	Spring
4/12/2018	BL	11A	4	2018	Year-1	0	0	Spring
4/12/2018	BL	13A	4	2018	Year-1	0	0	Spring
4/13/2018	BL	1M	4	2018	Year-1	0	0	Spring
4/13/2018	BL	2M	4	2018	Year-1		1	Spring
4/13/2018	BL	3M	4	2018	Year-1		1	Spring
4/13/2018	BL	4M	4	2018	Year-1		1	Spring
4/13/2018	BL	5M	4	2018	Year-1	0	0	Spring
4/13/2018	BL	6M	4	2018	Year-1	0	0	Spring
4/13/2018	BL	7M	4	2018	Year-1	0	0	Spring
4/13/2018	BL	10M	4	2018	Year-1	0	0	Spring
4/13/2018	BL	12M	4	2018	Year-1	0	0	Spring
4/13/2018	BL	14M	4	2018	Year-1	0	0	Spring
4/13/2018	BL	8A	4	2018	Year-1	0	0	Spring
4/13/2018	BL	9A	4	2018	Year-1	0	0	Spring
4/13/2018	BL	11A	4	2018	Year-1	0	0	Spring
4/13/2018	BL	13A	4	2018	Year-1	0	0	Spring
4/14/2018	BL	1M	4	2018	Year-1	0	0	Spring
4/14/2018	BL	2M	4	2018	Year-1		1	Spring
4/14/2018	BL	3M	4	2018	Year-1		1	Spring
4/14/2018	BL	4M	4	2018	Year-1		1	Spring
4/14/2018	BL	5M	4	2018	Year-1	0	0	Spring
4/14/2018	BL	6M	4	2018	Year-1	0	0	Spring
4/14/2018	BL	7M	4	2018	Year-1	0	0	Spring
4/14/2018	BL	10M	4	2018	Year-1	0	0	Spring
4/14/2018	BL	12M	4	2018	Year-1	0	0	Spring
4/14/2018	BL	14M	4	2018	Year-1	0	0	Spring
4/14/2018	BL	8A	4	2018	Year-1	0	0	Spring
4/14/2018	BL	9A	4	2018	Year-1	0	0	Spring
4/14/2018	BL	11A	4	2018	Year-1	0	0	Spring
4/14/2018	BL	13A	4	2018	Year-1	0	0	Spring
4/15/2018	BL	1M	4	2018	Year-1	0	0	Spring
4/15/2018	BL	2M	4	2018	Year-1		1	Spring
4/15/2018	BL	3M	4	2018	Year-1		1	Spring
4/15/2018	BL	4M	4	2018	Year-1		1	Spring
4/15/2018	BL	5M	4	2018	Year-1		1	Spring
4/15/2018	BL	6M	4	2018	Year-1	0	0	Spring
4/15/2018	BL	7M	4	2018	Year-1	0	0	Spring
4/15/2018	BL	10M	4	2018	Year-1	0	0	Spring
4/15/2018	BL	12M	4	2018	Year-1	0	0	Spring
4/15/2018	BL	14M	4	2018	Year-1	0	0	Spring
4/15/2018	BL	8A	4	2018	Year-1	0	0	Spring

Date	Species	Site	Month	Year	Survey Year	Presence	Data Gap	Season
4/15/2018	BL	9A	4	2018	Year-1	0	0	Spring
4/15/2018	BL	11A	4	2018	Year-1	0	0	Spring
4/15/2018	BL	13A	4	2018	Year-1	0	0	Spring
4/16/2018	BL	1M	4	2018	Year-1	0	0	Spring
4/16/2018	BL	2M	4	2018	Year-1		1	Spring
4/16/2018	BL	3M	4	2018	Year-1		1	Spring
4/16/2018	BL	4M	4	2018	Year-1		1	Spring
4/16/2018	BL	5M	4	2018	Year-1		1	Spring
4/16/2018	BL	6M	4	2018	Year-1	0	0	Spring
4/16/2018	BL	7M	4	2018	Year-1	0	0	Spring
4/16/2018	BL	10M	4	2018	Year-1	0	0	Spring
4/16/2018	BL	12M	4	2018	Year-1	0	0	Spring
4/16/2018	BL	14M	4	2018	Year-1	0	0	Spring
4/16/2018	BL	8A	4	2018	Year-1	0	0	Spring
4/16/2018	BL	9A	4	2018	Year-1	0	0	Spring
4/16/2018	BL	11A	4	2018	Year-1	0	0	Spring
4/16/2018	BL	13A	4	2018	Year-1	0	0	Spring
4/17/2018	BL	1M	4	2018	Year-1	0	0	Spring
4/17/2018	BL	2M	4	2018	Year-1		1	Spring
4/17/2018	BL	3M	4	2018	Year-1		1	Spring
4/17/2018	BL	4M	4	2018	Year-1		1	Spring
4/17/2018	BL	5M	4	2018	Year-1		1	Spring
4/17/2018	BL	6M	4	2018	Year-1	0	0	Spring
4/17/2018	BL	7M	4	2018	Year-1	0	0	Spring
4/17/2018	BL	10M	4	2018	Year-1	0	0	Spring
4/17/2018	BL	12M	4	2018	Year-1	0	0	Spring
4/17/2018	BL	14M	4	2018	Year-1	0	0	Spring
4/17/2018	BL	8A	4	2018	Year-1	0	0	Spring
4/17/2018	BL	9A	4	2018	Year-1	0	0	Spring
4/17/2018	BL	11A	4	2018	Year-1	0	0	Spring
4/17/2018	BL	13A	4	2018	Year-1	0	0	Spring
4/18/2018	BL	1M	4	2018	Year-1	0	0	Spring
4/18/2018	BL	2M	4	2018	Year-1		1	Spring
4/18/2018	BL	3M	4	2018	Year-1		1	Spring
4/18/2018	BL	4M	4	2018	Year-1		1	Spring
4/18/2018	BL	5M	4	2018	Year-1		1	Spring
4/18/2018	BL	6M	4	2018	Year-1	0	0	Spring
4/18/2018	BL	7M	4	2018	Year-1	0	0	Spring
4/18/2018	BL	10M	4	2018	Year-1	0	0	Spring
4/18/2018	BL	12M	4	2018	Year-1	0	0	Spring
4/18/2018	BL	14M	4	2018	Year-1	0	0	Spring
4/18/2018	BL	8A	4	2018	Year-1	0	0	Spring
4/18/2018	BL	9A	4	2018	Year-1	0	0	Spring
4/18/2018	BL	11A	4	2018	Year-1	0	0	Spring

Date	Species	Site	Month	Year	Survey Year	Presence	Data Gap	Season
4/18/2018	BL	13A	4	2018	Year-1	0	0	Spring
4/19/2018	BL	1M	4	2018	Year-1	0	0	Spring
4/19/2018	BL	2M	4	2018	Year-1		1	Spring
4/19/2018	BL	3M	4	2018	Year-1		1	Spring
4/19/2018	BL	4M	4	2018	Year-1		1	Spring
4/19/2018	BL	5M	4	2018	Year-1		1	Spring
4/19/2018	BL	6M	4	2018	Year-1	0	0	Spring
4/19/2018	BL	7M	4	2018	Year-1	0	0	Spring
4/19/2018	BL	10M	4	2018	Year-1	0	0	Spring
4/19/2018	BL	12M	4	2018	Year-1	0	0	Spring
4/19/2018	BL	14M	4	2018	Year-1	0	0	Spring
4/19/2018	BL	8A	4	2018	Year-1	0	0	Spring
4/19/2018	BL	9A	4	2018	Year-1	0	0	Spring
4/19/2018	BL	11A	4	2018	Year-1	0	0	Spring
4/19/2018	BL	13A	4	2018	Year-1	0	0	Spring
4/20/2018	BL	1M	4	2018	Year-1	0	0	Spring
4/20/2018	BL	2M	4	2018	Year-1		1	Spring
4/20/2018	BL	3M	4	2018	Year-1		1	Spring
4/20/2018	BL	4M	4	2018	Year-1		1	Spring
4/20/2018	BL	5M	4	2018	Year-1		1	Spring
4/20/2018	BL	6M	4	2018	Year-1	0	0	Spring
4/20/2018	BL	7M	4	2018	Year-1	0	0	Spring
4/20/2018	BL	10M	4	2018	Year-1	0	0	Spring
4/20/2018	BL	12M	4	2018	Year-1	0	0	Spring
4/20/2018	BL	14M	4	2018	Year-1	0	0	Spring
4/20/2018	BL	8A	4	2018	Year-1	0	0	Spring
4/20/2018	BL	9A	4	2018	Year-1	0	0	Spring
4/20/2018	BL	11A	4	2018	Year-1	0	0	Spring
4/20/2018	BL	13A	4	2018	Year-1	0	0	Spring
4/21/2018	BL	1M	4	2018	Year-1	0	0	Spring
4/21/2018	BL	2M	4	2018	Year-1		1	Spring
4/21/2018	BL	3M	4	2018	Year-1		1	Spring
4/21/2018	BL	4M	4	2018	Year-1		1	Spring
4/21/2018	BL	5M	4	2018	Year-1		1	Spring
4/21/2018	BL	6M	4	2018	Year-1	0	0	Spring
4/21/2018	BL	7M	4	2018	Year-1	0	0	Spring
4/21/2018	BL	10M	4	2018	Year-1	0	0	Spring
4/21/2018	BL	12M	4	2018	Year-1	0	0	Spring
4/21/2018	BL	14M	4	2018	Year-1	0	0	Spring
4/21/2018	BL	8A	4	2018	Year-1	0	0	Spring
4/21/2018	BL	9A	4	2018	Year-1	0	0	Spring
4/21/2018	BL	11A	4	2018	Year-1	0	0	Spring
4/21/2018	BL	13A	4	2018	Year-1	0	0	Spring
4/22/2018	BL	1M	4	2018	Year-1	0	0	Spring

Date	Species	Site	Month	Year	Survey Year	Presence	Data Gap	Season
4/22/2018	BL	2M	4	2018	Year-1		1	Spring
4/22/2018	BL	3M	4	2018	Year-1		1	Spring
4/22/2018	BL	4M	4	2018	Year-1		1	Spring
4/22/2018	BL	5M	4	2018	Year-1		1	Spring
4/22/2018	BL	6M	4	2018	Year-1	0	0	Spring
4/22/2018	BL	7M	4	2018	Year-1	0	0	Spring
4/22/2018	BL	10M	4	2018	Year-1	0	0	Spring
4/22/2018	BL	12M	4	2018	Year-1	0	0	Spring
4/22/2018	BL	14M	4	2018	Year-1	0	0	Spring
4/22/2018	BL	8A	4	2018	Year-1	0	0	Spring
4/22/2018	BL	9A	4	2018	Year-1	0	0	Spring
4/22/2018	BL	11A	4	2018	Year-1	0	0	Spring
4/22/2018	BL	13A	4	2018	Year-1	0	0	Spring
4/23/2018	BL	1M	4	2018	Year-1	0	0	Spring
4/23/2018	BL	2M	4	2018	Year-1		1	Spring
4/23/2018	BL	3M	4	2018	Year-1		1	Spring
4/23/2018	BL	4M	4	2018	Year-1		1	Spring
4/23/2018	BL	5M	4	2018	Year-1		1	Spring
4/23/2018	BL	6M	4	2018	Year-1	0	0	Spring
4/23/2018	BL	7M	4	2018	Year-1	0	0	Spring
4/23/2018	BL	10M	4	2018	Year-1	0	0	Spring
4/23/2018	BL	12M	4	2018	Year-1	0	0	Spring
4/23/2018	BL	14M	4	2018	Year-1	0	0	Spring
4/23/2018	BL	8A	4	2018	Year-1	0	0	Spring
4/23/2018	BL	9A	4	2018	Year-1	0	0	Spring
4/23/2018	BL	11A	4	2018	Year-1	0	0	Spring
4/23/2018	BL	13A	4	2018	Year-1	0	0	Spring
4/24/2018	BL	1M	4	2018	Year-1	0	0	Spring
4/24/2018	BL	2M	4	2018	Year-1		1	Spring
4/24/2018	BL	3M	4	2018	Year-1		1	Spring
4/24/2018	BL	4M	4	2018	Year-1		1	Spring
4/24/2018	BL	5M	4	2018	Year-1		1	Spring
4/24/2018	BL	6M	4	2018	Year-1	0	0	Spring
4/24/2018	BL	7M	4	2018	Year-1	0	0	Spring
4/24/2018	BL	10M	4	2018	Year-1	0	0	Spring
4/24/2018	BL	12M	4	2018	Year-1	0	0	Spring
4/24/2018	BL	14M	4	2018	Year-1	0	0	Spring
4/24/2018	BL	8A	4	2018	Year-1	0	0	Spring
4/24/2018	BL	9A	4	2018	Year-1	0	0	Spring
4/24/2018	BL	11A	4	2018	Year-1	0	0	Spring
4/24/2018	BL	13A	4	2018	Year-1	0	0	Spring
4/25/2018	BL	1M	4	2018	Year-1	0	0	Spring
4/25/2018	BL	2M	4	2018	Year-1		1	Spring
4/25/2018	BL	3M	4	2018	Year-1		1	Spring

Date	Species	Site	Month	Year	Survey Year	Presence	Data Gap	Season
4/25/2018	BL	4M	4	2018	Year-1		1	Spring
4/25/2018	BL	5M	4	2018	Year-1		1	Spring
4/25/2018	BL	6M	4	2018	Year-1	0	0	Spring
4/25/2018	BL	7M	4	2018	Year-1	0	0	Spring
4/25/2018	BL	10M	4	2018	Year-1	0	0	Spring
4/25/2018	BL	12M	4	2018	Year-1	0	0	Spring
4/25/2018	BL	14M	4	2018	Year-1	0	0	Spring
4/25/2018	BL	8A	4	2018	Year-1	0	0	Spring
4/25/2018	BL	9A	4	2018	Year-1	0	0	Spring
4/25/2018	BL	11A	4	2018	Year-1	0	0	Spring
4/25/2018	BL	13A	4	2018	Year-1	0	0	Spring
4/26/2018	BL	1M	4	2018	Year-1	0	0	Spring
4/26/2018	BL	2M	4	2018	Year-1		1	Spring
4/26/2018	BL	3M	4	2018	Year-1		1	Spring
4/26/2018	BL	4M	4	2018	Year-1		1	Spring
4/26/2018	BL	5M	4	2018	Year-1		1	Spring
4/26/2018	BL	6M	4	2018	Year-1	0	0	Spring
4/26/2018	BL	7M	4	2018	Year-1	0	0	Spring
4/26/2018	BL	10M	4	2018	Year-1	0	0	Spring
4/26/2018	BL	12M	4	2018	Year-1	0	0	Spring
4/26/2018	BL	14M	4	2018	Year-1	0	0	Spring
4/26/2018	BL	8A	4	2018	Year-1	0	0	Spring
4/26/2018	BL	9A	4	2018	Year-1	0	0	Spring
4/26/2018	BL	11A	4	2018	Year-1	0	0	Spring
4/26/2018	BL	13A	4	2018	Year-1	0	0	Spring
4/27/2018	BL	1M	4	2018	Year-1	0	0	Spring
4/27/2018	BL	2M	4	2018	Year-1		1	Spring
4/27/2018	BL	3M	4	2018	Year-1		1	Spring
4/27/2018	BL	4M	4	2018	Year-1		1	Spring
4/27/2018	BL	5M	4	2018	Year-1		1	Spring
4/27/2018	BL	6M	4	2018	Year-1	0	0	Spring
4/27/2018	BL	7M	4	2018	Year-1	0	0	Spring
4/27/2018	BL	10M	4	2018	Year-1	0	0	Spring
4/27/2018	BL	12M	4	2018	Year-1	0	0	Spring
4/27/2018	BL	14M	4	2018	Year-1	0	0	Spring
4/27/2018	BL	8A	4	2018	Year-1	0	0	Spring
4/27/2018	BL	9A	4	2018	Year-1	0	0	Spring
4/27/2018	BL	11A	4	2018	Year-1	0	0	Spring
4/27/2018	BL	13A	4	2018	Year-1	0	0	Spring
4/28/2018	BL	1M	4	2018	Year-1	0	0	Spring
4/28/2018	BL	2M	4	2018	Year-1		1	Spring
4/28/2018	BL	3M	4	2018	Year-1		1	Spring
4/28/2018	BL	4M	4	2018	Year-1		1	Spring
4/28/2018	BL	5M	4	2018	Year-1		1	Spring

Date	Species	Site	Month	Year	Survey Year	Presence	Data Gap	Season
4/28/2018	BL	6M	4	2018	Year-1	0	0	Spring
4/28/2018	BL	7M	4	2018	Year-1	0	0	Spring
4/28/2018	BL	10M	4	2018	Year-1	0	0	Spring
4/28/2018	BL	12M	4	2018	Year-1	0	0	Spring
4/28/2018	BL	14M	4	2018	Year-1	0	0	Spring
4/28/2018	BL	8A	4	2018	Year-1	0	0	Spring
4/28/2018	BL	9A	4	2018	Year-1	0	0	Spring
4/28/2018	BL	11A	4	2018	Year-1	0	0	Spring
4/28/2018	BL	13A	4	2018	Year-1	0	0	Spring
4/29/2018	BL	1M	4	2018	Year-1	0	0	Spring
4/29/2018	BL	2M	4	2018	Year-1		1	Spring
4/29/2018	BL	3M	4	2018	Year-1		1	Spring
4/29/2018	BL	4M	4	2018	Year-1		1	Spring
4/29/2018	BL	5M	4	2018	Year-1		1	Spring
4/29/2018	BL	6M	4	2018	Year-1	0	0	Spring
4/29/2018	BL	7M	4	2018	Year-1	0	0	Spring
4/29/2018	BL	10M	4	2018	Year-1	0	0	Spring
4/29/2018	BL	12M	4	2018	Year-1	0	0	Spring
4/29/2018	BL	14M	4	2018	Year-1	0	0	Spring
4/29/2018	BL	8A	4	2018	Year-1	0	0	Spring
4/29/2018	BL	9A	4	2018	Year-1	0	0	Spring
4/29/2018	BL	11A	4	2018	Year-1	0	0	Spring
4/29/2018	BL	13A	4	2018	Year-1	0	0	Spring
4/30/2018	BL	1M	4	2018	Year-1	0	0	Spring
4/30/2018	BL	2M	4	2018	Year-1		1	Spring
4/30/2018	BL	3M	4	2018	Year-1		1	Spring
4/30/2018	BL	4M	4	2018	Year-1		1	Spring
4/30/2018	BL	5M	4	2018	Year-1		1	Spring
4/30/2018	BL	6M	4	2018	Year-1	0	0	Spring
4/30/2018	BL	7M	4	2018	Year-1	0	0	Spring
4/30/2018	BL	10M	4	2018	Year-1	0	0	Spring
4/30/2018	BL	12M	4	2018	Year-1	0	0	Spring
4/30/2018	BL	14M	4	2018	Year-1	0	0	Spring
4/30/2018	BL	8A	4	2018	Year-1	0	0	Spring
4/30/2018	BL	9A	4	2018	Year-1	0	0	Spring
4/30/2018	BL	11A	4	2018	Year-1	0	0	Spring
4/30/2018	BL	13A	4	2018	Year-1	0	0	Spring
5/1/2018	BL	1M	5	2018	Year-1	0	0	Spring
5/1/2018	BL	2M	5	2018	Year-1		1	Spring
5/1/2018	BL	3M	5	2018	Year-1		1	Spring
5/1/2018	BL	4M	5	2018	Year-1		1	Spring
5/1/2018	BL	5M	5	2018	Year-1		1	Spring
5/1/2018	BL	6M	5	2018	Year-1	0	0	Spring
5/1/2018	BL	7M	5	2018	Year-1	0	0	Spring

Date	Species	Site	Month	Year	Survey Year	Presence	Data Gap	Season
5/1/2018	BL	10M	5	2018	Year-1	0	0	Spring
5/1/2018	BL	12M	5	2018	Year-1	0	0	Spring
5/1/2018	BL	14M	5	2018	Year-1	0	0	Spring
5/1/2018	BL	8A	5	2018	Year-1	0	0	Spring
5/1/2018	BL	9A	5	2018	Year-1	0	0	Spring
5/1/2018	BL	11A	5	2018	Year-1	0	0	Spring
5/1/2018	BL	13A	5	2018	Year-1	0	0	Spring
5/2/2018	BL	1M	5	2018	Year-1	0	0	Spring
5/2/2018	BL	2M	5	2018	Year-1		1	Spring
5/2/2018	BL	3M	5	2018	Year-1		1	Spring
5/2/2018	BL	4M	5	2018	Year-1		1	Spring
5/2/2018	BL	5M	5	2018	Year-1		1	Spring
5/2/2018	BL	6M	5	2018	Year-1	0	0	Spring
5/2/2018	BL	7M	5	2018	Year-1	0	0	Spring
5/2/2018	BL	10M	5	2018	Year-1	0	0	Spring
5/2/2018	BL	12M	5	2018	Year-1	0	0	Spring
5/2/2018	BL	14M	5	2018	Year-1	0	0	Spring
5/2/2018	BL	8A	5	2018	Year-1	0	0	Spring
5/2/2018	BL	9A	5	2018	Year-1	0	0	Spring
5/2/2018	BL	11A	5	2018	Year-1	0	0	Spring
5/2/2018	BL	13A	5	2018	Year-1	0	0	Spring
5/3/2018	BL	1M	5	2018	Year-1	0	0	Spring
5/3/2018	BL	2M	5	2018	Year-1		1	Spring
5/3/2018	BL	3M	5	2018	Year-1		1	Spring
5/3/2018	BL	4M	5	2018	Year-1		1	Spring
5/3/2018	BL	5M	5	2018	Year-1		1	Spring
5/3/2018	BL	6M	5	2018	Year-1	0	0	Spring
5/3/2018	BL	7M	5	2018	Year-1	0	0	Spring
5/3/2018	BL	10M	5	2018	Year-1	0	0	Spring
5/3/2018	BL	12M	5	2018	Year-1	0	0	Spring
5/3/2018	BL	14M	5	2018	Year-1	0	0	Spring
5/3/2018	BL	8A	5	2018	Year-1	0	0	Spring
5/3/2018	BL	9A	5	2018	Year-1	0	0	Spring
5/3/2018	BL	11A	5	2018	Year-1	0	0	Spring
5/3/2018	BL	13A	5	2018	Year-1	0	0	Spring
5/4/2018	BL	1M	5	2018	Year-1	0	0	Spring
5/4/2018	BL	2M	5	2018	Year-1		1	Spring
5/4/2018	BL	3M	5	2018	Year-1		1	Spring
5/4/2018	BL	4M	5	2018	Year-1		1	Spring
5/4/2018	BL	5M	5	2018	Year-1		1	Spring
5/4/2018	BL	6M	5	2018	Year-1	0	0	Spring
5/4/2018	BL	7M	5	2018	Year-1	0	0	Spring
5/4/2018	BL	10M	5	2018	Year-1	0	0	Spring
5/4/2018	BL	12M	5	2018	Year-1	0	0	Spring

Date	Species	Site	Month	Year	Survey Year	Presence	Data Gap	Season
5/4/2018	BL	14M	5	2018	Year-1	0	0	Spring
5/4/2018	BL	8A	5	2018	Year-1	0	0	Spring
5/4/2018	BL	9A	5	2018	Year-1	0	0	Spring
5/4/2018	BL	11A	5	2018	Year-1	0	0	Spring
5/4/2018	BL	13A	5	2018	Year-1	0	0	Spring
5/5/2018	BL	1M	5	2018	Year-1	0	0	Spring
5/5/2018	BL	2M	5	2018	Year-1		1	Spring
5/5/2018	BL	3M	5	2018	Year-1		1	Spring
5/5/2018	BL	4M	5	2018	Year-1		1	Spring
5/5/2018	BL	5M	5	2018	Year-1		1	Spring
5/5/2018	BL	6M	5	2018	Year-1	0	0	Spring
5/5/2018	BL	7M	5	2018	Year-1	0	0	Spring
5/5/2018	BL	10M	5	2018	Year-1	0	0	Spring
5/5/2018	BL	12M	5	2018	Year-1	0	0	Spring
5/5/2018	BL	14M	5	2018	Year-1	0	0	Spring
5/5/2018	BL	8A	5	2018	Year-1	0	0	Spring
5/5/2018	BL	9A	5	2018	Year-1	0	0	Spring
5/5/2018	BL	11A	5	2018	Year-1	0	0	Spring
5/5/2018	BL	13A	5	2018	Year-1	0	0	Spring
5/6/2018	BL	1M	5	2018	Year-1	0	0	Spring
5/6/2018	BL	2M	5	2018	Year-1		1	Spring
5/6/2018	BL	3M	5	2018	Year-1		1	Spring
5/6/2018	BL	4M	5	2018	Year-1		1	Spring
5/6/2018	BL	5M	5	2018	Year-1		1	Spring
5/6/2018	BL	6M	5	2018	Year-1	0	0	Spring
5/6/2018	BL	7M	5	2018	Year-1	0	0	Spring
5/6/2018	BL	10M	5	2018	Year-1	0	0	Spring
5/6/2018	BL	12M	5	2018	Year-1	0	0	Spring
5/6/2018	BL	14M	5	2018	Year-1	0	0	Spring
5/6/2018	BL	8A	5	2018	Year-1	0	0	Spring
5/6/2018	BL	9A	5	2018	Year-1	0	0	Spring
5/6/2018	BL	11A	5	2018	Year-1	0	0	Spring
5/6/2018	BL	13A	5	2018	Year-1	0	0	Spring
5/7/2018	BL	1M	5	2018	Year-1	0	0	Spring
5/7/2018	BL	2M	5	2018	Year-1		1	Spring
5/7/2018	BL	3M	5	2018	Year-1		1	Spring
5/7/2018	BL	4M	5	2018	Year-1		1	Spring
5/7/2018	BL	5M	5	2018	Year-1		1	Spring
5/7/2018	BL	6M	5	2018	Year-1	0	0	Spring
5/7/2018	BL	7M	5	2018	Year-1	0	0	Spring
5/7/2018	BL	10M	5	2018	Year-1	0	0	Spring
5/7/2018	BL	12M	5	2018	Year-1	0	0	Spring
5/7/2018	BL	14M	5	2018	Year-1	0	0	Spring
5/7/2018	BL	8A	5	2018	Year-1	0	0	Spring

Date	Species	Site	Month	Year	Survey Year	Presence	Data Gap	Season
5/7/2018	BL	9A	5	2018	Year-1	0	0	Spring
5/7/2018	BL	11A	5	2018	Year-1	0	0	Spring
5/7/2018	BL	13A	5	2018	Year-1	0	0	Spring
5/8/2018	BL	1M	5	2018	Year-1	0	0	Spring
5/8/2018	BL	2M	5	2018	Year-1		1	Spring
5/8/2018	BL	3M	5	2018	Year-1		1	Spring
5/8/2018	BL	4M	5	2018	Year-1		1	Spring
5/8/2018	BL	5M	5	2018	Year-1		1	Spring
5/8/2018	BL	6M	5	2018	Year-1	0	0	Spring
5/8/2018	BL	7M	5	2018	Year-1	0	0	Spring
5/8/2018	BL	10M	5	2018	Year-1	0	0	Spring
5/8/2018	BL	12M	5	2018	Year-1	0	0	Spring
5/8/2018	BL	14M	5	2018	Year-1	0	0	Spring
5/8/2018	BL	8A	5	2018	Year-1	0	0	Spring
5/8/2018	BL	9A	5	2018	Year-1	0	0	Spring
5/8/2018	BL	11A	5	2018	Year-1	0	0	Spring
5/8/2018	BL	13A	5	2018	Year-1	0	0	Spring
5/9/2018	BL	1M	5	2018	Year-1	0	0	Spring
5/9/2018	BL	2M	5	2018	Year-1		1	Spring
5/9/2018	BL	3M	5	2018	Year-1		1	Spring
5/9/2018	BL	4M	5	2018	Year-1		1	Spring
5/9/2018	BL	5M	5	2018	Year-1		1	Spring
5/9/2018	BL	6M	5	2018	Year-1	0	0	Spring
5/9/2018	BL	7M	5	2018	Year-1	0	0	Spring
5/9/2018	BL	10M	5	2018	Year-1	0	0	Spring
5/9/2018	BL	12M	5	2018	Year-1	0	0	Spring
5/9/2018	BL	14M	5	2018	Year-1	0	0	Spring
5/9/2018	BL	8A	5	2018	Year-1	0	0	Spring
5/9/2018	BL	9A	5	2018	Year-1	0	0	Spring
5/9/2018	BL	11A	5	2018	Year-1	0	0	Spring
5/9/2018	BL	13A	5	2018	Year-1	0	0	Spring
5/10/2018	BL	1M	5	2018	Year-1	0	0	Spring
5/10/2018	BL	2M	5	2018	Year-1		1	Spring
5/10/2018	BL	3M	5	2018	Year-1		1	Spring
5/10/2018	BL	4M	5	2018	Year-1		1	Spring
5/10/2018	BL	5M	5	2018	Year-1		1	Spring
5/10/2018	BL	6M	5	2018	Year-1	0	0	Spring
5/10/2018	BL	7M	5	2018	Year-1	0	0	Spring
5/10/2018	BL	10M	5	2018	Year-1	0	0	Spring
5/10/2018	BL	12M	5	2018	Year-1	0	0	Spring
5/10/2018	BL	14M	5	2018	Year-1	0	0	Spring
5/10/2018	BL	8A	5	2018	Year-1	0	0	Spring
5/10/2018	BL	9A	5	2018	Year-1	0	0	Spring
5/10/2018	BL	11A	5	2018	Year-1	0	0	Spring

Date	Species	Site	Month	Year	Survey Year	Presence	Data Gap	Season
5/10/2018	BL	13A	5	2018	Year-1	0	0	Spring
5/11/2018	BL	1M	5	2018	Year-1	0	0	Spring
5/11/2018	BL	2M	5	2018	Year-1		1	Spring
5/11/2018	BL	3M	5	2018	Year-1		1	Spring
5/11/2018	BL	4M	5	2018	Year-1		1	Spring
5/11/2018	BL	5M	5	2018	Year-1		1	Spring
5/11/2018	BL	6M	5	2018	Year-1	0	0	Spring
5/11/2018	BL	7M	5	2018	Year-1	0	0	Spring
5/11/2018	BL	10M	5	2018	Year-1	0	0	Spring
5/11/2018	BL	12M	5	2018	Year-1	0	0	Spring
5/11/2018	BL	14M	5	2018	Year-1	0	0	Spring
5/11/2018	BL	8A	5	2018	Year-1	0	0	Spring
5/11/2018	BL	9A	5	2018	Year-1	0	0	Spring
5/11/2018	BL	11A	5	2018	Year-1	0	0	Spring
5/11/2018	BL	13A	5	2018	Year-1	0	0	Spring
5/12/2018	BL	1M	5	2018	Year-1	0	0	Spring
5/12/2018	BL	2M	5	2018	Year-1		1	Spring
5/12/2018	BL	3M	5	2018	Year-1		1	Spring
5/12/2018	BL	4M	5	2018	Year-1		1	Spring
5/12/2018	BL	5M	5	2018	Year-1		1	Spring
5/12/2018	BL	6M	5	2018	Year-1	0	0	Spring
5/12/2018	BL	7M	5	2018	Year-1	0	0	Spring
5/12/2018	BL	10M	5	2018	Year-1	0	0	Spring
5/12/2018	BL	12M	5	2018	Year-1	0	0	Spring
5/12/2018	BL	14M	5	2018	Year-1	0	0	Spring
5/12/2018	BL	8A	5	2018	Year-1	0	0	Spring
5/12/2018	BL	9A	5	2018	Year-1	0	0	Spring
5/12/2018	BL	11A	5	2018	Year-1	0	0	Spring
5/12/2018	BL	13A	5	2018	Year-1	0	0	Spring
5/13/2018	BL	1M	5	2018	Year-1	0	0	Spring
5/13/2018	BL	2M	5	2018	Year-1		1	Spring
5/13/2018	BL	3M	5	2018	Year-1		1	Spring
5/13/2018	BL	4M	5	2018	Year-1		1	Spring
5/13/2018	BL	5M	5	2018	Year-1		1	Spring
5/13/2018	BL	6M	5	2018	Year-1	0	0	Spring
5/13/2018	BL	7M	5	2018	Year-1	0	0	Spring
5/13/2018	BL	10M	5	2018	Year-1	0	0	Spring
5/13/2018	BL	12M	5	2018	Year-1	0	0	Spring
5/13/2018	BL	14M	5	2018	Year-1	0	0	Spring
5/13/2018	BL	8A	5	2018	Year-1	0	0	Spring
5/13/2018	BL	9A	5	2018	Year-1	0	0	Spring
5/13/2018	BL	11A	5	2018	Year-1	0	0	Spring
5/13/2018	BL	13A	5	2018	Year-1	0	0	Spring
5/14/2018	BL	1M	5	2018	Year-1	0	0	Spring

Date	Species	Site	Month	Year	Survey Year	Presence	Data Gap	Season
5/14/2018	BL	2M	5	2018	Year-1		1	Spring
5/14/2018	BL	3M	5	2018	Year-1		1	Spring
5/14/2018	BL	4M	5	2018	Year-1		1	Spring
5/14/2018	BL	5M	5	2018	Year-1		1	Spring
5/14/2018	BL	6M	5	2018	Year-1	0	0	Spring
5/14/2018	BL	7M	5	2018	Year-1	0	0	Spring
5/14/2018	BL	10M	5	2018	Year-1	0	0	Spring
5/14/2018	BL	12M	5	2018	Year-1	0	0	Spring
5/14/2018	BL	14M	5	2018	Year-1	0	0	Spring
5/14/2018	BL	8A	5	2018	Year-1	0	0	Spring
5/14/2018	BL	9A	5	2018	Year-1	0	0	Spring
5/14/2018	BL	11A	5	2018	Year-1	0	0	Spring
5/14/2018	BL	13A	5	2018	Year-1	0	0	Spring
5/15/2018	BL	1M	5	2018	Year-1	0	0	Spring
5/15/2018	BL	2M	5	2018	Year-1		1	Spring
5/15/2018	BL	3M	5	2018	Year-1		1	Spring
5/15/2018	BL	4M	5	2018	Year-1		1	Spring
5/15/2018	BL	5M	5	2018	Year-1		1	Spring
5/15/2018	BL	6M	5	2018	Year-1	0	0	Spring
5/15/2018	BL	7M	5	2018	Year-1	0	0	Spring
5/15/2018	BL	10M	5	2018	Year-1	0	0	Spring
5/15/2018	BL	12M	5	2018	Year-1	0	0	Spring
5/15/2018	BL	14M	5	2018	Year-1	0	0	Spring
5/15/2018	BL	8A	5	2018	Year-1	0	0	Spring
5/15/2018	BL	9A	5	2018	Year-1	0	0	Spring
5/15/2018	BL	11A	5	2018	Year-1	0	0	Spring
5/15/2018	BL	13A	5	2018	Year-1	0	0	Spring
5/16/2018	BL	1M	5	2018	Year-1	0	0	Spring
5/16/2018	BL	2M	5	2018	Year-1		1	Spring
5/16/2018	BL	3M	5	2018	Year-1		1	Spring
5/16/2018	BL	4M	5	2018	Year-1		1	Spring
5/16/2018	BL	5M	5	2018	Year-1		1	Spring
5/16/2018	BL	6M	5	2018	Year-1	0	0	Spring
5/16/2018	BL	7M	5	2018	Year-1	0	0	Spring
5/16/2018	BL	10M	5	2018	Year-1	0	0	Spring
5/16/2018	BL	12M	5	2018	Year-1	0	0	Spring
5/16/2018	BL	14M	5	2018	Year-1	0	0	Spring
5/16/2018	BL	8A	5	2018	Year-1	0	0	Spring
5/16/2018	BL	9A	5	2018	Year-1	0	0	Spring
5/16/2018	BL	11A	5	2018	Year-1	0	0	Spring
5/16/2018	BL	13A	5	2018	Year-1	0	0	Spring
5/17/2018	BL	1M	5	2018	Year-1	0	0	Spring
5/17/2018	BL	2M	5	2018	Year-1		1	Spring
5/17/2018	BL	3M	5	2018	Year-1		1	Spring

Date	Species	Site	Month	Year	Survey Year	Presence	Data Gap	Season
5/17/2018	BL	4M	5	2018	Year-1		1	Spring
5/17/2018	BL	5M	5	2018	Year-1		1	Spring
5/17/2018	BL	6M	5	2018	Year-1	0	0	Spring
5/17/2018	BL	7M	5	2018	Year-1	0	0	Spring
5/17/2018	BL	10M	5	2018	Year-1	0	0	Spring
5/17/2018	BL	12M	5	2018	Year-1	0	0	Spring
5/17/2018	BL	14M	5	2018	Year-1	0	0	Spring
5/17/2018	BL	8A	5	2018	Year-1	0	0	Spring
5/17/2018	BL	9A	5	2018	Year-1	0	0	Spring
5/17/2018	BL	11A	5	2018	Year-1	0	0	Spring
5/17/2018	BL	13A	5	2018	Year-1	0	0	Spring
5/18/2018	BL	1M	5	2018	Year-1	0	0	Spring
5/18/2018	BL	2M	5	2018	Year-1		1	Spring
5/18/2018	BL	3M	5	2018	Year-1		1	Spring
5/18/2018	BL	4M	5	2018	Year-1		1	Spring
5/18/2018	BL	5M	5	2018	Year-1		1	Spring
5/18/2018	BL	6M	5	2018	Year-1	0	0	Spring
5/18/2018	BL	7M	5	2018	Year-1	0	0	Spring
5/18/2018	BL	10M	5	2018	Year-1	0	0	Spring
5/18/2018	BL	12M	5	2018	Year-1	0	0	Spring
5/18/2018	BL	14M	5	2018	Year-1	0	0	Spring
5/18/2018	BL	8A	5	2018	Year-1	0	0	Spring
5/18/2018	BL	9A	5	2018	Year-1	0	0	Spring
5/18/2018	BL	11A	5	2018	Year-1	0	0	Spring
5/18/2018	BL	13A	5	2018	Year-1	0	0	Spring
5/19/2018	BL	1M	5	2018	Year-1	0	0	Spring
5/19/2018	BL	2M	5	2018	Year-1		1	Spring
5/19/2018	BL	3M	5	2018	Year-1		1	Spring
5/19/2018	BL	4M	5	2018	Year-1		1	Spring
5/19/2018	BL	5M	5	2018	Year-1		1	Spring
5/19/2018	BL	6M	5	2018	Year-1	0	0	Spring
5/19/2018	BL	7M	5	2018	Year-1	0	0	Spring
5/19/2018	BL	10M	5	2018	Year-1	0	0	Spring
5/19/2018	BL	12M	5	2018	Year-1	0	0	Spring
5/19/2018	BL	14M	5	2018	Year-1	0	0	Spring
5/19/2018	BL	8A	5	2018	Year-1	0	0	Spring
5/19/2018	BL	9A	5	2018	Year-1	0	0	Spring
5/19/2018	BL	11A	5	2018	Year-1	0	0	Spring
5/19/2018	BL	13A	5	2018	Year-1	0	0	Spring
5/20/2018	BL	1M	5	2018	Year-1	0	0	Spring
5/20/2018	BL	2M	5	2018	Year-1		1	Spring
5/20/2018	BL	3M	5	2018	Year-1		1	Spring
5/20/2018	BL	4M	5	2018	Year-1		1	Spring
5/20/2018	BL	5M	5	2018	Year-1		1	Spring

Date	Species	Site	Month	Year	Survey Year	Presence	Data Gap	Season
5/20/2018	BL	6M	5	2018	Year-1	0	0	Spring
5/20/2018	BL	7M	5	2018	Year-1	0	0	Spring
5/20/2018	BL	10M	5	2018	Year-1	0	0	Spring
5/20/2018	BL	12M	5	2018	Year-1	0	0	Spring
5/20/2018	BL	14M	5	2018	Year-1	0	0	Spring
5/20/2018	BL	8A	5	2018	Year-1	0	0	Spring
5/20/2018	BL	9A	5	2018	Year-1	0	0	Spring
5/20/2018	BL	11A	5	2018	Year-1	0	0	Spring
5/20/2018	BL	13A	5	2018	Year-1	0	0	Spring
5/21/2018	BL	1M	5	2018	Year-1	0	0	Spring
5/21/2018	BL	2M	5	2018	Year-1		1	Spring
5/21/2018	BL	3M	5	2018	Year-1		1	Spring
5/21/2018	BL	4M	5	2018	Year-1		1	Spring
5/21/2018	BL	5M	5	2018	Year-1		1	Spring
5/21/2018	BL	6M	5	2018	Year-1	0	0	Spring
5/21/2018	BL	7M	5	2018	Year-1	0	0	Spring
5/21/2018	BL	10M	5	2018	Year-1	0	0	Spring
5/21/2018	BL	12M	5	2018	Year-1	0	0	Spring
5/21/2018	BL	14M	5	2018	Year-1	0	0	Spring
5/21/2018	BL	8A	5	2018	Year-1	0	0	Spring
5/21/2018	BL	9A	5	2018	Year-1	0	0	Spring
5/21/2018	BL	11A	5	2018	Year-1	0	0	Spring
5/21/2018	BL	13A	5	2018	Year-1	0	0	Spring
5/22/2018	BL	1M	5	2018	Year-1	0	0	Spring
5/22/2018	BL	2M	5	2018	Year-1		1	Spring
5/22/2018	BL	3M	5	2018	Year-1		1	Spring
5/22/2018	BL	4M	5	2018	Year-1		1	Spring
5/22/2018	BL	5M	5	2018	Year-1		1	Spring
5/22/2018	BL	6M	5	2018	Year-1	0	0	Spring
5/22/2018	BL	7M	5	2018	Year-1	0	0	Spring
5/22/2018	BL	10M	5	2018	Year-1	0	0	Spring
5/22/2018	BL	12M	5	2018	Year-1	0	0	Spring
5/22/2018	BL	14M	5	2018	Year-1	0	0	Spring
5/22/2018	BL	8A	5	2018	Year-1	0	0	Spring
5/22/2018	BL	9A	5	2018	Year-1	0	0	Spring
5/22/2018	BL	11A	5	2018	Year-1	0	0	Spring
5/22/2018	BL	13A	5	2018	Year-1	0	0	Spring
5/23/2018	BL	1M	5	2018	Year-1	0	0	Spring
5/23/2018	BL	2M	5	2018	Year-1		1	Spring
5/23/2018	BL	3M	5	2018	Year-1		1	Spring
5/23/2018	BL	4M	5	2018	Year-1		1	Spring
5/23/2018	BL	5M	5	2018	Year-1		1	Spring
5/23/2018	BL	6M	5	2018	Year-1	0	0	Spring
5/23/2018	BL	7M	5	2018	Year-1	0	0	Spring

Date	Species	Site	Month	Year	Survey Year	Presence	Data Gap	Season
5/23/2018	BL	10M	5	2018	Year-1	0	0	Spring
5/23/2018	BL	12M	5	2018	Year-1	0	0	Spring
5/23/2018	BL	14M	5	2018	Year-1	0	0	Spring
5/23/2018	BL	8A	5	2018	Year-1	0	0	Spring
5/23/2018	BL	9A	5	2018	Year-1	0	0	Spring
5/23/2018	BL	11A	5	2018	Year-1	0	0	Spring
5/23/2018	BL	13A	5	2018	Year-1	0	0	Spring
5/24/2018	BL	1M	5	2018	Year-1	0	0	Spring
5/24/2018	BL	2M	5	2018	Year-1		1	Spring
5/24/2018	BL	3M	5	2018	Year-1		1	Spring
5/24/2018	BL	4M	5	2018	Year-1		1	Spring
5/24/2018	BL	5M	5	2018	Year-1		1	Spring
5/24/2018	BL	6M	5	2018	Year-1	0	0	Spring
5/24/2018	BL	7M	5	2018	Year-1	0	0	Spring
5/24/2018	BL	10M	5	2018	Year-1	0	0	Spring
5/24/2018	BL	12M	5	2018	Year-1	0	0	Spring
5/24/2018	BL	14M	5	2018	Year-1	0	0	Spring
5/24/2018	BL	8A	5	2018	Year-1	0	0	Spring
5/24/2018	BL	9A	5	2018	Year-1	0	0	Spring
5/24/2018	BL	11A	5	2018	Year-1	0	0	Spring
5/24/2018	BL	13A	5	2018	Year-1	0	0	Spring
5/25/2018	BL	1M	5	2018	Year-1	0	0	Spring
5/25/2018	BL	2M	5	2018	Year-1		1	Spring
5/25/2018	BL	3M	5	2018	Year-1		1	Spring
5/25/2018	BL	4M	5	2018	Year-1		1	Spring
5/25/2018	BL	5M	5	2018	Year-1		1	Spring
5/25/2018	BL	6M	5	2018	Year-1	0	0	Spring
5/25/2018	BL	7M	5	2018	Year-1	0	0	Spring
5/25/2018	BL	10M	5	2018	Year-1	0	0	Spring
5/25/2018	BL	12M	5	2018	Year-1	0	0	Spring
5/25/2018	BL	14M	5	2018	Year-1	0	0	Spring
5/25/2018	BL	8A	5	2018	Year-1	0	0	Spring
5/25/2018	BL	9A	5	2018	Year-1	0	0	Spring
5/25/2018	BL	11A	5	2018	Year-1	0	0	Spring
5/25/2018	BL	13A	5	2018	Year-1	0	0	Spring
5/26/2018	BL	1M	5	2018	Year-1	0	0	Spring
5/26/2018	BL	2M	5	2018	Year-1		1	Spring
5/26/2018	BL	3M	5	2018	Year-1		1	Spring
5/26/2018	BL	4M	5	2018	Year-1		1	Spring
5/26/2018	BL	5M	5	2018	Year-1		1	Spring
5/26/2018	BL	6M	5	2018	Year-1	0	0	Spring
5/26/2018	BL	7M	5	2018	Year-1	0	0	Spring
5/26/2018	BL	10M	5	2018	Year-1	0	0	Spring
5/26/2018	BL	12M	5	2018	Year-1	0	0	Spring

Date	Species	Site	Month	Year	Survey Year	Presence	Data Gap	Season
5/26/2018	BL	14M	5	2018	Year-1	0	0	Spring
5/26/2018	BL	8A	5	2018	Year-1	0	0	Spring
5/26/2018	BL	9A	5	2018	Year-1	0	0	Spring
5/26/2018	BL	11A	5	2018	Year-1	0	0	Spring
5/26/2018	BL	13A	5	2018	Year-1	0	0	Spring
5/27/2018	BL	1M	5	2018	Year-1	0	0	Spring
5/27/2018	BL	2M	5	2018	Year-1		1	Spring
5/27/2018	BL	3M	5	2018	Year-1		1	Spring
5/27/2018	BL	4M	5	2018	Year-1		1	Spring
5/27/2018	BL	5M	5	2018	Year-1		1	Spring
5/27/2018	BL	6M	5	2018	Year-1	0	0	Spring
5/27/2018	BL	7M	5	2018	Year-1	0	0	Spring
5/27/2018	BL	10M	5	2018	Year-1	0	0	Spring
5/27/2018	BL	12M	5	2018	Year-1	0	0	Spring
5/27/2018	BL	14M	5	2018	Year-1	0	0	Spring
5/27/2018	BL	8A	5	2018	Year-1	0	0	Spring
5/27/2018	BL	9A	5	2018	Year-1	0	0	Spring
5/27/2018	BL	11A	5	2018	Year-1	0	0	Spring
5/27/2018	BL	13A	5	2018	Year-1	0	0	Spring
5/28/2018	BL	1M	5	2018	Year-1	0	0	Spring
5/28/2018	BL	2M	5	2018	Year-1		1	Spring
5/28/2018	BL	3M	5	2018	Year-1		1	Spring
5/28/2018	BL	4M	5	2018	Year-1		1	Spring
5/28/2018	BL	5M	5	2018	Year-1		1	Spring
5/28/2018	BL	6M	5	2018	Year-1	0	0	Spring
5/28/2018	BL	7M	5	2018	Year-1	0	0	Spring
5/28/2018	BL	10M	5	2018	Year-1	0	0	Spring
5/28/2018	BL	12M	5	2018	Year-1	0	0	Spring
5/28/2018	BL	14M	5	2018	Year-1	0	0	Spring
5/28/2018	BL	8A	5	2018	Year-1	0	0	Spring
5/28/2018	BL	9A	5	2018	Year-1	0	0	Spring
5/28/2018	BL	11A	5	2018	Year-1	0	0	Spring
5/28/2018	BL	13A	5	2018	Year-1	0	0	Spring
5/29/2018	BL	1M	5	2018	Year-1	0	0	Spring
5/29/2018	BL	2M	5	2018	Year-1		1	Spring
5/29/2018	BL	3M	5	2018	Year-1		1	Spring
5/29/2018	BL	4M	5	2018	Year-1		1	Spring
5/29/2018	BL	5M	5	2018	Year-1		1	Spring
5/29/2018	BL	6M	5	2018	Year-1	0	0	Spring
5/29/2018	BL	7M	5	2018	Year-1	0	0	Spring
5/29/2018	BL	10M	5	2018	Year-1	0	0	Spring
5/29/2018	BL	12M	5	2018	Year-1	0	0	Spring
5/29/2018	BL	14M	5	2018	Year-1	0	0	Spring
5/29/2018	BL	8A	5	2018	Year-1	0	0	Spring

Date	Species	Site	Month	Year	Survey Year	Presence	Data Gap	Season
5/29/2018	BL	9A	5	2018	Year-1	0	0	Spring
5/29/2018	BL	11A	5	2018	Year-1	0	0	Spring
5/29/2018	BL	13A	5	2018	Year-1	0	0	Spring
5/30/2018	BL	1M	5	2018	Year-1	0	0	Spring
5/30/2018	BL	2M	5	2018	Year-1		1	Spring
5/30/2018	BL	3M	5	2018	Year-1		1	Spring
5/30/2018	BL	4M	5	2018	Year-1		1	Spring
5/30/2018	BL	5M	5	2018	Year-1		1	Spring
5/30/2018	BL	6M	5	2018	Year-1	0	0	Spring
5/30/2018	BL	7M	5	2018	Year-1	0	0	Spring
5/30/2018	BL	10M	5	2018	Year-1	0	0	Spring
5/30/2018	BL	12M	5	2018	Year-1	0	0	Spring
5/30/2018	BL	14M	5	2018	Year-1	0	0	Spring
5/30/2018	BL	8A	5	2018	Year-1	0	0	Spring
5/30/2018	BL	9A	5	2018	Year-1	0	0	Spring
5/30/2018	BL	11A	5	2018	Year-1	0	0	Spring
5/30/2018	BL	13A	5	2018	Year-1	0	0	Spring
5/31/2018	BL	1M	5	2018	Year-1	0	0	Spring
5/31/2018	BL	2M	5	2018	Year-1		1	Spring
5/31/2018	BL	3M	5	2018	Year-1		1	Spring
5/31/2018	BL	4M	5	2018	Year-1		1	Spring
5/31/2018	BL	5M	5	2018	Year-1		1	Spring
5/31/2018	BL	6M	5	2018	Year-1	0	0	Spring
5/31/2018	BL	7M	5	2018	Year-1	0	0	Spring
5/31/2018	BL	10M	5	2018	Year-1	0	0	Spring
5/31/2018	BL	12M	5	2018	Year-1	0	0	Spring
5/31/2018	BL	14M	5	2018	Year-1	0	0	Spring
5/31/2018	BL	8A	5	2018	Year-1	0	0	Spring
5/31/2018	BL	9A	5	2018	Year-1	0	0	Spring
5/31/2018	BL	11A	5	2018	Year-1	0	0	Spring
5/31/2018	BL	13A	5	2018	Year-1	0	0	Spring
6/1/2018	BL	1M	6	2018	Year-1	0	0	Summer
6/1/2018	BL	2M	6	2018	Year-1		1	Summer
6/1/2018	BL	3M	6	2018	Year-1		1	Summer
6/1/2018	BL	4M	6	2018	Year-1		1	Summer
6/1/2018	BL	5M	6	2018	Year-1		1	Summer
6/1/2018	BL	6M	6	2018	Year-1	0	0	Summer
6/1/2018	BL	7M	6	2018	Year-1	0	0	Summer
6/1/2018	BL	10M	6	2018	Year-1	0	0	Summer
6/1/2018	BL	12M	6	2018	Year-1	0	0	Summer
6/1/2018	BL	14M	6	2018	Year-1	0	0	Summer
6/1/2018	BL	8A	6	2018	Year-1	0	0	Summer
6/1/2018	BL	9A	6	2018	Year-1	0	0	Summer
6/1/2018	BL	11A	6	2018	Year-1	0	0	Summer

Date	Species	Site	Month	Year	Survey Year	Presence	Data Gap	Season
6/1/2018	BL	13A	6	2018	Year-1	0	0	Summer
6/2/2018	BL	1M	6	2018	Year-1	0	0	Summer
6/2/2018	BL	2M	6	2018	Year-1		1	Summer
6/2/2018	BL	3M	6	2018	Year-1		1	Summer
6/2/2018	BL	4M	6	2018	Year-1		1	Summer
6/2/2018	BL	5M	6	2018	Year-1		1	Summer
6/2/2018	BL	6M	6	2018	Year-1	0	0	Summer
6/2/2018	BL	7M	6	2018	Year-1	0	0	Summer
6/2/2018	BL	10M	6	2018	Year-1	0	0	Summer
6/2/2018	BL	12M	6	2018	Year-1	0	0	Summer
6/2/2018	BL	14M	6	2018	Year-1	0	0	Summer
6/2/2018	BL	8A	6	2018	Year-1	0	0	Summer
6/2/2018	BL	9A	6	2018	Year-1	0	0	Summer
6/2/2018	BL	11A	6	2018	Year-1	0	0	Summer
6/2/2018	BL	13A	6	2018	Year-1	0	0	Summer
6/3/2018	BL	1M	6	2018	Year-1	0	0	Summer
6/3/2018	BL	2M	6	2018	Year-1		1	Summer
6/3/2018	BL	3M	6	2018	Year-1		1	Summer
6/3/2018	BL	4M	6	2018	Year-1		1	Summer
6/3/2018	BL	5M	6	2018	Year-1		1	Summer
6/3/2018	BL	6M	6	2018	Year-1	0	0	Summer
6/3/2018	BL	7M	6	2018	Year-1	0	0	Summer
6/3/2018	BL	10M	6	2018	Year-1	0	0	Summer
6/3/2018	BL	12M	6	2018	Year-1	0	0	Summer
6/3/2018	BL	14M	6	2018	Year-1	0	0	Summer
6/3/2018	BL	8A	6	2018	Year-1	0	0	Summer
6/3/2018	BL	9A	6	2018	Year-1	0	0	Summer
6/3/2018	BL	11A	6	2018	Year-1	0	0	Summer
6/3/2018	BL	13A	6	2018	Year-1	0	0	Summer
6/4/2018	BL	1M	6	2018	Year-1	0	0	Summer
6/4/2018	BL	2M	6	2018	Year-1		1	Summer
6/4/2018	BL	3M	6	2018	Year-1		1	Summer
6/4/2018	BL	4M	6	2018	Year-1		1	Summer
6/4/2018	BL	5M	6	2018	Year-1		1	Summer
6/4/2018	BL	6M	6	2018	Year-1	0	0	Summer
6/4/2018	BL	7M	6	2018	Year-1	0	0	Summer
6/4/2018	BL	10M	6	2018	Year-1	0	0	Summer
6/4/2018	BL	12M	6	2018	Year-1	0	0	Summer
6/4/2018	BL	14M	6	2018	Year-1	0	0	Summer
6/4/2018	BL	8A	6	2018	Year-1	0	0	Summer
6/4/2018	BL	9A	6	2018	Year-1	0	0	Summer
6/4/2018	BL	11A	6	2018	Year-1	0	0	Summer
6/4/2018	BL	13A	6	2018	Year-1	0	0	Summer
6/5/2018	BL	1M	6	2018	Year-1	0	0	Summer

Date	Species	Site	Month	Year	Survey Year	Presence	Data Gap	Season
6/5/2018	BL	2M	6	2018	Year-1		1	Summer
6/5/2018	BL	3M	6	2018	Year-1		1	Summer
6/5/2018	BL	4M	6	2018	Year-1		1	Summer
6/5/2018	BL	5M	6	2018	Year-1		1	Summer
6/5/2018	BL	6M	6	2018	Year-1	0	0	Summer
6/5/2018	BL	7M	6	2018	Year-1	0	0	Summer
6/5/2018	BL	10M	6	2018	Year-1	0	0	Summer
6/5/2018	BL	12M	6	2018	Year-1	0	0	Summer
6/5/2018	BL	14M	6	2018	Year-1	0	0	Summer
6/5/2018	BL	8A	6	2018	Year-1	0	0	Summer
6/5/2018	BL	9A	6	2018	Year-1	0	0	Summer
6/5/2018	BL	11A	6	2018	Year-1	0	0	Summer
6/5/2018	BL	13A	6	2018	Year-1	0	0	Summer
6/6/2018	BL	1M	6	2018	Year-1	0	0	Summer
6/6/2018	BL	2M	6	2018	Year-1		1	Summer
6/6/2018	BL	3M	6	2018	Year-1		1	Summer
6/6/2018	BL	4M	6	2018	Year-1		1	Summer
6/6/2018	BL	5M	6	2018	Year-1		1	Summer
6/6/2018	BL	6M	6	2018	Year-1	0	0	Summer
6/6/2018	BL	7M	6	2018	Year-1	0	0	Summer
6/6/2018	BL	10M	6	2018	Year-1	0	0	Summer
6/6/2018	BL	12M	6	2018	Year-1	0	0	Summer
6/6/2018	BL	14M	6	2018	Year-1	0	0	Summer
6/6/2018	BL	8A	6	2018	Year-1	0	0	Summer
6/6/2018	BL	9A	6	2018	Year-1	0	0	Summer
6/6/2018	BL	11A	6	2018	Year-1	0	0	Summer
6/6/2018	BL	13A	6	2018	Year-1	0	0	Summer
6/7/2018	BL	1M	6	2018	Year-1	0	0	Summer
6/7/2018	BL	2M	6	2018	Year-1		1	Summer
6/7/2018	BL	3M	6	2018	Year-1		1	Summer
6/7/2018	BL	4M	6	2018	Year-1		1	Summer
6/7/2018	BL	5M	6	2018	Year-1		1	Summer
6/7/2018	BL	6M	6	2018	Year-1	0	0	Summer
6/7/2018	BL	7M	6	2018	Year-1	0	0	Summer
6/7/2018	BL	10M	6	2018	Year-1	0	0	Summer
6/7/2018	BL	12M	6	2018	Year-1	0	0	Summer
6/7/2018	BL	14M	6	2018	Year-1	0	0	Summer
6/7/2018	BL	8A	6	2018	Year-1	0	0	Summer
6/7/2018	BL	9A	6	2018	Year-1	0	0	Summer
6/7/2018	BL	11A	6	2018	Year-1	0	0	Summer
6/7/2018	BL	13A	6	2018	Year-1	0	0	Summer
6/8/2018	BL	1M	6	2018	Year-1	0	0	Summer
6/8/2018	BL	2M	6	2018	Year-1		1	Summer
6/8/2018	BL	3M	6	2018	Year-1		1	Summer

Date	Species	Site	Month	Year	Survey Year	Presence	Data Gap	Season
6/8/2018	BL	4M	6	2018	Year-1		1	Summer
6/8/2018	BL	5M	6	2018	Year-1		1	Summer
6/8/2018	BL	6M	6	2018	Year-1	0	0	Summer
6/8/2018	BL	7M	6	2018	Year-1	0	0	Summer
6/8/2018	BL	10M	6	2018	Year-1	0	0	Summer
6/8/2018	BL	12M	6	2018	Year-1	0	0	Summer
6/8/2018	BL	14M	6	2018	Year-1	0	0	Summer
6/8/2018	BL	8A	6	2018	Year-1	0	0	Summer
6/8/2018	BL	9A	6	2018	Year-1	0	0	Summer
6/8/2018	BL	11A	6	2018	Year-1	0	0	Summer
6/8/2018	BL	13A	6	2018	Year-1	0	0	Summer
6/9/2018	BL	1M	6	2018	Year-1	0	0	Summer
6/9/2018	BL	2M	6	2018	Year-1		1	Summer
6/9/2018	BL	3M	6	2018	Year-1		1	Summer
6/9/2018	BL	4M	6	2018	Year-1		1	Summer
6/9/2018	BL	5M	6	2018	Year-1		1	Summer
6/9/2018	BL	6M	6	2018	Year-1	0	0	Summer
6/9/2018	BL	7M	6	2018	Year-1	0	0	Summer
6/9/2018	BL	10M	6	2018	Year-1	0	0	Summer
6/9/2018	BL	12M	6	2018	Year-1	0	0	Summer
6/9/2018	BL	14M	6	2018	Year-1	0	0	Summer
6/9/2018	BL	8A	6	2018	Year-1	0	0	Summer
6/9/2018	BL	9A	6	2018	Year-1	0	0	Summer
6/9/2018	BL	11A	6	2018	Year-1	0	0	Summer
6/9/2018	BL	13A	6	2018	Year-1	0	0	Summer
6/10/2018	BL	1M	6	2018	Year-1	0	0	Summer
6/10/2018	BL	2M	6	2018	Year-1		1	Summer
6/10/2018	BL	3M	6	2018	Year-1		1	Summer
6/10/2018	BL	4M	6	2018	Year-1		1	Summer
6/10/2018	BL	5M	6	2018	Year-1		1	Summer
6/10/2018	BL	6M	6	2018	Year-1	0	0	Summer
6/10/2018	BL	7M	6	2018	Year-1	0	0	Summer
6/10/2018	BL	10M	6	2018	Year-1	0	0	Summer
6/10/2018	BL	12M	6	2018	Year-1	0	0	Summer
6/10/2018	BL	14M	6	2018	Year-1	0	0	Summer
6/10/2018	BL	8A	6	2018	Year-1	0	0	Summer
6/10/2018	BL	9A	6	2018	Year-1	0	0	Summer
6/10/2018	BL	11A	6	2018	Year-1	0	0	Summer
6/10/2018	BL	13A	6	2018	Year-1	0	0	Summer
6/11/2018	BL	1M	6	2018	Year-1	0	0	Summer
6/11/2018	BL	2M	6	2018	Year-1		1	Summer
6/11/2018	BL	3M	6	2018	Year-1		1	Summer
6/11/2018	BL	4M	6	2018	Year-1		1	Summer
6/11/2018	BL	5M	6	2018	Year-1		1	Summer

Date	Species	Site	Month	Year	Survey Year	Presence	Data Gap	Season
6/11/2018	BL	6M	6	2018	Year-1	0	0	Summer
6/11/2018	BL	7M	6	2018	Year-1	0	0	Summer
6/11/2018	BL	10M	6	2018	Year-1	0	0	Summer
6/11/2018	BL	12M	6	2018	Year-1	0	0	Summer
6/11/2018	BL	14M	6	2018	Year-1	0	0	Summer
6/11/2018	BL	8A	6	2018	Year-1	0	0	Summer
6/11/2018	BL	9A	6	2018	Year-1	0	0	Summer
6/11/2018	BL	11A	6	2018	Year-1	0	0	Summer
6/11/2018	BL	13A	6	2018	Year-1	0	0	Summer
6/12/2018	BL	1M	6	2018	Year-1	0	0	Summer
6/12/2018	BL	2M	6	2018	Year-1		1	Summer
6/12/2018	BL	3M	6	2018	Year-1		1	Summer
6/12/2018	BL	4M	6	2018	Year-1		1	Summer
6/12/2018	BL	5M	6	2018	Year-1		1	Summer
6/12/2018	BL	6M	6	2018	Year-1	0	0	Summer
6/12/2018	BL	7M	6	2018	Year-1	0	0	Summer
6/12/2018	BL	10M	6	2018	Year-1	0	0	Summer
6/12/2018	BL	12M	6	2018	Year-1	0	0	Summer
6/12/2018	BL	14M	6	2018	Year-1	0	0	Summer
6/12/2018	BL	8A	6	2018	Year-1	0	0	Summer
6/12/2018	BL	9A	6	2018	Year-1	0	0	Summer
6/12/2018	BL	11A	6	2018	Year-1	0	0	Summer
6/12/2018	BL	13A	6	2018	Year-1	0	0	Summer
6/13/2018	BL	1M	6	2018	Year-1	0	0	Summer
6/13/2018	BL	2M	6	2018	Year-1		1	Summer
6/13/2018	BL	3M	6	2018	Year-1		1	Summer
6/13/2018	BL	4M	6	2018	Year-1		1	Summer
6/13/2018	BL	5M	6	2018	Year-1		1	Summer
6/13/2018	BL	6M	6	2018	Year-1	0	0	Summer
6/13/2018	BL	7M	6	2018	Year-1	0	0	Summer
6/13/2018	BL	10M	6	2018	Year-1	0	0	Summer
6/13/2018	BL	12M	6	2018	Year-1	0	0	Summer
6/13/2018	BL	14M	6	2018	Year-1	0	0	Summer
6/13/2018	BL	8A	6	2018	Year-1	0	0	Summer
6/13/2018	BL	9A	6	2018	Year-1	0	0	Summer
6/13/2018	BL	11A	6	2018	Year-1	0	0	Summer
6/13/2018	BL	13A	6	2018	Year-1	0	0	Summer
6/14/2018	BL	1M	6	2018	Year-1	0	0	Summer
6/14/2018	BL	2M	6	2018	Year-1		1	Summer
6/14/2018	BL	3M	6	2018	Year-1		1	Summer
6/14/2018	BL	4M	6	2018	Year-1		1	Summer
6/14/2018	BL	5M	6	2018	Year-1		1	Summer
6/14/2018	BL	6M	6	2018	Year-1	0	0	Summer
6/14/2018	BL	7M	6	2018	Year-1	0	0	Summer

Date	Species	Site	Month	Year	Survey Year	Presence	Data Gap	Season
6/14/2018	BL	10M	6	2018	Year-1	0	0	Summer
6/14/2018	BL	12M	6	2018	Year-1	0	0	Summer
6/14/2018	BL	14M	6	2018	Year-1	0	0	Summer
6/14/2018	BL	8A	6	2018	Year-1	0	0	Summer
6/14/2018	BL	9A	6	2018	Year-1	0	0	Summer
6/14/2018	BL	11A	6	2018	Year-1	0	0	Summer
6/14/2018	BL	13A	6	2018	Year-1	0	0	Summer
6/15/2018	BL	1M	6	2018	Year-1	0	0	Summer
6/15/2018	BL	2M	6	2018	Year-1		1	Summer
6/15/2018	BL	3M	6	2018	Year-1		1	Summer
6/15/2018	BL	4M	6	2018	Year-1		1	Summer
6/15/2018	BL	5M	6	2018	Year-1		1	Summer
6/15/2018	BL	6M	6	2018	Year-1	0	0	Summer
6/15/2018	BL	7M	6	2018	Year-1	0	0	Summer
6/15/2018	BL	10M	6	2018	Year-1	0	0	Summer
6/15/2018	BL	12M	6	2018	Year-1	0	0	Summer
6/15/2018	BL	14M	6	2018	Year-1	0	0	Summer
6/15/2018	BL	8A	6	2018	Year-1	0	0	Summer
6/15/2018	BL	9A	6	2018	Year-1	0	0	Summer
6/15/2018	BL	11A	6	2018	Year-1	0	0	Summer
6/15/2018	BL	13A	6	2018	Year-1	0	0	Summer
6/16/2018	BL	1M	6	2018	Year-1	0	0	Summer
6/16/2018	BL	2M	6	2018	Year-1		1	Summer
6/16/2018	BL	3M	6	2018	Year-1		1	Summer
6/16/2018	BL	4M	6	2018	Year-1		1	Summer
6/16/2018	BL	5M	6	2018	Year-1		1	Summer
6/16/2018	BL	6M	6	2018	Year-1	0	0	Summer
6/16/2018	BL	7M	6	2018	Year-1	0	0	Summer
6/16/2018	BL	10M	6	2018	Year-1	0	0	Summer
6/16/2018	BL	12M	6	2018	Year-1	0	0	Summer
6/16/2018	BL	14M	6	2018	Year-1	0	0	Summer
6/16/2018	BL	8A	6	2018	Year-1	0	0	Summer
6/16/2018	BL	9A	6	2018	Year-1	0	0	Summer
6/16/2018	BL	11A	6	2018	Year-1	0	0	Summer
6/16/2018	BL	13A	6	2018	Year-1	0	0	Summer
6/17/2018	BL	1M	6	2018	Year-1	0	0	Summer
6/17/2018	BL	2M	6	2018	Year-1		1	Summer
6/17/2018	BL	3M	6	2018	Year-1		1	Summer
6/17/2018	BL	4M	6	2018	Year-1		1	Summer
6/17/2018	BL	5M	6	2018	Year-1		1	Summer
6/17/2018	BL	6M	6	2018	Year-1	0	0	Summer
6/17/2018	BL	7M	6	2018	Year-1	0	0	Summer
6/17/2018	BL	10M	6	2018	Year-1	0	0	Summer
6/17/2018	BL	12M	6	2018	Year-1	0	0	Summer

Date	Species	Site	Month	Year	Survey Year	Presence	Data Gap	Season
6/17/2018	BL	14M	6	2018	Year-1	0	0	Summer
6/17/2018	BL	8A	6	2018	Year-1	0	0	Summer
6/17/2018	BL	9A	6	2018	Year-1	0	0	Summer
6/17/2018	BL	11A	6	2018	Year-1	0	0	Summer
6/17/2018	BL	13A	6	2018	Year-1	0	0	Summer
6/18/2018	BL	1M	6	2018	Year-1	0	0	Summer
6/18/2018	BL	2M	6	2018	Year-1		1	Summer
6/18/2018	BL	3M	6	2018	Year-1		1	Summer
6/18/2018	BL	4M	6	2018	Year-1		1	Summer
6/18/2018	BL	5M	6	2018	Year-1		1	Summer
6/18/2018	BL	6M	6	2018	Year-1	0	0	Summer
6/18/2018	BL	7M	6	2018	Year-1	0	0	Summer
6/18/2018	BL	10M	6	2018	Year-1	0	0	Summer
6/18/2018	BL	12M	6	2018	Year-1	0	0	Summer
6/18/2018	BL	14M	6	2018	Year-1	0	0	Summer
6/18/2018	BL	8A	6	2018	Year-1	0	0	Summer
6/18/2018	BL	9A	6	2018	Year-1	0	0	Summer
6/18/2018	BL	11A	6	2018	Year-1	0	0	Summer
6/18/2018	BL	13A	6	2018	Year-1	0	0	Summer
6/19/2018	BL	1M	6	2018	Year-1	0	0	Summer
6/19/2018	BL	2M	6	2018	Year-1		1	Summer
6/19/2018	BL	3M	6	2018	Year-1		1	Summer
6/19/2018	BL	4M	6	2018	Year-1		1	Summer
6/19/2018	BL	5M	6	2018	Year-1		1	Summer
6/19/2018	BL	6M	6	2018	Year-1	0	0	Summer
6/19/2018	BL	7M	6	2018	Year-1	0	0	Summer
6/19/2018	BL	10M	6	2018	Year-1	0	0	Summer
6/19/2018	BL	12M	6	2018	Year-1	0	0	Summer
6/19/2018	BL	14M	6	2018	Year-1	0	0	Summer
6/19/2018	BL	8A	6	2018	Year-1	0	0	Summer
6/19/2018	BL	9A	6	2018	Year-1	0	0	Summer
6/19/2018	BL	11A	6	2018	Year-1	0	0	Summer
6/19/2018	BL	13A	6	2018	Year-1	0	0	Summer
6/20/2018	BL	1M	6	2018	Year-1	0	0	Summer
6/20/2018	BL	2M	6	2018	Year-1		1	Summer
6/20/2018	BL	3M	6	2018	Year-1		1	Summer
6/20/2018	BL	4M	6	2018	Year-1		1	Summer
6/20/2018	BL	5M	6	2018	Year-1		1	Summer
6/20/2018	BL	6M	6	2018	Year-1	0	0	Summer
6/20/2018	BL	7M	6	2018	Year-1	0	0	Summer
6/20/2018	BL	10M	6	2018	Year-1	0	0	Summer
6/20/2018	BL	12M	6	2018	Year-1	0	0	Summer
6/20/2018	BL	14M	6	2018	Year-1	0	0	Summer
6/20/2018	BL	8A	6	2018	Year-1	0	0	Summer

Date	Species	Site	Month	Year	Survey Year	Presence	Data Gap	Season
6/20/2018	BL	9A	6	2018	Year-1	0	0	Summer
6/20/2018	BL	11A	6	2018	Year-1	0	0	Summer
6/20/2018	BL	13A	6	2018	Year-1	0	0	Summer
6/21/2018	BL	1M	6	2018	Year-1	0	0	Summer
6/21/2018	BL	2M	6	2018	Year-1		1	Summer
6/21/2018	BL	3M	6	2018	Year-1		1	Summer
6/21/2018	BL	4M	6	2018	Year-1		1	Summer
6/21/2018	BL	5M	6	2018	Year-1		1	Summer
6/21/2018	BL	6M	6	2018	Year-1	0	0	Summer
6/21/2018	BL	7M	6	2018	Year-1	0	0	Summer
6/21/2018	BL	10M	6	2018	Year-1	0	0	Summer
6/21/2018	BL	12M	6	2018	Year-1	0	0	Summer
6/21/2018	BL	14M	6	2018	Year-1	0	0	Summer
6/21/2018	BL	8A	6	2018	Year-1	0	0	Summer
6/21/2018	BL	9A	6	2018	Year-1	0	0	Summer
6/21/2018	BL	11A	6	2018	Year-1	0	0	Summer
6/21/2018	BL	13A	6	2018	Year-1	0	0	Summer
6/22/2018	BL	1M	6	2018	Year-1	0	0	Summer
6/22/2018	BL	2M	6	2018	Year-1		1	Summer
6/22/2018	BL	3M	6	2018	Year-1		1	Summer
6/22/2018	BL	4M	6	2018	Year-1		1	Summer
6/22/2018	BL	5M	6	2018	Year-1		1	Summer
6/22/2018	BL	6M	6	2018	Year-1	0	0	Summer
6/22/2018	BL	7M	6	2018	Year-1	0	0	Summer
6/22/2018	BL	10M	6	2018	Year-1	0	0	Summer
6/22/2018	BL	12M	6	2018	Year-1	0	0	Summer
6/22/2018	BL	14M	6	2018	Year-1	0	0	Summer
6/22/2018	BL	8A	6	2018	Year-1	0	0	Summer
6/22/2018	BL	9A	6	2018	Year-1	0	0	Summer
6/22/2018	BL	11A	6	2018	Year-1	0	0	Summer
6/22/2018	BL	13A	6	2018	Year-1	0	0	Summer
6/23/2018	BL	1M	6	2018	Year-1	0	0	Summer
6/23/2018	BL	2M	6	2018	Year-1		1	Summer
6/23/2018	BL	3M	6	2018	Year-1		1	Summer
6/23/2018	BL	4M	6	2018	Year-1		1	Summer
6/23/2018	BL	5M	6	2018	Year-1		1	Summer
6/23/2018	BL	6M	6	2018	Year-1	0	0	Summer
6/23/2018	BL	7M	6	2018	Year-1	0	0	Summer
6/23/2018	BL	10M	6	2018	Year-1	0	0	Summer
6/23/2018	BL	12M	6	2018	Year-1	0	0	Summer
6/23/2018	BL	14M	6	2018	Year-1	0	0	Summer
6/23/2018	BL	8A	6	2018	Year-1	0	0	Summer
6/23/2018	BL	9A	6	2018	Year-1	0	0	Summer
6/23/2018	BL	11A	6	2018	Year-1	0	0	Summer

Date	Species	Site	Month	Year	Survey Year	Presence	Data Gap	Season
6/23/2018	BL	13A	6	2018	Year-1	0	0	Summer
6/24/2018	BL	1M	6	2018	Year-1	0	0	Summer
6/24/2018	BL	2M	6	2018	Year-1		1	Summer
6/24/2018	BL	3M	6	2018	Year-1		1	Summer
6/24/2018	BL	4M	6	2018	Year-1		1	Summer
6/24/2018	BL	5M	6	2018	Year-1		1	Summer
6/24/2018	BL	6M	6	2018	Year-1	0	0	Summer
6/24/2018	BL	7M	6	2018	Year-1	0	0	Summer
6/24/2018	BL	10M	6	2018	Year-1	0	0	Summer
6/24/2018	BL	12M	6	2018	Year-1	0	0	Summer
6/24/2018	BL	14M	6	2018	Year-1	0	0	Summer
6/24/2018	BL	8A	6	2018	Year-1	0	0	Summer
6/24/2018	BL	9A	6	2018	Year-1	0	0	Summer
6/24/2018	BL	11A	6	2018	Year-1	0	0	Summer
6/24/2018	BL	13A	6	2018	Year-1	0	0	Summer
6/25/2018	BL	1M	6	2018	Year-1	0	0	Summer
6/25/2018	BL	2M	6	2018	Year-1		1	Summer
6/25/2018	BL	3M	6	2018	Year-1		1	Summer
6/25/2018	BL	4M	6	2018	Year-1		1	Summer
6/25/2018	BL	5M	6	2018	Year-1		1	Summer
6/25/2018	BL	6M	6	2018	Year-1	0	0	Summer
6/25/2018	BL	7M	6	2018	Year-1	0	0	Summer
6/25/2018	BL	10M	6	2018	Year-1	0	0	Summer
6/25/2018	BL	12M	6	2018	Year-1	0	0	Summer
6/25/2018	BL	14M	6	2018	Year-1	0	0	Summer
6/25/2018	BL	8A	6	2018	Year-1	0	0	Summer
6/25/2018	BL	9A	6	2018	Year-1	0	0	Summer
6/25/2018	BL	11A	6	2018	Year-1	0	0	Summer
6/25/2018	BL	13A	6	2018	Year-1	0	0	Summer
6/26/2018	BL	1M	6	2018	Year-1	0	0	Summer
6/26/2018	BL	2M	6	2018	Year-1		1	Summer
6/26/2018	BL	3M	6	2018	Year-1		1	Summer
6/26/2018	BL	4M	6	2018	Year-1		1	Summer
6/26/2018	BL	5M	6	2018	Year-1		1	Summer
6/26/2018	BL	6M	6	2018	Year-1	0	0	Summer
6/26/2018	BL	7M	6	2018	Year-1	0	0	Summer
6/26/2018	BL	10M	6	2018	Year-1	0	0	Summer
6/26/2018	BL	12M	6	2018	Year-1	0	0	Summer
6/26/2018	BL	14M	6	2018	Year-1	0	0	Summer
6/26/2018	BL	8A	6	2018	Year-1	0	0	Summer
6/26/2018	BL	9A	6	2018	Year-1	0	0	Summer
6/26/2018	BL	11A	6	2018	Year-1	0	0	Summer
6/26/2018	BL	13A	6	2018	Year-1	0	0	Summer
6/27/2018	BL	1M	6	2018	Year-1	0	0	Summer

Date	Species	Site	Month	Year	Survey Year	Presence	Data Gap	Season
6/27/2018	BL	2M	6	2018	Year-1		1	Summer
6/27/2018	BL	3M	6	2018	Year-1		1	Summer
6/27/2018	BL	4M	6	2018	Year-1		1	Summer
6/27/2018	BL	5M	6	2018	Year-1		1	Summer
6/27/2018	BL	6M	6	2018	Year-1	0	0	Summer
6/27/2018	BL	7M	6	2018	Year-1		1	Summer
6/27/2018	BL	10M	6	2018	Year-1	0	0	Summer
6/27/2018	BL	12M	6	2018	Year-1	0	0	Summer
6/27/2018	BL	14M	6	2018	Year-1	0	0	Summer
6/27/2018	BL	8A	6	2018	Year-1	0	0	Summer
6/27/2018	BL	9A	6	2018	Year-1	0	0	Summer
6/27/2018	BL	11A	6	2018	Year-1	0	0	Summer
6/27/2018	BL	13A	6	2018	Year-1	0	0	Summer
6/28/2018	BL	1M	6	2018	Year-1	0	0	Summer
6/28/2018	BL	2M	6	2018	Year-1		1	Summer
6/28/2018	BL	3M	6	2018	Year-1		1	Summer
6/28/2018	BL	4M	6	2018	Year-1		1	Summer
6/28/2018	BL	5M	6	2018	Year-1		1	Summer
6/28/2018	BL	6M	6	2018	Year-1	0	0	Summer
6/28/2018	BL	7M	6	2018	Year-1		1	Summer
6/28/2018	BL	10M	6	2018	Year-1	0	0	Summer
6/28/2018	BL	12M	6	2018	Year-1	0	0	Summer
6/28/2018	BL	14M	6	2018	Year-1	0	0	Summer
6/28/2018	BL	8A	6	2018	Year-1	0	0	Summer
6/28/2018	BL	9A	6	2018	Year-1	0	0	Summer
6/28/2018	BL	11A	6	2018	Year-1	0	0	Summer
6/28/2018	BL	13A	6	2018	Year-1	0	0	Summer
6/29/2018	BL	1M	6	2018	Year-1	0	0	Summer
6/29/2018	BL	2M	6	2018	Year-1		1	Summer
6/29/2018	BL	3M	6	2018	Year-1		1	Summer
6/29/2018	BL	4M	6	2018	Year-1		1	Summer
6/29/2018	BL	5M	6	2018	Year-1		1	Summer
6/29/2018	BL	6M	6	2018	Year-1	0	0	Summer
6/29/2018	BL	7M	6	2018	Year-1		1	Summer
6/29/2018	BL	10M	6	2018	Year-1	0	0	Summer
6/29/2018	BL	12M	6	2018	Year-1	0	0	Summer
6/29/2018	BL	14M	6	2018	Year-1	0	0	Summer
6/29/2018	BL	8A	6	2018	Year-1	0	0	Summer
6/29/2018	BL	9A	6	2018	Year-1	0	0	Summer
6/29/2018	BL	11A	6	2018	Year-1	0	0	Summer
6/29/2018	BL	13A	6	2018	Year-1	0	0	Summer
6/30/2018	BL	1M	6	2018	Year-1	0	0	Summer
6/30/2018	BL	2M	6	2018	Year-1		1	Summer
6/30/2018	BL	3M	6	2018	Year-1		1	Summer

Date	Species	Site	Month	Year	Survey Year	Presence	Data Gap	Season
6/30/2018	BL	4M	6	2018	Year-1		1	Summer
6/30/2018	BL	5M	6	2018	Year-1		1	Summer
6/30/2018	BL	6M	6	2018	Year-1	0	0	Summer
6/30/2018	BL	7M	6	2018	Year-1		1	Summer
6/30/2018	BL	10M	6	2018	Year-1	0	0	Summer
6/30/2018	BL	12M	6	2018	Year-1	0	0	Summer
6/30/2018	BL	14M	6	2018	Year-1	0	0	Summer
6/30/2018	BL	8A	6	2018	Year-1	0	0	Summer
6/30/2018	BL	9A	6	2018	Year-1	0	0	Summer
6/30/2018	BL	11A	6	2018	Year-1	0	0	Summer
6/30/2018	BL	13A	6	2018	Year-1	0	0	Summer
7/1/2018	BL	1M	7	2018	Year-1	0	0	Summer
7/1/2018	BL	2M	7	2018	Year-1		1	Summer
7/1/2018	BL	3M	7	2018	Year-1		1	Summer
7/1/2018	BL	4M	7	2018	Year-1		1	Summer
7/1/2018	BL	5M	7	2018	Year-1		1	Summer
7/1/2018	BL	6M	7	2018	Year-1	0	0	Summer
7/1/2018	BL	7M	7	2018	Year-1		1	Summer
7/1/2018	BL	10M	7	2018	Year-1	0	0	Summer
7/1/2018	BL	12M	7	2018	Year-1	0	0	Summer
7/1/2018	BL	14M	7	2018	Year-1	0	0	Summer
7/1/2018	BL	8A	7	2018	Year-1	0	0	Summer
7/1/2018	BL	9A	7	2018	Year-1	0	0	Summer
7/1/2018	BL	11A	7	2018	Year-1	0	0	Summer
7/1/2018	BL	13A	7	2018	Year-1	0	0	Summer
7/2/2018	BL	1M	7	2018	Year-1	0	0	Summer
7/2/2018	BL	2M	7	2018	Year-1		1	Summer
7/2/2018	BL	3M	7	2018	Year-1		1	Summer
7/2/2018	BL	4M	7	2018	Year-1		1	Summer
7/2/2018	BL	5M	7	2018	Year-1		1	Summer
7/2/2018	BL	6M	7	2018	Year-1	0	0	Summer
7/2/2018	BL	7M	7	2018	Year-1		1	Summer
7/2/2018	BL	10M	7	2018	Year-1	0	0	Summer
7/2/2018	BL	12M	7	2018	Year-1	0	0	Summer
7/2/2018	BL	14M	7	2018	Year-1	0	0	Summer
7/2/2018	BL	8A	7	2018	Year-1	0	0	Summer
7/2/2018	BL	9A	7	2018	Year-1	0	0	Summer
7/2/2018	BL	11A	7	2018	Year-1	0	0	Summer
7/2/2018	BL	13A	7	2018	Year-1	0	0	Summer
7/3/2018	BL	1M	7	2018	Year-1	0	0	Summer
7/3/2018	BL	2M	7	2018	Year-1		1	Summer
7/3/2018	BL	3M	7	2018	Year-1		1	Summer
7/3/2018	BL	4M	7	2018	Year-1		1	Summer
7/3/2018	BL	5M	7	2018	Year-1		1	Summer

Date	Species	Site	Month	Year	Survey Year	Presence	Data Gap	Season
7/3/2018	BL	6M	7	2018	Year-1	0	0	Summer
7/3/2018	BL	7M	7	2018	Year-1		1	Summer
7/3/2018	BL	10M	7	2018	Year-1	0	0	Summer
7/3/2018	BL	12M	7	2018	Year-1	0	0	Summer
7/3/2018	BL	14M	7	2018	Year-1	0	0	Summer
7/3/2018	BL	8A	7	2018	Year-1	0	0	Summer
7/3/2018	BL	9A	7	2018	Year-1	0	0	Summer
7/3/2018	BL	11A	7	2018	Year-1	0	0	Summer
7/3/2018	BL	13A	7	2018	Year-1	0	0	Summer
7/4/2018	BL	1M	7	2018	Year-1	0	0	Summer
7/4/2018	BL	2M	7	2018	Year-1		1	Summer
7/4/2018	BL	3M	7	2018	Year-1		1	Summer
7/4/2018	BL	4M	7	2018	Year-1		1	Summer
7/4/2018	BL	5M	7	2018	Year-1		1	Summer
7/4/2018	BL	6M	7	2018	Year-1	0	0	Summer
7/4/2018	BL	7M	7	2018	Year-1		1	Summer
7/4/2018	BL	10M	7	2018	Year-1	0	0	Summer
7/4/2018	BL	12M	7	2018	Year-1	0	0	Summer
7/4/2018	BL	14M	7	2018	Year-1	0	0	Summer
7/4/2018	BL	8A	7	2018	Year-1	0	0	Summer
7/4/2018	BL	9A	7	2018	Year-1	0	0	Summer
7/4/2018	BL	11A	7	2018	Year-1	0	0	Summer
7/4/2018	BL	13A	7	2018	Year-1	0	0	Summer
7/5/2018	BL	1M	7	2018	Year-1	0	0	Summer
7/5/2018	BL	2M	7	2018	Year-1		1	Summer
7/5/2018	BL	3M	7	2018	Year-1		1	Summer
7/5/2018	BL	4M	7	2018	Year-1		1	Summer
7/5/2018	BL	5M	7	2018	Year-1		1	Summer
7/5/2018	BL	6M	7	2018	Year-1	0	0	Summer
7/5/2018	BL	7M	7	2018	Year-1		1	Summer
7/5/2018	BL	10M	7	2018	Year-1	0	0	Summer
7/5/2018	BL	12M	7	2018	Year-1	0	0	Summer
7/5/2018	BL	14M	7	2018	Year-1	0	0	Summer
7/5/2018	BL	8A	7	2018	Year-1	0	0	Summer
7/5/2018	BL	9A	7	2018	Year-1	0	0	Summer
7/5/2018	BL	11A	7	2018	Year-1	0	0	Summer
7/5/2018	BL	13A	7	2018	Year-1	0	0	Summer
7/6/2018	BL	1M	7	2018	Year-1	0	0	Summer
7/6/2018	BL	2M	7	2018	Year-1		1	Summer
7/6/2018	BL	3M	7	2018	Year-1		1	Summer
7/6/2018	BL	4M	7	2018	Year-1		1	Summer
7/6/2018	BL	5M	7	2018	Year-1		1	Summer
7/6/2018	BL	6M	7	2018	Year-1	0	0	Summer
7/6/2018	BL	7M	7	2018	Year-1		1	Summer

Date	Species	Site	Month	Year	Survey Year	Presence	Data Gap	Season
7/6/2018	BL	10M	7	2018	Year-1	0	0	Summer
7/6/2018	BL	12M	7	2018	Year-1	0	0	Summer
7/6/2018	BL	14M	7	2018	Year-1	0	0	Summer
7/6/2018	BL	8A	7	2018	Year-1	0	0	Summer
7/6/2018	BL	9A	7	2018	Year-1	0	0	Summer
7/6/2018	BL	11A	7	2018	Year-1	0	0	Summer
7/6/2018	BL	13A	7	2018	Year-1	0	0	Summer
7/7/2018	BL	1M	7	2018	Year-1	0	0	Summer
7/7/2018	BL	2M	7	2018	Year-1		1	Summer
7/7/2018	BL	3M	7	2018	Year-1		1	Summer
7/7/2018	BL	4M	7	2018	Year-1		1	Summer
7/7/2018	BL	5M	7	2018	Year-1		1	Summer
7/7/2018	BL	6M	7	2018	Year-1	0	0	Summer
7/7/2018	BL	7M	7	2018	Year-1		1	Summer
7/7/2018	BL	10M	7	2018	Year-1	0	0	Summer
7/7/2018	BL	12M	7	2018	Year-1	0	0	Summer
7/7/2018	BL	14M	7	2018	Year-1	0	0	Summer
7/7/2018	BL	8A	7	2018	Year-1	0	0	Summer
7/7/2018	BL	9A	7	2018	Year-1	0	0	Summer
7/7/2018	BL	11A	7	2018	Year-1	0	0	Summer
7/7/2018	BL	13A	7	2018	Year-1	0	0	Summer
7/8/2018	BL	1M	7	2018	Year-1	0	0	Summer
7/8/2018	BL	2M	7	2018	Year-1		1	Summer
7/8/2018	BL	3M	7	2018	Year-1		1	Summer
7/8/2018	BL	4M	7	2018	Year-1		1	Summer
7/8/2018	BL	5M	7	2018	Year-1		1	Summer
7/8/2018	BL	6M	7	2018	Year-1	0	0	Summer
7/8/2018	BL	7M	7	2018	Year-1		1	Summer
7/8/2018	BL	10M	7	2018	Year-1	0	0	Summer
7/8/2018	BL	12M	7	2018	Year-1	0	0	Summer
7/8/2018	BL	14M	7	2018	Year-1	0	0	Summer
7/8/2018	BL	8A	7	2018	Year-1	0	0	Summer
7/8/2018	BL	9A	7	2018	Year-1	0	0	Summer
7/8/2018	BL	11A	7	2018	Year-1	0	0	Summer
7/8/2018	BL	13A	7	2018	Year-1	0	0	Summer
7/9/2018	BL	1M	7	2018	Year-1	0	0	Summer
7/9/2018	BL	2M	7	2018	Year-1		1	Summer
7/9/2018	BL	3M	7	2018	Year-1		1	Summer
7/9/2018	BL	4M	7	2018	Year-1		1	Summer
7/9/2018	BL	5M	7	2018	Year-1		1	Summer
7/9/2018	BL	6M	7	2018	Year-1	0	0	Summer
7/9/2018	BL	7M	7	2018	Year-1		1	Summer
7/9/2018	BL	10M	7	2018	Year-1	0	0	Summer
7/9/2018	BL	12M	7	2018	Year-1	0	0	Summer

Date	Species	Site	Month	Year	Survey Year	Presence	Data Gap	Season
7/9/2018	BL	14M	7	2018	Year-1	0	0	Summer
7/9/2018	BL	8A	7	2018	Year-1	0	0	Summer
7/9/2018	BL	9A	7	2018	Year-1	0	0	Summer
7/9/2018	BL	11A	7	2018	Year-1	0	0	Summer
7/9/2018	BL	13A	7	2018	Year-1	0	0	Summer
7/10/2018	BL	1M	7	2018	Year-1	0	0	Summer
7/10/2018	BL	2M	7	2018	Year-1		1	Summer
7/10/2018	BL	3M	7	2018	Year-1		1	Summer
7/10/2018	BL	4M	7	2018	Year-1		1	Summer
7/10/2018	BL	5M	7	2018	Year-1		1	Summer
7/10/2018	BL	6M	7	2018	Year-1	0	0	Summer
7/10/2018	BL	7M	7	2018	Year-1		1	Summer
7/10/2018	BL	10M	7	2018	Year-1	0	0	Summer
7/10/2018	BL	12M	7	2018	Year-1	0	0	Summer
7/10/2018	BL	14M	7	2018	Year-1	0	0	Summer
7/10/2018	BL	8A	7	2018	Year-1	0	0	Summer
7/10/2018	BL	9A	7	2018	Year-1	0	0	Summer
7/10/2018	BL	11A	7	2018	Year-1	0	0	Summer
7/10/2018	BL	13A	7	2018	Year-1	0	0	Summer
7/11/2018	BL	1M	7	2018	Year-1	0	0	Summer
7/11/2018	BL	2M	7	2018	Year-1		1	Summer
7/11/2018	BL	3M	7	2018	Year-1		1	Summer
7/11/2018	BL	4M	7	2018	Year-1		1	Summer
7/11/2018	BL	5M	7	2018	Year-1		1	Summer
7/11/2018	BL	6M	7	2018	Year-1	0	0	Summer
7/11/2018	BL	7M	7	2018	Year-1		1	Summer
7/11/2018	BL	10M	7	2018	Year-1	0	0	Summer
7/11/2018	BL	12M	7	2018	Year-1	0	0	Summer
7/11/2018	BL	14M	7	2018	Year-1	0	0	Summer
7/11/2018	BL	8A	7	2018	Year-1	0	0	Summer
7/11/2018	BL	9A	7	2018	Year-1	0	0	Summer
7/11/2018	BL	11A	7	2018	Year-1	0	0	Summer
7/11/2018	BL	13A	7	2018	Year-1	0	0	Summer
7/12/2018	BL	1M	7	2018	Year-1	0	0	Summer
7/12/2018	BL	2M	7	2018	Year-1		1	Summer
7/12/2018	BL	3M	7	2018	Year-1		1	Summer
7/12/2018	BL	4M	7	2018	Year-1		1	Summer
7/12/2018	BL	5M	7	2018	Year-1		1	Summer
7/12/2018	BL	6M	7	2018	Year-1	0	0	Summer
7/12/2018	BL	7M	7	2018	Year-1		1	Summer
7/12/2018	BL	10M	7	2018	Year-1	0	0	Summer
7/12/2018	BL	12M	7	2018	Year-1	0	0	Summer
7/12/2018	BL	14M	7	2018	Year-1	0	0	Summer
7/12/2018	BL	8A	7	2018	Year-1	0	0	Summer

Date	Species	Site	Month	Year	Survey Year	Presence	Data Gap	Season
7/12/2018	BL	9A	7	2018	Year-1	0	0	Summer
7/12/2018	BL	11A	7	2018	Year-1	0	0	Summer
7/12/2018	BL	13A	7	2018	Year-1	0	0	Summer
7/13/2018	BL	1M	7	2018	Year-1	0	0	Summer
7/13/2018	BL	2M	7	2018	Year-1		1	Summer
7/13/2018	BL	3M	7	2018	Year-1		1	Summer
7/13/2018	BL	4M	7	2018	Year-1		1	Summer
7/13/2018	BL	5M	7	2018	Year-1		1	Summer
7/13/2018	BL	6M	7	2018	Year-1	0	0	Summer
7/13/2018	BL	7M	7	2018	Year-1		1	Summer
7/13/2018	BL	10M	7	2018	Year-1	0	0	Summer
7/13/2018	BL	12M	7	2018	Year-1	0	0	Summer
7/13/2018	BL	14M	7	2018	Year-1	0	0	Summer
7/13/2018	BL	8A	7	2018	Year-1	0	0	Summer
7/13/2018	BL	9A	7	2018	Year-1	0	0	Summer
7/13/2018	BL	11A	7	2018	Year-1	0	0	Summer
7/13/2018	BL	13A	7	2018	Year-1	0	0	Summer
7/14/2018	BL	1M	7	2018	Year-1	0	0	Summer
7/14/2018	BL	2M	7	2018	Year-1		1	Summer
7/14/2018	BL	3M	7	2018	Year-1		1	Summer
7/14/2018	BL	4M	7	2018	Year-1		1	Summer
7/14/2018	BL	5M	7	2018	Year-1		1	Summer
7/14/2018	BL	6M	7	2018	Year-1	0	0	Summer
7/14/2018	BL	7M	7	2018	Year-1		1	Summer
7/14/2018	BL	10M	7	2018	Year-1	0	0	Summer
7/14/2018	BL	12M	7	2018	Year-1	0	0	Summer
7/14/2018	BL	14M	7	2018	Year-1	0	0	Summer
7/14/2018	BL	8A	7	2018	Year-1	0	0	Summer
7/14/2018	BL	9A	7	2018	Year-1	0	0	Summer
7/14/2018	BL	11A	7	2018	Year-1	0	0	Summer
7/14/2018	BL	13A	7	2018	Year-1	0	0	Summer
7/15/2018	BL	1M	7	2018	Year-1		1	Summer
7/15/2018	BL	2M	7	2018	Year-1		1	Summer
7/15/2018	BL	3M	7	2018	Year-1		1	Summer
7/15/2018	BL	4M	7	2018	Year-1		1	Summer
7/15/2018	BL	5M	7	2018	Year-1		1	Summer
7/15/2018	BL	6M	7	2018	Year-1		1	Summer
7/15/2018	BL	7M	7	2018	Year-1		1	Summer
7/15/2018	BL	10M	7	2018	Year-1		1	Summer
7/15/2018	BL	12M	7	2018	Year-1		1	Summer
7/15/2018	BL	14M	7	2018	Year-1		1	Summer
7/15/2018	BL	8A	7	2018	Year-1		1	Summer
7/15/2018	BL	9A	7	2018	Year-1		1	Summer
7/15/2018	BL	11A	7	2018	Year-1		1	Summer

Date	Species	Site	Month	Year	Survey Year	Presence	Data Gap	Season
7/15/2018	BL	13A	7	2018	Year-1		1	Summer
7/16/2018	BL	1M	7	2018	Year-1		1	Summer
7/16/2018	BL	2M	7	2018	Year-1		1	Summer
7/16/2018	BL	3M	7	2018	Year-1		1	Summer
7/16/2018	BL	4M	7	2018	Year-1		1	Summer
7/16/2018	BL	5M	7	2018	Year-1		1	Summer
7/16/2018	BL	6M	7	2018	Year-1		1	Summer
7/16/2018	BL	7M	7	2018	Year-1		1	Summer
7/16/2018	BL	10M	7	2018	Year-1		1	Summer
7/16/2018	BL	12M	7	2018	Year-1	0	0	Summer
7/16/2018	BL	14M	7	2018	Year-1		1	Summer
7/16/2018	BL	8A	7	2018	Year-1		1	Summer
7/16/2018	BL	9A	7	2018	Year-1	0	0	Summer
7/16/2018	BL	11A	7	2018	Year-1	0	0	Summer
7/16/2018	BL	13A	7	2018	Year-1	0	0	Summer
7/17/2018	BL	1M	7	2018	Year-1		1	Summer
7/17/2018	BL	2M	7	2018	Year-1		1	Summer
7/17/2018	BL	3M	7	2018	Year-1		1	Summer
7/17/2018	BL	4M	7	2018	Year-1		1	Summer
7/17/2018	BL	5M	7	2018	Year-1		1	Summer
7/17/2018	BL	6M	7	2018	Year-1		1	Summer
7/17/2018	BL	7M	7	2018	Year-1		1	Summer
7/17/2018	BL	10M	7	2018	Year-1		1	Summer
7/17/2018	BL	12M	7	2018	Year-1	0	0	Summer
7/17/2018	BL	14M	7	2018	Year-1		1	Summer
7/17/2018	BL	8A	7	2018	Year-1		1	Summer
7/17/2018	BL	9A	7	2018	Year-1	0	0	Summer
7/17/2018	BL	11A	7	2018	Year-1	0	0	Summer
7/17/2018	BL	13A	7	2018	Year-1	0	0	Summer
7/18/2018	BL	1M	7	2018	Year-1		1	Summer
7/18/2018	BL	2M	7	2018	Year-1		1	Summer
7/18/2018	BL	3M	7	2018	Year-1		1	Summer
7/18/2018	BL	4M	7	2018	Year-1		1	Summer
7/18/2018	BL	5M	7	2018	Year-1		1	Summer
7/18/2018	BL	6M	7	2018	Year-1		1	Summer
7/18/2018	BL	7M	7	2018	Year-1		1	Summer
7/18/2018	BL	10M	7	2018	Year-1		1	Summer
7/18/2018	BL	12M	7	2018	Year-1	0	0	Summer
7/18/2018	BL	14M	7	2018	Year-1		1	Summer
7/18/2018	BL	8A	7	2018	Year-1		1	Summer
7/18/2018	BL	9A	7	2018	Year-1	0	0	Summer
7/18/2018	BL	11A	7	2018	Year-1	0	0	Summer
7/18/2018	BL	13A	7	2018	Year-1	0	0	Summer
7/19/2018	BL	1M	7	2018	Year-1		1	Summer

Date	Species	Site	Month	Year	Survey Year	Presence	Data Gap	Season
7/19/2018	BL	2M	7	2018	Year-1		1	Summer
7/19/2018	BL	3M	7	2018	Year-1		1	Summer
7/19/2018	BL	4M	7	2018	Year-1		1	Summer
7/19/2018	BL	5M	7	2018	Year-1		1	Summer
7/19/2018	BL	6M	7	2018	Year-1		1	Summer
7/19/2018	BL	7M	7	2018	Year-1		1	Summer
7/19/2018	BL	10M	7	2018	Year-1		1	Summer
7/19/2018	BL	12M	7	2018	Year-1	0	0	Summer
7/19/2018	BL	14M	7	2018	Year-1		1	Summer
7/19/2018	BL	8A	7	2018	Year-1		1	Summer
7/19/2018	BL	9A	7	2018	Year-1	0	0	Summer
7/19/2018	BL	11A	7	2018	Year-1	0	0	Summer
7/19/2018	BL	13A	7	2018	Year-1	0	0	Summer
7/20/2018	BL	1M	7	2018	Year-1	0	0	Summer
7/20/2018	BL	2M	7	2018	Year-1		1	Summer
7/20/2018	BL	3M	7	2018	Year-1	0	0	Summer
7/20/2018	BL	4M	7	2018	Year-1	0	0	Summer
7/20/2018	BL	5M	7	2018	Year-1		1	Summer
7/20/2018	BL	6M	7	2018	Year-1	0	0	Summer
7/20/2018	BL	7M	7	2018	Year-1	0	0	Summer
7/20/2018	BL	10M	7	2018	Year-1		1	Summer
7/20/2018	BL	12M	7	2018	Year-1	0	0	Summer
7/20/2018	BL	14M	7	2018	Year-1	0	0	Summer
7/20/2018	BL	8A	7	2018	Year-1		1	Summer
7/20/2018	BL	9A	7	2018	Year-1	0	0	Summer
7/20/2018	BL	11A	7	2018	Year-1	0	0	Summer
7/20/2018	BL	13A	7	2018	Year-1	0	0	Summer
7/21/2018	BL	1M	7	2018	Year-1	0	0	Summer
7/21/2018	BL	2M	7	2018	Year-1		1	Summer
7/21/2018	BL	3M	7	2018	Year-1	0	0	Summer
7/21/2018	BL	4M	7	2018	Year-1	0	0	Summer
7/21/2018	BL	5M	7	2018	Year-1		1	Summer
7/21/2018	BL	6M	7	2018	Year-1	0	0	Summer
7/21/2018	BL	7M	7	2018	Year-1	0	0	Summer
7/21/2018	BL	10M	7	2018	Year-1		1	Summer
7/21/2018	BL	12M	7	2018	Year-1	0	0	Summer
7/21/2018	BL	14M	7	2018	Year-1	0	0	Summer
7/21/2018	BL	8A	7	2018	Year-1		1	Summer
7/21/2018	BL	9A	7	2018	Year-1	0	0	Summer
7/21/2018	BL	11A	7	2018	Year-1	0	0	Summer
7/21/2018	BL	13A	7	2018	Year-1	0	0	Summer
7/22/2018	BL	1M	7	2018	Year-1	0	0	Summer
7/22/2018	BL	2M	7	2018	Year-1		1	Summer
7/22/2018	BL	3M	7	2018	Year-1	0	0	Summer

Date	Species	Site	Month	Year	Survey Year	Presence	Data Gap	Season
7/22/2018	BL	4M	7	2018	Year-1	0	0	Summer
7/22/2018	BL	5M	7	2018	Year-1		1	Summer
7/22/2018	BL	6M	7	2018	Year-1	0	0	Summer
7/22/2018	BL	7M	7	2018	Year-1	0	0	Summer
7/22/2018	BL	10M	7	2018	Year-1		1	Summer
7/22/2018	BL	12M	7	2018	Year-1	0	0	Summer
7/22/2018	BL	14M	7	2018	Year-1	0	0	Summer
7/22/2018	BL	8A	7	2018	Year-1		1	Summer
7/22/2018	BL	9A	7	2018	Year-1	0	0	Summer
7/22/2018	BL	11A	7	2018	Year-1	0	0	Summer
7/22/2018	BL	13A	7	2018	Year-1	0	0	Summer
7/23/2018	BL	1M	7	2018	Year-1	0	0	Summer
7/23/2018	BL	2M	7	2018	Year-1		1	Summer
7/23/2018	BL	3M	7	2018	Year-1	0	0	Summer
7/23/2018	BL	4M	7	2018	Year-1	0	0	Summer
7/23/2018	BL	5M	7	2018	Year-1		1	Summer
7/23/2018	BL	6M	7	2018	Year-1	0	0	Summer
7/23/2018	BL	7M	7	2018	Year-1	0	0	Summer
7/23/2018	BL	10M	7	2018	Year-1		1	Summer
7/23/2018	BL	12M	7	2018	Year-1	0	0	Summer
7/23/2018	BL	14M	7	2018	Year-1	0	0	Summer
7/23/2018	BL	8A	7	2018	Year-1		1	Summer
7/23/2018	BL	9A	7	2018	Year-1	0	0	Summer
7/23/2018	BL	11A	7	2018	Year-1	0	0	Summer
7/23/2018	BL	13A	7	2018	Year-1	0	0	Summer
7/24/2018	BL	1M	7	2018	Year-1	0	0	Summer
7/24/2018	BL	2M	7	2018	Year-1		1	Summer
7/24/2018	BL	3M	7	2018	Year-1	0	0	Summer
7/24/2018	BL	4M	7	2018	Year-1	0	0	Summer
7/24/2018	BL	5M	7	2018	Year-1		1	Summer
7/24/2018	BL	6M	7	2018	Year-1	0	0	Summer
7/24/2018	BL	7M	7	2018	Year-1	0	0	Summer
7/24/2018	BL	10M	7	2018	Year-1		1	Summer
7/24/2018	BL	12M	7	2018	Year-1	0	0	Summer
7/24/2018	BL	14M	7	2018	Year-1	0	0	Summer
7/24/2018	BL	8A	7	2018	Year-1		1	Summer
7/24/2018	BL	9A	7	2018	Year-1	0	0	Summer
7/24/2018	BL	11A	7	2018	Year-1	0	0	Summer
7/24/2018	BL	13A	7	2018	Year-1	0	0	Summer
7/25/2018	BL	1M	7	2018	Year-1	0	0	Summer
7/25/2018	BL	2M	7	2018	Year-1		1	Summer
7/25/2018	BL	3M	7	2018	Year-1	0	0	Summer
7/25/2018	BL	4M	7	2018	Year-1	0	0	Summer
7/25/2018	BL	5M	7	2018	Year-1		1	Summer

Date	Species	Site	Month	Year	Survey Year	Presence	Data Gap	Season
7/25/2018	BL	6M	7	2018	Year-1	0	0	Summer
7/25/2018	BL	7M	7	2018	Year-1	0	0	Summer
7/25/2018	BL	10M	7	2018	Year-1		1	Summer
7/25/2018	BL	12M	7	2018	Year-1	0	0	Summer
7/25/2018	BL	14M	7	2018	Year-1	0	0	Summer
7/25/2018	BL	8A	7	2018	Year-1		1	Summer
7/25/2018	BL	9A	7	2018	Year-1	0	0	Summer
7/25/2018	BL	11A	7	2018	Year-1	0	0	Summer
7/25/2018	BL	13A	7	2018	Year-1	0	0	Summer
7/26/2018	BL	1M	7	2018	Year-1	0	0	Summer
7/26/2018	BL	2M	7	2018	Year-1		1	Summer
7/26/2018	BL	3M	7	2018	Year-1	0	0	Summer
7/26/2018	BL	4M	7	2018	Year-1	0	0	Summer
7/26/2018	BL	5M	7	2018	Year-1		1	Summer
7/26/2018	BL	6M	7	2018	Year-1	0	0	Summer
7/26/2018	BL	7M	7	2018	Year-1	0	0	Summer
7/26/2018	BL	10M	7	2018	Year-1		1	Summer
7/26/2018	BL	12M	7	2018	Year-1	0	0	Summer
7/26/2018	BL	14M	7	2018	Year-1	0	0	Summer
7/26/2018	BL	8A	7	2018	Year-1		1	Summer
7/26/2018	BL	9A	7	2018	Year-1	0	0	Summer
7/26/2018	BL	11A	7	2018	Year-1	0	0	Summer
7/26/2018	BL	13A	7	2018	Year-1	0	0	Summer
7/27/2018	BL	1M	7	2018	Year-1	0	0	Summer
7/27/2018	BL	2M	7	2018	Year-1		1	Summer
7/27/2018	BL	3M	7	2018	Year-1	0	0	Summer
7/27/2018	BL	4M	7	2018	Year-1	0	0	Summer
7/27/2018	BL	5M	7	2018	Year-1		1	Summer
7/27/2018	BL	6M	7	2018	Year-1	0	0	Summer
7/27/2018	BL	7M	7	2018	Year-1	0	0	Summer
7/27/2018	BL	10M	7	2018	Year-1		1	Summer
7/27/2018	BL	12M	7	2018	Year-1	0	0	Summer
7/27/2018	BL	14M	7	2018	Year-1	0	0	Summer
7/27/2018	BL	8A	7	2018	Year-1		1	Summer
7/27/2018	BL	9A	7	2018	Year-1	0	0	Summer
7/27/2018	BL	11A	7	2018	Year-1	0	0	Summer
7/27/2018	BL	13A	7	2018	Year-1	0	0	Summer
7/28/2018	BL	1M	7	2018	Year-1	0	0	Summer
7/28/2018	BL	2M	7	2018	Year-1		1	Summer
7/28/2018	BL	3M	7	2018	Year-1	0	0	Summer
7/28/2018	BL	4M	7	2018	Year-1	0	0	Summer
7/28/2018	BL	5M	7	2018	Year-1		1	Summer
7/28/2018	BL	6M	7	2018	Year-1	0	0	Summer
7/28/2018	BL	7M	7	2018	Year-1	0	0	Summer

Date	Species	Site	Month	Year	Survey Year	Presence	Data Gap	Season
7/28/2018	BL	10M	7	2018	Year-1		1	Summer
7/28/2018	BL	12M	7	2018	Year-1	0	0	Summer
7/28/2018	BL	14M	7	2018	Year-1	0	0	Summer
7/28/2018	BL	8A	7	2018	Year-1		1	Summer
7/28/2018	BL	9A	7	2018	Year-1	0	0	Summer
7/28/2018	BL	11A	7	2018	Year-1	0	0	Summer
7/28/2018	BL	13A	7	2018	Year-1	0	0	Summer
7/29/2018	BL	1M	7	2018	Year-1	0	0	Summer
7/29/2018	BL	2M	7	2018	Year-1		1	Summer
7/29/2018	BL	3M	7	2018	Year-1	0	0	Summer
7/29/2018	BL	4M	7	2018	Year-1	0	0	Summer
7/29/2018	BL	5M	7	2018	Year-1		1	Summer
7/29/2018	BL	6M	7	2018	Year-1	0	0	Summer
7/29/2018	BL	7M	7	2018	Year-1	0	0	Summer
7/29/2018	BL	10M	7	2018	Year-1		1	Summer
7/29/2018	BL	12M	7	2018	Year-1	0	0	Summer
7/29/2018	BL	14M	7	2018	Year-1	0	0	Summer
7/29/2018	BL	8A	7	2018	Year-1		1	Summer
7/29/2018	BL	9A	7	2018	Year-1	0	0	Summer
7/29/2018	BL	11A	7	2018	Year-1	0	0	Summer
7/29/2018	BL	13A	7	2018	Year-1	0	0	Summer
7/30/2018	BL	1M	7	2018	Year-1	0	0	Summer
7/30/2018	BL	2M	7	2018	Year-1		1	Summer
7/30/2018	BL	3M	7	2018	Year-1	0	0	Summer
7/30/2018	BL	4M	7	2018	Year-1	0	0	Summer
7/30/2018	BL	5M	7	2018	Year-1		1	Summer
7/30/2018	BL	6M	7	2018	Year-1	0	0	Summer
7/30/2018	BL	7M	7	2018	Year-1	0	0	Summer
7/30/2018	BL	10M	7	2018	Year-1		1	Summer
7/30/2018	BL	12M	7	2018	Year-1	0	0	Summer
7/30/2018	BL	14M	7	2018	Year-1	0	0	Summer
7/30/2018	BL	8A	7	2018	Year-1		1	Summer
7/30/2018	BL	9A	7	2018	Year-1	0	0	Summer
7/30/2018	BL	11A	7	2018	Year-1	0	0	Summer
7/30/2018	BL	13A	7	2018	Year-1	0	0	Summer
7/31/2018	BL	1M	7	2018	Year-1	0	0	Summer
7/31/2018	BL	2M	7	2018	Year-1		1	Summer
7/31/2018	BL	3M	7	2018	Year-1	0	0	Summer
7/31/2018	BL	4M	7	2018	Year-1	0	0	Summer
7/31/2018	BL	5M	7	2018	Year-1		1	Summer
7/31/2018	BL	6M	7	2018	Year-1	0	0	Summer
7/31/2018	BL	7M	7	2018	Year-1	0	0	Summer
7/31/2018	BL	10M	7	2018	Year-1		1	Summer
7/31/2018	BL	12M	7	2018	Year-1	0	0	Summer

Date	Species	Site	Month	Year	Survey Year	Presence	Data Gap	Season
7/31/2018	BL	14M	7	2018	Year-1	0	0	Summer
7/31/2018	BL	8A	7	2018	Year-1		1	Summer
7/31/2018	BL	9A	7	2018	Year-1	0	0	Summer
7/31/2018	BL	11A	7	2018	Year-1	0	0	Summer
7/31/2018	BL	13A	7	2018	Year-1	0	0	Summer
8/1/2018	BL	1M	8	2018	Year-1	0	0	Summer
8/1/2018	BL	2M	8	2018	Year-1		1	Summer
8/1/2018	BL	3M	8	2018	Year-1	0	0	Summer
8/1/2018	BL	4M	8	2018	Year-1	0	0	Summer
8/1/2018	BL	5M	8	2018	Year-1		1	Summer
8/1/2018	BL	6M	8	2018	Year-1	0	0	Summer
8/1/2018	BL	7M	8	2018	Year-1	0	0	Summer
8/1/2018	BL	10M	8	2018	Year-1		1	Summer
8/1/2018	BL	12M	8	2018	Year-1	0	0	Summer
8/1/2018	BL	14M	8	2018	Year-1	0	0	Summer
8/1/2018	BL	8A	8	2018	Year-1		1	Summer
8/1/2018	BL	9A	8	2018	Year-1	0	0	Summer
8/1/2018	BL	11A	8	2018	Year-1	0	0	Summer
8/1/2018	BL	13A	8	2018	Year-1	0	0	Summer
8/2/2018	BL	1M	8	2018	Year-1	0	0	Summer
8/2/2018	BL	2M	8	2018	Year-1		1	Summer
8/2/2018	BL	3M	8	2018	Year-1	0	0	Summer
8/2/2018	BL	4M	8	2018	Year-1	0	0	Summer
8/2/2018	BL	5M	8	2018	Year-1		1	Summer
8/2/2018	BL	6M	8	2018	Year-1	0	0	Summer
8/2/2018	BL	7M	8	2018	Year-1	0	0	Summer
8/2/2018	BL	10M	8	2018	Year-1		1	Summer
8/2/2018	BL	12M	8	2018	Year-1	0	0	Summer
8/2/2018	BL	14M	8	2018	Year-1	0	0	Summer
8/2/2018	BL	8A	8	2018	Year-1		1	Summer
8/2/2018	BL	9A	8	2018	Year-1	0	0	Summer
8/2/2018	BL	11A	8	2018	Year-1	0	0	Summer
8/2/2018	BL	13A	8	2018	Year-1	0	0	Summer
8/3/2018	BL	1M	8	2018	Year-1	0	0	Summer
8/3/2018	BL	2M	8	2018	Year-1		1	Summer
8/3/2018	BL	3M	8	2018	Year-1	0	0	Summer
8/3/2018	BL	4M	8	2018	Year-1	0	0	Summer
8/3/2018	BL	5M	8	2018	Year-1		1	Summer
8/3/2018	BL	6M	8	2018	Year-1	0	0	Summer
8/3/2018	BL	7M	8	2018	Year-1	0	0	Summer
8/3/2018	BL	10M	8	2018	Year-1		1	Summer
8/3/2018	BL	12M	8	2018	Year-1	0	0	Summer
8/3/2018	BL	14M	8	2018	Year-1	0	0	Summer
8/3/2018	BL	8A	8	2018	Year-1		1	Summer

Date	Species	Site	Month	Year	Survey Year	Presence	Data Gap	Season
8/3/2018	BL	9A	8	2018	Year-1	0	0	Summer
8/3/2018	BL	11A	8	2018	Year-1	0	0	Summer
8/3/2018	BL	13A	8	2018	Year-1	0	0	Summer
8/4/2018	BL	1M	8	2018	Year-1	0	0	Summer
8/4/2018	BL	2M	8	2018	Year-1		1	Summer
8/4/2018	BL	3M	8	2018	Year-1	0	0	Summer
8/4/2018	BL	4M	8	2018	Year-1	0	0	Summer
8/4/2018	BL	5M	8	2018	Year-1		1	Summer
8/4/2018	BL	6M	8	2018	Year-1	0	0	Summer
8/4/2018	BL	7M	8	2018	Year-1	0	0	Summer
8/4/2018	BL	10M	8	2018	Year-1		1	Summer
8/4/2018	BL	12M	8	2018	Year-1	0	0	Summer
8/4/2018	BL	14M	8	2018	Year-1	0	0	Summer
8/4/2018	BL	8A	8	2018	Year-1		1	Summer
8/4/2018	BL	9A	8	2018	Year-1	0	0	Summer
8/4/2018	BL	11A	8	2018	Year-1	0	0	Summer
8/4/2018	BL	13A	8	2018	Year-1	0	0	Summer
8/5/2018	BL	1M	8	2018	Year-1	0	0	Summer
8/5/2018	BL	2M	8	2018	Year-1		1	Summer
8/5/2018	BL	3M	8	2018	Year-1	0	0	Summer
8/5/2018	BL	4M	8	2018	Year-1	0	0	Summer
8/5/2018	BL	5M	8	2018	Year-1		1	Summer
8/5/2018	BL	6M	8	2018	Year-1	0	0	Summer
8/5/2018	BL	7M	8	2018	Year-1	0	0	Summer
8/5/2018	BL	10M	8	2018	Year-1		1	Summer
8/5/2018	BL	12M	8	2018	Year-1	0	0	Summer
8/5/2018	BL	14M	8	2018	Year-1	0	0	Summer
8/5/2018	BL	8A	8	2018	Year-1		1	Summer
8/5/2018	BL	9A	8	2018	Year-1	0	0	Summer
8/5/2018	BL	11A	8	2018	Year-1	0	0	Summer
8/5/2018	BL	13A	8	2018	Year-1	0	0	Summer
8/6/2018	BL	1M	8	2018	Year-1	0	0	Summer
8/6/2018	BL	2M	8	2018	Year-1		1	Summer
8/6/2018	BL	3M	8	2018	Year-1	0	0	Summer
8/6/2018	BL	4M	8	2018	Year-1	0	0	Summer
8/6/2018	BL	5M	8	2018	Year-1		1	Summer
8/6/2018	BL	6M	8	2018	Year-1	0	0	Summer
8/6/2018	BL	7M	8	2018	Year-1	0	0	Summer
8/6/2018	BL	10M	8	2018	Year-1		1	Summer
8/6/2018	BL	12M	8	2018	Year-1	0	0	Summer
8/6/2018	BL	14M	8	2018	Year-1	0	0	Summer
8/6/2018	BL	8A	8	2018	Year-1		1	Summer
8/6/2018	BL	9A	8	2018	Year-1	0	0	Summer
8/6/2018	BL	11A	8	2018	Year-1	0	0	Summer

Date	Species	Site	Month	Year	Survey Year	Presence	Data Gap	Season
8/6/2018	BL	13A	8	2018	Year-1	0	0	Summer
8/7/2018	BL	1M	8	2018	Year-1	0	0	Summer
8/7/2018	BL	2M	8	2018	Year-1		1	Summer
8/7/2018	BL	3M	8	2018	Year-1	0	0	Summer
8/7/2018	BL	4M	8	2018	Year-1	0	0	Summer
8/7/2018	BL	5M	8	2018	Year-1		1	Summer
8/7/2018	BL	6M	8	2018	Year-1	0	0	Summer
8/7/2018	BL	7M	8	2018	Year-1	0	0	Summer
8/7/2018	BL	10M	8	2018	Year-1		1	Summer
8/7/2018	BL	12M	8	2018	Year-1	0	0	Summer
8/7/2018	BL	14M	8	2018	Year-1	0	0	Summer
8/7/2018	BL	8A	8	2018	Year-1		1	Summer
8/7/2018	BL	9A	8	2018	Year-1	0	0	Summer
8/7/2018	BL	11A	8	2018	Year-1	0	0	Summer
8/7/2018	BL	13A	8	2018	Year-1	0	0	Summer
8/8/2018	BL	1M	8	2018	Year-1	0	0	Summer
8/8/2018	BL	2M	8	2018	Year-1		1	Summer
8/8/2018	BL	3M	8	2018	Year-1	0	0	Summer
8/8/2018	BL	4M	8	2018	Year-1	0	0	Summer
8/8/2018	BL	5M	8	2018	Year-1		1	Summer
8/8/2018	BL	6M	8	2018	Year-1	0	0	Summer
8/8/2018	BL	7M	8	2018	Year-1	0	0	Summer
8/8/2018	BL	10M	8	2018	Year-1		1	Summer
8/8/2018	BL	12M	8	2018	Year-1		1	Summer
8/8/2018	BL	14M	8	2018	Year-1	0	0	Summer
8/8/2018	BL	8A	8	2018	Year-1		1	Summer
8/8/2018	BL	9A	8	2018	Year-1	0	0	Summer
8/8/2018	BL	11A	8	2018	Year-1	0	0	Summer
8/8/2018	BL	13A	8	2018	Year-1	0	0	Summer
8/9/2018	BL	1M	8	2018	Year-1	0	0	Summer
8/9/2018	BL	2M	8	2018	Year-1		1	Summer
8/9/2018	BL	3M	8	2018	Year-1	0	0	Summer
8/9/2018	BL	4M	8	2018	Year-1	0	0	Summer
8/9/2018	BL	5M	8	2018	Year-1		1	Summer
8/9/2018	BL	6M	8	2018	Year-1	0	0	Summer
8/9/2018	BL	7M	8	2018	Year-1	0	0	Summer
8/9/2018	BL	10M	8	2018	Year-1		1	Summer
8/9/2018	BL	12M	8	2018	Year-1		1	Summer
8/9/2018	BL	14M	8	2018	Year-1	0	0	Summer
8/9/2018	BL	8A	8	2018	Year-1		1	Summer
8/9/2018	BL	9A	8	2018	Year-1	0	0	Summer
8/9/2018	BL	11A	8	2018	Year-1	0	0	Summer
8/9/2018	BL	13A	8	2018	Year-1	0	0	Summer
8/10/2018	BL	1M	8	2018	Year-1	0	0	Summer

Date	Species	Site	Month	Year	Survey Year	Presence	Data Gap	Season
8/10/2018	BL	2M	8	2018	Year-1		1	Summer
8/10/2018	BL	3M	8	2018	Year-1	0	0	Summer
8/10/2018	BL	4M	8	2018	Year-1	0	0	Summer
8/10/2018	BL	5M	8	2018	Year-1		1	Summer
8/10/2018	BL	6M	8	2018	Year-1	0	0	Summer
8/10/2018	BL	7M	8	2018	Year-1	0	0	Summer
8/10/2018	BL	10M	8	2018	Year-1		1	Summer
8/10/2018	BL	12M	8	2018	Year-1		1	Summer
8/10/2018	BL	14M	8	2018	Year-1	0	0	Summer
8/10/2018	BL	8A	8	2018	Year-1		1	Summer
8/10/2018	BL	9A	8	2018	Year-1	0	0	Summer
8/10/2018	BL	11A	8	2018	Year-1	0	0	Summer
8/10/2018	BL	13A	8	2018	Year-1	0	0	Summer
8/11/2018	BL	1M	8	2018	Year-1	0	0	Summer
8/11/2018	BL	2M	8	2018	Year-1		1	Summer
8/11/2018	BL	3M	8	2018	Year-1	0	0	Summer
8/11/2018	BL	4M	8	2018	Year-1	0	0	Summer
8/11/2018	BL	5M	8	2018	Year-1		1	Summer
8/11/2018	BL	6M	8	2018	Year-1	0	0	Summer
8/11/2018	BL	7M	8	2018	Year-1	0	0	Summer
8/11/2018	BL	10M	8	2018	Year-1		1	Summer
8/11/2018	BL	12M	8	2018	Year-1		1	Summer
8/11/2018	BL	14M	8	2018	Year-1	0	0	Summer
8/11/2018	BL	8A	8	2018	Year-1		1	Summer
8/11/2018	BL	9A	8	2018	Year-1	0	0	Summer
8/11/2018	BL	11A	8	2018	Year-1	0	0	Summer
8/11/2018	BL	13A	8	2018	Year-1	0	0	Summer
8/12/2018	BL	1M	8	2018	Year-1	0	0	Summer
8/12/2018	BL	2M	8	2018	Year-1		1	Summer
8/12/2018	BL	3M	8	2018	Year-1	0	0	Summer
8/12/2018	BL	4M	8	2018	Year-1	0	0	Summer
8/12/2018	BL	5M	8	2018	Year-1		1	Summer
8/12/2018	BL	6M	8	2018	Year-1	0	0	Summer
8/12/2018	BL	7M	8	2018	Year-1	0	0	Summer
8/12/2018	BL	10M	8	2018	Year-1		1	Summer
8/12/2018	BL	12M	8	2018	Year-1		1	Summer
8/12/2018	BL	14M	8	2018	Year-1	0	0	Summer
8/12/2018	BL	8A	8	2018	Year-1		1	Summer
8/12/2018	BL	9A	8	2018	Year-1	0	0	Summer
8/12/2018	BL	11A	8	2018	Year-1	0	0	Summer
8/12/2018	BL	13A	8	2018	Year-1	0	0	Summer
8/13/2018	BL	1M	8	2018	Year-1	0	0	Summer
8/13/2018	BL	2M	8	2018	Year-1		1	Summer
8/13/2018	BL	3M	8	2018	Year-1	0	0	Summer

Date	Species	Site	Month	Year	Survey Year	Presence	Data Gap	Season
8/13/2018	BL	4M	8	2018	Year-1	0	0	Summer
8/13/2018	BL	5M	8	2018	Year-1		1	Summer
8/13/2018	BL	6M	8	2018	Year-1	0	0	Summer
8/13/2018	BL	7M	8	2018	Year-1	0	0	Summer
8/13/2018	BL	10M	8	2018	Year-1		1	Summer
8/13/2018	BL	12M	8	2018	Year-1		1	Summer
8/13/2018	BL	14M	8	2018	Year-1	0	0	Summer
8/13/2018	BL	8A	8	2018	Year-1		1	Summer
8/13/2018	BL	9A	8	2018	Year-1	0	0	Summer
8/13/2018	BL	11A	8	2018	Year-1	0	0	Summer
8/13/2018	BL	13A	8	2018	Year-1	0	0	Summer
8/14/2018	BL	1M	8	2018	Year-1	0	0	Summer
8/14/2018	BL	2M	8	2018	Year-1		1	Summer
8/14/2018	BL	3M	8	2018	Year-1	0	0	Summer
8/14/2018	BL	4M	8	2018	Year-1	0	0	Summer
8/14/2018	BL	5M	8	2018	Year-1		1	Summer
8/14/2018	BL	6M	8	2018	Year-1	0	0	Summer
8/14/2018	BL	7M	8	2018	Year-1	0	0	Summer
8/14/2018	BL	10M	8	2018	Year-1		1	Summer
8/14/2018	BL	12M	8	2018	Year-1		1	Summer
8/14/2018	BL	14M	8	2018	Year-1	0	0	Summer
8/14/2018	BL	8A	8	2018	Year-1		1	Summer
8/14/2018	BL	9A	8	2018	Year-1	0	0	Summer
8/14/2018	BL	11A	8	2018	Year-1	0	0	Summer
8/14/2018	BL	13A	8	2018	Year-1	0	0	Summer
8/15/2018	BL	1M	8	2018	Year-1	0	0	Summer
8/15/2018	BL	2M	8	2018	Year-1		1	Summer
8/15/2018	BL	3M	8	2018	Year-1	0	0	Summer
8/15/2018	BL	4M	8	2018	Year-1	0	0	Summer
8/15/2018	BL	5M	8	2018	Year-1		1	Summer
8/15/2018	BL	6M	8	2018	Year-1	0	0	Summer
8/15/2018	BL	7M	8	2018	Year-1	0	0	Summer
8/15/2018	BL	10M	8	2018	Year-1		1	Summer
8/15/2018	BL	12M	8	2018	Year-1		1	Summer
8/15/2018	BL	14M	8	2018	Year-1	0	0	Summer
8/15/2018	BL	8A	8	2018	Year-1		1	Summer
8/15/2018	BL	9A	8	2018	Year-1	0	0	Summer
8/15/2018	BL	11A	8	2018	Year-1	0	0	Summer
8/15/2018	BL	13A	8	2018	Year-1	0	0	Summer
8/16/2018	BL	1M	8	2018	Year-1	0	0	Summer
8/16/2018	BL	2M	8	2018	Year-1		1	Summer
8/16/2018	BL	3M	8	2018	Year-1	0	0	Summer
8/16/2018	BL	4M	8	2018	Year-1	0	0	Summer
8/16/2018	BL	5M	8	2018	Year-1		1	Summer

Date	Species	Site	Month	Year	Survey Year	Presence	Data Gap	Season
8/16/2018	BL	6M	8	2018	Year-1	0	0	Summer
8/16/2018	BL	7M	8	2018	Year-1	0	0	Summer
8/16/2018	BL	10M	8	2018	Year-1		1	Summer
8/16/2018	BL	12M	8	2018	Year-1		1	Summer
8/16/2018	BL	14M	8	2018	Year-1	0	0	Summer
8/16/2018	BL	8A	8	2018	Year-1		1	Summer
8/16/2018	BL	9A	8	2018	Year-1	0	0	Summer
8/16/2018	BL	11A	8	2018	Year-1	0	0	Summer
8/16/2018	BL	13A	8	2018	Year-1	0	0	Summer
8/17/2018	BL	1M	8	2018	Year-1	0	0	Summer
8/17/2018	BL	2M	8	2018	Year-1		1	Summer
8/17/2018	BL	3M	8	2018	Year-1	0	0	Summer
8/17/2018	BL	4M	8	2018	Year-1	0	0	Summer
8/17/2018	BL	5M	8	2018	Year-1		1	Summer
8/17/2018	BL	6M	8	2018	Year-1	0	0	Summer
8/17/2018	BL	7M	8	2018	Year-1	0	0	Summer
8/17/2018	BL	10M	8	2018	Year-1		1	Summer
8/17/2018	BL	12M	8	2018	Year-1		1	Summer
8/17/2018	BL	14M	8	2018	Year-1	0	0	Summer
8/17/2018	BL	8A	8	2018	Year-1		1	Summer
8/17/2018	BL	9A	8	2018	Year-1	0	0	Summer
8/17/2018	BL	11A	8	2018	Year-1	0	0	Summer
8/17/2018	BL	13A	8	2018	Year-1	0	0	Summer
8/18/2018	BL	1M	8	2018	Year-1	0	0	Summer
8/18/2018	BL	2M	8	2018	Year-1		1	Summer
8/18/2018	BL	3M	8	2018	Year-1	0	0	Summer
8/18/2018	BL	4M	8	2018	Year-1	0	0	Summer
8/18/2018	BL	5M	8	2018	Year-1		1	Summer
8/18/2018	BL	6M	8	2018	Year-1	0	0	Summer
8/18/2018	BL	7M	8	2018	Year-1	0	0	Summer
8/18/2018	BL	10M	8	2018	Year-1		1	Summer
8/18/2018	BL	12M	8	2018	Year-1		1	Summer
8/18/2018	BL	14M	8	2018	Year-1	0	0	Summer
8/18/2018	BL	8A	8	2018	Year-1		1	Summer
8/18/2018	BL	9A	8	2018	Year-1	0	0	Summer
8/18/2018	BL	11A	8	2018	Year-1	0	0	Summer
8/18/2018	BL	13A	8	2018	Year-1	0	0	Summer
8/19/2018	BL	1M	8	2018	Year-1	0	0	Summer
8/19/2018	BL	2M	8	2018	Year-1		1	Summer
8/19/2018	BL	3M	8	2018	Year-1	0	0	Summer
8/19/2018	BL	4M	8	2018	Year-1	0	0	Summer
8/19/2018	BL	5M	8	2018	Year-1		1	Summer
8/19/2018	BL	6M	8	2018	Year-1	0	0	Summer
8/19/2018	BL	7M	8	2018	Year-1	0	0	Summer

Date	Species	Site	Month	Year	Survey Year	Presence	Data Gap	Season
8/19/2018	BL	10M	8	2018	Year-1		1	Summer
8/19/2018	BL	12M	8	2018	Year-1		1	Summer
8/19/2018	BL	14M	8	2018	Year-1	0	0	Summer
8/19/2018	BL	8A	8	2018	Year-1		1	Summer
8/19/2018	BL	9A	8	2018	Year-1	0	0	Summer
8/19/2018	BL	11A	8	2018	Year-1	0	0	Summer
8/19/2018	BL	13A	8	2018	Year-1	0	0	Summer
8/20/2018	BL	1M	8	2018	Year-1	0	0	Summer
8/20/2018	BL	2M	8	2018	Year-1		1	Summer
8/20/2018	BL	3M	8	2018	Year-1	0	0	Summer
8/20/2018	BL	4M	8	2018	Year-1	0	0	Summer
8/20/2018	BL	5M	8	2018	Year-1		1	Summer
8/20/2018	BL	6M	8	2018	Year-1	0	0	Summer
8/20/2018	BL	7M	8	2018	Year-1	0	0	Summer
8/20/2018	BL	10M	8	2018	Year-1		1	Summer
8/20/2018	BL	12M	8	2018	Year-1		1	Summer
8/20/2018	BL	14M	8	2018	Year-1	0	0	Summer
8/20/2018	BL	8A	8	2018	Year-1		1	Summer
8/20/2018	BL	9A	8	2018	Year-1	0	0	Summer
8/20/2018	BL	11A	8	2018	Year-1	0	0	Summer
8/20/2018	BL	13A	8	2018	Year-1	0	0	Summer
8/21/2018	BL	1M	8	2018	Year-1	0	0	Summer
8/21/2018	BL	2M	8	2018	Year-1		1	Summer
8/21/2018	BL	3M	8	2018	Year-1	0	0	Summer
8/21/2018	BL	4M	8	2018	Year-1	0	0	Summer
8/21/2018	BL	5M	8	2018	Year-1		1	Summer
8/21/2018	BL	6M	8	2018	Year-1	0	0	Summer
8/21/2018	BL	7M	8	2018	Year-1	0	0	Summer
8/21/2018	BL	10M	8	2018	Year-1		1	Summer
8/21/2018	BL	12M	8	2018	Year-1		1	Summer
8/21/2018	BL	14M	8	2018	Year-1	0	0	Summer
8/21/2018	BL	8A	8	2018	Year-1		1	Summer
8/21/2018	BL	9A	8	2018	Year-1	0	0	Summer
8/21/2018	BL	11A	8	2018	Year-1	0	0	Summer
8/21/2018	BL	13A	8	2018	Year-1	0	0	Summer
8/22/2018	BL	1M	8	2018	Year-1	0	0	Summer
8/22/2018	BL	2M	8	2018	Year-1		1	Summer
8/22/2018	BL	3M	8	2018	Year-1	0	0	Summer
8/22/2018	BL	4M	8	2018	Year-1	0	0	Summer
8/22/2018	BL	5M	8	2018	Year-1		1	Summer
8/22/2018	BL	6M	8	2018	Year-1	0	0	Summer
8/22/2018	BL	7M	8	2018	Year-1	0	0	Summer
8/22/2018	BL	10M	8	2018	Year-1		1	Summer
8/22/2018	BL	12M	8	2018	Year-1		1	Summer

Date	Species	Site	Month	Year	Survey Year	Presence	Data Gap	Season
8/22/2018	BL	14M	8	2018	Year-1	0	0	Summer
8/22/2018	BL	8A	8	2018	Year-1		1	Summer
8/22/2018	BL	9A	8	2018	Year-1	0	0	Summer
8/22/2018	BL	11A	8	2018	Year-1	0	0	Summer
8/22/2018	BL	13A	8	2018	Year-1	0	0	Summer
8/23/2018	BL	1M	8	2018	Year-1	0	0	Summer
8/23/2018	BL	2M	8	2018	Year-1		1	Summer
8/23/2018	BL	3M	8	2018	Year-1	0	0	Summer
8/23/2018	BL	4M	8	2018	Year-1	0	0	Summer
8/23/2018	BL	5M	8	2018	Year-1		1	Summer
8/23/2018	BL	6M	8	2018	Year-1	0	0	Summer
8/23/2018	BL	7M	8	2018	Year-1	0	0	Summer
8/23/2018	BL	10M	8	2018	Year-1		1	Summer
8/23/2018	BL	12M	8	2018	Year-1		1	Summer
8/23/2018	BL	14M	8	2018	Year-1	0	0	Summer
8/23/2018	BL	8A	8	2018	Year-1		1	Summer
8/23/2018	BL	9A	8	2018	Year-1	0	0	Summer
8/23/2018	BL	11A	8	2018	Year-1	0	0	Summer
8/23/2018	BL	13A	8	2018	Year-1	0	0	Summer
8/24/2018	BL	1M	8	2018	Year-1	0	0	Summer
8/24/2018	BL	2M	8	2018	Year-1		1	Summer
8/24/2018	BL	3M	8	2018	Year-1	0	0	Summer
8/24/2018	BL	4M	8	2018	Year-1	0	0	Summer
8/24/2018	BL	5M	8	2018	Year-1		1	Summer
8/24/2018	BL	6M	8	2018	Year-1	0	0	Summer
8/24/2018	BL	7M	8	2018	Year-1	0	0	Summer
8/24/2018	BL	10M	8	2018	Year-1		1	Summer
8/24/2018	BL	12M	8	2018	Year-1		1	Summer
8/24/2018	BL	14M	8	2018	Year-1	0	0	Summer
8/24/2018	BL	8A	8	2018	Year-1		1	Summer
8/24/2018	BL	9A	8	2018	Year-1	0	0	Summer
8/24/2018	BL	11A	8	2018	Year-1	0	0	Summer
8/24/2018	BL	13A	8	2018	Year-1	0	0	Summer
8/25/2018	BL	1M	8	2018	Year-1	0	0	Summer
8/25/2018	BL	2M	8	2018	Year-1		1	Summer
8/25/2018	BL	3M	8	2018	Year-1	0	0	Summer
8/25/2018	BL	4M	8	2018	Year-1	0	0	Summer
8/25/2018	BL	5M	8	2018	Year-1		1	Summer
8/25/2018	BL	6M	8	2018	Year-1	0	0	Summer
8/25/2018	BL	7M	8	2018	Year-1	0	0	Summer
8/25/2018	BL	10M	8	2018	Year-1		1	Summer
8/25/2018	BL	12M	8	2018	Year-1		1	Summer
8/25/2018	BL	14M	8	2018	Year-1	0	0	Summer
8/25/2018	BL	8A	8	2018	Year-1		1	Summer

Date	Species	Site	Month	Year	Survey Year	Presence	Data Gap	Season
8/25/2018	BL	9A	8	2018	Year-1	0	0	Summer
8/25/2018	BL	11A	8	2018	Year-1	0	0	Summer
8/25/2018	BL	13A	8	2018	Year-1	0	0	Summer
8/26/2018	BL	1M	8	2018	Year-1	0	0	Summer
8/26/2018	BL	2M	8	2018	Year-1		1	Summer
8/26/2018	BL	3M	8	2018	Year-1	0	0	Summer
8/26/2018	BL	4M	8	2018	Year-1	0	0	Summer
8/26/2018	BL	5M	8	2018	Year-1		1	Summer
8/26/2018	BL	6M	8	2018	Year-1	0	0	Summer
8/26/2018	BL	7M	8	2018	Year-1	0	0	Summer
8/26/2018	BL	10M	8	2018	Year-1		1	Summer
8/26/2018	BL	12M	8	2018	Year-1		1	Summer
8/26/2018	BL	14M	8	2018	Year-1	0	0	Summer
8/26/2018	BL	8A	8	2018	Year-1		1	Summer
8/26/2018	BL	9A	8	2018	Year-1	0	0	Summer
8/26/2018	BL	11A	8	2018	Year-1	0	0	Summer
8/26/2018	BL	13A	8	2018	Year-1	0	0	Summer
8/27/2018	BL	1M	8	2018	Year-1	0	0	Summer
8/27/2018	BL	2M	8	2018	Year-1		1	Summer
8/27/2018	BL	3M	8	2018	Year-1	0	0	Summer
8/27/2018	BL	4M	8	2018	Year-1	0	0	Summer
8/27/2018	BL	5M	8	2018	Year-1		1	Summer
8/27/2018	BL	6M	8	2018	Year-1	0	0	Summer
8/27/2018	BL	7M	8	2018	Year-1	0	0	Summer
8/27/2018	BL	10M	8	2018	Year-1		1	Summer
8/27/2018	BL	12M	8	2018	Year-1		1	Summer
8/27/2018	BL	14M	8	2018	Year-1	0	0	Summer
8/27/2018	BL	8A	8	2018	Year-1		1	Summer
8/27/2018	BL	9A	8	2018	Year-1	0	0	Summer
8/27/2018	BL	11A	8	2018	Year-1	0	0	Summer
8/27/2018	BL	13A	8	2018	Year-1	0	0	Summer
8/28/2018	BL	1M	8	2018	Year-1	0	0	Summer
8/28/2018	BL	2M	8	2018	Year-1		1	Summer
8/28/2018	BL	3M	8	2018	Year-1	0	0	Summer
8/28/2018	BL	4M	8	2018	Year-1	0	0	Summer
8/28/2018	BL	5M	8	2018	Year-1		1	Summer
8/28/2018	BL	6M	8	2018	Year-1	0	0	Summer
8/28/2018	BL	7M	8	2018	Year-1	0	0	Summer
8/28/2018	BL	10M	8	2018	Year-1		1	Summer
8/28/2018	BL	12M	8	2018	Year-1		1	Summer
8/28/2018	BL	14M	8	2018	Year-1	0	0	Summer
8/28/2018	BL	8A	8	2018	Year-1		1	Summer
8/28/2018	BL	9A	8	2018	Year-1	0	0	Summer
8/28/2018	BL	11A	8	2018	Year-1	0	0	Summer

Date	Species	Site	Month	Year	Survey Year	Presence	Data Gap	Season
8/28/2018	BL	13A	8	2018	Year-1	0	0	Summer
8/29/2018	BL	1M	8	2018	Year-1	0	0	Summer
8/29/2018	BL	2M	8	2018	Year-1		1	Summer
8/29/2018	BL	3M	8	2018	Year-1	0	0	Summer
8/29/2018	BL	4M	8	2018	Year-1	0	0	Summer
8/29/2018	BL	5M	8	2018	Year-1		1	Summer
8/29/2018	BL	6M	8	2018	Year-1	0	0	Summer
8/29/2018	BL	7M	8	2018	Year-1	0	0	Summer
8/29/2018	BL	10M	8	2018	Year-1		1	Summer
8/29/2018	BL	12M	8	2018	Year-1		1	Summer
8/29/2018	BL	14M	8	2018	Year-1	0	0	Summer
8/29/2018	BL	8A	8	2018	Year-1		1	Summer
8/29/2018	BL	9A	8	2018	Year-1	0	0	Summer
8/29/2018	BL	11A	8	2018	Year-1	0	0	Summer
8/29/2018	BL	13A	8	2018	Year-1	0	0	Summer
8/30/2018	BL	1M	8	2018	Year-1	0	0	Summer
8/30/2018	BL	2M	8	2018	Year-1		1	Summer
8/30/2018	BL	3M	8	2018	Year-1	0	0	Summer
8/30/2018	BL	4M	8	2018	Year-1	0	0	Summer
8/30/2018	BL	5M	8	2018	Year-1		1	Summer
8/30/2018	BL	6M	8	2018	Year-1	0	0	Summer
8/30/2018	BL	7M	8	2018	Year-1	0	0	Summer
8/30/2018	BL	10M	8	2018	Year-1		1	Summer
8/30/2018	BL	12M	8	2018	Year-1		1	Summer
8/30/2018	BL	14M	8	2018	Year-1	0	0	Summer
8/30/2018	BL	8A	8	2018	Year-1		1	Summer
8/30/2018	BL	9A	8	2018	Year-1	0	0	Summer
8/30/2018	BL	11A	8	2018	Year-1	0	0	Summer
8/30/2018	BL	13A	8	2018	Year-1	0	0	Summer
8/31/2018	BL	1M	8	2018	Year-1	0	0	Summer
8/31/2018	BL	2M	8	2018	Year-1		1	Summer
8/31/2018	BL	3M	8	2018	Year-1	0	0	Summer
8/31/2018	BL	4M	8	2018	Year-1	0	0	Summer
8/31/2018	BL	5M	8	2018	Year-1		1	Summer
8/31/2018	BL	6M	8	2018	Year-1	0	0	Summer
8/31/2018	BL	7M	8	2018	Year-1	0	0	Summer
8/31/2018	BL	10M	8	2018	Year-1		1	Summer
8/31/2018	BL	12M	8	2018	Year-1		1	Summer
8/31/2018	BL	14M	8	2018	Year-1	0	0	Summer
8/31/2018	BL	8A	8	2018	Year-1		1	Summer
8/31/2018	BL	9A	8	2018	Year-1	0	0	Summer
8/31/2018	BL	11A	8	2018	Year-1	0	0	Summer
8/31/2018	BL	13A	8	2018	Year-1	0	0	Summer
9/1/2018	BL	1M	9	2018	Year-1	0	0	Fall

Date	Species	Site	Month	Year	Survey Year	Presence	Data Gap	Season
9/1/2018	BL	2M	9	2018	Year-1		1	Fall
9/1/2018	BL	3M	9	2018	Year-1	0	0	Fall
9/1/2018	BL	4M	9	2018	Year-1	0	0	Fall
9/1/2018	BL	5M	9	2018	Year-1		1	Fall
9/1/2018	BL	6M	9	2018	Year-1	0	0	Fall
9/1/2018	BL	7M	9	2018	Year-1	0	0	Fall
9/1/2018	BL	10M	9	2018	Year-1		1	Fall
9/1/2018	BL	12M	9	2018	Year-1		1	Fall
9/1/2018	BL	14M	9	2018	Year-1	0	0	Fall
9/1/2018	BL	8A	9	2018	Year-1		1	Fall
9/1/2018	BL	9A	9	2018	Year-1	0	0	Fall
9/1/2018	BL	11A	9	2018	Year-1	0	0	Fall
9/1/2018	BL	13A	9	2018	Year-1	0	0	Fall
9/2/2018	BL	1M	9	2018	Year-1	0	0	Fall
9/2/2018	BL	2M	9	2018	Year-1		1	Fall
9/2/2018	BL	3M	9	2018	Year-1	0	0	Fall
9/2/2018	BL	4M	9	2018	Year-1	0	0	Fall
9/2/2018	BL	5M	9	2018	Year-1		1	Fall
9/2/2018	BL	6M	9	2018	Year-1	0	0	Fall
9/2/2018	BL	7M	9	2018	Year-1	0	0	Fall
9/2/2018	BL	10M	9	2018	Year-1		1	Fall
9/2/2018	BL	12M	9	2018	Year-1		1	Fall
9/2/2018	BL	14M	9	2018	Year-1	0	0	Fall
9/2/2018	BL	8A	9	2018	Year-1		1	Fall
9/2/2018	BL	9A	9	2018	Year-1	0	0	Fall
9/2/2018	BL	11A	9	2018	Year-1	0	0	Fall
9/2/2018	BL	13A	9	2018	Year-1	0	0	Fall
9/3/2018	BL	1M	9	2018	Year-1	0	0	Fall
9/3/2018	BL	2M	9	2018	Year-1		1	Fall
9/3/2018	BL	3M	9	2018	Year-1	0	0	Fall
9/3/2018	BL	4M	9	2018	Year-1	0	0	Fall
9/3/2018	BL	5M	9	2018	Year-1		1	Fall
9/3/2018	BL	6M	9	2018	Year-1	0	0	Fall
9/3/2018	BL	7M	9	2018	Year-1	0	0	Fall
9/3/2018	BL	10M	9	2018	Year-1		1	Fall
9/3/2018	BL	12M	9	2018	Year-1		1	Fall
9/3/2018	BL	14M	9	2018	Year-1	0	0	Fall
9/3/2018	BL	8A	9	2018	Year-1		1	Fall
9/3/2018	BL	9A	9	2018	Year-1	0	0	Fall
9/3/2018	BL	11A	9	2018	Year-1	0	0	Fall
9/3/2018	BL	13A	9	2018	Year-1	0	0	Fall
9/4/2018	BL	1M	9	2018	Year-1	0	0	Fall
9/4/2018	BL	2M	9	2018	Year-1		1	Fall
9/4/2018	BL	3M	9	2018	Year-1	0	0	Fall

Date	Species	Site	Month	Year	Survey Year	Presence	Data Gap	Season
9/4/2018	BL	4M	9	2018	Year-1	0	0	Fall
9/4/2018	BL	5M	9	2018	Year-1		1	Fall
9/4/2018	BL	6M	9	2018	Year-1	0	0	Fall
9/4/2018	BL	7M	9	2018	Year-1	0	0	Fall
9/4/2018	BL	10M	9	2018	Year-1		1	Fall
9/4/2018	BL	12M	9	2018	Year-1		1	Fall
9/4/2018	BL	14M	9	2018	Year-1	0	0	Fall
9/4/2018	BL	8A	9	2018	Year-1		1	Fall
9/4/2018	BL	9A	9	2018	Year-1	0	0	Fall
9/4/2018	BL	11A	9	2018	Year-1	0	0	Fall
9/4/2018	BL	13A	9	2018	Year-1	0	0	Fall
9/5/2018	BL	1M	9	2018	Year-1	0	0	Fall
9/5/2018	BL	2M	9	2018	Year-1		1	Fall
9/5/2018	BL	3M	9	2018	Year-1	0	0	Fall
9/5/2018	BL	4M	9	2018	Year-1	0	0	Fall
9/5/2018	BL	5M	9	2018	Year-1		1	Fall
9/5/2018	BL	6M	9	2018	Year-1	0	0	Fall
9/5/2018	BL	7M	9	2018	Year-1	0	0	Fall
9/5/2018	BL	10M	9	2018	Year-1		1	Fall
9/5/2018	BL	12M	9	2018	Year-1		1	Fall
9/5/2018	BL	14M	9	2018	Year-1	0	0	Fall
9/5/2018	BL	8A	9	2018	Year-1		1	Fall
9/5/2018	BL	9A	9	2018	Year-1	0	0	Fall
9/5/2018	BL	11A	9	2018	Year-1	0	0	Fall
9/5/2018	BL	13A	9	2018	Year-1	0	0	Fall
9/6/2018	BL	1M	9	2018	Year-1	0	0	Fall
9/6/2018	BL	2M	9	2018	Year-1		1	Fall
9/6/2018	BL	3M	9	2018	Year-1	0	0	Fall
9/6/2018	BL	4M	9	2018	Year-1	0	0	Fall
9/6/2018	BL	5M	9	2018	Year-1		1	Fall
9/6/2018	BL	6M	9	2018	Year-1	0	0	Fall
9/6/2018	BL	7M	9	2018	Year-1	0	0	Fall
9/6/2018	BL	10M	9	2018	Year-1		1	Fall
9/6/2018	BL	12M	9	2018	Year-1		1	Fall
9/6/2018	BL	14M	9	2018	Year-1	0	0	Fall
9/6/2018	BL	8A	9	2018	Year-1		1	Fall
9/6/2018	BL	9A	9	2018	Year-1	0	0	Fall
9/6/2018	BL	11A	9	2018	Year-1	0	0	Fall
9/6/2018	BL	13A	9	2018	Year-1	0	0	Fall
9/7/2018	BL	1M	9	2018	Year-1	0	0	Fall
9/7/2018	BL	2M	9	2018	Year-1		1	Fall
9/7/2018	BL	3M	9	2018	Year-1	0	0	Fall
9/7/2018	BL	4M	9	2018	Year-1	0	0	Fall
9/7/2018	BL	5M	9	2018	Year-1		1	Fall

Date	Species	Site	Month	Year	Survey Year	Presence	Data Gap	Season
9/7/2018	BL	6M	9	2018	Year-1	0	0	Fall
9/7/2018	BL	7M	9	2018	Year-1	0	0	Fall
9/7/2018	BL	10M	9	2018	Year-1		1	Fall
9/7/2018	BL	12M	9	2018	Year-1		1	Fall
9/7/2018	BL	14M	9	2018	Year-1	0	0	Fall
9/7/2018	BL	8A	9	2018	Year-1		1	Fall
9/7/2018	BL	9A	9	2018	Year-1	0	0	Fall
9/7/2018	BL	11A	9	2018	Year-1	0	0	Fall
9/7/2018	BL	13A	9	2018	Year-1	0	0	Fall
9/8/2018	BL	1M	9	2018	Year-1	0	0	Fall
9/8/2018	BL	2M	9	2018	Year-1		1	Fall
9/8/2018	BL	3M	9	2018	Year-1	0	0	Fall
9/8/2018	BL	4M	9	2018	Year-1	0	0	Fall
9/8/2018	BL	5M	9	2018	Year-1		1	Fall
9/8/2018	BL	6M	9	2018	Year-1	0	0	Fall
9/8/2018	BL	7M	9	2018	Year-1	0	0	Fall
9/8/2018	BL	10M	9	2018	Year-1		1	Fall
9/8/2018	BL	12M	9	2018	Year-1		1	Fall
9/8/2018	BL	14M	9	2018	Year-1	0	0	Fall
9/8/2018	BL	8A	9	2018	Year-1		1	Fall
9/8/2018	BL	9A	9	2018	Year-1	0	0	Fall
9/8/2018	BL	11A	9	2018	Year-1	0	0	Fall
9/8/2018	BL	13A	9	2018	Year-1	0	0	Fall
9/9/2018	BL	1M	9	2018	Year-1	0	0	Fall
9/9/2018	BL	2M	9	2018	Year-1		1	Fall
9/9/2018	BL	3M	9	2018	Year-1	0	0	Fall
9/9/2018	BL	4M	9	2018	Year-1	0	0	Fall
9/9/2018	BL	5M	9	2018	Year-1		1	Fall
9/9/2018	BL	6M	9	2018	Year-1	0	0	Fall
9/9/2018	BL	7M	9	2018	Year-1	0	0	Fall
9/9/2018	BL	10M	9	2018	Year-1		1	Fall
9/9/2018	BL	12M	9	2018	Year-1		1	Fall
9/9/2018	BL	14M	9	2018	Year-1	0	0	Fall
9/9/2018	BL	8A	9	2018	Year-1		1	Fall
9/9/2018	BL	9A	9	2018	Year-1	0	0	Fall
9/9/2018	BL	11A	9	2018	Year-1	0	0	Fall
9/9/2018	BL	13A	9	2018	Year-1	0	0	Fall
9/10/2018	BL	1M	9	2018	Year-1	0	0	Fall
9/10/2018	BL	2M	9	2018	Year-1		1	Fall
9/10/2018	BL	3M	9	2018	Year-1	0	0	Fall
9/10/2018	BL	4M	9	2018	Year-1	0	0	Fall
9/10/2018	BL	5M	9	2018	Year-1		1	Fall
9/10/2018	BL	6M	9	2018	Year-1	0	0	Fall
9/10/2018	BL	7M	9	2018	Year-1	0	0	Fall

Date	Species	Site	Month	Year	Survey Year	Presence	Data Gap	Season
9/10/2018	BL	10M	9	2018	Year-1		1	Fall
9/10/2018	BL	12M	9	2018	Year-1		1	Fall
9/10/2018	BL	14M	9	2018	Year-1	0	0	Fall
9/10/2018	BL	8A	9	2018	Year-1		1	Fall
9/10/2018	BL	9A	9	2018	Year-1	0	0	Fall
9/10/2018	BL	11A	9	2018	Year-1	0	0	Fall
9/10/2018	BL	13A	9	2018	Year-1	0	0	Fall
9/11/2018	BL	1M	9	2018	Year-1	0	0	Fall
9/11/2018	BL	2M	9	2018	Year-1		1	Fall
9/11/2018	BL	3M	9	2018	Year-1	0	0	Fall
9/11/2018	BL	4M	9	2018	Year-1	0	0	Fall
9/11/2018	BL	5M	9	2018	Year-1		1	Fall
9/11/2018	BL	6M	9	2018	Year-1	0	0	Fall
9/11/2018	BL	7M	9	2018	Year-1	0	0	Fall
9/11/2018	BL	10M	9	2018	Year-1		1	Fall
9/11/2018	BL	12M	9	2018	Year-1		1	Fall
9/11/2018	BL	14M	9	2018	Year-1	0	0	Fall
9/11/2018	BL	8A	9	2018	Year-1		1	Fall
9/11/2018	BL	9A	9	2018	Year-1	0	0	Fall
9/11/2018	BL	11A	9	2018	Year-1	0	0	Fall
9/11/2018	BL	13A	9	2018	Year-1	0	0	Fall
9/12/2018	BL	1M	9	2018	Year-1	0	0	Fall
9/12/2018	BL	2M	9	2018	Year-1		1	Fall
9/12/2018	BL	3M	9	2018	Year-1	0	0	Fall
9/12/2018	BL	4M	9	2018	Year-1	0	0	Fall
9/12/2018	BL	5M	9	2018	Year-1		1	Fall
9/12/2018	BL	6M	9	2018	Year-1	0	0	Fall
9/12/2018	BL	7M	9	2018	Year-1	0	0	Fall
9/12/2018	BL	10M	9	2018	Year-1		1	Fall
9/12/2018	BL	12M	9	2018	Year-1		1	Fall
9/12/2018	BL	14M	9	2018	Year-1	0	0	Fall
9/12/2018	BL	8A	9	2018	Year-1		1	Fall
9/12/2018	BL	9A	9	2018	Year-1	0	0	Fall
9/12/2018	BL	11A	9	2018	Year-1	0	0	Fall
9/12/2018	BL	13A	9	2018	Year-1	0	0	Fall
9/13/2018	BL	1M	9	2018	Year-1	0	0	Fall
9/13/2018	BL	2M	9	2018	Year-1		1	Fall
9/13/2018	BL	3M	9	2018	Year-1	0	0	Fall
9/13/2018	BL	4M	9	2018	Year-1	0	0	Fall
9/13/2018	BL	5M	9	2018	Year-1		1	Fall
9/13/2018	BL	6M	9	2018	Year-1	0	0	Fall
9/13/2018	BL	7M	9	2018	Year-1	0	0	Fall
9/13/2018	BL	10M	9	2018	Year-1		1	Fall
9/13/2018	BL	12M	9	2018	Year-1		1	Fall

Date	Species	Site	Month	Year	Survey Year	Presence	Data Gap	Season
9/13/2018	BL	14M	9	2018	Year-1	0	0	Fall
9/13/2018	BL	8A	9	2018	Year-1		1	Fall
9/13/2018	BL	9A	9	2018	Year-1	0	0	Fall
9/13/2018	BL	11A	9	2018	Year-1	0	0	Fall
9/13/2018	BL	13A	9	2018	Year-1	0	0	Fall
9/14/2018	BL	1M	9	2018	Year-1	0	0	Fall
9/14/2018	BL	2M	9	2018	Year-1		1	Fall
9/14/2018	BL	3M	9	2018	Year-1	0	0	Fall
9/14/2018	BL	4M	9	2018	Year-1	0	0	Fall
9/14/2018	BL	5M	9	2018	Year-1		1	Fall
9/14/2018	BL	6M	9	2018	Year-1	0	0	Fall
9/14/2018	BL	7M	9	2018	Year-1	0	0	Fall
9/14/2018	BL	10M	9	2018	Year-1		1	Fall
9/14/2018	BL	12M	9	2018	Year-1		1	Fall
9/14/2018	BL	14M	9	2018	Year-1	0	0	Fall
9/14/2018	BL	8A	9	2018	Year-1		1	Fall
9/14/2018	BL	9A	9	2018	Year-1	0	0	Fall
9/14/2018	BL	11A	9	2018	Year-1	0	0	Fall
9/14/2018	BL	13A	9	2018	Year-1	0	0	Fall
9/15/2018	BL	1M	9	2018	Year-1	0	0	Fall
9/15/2018	BL	2M	9	2018	Year-1		1	Fall
9/15/2018	BL	3M	9	2018	Year-1	0	0	Fall
9/15/2018	BL	4M	9	2018	Year-1	0	0	Fall
9/15/2018	BL	5M	9	2018	Year-1		1	Fall
9/15/2018	BL	6M	9	2018	Year-1	0	0	Fall
9/15/2018	BL	7M	9	2018	Year-1	0	0	Fall
9/15/2018	BL	10M	9	2018	Year-1		1	Fall
9/15/2018	BL	12M	9	2018	Year-1		1	Fall
9/15/2018	BL	14M	9	2018	Year-1	0	0	Fall
9/15/2018	BL	8A	9	2018	Year-1		1	Fall
9/15/2018	BL	9A	9	2018	Year-1	0	0	Fall
9/15/2018	BL	11A	9	2018	Year-1	0	0	Fall
9/15/2018	BL	13A	9	2018	Year-1	0	0	Fall
9/16/2018	BL	1M	9	2018	Year-1	0	0	Fall
9/16/2018	BL	2M	9	2018	Year-1		1	Fall
9/16/2018	BL	3M	9	2018	Year-1	0	0	Fall
9/16/2018	BL	4M	9	2018	Year-1		1	Fall
9/16/2018	BL	5M	9	2018	Year-1		1	Fall
9/16/2018	BL	6M	9	2018	Year-1	0	0	Fall
9/16/2018	BL	7M	9	2018	Year-1	0	0	Fall
9/16/2018	BL	10M	9	2018	Year-1		1	Fall
9/16/2018	BL	12M	9	2018	Year-1		1	Fall
9/16/2018	BL	14M	9	2018	Year-1	0	0	Fall
9/16/2018	BL	8A	9	2018	Year-1		1	Fall

Date	Species	Site	Month	Year	Survey Year	Presence	Data Gap	Season
9/16/2018	BL	9A	9	2018	Year-1	0	0	Fall
9/16/2018	BL	11A	9	2018	Year-1	0	0	Fall
9/16/2018	BL	13A	9	2018	Year-1	0	0	Fall
9/17/2018	BL	1M	9	2018	Year-1	0	0	Fall
9/17/2018	BL	2M	9	2018	Year-1		1	Fall
9/17/2018	BL	3M	9	2018	Year-1	0	0	Fall
9/17/2018	BL	4M	9	2018	Year-1		1	Fall
9/17/2018	BL	5M	9	2018	Year-1		1	Fall
9/17/2018	BL	6M	9	2018	Year-1	0	0	Fall
9/17/2018	BL	7M	9	2018	Year-1	0	0	Fall
9/17/2018	BL	10M	9	2018	Year-1		1	Fall
9/17/2018	BL	12M	9	2018	Year-1		1	Fall
9/17/2018	BL	14M	9	2018	Year-1	0	0	Fall
9/17/2018	BL	8A	9	2018	Year-1		1	Fall
9/17/2018	BL	9A	9	2018	Year-1	0	0	Fall
9/17/2018	BL	11A	9	2018	Year-1	0	0	Fall
9/17/2018	BL	13A	9	2018	Year-1	0	0	Fall
9/18/2018	BL	1M	9	2018	Year-1	0	0	Fall
9/18/2018	BL	2M	9	2018	Year-1		1	Fall
9/18/2018	BL	3M	9	2018	Year-1	0	0	Fall
9/18/2018	BL	4M	9	2018	Year-1		1	Fall
9/18/2018	BL	5M	9	2018	Year-1		1	Fall
9/18/2018	BL	6M	9	2018	Year-1	0	0	Fall
9/18/2018	BL	7M	9	2018	Year-1	0	0	Fall
9/18/2018	BL	10M	9	2018	Year-1		1	Fall
9/18/2018	BL	12M	9	2018	Year-1		1	Fall
9/18/2018	BL	14M	9	2018	Year-1	0	0	Fall
9/18/2018	BL	8A	9	2018	Year-1		1	Fall
9/18/2018	BL	9A	9	2018	Year-1	0	0	Fall
9/18/2018	BL	11A	9	2018	Year-1	0	0	Fall
9/18/2018	BL	13A	9	2018	Year-1	0	0	Fall
9/19/2018	BL	1M	9	2018	Year-1	0	0	Fall
9/19/2018	BL	2M	9	2018	Year-1		1	Fall
9/19/2018	BL	3M	9	2018	Year-1	0	0	Fall
9/19/2018	BL	4M	9	2018	Year-1		1	Fall
9/19/2018	BL	5M	9	2018	Year-1		1	Fall
9/19/2018	BL	6M	9	2018	Year-1	0	0	Fall
9/19/2018	BL	7M	9	2018	Year-1	0	0	Fall
9/19/2018	BL	10M	9	2018	Year-1		1	Fall
9/19/2018	BL	12M	9	2018	Year-1		1	Fall
9/19/2018	BL	14M	9	2018	Year-1	0	0	Fall
9/19/2018	BL	8A	9	2018	Year-1		1	Fall
9/19/2018	BL	9A	9	2018	Year-1	0	0	Fall
9/19/2018	BL	11A	9	2018	Year-1	0	0	Fall

Date	Species	Site	Month	Year	Survey Year	Presence	Data Gap	Season
9/19/2018	BL	13A	9	2018	Year-1	0	0	Fall
9/20/2018	BL	1M	9	2018	Year-1	0	0	Fall
9/20/2018	BL	2M	9	2018	Year-1		1	Fall
9/20/2018	BL	3M	9	2018	Year-1	0	0	Fall
9/20/2018	BL	4M	9	2018	Year-1		1	Fall
9/20/2018	BL	5M	9	2018	Year-1		1	Fall
9/20/2018	BL	6M	9	2018	Year-1	0	0	Fall
9/20/2018	BL	7M	9	2018	Year-1	0	0	Fall
9/20/2018	BL	10M	9	2018	Year-1		1	Fall
9/20/2018	BL	12M	9	2018	Year-1		1	Fall
9/20/2018	BL	14M	9	2018	Year-1	0	0	Fall
9/20/2018	BL	8A	9	2018	Year-1		1	Fall
9/20/2018	BL	9A	9	2018	Year-1	0	0	Fall
9/20/2018	BL	11A	9	2018	Year-1	0	0	Fall
9/20/2018	BL	13A	9	2018	Year-1	0	0	Fall
9/21/2018	BL	1M	9	2018	Year-1	0	0	Fall
9/21/2018	BL	2M	9	2018	Year-1		1	Fall
9/21/2018	BL	3M	9	2018	Year-1	0	0	Fall
9/21/2018	BL	4M	9	2018	Year-1		1	Fall
9/21/2018	BL	5M	9	2018	Year-1		1	Fall
9/21/2018	BL	6M	9	2018	Year-1	0	0	Fall
9/21/2018	BL	7M	9	2018	Year-1	0	0	Fall
9/21/2018	BL	10M	9	2018	Year-1		1	Fall
9/21/2018	BL	12M	9	2018	Year-1		1	Fall
9/21/2018	BL	14M	9	2018	Year-1	0	0	Fall
9/21/2018	BL	8A	9	2018	Year-1		1	Fall
9/21/2018	BL	9A	9	2018	Year-1	0	0	Fall
9/21/2018	BL	11A	9	2018	Year-1	0	0	Fall
9/21/2018	BL	13A	9	2018	Year-1	0	0	Fall
9/22/2018	BL	1M	9	2018	Year-1	0	0	Fall
9/22/2018	BL	2M	9	2018	Year-1		1	Fall
9/22/2018	BL	3M	9	2018	Year-1	0	0	Fall
9/22/2018	BL	4M	9	2018	Year-1		1	Fall
9/22/2018	BL	5M	9	2018	Year-1		1	Fall
9/22/2018	BL	6M	9	2018	Year-1	0	0	Fall
9/22/2018	BL	7M	9	2018	Year-1	0	0	Fall
9/22/2018	BL	10M	9	2018	Year-1		1	Fall
9/22/2018	BL	12M	9	2018	Year-1		1	Fall
9/22/2018	BL	14M	9	2018	Year-1	0	0	Fall
9/22/2018	BL	8A	9	2018	Year-1		1	Fall
9/22/2018	BL	9A	9	2018	Year-1	0	0	Fall
9/22/2018	BL	11A	9	2018	Year-1	0	0	Fall
9/22/2018	BL	13A	9	2018	Year-1	0	0	Fall
9/23/2018	BL	1M	9	2018	Year-1	0	0	Fall

Date	Species	Site	Month	Year	Survey Year	Presence	Data Gap	Season
9/23/2018	BL	2M	9	2018	Year-1		1	Fall
9/23/2018	BL	3M	9	2018	Year-1	0	0	Fall
9/23/2018	BL	4M	9	2018	Year-1		1	Fall
9/23/2018	BL	5M	9	2018	Year-1		1	Fall
9/23/2018	BL	6M	9	2018	Year-1	0	0	Fall
9/23/2018	BL	7M	9	2018	Year-1	0	0	Fall
9/23/2018	BL	10M	9	2018	Year-1		1	Fall
9/23/2018	BL	12M	9	2018	Year-1		1	Fall
9/23/2018	BL	14M	9	2018	Year-1	0	0	Fall
9/23/2018	BL	8A	9	2018	Year-1		1	Fall
9/23/2018	BL	9A	9	2018	Year-1	0	0	Fall
9/23/2018	BL	11A	9	2018	Year-1	0	0	Fall
9/23/2018	BL	13A	9	2018	Year-1	0	0	Fall
9/24/2018	BL	1M	9	2018	Year-1	0	0	Fall
9/24/2018	BL	2M	9	2018	Year-1		1	Fall
9/24/2018	BL	3M	9	2018	Year-1	0	0	Fall
9/24/2018	BL	4M	9	2018	Year-1		1	Fall
9/24/2018	BL	5M	9	2018	Year-1		1	Fall
9/24/2018	BL	6M	9	2018	Year-1	0	0	Fall
9/24/2018	BL	7M	9	2018	Year-1	0	0	Fall
9/24/2018	BL	10M	9	2018	Year-1		1	Fall
9/24/2018	BL	12M	9	2018	Year-1		1	Fall
9/24/2018	BL	14M	9	2018	Year-1	0	0	Fall
9/24/2018	BL	8A	9	2018	Year-1		1	Fall
9/24/2018	BL	9A	9	2018	Year-1	0	0	Fall
9/24/2018	BL	11A	9	2018	Year-1	0	0	Fall
9/24/2018	BL	13A	9	2018	Year-1	0	0	Fall
9/25/2018	BL	1M	9	2018	Year-1	0	0	Fall
9/25/2018	BL	2M	9	2018	Year-1		1	Fall
9/25/2018	BL	3M	9	2018	Year-1	0	0	Fall
9/25/2018	BL	4M	9	2018	Year-1		1	Fall
9/25/2018	BL	5M	9	2018	Year-1		1	Fall
9/25/2018	BL	6M	9	2018	Year-1	0	0	Fall
9/25/2018	BL	7M	9	2018	Year-1	0	0	Fall
9/25/2018	BL	10M	9	2018	Year-1		1	Fall
9/25/2018	BL	12M	9	2018	Year-1		1	Fall
9/25/2018	BL	14M	9	2018	Year-1	0	0	Fall
9/25/2018	BL	8A	9	2018	Year-1		1	Fall
9/25/2018	BL	9A	9	2018	Year-1	0	0	Fall
9/25/2018	BL	11A	9	2018	Year-1	0	0	Fall
9/25/2018	BL	13A	9	2018	Year-1	0	0	Fall
9/26/2018	BL	1M	9	2018	Year-1	0	0	Fall
9/26/2018	BL	2M	9	2018	Year-1		1	Fall
9/26/2018	BL	3M	9	2018	Year-1	0	0	Fall

Date	Species	Site	Month	Year	Survey Year	Presence	Data Gap	Season
9/26/2018	BL	4M	9	2018	Year-1		1	Fall
9/26/2018	BL	5M	9	2018	Year-1		1	Fall
9/26/2018	BL	6M	9	2018	Year-1	0	0	Fall
9/26/2018	BL	7M	9	2018	Year-1	0	0	Fall
9/26/2018	BL	10M	9	2018	Year-1		1	Fall
9/26/2018	BL	12M	9	2018	Year-1		1	Fall
9/26/2018	BL	14M	9	2018	Year-1	0	0	Fall
9/26/2018	BL	8A	9	2018	Year-1		1	Fall
9/26/2018	BL	9A	9	2018	Year-1	0	0	Fall
9/26/2018	BL	11A	9	2018	Year-1	0	0	Fall
9/26/2018	BL	13A	9	2018	Year-1	0	0	Fall
9/27/2018	BL	1M	9	2018	Year-1	0	0	Fall
9/27/2018	BL	2M	9	2018	Year-1		1	Fall
9/27/2018	BL	3M	9	2018	Year-1	0	0	Fall
9/27/2018	BL	4M	9	2018	Year-1		1	Fall
9/27/2018	BL	5M	9	2018	Year-1		1	Fall
9/27/2018	BL	6M	9	2018	Year-1	0	0	Fall
9/27/2018	BL	7M	9	2018	Year-1	0	0	Fall
9/27/2018	BL	10M	9	2018	Year-1		1	Fall
9/27/2018	BL	12M	9	2018	Year-1		1	Fall
9/27/2018	BL	14M	9	2018	Year-1	0	0	Fall
9/27/2018	BL	8A	9	2018	Year-1		1	Fall
9/27/2018	BL	9A	9	2018	Year-1	0	0	Fall
9/27/2018	BL	11A	9	2018	Year-1	0	0	Fall
9/27/2018	BL	13A	9	2018	Year-1	0	0	Fall
9/28/2018	BL	1M	9	2018	Year-1	0	0	Fall
9/28/2018	BL	2M	9	2018	Year-1		1	Fall
9/28/2018	BL	3M	9	2018	Year-1	0	0	Fall
9/28/2018	BL	4M	9	2018	Year-1		1	Fall
9/28/2018	BL	5M	9	2018	Year-1		1	Fall
9/28/2018	BL	6M	9	2018	Year-1	0	0	Fall
9/28/2018	BL	7M	9	2018	Year-1	0	0	Fall
9/28/2018	BL	10M	9	2018	Year-1		1	Fall
9/28/2018	BL	12M	9	2018	Year-1		1	Fall
9/28/2018	BL	14M	9	2018	Year-1	0	0	Fall
9/28/2018	BL	8A	9	2018	Year-1		1	Fall
9/28/2018	BL	9A	9	2018	Year-1	0	0	Fall
9/28/2018	BL	11A	9	2018	Year-1	0	0	Fall
9/28/2018	BL	13A	9	2018	Year-1	0	0	Fall
9/29/2018	BL	1M	9	2018	Year-1	0	0	Fall
9/29/2018	BL	2M	9	2018	Year-1		1	Fall
9/29/2018	BL	3M	9	2018	Year-1	0	0	Fall
9/29/2018	BL	4M	9	2018	Year-1		1	Fall
9/29/2018	BL	5M	9	2018	Year-1		1	Fall

Date	Species	Site	Month	Year	Survey Year	Presence	Data Gap	Season
9/29/2018	BL	6M	9	2018	Year-1	0	0	Fall
9/29/2018	BL	7M	9	2018	Year-1	0	0	Fall
9/29/2018	BL	10M	9	2018	Year-1		1	Fall
9/29/2018	BL	12M	9	2018	Year-1		1	Fall
9/29/2018	BL	14M	9	2018	Year-1	0	0	Fall
9/29/2018	BL	8A	9	2018	Year-1		1	Fall
9/29/2018	BL	9A	9	2018	Year-1	0	0	Fall
9/29/2018	BL	11A	9	2018	Year-1	0	0	Fall
9/29/2018	BL	13A	9	2018	Year-1	0	0	Fall
9/30/2018	BL	1M	9	2018	Year-1	0	0	Fall
9/30/2018	BL	2M	9	2018	Year-1		1	Fall
9/30/2018	BL	3M	9	2018	Year-1	0	0	Fall
9/30/2018	BL	4M	9	2018	Year-1		1	Fall
9/30/2018	BL	5M	9	2018	Year-1		1	Fall
9/30/2018	BL	6M	9	2018	Year-1	0	0	Fall
9/30/2018	BL	7M	9	2018	Year-1	0	0	Fall
9/30/2018	BL	10M	9	2018	Year-1		1	Fall
9/30/2018	BL	12M	9	2018	Year-1		1	Fall
9/30/2018	BL	14M	9	2018	Year-1	0	0	Fall
9/30/2018	BL	8A	9	2018	Year-1		1	Fall
9/30/2018	BL	9A	9	2018	Year-1	0	0	Fall
9/30/2018	BL	11A	9	2018	Year-1	0	0	Fall
9/30/2018	BL	13A	9	2018	Year-1	0	0	Fall
10/1/2018	BL	1M	10	2018	Year-1	0	0	Fall
10/1/2018	BL	2M	10	2018	Year-1		1	Fall
10/1/2018	BL	3M	10	2018	Year-1	0	0	Fall
10/1/2018	BL	4M	10	2018	Year-1		1	Fall
10/1/2018	BL	5M	10	2018	Year-1		1	Fall
10/1/2018	BL	6M	10	2018	Year-1		1	Fall
10/1/2018	BL	7M	10	2018	Year-1	0	0	Fall
10/1/2018	BL	10M	10	2018	Year-1		1	Fall
10/1/2018	BL	12M	10	2018	Year-1		1	Fall
10/1/2018	BL	14M	10	2018	Year-1	0	0	Fall
10/1/2018	BL	8A	10	2018	Year-1		1	Fall
10/1/2018	BL	9A	10	2018	Year-1	0	0	Fall
10/1/2018	BL	11A	10	2018	Year-1	0	0	Fall
10/1/2018	BL	13A	10	2018	Year-1	0	0	Fall
10/2/2018	BL	1M	10	2018	Year-1	0	0	Fall
10/2/2018	BL	2M	10	2018	Year-1		1	Fall
10/2/2018	BL	3M	10	2018	Year-1	0	0	Fall
10/2/2018	BL	4M	10	2018	Year-1		1	Fall
10/2/2018	BL	5M	10	2018	Year-1		1	Fall
10/2/2018	BL	6M	10	2018	Year-1		1	Fall
10/2/2018	BL	7M	10	2018	Year-1	0	0	Fall

Date	Species	Site	Month	Year	Survey Year	Presence	Data Gap	Season
10/2/2018	BL	10M	10	2018	Year-1		1	Fall
10/2/2018	BL	12M	10	2018	Year-1		1	Fall
10/2/2018	BL	14M	10	2018	Year-1	0	0	Fall
10/2/2018	BL	8A	10	2018	Year-1		1	Fall
10/2/2018	BL	9A	10	2018	Year-1	0	0	Fall
10/2/2018	BL	11A	10	2018	Year-1	0	0	Fall
10/2/2018	BL	13A	10	2018	Year-1	0	0	Fall
10/3/2018	BL	1M	10	2018	Year-1	0	0	Fall
10/3/2018	BL	2M	10	2018	Year-1		1	Fall
10/3/2018	BL	3M	10	2018	Year-1	0	0	Fall
10/3/2018	BL	4M	10	2018	Year-1		1	Fall
10/3/2018	BL	5M	10	2018	Year-1		1	Fall
10/3/2018	BL	6M	10	2018	Year-1		1	Fall
10/3/2018	BL	7M	10	2018	Year-1	0	0	Fall
10/3/2018	BL	10M	10	2018	Year-1		1	Fall
10/3/2018	BL	12M	10	2018	Year-1		1	Fall
10/3/2018	BL	14M	10	2018	Year-1	0	0	Fall
10/3/2018	BL	8A	10	2018	Year-1		1	Fall
10/3/2018	BL	9A	10	2018	Year-1	0	0	Fall
10/3/2018	BL	11A	10	2018	Year-1	0	0	Fall
10/3/2018	BL	13A	10	2018	Year-1	0	0	Fall
10/4/2018	BL	1M	10	2018	Year-1	0	0	Fall
10/4/2018	BL	2M	10	2018	Year-1		1	Fall
10/4/2018	BL	3M	10	2018	Year-1	0	0	Fall
10/4/2018	BL	4M	10	2018	Year-1		1	Fall
10/4/2018	BL	5M	10	2018	Year-1		1	Fall
10/4/2018	BL	6M	10	2018	Year-1		1	Fall
10/4/2018	BL	7M	10	2018	Year-1	0	0	Fall
10/4/2018	BL	10M	10	2018	Year-1		1	Fall
10/4/2018	BL	12M	10	2018	Year-1		1	Fall
10/4/2018	BL	14M	10	2018	Year-1	0	0	Fall
10/4/2018	BL	8A	10	2018	Year-1		1	Fall
10/4/2018	BL	9A	10	2018	Year-1	0	0	Fall
10/4/2018	BL	11A	10	2018	Year-1	0	0	Fall
10/4/2018	BL	13A	10	2018	Year-1	0	0	Fall
10/5/2018	BL	1M	10	2018	Year-1	0	0	Fall
10/5/2018	BL	2M	10	2018	Year-1		1	Fall
10/5/2018	BL	3M	10	2018	Year-1	0	0	Fall
10/5/2018	BL	4M	10	2018	Year-1		1	Fall
10/5/2018	BL	5M	10	2018	Year-1		1	Fall
10/5/2018	BL	6M	10	2018	Year-1		1	Fall
10/5/2018	BL	7M	10	2018	Year-1	0	0	Fall
10/5/2018	BL	10M	10	2018	Year-1		1	Fall
10/5/2018	BL	12M	10	2018	Year-1		1	Fall

Date	Species	Site	Month	Year	Survey Year	Presence	Data Gap	Season
10/5/2018	BL	14M	10	2018	Year-1	0	0	Fall
10/5/2018	BL	8A	10	2018	Year-1		1	Fall
10/5/2018	BL	9A	10	2018	Year-1	0	0	Fall
10/5/2018	BL	11A	10	2018	Year-1	0	0	Fall
10/5/2018	BL	13A	10	2018	Year-1	0	0	Fall
10/6/2018	BL	1M	10	2018	Year-1	0	0	Fall
10/6/2018	BL	2M	10	2018	Year-1		1	Fall
10/6/2018	BL	3M	10	2018	Year-1	0	0	Fall
10/6/2018	BL	4M	10	2018	Year-1		1	Fall
10/6/2018	BL	5M	10	2018	Year-1		1	Fall
10/6/2018	BL	6M	10	2018	Year-1		1	Fall
10/6/2018	BL	7M	10	2018	Year-1	0	0	Fall
10/6/2018	BL	10M	10	2018	Year-1		1	Fall
10/6/2018	BL	12M	10	2018	Year-1		1	Fall
10/6/2018	BL	14M	10	2018	Year-1	0	0	Fall
10/6/2018	BL	8A	10	2018	Year-1		1	Fall
10/6/2018	BL	9A	10	2018	Year-1	0	0	Fall
10/6/2018	BL	11A	10	2018	Year-1	0	0	Fall
10/6/2018	BL	13A	10	2018	Year-1	0	0	Fall
10/7/2018	BL	1M	10	2018	Year-1	0	0	Fall
10/7/2018	BL	2M	10	2018	Year-1		1	Fall
10/7/2018	BL	3M	10	2018	Year-1	0	0	Fall
10/7/2018	BL	4M	10	2018	Year-1		1	Fall
10/7/2018	BL	5M	10	2018	Year-1		1	Fall
10/7/2018	BL	6M	10	2018	Year-1		1	Fall
10/7/2018	BL	7M	10	2018	Year-1	0	0	Fall
10/7/2018	BL	10M	10	2018	Year-1		1	Fall
10/7/2018	BL	12M	10	2018	Year-1		1	Fall
10/7/2018	BL	14M	10	2018	Year-1	0	0	Fall
10/7/2018	BL	8A	10	2018	Year-1		1	Fall
10/7/2018	BL	9A	10	2018	Year-1	0	0	Fall
10/7/2018	BL	11A	10	2018	Year-1	0	0	Fall
10/7/2018	BL	13A	10	2018	Year-1	0	0	Fall
10/8/2018	BL	1M	10	2018	Year-1	0	0	Fall
10/8/2018	BL	2M	10	2018	Year-1		1	Fall
10/8/2018	BL	3M	10	2018	Year-1	0	0	Fall
10/8/2018	BL	4M	10	2018	Year-1		1	Fall
10/8/2018	BL	5M	10	2018	Year-1		1	Fall
10/8/2018	BL	6M	10	2018	Year-1		1	Fall
10/8/2018	BL	7M	10	2018	Year-1	0	0	Fall
10/8/2018	BL	10M	10	2018	Year-1		1	Fall
10/8/2018	BL	12M	10	2018	Year-1		1	Fall
10/8/2018	BL	14M	10	2018	Year-1	0	0	Fall
10/8/2018	BL	8A	10	2018	Year-1		1	Fall

Date	Species	Site	Month	Year	Survey Year	Presence	Data Gap	Season
10/8/2018	BL	9A	10	2018	Year-1	0	0	Fall
10/8/2018	BL	11A	10	2018	Year-1	0	0	Fall
10/8/2018	BL	13A	10	2018	Year-1	0	0	Fall
10/9/2018	BL	1M	10	2018	Year-1	0	0	Fall
10/9/2018	BL	2M	10	2018	Year-1		1	Fall
10/9/2018	BL	3M	10	2018	Year-1	0	0	Fall
10/9/2018	BL	4M	10	2018	Year-1		1	Fall
10/9/2018	BL	5M	10	2018	Year-1		1	Fall
10/9/2018	BL	6M	10	2018	Year-1		1	Fall
10/9/2018	BL	7M	10	2018	Year-1	0	0	Fall
10/9/2018	BL	10M	10	2018	Year-1		1	Fall
10/9/2018	BL	12M	10	2018	Year-1		1	Fall
10/9/2018	BL	14M	10	2018	Year-1	0	0	Fall
10/9/2018	BL	8A	10	2018	Year-1		1	Fall
10/9/2018	BL	9A	10	2018	Year-1	0	0	Fall
10/9/2018	BL	11A	10	2018	Year-1	0	0	Fall
10/9/2018	BL	13A	10	2018	Year-1	0	0	Fall
10/10/2018	BL	1M	10	2018	Year-1	0	0	Fall
10/10/2018	BL	2M	10	2018	Year-1		1	Fall
10/10/2018	BL	3M	10	2018	Year-1	0	0	Fall
10/10/2018	BL	4M	10	2018	Year-1		1	Fall
10/10/2018	BL	5M	10	2018	Year-1		1	Fall
10/10/2018	BL	6M	10	2018	Year-1		1	Fall
10/10/2018	BL	7M	10	2018	Year-1	0	0	Fall
10/10/2018	BL	10M	10	2018	Year-1		1	Fall
10/10/2018	BL	12M	10	2018	Year-1		1	Fall
10/10/2018	BL	14M	10	2018	Year-1	0	0	Fall
10/10/2018	BL	8A	10	2018	Year-1		1	Fall
10/10/2018	BL	9A	10	2018	Year-1	0	0	Fall
10/10/2018	BL	11A	10	2018	Year-1	0	0	Fall
10/10/2018	BL	13A	10	2018	Year-1	0	0	Fall
10/11/2018	BL	1M	10	2018	Year-1	0	0	Fall
10/11/2018	BL	2M	10	2018	Year-1		1	Fall
10/11/2018	BL	3M	10	2018	Year-1	0	0	Fall
10/11/2018	BL	4M	10	2018	Year-1		1	Fall
10/11/2018	BL	5M	10	2018	Year-1		1	Fall
10/11/2018	BL	6M	10	2018	Year-1		1	Fall
10/11/2018	BL	7M	10	2018	Year-1	0	0	Fall
10/11/2018	BL	10M	10	2018	Year-1		1	Fall
10/11/2018	BL	12M	10	2018	Year-1		1	Fall
10/11/2018	BL	14M	10	2018	Year-1	0	0	Fall
10/11/2018	BL	8A	10	2018	Year-1		1	Fall
10/11/2018	BL	9A	10	2018	Year-1	0	0	Fall
10/11/2018	BL	11A	10	2018	Year-1	0	0	Fall

Date	Species	Site	Month	Year	Survey Year	Presence	Data Gap	Season
10/11/2018	BL	13A	10	2018	Year-1	0	0	Fall
10/12/2018	BL	1M	10	2018	Year-1	0	0	Fall
10/12/2018	BL	2M	10	2018	Year-1		1	Fall
10/12/2018	BL	3M	10	2018	Year-1	0	0	Fall
10/12/2018	BL	4M	10	2018	Year-1		1	Fall
10/12/2018	BL	5M	10	2018	Year-1		1	Fall
10/12/2018	BL	6M	10	2018	Year-1		1	Fall
10/12/2018	BL	7M	10	2018	Year-1	0	0	Fall
10/12/2018	BL	10M	10	2018	Year-1		1	Fall
10/12/2018	BL	12M	10	2018	Year-1		1	Fall
10/12/2018	BL	14M	10	2018	Year-1	0	0	Fall
10/12/2018	BL	8A	10	2018	Year-1		1	Fall
10/12/2018	BL	9A	10	2018	Year-1	0	0	Fall
10/12/2018	BL	11A	10	2018	Year-1	0	0	Fall
10/12/2018	BL	13A	10	2018	Year-1	0	0	Fall
10/13/2018	BL	1M	10	2018	Year-1	0	0	Fall
10/13/2018	BL	2M	10	2018	Year-1		1	Fall
10/13/2018	BL	3M	10	2018	Year-1	0	0	Fall
10/13/2018	BL	4M	10	2018	Year-1		1	Fall
10/13/2018	BL	5M	10	2018	Year-1		1	Fall
10/13/2018	BL	6M	10	2018	Year-1		1	Fall
10/13/2018	BL	7M	10	2018	Year-1	0	0	Fall
10/13/2018	BL	10M	10	2018	Year-1		1	Fall
10/13/2018	BL	12M	10	2018	Year-1		1	Fall
10/13/2018	BL	14M	10	2018	Year-1	0	0	Fall
10/13/2018	BL	8A	10	2018	Year-1		1	Fall
10/13/2018	BL	9A	10	2018	Year-1	0	0	Fall
10/13/2018	BL	11A	10	2018	Year-1	0	0	Fall
10/13/2018	BL	13A	10	2018	Year-1	0	0	Fall
10/14/2018	BL	1M	10	2018	Year-1	0	0	Fall
10/14/2018	BL	2M	10	2018	Year-1		1	Fall
10/14/2018	BL	3M	10	2018	Year-1	0	0	Fall
10/14/2018	BL	4M	10	2018	Year-1		1	Fall
10/14/2018	BL	5M	10	2018	Year-1		1	Fall
10/14/2018	BL	6M	10	2018	Year-1		1	Fall
10/14/2018	BL	7M	10	2018	Year-1	0	0	Fall
10/14/2018	BL	10M	10	2018	Year-1		1	Fall
10/14/2018	BL	12M	10	2018	Year-1		1	Fall
10/14/2018	BL	14M	10	2018	Year-1	0	0	Fall
10/14/2018	BL	8A	10	2018	Year-1		1	Fall
10/14/2018	BL	9A	10	2018	Year-1	0	0	Fall
10/14/2018	BL	11A	10	2018	Year-1	0	0	Fall
10/14/2018	BL	13A	10	2018	Year-1	0	0	Fall
10/15/2018	BL	1M	10	2018	Year-1	0	0	Fall

Date	Species	Site	Month	Year	Survey Year	Presence	Data Gap	Season
10/15/2018	BL	2M	10	2018	Year-1		1	Fall
10/15/2018	BL	3M	10	2018	Year-1	0	0	Fall
10/15/2018	BL	4M	10	2018	Year-1		1	Fall
10/15/2018	BL	5M	10	2018	Year-1		1	Fall
10/15/2018	BL	6M	10	2018	Year-1		1	Fall
10/15/2018	BL	7M	10	2018	Year-1	0	0	Fall
10/15/2018	BL	10M	10	2018	Year-1		1	Fall
10/15/2018	BL	12M	10	2018	Year-1		1	Fall
10/15/2018	BL	14M	10	2018	Year-1	0	0	Fall
10/15/2018	BL	8A	10	2018	Year-1		1	Fall
10/15/2018	BL	9A	10	2018	Year-1	0	0	Fall
10/15/2018	BL	11A	10	2018	Year-1	0	0	Fall
10/15/2018	BL	13A	10	2018	Year-1	0	0	Fall
10/16/2018	BL	1M	10	2018	Year-2	0	0	Fall
10/16/2018	BL	2M	10	2018	Year-2		1	Fall
10/16/2018	BL	3M	10	2018	Year-2	0	0	Fall
10/16/2018	BL	4M	10	2018	Year-2		1	Fall
10/16/2018	BL	5M	10	2018	Year-2		1	Fall
10/16/2018	BL	6M	10	2018	Year-2		1	Fall
10/16/2018	BL	7M	10	2018	Year-2	0	0	Fall
10/16/2018	BL	10M	10	2018	Year-2		1	Fall
10/16/2018	BL	12M	10	2018	Year-2		1	Fall
10/16/2018	BL	14M	10	2018	Year-2	0	0	Fall
10/16/2018	BL	8A	10	2018	Year-2		1	Fall
10/16/2018	BL	9A	10	2018	Year-2	0	0	Fall
10/16/2018	BL	11A	10	2018	Year-2	0	0	Fall
10/16/2018	BL	13A	10	2018	Year-2	0	0	Fall
10/17/2018	BL	1M	10	2018	Year-2	0	0	Fall
10/17/2018	BL	2M	10	2018	Year-2		1	Fall
10/17/2018	BL	3M	10	2018	Year-2	0	0	Fall
10/17/2018	BL	4M	10	2018	Year-2		1	Fall
10/17/2018	BL	5M	10	2018	Year-2		1	Fall
10/17/2018	BL	6M	10	2018	Year-2		1	Fall
10/17/2018	BL	7M	10	2018	Year-2	0	0	Fall
10/17/2018	BL	10M	10	2018	Year-2		1	Fall
10/17/2018	BL	12M	10	2018	Year-2		1	Fall
10/17/2018	BL	14M	10	2018	Year-2	0	0	Fall
10/17/2018	BL	8A	10	2018	Year-2		1	Fall
10/17/2018	BL	9A	10	2018	Year-2	0	0	Fall
10/17/2018	BL	11A	10	2018	Year-2	0	0	Fall
10/17/2018	BL	13A	10	2018	Year-2	0	0	Fall
10/18/2018	BL	1M	10	2018	Year-2	0	0	Fall
10/18/2018	BL	2M	10	2018	Year-2		1	Fall
10/18/2018	BL	3M	10	2018	Year-2	0	0	Fall

Date	Species	Site	Month	Year	Survey Year	Presence	Data Gap	Season
10/18/2018	BL	4M	10	2018	Year-2		1	Fall
10/18/2018	BL	5M	10	2018	Year-2		1	Fall
10/18/2018	BL	6M	10	2018	Year-2		1	Fall
10/18/2018	BL	7M	10	2018	Year-2	0	0	Fall
10/18/2018	BL	10M	10	2018	Year-2		1	Fall
10/18/2018	BL	12M	10	2018	Year-2		1	Fall
10/18/2018	BL	14M	10	2018	Year-2	0	0	Fall
10/18/2018	BL	8A	10	2018	Year-2		1	Fall
10/18/2018	BL	9A	10	2018	Year-2	0	0	Fall
10/18/2018	BL	11A	10	2018	Year-2	0	0	Fall
10/18/2018	BL	13A	10	2018	Year-2	0	0	Fall
10/19/2018	BL	1M	10	2018	Year-2	0	0	Fall
10/19/2018	BL	2M	10	2018	Year-2		1	Fall
10/19/2018	BL	3M	10	2018	Year-2	0	0	Fall
10/19/2018	BL	4M	10	2018	Year-2		1	Fall
10/19/2018	BL	5M	10	2018	Year-2		1	Fall
10/19/2018	BL	6M	10	2018	Year-2		1	Fall
10/19/2018	BL	7M	10	2018	Year-2	0	0	Fall
10/19/2018	BL	10M	10	2018	Year-2		1	Fall
10/19/2018	BL	12M	10	2018	Year-2		1	Fall
10/19/2018	BL	14M	10	2018	Year-2	0	0	Fall
10/19/2018	BL	8A	10	2018	Year-2		1	Fall
10/19/2018	BL	9A	10	2018	Year-2	0	0	Fall
10/19/2018	BL	11A	10	2018	Year-2	0	0	Fall
10/19/2018	BL	13A	10	2018	Year-2	0	0	Fall
10/20/2018	BL	1M	10	2018	Year-2	0	0	Fall
10/20/2018	BL	2M	10	2018	Year-2		1	Fall
10/20/2018	BL	3M	10	2018	Year-2	0	0	Fall
10/20/2018	BL	4M	10	2018	Year-2		1	Fall
10/20/2018	BL	5M	10	2018	Year-2		1	Fall
10/20/2018	BL	6M	10	2018	Year-2		1	Fall
10/20/2018	BL	7M	10	2018	Year-2	0	0	Fall
10/20/2018	BL	10M	10	2018	Year-2		1	Fall
10/20/2018	BL	12M	10	2018	Year-2		1	Fall
10/20/2018	BL	14M	10	2018	Year-2	0	0	Fall
10/20/2018	BL	8A	10	2018	Year-2		1	Fall
10/20/2018	BL	9A	10	2018	Year-2	0	0	Fall
10/20/2018	BL	11A	10	2018	Year-2	0	0	Fall
10/20/2018	BL	13A	10	2018	Year-2	0	0	Fall
10/21/2018	BL	1M	10	2018	Year-2	0	0	Fall
10/21/2018	BL	2M	10	2018	Year-2		1	Fall
10/21/2018	BL	3M	10	2018	Year-2	0	0	Fall
10/21/2018	BL	4M	10	2018	Year-2		1	Fall
10/21/2018	BL	5M	10	2018	Year-2		1	Fall

Date	Species	Site	Month	Year	Survey Year	Presence	Data Gap	Season
10/21/2018	BL	6M	10	2018	Year-2		1	Fall
10/21/2018	BL	7M	10	2018	Year-2	0	0	Fall
10/21/2018	BL	10M	10	2018	Year-2		1	Fall
10/21/2018	BL	12M	10	2018	Year-2		1	Fall
10/21/2018	BL	14M	10	2018	Year-2	0	0	Fall
10/21/2018	BL	8A	10	2018	Year-2		1	Fall
10/21/2018	BL	9A	10	2018	Year-2	0	0	Fall
10/21/2018	BL	11A	10	2018	Year-2	0	0	Fall
10/21/2018	BL	13A	10	2018	Year-2	0	0	Fall
10/22/2018	BL	1M	10	2018	Year-2	0	0	Fall
10/22/2018	BL	2M	10	2018	Year-2		1	Fall
10/22/2018	BL	3M	10	2018	Year-2	0	0	Fall
10/22/2018	BL	4M	10	2018	Year-2		1	Fall
10/22/2018	BL	5M	10	2018	Year-2		1	Fall
10/22/2018	BL	6M	10	2018	Year-2		1	Fall
10/22/2018	BL	7M	10	2018	Year-2	0	0	Fall
10/22/2018	BL	10M	10	2018	Year-2		1	Fall
10/22/2018	BL	12M	10	2018	Year-2		1	Fall
10/22/2018	BL	14M	10	2018	Year-2	0	0	Fall
10/22/2018	BL	8A	10	2018	Year-2		1	Fall
10/22/2018	BL	9A	10	2018	Year-2	0	0	Fall
10/22/2018	BL	11A	10	2018	Year-2	0	0	Fall
10/22/2018	BL	13A	10	2018	Year-2	0	0	Fall
10/23/2018	BL	1M	10	2018	Year-2	0	0	Fall
10/23/2018	BL	2M	10	2018	Year-2		1	Fall
10/23/2018	BL	3M	10	2018	Year-2	0	0	Fall
10/23/2018	BL	4M	10	2018	Year-2		1	Fall
10/23/2018	BL	5M	10	2018	Year-2		1	Fall
10/23/2018	BL	6M	10	2018	Year-2		1	Fall
10/23/2018	BL	7M	10	2018	Year-2	0	0	Fall
10/23/2018	BL	10M	10	2018	Year-2		1	Fall
10/23/2018	BL	12M	10	2018	Year-2		1	Fall
10/23/2018	BL	14M	10	2018	Year-2	0	0	Fall
10/23/2018	BL	8A	10	2018	Year-2		1	Fall
10/23/2018	BL	9A	10	2018	Year-2	0	0	Fall
10/23/2018	BL	11A	10	2018	Year-2	0	0	Fall
10/23/2018	BL	13A	10	2018	Year-2	0	0	Fall
10/24/2018	BL	1M	10	2018	Year-2	0	0	Fall
10/24/2018	BL	2M	10	2018	Year-2		1	Fall
10/24/2018	BL	3M	10	2018	Year-2	0	0	Fall
10/24/2018	BL	4M	10	2018	Year-2		1	Fall
10/24/2018	BL	5M	10	2018	Year-2		1	Fall
10/24/2018	BL	6M	10	2018	Year-2		1	Fall
10/24/2018	BL	7M	10	2018	Year-2	0	0	Fall

Date	Species	Site	Month	Year	Survey Year	Presence	Data Gap	Season
10/24/2018	BL	10M	10	2018	Year-2		1	Fall
10/24/2018	BL	12M	10	2018	Year-2		1	Fall
10/24/2018	BL	14M	10	2018	Year-2	0	0	Fall
10/24/2018	BL	8A	10	2018	Year-2		1	Fall
10/24/2018	BL	9A	10	2018	Year-2	0	0	Fall
10/24/2018	BL	11A	10	2018	Year-2	0	0	Fall
10/24/2018	BL	13A	10	2018	Year-2	0	0	Fall
10/25/2018	BL	1M	10	2018	Year-2	0	0	Fall
10/25/2018	BL	2M	10	2018	Year-2		1	Fall
10/25/2018	BL	3M	10	2018	Year-2	0	0	Fall
10/25/2018	BL	4M	10	2018	Year-2		1	Fall
10/25/2018	BL	5M	10	2018	Year-2		1	Fall
10/25/2018	BL	6M	10	2018	Year-2		1	Fall
10/25/2018	BL	7M	10	2018	Year-2	0	0	Fall
10/25/2018	BL	10M	10	2018	Year-2		1	Fall
10/25/2018	BL	12M	10	2018	Year-2		1	Fall
10/25/2018	BL	14M	10	2018	Year-2	0	0	Fall
10/25/2018	BL	8A	10	2018	Year-2		1	Fall
10/25/2018	BL	9A	10	2018	Year-2	0	0	Fall
10/25/2018	BL	11A	10	2018	Year-2	0	0	Fall
10/25/2018	BL	13A	10	2018	Year-2	0	0	Fall
10/26/2018	BL	1M	10	2018	Year-2	0	0	Fall
10/26/2018	BL	2M	10	2018	Year-2		1	Fall
10/26/2018	BL	3M	10	2018	Year-2	0	0	Fall
10/26/2018	BL	4M	10	2018	Year-2		1	Fall
10/26/2018	BL	5M	10	2018	Year-2		1	Fall
10/26/2018	BL	6M	10	2018	Year-2		1	Fall
10/26/2018	BL	7M	10	2018	Year-2	0	0	Fall
10/26/2018	BL	10M	10	2018	Year-2		1	Fall
10/26/2018	BL	12M	10	2018	Year-2		1	Fall
10/26/2018	BL	14M	10	2018	Year-2	0	0	Fall
10/26/2018	BL	8A	10	2018	Year-2		1	Fall
10/26/2018	BL	9A	10	2018	Year-2	0	0	Fall
10/26/2018	BL	11A	10	2018	Year-2	0	0	Fall
10/26/2018	BL	13A	10	2018	Year-2	0	0	Fall
10/27/2018	BL	1M	10	2018	Year-2	0	0	Fall
10/27/2018	BL	2M	10	2018	Year-2		1	Fall
10/27/2018	BL	3M	10	2018	Year-2	0	0	Fall
10/27/2018	BL	4M	10	2018	Year-2		1	Fall
10/27/2018	BL	5M	10	2018	Year-2		1	Fall
10/27/2018	BL	6M	10	2018	Year-2		1	Fall
10/27/2018	BL	7M	10	2018	Year-2	0	0	Fall
10/27/2018	BL	10M	10	2018	Year-2		1	Fall
10/27/2018	BL	12M	10	2018	Year-2		1	Fall

Date	Species	Site	Month	Year	Survey Year	Presence	Data Gap	Season
10/27/2018	BL	14M	10	2018	Year-2	0	0	Fall
10/27/2018	BL	8A	10	2018	Year-2		1	Fall
10/27/2018	BL	9A	10	2018	Year-2	0	0	Fall
10/27/2018	BL	11A	10	2018	Year-2	0	0	Fall
10/27/2018	BL	13A	10	2018	Year-2	0	0	Fall
10/28/2018	BL	1M	10	2018	Year-2	0	0	Fall
10/28/2018	BL	2M	10	2018	Year-2		1	Fall
10/28/2018	BL	3M	10	2018	Year-2	0	0	Fall
10/28/2018	BL	4M	10	2018	Year-2		1	Fall
10/28/2018	BL	5M	10	2018	Year-2		1	Fall
10/28/2018	BL	6M	10	2018	Year-2		1	Fall
10/28/2018	BL	7M	10	2018	Year-2	0	0	Fall
10/28/2018	BL	10M	10	2018	Year-2		1	Fall
10/28/2018	BL	12M	10	2018	Year-2		1	Fall
10/28/2018	BL	14M	10	2018	Year-2	0	0	Fall
10/28/2018	BL	8A	10	2018	Year-2		1	Fall
10/28/2018	BL	9A	10	2018	Year-2	0	0	Fall
10/28/2018	BL	11A	10	2018	Year-2	0	0	Fall
10/28/2018	BL	13A	10	2018	Year-2	0	0	Fall
10/29/2018	BL	1M	10	2018	Year-2	0	0	Fall
10/29/2018	BL	2M	10	2018	Year-2		1	Fall
10/29/2018	BL	3M	10	2018	Year-2	0	0	Fall
10/29/2018	BL	4M	10	2018	Year-2		1	Fall
10/29/2018	BL	5M	10	2018	Year-2		1	Fall
10/29/2018	BL	6M	10	2018	Year-2		1	Fall
10/29/2018	BL	7M	10	2018	Year-2	0	0	Fall
10/29/2018	BL	10M	10	2018	Year-2		1	Fall
10/29/2018	BL	12M	10	2018	Year-2		1	Fall
10/29/2018	BL	14M	10	2018	Year-2	0	0	Fall
10/29/2018	BL	8A	10	2018	Year-2		1	Fall
10/29/2018	BL	9A	10	2018	Year-2	0	0	Fall
10/29/2018	BL	11A	10	2018	Year-2	0	0	Fall
10/29/2018	BL	13A	10	2018	Year-2	0	0	Fall
10/30/2018	BL	1M	10	2018	Year-2	0	0	Fall
10/30/2018	BL	2M	10	2018	Year-2		1	Fall
10/30/2018	BL	3M	10	2018	Year-2	0	0	Fall
10/30/2018	BL	4M	10	2018	Year-2		1	Fall
10/30/2018	BL	5M	10	2018	Year-2		1	Fall
10/30/2018	BL	6M	10	2018	Year-2		1	Fall
10/30/2018	BL	7M	10	2018	Year-2	0	0	Fall
10/30/2018	BL	10M	10	2018	Year-2		1	Fall
10/30/2018	BL	12M	10	2018	Year-2		1	Fall
10/30/2018	BL	14M	10	2018	Year-2	0	0	Fall
10/30/2018	BL	8A	10	2018	Year-2		1	Fall

Date	Species	Site	Month	Year	Survey Year	Presence	Data Gap	Season
10/30/2018	BL	9A	10	2018	Year-2	0	0	Fall
10/30/2018	BL	11A	10	2018	Year-2	0	0	Fall
10/30/2018	BL	13A	10	2018	Year-2	0	0	Fall
10/31/2018	BL	1M	10	2018	Year-2	0	0	Fall
10/31/2018	BL	2M	10	2018	Year-2		1	Fall
10/31/2018	BL	3M	10	2018	Year-2	0	0	Fall
10/31/2018	BL	4M	10	2018	Year-2		1	Fall
10/31/2018	BL	5M	10	2018	Year-2		1	Fall
10/31/2018	BL	6M	10	2018	Year-2		1	Fall
10/31/2018	BL	7M	10	2018	Year-2	0	0	Fall
10/31/2018	BL	10M	10	2018	Year-2		1	Fall
10/31/2018	BL	12M	10	2018	Year-2		1	Fall
10/31/2018	BL	14M	10	2018	Year-2	0	0	Fall
10/31/2018	BL	8A	10	2018	Year-2		1	Fall
10/31/2018	BL	9A	10	2018	Year-2	0	0	Fall
10/31/2018	BL	11A	10	2018	Year-2	0	0	Fall
10/31/2018	BL	13A	10	2018	Year-2	0	0	Fall
11/1/2018	BL	1M	11	2018	Year-2	0	0	Fall
11/1/2018	BL	2M	11	2018	Year-2		1	Fall
11/1/2018	BL	3M	11	2018	Year-2	0	0	Fall
11/1/2018	BL	4M	11	2018	Year-2		1	Fall
11/1/2018	BL	5M	11	2018	Year-2		1	Fall
11/1/2018	BL	6M	11	2018	Year-2		1	Fall
11/1/2018	BL	7M	11	2018	Year-2	0	0	Fall
11/1/2018	BL	10M	11	2018	Year-2		1	Fall
11/1/2018	BL	12M	11	2018	Year-2		1	Fall
11/1/2018	BL	14M	11	2018	Year-2	0	0	Fall
11/1/2018	BL	8A	11	2018	Year-2		1	Fall
11/1/2018	BL	9A	11	2018	Year-2	0	0	Fall
11/1/2018	BL	11A	11	2018	Year-2	0	0	Fall
11/1/2018	BL	13A	11	2018	Year-2	0	0	Fall
11/2/2018	BL	1M	11	2018	Year-2	0	0	Fall
11/2/2018	BL	2M	11	2018	Year-2		1	Fall
11/2/2018	BL	3M	11	2018	Year-2	0	0	Fall
11/2/2018	BL	4M	11	2018	Year-2		1	Fall
11/2/2018	BL	5M	11	2018	Year-2		1	Fall
11/2/2018	BL	6M	11	2018	Year-2		1	Fall
11/2/2018	BL	7M	11	2018	Year-2	0	0	Fall
11/2/2018	BL	10M	11	2018	Year-2		1	Fall
11/2/2018	BL	12M	11	2018	Year-2		1	Fall
11/2/2018	BL	14M	11	2018	Year-2	0	0	Fall
11/2/2018	BL	8A	11	2018	Year-2		1	Fall
11/2/2018	BL	9A	11	2018	Year-2	0	0	Fall
11/2/2018	BL	11A	11	2018	Year-2	0	0	Fall

Date	Species	Site	Month	Year	Survey Year	Presence	Data Gap	Season
11/2/2018	BL	13A	11	2018	Year-2	0	0	Fall
11/3/2018	BL	1M	11	2018	Year-2	0	0	Fall
11/3/2018	BL	2M	11	2018	Year-2		1	Fall
11/3/2018	BL	3M	11	2018	Year-2	0	0	Fall
11/3/2018	BL	4M	11	2018	Year-2		1	Fall
11/3/2018	BL	5M	11	2018	Year-2		1	Fall
11/3/2018	BL	6M	11	2018	Year-2		1	Fall
11/3/2018	BL	7M	11	2018	Year-2	0	0	Fall
11/3/2018	BL	10M	11	2018	Year-2		1	Fall
11/3/2018	BL	12M	11	2018	Year-2		1	Fall
11/3/2018	BL	14M	11	2018	Year-2	0	0	Fall
11/3/2018	BL	8A	11	2018	Year-2		1	Fall
11/3/2018	BL	9A	11	2018	Year-2	0	0	Fall
11/3/2018	BL	11A	11	2018	Year-2	0	0	Fall
11/3/2018	BL	13A	11	2018	Year-2	0	0	Fall
11/4/2018	BL	1M	11	2018	Year-2	0	0	Fall
11/4/2018	BL	2M	11	2018	Year-2		1	Fall
11/4/2018	BL	3M	11	2018	Year-2	0	0	Fall
11/4/2018	BL	4M	11	2018	Year-2		1	Fall
11/4/2018	BL	5M	11	2018	Year-2		1	Fall
11/4/2018	BL	6M	11	2018	Year-2		1	Fall
11/4/2018	BL	7M	11	2018	Year-2	0	0	Fall
11/4/2018	BL	10M	11	2018	Year-2		1	Fall
11/4/2018	BL	12M	11	2018	Year-2		1	Fall
11/4/2018	BL	14M	11	2018	Year-2	0	0	Fall
11/4/2018	BL	8A	11	2018	Year-2		1	Fall
11/4/2018	BL	9A	11	2018	Year-2	0	0	Fall
11/4/2018	BL	11A	11	2018	Year-2	0	0	Fall
11/4/2018	BL	13A	11	2018	Year-2	0	0	Fall
11/5/2018	BL	1M	11	2018	Year-2	0	0	Fall
11/5/2018	BL	2M	11	2018	Year-2		1	Fall
11/5/2018	BL	3M	11	2018	Year-2	0	0	Fall
11/5/2018	BL	4M	11	2018	Year-2		1	Fall
11/5/2018	BL	5M	11	2018	Year-2		1	Fall
11/5/2018	BL	6M	11	2018	Year-2		1	Fall
11/5/2018	BL	7M	11	2018	Year-2	0	0	Fall
11/5/2018	BL	10M	11	2018	Year-2		1	Fall
11/5/2018	BL	12M	11	2018	Year-2		1	Fall
11/5/2018	BL	14M	11	2018	Year-2	0	0	Fall
11/5/2018	BL	8A	11	2018	Year-2		1	Fall
11/5/2018	BL	9A	11	2018	Year-2	0	0	Fall
11/5/2018	BL	11A	11	2018	Year-2	0	0	Fall
11/5/2018	BL	13A	11	2018	Year-2	0	0	Fall
11/6/2018	BL	1M	11	2018	Year-2	0	0	Fall

Date	Species	Site	Month	Year	Survey Year	Presence	Data Gap	Season
11/6/2018	BL	2M	11	2018	Year-2		1	Fall
11/6/2018	BL	3M	11	2018	Year-2	0	0	Fall
11/6/2018	BL	4M	11	2018	Year-2		1	Fall
11/6/2018	BL	5M	11	2018	Year-2		1	Fall
11/6/2018	BL	6M	11	2018	Year-2		1	Fall
11/6/2018	BL	7M	11	2018	Year-2	0	0	Fall
11/6/2018	BL	10M	11	2018	Year-2		1	Fall
11/6/2018	BL	12M	11	2018	Year-2		1	Fall
11/6/2018	BL	14M	11	2018	Year-2	0	0	Fall
11/6/2018	BL	8A	11	2018	Year-2		1	Fall
11/6/2018	BL	9A	11	2018	Year-2	0	0	Fall
11/6/2018	BL	11A	11	2018	Year-2	0	0	Fall
11/6/2018	BL	13A	11	2018	Year-2	0	0	Fall
11/7/2018	BL	1M	11	2018	Year-2	0	0	Fall
11/7/2018	BL	2M	11	2018	Year-2		1	Fall
11/7/2018	BL	3M	11	2018	Year-2	0	0	Fall
11/7/2018	BL	4M	11	2018	Year-2		1	Fall
11/7/2018	BL	5M	11	2018	Year-2		1	Fall
11/7/2018	BL	6M	11	2018	Year-2		1	Fall
11/7/2018	BL	7M	11	2018	Year-2	0	0	Fall
11/7/2018	BL	10M	11	2018	Year-2		1	Fall
11/7/2018	BL	12M	11	2018	Year-2		1	Fall
11/7/2018	BL	14M	11	2018	Year-2	0	0	Fall
11/7/2018	BL	8A	11	2018	Year-2		1	Fall
11/7/2018	BL	9A	11	2018	Year-2	0	0	Fall
11/7/2018	BL	11A	11	2018	Year-2	0	0	Fall
11/7/2018	BL	13A	11	2018	Year-2	0	0	Fall
11/8/2018	BL	1M	11	2018	Year-2	0	0	Fall
11/8/2018	BL	2M	11	2018	Year-2		1	Fall
11/8/2018	BL	3M	11	2018	Year-2	0	0	Fall
11/8/2018	BL	4M	11	2018	Year-2		1	Fall
11/8/2018	BL	5M	11	2018	Year-2		1	Fall
11/8/2018	BL	6M	11	2018	Year-2		1	Fall
11/8/2018	BL	7M	11	2018	Year-2	0	0	Fall
11/8/2018	BL	10M	11	2018	Year-2		1	Fall
11/8/2018	BL	12M	11	2018	Year-2		1	Fall
11/8/2018	BL	14M	11	2018	Year-2	0	0	Fall
11/8/2018	BL	8A	11	2018	Year-2		1	Fall
11/8/2018	BL	9A	11	2018	Year-2	0	0	Fall
11/8/2018	BL	11A	11	2018	Year-2	0	0	Fall
11/8/2018	BL	13A	11	2018	Year-2	0	0	Fall
11/9/2018	BL	1M	11	2018	Year-2	0	0	Fall
11/9/2018	BL	2M	11	2018	Year-2		1	Fall
11/9/2018	BL	3M	11	2018	Year-2	0	0	Fall

Date	Species	Site	Month	Year	Survey Year	Presence	Data Gap	Season
11/9/2018	BL	4M	11	2018	Year-2		1	Fall
11/9/2018	BL	5M	11	2018	Year-2		1	Fall
11/9/2018	BL	6M	11	2018	Year-2		1	Fall
11/9/2018	BL	7M	11	2018	Year-2	0	0	Fall
11/9/2018	BL	10M	11	2018	Year-2		1	Fall
11/9/2018	BL	12M	11	2018	Year-2		1	Fall
11/9/2018	BL	14M	11	2018	Year-2	0	0	Fall
11/9/2018	BL	8A	11	2018	Year-2		1	Fall
11/9/2018	BL	9A	11	2018	Year-2	0	0	Fall
11/9/2018	BL	11A	11	2018	Year-2	0	0	Fall
11/9/2018	BL	13A	11	2018	Year-2	0	0	Fall
11/10/2018	BL	1M	11	2018	Year-2	0	0	Fall
11/10/2018	BL	2M	11	2018	Year-2		1	Fall
11/10/2018	BL	3M	11	2018	Year-2	0	0	Fall
11/10/2018	BL	4M	11	2018	Year-2		1	Fall
11/10/2018	BL	5M	11	2018	Year-2		1	Fall
11/10/2018	BL	6M	11	2018	Year-2		1	Fall
11/10/2018	BL	7M	11	2018	Year-2	0	0	Fall
11/10/2018	BL	10M	11	2018	Year-2		1	Fall
11/10/2018	BL	12M	11	2018	Year-2		1	Fall
11/10/2018	BL	14M	11	2018	Year-2	0	0	Fall
11/10/2018	BL	8A	11	2018	Year-2		1	Fall
11/10/2018	BL	9A	11	2018	Year-2	0	0	Fall
11/10/2018	BL	11A	11	2018	Year-2	0	0	Fall
11/10/2018	BL	13A	11	2018	Year-2	0	0	Fall
11/11/2018	BL	1M	11	2018	Year-2	0	0	Fall
11/11/2018	BL	2M	11	2018	Year-2		1	Fall
11/11/2018	BL	3M	11	2018	Year-2	0	0	Fall
11/11/2018	BL	4M	11	2018	Year-2		1	Fall
11/11/2018	BL	5M	11	2018	Year-2		1	Fall
11/11/2018	BL	6M	11	2018	Year-2		1	Fall
11/11/2018	BL	7M	11	2018	Year-2	0	0	Fall
11/11/2018	BL	10M	11	2018	Year-2		1	Fall
11/11/2018	BL	12M	11	2018	Year-2		1	Fall
11/11/2018	BL	14M	11	2018	Year-2	0	0	Fall
11/11/2018	BL	8A	11	2018	Year-2		1	Fall
11/11/2018	BL	9A	11	2018	Year-2	0	0	Fall
11/11/2018	BL	11A	11	2018	Year-2	0	0	Fall
11/11/2018	BL	13A	11	2018	Year-2	0	0	Fall
11/12/2018	BL	1M	11	2018	Year-2	0	0	Fall
11/12/2018	BL	2M	11	2018	Year-2		1	Fall
11/12/2018	BL	3M	11	2018	Year-2	0	0	Fall
11/12/2018	BL	4M	11	2018	Year-2		1	Fall
11/12/2018	BL	5M	11	2018	Year-2		1	Fall

Date	Species	Site	Month	Year	Survey Year	Presence	Data Gap	Season
11/12/2018	BL	6M	11	2018	Year-2		1	Fall
11/12/2018	BL	7M	11	2018	Year-2	0	0	Fall
11/12/2018	BL	10M	11	2018	Year-2		1	Fall
11/12/2018	BL	12M	11	2018	Year-2		1	Fall
11/12/2018	BL	14M	11	2018	Year-2	0	0	Fall
11/12/2018	BL	8A	11	2018	Year-2		1	Fall
11/12/2018	BL	9A	11	2018	Year-2	0	0	Fall
11/12/2018	BL	11A	11	2018	Year-2	0	0	Fall
11/12/2018	BL	13A	11	2018	Year-2	0	0	Fall
11/13/2018	BL	1M	11	2018	Year-2	0	0	Fall
11/13/2018	BL	2M	11	2018	Year-2		1	Fall
11/13/2018	BL	3M	11	2018	Year-2	0	0	Fall
11/13/2018	BL	4M	11	2018	Year-2		1	Fall
11/13/2018	BL	5M	11	2018	Year-2		1	Fall
11/13/2018	BL	6M	11	2018	Year-2		1	Fall
11/13/2018	BL	7M	11	2018	Year-2	0	0	Fall
11/13/2018	BL	10M	11	2018	Year-2		1	Fall
11/13/2018	BL	12M	11	2018	Year-2		1	Fall
11/13/2018	BL	14M	11	2018	Year-2	0	0	Fall
11/13/2018	BL	8A	11	2018	Year-2		1	Fall
11/13/2018	BL	9A	11	2018	Year-2	0	0	Fall
11/13/2018	BL	11A	11	2018	Year-2	0	0	Fall
11/13/2018	BL	13A	11	2018	Year-2	0	0	Fall
11/14/2018	BL	1M	11	2018	Year-2	0	0	Fall
11/14/2018	BL	2M	11	2018	Year-2		1	Fall
11/14/2018	BL	3M	11	2018	Year-2	0	0	Fall
11/14/2018	BL	4M	11	2018	Year-2		1	Fall
11/14/2018	BL	5M	11	2018	Year-2		1	Fall
11/14/2018	BL	6M	11	2018	Year-2		1	Fall
11/14/2018	BL	7M	11	2018	Year-2	0	0	Fall
11/14/2018	BL	10M	11	2018	Year-2		1	Fall
11/14/2018	BL	12M	11	2018	Year-2		1	Fall
11/14/2018	BL	14M	11	2018	Year-2	0	0	Fall
11/14/2018	BL	8A	11	2018	Year-2		1	Fall
11/14/2018	BL	9A	11	2018	Year-2	0	0	Fall
11/14/2018	BL	11A	11	2018	Year-2	0	0	Fall
11/14/2018	BL	13A	11	2018	Year-2	0	0	Fall
11/15/2018	BL	1M	11	2018	Year-2	0	0	Fall
11/15/2018	BL	2M	11	2018	Year-2		1	Fall
11/15/2018	BL	3M	11	2018	Year-2	0	0	Fall
11/15/2018	BL	4M	11	2018	Year-2		1	Fall
11/15/2018	BL	5M	11	2018	Year-2		1	Fall
11/15/2018	BL	6M	11	2018	Year-2		1	Fall
11/15/2018	BL	7M	11	2018	Year-2	0	0	Fall

Date	Species	Site	Month	Year	Survey Year	Presence	Data Gap	Season
11/15/2018	BL	10M	11	2018	Year-2		1	Fall
11/15/2018	BL	12M	11	2018	Year-2		1	Fall
11/15/2018	BL	14M	11	2018	Year-2	0	0	Fall
11/15/2018	BL	8A	11	2018	Year-2		1	Fall
11/15/2018	BL	9A	11	2018	Year-2	0	0	Fall
11/15/2018	BL	11A	11	2018	Year-2	0	0	Fall
11/15/2018	BL	13A	11	2018	Year-2	0	0	Fall
11/16/2018	BL	1M	11	2018	Year-2	0	0	Fall
11/16/2018	BL	2M	11	2018	Year-2		1	Fall
11/16/2018	BL	3M	11	2018	Year-2	0	0	Fall
11/16/2018	BL	4M	11	2018	Year-2		1	Fall
11/16/2018	BL	5M	11	2018	Year-2		1	Fall
11/16/2018	BL	6M	11	2018	Year-2		1	Fall
11/16/2018	BL	7M	11	2018	Year-2	0	0	Fall
11/16/2018	BL	10M	11	2018	Year-2		1	Fall
11/16/2018	BL	12M	11	2018	Year-2		1	Fall
11/16/2018	BL	14M	11	2018	Year-2	0	0	Fall
11/16/2018	BL	8A	11	2018	Year-2		1	Fall
11/16/2018	BL	9A	11	2018	Year-2	0	0	Fall
11/16/2018	BL	11A	11	2018	Year-2	0	0	Fall
11/16/2018	BL	13A	11	2018	Year-2	0	0	Fall
11/17/2018	BL	1M	11	2018	Year-2	0	0	Fall
11/17/2018	BL	2M	11	2018	Year-2		1	Fall
11/17/2018	BL	3M	11	2018	Year-2	0	0	Fall
11/17/2018	BL	4M	11	2018	Year-2		1	Fall
11/17/2018	BL	5M	11	2018	Year-2		1	Fall
11/17/2018	BL	6M	11	2018	Year-2		1	Fall
11/17/2018	BL	7M	11	2018	Year-2	0	0	Fall
11/17/2018	BL	10M	11	2018	Year-2		1	Fall
11/17/2018	BL	12M	11	2018	Year-2		1	Fall
11/17/2018	BL	14M	11	2018	Year-2	0	0	Fall
11/17/2018	BL	8A	11	2018	Year-2		1	Fall
11/17/2018	BL	9A	11	2018	Year-2	0	0	Fall
11/17/2018	BL	11A	11	2018	Year-2	0	0	Fall
11/17/2018	BL	13A	11	2018	Year-2	0	0	Fall
11/18/2018	BL	1M	11	2018	Year-2	0	0	Fall
11/18/2018	BL	2M	11	2018	Year-2		1	Fall
11/18/2018	BL	3M	11	2018	Year-2	0	0	Fall
11/18/2018	BL	4M	11	2018	Year-2		1	Fall
11/18/2018	BL	5M	11	2018	Year-2		1	Fall
11/18/2018	BL	6M	11	2018	Year-2		1	Fall
11/18/2018	BL	7M	11	2018	Year-2	0	0	Fall
11/18/2018	BL	10M	11	2018	Year-2		1	Fall
11/18/2018	BL	12M	11	2018	Year-2		1	Fall

Date	Species	Site	Month	Year	Survey Year	Presence	Data Gap	Season
11/18/2018	BL	14M	11	2018	Year-2	0	0	Fall
11/18/2018	BL	8A	11	2018	Year-2		1	Fall
11/18/2018	BL	9A	11	2018	Year-2	0	0	Fall
11/18/2018	BL	11A	11	2018	Year-2	0	0	Fall
11/18/2018	BL	13A	11	2018	Year-2	0	0	Fall
11/19/2018	BL	1M	11	2018	Year-2	0	0	Fall
11/19/2018	BL	2M	11	2018	Year-2		1	Fall
11/19/2018	BL	3M	11	2018	Year-2	0	0	Fall
11/19/2018	BL	4M	11	2018	Year-2		1	Fall
11/19/2018	BL	5M	11	2018	Year-2		1	Fall
11/19/2018	BL	6M	11	2018	Year-2		1	Fall
11/19/2018	BL	7M	11	2018	Year-2	0	0	Fall
11/19/2018	BL	10M	11	2018	Year-2		1	Fall
11/19/2018	BL	12M	11	2018	Year-2		1	Fall
11/19/2018	BL	14M	11	2018	Year-2	0	0	Fall
11/19/2018	BL	8A	11	2018	Year-2		1	Fall
11/19/2018	BL	9A	11	2018	Year-2	0	0	Fall
11/19/2018	BL	11A	11	2018	Year-2	0	0	Fall
11/19/2018	BL	13A	11	2018	Year-2	0	0	Fall
11/20/2018	BL	1M	11	2018	Year-2	0	0	Fall
11/20/2018	BL	2M	11	2018	Year-2		1	Fall
11/20/2018	BL	3M	11	2018	Year-2	0	0	Fall
11/20/2018	BL	4M	11	2018	Year-2		1	Fall
11/20/2018	BL	5M	11	2018	Year-2		1	Fall
11/20/2018	BL	6M	11	2018	Year-2		1	Fall
11/20/2018	BL	7M	11	2018	Year-2	0	0	Fall
11/20/2018	BL	10M	11	2018	Year-2		1	Fall
11/20/2018	BL	12M	11	2018	Year-2		1	Fall
11/20/2018	BL	14M	11	2018	Year-2	0	0	Fall
11/20/2018	BL	8A	11	2018	Year-2		1	Fall
11/20/2018	BL	9A	11	2018	Year-2	0	0	Fall
11/20/2018	BL	11A	11	2018	Year-2	0	0	Fall
11/20/2018	BL	13A	11	2018	Year-2	0	0	Fall
11/21/2018	BL	1M	11	2018	Year-2	0	0	Fall
11/21/2018	BL	2M	11	2018	Year-2		1	Fall
11/21/2018	BL	3M	11	2018	Year-2	0	0	Fall
11/21/2018	BL	4M	11	2018	Year-2		1	Fall
11/21/2018	BL	5M	11	2018	Year-2		1	Fall
11/21/2018	BL	6M	11	2018	Year-2		1	Fall
11/21/2018	BL	7M	11	2018	Year-2	0	0	Fall
11/21/2018	BL	10M	11	2018	Year-2		1	Fall
11/21/2018	BL	12M	11	2018	Year-2		1	Fall
11/21/2018	BL	14M	11	2018	Year-2	0	0	Fall
11/21/2018	BL	8A	11	2018	Year-2		1	Fall

Date	Species	Site	Month	Year	Survey Year	Presence	Data Gap	Season
11/21/2018	BL	9A	11	2018	Year-2	0	0	Fall
11/21/2018	BL	11A	11	2018	Year-2	0	0	Fall
11/21/2018	BL	13A	11	2018	Year-2	0	0	Fall
11/22/2018	BL	1M	11	2018	Year-2	0	0	Fall
11/22/2018	BL	2M	11	2018	Year-2		1	Fall
11/22/2018	BL	3M	11	2018	Year-2	0	0	Fall
11/22/2018	BL	4M	11	2018	Year-2		1	Fall
11/22/2018	BL	5M	11	2018	Year-2		1	Fall
11/22/2018	BL	6M	11	2018	Year-2		1	Fall
11/22/2018	BL	7M	11	2018	Year-2	0	0	Fall
11/22/2018	BL	10M	11	2018	Year-2		1	Fall
11/22/2018	BL	12M	11	2018	Year-2		1	Fall
11/22/2018	BL	14M	11	2018	Year-2	0	0	Fall
11/22/2018	BL	8A	11	2018	Year-2		1	Fall
11/22/2018	BL	9A	11	2018	Year-2	0	0	Fall
11/22/2018	BL	11A	11	2018	Year-2	0	0	Fall
11/22/2018	BL	13A	11	2018	Year-2	0	0	Fall
11/23/2018	BL	1M	11	2018	Year-2	0	0	Fall
11/23/2018	BL	2M	11	2018	Year-2		1	Fall
11/23/2018	BL	3M	11	2018	Year-2	0	0	Fall
11/23/2018	BL	4M	11	2018	Year-2		1	Fall
11/23/2018	BL	5M	11	2018	Year-2		1	Fall
11/23/2018	BL	6M	11	2018	Year-2		1	Fall
11/23/2018	BL	7M	11	2018	Year-2	0	0	Fall
11/23/2018	BL	10M	11	2018	Year-2		1	Fall
11/23/2018	BL	12M	11	2018	Year-2		1	Fall
11/23/2018	BL	14M	11	2018	Year-2	0	0	Fall
11/23/2018	BL	8A	11	2018	Year-2		1	Fall
11/23/2018	BL	9A	11	2018	Year-2	0	0	Fall
11/23/2018	BL	11A	11	2018	Year-2	0	0	Fall
11/23/2018	BL	13A	11	2018	Year-2	0	0	Fall
11/24/2018	BL	1M	11	2018	Year-2	0	0	Fall
11/24/2018	BL	2M	11	2018	Year-2		1	Fall
11/24/2018	BL	3M	11	2018	Year-2	0	0	Fall
11/24/2018	BL	4M	11	2018	Year-2		1	Fall
11/24/2018	BL	5M	11	2018	Year-2		1	Fall
11/24/2018	BL	6M	11	2018	Year-2		1	Fall
11/24/2018	BL	7M	11	2018	Year-2	0	0	Fall
11/24/2018	BL	10M	11	2018	Year-2		1	Fall
11/24/2018	BL	12M	11	2018	Year-2		1	Fall
11/24/2018	BL	14M	11	2018	Year-2	0	0	Fall
11/24/2018	BL	8A	11	2018	Year-2		1	Fall
11/24/2018	BL	9A	11	2018	Year-2	0	0	Fall
11/24/2018	BL	11A	11	2018	Year-2	0	0	Fall

Date	Species	Site	Month	Year	Survey Year	Presence	Data Gap	Season
11/24/2018	BL	13A	11	2018	Year-2	0	0	Fall
11/25/2018	BL	1M	11	2018	Year-2	0	0	Fall
11/25/2018	BL	2M	11	2018	Year-2		1	Fall
11/25/2018	BL	3M	11	2018	Year-2	0	0	Fall
11/25/2018	BL	4M	11	2018	Year-2		1	Fall
11/25/2018	BL	5M	11	2018	Year-2		1	Fall
11/25/2018	BL	6M	11	2018	Year-2		1	Fall
11/25/2018	BL	7M	11	2018	Year-2	0	0	Fall
11/25/2018	BL	10M	11	2018	Year-2		1	Fall
11/25/2018	BL	12M	11	2018	Year-2		1	Fall
11/25/2018	BL	14M	11	2018	Year-2	0	0	Fall
11/25/2018	BL	8A	11	2018	Year-2		1	Fall
11/25/2018	BL	9A	11	2018	Year-2	0	0	Fall
11/25/2018	BL	11A	11	2018	Year-2	0	0	Fall
11/25/2018	BL	13A	11	2018	Year-2	0	0	Fall
11/26/2018	BL	1M	11	2018	Year-2	0	0	Fall
11/26/2018	BL	2M	11	2018	Year-2		1	Fall
11/26/2018	BL	3M	11	2018	Year-2	0	0	Fall
11/26/2018	BL	4M	11	2018	Year-2		1	Fall
11/26/2018	BL	5M	11	2018	Year-2		1	Fall
11/26/2018	BL	6M	11	2018	Year-2		1	Fall
11/26/2018	BL	7M	11	2018	Year-2	0	0	Fall
11/26/2018	BL	10M	11	2018	Year-2		1	Fall
11/26/2018	BL	12M	11	2018	Year-2		1	Fall
11/26/2018	BL	14M	11	2018	Year-2	0	0	Fall
11/26/2018	BL	8A	11	2018	Year-2		1	Fall
11/26/2018	BL	9A	11	2018	Year-2	0	0	Fall
11/26/2018	BL	11A	11	2018	Year-2	0	0	Fall
11/26/2018	BL	13A	11	2018	Year-2	0	0	Fall
11/27/2018	BL	1M	11	2018	Year-2	0	0	Fall
11/27/2018	BL	2M	11	2018	Year-2		1	Fall
11/27/2018	BL	3M	11	2018	Year-2	0	0	Fall
11/27/2018	BL	4M	11	2018	Year-2		1	Fall
11/27/2018	BL	5M	11	2018	Year-2		1	Fall
11/27/2018	BL	6M	11	2018	Year-2		1	Fall
11/27/2018	BL	7M	11	2018	Year-2	0	0	Fall
11/27/2018	BL	10M	11	2018	Year-2		1	Fall
11/27/2018	BL	12M	11	2018	Year-2		1	Fall
11/27/2018	BL	14M	11	2018	Year-2	0	0	Fall
11/27/2018	BL	8A	11	2018	Year-2		1	Fall
11/27/2018	BL	9A	11	2018	Year-2	0	0	Fall
11/27/2018	BL	11A	11	2018	Year-2	0	0	Fall
11/27/2018	BL	13A	11	2018	Year-2	0	0	Fall
11/28/2018	BL	1M	11	2018	Year-2	0	0	Fall

Date	Species	Site	Month	Year	Survey Year	Presence	Data Gap	Season
11/28/2018	BL	2M	11	2018	Year-2		1	Fall
11/28/2018	BL	3M	11	2018	Year-2	0	0	Fall
11/28/2018	BL	4M	11	2018	Year-2		1	Fall
11/28/2018	BL	5M	11	2018	Year-2		1	Fall
11/28/2018	BL	6M	11	2018	Year-2		1	Fall
11/28/2018	BL	7M	11	2018	Year-2	0	0	Fall
11/28/2018	BL	10M	11	2018	Year-2		1	Fall
11/28/2018	BL	12M	11	2018	Year-2		1	Fall
11/28/2018	BL	14M	11	2018	Year-2	0	0	Fall
11/28/2018	BL	8A	11	2018	Year-2		1	Fall
11/28/2018	BL	9A	11	2018	Year-2	0	0	Fall
11/28/2018	BL	11A	11	2018	Year-2	0	0	Fall
11/28/2018	BL	13A	11	2018	Year-2	0	0	Fall
11/29/2018	BL	1M	11	2018	Year-2	0	0	Fall
11/29/2018	BL	2M	11	2018	Year-2		1	Fall
11/29/2018	BL	3M	11	2018	Year-2	0	0	Fall
11/29/2018	BL	4M	11	2018	Year-2		1	Fall
11/29/2018	BL	5M	11	2018	Year-2		1	Fall
11/29/2018	BL	6M	11	2018	Year-2		1	Fall
11/29/2018	BL	7M	11	2018	Year-2	0	0	Fall
11/29/2018	BL	10M	11	2018	Year-2		1	Fall
11/29/2018	BL	12M	11	2018	Year-2		1	Fall
11/29/2018	BL	14M	11	2018	Year-2	0	0	Fall
11/29/2018	BL	8A	11	2018	Year-2		1	Fall
11/29/2018	BL	9A	11	2018	Year-2	0	0	Fall
11/29/2018	BL	11A	11	2018	Year-2	0	0	Fall
11/29/2018	BL	13A	11	2018	Year-2	0	0	Fall
11/30/2018	BL	1M	11	2018	Year-2	0	0	Fall
11/30/2018	BL	2M	11	2018	Year-2		1	Fall
11/30/2018	BL	3M	11	2018	Year-2	0	0	Fall
11/30/2018	BL	4M	11	2018	Year-2		1	Fall
11/30/2018	BL	5M	11	2018	Year-2		1	Fall
11/30/2018	BL	6M	11	2018	Year-2		1	Fall
11/30/2018	BL	7M	11	2018	Year-2	0	0	Fall
11/30/2018	BL	10M	11	2018	Year-2		1	Fall
11/30/2018	BL	12M	11	2018	Year-2		1	Fall
11/30/2018	BL	14M	11	2018	Year-2	0	0	Fall
11/30/2018	BL	8A	11	2018	Year-2		1	Fall
11/30/2018	BL	9A	11	2018	Year-2	0	0	Fall
11/30/2018	BL	11A	11	2018	Year-2	0	0	Fall
11/30/2018	BL	13A	11	2018	Year-2	0	0	Fall
12/1/2018	BL	1M	12	2018	Year-2	0	0	Winter
12/1/2018	BL	2M	12	2018	Year-2		1	Winter
12/1/2018	BL	3M	12	2018	Year-2	0	0	Winter

Date	Species	Site	Month	Year	Survey Year	Presence	Data Gap	Season
12/1/2018	BL	4M	12	2018	Year-2		1	Winter
12/1/2018	BL	5M	12	2018	Year-2		1	Winter
12/1/2018	BL	6M	12	2018	Year-2		1	Winter
12/1/2018	BL	7M	12	2018	Year-2	0	0	Winter
12/1/2018	BL	10M	12	2018	Year-2		1	Winter
12/1/2018	BL	12M	12	2018	Year-2		1	Winter
12/1/2018	BL	14M	12	2018	Year-2	0	0	Winter
12/1/2018	BL	8A	12	2018	Year-2		1	Winter
12/1/2018	BL	9A	12	2018	Year-2	0	0	Winter
12/1/2018	BL	11A	12	2018	Year-2	0	0	Winter
12/1/2018	BL	13A	12	2018	Year-2	0	0	Winter
12/2/2018	BL	1M	12	2018	Year-2	0	0	Winter
12/2/2018	BL	2M	12	2018	Year-2		1	Winter
12/2/2018	BL	3M	12	2018	Year-2	0	0	Winter
12/2/2018	BL	4M	12	2018	Year-2		1	Winter
12/2/2018	BL	5M	12	2018	Year-2		1	Winter
12/2/2018	BL	6M	12	2018	Year-2		1	Winter
12/2/2018	BL	7M	12	2018	Year-2	0	0	Winter
12/2/2018	BL	10M	12	2018	Year-2		1	Winter
12/2/2018	BL	12M	12	2018	Year-2		1	Winter
12/2/2018	BL	14M	12	2018	Year-2	0	0	Winter
12/2/2018	BL	8A	12	2018	Year-2		1	Winter
12/2/2018	BL	9A	12	2018	Year-2	0	0	Winter
12/2/2018	BL	11A	12	2018	Year-2	0	0	Winter
12/2/2018	BL	13A	12	2018	Year-2	0	0	Winter
12/3/2018	BL	1M	12	2018	Year-2	0	0	Winter
12/3/2018	BL	2M	12	2018	Year-2		1	Winter
12/3/2018	BL	3M	12	2018	Year-2	0	0	Winter
12/3/2018	BL	4M	12	2018	Year-2		1	Winter
12/3/2018	BL	5M	12	2018	Year-2		1	Winter
12/3/2018	BL	6M	12	2018	Year-2		1	Winter
12/3/2018	BL	7M	12	2018	Year-2	0	0	Winter
12/3/2018	BL	10M	12	2018	Year-2		1	Winter
12/3/2018	BL	12M	12	2018	Year-2		1	Winter
12/3/2018	BL	14M	12	2018	Year-2	0	0	Winter
12/3/2018	BL	8A	12	2018	Year-2		1	Winter
12/3/2018	BL	9A	12	2018	Year-2	0	0	Winter
12/3/2018	BL	11A	12	2018	Year-2	0	0	Winter
12/3/2018	BL	13A	12	2018	Year-2	0	0	Winter
12/4/2018	BL	1M	12	2018	Year-2	0	0	Winter
12/4/2018	BL	2M	12	2018	Year-2		1	Winter
12/4/2018	BL	3M	12	2018	Year-2	0	0	Winter
12/4/2018	BL	4M	12	2018	Year-2		1	Winter
12/4/2018	BL	5M	12	2018	Year-2		1	Winter

Date	Species	Site	Month	Year	Survey Year	Presence	Data Gap	Season
12/4/2018	BL	6M	12	2018	Year-2		1	Winter
12/4/2018	BL	7M	12	2018	Year-2	0	0	Winter
12/4/2018	BL	10M	12	2018	Year-2		1	Winter
12/4/2018	BL	12M	12	2018	Year-2		1	Winter
12/4/2018	BL	14M	12	2018	Year-2	0	0	Winter
12/4/2018	BL	8A	12	2018	Year-2		1	Winter
12/4/2018	BL	9A	12	2018	Year-2	0	0	Winter
12/4/2018	BL	11A	12	2018	Year-2	0	0	Winter
12/4/2018	BL	13A	12	2018	Year-2	0	0	Winter
12/5/2018	BL	1M	12	2018	Year-2	0	0	Winter
12/5/2018	BL	2M	12	2018	Year-2		1	Winter
12/5/2018	BL	3M	12	2018	Year-2	0	0	Winter
12/5/2018	BL	4M	12	2018	Year-2		1	Winter
12/5/2018	BL	5M	12	2018	Year-2		1	Winter
12/5/2018	BL	6M	12	2018	Year-2		1	Winter
12/5/2018	BL	7M	12	2018	Year-2	0	0	Winter
12/5/2018	BL	10M	12	2018	Year-2		1	Winter
12/5/2018	BL	12M	12	2018	Year-2		1	Winter
12/5/2018	BL	14M	12	2018	Year-2	0	0	Winter
12/5/2018	BL	8A	12	2018	Year-2		1	Winter
12/5/2018	BL	9A	12	2018	Year-2	0	0	Winter
12/5/2018	BL	11A	12	2018	Year-2	0	0	Winter
12/5/2018	BL	13A	12	2018	Year-2	0	0	Winter
12/6/2018	BL	1M	12	2018	Year-2	0	0	Winter
12/6/2018	BL	2M	12	2018	Year-2		1	Winter
12/6/2018	BL	3M	12	2018	Year-2	0	0	Winter
12/6/2018	BL	4M	12	2018	Year-2		1	Winter
12/6/2018	BL	5M	12	2018	Year-2		1	Winter
12/6/2018	BL	6M	12	2018	Year-2		1	Winter
12/6/2018	BL	7M	12	2018	Year-2	0	0	Winter
12/6/2018	BL	10M	12	2018	Year-2		1	Winter
12/6/2018	BL	12M	12	2018	Year-2		1	Winter
12/6/2018	BL	14M	12	2018	Year-2	0	0	Winter
12/6/2018	BL	8A	12	2018	Year-2		1	Winter
12/6/2018	BL	9A	12	2018	Year-2	0	0	Winter
12/6/2018	BL	11A	12	2018	Year-2	0	0	Winter
12/6/2018	BL	13A	12	2018	Year-2	0	0	Winter
12/7/2018	BL	1M	12	2018	Year-2	0	0	Winter
12/7/2018	BL	2M	12	2018	Year-2		1	Winter
12/7/2018	BL	3M	12	2018	Year-2	0	0	Winter
12/7/2018	BL	4M	12	2018	Year-2		1	Winter
12/7/2018	BL	5M	12	2018	Year-2		1	Winter
12/7/2018	BL	6M	12	2018	Year-2		1	Winter
12/7/2018	BL	7M	12	2018	Year-2	0	0	Winter

Date	Species	Site	Month	Year	Survey Year	Presence	Data Gap	Season
12/7/2018	BL	10M	12	2018	Year-2		1	Winter
12/7/2018	BL	12M	12	2018	Year-2		1	Winter
12/7/2018	BL	14M	12	2018	Year-2	0	0	Winter
12/7/2018	BL	8A	12	2018	Year-2		1	Winter
12/7/2018	BL	9A	12	2018	Year-2	0	0	Winter
12/7/2018	BL	11A	12	2018	Year-2	0	0	Winter
12/7/2018	BL	13A	12	2018	Year-2	0	0	Winter
12/8/2018	BL	1M	12	2018	Year-2	0	0	Winter
12/8/2018	BL	2M	12	2018	Year-2		1	Winter
12/8/2018	BL	3M	12	2018	Year-2	0	0	Winter
12/8/2018	BL	4M	12	2018	Year-2		1	Winter
12/8/2018	BL	5M	12	2018	Year-2		1	Winter
12/8/2018	BL	6M	12	2018	Year-2		1	Winter
12/8/2018	BL	7M	12	2018	Year-2	0	0	Winter
12/8/2018	BL	10M	12	2018	Year-2		1	Winter
12/8/2018	BL	12M	12	2018	Year-2		1	Winter
12/8/2018	BL	14M	12	2018	Year-2	0	0	Winter
12/8/2018	BL	8A	12	2018	Year-2		1	Winter
12/8/2018	BL	9A	12	2018	Year-2	0	0	Winter
12/8/2018	BL	11A	12	2018	Year-2	0	0	Winter
12/8/2018	BL	13A	12	2018	Year-2	0	0	Winter
12/9/2018	BL	1M	12	2018	Year-2	0	0	Winter
12/9/2018	BL	2M	12	2018	Year-2		1	Winter
12/9/2018	BL	3M	12	2018	Year-2	0	0	Winter
12/9/2018	BL	4M	12	2018	Year-2		1	Winter
12/9/2018	BL	5M	12	2018	Year-2		1	Winter
12/9/2018	BL	6M	12	2018	Year-2		1	Winter
12/9/2018	BL	7M	12	2018	Year-2	0	0	Winter
12/9/2018	BL	10M	12	2018	Year-2		1	Winter
12/9/2018	BL	12M	12	2018	Year-2		1	Winter
12/9/2018	BL	14M	12	2018	Year-2	0	0	Winter
12/9/2018	BL	8A	12	2018	Year-2		1	Winter
12/9/2018	BL	9A	12	2018	Year-2	0	0	Winter
12/9/2018	BL	11A	12	2018	Year-2	0	0	Winter
12/9/2018	BL	13A	12	2018	Year-2	0	0	Winter
12/10/2018	BL	1M	12	2018	Year-2	0	0	Winter
12/10/2018	BL	2M	12	2018	Year-2		1	Winter
12/10/2018	BL	3M	12	2018	Year-2	0	0	Winter
12/10/2018	BL	4M	12	2018	Year-2		1	Winter
12/10/2018	BL	5M	12	2018	Year-2		1	Winter
12/10/2018	BL	6M	12	2018	Year-2		1	Winter
12/10/2018	BL	7M	12	2018	Year-2	0	0	Winter
12/10/2018	BL	10M	12	2018	Year-2		1	Winter
12/10/2018	BL	12M	12	2018	Year-2		1	Winter

Date	Species	Site	Month	Year	Survey Year	Presence	Data Gap	Season
12/10/2018	BL	14M	12	2018	Year-2	0	0	Winter
12/10/2018	BL	8A	12	2018	Year-2		1	Winter
12/10/2018	BL	9A	12	2018	Year-2	0	0	Winter
12/10/2018	BL	11A	12	2018	Year-2	0	0	Winter
12/10/2018	BL	13A	12	2018	Year-2	0	0	Winter
12/11/2018	BL	1M	12	2018	Year-2	0	0	Winter
12/11/2018	BL	2M	12	2018	Year-2		1	Winter
12/11/2018	BL	3M	12	2018	Year-2	0	0	Winter
12/11/2018	BL	4M	12	2018	Year-2		1	Winter
12/11/2018	BL	5M	12	2018	Year-2		1	Winter
12/11/2018	BL	6M	12	2018	Year-2		1	Winter
12/11/2018	BL	7M	12	2018	Year-2	0	0	Winter
12/11/2018	BL	10M	12	2018	Year-2		1	Winter
12/11/2018	BL	12M	12	2018	Year-2		1	Winter
12/11/2018	BL	14M	12	2018	Year-2	0	0	Winter
12/11/2018	BL	8A	12	2018	Year-2		1	Winter
12/11/2018	BL	9A	12	2018	Year-2	0	0	Winter
12/11/2018	BL	11A	12	2018	Year-2	0	0	Winter
12/11/2018	BL	13A	12	2018	Year-2	0	0	Winter
12/12/2018	BL	1M	12	2018	Year-2	0	0	Winter
12/12/2018	BL	2M	12	2018	Year-2		1	Winter
12/12/2018	BL	3M	12	2018	Year-2	0	0	Winter
12/12/2018	BL	4M	12	2018	Year-2		1	Winter
12/12/2018	BL	5M	12	2018	Year-2		1	Winter
12/12/2018	BL	6M	12	2018	Year-2		1	Winter
12/12/2018	BL	7M	12	2018	Year-2	0	0	Winter
12/12/2018	BL	10M	12	2018	Year-2		1	Winter
12/12/2018	BL	12M	12	2018	Year-2		1	Winter
12/12/2018	BL	14M	12	2018	Year-2	0	0	Winter
12/12/2018	BL	8A	12	2018	Year-2		1	Winter
12/12/2018	BL	9A	12	2018	Year-2	0	0	Winter
12/12/2018	BL	11A	12	2018	Year-2	0	0	Winter
12/12/2018	BL	13A	12	2018	Year-2	0	0	Winter
12/13/2018	BL	1M	12	2018	Year-2	0	0	Winter
12/13/2018	BL	2M	12	2018	Year-2		1	Winter
12/13/2018	BL	3M	12	2018	Year-2	0	0	Winter
12/13/2018	BL	4M	12	2018	Year-2		1	Winter
12/13/2018	BL	5M	12	2018	Year-2		1	Winter
12/13/2018	BL	6M	12	2018	Year-2		1	Winter
12/13/2018	BL	7M	12	2018	Year-2	0	0	Winter
12/13/2018	BL	10M	12	2018	Year-2		1	Winter
12/13/2018	BL	12M	12	2018	Year-2		1	Winter
12/13/2018	BL	14M	12	2018	Year-2	0	0	Winter
12/13/2018	BL	8A	12	2018	Year-2		1	Winter

Date	Species	Site	Month	Year	Survey Year	Presence	Data Gap	Season
12/13/2018	BL	9A	12	2018	Year-2	0	0	Winter
12/13/2018	BL	11A	12	2018	Year-2	0	0	Winter
12/13/2018	BL	13A	12	2018	Year-2	0	0	Winter
12/14/2018	BL	1M	12	2018	Year-2	0	0	Winter
12/14/2018	BL	2M	12	2018	Year-2		1	Winter
12/14/2018	BL	3M	12	2018	Year-2	0	0	Winter
12/14/2018	BL	4M	12	2018	Year-2		1	Winter
12/14/2018	BL	5M	12	2018	Year-2		1	Winter
12/14/2018	BL	6M	12	2018	Year-2		1	Winter
12/14/2018	BL	7M	12	2018	Year-2	0	0	Winter
12/14/2018	BL	10M	12	2018	Year-2		1	Winter
12/14/2018	BL	12M	12	2018	Year-2		1	Winter
12/14/2018	BL	14M	12	2018	Year-2	0	0	Winter
12/14/2018	BL	8A	12	2018	Year-2		1	Winter
12/14/2018	BL	9A	12	2018	Year-2	0	0	Winter
12/14/2018	BL	11A	12	2018	Year-2	0	0	Winter
12/14/2018	BL	13A	12	2018	Year-2	0	0	Winter
12/15/2018	BL	1M	12	2018	Year-2	0	0	Winter
12/15/2018	BL	2M	12	2018	Year-2		1	Winter
12/15/2018	BL	3M	12	2018	Year-2	0	0	Winter
12/15/2018	BL	4M	12	2018	Year-2		1	Winter
12/15/2018	BL	5M	12	2018	Year-2		1	Winter
12/15/2018	BL	6M	12	2018	Year-2		1	Winter
12/15/2018	BL	7M	12	2018	Year-2	0	0	Winter
12/15/2018	BL	10M	12	2018	Year-2		1	Winter
12/15/2018	BL	12M	12	2018	Year-2		1	Winter
12/15/2018	BL	14M	12	2018	Year-2	0	0	Winter
12/15/2018	BL	8A	12	2018	Year-2		1	Winter
12/15/2018	BL	9A	12	2018	Year-2	0	0	Winter
12/15/2018	BL	11A	12	2018	Year-2	0	0	Winter
12/15/2018	BL	13A	12	2018	Year-2	0	0	Winter
12/16/2018	BL	1M	12	2018	Year-2	0	0	Winter
12/16/2018	BL	2M	12	2018	Year-2		1	Winter
12/16/2018	BL	3M	12	2018	Year-2	0	0	Winter
12/16/2018	BL	4M	12	2018	Year-2		1	Winter
12/16/2018	BL	5M	12	2018	Year-2		1	Winter
12/16/2018	BL	6M	12	2018	Year-2		1	Winter
12/16/2018	BL	7M	12	2018	Year-2	0	0	Winter
12/16/2018	BL	10M	12	2018	Year-2		1	Winter
12/16/2018	BL	12M	12	2018	Year-2		1	Winter
12/16/2018	BL	14M	12	2018	Year-2	0	0	Winter
12/16/2018	BL	8A	12	2018	Year-2		1	Winter
12/16/2018	BL	9A	12	2018	Year-2	0	0	Winter
12/16/2018	BL	11A	12	2018	Year-2	0	0	Winter

Date	Species	Site	Month	Year	Survey Year	Presence	Data Gap	Season
12/16/2018	BL	13A	12	2018	Year-2	0	0	Winter
12/17/2018	BL	1M	12	2018	Year-2	0	0	Winter
12/17/2018	BL	2M	12	2018	Year-2		1	Winter
12/17/2018	BL	3M	12	2018	Year-2	0	0	Winter
12/17/2018	BL	4M	12	2018	Year-2		1	Winter
12/17/2018	BL	5M	12	2018	Year-2		1	Winter
12/17/2018	BL	6M	12	2018	Year-2		1	Winter
12/17/2018	BL	7M	12	2018	Year-2	0	0	Winter
12/17/2018	BL	10M	12	2018	Year-2		1	Winter
12/17/2018	BL	12M	12	2018	Year-2		1	Winter
12/17/2018	BL	14M	12	2018	Year-2	0	0	Winter
12/17/2018	BL	8A	12	2018	Year-2		1	Winter
12/17/2018	BL	9A	12	2018	Year-2	0	0	Winter
12/17/2018	BL	11A	12	2018	Year-2	0	0	Winter
12/17/2018	BL	13A	12	2018	Year-2	0	0	Winter
12/18/2018	BL	1M	12	2018	Year-2	0	0	Winter
12/18/2018	BL	2M	12	2018	Year-2		1	Winter
12/18/2018	BL	3M	12	2018	Year-2	0	0	Winter
12/18/2018	BL	4M	12	2018	Year-2		1	Winter
12/18/2018	BL	5M	12	2018	Year-2		1	Winter
12/18/2018	BL	6M	12	2018	Year-2		1	Winter
12/18/2018	BL	7M	12	2018	Year-2	0	0	Winter
12/18/2018	BL	10M	12	2018	Year-2		1	Winter
12/18/2018	BL	12M	12	2018	Year-2		1	Winter
12/18/2018	BL	14M	12	2018	Year-2	0	0	Winter
12/18/2018	BL	8A	12	2018	Year-2		1	Winter
12/18/2018	BL	9A	12	2018	Year-2	0	0	Winter
12/18/2018	BL	11A	12	2018	Year-2	0	0	Winter
12/18/2018	BL	13A	12	2018	Year-2	0	0	Winter
12/19/2018	BL	1M	12	2018	Year-2	0	0	Winter
12/19/2018	BL	2M	12	2018	Year-2		1	Winter
12/19/2018	BL	3M	12	2018	Year-2	0	0	Winter
12/19/2018	BL	4M	12	2018	Year-2		1	Winter
12/19/2018	BL	5M	12	2018	Year-2		1	Winter
12/19/2018	BL	6M	12	2018	Year-2		1	Winter
12/19/2018	BL	7M	12	2018	Year-2	0	0	Winter
12/19/2018	BL	10M	12	2018	Year-2		1	Winter
12/19/2018	BL	12M	12	2018	Year-2		1	Winter
12/19/2018	BL	14M	12	2018	Year-2	0	0	Winter
12/19/2018	BL	8A	12	2018	Year-2		1	Winter
12/19/2018	BL	9A	12	2018	Year-2	0	0	Winter
12/19/2018	BL	11A	12	2018	Year-2	0	0	Winter
12/19/2018	BL	13A	12	2018	Year-2	0	0	Winter
12/20/2018	BL	1M	12	2018	Year-2	0	0	Winter

Date	Species	Site	Month	Year	Survey Year	Presence	Data Gap	Season
12/20/2018	BL	2M	12	2018	Year-2		1	Winter
12/20/2018	BL	3M	12	2018	Year-2	0	0	Winter
12/20/2018	BL	4M	12	2018	Year-2		1	Winter
12/20/2018	BL	5M	12	2018	Year-2		1	Winter
12/20/2018	BL	6M	12	2018	Year-2		1	Winter
12/20/2018	BL	7M	12	2018	Year-2	0	0	Winter
12/20/2018	BL	10M	12	2018	Year-2		1	Winter
12/20/2018	BL	12M	12	2018	Year-2		1	Winter
12/20/2018	BL	14M	12	2018	Year-2		1	Winter
12/20/2018	BL	8A	12	2018	Year-2		1	Winter
12/20/2018	BL	9A	12	2018	Year-2	0	0	Winter
12/20/2018	BL	11A	12	2018	Year-2	0	0	Winter
12/20/2018	BL	13A	12	2018	Year-2	0	0	Winter
12/21/2018	BL	1M	12	2018	Year-2	0	0	Winter
12/21/2018	BL	2M	12	2018	Year-2		1	Winter
12/21/2018	BL	3M	12	2018	Year-2	0	0	Winter
12/21/2018	BL	4M	12	2018	Year-2		1	Winter
12/21/2018	BL	5M	12	2018	Year-2		1	Winter
12/21/2018	BL	6M	12	2018	Year-2		1	Winter
12/21/2018	BL	7M	12	2018	Year-2	0	0	Winter
12/21/2018	BL	10M	12	2018	Year-2		1	Winter
12/21/2018	BL	12M	12	2018	Year-2		1	Winter
12/21/2018	BL	14M	12	2018	Year-2		1	Winter
12/21/2018	BL	8A	12	2018	Year-2		1	Winter
12/21/2018	BL	9A	12	2018	Year-2	0	0	Winter
12/21/2018	BL	11A	12	2018	Year-2	0	0	Winter
12/21/2018	BL	13A	12	2018	Year-2	0	0	Winter
12/22/2018	BL	1M	12	2018	Year-2	0	0	Winter
12/22/2018	BL	2M	12	2018	Year-2		1	Winter
12/22/2018	BL	3M	12	2018	Year-2	0	0	Winter
12/22/2018	BL	4M	12	2018	Year-2		1	Winter
12/22/2018	BL	5M	12	2018	Year-2		1	Winter
12/22/2018	BL	6M	12	2018	Year-2		1	Winter
12/22/2018	BL	7M	12	2018	Year-2	0	0	Winter
12/22/2018	BL	10M	12	2018	Year-2		1	Winter
12/22/2018	BL	12M	12	2018	Year-2		1	Winter
12/22/2018	BL	14M	12	2018	Year-2		1	Winter
12/22/2018	BL	8A	12	2018	Year-2		1	Winter
12/22/2018	BL	9A	12	2018	Year-2	0	0	Winter
12/22/2018	BL	11A	12	2018	Year-2	0	0	Winter
12/22/2018	BL	13A	12	2018	Year-2	0	0	Winter
12/23/2018	BL	1M	12	2018	Year-2	0	0	Winter
12/23/2018	BL	2M	12	2018	Year-2		1	Winter
12/23/2018	BL	3M	12	2018	Year-2		1	Winter

Date	Species	Site	Month	Year	Survey Year	Presence	Data Gap	Season
12/23/2018	BL	4M	12	2018	Year-2		1	Winter
12/23/2018	BL	5M	12	2018	Year-2		1	Winter
12/23/2018	BL	6M	12	2018	Year-2		1	Winter
12/23/2018	BL	7M	12	2018	Year-2	0	0	Winter
12/23/2018	BL	10M	12	2018	Year-2		1	Winter
12/23/2018	BL	12M	12	2018	Year-2		1	Winter
12/23/2018	BL	14M	12	2018	Year-2		1	Winter
12/23/2018	BL	8A	12	2018	Year-2		1	Winter
12/23/2018	BL	9A	12	2018	Year-2	0	0	Winter
12/23/2018	BL	11A	12	2018	Year-2	0	0	Winter
12/23/2018	BL	13A	12	2018	Year-2	0	0	Winter
12/24/2018	BL	1M	12	2018	Year-2		1	Winter
12/24/2018	BL	2M	12	2018	Year-2		1	Winter
12/24/2018	BL	3M	12	2018	Year-2		1	Winter
12/24/2018	BL	4M	12	2018	Year-2		1	Winter
12/24/2018	BL	5M	12	2018	Year-2		1	Winter
12/24/2018	BL	6M	12	2018	Year-2		1	Winter
12/24/2018	BL	7M	12	2018	Year-2		1	Winter
12/24/2018	BL	10M	12	2018	Year-2		1	Winter
12/24/2018	BL	12M	12	2018	Year-2		1	Winter
12/24/2018	BL	14M	12	2018	Year-2		1	Winter
12/24/2018	BL	8A	12	2018	Year-2		1	Winter
12/24/2018	BL	9A	12	2018	Year-2	0	0	Winter
12/24/2018	BL	11A	12	2018	Year-2	0	0	Winter
12/24/2018	BL	13A	12	2018	Year-2	0	0	Winter
12/25/2018	BL	1M	12	2018	Year-2		1	Winter
12/25/2018	BL	2M	12	2018	Year-2		1	Winter
12/25/2018	BL	3M	12	2018	Year-2		1	Winter
12/25/2018	BL	4M	12	2018	Year-2		1	Winter
12/25/2018	BL	5M	12	2018	Year-2		1	Winter
12/25/2018	BL	6M	12	2018	Year-2		1	Winter
12/25/2018	BL	7M	12	2018	Year-2		1	Winter
12/25/2018	BL	10M	12	2018	Year-2		1	Winter
12/25/2018	BL	12M	12	2018	Year-2		1	Winter
12/25/2018	BL	14M	12	2018	Year-2		1	Winter
12/25/2018	BL	8A	12	2018	Year-2		1	Winter
12/25/2018	BL	9A	12	2018	Year-2	0	0	Winter
12/25/2018	BL	11A	12	2018	Year-2	0	0	Winter
12/25/2018	BL	13A	12	2018	Year-2	0	0	Winter
12/26/2018	BL	1M	12	2018	Year-2		1	Winter
12/26/2018	BL	2M	12	2018	Year-2		1	Winter
12/26/2018	BL	3M	12	2018	Year-2		1	Winter
12/26/2018	BL	4M	12	2018	Year-2		1	Winter
12/26/2018	BL	5M	12	2018	Year-2		1	Winter

Date	Species	Site	Month	Year	Survey Year	Presence	Data Gap	Season
12/26/2018	BL	6M	12	2018	Year-2		1	Winter
12/26/2018	BL	7M	12	2018	Year-2		1	Winter
12/26/2018	BL	10M	12	2018	Year-2		1	Winter
12/26/2018	BL	12M	12	2018	Year-2		1	Winter
12/26/2018	BL	14M	12	2018	Year-2		1	Winter
12/26/2018	BL	8A	12	2018	Year-2		1	Winter
12/26/2018	BL	9A	12	2018	Year-2	0	0	Winter
12/26/2018	BL	11A	12	2018	Year-2	0	0	Winter
12/26/2018	BL	13A	12	2018	Year-2	0	0	Winter
12/27/2018	BL	1M	12	2018	Year-2		1	Winter
12/27/2018	BL	2M	12	2018	Year-2		1	Winter
12/27/2018	BL	3M	12	2018	Year-2		1	Winter
12/27/2018	BL	4M	12	2018	Year-2		1	Winter
12/27/2018	BL	5M	12	2018	Year-2		1	Winter
12/27/2018	BL	6M	12	2018	Year-2		1	Winter
12/27/2018	BL	7M	12	2018	Year-2		1	Winter
12/27/2018	BL	10M	12	2018	Year-2		1	Winter
12/27/2018	BL	12M	12	2018	Year-2		1	Winter
12/27/2018	BL	14M	12	2018	Year-2		1	Winter
12/27/2018	BL	8A	12	2018	Year-2		1	Winter
12/27/2018	BL	9A	12	2018	Year-2	0	0	Winter
12/27/2018	BL	11A	12	2018	Year-2	0	0	Winter
12/27/2018	BL	13A	12	2018	Year-2	0	0	Winter
12/28/2018	BL	1M	12	2018	Year-2		1	Winter
12/28/2018	BL	2M	12	2018	Year-2		1	Winter
12/28/2018	BL	3M	12	2018	Year-2		1	Winter
12/28/2018	BL	4M	12	2018	Year-2		1	Winter
12/28/2018	BL	5M	12	2018	Year-2		1	Winter
12/28/2018	BL	6M	12	2018	Year-2		1	Winter
12/28/2018	BL	7M	12	2018	Year-2		1	Winter
12/28/2018	BL	10M	12	2018	Year-2		1	Winter
12/28/2018	BL	12M	12	2018	Year-2		1	Winter
12/28/2018	BL	14M	12	2018	Year-2		1	Winter
12/28/2018	BL	8A	12	2018	Year-2		1	Winter
12/28/2018	BL	9A	12	2018	Year-2	0	0	Winter
12/28/2018	BL	11A	12	2018	Year-2	0	0	Winter
12/28/2018	BL	13A	12	2018	Year-2	0	0	Winter
12/29/2018	BL	1M	12	2018	Year-2		1	Winter
12/29/2018	BL	2M	12	2018	Year-2		1	Winter
12/29/2018	BL	3M	12	2018	Year-2		1	Winter
12/29/2018	BL	4M	12	2018	Year-2		1	Winter
12/29/2018	BL	5M	12	2018	Year-2		1	Winter
12/29/2018	BL	6M	12	2018	Year-2		1	Winter
12/29/2018	BL	7M	12	2018	Year-2		1	Winter

Date	Species	Site	Month	Year	Survey Year	Presence	Data Gap	Season
12/29/2018	BL	10M	12	2018	Year-2		1	Winter
12/29/2018	BL	12M	12	2018	Year-2		1	Winter
12/29/2018	BL	14M	12	2018	Year-2		1	Winter
12/29/2018	BL	8A	12	2018	Year-2		1	Winter
12/29/2018	BL	9A	12	2018	Year-2	0	0	Winter
12/29/2018	BL	11A	12	2018	Year-2	0	0	Winter
12/29/2018	BL	13A	12	2018	Year-2	0	0	Winter
12/30/2018	BL	1M	12	2018	Year-2		1	Winter
12/30/2018	BL	2M	12	2018	Year-2		1	Winter
12/30/2018	BL	3M	12	2018	Year-2		1	Winter
12/30/2018	BL	4M	12	2018	Year-2		1	Winter
12/30/2018	BL	5M	12	2018	Year-2		1	Winter
12/30/2018	BL	6M	12	2018	Year-2		1	Winter
12/30/2018	BL	7M	12	2018	Year-2		1	Winter
12/30/2018	BL	10M	12	2018	Year-2		1	Winter
12/30/2018	BL	12M	12	2018	Year-2		1	Winter
12/30/2018	BL	14M	12	2018	Year-2		1	Winter
12/30/2018	BL	8A	12	2018	Year-2		1	Winter
12/30/2018	BL	9A	12	2018	Year-2	0	0	Winter
12/30/2018	BL	11A	12	2018	Year-2	0	0	Winter
12/30/2018	BL	13A	12	2018	Year-2	0	0	Winter
12/31/2018	BL	1M	12	2018	Year-2		1	Winter
12/31/2018	BL	2M	12	2018	Year-2		1	Winter
12/31/2018	BL	3M	12	2018	Year-2		1	Winter
12/31/2018	BL	4M	12	2018	Year-2		1	Winter
12/31/2018	BL	5M	12	2018	Year-2		1	Winter
12/31/2018	BL	6M	12	2018	Year-2		1	Winter
12/31/2018	BL	7M	12	2018	Year-2		1	Winter
12/31/2018	BL	10M	12	2018	Year-2		1	Winter
12/31/2018	BL	12M	12	2018	Year-2		1	Winter
12/31/2018	BL	14M	12	2018	Year-2		1	Winter
12/31/2018	BL	8A	12	2018	Year-2		1	Winter
12/31/2018	BL	9A	12	2018	Year-2	0	0	Winter
12/31/2018	BL	11A	12	2018	Year-2	0	0	Winter
12/31/2018	BL	13A	12	2018	Year-2	0	0	Winter
1/1/2019	BL	1M	1	2019	Year-2		1	Winter
1/1/2019	BL	2M	1	2019	Year-2		1	Winter
1/1/2019	BL	3M	1	2019	Year-2		1	Winter
1/1/2019	BL	4M	1	2019	Year-2		1	Winter
1/1/2019	BL	5M	1	2019	Year-2		1	Winter
1/1/2019	BL	6M	1	2019	Year-2		1	Winter
1/1/2019	BL	7M	1	2019	Year-2		1	Winter
1/1/2019	BL	10M	1	2019	Year-2		1	Winter
1/1/2019	BL	12M	1	2019	Year-2		1	Winter

Date	Species	Site	Month	Year	Survey Year	Presence	Data Gap	Season
1/1/2019	BL	14M	1	2019	Year-2		1	Winter
1/1/2019	BL	8A	1	2019	Year-2		1	Winter
1/1/2019	BL	9A	1	2019	Year-2	0	0	Winter
1/1/2019	BL	11A	1	2019	Year-2	0	0	Winter
1/1/2019	BL	13A	1	2019	Year-2	0	0	Winter
1/2/2019	BL	1M	1	2019	Year-2		1	Winter
1/2/2019	BL	2M	1	2019	Year-2		1	Winter
1/2/2019	BL	3M	1	2019	Year-2		1	Winter
1/2/2019	BL	4M	1	2019	Year-2		1	Winter
1/2/2019	BL	5M	1	2019	Year-2		1	Winter
1/2/2019	BL	6M	1	2019	Year-2		1	Winter
1/2/2019	BL	7M	1	2019	Year-2		1	Winter
1/2/2019	BL	10M	1	2019	Year-2		1	Winter
1/2/2019	BL	12M	1	2019	Year-2		1	Winter
1/2/2019	BL	14M	1	2019	Year-2		1	Winter
1/2/2019	BL	8A	1	2019	Year-2		1	Winter
1/2/2019	BL	9A	1	2019	Year-2	0	0	Winter
1/2/2019	BL	11A	1	2019	Year-2	0	0	Winter
1/2/2019	BL	13A	1	2019	Year-2	0	0	Winter
1/3/2019	BL	1M	1	2019	Year-2		1	Winter
1/3/2019	BL	2M	1	2019	Year-2		1	Winter
1/3/2019	BL	3M	1	2019	Year-2		1	Winter
1/3/2019	BL	4M	1	2019	Year-2		1	Winter
1/3/2019	BL	5M	1	2019	Year-2		1	Winter
1/3/2019	BL	6M	1	2019	Year-2		1	Winter
1/3/2019	BL	7M	1	2019	Year-2		1	Winter
1/3/2019	BL	10M	1	2019	Year-2		1	Winter
1/3/2019	BL	12M	1	2019	Year-2		1	Winter
1/3/2019	BL	14M	1	2019	Year-2		1	Winter
1/3/2019	BL	8A	1	2019	Year-2		1	Winter
1/3/2019	BL	9A	1	2019	Year-2	0	0	Winter
1/3/2019	BL	11A	1	2019	Year-2	0	0	Winter
1/3/2019	BL	13A	1	2019	Year-2	0	0	Winter
1/4/2019	BL	1M	1	2019	Year-2		1	Winter
1/4/2019	BL	2M	1	2019	Year-2		1	Winter
1/4/2019	BL	3M	1	2019	Year-2		1	Winter
1/4/2019	BL	4M	1	2019	Year-2		1	Winter
1/4/2019	BL	5M	1	2019	Year-2		1	Winter
1/4/2019	BL	6M	1	2019	Year-2		1	Winter
1/4/2019	BL	7M	1	2019	Year-2		1	Winter
1/4/2019	BL	10M	1	2019	Year-2		1	Winter
1/4/2019	BL	12M	1	2019	Year-2		1	Winter
1/4/2019	BL	14M	1	2019	Year-2		1	Winter
1/4/2019	BL	8A	1	2019	Year-2		1	Winter

Date	Species	Site	Month	Year	Survey Year	Presence	Data Gap	Season
1/4/2019	BL	9A	1	2019	Year-2	0	0	Winter
1/4/2019	BL	11A	1	2019	Year-2	0	0	Winter
1/4/2019	BL	13A	1	2019	Year-2	0	0	Winter
1/5/2019	BL	1M	1	2019	Year-2		1	Winter
1/5/2019	BL	2M	1	2019	Year-2		1	Winter
1/5/2019	BL	3M	1	2019	Year-2		1	Winter
1/5/2019	BL	4M	1	2019	Year-2		1	Winter
1/5/2019	BL	5M	1	2019	Year-2		1	Winter
1/5/2019	BL	6M	1	2019	Year-2		1	Winter
1/5/2019	BL	7M	1	2019	Year-2		1	Winter
1/5/2019	BL	10M	1	2019	Year-2		1	Winter
1/5/2019	BL	12M	1	2019	Year-2		1	Winter
1/5/2019	BL	14M	1	2019	Year-2		1	Winter
1/5/2019	BL	8A	1	2019	Year-2		1	Winter
1/5/2019	BL	9A	1	2019	Year-2	0	0	Winter
1/5/2019	BL	11A	1	2019	Year-2	0	0	Winter
1/5/2019	BL	13A	1	2019	Year-2	0	0	Winter
1/6/2019	BL	1M	1	2019	Year-2		1	Winter
1/6/2019	BL	2M	1	2019	Year-2		1	Winter
1/6/2019	BL	3M	1	2019	Year-2		1	Winter
1/6/2019	BL	4M	1	2019	Year-2		1	Winter
1/6/2019	BL	5M	1	2019	Year-2		1	Winter
1/6/2019	BL	6M	1	2019	Year-2		1	Winter
1/6/2019	BL	7M	1	2019	Year-2		1	Winter
1/6/2019	BL	10M	1	2019	Year-2		1	Winter
1/6/2019	BL	12M	1	2019	Year-2		1	Winter
1/6/2019	BL	14M	1	2019	Year-2		1	Winter
1/6/2019	BL	8A	1	2019	Year-2		1	Winter
1/6/2019	BL	9A	1	2019	Year-2	0	0	Winter
1/6/2019	BL	11A	1	2019	Year-2	0	0	Winter
1/6/2019	BL	13A	1	2019	Year-2	0	0	Winter
1/7/2019	BL	1M	1	2019	Year-2		1	Winter
1/7/2019	BL	2M	1	2019	Year-2		1	Winter
1/7/2019	BL	3M	1	2019	Year-2		1	Winter
1/7/2019	BL	4M	1	2019	Year-2		1	Winter
1/7/2019	BL	5M	1	2019	Year-2		1	Winter
1/7/2019	BL	6M	1	2019	Year-2		1	Winter
1/7/2019	BL	7M	1	2019	Year-2		1	Winter
1/7/2019	BL	10M	1	2019	Year-2		1	Winter
1/7/2019	BL	12M	1	2019	Year-2		1	Winter
1/7/2019	BL	14M	1	2019	Year-2		1	Winter
1/7/2019	BL	8A	1	2019	Year-2		1	Winter
1/7/2019	BL	9A	1	2019	Year-2	0	0	Winter
1/7/2019	BL	11A	1	2019	Year-2	0	0	Winter

Date	Species	Site	Month	Year	Survey Year	Presence	Data Gap	Season
1/7/2019	BL	13A	1	2019	Year-2	0	0	Winter
1/8/2019	BL	1M	1	2019	Year-2		1	Winter
1/8/2019	BL	2M	1	2019	Year-2		1	Winter
1/8/2019	BL	3M	1	2019	Year-2		1	Winter
1/8/2019	BL	4M	1	2019	Year-2		1	Winter
1/8/2019	BL	5M	1	2019	Year-2		1	Winter
1/8/2019	BL	6M	1	2019	Year-2		1	Winter
1/8/2019	BL	7M	1	2019	Year-2		1	Winter
1/8/2019	BL	10M	1	2019	Year-2		1	Winter
1/8/2019	BL	12M	1	2019	Year-2		1	Winter
1/8/2019	BL	14M	1	2019	Year-2		1	Winter
1/8/2019	BL	8A	1	2019	Year-2		1	Winter
1/8/2019	BL	9A	1	2019	Year-2	0	0	Winter
1/8/2019	BL	11A	1	2019	Year-2	0	0	Winter
1/8/2019	BL	13A	1	2019	Year-2	0	0	Winter
1/9/2019	BL	1M	1	2019	Year-2		1	Winter
1/9/2019	BL	2M	1	2019	Year-2		1	Winter
1/9/2019	BL	3M	1	2019	Year-2		1	Winter
1/9/2019	BL	4M	1	2019	Year-2		1	Winter
1/9/2019	BL	5M	1	2019	Year-2		1	Winter
1/9/2019	BL	6M	1	2019	Year-2		1	Winter
1/9/2019	BL	7M	1	2019	Year-2		1	Winter
1/9/2019	BL	10M	1	2019	Year-2		1	Winter
1/9/2019	BL	12M	1	2019	Year-2		1	Winter
1/9/2019	BL	14M	1	2019	Year-2		1	Winter
1/9/2019	BL	8A	1	2019	Year-2		1	Winter
1/9/2019	BL	9A	1	2019	Year-2	0	0	Winter
1/9/2019	BL	11A	1	2019	Year-2	0	0	Winter
1/9/2019	BL	13A	1	2019	Year-2	0	0	Winter
1/10/2019	BL	1M	1	2019	Year-2		1	Winter
1/10/2019	BL	2M	1	2019	Year-2		1	Winter
1/10/2019	BL	3M	1	2019	Year-2		1	Winter
1/10/2019	BL	4M	1	2019	Year-2		1	Winter
1/10/2019	BL	5M	1	2019	Year-2		1	Winter
1/10/2019	BL	6M	1	2019	Year-2		1	Winter
1/10/2019	BL	7M	1	2019	Year-2		1	Winter
1/10/2019	BL	10M	1	2019	Year-2		1	Winter
1/10/2019	BL	12M	1	2019	Year-2		1	Winter
1/10/2019	BL	14M	1	2019	Year-2		1	Winter
1/10/2019	BL	8A	1	2019	Year-2		1	Winter
1/10/2019	BL	9A	1	2019	Year-2	0	0	Winter
1/10/2019	BL	11A	1	2019	Year-2	0	0	Winter
1/10/2019	BL	13A	1	2019	Year-2	0	0	Winter
1/11/2019	BL	1M	1	2019	Year-2		1	Winter

Date	Species	Site	Month	Year	Survey Year	Presence	Data Gap	Season
1/11/2019	BL	2M	1	2019	Year-2		1	Winter
1/11/2019	BL	3M	1	2019	Year-2		1	Winter
1/11/2019	BL	4M	1	2019	Year-2		1	Winter
1/11/2019	BL	5M	1	2019	Year-2		1	Winter
1/11/2019	BL	6M	1	2019	Year-2		1	Winter
1/11/2019	BL	7M	1	2019	Year-2		1	Winter
1/11/2019	BL	10M	1	2019	Year-2		1	Winter
1/11/2019	BL	12M	1	2019	Year-2		1	Winter
1/11/2019	BL	14M	1	2019	Year-2		1	Winter
1/11/2019	BL	8A	1	2019	Year-2		1	Winter
1/11/2019	BL	9A	1	2019	Year-2	0	0	Winter
1/11/2019	BL	11A	1	2019	Year-2	0	0	Winter
1/11/2019	BL	13A	1	2019	Year-2	0	0	Winter
1/12/2019	BL	1M	1	2019	Year-2		1	Winter
1/12/2019	BL	2M	1	2019	Year-2		1	Winter
1/12/2019	BL	3M	1	2019	Year-2		1	Winter
1/12/2019	BL	4M	1	2019	Year-2		1	Winter
1/12/2019	BL	5M	1	2019	Year-2		1	Winter
1/12/2019	BL	6M	1	2019	Year-2		1	Winter
1/12/2019	BL	7M	1	2019	Year-2		1	Winter
1/12/2019	BL	10M	1	2019	Year-2		1	Winter
1/12/2019	BL	12M	1	2019	Year-2		1	Winter
1/12/2019	BL	14M	1	2019	Year-2		1	Winter
1/12/2019	BL	8A	1	2019	Year-2		1	Winter
1/12/2019	BL	9A	1	2019	Year-2	0	0	Winter
1/12/2019	BL	11A	1	2019	Year-2	0	0	Winter
1/12/2019	BL	13A	1	2019	Year-2	0	0	Winter
1/13/2019	BL	1M	1	2019	Year-2	0	0	Winter
1/13/2019	BL	2M	1	2019	Year-2		1	Winter
1/13/2019	BL	3M	1	2019	Year-2		1	Winter
1/13/2019	BL	4M	1	2019	Year-2		1	Winter
1/13/2019	BL	5M	1	2019	Year-2		1	Winter
1/13/2019	BL	6M	1	2019	Year-2		1	Winter
1/13/2019	BL	7M	1	2019	Year-2		1	Winter
1/13/2019	BL	10M	1	2019	Year-2		1	Winter
1/13/2019	BL	12M	1	2019	Year-2		1	Winter
1/13/2019	BL	14M	1	2019	Year-2		1	Winter
1/13/2019	BL	8A	1	2019	Year-2		1	Winter
1/13/2019	BL	9A	1	2019	Year-2	0	0	Winter
1/13/2019	BL	11A	1	2019	Year-2	0	0	Winter
1/13/2019	BL	13A	1	2019	Year-2	0	0	Winter
1/14/2019	BL	1M	1	2019	Year-2	0	0	Winter
1/14/2019	BL	2M	1	2019	Year-2	0	0	Winter
1/14/2019	BL	3M	1	2019	Year-2	0	0	Winter

Date	Species	Site	Month	Year	Survey Year	Presence	Data Gap	Season
1/14/2019	BL	4M	1	2019	Year-2	0	0	Winter
1/14/2019	BL	5M	1	2019	Year-2	0	0	Winter
1/14/2019	BL	6M	1	2019	Year-2	0	0	Winter
1/14/2019	BL	7M	1	2019	Year-2	0	0	Winter
1/14/2019	BL	10M	1	2019	Year-2		1	Winter
1/14/2019	BL	12M	1	2019	Year-2		1	Winter
1/14/2019	BL	14M	1	2019	Year-2		1	Winter
1/14/2019	BL	8A	1	2019	Year-2		1	Winter
1/14/2019	BL	9A	1	2019	Year-2	0	0	Winter
1/14/2019	BL	11A	1	2019	Year-2	0	0	Winter
1/14/2019	BL	13A	1	2019	Year-2	0	0	Winter
1/15/2019	BL	1M	1	2019	Year-2	0	0	Winter
1/15/2019	BL	2M	1	2019	Year-2	0	0	Winter
1/15/2019	BL	3M	1	2019	Year-2	0	0	Winter
1/15/2019	BL	4M	1	2019	Year-2	0	0	Winter
1/15/2019	BL	5M	1	2019	Year-2	0	0	Winter
1/15/2019	BL	6M	1	2019	Year-2	0	0	Winter
1/15/2019	BL	7M	1	2019	Year-2	0	0	Winter
1/15/2019	BL	10M	1	2019	Year-2		1	Winter
1/15/2019	BL	12M	1	2019	Year-2		1	Winter
1/15/2019	BL	14M	1	2019	Year-2		1	Winter
1/15/2019	BL	8A	1	2019	Year-2		1	Winter
1/15/2019	BL	9A	1	2019	Year-2	0	0	Winter
1/15/2019	BL	11A	1	2019	Year-2	0	0	Winter
1/15/2019	BL	13A	1	2019	Year-2	0	0	Winter
1/16/2019	BL	1M	1	2019	Year-2	0	0	Winter
1/16/2019	BL	2M	1	2019	Year-2	0	0	Winter
1/16/2019	BL	3M	1	2019	Year-2	0	0	Winter
1/16/2019	BL	4M	1	2019	Year-2	0	0	Winter
1/16/2019	BL	5M	1	2019	Year-2	0	0	Winter
1/16/2019	BL	6M	1	2019	Year-2	0	0	Winter
1/16/2019	BL	7M	1	2019	Year-2	0	0	Winter
1/16/2019	BL	10M	1	2019	Year-2		1	Winter
1/16/2019	BL	12M	1	2019	Year-2		1	Winter
1/16/2019	BL	14M	1	2019	Year-2		1	Winter
1/16/2019	BL	8A	1	2019	Year-2		1	Winter
1/16/2019	BL	9A	1	2019	Year-2	0	0	Winter
1/16/2019	BL	11A	1	2019	Year-2	0	0	Winter
1/16/2019	BL	13A	1	2019	Year-2	0	0	Winter
1/17/2019	BL	1M	1	2019	Year-2	0	0	Winter
1/17/2019	BL	2M	1	2019	Year-2	0	0	Winter
1/17/2019	BL	3M	1	2019	Year-2	0	0	Winter
1/17/2019	BL	4M	1	2019	Year-2	0	0	Winter
1/17/2019	BL	5M	1	2019	Year-2	0	0	Winter

Date	Species	Site	Month	Year	Survey Year	Presence	Data Gap	Season
1/17/2019	BL	6M	1	2019	Year-2	0	0	Winter
1/17/2019	BL	7M	1	2019	Year-2	0	0	Winter
1/17/2019	BL	10M	1	2019	Year-2		1	Winter
1/17/2019	BL	12M	1	2019	Year-2		1	Winter
1/17/2019	BL	14M	1	2019	Year-2		1	Winter
1/17/2019	BL	8A	1	2019	Year-2		1	Winter
1/17/2019	BL	9A	1	2019	Year-2	0	0	Winter
1/17/2019	BL	11A	1	2019	Year-2	0	0	Winter
1/17/2019	BL	13A	1	2019	Year-2	0	0	Winter
1/18/2019	BL	1M	1	2019	Year-2	0	0	Winter
1/18/2019	BL	2M	1	2019	Year-2	0	0	Winter
1/18/2019	BL	3M	1	2019	Year-2	0	0	Winter
1/18/2019	BL	4M	1	2019	Year-2	0	0	Winter
1/18/2019	BL	5M	1	2019	Year-2	0	0	Winter
1/18/2019	BL	6M	1	2019	Year-2	0	0	Winter
1/18/2019	BL	7M	1	2019	Year-2	0	0	Winter
1/18/2019	BL	10M	1	2019	Year-2		1	Winter
1/18/2019	BL	12M	1	2019	Year-2		1	Winter
1/18/2019	BL	14M	1	2019	Year-2	0	0	Winter
1/18/2019	BL	8A	1	2019	Year-2		1	Winter
1/18/2019	BL	9A	1	2019	Year-2	0	0	Winter
1/18/2019	BL	11A	1	2019	Year-2	0	0	Winter
1/18/2019	BL	13A	1	2019	Year-2	0	0	Winter
1/19/2019	BL	1M	1	2019	Year-2	0	0	Winter
1/19/2019	BL	2M	1	2019	Year-2	0	0	Winter
1/19/2019	BL	3M	1	2019	Year-2	0	0	Winter
1/19/2019	BL	4M	1	2019	Year-2	0	0	Winter
1/19/2019	BL	5M	1	2019	Year-2	0	0	Winter
1/19/2019	BL	6M	1	2019	Year-2	0	0	Winter
1/19/2019	BL	7M	1	2019	Year-2	0	0	Winter
1/19/2019	BL	10M	1	2019	Year-2	0	0	Winter
1/19/2019	BL	12M	1	2019	Year-2	0	0	Winter
1/19/2019	BL	14M	1	2019	Year-2	0	0	Winter
1/19/2019	BL	8A	1	2019	Year-2		1	Winter
1/19/2019	BL	9A	1	2019	Year-2	0	0	Winter
1/19/2019	BL	11A	1	2019	Year-2	0	0	Winter
1/19/2019	BL	13A	1	2019	Year-2	0	0	Winter
1/20/2019	BL	1M	1	2019	Year-2	0	0	Winter
1/20/2019	BL	2M	1	2019	Year-2	0	0	Winter
1/20/2019	BL	3M	1	2019	Year-2	0	0	Winter
1/20/2019	BL	4M	1	2019	Year-2	0	0	Winter
1/20/2019	BL	5M	1	2019	Year-2	0	0	Winter
1/20/2019	BL	6M	1	2019	Year-2	0	0	Winter
1/20/2019	BL	7M	1	2019	Year-2	0	0	Winter

Date	Species	Site	Month	Year	Survey Year	Presence	Data Gap	Season
1/20/2019	BL	10M	1	2019	Year-2	0	0	Winter
1/20/2019	BL	12M	1	2019	Year-2	0	0	Winter
1/20/2019	BL	14M	1	2019	Year-2	0	0	Winter
1/20/2019	BL	8A	1	2019	Year-2		1	Winter
1/20/2019	BL	9A	1	2019	Year-2	0	0	Winter
1/20/2019	BL	11A	1	2019	Year-2	0	0	Winter
1/20/2019	BL	13A	1	2019	Year-2	0	0	Winter
1/21/2019	BL	1M	1	2019	Year-2	0	0	Winter
1/21/2019	BL	2M	1	2019	Year-2	0	0	Winter
1/21/2019	BL	3M	1	2019	Year-2	0	0	Winter
1/21/2019	BL	4M	1	2019	Year-2	0	0	Winter
1/21/2019	BL	5M	1	2019	Year-2	0	0	Winter
1/21/2019	BL	6M	1	2019	Year-2	0	0	Winter
1/21/2019	BL	7M	1	2019	Year-2	0	0	Winter
1/21/2019	BL	10M	1	2019	Year-2	0	0	Winter
1/21/2019	BL	12M	1	2019	Year-2	0	0	Winter
1/21/2019	BL	14M	1	2019	Year-2	0	0	Winter
1/21/2019	BL	8A	1	2019	Year-2		1	Winter
1/21/2019	BL	9A	1	2019	Year-2	0	0	Winter
1/21/2019	BL	11A	1	2019	Year-2	0	0	Winter
1/21/2019	BL	13A	1	2019	Year-2	0	0	Winter
1/22/2019	BL	1M	1	2019	Year-2	0	0	Winter
1/22/2019	BL	2M	1	2019	Year-2	0	0	Winter
1/22/2019	BL	3M	1	2019	Year-2	0	0	Winter
1/22/2019	BL	4M	1	2019	Year-2	0	0	Winter
1/22/2019	BL	5M	1	2019	Year-2	0	0	Winter
1/22/2019	BL	6M	1	2019	Year-2	0	0	Winter
1/22/2019	BL	7M	1	2019	Year-2	0	0	Winter
1/22/2019	BL	10M	1	2019	Year-2	0	0	Winter
1/22/2019	BL	12M	1	2019	Year-2	0	0	Winter
1/22/2019	BL	14M	1	2019	Year-2	0	0	Winter
1/22/2019	BL	8A	1	2019	Year-2		1	Winter
1/22/2019	BL	9A	1	2019	Year-2	0	0	Winter
1/22/2019	BL	11A	1	2019	Year-2	0	0	Winter
1/22/2019	BL	13A	1	2019	Year-2	0	0	Winter
1/23/2019	BL	1M	1	2019	Year-2	1	0	Winter
1/23/2019	BL	2M	1	2019	Year-2	1	0	Winter
1/23/2019	BL	3M	1	2019	Year-2	0	0	Winter
1/23/2019	BL	4M	1	2019	Year-2	0	0	Winter
1/23/2019	BL	5M	1	2019	Year-2	0	0	Winter
1/23/2019	BL	6M	1	2019	Year-2	0	0	Winter
1/23/2019	BL	7M	1	2019	Year-2	0	0	Winter
1/23/2019	BL	10M	1	2019	Year-2	0	0	Winter
1/23/2019	BL	12M	1	2019	Year-2	0	0	Winter

Date	Species	Site	Month	Year	Survey Year	Presence	Data Gap	Season
1/23/2019	BL	14M	1	2019	Year-2	1	0	Winter
1/23/2019	BL	8A	1	2019	Year-2		1	Winter
1/23/2019	BL	9A	1	2019	Year-2	0	0	Winter
1/23/2019	BL	11A	1	2019	Year-2	0	0	Winter
1/23/2019	BL	13A	1	2019	Year-2	0	0	Winter
1/24/2019	BL	1M	1	2019	Year-2	0	0	Winter
1/24/2019	BL	2M	1	2019	Year-2	1	0	Winter
1/24/2019	BL	3M	1	2019	Year-2	0	0	Winter
1/24/2019	BL	4M	1	2019	Year-2	0	0	Winter
1/24/2019	BL	5M	1	2019	Year-2	0	0	Winter
1/24/2019	BL	6M	1	2019	Year-2	0	0	Winter
1/24/2019	BL	7M	1	2019	Year-2	0	0	Winter
1/24/2019	BL	10M	1	2019	Year-2	0	0	Winter
1/24/2019	BL	12M	1	2019	Year-2	0	0	Winter
1/24/2019	BL	14M	1	2019	Year-2	0	0	Winter
1/24/2019	BL	8A	1	2019	Year-2		1	Winter
1/24/2019	BL	9A	1	2019	Year-2	0	0	Winter
1/24/2019	BL	11A	1	2019	Year-2	0	0	Winter
1/24/2019	BL	13A	1	2019	Year-2	0	0	Winter
1/25/2019	BL	1M	1	2019	Year-2	0	0	Winter
1/25/2019	BL	2M	1	2019	Year-2	0	0	Winter
1/25/2019	BL	3M	1	2019	Year-2	0	0	Winter
1/25/2019	BL	4M	1	2019	Year-2	0	0	Winter
1/25/2019	BL	5M	1	2019	Year-2	0	0	Winter
1/25/2019	BL	6M	1	2019	Year-2	0	0	Winter
1/25/2019	BL	7M	1	2019	Year-2	0	0	Winter
1/25/2019	BL	10M	1	2019	Year-2	0	0	Winter
1/25/2019	BL	12M	1	2019	Year-2	0	0	Winter
1/25/2019	BL	14M	1	2019	Year-2	0	0	Winter
1/25/2019	BL	8A	1	2019	Year-2		1	Winter
1/25/2019	BL	9A	1	2019	Year-2	0	0	Winter
1/25/2019	BL	11A	1	2019	Year-2	0	0	Winter
1/25/2019	BL	13A	1	2019	Year-2	0	0	Winter
1/26/2019	BL	1M	1	2019	Year-2	1	0	Winter
1/26/2019	BL	2M	1	2019	Year-2	1	0	Winter
1/26/2019	BL	3M	1	2019	Year-2	0	0	Winter
1/26/2019	BL	4M	1	2019	Year-2	0	0	Winter
1/26/2019	BL	5M	1	2019	Year-2	0	0	Winter
1/26/2019	BL	6M	1	2019	Year-2	0	0	Winter
1/26/2019	BL	7M	1	2019	Year-2	0	0	Winter
1/26/2019	BL	10M	1	2019	Year-2	0	0	Winter
1/26/2019	BL	12M	1	2019	Year-2	0	0	Winter
1/26/2019	BL	14M	1	2019	Year-2	1	0	Winter
1/26/2019	BL	8A	1	2019	Year-2		1	Winter

Date	Species	Site	Month	Year	Survey Year	Presence	Data Gap	Season
1/26/2019	BL	9A	1	2019	Year-2	0	0	Winter
1/26/2019	BL	11A	1	2019	Year-2	0	0	Winter
1/26/2019	BL	13A	1	2019	Year-2	0	0	Winter
1/27/2019	BL	1M	1	2019	Year-2	1	0	Winter
1/27/2019	BL	2M	1	2019	Year-2	1	0	Winter
1/27/2019	BL	3M	1	2019	Year-2	0	0	Winter
1/27/2019	BL	4M	1	2019	Year-2	0	0	Winter
1/27/2019	BL	5M	1	2019	Year-2	0	0	Winter
1/27/2019	BL	6M	1	2019	Year-2	0	0	Winter
1/27/2019	BL	7M	1	2019	Year-2	0	0	Winter
1/27/2019	BL	10M	1	2019	Year-2	0	0	Winter
1/27/2019	BL	12M	1	2019	Year-2	0	0	Winter
1/27/2019	BL	14M	1	2019	Year-2	0	0	Winter
1/27/2019	BL	8A	1	2019	Year-2		1	Winter
1/27/2019	BL	9A	1	2019	Year-2	0	0	Winter
1/27/2019	BL	11A	1	2019	Year-2	0	0	Winter
1/27/2019	BL	13A	1	2019	Year-2	0	0	Winter
1/28/2019	BL	1M	1	2019	Year-2	1	0	Winter
1/28/2019	BL	2M	1	2019	Year-2	1	0	Winter
1/28/2019	BL	3M	1	2019	Year-2	0	0	Winter
1/28/2019	BL	4M	1	2019	Year-2	0	0	Winter
1/28/2019	BL	5M	1	2019	Year-2	0	0	Winter
1/28/2019	BL	6M	1	2019	Year-2	0	0	Winter
1/28/2019	BL	7M	1	2019	Year-2	0	0	Winter
1/28/2019	BL	10M	1	2019	Year-2	0	0	Winter
1/28/2019	BL	12M	1	2019	Year-2	0	0	Winter
1/28/2019	BL	14M	1	2019	Year-2	0	0	Winter
1/28/2019	BL	8A	1	2019	Year-2		1	Winter
1/28/2019	BL	9A	1	2019	Year-2	0	0	Winter
1/28/2019	BL	11A	1	2019	Year-2	0	0	Winter
1/28/2019	BL	13A	1	2019	Year-2	0	0	Winter
1/29/2019	BL	1M	1	2019	Year-2	0	0	Winter
1/29/2019	BL	2M	1	2019	Year-2	0	0	Winter
1/29/2019	BL	3M	1	2019	Year-2	1	0	Winter
1/29/2019	BL	4M	1	2019	Year-2	0	0	Winter
1/29/2019	BL	5M	1	2019	Year-2	0	0	Winter
1/29/2019	BL	6M	1	2019	Year-2	0	0	Winter
1/29/2019	BL	7M	1	2019	Year-2	0	0	Winter
1/29/2019	BL	10M	1	2019	Year-2	0	0	Winter
1/29/2019	BL	12M	1	2019	Year-2	0	0	Winter
1/29/2019	BL	14M	1	2019	Year-2	1	0	Winter
1/29/2019	BL	8A	1	2019	Year-2		1	Winter
1/29/2019	BL	9A	1	2019	Year-2	0	0	Winter
1/29/2019	BL	11A	1	2019	Year-2	0	0	Winter

Date	Species	Site	Month	Year	Survey Year	Presence	Data Gap	Season
1/29/2019	BL	13A	1	2019	Year-2	0	0	Winter
1/30/2019	BL	1M	1	2019	Year-2	0	0	Winter
1/30/2019	BL	2M	1	2019	Year-2	0	0	Winter
1/30/2019	BL	3M	1	2019	Year-2	0	0	Winter
1/30/2019	BL	4M	1	2019	Year-2	0	0	Winter
1/30/2019	BL	5M	1	2019	Year-2	0	0	Winter
1/30/2019	BL	6M	1	2019	Year-2	0	0	Winter
1/30/2019	BL	7M	1	2019	Year-2	0	0	Winter
1/30/2019	BL	10M	1	2019	Year-2	0	0	Winter
1/30/2019	BL	12M	1	2019	Year-2	0	0	Winter
1/30/2019	BL	14M	1	2019	Year-2	0	0	Winter
1/30/2019	BL	8A	1	2019	Year-2		1	Winter
1/30/2019	BL	9A	1	2019	Year-2	0	0	Winter
1/30/2019	BL	11A	1	2019	Year-2	0	0	Winter
1/30/2019	BL	13A	1	2019	Year-2	0	0	Winter
1/31/2019	BL	1M	1	2019	Year-2	0	0	Winter
1/31/2019	BL	2M	1	2019	Year-2	0	0	Winter
1/31/2019	BL	3M	1	2019	Year-2	0	0	Winter
1/31/2019	BL	4M	1	2019	Year-2	0	0	Winter
1/31/2019	BL	5M	1	2019	Year-2	0	0	Winter
1/31/2019	BL	6M	1	2019	Year-2	0	0	Winter
1/31/2019	BL	7M	1	2019	Year-2	0	0	Winter
1/31/2019	BL	10M	1	2019	Year-2	0	0	Winter
1/31/2019	BL	12M	1	2019	Year-2	0	0	Winter
1/31/2019	BL	14M	1	2019	Year-2	0	0	Winter
1/31/2019	BL	8A	1	2019	Year-2		1	Winter
1/31/2019	BL	9A	1	2019	Year-2	0	0	Winter
1/31/2019	BL	11A	1	2019	Year-2	0	0	Winter
1/31/2019	BL	13A	1	2019	Year-2	0	0	Winter
2/1/2019	BL	1M	2	2019	Year-2	0	0	Winter
2/1/2019	BL	2M	2	2019	Year-2	0	0	Winter
2/1/2019	BL	3M	2	2019	Year-2	0	0	Winter
2/1/2019	BL	4M	2	2019	Year-2	0	0	Winter
2/1/2019	BL	5M	2	2019	Year-2	0	0	Winter
2/1/2019	BL	6M	2	2019	Year-2	0	0	Winter
2/1/2019	BL	7M	2	2019	Year-2	0	0	Winter
2/1/2019	BL	10M	2	2019	Year-2	0	0	Winter
2/1/2019	BL	12M	2	2019	Year-2	0	0	Winter
2/1/2019	BL	14M	2	2019	Year-2	0	0	Winter
2/1/2019	BL	8A	2	2019	Year-2		1	Winter
2/1/2019	BL	9A	2	2019	Year-2	0	0	Winter
2/1/2019	BL	11A	2	2019	Year-2	0	0	Winter
2/1/2019	BL	13A	2	2019	Year-2	0	0	Winter
2/2/2019	BL	1M	2	2019	Year-2	0	0	Winter

Date	Species	Site	Month	Year	Survey Year	Presence	Data Gap	Season
2/2/2019	BL	2M	2	2019	Year-2	0	0	Winter
2/2/2019	BL	3M	2	2019	Year-2	0	0	Winter
2/2/2019	BL	4M	2	2019	Year-2	0	0	Winter
2/2/2019	BL	5M	2	2019	Year-2	0	0	Winter
2/2/2019	BL	6M	2	2019	Year-2	0	0	Winter
2/2/2019	BL	7M	2	2019	Year-2	0	0	Winter
2/2/2019	BL	10M	2	2019	Year-2	0	0	Winter
2/2/2019	BL	12M	2	2019	Year-2	0	0	Winter
2/2/2019	BL	14M	2	2019	Year-2	0	0	Winter
2/2/2019	BL	8A	2	2019	Year-2		1	Winter
2/2/2019	BL	9A	2	2019	Year-2	0	0	Winter
2/2/2019	BL	11A	2	2019	Year-2	0	0	Winter
2/2/2019	BL	13A	2	2019	Year-2	0	0	Winter
2/3/2019	BL	1M	2	2019	Year-2	0	0	Winter
2/3/2019	BL	2M	2	2019	Year-2	0	0	Winter
2/3/2019	BL	3M	2	2019	Year-2	0	0	Winter
2/3/2019	BL	4M	2	2019	Year-2	0	0	Winter
2/3/2019	BL	5M	2	2019	Year-2	0	0	Winter
2/3/2019	BL	6M	2	2019	Year-2	0	0	Winter
2/3/2019	BL	7M	2	2019	Year-2	0	0	Winter
2/3/2019	BL	10M	2	2019	Year-2	0	0	Winter
2/3/2019	BL	12M	2	2019	Year-2	0	0	Winter
2/3/2019	BL	14M	2	2019	Year-2	0	0	Winter
2/3/2019	BL	8A	2	2019	Year-2		1	Winter
2/3/2019	BL	9A	2	2019	Year-2	0	0	Winter
2/3/2019	BL	11A	2	2019	Year-2	0	0	Winter
2/3/2019	BL	13A	2	2019	Year-2	0	0	Winter
2/4/2019	BL	1M	2	2019	Year-2	0	0	Winter
2/4/2019	BL	2M	2	2019	Year-2	0	0	Winter
2/4/2019	BL	3M	2	2019	Year-2	0	0	Winter
2/4/2019	BL	4M	2	2019	Year-2	0	0	Winter
2/4/2019	BL	5M	2	2019	Year-2	0	0	Winter
2/4/2019	BL	6M	2	2019	Year-2	0	0	Winter
2/4/2019	BL	7M	2	2019	Year-2	0	0	Winter
2/4/2019	BL	10M	2	2019	Year-2	0	0	Winter
2/4/2019	BL	12M	2	2019	Year-2	0	0	Winter
2/4/2019	BL	14M	2	2019	Year-2	0	0	Winter
2/4/2019	BL	8A	2	2019	Year-2		1	Winter
2/4/2019	BL	9A	2	2019	Year-2	0	0	Winter
2/4/2019	BL	11A	2	2019	Year-2	0	0	Winter
2/4/2019	BL	13A	2	2019	Year-2	0	0	Winter
2/5/2019	BL	1M	2	2019	Year-2	0	0	Winter
2/5/2019	BL	2M	2	2019	Year-2	0	0	Winter
2/5/2019	BL	3M	2	2019	Year-2	0	0	Winter

Date	Species	Site	Month	Year	Survey Year	Presence	Data Gap	Season
2/5/2019	BL	4M	2	2019	Year-2	0	0	Winter
2/5/2019	BL	5M	2	2019	Year-2	0	0	Winter
2/5/2019	BL	6M	2	2019	Year-2	0	0	Winter
2/5/2019	BL	7M	2	2019	Year-2	0	0	Winter
2/5/2019	BL	10M	2	2019	Year-2	0	0	Winter
2/5/2019	BL	12M	2	2019	Year-2	0	0	Winter
2/5/2019	BL	14M	2	2019	Year-2	0	0	Winter
2/5/2019	BL	8A	2	2019	Year-2		1	Winter
2/5/2019	BL	9A	2	2019	Year-2	0	0	Winter
2/5/2019	BL	11A	2	2019	Year-2	0	0	Winter
2/5/2019	BL	13A	2	2019	Year-2	0	0	Winter
2/6/2019	BL	1M	2	2019	Year-2	0	0	Winter
2/6/2019	BL	2M	2	2019	Year-2	0	0	Winter
2/6/2019	BL	3M	2	2019	Year-2	0	0	Winter
2/6/2019	BL	4M	2	2019	Year-2	0	0	Winter
2/6/2019	BL	5M	2	2019	Year-2	0	0	Winter
2/6/2019	BL	6M	2	2019	Year-2	0	0	Winter
2/6/2019	BL	7M	2	2019	Year-2	0	0	Winter
2/6/2019	BL	10M	2	2019	Year-2	0	0	Winter
2/6/2019	BL	12M	2	2019	Year-2	0	0	Winter
2/6/2019	BL	14M	2	2019	Year-2	0	0	Winter
2/6/2019	BL	8A	2	2019	Year-2		1	Winter
2/6/2019	BL	9A	2	2019	Year-2	0	0	Winter
2/6/2019	BL	11A	2	2019	Year-2	0	0	Winter
2/6/2019	BL	13A	2	2019	Year-2	0	0	Winter
2/7/2019	BL	1M	2	2019	Year-2	0	0	Winter
2/7/2019	BL	2M	2	2019	Year-2	0	0	Winter
2/7/2019	BL	3M	2	2019	Year-2	0	0	Winter
2/7/2019	BL	4M	2	2019	Year-2	0	0	Winter
2/7/2019	BL	5M	2	2019	Year-2	0	0	Winter
2/7/2019	BL	6M	2	2019	Year-2	0	0	Winter
2/7/2019	BL	7M	2	2019	Year-2	0	0	Winter
2/7/2019	BL	10M	2	2019	Year-2	0	0	Winter
2/7/2019	BL	12M	2	2019	Year-2	0	0	Winter
2/7/2019	BL	14M	2	2019	Year-2	0	0	Winter
2/7/2019	BL	8A	2	2019	Year-2		1	Winter
2/7/2019	BL	9A	2	2019	Year-2	0	0	Winter
2/7/2019	BL	11A	2	2019	Year-2	0	0	Winter
2/7/2019	BL	13A	2	2019	Year-2	0	0	Winter
2/8/2019	BL	1M	2	2019	Year-2	0	0	Winter
2/8/2019	BL	2M	2	2019	Year-2	0	0	Winter
2/8/2019	BL	3M	2	2019	Year-2	0	0	Winter
2/8/2019	BL	4M	2	2019	Year-2	0	0	Winter
2/8/2019	BL	5M	2	2019	Year-2	0	0	Winter

Date	Species	Site	Month	Year	Survey Year	Presence	Data Gap	Season
2/8/2019	BL	6M	2	2019	Year-2	0	0	Winter
2/8/2019	BL	7M	2	2019	Year-2	0	0	Winter
2/8/2019	BL	10M	2	2019	Year-2	0	0	Winter
2/8/2019	BL	12M	2	2019	Year-2	0	0	Winter
2/8/2019	BL	14M	2	2019	Year-2	0	0	Winter
2/8/2019	BL	8A	2	2019	Year-2		1	Winter
2/8/2019	BL	9A	2	2019	Year-2	0	0	Winter
2/8/2019	BL	11A	2	2019	Year-2	0	0	Winter
2/8/2019	BL	13A	2	2019	Year-2	0	0	Winter
2/9/2019	BL	1M	2	2019	Year-2	0	0	Winter
2/9/2019	BL	2M	2	2019	Year-2	0	0	Winter
2/9/2019	BL	3M	2	2019	Year-2	0	0	Winter
2/9/2019	BL	4M	2	2019	Year-2	0	0	Winter
2/9/2019	BL	5M	2	2019	Year-2	0	0	Winter
2/9/2019	BL	6M	2	2019	Year-2	0	0	Winter
2/9/2019	BL	7M	2	2019	Year-2	0	0	Winter
2/9/2019	BL	10M	2	2019	Year-2	0	0	Winter
2/9/2019	BL	12M	2	2019	Year-2	0	0	Winter
2/9/2019	BL	14M	2	2019	Year-2	0	0	Winter
2/9/2019	BL	8A	2	2019	Year-2		1	Winter
2/9/2019	BL	9A	2	2019	Year-2	0	0	Winter
2/9/2019	BL	11A	2	2019	Year-2	0	0	Winter
2/9/2019	BL	13A	2	2019	Year-2	0	0	Winter
2/10/2019	BL	1M	2	2019	Year-2	0	0	Winter
2/10/2019	BL	2M	2	2019	Year-2	0	0	Winter
2/10/2019	BL	3M	2	2019	Year-2	0	0	Winter
2/10/2019	BL	4M	2	2019	Year-2	0	0	Winter
2/10/2019	BL	5M	2	2019	Year-2	0	0	Winter
2/10/2019	BL	6M	2	2019	Year-2	0	0	Winter
2/10/2019	BL	7M	2	2019	Year-2	0	0	Winter
2/10/2019	BL	10M	2	2019	Year-2	0	0	Winter
2/10/2019	BL	12M	2	2019	Year-2	0	0	Winter
2/10/2019	BL	14M	2	2019	Year-2	0	0	Winter
2/10/2019	BL	8A	2	2019	Year-2		1	Winter
2/10/2019	BL	9A	2	2019	Year-2	0	0	Winter
2/10/2019	BL	11A	2	2019	Year-2	0	0	Winter
2/10/2019	BL	13A	2	2019	Year-2	0	0	Winter
2/11/2019	BL	1M	2	2019	Year-2	0	0	Winter
2/11/2019	BL	2M	2	2019	Year-2	0	0	Winter
2/11/2019	BL	3M	2	2019	Year-2	0	0	Winter
2/11/2019	BL	4M	2	2019	Year-2	0	0	Winter
2/11/2019	BL	5M	2	2019	Year-2	0	0	Winter
2/11/2019	BL	6M	2	2019	Year-2	0	0	Winter
2/11/2019	BL	7M	2	2019	Year-2	0	0	Winter

Date	Species	Site	Month	Year	Survey Year	Presence	Data Gap	Season
2/11/2019	BL	10M	2	2019	Year-2	0	0	Winter
2/11/2019	BL	12M	2	2019	Year-2	0	0	Winter
2/11/2019	BL	14M	2	2019	Year-2	0	0	Winter
2/11/2019	BL	8A	2	2019	Year-2		1	Winter
2/11/2019	BL	9A	2	2019	Year-2	0	0	Winter
2/11/2019	BL	11A	2	2019	Year-2	0	0	Winter
2/11/2019	BL	13A	2	2019	Year-2	0	0	Winter
2/12/2019	BL	1M	2	2019	Year-2	0	0	Winter
2/12/2019	BL	2M	2	2019	Year-2	0	0	Winter
2/12/2019	BL	3M	2	2019	Year-2	0	0	Winter
2/12/2019	BL	4M	2	2019	Year-2	0	0	Winter
2/12/2019	BL	5M	2	2019	Year-2	0	0	Winter
2/12/2019	BL	6M	2	2019	Year-2	0	0	Winter
2/12/2019	BL	7M	2	2019	Year-2	0	0	Winter
2/12/2019	BL	10M	2	2019	Year-2	0	0	Winter
2/12/2019	BL	12M	2	2019	Year-2	0	0	Winter
2/12/2019	BL	14M	2	2019	Year-2	0	0	Winter
2/12/2019	BL	8A	2	2019	Year-2		1	Winter
2/12/2019	BL	9A	2	2019	Year-2	0	0	Winter
2/12/2019	BL	11A	2	2019	Year-2	0	0	Winter
2/12/2019	BL	13A	2	2019	Year-2	0	0	Winter
2/13/2019	BL	1M	2	2019	Year-2	0	0	Winter
2/13/2019	BL	2M	2	2019	Year-2	0	0	Winter
2/13/2019	BL	3M	2	2019	Year-2	0	0	Winter
2/13/2019	BL	4M	2	2019	Year-2	0	0	Winter
2/13/2019	BL	5M	2	2019	Year-2	0	0	Winter
2/13/2019	BL	6M	2	2019	Year-2	0	0	Winter
2/13/2019	BL	7M	2	2019	Year-2	0	0	Winter
2/13/2019	BL	10M	2	2019	Year-2	0	0	Winter
2/13/2019	BL	12M	2	2019	Year-2	0	0	Winter
2/13/2019	BL	14M	2	2019	Year-2	0	0	Winter
2/13/2019	BL	8A	2	2019	Year-2		1	Winter
2/13/2019	BL	9A	2	2019	Year-2	0	0	Winter
2/13/2019	BL	11A	2	2019	Year-2	0	0	Winter
2/13/2019	BL	13A	2	2019	Year-2	0	0	Winter
2/14/2019	BL	1M	2	2019	Year-2	0	0	Winter
2/14/2019	BL	2M	2	2019	Year-2	0	0	Winter
2/14/2019	BL	3M	2	2019	Year-2	0	0	Winter
2/14/2019	BL	4M	2	2019	Year-2	0	0	Winter
2/14/2019	BL	5M	2	2019	Year-2	0	0	Winter
2/14/2019	BL	6M	2	2019	Year-2	0	0	Winter
2/14/2019	BL	7M	2	2019	Year-2	0	0	Winter
2/14/2019	BL	10M	2	2019	Year-2	0	0	Winter
2/14/2019	BL	12M	2	2019	Year-2	0	0	Winter

Date	Species	Site	Month	Year	Survey Year	Presence	Data Gap	Season
2/14/2019	BL	14M	2	2019	Year-2	0	0	Winter
2/14/2019	BL	8A	2	2019	Year-2		1	Winter
2/14/2019	BL	9A	2	2019	Year-2	0	0	Winter
2/14/2019	BL	11A	2	2019	Year-2	0	0	Winter
2/14/2019	BL	13A	2	2019	Year-2	0	0	Winter
2/15/2019	BL	1M	2	2019	Year-2	0	0	Winter
2/15/2019	BL	2M	2	2019	Year-2	0	0	Winter
2/15/2019	BL	3M	2	2019	Year-2	0	0	Winter
2/15/2019	BL	4M	2	2019	Year-2	0	0	Winter
2/15/2019	BL	5M	2	2019	Year-2	0	0	Winter
2/15/2019	BL	6M	2	2019	Year-2	0	0	Winter
2/15/2019	BL	7M	2	2019	Year-2	0	0	Winter
2/15/2019	BL	10M	2	2019	Year-2	0	0	Winter
2/15/2019	BL	12M	2	2019	Year-2	0	0	Winter
2/15/2019	BL	14M	2	2019	Year-2	0	0	Winter
2/15/2019	BL	8A	2	2019	Year-2		1	Winter
2/15/2019	BL	9A	2	2019	Year-2	0	0	Winter
2/15/2019	BL	11A	2	2019	Year-2	0	0	Winter
2/15/2019	BL	13A	2	2019	Year-2	0	0	Winter
2/16/2019	BL	1M	2	2019	Year-2	0	0	Winter
2/16/2019	BL	2M	2	2019	Year-2	0	0	Winter
2/16/2019	BL	3M	2	2019	Year-2	0	0	Winter
2/16/2019	BL	4M	2	2019	Year-2	0	0	Winter
2/16/2019	BL	5M	2	2019	Year-2	0	0	Winter
2/16/2019	BL	6M	2	2019	Year-2	0	0	Winter
2/16/2019	BL	7M	2	2019	Year-2	0	0	Winter
2/16/2019	BL	10M	2	2019	Year-2	0	0	Winter
2/16/2019	BL	12M	2	2019	Year-2	0	0	Winter
2/16/2019	BL	14M	2	2019	Year-2	0	0	Winter
2/16/2019	BL	8A	2	2019	Year-2		1	Winter
2/16/2019	BL	9A	2	2019	Year-2	0	0	Winter
2/16/2019	BL	11A	2	2019	Year-2	0	0	Winter
2/16/2019	BL	13A	2	2019	Year-2	0	0	Winter
2/17/2019	BL	1M	2	2019	Year-2	0	0	Winter
2/17/2019	BL	2M	2	2019	Year-2	0	0	Winter
2/17/2019	BL	3M	2	2019	Year-2	0	0	Winter
2/17/2019	BL	4M	2	2019	Year-2	0	0	Winter
2/17/2019	BL	5M	2	2019	Year-2	0	0	Winter
2/17/2019	BL	6M	2	2019	Year-2	0	0	Winter
2/17/2019	BL	7M	2	2019	Year-2	0	0	Winter
2/17/2019	BL	10M	2	2019	Year-2	0	0	Winter
2/17/2019	BL	12M	2	2019	Year-2	0	0	Winter
2/17/2019	BL	14M	2	2019	Year-2	1	0	Winter
2/17/2019	BL	8A	2	2019	Year-2		1	Winter

Date	Species	Site	Month	Year	Survey Year	Presence	Data Gap	Season
2/17/2019	BL	9A	2	2019	Year-2	0	0	Winter
2/17/2019	BL	11A	2	2019	Year-2	0	0	Winter
2/17/2019	BL	13A	2	2019	Year-2	0	0	Winter
2/18/2019	BL	1M	2	2019	Year-2	0	0	Winter
2/18/2019	BL	2M	2	2019	Year-2	0	0	Winter
2/18/2019	BL	3M	2	2019	Year-2	0	0	Winter
2/18/2019	BL	4M	2	2019	Year-2	0	0	Winter
2/18/2019	BL	5M	2	2019	Year-2	0	0	Winter
2/18/2019	BL	6M	2	2019	Year-2	0	0	Winter
2/18/2019	BL	7M	2	2019	Year-2	0	0	Winter
2/18/2019	BL	10M	2	2019	Year-2	0	0	Winter
2/18/2019	BL	12M	2	2019	Year-2	0	0	Winter
2/18/2019	BL	14M	2	2019	Year-2	0	0	Winter
2/18/2019	BL	8A	2	2019	Year-2		1	Winter
2/18/2019	BL	9A	2	2019	Year-2	0	0	Winter
2/18/2019	BL	11A	2	2019	Year-2	0	0	Winter
2/18/2019	BL	13A	2	2019	Year-2	0	0	Winter
2/19/2019	BL	1M	2	2019	Year-2	0	0	Winter
2/19/2019	BL	2M	2	2019	Year-2	0	0	Winter
2/19/2019	BL	3M	2	2019	Year-2	0	0	Winter
2/19/2019	BL	4M	2	2019	Year-2	0	0	Winter
2/19/2019	BL	5M	2	2019	Year-2	0	0	Winter
2/19/2019	BL	6M	2	2019	Year-2	0	0	Winter
2/19/2019	BL	7M	2	2019	Year-2	0	0	Winter
2/19/2019	BL	10M	2	2019	Year-2	0	0	Winter
2/19/2019	BL	12M	2	2019	Year-2	0	0	Winter
2/19/2019	BL	14M	2	2019	Year-2	0	0	Winter
2/19/2019	BL	8A	2	2019	Year-2		1	Winter
2/19/2019	BL	9A	2	2019	Year-2	0	0	Winter
2/19/2019	BL	11A	2	2019	Year-2	0	0	Winter
2/19/2019	BL	13A	2	2019	Year-2	0	0	Winter
2/20/2019	BL	1M	2	2019	Year-2	0	0	Winter
2/20/2019	BL	2M	2	2019	Year-2	0	0	Winter
2/20/2019	BL	3M	2	2019	Year-2	0	0	Winter
2/20/2019	BL	4M	2	2019	Year-2	0	0	Winter
2/20/2019	BL	5M	2	2019	Year-2	0	0	Winter
2/20/2019	BL	6M	2	2019	Year-2	0	0	Winter
2/20/2019	BL	7M	2	2019	Year-2	0	0	Winter
2/20/2019	BL	10M	2	2019	Year-2	0	0	Winter
2/20/2019	BL	12M	2	2019	Year-2	0	0	Winter
2/20/2019	BL	14M	2	2019	Year-2	0	0	Winter
2/20/2019	BL	8A	2	2019	Year-2		1	Winter
2/20/2019	BL	9A	2	2019	Year-2	0	0	Winter
2/20/2019	BL	11A	2	2019	Year-2	0	0	Winter

Date	Species	Site	Month	Year	Survey Year	Presence	Data Gap	Season
2/20/2019	BL	13A	2	2019	Year-2	0	0	Winter
2/21/2019	BL	1M	2	2019	Year-2	0	0	Winter
2/21/2019	BL	2M	2	2019	Year-2	0	0	Winter
2/21/2019	BL	3M	2	2019	Year-2	0	0	Winter
2/21/2019	BL	4M	2	2019	Year-2	0	0	Winter
2/21/2019	BL	5M	2	2019	Year-2	0	0	Winter
2/21/2019	BL	6M	2	2019	Year-2	0	0	Winter
2/21/2019	BL	7M	2	2019	Year-2	0	0	Winter
2/21/2019	BL	10M	2	2019	Year-2	0	0	Winter
2/21/2019	BL	12M	2	2019	Year-2	0	0	Winter
2/21/2019	BL	14M	2	2019	Year-2	0	0	Winter
2/21/2019	BL	8A	2	2019	Year-2		1	Winter
2/21/2019	BL	9A	2	2019	Year-2	0	0	Winter
2/21/2019	BL	11A	2	2019	Year-2	0	0	Winter
2/21/2019	BL	13A	2	2019	Year-2	0	0	Winter
2/22/2019	BL	1M	2	2019	Year-2	0	0	Winter
2/22/2019	BL	2M	2	2019	Year-2	0	0	Winter
2/22/2019	BL	3M	2	2019	Year-2	0	0	Winter
2/22/2019	BL	4M	2	2019	Year-2	0	0	Winter
2/22/2019	BL	5M	2	2019	Year-2	0	0	Winter
2/22/2019	BL	6M	2	2019	Year-2	0	0	Winter
2/22/2019	BL	7M	2	2019	Year-2	0	0	Winter
2/22/2019	BL	10M	2	2019	Year-2	0	0	Winter
2/22/2019	BL	12M	2	2019	Year-2	0	0	Winter
2/22/2019	BL	14M	2	2019	Year-2	0	0	Winter
2/22/2019	BL	8A	2	2019	Year-2		1	Winter
2/22/2019	BL	9A	2	2019	Year-2	0	0	Winter
2/22/2019	BL	11A	2	2019	Year-2	0	0	Winter
2/22/2019	BL	13A	2	2019	Year-2	0	0	Winter
2/23/2019	BL	1M	2	2019	Year-2	0	0	Winter
2/23/2019	BL	2M	2	2019	Year-2	0	0	Winter
2/23/2019	BL	3M	2	2019	Year-2	0	0	Winter
2/23/2019	BL	4M	2	2019	Year-2	0	0	Winter
2/23/2019	BL	5M	2	2019	Year-2	0	0	Winter
2/23/2019	BL	6M	2	2019	Year-2	0	0	Winter
2/23/2019	BL	7M	2	2019	Year-2	0	0	Winter
2/23/2019	BL	10M	2	2019	Year-2	0	0	Winter
2/23/2019	BL	12M	2	2019	Year-2	0	0	Winter
2/23/2019	BL	14M	2	2019	Year-2	0	0	Winter
2/23/2019	BL	8A	2	2019	Year-2		1	Winter
2/23/2019	BL	9A	2	2019	Year-2	0	0	Winter
2/23/2019	BL	11A	2	2019	Year-2	0	0	Winter
2/23/2019	BL	13A	2	2019	Year-2	0	0	Winter
2/24/2019	BL	1M	2	2019	Year-2	0	0	Winter

Date	Species	Site	Month	Year	Survey Year	Presence	Data Gap	Season
2/24/2019	BL	2M	2	2019	Year-2	0	0	Winter
2/24/2019	BL	3M	2	2019	Year-2	0	0	Winter
2/24/2019	BL	4M	2	2019	Year-2	0	0	Winter
2/24/2019	BL	5M	2	2019	Year-2	0	0	Winter
2/24/2019	BL	6M	2	2019	Year-2	0	0	Winter
2/24/2019	BL	7M	2	2019	Year-2	0	0	Winter
2/24/2019	BL	10M	2	2019	Year-2	0	0	Winter
2/24/2019	BL	12M	2	2019	Year-2	0	0	Winter
2/24/2019	BL	14M	2	2019	Year-2	0	0	Winter
2/24/2019	BL	8A	2	2019	Year-2		1	Winter
2/24/2019	BL	9A	2	2019	Year-2	0	0	Winter
2/24/2019	BL	11A	2	2019	Year-2	0	0	Winter
2/24/2019	BL	13A	2	2019	Year-2	0	0	Winter
2/25/2019	BL	1M	2	2019	Year-2	0	0	Winter
2/25/2019	BL	2M	2	2019	Year-2	0	0	Winter
2/25/2019	BL	3M	2	2019	Year-2	0	0	Winter
2/25/2019	BL	4M	2	2019	Year-2	0	0	Winter
2/25/2019	BL	5M	2	2019	Year-2	0	0	Winter
2/25/2019	BL	6M	2	2019	Year-2	0	0	Winter
2/25/2019	BL	7M	2	2019	Year-2	0	0	Winter
2/25/2019	BL	10M	2	2019	Year-2	0	0	Winter
2/25/2019	BL	12M	2	2019	Year-2	0	0	Winter
2/25/2019	BL	14M	2	2019	Year-2	0	0	Winter
2/25/2019	BL	8A	2	2019	Year-2		1	Winter
2/25/2019	BL	9A	2	2019	Year-2	0	0	Winter
2/25/2019	BL	11A	2	2019	Year-2	0	0	Winter
2/25/2019	BL	13A	2	2019	Year-2	0	0	Winter
2/26/2019	BL	1M	2	2019	Year-2	0	0	Winter
2/26/2019	BL	2M	2	2019	Year-2	0	0	Winter
2/26/2019	BL	3M	2	2019	Year-2	0	0	Winter
2/26/2019	BL	4M	2	2019	Year-2	0	0	Winter
2/26/2019	BL	5M	2	2019	Year-2	0	0	Winter
2/26/2019	BL	6M	2	2019	Year-2	0	0	Winter
2/26/2019	BL	7M	2	2019	Year-2	0	0	Winter
2/26/2019	BL	10M	2	2019	Year-2	0	0	Winter
2/26/2019	BL	12M	2	2019	Year-2	0	0	Winter
2/26/2019	BL	14M	2	2019	Year-2	0	0	Winter
2/26/2019	BL	8A	2	2019	Year-2		1	Winter
2/26/2019	BL	9A	2	2019	Year-2	0	0	Winter
2/26/2019	BL	11A	2	2019	Year-2	0	0	Winter
2/26/2019	BL	13A	2	2019	Year-2	0	0	Winter
2/27/2019	BL	1M	2	2019	Year-2	0	0	Winter
2/27/2019	BL	2M	2	2019	Year-2	0	0	Winter
2/27/2019	BL	3M	2	2019	Year-2	0	0	Winter

Date	Species	Site	Month	Year	Survey Year	Presence	Data Gap	Season
2/27/2019	BL	4M	2	2019	Year-2	0	0	Winter
2/27/2019	BL	5M	2	2019	Year-2	0	0	Winter
2/27/2019	BL	6M	2	2019	Year-2	0	0	Winter
2/27/2019	BL	7M	2	2019	Year-2	0	0	Winter
2/27/2019	BL	10M	2	2019	Year-2	0	0	Winter
2/27/2019	BL	12M	2	2019	Year-2	0	0	Winter
2/27/2019	BL	14M	2	2019	Year-2	0	0	Winter
2/27/2019	BL	8A	2	2019	Year-2		1	Winter
2/27/2019	BL	9A	2	2019	Year-2	0	0	Winter
2/27/2019	BL	11A	2	2019	Year-2	0	0	Winter
2/27/2019	BL	13A	2	2019	Year-2	0	0	Winter
2/28/2019	BL	1M	2	2019	Year-2	0	0	Winter
2/28/2019	BL	2M	2	2019	Year-2	0	0	Winter
2/28/2019	BL	3M	2	2019	Year-2	0	0	Winter
2/28/2019	BL	4M	2	2019	Year-2	0	0	Winter
2/28/2019	BL	5M	2	2019	Year-2	0	0	Winter
2/28/2019	BL	6M	2	2019	Year-2	0	0	Winter
2/28/2019	BL	7M	2	2019	Year-2	0	0	Winter
2/28/2019	BL	10M	2	2019	Year-2	0	0	Winter
2/28/2019	BL	12M	2	2019	Year-2	0	0	Winter
2/28/2019	BL	14M	2	2019	Year-2	0	0	Winter
2/28/2019	BL	8A	2	2019	Year-2		1	Winter
2/28/2019	BL	9A	2	2019	Year-2	0	0	Winter
2/28/2019	BL	11A	2	2019	Year-2	0	0	Winter
2/28/2019	BL	13A	2	2019	Year-2	0	0	Winter
3/1/2019	BL	1M	3	2019	Year-2	0	0	Spring
3/1/2019	BL	2M	3	2019	Year-2	0	0	Spring
3/1/2019	BL	3M	3	2019	Year-2	0	0	Spring
3/1/2019	BL	4M	3	2019	Year-2	0	0	Spring
3/1/2019	BL	5M	3	2019	Year-2	0	0	Spring
3/1/2019	BL	6M	3	2019	Year-2	0	0	Spring
3/1/2019	BL	7M	3	2019	Year-2	0	0	Spring
3/1/2019	BL	10M	3	2019	Year-2	0	0	Spring
3/1/2019	BL	12M	3	2019	Year-2	0	0	Spring
3/1/2019	BL	14M	3	2019	Year-2	0	0	Spring
3/1/2019	BL	8A	3	2019	Year-2		1	Spring
3/1/2019	BL	9A	3	2019	Year-2	0	0	Spring
3/1/2019	BL	11A	3	2019	Year-2	0	0	Spring
3/1/2019	BL	13A	3	2019	Year-2	0	0	Spring
3/2/2019	BL	1M	3	2019	Year-2	0	0	Spring
3/2/2019	BL	2M	3	2019	Year-2	0	0	Spring
3/2/2019	BL	3M	3	2019	Year-2	0	0	Spring
3/2/2019	BL	4M	3	2019	Year-2	0	0	Spring
3/2/2019	BL	5M	3	2019	Year-2	0	0	Spring

Date	Species	Site	Month	Year	Survey Year	Presence	Data Gap	Season
3/2/2019	BL	6M	3	2019	Year-2	0	0	Spring
3/2/2019	BL	7M	3	2019	Year-2	0	0	Spring
3/2/2019	BL	10M	3	2019	Year-2	0	0	Spring
3/2/2019	BL	12M	3	2019	Year-2	0	0	Spring
3/2/2019	BL	14M	3	2019	Year-2	0	0	Spring
3/2/2019	BL	8A	3	2019	Year-2		1	Spring
3/2/2019	BL	9A	3	2019	Year-2	0	0	Spring
3/2/2019	BL	11A	3	2019	Year-2	0	0	Spring
3/2/2019	BL	13A	3	2019	Year-2	0	0	Spring
3/3/2019	BL	1M	3	2019	Year-2	0	0	Spring
3/3/2019	BL	2M	3	2019	Year-2	0	0	Spring
3/3/2019	BL	3M	3	2019	Year-2	0	0	Spring
3/3/2019	BL	4M	3	2019	Year-2	0	0	Spring
3/3/2019	BL	5M	3	2019	Year-2	0	0	Spring
3/3/2019	BL	6M	3	2019	Year-2	0	0	Spring
3/3/2019	BL	7M	3	2019	Year-2	0	0	Spring
3/3/2019	BL	10M	3	2019	Year-2	0	0	Spring
3/3/2019	BL	12M	3	2019	Year-2	0	0	Spring
3/3/2019	BL	14M	3	2019	Year-2	0	0	Spring
3/3/2019	BL	8A	3	2019	Year-2		1	Spring
3/3/2019	BL	9A	3	2019	Year-2	0	0	Spring
3/3/2019	BL	11A	3	2019	Year-2	0	0	Spring
3/3/2019	BL	13A	3	2019	Year-2	0	0	Spring
3/4/2019	BL	1M	3	2019	Year-2	0	0	Spring
3/4/2019	BL	2M	3	2019	Year-2	0	0	Spring
3/4/2019	BL	3M	3	2019	Year-2	0	0	Spring
3/4/2019	BL	4M	3	2019	Year-2	0	0	Spring
3/4/2019	BL	5M	3	2019	Year-2	0	0	Spring
3/4/2019	BL	6M	3	2019	Year-2	0	0	Spring
3/4/2019	BL	7M	3	2019	Year-2	0	0	Spring
3/4/2019	BL	10M	3	2019	Year-2	0	0	Spring
3/4/2019	BL	12M	3	2019	Year-2	0	0	Spring
3/4/2019	BL	14M	3	2019	Year-2	0	0	Spring
3/4/2019	BL	8A	3	2019	Year-2		1	Spring
3/4/2019	BL	9A	3	2019	Year-2	0	0	Spring
3/4/2019	BL	11A	3	2019	Year-2	0	0	Spring
3/4/2019	BL	13A	3	2019	Year-2	0	0	Spring
3/5/2019	BL	1M	3	2019	Year-2	0	0	Spring
3/5/2019	BL	2M	3	2019	Year-2	0	0	Spring
3/5/2019	BL	3M	3	2019	Year-2	0	0	Spring
3/5/2019	BL	4M	3	2019	Year-2	0	0	Spring
3/5/2019	BL	5M	3	2019	Year-2	0	0	Spring
3/5/2019	BL	6M	3	2019	Year-2	0	0	Spring
3/5/2019	BL	7M	3	2019	Year-2	0	0	Spring

Date	Species	Site	Month	Year	Survey Year	Presence	Data Gap	Season
3/5/2019	BL	10M	3	2019	Year-2	0	0	Spring
3/5/2019	BL	12M	3	2019	Year-2	0	0	Spring
3/5/2019	BL	14M	3	2019	Year-2	0	0	Spring
3/5/2019	BL	8A	3	2019	Year-2		1	Spring
3/5/2019	BL	9A	3	2019	Year-2	0	0	Spring
3/5/2019	BL	11A	3	2019	Year-2	0	0	Spring
3/5/2019	BL	13A	3	2019	Year-2	0	0	Spring
3/6/2019	BL	1M	3	2019	Year-2	0	0	Spring
3/6/2019	BL	2M	3	2019	Year-2	0	0	Spring
3/6/2019	BL	3M	3	2019	Year-2	0	0	Spring
3/6/2019	BL	4M	3	2019	Year-2	0	0	Spring
3/6/2019	BL	5M	3	2019	Year-2	0	0	Spring
3/6/2019	BL	6M	3	2019	Year-2	0	0	Spring
3/6/2019	BL	7M	3	2019	Year-2	0	0	Spring
3/6/2019	BL	10M	3	2019	Year-2	0	0	Spring
3/6/2019	BL	12M	3	2019	Year-2	0	0	Spring
3/6/2019	BL	14M	3	2019	Year-2	0	0	Spring
3/6/2019	BL	8A	3	2019	Year-2		1	Spring
3/6/2019	BL	9A	3	2019	Year-2	0	0	Spring
3/6/2019	BL	11A	3	2019	Year-2	0	0	Spring
3/6/2019	BL	13A	3	2019	Year-2	0	0	Spring
3/7/2019	BL	1M	3	2019	Year-2	0	0	Spring
3/7/2019	BL	2M	3	2019	Year-2	0	0	Spring
3/7/2019	BL	3M	3	2019	Year-2	0	0	Spring
3/7/2019	BL	4M	3	2019	Year-2	0	0	Spring
3/7/2019	BL	5M	3	2019	Year-2	0	0	Spring
3/7/2019	BL	6M	3	2019	Year-2	0	0	Spring
3/7/2019	BL	7M	3	2019	Year-2	0	0	Spring
3/7/2019	BL	10M	3	2019	Year-2	0	0	Spring
3/7/2019	BL	12M	3	2019	Year-2	0	0	Spring
3/7/2019	BL	14M	3	2019	Year-2	0	0	Spring
3/7/2019	BL	8A	3	2019	Year-2		1	Spring
3/7/2019	BL	9A	3	2019	Year-2	0	0	Spring
3/7/2019	BL	11A	3	2019	Year-2	0	0	Spring
3/7/2019	BL	13A	3	2019	Year-2	0	0	Spring
3/8/2019	BL	1M	3	2019	Year-2	0	0	Spring
3/8/2019	BL	2M	3	2019	Year-2	0	0	Spring
3/8/2019	BL	3M	3	2019	Year-2	0	0	Spring
3/8/2019	BL	4M	3	2019	Year-2	0	0	Spring
3/8/2019	BL	5M	3	2019	Year-2	0	0	Spring
3/8/2019	BL	6M	3	2019	Year-2	0	0	Spring
3/8/2019	BL	7M	3	2019	Year-2	0	0	Spring
3/8/2019	BL	10M	3	2019	Year-2	0	0	Spring
3/8/2019	BL	12M	3	2019	Year-2	0	0	Spring

Date	Species	Site	Month	Year	Survey Year	Presence	Data Gap	Season
3/8/2019	BL	14M	3	2019	Year-2	0	0	Spring
3/8/2019	BL	8A	3	2019	Year-2		1	Spring
3/8/2019	BL	9A	3	2019	Year-2	0	0	Spring
3/8/2019	BL	11A	3	2019	Year-2	0	0	Spring
3/8/2019	BL	13A	3	2019	Year-2	0	0	Spring
3/9/2019	BL	1M	3	2019	Year-2	0	0	Spring
3/9/2019	BL	2M	3	2019	Year-2	0	0	Spring
3/9/2019	BL	3M	3	2019	Year-2	0	0	Spring
3/9/2019	BL	4M	3	2019	Year-2	0	0	Spring
3/9/2019	BL	5M	3	2019	Year-2	0	0	Spring
3/9/2019	BL	6M	3	2019	Year-2	0	0	Spring
3/9/2019	BL	7M	3	2019	Year-2	0	0	Spring
3/9/2019	BL	10M	3	2019	Year-2	0	0	Spring
3/9/2019	BL	12M	3	2019	Year-2	0	0	Spring
3/9/2019	BL	14M	3	2019	Year-2	0	0	Spring
3/9/2019	BL	8A	3	2019	Year-2		1	Spring
3/9/2019	BL	9A	3	2019	Year-2	0	0	Spring
3/9/2019	BL	11A	3	2019	Year-2	0	0	Spring
3/9/2019	BL	13A	3	2019	Year-2	0	0	Spring
3/10/2019	BL	1M	3	2019	Year-2	0	0	Spring
3/10/2019	BL	2M	3	2019	Year-2	0	0	Spring
3/10/2019	BL	3M	3	2019	Year-2	0	0	Spring
3/10/2019	BL	4M	3	2019	Year-2	0	0	Spring
3/10/2019	BL	5M	3	2019	Year-2	0	0	Spring
3/10/2019	BL	6M	3	2019	Year-2	0	0	Spring
3/10/2019	BL	7M	3	2019	Year-2	0	0	Spring
3/10/2019	BL	10M	3	2019	Year-2	0	0	Spring
3/10/2019	BL	12M	3	2019	Year-2	0	0	Spring
3/10/2019	BL	14M	3	2019	Year-2	0	0	Spring
3/10/2019	BL	8A	3	2019	Year-2		1	Spring
3/10/2019	BL	9A	3	2019	Year-2	0	0	Spring
3/10/2019	BL	11A	3	2019	Year-2	0	0	Spring
3/10/2019	BL	13A	3	2019	Year-2	0	0	Spring
3/11/2019	BL	1M	3	2019	Year-2	0	0	Spring
3/11/2019	BL	2M	3	2019	Year-2	0	0	Spring
3/11/2019	BL	3M	3	2019	Year-2	0	0	Spring
3/11/2019	BL	4M	3	2019	Year-2	0	0	Spring
3/11/2019	BL	5M	3	2019	Year-2	0	0	Spring
3/11/2019	BL	6M	3	2019	Year-2	0	0	Spring
3/11/2019	BL	7M	3	2019	Year-2	0	0	Spring
3/11/2019	BL	10M	3	2019	Year-2	0	0	Spring
3/11/2019	BL	12M	3	2019	Year-2	0	0	Spring
3/11/2019	BL	14M	3	2019	Year-2	0	0	Spring
3/11/2019	BL	8A	3	2019	Year-2		1	Spring

Date	Species	Site	Month	Year	Survey Year	Presence	Data Gap	Season
3/11/2019	BL	9A	3	2019	Year-2	0	0	Spring
3/11/2019	BL	11A	3	2019	Year-2	0	0	Spring
3/11/2019	BL	13A	3	2019	Year-2	0	0	Spring
3/12/2019	BL	1M	3	2019	Year-2	0	0	Spring
3/12/2019	BL	2M	3	2019	Year-2	0	0	Spring
3/12/2019	BL	3M	3	2019	Year-2	0	0	Spring
3/12/2019	BL	4M	3	2019	Year-2	0	0	Spring
3/12/2019	BL	5M	3	2019	Year-2	0	0	Spring
3/12/2019	BL	6M	3	2019	Year-2	0	0	Spring
3/12/2019	BL	7M	3	2019	Year-2	0	0	Spring
3/12/2019	BL	10M	3	2019	Year-2	0	0	Spring
3/12/2019	BL	12M	3	2019	Year-2	0	0	Spring
3/12/2019	BL	14M	3	2019	Year-2	0	0	Spring
3/12/2019	BL	8A	3	2019	Year-2		1	Spring
3/12/2019	BL	9A	3	2019	Year-2	0	0	Spring
3/12/2019	BL	11A	3	2019	Year-2	0	0	Spring
3/12/2019	BL	13A	3	2019	Year-2	0	0	Spring
3/13/2019	BL	1M	3	2019	Year-2	0	0	Spring
3/13/2019	BL	2M	3	2019	Year-2	0	0	Spring
3/13/2019	BL	3M	3	2019	Year-2	0	0	Spring
3/13/2019	BL	4M	3	2019	Year-2	0	0	Spring
3/13/2019	BL	5M	3	2019	Year-2	0	0	Spring
3/13/2019	BL	6M	3	2019	Year-2	0	0	Spring
3/13/2019	BL	7M	3	2019	Year-2	0	0	Spring
3/13/2019	BL	10M	3	2019	Year-2	0	0	Spring
3/13/2019	BL	12M	3	2019	Year-2	0	0	Spring
3/13/2019	BL	14M	3	2019	Year-2	0	0	Spring
3/13/2019	BL	8A	3	2019	Year-2		1	Spring
3/13/2019	BL	9A	3	2019	Year-2	0	0	Spring
3/13/2019	BL	11A	3	2019	Year-2	0	0	Spring
3/13/2019	BL	13A	3	2019	Year-2	0	0	Spring
3/14/2019	BL	1M	3	2019	Year-2	0	0	Spring
3/14/2019	BL	2M	3	2019	Year-2	0	0	Spring
3/14/2019	BL	3M	3	2019	Year-2	0	0	Spring
3/14/2019	BL	4M	3	2019	Year-2	0	0	Spring
3/14/2019	BL	5M	3	2019	Year-2	0	0	Spring
3/14/2019	BL	6M	3	2019	Year-2	0	0	Spring
3/14/2019	BL	7M	3	2019	Year-2	0	0	Spring
3/14/2019	BL	10M	3	2019	Year-2	0	0	Spring
3/14/2019	BL	12M	3	2019	Year-2	0	0	Spring
3/14/2019	BL	14M	3	2019	Year-2	0	0	Spring
3/14/2019	BL	8A	3	2019	Year-2	0	0	Spring
3/14/2019	BL	9A	3	2019	Year-2	0	0	Spring
3/14/2019	BL	11A	3	2019	Year-2		1	Spring

Date	Species	Site	Month	Year	Survey Year	Presence	Data Gap	Season
3/14/2019	BL	13A	3	2019	Year-2		1	Spring
3/15/2019	BL	1M	3	2019	Year-2	0	0	Spring
3/15/2019	BL	2M	3	2019	Year-2	0	0	Spring
3/15/2019	BL	3M	3	2019	Year-2	0	0	Spring
3/15/2019	BL	4M	3	2019	Year-2	0	0	Spring
3/15/2019	BL	5M	3	2019	Year-2	0	0	Spring
3/15/2019	BL	6M	3	2019	Year-2	0	0	Spring
3/15/2019	BL	7M	3	2019	Year-2	0	0	Spring
3/15/2019	BL	10M	3	2019	Year-2	0	0	Spring
3/15/2019	BL	12M	3	2019	Year-2	0	0	Spring
3/15/2019	BL	14M	3	2019	Year-2	0	0	Spring
3/15/2019	BL	8A	3	2019	Year-2	0	0	Spring
3/15/2019	BL	9A	3	2019	Year-2	0	0	Spring
3/15/2019	BL	11A	3	2019	Year-2	0	0	Spring
3/15/2019	BL	13A	3	2019	Year-2	0	0	Spring
3/16/2019	BL	1M	3	2019	Year-2	0	0	Spring
3/16/2019	BL	2M	3	2019	Year-2	0	0	Spring
3/16/2019	BL	3M	3	2019	Year-2	0	0	Spring
3/16/2019	BL	4M	3	2019	Year-2	0	0	Spring
3/16/2019	BL	5M	3	2019	Year-2	0	0	Spring
3/16/2019	BL	6M	3	2019	Year-2	0	0	Spring
3/16/2019	BL	7M	3	2019	Year-2	0	0	Spring
3/16/2019	BL	10M	3	2019	Year-2	0	0	Spring
3/16/2019	BL	12M	3	2019	Year-2	0	0	Spring
3/16/2019	BL	14M	3	2019	Year-2	0	0	Spring
3/16/2019	BL	8A	3	2019	Year-2	0	0	Spring
3/16/2019	BL	9A	3	2019	Year-2	0	0	Spring
3/16/2019	BL	11A	3	2019	Year-2	0	0	Spring
3/16/2019	BL	13A	3	2019	Year-2	0	0	Spring
3/17/2019	BL	1M	3	2019	Year-2	0	0	Spring
3/17/2019	BL	2M	3	2019	Year-2	0	0	Spring
3/17/2019	BL	3M	3	2019	Year-2	0	0	Spring
3/17/2019	BL	4M	3	2019	Year-2	0	0	Spring
3/17/2019	BL	5M	3	2019	Year-2	0	0	Spring
3/17/2019	BL	6M	3	2019	Year-2	0	0	Spring
3/17/2019	BL	7M	3	2019	Year-2	0	0	Spring
3/17/2019	BL	10M	3	2019	Year-2	0	0	Spring
3/17/2019	BL	12M	3	2019	Year-2	0	0	Spring
3/17/2019	BL	14M	3	2019	Year-2	0	0	Spring
3/17/2019	BL	8A	3	2019	Year-2	0	0	Spring
3/17/2019	BL	9A	3	2019	Year-2	0	0	Spring
3/17/2019	BL	11A	3	2019	Year-2	0	0	Spring
3/17/2019	BL	13A	3	2019	Year-2	0	0	Spring
3/18/2019	BL	1M	3	2019	Year-2	0	0	Spring

Date	Species	Site	Month	Year	Survey Year	Presence	Data Gap	Season
3/18/2019	BL	2M	3	2019	Year-2	0	0	Spring
3/18/2019	BL	3M	3	2019	Year-2	0	0	Spring
3/18/2019	BL	4M	3	2019	Year-2	0	0	Spring
3/18/2019	BL	5M	3	2019	Year-2	0	0	Spring
3/18/2019	BL	6M	3	2019	Year-2	0	0	Spring
3/18/2019	BL	7M	3	2019	Year-2	0	0	Spring
3/18/2019	BL	10M	3	2019	Year-2	0	0	Spring
3/18/2019	BL	12M	3	2019	Year-2	0	0	Spring
3/18/2019	BL	14M	3	2019	Year-2	0	0	Spring
3/18/2019	BL	8A	3	2019	Year-2	0	0	Spring
3/18/2019	BL	9A	3	2019	Year-2	0	0	Spring
3/18/2019	BL	11A	3	2019	Year-2	0	0	Spring
3/18/2019	BL	13A	3	2019	Year-2	0	0	Spring
3/19/2019	BL	1M	3	2019	Year-2	0	0	Spring
3/19/2019	BL	2M	3	2019	Year-2	0	0	Spring
3/19/2019	BL	3M	3	2019	Year-2	0	0	Spring
3/19/2019	BL	4M	3	2019	Year-2	0	0	Spring
3/19/2019	BL	5M	3	2019	Year-2	0	0	Spring
3/19/2019	BL	6M	3	2019	Year-2	0	0	Spring
3/19/2019	BL	7M	3	2019	Year-2	0	0	Spring
3/19/2019	BL	10M	3	2019	Year-2	0	0	Spring
3/19/2019	BL	12M	3	2019	Year-2	0	0	Spring
3/19/2019	BL	14M	3	2019	Year-2	0	0	Spring
3/19/2019	BL	8A	3	2019	Year-2	0	0	Spring
3/19/2019	BL	9A	3	2019	Year-2	0	0	Spring
3/19/2019	BL	11A	3	2019	Year-2	0	0	Spring
3/19/2019	BL	13A	3	2019	Year-2	0	0	Spring
3/20/2019	BL	1M	3	2019	Year-2	0	0	Spring
3/20/2019	BL	2M	3	2019	Year-2	0	0	Spring
3/20/2019	BL	3M	3	2019	Year-2	0	0	Spring
3/20/2019	BL	4M	3	2019	Year-2	0	0	Spring
3/20/2019	BL	5M	3	2019	Year-2	0	0	Spring
3/20/2019	BL	6M	3	2019	Year-2	0	0	Spring
3/20/2019	BL	7M	3	2019	Year-2	0	0	Spring
3/20/2019	BL	10M	3	2019	Year-2	0	0	Spring
3/20/2019	BL	12M	3	2019	Year-2	0	0	Spring
3/20/2019	BL	14M	3	2019	Year-2	0	0	Spring
3/20/2019	BL	8A	3	2019	Year-2	0	0	Spring
3/20/2019	BL	9A	3	2019	Year-2	0	0	Spring
3/20/2019	BL	11A	3	2019	Year-2	0	0	Spring
3/20/2019	BL	13A	3	2019	Year-2	0	0	Spring
3/21/2019	BL	1M	3	2019	Year-2	0	0	Spring
3/21/2019	BL	2M	3	2019	Year-2	0	0	Spring
3/21/2019	BL	3M	3	2019	Year-2	0	0	Spring

Date	Species	Site	Month	Year	Survey Year	Presence	Data Gap	Season
3/21/2019	BL	4M	3	2019	Year-2	0	0	Spring
3/21/2019	BL	5M	3	2019	Year-2	0	0	Spring
3/21/2019	BL	6M	3	2019	Year-2	0	0	Spring
3/21/2019	BL	7M	3	2019	Year-2	0	0	Spring
3/21/2019	BL	10M	3	2019	Year-2	0	0	Spring
3/21/2019	BL	12M	3	2019	Year-2	0	0	Spring
3/21/2019	BL	14M	3	2019	Year-2	0	0	Spring
3/21/2019	BL	8A	3	2019	Year-2	0	0	Spring
3/21/2019	BL	9A	3	2019	Year-2	0	0	Spring
3/21/2019	BL	11A	3	2019	Year-2	0	0	Spring
3/21/2019	BL	13A	3	2019	Year-2	0	0	Spring
3/22/2019	BL	1M	3	2019	Year-2	0	0	Spring
3/22/2019	BL	2M	3	2019	Year-2	0	0	Spring
3/22/2019	BL	3M	3	2019	Year-2	0	0	Spring
3/22/2019	BL	4M	3	2019	Year-2	0	0	Spring
3/22/2019	BL	5M	3	2019	Year-2	0	0	Spring
3/22/2019	BL	6M	3	2019	Year-2	0	0	Spring
3/22/2019	BL	7M	3	2019	Year-2	0	0	Spring
3/22/2019	BL	10M	3	2019	Year-2	0	0	Spring
3/22/2019	BL	12M	3	2019	Year-2	0	0	Spring
3/22/2019	BL	14M	3	2019	Year-2	0	0	Spring
3/22/2019	BL	8A	3	2019	Year-2	0	0	Spring
3/22/2019	BL	9A	3	2019	Year-2	0	0	Spring
3/22/2019	BL	11A	3	2019	Year-2	0	0	Spring
3/22/2019	BL	13A	3	2019	Year-2	0	0	Spring
3/23/2019	BL	1M	3	2019	Year-2	0	0	Spring
3/23/2019	BL	2M	3	2019	Year-2	0	0	Spring
3/23/2019	BL	3M	3	2019	Year-2	0	0	Spring
3/23/2019	BL	4M	3	2019	Year-2	0	0	Spring
3/23/2019	BL	5M	3	2019	Year-2	0	0	Spring
3/23/2019	BL	6M	3	2019	Year-2	0	0	Spring
3/23/2019	BL	7M	3	2019	Year-2	0	0	Spring
3/23/2019	BL	10M	3	2019	Year-2	0	0	Spring
3/23/2019	BL	12M	3	2019	Year-2	0	0	Spring
3/23/2019	BL	14M	3	2019	Year-2	0	0	Spring
3/23/2019	BL	8A	3	2019	Year-2	0	0	Spring
3/23/2019	BL	9A	3	2019	Year-2	0	0	Spring
3/23/2019	BL	11A	3	2019	Year-2	0	0	Spring
3/23/2019	BL	13A	3	2019	Year-2	0	0	Spring
3/24/2019	BL	1M	3	2019	Year-2	0	0	Spring
3/24/2019	BL	2M	3	2019	Year-2	0	0	Spring
3/24/2019	BL	3M	3	2019	Year-2	0	0	Spring
3/24/2019	BL	4M	3	2019	Year-2	0	0	Spring
3/24/2019	BL	5M	3	2019	Year-2	0	0	Spring

Date	Species	Site	Month	Year	Survey Year	Presence	Data Gap	Season
3/24/2019	BL	6M	3	2019	Year-2	0	0	Spring
3/24/2019	BL	7M	3	2019	Year-2	0	0	Spring
3/24/2019	BL	10M	3	2019	Year-2	0	0	Spring
3/24/2019	BL	12M	3	2019	Year-2	0	0	Spring
3/24/2019	BL	14M	3	2019	Year-2	0	0	Spring
3/24/2019	BL	8A	3	2019	Year-2	0	0	Spring
3/24/2019	BL	9A	3	2019	Year-2	0	0	Spring
3/24/2019	BL	11A	3	2019	Year-2	0	0	Spring
3/24/2019	BL	13A	3	2019	Year-2	0	0	Spring
3/25/2019	BL	1M	3	2019	Year-2	0	0	Spring
3/25/2019	BL	2M	3	2019	Year-2	0	0	Spring
3/25/2019	BL	3M	3	2019	Year-2	0	0	Spring
3/25/2019	BL	4M	3	2019	Year-2	0	0	Spring
3/25/2019	BL	5M	3	2019	Year-2	0	0	Spring
3/25/2019	BL	6M	3	2019	Year-2	0	0	Spring
3/25/2019	BL	7M	3	2019	Year-2	0	0	Spring
3/25/2019	BL	10M	3	2019	Year-2	0	0	Spring
3/25/2019	BL	12M	3	2019	Year-2	0	0	Spring
3/25/2019	BL	14M	3	2019	Year-2	0	0	Spring
3/25/2019	BL	8A	3	2019	Year-2	0	0	Spring
3/25/2019	BL	9A	3	2019	Year-2	0	0	Spring
3/25/2019	BL	11A	3	2019	Year-2	0	0	Spring
3/25/2019	BL	13A	3	2019	Year-2	0	0	Spring
3/26/2019	BL	1M	3	2019	Year-2	0	0	Spring
3/26/2019	BL	2M	3	2019	Year-2	0	0	Spring
3/26/2019	BL	3M	3	2019	Year-2	0	0	Spring
3/26/2019	BL	4M	3	2019	Year-2	0	0	Spring
3/26/2019	BL	5M	3	2019	Year-2	0	0	Spring
3/26/2019	BL	6M	3	2019	Year-2	0	0	Spring
3/26/2019	BL	7M	3	2019	Year-2	0	0	Spring
3/26/2019	BL	10M	3	2019	Year-2	0	0	Spring
3/26/2019	BL	12M	3	2019	Year-2	0	0	Spring
3/26/2019	BL	14M	3	2019	Year-2	0	0	Spring
3/26/2019	BL	8A	3	2019	Year-2	0	0	Spring
3/26/2019	BL	9A	3	2019	Year-2	0	0	Spring
3/26/2019	BL	11A	3	2019	Year-2	0	0	Spring
3/26/2019	BL	13A	3	2019	Year-2	0	0	Spring
3/27/2019	BL	1M	3	2019	Year-2	0	0	Spring
3/27/2019	BL	2M	3	2019	Year-2	0	0	Spring
3/27/2019	BL	3M	3	2019	Year-2	0	0	Spring
3/27/2019	BL	4M	3	2019	Year-2	0	0	Spring
3/27/2019	BL	5M	3	2019	Year-2	0	0	Spring
3/27/2019	BL	6M	3	2019	Year-2	0	0	Spring
3/27/2019	BL	7M	3	2019	Year-2	0	0	Spring

Date	Species	Site	Month	Year	Survey Year	Presence	Data Gap	Season
3/27/2019	BL	10M	3	2019	Year-2	0	0	Spring
3/27/2019	BL	12M	3	2019	Year-2	0	0	Spring
3/27/2019	BL	14M	3	2019	Year-2	0	0	Spring
3/27/2019	BL	8A	3	2019	Year-2	0	0	Spring
3/27/2019	BL	9A	3	2019	Year-2	0	0	Spring
3/27/2019	BL	11A	3	2019	Year-2	0	0	Spring
3/27/2019	BL	13A	3	2019	Year-2	0	0	Spring
3/28/2019	BL	1M	3	2019	Year-2	0	0	Spring
3/28/2019	BL	2M	3	2019	Year-2	0	0	Spring
3/28/2019	BL	3M	3	2019	Year-2	0	0	Spring
3/28/2019	BL	4M	3	2019	Year-2	0	0	Spring
3/28/2019	BL	5M	3	2019	Year-2	0	0	Spring
3/28/2019	BL	6M	3	2019	Year-2	0	0	Spring
3/28/2019	BL	7M	3	2019	Year-2	0	0	Spring
3/28/2019	BL	10M	3	2019	Year-2	0	0	Spring
3/28/2019	BL	12M	3	2019	Year-2	0	0	Spring
3/28/2019	BL	14M	3	2019	Year-2	0	0	Spring
3/28/2019	BL	8A	3	2019	Year-2	0	0	Spring
3/28/2019	BL	9A	3	2019	Year-2	0	0	Spring
3/28/2019	BL	11A	3	2019	Year-2	0	0	Spring
3/28/2019	BL	13A	3	2019	Year-2	0	0	Spring
3/29/2019	BL	1M	3	2019	Year-2	0	0	Spring
3/29/2019	BL	2M	3	2019	Year-2	0	0	Spring
3/29/2019	BL	3M	3	2019	Year-2	0	0	Spring
3/29/2019	BL	4M	3	2019	Year-2	0	0	Spring
3/29/2019	BL	5M	3	2019	Year-2	0	0	Spring
3/29/2019	BL	6M	3	2019	Year-2	0	0	Spring
3/29/2019	BL	7M	3	2019	Year-2	0	0	Spring
3/29/2019	BL	10M	3	2019	Year-2	0	0	Spring
3/29/2019	BL	12M	3	2019	Year-2	0	0	Spring
3/29/2019	BL	14M	3	2019	Year-2	0	0	Spring
3/29/2019	BL	8A	3	2019	Year-2	0	0	Spring
3/29/2019	BL	9A	3	2019	Year-2	0	0	Spring
3/29/2019	BL	11A	3	2019	Year-2	0	0	Spring
3/29/2019	BL	13A	3	2019	Year-2	0	0	Spring
3/30/2019	BL	1M	3	2019	Year-2	0	0	Spring
3/30/2019	BL	2M	3	2019	Year-2	0	0	Spring
3/30/2019	BL	3M	3	2019	Year-2	0	0	Spring
3/30/2019	BL	4M	3	2019	Year-2	0	0	Spring
3/30/2019	BL	5M	3	2019	Year-2	0	0	Spring
3/30/2019	BL	6M	3	2019	Year-2	0	0	Spring
3/30/2019	BL	7M	3	2019	Year-2	0	0	Spring
3/30/2019	BL	10M	3	2019	Year-2	0	0	Spring
3/30/2019	BL	12M	3	2019	Year-2	0	0	Spring

Date	Species	Site	Month	Year	Survey Year	Presence	Data Gap	Season
3/30/2019	BL	14M	3	2019	Year-2	0	0	Spring
3/30/2019	BL	8A	3	2019	Year-2	0	0	Spring
3/30/2019	BL	9A	3	2019	Year-2	0	0	Spring
3/30/2019	BL	11A	3	2019	Year-2	0	0	Spring
3/30/2019	BL	13A	3	2019	Year-2	0	0	Spring
3/31/2019	BL	1M	3	2019	Year-2	0	0	Spring
3/31/2019	BL	2M	3	2019	Year-2	0	0	Spring
3/31/2019	BL	3M	3	2019	Year-2	0	0	Spring
3/31/2019	BL	4M	3	2019	Year-2	0	0	Spring
3/31/2019	BL	5M	3	2019	Year-2	0	0	Spring
3/31/2019	BL	6M	3	2019	Year-2	0	0	Spring
3/31/2019	BL	7M	3	2019	Year-2	0	0	Spring
3/31/2019	BL	10M	3	2019	Year-2	0	0	Spring
3/31/2019	BL	12M	3	2019	Year-2	0	0	Spring
3/31/2019	BL	14M	3	2019	Year-2	0	0	Spring
3/31/2019	BL	8A	3	2019	Year-2	0	0	Spring
3/31/2019	BL	9A	3	2019	Year-2	0	0	Spring
3/31/2019	BL	11A	3	2019	Year-2	0	0	Spring
3/31/2019	BL	13A	3	2019	Year-2	0	0	Spring
4/1/2019	BL	1M	4	2019	Year-2	0	0	Spring
4/1/2019	BL	2M	4	2019	Year-2	0	0	Spring
4/1/2019	BL	3M	4	2019	Year-2	0	0	Spring
4/1/2019	BL	4M	4	2019	Year-2	0	0	Spring
4/1/2019	BL	5M	4	2019	Year-2	0	0	Spring
4/1/2019	BL	6M	4	2019	Year-2	0	0	Spring
4/1/2019	BL	7M	4	2019	Year-2	0	0	Spring
4/1/2019	BL	10M	4	2019	Year-2	0	0	Spring
4/1/2019	BL	12M	4	2019	Year-2	0	0	Spring
4/1/2019	BL	14M	4	2019	Year-2	0	0	Spring
4/1/2019	BL	8A	4	2019	Year-2	0	0	Spring
4/1/2019	BL	9A	4	2019	Year-2	0	0	Spring
4/1/2019	BL	11A	4	2019	Year-2	0	0	Spring
4/1/2019	BL	13A	4	2019	Year-2	0	0	Spring
4/2/2019	BL	1M	4	2019	Year-2	0	0	Spring
4/2/2019	BL	2M	4	2019	Year-2	0	0	Spring
4/2/2019	BL	3M	4	2019	Year-2	0	0	Spring
4/2/2019	BL	4M	4	2019	Year-2	0	0	Spring
4/2/2019	BL	5M	4	2019	Year-2	0	0	Spring
4/2/2019	BL	6M	4	2019	Year-2	0	0	Spring
4/2/2019	BL	7M	4	2019	Year-2	0	0	Spring
4/2/2019	BL	10M	4	2019	Year-2	0	0	Spring
4/2/2019	BL	12M	4	2019	Year-2	0	0	Spring
4/2/2019	BL	14M	4	2019	Year-2	0	0	Spring
4/2/2019	BL	8A	4	2019	Year-2	0	0	Spring

Date	Species	Site	Month	Year	Survey Year	Presence	Data Gap	Season
4/2/2019	BL	9A	4	2019	Year-2	0	0	Spring
4/2/2019	BL	11A	4	2019	Year-2	0	0	Spring
4/2/2019	BL	13A	4	2019	Year-2	0	0	Spring
4/3/2019	BL	1M	4	2019	Year-2	0	0	Spring
4/3/2019	BL	2M	4	2019	Year-2	0	0	Spring
4/3/2019	BL	3M	4	2019	Year-2	0	0	Spring
4/3/2019	BL	4M	4	2019	Year-2	0	0	Spring
4/3/2019	BL	5M	4	2019	Year-2	0	0	Spring
4/3/2019	BL	6M	4	2019	Year-2	0	0	Spring
4/3/2019	BL	7M	4	2019	Year-2	0	0	Spring
4/3/2019	BL	10M	4	2019	Year-2	0	0	Spring
4/3/2019	BL	12M	4	2019	Year-2	0	0	Spring
4/3/2019	BL	14M	4	2019	Year-2	0	0	Spring
4/3/2019	BL	8A	4	2019	Year-2	0	0	Spring
4/3/2019	BL	9A	4	2019	Year-2	0	0	Spring
4/3/2019	BL	11A	4	2019	Year-2	0	0	Spring
4/3/2019	BL	13A	4	2019	Year-2	0	0	Spring
4/4/2019	BL	1M	4	2019	Year-2	0	0	Spring
4/4/2019	BL	2M	4	2019	Year-2	0	0	Spring
4/4/2019	BL	3M	4	2019	Year-2	0	0	Spring
4/4/2019	BL	4M	4	2019	Year-2	0	0	Spring
4/4/2019	BL	5M	4	2019	Year-2	0	0	Spring
4/4/2019	BL	6M	4	2019	Year-2	0	0	Spring
4/4/2019	BL	7M	4	2019	Year-2	0	0	Spring
4/4/2019	BL	10M	4	2019	Year-2	0	0	Spring
4/4/2019	BL	12M	4	2019	Year-2	0	0	Spring
4/4/2019	BL	14M	4	2019	Year-2	0	0	Spring
4/4/2019	BL	8A	4	2019	Year-2	0	0	Spring
4/4/2019	BL	9A	4	2019	Year-2	0	0	Spring
4/4/2019	BL	11A	4	2019	Year-2	0	0	Spring
4/4/2019	BL	13A	4	2019	Year-2	0	0	Spring
4/5/2019	BL	1M	4	2019	Year-2	0	0	Spring
4/5/2019	BL	2M	4	2019	Year-2	0	0	Spring
4/5/2019	BL	3M	4	2019	Year-2	0	0	Spring
4/5/2019	BL	4M	4	2019	Year-2	0	0	Spring
4/5/2019	BL	5M	4	2019	Year-2	0	0	Spring
4/5/2019	BL	6M	4	2019	Year-2	0	0	Spring
4/5/2019	BL	7M	4	2019	Year-2	0	0	Spring
4/5/2019	BL	10M	4	2019	Year-2	0	0	Spring
4/5/2019	BL	12M	4	2019	Year-2	0	0	Spring
4/5/2019	BL	14M	4	2019	Year-2	0	0	Spring
4/5/2019	BL	8A	4	2019	Year-2	0	0	Spring
4/5/2019	BL	9A	4	2019	Year-2	0	0	Spring
4/5/2019	BL	11A	4	2019	Year-2	0	0	Spring

Date	Species	Site	Month	Year	Survey Year	Presence	Data Gap	Season
4/5/2019	BL	13A	4	2019	Year-2	0	0	Spring
4/6/2019	BL	1M	4	2019	Year-2	0	0	Spring
4/6/2019	BL	2M	4	2019	Year-2	0	0	Spring
4/6/2019	BL	3M	4	2019	Year-2	0	0	Spring
4/6/2019	BL	4M	4	2019	Year-2	0	0	Spring
4/6/2019	BL	5M	4	2019	Year-2	0	0	Spring
4/6/2019	BL	6M	4	2019	Year-2	0	0	Spring
4/6/2019	BL	7M	4	2019	Year-2	0	0	Spring
4/6/2019	BL	10M	4	2019	Year-2	0	0	Spring
4/6/2019	BL	12M	4	2019	Year-2	0	0	Spring
4/6/2019	BL	14M	4	2019	Year-2	0	0	Spring
4/6/2019	BL	8A	4	2019	Year-2	0	0	Spring
4/6/2019	BL	9A	4	2019	Year-2	0	0	Spring
4/6/2019	BL	11A	4	2019	Year-2	0	0	Spring
4/6/2019	BL	13A	4	2019	Year-2	0	0	Spring
4/7/2019	BL	1M	4	2019	Year-2	0	0	Spring
4/7/2019	BL	2M	4	2019	Year-2	0	0	Spring
4/7/2019	BL	3M	4	2019	Year-2	0	0	Spring
4/7/2019	BL	4M	4	2019	Year-2	0	0	Spring
4/7/2019	BL	5M	4	2019	Year-2	0	0	Spring
4/7/2019	BL	6M	4	2019	Year-2	0	0	Spring
4/7/2019	BL	7M	4	2019	Year-2	0	0	Spring
4/7/2019	BL	10M	4	2019	Year-2	0	0	Spring
4/7/2019	BL	12M	4	2019	Year-2	0	0	Spring
4/7/2019	BL	14M	4	2019	Year-2	0	0	Spring
4/7/2019	BL	8A	4	2019	Year-2	0	0	Spring
4/7/2019	BL	9A	4	2019	Year-2	0	0	Spring
4/7/2019	BL	11A	4	2019	Year-2	0	0	Spring
4/7/2019	BL	13A	4	2019	Year-2	0	0	Spring
4/8/2019	BL	1M	4	2019	Year-2	0	0	Spring
4/8/2019	BL	2M	4	2019	Year-2	0	0	Spring
4/8/2019	BL	3M	4	2019	Year-2	0	0	Spring
4/8/2019	BL	4M	4	2019	Year-2	0	0	Spring
4/8/2019	BL	5M	4	2019	Year-2	0	0	Spring
4/8/2019	BL	6M	4	2019	Year-2	0	0	Spring
4/8/2019	BL	7M	4	2019	Year-2	0	0	Spring
4/8/2019	BL	10M	4	2019	Year-2	0	0	Spring
4/8/2019	BL	12M	4	2019	Year-2	0	0	Spring
4/8/2019	BL	14M	4	2019	Year-2	0	0	Spring
4/8/2019	BL	8A	4	2019	Year-2	0	0	Spring
4/8/2019	BL	9A	4	2019	Year-2	0	0	Spring
4/8/2019	BL	11A	4	2019	Year-2	0	0	Spring
4/8/2019	BL	13A	4	2019	Year-2	0	0	Spring
4/9/2019	BL	1M	4	2019	Year-2	0	0	Spring

Date	Species	Site	Month	Year	Survey Year	Presence	Data Gap	Season
4/9/2019	BL	2M	4	2019	Year-2	0	0	Spring
4/9/2019	BL	3M	4	2019	Year-2	0	0	Spring
4/9/2019	BL	4M	4	2019	Year-2	0	0	Spring
4/9/2019	BL	5M	4	2019	Year-2	0	0	Spring
4/9/2019	BL	6M	4	2019	Year-2	0	0	Spring
4/9/2019	BL	7M	4	2019	Year-2	0	0	Spring
4/9/2019	BL	10M	4	2019	Year-2	0	0	Spring
4/9/2019	BL	12M	4	2019	Year-2	0	0	Spring
4/9/2019	BL	14M	4	2019	Year-2	0	0	Spring
4/9/2019	BL	8A	4	2019	Year-2	0	0	Spring
4/9/2019	BL	9A	4	2019	Year-2	0	0	Spring
4/9/2019	BL	11A	4	2019	Year-2	0	0	Spring
4/9/2019	BL	13A	4	2019	Year-2	0	0	Spring
4/10/2019	BL	1M	4	2019	Year-2	0	0	Spring
4/10/2019	BL	2M	4	2019	Year-2	0	0	Spring
4/10/2019	BL	3M	4	2019	Year-2	0	0	Spring
4/10/2019	BL	4M	4	2019	Year-2	0	0	Spring
4/10/2019	BL	5M	4	2019	Year-2	0	0	Spring
4/10/2019	BL	6M	4	2019	Year-2		1	Spring
4/10/2019	BL	7M	4	2019	Year-2		1	Spring
4/10/2019	BL	10M	4	2019	Year-2	0	0	Spring
4/10/2019	BL	12M	4	2019	Year-2	0	0	Spring
4/10/2019	BL	14M	4	2019	Year-2	0	0	Spring
4/10/2019	BL	8A	4	2019	Year-2	0	0	Spring
4/10/2019	BL	9A	4	2019	Year-2	0	0	Spring
4/10/2019	BL	11A	4	2019	Year-2	0	0	Spring
4/10/2019	BL	13A	4	2019	Year-2	0	0	Spring
4/11/2019	BL	1M	4	2019	Year-2		1	Spring
4/11/2019	BL	2M	4	2019	Year-2		1	Spring
4/11/2019	BL	3M	4	2019	Year-2		1	Spring
4/11/2019	BL	4M	4	2019	Year-2	0	0	Spring
4/11/2019	BL	5M	4	2019	Year-2	0	0	Spring
4/11/2019	BL	6M	4	2019	Year-2		1	Spring
4/11/2019	BL	7M	4	2019	Year-2	0	0	Spring
4/11/2019	BL	10M	4	2019	Year-2	0	0	Spring
4/11/2019	BL	12M	4	2019	Year-2	0	0	Spring
4/11/2019	BL	14M	4	2019	Year-2	0	0	Spring
4/11/2019	BL	8A	4	2019	Year-2	0	0	Spring
4/11/2019	BL	9A	4	2019	Year-2	0	0	Spring
4/11/2019	BL	11A	4	2019	Year-2	0	0	Spring
4/11/2019	BL	13A	4	2019	Year-2	0	0	Spring
4/12/2019	BL	1M	4	2019	Year-2	0	0	Spring
4/12/2019	BL	2M	4	2019	Year-2	0	0	Spring
4/12/2019	BL	3M	4	2019	Year-2	0	0	Spring

Date	Species	Site	Month	Year	Survey Year	Presence	Data Gap	Season
4/12/2019	BL	4M	4	2019	Year-2	0	0	Spring
4/12/2019	BL	5M	4	2019	Year-2	0	0	Spring
4/12/2019	BL	6M	4	2019	Year-2		1	Spring
4/12/2019	BL	7M	4	2019	Year-2	0	0	Spring
4/12/2019	BL	10M	4	2019	Year-2	0	0	Spring
4/12/2019	BL	12M	4	2019	Year-2	0	0	Spring
4/12/2019	BL	14M	4	2019	Year-2	0	0	Spring
4/12/2019	BL	8A	4	2019	Year-2	0	0	Spring
4/12/2019	BL	9A	4	2019	Year-2	0	0	Spring
4/12/2019	BL	11A	4	2019	Year-2	0	0	Spring
4/12/2019	BL	13A	4	2019	Year-2	0	0	Spring
4/13/2019	BL	1M	4	2019	Year-2	0	0	Spring
4/13/2019	BL	2M	4	2019	Year-2	0	0	Spring
4/13/2019	BL	3M	4	2019	Year-2	0	0	Spring
4/13/2019	BL	4M	4	2019	Year-2	0	0	Spring
4/13/2019	BL	5M	4	2019	Year-2	0	0	Spring
4/13/2019	BL	6M	4	2019	Year-2		1	Spring
4/13/2019	BL	7M	4	2019	Year-2	0	0	Spring
4/13/2019	BL	10M	4	2019	Year-2	0	0	Spring
4/13/2019	BL	12M	4	2019	Year-2	0	0	Spring
4/13/2019	BL	14M	4	2019	Year-2	0	0	Spring
4/13/2019	BL	8A	4	2019	Year-2	0	0	Spring
4/13/2019	BL	9A	4	2019	Year-2	0	0	Spring
4/13/2019	BL	11A	4	2019	Year-2	0	0	Spring
4/13/2019	BL	13A	4	2019	Year-2	0	0	Spring
4/14/2019	BL	1M	4	2019	Year-2	0	0	Spring
4/14/2019	BL	2M	4	2019	Year-2	0	0	Spring
4/14/2019	BL	3M	4	2019	Year-2	0	0	Spring
4/14/2019	BL	4M	4	2019	Year-2	0	0	Spring
4/14/2019	BL	5M	4	2019	Year-2	0	0	Spring
4/14/2019	BL	6M	4	2019	Year-2		1	Spring
4/14/2019	BL	7M	4	2019	Year-2	0	0	Spring
4/14/2019	BL	10M	4	2019	Year-2	0	0	Spring
4/14/2019	BL	12M	4	2019	Year-2	0	0	Spring
4/14/2019	BL	14M	4	2019	Year-2	0	0	Spring
4/14/2019	BL	8A	4	2019	Year-2	0	0	Spring
4/14/2019	BL	9A	4	2019	Year-2	0	0	Spring
4/14/2019	BL	11A	4	2019	Year-2	0	0	Spring
4/14/2019	BL	13A	4	2019	Year-2	0	0	Spring
4/15/2019	BL	1M	4	2019	Year-2	0	0	Spring
4/15/2019	BL	2M	4	2019	Year-2	0	0	Spring
4/15/2019	BL	3M	4	2019	Year-2	0	0	Spring
4/15/2019	BL	4M	4	2019	Year-2	0	0	Spring
4/15/2019	BL	5M	4	2019	Year-2	0	0	Spring

Date	Species	Site	Month	Year	Survey Year	Presence	Data Gap	Season
4/15/2019	BL	6M	4	2019	Year-2		1	Spring
4/15/2019	BL	7M	4	2019	Year-2	0	0	Spring
4/15/2019	BL	10M	4	2019	Year-2	0	0	Spring
4/15/2019	BL	12M	4	2019	Year-2	0	0	Spring
4/15/2019	BL	14M	4	2019	Year-2	0	0	Spring
4/15/2019	BL	8A	4	2019	Year-2	0	0	Spring
4/15/2019	BL	9A	4	2019	Year-2	0	0	Spring
4/15/2019	BL	11A	4	2019	Year-2	0	0	Spring
4/15/2019	BL	13A	4	2019	Year-2	0	0	Spring
4/16/2019	BL	1M	4	2019	Year-2	0	0	Spring
4/16/2019	BL	2M	4	2019	Year-2	0	0	Spring
4/16/2019	BL	3M	4	2019	Year-2	0	0	Spring
4/16/2019	BL	4M	4	2019	Year-2	0	0	Spring
4/16/2019	BL	5M	4	2019	Year-2	0	0	Spring
4/16/2019	BL	6M	4	2019	Year-2		1	Spring
4/16/2019	BL	7M	4	2019	Year-2	0	0	Spring
4/16/2019	BL	10M	4	2019	Year-2	0	0	Spring
4/16/2019	BL	12M	4	2019	Year-2	0	0	Spring
4/16/2019	BL	14M	4	2019	Year-2	0	0	Spring
4/16/2019	BL	8A	4	2019	Year-2	0	0	Spring
4/16/2019	BL	9A	4	2019	Year-2	0	0	Spring
4/16/2019	BL	11A	4	2019	Year-2	0	0	Spring
4/16/2019	BL	13A	4	2019	Year-2	0	0	Spring
4/17/2019	BL	1M	4	2019	Year-2	0	0	Spring
4/17/2019	BL	2M	4	2019	Year-2	0	0	Spring
4/17/2019	BL	3M	4	2019	Year-2	0	0	Spring
4/17/2019	BL	4M	4	2019	Year-2	0	0	Spring
4/17/2019	BL	5M	4	2019	Year-2	0	0	Spring
4/17/2019	BL	6M	4	2019	Year-2		1	Spring
4/17/2019	BL	7M	4	2019	Year-2	0	0	Spring
4/17/2019	BL	10M	4	2019	Year-2	0	0	Spring
4/17/2019	BL	12M	4	2019	Year-2	0	0	Spring
4/17/2019	BL	14M	4	2019	Year-2	0	0	Spring
4/17/2019	BL	8A	4	2019	Year-2	0	0	Spring
4/17/2019	BL	9A	4	2019	Year-2	0	0	Spring
4/17/2019	BL	11A	4	2019	Year-2	0	0	Spring
4/17/2019	BL	13A	4	2019	Year-2	0	0	Spring
4/18/2019	BL	1M	4	2019	Year-2	0	0	Spring
4/18/2019	BL	2M	4	2019	Year-2	0	0	Spring
4/18/2019	BL	3M	4	2019	Year-2	0	0	Spring
4/18/2019	BL	4M	4	2019	Year-2	0	0	Spring
4/18/2019	BL	5M	4	2019	Year-2	0	0	Spring
4/18/2019	BL	6M	4	2019	Year-2		1	Spring
4/18/2019	BL	7M	4	2019	Year-2	0	0	Spring

Date	Species	Site	Month	Year	Survey Year	Presence	Data Gap	Season
4/18/2019	BL	10M	4	2019	Year-2	0	0	Spring
4/18/2019	BL	12M	4	2019	Year-2	0	0	Spring
4/18/2019	BL	14M	4	2019	Year-2	0	0	Spring
4/18/2019	BL	8A	4	2019	Year-2	0	0	Spring
4/18/2019	BL	9A	4	2019	Year-2	0	0	Spring
4/18/2019	BL	11A	4	2019	Year-2	0	0	Spring
4/18/2019	BL	13A	4	2019	Year-2	0	0	Spring
4/19/2019	BL	1M	4	2019	Year-2	0	0	Spring
4/19/2019	BL	2M	4	2019	Year-2	0	0	Spring
4/19/2019	BL	3M	4	2019	Year-2	0	0	Spring
4/19/2019	BL	4M	4	2019	Year-2	0	0	Spring
4/19/2019	BL	5M	4	2019	Year-2	0	0	Spring
4/19/2019	BL	6M	4	2019	Year-2		1	Spring
4/19/2019	BL	7M	4	2019	Year-2	0	0	Spring
4/19/2019	BL	10M	4	2019	Year-2	0	0	Spring
4/19/2019	BL	12M	4	2019	Year-2	0	0	Spring
4/19/2019	BL	14M	4	2019	Year-2	0	0	Spring
4/19/2019	BL	8A	4	2019	Year-2	0	0	Spring
4/19/2019	BL	9A	4	2019	Year-2	0	0	Spring
4/19/2019	BL	11A	4	2019	Year-2	0	0	Spring
4/19/2019	BL	13A	4	2019	Year-2	0	0	Spring
4/20/2019	BL	1M	4	2019	Year-2	0	0	Spring
4/20/2019	BL	2M	4	2019	Year-2	0	0	Spring
4/20/2019	BL	3M	4	2019	Year-2	0	0	Spring
4/20/2019	BL	4M	4	2019	Year-2	0	0	Spring
4/20/2019	BL	5M	4	2019	Year-2	0	0	Spring
4/20/2019	BL	6M	4	2019	Year-2		1	Spring
4/20/2019	BL	7M	4	2019	Year-2	0	0	Spring
4/20/2019	BL	10M	4	2019	Year-2	0	0	Spring
4/20/2019	BL	12M	4	2019	Year-2	0	0	Spring
4/20/2019	BL	14M	4	2019	Year-2	0	0	Spring
4/20/2019	BL	8A	4	2019	Year-2	0	0	Spring
4/20/2019	BL	9A	4	2019	Year-2	0	0	Spring
4/20/2019	BL	11A	4	2019	Year-2	0	0	Spring
4/20/2019	BL	13A	4	2019	Year-2	0	0	Spring
4/21/2019	BL	1M	4	2019	Year-2	0	0	Spring
4/21/2019	BL	2M	4	2019	Year-2	0	0	Spring
4/21/2019	BL	3M	4	2019	Year-2	0	0	Spring
4/21/2019	BL	4M	4	2019	Year-2	0	0	Spring
4/21/2019	BL	5M	4	2019	Year-2	0	0	Spring
4/21/2019	BL	6M	4	2019	Year-2		1	Spring
4/21/2019	BL	7M	4	2019	Year-2	0	0	Spring
4/21/2019	BL	10M	4	2019	Year-2	0	0	Spring
4/21/2019	BL	12M	4	2019	Year-2	0	0	Spring

Date	Species	Site	Month	Year	Survey Year	Presence	Data Gap	Season
4/21/2019	BL	14M	4	2019	Year-2	0	0	Spring
4/21/2019	BL	8A	4	2019	Year-2	0	0	Spring
4/21/2019	BL	9A	4	2019	Year-2	0	0	Spring
4/21/2019	BL	11A	4	2019	Year-2	0	0	Spring
4/21/2019	BL	13A	4	2019	Year-2	0	0	Spring
4/22/2019	BL	1M	4	2019	Year-2	0	0	Spring
4/22/2019	BL	2M	4	2019	Year-2	0	0	Spring
4/22/2019	BL	3M	4	2019	Year-2	0	0	Spring
4/22/2019	BL	4M	4	2019	Year-2	0	0	Spring
4/22/2019	BL	5M	4	2019	Year-2	0	0	Spring
4/22/2019	BL	6M	4	2019	Year-2		1	Spring
4/22/2019	BL	7M	4	2019	Year-2	0	0	Spring
4/22/2019	BL	10M	4	2019	Year-2	0	0	Spring
4/22/2019	BL	12M	4	2019	Year-2	0	0	Spring
4/22/2019	BL	14M	4	2019	Year-2	0	0	Spring
4/22/2019	BL	8A	4	2019	Year-2	0	0	Spring
4/22/2019	BL	9A	4	2019	Year-2	0	0	Spring
4/22/2019	BL	11A	4	2019	Year-2	0	0	Spring
4/22/2019	BL	13A	4	2019	Year-2	0	0	Spring
4/23/2019	BL	1M	4	2019	Year-2	0	0	Spring
4/23/2019	BL	2M	4	2019	Year-2	0	0	Spring
4/23/2019	BL	3M	4	2019	Year-2	0	0	Spring
4/23/2019	BL	4M	4	2019	Year-2	0	0	Spring
4/23/2019	BL	5M	4	2019	Year-2	0	0	Spring
4/23/2019	BL	6M	4	2019	Year-2		1	Spring
4/23/2019	BL	7M	4	2019	Year-2	0	0	Spring
4/23/2019	BL	10M	4	2019	Year-2	0	0	Spring
4/23/2019	BL	12M	4	2019	Year-2	0	0	Spring
4/23/2019	BL	14M	4	2019	Year-2	0	0	Spring
4/23/2019	BL	8A	4	2019	Year-2	0	0	Spring
4/23/2019	BL	9A	4	2019	Year-2	0	0	Spring
4/23/2019	BL	11A	4	2019	Year-2	0	0	Spring
4/23/2019	BL	13A	4	2019	Year-2	0	0	Spring
4/24/2019	BL	1M	4	2019	Year-2	0	0	Spring
4/24/2019	BL	2M	4	2019	Year-2	0	0	Spring
4/24/2019	BL	3M	4	2019	Year-2	0	0	Spring
4/24/2019	BL	4M	4	2019	Year-2	0	0	Spring
4/24/2019	BL	5M	4	2019	Year-2	0	0	Spring
4/24/2019	BL	6M	4	2019	Year-2		1	Spring
4/24/2019	BL	7M	4	2019	Year-2	0	0	Spring
4/24/2019	BL	10M	4	2019	Year-2	0	0	Spring
4/24/2019	BL	12M	4	2019	Year-2	0	0	Spring
4/24/2019	BL	14M	4	2019	Year-2	0	0	Spring
4/24/2019	BL	8A	4	2019	Year-2	0	0	Spring

Date	Species	Site	Month	Year	Survey Year	Presence	Data Gap	Season
4/24/2019	BL	9A	4	2019	Year-2	0	0	Spring
4/24/2019	BL	11A	4	2019	Year-2	0	0	Spring
4/24/2019	BL	13A	4	2019	Year-2	0	0	Spring
4/25/2019	BL	1M	4	2019	Year-2	0	0	Spring
4/25/2019	BL	2M	4	2019	Year-2	0	0	Spring
4/25/2019	BL	3M	4	2019	Year-2	0	0	Spring
4/25/2019	BL	4M	4	2019	Year-2	0	0	Spring
4/25/2019	BL	5M	4	2019	Year-2	0	0	Spring
4/25/2019	BL	6M	4	2019	Year-2		1	Spring
4/25/2019	BL	7M	4	2019	Year-2	0	0	Spring
4/25/2019	BL	10M	4	2019	Year-2		1	Spring
4/25/2019	BL	12M	4	2019	Year-2		1	Spring
4/25/2019	BL	14M	4	2019	Year-2		1	Spring
4/25/2019	BL	8A	4	2019	Year-2	0	0	Spring
4/25/2019	BL	9A	4	2019	Year-2	0	0	Spring
4/25/2019	BL	11A	4	2019	Year-2	0	0	Spring
4/25/2019	BL	13A	4	2019	Year-2	0	0	Spring
4/26/2019	BL	1M	4	2019	Year-2	0	0	Spring
4/26/2019	BL	2M	4	2019	Year-2	0	0	Spring
4/26/2019	BL	3M	4	2019	Year-2	0	0	Spring
4/26/2019	BL	4M	4	2019	Year-2	0	0	Spring
4/26/2019	BL	5M	4	2019	Year-2	0	0	Spring
4/26/2019	BL	6M	4	2019	Year-2		1	Spring
4/26/2019	BL	7M	4	2019	Year-2	0	0	Spring
4/26/2019	BL	10M	4	2019	Year-2	0	0	Spring
4/26/2019	BL	12M	4	2019	Year-2	0	0	Spring
4/26/2019	BL	14M	4	2019	Year-2	0	0	Spring
4/26/2019	BL	8A	4	2019	Year-2	0	0	Spring
4/26/2019	BL	9A	4	2019	Year-2	0	0	Spring
4/26/2019	BL	11A	4	2019	Year-2	0	0	Spring
4/26/2019	BL	13A	4	2019	Year-2	0	0	Spring
4/27/2019	BL	1M	4	2019	Year-2	0	0	Spring
4/27/2019	BL	2M	4	2019	Year-2	0	0	Spring
4/27/2019	BL	3M	4	2019	Year-2	0	0	Spring
4/27/2019	BL	4M	4	2019	Year-2	0	0	Spring
4/27/2019	BL	5M	4	2019	Year-2	0	0	Spring
4/27/2019	BL	6M	4	2019	Year-2		1	Spring
4/27/2019	BL	7M	4	2019	Year-2	0	0	Spring
4/27/2019	BL	10M	4	2019	Year-2	0	0	Spring
4/27/2019	BL	12M	4	2019	Year-2	0	0	Spring
4/27/2019	BL	14M	4	2019	Year-2	0	0	Spring
4/27/2019	BL	8A	4	2019	Year-2	0	0	Spring
4/27/2019	BL	9A	4	2019	Year-2	0	0	Spring
4/27/2019	BL	11A	4	2019	Year-2	0	0	Spring

Date	Species	Site	Month	Year	Survey Year	Presence	Data Gap	Season
4/27/2019	BL	13A	4	2019	Year-2	0	0	Spring
4/28/2019	BL	1M	4	2019	Year-2	0	0	Spring
4/28/2019	BL	2M	4	2019	Year-2	0	0	Spring
4/28/2019	BL	3M	4	2019	Year-2	0	0	Spring
4/28/2019	BL	4M	4	2019	Year-2	0	0	Spring
4/28/2019	BL	5M	4	2019	Year-2	0	0	Spring
4/28/2019	BL	6M	4	2019	Year-2		1	Spring
4/28/2019	BL	7M	4	2019	Year-2	0	0	Spring
4/28/2019	BL	10M	4	2019	Year-2	0	0	Spring
4/28/2019	BL	12M	4	2019	Year-2	0	0	Spring
4/28/2019	BL	14M	4	2019	Year-2	0	0	Spring
4/28/2019	BL	8A	4	2019	Year-2	0	0	Spring
4/28/2019	BL	9A	4	2019	Year-2	0	0	Spring
4/28/2019	BL	11A	4	2019	Year-2	0	0	Spring
4/28/2019	BL	13A	4	2019	Year-2	0	0	Spring
4/29/2019	BL	1M	4	2019	Year-2	0	0	Spring
4/29/2019	BL	2M	4	2019	Year-2	0	0	Spring
4/29/2019	BL	3M	4	2019	Year-2	0	0	Spring
4/29/2019	BL	4M	4	2019	Year-2	0	0	Spring
4/29/2019	BL	5M	4	2019	Year-2	0	0	Spring
4/29/2019	BL	6M	4	2019	Year-2		1	Spring
4/29/2019	BL	7M	4	2019	Year-2	0	0	Spring
4/29/2019	BL	10M	4	2019	Year-2	0	0	Spring
4/29/2019	BL	12M	4	2019	Year-2	0	0	Spring
4/29/2019	BL	14M	4	2019	Year-2	0	0	Spring
4/29/2019	BL	8A	4	2019	Year-2	0	0	Spring
4/29/2019	BL	9A	4	2019	Year-2	0	0	Spring
4/29/2019	BL	11A	4	2019	Year-2	0	0	Spring
4/29/2019	BL	13A	4	2019	Year-2	0	0	Spring
4/30/2019	BL	1M	4	2019	Year-2	0	0	Spring
4/30/2019	BL	2M	4	2019	Year-2	0	0	Spring
4/30/2019	BL	3M	4	2019	Year-2	0	0	Spring
4/30/2019	BL	4M	4	2019	Year-2	0	0	Spring
4/30/2019	BL	5M	4	2019	Year-2	0	0	Spring
4/30/2019	BL	6M	4	2019	Year-2		1	Spring
4/30/2019	BL	7M	4	2019	Year-2	0	0	Spring
4/30/2019	BL	10M	4	2019	Year-2	0	0	Spring
4/30/2019	BL	12M	4	2019	Year-2	0	0	Spring
4/30/2019	BL	14M	4	2019	Year-2	0	0	Spring
4/30/2019	BL	8A	4	2019	Year-2	0	0	Spring
4/30/2019	BL	9A	4	2019	Year-2	0	0	Spring
4/30/2019	BL	11A	4	2019	Year-2	0	0	Spring
4/30/2019	BL	13A	4	2019	Year-2	0	0	Spring
5/1/2019	BL	1M	5	2019	Year-2	0	0	Spring

Date	Species	Site	Month	Year	Survey Year	Presence	Data Gap	Season
5/1/2019	BL	2M	5	2019	Year-2	0	0	Spring
5/1/2019	BL	3M	5	2019	Year-2	0	0	Spring
5/1/2019	BL	4M	5	2019	Year-2	0	0	Spring
5/1/2019	BL	5M	5	2019	Year-2	0	0	Spring
5/1/2019	BL	6M	5	2019	Year-2		1	Spring
5/1/2019	BL	7M	5	2019	Year-2	0	0	Spring
5/1/2019	BL	10M	5	2019	Year-2	0	0	Spring
5/1/2019	BL	12M	5	2019	Year-2	0	0	Spring
5/1/2019	BL	14M	5	2019	Year-2	0	0	Spring
5/1/2019	BL	8A	5	2019	Year-2	0	0	Spring
5/1/2019	BL	9A	5	2019	Year-2	0	0	Spring
5/1/2019	BL	11A	5	2019	Year-2	0	0	Spring
5/1/2019	BL	13A	5	2019	Year-2	0	0	Spring
5/2/2019	BL	1M	5	2019	Year-2	0	0	Spring
5/2/2019	BL	2M	5	2019	Year-2	0	0	Spring
5/2/2019	BL	3M	5	2019	Year-2	0	0	Spring
5/2/2019	BL	4M	5	2019	Year-2	0	0	Spring
5/2/2019	BL	5M	5	2019	Year-2	0	0	Spring
5/2/2019	BL	6M	5	2019	Year-2		1	Spring
5/2/2019	BL	7M	5	2019	Year-2	0	0	Spring
5/2/2019	BL	10M	5	2019	Year-2	0	0	Spring
5/2/2019	BL	12M	5	2019	Year-2	0	0	Spring
5/2/2019	BL	14M	5	2019	Year-2	0	0	Spring
5/2/2019	BL	8A	5	2019	Year-2	0	0	Spring
5/2/2019	BL	9A	5	2019	Year-2	0	0	Spring
5/2/2019	BL	11A	5	2019	Year-2	0	0	Spring
5/2/2019	BL	13A	5	2019	Year-2	0	0	Spring
5/3/2019	BL	1M	5	2019	Year-2	0	0	Spring
5/3/2019	BL	2M	5	2019	Year-2	0	0	Spring
5/3/2019	BL	3M	5	2019	Year-2	0	0	Spring
5/3/2019	BL	4M	5	2019	Year-2	0	0	Spring
5/3/2019	BL	5M	5	2019	Year-2	0	0	Spring
5/3/2019	BL	6M	5	2019	Year-2		1	Spring
5/3/2019	BL	7M	5	2019	Year-2	0	0	Spring
5/3/2019	BL	10M	5	2019	Year-2	0	0	Spring
5/3/2019	BL	12M	5	2019	Year-2	0	0	Spring
5/3/2019	BL	14M	5	2019	Year-2	0	0	Spring
5/3/2019	BL	8A	5	2019	Year-2	0	0	Spring
5/3/2019	BL	9A	5	2019	Year-2	0	0	Spring
5/3/2019	BL	11A	5	2019	Year-2	0	0	Spring
5/3/2019	BL	13A	5	2019	Year-2	0	0	Spring
5/4/2019	BL	1M	5	2019	Year-2	0	0	Spring
5/4/2019	BL	2M	5	2019	Year-2	0	0	Spring
5/4/2019	BL	3M	5	2019	Year-2	0	0	Spring

Date	Species	Site	Month	Year	Survey Year	Presence	Data Gap	Season
5/4/2019	BL	4M	5	2019	Year-2	0	0	Spring
5/4/2019	BL	5M	5	2019	Year-2	0	0	Spring
5/4/2019	BL	6M	5	2019	Year-2		1	Spring
5/4/2019	BL	7M	5	2019	Year-2	0	0	Spring
5/4/2019	BL	10M	5	2019	Year-2	0	0	Spring
5/4/2019	BL	12M	5	2019	Year-2	0	0	Spring
5/4/2019	BL	14M	5	2019	Year-2	0	0	Spring
5/4/2019	BL	8A	5	2019	Year-2	0	0	Spring
5/4/2019	BL	9A	5	2019	Year-2	0	0	Spring
5/4/2019	BL	11A	5	2019	Year-2	0	0	Spring
5/4/2019	BL	13A	5	2019	Year-2	0	0	Spring
5/5/2019	BL	1M	5	2019	Year-2	0	0	Spring
5/5/2019	BL	2M	5	2019	Year-2	0	0	Spring
5/5/2019	BL	3M	5	2019	Year-2	0	0	Spring
5/5/2019	BL	4M	5	2019	Year-2	0	0	Spring
5/5/2019	BL	5M	5	2019	Year-2	0	0	Spring
5/5/2019	BL	6M	5	2019	Year-2		1	Spring
5/5/2019	BL	7M	5	2019	Year-2	0	0	Spring
5/5/2019	BL	10M	5	2019	Year-2	0	0	Spring
5/5/2019	BL	12M	5	2019	Year-2	0	0	Spring
5/5/2019	BL	14M	5	2019	Year-2	0	0	Spring
5/5/2019	BL	8A	5	2019	Year-2	0	0	Spring
5/5/2019	BL	9A	5	2019	Year-2	0	0	Spring
5/5/2019	BL	11A	5	2019	Year-2	0	0	Spring
5/5/2019	BL	13A	5	2019	Year-2	0	0	Spring
5/6/2019	BL	1M	5	2019	Year-2	0	0	Spring
5/6/2019	BL	2M	5	2019	Year-2	0	0	Spring
5/6/2019	BL	3M	5	2019	Year-2	0	0	Spring
5/6/2019	BL	4M	5	2019	Year-2	0	0	Spring
5/6/2019	BL	5M	5	2019	Year-2	0	0	Spring
5/6/2019	BL	6M	5	2019	Year-2		1	Spring
5/6/2019	BL	7M	5	2019	Year-2	0	0	Spring
5/6/2019	BL	10M	5	2019	Year-2	0	0	Spring
5/6/2019	BL	12M	5	2019	Year-2	0	0	Spring
5/6/2019	BL	14M	5	2019	Year-2	0	0	Spring
5/6/2019	BL	8A	5	2019	Year-2	0	0	Spring
5/6/2019	BL	9A	5	2019	Year-2	0	0	Spring
5/6/2019	BL	11A	5	2019	Year-2	0	0	Spring
5/6/2019	BL	13A	5	2019	Year-2	0	0	Spring
5/7/2019	BL	1M	5	2019	Year-2	0	0	Spring
5/7/2019	BL	2M	5	2019	Year-2	0	0	Spring
5/7/2019	BL	3M	5	2019	Year-2	0	0	Spring
5/7/2019	BL	4M	5	2019	Year-2	0	0	Spring
5/7/2019	BL	5M	5	2019	Year-2	0	0	Spring

Date	Species	Site	Month	Year	Survey Year	Presence	Data Gap	Season
5/7/2019	BL	6M	5	2019	Year-2		1	Spring
5/7/2019	BL	7M	5	2019	Year-2	0	0	Spring
5/7/2019	BL	10M	5	2019	Year-2	0	0	Spring
5/7/2019	BL	12M	5	2019	Year-2	0	0	Spring
5/7/2019	BL	14M	5	2019	Year-2	0	0	Spring
5/7/2019	BL	8A	5	2019	Year-2	0	0	Spring
5/7/2019	BL	9A	5	2019	Year-2	0	0	Spring
5/7/2019	BL	11A	5	2019	Year-2	0	0	Spring
5/7/2019	BL	13A	5	2019	Year-2	0	0	Spring
5/8/2019	BL	1M	5	2019	Year-2	0	0	Spring
5/8/2019	BL	2M	5	2019	Year-2	0	0	Spring
5/8/2019	BL	3M	5	2019	Year-2	0	0	Spring
5/8/2019	BL	4M	5	2019	Year-2	0	0	Spring
5/8/2019	BL	5M	5	2019	Year-2	0	0	Spring
5/8/2019	BL	6M	5	2019	Year-2		1	Spring
5/8/2019	BL	7M	5	2019	Year-2	0	0	Spring
5/8/2019	BL	10M	5	2019	Year-2	0	0	Spring
5/8/2019	BL	12M	5	2019	Year-2	0	0	Spring
5/8/2019	BL	14M	5	2019	Year-2	0	0	Spring
5/8/2019	BL	8A	5	2019	Year-2	0	0	Spring
5/8/2019	BL	9A	5	2019	Year-2	0	0	Spring
5/8/2019	BL	11A	5	2019	Year-2	0	0	Spring
5/8/2019	BL	13A	5	2019	Year-2	0	0	Spring
5/9/2019	BL	1M	5	2019	Year-2	0	0	Spring
5/9/2019	BL	2M	5	2019	Year-2	0	0	Spring
5/9/2019	BL	3M	5	2019	Year-2	0	0	Spring
5/9/2019	BL	4M	5	2019	Year-2	0	0	Spring
5/9/2019	BL	5M	5	2019	Year-2	0	0	Spring
5/9/2019	BL	6M	5	2019	Year-2		1	Spring
5/9/2019	BL	7M	5	2019	Year-2	0	0	Spring
5/9/2019	BL	10M	5	2019	Year-2	0	0	Spring
5/9/2019	BL	12M	5	2019	Year-2	0	0	Spring
5/9/2019	BL	14M	5	2019	Year-2	0	0	Spring
5/9/2019	BL	8A	5	2019	Year-2	0	0	Spring
5/9/2019	BL	9A	5	2019	Year-2	0	0	Spring
5/9/2019	BL	11A	5	2019	Year-2	0	0	Spring
5/9/2019	BL	13A	5	2019	Year-2	0	0	Spring
5/10/2019	BL	1M	5	2019	Year-2	0	0	Spring
5/10/2019	BL	2M	5	2019	Year-2	0	0	Spring
5/10/2019	BL	3M	5	2019	Year-2	0	0	Spring
5/10/2019	BL	4M	5	2019	Year-2	0	0	Spring
5/10/2019	BL	5M	5	2019	Year-2	0	0	Spring
5/10/2019	BL	6M	5	2019	Year-2		1	Spring
5/10/2019	BL	7M	5	2019	Year-2	0	0	Spring

Date	Species	Site	Month	Year	Survey Year	Presence	Data Gap	Season
5/10/2019	BL	10M	5	2019	Year-2	0	0	Spring
5/10/2019	BL	12M	5	2019	Year-2	0	0	Spring
5/10/2019	BL	14M	5	2019	Year-2	0	0	Spring
5/10/2019	BL	8A	5	2019	Year-2	0	0	Spring
5/10/2019	BL	9A	5	2019	Year-2	0	0	Spring
5/10/2019	BL	11A	5	2019	Year-2	0	0	Spring
5/10/2019	BL	13A	5	2019	Year-2	0	0	Spring
5/11/2019	BL	1M	5	2019	Year-2	0	0	Spring
5/11/2019	BL	2M	5	2019	Year-2	0	0	Spring
5/11/2019	BL	3M	5	2019	Year-2	0	0	Spring
5/11/2019	BL	4M	5	2019	Year-2	0	0	Spring
5/11/2019	BL	5M	5	2019	Year-2	0	0	Spring
5/11/2019	BL	6M	5	2019	Year-2		1	Spring
5/11/2019	BL	7M	5	2019	Year-2	0	0	Spring
5/11/2019	BL	10M	5	2019	Year-2	0	0	Spring
5/11/2019	BL	12M	5	2019	Year-2	0	0	Spring
5/11/2019	BL	14M	5	2019	Year-2	0	0	Spring
5/11/2019	BL	8A	5	2019	Year-2	0	0	Spring
5/11/2019	BL	9A	5	2019	Year-2	0	0	Spring
5/11/2019	BL	11A	5	2019	Year-2	0	0	Spring
5/11/2019	BL	13A	5	2019	Year-2	0	0	Spring
5/12/2019	BL	1M	5	2019	Year-2	0	0	Spring
5/12/2019	BL	2M	5	2019	Year-2	0	0	Spring
5/12/2019	BL	3M	5	2019	Year-2	0	0	Spring
5/12/2019	BL	4M	5	2019	Year-2	0	0	Spring
5/12/2019	BL	5M	5	2019	Year-2	0	0	Spring
5/12/2019	BL	6M	5	2019	Year-2		1	Spring
5/12/2019	BL	7M	5	2019	Year-2	0	0	Spring
5/12/2019	BL	10M	5	2019	Year-2	0	0	Spring
5/12/2019	BL	12M	5	2019	Year-2	0	0	Spring
5/12/2019	BL	14M	5	2019	Year-2	0	0	Spring
5/12/2019	BL	8A	5	2019	Year-2	0	0	Spring
5/12/2019	BL	9A	5	2019	Year-2	0	0	Spring
5/12/2019	BL	11A	5	2019	Year-2	0	0	Spring
5/12/2019	BL	13A	5	2019	Year-2	0	0	Spring
5/13/2019	BL	1M	5	2019	Year-2	0	0	Spring
5/13/2019	BL	2M	5	2019	Year-2	0	0	Spring
5/13/2019	BL	3M	5	2019	Year-2	0	0	Spring
5/13/2019	BL	4M	5	2019	Year-2	0	0	Spring
5/13/2019	BL	5M	5	2019	Year-2	0	0	Spring
5/13/2019	BL	6M	5	2019	Year-2		1	Spring
5/13/2019	BL	7M	5	2019	Year-2	0	0	Spring
5/13/2019	BL	10M	5	2019	Year-2	0	0	Spring
5/13/2019	BL	12M	5	2019	Year-2	0	0	Spring

Date	Species	Site	Month	Year	Survey Year	Presence	Data Gap	Season
5/13/2019	BL	14M	5	2019	Year-2	0	0	Spring
5/13/2019	BL	8A	5	2019	Year-2	0	0	Spring
5/13/2019	BL	9A	5	2019	Year-2	0	0	Spring
5/13/2019	BL	11A	5	2019	Year-2	0	0	Spring
5/13/2019	BL	13A	5	2019	Year-2	0	0	Spring
5/14/2019	BL	1M	5	2019	Year-2	0	0	Spring
5/14/2019	BL	2M	5	2019	Year-2	0	0	Spring
5/14/2019	BL	3M	5	2019	Year-2	0	0	Spring
5/14/2019	BL	4M	5	2019	Year-2	0	0	Spring
5/14/2019	BL	5M	5	2019	Year-2	0	0	Spring
5/14/2019	BL	6M	5	2019	Year-2		1	Spring
5/14/2019	BL	7M	5	2019	Year-2	0	0	Spring
5/14/2019	BL	10M	5	2019	Year-2	0	0	Spring
5/14/2019	BL	12M	5	2019	Year-2	0	0	Spring
5/14/2019	BL	14M	5	2019	Year-2	0	0	Spring
5/14/2019	BL	8A	5	2019	Year-2	0	0	Spring
5/14/2019	BL	9A	5	2019	Year-2	0	0	Spring
5/14/2019	BL	11A	5	2019	Year-2	0	0	Spring
5/14/2019	BL	13A	5	2019	Year-2	0	0	Spring
5/15/2019	BL	1M	5	2019	Year-2	0	0	Spring
5/15/2019	BL	2M	5	2019	Year-2	0	0	Spring
5/15/2019	BL	3M	5	2019	Year-2	0	0	Spring
5/15/2019	BL	4M	5	2019	Year-2	0	0	Spring
5/15/2019	BL	5M	5	2019	Year-2	0	0	Spring
5/15/2019	BL	6M	5	2019	Year-2		1	Spring
5/15/2019	BL	7M	5	2019	Year-2	0	0	Spring
5/15/2019	BL	10M	5	2019	Year-2	0	0	Spring
5/15/2019	BL	12M	5	2019	Year-2	0	0	Spring
5/15/2019	BL	14M	5	2019	Year-2	0	0	Spring
5/15/2019	BL	8A	5	2019	Year-2	0	0	Spring
5/15/2019	BL	9A	5	2019	Year-2	0	0	Spring
5/15/2019	BL	11A	5	2019	Year-2	0	0	Spring
5/15/2019	BL	13A	5	2019	Year-2	0	0	Spring
5/16/2019	BL	1M	5	2019	Year-2	0	0	Spring
5/16/2019	BL	2M	5	2019	Year-2	0	0	Spring
5/16/2019	BL	3M	5	2019	Year-2	0	0	Spring
5/16/2019	BL	4M	5	2019	Year-2	0	0	Spring
5/16/2019	BL	5M	5	2019	Year-2	0	0	Spring
5/16/2019	BL	6M	5	2019	Year-2		1	Spring
5/16/2019	BL	7M	5	2019	Year-2	0	0	Spring
5/16/2019	BL	10M	5	2019	Year-2	0	0	Spring
5/16/2019	BL	12M	5	2019	Year-2	0	0	Spring
5/16/2019	BL	14M	5	2019	Year-2	0	0	Spring
5/16/2019	BL	8A	5	2019	Year-2	0	0	Spring

Date	Species	Site	Month	Year	Survey Year	Presence	Data Gap	Season
5/16/2019	BL	9A	5	2019	Year-2	0	0	Spring
5/16/2019	BL	11A	5	2019	Year-2		1	Spring
5/16/2019	BL	13A	5	2019	Year-2	0	0	Spring
5/17/2019	BL	1M	5	2019	Year-2	0	0	Spring
5/17/2019	BL	2M	5	2019	Year-2	0	0	Spring
5/17/2019	BL	3M	5	2019	Year-2	0	0	Spring
5/17/2019	BL	4M	5	2019	Year-2	0	0	Spring
5/17/2019	BL	5M	5	2019	Year-2	0	0	Spring
5/17/2019	BL	6M	5	2019	Year-2		1	Spring
5/17/2019	BL	7M	5	2019	Year-2	0	0	Spring
5/17/2019	BL	10M	5	2019	Year-2	0	0	Spring
5/17/2019	BL	12M	5	2019	Year-2	0	0	Spring
5/17/2019	BL	14M	5	2019	Year-2	0	0	Spring
5/17/2019	BL	8A	5	2019	Year-2	0	0	Spring
5/17/2019	BL	9A	5	2019	Year-2	0	0	Spring
5/17/2019	BL	11A	5	2019	Year-2	0	0	Spring
5/17/2019	BL	13A	5	2019	Year-2	0	0	Spring
5/18/2019	BL	1M	5	2019	Year-2	0	0	Spring
5/18/2019	BL	2M	5	2019	Year-2	0	0	Spring
5/18/2019	BL	3M	5	2019	Year-2	0	0	Spring
5/18/2019	BL	4M	5	2019	Year-2	0	0	Spring
5/18/2019	BL	5M	5	2019	Year-2	0	0	Spring
5/18/2019	BL	6M	5	2019	Year-2		1	Spring
5/18/2019	BL	7M	5	2019	Year-2	0	0	Spring
5/18/2019	BL	10M	5	2019	Year-2	0	0	Spring
5/18/2019	BL	12M	5	2019	Year-2	0	0	Spring
5/18/2019	BL	14M	5	2019	Year-2	0	0	Spring
5/18/2019	BL	8A	5	2019	Year-2	0	0	Spring
5/18/2019	BL	9A	5	2019	Year-2	0	0	Spring
5/18/2019	BL	11A	5	2019	Year-2	0	0	Spring
5/18/2019	BL	13A	5	2019	Year-2	0	0	Spring
5/19/2019	BL	1M	5	2019	Year-2	0	0	Spring
5/19/2019	BL	2M	5	2019	Year-2	0	0	Spring
5/19/2019	BL	3M	5	2019	Year-2	0	0	Spring
5/19/2019	BL	4M	5	2019	Year-2	0	0	Spring
5/19/2019	BL	5M	5	2019	Year-2	0	0	Spring
5/19/2019	BL	6M	5	2019	Year-2		1	Spring
5/19/2019	BL	7M	5	2019	Year-2	0	0	Spring
5/19/2019	BL	10M	5	2019	Year-2	0	0	Spring
5/19/2019	BL	12M	5	2019	Year-2	0	0	Spring
5/19/2019	BL	14M	5	2019	Year-2	0	0	Spring
5/19/2019	BL	8A	5	2019	Year-2	0	0	Spring
5/19/2019	BL	9A	5	2019	Year-2	0	0	Spring
5/19/2019	BL	11A	5	2019	Year-2	0	0	Spring

Date	Species	Site	Month	Year	Survey Year	Presence	Data Gap	Season
5/19/2019	BL	13A	5	2019	Year-2	0	0	Spring
5/20/2019	BL	1M	5	2019	Year-2	0	0	Spring
5/20/2019	BL	2M	5	2019	Year-2	0	0	Spring
5/20/2019	BL	3M	5	2019	Year-2	0	0	Spring
5/20/2019	BL	4M	5	2019	Year-2	0	0	Spring
5/20/2019	BL	5M	5	2019	Year-2	0	0	Spring
5/20/2019	BL	6M	5	2019	Year-2		1	Spring
5/20/2019	BL	7M	5	2019	Year-2	0	0	Spring
5/20/2019	BL	10M	5	2019	Year-2	0	0	Spring
5/20/2019	BL	12M	5	2019	Year-2	0	0	Spring
5/20/2019	BL	14M	5	2019	Year-2	0	0	Spring
5/20/2019	BL	8A	5	2019	Year-2	0	0	Spring
5/20/2019	BL	9A	5	2019	Year-2	0	0	Spring
5/20/2019	BL	11A	5	2019	Year-2	0	0	Spring
5/20/2019	BL	13A	5	2019	Year-2	0	0	Spring
5/21/2019	BL	1M	5	2019	Year-2	0	0	Spring
5/21/2019	BL	2M	5	2019	Year-2	0	0	Spring
5/21/2019	BL	3M	5	2019	Year-2	0	0	Spring
5/21/2019	BL	4M	5	2019	Year-2	0	0	Spring
5/21/2019	BL	5M	5	2019	Year-2	0	0	Spring
5/21/2019	BL	6M	5	2019	Year-2		1	Spring
5/21/2019	BL	7M	5	2019	Year-2	0	0	Spring
5/21/2019	BL	10M	5	2019	Year-2	0	0	Spring
5/21/2019	BL	12M	5	2019	Year-2	0	0	Spring
5/21/2019	BL	14M	5	2019	Year-2	0	0	Spring
5/21/2019	BL	8A	5	2019	Year-2	0	0	Spring
5/21/2019	BL	9A	5	2019	Year-2	0	0	Spring
5/21/2019	BL	11A	5	2019	Year-2	0	0	Spring
5/21/2019	BL	13A	5	2019	Year-2	0	0	Spring
5/22/2019	BL	1M	5	2019	Year-2	0	0	Spring
5/22/2019	BL	2M	5	2019	Year-2	0	0	Spring
5/22/2019	BL	3M	5	2019	Year-2	0	0	Spring
5/22/2019	BL	4M	5	2019	Year-2	0	0	Spring
5/22/2019	BL	5M	5	2019	Year-2	0	0	Spring
5/22/2019	BL	6M	5	2019	Year-2		1	Spring
5/22/2019	BL	7M	5	2019	Year-2	0	0	Spring
5/22/2019	BL	10M	5	2019	Year-2	0	0	Spring
5/22/2019	BL	12M	5	2019	Year-2	0	0	Spring
5/22/2019	BL	14M	5	2019	Year-2	0	0	Spring
5/22/2019	BL	8A	5	2019	Year-2	0	0	Spring
5/22/2019	BL	9A	5	2019	Year-2	0	0	Spring
5/22/2019	BL	11A	5	2019	Year-2	0	0	Spring
5/22/2019	BL	13A	5	2019	Year-2	0	0	Spring
5/23/2019	BL	1M	5	2019	Year-2	0	0	Spring

Date	Species	Site	Month	Year	Survey Year	Presence	Data Gap	Season
5/23/2019	BL	2M	5	2019	Year-2	0	0	Spring
5/23/2019	BL	3M	5	2019	Year-2	0	0	Spring
5/23/2019	BL	4M	5	2019	Year-2	0	0	Spring
5/23/2019	BL	5M	5	2019	Year-2	0	0	Spring
5/23/2019	BL	6M	5	2019	Year-2		1	Spring
5/23/2019	BL	7M	5	2019	Year-2	0	0	Spring
5/23/2019	BL	10M	5	2019	Year-2	0	0	Spring
5/23/2019	BL	12M	5	2019	Year-2	0	0	Spring
5/23/2019	BL	14M	5	2019	Year-2	0	0	Spring
5/23/2019	BL	8A	5	2019	Year-2	0	0	Spring
5/23/2019	BL	9A	5	2019	Year-2	0	0	Spring
5/23/2019	BL	11A	5	2019	Year-2	0	0	Spring
5/23/2019	BL	13A	5	2019	Year-2	0	0	Spring
5/24/2019	BL	1M	5	2019	Year-2	0	0	Spring
5/24/2019	BL	2M	5	2019	Year-2	0	0	Spring
5/24/2019	BL	3M	5	2019	Year-2	0	0	Spring
5/24/2019	BL	4M	5	2019	Year-2	0	0	Spring
5/24/2019	BL	5M	5	2019	Year-2	0	0	Spring
5/24/2019	BL	6M	5	2019	Year-2		1	Spring
5/24/2019	BL	7M	5	2019	Year-2	0	0	Spring
5/24/2019	BL	10M	5	2019	Year-2	0	0	Spring
5/24/2019	BL	12M	5	2019	Year-2	0	0	Spring
5/24/2019	BL	14M	5	2019	Year-2	0	0	Spring
5/24/2019	BL	8A	5	2019	Year-2	0	0	Spring
5/24/2019	BL	9A	5	2019	Year-2	0	0	Spring
5/24/2019	BL	11A	5	2019	Year-2	0	0	Spring
5/24/2019	BL	13A	5	2019	Year-2	0	0	Spring
5/25/2019	BL	1M	5	2019	Year-2	0	0	Spring
5/25/2019	BL	2M	5	2019	Year-2	0	0	Spring
5/25/2019	BL	3M	5	2019	Year-2	0	0	Spring
5/25/2019	BL	4M	5	2019	Year-2	0	0	Spring
5/25/2019	BL	5M	5	2019	Year-2	0	0	Spring
5/25/2019	BL	6M	5	2019	Year-2		1	Spring
5/25/2019	BL	7M	5	2019	Year-2	0	0	Spring
5/25/2019	BL	10M	5	2019	Year-2	0	0	Spring
5/25/2019	BL	12M	5	2019	Year-2	0	0	Spring
5/25/2019	BL	14M	5	2019	Year-2	0	0	Spring
5/25/2019	BL	8A	5	2019	Year-2	0	0	Spring
5/25/2019	BL	9A	5	2019	Year-2	0	0	Spring
5/25/2019	BL	11A	5	2019	Year-2	0	0	Spring
5/25/2019	BL	13A	5	2019	Year-2	0	0	Spring
5/26/2019	BL	1M	5	2019	Year-2	0	0	Spring
5/26/2019	BL	2M	5	2019	Year-2	0	0	Spring
5/26/2019	BL	3M	5	2019	Year-2	0	0	Spring

Date	Species	Site	Month	Year	Survey Year	Presence	Data Gap	Season
5/26/2019	BL	4M	5	2019	Year-2	0	0	Spring
5/26/2019	BL	5M	5	2019	Year-2	0	0	Spring
5/26/2019	BL	6M	5	2019	Year-2		1	Spring
5/26/2019	BL	7M	5	2019	Year-2	0	0	Spring
5/26/2019	BL	10M	5	2019	Year-2	0	0	Spring
5/26/2019	BL	12M	5	2019	Year-2	0	0	Spring
5/26/2019	BL	14M	5	2019	Year-2	0	0	Spring
5/26/2019	BL	8A	5	2019	Year-2	0	0	Spring
5/26/2019	BL	9A	5	2019	Year-2	0	0	Spring
5/26/2019	BL	11A	5	2019	Year-2	0	0	Spring
5/26/2019	BL	13A	5	2019	Year-2	0	0	Spring
5/27/2019	BL	1M	5	2019	Year-2	0	0	Spring
5/27/2019	BL	2M	5	2019	Year-2	0	0	Spring
5/27/2019	BL	3M	5	2019	Year-2	0	0	Spring
5/27/2019	BL	4M	5	2019	Year-2	0	0	Spring
5/27/2019	BL	5M	5	2019	Year-2	0	0	Spring
5/27/2019	BL	6M	5	2019	Year-2		1	Spring
5/27/2019	BL	7M	5	2019	Year-2	0	0	Spring
5/27/2019	BL	10M	5	2019	Year-2	0	0	Spring
5/27/2019	BL	12M	5	2019	Year-2	0	0	Spring
5/27/2019	BL	14M	5	2019	Year-2	0	0	Spring
5/27/2019	BL	8A	5	2019	Year-2	0	0	Spring
5/27/2019	BL	9A	5	2019	Year-2	0	0	Spring
5/27/2019	BL	11A	5	2019	Year-2	0	0	Spring
5/27/2019	BL	13A	5	2019	Year-2	0	0	Spring
5/28/2019	BL	1M	5	2019	Year-2	0	0	Spring
5/28/2019	BL	2M	5	2019	Year-2	0	0	Spring
5/28/2019	BL	3M	5	2019	Year-2	0	0	Spring
5/28/2019	BL	4M	5	2019	Year-2	0	0	Spring
5/28/2019	BL	5M	5	2019	Year-2	0	0	Spring
5/28/2019	BL	6M	5	2019	Year-2		1	Spring
5/28/2019	BL	7M	5	2019	Year-2	0	0	Spring
5/28/2019	BL	10M	5	2019	Year-2	0	0	Spring
5/28/2019	BL	12M	5	2019	Year-2	0	0	Spring
5/28/2019	BL	14M	5	2019	Year-2	0	0	Spring
5/28/2019	BL	8A	5	2019	Year-2	0	0	Spring
5/28/2019	BL	9A	5	2019	Year-2	0	0	Spring
5/28/2019	BL	11A	5	2019	Year-2	0	0	Spring
5/28/2019	BL	13A	5	2019	Year-2	0	0	Spring
5/29/2019	BL	1M	5	2019	Year-2	0	0	Spring
5/29/2019	BL	2M	5	2019	Year-2	0	0	Spring
5/29/2019	BL	3M	5	2019	Year-2	0	0	Spring
5/29/2019	BL	4M	5	2019	Year-2	0	0	Spring
5/29/2019	BL	5M	5	2019	Year-2	0	0	Spring

Date	Species	Site	Month	Year	Survey Year	Presence	Data Gap	Season
5/29/2019	BL	6M	5	2019	Year-2		1	Spring
5/29/2019	BL	7M	5	2019	Year-2	0	0	Spring
5/29/2019	BL	10M	5	2019	Year-2	0	0	Spring
5/29/2019	BL	12M	5	2019	Year-2	0	0	Spring
5/29/2019	BL	14M	5	2019	Year-2	0	0	Spring
5/29/2019	BL	8A	5	2019	Year-2	0	0	Spring
5/29/2019	BL	9A	5	2019	Year-2	0	0	Spring
5/29/2019	BL	11A	5	2019	Year-2	0	0	Spring
5/29/2019	BL	13A	5	2019	Year-2	0	0	Spring
5/30/2019	BL	1M	5	2019	Year-2	0	0	Spring
5/30/2019	BL	2M	5	2019	Year-2	0	0	Spring
5/30/2019	BL	3M	5	2019	Year-2	0	0	Spring
5/30/2019	BL	4M	5	2019	Year-2	0	0	Spring
5/30/2019	BL	5M	5	2019	Year-2	0	0	Spring
5/30/2019	BL	6M	5	2019	Year-2		1	Spring
5/30/2019	BL	7M	5	2019	Year-2	0	0	Spring
5/30/2019	BL	10M	5	2019	Year-2	0	0	Spring
5/30/2019	BL	12M	5	2019	Year-2	0	0	Spring
5/30/2019	BL	14M	5	2019	Year-2	0	0	Spring
5/30/2019	BL	8A	5	2019	Year-2	0	0	Spring
5/30/2019	BL	9A	5	2019	Year-2	0	0	Spring
5/30/2019	BL	11A	5	2019	Year-2	0	0	Spring
5/30/2019	BL	13A	5	2019	Year-2	0	0	Spring
5/31/2019	BL	1M	5	2019	Year-2	0	0	Spring
5/31/2019	BL	2M	5	2019	Year-2	0	0	Spring
5/31/2019	BL	3M	5	2019	Year-2	0	0	Spring
5/31/2019	BL	4M	5	2019	Year-2	0	0	Spring
5/31/2019	BL	5M	5	2019	Year-2	0	0	Spring
5/31/2019	BL	6M	5	2019	Year-2		1	Spring
5/31/2019	BL	7M	5	2019	Year-2	0	0	Spring
5/31/2019	BL	10M	5	2019	Year-2	0	0	Spring
5/31/2019	BL	12M	5	2019	Year-2	0	0	Spring
5/31/2019	BL	14M	5	2019	Year-2	0	0	Spring
5/31/2019	BL	8A	5	2019	Year-2	0	0	Spring
5/31/2019	BL	9A	5	2019	Year-2	0	0	Spring
5/31/2019	BL	11A	5	2019	Year-2	0	0	Spring
5/31/2019	BL	13A	5	2019	Year-2	0	0	Spring
6/1/2019	BL	1M	6	2019	Year-2	0	0	Summer
6/1/2019	BL	2M	6	2019	Year-2	0	0	Summer
6/1/2019	BL	3M	6	2019	Year-2	0	0	Summer
6/1/2019	BL	4M	6	2019	Year-2	0	0	Summer
6/1/2019	BL	5M	6	2019	Year-2	0	0	Summer
6/1/2019	BL	6M	6	2019	Year-2		1	Summer
6/1/2019	BL	7M	6	2019	Year-2	0	0	Summer

Date	Species	Site	Month	Year	Survey Year	Presence	Data Gap	Season
6/1/2019	BL	10M	6	2019	Year-2	0	0	Summer
6/1/2019	BL	12M	6	2019	Year-2	0	0	Summer
6/1/2019	BL	14M	6	2019	Year-2	0	0	Summer
6/1/2019	BL	8A	6	2019	Year-2	0	0	Summer
6/1/2019	BL	9A	6	2019	Year-2	0	0	Summer
6/1/2019	BL	11A	6	2019	Year-2	0	0	Summer
6/1/2019	BL	13A	6	2019	Year-2	0	0	Summer
6/2/2019	BL	1M	6	2019	Year-2	0	0	Summer
6/2/2019	BL	2M	6	2019	Year-2	0	0	Summer
6/2/2019	BL	3M	6	2019	Year-2	0	0	Summer
6/2/2019	BL	4M	6	2019	Year-2	0	0	Summer
6/2/2019	BL	5M	6	2019	Year-2	0	0	Summer
6/2/2019	BL	6M	6	2019	Year-2		1	Summer
6/2/2019	BL	7M	6	2019	Year-2	0	0	Summer
6/2/2019	BL	10M	6	2019	Year-2	0	0	Summer
6/2/2019	BL	12M	6	2019	Year-2	0	0	Summer
6/2/2019	BL	14M	6	2019	Year-2	0	0	Summer
6/2/2019	BL	8A	6	2019	Year-2	0	0	Summer
6/2/2019	BL	9A	6	2019	Year-2	0	0	Summer
6/2/2019	BL	11A	6	2019	Year-2	0	0	Summer
6/2/2019	BL	13A	6	2019	Year-2	0	0	Summer
6/3/2019	BL	1M	6	2019	Year-2	0	0	Summer
6/3/2019	BL	2M	6	2019	Year-2	0	0	Summer
6/3/2019	BL	3M	6	2019	Year-2	0	0	Summer
6/3/2019	BL	4M	6	2019	Year-2	0	0	Summer
6/3/2019	BL	5M	6	2019	Year-2	0	0	Summer
6/3/2019	BL	6M	6	2019	Year-2		1	Summer
6/3/2019	BL	7M	6	2019	Year-2	0	0	Summer
6/3/2019	BL	10M	6	2019	Year-2	0	0	Summer
6/3/2019	BL	12M	6	2019	Year-2	0	0	Summer
6/3/2019	BL	14M	6	2019	Year-2	0	0	Summer
6/3/2019	BL	8A	6	2019	Year-2	0	0	Summer
6/3/2019	BL	9A	6	2019	Year-2	0	0	Summer
6/3/2019	BL	11A	6	2019	Year-2	0	0	Summer
6/3/2019	BL	13A	6	2019	Year-2	0	0	Summer
6/4/2019	BL	1M	6	2019	Year-2	0	0	Summer
6/4/2019	BL	2M	6	2019	Year-2	0	0	Summer
6/4/2019	BL	3M	6	2019	Year-2	0	0	Summer
6/4/2019	BL	4M	6	2019	Year-2	0	0	Summer
6/4/2019	BL	5M	6	2019	Year-2	0	0	Summer
6/4/2019	BL	6M	6	2019	Year-2		1	Summer
6/4/2019	BL	7M	6	2019	Year-2	0	0	Summer
6/4/2019	BL	10M	6	2019	Year-2	0	0	Summer
6/4/2019	BL	12M	6	2019	Year-2	0	0	Summer

Date	Species	Site	Month	Year	Survey Year	Presence	Data Gap	Season
6/4/2019	BL	14M	6	2019	Year-2	0	0	Summer
6/4/2019	BL	8A	6	2019	Year-2	0	0	Summer
6/4/2019	BL	9A	6	2019	Year-2	0	0	Summer
6/4/2019	BL	11A	6	2019	Year-2	0	0	Summer
6/4/2019	BL	13A	6	2019	Year-2	0	0	Summer
6/5/2019	BL	1M	6	2019	Year-2	0	0	Summer
6/5/2019	BL	2M	6	2019	Year-2	0	0	Summer
6/5/2019	BL	3M	6	2019	Year-2	0	0	Summer
6/5/2019	BL	4M	6	2019	Year-2	0	0	Summer
6/5/2019	BL	5M	6	2019	Year-2	0	0	Summer
6/5/2019	BL	6M	6	2019	Year-2		1	Summer
6/5/2019	BL	7M	6	2019	Year-2	0	0	Summer
6/5/2019	BL	10M	6	2019	Year-2	0	0	Summer
6/5/2019	BL	12M	6	2019	Year-2	0	0	Summer
6/5/2019	BL	14M	6	2019	Year-2	0	0	Summer
6/5/2019	BL	8A	6	2019	Year-2	0	0	Summer
6/5/2019	BL	9A	6	2019	Year-2	0	0	Summer
6/5/2019	BL	11A	6	2019	Year-2	0	0	Summer
6/5/2019	BL	13A	6	2019	Year-2	0	0	Summer
6/6/2019	BL	1M	6	2019	Year-2	0	0	Summer
6/6/2019	BL	2M	6	2019	Year-2	0	0	Summer
6/6/2019	BL	3M	6	2019	Year-2	0	0	Summer
6/6/2019	BL	4M	6	2019	Year-2	0	0	Summer
6/6/2019	BL	5M	6	2019	Year-2	0	0	Summer
6/6/2019	BL	6M	6	2019	Year-2		1	Summer
6/6/2019	BL	7M	6	2019	Year-2	0	0	Summer
6/6/2019	BL	10M	6	2019	Year-2	0	0	Summer
6/6/2019	BL	12M	6	2019	Year-2	0	0	Summer
6/6/2019	BL	14M	6	2019	Year-2	0	0	Summer
6/6/2019	BL	8A	6	2019	Year-2	0	0	Summer
6/6/2019	BL	9A	6	2019	Year-2	0	0	Summer
6/6/2019	BL	11A	6	2019	Year-2	0	0	Summer
6/6/2019	BL	13A	6	2019	Year-2	0	0	Summer
6/7/2019	BL	1M	6	2019	Year-2	0	0	Summer
6/7/2019	BL	2M	6	2019	Year-2	0	0	Summer
6/7/2019	BL	3M	6	2019	Year-2	0	0	Summer
6/7/2019	BL	4M	6	2019	Year-2	0	0	Summer
6/7/2019	BL	5M	6	2019	Year-2	0	0	Summer
6/7/2019	BL	6M	6	2019	Year-2		1	Summer
6/7/2019	BL	7M	6	2019	Year-2	0	0	Summer
6/7/2019	BL	10M	6	2019	Year-2	0	0	Summer
6/7/2019	BL	12M	6	2019	Year-2	0	0	Summer
6/7/2019	BL	14M	6	2019	Year-2	0	0	Summer
6/7/2019	BL	8A	6	2019	Year-2	0	0	Summer

Date	Species	Site	Month	Year	Survey Year	Presence	Data Gap	Season
6/7/2019	BL	9A	6	2019	Year-2	0	0	Summer
6/7/2019	BL	11A	6	2019	Year-2	0	0	Summer
6/7/2019	BL	13A	6	2019	Year-2	0	0	Summer
6/8/2019	BL	1M	6	2019	Year-2	0	0	Summer
6/8/2019	BL	2M	6	2019	Year-2	0	0	Summer
6/8/2019	BL	3M	6	2019	Year-2	0	0	Summer
6/8/2019	BL	4M	6	2019	Year-2	0	0	Summer
6/8/2019	BL	5M	6	2019	Year-2	0	0	Summer
6/8/2019	BL	6M	6	2019	Year-2		1	Summer
6/8/2019	BL	7M	6	2019	Year-2	0	0	Summer
6/8/2019	BL	10M	6	2019	Year-2	0	0	Summer
6/8/2019	BL	12M	6	2019	Year-2	0	0	Summer
6/8/2019	BL	14M	6	2019	Year-2	0	0	Summer
6/8/2019	BL	8A	6	2019	Year-2	0	0	Summer
6/8/2019	BL	9A	6	2019	Year-2	0	0	Summer
6/8/2019	BL	11A	6	2019	Year-2	0	0	Summer
6/8/2019	BL	13A	6	2019	Year-2	0	0	Summer
6/9/2019	BL	1M	6	2019	Year-2	0	0	Summer
6/9/2019	BL	2M	6	2019	Year-2	0	0	Summer
6/9/2019	BL	3M	6	2019	Year-2	0	0	Summer
6/9/2019	BL	4M	6	2019	Year-2	0	0	Summer
6/9/2019	BL	5M	6	2019	Year-2	0	0	Summer
6/9/2019	BL	6M	6	2019	Year-2		1	Summer
6/9/2019	BL	7M	6	2019	Year-2	0	0	Summer
6/9/2019	BL	10M	6	2019	Year-2	0	0	Summer
6/9/2019	BL	12M	6	2019	Year-2	0	0	Summer
6/9/2019	BL	14M	6	2019	Year-2	0	0	Summer
6/9/2019	BL	8A	6	2019	Year-2	0	0	Summer
6/9/2019	BL	9A	6	2019	Year-2	0	0	Summer
6/9/2019	BL	11A	6	2019	Year-2	0	0	Summer
6/9/2019	BL	13A	6	2019	Year-2	0	0	Summer
6/10/2019	BL	1M	6	2019	Year-2	0	0	Summer
6/10/2019	BL	2M	6	2019	Year-2	0	0	Summer
6/10/2019	BL	3M	6	2019	Year-2	0	0	Summer
6/10/2019	BL	4M	6	2019	Year-2	0	0	Summer
6/10/2019	BL	5M	6	2019	Year-2	0	0	Summer
6/10/2019	BL	6M	6	2019	Year-2		1	Summer
6/10/2019	BL	7M	6	2019	Year-2	0	0	Summer
6/10/2019	BL	10M	6	2019	Year-2	0	0	Summer
6/10/2019	BL	12M	6	2019	Year-2	0	0	Summer
6/10/2019	BL	14M	6	2019	Year-2	0	0	Summer
6/10/2019	BL	8A	6	2019	Year-2	0	0	Summer
6/10/2019	BL	9A	6	2019	Year-2	0	0	Summer
6/10/2019	BL	11A	6	2019	Year-2	0	0	Summer

Date	Species	Site	Month	Year	Survey Year	Presence	Data Gap	Season
6/10/2019	BL	13A	6	2019	Year-2	0	0	Summer
6/11/2019	BL	1M	6	2019	Year-2	0	0	Summer
6/11/2019	BL	2M	6	2019	Year-2	0	0	Summer
6/11/2019	BL	3M	6	2019	Year-2	0	0	Summer
6/11/2019	BL	4M	6	2019	Year-2	0	0	Summer
6/11/2019	BL	5M	6	2019	Year-2	0	0	Summer
6/11/2019	BL	6M	6	2019	Year-2		1	Summer
6/11/2019	BL	7M	6	2019	Year-2	0	0	Summer
6/11/2019	BL	10M	6	2019	Year-2	0	0	Summer
6/11/2019	BL	12M	6	2019	Year-2	0	0	Summer
6/11/2019	BL	14M	6	2019	Year-2	0	0	Summer
6/11/2019	BL	8A	6	2019	Year-2	0	0	Summer
6/11/2019	BL	9A	6	2019	Year-2	0	0	Summer
6/11/2019	BL	11A	6	2019	Year-2	0	0	Summer
6/11/2019	BL	13A	6	2019	Year-2	0	0	Summer
6/12/2019	BL	1M	6	2019	Year-2	0	0	Summer
6/12/2019	BL	2M	6	2019	Year-2	0	0	Summer
6/12/2019	BL	3M	6	2019	Year-2	0	0	Summer
6/12/2019	BL	4M	6	2019	Year-2	0	0	Summer
6/12/2019	BL	5M	6	2019	Year-2	0	0	Summer
6/12/2019	BL	6M	6	2019	Year-2		1	Summer
6/12/2019	BL	7M	6	2019	Year-2	0	0	Summer
6/12/2019	BL	10M	6	2019	Year-2	0	0	Summer
6/12/2019	BL	12M	6	2019	Year-2	0	0	Summer
6/12/2019	BL	14M	6	2019	Year-2	0	0	Summer
6/12/2019	BL	8A	6	2019	Year-2	0	0	Summer
6/12/2019	BL	9A	6	2019	Year-2	0	0	Summer
6/12/2019	BL	11A	6	2019	Year-2	0	0	Summer
6/12/2019	BL	13A	6	2019	Year-2	0	0	Summer
6/13/2019	BL	1M	6	2019	Year-2	0	0	Summer
6/13/2019	BL	2M	6	2019	Year-2	0	0	Summer
6/13/2019	BL	3M	6	2019	Year-2	0	0	Summer
6/13/2019	BL	4M	6	2019	Year-2	0	0	Summer
6/13/2019	BL	5M	6	2019	Year-2	0	0	Summer
6/13/2019	BL	6M	6	2019	Year-2		1	Summer
6/13/2019	BL	7M	6	2019	Year-2	0	0	Summer
6/13/2019	BL	10M	6	2019	Year-2	0	0	Summer
6/13/2019	BL	12M	6	2019	Year-2	0	0	Summer
6/13/2019	BL	14M	6	2019	Year-2	0	0	Summer
6/13/2019	BL	8A	6	2019	Year-2	0	0	Summer
6/13/2019	BL	9A	6	2019	Year-2	0	0	Summer
6/13/2019	BL	11A	6	2019	Year-2	0	0	Summer
6/13/2019	BL	13A	6	2019	Year-2	0	0	Summer
6/14/2019	BL	1M	6	2019	Year-2	0	0	Summer

Date	Species	Site	Month	Year	Survey Year	Presence	Data Gap	Season
6/14/2019	BL	2M	6	2019	Year-2	0	0	Summer
6/14/2019	BL	3M	6	2019	Year-2	0	0	Summer
6/14/2019	BL	4M	6	2019	Year-2	0	0	Summer
6/14/2019	BL	5M	6	2019	Year-2	0	0	Summer
6/14/2019	BL	6M	6	2019	Year-2		1	Summer
6/14/2019	BL	7M	6	2019	Year-2	0	0	Summer
6/14/2019	BL	10M	6	2019	Year-2	0	0	Summer
6/14/2019	BL	12M	6	2019	Year-2	0	0	Summer
6/14/2019	BL	14M	6	2019	Year-2	0	0	Summer
6/14/2019	BL	8A	6	2019	Year-2	0	0	Summer
6/14/2019	BL	9A	6	2019	Year-2	0	0	Summer
6/14/2019	BL	11A	6	2019	Year-2	0	0	Summer
6/14/2019	BL	13A	6	2019	Year-2	0	0	Summer
6/15/2019	BL	1M	6	2019	Year-2	0	0	Summer
6/15/2019	BL	2M	6	2019	Year-2	0	0	Summer
6/15/2019	BL	3M	6	2019	Year-2	0	0	Summer
6/15/2019	BL	4M	6	2019	Year-2	0	0	Summer
6/15/2019	BL	5M	6	2019	Year-2	0	0	Summer
6/15/2019	BL	6M	6	2019	Year-2		1	Summer
6/15/2019	BL	7M	6	2019	Year-2	0	0	Summer
6/15/2019	BL	10M	6	2019	Year-2	0	0	Summer
6/15/2019	BL	12M	6	2019	Year-2	0	0	Summer
6/15/2019	BL	14M	6	2019	Year-2	0	0	Summer
6/15/2019	BL	8A	6	2019	Year-2	0	0	Summer
6/15/2019	BL	9A	6	2019	Year-2	0	0	Summer
6/15/2019	BL	11A	6	2019	Year-2	0	0	Summer
6/15/2019	BL	13A	6	2019	Year-2	0	0	Summer
6/16/2019	BL	1M	6	2019	Year-2	0	0	Summer
6/16/2019	BL	2M	6	2019	Year-2	0	0	Summer
6/16/2019	BL	3M	6	2019	Year-2	0	0	Summer
6/16/2019	BL	4M	6	2019	Year-2	0	0	Summer
6/16/2019	BL	5M	6	2019	Year-2	0	0	Summer
6/16/2019	BL	6M	6	2019	Year-2		1	Summer
6/16/2019	BL	7M	6	2019	Year-2	0	0	Summer
6/16/2019	BL	10M	6	2019	Year-2	0	0	Summer
6/16/2019	BL	12M	6	2019	Year-2	0	0	Summer
6/16/2019	BL	14M	6	2019	Year-2	0	0	Summer
6/16/2019	BL	8A	6	2019	Year-2	0	0	Summer
6/16/2019	BL	9A	6	2019	Year-2	0	0	Summer
6/16/2019	BL	11A	6	2019	Year-2	0	0	Summer
6/16/2019	BL	13A	6	2019	Year-2	0	0	Summer
6/17/2019	BL	1M	6	2019	Year-2	0	0	Summer
6/17/2019	BL	2M	6	2019	Year-2	0	0	Summer
6/17/2019	BL	3M	6	2019	Year-2	0	0	Summer

Date	Species	Site	Month	Year	Survey Year	Presence	Data Gap	Season
6/17/2019	BL	4M	6	2019	Year-2	0	0	Summer
6/17/2019	BL	5M	6	2019	Year-2	0	0	Summer
6/17/2019	BL	6M	6	2019	Year-2		1	Summer
6/17/2019	BL	7M	6	2019	Year-2	0	0	Summer
6/17/2019	BL	10M	6	2019	Year-2	0	0	Summer
6/17/2019	BL	12M	6	2019	Year-2	0	0	Summer
6/17/2019	BL	14M	6	2019	Year-2	0	0	Summer
6/17/2019	BL	8A	6	2019	Year-2	0	0	Summer
6/17/2019	BL	9A	6	2019	Year-2	0	0	Summer
6/17/2019	BL	11A	6	2019	Year-2	0	0	Summer
6/17/2019	BL	13A	6	2019	Year-2	0	0	Summer
6/18/2019	BL	1M	6	2019	Year-2	0	0	Summer
6/18/2019	BL	2M	6	2019	Year-2	0	0	Summer
6/18/2019	BL	3M	6	2019	Year-2	0	0	Summer
6/18/2019	BL	4M	6	2019	Year-2	0	0	Summer
6/18/2019	BL	5M	6	2019	Year-2	0	0	Summer
6/18/2019	BL	6M	6	2019	Year-2		1	Summer
6/18/2019	BL	7M	6	2019	Year-2	0	0	Summer
6/18/2019	BL	10M	6	2019	Year-2	0	0	Summer
6/18/2019	BL	12M	6	2019	Year-2	0	0	Summer
6/18/2019	BL	14M	6	2019	Year-2	0	0	Summer
6/18/2019	BL	8A	6	2019	Year-2	0	0	Summer
6/18/2019	BL	9A	6	2019	Year-2	0	0	Summer
6/18/2019	BL	11A	6	2019	Year-2	0	0	Summer
6/18/2019	BL	13A	6	2019	Year-2	0	0	Summer
6/19/2019	BL	1M	6	2019	Year-2	0	0	Summer
6/19/2019	BL	2M	6	2019	Year-2	0	0	Summer
6/19/2019	BL	3M	6	2019	Year-2	0	0	Summer
6/19/2019	BL	4M	6	2019	Year-2	0	0	Summer
6/19/2019	BL	5M	6	2019	Year-2	0	0	Summer
6/19/2019	BL	6M	6	2019	Year-2		1	Summer
6/19/2019	BL	7M	6	2019	Year-2	0	0	Summer
6/19/2019	BL	10M	6	2019	Year-2	0	0	Summer
6/19/2019	BL	12M	6	2019	Year-2	0	0	Summer
6/19/2019	BL	14M	6	2019	Year-2	0	0	Summer
6/19/2019	BL	8A	6	2019	Year-2	0	0	Summer
6/19/2019	BL	9A	6	2019	Year-2	0	0	Summer
6/19/2019	BL	11A	6	2019	Year-2	0	0	Summer
6/19/2019	BL	13A	6	2019	Year-2	0	0	Summer
6/20/2019	BL	1M	6	2019	Year-2	0	0	Summer
6/20/2019	BL	2M	6	2019	Year-2	0	0	Summer
6/20/2019	BL	3M	6	2019	Year-2	0	0	Summer
6/20/2019	BL	4M	6	2019	Year-2	0	0	Summer
6/20/2019	BL	5M	6	2019	Year-2	0	0	Summer

Date	Species	Site	Month	Year	Survey Year	Presence	Data Gap	Season
6/20/2019	BL	6M	6	2019	Year-2		1	Summer
6/20/2019	BL	7M	6	2019	Year-2	0	0	Summer
6/20/2019	BL	10M	6	2019	Year-2	0	0	Summer
6/20/2019	BL	12M	6	2019	Year-2	0	0	Summer
6/20/2019	BL	14M	6	2019	Year-2	0	0	Summer
6/20/2019	BL	8A	6	2019	Year-2	0	0	Summer
6/20/2019	BL	9A	6	2019	Year-2	0	0	Summer
6/20/2019	BL	11A	6	2019	Year-2	0	0	Summer
6/20/2019	BL	13A	6	2019	Year-2	0	0	Summer
6/21/2019	BL	1M	6	2019	Year-2	0	0	Summer
6/21/2019	BL	2M	6	2019	Year-2	0	0	Summer
6/21/2019	BL	3M	6	2019	Year-2	0	0	Summer
6/21/2019	BL	4M	6	2019	Year-2	0	0	Summer
6/21/2019	BL	5M	6	2019	Year-2	0	0	Summer
6/21/2019	BL	6M	6	2019	Year-2		1	Summer
6/21/2019	BL	7M	6	2019	Year-2	0	0	Summer
6/21/2019	BL	10M	6	2019	Year-2	0	0	Summer
6/21/2019	BL	12M	6	2019	Year-2	0	0	Summer
6/21/2019	BL	14M	6	2019	Year-2	0	0	Summer
6/21/2019	BL	8A	6	2019	Year-2	0	0	Summer
6/21/2019	BL	9A	6	2019	Year-2	0	0	Summer
6/21/2019	BL	11A	6	2019	Year-2	0	0	Summer
6/21/2019	BL	13A	6	2019	Year-2	0	0	Summer
6/22/2019	BL	1M	6	2019	Year-2	0	0	Summer
6/22/2019	BL	2M	6	2019	Year-2	0	0	Summer
6/22/2019	BL	3M	6	2019	Year-2	0	0	Summer
6/22/2019	BL	4M	6	2019	Year-2	0	0	Summer
6/22/2019	BL	5M	6	2019	Year-2	0	0	Summer
6/22/2019	BL	6M	6	2019	Year-2		1	Summer
6/22/2019	BL	7M	6	2019	Year-2	0	0	Summer
6/22/2019	BL	10M	6	2019	Year-2	0	0	Summer
6/22/2019	BL	12M	6	2019	Year-2	0	0	Summer
6/22/2019	BL	14M	6	2019	Year-2	0	0	Summer
6/22/2019	BL	8A	6	2019	Year-2	0	0	Summer
6/22/2019	BL	9A	6	2019	Year-2	0	0	Summer
6/22/2019	BL	11A	6	2019	Year-2	0	0	Summer
6/22/2019	BL	13A	6	2019	Year-2	0	0	Summer
6/23/2019	BL	1M	6	2019	Year-2	0	0	Summer
6/23/2019	BL	2M	6	2019	Year-2	0	0	Summer
6/23/2019	BL	3M	6	2019	Year-2	0	0	Summer
6/23/2019	BL	4M	6	2019	Year-2	0	0	Summer
6/23/2019	BL	5M	6	2019	Year-2	0	0	Summer
6/23/2019	BL	6M	6	2019	Year-2		1	Summer
6/23/2019	BL	7M	6	2019	Year-2	0	0	Summer

Date	Species	Site	Month	Year	Survey Year	Presence	Data Gap	Season
6/23/2019	BL	10M	6	2019	Year-2	0	0	Summer
6/23/2019	BL	12M	6	2019	Year-2	0	0	Summer
6/23/2019	BL	14M	6	2019	Year-2	0	0	Summer
6/23/2019	BL	8A	6	2019	Year-2	0	0	Summer
6/23/2019	BL	9A	6	2019	Year-2	0	0	Summer
6/23/2019	BL	11A	6	2019	Year-2	0	0	Summer
6/23/2019	BL	13A	6	2019	Year-2	0	0	Summer
6/24/2019	BL	1M	6	2019	Year-2	0	0	Summer
6/24/2019	BL	2M	6	2019	Year-2	0	0	Summer
6/24/2019	BL	3M	6	2019	Year-2	0	0	Summer
6/24/2019	BL	4M	6	2019	Year-2	0	0	Summer
6/24/2019	BL	5M	6	2019	Year-2	0	0	Summer
6/24/2019	BL	6M	6	2019	Year-2		1	Summer
6/24/2019	BL	7M	6	2019	Year-2	0	0	Summer
6/24/2019	BL	10M	6	2019	Year-2	0	0	Summer
6/24/2019	BL	12M	6	2019	Year-2	0	0	Summer
6/24/2019	BL	14M	6	2019	Year-2	0	0	Summer
6/24/2019	BL	8A	6	2019	Year-2	0	0	Summer
6/24/2019	BL	9A	6	2019	Year-2	0	0	Summer
6/24/2019	BL	11A	6	2019	Year-2	0	0	Summer
6/24/2019	BL	13A	6	2019	Year-2	0	0	Summer
6/25/2019	BL	1M	6	2019	Year-2	0	0	Summer
6/25/2019	BL	2M	6	2019	Year-2	0	0	Summer
6/25/2019	BL	3M	6	2019	Year-2	0	0	Summer
6/25/2019	BL	4M	6	2019	Year-2	0	0	Summer
6/25/2019	BL	5M	6	2019	Year-2	0	0	Summer
6/25/2019	BL	6M	6	2019	Year-2		1	Summer
6/25/2019	BL	7M	6	2019	Year-2	0	0	Summer
6/25/2019	BL	10M	6	2019	Year-2	0	0	Summer
6/25/2019	BL	12M	6	2019	Year-2	0	0	Summer
6/25/2019	BL	14M	6	2019	Year-2	0	0	Summer
6/25/2019	BL	8A	6	2019	Year-2	0	0	Summer
6/25/2019	BL	9A	6	2019	Year-2	0	0	Summer
6/25/2019	BL	11A	6	2019	Year-2	0	0	Summer
6/25/2019	BL	13A	6	2019	Year-2	0	0	Summer
6/26/2019	BL	1M	6	2019	Year-2	0	0	Summer
6/26/2019	BL	2M	6	2019	Year-2	0	0	Summer
6/26/2019	BL	3M	6	2019	Year-2	0	0	Summer
6/26/2019	BL	4M	6	2019	Year-2	0	0	Summer
6/26/2019	BL	5M	6	2019	Year-2	0	0	Summer
6/26/2019	BL	6M	6	2019	Year-2		1	Summer
6/26/2019	BL	7M	6	2019	Year-2	0	0	Summer
6/26/2019	BL	10M	6	2019	Year-2	0	0	Summer
6/26/2019	BL	12M	6	2019	Year-2	0	0	Summer

Date	Species	Site	Month	Year	Survey Year	Presence	Data Gap	Season
6/26/2019	BL	14M	6	2019	Year-2	0	0	Summer
6/26/2019	BL	8A	6	2019	Year-2	0	0	Summer
6/26/2019	BL	9A	6	2019	Year-2	0	0	Summer
6/26/2019	BL	11A	6	2019	Year-2	0	0	Summer
6/26/2019	BL	13A	6	2019	Year-2	0	0	Summer
6/27/2019	BL	1M	6	2019	Year-2	0	0	Summer
6/27/2019	BL	2M	6	2019	Year-2	0	0	Summer
6/27/2019	BL	3M	6	2019	Year-2	0	0	Summer
6/27/2019	BL	4M	6	2019	Year-2	0	0	Summer
6/27/2019	BL	5M	6	2019	Year-2	0	0	Summer
6/27/2019	BL	6M	6	2019	Year-2		1	Summer
6/27/2019	BL	7M	6	2019	Year-2	0	0	Summer
6/27/2019	BL	10M	6	2019	Year-2	0	0	Summer
6/27/2019	BL	12M	6	2019	Year-2	0	0	Summer
6/27/2019	BL	14M	6	2019	Year-2	0	0	Summer
6/27/2019	BL	8A	6	2019	Year-2	0	0	Summer
6/27/2019	BL	9A	6	2019	Year-2	0	0	Summer
6/27/2019	BL	11A	6	2019	Year-2	0	0	Summer
6/27/2019	BL	13A	6	2019	Year-2	0	0	Summer
6/28/2019	BL	1M	6	2019	Year-2	0	0	Summer
6/28/2019	BL	2M	6	2019	Year-2	0	0	Summer
6/28/2019	BL	3M	6	2019	Year-2	0	0	Summer
6/28/2019	BL	4M	6	2019	Year-2	0	0	Summer
6/28/2019	BL	5M	6	2019	Year-2	0	0	Summer
6/28/2019	BL	6M	6	2019	Year-2		1	Summer
6/28/2019	BL	7M	6	2019	Year-2	0	0	Summer
6/28/2019	BL	10M	6	2019	Year-2	0	0	Summer
6/28/2019	BL	12M	6	2019	Year-2	0	0	Summer
6/28/2019	BL	14M	6	2019	Year-2	0	0	Summer
6/28/2019	BL	8A	6	2019	Year-2	0	0	Summer
6/28/2019	BL	9A	6	2019	Year-2	0	0	Summer
6/28/2019	BL	11A	6	2019	Year-2	0	0	Summer
6/28/2019	BL	13A	6	2019	Year-2	0	0	Summer
6/29/2019	BL	1M	6	2019	Year-2	0	0	Summer
6/29/2019	BL	2M	6	2019	Year-2	0	0	Summer
6/29/2019	BL	3M	6	2019	Year-2	0	0	Summer
6/29/2019	BL	4M	6	2019	Year-2	0	0	Summer
6/29/2019	BL	5M	6	2019	Year-2	0	0	Summer
6/29/2019	BL	6M	6	2019	Year-2		1	Summer
6/29/2019	BL	7M	6	2019	Year-2	0	0	Summer
6/29/2019	BL	10M	6	2019	Year-2	0	0	Summer
6/29/2019	BL	12M	6	2019	Year-2	0	0	Summer
6/29/2019	BL	14M	6	2019	Year-2	0	0	Summer
6/29/2019	BL	8A	6	2019	Year-2	0	0	Summer

Date	Species	Site	Month	Year	Survey Year	Presence	Data Gap	Season
6/29/2019	BL	9A	6	2019	Year-2	0	0	Summer
6/29/2019	BL	11A	6	2019	Year-2	0	0	Summer
6/29/2019	BL	13A	6	2019	Year-2	0	0	Summer
6/30/2019	BL	1M	6	2019	Year-2	0	0	Summer
6/30/2019	BL	2M	6	2019	Year-2	0	0	Summer
6/30/2019	BL	3M	6	2019	Year-2	0	0	Summer
6/30/2019	BL	4M	6	2019	Year-2	0	0	Summer
6/30/2019	BL	5M	6	2019	Year-2	0	0	Summer
6/30/2019	BL	6M	6	2019	Year-2		1	Summer
6/30/2019	BL	7M	6	2019	Year-2	0	0	Summer
6/30/2019	BL	10M	6	2019	Year-2	0	0	Summer
6/30/2019	BL	12M	6	2019	Year-2	0	0	Summer
6/30/2019	BL	14M	6	2019	Year-2	0	0	Summer
6/30/2019	BL	8A	6	2019	Year-2	0	0	Summer
6/30/2019	BL	9A	6	2019	Year-2	0	0	Summer
6/30/2019	BL	11A	6	2019	Year-2	0	0	Summer
6/30/2019	BL	13A	6	2019	Year-2	0	0	Summer
7/1/2019	BL	1M	7	2019	Year-2	0	0	Summer
7/1/2019	BL	2M	7	2019	Year-2	0	0	Summer
7/1/2019	BL	3M	7	2019	Year-2	0	0	Summer
7/1/2019	BL	4M	7	2019	Year-2	0	0	Summer
7/1/2019	BL	5M	7	2019	Year-2	0	0	Summer
7/1/2019	BL	6M	7	2019	Year-2		1	Summer
7/1/2019	BL	7M	7	2019	Year-2	0	0	Summer
7/1/2019	BL	10M	7	2019	Year-2	0	0	Summer
7/1/2019	BL	12M	7	2019	Year-2	0	0	Summer
7/1/2019	BL	14M	7	2019	Year-2	0	0	Summer
7/1/2019	BL	8A	7	2019	Year-2	0	0	Summer
7/1/2019	BL	9A	7	2019	Year-2	0	0	Summer
7/1/2019	BL	11A	7	2019	Year-2	0	0	Summer
7/1/2019	BL	13A	7	2019	Year-2	0	0	Summer
7/2/2019	BL	1M	7	2019	Year-2	0	0	Summer
7/2/2019	BL	2M	7	2019	Year-2	0	0	Summer
7/2/2019	BL	3M	7	2019	Year-2	0	0	Summer
7/2/2019	BL	4M	7	2019	Year-2	0	0	Summer
7/2/2019	BL	5M	7	2019	Year-2	0	0	Summer
7/2/2019	BL	6M	7	2019	Year-2		1	Summer
7/2/2019	BL	7M	7	2019	Year-2	0	0	Summer
7/2/2019	BL	10M	7	2019	Year-2	0	0	Summer
7/2/2019	BL	12M	7	2019	Year-2	0	0	Summer
7/2/2019	BL	14M	7	2019	Year-2	0	0	Summer
7/2/2019	BL	8A	7	2019	Year-2	0	0	Summer
7/2/2019	BL	9A	7	2019	Year-2	0	0	Summer
7/2/2019	BL	11A	7	2019	Year-2	0	0	Summer

Date	Species	Site	Month	Year	Survey Year	Presence	Data Gap	Season
7/2/2019	BL	13A	7	2019	Year-2	0	0	Summer
7/3/2019	BL	1M	7	2019	Year-2	0	0	Summer
7/3/2019	BL	2M	7	2019	Year-2	0	0	Summer
7/3/2019	BL	3M	7	2019	Year-2	0	0	Summer
7/3/2019	BL	4M	7	2019	Year-2	0	0	Summer
7/3/2019	BL	5M	7	2019	Year-2	0	0	Summer
7/3/2019	BL	6M	7	2019	Year-2		1	Summer
7/3/2019	BL	7M	7	2019	Year-2	0	0	Summer
7/3/2019	BL	10M	7	2019	Year-2	0	0	Summer
7/3/2019	BL	12M	7	2019	Year-2	0	0	Summer
7/3/2019	BL	14M	7	2019	Year-2	0	0	Summer
7/3/2019	BL	8A	7	2019	Year-2	0	0	Summer
7/3/2019	BL	9A	7	2019	Year-2	0	0	Summer
7/3/2019	BL	11A	7	2019	Year-2	0	0	Summer
7/3/2019	BL	13A	7	2019	Year-2	0	0	Summer
7/4/2019	BL	1M	7	2019	Year-2	0	0	Summer
7/4/2019	BL	2M	7	2019	Year-2	0	0	Summer
7/4/2019	BL	3M	7	2019	Year-2	0	0	Summer
7/4/2019	BL	4M	7	2019	Year-2	0	0	Summer
7/4/2019	BL	5M	7	2019	Year-2	0	0	Summer
7/4/2019	BL	6M	7	2019	Year-2		1	Summer
7/4/2019	BL	7M	7	2019	Year-2	0	0	Summer
7/4/2019	BL	10M	7	2019	Year-2	0	0	Summer
7/4/2019	BL	12M	7	2019	Year-2	0	0	Summer
7/4/2019	BL	14M	7	2019	Year-2	0	0	Summer
7/4/2019	BL	8A	7	2019	Year-2	0	0	Summer
7/4/2019	BL	9A	7	2019	Year-2	0	0	Summer
7/4/2019	BL	11A	7	2019	Year-2	0	0	Summer
7/4/2019	BL	13A	7	2019	Year-2	0	0	Summer
7/5/2019	BL	1M	7	2019	Year-2	0	0	Summer
7/5/2019	BL	2M	7	2019	Year-2	0	0	Summer
7/5/2019	BL	3M	7	2019	Year-2	0	0	Summer
7/5/2019	BL	4M	7	2019	Year-2	0	0	Summer
7/5/2019	BL	5M	7	2019	Year-2	0	0	Summer
7/5/2019	BL	6M	7	2019	Year-2		1	Summer
7/5/2019	BL	7M	7	2019	Year-2	0	0	Summer
7/5/2019	BL	10M	7	2019	Year-2	0	0	Summer
7/5/2019	BL	12M	7	2019	Year-2	0	0	Summer
7/5/2019	BL	14M	7	2019	Year-2	0	0	Summer
7/5/2019	BL	8A	7	2019	Year-2	0	0	Summer
7/5/2019	BL	9A	7	2019	Year-2	0	0	Summer
7/5/2019	BL	11A	7	2019	Year-2	0	0	Summer
7/5/2019	BL	13A	7	2019	Year-2	0	0	Summer
7/6/2019	BL	1M	7	2019	Year-2	0	0	Summer

Date	Species	Site	Month	Year	Survey Year	Presence	Data Gap	Season
7/6/2019	BL	2M	7	2019	Year-2	0	0	Summer
7/6/2019	BL	3M	7	2019	Year-2	0	0	Summer
7/6/2019	BL	4M	7	2019	Year-2	0	0	Summer
7/6/2019	BL	5M	7	2019	Year-2	0	0	Summer
7/6/2019	BL	6M	7	2019	Year-2		1	Summer
7/6/2019	BL	7M	7	2019	Year-2	0	0	Summer
7/6/2019	BL	10M	7	2019	Year-2	0	0	Summer
7/6/2019	BL	12M	7	2019	Year-2	0	0	Summer
7/6/2019	BL	14M	7	2019	Year-2	0	0	Summer
7/6/2019	BL	8A	7	2019	Year-2	0	0	Summer
7/6/2019	BL	9A	7	2019	Year-2	0	0	Summer
7/6/2019	BL	11A	7	2019	Year-2	0	0	Summer
7/6/2019	BL	13A	7	2019	Year-2	0	0	Summer
7/7/2019	BL	1M	7	2019	Year-2	0	0	Summer
7/7/2019	BL	2M	7	2019	Year-2	0	0	Summer
7/7/2019	BL	3M	7	2019	Year-2	0	0	Summer
7/7/2019	BL	4M	7	2019	Year-2	0	0	Summer
7/7/2019	BL	5M	7	2019	Year-2	0	0	Summer
7/7/2019	BL	6M	7	2019	Year-2		1	Summer
7/7/2019	BL	7M	7	2019	Year-2	0	0	Summer
7/7/2019	BL	10M	7	2019	Year-2	0	0	Summer
7/7/2019	BL	12M	7	2019	Year-2	0	0	Summer
7/7/2019	BL	14M	7	2019	Year-2	0	0	Summer
7/7/2019	BL	8A	7	2019	Year-2	0	0	Summer
7/7/2019	BL	9A	7	2019	Year-2	0	0	Summer
7/7/2019	BL	11A	7	2019	Year-2	0	0	Summer
7/7/2019	BL	13A	7	2019	Year-2	0	0	Summer
7/8/2019	BL	1M	7	2019	Year-2	0	0	Summer
7/8/2019	BL	2M	7	2019	Year-2	0	0	Summer
7/8/2019	BL	3M	7	2019	Year-2	0	0	Summer
7/8/2019	BL	4M	7	2019	Year-2	0	0	Summer
7/8/2019	BL	5M	7	2019	Year-2	0	0	Summer
7/8/2019	BL	6M	7	2019	Year-2		1	Summer
7/8/2019	BL	7M	7	2019	Year-2	0	0	Summer
7/8/2019	BL	10M	7	2019	Year-2	0	0	Summer
7/8/2019	BL	12M	7	2019	Year-2	0	0	Summer
7/8/2019	BL	14M	7	2019	Year-2	0	0	Summer
7/8/2019	BL	8A	7	2019	Year-2	0	0	Summer
7/8/2019	BL	9A	7	2019	Year-2	0	0	Summer
7/8/2019	BL	11A	7	2019	Year-2	0	0	Summer
7/8/2019	BL	13A	7	2019	Year-2	0	0	Summer
7/9/2019	BL	1M	7	2019	Year-2	0	0	Summer
7/9/2019	BL	2M	7	2019	Year-2	0	0	Summer
7/9/2019	BL	3M	7	2019	Year-2	0	0	Summer

Date	Species	Site	Month	Year	Survey Year	Presence	Data Gap	Season
7/9/2019	BL	4M	7	2019	Year-2	0	0	Summer
7/9/2019	BL	5M	7	2019	Year-2	0	0	Summer
7/9/2019	BL	6M	7	2019	Year-2		1	Summer
7/9/2019	BL	7M	7	2019	Year-2	0	0	Summer
7/9/2019	BL	10M	7	2019	Year-2	0	0	Summer
7/9/2019	BL	12M	7	2019	Year-2	0	0	Summer
7/9/2019	BL	14M	7	2019	Year-2	0	0	Summer
7/9/2019	BL	8A	7	2019	Year-2	0	0	Summer
7/9/2019	BL	9A	7	2019	Year-2	0	0	Summer
7/9/2019	BL	11A	7	2019	Year-2	0	0	Summer
7/9/2019	BL	13A	7	2019	Year-2	0	0	Summer
7/10/2019	BL	1M	7	2019	Year-2	0	0	Summer
7/10/2019	BL	2M	7	2019	Year-2	0	0	Summer
7/10/2019	BL	3M	7	2019	Year-2	0	0	Summer
7/10/2019	BL	4M	7	2019	Year-2	0	0	Summer
7/10/2019	BL	5M	7	2019	Year-2	0	0	Summer
7/10/2019	BL	6M	7	2019	Year-2		1	Summer
7/10/2019	BL	7M	7	2019	Year-2	0	0	Summer
7/10/2019	BL	10M	7	2019	Year-2	0	0	Summer
7/10/2019	BL	12M	7	2019	Year-2	0	0	Summer
7/10/2019	BL	14M	7	2019	Year-2	0	0	Summer
7/10/2019	BL	8A	7	2019	Year-2	0	0	Summer
7/10/2019	BL	9A	7	2019	Year-2	0	0	Summer
7/10/2019	BL	11A	7	2019	Year-2	0	0	Summer
7/10/2019	BL	13A	7	2019	Year-2	0	0	Summer
7/11/2019	BL	1M	7	2019	Year-2	0	0	Summer
7/11/2019	BL	2M	7	2019	Year-2	0	0	Summer
7/11/2019	BL	3M	7	2019	Year-2	0	0	Summer
7/11/2019	BL	4M	7	2019	Year-2	0	0	Summer
7/11/2019	BL	5M	7	2019	Year-2	0	0	Summer
7/11/2019	BL	6M	7	2019	Year-2		1	Summer
7/11/2019	BL	7M	7	2019	Year-2	0	0	Summer
7/11/2019	BL	10M	7	2019	Year-2	0	0	Summer
7/11/2019	BL	12M	7	2019	Year-2	0	0	Summer
7/11/2019	BL	14M	7	2019	Year-2	0	0	Summer
7/11/2019	BL	8A	7	2019	Year-2	0	0	Summer
7/11/2019	BL	9A	7	2019	Year-2	0	0	Summer
7/11/2019	BL	11A	7	2019	Year-2	0	0	Summer
7/11/2019	BL	13A	7	2019	Year-2	0	0	Summer
7/12/2019	BL	1M	7	2019	Year-2	0	0	Summer
7/12/2019	BL	2M	7	2019	Year-2	0	0	Summer
7/12/2019	BL	3M	7	2019	Year-2	0	0	Summer
7/12/2019	BL	4M	7	2019	Year-2	0	0	Summer
7/12/2019	BL	5M	7	2019	Year-2	0	0	Summer

Date	Species	Site	Month	Year	Survey Year	Presence	Data Gap	Season
7/12/2019	BL	6M	7	2019	Year-2		1	Summer
7/12/2019	BL	7M	7	2019	Year-2	0	0	Summer
7/12/2019	BL	10M	7	2019	Year-2	0	0	Summer
7/12/2019	BL	12M	7	2019	Year-2	0	0	Summer
7/12/2019	BL	14M	7	2019	Year-2	0	0	Summer
7/12/2019	BL	8A	7	2019	Year-2	0	0	Summer
7/12/2019	BL	9A	7	2019	Year-2	0	0	Summer
7/12/2019	BL	11A	7	2019	Year-2	0	0	Summer
7/12/2019	BL	13A	7	2019	Year-2	0	0	Summer
7/13/2019	BL	1M	7	2019	Year-2	0	0	Summer
7/13/2019	BL	2M	7	2019	Year-2	0	0	Summer
7/13/2019	BL	3M	7	2019	Year-2	0	0	Summer
7/13/2019	BL	4M	7	2019	Year-2	0	0	Summer
7/13/2019	BL	5M	7	2019	Year-2	0	0	Summer
7/13/2019	BL	6M	7	2019	Year-2		1	Summer
7/13/2019	BL	7M	7	2019	Year-2	0	0	Summer
7/13/2019	BL	10M	7	2019	Year-2	0	0	Summer
7/13/2019	BL	12M	7	2019	Year-2	0	0	Summer
7/13/2019	BL	14M	7	2019	Year-2	0	0	Summer
7/13/2019	BL	8A	7	2019	Year-2	0	0	Summer
7/13/2019	BL	9A	7	2019	Year-2	0	0	Summer
7/13/2019	BL	11A	7	2019	Year-2	0	0	Summer
7/13/2019	BL	13A	7	2019	Year-2	0	0	Summer
7/14/2019	BL	1M	7	2019	Year-2	0	0	Summer
7/14/2019	BL	2M	7	2019	Year-2	0	0	Summer
7/14/2019	BL	3M	7	2019	Year-2	0	0	Summer
7/14/2019	BL	4M	7	2019	Year-2	0	0	Summer
7/14/2019	BL	5M	7	2019	Year-2	0	0	Summer
7/14/2019	BL	6M	7	2019	Year-2		1	Summer
7/14/2019	BL	7M	7	2019	Year-2	0	0	Summer
7/14/2019	BL	10M	7	2019	Year-2	0	0	Summer
7/14/2019	BL	12M	7	2019	Year-2	0	0	Summer
7/14/2019	BL	14M	7	2019	Year-2	0	0	Summer
7/14/2019	BL	8A	7	2019	Year-2	0	0	Summer
7/14/2019	BL	9A	7	2019	Year-2	0	0	Summer
7/14/2019	BL	11A	7	2019	Year-2	0	0	Summer
7/14/2019	BL	13A	7	2019	Year-2	0	0	Summer
7/15/2019	BL	1M	7	2019	Year-2	0	0	Summer
7/15/2019	BL	2M	7	2019	Year-2	0	0	Summer
7/15/2019	BL	3M	7	2019	Year-2	0	0	Summer
7/15/2019	BL	4M	7	2019	Year-2	0	0	Summer
7/15/2019	BL	5M	7	2019	Year-2	0	0	Summer
7/15/2019	BL	6M	7	2019	Year-2		1	Summer
7/15/2019	BL	7M	7	2019	Year-2	0	0	Summer

Date	Species	Site	Month	Year	Survey Year	Presence	Data Gap	Season
7/15/2019	BL	10M	7	2019	Year-2	0	0	Summer
7/15/2019	BL	12M	7	2019	Year-2	0	0	Summer
7/15/2019	BL	14M	7	2019	Year-2	0	0	Summer
7/15/2019	BL	8A	7	2019	Year-2	0	0	Summer
7/15/2019	BL	9A	7	2019	Year-2	0	0	Summer
7/15/2019	BL	11A	7	2019	Year-2	0	0	Summer
7/15/2019	BL	13A	7	2019	Year-2	0	0	Summer
7/16/2019	BL	1M	7	2019	Year-2	0	0	Summer
7/16/2019	BL	2M	7	2019	Year-2	0	0	Summer
7/16/2019	BL	3M	7	2019	Year-2	0	0	Summer
7/16/2019	BL	4M	7	2019	Year-2	0	0	Summer
7/16/2019	BL	5M	7	2019	Year-2	0	0	Summer
7/16/2019	BL	6M	7	2019	Year-2		1	Summer
7/16/2019	BL	7M	7	2019	Year-2	0	0	Summer
7/16/2019	BL	10M	7	2019	Year-2	0	0	Summer
7/16/2019	BL	12M	7	2019	Year-2	0	0	Summer
7/16/2019	BL	14M	7	2019	Year-2	0	0	Summer
7/16/2019	BL	8A	7	2019	Year-2	0	0	Summer
7/16/2019	BL	9A	7	2019	Year-2	0	0	Summer
7/16/2019	BL	11A	7	2019	Year-2	0	0	Summer
7/16/2019	BL	13A	7	2019	Year-2	0	0	Summer
7/17/2019	BL	1M	7	2019	Year-2	0	0	Summer
7/17/2019	BL	2M	7	2019	Year-2	0	0	Summer
7/17/2019	BL	3M	7	2019	Year-2	0	0	Summer
7/17/2019	BL	4M	7	2019	Year-2	0	0	Summer
7/17/2019	BL	5M	7	2019	Year-2	0	0	Summer
7/17/2019	BL	6M	7	2019	Year-2		1	Summer
7/17/2019	BL	7M	7	2019	Year-2	0	0	Summer
7/17/2019	BL	10M	7	2019	Year-2	0	0	Summer
7/17/2019	BL	12M	7	2019	Year-2	0	0	Summer
7/17/2019	BL	14M	7	2019	Year-2	0	0	Summer
7/17/2019	BL	8A	7	2019	Year-2	0	0	Summer
7/17/2019	BL	9A	7	2019	Year-2	0	0	Summer
7/17/2019	BL	11A	7	2019	Year-2	0	0	Summer
7/17/2019	BL	13A	7	2019	Year-2	0	0	Summer
7/18/2019	BL	1M	7	2019	Year-2	0	0	Summer
7/18/2019	BL	2M	7	2019	Year-2	0	0	Summer
7/18/2019	BL	3M	7	2019	Year-2	0	0	Summer
7/18/2019	BL	4M	7	2019	Year-2	0	0	Summer
7/18/2019	BL	5M	7	2019	Year-2	0	0	Summer
7/18/2019	BL	6M	7	2019	Year-2		1	Summer
7/18/2019	BL	7M	7	2019	Year-2	0	0	Summer
7/18/2019	BL	10M	7	2019	Year-2	0	0	Summer
7/18/2019	BL	12M	7	2019	Year-2	0	0	Summer

Date	Species	Site	Month	Year	Survey Year	Presence	Data Gap	Season
7/18/2019	BL	14M	7	2019	Year-2	0	0	Summer
7/18/2019	BL	8A	7	2019	Year-2	0	0	Summer
7/18/2019	BL	9A	7	2019	Year-2	0	0	Summer
7/18/2019	BL	11A	7	2019	Year-2	0	0	Summer
7/18/2019	BL	13A	7	2019	Year-2	0	0	Summer
7/19/2019	BL	1M	7	2019	Year-2	0	0	Summer
7/19/2019	BL	2M	7	2019	Year-2	0	0	Summer
7/19/2019	BL	3M	7	2019	Year-2	0	0	Summer
7/19/2019	BL	4M	7	2019	Year-2	0	0	Summer
7/19/2019	BL	5M	7	2019	Year-2	0	0	Summer
7/19/2019	BL	6M	7	2019	Year-2		1	Summer
7/19/2019	BL	7M	7	2019	Year-2	0	0	Summer
7/19/2019	BL	10M	7	2019	Year-2	0	0	Summer
7/19/2019	BL	12M	7	2019	Year-2	0	0	Summer
7/19/2019	BL	14M	7	2019	Year-2	0	0	Summer
7/19/2019	BL	8A	7	2019	Year-2	0	0	Summer
7/19/2019	BL	9A	7	2019	Year-2	0	0	Summer
7/19/2019	BL	11A	7	2019	Year-2	0	0	Summer
7/19/2019	BL	13A	7	2019	Year-2	0	0	Summer
7/20/2019	BL	1M	7	2019	Year-2	0	0	Summer
7/20/2019	BL	2M	7	2019	Year-2	0	0	Summer
7/20/2019	BL	3M	7	2019	Year-2	0	0	Summer
7/20/2019	BL	4M	7	2019	Year-2	0	0	Summer
7/20/2019	BL	5M	7	2019	Year-2	0	0	Summer
7/20/2019	BL	6M	7	2019	Year-2		1	Summer
7/20/2019	BL	7M	7	2019	Year-2	0	0	Summer
7/20/2019	BL	10M	7	2019	Year-2	0	0	Summer
7/20/2019	BL	12M	7	2019	Year-2	0	0	Summer
7/20/2019	BL	14M	7	2019	Year-2	0	0	Summer
7/20/2019	BL	8A	7	2019	Year-2	0	0	Summer
7/20/2019	BL	9A	7	2019	Year-2	0	0	Summer
7/20/2019	BL	11A	7	2019	Year-2	0	0	Summer
7/20/2019	BL	13A	7	2019	Year-2	0	0	Summer
7/21/2019	BL	1M	7	2019	Year-2	0	0	Summer
7/21/2019	BL	2M	7	2019	Year-2	0	0	Summer
7/21/2019	BL	3M	7	2019	Year-2	0	0	Summer
7/21/2019	BL	4M	7	2019	Year-2	0	0	Summer
7/21/2019	BL	5M	7	2019	Year-2	0	0	Summer
7/21/2019	BL	6M	7	2019	Year-2		1	Summer
7/21/2019	BL	7M	7	2019	Year-2	0	0	Summer
7/21/2019	BL	10M	7	2019	Year-2	0	0	Summer
7/21/2019	BL	12M	7	2019	Year-2	0	0	Summer
7/21/2019	BL	14M	7	2019	Year-2	0	0	Summer
7/21/2019	BL	8A	7	2019	Year-2	0	0	Summer

Date	Species	Site	Month	Year	Survey Year	Presence	Data Gap	Season
7/21/2019	BL	9A	7	2019	Year-2	0	0	Summer
7/21/2019	BL	11A	7	2019	Year-2	0	0	Summer
7/21/2019	BL	13A	7	2019	Year-2	0	0	Summer
7/22/2019	BL	1M	7	2019	Year-2	0	0	Summer
7/22/2019	BL	2M	7	2019	Year-2	0	0	Summer
7/22/2019	BL	3M	7	2019	Year-2	0	0	Summer
7/22/2019	BL	4M	7	2019	Year-2	0	0	Summer
7/22/2019	BL	5M	7	2019	Year-2	0	0	Summer
7/22/2019	BL	6M	7	2019	Year-2		1	Summer
7/22/2019	BL	7M	7	2019	Year-2	0	0	Summer
7/22/2019	BL	10M	7	2019	Year-2	0	0	Summer
7/22/2019	BL	12M	7	2019	Year-2	0	0	Summer
7/22/2019	BL	14M	7	2019	Year-2	0	0	Summer
7/22/2019	BL	8A	7	2019	Year-2	0	0	Summer
7/22/2019	BL	9A	7	2019	Year-2	0	0	Summer
7/22/2019	BL	11A	7	2019	Year-2	0	0	Summer
7/22/2019	BL	13A	7	2019	Year-2	0	0	Summer
7/23/2019	BL	1M	7	2019	Year-2	0	0	Summer
7/23/2019	BL	2M	7	2019	Year-2	0	0	Summer
7/23/2019	BL	3M	7	2019	Year-2	0	0	Summer
7/23/2019	BL	4M	7	2019	Year-2	0	0	Summer
7/23/2019	BL	5M	7	2019	Year-2	0	0	Summer
7/23/2019	BL	6M	7	2019	Year-2		1	Summer
7/23/2019	BL	7M	7	2019	Year-2	0	0	Summer
7/23/2019	BL	10M	7	2019	Year-2	0	0	Summer
7/23/2019	BL	12M	7	2019	Year-2	0	0	Summer
7/23/2019	BL	14M	7	2019	Year-2	0	0	Summer
7/23/2019	BL	8A	7	2019	Year-2	0	0	Summer
7/23/2019	BL	9A	7	2019	Year-2	0	0	Summer
7/23/2019	BL	11A	7	2019	Year-2	0	0	Summer
7/23/2019	BL	13A	7	2019	Year-2	0	0	Summer
7/24/2019	BL	1M	7	2019	Year-2	0	0	Summer
7/24/2019	BL	2M	7	2019	Year-2	0	0	Summer
7/24/2019	BL	3M	7	2019	Year-2	0	0	Summer
7/24/2019	BL	4M	7	2019	Year-2	0	0	Summer
7/24/2019	BL	5M	7	2019	Year-2	0	0	Summer
7/24/2019	BL	6M	7	2019	Year-2		1	Summer
7/24/2019	BL	7M	7	2019	Year-2	0	0	Summer
7/24/2019	BL	10M	7	2019	Year-2	0	0	Summer
7/24/2019	BL	12M	7	2019	Year-2	0	0	Summer
7/24/2019	BL	14M	7	2019	Year-2	0	0	Summer
7/24/2019	BL	8A	7	2019	Year-2	0	0	Summer
7/24/2019	BL	9A	7	2019	Year-2	0	0	Summer
7/24/2019	BL	11A	7	2019	Year-2	0	0	Summer

Date	Species	Site	Month	Year	Survey Year	Presence	Data Gap	Season
7/24/2019	BL	13A	7	2019	Year-2	0	0	Summer
7/25/2019	BL	1M	7	2019	Year-2	0	0	Summer
7/25/2019	BL	2M	7	2019	Year-2	0	0	Summer
7/25/2019	BL	3M	7	2019	Year-2	0	0	Summer
7/25/2019	BL	4M	7	2019	Year-2	0	0	Summer
7/25/2019	BL	5M	7	2019	Year-2	0	0	Summer
7/25/2019	BL	6M	7	2019	Year-2		1	Summer
7/25/2019	BL	7M	7	2019	Year-2	0	0	Summer
7/25/2019	BL	10M	7	2019	Year-2	0	0	Summer
7/25/2019	BL	12M	7	2019	Year-2	0	0	Summer
7/25/2019	BL	14M	7	2019	Year-2	0	0	Summer
7/25/2019	BL	8A	7	2019	Year-2	0	0	Summer
7/25/2019	BL	9A	7	2019	Year-2	0	0	Summer
7/25/2019	BL	11A	7	2019	Year-2	0	0	Summer
7/25/2019	BL	13A	7	2019	Year-2	0	0	Summer
7/26/2019	BL	1M	7	2019	Year-2	0	0	Summer
7/26/2019	BL	2M	7	2019	Year-2	0	0	Summer
7/26/2019	BL	3M	7	2019	Year-2	0	0	Summer
7/26/2019	BL	4M	7	2019	Year-2	0	0	Summer
7/26/2019	BL	5M	7	2019	Year-2	0	0	Summer
7/26/2019	BL	6M	7	2019	Year-2		1	Summer
7/26/2019	BL	7M	7	2019	Year-2	0	0	Summer
7/26/2019	BL	10M	7	2019	Year-2	0	0	Summer
7/26/2019	BL	12M	7	2019	Year-2	0	0	Summer
7/26/2019	BL	14M	7	2019	Year-2	0	0	Summer
7/26/2019	BL	8A	7	2019	Year-2	0	0	Summer
7/26/2019	BL	9A	7	2019	Year-2	0	0	Summer
7/26/2019	BL	11A	7	2019	Year-2	0	0	Summer
7/26/2019	BL	13A	7	2019	Year-2	0	0	Summer
7/27/2019	BL	1M	7	2019	Year-2	0	0	Summer
7/27/2019	BL	2M	7	2019	Year-2	0	0	Summer
7/27/2019	BL	3M	7	2019	Year-2	0	0	Summer
7/27/2019	BL	4M	7	2019	Year-2	0	0	Summer
7/27/2019	BL	5M	7	2019	Year-2	0	0	Summer
7/27/2019	BL	6M	7	2019	Year-2		1	Summer
7/27/2019	BL	7M	7	2019	Year-2	0	0	Summer
7/27/2019	BL	10M	7	2019	Year-2	0	0	Summer
7/27/2019	BL	12M	7	2019	Year-2	0	0	Summer
7/27/2019	BL	14M	7	2019	Year-2	0	0	Summer
7/27/2019	BL	8A	7	2019	Year-2	0	0	Summer
7/27/2019	BL	9A	7	2019	Year-2	0	0	Summer
7/27/2019	BL	11A	7	2019	Year-2	0	0	Summer
7/27/2019	BL	13A	7	2019	Year-2	0	0	Summer
7/28/2019	BL	1M	7	2019	Year-2	0	0	Summer

Date	Species	Site	Month	Year	Survey Year	Presence	Data Gap	Season
7/28/2019	BL	2M	7	2019	Year-2	0	0	Summer
7/28/2019	BL	3M	7	2019	Year-2	0	0	Summer
7/28/2019	BL	4M	7	2019	Year-2	0	0	Summer
7/28/2019	BL	5M	7	2019	Year-2	0	0	Summer
7/28/2019	BL	6M	7	2019	Year-2		1	Summer
7/28/2019	BL	7M	7	2019	Year-2	0	0	Summer
7/28/2019	BL	10M	7	2019	Year-2	0	0	Summer
7/28/2019	BL	12M	7	2019	Year-2	0	0	Summer
7/28/2019	BL	14M	7	2019	Year-2	0	0	Summer
7/28/2019	BL	8A	7	2019	Year-2	0	0	Summer
7/28/2019	BL	9A	7	2019	Year-2	0	0	Summer
7/28/2019	BL	11A	7	2019	Year-2	0	0	Summer
7/28/2019	BL	13A	7	2019	Year-2	0	0	Summer
7/29/2019	BL	1M	7	2019	Year-2	0	0	Summer
7/29/2019	BL	2M	7	2019	Year-2	0	0	Summer
7/29/2019	BL	3M	7	2019	Year-2	0	0	Summer
7/29/2019	BL	4M	7	2019	Year-2	0	0	Summer
7/29/2019	BL	5M	7	2019	Year-2	0	0	Summer
7/29/2019	BL	6M	7	2019	Year-2		1	Summer
7/29/2019	BL	7M	7	2019	Year-2	0	0	Summer
7/29/2019	BL	10M	7	2019	Year-2	0	0	Summer
7/29/2019	BL	12M	7	2019	Year-2	0	0	Summer
7/29/2019	BL	14M	7	2019	Year-2	0	0	Summer
7/29/2019	BL	8A	7	2019	Year-2	0	0	Summer
7/29/2019	BL	9A	7	2019	Year-2	0	0	Summer
7/29/2019	BL	11A	7	2019	Year-2	0	0	Summer
7/29/2019	BL	13A	7	2019	Year-2	0	0	Summer
7/30/2019	BL	1M	7	2019	Year-2	0	0	Summer
7/30/2019	BL	2M	7	2019	Year-2	0	0	Summer
7/30/2019	BL	3M	7	2019	Year-2	0	0	Summer
7/30/2019	BL	4M	7	2019	Year-2	0	0	Summer
7/30/2019	BL	5M	7	2019	Year-2	0	0	Summer
7/30/2019	BL	6M	7	2019	Year-2		1	Summer
7/30/2019	BL	7M	7	2019	Year-2	0	0	Summer
7/30/2019	BL	10M	7	2019	Year-2	0	0	Summer
7/30/2019	BL	12M	7	2019	Year-2	0	0	Summer
7/30/2019	BL	14M	7	2019	Year-2	0	0	Summer
7/30/2019	BL	8A	7	2019	Year-2	0	0	Summer
7/30/2019	BL	9A	7	2019	Year-2	0	0	Summer
7/30/2019	BL	11A	7	2019	Year-2	0	0	Summer
7/30/2019	BL	13A	7	2019	Year-2	0	0	Summer
7/31/2019	BL	1M	7	2019	Year-2	0	0	Summer
7/31/2019	BL	2M	7	2019	Year-2	0	0	Summer
7/31/2019	BL	3M	7	2019	Year-2	0	0	Summer

Date	Species	Site	Month	Year	Survey Year	Presence	Data Gap	Season
7/31/2019	BL	4M	7	2019	Year-2	0	0	Summer
7/31/2019	BL	5M	7	2019	Year-2	0	0	Summer
7/31/2019	BL	6M	7	2019	Year-2		1	Summer
7/31/2019	BL	7M	7	2019	Year-2	0	0	Summer
7/31/2019	BL	10M	7	2019	Year-2	0	0	Summer
7/31/2019	BL	12M	7	2019	Year-2	0	0	Summer
7/31/2019	BL	14M	7	2019	Year-2	0	0	Summer
7/31/2019	BL	8A	7	2019	Year-2	0	0	Summer
7/31/2019	BL	9A	7	2019	Year-2	0	0	Summer
7/31/2019	BL	11A	7	2019	Year-2	0	0	Summer
7/31/2019	BL	13A	7	2019	Year-2	0	0	Summer
8/1/2019	BL	1M	8	2019	Year-2	0	0	Summer
8/1/2019	BL	2M	8	2019	Year-2	0	0	Summer
8/1/2019	BL	3M	8	2019	Year-2	0	0	Summer
8/1/2019	BL	4M	8	2019	Year-2	0	0	Summer
8/1/2019	BL	5M	8	2019	Year-2	0	0	Summer
8/1/2019	BL	6M	8	2019	Year-2		1	Summer
8/1/2019	BL	7M	8	2019	Year-2	0	0	Summer
8/1/2019	BL	10M	8	2019	Year-2	0	0	Summer
8/1/2019	BL	12M	8	2019	Year-2	0	0	Summer
8/1/2019	BL	14M	8	2019	Year-2	0	0	Summer
8/1/2019	BL	8A	8	2019	Year-2	0	0	Summer
8/1/2019	BL	9A	8	2019	Year-2	0	0	Summer
8/1/2019	BL	11A	8	2019	Year-2	0	0	Summer
8/1/2019	BL	13A	8	2019	Year-2	0	0	Summer
8/2/2019	BL	1M	8	2019	Year-2	0	0	Summer
8/2/2019	BL	2M	8	2019	Year-2	0	0	Summer
8/2/2019	BL	3M	8	2019	Year-2	0	0	Summer
8/2/2019	BL	4M	8	2019	Year-2	0	0	Summer
8/2/2019	BL	5M	8	2019	Year-2	0	0	Summer
8/2/2019	BL	6M	8	2019	Year-2		1	Summer
8/2/2019	BL	7M	8	2019	Year-2	0	0	Summer
8/2/2019	BL	10M	8	2019	Year-2		1	Summer
8/2/2019	BL	12M	8	2019	Year-2		1	Summer
8/2/2019	BL	14M	8	2019	Year-2		1	Summer
8/2/2019	BL	8A	8	2019	Year-2	0	0	Summer
8/2/2019	BL	9A	8	2019	Year-2	0	0	Summer
8/2/2019	BL	11A	8	2019	Year-2	0	0	Summer
8/2/2019	BL	13A	8	2019	Year-2	0	0	Summer
8/3/2019	BL	1M	8	2019	Year-2	0	0	Summer
8/3/2019	BL	2M	8	2019	Year-2	0	0	Summer
8/3/2019	BL	3M	8	2019	Year-2	0	0	Summer
8/3/2019	BL	4M	8	2019	Year-2	0	0	Summer
8/3/2019	BL	5M	8	2019	Year-2		1	Summer

Date	Species	Site	Month	Year	Survey Year	Presence	Data Gap	Season
8/3/2019	BL	6M	8	2019	Year-2		1	Summer
8/3/2019	BL	7M	8	2019	Year-2		1	Summer
8/3/2019	BL	10M	8	2019	Year-2	0	0	Summer
8/3/2019	BL	12M	8	2019	Year-2	0	0	Summer
8/3/2019	BL	14M	8	2019	Year-2		1	Summer
8/3/2019	BL	8A	8	2019	Year-2	0	0	Summer
8/3/2019	BL	9A	8	2019	Year-2	0	0	Summer
8/3/2019	BL	11A	8	2019	Year-2	0	0	Summer
8/3/2019	BL	13A	8	2019	Year-2	0	0	Summer
8/4/2019	BL	1M	8	2019	Year-2		1	Summer
8/4/2019	BL	2M	8	2019	Year-2		1	Summer
8/4/2019	BL	3M	8	2019	Year-2		1	Summer
8/4/2019	BL	4M	8	2019	Year-2		1	Summer
8/4/2019	BL	5M	8	2019	Year-2	0	0	Summer
8/4/2019	BL	6M	8	2019	Year-2	0	0	Summer
8/4/2019	BL	7M	8	2019	Year-2	0	0	Summer
8/4/2019	BL	10M	8	2019	Year-2	0	0	Summer
8/4/2019	BL	12M	8	2019	Year-2	0	0	Summer
8/4/2019	BL	14M	8	2019	Year-2		1	Summer
8/4/2019	BL	8A	8	2019	Year-2	0	0	Summer
8/4/2019	BL	9A	8	2019	Year-2	0	0	Summer
8/4/2019	BL	11A	8	2019	Year-2	0	0	Summer
8/4/2019	BL	13A	8	2019	Year-2	0	0	Summer
8/5/2019	BL	1M	8	2019	Year-2	0	0	Summer
8/5/2019	BL	2M	8	2019	Year-2	0	0	Summer
8/5/2019	BL	3M	8	2019	Year-2	0	0	Summer
8/5/2019	BL	4M	8	2019	Year-2	0	0	Summer
8/5/2019	BL	5M	8	2019	Year-2	0	0	Summer
8/5/2019	BL	6M	8	2019	Year-2	0	0	Summer
8/5/2019	BL	7M	8	2019	Year-2	0	0	Summer
8/5/2019	BL	10M	8	2019	Year-2	0	0	Summer
8/5/2019	BL	12M	8	2019	Year-2	0	0	Summer
8/5/2019	BL	14M	8	2019	Year-2		1	Summer
8/5/2019	BL	8A	8	2019	Year-2	0	0	Summer
8/5/2019	BL	9A	8	2019	Year-2	0	0	Summer
8/5/2019	BL	11A	8	2019	Year-2	0	0	Summer
8/5/2019	BL	13A	8	2019	Year-2	0	0	Summer
8/6/2019	BL	1M	8	2019	Year-2	0	0	Summer
8/6/2019	BL	2M	8	2019	Year-2	0	0	Summer
8/6/2019	BL	3M	8	2019	Year-2	0	0	Summer
8/6/2019	BL	4M	8	2019	Year-2	0	0	Summer
8/6/2019	BL	5M	8	2019	Year-2	0	0	Summer
8/6/2019	BL	6M	8	2019	Year-2	0	0	Summer
8/6/2019	BL	7M	8	2019	Year-2	0	0	Summer

Date	Species	Site	Month	Year	Survey Year	Presence	Data Gap	Season
8/6/2019	BL	10M	8	2019	Year-2	0	0	Summer
8/6/2019	BL	12M	8	2019	Year-2	0	0	Summer
8/6/2019	BL	14M	8	2019	Year-2		1	Summer
8/6/2019	BL	8A	8	2019	Year-2	0	0	Summer
8/6/2019	BL	9A	8	2019	Year-2	0	0	Summer
8/6/2019	BL	11A	8	2019	Year-2	0	0	Summer
8/6/2019	BL	13A	8	2019	Year-2	0	0	Summer
8/7/2019	BL	1M	8	2019	Year-2	0	0	Summer
8/7/2019	BL	2M	8	2019	Year-2	0	0	Summer
8/7/2019	BL	3M	8	2019	Year-2	0	0	Summer
8/7/2019	BL	4M	8	2019	Year-2	0	0	Summer
8/7/2019	BL	5M	8	2019	Year-2	0	0	Summer
8/7/2019	BL	6M	8	2019	Year-2	0	0	Summer
8/7/2019	BL	7M	8	2019	Year-2	0	0	Summer
8/7/2019	BL	10M	8	2019	Year-2	0	0	Summer
8/7/2019	BL	12M	8	2019	Year-2	0	0	Summer
8/7/2019	BL	14M	8	2019	Year-2		1	Summer
8/7/2019	BL	8A	8	2019	Year-2	0	0	Summer
8/7/2019	BL	9A	8	2019	Year-2	0	0	Summer
8/7/2019	BL	11A	8	2019	Year-2	0	0	Summer
8/7/2019	BL	13A	8	2019	Year-2	0	0	Summer
8/8/2019	BL	1M	8	2019	Year-2	0	0	Summer
8/8/2019	BL	2M	8	2019	Year-2	0	0	Summer
8/8/2019	BL	3M	8	2019	Year-2	0	0	Summer
8/8/2019	BL	4M	8	2019	Year-2	0	0	Summer
8/8/2019	BL	5M	8	2019	Year-2	0	0	Summer
8/8/2019	BL	6M	8	2019	Year-2	0	0	Summer
8/8/2019	BL	7M	8	2019	Year-2	0	0	Summer
8/8/2019	BL	10M	8	2019	Year-2	0	0	Summer
8/8/2019	BL	12M	8	2019	Year-2	0	0	Summer
8/8/2019	BL	14M	8	2019	Year-2		1	Summer
8/8/2019	BL	8A	8	2019	Year-2	0	0	Summer
8/8/2019	BL	9A	8	2019	Year-2	0	0	Summer
8/8/2019	BL	11A	8	2019	Year-2	0	0	Summer
8/8/2019	BL	13A	8	2019	Year-2	0	0	Summer
8/9/2019	BL	1M	8	2019	Year-2	0	0	Summer
8/9/2019	BL	2M	8	2019	Year-2	0	0	Summer
8/9/2019	BL	3M	8	2019	Year-2	0	0	Summer
8/9/2019	BL	4M	8	2019	Year-2	0	0	Summer
8/9/2019	BL	5M	8	2019	Year-2	0	0	Summer
8/9/2019	BL	6M	8	2019	Year-2	0	0	Summer
8/9/2019	BL	7M	8	2019	Year-2	0	0	Summer
8/9/2019	BL	10M	8	2019	Year-2	0	0	Summer
8/9/2019	BL	12M	8	2019	Year-2	0	0	Summer

Date	Species	Site	Month	Year	Survey Year	Presence	Data Gap	Season
8/9/2019	BL	14M	8	2019	Year-2		1	Summer
8/9/2019	BL	8A	8	2019	Year-2	0	0	Summer
8/9/2019	BL	9A	8	2019	Year-2	0	0	Summer
8/9/2019	BL	11A	8	2019	Year-2	0	0	Summer
8/9/2019	BL	13A	8	2019	Year-2	0	0	Summer
8/10/2019	BL	1M	8	2019	Year-2	0	0	Summer
8/10/2019	BL	2M	8	2019	Year-2	0	0	Summer
8/10/2019	BL	3M	8	2019	Year-2	0	0	Summer
8/10/2019	BL	4M	8	2019	Year-2	0	0	Summer
8/10/2019	BL	5M	8	2019	Year-2	0	0	Summer
8/10/2019	BL	6M	8	2019	Year-2	0	0	Summer
8/10/2019	BL	7M	8	2019	Year-2	0	0	Summer
8/10/2019	BL	10M	8	2019	Year-2	0	0	Summer
8/10/2019	BL	12M	8	2019	Year-2	0	0	Summer
8/10/2019	BL	14M	8	2019	Year-2		1	Summer
8/10/2019	BL	8A	8	2019	Year-2	0	0	Summer
8/10/2019	BL	9A	8	2019	Year-2	0	0	Summer
8/10/2019	BL	11A	8	2019	Year-2	0	0	Summer
8/10/2019	BL	13A	8	2019	Year-2	0	0	Summer
8/11/2019	BL	1M	8	2019	Year-2	0	0	Summer
8/11/2019	BL	2M	8	2019	Year-2	0	0	Summer
8/11/2019	BL	3M	8	2019	Year-2	0	0	Summer
8/11/2019	BL	4M	8	2019	Year-2	0	0	Summer
8/11/2019	BL	5M	8	2019	Year-2	0	0	Summer
8/11/2019	BL	6M	8	2019	Year-2	0	0	Summer
8/11/2019	BL	7M	8	2019	Year-2	0	0	Summer
8/11/2019	BL	10M	8	2019	Year-2	0	0	Summer
8/11/2019	BL	12M	8	2019	Year-2	0	0	Summer
8/11/2019	BL	14M	8	2019	Year-2		1	Summer
8/11/2019	BL	8A	8	2019	Year-2	0	0	Summer
8/11/2019	BL	9A	8	2019	Year-2	0	0	Summer
8/11/2019	BL	11A	8	2019	Year-2	0	0	Summer
8/11/2019	BL	13A	8	2019	Year-2	0	0	Summer
8/12/2019	BL	1M	8	2019	Year-2	0	0	Summer
8/12/2019	BL	2M	8	2019	Year-2	0	0	Summer
8/12/2019	BL	3M	8	2019	Year-2	0	0	Summer
8/12/2019	BL	4M	8	2019	Year-2	0	0	Summer
8/12/2019	BL	5M	8	2019	Year-2	0	0	Summer
8/12/2019	BL	6M	8	2019	Year-2	0	0	Summer
8/12/2019	BL	7M	8	2019	Year-2	0	0	Summer
8/12/2019	BL	10M	8	2019	Year-2	0	0	Summer
8/12/2019	BL	12M	8	2019	Year-2	0	0	Summer
8/12/2019	BL	14M	8	2019	Year-2		1	Summer
8/12/2019	BL	8A	8	2019	Year-2	0	0	Summer

Date	Species	Site	Month	Year	Survey Year	Presence	Data Gap	Season
8/12/2019	BL	9A	8	2019	Year-2	0	0	Summer
8/12/2019	BL	11A	8	2019	Year-2	0	0	Summer
8/12/2019	BL	13A	8	2019	Year-2	0	0	Summer
8/13/2019	BL	1M	8	2019	Year-2	0	0	Summer
8/13/2019	BL	2M	8	2019	Year-2	0	0	Summer
8/13/2019	BL	3M	8	2019	Year-2	0	0	Summer
8/13/2019	BL	4M	8	2019	Year-2	0	0	Summer
8/13/2019	BL	5M	8	2019	Year-2	0	0	Summer
8/13/2019	BL	6M	8	2019	Year-2	0	0	Summer
8/13/2019	BL	7M	8	2019	Year-2	0	0	Summer
8/13/2019	BL	10M	8	2019	Year-2	0	0	Summer
8/13/2019	BL	12M	8	2019	Year-2	0	0	Summer
8/13/2019	BL	14M	8	2019	Year-2		1	Summer
8/13/2019	BL	8A	8	2019	Year-2	0	0	Summer
8/13/2019	BL	9A	8	2019	Year-2	0	0	Summer
8/13/2019	BL	11A	8	2019	Year-2	0	0	Summer
8/13/2019	BL	13A	8	2019	Year-2	0	0	Summer
8/14/2019	BL	1M	8	2019	Year-2	0	0	Summer
8/14/2019	BL	2M	8	2019	Year-2	0	0	Summer
8/14/2019	BL	3M	8	2019	Year-2	0	0	Summer
8/14/2019	BL	4M	8	2019	Year-2	0	0	Summer
8/14/2019	BL	5M	8	2019	Year-2	0	0	Summer
8/14/2019	BL	6M	8	2019	Year-2	0	0	Summer
8/14/2019	BL	7M	8	2019	Year-2	0	0	Summer
8/14/2019	BL	10M	8	2019	Year-2	0	0	Summer
8/14/2019	BL	12M	8	2019	Year-2	0	0	Summer
8/14/2019	BL	14M	8	2019	Year-2		1	Summer
8/14/2019	BL	8A	8	2019	Year-2	0	0	Summer
8/14/2019	BL	9A	8	2019	Year-2	0	0	Summer
8/14/2019	BL	11A	8	2019	Year-2	0	0	Summer
8/14/2019	BL	13A	8	2019	Year-2	0	0	Summer
8/15/2019	BL	1M	8	2019	Year-2	0	0	Summer
8/15/2019	BL	2M	8	2019	Year-2	0	0	Summer
8/15/2019	BL	3M	8	2019	Year-2	0	0	Summer
8/15/2019	BL	4M	8	2019	Year-2	0	0	Summer
8/15/2019	BL	5M	8	2019	Year-2	0	0	Summer
8/15/2019	BL	6M	8	2019	Year-2	0	0	Summer
8/15/2019	BL	7M	8	2019	Year-2	0	0	Summer
8/15/2019	BL	10M	8	2019	Year-2	0	0	Summer
8/15/2019	BL	12M	8	2019	Year-2	0	0	Summer
8/15/2019	BL	14M	8	2019	Year-2		1	Summer
8/15/2019	BL	8A	8	2019	Year-2	0	0	Summer
8/15/2019	BL	9A	8	2019	Year-2	0	0	Summer
8/15/2019	BL	11A	8	2019	Year-2	0	0	Summer

Date	Species	Site	Month	Year	Survey Year	Presence	Data Gap	Season
8/15/2019	BL	13A	8	2019	Year-2	0	0	Summer
8/16/2019	BL	1M	8	2019	Year-2	0	0	Summer
8/16/2019	BL	2M	8	2019	Year-2	0	0	Summer
8/16/2019	BL	3M	8	2019	Year-2	0	0	Summer
8/16/2019	BL	4M	8	2019	Year-2	0	0	Summer
8/16/2019	BL	5M	8	2019	Year-2	0	0	Summer
8/16/2019	BL	6M	8	2019	Year-2	0	0	Summer
8/16/2019	BL	7M	8	2019	Year-2	0	0	Summer
8/16/2019	BL	10M	8	2019	Year-2	0	0	Summer
8/16/2019	BL	12M	8	2019	Year-2	0	0	Summer
8/16/2019	BL	14M	8	2019	Year-2		1	Summer
8/16/2019	BL	8A	8	2019	Year-2	0	0	Summer
8/16/2019	BL	9A	8	2019	Year-2	0	0	Summer
8/16/2019	BL	11A	8	2019	Year-2	0	0	Summer
8/16/2019	BL	13A	8	2019	Year-2	0	0	Summer
8/17/2019	BL	1M	8	2019	Year-2	0	0	Summer
8/17/2019	BL	2M	8	2019	Year-2	0	0	Summer
8/17/2019	BL	3M	8	2019	Year-2	0	0	Summer
8/17/2019	BL	4M	8	2019	Year-2	0	0	Summer
8/17/2019	BL	5M	8	2019	Year-2	0	0	Summer
8/17/2019	BL	6M	8	2019	Year-2	0	0	Summer
8/17/2019	BL	7M	8	2019	Year-2	0	0	Summer
8/17/2019	BL	10M	8	2019	Year-2	0	0	Summer
8/17/2019	BL	12M	8	2019	Year-2	0	0	Summer
8/17/2019	BL	14M	8	2019	Year-2		1	Summer
8/17/2019	BL	8A	8	2019	Year-2	0	0	Summer
8/17/2019	BL	9A	8	2019	Year-2	0	0	Summer
8/17/2019	BL	11A	8	2019	Year-2	0	0	Summer
8/17/2019	BL	13A	8	2019	Year-2	0	0	Summer
8/18/2019	BL	1M	8	2019	Year-2	0	0	Summer
8/18/2019	BL	2M	8	2019	Year-2	0	0	Summer
8/18/2019	BL	3M	8	2019	Year-2	0	0	Summer
8/18/2019	BL	4M	8	2019	Year-2	0	0	Summer
8/18/2019	BL	5M	8	2019	Year-2	0	0	Summer
8/18/2019	BL	6M	8	2019	Year-2	0	0	Summer
8/18/2019	BL	7M	8	2019	Year-2	0	0	Summer
8/18/2019	BL	10M	8	2019	Year-2	0	0	Summer
8/18/2019	BL	12M	8	2019	Year-2	0	0	Summer
8/18/2019	BL	14M	8	2019	Year-2		1	Summer
8/18/2019	BL	8A	8	2019	Year-2	0	0	Summer
8/18/2019	BL	9A	8	2019	Year-2	0	0	Summer
8/18/2019	BL	11A	8	2019	Year-2	0	0	Summer
8/18/2019	BL	13A	8	2019	Year-2	0	0	Summer
8/19/2019	BL	1M	8	2019	Year-2	0	0	Summer

Date	Species	Site	Month	Year	Survey Year	Presence	Data Gap	Season
8/19/2019	BL	2M	8	2019	Year-2	0	0	Summer
8/19/2019	BL	3M	8	2019	Year-2	0	0	Summer
8/19/2019	BL	4M	8	2019	Year-2	0	0	Summer
8/19/2019	BL	5M	8	2019	Year-2	0	0	Summer
8/19/2019	BL	6M	8	2019	Year-2	0	0	Summer
8/19/2019	BL	7M	8	2019	Year-2	0	0	Summer
8/19/2019	BL	10M	8	2019	Year-2	0	0	Summer
8/19/2019	BL	12M	8	2019	Year-2	0	0	Summer
8/19/2019	BL	14M	8	2019	Year-2		1	Summer
8/19/2019	BL	8A	8	2019	Year-2	0	0	Summer
8/19/2019	BL	9A	8	2019	Year-2	0	0	Summer
8/19/2019	BL	11A	8	2019	Year-2	0	0	Summer
8/19/2019	BL	13A	8	2019	Year-2	0	0	Summer
8/20/2019	BL	1M	8	2019	Year-2	0	0	Summer
8/20/2019	BL	2M	8	2019	Year-2	0	0	Summer
8/20/2019	BL	3M	8	2019	Year-2	0	0	Summer
8/20/2019	BL	4M	8	2019	Year-2	0	0	Summer
8/20/2019	BL	5M	8	2019	Year-2	0	0	Summer
8/20/2019	BL	6M	8	2019	Year-2	0	0	Summer
8/20/2019	BL	7M	8	2019	Year-2	0	0	Summer
8/20/2019	BL	10M	8	2019	Year-2	0	0	Summer
8/20/2019	BL	12M	8	2019	Year-2	0	0	Summer
8/20/2019	BL	14M	8	2019	Year-2		1	Summer
8/20/2019	BL	8A	8	2019	Year-2	0	0	Summer
8/20/2019	BL	9A	8	2019	Year-2	0	0	Summer
8/20/2019	BL	11A	8	2019	Year-2	0	0	Summer
8/20/2019	BL	13A	8	2019	Year-2	0	0	Summer
8/21/2019	BL	1M	8	2019	Year-2	0	0	Summer
8/21/2019	BL	2M	8	2019	Year-2	0	0	Summer
8/21/2019	BL	3M	8	2019	Year-2	0	0	Summer
8/21/2019	BL	4M	8	2019	Year-2	0	0	Summer
8/21/2019	BL	5M	8	2019	Year-2	0	0	Summer
8/21/2019	BL	6M	8	2019	Year-2	0	0	Summer
8/21/2019	BL	7M	8	2019	Year-2	0	0	Summer
8/21/2019	BL	10M	8	2019	Year-2	0	0	Summer
8/21/2019	BL	12M	8	2019	Year-2	0	0	Summer
8/21/2019	BL	14M	8	2019	Year-2		1	Summer
8/21/2019	BL	8A	8	2019	Year-2	0	0	Summer
8/21/2019	BL	9A	8	2019	Year-2	0	0	Summer
8/21/2019	BL	11A	8	2019	Year-2	0	0	Summer
8/21/2019	BL	13A	8	2019	Year-2	0	0	Summer
8/22/2019	BL	1M	8	2019	Year-2	0	0	Summer
8/22/2019	BL	2M	8	2019	Year-2	0	0	Summer
8/22/2019	BL	3M	8	2019	Year-2	0	0	Summer

Date	Species	Site	Month	Year	Survey Year	Presence	Data Gap	Season
8/22/2019	BL	4M	8	2019	Year-2	0	0	Summer
8/22/2019	BL	5M	8	2019	Year-2	0	0	Summer
8/22/2019	BL	6M	8	2019	Year-2	0	0	Summer
8/22/2019	BL	7M	8	2019	Year-2	0	0	Summer
8/22/2019	BL	10M	8	2019	Year-2	0	0	Summer
8/22/2019	BL	12M	8	2019	Year-2	0	0	Summer
8/22/2019	BL	14M	8	2019	Year-2		1	Summer
8/22/2019	BL	8A	8	2019	Year-2	0	0	Summer
8/22/2019	BL	9A	8	2019	Year-2	0	0	Summer
8/22/2019	BL	11A	8	2019	Year-2	0	0	Summer
8/22/2019	BL	13A	8	2019	Year-2	0	0	Summer
8/23/2019	BL	1M	8	2019	Year-2	0	0	Summer
8/23/2019	BL	2M	8	2019	Year-2	0	0	Summer
8/23/2019	BL	3M	8	2019	Year-2	0	0	Summer
8/23/2019	BL	4M	8	2019	Year-2	0	0	Summer
8/23/2019	BL	5M	8	2019	Year-2	0	0	Summer
8/23/2019	BL	6M	8	2019	Year-2	0	0	Summer
8/23/2019	BL	7M	8	2019	Year-2	0	0	Summer
8/23/2019	BL	10M	8	2019	Year-2	0	0	Summer
8/23/2019	BL	12M	8	2019	Year-2	0	0	Summer
8/23/2019	BL	14M	8	2019	Year-2		1	Summer
8/23/2019	BL	8A	8	2019	Year-2	0	0	Summer
8/23/2019	BL	9A	8	2019	Year-2	0	0	Summer
8/23/2019	BL	11A	8	2019	Year-2	0	0	Summer
8/23/2019	BL	13A	8	2019	Year-2	0	0	Summer
8/24/2019	BL	1M	8	2019	Year-2	0	0	Summer
8/24/2019	BL	2M	8	2019	Year-2	0	0	Summer
8/24/2019	BL	3M	8	2019	Year-2	0	0	Summer
8/24/2019	BL	4M	8	2019	Year-2	0	0	Summer
8/24/2019	BL	5M	8	2019	Year-2	0	0	Summer
8/24/2019	BL	6M	8	2019	Year-2	0	0	Summer
8/24/2019	BL	7M	8	2019	Year-2	0	0	Summer
8/24/2019	BL	10M	8	2019	Year-2	0	0	Summer
8/24/2019	BL	12M	8	2019	Year-2	0	0	Summer
8/24/2019	BL	14M	8	2019	Year-2		1	Summer
8/24/2019	BL	8A	8	2019	Year-2	0	0	Summer
8/24/2019	BL	9A	8	2019	Year-2	0	0	Summer
8/24/2019	BL	11A	8	2019	Year-2	0	0	Summer
8/24/2019	BL	13A	8	2019	Year-2	0	0	Summer
8/25/2019	BL	1M	8	2019	Year-2	0	0	Summer
8/25/2019	BL	2M	8	2019	Year-2	0	0	Summer
8/25/2019	BL	3M	8	2019	Year-2	0	0	Summer
8/25/2019	BL	4M	8	2019	Year-2	0	0	Summer
8/25/2019	BL	5M	8	2019	Year-2	0	0	Summer

Date	Species	Site	Month	Year	Survey Year	Presence	Data Gap	Season
8/25/2019	BL	6M	8	2019	Year-2	0	0	Summer
8/25/2019	BL	7M	8	2019	Year-2	0	0	Summer
8/25/2019	BL	10M	8	2019	Year-2	0	0	Summer
8/25/2019	BL	12M	8	2019	Year-2	0	0	Summer
8/25/2019	BL	14M	8	2019	Year-2		1	Summer
8/25/2019	BL	8A	8	2019	Year-2	0	0	Summer
8/25/2019	BL	9A	8	2019	Year-2	0	0	Summer
8/25/2019	BL	11A	8	2019	Year-2	0	0	Summer
8/25/2019	BL	13A	8	2019	Year-2	0	0	Summer
8/26/2019	BL	1M	8	2019	Year-2	0	0	Summer
8/26/2019	BL	2M	8	2019	Year-2	0	0	Summer
8/26/2019	BL	3M	8	2019	Year-2	0	0	Summer
8/26/2019	BL	4M	8	2019	Year-2	0	0	Summer
8/26/2019	BL	5M	8	2019	Year-2	0	0	Summer
8/26/2019	BL	6M	8	2019	Year-2	0	0	Summer
8/26/2019	BL	7M	8	2019	Year-2	0	0	Summer
8/26/2019	BL	10M	8	2019	Year-2	0	0	Summer
8/26/2019	BL	12M	8	2019	Year-2	0	0	Summer
8/26/2019	BL	14M	8	2019	Year-2		1	Summer
8/26/2019	BL	8A	8	2019	Year-2	0	0	Summer
8/26/2019	BL	9A	8	2019	Year-2	0	0	Summer
8/26/2019	BL	11A	8	2019	Year-2	0	0	Summer
8/26/2019	BL	13A	8	2019	Year-2	0	0	Summer
8/27/2019	BL	1M	8	2019	Year-2	0	0	Summer
8/27/2019	BL	2M	8	2019	Year-2	0	0	Summer
8/27/2019	BL	3M	8	2019	Year-2	0	0	Summer
8/27/2019	BL	4M	8	2019	Year-2	0	0	Summer
8/27/2019	BL	5M	8	2019	Year-2	0	0	Summer
8/27/2019	BL	6M	8	2019	Year-2	0	0	Summer
8/27/2019	BL	7M	8	2019	Year-2	0	0	Summer
8/27/2019	BL	10M	8	2019	Year-2	0	0	Summer
8/27/2019	BL	12M	8	2019	Year-2	0	0	Summer
8/27/2019	BL	14M	8	2019	Year-2		1	Summer
8/27/2019	BL	8A	8	2019	Year-2	0	0	Summer
8/27/2019	BL	9A	8	2019	Year-2	0	0	Summer
8/27/2019	BL	11A	8	2019	Year-2	0	0	Summer
8/27/2019	BL	13A	8	2019	Year-2	0	0	Summer
8/28/2019	BL	1M	8	2019	Year-2	0	0	Summer
8/28/2019	BL	2M	8	2019	Year-2	0	0	Summer
8/28/2019	BL	3M	8	2019	Year-2	0	0	Summer
8/28/2019	BL	4M	8	2019	Year-2	0	0	Summer
8/28/2019	BL	5M	8	2019	Year-2	0	0	Summer
8/28/2019	BL	6M	8	2019	Year-2	0	0	Summer
8/28/2019	BL	7M	8	2019	Year-2	0	0	Summer

Date	Species	Site	Month	Year	Survey Year	Presence	Data Gap	Season
8/28/2019	BL	10M	8	2019	Year-2	0	0	Summer
8/28/2019	BL	12M	8	2019	Year-2	0	0	Summer
8/28/2019	BL	14M	8	2019	Year-2		1	Summer
8/28/2019	BL	8A	8	2019	Year-2	0	0	Summer
8/28/2019	BL	9A	8	2019	Year-2	0	0	Summer
8/28/2019	BL	11A	8	2019	Year-2	0	0	Summer
8/28/2019	BL	13A	8	2019	Year-2	0	0	Summer
8/29/2019	BL	1M	8	2019	Year-2	0	0	Summer
8/29/2019	BL	2M	8	2019	Year-2	0	0	Summer
8/29/2019	BL	3M	8	2019	Year-2	0	0	Summer
8/29/2019	BL	4M	8	2019	Year-2	0	0	Summer
8/29/2019	BL	5M	8	2019	Year-2	0	0	Summer
8/29/2019	BL	6M	8	2019	Year-2	0	0	Summer
8/29/2019	BL	7M	8	2019	Year-2	0	0	Summer
8/29/2019	BL	10M	8	2019	Year-2	0	0	Summer
8/29/2019	BL	12M	8	2019	Year-2	0	0	Summer
8/29/2019	BL	14M	8	2019	Year-2		1	Summer
8/29/2019	BL	8A	8	2019	Year-2	0	0	Summer
8/29/2019	BL	9A	8	2019	Year-2	0	0	Summer
8/29/2019	BL	11A	8	2019	Year-2	0	0	Summer
8/29/2019	BL	13A	8	2019	Year-2	0	0	Summer
8/30/2019	BL	1M	8	2019	Year-2	0	0	Summer
8/30/2019	BL	2M	8	2019	Year-2	0	0	Summer
8/30/2019	BL	3M	8	2019	Year-2	0	0	Summer
8/30/2019	BL	4M	8	2019	Year-2	0	0	Summer
8/30/2019	BL	5M	8	2019	Year-2	0	0	Summer
8/30/2019	BL	6M	8	2019	Year-2	0	0	Summer
8/30/2019	BL	7M	8	2019	Year-2	0	0	Summer
8/30/2019	BL	10M	8	2019	Year-2	0	0	Summer
8/30/2019	BL	12M	8	2019	Year-2	0	0	Summer
8/30/2019	BL	14M	8	2019	Year-2		1	Summer
8/30/2019	BL	8A	8	2019	Year-2	0	0	Summer
8/30/2019	BL	9A	8	2019	Year-2	0	0	Summer
8/30/2019	BL	11A	8	2019	Year-2	0	0	Summer
8/30/2019	BL	13A	8	2019	Year-2	0	0	Summer
8/31/2019	BL	1M	8	2019	Year-2	0	0	Summer
8/31/2019	BL	2M	8	2019	Year-2	0	0	Summer
8/31/2019	BL	3M	8	2019	Year-2	0	0	Summer
8/31/2019	BL	4M	8	2019	Year-2	0	0	Summer
8/31/2019	BL	5M	8	2019	Year-2	0	0	Summer
8/31/2019	BL	6M	8	2019	Year-2	0	0	Summer
8/31/2019	BL	7M	8	2019	Year-2	0	0	Summer
8/31/2019	BL	10M	8	2019	Year-2	0	0	Summer
8/31/2019	BL	12M	8	2019	Year-2	0	0	Summer

Date	Species	Site	Month	Year	Survey Year	Presence	Data Gap	Season
8/31/2019	BL	14M	8	2019	Year-2		1	Summer
8/31/2019	BL	8A	8	2019	Year-2	0	0	Summer
8/31/2019	BL	9A	8	2019	Year-2	0	0	Summer
8/31/2019	BL	11A	8	2019	Year-2	0	0	Summer
8/31/2019	BL	13A	8	2019	Year-2	0	0	Summer
9/1/2019	BL	1M	9	2019	Year-2	0	0	Fall
9/1/2019	BL	2M	9	2019	Year-2	0	0	Fall
9/1/2019	BL	3M	9	2019	Year-2	0	0	Fall
9/1/2019	BL	4M	9	2019	Year-2	0	0	Fall
9/1/2019	BL	5M	9	2019	Year-2	0	0	Fall
9/1/2019	BL	6M	9	2019	Year-2	0	0	Fall
9/1/2019	BL	7M	9	2019	Year-2	0	0	Fall
9/1/2019	BL	10M	9	2019	Year-2	0	0	Fall
9/1/2019	BL	12M	9	2019	Year-2	0	0	Fall
9/1/2019	BL	14M	9	2019	Year-2		1	Fall
9/1/2019	BL	8A	9	2019	Year-2	0	0	Fall
9/1/2019	BL	9A	9	2019	Year-2	0	0	Fall
9/1/2019	BL	11A	9	2019	Year-2	0	0	Fall
9/1/2019	BL	13A	9	2019	Year-2	0	0	Fall
9/2/2019	BL	1M	9	2019	Year-2	0	0	Fall
9/2/2019	BL	2M	9	2019	Year-2	0	0	Fall
9/2/2019	BL	3M	9	2019	Year-2	0	0	Fall
9/2/2019	BL	4M	9	2019	Year-2	0	0	Fall
9/2/2019	BL	5M	9	2019	Year-2	0	0	Fall
9/2/2019	BL	6M	9	2019	Year-2	0	0	Fall
9/2/2019	BL	7M	9	2019	Year-2	0	0	Fall
9/2/2019	BL	10M	9	2019	Year-2	0	0	Fall
9/2/2019	BL	12M	9	2019	Year-2	0	0	Fall
9/2/2019	BL	14M	9	2019	Year-2		1	Fall
9/2/2019	BL	8A	9	2019	Year-2	0	0	Fall
9/2/2019	BL	9A	9	2019	Year-2	0	0	Fall
9/2/2019	BL	11A	9	2019	Year-2	0	0	Fall
9/2/2019	BL	13A	9	2019	Year-2	0	0	Fall
9/3/2019	BL	1M	9	2019	Year-2	0	0	Fall
9/3/2019	BL	2M	9	2019	Year-2	0	0	Fall
9/3/2019	BL	3M	9	2019	Year-2	0	0	Fall
9/3/2019	BL	4M	9	2019	Year-2	0	0	Fall
9/3/2019	BL	5M	9	2019	Year-2	0	0	Fall
9/3/2019	BL	6M	9	2019	Year-2	0	0	Fall
9/3/2019	BL	7M	9	2019	Year-2	0	0	Fall
9/3/2019	BL	10M	9	2019	Year-2	0	0	Fall
9/3/2019	BL	12M	9	2019	Year-2	0	0	Fall
9/3/2019	BL	14M	9	2019	Year-2		1	Fall
9/3/2019	BL	8A	9	2019	Year-2	0	0	Fall

Date	Species	Site	Month	Year	Survey Year	Presence	Data Gap	Season
9/3/2019	BL	9A	9	2019	Year-2	0	0	Fall
9/3/2019	BL	11A	9	2019	Year-2	0	0	Fall
9/3/2019	BL	13A	9	2019	Year-2	0	0	Fall
9/4/2019	BL	1M	9	2019	Year-2	0	0	Fall
9/4/2019	BL	2M	9	2019	Year-2	0	0	Fall
9/4/2019	BL	3M	9	2019	Year-2	0	0	Fall
9/4/2019	BL	4M	9	2019	Year-2	0	0	Fall
9/4/2019	BL	5M	9	2019	Year-2	0	0	Fall
9/4/2019	BL	6M	9	2019	Year-2	0	0	Fall
9/4/2019	BL	7M	9	2019	Year-2	0	0	Fall
9/4/2019	BL	10M	9	2019	Year-2	0	0	Fall
9/4/2019	BL	12M	9	2019	Year-2	0	0	Fall
9/4/2019	BL	14M	9	2019	Year-2		1	Fall
9/4/2019	BL	8A	9	2019	Year-2	0	0	Fall
9/4/2019	BL	9A	9	2019	Year-2	0	0	Fall
9/4/2019	BL	11A	9	2019	Year-2	0	0	Fall
9/4/2019	BL	13A	9	2019	Year-2	0	0	Fall
9/5/2019	BL	1M	9	2019	Year-2	0	0	Fall
9/5/2019	BL	2M	9	2019	Year-2	0	0	Fall
9/5/2019	BL	3M	9	2019	Year-2	0	0	Fall
9/5/2019	BL	4M	9	2019	Year-2	0	0	Fall
9/5/2019	BL	5M	9	2019	Year-2	0	0	Fall
9/5/2019	BL	6M	9	2019	Year-2	0	0	Fall
9/5/2019	BL	7M	9	2019	Year-2	0	0	Fall
9/5/2019	BL	10M	9	2019	Year-2	0	0	Fall
9/5/2019	BL	12M	9	2019	Year-2	0	0	Fall
9/5/2019	BL	14M	9	2019	Year-2		1	Fall
9/5/2019	BL	8A	9	2019	Year-2	0	0	Fall
9/5/2019	BL	9A	9	2019	Year-2	0	0	Fall
9/5/2019	BL	11A	9	2019	Year-2	0	0	Fall
9/5/2019	BL	13A	9	2019	Year-2	0	0	Fall
9/6/2019	BL	1M	9	2019	Year-2	0	0	Fall
9/6/2019	BL	2M	9	2019	Year-2	0	0	Fall
9/6/2019	BL	3M	9	2019	Year-2	0	0	Fall
9/6/2019	BL	4M	9	2019	Year-2	0	0	Fall
9/6/2019	BL	5M	9	2019	Year-2	0	0	Fall
9/6/2019	BL	6M	9	2019	Year-2	0	0	Fall
9/6/2019	BL	7M	9	2019	Year-2	0	0	Fall
9/6/2019	BL	10M	9	2019	Year-2	0	0	Fall
9/6/2019	BL	12M	9	2019	Year-2	0	0	Fall
9/6/2019	BL	14M	9	2019	Year-2		1	Fall
9/6/2019	BL	8A	9	2019	Year-2	0	0	Fall
9/6/2019	BL	9A	9	2019	Year-2	0	0	Fall
9/6/2019	BL	11A	9	2019	Year-2	0	0	Fall

Date	Species	Site	Month	Year	Survey Year	Presence	Data Gap	Season
9/6/2019	BL	13A	9	2019	Year-2	0	0	Fall
9/7/2019	BL	1M	9	2019	Year-2	0	0	Fall
9/7/2019	BL	2M	9	2019	Year-2	0	0	Fall
9/7/2019	BL	3M	9	2019	Year-2	0	0	Fall
9/7/2019	BL	4M	9	2019	Year-2	0	0	Fall
9/7/2019	BL	5M	9	2019	Year-2	0	0	Fall
9/7/2019	BL	6M	9	2019	Year-2	0	0	Fall
9/7/2019	BL	7M	9	2019	Year-2	0	0	Fall
9/7/2019	BL	10M	9	2019	Year-2	0	0	Fall
9/7/2019	BL	12M	9	2019	Year-2	0	0	Fall
9/7/2019	BL	14M	9	2019	Year-2		1	Fall
9/7/2019	BL	8A	9	2019	Year-2	0	0	Fall
9/7/2019	BL	9A	9	2019	Year-2	0	0	Fall
9/7/2019	BL	11A	9	2019	Year-2	0	0	Fall
9/7/2019	BL	13A	9	2019	Year-2	0	0	Fall
9/8/2019	BL	1M	9	2019	Year-2	0	0	Fall
9/8/2019	BL	2M	9	2019	Year-2	0	0	Fall
9/8/2019	BL	3M	9	2019	Year-2	0	0	Fall
9/8/2019	BL	4M	9	2019	Year-2	0	0	Fall
9/8/2019	BL	5M	9	2019	Year-2	0	0	Fall
9/8/2019	BL	6M	9	2019	Year-2	0	0	Fall
9/8/2019	BL	7M	9	2019	Year-2	0	0	Fall
9/8/2019	BL	10M	9	2019	Year-2	0	0	Fall
9/8/2019	BL	12M	9	2019	Year-2	0	0	Fall
9/8/2019	BL	14M	9	2019	Year-2		1	Fall
9/8/2019	BL	8A	9	2019	Year-2	0	0	Fall
9/8/2019	BL	9A	9	2019	Year-2	0	0	Fall
9/8/2019	BL	11A	9	2019	Year-2	0	0	Fall
9/8/2019	BL	13A	9	2019	Year-2	0	0	Fall
9/9/2019	BL	1M	9	2019	Year-2	0	0	Fall
9/9/2019	BL	2M	9	2019	Year-2	0	0	Fall
9/9/2019	BL	3M	9	2019	Year-2	0	0	Fall
9/9/2019	BL	4M	9	2019	Year-2	0	0	Fall
9/9/2019	BL	5M	9	2019	Year-2	0	0	Fall
9/9/2019	BL	6M	9	2019	Year-2	0	0	Fall
9/9/2019	BL	7M	9	2019	Year-2	0	0	Fall
9/9/2019	BL	10M	9	2019	Year-2	0	0	Fall
9/9/2019	BL	12M	9	2019	Year-2	0	0	Fall
9/9/2019	BL	14M	9	2019	Year-2		1	Fall
9/9/2019	BL	8A	9	2019	Year-2	0	0	Fall
9/9/2019	BL	9A	9	2019	Year-2	0	0	Fall
9/9/2019	BL	11A	9	2019	Year-2	0	0	Fall
9/9/2019	BL	13A	9	2019	Year-2	0	0	Fall
9/10/2019	BL	1M	9	2019	Year-2	0	0	Fall

Date	Species	Site	Month	Year	Survey Year	Presence	Data Gap	Season
9/10/2019	BL	2M	9	2019	Year-2	0	0	Fall
9/10/2019	BL	3M	9	2019	Year-2	0	0	Fall
9/10/2019	BL	4M	9	2019	Year-2	0	0	Fall
9/10/2019	BL	5M	9	2019	Year-2	0	0	Fall
9/10/2019	BL	6M	9	2019	Year-2	0	0	Fall
9/10/2019	BL	7M	9	2019	Year-2	0	0	Fall
9/10/2019	BL	10M	9	2019	Year-2	0	0	Fall
9/10/2019	BL	12M	9	2019	Year-2	0	0	Fall
9/10/2019	BL	14M	9	2019	Year-2		1	Fall
9/10/2019	BL	8A	9	2019	Year-2	0	0	Fall
9/10/2019	BL	9A	9	2019	Year-2	0	0	Fall
9/10/2019	BL	11A	9	2019	Year-2	0	0	Fall
9/10/2019	BL	13A	9	2019	Year-2	0	0	Fall
9/11/2019	BL	1M	9	2019	Year-2	0	0	Fall
9/11/2019	BL	2M	9	2019	Year-2	0	0	Fall
9/11/2019	BL	3M	9	2019	Year-2	0	0	Fall
9/11/2019	BL	4M	9	2019	Year-2	0	0	Fall
9/11/2019	BL	5M	9	2019	Year-2	0	0	Fall
9/11/2019	BL	6M	9	2019	Year-2	0	0	Fall
9/11/2019	BL	7M	9	2019	Year-2	0	0	Fall
9/11/2019	BL	10M	9	2019	Year-2	0	0	Fall
9/11/2019	BL	12M	9	2019	Year-2	0	0	Fall
9/11/2019	BL	14M	9	2019	Year-2		1	Fall
9/11/2019	BL	8A	9	2019	Year-2	0	0	Fall
9/11/2019	BL	9A	9	2019	Year-2	0	0	Fall
9/11/2019	BL	11A	9	2019	Year-2	0	0	Fall
9/11/2019	BL	13A	9	2019	Year-2	0	0	Fall
9/12/2019	BL	1M	9	2019	Year-2	0	0	Fall
9/12/2019	BL	2M	9	2019	Year-2	0	0	Fall
9/12/2019	BL	3M	9	2019	Year-2	0	0	Fall
9/12/2019	BL	4M	9	2019	Year-2	0	0	Fall
9/12/2019	BL	5M	9	2019	Year-2	0	0	Fall
9/12/2019	BL	6M	9	2019	Year-2	0	0	Fall
9/12/2019	BL	7M	9	2019	Year-2	0	0	Fall
9/12/2019	BL	10M	9	2019	Year-2	0	0	Fall
9/12/2019	BL	12M	9	2019	Year-2	0	0	Fall
9/12/2019	BL	14M	9	2019	Year-2		1	Fall
9/12/2019	BL	8A	9	2019	Year-2	0	0	Fall
9/12/2019	BL	9A	9	2019	Year-2	0	0	Fall
9/12/2019	BL	11A	9	2019	Year-2	0	0	Fall
9/12/2019	BL	13A	9	2019	Year-2	0	0	Fall
9/13/2019	BL	1M	9	2019	Year-2	0	0	Fall
9/13/2019	BL	2M	9	2019	Year-2	0	0	Fall
9/13/2019	BL	3M	9	2019	Year-2	0	0	Fall

Date	Species	Site	Month	Year	Survey Year	Presence	Data Gap	Season
9/13/2019	BL	4M	9	2019	Year-2	0	0	Fall
9/13/2019	BL	5M	9	2019	Year-2	0	0	Fall
9/13/2019	BL	6M	9	2019	Year-2	0	0	Fall
9/13/2019	BL	7M	9	2019	Year-2	0	0	Fall
9/13/2019	BL	10M	9	2019	Year-2	0	0	Fall
9/13/2019	BL	12M	9	2019	Year-2	0	0	Fall
9/13/2019	BL	14M	9	2019	Year-2		1	Fall
9/13/2019	BL	8A	9	2019	Year-2	0	0	Fall
9/13/2019	BL	9A	9	2019	Year-2	0	0	Fall
9/13/2019	BL	11A	9	2019	Year-2	0	0	Fall
9/13/2019	BL	13A	9	2019	Year-2	0	0	Fall
9/14/2019	BL	1M	9	2019	Year-2	0	0	Fall
9/14/2019	BL	2M	9	2019	Year-2	0	0	Fall
9/14/2019	BL	3M	9	2019	Year-2	0	0	Fall
9/14/2019	BL	4M	9	2019	Year-2	0	0	Fall
9/14/2019	BL	5M	9	2019	Year-2	0	0	Fall
9/14/2019	BL	6M	9	2019	Year-2	0	0	Fall
9/14/2019	BL	7M	9	2019	Year-2	0	0	Fall
9/14/2019	BL	10M	9	2019	Year-2	0	0	Fall
9/14/2019	BL	12M	9	2019	Year-2	0	0	Fall
9/14/2019	BL	14M	9	2019	Year-2		1	Fall
9/14/2019	BL	8A	9	2019	Year-2	0	0	Fall
9/14/2019	BL	9A	9	2019	Year-2	0	0	Fall
9/14/2019	BL	11A	9	2019	Year-2	0	0	Fall
9/14/2019	BL	13A	9	2019	Year-2	0	0	Fall
9/15/2019	BL	1M	9	2019	Year-2	0	0	Fall
9/15/2019	BL	2M	9	2019	Year-2	0	0	Fall
9/15/2019	BL	3M	9	2019	Year-2	0	0	Fall
9/15/2019	BL	4M	9	2019	Year-2	0	0	Fall
9/15/2019	BL	5M	9	2019	Year-2	0	0	Fall
9/15/2019	BL	6M	9	2019	Year-2	0	0	Fall
9/15/2019	BL	7M	9	2019	Year-2	0	0	Fall
9/15/2019	BL	10M	9	2019	Year-2	0	0	Fall
9/15/2019	BL	12M	9	2019	Year-2	0	0	Fall
9/15/2019	BL	14M	9	2019	Year-2		1	Fall
9/15/2019	BL	8A	9	2019	Year-2	0	0	Fall
9/15/2019	BL	9A	9	2019	Year-2	0	0	Fall
9/15/2019	BL	11A	9	2019	Year-2	0	0	Fall
9/15/2019	BL	13A	9	2019	Year-2	0	0	Fall
9/16/2019	BL	1M	9	2019	Year-2	0	0	Fall
9/16/2019	BL	2M	9	2019	Year-2	0	0	Fall
9/16/2019	BL	3M	9	2019	Year-2	0	0	Fall
9/16/2019	BL	4M	9	2019	Year-2	0	0	Fall
9/16/2019	BL	5M	9	2019	Year-2	0	0	Fall

Date	Species	Site	Month	Year	Survey Year	Presence	Data Gap	Season
9/16/2019	BL	6M	9	2019	Year-2	0	0	Fall
9/16/2019	BL	7M	9	2019	Year-2	0	0	Fall
9/16/2019	BL	10M	9	2019	Year-2	0	0	Fall
9/16/2019	BL	12M	9	2019	Year-2	0	0	Fall
9/16/2019	BL	14M	9	2019	Year-2		1	Fall
9/16/2019	BL	8A	9	2019	Year-2	0	0	Fall
9/16/2019	BL	9A	9	2019	Year-2	0	0	Fall
9/16/2019	BL	11A	9	2019	Year-2	0	0	Fall
9/16/2019	BL	13A	9	2019	Year-2	0	0	Fall
9/17/2019	BL	1M	9	2019	Year-2	0	0	Fall
9/17/2019	BL	2M	9	2019	Year-2	0	0	Fall
9/17/2019	BL	3M	9	2019	Year-2	0	0	Fall
9/17/2019	BL	4M	9	2019	Year-2	0	0	Fall
9/17/2019	BL	5M	9	2019	Year-2	0	0	Fall
9/17/2019	BL	6M	9	2019	Year-2	0	0	Fall
9/17/2019	BL	7M	9	2019	Year-2	0	0	Fall
9/17/2019	BL	10M	9	2019	Year-2	0	0	Fall
9/17/2019	BL	12M	9	2019	Year-2	0	0	Fall
9/17/2019	BL	14M	9	2019	Year-2		1	Fall
9/17/2019	BL	8A	9	2019	Year-2	0	0	Fall
9/17/2019	BL	9A	9	2019	Year-2	0	0	Fall
9/17/2019	BL	11A	9	2019	Year-2	0	0	Fall
9/17/2019	BL	13A	9	2019	Year-2	0	0	Fall
9/18/2019	BL	1M	9	2019	Year-2	0	0	Fall
9/18/2019	BL	2M	9	2019	Year-2	0	0	Fall
9/18/2019	BL	3M	9	2019	Year-2	0	0	Fall
9/18/2019	BL	4M	9	2019	Year-2	0	0	Fall
9/18/2019	BL	5M	9	2019	Year-2	0	0	Fall
9/18/2019	BL	6M	9	2019	Year-2	0	0	Fall
9/18/2019	BL	7M	9	2019	Year-2	0	0	Fall
9/18/2019	BL	10M	9	2019	Year-2	0	0	Fall
9/18/2019	BL	12M	9	2019	Year-2	0	0	Fall
9/18/2019	BL	14M	9	2019	Year-2		1	Fall
9/18/2019	BL	8A	9	2019	Year-2	0	0	Fall
9/18/2019	BL	9A	9	2019	Year-2	0	0	Fall
9/18/2019	BL	11A	9	2019	Year-2	0	0	Fall
9/18/2019	BL	13A	9	2019	Year-2	0	0	Fall
9/19/2019	BL	1M	9	2019	Year-2	0	0	Fall
9/19/2019	BL	2M	9	2019	Year-2	0	0	Fall
9/19/2019	BL	3M	9	2019	Year-2	0	0	Fall
9/19/2019	BL	4M	9	2019	Year-2	0	0	Fall
9/19/2019	BL	5M	9	2019	Year-2	0	0	Fall
9/19/2019	BL	6M	9	2019	Year-2	0	0	Fall
9/19/2019	BL	7M	9	2019	Year-2	0	0	Fall

Date	Species	Site	Month	Year	Survey Year	Presence	Data Gap	Season
9/19/2019	BL	10M	9	2019	Year-2	0	0	Fall
9/19/2019	BL	12M	9	2019	Year-2	0	0	Fall
9/19/2019	BL	14M	9	2019	Year-2		1	Fall
9/19/2019	BL	8A	9	2019	Year-2	0	0	Fall
9/19/2019	BL	9A	9	2019	Year-2	0	0	Fall
9/19/2019	BL	11A	9	2019	Year-2	0	0	Fall
9/19/2019	BL	13A	9	2019	Year-2	0	0	Fall
9/20/2019	BL	1M	9	2019	Year-2	0	0	Fall
9/20/2019	BL	2M	9	2019	Year-2	0	0	Fall
9/20/2019	BL	3M	9	2019	Year-2	0	0	Fall
9/20/2019	BL	4M	9	2019	Year-2	0	0	Fall
9/20/2019	BL	5M	9	2019	Year-2	0	0	Fall
9/20/2019	BL	6M	9	2019	Year-2	0	0	Fall
9/20/2019	BL	7M	9	2019	Year-2	0	0	Fall
9/20/2019	BL	10M	9	2019	Year-2	0	0	Fall
9/20/2019	BL	12M	9	2019	Year-2	0	0	Fall
9/20/2019	BL	14M	9	2019	Year-2		1	Fall
9/20/2019	BL	8A	9	2019	Year-2	0	0	Fall
9/20/2019	BL	9A	9	2019	Year-2	0	0	Fall
9/20/2019	BL	11A	9	2019	Year-2	0	0	Fall
9/20/2019	BL	13A	9	2019	Year-2	0	0	Fall
9/21/2019	BL	1M	9	2019	Year-2	0	0	Fall
9/21/2019	BL	2M	9	2019	Year-2	0	0	Fall
9/21/2019	BL	3M	9	2019	Year-2	0	0	Fall
9/21/2019	BL	4M	9	2019	Year-2	0	0	Fall
9/21/2019	BL	5M	9	2019	Year-2	0	0	Fall
9/21/2019	BL	6M	9	2019	Year-2	0	0	Fall
9/21/2019	BL	7M	9	2019	Year-2	0	0	Fall
9/21/2019	BL	10M	9	2019	Year-2	0	0	Fall
9/21/2019	BL	12M	9	2019	Year-2	0	0	Fall
9/21/2019	BL	14M	9	2019	Year-2		1	Fall
9/21/2019	BL	8A	9	2019	Year-2	0	0	Fall
9/21/2019	BL	9A	9	2019	Year-2	0	0	Fall
9/21/2019	BL	11A	9	2019	Year-2	0	0	Fall
9/21/2019	BL	13A	9	2019	Year-2	0	0	Fall
9/22/2019	BL	1M	9	2019	Year-2	0	0	Fall
9/22/2019	BL	2M	9	2019	Year-2	0	0	Fall
9/22/2019	BL	3M	9	2019	Year-2	0	0	Fall
9/22/2019	BL	4M	9	2019	Year-2	0	0	Fall
9/22/2019	BL	5M	9	2019	Year-2	0	0	Fall
9/22/2019	BL	6M	9	2019	Year-2	0	0	Fall
9/22/2019	BL	7M	9	2019	Year-2	0	0	Fall
9/22/2019	BL	10M	9	2019	Year-2	0	0	Fall
9/22/2019	BL	12M	9	2019	Year-2	0	0	Fall

Date	Species	Site	Month	Year	Survey Year	Presence	Data Gap	Season
9/22/2019	BL	14M	9	2019	Year-2		1	Fall
9/22/2019	BL	8A	9	2019	Year-2	0	0	Fall
9/22/2019	BL	9A	9	2019	Year-2	0	0	Fall
9/22/2019	BL	11A	9	2019	Year-2	0	0	Fall
9/22/2019	BL	13A	9	2019	Year-2	0	0	Fall
9/23/2019	BL	1M	9	2019	Year-2	0	0	Fall
9/23/2019	BL	2M	9	2019	Year-2	0	0	Fall
9/23/2019	BL	3M	9	2019	Year-2	0	0	Fall
9/23/2019	BL	4M	9	2019	Year-2	0	0	Fall
9/23/2019	BL	5M	9	2019	Year-2	0	0	Fall
9/23/2019	BL	6M	9	2019	Year-2	0	0	Fall
9/23/2019	BL	7M	9	2019	Year-2	0	0	Fall
9/23/2019	BL	10M	9	2019	Year-2	0	0	Fall
9/23/2019	BL	12M	9	2019	Year-2	0	0	Fall
9/23/2019	BL	14M	9	2019	Year-2		1	Fall
9/23/2019	BL	8A	9	2019	Year-2	0	0	Fall
9/23/2019	BL	9A	9	2019	Year-2	0	0	Fall
9/23/2019	BL	11A	9	2019	Year-2	0	0	Fall
9/23/2019	BL	13A	9	2019	Year-2	0	0	Fall
9/24/2019	BL	1M	9	2019	Year-2	0	0	Fall
9/24/2019	BL	2M	9	2019	Year-2	0	0	Fall
9/24/2019	BL	3M	9	2019	Year-2	0	0	Fall
9/24/2019	BL	4M	9	2019	Year-2	0	0	Fall
9/24/2019	BL	5M	9	2019	Year-2	0	0	Fall
9/24/2019	BL	6M	9	2019	Year-2	0	0	Fall
9/24/2019	BL	7M	9	2019	Year-2	0	0	Fall
9/24/2019	BL	10M	9	2019	Year-2	0	0	Fall
9/24/2019	BL	12M	9	2019	Year-2	0	0	Fall
9/24/2019	BL	14M	9	2019	Year-2		1	Fall
9/24/2019	BL	8A	9	2019	Year-2	0	0	Fall
9/24/2019	BL	9A	9	2019	Year-2	0	0	Fall
9/24/2019	BL	11A	9	2019	Year-2	0	0	Fall
9/24/2019	BL	13A	9	2019	Year-2	0	0	Fall
9/25/2019	BL	1M	9	2019	Year-2	0	0	Fall
9/25/2019	BL	2M	9	2019	Year-2	0	0	Fall
9/25/2019	BL	3M	9	2019	Year-2	0	0	Fall
9/25/2019	BL	4M	9	2019	Year-2	0	0	Fall
9/25/2019	BL	5M	9	2019	Year-2	0	0	Fall
9/25/2019	BL	6M	9	2019	Year-2	0	0	Fall
9/25/2019	BL	7M	9	2019	Year-2	0	0	Fall
9/25/2019	BL	10M	9	2019	Year-2	0	0	Fall
9/25/2019	BL	12M	9	2019	Year-2	0	0	Fall
9/25/2019	BL	14M	9	2019	Year-2		1	Fall
9/25/2019	BL	8A	9	2019	Year-2	0	0	Fall

Date	Species	Site	Month	Year	Survey Year	Presence	Data Gap	Season
9/25/2019	BL	9A	9	2019	Year-2	0	0	Fall
9/25/2019	BL	11A	9	2019	Year-2	0	0	Fall
9/25/2019	BL	13A	9	2019	Year-2	0	0	Fall
9/26/2019	BL	1M	9	2019	Year-2	0	0	Fall
9/26/2019	BL	2M	9	2019	Year-2	0	0	Fall
9/26/2019	BL	3M	9	2019	Year-2	0	0	Fall
9/26/2019	BL	4M	9	2019	Year-2	0	0	Fall
9/26/2019	BL	5M	9	2019	Year-2	0	0	Fall
9/26/2019	BL	6M	9	2019	Year-2	0	0	Fall
9/26/2019	BL	7M	9	2019	Year-2	0	0	Fall
9/26/2019	BL	10M	9	2019	Year-2	0	0	Fall
9/26/2019	BL	12M	9	2019	Year-2	0	0	Fall
9/26/2019	BL	14M	9	2019	Year-2		1	Fall
9/26/2019	BL	8A	9	2019	Year-2	0	0	Fall
9/26/2019	BL	9A	9	2019	Year-2	0	0	Fall
9/26/2019	BL	11A	9	2019	Year-2	0	0	Fall
9/26/2019	BL	13A	9	2019	Year-2	0	0	Fall
9/27/2019	BL	1M	9	2019	Year-2	0	0	Fall
9/27/2019	BL	2M	9	2019	Year-2	0	0	Fall
9/27/2019	BL	3M	9	2019	Year-2	0	0	Fall
9/27/2019	BL	4M	9	2019	Year-2	0	0	Fall
9/27/2019	BL	5M	9	2019	Year-2	0	0	Fall
9/27/2019	BL	6M	9	2019	Year-2	0	0	Fall
9/27/2019	BL	7M	9	2019	Year-2	0	0	Fall
9/27/2019	BL	10M	9	2019	Year-2	0	0	Fall
9/27/2019	BL	12M	9	2019	Year-2	0	0	Fall
9/27/2019	BL	14M	9	2019	Year-2		1	Fall
9/27/2019	BL	8A	9	2019	Year-2	0	0	Fall
9/27/2019	BL	9A	9	2019	Year-2	0	0	Fall
9/27/2019	BL	11A	9	2019	Year-2	0	0	Fall
9/27/2019	BL	13A	9	2019	Year-2	0	0	Fall
9/28/2019	BL	1M	9	2019	Year-2	0	0	Fall
9/28/2019	BL	2M	9	2019	Year-2	0	0	Fall
9/28/2019	BL	3M	9	2019	Year-2	0	0	Fall
9/28/2019	BL	4M	9	2019	Year-2	0	0	Fall
9/28/2019	BL	5M	9	2019	Year-2	0	0	Fall
9/28/2019	BL	6M	9	2019	Year-2	0	0	Fall
9/28/2019	BL	7M	9	2019	Year-2	0	0	Fall
9/28/2019	BL	10M	9	2019	Year-2	0	0	Fall
9/28/2019	BL	12M	9	2019	Year-2	0	0	Fall
9/28/2019	BL	14M	9	2019	Year-2		1	Fall
9/28/2019	BL	8A	9	2019	Year-2	0	0	Fall
9/28/2019	BL	9A	9	2019	Year-2	0	0	Fall
9/28/2019	BL	11A	9	2019	Year-2	0	0	Fall

Date	Species	Site	Month	Year	Survey Year	Presence	Data Gap	Season
9/28/2019	BL	13A	9	2019	Year-2	0	0	Fall
9/29/2019	BL	1M	9	2019	Year-2	0	0	Fall
9/29/2019	BL	2M	9	2019	Year-2	0	0	Fall
9/29/2019	BL	3M	9	2019	Year-2	0	0	Fall
9/29/2019	BL	4M	9	2019	Year-2	0	0	Fall
9/29/2019	BL	5M	9	2019	Year-2	0	0	Fall
9/29/2019	BL	6M	9	2019	Year-2	0	0	Fall
9/29/2019	BL	7M	9	2019	Year-2	0	0	Fall
9/29/2019	BL	10M	9	2019	Year-2	0	0	Fall
9/29/2019	BL	12M	9	2019	Year-2	0	0	Fall
9/29/2019	BL	14M	9	2019	Year-2		1	Fall
9/29/2019	BL	8A	9	2019	Year-2	0	0	Fall
9/29/2019	BL	9A	9	2019	Year-2	0	0	Fall
9/29/2019	BL	11A	9	2019	Year-2	0	0	Fall
9/29/2019	BL	13A	9	2019	Year-2	0	0	Fall
9/30/2019	BL	1M	9	2019	Year-2	0	0	Fall
9/30/2019	BL	2M	9	2019	Year-2	0	0	Fall
9/30/2019	BL	3M	9	2019	Year-2	0	0	Fall
9/30/2019	BL	4M	9	2019	Year-2	0	0	Fall
9/30/2019	BL	5M	9	2019	Year-2	0	0	Fall
9/30/2019	BL	6M	9	2019	Year-2	0	0	Fall
9/30/2019	BL	7M	9	2019	Year-2	0	0	Fall
9/30/2019	BL	10M	9	2019	Year-2	0	0	Fall
9/30/2019	BL	12M	9	2019	Year-2	0	0	Fall
9/30/2019	BL	14M	9	2019	Year-2		1	Fall
9/30/2019	BL	8A	9	2019	Year-2	0	0	Fall
9/30/2019	BL	9A	9	2019	Year-2	0	0	Fall
9/30/2019	BL	11A	9	2019	Year-2	0	0	Fall
9/30/2019	BL	13A	9	2019	Year-2	0	0	Fall
10/1/2019	BL	1M	10	2019	Year-2	0	0	Fall
10/1/2019	BL	2M	10	2019	Year-2	0	0	Fall
10/1/2019	BL	3M	10	2019	Year-2	0	0	Fall
10/1/2019	BL	4M	10	2019	Year-2	0	0	Fall
10/1/2019	BL	5M	10	2019	Year-2	0	0	Fall
10/1/2019	BL	6M	10	2019	Year-2	0	0	Fall
10/1/2019	BL	7M	10	2019	Year-2	0	0	Fall
10/1/2019	BL	10M	10	2019	Year-2	0	0	Fall
10/1/2019	BL	12M	10	2019	Year-2	0	0	Fall
10/1/2019	BL	14M	10	2019	Year-2		1	Fall
10/1/2019	BL	8A	10	2019	Year-2	0	0	Fall
10/1/2019	BL	9A	10	2019	Year-2	0	0	Fall
10/1/2019	BL	11A	10	2019	Year-2	0	0	Fall
10/1/2019	BL	13A	10	2019	Year-2	0	0	Fall
10/2/2019	BL	1M	10	2019	Year-2	0	0	Fall

Date	Species	Site	Month	Year	Survey Year	Presence	Data Gap	Season
10/2/2019	BL	2M	10	2019	Year-2	0	0	Fall
10/2/2019	BL	3M	10	2019	Year-2	0	0	Fall
10/2/2019	BL	4M	10	2019	Year-2	0	0	Fall
10/2/2019	BL	5M	10	2019	Year-2	0	0	Fall
10/2/2019	BL	6M	10	2019	Year-2	0	0	Fall
10/2/2019	BL	7M	10	2019	Year-2	0	0	Fall
10/2/2019	BL	10M	10	2019	Year-2	0	0	Fall
10/2/2019	BL	12M	10	2019	Year-2	0	0	Fall
10/2/2019	BL	14M	10	2019	Year-2		1	Fall
10/2/2019	BL	8A	10	2019	Year-2	0	0	Fall
10/2/2019	BL	9A	10	2019	Year-2	0	0	Fall
10/2/2019	BL	11A	10	2019	Year-2	0	0	Fall
10/2/2019	BL	13A	10	2019	Year-2	0	0	Fall
10/3/2019	BL	1M	10	2019	Year-2	0	0	Fall
10/3/2019	BL	2M	10	2019	Year-2	0	0	Fall
10/3/2019	BL	3M	10	2019	Year-2	0	0	Fall
10/3/2019	BL	4M	10	2019	Year-2	0	0	Fall
10/3/2019	BL	5M	10	2019	Year-2	0	0	Fall
10/3/2019	BL	6M	10	2019	Year-2	0	0	Fall
10/3/2019	BL	7M	10	2019	Year-2	0	0	Fall
10/3/2019	BL	10M	10	2019	Year-2	0	0	Fall
10/3/2019	BL	12M	10	2019	Year-2	0	0	Fall
10/3/2019	BL	14M	10	2019	Year-2		1	Fall
10/3/2019	BL	8A	10	2019	Year-2	0	0	Fall
10/3/2019	BL	9A	10	2019	Year-2	0	0	Fall
10/3/2019	BL	11A	10	2019	Year-2	0	0	Fall
10/3/2019	BL	13A	10	2019	Year-2	0	0	Fall
10/4/2019	BL	1M	10	2019	Year-2	0	0	Fall
10/4/2019	BL	2M	10	2019	Year-2	0	0	Fall
10/4/2019	BL	3M	10	2019	Year-2	0	0	Fall
10/4/2019	BL	4M	10	2019	Year-2	0	0	Fall
10/4/2019	BL	5M	10	2019	Year-2	0	0	Fall
10/4/2019	BL	6M	10	2019	Year-2	0	0	Fall
10/4/2019	BL	7M	10	2019	Year-2	0	0	Fall
10/4/2019	BL	10M	10	2019	Year-2	0	0	Fall
10/4/2019	BL	12M	10	2019	Year-2	0	0	Fall
10/4/2019	BL	14M	10	2019	Year-2		1	Fall
10/4/2019	BL	8A	10	2019	Year-2	0	0	Fall
10/4/2019	BL	9A	10	2019	Year-2	0	0	Fall
10/4/2019	BL	11A	10	2019	Year-2	0	0	Fall
10/4/2019	BL	13A	10	2019	Year-2	0	0	Fall
10/5/2019	BL	1M	10	2019	Year-2	0	0	Fall
10/5/2019	BL	2M	10	2019	Year-2	0	0	Fall
10/5/2019	BL	3M	10	2019	Year-2	0	0	Fall

Date	Species	Site	Month	Year	Survey Year	Presence	Data Gap	Season
10/5/2019	BL	4M	10	2019	Year-2	0	0	Fall
10/5/2019	BL	5M	10	2019	Year-2	0	0	Fall
10/5/2019	BL	6M	10	2019	Year-2	0	0	Fall
10/5/2019	BL	7M	10	2019	Year-2	0	0	Fall
10/5/2019	BL	10M	10	2019	Year-2	0	0	Fall
10/5/2019	BL	12M	10	2019	Year-2	0	0	Fall
10/5/2019	BL	14M	10	2019	Year-2		1	Fall
10/5/2019	BL	8A	10	2019	Year-2	0	0	Fall
10/5/2019	BL	9A	10	2019	Year-2	0	0	Fall
10/5/2019	BL	11A	10	2019	Year-2	0	0	Fall
10/5/2019	BL	13A	10	2019	Year-2	0	0	Fall
10/6/2019	BL	1M	10	2019	Year-2	0	0	Fall
10/6/2019	BL	2M	10	2019	Year-2	0	0	Fall
10/6/2019	BL	3M	10	2019	Year-2	0	0	Fall
10/6/2019	BL	4M	10	2019	Year-2	0	0	Fall
10/6/2019	BL	5M	10	2019	Year-2	0	0	Fall
10/6/2019	BL	6M	10	2019	Year-2	0	0	Fall
10/6/2019	BL	7M	10	2019	Year-2	0	0	Fall
10/6/2019	BL	10M	10	2019	Year-2	0	0	Fall
10/6/2019	BL	12M	10	2019	Year-2	0	0	Fall
10/6/2019	BL	14M	10	2019	Year-2		1	Fall
10/6/2019	BL	8A	10	2019	Year-2	0	0	Fall
10/6/2019	BL	9A	10	2019	Year-2	0	0	Fall
10/6/2019	BL	11A	10	2019	Year-2	0	0	Fall
10/6/2019	BL	13A	10	2019	Year-2	0	0	Fall
10/7/2019	BL	1M	10	2019	Year-2	0	0	Fall
10/7/2019	BL	2M	10	2019	Year-2	0	0	Fall
10/7/2019	BL	3M	10	2019	Year-2	0	0	Fall
10/7/2019	BL	4M	10	2019	Year-2	0	0	Fall
10/7/2019	BL	5M	10	2019	Year-2	0	0	Fall
10/7/2019	BL	6M	10	2019	Year-2	0	0	Fall
10/7/2019	BL	7M	10	2019	Year-2	0	0	Fall
10/7/2019	BL	10M	10	2019	Year-2	0	0	Fall
10/7/2019	BL	12M	10	2019	Year-2	0	0	Fall
10/7/2019	BL	14M	10	2019	Year-2		1	Fall
10/7/2019	BL	8A	10	2019	Year-2	0	0	Fall
10/7/2019	BL	9A	10	2019	Year-2	0	0	Fall
10/7/2019	BL	11A	10	2019	Year-2	0	0	Fall
10/7/2019	BL	13A	10	2019	Year-2	0	0	Fall
10/8/2019	BL	1M	10	2019	Year-2	0	0	Fall
10/8/2019	BL	2M	10	2019	Year-2	0	0	Fall
10/8/2019	BL	3M	10	2019	Year-2	0	0	Fall
10/8/2019	BL	4M	10	2019	Year-2	0	0	Fall
10/8/2019	BL	5M	10	2019	Year-2	0	0	Fall

Date	Species	Site	Month	Year	Survey Year	Presence	Data Gap	Season
10/8/2019	BL	6M	10	2019	Year-2	0	0	Fall
10/8/2019	BL	7M	10	2019	Year-2	0	0	Fall
10/8/2019	BL	10M	10	2019	Year-2	0	0	Fall
10/8/2019	BL	12M	10	2019	Year-2	0	0	Fall
10/8/2019	BL	14M	10	2019	Year-2		1	Fall
10/8/2019	BL	8A	10	2019	Year-2	0	0	Fall
10/8/2019	BL	9A	10	2019	Year-2	0	0	Fall
10/8/2019	BL	11A	10	2019	Year-2	0	0	Fall
10/8/2019	BL	13A	10	2019	Year-2	0	0	Fall
10/9/2019	BL	1M	10	2019	Year-2	0	0	Fall
10/9/2019	BL	2M	10	2019	Year-2	0	0	Fall
10/9/2019	BL	3M	10	2019	Year-2	0	0	Fall
10/9/2019	BL	4M	10	2019	Year-2	0	0	Fall
10/9/2019	BL	5M	10	2019	Year-2	0	0	Fall
10/9/2019	BL	6M	10	2019	Year-2	0	0	Fall
10/9/2019	BL	7M	10	2019	Year-2	0	0	Fall
10/9/2019	BL	10M	10	2019	Year-2	0	0	Fall
10/9/2019	BL	12M	10	2019	Year-2	0	0	Fall
10/9/2019	BL	14M	10	2019	Year-2		1	Fall
10/9/2019	BL	8A	10	2019	Year-2	0	0	Fall
10/9/2019	BL	9A	10	2019	Year-2	0	0	Fall
10/9/2019	BL	11A	10	2019	Year-2	0	0	Fall
10/9/2019	BL	13A	10	2019	Year-2	0	0	Fall
10/10/2019	BL	1M	10	2019	Year-2	0	0	Fall
10/10/2019	BL	2M	10	2019	Year-2	0	0	Fall
10/10/2019	BL	3M	10	2019	Year-2	0	0	Fall
10/10/2019	BL	4M	10	2019	Year-2	0	0	Fall
10/10/2019	BL	5M	10	2019	Year-2	0	0	Fall
10/10/2019	BL	6M	10	2019	Year-2	0	0	Fall
10/10/2019	BL	7M	10	2019	Year-2	0	0	Fall
10/10/2019	BL	10M	10	2019	Year-2	0	0	Fall
10/10/2019	BL	12M	10	2019	Year-2	0	0	Fall
10/10/2019	BL	14M	10	2019	Year-2		1	Fall
10/10/2019	BL	8A	10	2019	Year-2	0	0	Fall
10/10/2019	BL	9A	10	2019	Year-2	0	0	Fall
10/10/2019	BL	11A	10	2019	Year-2	0	0	Fall
10/10/2019	BL	13A	10	2019	Year-2	0	0	Fall
10/11/2019	BL	1M	10	2019	Year-2	0	0	Fall
10/11/2019	BL	2M	10	2019	Year-2	0	0	Fall
10/11/2019	BL	3M	10	2019	Year-2	0	0	Fall
10/11/2019	BL	4M	10	2019	Year-2	0	0	Fall
10/11/2019	BL	5M	10	2019	Year-2	0	0	Fall
10/11/2019	BL	6M	10	2019	Year-2	0	0	Fall
10/11/2019	BL	7M	10	2019	Year-2	0	0	Fall

Date	Species	Site	Month	Year	Survey Year	Presence	Data Gap	Season
10/11/2019	BL	10M	10	2019	Year-2	0	0	Fall
10/11/2019	BL	12M	10	2019	Year-2	0	0	Fall
10/11/2019	BL	14M	10	2019	Year-2		1	Fall
10/11/2019	BL	8A	10	2019	Year-2	0	0	Fall
10/11/2019	BL	9A	10	2019	Year-2	0	0	Fall
10/11/2019	BL	11A	10	2019	Year-2	0	0	Fall
10/11/2019	BL	13A	10	2019	Year-2	0	0	Fall
10/12/2019	BL	1M	10	2019	Year-2	0	0	Fall
10/12/2019	BL	2M	10	2019	Year-2	0	0	Fall
10/12/2019	BL	3M	10	2019	Year-2	0	0	Fall
10/12/2019	BL	4M	10	2019	Year-2	0	0	Fall
10/12/2019	BL	5M	10	2019	Year-2	0	0	Fall
10/12/2019	BL	6M	10	2019	Year-2	0	0	Fall
10/12/2019	BL	7M	10	2019	Year-2	0	0	Fall
10/12/2019	BL	10M	10	2019	Year-2	0	0	Fall
10/12/2019	BL	12M	10	2019	Year-2	0	0	Fall
10/12/2019	BL	14M	10	2019	Year-2		1	Fall
10/12/2019	BL	8A	10	2019	Year-2	0	0	Fall
10/12/2019	BL	9A	10	2019	Year-2	0	0	Fall
10/12/2019	BL	11A	10	2019	Year-2	0	0	Fall
10/12/2019	BL	13A	10	2019	Year-2	0	0	Fall
10/13/2019	BL	1M	10	2019	Year-2	0	0	Fall
10/13/2019	BL	2M	10	2019	Year-2	0	0	Fall
10/13/2019	BL	3M	10	2019	Year-2	0	0	Fall
10/13/2019	BL	4M	10	2019	Year-2	0	0	Fall
10/13/2019	BL	5M	10	2019	Year-2	0	0	Fall
10/13/2019	BL	6M	10	2019	Year-2	0	0	Fall
10/13/2019	BL	7M	10	2019	Year-2	0	0	Fall
10/13/2019	BL	10M	10	2019	Year-2	0	0	Fall
10/13/2019	BL	12M	10	2019	Year-2	0	0	Fall
10/13/2019	BL	14M	10	2019	Year-2		1	Fall
10/13/2019	BL	8A	10	2019	Year-2	0	0	Fall
10/13/2019	BL	9A	10	2019	Year-2	0	0	Fall
10/13/2019	BL	11A	10	2019	Year-2	0	0	Fall
10/13/2019	BL	13A	10	2019	Year-2	0	0	Fall
10/14/2019	BL	1M	10	2019	Year-2	0	0	Fall
10/14/2019	BL	2M	10	2019	Year-2	0	0	Fall
10/14/2019	BL	3M	10	2019	Year-2	0	0	Fall
10/14/2019	BL	4M	10	2019	Year-2	0	0	Fall
10/14/2019	BL	5M	10	2019	Year-2	0	0	Fall
10/14/2019	BL	6M	10	2019	Year-2	0	0	Fall
10/14/2019	BL	7M	10	2019	Year-2	0	0	Fall
10/14/2019	BL	10M	10	2019	Year-2	0	0	Fall
10/14/2019	BL	12M	10	2019	Year-2	0	0	Fall

Date	Species	Site	Month	Year	Survey Year	Presence	Data Gap	Season
10/14/2019	BL	14M	10	2019	Year-2		1	Fall
10/14/2019	BL	8A	10	2019	Year-2	0	0	Fall
10/14/2019	BL	9A	10	2019	Year-2	0	0	Fall
10/14/2019	BL	11A	10	2019	Year-2	0	0	Fall
10/14/2019	BL	13A	10	2019	Year-2	0	0	Fall
10/15/2019	BL	1M	10	2019	Year-2	0	0	Fall
10/15/2019	BL	2M	10	2019	Year-2	0	0	Fall
10/15/2019	BL	3M	10	2019	Year-2	0	0	Fall
10/15/2019	BL	4M	10	2019	Year-2	0	0	Fall
10/15/2019	BL	5M	10	2019	Year-2	0	0	Fall
10/15/2019	BL	6M	10	2019	Year-2	0	0	Fall
10/15/2019	BL	7M	10	2019	Year-2	0	0	Fall
10/15/2019	BL	10M	10	2019	Year-2	0	0	Fall
10/15/2019	BL	12M	10	2019	Year-2	0	0	Fall
10/15/2019	BL	14M	10	2019	Year-2		1	Fall
10/15/2019	BL	8A	10	2019	Year-2	0	0	Fall
10/15/2019	BL	9A	10	2019	Year-2	0	0	Fall
10/15/2019	BL	11A	10	2019	Year-2	0	0	Fall
10/15/2019	BL	13A	10	2019	Year-2	0	0	Fall
10/16/2019	BL	1M	10	2019	Year-3	0	0	Fall
10/16/2019	BL	2M	10	2019	Year-3	0	0	Fall
10/16/2019	BL	3M	10	2019	Year-3	0	0	Fall
10/16/2019	BL	4M	10	2019	Year-3	0	0	Fall
10/16/2019	BL	5M	10	2019	Year-3	0	0	Fall
10/16/2019	BL	6M	10	2019	Year-3	0	0	Fall
10/16/2019	BL	7M	10	2019	Year-3	0	0	Fall
10/16/2019	BL	10M	10	2019	Year-3	0	0	Fall
10/16/2019	BL	12M	10	2019	Year-3	0	0	Fall
10/16/2019	BL	14M	10	2019	Year-3		1	Fall
10/16/2019	BL	8A	10	2019	Year-3	0	0	Fall
10/16/2019	BL	9A	10	2019	Year-3	0	0	Fall
10/16/2019	BL	11A	10	2019	Year-3	0	0	Fall
10/16/2019	BL	13A	10	2019	Year-3	0	0	Fall
10/17/2019	BL	1M	10	2019	Year-3	0	0	Fall
10/17/2019	BL	2M	10	2019	Year-3	0	0	Fall
10/17/2019	BL	3M	10	2019	Year-3	0	0	Fall
10/17/2019	BL	4M	10	2019	Year-3	0	0	Fall
10/17/2019	BL	5M	10	2019	Year-3	0	0	Fall
10/17/2019	BL	6M	10	2019	Year-3	0	0	Fall
10/17/2019	BL	7M	10	2019	Year-3	0	0	Fall
10/17/2019	BL	10M	10	2019	Year-3	0	0	Fall
10/17/2019	BL	12M	10	2019	Year-3	0	0	Fall
10/17/2019	BL	14M	10	2019	Year-3		1	Fall
10/17/2019	BL	8A	10	2019	Year-3	0	0	Fall

Date	Species	Site	Month	Year	Survey Year	Presence	Data Gap	Season
10/17/2019	BL	9A	10	2019	Year-3	0	0	Fall
10/17/2019	BL	11A	10	2019	Year-3	0	0	Fall
10/17/2019	BL	13A	10	2019	Year-3	0	0	Fall
10/18/2019	BL	1M	10	2019	Year-3	0	0	Fall
10/18/2019	BL	2M	10	2019	Year-3	0	0	Fall
10/18/2019	BL	3M	10	2019	Year-3	0	0	Fall
10/18/2019	BL	4M	10	2019	Year-3	0	0	Fall
10/18/2019	BL	5M	10	2019	Year-3	0	0	Fall
10/18/2019	BL	6M	10	2019	Year-3	0	0	Fall
10/18/2019	BL	7M	10	2019	Year-3	0	0	Fall
10/18/2019	BL	10M	10	2019	Year-3	0	0	Fall
10/18/2019	BL	12M	10	2019	Year-3	0	0	Fall
10/18/2019	BL	14M	10	2019	Year-3		1	Fall
10/18/2019	BL	8A	10	2019	Year-3	0	0	Fall
10/18/2019	BL	9A	10	2019	Year-3	0	0	Fall
10/18/2019	BL	11A	10	2019	Year-3	0	0	Fall
10/18/2019	BL	13A	10	2019	Year-3	0	0	Fall
10/19/2019	BL	1M	10	2019	Year-3	0	0	Fall
10/19/2019	BL	2M	10	2019	Year-3	0	0	Fall
10/19/2019	BL	3M	10	2019	Year-3	0	0	Fall
10/19/2019	BL	4M	10	2019	Year-3	0	0	Fall
10/19/2019	BL	5M	10	2019	Year-3	0	0	Fall
10/19/2019	BL	6M	10	2019	Year-3	0	0	Fall
10/19/2019	BL	7M	10	2019	Year-3	0	0	Fall
10/19/2019	BL	10M	10	2019	Year-3	0	0	Fall
10/19/2019	BL	12M	10	2019	Year-3	0	0	Fall
10/19/2019	BL	14M	10	2019	Year-3		1	Fall
10/19/2019	BL	8A	10	2019	Year-3	0	0	Fall
10/19/2019	BL	9A	10	2019	Year-3	0	0	Fall
10/19/2019	BL	11A	10	2019	Year-3	0	0	Fall
10/19/2019	BL	13A	10	2019	Year-3	0	0	Fall
10/20/2019	BL	1M	10	2019	Year-3	0	0	Fall
10/20/2019	BL	2M	10	2019	Year-3	0	0	Fall
10/20/2019	BL	3M	10	2019	Year-3	0	0	Fall
10/20/2019	BL	4M	10	2019	Year-3	0	0	Fall
10/20/2019	BL	5M	10	2019	Year-3	0	0	Fall
10/20/2019	BL	6M	10	2019	Year-3	0	0	Fall
10/20/2019	BL	7M	10	2019	Year-3	0	0	Fall
10/20/2019	BL	10M	10	2019	Year-3		1	Fall
10/20/2019	BL	12M	10	2019	Year-3		1	Fall
10/20/2019	BL	14M	10	2019	Year-3		1	Fall
10/20/2019	BL	8A	10	2019	Year-3		1	Fall
10/20/2019	BL	9A	10	2019	Year-3		1	Fall
10/20/2019	BL	11A	10	2019	Year-3	0	0	Fall

Date	Species	Site	Month	Year	Survey Year	Presence	Data Gap	Season
10/20/2019	BL	13A	10	2019	Year-3		1	Fall
10/21/2019	BL	1M	10	2019	Year-3	0	0	Fall
10/21/2019	BL	2M	10	2019	Year-3	0	0	Fall
10/21/2019	BL	3M	10	2019	Year-3	0	0	Fall
10/21/2019	BL	4M	10	2019	Year-3	0	0	Fall
10/21/2019	BL	5M	10	2019	Year-3	0	0	Fall
10/21/2019	BL	6M	10	2019	Year-3	0	0	Fall
10/21/2019	BL	7M	10	2019	Year-3	0	0	Fall
10/21/2019	BL	10M	10	2019	Year-3	0	0	Fall
10/21/2019	BL	12M	10	2019	Year-3	0	0	Fall
10/21/2019	BL	14M	10	2019	Year-3	0	0	Fall
10/21/2019	BL	8A	10	2019	Year-3		1	Fall
10/21/2019	BL	9A	10	2019	Year-3		1	Fall
10/21/2019	BL	11A	10	2019	Year-3	0	0	Fall
10/21/2019	BL	13A	10	2019	Year-3		1	Fall
10/22/2019	BL	1M	10	2019	Year-3	0	0	Fall
10/22/2019	BL	2M	10	2019	Year-3	0	0	Fall
10/22/2019	BL	3M	10	2019	Year-3	0	0	Fall
10/22/2019	BL	4M	10	2019	Year-3	0	0	Fall
10/22/2019	BL	5M	10	2019	Year-3	0	0	Fall
10/22/2019	BL	6M	10	2019	Year-3	0	0	Fall
10/22/2019	BL	7M	10	2019	Year-3	0	0	Fall
10/22/2019	BL	10M	10	2019	Year-3	0	0	Fall
10/22/2019	BL	12M	10	2019	Year-3	0	0	Fall
10/22/2019	BL	14M	10	2019	Year-3	0	0	Fall
10/22/2019	BL	8A	10	2019	Year-3		1	Fall
10/22/2019	BL	9A	10	2019	Year-3		1	Fall
10/22/2019	BL	11A	10	2019	Year-3	0	0	Fall
10/22/2019	BL	13A	10	2019	Year-3		1	Fall
10/23/2019	BL	1M	10	2019	Year-3	0	0	Fall
10/23/2019	BL	2M	10	2019	Year-3	0	0	Fall
10/23/2019	BL	3M	10	2019	Year-3	0	0	Fall
10/23/2019	BL	4M	10	2019	Year-3	0	0	Fall
10/23/2019	BL	5M	10	2019	Year-3	0	0	Fall
10/23/2019	BL	6M	10	2019	Year-3	0	0	Fall
10/23/2019	BL	7M	10	2019	Year-3	0	0	Fall
10/23/2019	BL	10M	10	2019	Year-3	0	0	Fall
10/23/2019	BL	12M	10	2019	Year-3	0	0	Fall
10/23/2019	BL	14M	10	2019	Year-3	0	0	Fall
10/23/2019	BL	8A	10	2019	Year-3		1	Fall
10/23/2019	BL	9A	10	2019	Year-3		1	Fall
10/23/2019	BL	11A	10	2019	Year-3	0	0	Fall
10/23/2019	BL	13A	10	2019	Year-3		1	Fall
10/24/2019	BL	1M	10	2019	Year-3	0	0	Fall

Date	Species	Site	Month	Year	Survey Year	Presence	Data Gap	Season
10/24/2019	BL	2M	10	2019	Year-3	0	0	Fall
10/24/2019	BL	3M	10	2019	Year-3		1	Fall
10/24/2019	BL	4M	10	2019	Year-3		1	Fall
10/24/2019	BL	5M	10	2019	Year-3		1	Fall
10/24/2019	BL	6M	10	2019	Year-3		1	Fall
10/24/2019	BL	7M	10	2019	Year-3		1	Fall
10/24/2019	BL	10M	10	2019	Year-3	0	0	Fall
10/24/2019	BL	12M	10	2019	Year-3	0	0	Fall
10/24/2019	BL	14M	10	2019	Year-3	0	0	Fall
10/24/2019	BL	8A	10	2019	Year-3		1	Fall
10/24/2019	BL	9A	10	2019	Year-3		1	Fall
10/24/2019	BL	11A	10	2019	Year-3	0	0	Fall
10/24/2019	BL	13A	10	2019	Year-3		1	Fall
10/25/2019	BL	1M	10	2019	Year-3		1	Fall
10/25/2019	BL	2M	10	2019	Year-3		1	Fall
10/25/2019	BL	3M	10	2019	Year-3		1	Fall
10/25/2019	BL	4M	10	2019	Year-3	0	0	Fall
10/25/2019	BL	5M	10	2019	Year-3	0	0	Fall
10/25/2019	BL	6M	10	2019	Year-3	0	0	Fall
10/25/2019	BL	7M	10	2019	Year-3	0	0	Fall
10/25/2019	BL	10M	10	2019	Year-3	0	0	Fall
10/25/2019	BL	12M	10	2019	Year-3	0	0	Fall
10/25/2019	BL	14M	10	2019	Year-3	0	0	Fall
10/25/2019	BL	8A	10	2019	Year-3		1	Fall
10/25/2019	BL	9A	10	2019	Year-3		1	Fall
10/25/2019	BL	11A	10	2019	Year-3	0	0	Fall
10/25/2019	BL	13A	10	2019	Year-3		1	Fall
10/26/2019	BL	1M	10	2019	Year-3	0	0	Fall
10/26/2019	BL	2M	10	2019	Year-3	0	0	Fall
10/26/2019	BL	3M	10	2019	Year-3		1	Fall
10/26/2019	BL	4M	10	2019	Year-3	0	0	Fall
10/26/2019	BL	5M	10	2019	Year-3	0	0	Fall
10/26/2019	BL	6M	10	2019	Year-3	0	0	Fall
10/26/2019	BL	7M	10	2019	Year-3	0	0	Fall
10/26/2019	BL	10M	10	2019	Year-3	0	0	Fall
10/26/2019	BL	12M	10	2019	Year-3	0	0	Fall
10/26/2019	BL	14M	10	2019	Year-3	0	0	Fall
10/26/2019	BL	8A	10	2019	Year-3		1	Fall
10/26/2019	BL	9A	10	2019	Year-3		1	Fall
10/26/2019	BL	11A	10	2019	Year-3	0	0	Fall
10/26/2019	BL	13A	10	2019	Year-3		1	Fall
10/27/2019	BL	1M	10	2019	Year-3	0	0	Fall
10/27/2019	BL	2M	10	2019	Year-3	0	0	Fall
10/27/2019	BL	3M	10	2019	Year-3		1	Fall

Date	Species	Site	Month	Year	Survey Year	Presence	Data Gap	Season
10/27/2019	BL	4M	10	2019	Year-3	0	0	Fall
10/27/2019	BL	5M	10	2019	Year-3	0	0	Fall
10/27/2019	BL	6M	10	2019	Year-3	0	0	Fall
10/27/2019	BL	7M	10	2019	Year-3	0	0	Fall
10/27/2019	BL	10M	10	2019	Year-3	0	0	Fall
10/27/2019	BL	12M	10	2019	Year-3	0	0	Fall
10/27/2019	BL	14M	10	2019	Year-3	0	0	Fall
10/27/2019	BL	8A	10	2019	Year-3		1	Fall
10/27/2019	BL	9A	10	2019	Year-3		1	Fall
10/27/2019	BL	11A	10	2019	Year-3	0	0	Fall
10/27/2019	BL	13A	10	2019	Year-3		1	Fall
10/28/2019	BL	1M	10	2019	Year-3	0	0	Fall
10/28/2019	BL	2M	10	2019	Year-3	0	0	Fall
10/28/2019	BL	3M	10	2019	Year-3		1	Fall
10/28/2019	BL	4M	10	2019	Year-3	0	0	Fall
10/28/2019	BL	5M	10	2019	Year-3	0	0	Fall
10/28/2019	BL	6M	10	2019	Year-3	0	0	Fall
10/28/2019	BL	7M	10	2019	Year-3	0	0	Fall
10/28/2019	BL	10M	10	2019	Year-3	0	0	Fall
10/28/2019	BL	12M	10	2019	Year-3	0	0	Fall
10/28/2019	BL	14M	10	2019	Year-3	0	0	Fall
10/28/2019	BL	8A	10	2019	Year-3		1	Fall
10/28/2019	BL	9A	10	2019	Year-3		1	Fall
10/28/2019	BL	11A	10	2019	Year-3	0	0	Fall
10/28/2019	BL	13A	10	2019	Year-3		1	Fall
10/29/2019	BL	1M	10	2019	Year-3	0	0	Fall
10/29/2019	BL	2M	10	2019	Year-3	0	0	Fall
10/29/2019	BL	3M	10	2019	Year-3		1	Fall
10/29/2019	BL	4M	10	2019	Year-3	0	0	Fall
10/29/2019	BL	5M	10	2019	Year-3	0	0	Fall
10/29/2019	BL	6M	10	2019	Year-3	0	0	Fall
10/29/2019	BL	7M	10	2019	Year-3	0	0	Fall
10/29/2019	BL	10M	10	2019	Year-3	0	0	Fall
10/29/2019	BL	12M	10	2019	Year-3	0	0	Fall
10/29/2019	BL	14M	10	2019	Year-3	0	0	Fall
10/29/2019	BL	8A	10	2019	Year-3		1	Fall
10/29/2019	BL	9A	10	2019	Year-3		1	Fall
10/29/2019	BL	11A	10	2019	Year-3	0	0	Fall
10/29/2019	BL	13A	10	2019	Year-3		1	Fall
10/30/2019	BL	1M	10	2019	Year-3	0	0	Fall
10/30/2019	BL	2M	10	2019	Year-3	0	0	Fall
10/30/2019	BL	3M	10	2019	Year-3		1	Fall
10/30/2019	BL	4M	10	2019	Year-3	0	0	Fall
10/30/2019	BL	5M	10	2019	Year-3	0	0	Fall

Date	Species	Site	Month	Year	Survey Year	Presence	Data Gap	Season
10/30/2019	BL	6M	10	2019	Year-3	0	0	Fall
10/30/2019	BL	7M	10	2019	Year-3	0	0	Fall
10/30/2019	BL	10M	10	2019	Year-3	0	0	Fall
10/30/2019	BL	12M	10	2019	Year-3	0	0	Fall
10/30/2019	BL	14M	10	2019	Year-3	0	0	Fall
10/30/2019	BL	8A	10	2019	Year-3		1	Fall
10/30/2019	BL	9A	10	2019	Year-3		1	Fall
10/30/2019	BL	11A	10	2019	Year-3	0	0	Fall
10/30/2019	BL	13A	10	2019	Year-3		1	Fall
10/31/2019	BL	1M	10	2019	Year-3	0	0	Fall
10/31/2019	BL	2M	10	2019	Year-3	0	0	Fall
10/31/2019	BL	3M	10	2019	Year-3		1	Fall
10/31/2019	BL	4M	10	2019	Year-3	0	0	Fall
10/31/2019	BL	5M	10	2019	Year-3	0	0	Fall
10/31/2019	BL	6M	10	2019	Year-3	0	0	Fall
10/31/2019	BL	7M	10	2019	Year-3	0	0	Fall
10/31/2019	BL	10M	10	2019	Year-3	0	0	Fall
10/31/2019	BL	12M	10	2019	Year-3	0	0	Fall
10/31/2019	BL	14M	10	2019	Year-3	0	0	Fall
10/31/2019	BL	8A	10	2019	Year-3		1	Fall
10/31/2019	BL	9A	10	2019	Year-3		1	Fall
10/31/2019	BL	11A	10	2019	Year-3	0	0	Fall
10/31/2019	BL	13A	10	2019	Year-3		1	Fall
11/1/2019	BL	1M	11	2019	Year-3	0	0	Fall
11/1/2019	BL	2M	11	2019	Year-3	0	0	Fall
11/1/2019	BL	3M	11	2019	Year-3		1	Fall
11/1/2019	BL	4M	11	2019	Year-3	0	0	Fall
11/1/2019	BL	5M	11	2019	Year-3	0	0	Fall
11/1/2019	BL	6M	11	2019	Year-3	0	0	Fall
11/1/2019	BL	7M	11	2019	Year-3	0	0	Fall
11/1/2019	BL	10M	11	2019	Year-3	0	0	Fall
11/1/2019	BL	12M	11	2019	Year-3	0	0	Fall
11/1/2019	BL	14M	11	2019	Year-3	0	0	Fall
11/1/2019	BL	8A	11	2019	Year-3		1	Fall
11/1/2019	BL	9A	11	2019	Year-3		1	Fall
11/1/2019	BL	11A	11	2019	Year-3	0	0	Fall
11/1/2019	BL	13A	11	2019	Year-3		1	Fall
11/2/2019	BL	1M	11	2019	Year-3	0	0	Fall
11/2/2019	BL	2M	11	2019	Year-3	0	0	Fall
11/2/2019	BL	3M	11	2019	Year-3		1	Fall
11/2/2019	BL	4M	11	2019	Year-3	0	0	Fall
11/2/2019	BL	5M	11	2019	Year-3	0	0	Fall
11/2/2019	BL	6M	11	2019	Year-3	0	0	Fall
11/2/2019	BL	7M	11	2019	Year-3	0	0	Fall

Date	Species	Site	Month	Year	Survey Year	Presence	Data Gap	Season
11/2/2019	BL	10M	11	2019	Year-3	0	0	Fall
11/2/2019	BL	12M	11	2019	Year-3	0	0	Fall
11/2/2019	BL	14M	11	2019	Year-3	0	0	Fall
11/2/2019	BL	8A	11	2019	Year-3		1	Fall
11/2/2019	BL	9A	11	2019	Year-3		1	Fall
11/2/2019	BL	11A	11	2019	Year-3	0	0	Fall
11/2/2019	BL	13A	11	2019	Year-3		1	Fall
11/3/2019	BL	1M	11	2019	Year-3	0	0	Fall
11/3/2019	BL	2M	11	2019	Year-3	0	0	Fall
11/3/2019	BL	3M	11	2019	Year-3		1	Fall
11/3/2019	BL	4M	11	2019	Year-3	0	0	Fall
11/3/2019	BL	5M	11	2019	Year-3	0	0	Fall
11/3/2019	BL	6M	11	2019	Year-3	0	0	Fall
11/3/2019	BL	7M	11	2019	Year-3	0	0	Fall
11/3/2019	BL	10M	11	2019	Year-3	0	0	Fall
11/3/2019	BL	12M	11	2019	Year-3	0	0	Fall
11/3/2019	BL	14M	11	2019	Year-3	0	0	Fall
11/3/2019	BL	8A	11	2019	Year-3		1	Fall
11/3/2019	BL	9A	11	2019	Year-3		1	Fall
11/3/2019	BL	11A	11	2019	Year-3	0	0	Fall
11/3/2019	BL	13A	11	2019	Year-3		1	Fall
11/4/2019	BL	1M	11	2019	Year-3	0	0	Fall
11/4/2019	BL	2M	11	2019	Year-3	0	0	Fall
11/4/2019	BL	3M	11	2019	Year-3		1	Fall
11/4/2019	BL	4M	11	2019	Year-3	0	0	Fall
11/4/2019	BL	5M	11	2019	Year-3	0	0	Fall
11/4/2019	BL	6M	11	2019	Year-3	0	0	Fall
11/4/2019	BL	7M	11	2019	Year-3	0	0	Fall
11/4/2019	BL	10M	11	2019	Year-3	0	0	Fall
11/4/2019	BL	12M	11	2019	Year-3	0	0	Fall
11/4/2019	BL	14M	11	2019	Year-3	0	0	Fall
11/4/2019	BL	8A	11	2019	Year-3		1	Fall
11/4/2019	BL	9A	11	2019	Year-3		1	Fall
11/4/2019	BL	11A	11	2019	Year-3	0	0	Fall
11/4/2019	BL	13A	11	2019	Year-3		1	Fall
11/5/2019	BL	1M	11	2019	Year-3	0	0	Fall
11/5/2019	BL	2M	11	2019	Year-3	0	0	Fall
11/5/2019	BL	3M	11	2019	Year-3		1	Fall
11/5/2019	BL	4M	11	2019	Year-3	0	0	Fall
11/5/2019	BL	5M	11	2019	Year-3	0	0	Fall
11/5/2019	BL	6M	11	2019	Year-3	0	0	Fall
11/5/2019	BL	7M	11	2019	Year-3	0	0	Fall
11/5/2019	BL	10M	11	2019	Year-3	0	0	Fall
11/5/2019	BL	12M	11	2019	Year-3	0	0	Fall

Date	Species	Site	Month	Year	Survey Year	Presence	Data Gap	Season
11/5/2019	BL	14M	11	2019	Year-3	0	0	Fall
11/5/2019	BL	8A	11	2019	Year-3		1	Fall
11/5/2019	BL	9A	11	2019	Year-3		1	Fall
11/5/2019	BL	11A	11	2019	Year-3	0	0	Fall
11/5/2019	BL	13A	11	2019	Year-3		1	Fall
11/6/2019	BL	1M	11	2019	Year-3	0	0	Fall
11/6/2019	BL	2M	11	2019	Year-3	0	0	Fall
11/6/2019	BL	3M	11	2019	Year-3		1	Fall
11/6/2019	BL	4M	11	2019	Year-3	0	0	Fall
11/6/2019	BL	5M	11	2019	Year-3	0	0	Fall
11/6/2019	BL	6M	11	2019	Year-3	0	0	Fall
11/6/2019	BL	7M	11	2019	Year-3	0	0	Fall
11/6/2019	BL	10M	11	2019	Year-3	0	0	Fall
11/6/2019	BL	12M	11	2019	Year-3	0	0	Fall
11/6/2019	BL	14M	11	2019	Year-3	0	0	Fall
11/6/2019	BL	8A	11	2019	Year-3		1	Fall
11/6/2019	BL	9A	11	2019	Year-3		1	Fall
11/6/2019	BL	11A	11	2019	Year-3	0	0	Fall
11/6/2019	BL	13A	11	2019	Year-3		1	Fall
11/7/2019	BL	1M	11	2019	Year-3	0	0	Fall
11/7/2019	BL	2M	11	2019	Year-3	0	0	Fall
11/7/2019	BL	3M	11	2019	Year-3		1	Fall
11/7/2019	BL	4M	11	2019	Year-3	0	0	Fall
11/7/2019	BL	5M	11	2019	Year-3	0	0	Fall
11/7/2019	BL	6M	11	2019	Year-3	0	0	Fall
11/7/2019	BL	7M	11	2019	Year-3	0	0	Fall
11/7/2019	BL	10M	11	2019	Year-3	0	0	Fall
11/7/2019	BL	12M	11	2019	Year-3	0	0	Fall
11/7/2019	BL	14M	11	2019	Year-3	0	0	Fall
11/7/2019	BL	8A	11	2019	Year-3		1	Fall
11/7/2019	BL	9A	11	2019	Year-3		1	Fall
11/7/2019	BL	11A	11	2019	Year-3	0	0	Fall
11/7/2019	BL	13A	11	2019	Year-3		1	Fall
11/8/2019	BL	1M	11	2019	Year-3	0	0	Fall
11/8/2019	BL	2M	11	2019	Year-3	0	0	Fall
11/8/2019	BL	3M	11	2019	Year-3		1	Fall
11/8/2019	BL	4M	11	2019	Year-3	0	0	Fall
11/8/2019	BL	5M	11	2019	Year-3	0	0	Fall
11/8/2019	BL	6M	11	2019	Year-3	0	0	Fall
11/8/2019	BL	7M	11	2019	Year-3	0	0	Fall
11/8/2019	BL	10M	11	2019	Year-3	0	0	Fall
11/8/2019	BL	12M	11	2019	Year-3	0	0	Fall
11/8/2019	BL	14M	11	2019	Year-3	0	0	Fall
11/8/2019	BL	8A	11	2019	Year-3		1	Fall

Date	Species	Site	Month	Year	Survey Year	Presence	Data Gap	Season
11/8/2019	BL	9A	11	2019	Year-3		1	Fall
11/8/2019	BL	11A	11	2019	Year-3	0	0	Fall
11/8/2019	BL	13A	11	2019	Year-3		1	Fall
11/9/2019	BL	1M	11	2019	Year-3	0	0	Fall
11/9/2019	BL	2M	11	2019	Year-3	0	0	Fall
11/9/2019	BL	3M	11	2019	Year-3		1	Fall
11/9/2019	BL	4M	11	2019	Year-3	0	0	Fall
11/9/2019	BL	5M	11	2019	Year-3	0	0	Fall
11/9/2019	BL	6M	11	2019	Year-3	0	0	Fall
11/9/2019	BL	7M	11	2019	Year-3	0	0	Fall
11/9/2019	BL	10M	11	2019	Year-3	0	0	Fall
11/9/2019	BL	12M	11	2019	Year-3	0	0	Fall
11/9/2019	BL	14M	11	2019	Year-3	0	0	Fall
11/9/2019	BL	8A	11	2019	Year-3		1	Fall
11/9/2019	BL	9A	11	2019	Year-3		1	Fall
11/9/2019	BL	11A	11	2019	Year-3	0	0	Fall
11/9/2019	BL	13A	11	2019	Year-3		1	Fall
11/10/2019	BL	1M	11	2019	Year-3	0	0	Fall
11/10/2019	BL	2M	11	2019	Year-3	0	0	Fall
11/10/2019	BL	3M	11	2019	Year-3		1	Fall
11/10/2019	BL	4M	11	2019	Year-3	0	0	Fall
11/10/2019	BL	5M	11	2019	Year-3	0	0	Fall
11/10/2019	BL	6M	11	2019	Year-3	0	0	Fall
11/10/2019	BL	7M	11	2019	Year-3	0	0	Fall
11/10/2019	BL	10M	11	2019	Year-3	0	0	Fall
11/10/2019	BL	12M	11	2019	Year-3	0	0	Fall
11/10/2019	BL	14M	11	2019	Year-3	0	0	Fall
11/10/2019	BL	8A	11	2019	Year-3		1	Fall
11/10/2019	BL	9A	11	2019	Year-3		1	Fall
11/10/2019	BL	11A	11	2019	Year-3	0	0	Fall
11/10/2019	BL	13A	11	2019	Year-3		1	Fall
11/11/2019	BL	1M	11	2019	Year-3	0	0	Fall
11/11/2019	BL	2M	11	2019	Year-3	0	0	Fall
11/11/2019	BL	3M	11	2019	Year-3		1	Fall
11/11/2019	BL	4M	11	2019	Year-3	0	0	Fall
11/11/2019	BL	5M	11	2019	Year-3	0	0	Fall
11/11/2019	BL	6M	11	2019	Year-3	0	0	Fall
11/11/2019	BL	7M	11	2019	Year-3	0	0	Fall
11/11/2019	BL	10M	11	2019	Year-3	0	0	Fall
11/11/2019	BL	12M	11	2019	Year-3	0	0	Fall
11/11/2019	BL	14M	11	2019	Year-3	0	0	Fall
11/11/2019	BL	8A	11	2019	Year-3		1	Fall
11/11/2019	BL	9A	11	2019	Year-3		1	Fall
11/11/2019	BL	11A	11	2019	Year-3	0	0	Fall

Date	Species	Site	Month	Year	Survey Year	Presence	Data Gap	Season
11/11/2019	BL	13A	11	2019	Year-3		1	Fall
11/12/2019	BL	1M	11	2019	Year-3	0	0	Fall
11/12/2019	BL	2M	11	2019	Year-3	0	0	Fall
11/12/2019	BL	3M	11	2019	Year-3		1	Fall
11/12/2019	BL	4M	11	2019	Year-3	0	0	Fall
11/12/2019	BL	5M	11	2019	Year-3	0	0	Fall
11/12/2019	BL	6M	11	2019	Year-3	0	0	Fall
11/12/2019	BL	7M	11	2019	Year-3	0	0	Fall
11/12/2019	BL	10M	11	2019	Year-3	0	0	Fall
11/12/2019	BL	12M	11	2019	Year-3	0	0	Fall
11/12/2019	BL	14M	11	2019	Year-3	0	0	Fall
11/12/2019	BL	8A	11	2019	Year-3		1	Fall
11/12/2019	BL	9A	11	2019	Year-3		1	Fall
11/12/2019	BL	11A	11	2019	Year-3	0	0	Fall
11/12/2019	BL	13A	11	2019	Year-3		1	Fall
11/13/2019	BL	1M	11	2019	Year-3	0	0	Fall
11/13/2019	BL	2M	11	2019	Year-3	0	0	Fall
11/13/2019	BL	3M	11	2019	Year-3		1	Fall
11/13/2019	BL	4M	11	2019	Year-3	0	0	Fall
11/13/2019	BL	5M	11	2019	Year-3	0	0	Fall
11/13/2019	BL	6M	11	2019	Year-3	0	0	Fall
11/13/2019	BL	7M	11	2019	Year-3	0	0	Fall
11/13/2019	BL	10M	11	2019	Year-3	0	0	Fall
11/13/2019	BL	12M	11	2019	Year-3	0	0	Fall
11/13/2019	BL	14M	11	2019	Year-3	0	0	Fall
11/13/2019	BL	8A	11	2019	Year-3		1	Fall
11/13/2019	BL	9A	11	2019	Year-3		1	Fall
11/13/2019	BL	11A	11	2019	Year-3	0	0	Fall
11/13/2019	BL	13A	11	2019	Year-3		1	Fall
11/14/2019	BL	1M	11	2019	Year-3	0	0	Fall
11/14/2019	BL	2M	11	2019	Year-3	0	0	Fall
11/14/2019	BL	3M	11	2019	Year-3		1	Fall
11/14/2019	BL	4M	11	2019	Year-3	0	0	Fall
11/14/2019	BL	5M	11	2019	Year-3	0	0	Fall
11/14/2019	BL	6M	11	2019	Year-3	0	0	Fall
11/14/2019	BL	7M	11	2019	Year-3	0	0	Fall
11/14/2019	BL	10M	11	2019	Year-3	0	0	Fall
11/14/2019	BL	12M	11	2019	Year-3	0	0	Fall
11/14/2019	BL	14M	11	2019	Year-3	0	0	Fall
11/14/2019	BL	8A	11	2019	Year-3		1	Fall
11/14/2019	BL	9A	11	2019	Year-3		1	Fall
11/14/2019	BL	11A	11	2019	Year-3	0	0	Fall
11/14/2019	BL	13A	11	2019	Year-3		1	Fall
11/15/2019	BL	1M	11	2019	Year-3	0	0	Fall

Date	Species	Site	Month	Year	Survey Year	Presence	Data Gap	Season
11/15/2019	BL	2M	11	2019	Year-3	0	0	Fall
11/15/2019	BL	3M	11	2019	Year-3		1	Fall
11/15/2019	BL	4M	11	2019	Year-3	0	0	Fall
11/15/2019	BL	5M	11	2019	Year-3	0	0	Fall
11/15/2019	BL	6M	11	2019	Year-3	0	0	Fall
11/15/2019	BL	7M	11	2019	Year-3	0	0	Fall
11/15/2019	BL	10M	11	2019	Year-3	0	0	Fall
11/15/2019	BL	12M	11	2019	Year-3	0	0	Fall
11/15/2019	BL	14M	11	2019	Year-3	0	0	Fall
11/15/2019	BL	8A	11	2019	Year-3		1	Fall
11/15/2019	BL	9A	11	2019	Year-3		1	Fall
11/15/2019	BL	11A	11	2019	Year-3	0	0	Fall
11/15/2019	BL	13A	11	2019	Year-3		1	Fall
11/16/2019	BL	1M	11	2019	Year-3	0	0	Fall
11/16/2019	BL	2M	11	2019	Year-3	0	0	Fall
11/16/2019	BL	3M	11	2019	Year-3		1	Fall
11/16/2019	BL	4M	11	2019	Year-3	0	0	Fall
11/16/2019	BL	5M	11	2019	Year-3	0	0	Fall
11/16/2019	BL	6M	11	2019	Year-3	0	0	Fall
11/16/2019	BL	7M	11	2019	Year-3	0	0	Fall
11/16/2019	BL	10M	11	2019	Year-3	0	0	Fall
11/16/2019	BL	12M	11	2019	Year-3	0	0	Fall
11/16/2019	BL	14M	11	2019	Year-3	0	0	Fall
11/16/2019	BL	8A	11	2019	Year-3		1	Fall
11/16/2019	BL	9A	11	2019	Year-3		1	Fall
11/16/2019	BL	11A	11	2019	Year-3	0	0	Fall
11/16/2019	BL	13A	11	2019	Year-3		1	Fall
11/17/2019	BL	1M	11	2019	Year-3	0	0	Fall
11/17/2019	BL	2M	11	2019	Year-3	0	0	Fall
11/17/2019	BL	3M	11	2019	Year-3		1	Fall
11/17/2019	BL	4M	11	2019	Year-3	0	0	Fall
11/17/2019	BL	5M	11	2019	Year-3	0	0	Fall
11/17/2019	BL	6M	11	2019	Year-3	0	0	Fall
11/17/2019	BL	7M	11	2019	Year-3	0	0	Fall
11/17/2019	BL	10M	11	2019	Year-3	0	0	Fall
11/17/2019	BL	12M	11	2019	Year-3	0	0	Fall
11/17/2019	BL	14M	11	2019	Year-3	0	0	Fall
11/17/2019	BL	8A	11	2019	Year-3		1	Fall
11/17/2019	BL	9A	11	2019	Year-3		1	Fall
11/17/2019	BL	11A	11	2019	Year-3	0	0	Fall
11/17/2019	BL	13A	11	2019	Year-3		1	Fall
11/18/2019	BL	1M	11	2019	Year-3	0	0	Fall
11/18/2019	BL	2M	11	2019	Year-3	0	0	Fall
11/18/2019	BL	3M	11	2019	Year-3		1	Fall

Date	Species	Site	Month	Year	Survey Year	Presence	Data Gap	Season
11/18/2019	BL	4M	11	2019	Year-3	0	0	Fall
11/18/2019	BL	5M	11	2019	Year-3	0	0	Fall
11/18/2019	BL	6M	11	2019	Year-3	0	0	Fall
11/18/2019	BL	7M	11	2019	Year-3	0	0	Fall
11/18/2019	BL	10M	11	2019	Year-3	0	0	Fall
11/18/2019	BL	12M	11	2019	Year-3	0	0	Fall
11/18/2019	BL	14M	11	2019	Year-3	0	0	Fall
11/18/2019	BL	8A	11	2019	Year-3		1	Fall
11/18/2019	BL	9A	11	2019	Year-3		1	Fall
11/18/2019	BL	11A	11	2019	Year-3	0	0	Fall
11/18/2019	BL	13A	11	2019	Year-3		1	Fall
11/19/2019	BL	1M	11	2019	Year-3	0	0	Fall
11/19/2019	BL	2M	11	2019	Year-3	0	0	Fall
11/19/2019	BL	3M	11	2019	Year-3		1	Fall
11/19/2019	BL	4M	11	2019	Year-3	0	0	Fall
11/19/2019	BL	5M	11	2019	Year-3	0	0	Fall
11/19/2019	BL	6M	11	2019	Year-3	0	0	Fall
11/19/2019	BL	7M	11	2019	Year-3	0	0	Fall
11/19/2019	BL	10M	11	2019	Year-3	0	0	Fall
11/19/2019	BL	12M	11	2019	Year-3	0	0	Fall
11/19/2019	BL	14M	11	2019	Year-3	0	0	Fall
11/19/2019	BL	8A	11	2019	Year-3		1	Fall
11/19/2019	BL	9A	11	2019	Year-3		1	Fall
11/19/2019	BL	11A	11	2019	Year-3	0	0	Fall
11/19/2019	BL	13A	11	2019	Year-3		1	Fall
11/20/2019	BL	1M	11	2019	Year-3	0	0	Fall
11/20/2019	BL	2M	11	2019	Year-3	0	0	Fall
11/20/2019	BL	3M	11	2019	Year-3		1	Fall
11/20/2019	BL	4M	11	2019	Year-3	0	0	Fall
11/20/2019	BL	5M	11	2019	Year-3	0	0	Fall
11/20/2019	BL	6M	11	2019	Year-3	0	0	Fall
11/20/2019	BL	7M	11	2019	Year-3	0	0	Fall
11/20/2019	BL	10M	11	2019	Year-3	0	0	Fall
11/20/2019	BL	12M	11	2019	Year-3	0	0	Fall
11/20/2019	BL	14M	11	2019	Year-3	0	0	Fall
11/20/2019	BL	8A	11	2019	Year-3		1	Fall
11/20/2019	BL	9A	11	2019	Year-3		1	Fall
11/20/2019	BL	11A	11	2019	Year-3	0	0	Fall
11/20/2019	BL	13A	11	2019	Year-3		1	Fall
11/21/2019	BL	1M	11	2019	Year-3	0	0	Fall
11/21/2019	BL	2M	11	2019	Year-3	0	0	Fall
11/21/2019	BL	3M	11	2019	Year-3		1	Fall
11/21/2019	BL	4M	11	2019	Year-3	0	0	Fall
11/21/2019	BL	5M	11	2019	Year-3	0	0	Fall

Date	Species	Site	Month	Year	Survey Year	Presence	Data Gap	Season
11/21/2019	BL	6M	11	2019	Year-3	0	0	Fall
11/21/2019	BL	7M	11	2019	Year-3	0	0	Fall
11/21/2019	BL	10M	11	2019	Year-3	0	0	Fall
11/21/2019	BL	12M	11	2019	Year-3	0	0	Fall
11/21/2019	BL	14M	11	2019	Year-3	0	0	Fall
11/21/2019	BL	8A	11	2019	Year-3		1	Fall
11/21/2019	BL	9A	11	2019	Year-3		1	Fall
11/21/2019	BL	11A	11	2019	Year-3	0	0	Fall
11/21/2019	BL	13A	11	2019	Year-3		1	Fall
11/22/2019	BL	1M	11	2019	Year-3	0	0	Fall
11/22/2019	BL	2M	11	2019	Year-3	0	0	Fall
11/22/2019	BL	3M	11	2019	Year-3		1	Fall
11/22/2019	BL	4M	11	2019	Year-3	0	0	Fall
11/22/2019	BL	5M	11	2019	Year-3	0	0	Fall
11/22/2019	BL	6M	11	2019	Year-3	0	0	Fall
11/22/2019	BL	7M	11	2019	Year-3	0	0	Fall
11/22/2019	BL	10M	11	2019	Year-3	0	0	Fall
11/22/2019	BL	12M	11	2019	Year-3	0	0	Fall
11/22/2019	BL	14M	11	2019	Year-3	0	0	Fall
11/22/2019	BL	8A	11	2019	Year-3		1	Fall
11/22/2019	BL	9A	11	2019	Year-3		1	Fall
11/22/2019	BL	11A	11	2019	Year-3	0	0	Fall
11/22/2019	BL	13A	11	2019	Year-3		1	Fall
11/23/2019	BL	1M	11	2019	Year-3	0	0	Fall
11/23/2019	BL	2M	11	2019	Year-3	0	0	Fall
11/23/2019	BL	3M	11	2019	Year-3		1	Fall
11/23/2019	BL	4M	11	2019	Year-3	0	0	Fall
11/23/2019	BL	5M	11	2019	Year-3	0	0	Fall
11/23/2019	BL	6M	11	2019	Year-3	0	0	Fall
11/23/2019	BL	7M	11	2019	Year-3	0	0	Fall
11/23/2019	BL	10M	11	2019	Year-3	0	0	Fall
11/23/2019	BL	12M	11	2019	Year-3	0	0	Fall
11/23/2019	BL	14M	11	2019	Year-3	0	0	Fall
11/23/2019	BL	8A	11	2019	Year-3		1	Fall
11/23/2019	BL	9A	11	2019	Year-3		1	Fall
11/23/2019	BL	11A	11	2019	Year-3	0	0	Fall
11/23/2019	BL	13A	11	2019	Year-3		1	Fall
11/24/2019	BL	1M	11	2019	Year-3	0	0	Fall
11/24/2019	BL	2M	11	2019	Year-3	0	0	Fall
11/24/2019	BL	3M	11	2019	Year-3		1	Fall
11/24/2019	BL	4M	11	2019	Year-3	0	0	Fall
11/24/2019	BL	5M	11	2019	Year-3	0	0	Fall
11/24/2019	BL	6M	11	2019	Year-3	0	0	Fall
11/24/2019	BL	7M	11	2019	Year-3	0	0	Fall

Date	Species	Site	Month	Year	Survey Year	Presence	Data Gap	Season
11/24/2019	BL	10M	11	2019	Year-3	0	0	Fall
11/24/2019	BL	12M	11	2019	Year-3	0	0	Fall
11/24/2019	BL	14M	11	2019	Year-3	0	0	Fall
11/24/2019	BL	8A	11	2019	Year-3		1	Fall
11/24/2019	BL	9A	11	2019	Year-3		1	Fall
11/24/2019	BL	11A	11	2019	Year-3	0	0	Fall
11/24/2019	BL	13A	11	2019	Year-3		1	Fall
11/25/2019	BL	1M	11	2019	Year-3	0	0	Fall
11/25/2019	BL	2M	11	2019	Year-3	0	0	Fall
11/25/2019	BL	3M	11	2019	Year-3		1	Fall
11/25/2019	BL	4M	11	2019	Year-3	0	0	Fall
11/25/2019	BL	5M	11	2019	Year-3	0	0	Fall
11/25/2019	BL	6M	11	2019	Year-3	0	0	Fall
11/25/2019	BL	7M	11	2019	Year-3	0	0	Fall
11/25/2019	BL	10M	11	2019	Year-3	0	0	Fall
11/25/2019	BL	12M	11	2019	Year-3	0	0	Fall
11/25/2019	BL	14M	11	2019	Year-3	0	0	Fall
11/25/2019	BL	8A	11	2019	Year-3		1	Fall
11/25/2019	BL	9A	11	2019	Year-3		1	Fall
11/25/2019	BL	11A	11	2019	Year-3	0	0	Fall
11/25/2019	BL	13A	11	2019	Year-3		1	Fall
11/26/2019	BL	1M	11	2019	Year-3	0	0	Fall
11/26/2019	BL	2M	11	2019	Year-3	0	0	Fall
11/26/2019	BL	3M	11	2019	Year-3		1	Fall
11/26/2019	BL	4M	11	2019	Year-3	0	0	Fall
11/26/2019	BL	5M	11	2019	Year-3	0	0	Fall
11/26/2019	BL	6M	11	2019	Year-3	0	0	Fall
11/26/2019	BL	7M	11	2019	Year-3	0	0	Fall
11/26/2019	BL	10M	11	2019	Year-3	0	0	Fall
11/26/2019	BL	12M	11	2019	Year-3	0	0	Fall
11/26/2019	BL	14M	11	2019	Year-3	0	0	Fall
11/26/2019	BL	8A	11	2019	Year-3		1	Fall
11/26/2019	BL	9A	11	2019	Year-3		1	Fall
11/26/2019	BL	11A	11	2019	Year-3	0	0	Fall
11/26/2019	BL	13A	11	2019	Year-3		1	Fall
11/27/2019	BL	1M	11	2019	Year-3	0	0	Fall
11/27/2019	BL	2M	11	2019	Year-3	0	0	Fall
11/27/2019	BL	3M	11	2019	Year-3		1	Fall
11/27/2019	BL	4M	11	2019	Year-3	0	0	Fall
11/27/2019	BL	5M	11	2019	Year-3	0	0	Fall
11/27/2019	BL	6M	11	2019	Year-3	0	0	Fall
11/27/2019	BL	7M	11	2019	Year-3	0	0	Fall
11/27/2019	BL	10M	11	2019	Year-3	0	0	Fall
11/27/2019	BL	12M	11	2019	Year-3	0	0	Fall

Date	Species	Site	Month	Year	Survey Year	Presence	Data Gap	Season
11/27/2019	BL	14M	11	2019	Year-3	0	0	Fall
11/27/2019	BL	8A	11	2019	Year-3		1	Fall
11/27/2019	BL	9A	11	2019	Year-3		1	Fall
11/27/2019	BL	11A	11	2019	Year-3	0	0	Fall
11/27/2019	BL	13A	11	2019	Year-3		1	Fall
11/28/2019	BL	1M	11	2019	Year-3	0	0	Fall
11/28/2019	BL	2M	11	2019	Year-3	0	0	Fall
11/28/2019	BL	3M	11	2019	Year-3		1	Fall
11/28/2019	BL	4M	11	2019	Year-3	0	0	Fall
11/28/2019	BL	5M	11	2019	Year-3	0	0	Fall
11/28/2019	BL	6M	11	2019	Year-3	0	0	Fall
11/28/2019	BL	7M	11	2019	Year-3	0	0	Fall
11/28/2019	BL	10M	11	2019	Year-3	0	0	Fall
11/28/2019	BL	12M	11	2019	Year-3	0	0	Fall
11/28/2019	BL	14M	11	2019	Year-3	0	0	Fall
11/28/2019	BL	8A	11	2019	Year-3		1	Fall
11/28/2019	BL	9A	11	2019	Year-3		1	Fall
11/28/2019	BL	11A	11	2019	Year-3	0	0	Fall
11/28/2019	BL	13A	11	2019	Year-3		1	Fall
11/29/2019	BL	1M	11	2019	Year-3	0	0	Fall
11/29/2019	BL	2M	11	2019	Year-3	0	0	Fall
11/29/2019	BL	3M	11	2019	Year-3		1	Fall
11/29/2019	BL	4M	11	2019	Year-3	0	0	Fall
11/29/2019	BL	5M	11	2019	Year-3	0	0	Fall
11/29/2019	BL	6M	11	2019	Year-3	0	0	Fall
11/29/2019	BL	7M	11	2019	Year-3	0	0	Fall
11/29/2019	BL	10M	11	2019	Year-3	0	0	Fall
11/29/2019	BL	12M	11	2019	Year-3	0	0	Fall
11/29/2019	BL	14M	11	2019	Year-3	0	0	Fall
11/29/2019	BL	8A	11	2019	Year-3		1	Fall
11/29/2019	BL	9A	11	2019	Year-3		1	Fall
11/29/2019	BL	11A	11	2019	Year-3	0	0	Fall
11/29/2019	BL	13A	11	2019	Year-3		1	Fall
11/30/2019	BL	1M	11	2019	Year-3	0	0	Fall
11/30/2019	BL	2M	11	2019	Year-3	0	0	Fall
11/30/2019	BL	3M	11	2019	Year-3		1	Fall
11/30/2019	BL	4M	11	2019	Year-3	0	0	Fall
11/30/2019	BL	5M	11	2019	Year-3	0	0	Fall
11/30/2019	BL	6M	11	2019	Year-3	0	0	Fall
11/30/2019	BL	7M	11	2019	Year-3	0	0	Fall
11/30/2019	BL	10M	11	2019	Year-3	0	0	Fall
11/30/2019	BL	12M	11	2019	Year-3	0	0	Fall
11/30/2019	BL	14M	11	2019	Year-3	0	0	Fall
11/30/2019	BL	8A	11	2019	Year-3		1	Fall

Date	Species	Site	Month	Year	Survey Year	Presence	Data Gap	Season
11/30/2019	BL	9A	11	2019	Year-3		1	Fall
11/30/2019	BL	11A	11	2019	Year-3	0	0	Fall
11/30/2019	BL	13A	11	2019	Year-3		1	Fall
12/1/2019	BL	1M	12	2019	Year-3	0	0	Winter
12/1/2019	BL	2M	12	2019	Year-3	0	0	Winter
12/1/2019	BL	3M	12	2019	Year-3		1	Winter
12/1/2019	BL	4M	12	2019	Year-3	0	0	Winter
12/1/2019	BL	5M	12	2019	Year-3	0	0	Winter
12/1/2019	BL	6M	12	2019	Year-3	0	0	Winter
12/1/2019	BL	7M	12	2019	Year-3	0	0	Winter
12/1/2019	BL	10M	12	2019	Year-3	0	0	Winter
12/1/2019	BL	12M	12	2019	Year-3	0	0	Winter
12/1/2019	BL	14M	12	2019	Year-3	0	0	Winter
12/1/2019	BL	8A	12	2019	Year-3		1	Winter
12/1/2019	BL	9A	12	2019	Year-3		1	Winter
12/1/2019	BL	11A	12	2019	Year-3	0	0	Winter
12/1/2019	BL	13A	12	2019	Year-3		1	Winter
12/2/2019	BL	1M	12	2019	Year-3	0	0	Winter
12/2/2019	BL	2M	12	2019	Year-3	0	0	Winter
12/2/2019	BL	3M	12	2019	Year-3		1	Winter
12/2/2019	BL	4M	12	2019	Year-3	0	0	Winter
12/2/2019	BL	5M	12	2019	Year-3	0	0	Winter
12/2/2019	BL	6M	12	2019	Year-3	0	0	Winter
12/2/2019	BL	7M	12	2019	Year-3	0	0	Winter
12/2/2019	BL	10M	12	2019	Year-3	0	0	Winter
12/2/2019	BL	12M	12	2019	Year-3	0	0	Winter
12/2/2019	BL	14M	12	2019	Year-3	0	0	Winter
12/2/2019	BL	8A	12	2019	Year-3		1	Winter
12/2/2019	BL	9A	12	2019	Year-3		1	Winter
12/2/2019	BL	11A	12	2019	Year-3	0	0	Winter
12/2/2019	BL	13A	12	2019	Year-3		1	Winter
12/3/2019	BL	1M	12	2019	Year-3	0	0	Winter
12/3/2019	BL	2M	12	2019	Year-3	0	0	Winter
12/3/2019	BL	3M	12	2019	Year-3		1	Winter
12/3/2019	BL	4M	12	2019	Year-3	0	0	Winter
12/3/2019	BL	5M	12	2019	Year-3	0	0	Winter
12/3/2019	BL	6M	12	2019	Year-3	0	0	Winter
12/3/2019	BL	7M	12	2019	Year-3	0	0	Winter
12/3/2019	BL	10M	12	2019	Year-3	0	0	Winter
12/3/2019	BL	12M	12	2019	Year-3	0	0	Winter
12/3/2019	BL	14M	12	2019	Year-3	0	0	Winter
12/3/2019	BL	8A	12	2019	Year-3		1	Winter
12/3/2019	BL	9A	12	2019	Year-3		1	Winter
12/3/2019	BL	11A	12	2019	Year-3	0	0	Winter

Date	Species	Site	Month	Year	Survey Year	Presence	Data Gap	Season
12/3/2019	BL	13A	12	2019	Year-3		1	Winter
12/4/2019	BL	1M	12	2019	Year-3	0	0	Winter
12/4/2019	BL	2M	12	2019	Year-3	0	0	Winter
12/4/2019	BL	3M	12	2019	Year-3		1	Winter
12/4/2019	BL	4M	12	2019	Year-3	0	0	Winter
12/4/2019	BL	5M	12	2019	Year-3	0	0	Winter
12/4/2019	BL	6M	12	2019	Year-3	0	0	Winter
12/4/2019	BL	7M	12	2019	Year-3	0	0	Winter
12/4/2019	BL	10M	12	2019	Year-3	0	0	Winter
12/4/2019	BL	12M	12	2019	Year-3	0	0	Winter
12/4/2019	BL	14M	12	2019	Year-3	0	0	Winter
12/4/2019	BL	8A	12	2019	Year-3		1	Winter
12/4/2019	BL	9A	12	2019	Year-3		1	Winter
12/4/2019	BL	11A	12	2019	Year-3	0	0	Winter
12/4/2019	BL	13A	12	2019	Year-3		1	Winter
12/5/2019	BL	1M	12	2019	Year-3	0	0	Winter
12/5/2019	BL	2M	12	2019	Year-3	0	0	Winter
12/5/2019	BL	3M	12	2019	Year-3		1	Winter
12/5/2019	BL	4M	12	2019	Year-3	0	0	Winter
12/5/2019	BL	5M	12	2019	Year-3	0	0	Winter
12/5/2019	BL	6M	12	2019	Year-3	0	0	Winter
12/5/2019	BL	7M	12	2019	Year-3	0	0	Winter
12/5/2019	BL	10M	12	2019	Year-3	0	0	Winter
12/5/2019	BL	12M	12	2019	Year-3	0	0	Winter
12/5/2019	BL	14M	12	2019	Year-3	0	0	Winter
12/5/2019	BL	8A	12	2019	Year-3		1	Winter
12/5/2019	BL	9A	12	2019	Year-3		1	Winter
12/5/2019	BL	11A	12	2019	Year-3	0	0	Winter
12/5/2019	BL	13A	12	2019	Year-3		1	Winter
12/6/2019	BL	1M	12	2019	Year-3	0	0	Winter
12/6/2019	BL	2M	12	2019	Year-3	0	0	Winter
12/6/2019	BL	3M	12	2019	Year-3		1	Winter
12/6/2019	BL	4M	12	2019	Year-3	0	0	Winter
12/6/2019	BL	5M	12	2019	Year-3	0	0	Winter
12/6/2019	BL	6M	12	2019	Year-3	0	0	Winter
12/6/2019	BL	7M	12	2019	Year-3	0	0	Winter
12/6/2019	BL	10M	12	2019	Year-3	0	0	Winter
12/6/2019	BL	12M	12	2019	Year-3	0	0	Winter
12/6/2019	BL	14M	12	2019	Year-3	0	0	Winter
12/6/2019	BL	8A	12	2019	Year-3		1	Winter
12/6/2019	BL	9A	12	2019	Year-3		1	Winter
12/6/2019	BL	11A	12	2019	Year-3	0	0	Winter
12/6/2019	BL	13A	12	2019	Year-3		1	Winter
12/7/2019	BL	1M	12	2019	Year-3	0	0	Winter

Date	Species	Site	Month	Year	Survey Year	Presence	Data Gap	Season
12/7/2019	BL	2M	12	2019	Year-3	0	0	Winter
12/7/2019	BL	3M	12	2019	Year-3		1	Winter
12/7/2019	BL	4M	12	2019	Year-3	0	0	Winter
12/7/2019	BL	5M	12	2019	Year-3	0	0	Winter
12/7/2019	BL	6M	12	2019	Year-3	0	0	Winter
12/7/2019	BL	7M	12	2019	Year-3	0	0	Winter
12/7/2019	BL	10M	12	2019	Year-3	0	0	Winter
12/7/2019	BL	12M	12	2019	Year-3	0	0	Winter
12/7/2019	BL	14M	12	2019	Year-3	0	0	Winter
12/7/2019	BL	8A	12	2019	Year-3		1	Winter
12/7/2019	BL	9A	12	2019	Year-3		1	Winter
12/7/2019	BL	11A	12	2019	Year-3	0	0	Winter
12/7/2019	BL	13A	12	2019	Year-3		1	Winter
12/8/2019	BL	1M	12	2019	Year-3	0	0	Winter
12/8/2019	BL	2M	12	2019	Year-3	0	0	Winter
12/8/2019	BL	3M	12	2019	Year-3		1	Winter
12/8/2019	BL	4M	12	2019	Year-3	0	0	Winter
12/8/2019	BL	5M	12	2019	Year-3	0	0	Winter
12/8/2019	BL	6M	12	2019	Year-3	0	0	Winter
12/8/2019	BL	7M	12	2019	Year-3	0	0	Winter
12/8/2019	BL	10M	12	2019	Year-3	0	0	Winter
12/8/2019	BL	12M	12	2019	Year-3	0	0	Winter
12/8/2019	BL	14M	12	2019	Year-3	0	0	Winter
12/8/2019	BL	8A	12	2019	Year-3		1	Winter
12/8/2019	BL	9A	12	2019	Year-3		1	Winter
12/8/2019	BL	11A	12	2019	Year-3	0	0	Winter
12/8/2019	BL	13A	12	2019	Year-3		1	Winter
12/9/2019	BL	1M	12	2019	Year-3	0	0	Winter
12/9/2019	BL	2M	12	2019	Year-3	0	0	Winter
12/9/2019	BL	3M	12	2019	Year-3		1	Winter
12/9/2019	BL	4M	12	2019	Year-3	0	0	Winter
12/9/2019	BL	5M	12	2019	Year-3	0	0	Winter
12/9/2019	BL	6M	12	2019	Year-3	0	0	Winter
12/9/2019	BL	7M	12	2019	Year-3	0	0	Winter
12/9/2019	BL	10M	12	2019	Year-3	0	0	Winter
12/9/2019	BL	12M	12	2019	Year-3	0	0	Winter
12/9/2019	BL	14M	12	2019	Year-3	0	0	Winter
12/9/2019	BL	8A	12	2019	Year-3		1	Winter
12/9/2019	BL	9A	12	2019	Year-3		1	Winter
12/9/2019	BL	11A	12	2019	Year-3	0	0	Winter
12/9/2019	BL	13A	12	2019	Year-3		1	Winter
12/10/2019	BL	1M	12	2019	Year-3	0	0	Winter
12/10/2019	BL	2M	12	2019	Year-3	0	0	Winter
12/10/2019	BL	3M	12	2019	Year-3		1	Winter

Date	Species	Site	Month	Year	Survey Year	Presence	Data Gap	Season
12/10/2019	BL	4M	12	2019	Year-3	0	0	Winter
12/10/2019	BL	5M	12	2019	Year-3	0	0	Winter
12/10/2019	BL	6M	12	2019	Year-3	0	0	Winter
12/10/2019	BL	7M	12	2019	Year-3	0	0	Winter
12/10/2019	BL	10M	12	2019	Year-3	0	0	Winter
12/10/2019	BL	12M	12	2019	Year-3	0	0	Winter
12/10/2019	BL	14M	12	2019	Year-3	0	0	Winter
12/10/2019	BL	8A	12	2019	Year-3		1	Winter
12/10/2019	BL	9A	12	2019	Year-3		1	Winter
12/10/2019	BL	11A	12	2019	Year-3	0	0	Winter
12/10/2019	BL	13A	12	2019	Year-3		1	Winter
12/11/2019	BL	1M	12	2019	Year-3	0	0	Winter
12/11/2019	BL	2M	12	2019	Year-3	0	0	Winter
12/11/2019	BL	3M	12	2019	Year-3		1	Winter
12/11/2019	BL	4M	12	2019	Year-3	0	0	Winter
12/11/2019	BL	5M	12	2019	Year-3	0	0	Winter
12/11/2019	BL	6M	12	2019	Year-3	0	0	Winter
12/11/2019	BL	7M	12	2019	Year-3	0	0	Winter
12/11/2019	BL	10M	12	2019	Year-3	0	0	Winter
12/11/2019	BL	12M	12	2019	Year-3	0	0	Winter
12/11/2019	BL	14M	12	2019	Year-3	0	0	Winter
12/11/2019	BL	8A	12	2019	Year-3		1	Winter
12/11/2019	BL	9A	12	2019	Year-3		1	Winter
12/11/2019	BL	11A	12	2019	Year-3	0	0	Winter
12/11/2019	BL	13A	12	2019	Year-3		1	Winter
12/12/2019	BL	1M	12	2019	Year-3	0	0	Winter
12/12/2019	BL	2M	12	2019	Year-3	0	0	Winter
12/12/2019	BL	3M	12	2019	Year-3		1	Winter
12/12/2019	BL	4M	12	2019	Year-3	0	0	Winter
12/12/2019	BL	5M	12	2019	Year-3	0	0	Winter
12/12/2019	BL	6M	12	2019	Year-3	0	0	Winter
12/12/2019	BL	7M	12	2019	Year-3	0	0	Winter
12/12/2019	BL	10M	12	2019	Year-3	0	0	Winter
12/12/2019	BL	12M	12	2019	Year-3	0	0	Winter
12/12/2019	BL	14M	12	2019	Year-3	0	0	Winter
12/12/2019	BL	8A	12	2019	Year-3		1	Winter
12/12/2019	BL	9A	12	2019	Year-3		1	Winter
12/12/2019	BL	11A	12	2019	Year-3	0	0	Winter
12/12/2019	BL	13A	12	2019	Year-3		1	Winter
12/13/2019	BL	1M	12	2019	Year-3	0	0	Winter
12/13/2019	BL	2M	12	2019	Year-3	0	0	Winter
12/13/2019	BL	3M	12	2019	Year-3		1	Winter
12/13/2019	BL	4M	12	2019	Year-3	0	0	Winter
12/13/2019	BL	5M	12	2019	Year-3	0	0	Winter

Date	Species	Site	Month	Year	Survey Year	Presence	Data Gap	Season
12/13/2019	BL	6M	12	2019	Year-3	0	0	Winter
12/13/2019	BL	7M	12	2019	Year-3	0	0	Winter
12/13/2019	BL	10M	12	2019	Year-3	0	0	Winter
12/13/2019	BL	12M	12	2019	Year-3	0	0	Winter
12/13/2019	BL	14M	12	2019	Year-3	0	0	Winter
12/13/2019	BL	8A	12	2019	Year-3		1	Winter
12/13/2019	BL	9A	12	2019	Year-3		1	Winter
12/13/2019	BL	11A	12	2019	Year-3	0	0	Winter
12/13/2019	BL	13A	12	2019	Year-3		1	Winter
12/14/2019	BL	1M	12	2019	Year-3	0	0	Winter
12/14/2019	BL	2M	12	2019	Year-3	0	0	Winter
12/14/2019	BL	3M	12	2019	Year-3		1	Winter
12/14/2019	BL	4M	12	2019	Year-3	0	0	Winter
12/14/2019	BL	5M	12	2019	Year-3	0	0	Winter
12/14/2019	BL	6M	12	2019	Year-3	0	0	Winter
12/14/2019	BL	7M	12	2019	Year-3	0	0	Winter
12/14/2019	BL	10M	12	2019	Year-3	0	0	Winter
12/14/2019	BL	12M	12	2019	Year-3	0	0	Winter
12/14/2019	BL	14M	12	2019	Year-3	0	0	Winter
12/14/2019	BL	8A	12	2019	Year-3		1	Winter
12/14/2019	BL	9A	12	2019	Year-3		1	Winter
12/14/2019	BL	11A	12	2019	Year-3	0	0	Winter
12/14/2019	BL	13A	12	2019	Year-3		1	Winter
12/15/2019	BL	1M	12	2019	Year-3	0	0	Winter
12/15/2019	BL	2M	12	2019	Year-3	0	0	Winter
12/15/2019	BL	3M	12	2019	Year-3		1	Winter
12/15/2019	BL	4M	12	2019	Year-3	0	0	Winter
12/15/2019	BL	5M	12	2019	Year-3	0	0	Winter
12/15/2019	BL	6M	12	2019	Year-3	0	0	Winter
12/15/2019	BL	7M	12	2019	Year-3	0	0	Winter
12/15/2019	BL	10M	12	2019	Year-3	0	0	Winter
12/15/2019	BL	12M	12	2019	Year-3	0	0	Winter
12/15/2019	BL	14M	12	2019	Year-3	0	0	Winter
12/15/2019	BL	8A	12	2019	Year-3		1	Winter
12/15/2019	BL	9A	12	2019	Year-3		1	Winter
12/15/2019	BL	11A	12	2019	Year-3	0	0	Winter
12/15/2019	BL	13A	12	2019	Year-3		1	Winter
12/16/2019	BL	1M	12	2019	Year-3	0	0	Winter
12/16/2019	BL	2M	12	2019	Year-3	0	0	Winter
12/16/2019	BL	3M	12	2019	Year-3		1	Winter
12/16/2019	BL	4M	12	2019	Year-3	0	0	Winter
12/16/2019	BL	5M	12	2019	Year-3	0	0	Winter
12/16/2019	BL	6M	12	2019	Year-3	0	0	Winter
12/16/2019	BL	7M	12	2019	Year-3	0	0	Winter

Date	Species	Site	Month	Year	Survey Year	Presence	Data Gap	Season
12/16/2019	BL	10M	12	2019	Year-3	0	0	Winter
12/16/2019	BL	12M	12	2019	Year-3	0	0	Winter
12/16/2019	BL	14M	12	2019	Year-3	0	0	Winter
12/16/2019	BL	8A	12	2019	Year-3		1	Winter
12/16/2019	BL	9A	12	2019	Year-3		1	Winter
12/16/2019	BL	11A	12	2019	Year-3	0	0	Winter
12/16/2019	BL	13A	12	2019	Year-3		1	Winter
12/17/2019	BL	1M	12	2019	Year-3	0	0	Winter
12/17/2019	BL	2M	12	2019	Year-3	0	0	Winter
12/17/2019	BL	3M	12	2019	Year-3		1	Winter
12/17/2019	BL	4M	12	2019	Year-3	0	0	Winter
12/17/2019	BL	5M	12	2019	Year-3	0	0	Winter
12/17/2019	BL	6M	12	2019	Year-3	0	0	Winter
12/17/2019	BL	7M	12	2019	Year-3	0	0	Winter
12/17/2019	BL	10M	12	2019	Year-3	0	0	Winter
12/17/2019	BL	12M	12	2019	Year-3	0	0	Winter
12/17/2019	BL	14M	12	2019	Year-3	0	0	Winter
12/17/2019	BL	8A	12	2019	Year-3		1	Winter
12/17/2019	BL	9A	12	2019	Year-3		1	Winter
12/17/2019	BL	11A	12	2019	Year-3	0	0	Winter
12/17/2019	BL	13A	12	2019	Year-3		1	Winter
12/18/2019	BL	1M	12	2019	Year-3	0	0	Winter
12/18/2019	BL	2M	12	2019	Year-3	0	0	Winter
12/18/2019	BL	3M	12	2019	Year-3		1	Winter
12/18/2019	BL	4M	12	2019	Year-3	0	0	Winter
12/18/2019	BL	5M	12	2019	Year-3	0	0	Winter
12/18/2019	BL	6M	12	2019	Year-3	0	0	Winter
12/18/2019	BL	7M	12	2019	Year-3	0	0	Winter
12/18/2019	BL	10M	12	2019	Year-3	0	0	Winter
12/18/2019	BL	12M	12	2019	Year-3	0	0	Winter
12/18/2019	BL	14M	12	2019	Year-3	0	0	Winter
12/18/2019	BL	8A	12	2019	Year-3		1	Winter
12/18/2019	BL	9A	12	2019	Year-3		1	Winter
12/18/2019	BL	11A	12	2019	Year-3	0	0	Winter
12/18/2019	BL	13A	12	2019	Year-3		1	Winter
12/19/2019	BL	1M	12	2019	Year-3	0	0	Winter
12/19/2019	BL	2M	12	2019	Year-3	0	0	Winter
12/19/2019	BL	3M	12	2019	Year-3		1	Winter
12/19/2019	BL	4M	12	2019	Year-3	0	0	Winter
12/19/2019	BL	5M	12	2019	Year-3	0	0	Winter
12/19/2019	BL	6M	12	2019	Year-3	0	0	Winter
12/19/2019	BL	7M	12	2019	Year-3	0	0	Winter
12/19/2019	BL	10M	12	2019	Year-3	0	0	Winter
12/19/2019	BL	12M	12	2019	Year-3	0	0	Winter

Date	Species	Site	Month	Year	Survey Year	Presence	Data Gap	Season
12/19/2019	BL	14M	12	2019	Year-3	0	0	Winter
12/19/2019	BL	8A	12	2019	Year-3		1	Winter
12/19/2019	BL	9A	12	2019	Year-3		1	Winter
12/19/2019	BL	11A	12	2019	Year-3	0	0	Winter
12/19/2019	BL	13A	12	2019	Year-3		1	Winter
12/20/2019	BL	1M	12	2019	Year-3	0	0	Winter
12/20/2019	BL	2M	12	2019	Year-3	0	0	Winter
12/20/2019	BL	3M	12	2019	Year-3		1	Winter
12/20/2019	BL	4M	12	2019	Year-3	0	0	Winter
12/20/2019	BL	5M	12	2019	Year-3	0	0	Winter
12/20/2019	BL	6M	12	2019	Year-3	0	0	Winter
12/20/2019	BL	7M	12	2019	Year-3	0	0	Winter
12/20/2019	BL	10M	12	2019	Year-3	0	0	Winter
12/20/2019	BL	12M	12	2019	Year-3	0	0	Winter
12/20/2019	BL	14M	12	2019	Year-3	0	0	Winter
12/20/2019	BL	8A	12	2019	Year-3		1	Winter
12/20/2019	BL	9A	12	2019	Year-3		1	Winter
12/20/2019	BL	11A	12	2019	Year-3	0	0	Winter
12/20/2019	BL	13A	12	2019	Year-3		1	Winter
12/21/2019	BL	1M	12	2019	Year-3	0	0	Winter
12/21/2019	BL	2M	12	2019	Year-3	0	0	Winter
12/21/2019	BL	3M	12	2019	Year-3		1	Winter
12/21/2019	BL	4M	12	2019	Year-3	0	0	Winter
12/21/2019	BL	5M	12	2019	Year-3	0	0	Winter
12/21/2019	BL	6M	12	2019	Year-3	0	0	Winter
12/21/2019	BL	7M	12	2019	Year-3	0	0	Winter
12/21/2019	BL	10M	12	2019	Year-3	0	0	Winter
12/21/2019	BL	12M	12	2019	Year-3	0	0	Winter
12/21/2019	BL	14M	12	2019	Year-3	0	0	Winter
12/21/2019	BL	8A	12	2019	Year-3		1	Winter
12/21/2019	BL	9A	12	2019	Year-3		1	Winter
12/21/2019	BL	11A	12	2019	Year-3	0	0	Winter
12/21/2019	BL	13A	12	2019	Year-3		1	Winter
12/22/2019	BL	1M	12	2019	Year-3	0	0	Winter
12/22/2019	BL	2M	12	2019	Year-3	0	0	Winter
12/22/2019	BL	3M	12	2019	Year-3		1	Winter
12/22/2019	BL	4M	12	2019	Year-3	0	0	Winter
12/22/2019	BL	5M	12	2019	Year-3	0	0	Winter
12/22/2019	BL	6M	12	2019	Year-3	0	0	Winter
12/22/2019	BL	7M	12	2019	Year-3	0	0	Winter
12/22/2019	BL	10M	12	2019	Year-3	0	0	Winter
12/22/2019	BL	12M	12	2019	Year-3	0	0	Winter
12/22/2019	BL	14M	12	2019	Year-3	0	0	Winter
12/22/2019	BL	8A	12	2019	Year-3		1	Winter

Date	Species	Site	Month	Year	Survey Year	Presence	Data Gap	Season
12/22/2019	BL	9A	12	2019	Year-3		1	Winter
12/22/2019	BL	11A	12	2019	Year-3	0	0	Winter
12/22/2019	BL	13A	12	2019	Year-3		1	Winter
12/23/2019	BL	1M	12	2019	Year-3	0	0	Winter
12/23/2019	BL	2M	12	2019	Year-3	0	0	Winter
12/23/2019	BL	3M	12	2019	Year-3		1	Winter
12/23/2019	BL	4M	12	2019	Year-3	0	0	Winter
12/23/2019	BL	5M	12	2019	Year-3	0	0	Winter
12/23/2019	BL	6M	12	2019	Year-3	0	0	Winter
12/23/2019	BL	7M	12	2019	Year-3	0	0	Winter
12/23/2019	BL	10M	12	2019	Year-3	0	0	Winter
12/23/2019	BL	12M	12	2019	Year-3	0	0	Winter
12/23/2019	BL	14M	12	2019	Year-3	0	0	Winter
12/23/2019	BL	8A	12	2019	Year-3		1	Winter
12/23/2019	BL	9A	12	2019	Year-3		1	Winter
12/23/2019	BL	11A	12	2019	Year-3	0	0	Winter
12/23/2019	BL	13A	12	2019	Year-3		1	Winter
12/24/2019	BL	1M	12	2019	Year-3	0	0	Winter
12/24/2019	BL	2M	12	2019	Year-3	0	0	Winter
12/24/2019	BL	3M	12	2019	Year-3		1	Winter
12/24/2019	BL	4M	12	2019	Year-3	0	0	Winter
12/24/2019	BL	5M	12	2019	Year-3	0	0	Winter
12/24/2019	BL	6M	12	2019	Year-3	0	0	Winter
12/24/2019	BL	7M	12	2019	Year-3	0	0	Winter
12/24/2019	BL	10M	12	2019	Year-3	0	0	Winter
12/24/2019	BL	12M	12	2019	Year-3	0	0	Winter
12/24/2019	BL	14M	12	2019	Year-3	0	0	Winter
12/24/2019	BL	8A	12	2019	Year-3		1	Winter
12/24/2019	BL	9A	12	2019	Year-3		1	Winter
12/24/2019	BL	11A	12	2019	Year-3	0	0	Winter
12/24/2019	BL	13A	12	2019	Year-3		1	Winter
12/25/2019	BL	1M	12	2019	Year-3	0	0	Winter
12/25/2019	BL	2M	12	2019	Year-3	0	0	Winter
12/25/2019	BL	3M	12	2019	Year-3		1	Winter
12/25/2019	BL	4M	12	2019	Year-3	0	0	Winter
12/25/2019	BL	5M	12	2019	Year-3	0	0	Winter
12/25/2019	BL	6M	12	2019	Year-3	0	0	Winter
12/25/2019	BL	7M	12	2019	Year-3	0	0	Winter
12/25/2019	BL	10M	12	2019	Year-3	0	0	Winter
12/25/2019	BL	12M	12	2019	Year-3	0	0	Winter
12/25/2019	BL	14M	12	2019	Year-3	0	0	Winter
12/25/2019	BL	8A	12	2019	Year-3		1	Winter
12/25/2019	BL	9A	12	2019	Year-3		1	Winter
12/25/2019	BL	11A	12	2019	Year-3	0	0	Winter

Date	Species	Site	Month	Year	Survey Year	Presence	Data Gap	Season
12/25/2019	BL	13A	12	2019	Year-3		1	Winter
12/26/2019	BL	1M	12	2019	Year-3	0	0	Winter
12/26/2019	BL	2M	12	2019	Year-3	0	0	Winter
12/26/2019	BL	3M	12	2019	Year-3		1	Winter
12/26/2019	BL	4M	12	2019	Year-3	0	0	Winter
12/26/2019	BL	5M	12	2019	Year-3	0	0	Winter
12/26/2019	BL	6M	12	2019	Year-3	0	0	Winter
12/26/2019	BL	7M	12	2019	Year-3	0	0	Winter
12/26/2019	BL	10M	12	2019	Year-3	0	0	Winter
12/26/2019	BL	12M	12	2019	Year-3	0	0	Winter
12/26/2019	BL	14M	12	2019	Year-3	0	0	Winter
12/26/2019	BL	8A	12	2019	Year-3		1	Winter
12/26/2019	BL	9A	12	2019	Year-3		1	Winter
12/26/2019	BL	11A	12	2019	Year-3	0	0	Winter
12/26/2019	BL	13A	12	2019	Year-3		1	Winter
12/27/2019	BL	1M	12	2019	Year-3	0	0	Winter
12/27/2019	BL	2M	12	2019	Year-3	0	0	Winter
12/27/2019	BL	3M	12	2019	Year-3		1	Winter
12/27/2019	BL	4M	12	2019	Year-3	0	0	Winter
12/27/2019	BL	5M	12	2019	Year-3	0	0	Winter
12/27/2019	BL	6M	12	2019	Year-3	0	0	Winter
12/27/2019	BL	7M	12	2019	Year-3	0	0	Winter
12/27/2019	BL	10M	12	2019	Year-3	0	0	Winter
12/27/2019	BL	12M	12	2019	Year-3	0	0	Winter
12/27/2019	BL	14M	12	2019	Year-3	0	0	Winter
12/27/2019	BL	8A	12	2019	Year-3		1	Winter
12/27/2019	BL	9A	12	2019	Year-3		1	Winter
12/27/2019	BL	11A	12	2019	Year-3	0	0	Winter
12/27/2019	BL	13A	12	2019	Year-3		1	Winter
12/28/2019	BL	1M	12	2019	Year-3	0	0	Winter
12/28/2019	BL	2M	12	2019	Year-3	0	0	Winter
12/28/2019	BL	3M	12	2019	Year-3		1	Winter
12/28/2019	BL	4M	12	2019	Year-3	0	0	Winter
12/28/2019	BL	5M	12	2019	Year-3	0	0	Winter
12/28/2019	BL	6M	12	2019	Year-3	0	0	Winter
12/28/2019	BL	7M	12	2019	Year-3	0	0	Winter
12/28/2019	BL	10M	12	2019	Year-3	0	0	Winter
12/28/2019	BL	12M	12	2019	Year-3	0	0	Winter
12/28/2019	BL	14M	12	2019	Year-3	0	0	Winter
12/28/2019	BL	8A	12	2019	Year-3		1	Winter
12/28/2019	BL	9A	12	2019	Year-3		1	Winter
12/28/2019	BL	11A	12	2019	Year-3	0	0	Winter
12/28/2019	BL	13A	12	2019	Year-3		1	Winter
12/29/2019	BL	1M	12	2019	Year-3	0	0	Winter

Date	Species	Site	Month	Year	Survey Year	Presence	Data Gap	Season
12/29/2019	BL	2M	12	2019	Year-3	0	0	Winter
12/29/2019	BL	3M	12	2019	Year-3		1	Winter
12/29/2019	BL	4M	12	2019	Year-3	0	0	Winter
12/29/2019	BL	5M	12	2019	Year-3	0	0	Winter
12/29/2019	BL	6M	12	2019	Year-3	0	0	Winter
12/29/2019	BL	7M	12	2019	Year-3	0	0	Winter
12/29/2019	BL	10M	12	2019	Year-3	0	0	Winter
12/29/2019	BL	12M	12	2019	Year-3	0	0	Winter
12/29/2019	BL	14M	12	2019	Year-3	0	0	Winter
12/29/2019	BL	8A	12	2019	Year-3		1	Winter
12/29/2019	BL	9A	12	2019	Year-3		1	Winter
12/29/2019	BL	11A	12	2019	Year-3	0	0	Winter
12/29/2019	BL	13A	12	2019	Year-3		1	Winter
12/30/2019	BL	1M	12	2019	Year-3	0	0	Winter
12/30/2019	BL	2M	12	2019	Year-3	0	0	Winter
12/30/2019	BL	3M	12	2019	Year-3		1	Winter
12/30/2019	BL	4M	12	2019	Year-3	0	0	Winter
12/30/2019	BL	5M	12	2019	Year-3	0	0	Winter
12/30/2019	BL	6M	12	2019	Year-3	0	0	Winter
12/30/2019	BL	7M	12	2019	Year-3	0	0	Winter
12/30/2019	BL	10M	12	2019	Year-3	0	0	Winter
12/30/2019	BL	12M	12	2019	Year-3	0	0	Winter
12/30/2019	BL	14M	12	2019	Year-3	0	0	Winter
12/30/2019	BL	8A	12	2019	Year-3		1	Winter
12/30/2019	BL	9A	12	2019	Year-3		1	Winter
12/30/2019	BL	11A	12	2019	Year-3	0	0	Winter
12/30/2019	BL	13A	12	2019	Year-3		1	Winter
12/31/2019	BL	1M	12	2019	Year-3	0	0	Winter
12/31/2019	BL	2M	12	2019	Year-3	0	0	Winter
12/31/2019	BL	3M	12	2019	Year-3		1	Winter
12/31/2019	BL	4M	12	2019	Year-3	0	0	Winter
12/31/2019	BL	5M	12	2019	Year-3	0	0	Winter
12/31/2019	BL	6M	12	2019	Year-3	0	0	Winter
12/31/2019	BL	7M	12	2019	Year-3	0	0	Winter
12/31/2019	BL	10M	12	2019	Year-3	0	0	Winter
12/31/2019	BL	12M	12	2019	Year-3	0	0	Winter
12/31/2019	BL	14M	12	2019	Year-3	0	0	Winter
12/31/2019	BL	8A	12	2019	Year-3		1	Winter
12/31/2019	BL	9A	12	2019	Year-3		1	Winter
12/31/2019	BL	11A	12	2019	Year-3	0	0	Winter
12/31/2019	BL	13A	12	2019	Year-3		1	Winter
1/1/2020	BL	1M	1	2020	Year-3	0	0	Winter
1/1/2020	BL	2M	1	2020	Year-3	0	0	Winter
1/1/2020	BL	3M	1	2020	Year-3		1	Winter

Date	Species	Site	Month	Year	Survey Year	Presence	Data Gap	Season
1/1/2020	BL	4M	1	2020	Year-3	0	0	Winter
1/1/2020	BL	5M	1	2020	Year-3	0	0	Winter
1/1/2020	BL	6M	1	2020	Year-3	0	0	Winter
1/1/2020	BL	7M	1	2020	Year-3	0	0	Winter
1/1/2020	BL	10M	1	2020	Year-3	0	0	Winter
1/1/2020	BL	12M	1	2020	Year-3	0	0	Winter
1/1/2020	BL	14M	1	2020	Year-3	0	0	Winter
1/1/2020	BL	8A	1	2020	Year-3		1	Winter
1/1/2020	BL	9A	1	2020	Year-3		1	Winter
1/1/2020	BL	11A	1	2020	Year-3	0	0	Winter
1/1/2020	BL	13A	1	2020	Year-3		1	Winter
1/2/2020	BL	1M	1	2020	Year-3	0	0	Winter
1/2/2020	BL	2M	1	2020	Year-3	0	0	Winter
1/2/2020	BL	3M	1	2020	Year-3		1	Winter
1/2/2020	BL	4M	1	2020	Year-3	0	0	Winter
1/2/2020	BL	5M	1	2020	Year-3	0	0	Winter
1/2/2020	BL	6M	1	2020	Year-3	0	0	Winter
1/2/2020	BL	7M	1	2020	Year-3	0	0	Winter
1/2/2020	BL	10M	1	2020	Year-3	0	0	Winter
1/2/2020	BL	12M	1	2020	Year-3	0	0	Winter
1/2/2020	BL	14M	1	2020	Year-3	0	0	Winter
1/2/2020	BL	8A	1	2020	Year-3		1	Winter
1/2/2020	BL	9A	1	2020	Year-3		1	Winter
1/2/2020	BL	11A	1	2020	Year-3	0	0	Winter
1/2/2020	BL	13A	1	2020	Year-3		1	Winter
1/3/2020	BL	1M	1	2020	Year-3	0	0	Winter
1/3/2020	BL	2M	1	2020	Year-3	0	0	Winter
1/3/2020	BL	3M	1	2020	Year-3		1	Winter
1/3/2020	BL	4M	1	2020	Year-3	0	0	Winter
1/3/2020	BL	5M	1	2020	Year-3	0	0	Winter
1/3/2020	BL	6M	1	2020	Year-3	0	0	Winter
1/3/2020	BL	7M	1	2020	Year-3	0	0	Winter
1/3/2020	BL	10M	1	2020	Year-3	0	0	Winter
1/3/2020	BL	12M	1	2020	Year-3	0	0	Winter
1/3/2020	BL	14M	1	2020	Year-3	0	0	Winter
1/3/2020	BL	8A	1	2020	Year-3		1	Winter
1/3/2020	BL	9A	1	2020	Year-3		1	Winter
1/3/2020	BL	11A	1	2020	Year-3	0	0	Winter
1/3/2020	BL	13A	1	2020	Year-3		1	Winter
1/4/2020	BL	1M	1	2020	Year-3	0	0	Winter
1/4/2020	BL	2M	1	2020	Year-3	0	0	Winter
1/4/2020	BL	3M	1	2020	Year-3		1	Winter
1/4/2020	BL	4M	1	2020	Year-3	0	0	Winter
1/4/2020	BL	5M	1	2020	Year-3	0	0	Winter

Date	Species	Site	Month	Year	Survey Year	Presence	Data Gap	Season
1/4/2020	BL	6M	1	2020	Year-3	0	0	Winter
1/4/2020	BL	7M	1	2020	Year-3	0	0	Winter
1/4/2020	BL	10M	1	2020	Year-3	0	0	Winter
1/4/2020	BL	12M	1	2020	Year-3	0	0	Winter
1/4/2020	BL	14M	1	2020	Year-3	0	0	Winter
1/4/2020	BL	8A	1	2020	Year-3		1	Winter
1/4/2020	BL	9A	1	2020	Year-3		1	Winter
1/4/2020	BL	11A	1	2020	Year-3	0	0	Winter
1/4/2020	BL	13A	1	2020	Year-3		1	Winter
1/5/2020	BL	1M	1	2020	Year-3	0	0	Winter
1/5/2020	BL	2M	1	2020	Year-3	0	0	Winter
1/5/2020	BL	3M	1	2020	Year-3		1	Winter
1/5/2020	BL	4M	1	2020	Year-3	0	0	Winter
1/5/2020	BL	5M	1	2020	Year-3	0	0	Winter
1/5/2020	BL	6M	1	2020	Year-3	0	0	Winter
1/5/2020	BL	7M	1	2020	Year-3	0	0	Winter
1/5/2020	BL	10M	1	2020	Year-3	0	0	Winter
1/5/2020	BL	12M	1	2020	Year-3	0	0	Winter
1/5/2020	BL	14M	1	2020	Year-3	0	0	Winter
1/5/2020	BL	8A	1	2020	Year-3		1	Winter
1/5/2020	BL	9A	1	2020	Year-3		1	Winter
1/5/2020	BL	11A	1	2020	Year-3	0	0	Winter
1/5/2020	BL	13A	1	2020	Year-3		1	Winter
1/6/2020	BL	1M	1	2020	Year-3	0	0	Winter
1/6/2020	BL	2M	1	2020	Year-3	0	0	Winter
1/6/2020	BL	3M	1	2020	Year-3		1	Winter
1/6/2020	BL	4M	1	2020	Year-3	0	0	Winter
1/6/2020	BL	5M	1	2020	Year-3	0	0	Winter
1/6/2020	BL	6M	1	2020	Year-3	0	0	Winter
1/6/2020	BL	7M	1	2020	Year-3	0	0	Winter
1/6/2020	BL	10M	1	2020	Year-3	0	0	Winter
1/6/2020	BL	12M	1	2020	Year-3	0	0	Winter
1/6/2020	BL	14M	1	2020	Year-3	0	0	Winter
1/6/2020	BL	8A	1	2020	Year-3		1	Winter
1/6/2020	BL	9A	1	2020	Year-3		1	Winter
1/6/2020	BL	11A	1	2020	Year-3	0	0	Winter
1/6/2020	BL	13A	1	2020	Year-3		1	Winter
1/7/2020	BL	1M	1	2020	Year-3	0	0	Winter
1/7/2020	BL	2M	1	2020	Year-3	0	0	Winter
1/7/2020	BL	3M	1	2020	Year-3		1	Winter
1/7/2020	BL	4M	1	2020	Year-3	0	0	Winter
1/7/2020	BL	5M	1	2020	Year-3	0	0	Winter
1/7/2020	BL	6M	1	2020	Year-3	0	0	Winter
1/7/2020	BL	7M	1	2020	Year-3	0	0	Winter

Date	Species	Site	Month	Year	Survey Year	Presence	Data Gap	Season
1/7/2020	BL	10M	1	2020	Year-3	0	0	Winter
1/7/2020	BL	12M	1	2020	Year-3	0	0	Winter
1/7/2020	BL	14M	1	2020	Year-3	0	0	Winter
1/7/2020	BL	8A	1	2020	Year-3		1	Winter
1/7/2020	BL	9A	1	2020	Year-3		1	Winter
1/7/2020	BL	11A	1	2020	Year-3	0	0	Winter
1/7/2020	BL	13A	1	2020	Year-3		1	Winter
1/8/2020	BL	1M	1	2020	Year-3	0	0	Winter
1/8/2020	BL	2M	1	2020	Year-3	0	0	Winter
1/8/2020	BL	3M	1	2020	Year-3		1	Winter
1/8/2020	BL	4M	1	2020	Year-3	0	0	Winter
1/8/2020	BL	5M	1	2020	Year-3	0	0	Winter
1/8/2020	BL	6M	1	2020	Year-3	0	0	Winter
1/8/2020	BL	7M	1	2020	Year-3	0	0	Winter
1/8/2020	BL	10M	1	2020	Year-3	0	0	Winter
1/8/2020	BL	12M	1	2020	Year-3	0	0	Winter
1/8/2020	BL	14M	1	2020	Year-3	0	0	Winter
1/8/2020	BL	8A	1	2020	Year-3		1	Winter
1/8/2020	BL	9A	1	2020	Year-3		1	Winter
1/8/2020	BL	11A	1	2020	Year-3	0	0	Winter
1/8/2020	BL	13A	1	2020	Year-3		1	Winter
1/9/2020	BL	1M	1	2020	Year-3	0	0	Winter
1/9/2020	BL	2M	1	2020	Year-3	0	0	Winter
1/9/2020	BL	3M	1	2020	Year-3		1	Winter
1/9/2020	BL	4M	1	2020	Year-3	0	0	Winter
1/9/2020	BL	5M	1	2020	Year-3	0	0	Winter
1/9/2020	BL	6M	1	2020	Year-3	0	0	Winter
1/9/2020	BL	7M	1	2020	Year-3	0	0	Winter
1/9/2020	BL	10M	1	2020	Year-3	0	0	Winter
1/9/2020	BL	12M	1	2020	Year-3	0	0	Winter
1/9/2020	BL	14M	1	2020	Year-3	0	0	Winter
1/9/2020	BL	8A	1	2020	Year-3		1	Winter
1/9/2020	BL	9A	1	2020	Year-3		1	Winter
1/9/2020	BL	11A	1	2020	Year-3	0	0	Winter
1/9/2020	BL	13A	1	2020	Year-3		1	Winter
1/10/2020	BL	1M	1	2020	Year-3	0	0	Winter
1/10/2020	BL	2M	1	2020	Year-3	0	0	Winter
1/10/2020	BL	3M	1	2020	Year-3		1	Winter
1/10/2020	BL	4M	1	2020	Year-3	0	0	Winter
1/10/2020	BL	5M	1	2020	Year-3	0	0	Winter
1/10/2020	BL	6M	1	2020	Year-3	0	0	Winter
1/10/2020	BL	7M	1	2020	Year-3	0	0	Winter
1/10/2020	BL	10M	1	2020	Year-3	0	0	Winter
1/10/2020	BL	12M	1	2020	Year-3	0	0	Winter

Date	Species	Site	Month	Year	Survey Year	Presence	Data Gap	Season
1/10/2020	BL	14M	1	2020	Year-3	0	0	Winter
1/10/2020	BL	8A	1	2020	Year-3		1	Winter
1/10/2020	BL	9A	1	2020	Year-3		1	Winter
1/10/2020	BL	11A	1	2020	Year-3	0	0	Winter
1/10/2020	BL	13A	1	2020	Year-3		1	Winter
1/11/2020	BL	1M	1	2020	Year-3	0	0	Winter
1/11/2020	BL	2M	1	2020	Year-3	0	0	Winter
1/11/2020	BL	3M	1	2020	Year-3		1	Winter
1/11/2020	BL	4M	1	2020	Year-3	0	0	Winter
1/11/2020	BL	5M	1	2020	Year-3	0	0	Winter
1/11/2020	BL	6M	1	2020	Year-3	0	0	Winter
1/11/2020	BL	7M	1	2020	Year-3	0	0	Winter
1/11/2020	BL	10M	1	2020	Year-3	0	0	Winter
1/11/2020	BL	12M	1	2020	Year-3	0	0	Winter
1/11/2020	BL	14M	1	2020	Year-3	0	0	Winter
1/11/2020	BL	8A	1	2020	Year-3		1	Winter
1/11/2020	BL	9A	1	2020	Year-3		1	Winter
1/11/2020	BL	11A	1	2020	Year-3		1	Winter
1/11/2020	BL	13A	1	2020	Year-3		1	Winter
1/12/2020	BL	1M	1	2020	Year-3	0	0	Winter
1/12/2020	BL	2M	1	2020	Year-3	0	0	Winter
1/12/2020	BL	3M	1	2020	Year-3		1	Winter
1/12/2020	BL	4M	1	2020	Year-3	0	0	Winter
1/12/2020	BL	5M	1	2020	Year-3	0	0	Winter
1/12/2020	BL	6M	1	2020	Year-3	0	0	Winter
1/12/2020	BL	7M	1	2020	Year-3	0	0	Winter
1/12/2020	BL	10M	1	2020	Year-3	0	0	Winter
1/12/2020	BL	12M	1	2020	Year-3	0	0	Winter
1/12/2020	BL	14M	1	2020	Year-3	0	0	Winter
1/12/2020	BL	8A	1	2020	Year-3		1	Winter
1/12/2020	BL	9A	1	2020	Year-3		1	Winter
1/12/2020	BL	11A	1	2020	Year-3		1	Winter
1/12/2020	BL	13A	1	2020	Year-3		1	Winter
1/13/2020	BL	1M	1	2020	Year-3	0	0	Winter
1/13/2020	BL	2M	1	2020	Year-3	0	0	Winter
1/13/2020	BL	3M	1	2020	Year-3		1	Winter
1/13/2020	BL	4M	1	2020	Year-3	0	0	Winter
1/13/2020	BL	5M	1	2020	Year-3	0	0	Winter
1/13/2020	BL	6M	1	2020	Year-3	0	0	Winter
1/13/2020	BL	7M	1	2020	Year-3	0	0	Winter
1/13/2020	BL	10M	1	2020	Year-3	0	0	Winter
1/13/2020	BL	12M	1	2020	Year-3	0	0	Winter
1/13/2020	BL	14M	1	2020	Year-3	0	0	Winter
1/13/2020	BL	8A	1	2020	Year-3		1	Winter

Date	Species	Site	Month	Year	Survey Year	Presence	Data Gap	Season
1/13/2020	BL	9A	1	2020	Year-3		1	Winter
1/13/2020	BL	11A	1	2020	Year-3		1	Winter
1/13/2020	BL	13A	1	2020	Year-3		1	Winter
1/14/2020	BL	1M	1	2020	Year-3	0	0	Winter
1/14/2020	BL	2M	1	2020	Year-3	0	0	Winter
1/14/2020	BL	3M	1	2020	Year-3		1	Winter
1/14/2020	BL	4M	1	2020	Year-3	0	0	Winter
1/14/2020	BL	5M	1	2020	Year-3	0	0	Winter
1/14/2020	BL	6M	1	2020	Year-3	0	0	Winter
1/14/2020	BL	7M	1	2020	Year-3	0	0	Winter
1/14/2020	BL	10M	1	2020	Year-3	0	0	Winter
1/14/2020	BL	12M	1	2020	Year-3	0	0	Winter
1/14/2020	BL	14M	1	2020	Year-3	0	0	Winter
1/14/2020	BL	8A	1	2020	Year-3		1	Winter
1/14/2020	BL	9A	1	2020	Year-3		1	Winter
1/14/2020	BL	11A	1	2020	Year-3		1	Winter
1/14/2020	BL	13A	1	2020	Year-3		1	Winter
1/15/2020	BL	1M	1	2020	Year-3	0	0	Winter
1/15/2020	BL	2M	1	2020	Year-3	0	0	Winter
1/15/2020	BL	3M	1	2020	Year-3		1	Winter
1/15/2020	BL	4M	1	2020	Year-3	0	0	Winter
1/15/2020	BL	5M	1	2020	Year-3	0	0	Winter
1/15/2020	BL	6M	1	2020	Year-3	0	0	Winter
1/15/2020	BL	7M	1	2020	Year-3	0	0	Winter
1/15/2020	BL	10M	1	2020	Year-3	0	0	Winter
1/15/2020	BL	12M	1	2020	Year-3	0	0	Winter
1/15/2020	BL	14M	1	2020	Year-3	0	0	Winter
1/15/2020	BL	8A	1	2020	Year-3		1	Winter
1/15/2020	BL	9A	1	2020	Year-3		1	Winter
1/15/2020	BL	11A	1	2020	Year-3		1	Winter
1/15/2020	BL	13A	1	2020	Year-3		1	Winter
1/16/2020	BL	1M	1	2020	Year-3	0	0	Winter
1/16/2020	BL	2M	1	2020	Year-3	0	0	Winter
1/16/2020	BL	3M	1	2020	Year-3		1	Winter
1/16/2020	BL	4M	1	2020	Year-3	0	0	Winter
1/16/2020	BL	5M	1	2020	Year-3	0	0	Winter
1/16/2020	BL	6M	1	2020	Year-3	0	0	Winter
1/16/2020	BL	7M	1	2020	Year-3	0	0	Winter
1/16/2020	BL	10M	1	2020	Year-3	0	0	Winter
1/16/2020	BL	12M	1	2020	Year-3	0	0	Winter
1/16/2020	BL	14M	1	2020	Year-3	0	0	Winter
1/16/2020	BL	8A	1	2020	Year-3		1	Winter
1/16/2020	BL	9A	1	2020	Year-3		1	Winter
1/16/2020	BL	11A	1	2020	Year-3		1	Winter

Date	Species	Site	Month	Year	Survey Year	Presence	Data Gap	Season
1/16/2020	BL	13A	1	2020	Year-3		1	Winter
1/17/2020	BL	1M	1	2020	Year-3	0	0	Winter
1/17/2020	BL	2M	1	2020	Year-3	0	0	Winter
1/17/2020	BL	3M	1	2020	Year-3		1	Winter
1/17/2020	BL	4M	1	2020	Year-3	0	0	Winter
1/17/2020	BL	5M	1	2020	Year-3	0	0	Winter
1/17/2020	BL	6M	1	2020	Year-3	0	0	Winter
1/17/2020	BL	7M	1	2020	Year-3	0	0	Winter
1/17/2020	BL	10M	1	2020	Year-3	0	0	Winter
1/17/2020	BL	12M	1	2020	Year-3	0	0	Winter
1/17/2020	BL	14M	1	2020	Year-3	0	0	Winter
1/17/2020	BL	8A	1	2020	Year-3		1	Winter
1/17/2020	BL	9A	1	2020	Year-3		1	Winter
1/17/2020	BL	11A	1	2020	Year-3		1	Winter
1/17/2020	BL	13A	1	2020	Year-3		1	Winter
1/18/2020	BL	1M	1	2020	Year-3	0	0	Winter
1/18/2020	BL	2M	1	2020	Year-3	0	0	Winter
1/18/2020	BL	3M	1	2020	Year-3		1	Winter
1/18/2020	BL	4M	1	2020	Year-3	0	0	Winter
1/18/2020	BL	5M	1	2020	Year-3	0	0	Winter
1/18/2020	BL	6M	1	2020	Year-3	0	0	Winter
1/18/2020	BL	7M	1	2020	Year-3	0	0	Winter
1/18/2020	BL	10M	1	2020	Year-3	0	0	Winter
1/18/2020	BL	12M	1	2020	Year-3	0	0	Winter
1/18/2020	BL	14M	1	2020	Year-3	0	0	Winter
1/18/2020	BL	8A	1	2020	Year-3		1	Winter
1/18/2020	BL	9A	1	2020	Year-3		1	Winter
1/18/2020	BL	11A	1	2020	Year-3		1	Winter
1/18/2020	BL	13A	1	2020	Year-3		1	Winter
1/19/2020	BL	1M	1	2020	Year-3	0	0	Winter
1/19/2020	BL	2M	1	2020	Year-3	0	0	Winter
1/19/2020	BL	3M	1	2020	Year-3		1	Winter
1/19/2020	BL	4M	1	2020	Year-3	0	0	Winter
1/19/2020	BL	5M	1	2020	Year-3	0	0	Winter
1/19/2020	BL	6M	1	2020	Year-3	0	0	Winter
1/19/2020	BL	7M	1	2020	Year-3	0	0	Winter
1/19/2020	BL	10M	1	2020	Year-3	0	0	Winter
1/19/2020	BL	12M	1	2020	Year-3	0	0	Winter
1/19/2020	BL	14M	1	2020	Year-3	0	0	Winter
1/19/2020	BL	8A	1	2020	Year-3		1	Winter
1/19/2020	BL	9A	1	2020	Year-3		1	Winter
1/19/2020	BL	11A	1	2020	Year-3		1	Winter
1/19/2020	BL	13A	1	2020	Year-3		1	Winter
1/20/2020	BL	1M	1	2020	Year-3	0	0	Winter

Date	Species	Site	Month	Year	Survey Year	Presence	Data Gap	Season
1/20/2020	BL	2M	1	2020	Year-3	0	0	Winter
1/20/2020	BL	3M	1	2020	Year-3		1	Winter
1/20/2020	BL	4M	1	2020	Year-3	0	0	Winter
1/20/2020	BL	5M	1	2020	Year-3	0	0	Winter
1/20/2020	BL	6M	1	2020	Year-3	0	0	Winter
1/20/2020	BL	7M	1	2020	Year-3	0	0	Winter
1/20/2020	BL	10M	1	2020	Year-3	0	0	Winter
1/20/2020	BL	12M	1	2020	Year-3	0	0	Winter
1/20/2020	BL	14M	1	2020	Year-3	0	0	Winter
1/20/2020	BL	8A	1	2020	Year-3		1	Winter
1/20/2020	BL	9A	1	2020	Year-3		1	Winter
1/20/2020	BL	11A	1	2020	Year-3		1	Winter
1/20/2020	BL	13A	1	2020	Year-3		1	Winter
1/21/2020	BL	1M	1	2020	Year-3	0	0	Winter
1/21/2020	BL	2M	1	2020	Year-3	0	0	Winter
1/21/2020	BL	3M	1	2020	Year-3		1	Winter
1/21/2020	BL	4M	1	2020	Year-3	0	0	Winter
1/21/2020	BL	5M	1	2020	Year-3	0	0	Winter
1/21/2020	BL	6M	1	2020	Year-3	0	0	Winter
1/21/2020	BL	7M	1	2020	Year-3	0	0	Winter
1/21/2020	BL	10M	1	2020	Year-3		1	Winter
1/21/2020	BL	12M	1	2020	Year-3	0	0	Winter
1/21/2020	BL	14M	1	2020	Year-3	0	0	Winter
1/21/2020	BL	8A	1	2020	Year-3		1	Winter
1/21/2020	BL	9A	1	2020	Year-3		1	Winter
1/21/2020	BL	11A	1	2020	Year-3		1	Winter
1/21/2020	BL	13A	1	2020	Year-3		1	Winter
1/22/2020	BL	1M	1	2020	Year-3	0	0	Winter
1/22/2020	BL	2M	1	2020	Year-3	0	0	Winter
1/22/2020	BL	3M	1	2020	Year-3		1	Winter
1/22/2020	BL	4M	1	2020	Year-3		1	Winter
1/22/2020	BL	5M	1	2020	Year-3		1	Winter
1/22/2020	BL	6M	1	2020	Year-3		1	Winter
1/22/2020	BL	7M	1	2020	Year-3		1	Winter
1/22/2020	BL	10M	1	2020	Year-3	0	0	Winter
1/22/2020	BL	12M	1	2020	Year-3		1	Winter
1/22/2020	BL	14M	1	2020	Year-3		1	Winter
1/22/2020	BL	8A	1	2020	Year-3		1	Winter
1/22/2020	BL	9A	1	2020	Year-3		1	Winter
1/22/2020	BL	11A	1	2020	Year-3		1	Winter
1/22/2020	BL	13A	1	2020	Year-3		1	Winter
1/23/2020	BL	1M	1	2020	Year-3		1	Winter
1/23/2020	BL	2M	1	2020	Year-3		1	Winter
1/23/2020	BL	3M	1	2020	Year-3	0	0	Winter

Date	Species	Site	Month	Year	Survey Year	Presence	Data Gap	Season
1/23/2020	BL	4M	1	2020	Year-3	0	0	Winter
1/23/2020	BL	5M	1	2020	Year-3	0	0	Winter
1/23/2020	BL	6M	1	2020	Year-3	0	0	Winter
1/23/2020	BL	7M	1	2020	Year-3		1	Winter
1/23/2020	BL	10M	1	2020	Year-3	0	0	Winter
1/23/2020	BL	12M	1	2020	Year-3	0	0	Winter
1/23/2020	BL	14M	1	2020	Year-3	0	0	Winter
1/23/2020	BL	8A	1	2020	Year-3		1	Winter
1/23/2020	BL	9A	1	2020	Year-3		1	Winter
1/23/2020	BL	11A	1	2020	Year-3		1	Winter
1/23/2020	BL	13A	1	2020	Year-3		1	Winter
1/24/2020	BL	1M	1	2020	Year-3	0	0	Winter
1/24/2020	BL	2M	1	2020	Year-3	0	0	Winter
1/24/2020	BL	3M	1	2020	Year-3	0	0	Winter
1/24/2020	BL	4M	1	2020	Year-3	0	0	Winter
1/24/2020	BL	5M	1	2020	Year-3	0	0	Winter
1/24/2020	BL	6M	1	2020	Year-3	0	0	Winter
1/24/2020	BL	7M	1	2020	Year-3		1	Winter
1/24/2020	BL	10M	1	2020	Year-3	0	0	Winter
1/24/2020	BL	12M	1	2020	Year-3	0	0	Winter
1/24/2020	BL	14M	1	2020	Year-3	1	0	Winter
1/24/2020	BL	8A	1	2020	Year-3		1	Winter
1/24/2020	BL	9A	1	2020	Year-3		1	Winter
1/24/2020	BL	11A	1	2020	Year-3		1	Winter
1/24/2020	BL	13A	1	2020	Year-3		1	Winter
1/25/2020	BL	1M	1	2020	Year-3	1	0	Winter
1/25/2020	BL	2M	1	2020	Year-3	0	0	Winter
1/25/2020	BL	3M	1	2020	Year-3	0	0	Winter
1/25/2020	BL	4M	1	2020	Year-3	0	0	Winter
1/25/2020	BL	5M	1	2020	Year-3	0	0	Winter
1/25/2020	BL	6M	1	2020	Year-3	0	0	Winter
1/25/2020	BL	7M	1	2020	Year-3		1	Winter
1/25/2020	BL	10M	1	2020	Year-3	0	0	Winter
1/25/2020	BL	12M	1	2020	Year-3	0	0	Winter
1/25/2020	BL	14M	1	2020	Year-3	1	0	Winter
1/25/2020	BL	8A	1	2020	Year-3		1	Winter
1/25/2020	BL	9A	1	2020	Year-3		1	Winter
1/25/2020	BL	11A	1	2020	Year-3		1	Winter
1/25/2020	BL	13A	1	2020	Year-3		1	Winter
1/26/2020	BL	1M	1	2020	Year-3	1	0	Winter
1/26/2020	BL	2M	1	2020	Year-3	1	0	Winter
1/26/2020	BL	3M	1	2020	Year-3	0	0	Winter
1/26/2020	BL	4M	1	2020	Year-3	0	0	Winter
1/26/2020	BL	5M	1	2020	Year-3	0	0	Winter

Date	Species	Site	Month	Year	Survey Year	Presence	Data Gap	Season
1/26/2020	BL	6M	1	2020	Year-3	0	0	Winter
1/26/2020	BL	7M	1	2020	Year-3		1	Winter
1/26/2020	BL	10M	1	2020	Year-3	0	0	Winter
1/26/2020	BL	12M	1	2020	Year-3	0	0	Winter
1/26/2020	BL	14M	1	2020	Year-3	1	0	Winter
1/26/2020	BL	8A	1	2020	Year-3		1	Winter
1/26/2020	BL	9A	1	2020	Year-3		1	Winter
1/26/2020	BL	11A	1	2020	Year-3		1	Winter
1/26/2020	BL	13A	1	2020	Year-3		1	Winter
1/27/2020	BL	1M	1	2020	Year-3	0	0	Winter
1/27/2020	BL	2M	1	2020	Year-3	1	0	Winter
1/27/2020	BL	3M	1	2020	Year-3	1	0	Winter
1/27/2020	BL	4M	1	2020	Year-3	0	0	Winter
1/27/2020	BL	5M	1	2020	Year-3	0	0	Winter
1/27/2020	BL	6M	1	2020	Year-3	0	0	Winter
1/27/2020	BL	7M	1	2020	Year-3		1	Winter
1/27/2020	BL	10M	1	2020	Year-3	0	0	Winter
1/27/2020	BL	12M	1	2020	Year-3	0	0	Winter
1/27/2020	BL	14M	1	2020	Year-3	1	0	Winter
1/27/2020	BL	8A	1	2020	Year-3		1	Winter
1/27/2020	BL	9A	1	2020	Year-3		1	Winter
1/27/2020	BL	11A	1	2020	Year-3		1	Winter
1/27/2020	BL	13A	1	2020	Year-3		1	Winter
1/28/2020	BL	1M	1	2020	Year-3	0	0	Winter
1/28/2020	BL	2M	1	2020	Year-3	0	0	Winter
1/28/2020	BL	3M	1	2020	Year-3	0	0	Winter
1/28/2020	BL	4M	1	2020	Year-3	0	0	Winter
1/28/2020	BL	5M	1	2020	Year-3	0	0	Winter
1/28/2020	BL	6M	1	2020	Year-3	0	0	Winter
1/28/2020	BL	7M	1	2020	Year-3		1	Winter
1/28/2020	BL	10M	1	2020	Year-3	0	0	Winter
1/28/2020	BL	12M	1	2020	Year-3	0	0	Winter
1/28/2020	BL	14M	1	2020	Year-3	0	0	Winter
1/28/2020	BL	8A	1	2020	Year-3		1	Winter
1/28/2020	BL	9A	1	2020	Year-3		1	Winter
1/28/2020	BL	11A	1	2020	Year-3		1	Winter
1/28/2020	BL	13A	1	2020	Year-3		1	Winter
1/29/2020	BL	1M	1	2020	Year-3	0	0	Winter
1/29/2020	BL	2M	1	2020	Year-3	0	0	Winter
1/29/2020	BL	3M	1	2020	Year-3	0	0	Winter
1/29/2020	BL	4M	1	2020	Year-3	0	0	Winter
1/29/2020	BL	5M	1	2020	Year-3	0	0	Winter
1/29/2020	BL	6M	1	2020	Year-3	0	0	Winter
1/29/2020	BL	7M	1	2020	Year-3		1	Winter

Date	Species	Site	Month	Year	Survey Year	Presence	Data Gap	Season
1/29/2020	BL	10M	1	2020	Year-3	0	0	Winter
1/29/2020	BL	12M	1	2020	Year-3	0	0	Winter
1/29/2020	BL	14M	1	2020	Year-3	0	0	Winter
1/29/2020	BL	8A	1	2020	Year-3		1	Winter
1/29/2020	BL	9A	1	2020	Year-3		1	Winter
1/29/2020	BL	11A	1	2020	Year-3		1	Winter
1/29/2020	BL	13A	1	2020	Year-3		1	Winter
1/30/2020	BL	1M	1	2020	Year-3	1	0	Winter
1/30/2020	BL	2M	1	2020	Year-3	1	0	Winter
1/30/2020	BL	3M	1	2020	Year-3	1	0	Winter
1/30/2020	BL	4M	1	2020	Year-3	1	0	Winter
1/30/2020	BL	5M	1	2020	Year-3	0	0	Winter
1/30/2020	BL	6M	1	2020	Year-3	0	0	Winter
1/30/2020	BL	7M	1	2020	Year-3		1	Winter
1/30/2020	BL	10M	1	2020	Year-3	0	0	Winter
1/30/2020	BL	12M	1	2020	Year-3	1	0	Winter
1/30/2020	BL	14M	1	2020	Year-3	1	0	Winter
1/30/2020	BL	8A	1	2020	Year-3		1	Winter
1/30/2020	BL	9A	1	2020	Year-3		1	Winter
1/30/2020	BL	11A	1	2020	Year-3		1	Winter
1/30/2020	BL	13A	1	2020	Year-3		1	Winter
1/31/2020	BL	1M	1	2020	Year-3	1	0	Winter
1/31/2020	BL	2M	1	2020	Year-3	1	0	Winter
1/31/2020	BL	3M	1	2020	Year-3	1	0	Winter
1/31/2020	BL	4M	1	2020	Year-3	1	0	Winter
1/31/2020	BL	5M	1	2020	Year-3	0	0	Winter
1/31/2020	BL	6M	1	2020	Year-3	0	0	Winter
1/31/2020	BL	7M	1	2020	Year-3		1	Winter
1/31/2020	BL	10M	1	2020	Year-3	0	0	Winter
1/31/2020	BL	12M	1	2020	Year-3	1	0	Winter
1/31/2020	BL	14M	1	2020	Year-3	1	0	Winter
1/31/2020	BL	8A	1	2020	Year-3		1	Winter
1/31/2020	BL	9A	1	2020	Year-3		1	Winter
1/31/2020	BL	11A	1	2020	Year-3		1	Winter
1/31/2020	BL	13A	1	2020	Year-3		1	Winter
2/1/2020	BL	1M	2	2020	Year-3	1	0	Winter
2/1/2020	BL	2M	2	2020	Year-3	1	0	Winter
2/1/2020	BL	3M	2	2020	Year-3	1	0	Winter
2/1/2020	BL	4M	2	2020	Year-3	0	0	Winter
2/1/2020	BL	5M	2	2020	Year-3	0	0	Winter
2/1/2020	BL	6M	2	2020	Year-3	0	0	Winter
2/1/2020	BL	7M	2	2020	Year-3		1	Winter
2/1/2020	BL	10M	2	2020	Year-3	0	0	Winter
2/1/2020	BL	12M	2	2020	Year-3	0	0	Winter

Date	Species	Site	Month	Year	Survey Year	Presence	Data Gap	Season
2/1/2020	BL	14M	2	2020	Year-3	1	0	Winter
2/1/2020	BL	8A	2	2020	Year-3		1	Winter
2/1/2020	BL	9A	2	2020	Year-3		1	Winter
2/1/2020	BL	11A	2	2020	Year-3		1	Winter
2/1/2020	BL	13A	2	2020	Year-3		1	Winter
2/2/2020	BL	1M	2	2020	Year-3	0	0	Winter
2/2/2020	BL	2M	2	2020	Year-3	0	0	Winter
2/2/2020	BL	3M	2	2020	Year-3	0	0	Winter
2/2/2020	BL	4M	2	2020	Year-3	0	0	Winter
2/2/2020	BL	5M	2	2020	Year-3	0	0	Winter
2/2/2020	BL	6M	2	2020	Year-3	0	0	Winter
2/2/2020	BL	7M	2	2020	Year-3		1	Winter
2/2/2020	BL	10M	2	2020	Year-3	0	0	Winter
2/2/2020	BL	12M	2	2020	Year-3	0	0	Winter
2/2/2020	BL	14M	2	2020	Year-3	0	0	Winter
2/2/2020	BL	8A	2	2020	Year-3		1	Winter
2/2/2020	BL	9A	2	2020	Year-3		1	Winter
2/2/2020	BL	11A	2	2020	Year-3		1	Winter
2/2/2020	BL	13A	2	2020	Year-3		1	Winter
2/3/2020	BL	1M	2	2020	Year-3	1	0	Winter
2/3/2020	BL	2M	2	2020	Year-3	1	0	Winter
2/3/2020	BL	3M	2	2020	Year-3	1	0	Winter
2/3/2020	BL	4M	2	2020	Year-3	1	0	Winter
2/3/2020	BL	5M	2	2020	Year-3	0	0	Winter
2/3/2020	BL	6M	2	2020	Year-3	0	0	Winter
2/3/2020	BL	7M	2	2020	Year-3		1	Winter
2/3/2020	BL	10M	2	2020	Year-3	0	0	Winter
2/3/2020	BL	12M	2	2020	Year-3	0	0	Winter
2/3/2020	BL	14M	2	2020	Year-3	1	0	Winter
2/3/2020	BL	8A	2	2020	Year-3		1	Winter
2/3/2020	BL	9A	2	2020	Year-3		1	Winter
2/3/2020	BL	11A	2	2020	Year-3		1	Winter
2/3/2020	BL	13A	2	2020	Year-3		1	Winter
2/4/2020	BL	1M	2	2020	Year-3	1	0	Winter
2/4/2020	BL	2M	2	2020	Year-3	1	0	Winter
2/4/2020	BL	3M	2	2020	Year-3	1	0	Winter
2/4/2020	BL	4M	2	2020	Year-3	1	0	Winter
2/4/2020	BL	5M	2	2020	Year-3	0	0	Winter
2/4/2020	BL	6M	2	2020	Year-3	0	0	Winter
2/4/2020	BL	7M	2	2020	Year-3		1	Winter
2/4/2020	BL	10M	2	2020	Year-3	0	0	Winter
2/4/2020	BL	12M	2	2020	Year-3	1	0	Winter
2/4/2020	BL	14M	2	2020	Year-3	1	0	Winter
2/4/2020	BL	8A	2	2020	Year-3		1	Winter

Date	Species	Site	Month	Year	Survey Year	Presence	Data Gap	Season
2/4/2020	BL	9A	2	2020	Year-3		1	Winter
2/4/2020	BL	11A	2	2020	Year-3		1	Winter
2/4/2020	BL	13A	2	2020	Year-3		1	Winter
2/5/2020	BL	1M	2	2020	Year-3	1	0	Winter
2/5/2020	BL	2M	2	2020	Year-3	1	0	Winter
2/5/2020	BL	3M	2	2020	Year-3	1	0	Winter
2/5/2020	BL	4M	2	2020	Year-3	1	0	Winter
2/5/2020	BL	5M	2	2020	Year-3	0	0	Winter
2/5/2020	BL	6M	2	2020	Year-3	0	0	Winter
2/5/2020	BL	7M	2	2020	Year-3		1	Winter
2/5/2020	BL	10M	2	2020	Year-3	0	0	Winter
2/5/2020	BL	12M	2	2020	Year-3	1	0	Winter
2/5/2020	BL	14M	2	2020	Year-3	1	0	Winter
2/5/2020	BL	8A	2	2020	Year-3		1	Winter
2/5/2020	BL	9A	2	2020	Year-3		1	Winter
2/5/2020	BL	11A	2	2020	Year-3		1	Winter
2/5/2020	BL	13A	2	2020	Year-3		1	Winter
2/6/2020	BL	1M	2	2020	Year-3	1	0	Winter
2/6/2020	BL	2M	2	2020	Year-3	1	0	Winter
2/6/2020	BL	3M	2	2020	Year-3	1	0	Winter
2/6/2020	BL	4M	2	2020	Year-3	1	0	Winter
2/6/2020	BL	5M	2	2020	Year-3	0	0	Winter
2/6/2020	BL	6M	2	2020	Year-3	0	0	Winter
2/6/2020	BL	7M	2	2020	Year-3		1	Winter
2/6/2020	BL	10M	2	2020	Year-3	0	0	Winter
2/6/2020	BL	12M	2	2020	Year-3	0	0	Winter
2/6/2020	BL	14M	2	2020	Year-3	1	0	Winter
2/6/2020	BL	8A	2	2020	Year-3		1	Winter
2/6/2020	BL	9A	2	2020	Year-3		1	Winter
2/6/2020	BL	11A	2	2020	Year-3		1	Winter
2/6/2020	BL	13A	2	2020	Year-3		1	Winter
2/7/2020	BL	1M	2	2020	Year-3	1	0	Winter
2/7/2020	BL	2M	2	2020	Year-3	1	0	Winter
2/7/2020	BL	3M	2	2020	Year-3	1	0	Winter
2/7/2020	BL	4M	2	2020	Year-3	1	0	Winter
2/7/2020	BL	5M	2	2020	Year-3	0	0	Winter
2/7/2020	BL	6M	2	2020	Year-3	0	0	Winter
2/7/2020	BL	7M	2	2020	Year-3		1	Winter
2/7/2020	BL	10M	2	2020	Year-3	0	0	Winter
2/7/2020	BL	12M	2	2020	Year-3	1	0	Winter
2/7/2020	BL	14M	2	2020	Year-3	1	0	Winter
2/7/2020	BL	8A	2	2020	Year-3		1	Winter
2/7/2020	BL	9A	2	2020	Year-3		1	Winter
2/7/2020	BL	11A	2	2020	Year-3		1	Winter

Date	Species	Site	Month	Year	Survey Year	Presence	Data Gap	Season
2/7/2020	BL	13A	2	2020	Year-3		1	Winter
2/8/2020	BL	1M	2	2020	Year-3	1	0	Winter
2/8/2020	BL	2M	2	2020	Year-3	1	0	Winter
2/8/2020	BL	3M	2	2020	Year-3	0	0	Winter
2/8/2020	BL	4M	2	2020	Year-3	0	0	Winter
2/8/2020	BL	5M	2	2020	Year-3	0	0	Winter
2/8/2020	BL	6M	2	2020	Year-3	0	0	Winter
2/8/2020	BL	7M	2	2020	Year-3		1	Winter
2/8/2020	BL	10M	2	2020	Year-3	0	0	Winter
2/8/2020	BL	12M	2	2020	Year-3	0	0	Winter
2/8/2020	BL	14M	2	2020	Year-3	0	0	Winter
2/8/2020	BL	8A	2	2020	Year-3		1	Winter
2/8/2020	BL	9A	2	2020	Year-3		1	Winter
2/8/2020	BL	11A	2	2020	Year-3		1	Winter
2/8/2020	BL	13A	2	2020	Year-3		1	Winter
2/9/2020	BL	1M	2	2020	Year-3	1	0	Winter
2/9/2020	BL	2M	2	2020	Year-3	1	0	Winter
2/9/2020	BL	3M	2	2020	Year-3	1	0	Winter
2/9/2020	BL	4M	2	2020	Year-3	0	0	Winter
2/9/2020	BL	5M	2	2020	Year-3	0	0	Winter
2/9/2020	BL	6M	2	2020	Year-3	0	0	Winter
2/9/2020	BL	7M	2	2020	Year-3		1	Winter
2/9/2020	BL	10M	2	2020	Year-3	0	0	Winter
2/9/2020	BL	12M	2	2020	Year-3	0	0	Winter
2/9/2020	BL	14M	2	2020	Year-3	0	0	Winter
2/9/2020	BL	8A	2	2020	Year-3		1	Winter
2/9/2020	BL	9A	2	2020	Year-3		1	Winter
2/9/2020	BL	11A	2	2020	Year-3		1	Winter
2/9/2020	BL	13A	2	2020	Year-3		1	Winter
2/10/2020	BL	1M	2	2020	Year-3	1	0	Winter
2/10/2020	BL	2M	2	2020	Year-3	1	0	Winter
2/10/2020	BL	3M	2	2020	Year-3	0	0	Winter
2/10/2020	BL	4M	2	2020	Year-3	0	0	Winter
2/10/2020	BL	5M	2	2020	Year-3	0	0	Winter
2/10/2020	BL	6M	2	2020	Year-3	0	0	Winter
2/10/2020	BL	7M	2	2020	Year-3		1	Winter
2/10/2020	BL	10M	2	2020	Year-3	0	0	Winter
2/10/2020	BL	12M	2	2020	Year-3	0	0	Winter
2/10/2020	BL	14M	2	2020	Year-3	0	0	Winter
2/10/2020	BL	8A	2	2020	Year-3		1	Winter
2/10/2020	BL	9A	2	2020	Year-3		1	Winter
2/10/2020	BL	11A	2	2020	Year-3		1	Winter
2/10/2020	BL	13A	2	2020	Year-3		1	Winter
2/11/2020	BL	1M	2	2020	Year-3	0	0	Winter

Date	Species	Site	Month	Year	Survey Year	Presence	Data Gap	Season
2/11/2020	BL	2M	2	2020	Year-3	0	0	Winter
2/11/2020	BL	3M	2	2020	Year-3	0	0	Winter
2/11/2020	BL	4M	2	2020	Year-3	0	0	Winter
2/11/2020	BL	5M	2	2020	Year-3	0	0	Winter
2/11/2020	BL	6M	2	2020	Year-3	0	0	Winter
2/11/2020	BL	7M	2	2020	Year-3		1	Winter
2/11/2020	BL	10M	2	2020	Year-3	0	0	Winter
2/11/2020	BL	12M	2	2020	Year-3	0	0	Winter
2/11/2020	BL	14M	2	2020	Year-3	0	0	Winter
2/11/2020	BL	8A	2	2020	Year-3		1	Winter
2/11/2020	BL	9A	2	2020	Year-3		1	Winter
2/11/2020	BL	11A	2	2020	Year-3		1	Winter
2/11/2020	BL	13A	2	2020	Year-3		1	Winter
2/12/2020	BL	1M	2	2020	Year-3	0	0	Winter
2/12/2020	BL	2M	2	2020	Year-3	0	0	Winter
2/12/2020	BL	3M	2	2020	Year-3	0	0	Winter
2/12/2020	BL	4M	2	2020	Year-3	0	0	Winter
2/12/2020	BL	5M	2	2020	Year-3	0	0	Winter
2/12/2020	BL	6M	2	2020	Year-3	0	0	Winter
2/12/2020	BL	7M	2	2020	Year-3		1	Winter
2/12/2020	BL	10M	2	2020	Year-3	0	0	Winter
2/12/2020	BL	12M	2	2020	Year-3	0	0	Winter
2/12/2020	BL	14M	2	2020	Year-3	0	0	Winter
2/12/2020	BL	8A	2	2020	Year-3		1	Winter
2/12/2020	BL	9A	2	2020	Year-3		1	Winter
2/12/2020	BL	11A	2	2020	Year-3		1	Winter
2/12/2020	BL	13A	2	2020	Year-3		1	Winter
2/13/2020	BL	1M	2	2020	Year-3	0	0	Winter
2/13/2020	BL	2M	2	2020	Year-3	0	0	Winter
2/13/2020	BL	3M	2	2020	Year-3	0	0	Winter
2/13/2020	BL	4M	2	2020	Year-3	0	0	Winter
2/13/2020	BL	5M	2	2020	Year-3	0	0	Winter
2/13/2020	BL	6M	2	2020	Year-3	0	0	Winter
2/13/2020	BL	7M	2	2020	Year-3		1	Winter
2/13/2020	BL	10M	2	2020	Year-3	0	0	Winter
2/13/2020	BL	12M	2	2020	Year-3	0	0	Winter
2/13/2020	BL	14M	2	2020	Year-3	0	0	Winter
2/13/2020	BL	8A	2	2020	Year-3		1	Winter
2/13/2020	BL	9A	2	2020	Year-3		1	Winter
2/13/2020	BL	11A	2	2020	Year-3		1	Winter
2/13/2020	BL	13A	2	2020	Year-3		1	Winter
2/14/2020	BL	1M	2	2020	Year-3	0	0	Winter
2/14/2020	BL	2M	2	2020	Year-3	0	0	Winter
2/14/2020	BL	3M	2	2020	Year-3	0	0	Winter

Date	Species	Site	Month	Year	Survey Year	Presence	Data Gap	Season
2/14/2020	BL	4M	2	2020	Year-3	0	0	Winter
2/14/2020	BL	5M	2	2020	Year-3	0	0	Winter
2/14/2020	BL	6M	2	2020	Year-3	0	0	Winter
2/14/2020	BL	7M	2	2020	Year-3		1	Winter
2/14/2020	BL	10M	2	2020	Year-3	0	0	Winter
2/14/2020	BL	12M	2	2020	Year-3	0	0	Winter
2/14/2020	BL	14M	2	2020	Year-3	0	0	Winter
2/14/2020	BL	8A	2	2020	Year-3		1	Winter
2/14/2020	BL	9A	2	2020	Year-3		1	Winter
2/14/2020	BL	11A	2	2020	Year-3		1	Winter
2/14/2020	BL	13A	2	2020	Year-3		1	Winter
2/15/2020	BL	1M	2	2020	Year-3	0	0	Winter
2/15/2020	BL	2M	2	2020	Year-3	0	0	Winter
2/15/2020	BL	3M	2	2020	Year-3	0	0	Winter
2/15/2020	BL	4M	2	2020	Year-3	0	0	Winter
2/15/2020	BL	5M	2	2020	Year-3	0	0	Winter
2/15/2020	BL	6M	2	2020	Year-3	0	0	Winter
2/15/2020	BL	7M	2	2020	Year-3		1	Winter
2/15/2020	BL	10M	2	2020	Year-3	0	0	Winter
2/15/2020	BL	12M	2	2020	Year-3	0	0	Winter
2/15/2020	BL	14M	2	2020	Year-3	0	0	Winter
2/15/2020	BL	8A	2	2020	Year-3		1	Winter
2/15/2020	BL	9A	2	2020	Year-3		1	Winter
2/15/2020	BL	11A	2	2020	Year-3		1	Winter
2/15/2020	BL	13A	2	2020	Year-3		1	Winter
2/16/2020	BL	1M	2	2020	Year-3	0	0	Winter
2/16/2020	BL	2M	2	2020	Year-3	0	0	Winter
2/16/2020	BL	3M	2	2020	Year-3	0	0	Winter
2/16/2020	BL	4M	2	2020	Year-3	0	0	Winter
2/16/2020	BL	5M	2	2020	Year-3	0	0	Winter
2/16/2020	BL	6M	2	2020	Year-3	0	0	Winter
2/16/2020	BL	7M	2	2020	Year-3		1	Winter
2/16/2020	BL	10M	2	2020	Year-3	0	0	Winter
2/16/2020	BL	12M	2	2020	Year-3	0	0	Winter
2/16/2020	BL	14M	2	2020	Year-3	0	0	Winter
2/16/2020	BL	8A	2	2020	Year-3		1	Winter
2/16/2020	BL	9A	2	2020	Year-3		1	Winter
2/16/2020	BL	11A	2	2020	Year-3		1	Winter
2/16/2020	BL	13A	2	2020	Year-3		1	Winter
2/17/2020	BL	1M	2	2020	Year-3	0	0	Winter
2/17/2020	BL	2M	2	2020	Year-3	0	0	Winter
2/17/2020	BL	3M	2	2020	Year-3	0	0	Winter
2/17/2020	BL	4M	2	2020	Year-3	0	0	Winter
2/17/2020	BL	5M	2	2020	Year-3	0	0	Winter

Date	Species	Site	Month	Year	Survey Year	Presence	Data Gap	Season
2/17/2020	BL	6M	2	2020	Year-3	0	0	Winter
2/17/2020	BL	7M	2	2020	Year-3		1	Winter
2/17/2020	BL	10M	2	2020	Year-3	0	0	Winter
2/17/2020	BL	12M	2	2020	Year-3	0	0	Winter
2/17/2020	BL	14M	2	2020	Year-3	0	0	Winter
2/17/2020	BL	8A	2	2020	Year-3		1	Winter
2/17/2020	BL	9A	2	2020	Year-3		1	Winter
2/17/2020	BL	11A	2	2020	Year-3		1	Winter
2/17/2020	BL	13A	2	2020	Year-3		1	Winter
2/18/2020	BL	1M	2	2020	Year-3	0	0	Winter
2/18/2020	BL	2M	2	2020	Year-3	0	0	Winter
2/18/2020	BL	3M	2	2020	Year-3	0	0	Winter
2/18/2020	BL	4M	2	2020	Year-3	0	0	Winter
2/18/2020	BL	5M	2	2020	Year-3	0	0	Winter
2/18/2020	BL	6M	2	2020	Year-3	0	0	Winter
2/18/2020	BL	7M	2	2020	Year-3		1	Winter
2/18/2020	BL	10M	2	2020	Year-3	0	0	Winter
2/18/2020	BL	12M	2	2020	Year-3	0	0	Winter
2/18/2020	BL	14M	2	2020	Year-3	1	0	Winter
2/18/2020	BL	8A	2	2020	Year-3		1	Winter
2/18/2020	BL	9A	2	2020	Year-3		1	Winter
2/18/2020	BL	11A	2	2020	Year-3		1	Winter
2/18/2020	BL	13A	2	2020	Year-3		1	Winter
2/19/2020	BL	1M	2	2020	Year-3	0	0	Winter
2/19/2020	BL	2M	2	2020	Year-3	0	0	Winter
2/19/2020	BL	3M	2	2020	Year-3	0	0	Winter
2/19/2020	BL	4M	2	2020	Year-3	0	0	Winter
2/19/2020	BL	5M	2	2020	Year-3	0	0	Winter
2/19/2020	BL	6M	2	2020	Year-3	0	0	Winter
2/19/2020	BL	7M	2	2020	Year-3		1	Winter
2/19/2020	BL	10M	2	2020	Year-3	0	0	Winter
2/19/2020	BL	12M	2	2020	Year-3	0	0	Winter
2/19/2020	BL	14M	2	2020	Year-3	0	0	Winter
2/19/2020	BL	8A	2	2020	Year-3		1	Winter
2/19/2020	BL	9A	2	2020	Year-3		1	Winter
2/19/2020	BL	11A	2	2020	Year-3		1	Winter
2/19/2020	BL	13A	2	2020	Year-3		1	Winter
2/20/2020	BL	1M	2	2020	Year-3	0	0	Winter
2/20/2020	BL	2M	2	2020	Year-3	0	0	Winter
2/20/2020	BL	3M	2	2020	Year-3	0	0	Winter
2/20/2020	BL	4M	2	2020	Year-3	0	0	Winter
2/20/2020	BL	5M	2	2020	Year-3	0	0	Winter
2/20/2020	BL	6M	2	2020	Year-3	0	0	Winter
2/20/2020	BL	7M	2	2020	Year-3		1	Winter

Date	Species	Site	Month	Year	Survey Year	Presence	Data Gap	Season
2/20/2020	BL	10M	2	2020	Year-3	0	0	Winter
2/20/2020	BL	12M	2	2020	Year-3	0	0	Winter
2/20/2020	BL	14M	2	2020	Year-3	0	0	Winter
2/20/2020	BL	8A	2	2020	Year-3		1	Winter
2/20/2020	BL	9A	2	2020	Year-3		1	Winter
2/20/2020	BL	11A	2	2020	Year-3		1	Winter
2/20/2020	BL	13A	2	2020	Year-3		1	Winter
2/21/2020	BL	1M	2	2020	Year-3	1	0	Winter
2/21/2020	BL	2M	2	2020	Year-3	1	0	Winter
2/21/2020	BL	3M	2	2020	Year-3	0	0	Winter
2/21/2020	BL	4M	2	2020	Year-3	0	0	Winter
2/21/2020	BL	5M	2	2020	Year-3	0	0	Winter
2/21/2020	BL	6M	2	2020	Year-3	0	0	Winter
2/21/2020	BL	7M	2	2020	Year-3		1	Winter
2/21/2020	BL	10M	2	2020	Year-3	0	0	Winter
2/21/2020	BL	12M	2	2020	Year-3	0	0	Winter
2/21/2020	BL	14M	2	2020	Year-3	0	0	Winter
2/21/2020	BL	8A	2	2020	Year-3		1	Winter
2/21/2020	BL	9A	2	2020	Year-3		1	Winter
2/21/2020	BL	11A	2	2020	Year-3		1	Winter
2/21/2020	BL	13A	2	2020	Year-3		1	Winter
2/22/2020	BL	1M	2	2020	Year-3	0	0	Winter
2/22/2020	BL	2M	2	2020	Year-3	0	0	Winter
2/22/2020	BL	3M	2	2020	Year-3	0	0	Winter
2/22/2020	BL	4M	2	2020	Year-3	0	0	Winter
2/22/2020	BL	5M	2	2020	Year-3	0	0	Winter
2/22/2020	BL	6M	2	2020	Year-3	0	0	Winter
2/22/2020	BL	7M	2	2020	Year-3		1	Winter
2/22/2020	BL	10M	2	2020	Year-3	0	0	Winter
2/22/2020	BL	12M	2	2020	Year-3	0	0	Winter
2/22/2020	BL	14M	2	2020	Year-3	0	0	Winter
2/22/2020	BL	8A	2	2020	Year-3		1	Winter
2/22/2020	BL	9A	2	2020	Year-3		1	Winter
2/22/2020	BL	11A	2	2020	Year-3		1	Winter
2/22/2020	BL	13A	2	2020	Year-3		1	Winter
2/23/2020	BL	1M	2	2020	Year-3	0	0	Winter
2/23/2020	BL	2M	2	2020	Year-3	0	0	Winter
2/23/2020	BL	3M	2	2020	Year-3	0	0	Winter
2/23/2020	BL	4M	2	2020	Year-3	0	0	Winter
2/23/2020	BL	5M	2	2020	Year-3	0	0	Winter
2/23/2020	BL	6M	2	2020	Year-3	0	0	Winter
2/23/2020	BL	7M	2	2020	Year-3		1	Winter
2/23/2020	BL	10M	2	2020	Year-3	0	0	Winter
2/23/2020	BL	12M	2	2020	Year-3	0	0	Winter

Date	Species	Site	Month	Year	Survey Year	Presence	Data Gap	Season
2/23/2020	BL	14M	2	2020	Year-3	0	0	Winter
2/23/2020	BL	8A	2	2020	Year-3		1	Winter
2/23/2020	BL	9A	2	2020	Year-3		1	Winter
2/23/2020	BL	11A	2	2020	Year-3		1	Winter
2/23/2020	BL	13A	2	2020	Year-3		1	Winter
2/24/2020	BL	1M	2	2020	Year-3	0	0	Winter
2/24/2020	BL	2M	2	2020	Year-3	0	0	Winter
2/24/2020	BL	3M	2	2020	Year-3	0	0	Winter
2/24/2020	BL	4M	2	2020	Year-3	0	0	Winter
2/24/2020	BL	5M	2	2020	Year-3	0	0	Winter
2/24/2020	BL	6M	2	2020	Year-3	0	0	Winter
2/24/2020	BL	7M	2	2020	Year-3		1	Winter
2/24/2020	BL	10M	2	2020	Year-3	0	0	Winter
2/24/2020	BL	12M	2	2020	Year-3	0	0	Winter
2/24/2020	BL	14M	2	2020	Year-3	0	0	Winter
2/24/2020	BL	8A	2	2020	Year-3		1	Winter
2/24/2020	BL	9A	2	2020	Year-3		1	Winter
2/24/2020	BL	11A	2	2020	Year-3		1	Winter
2/24/2020	BL	13A	2	2020	Year-3		1	Winter
2/25/2020	BL	1M	2	2020	Year-3	0	0	Winter
2/25/2020	BL	2M	2	2020	Year-3	0	0	Winter
2/25/2020	BL	3M	2	2020	Year-3	0	0	Winter
2/25/2020	BL	4M	2	2020	Year-3	0	0	Winter
2/25/2020	BL	5M	2	2020	Year-3	0	0	Winter
2/25/2020	BL	6M	2	2020	Year-3	0	0	Winter
2/25/2020	BL	7M	2	2020	Year-3		1	Winter
2/25/2020	BL	10M	2	2020	Year-3	0	0	Winter
2/25/2020	BL	12M	2	2020	Year-3	0	0	Winter
2/25/2020	BL	14M	2	2020	Year-3	0	0	Winter
2/25/2020	BL	8A	2	2020	Year-3		1	Winter
2/25/2020	BL	9A	2	2020	Year-3		1	Winter
2/25/2020	BL	11A	2	2020	Year-3		1	Winter
2/25/2020	BL	13A	2	2020	Year-3		1	Winter
2/26/2020	BL	1M	2	2020	Year-3	0	0	Winter
2/26/2020	BL	2M	2	2020	Year-3	0	0	Winter
2/26/2020	BL	3M	2	2020	Year-3	0	0	Winter
2/26/2020	BL	4M	2	2020	Year-3	0	0	Winter
2/26/2020	BL	5M	2	2020	Year-3	0	0	Winter
2/26/2020	BL	6M	2	2020	Year-3	0	0	Winter
2/26/2020	BL	7M	2	2020	Year-3		1	Winter
2/26/2020	BL	10M	2	2020	Year-3	0	0	Winter
2/26/2020	BL	12M	2	2020	Year-3	0	0	Winter
2/26/2020	BL	14M	2	2020	Year-3	0	0	Winter
2/26/2020	BL	8A	2	2020	Year-3		1	Winter

Date	Species	Site	Month	Year	Survey Year	Presence	Data Gap	Season
2/26/2020	BL	9A	2	2020	Year-3		1	Winter
2/26/2020	BL	11A	2	2020	Year-3		1	Winter
2/26/2020	BL	13A	2	2020	Year-3		1	Winter
2/27/2020	BL	1M	2	2020	Year-3	0	0	Winter
2/27/2020	BL	2M	2	2020	Year-3	0	0	Winter
2/27/2020	BL	3M	2	2020	Year-3	0	0	Winter
2/27/2020	BL	4M	2	2020	Year-3	0	0	Winter
2/27/2020	BL	5M	2	2020	Year-3	0	0	Winter
2/27/2020	BL	6M	2	2020	Year-3	0	0	Winter
2/27/2020	BL	7M	2	2020	Year-3		1	Winter
2/27/2020	BL	10M	2	2020	Year-3	0	0	Winter
2/27/2020	BL	12M	2	2020	Year-3	0	0	Winter
2/27/2020	BL	14M	2	2020	Year-3	0	0	Winter
2/27/2020	BL	8A	2	2020	Year-3		1	Winter
2/27/2020	BL	9A	2	2020	Year-3		1	Winter
2/27/2020	BL	11A	2	2020	Year-3		1	Winter
2/27/2020	BL	13A	2	2020	Year-3		1	Winter
2/28/2020	BL	1M	2	2020	Year-3	0	0	Winter
2/28/2020	BL	2M	2	2020	Year-3	0	0	Winter
2/28/2020	BL	3M	2	2020	Year-3	0	0	Winter
2/28/2020	BL	4M	2	2020	Year-3	0	0	Winter
2/28/2020	BL	5M	2	2020	Year-3	0	0	Winter
2/28/2020	BL	6M	2	2020	Year-3	0	0	Winter
2/28/2020	BL	7M	2	2020	Year-3		1	Winter
2/28/2020	BL	10M	2	2020	Year-3	0	0	Winter
2/28/2020	BL	12M	2	2020	Year-3	0	0	Winter
2/28/2020	BL	14M	2	2020	Year-3	0	0	Winter
2/28/2020	BL	8A	2	2020	Year-3		1	Winter
2/28/2020	BL	9A	2	2020	Year-3		1	Winter
2/28/2020	BL	11A	2	2020	Year-3		1	Winter
2/28/2020	BL	13A	2	2020	Year-3		1	Winter
2/29/2020	BL	1M	2	2020	Year-3	0	0	Winter
2/29/2020	BL	2M	2	2020	Year-3	0	0	Winter
2/29/2020	BL	3M	2	2020	Year-3	0	0	Winter
2/29/2020	BL	4M	2	2020	Year-3	0	0	Winter
2/29/2020	BL	5M	2	2020	Year-3	0	0	Winter
2/29/2020	BL	6M	2	2020	Year-3	0	0	Winter
2/29/2020	BL	7M	2	2020	Year-3		1	Winter
2/29/2020	BL	10M	2	2020	Year-3	0	0	Winter
2/29/2020	BL	12M	2	2020	Year-3	0	0	Winter
2/29/2020	BL	14M	2	2020	Year-3	0	0	Winter
2/29/2020	BL	8A	2	2020	Year-3		1	Winter
2/29/2020	BL	9A	2	2020	Year-3		1	Winter
2/29/2020	BL	11A	2	2020	Year-3		1	Winter

Date	Species	Site	Month	Year	Survey Year	Presence	Data Gap	Season
2/29/2020	BL	13A	2	2020	Year-3		1	Winter
3/1/2020	BL	1M	3	2020	Year-3	0	0	Spring
3/1/2020	BL	2M	3	2020	Year-3	0	0	Spring
3/1/2020	BL	3M	3	2020	Year-3	0	0	Spring
3/1/2020	BL	4M	3	2020	Year-3	0	0	Spring
3/1/2020	BL	5M	3	2020	Year-3	0	0	Spring
3/1/2020	BL	6M	3	2020	Year-3	0	0	Spring
3/1/2020	BL	7M	3	2020	Year-3		1	Spring
3/1/2020	BL	10M	3	2020	Year-3	0	0	Spring
3/1/2020	BL	12M	3	2020	Year-3	0	0	Spring
3/1/2020	BL	14M	3	2020	Year-3	0	0	Spring
3/1/2020	BL	8A	3	2020	Year-3		1	Spring
3/1/2020	BL	9A	3	2020	Year-3		1	Spring
3/1/2020	BL	11A	3	2020	Year-3		1	Spring
3/1/2020	BL	13A	3	2020	Year-3		1	Spring
3/2/2020	BL	1M	3	2020	Year-3	0	0	Spring
3/2/2020	BL	2M	3	2020	Year-3	0	0	Spring
3/2/2020	BL	3M	3	2020	Year-3	0	0	Spring
3/2/2020	BL	4M	3	2020	Year-3	0	0	Spring
3/2/2020	BL	5M	3	2020	Year-3	0	0	Spring
3/2/2020	BL	6M	3	2020	Year-3	0	0	Spring
3/2/2020	BL	7M	3	2020	Year-3		1	Spring
3/2/2020	BL	10M	3	2020	Year-3	0	0	Spring
3/2/2020	BL	12M	3	2020	Year-3	0	0	Spring
3/2/2020	BL	14M	3	2020	Year-3	0	0	Spring
3/2/2020	BL	8A	3	2020	Year-3		1	Spring
3/2/2020	BL	9A	3	2020	Year-3		1	Spring
3/2/2020	BL	11A	3	2020	Year-3		1	Spring
3/2/2020	BL	13A	3	2020	Year-3		1	Spring
3/3/2020	BL	1M	3	2020	Year-3	0	0	Spring
3/3/2020	BL	2M	3	2020	Year-3	0	0	Spring
3/3/2020	BL	3M	3	2020	Year-3	0	0	Spring
3/3/2020	BL	4M	3	2020	Year-3	0	0	Spring
3/3/2020	BL	5M	3	2020	Year-3	0	0	Spring
3/3/2020	BL	6M	3	2020	Year-3	0	0	Spring
3/3/2020	BL	7M	3	2020	Year-3		1	Spring
3/3/2020	BL	10M	3	2020	Year-3	0	0	Spring
3/3/2020	BL	12M	3	2020	Year-3	0	0	Spring
3/3/2020	BL	14M	3	2020	Year-3	0	0	Spring
3/3/2020	BL	8A	3	2020	Year-3		1	Spring
3/3/2020	BL	9A	3	2020	Year-3		1	Spring
3/3/2020	BL	11A	3	2020	Year-3		1	Spring
3/3/2020	BL	13A	3	2020	Year-3		1	Spring
3/4/2020	BL	1M	3	2020	Year-3	0	0	Spring

Date	Species	Site	Month	Year	Survey Year	Presence	Data Gap	Season
3/4/2020	BL	2M	3	2020	Year-3	0	0	Spring
3/4/2020	BL	3M	3	2020	Year-3	0	0	Spring
3/4/2020	BL	4M	3	2020	Year-3	0	0	Spring
3/4/2020	BL	5M	3	2020	Year-3	0	0	Spring
3/4/2020	BL	6M	3	2020	Year-3	0	0	Spring
3/4/2020	BL	7M	3	2020	Year-3		1	Spring
3/4/2020	BL	10M	3	2020	Year-3	0	0	Spring
3/4/2020	BL	12M	3	2020	Year-3	0	0	Spring
3/4/2020	BL	14M	3	2020	Year-3	0	0	Spring
3/4/2020	BL	8A	3	2020	Year-3		1	Spring
3/4/2020	BL	9A	3	2020	Year-3		1	Spring
3/4/2020	BL	11A	3	2020	Year-3		1	Spring
3/4/2020	BL	13A	3	2020	Year-3		1	Spring
3/5/2020	BL	1M	3	2020	Year-3	0	0	Spring
3/5/2020	BL	2M	3	2020	Year-3	0	0	Spring
3/5/2020	BL	3M	3	2020	Year-3	0	0	Spring
3/5/2020	BL	4M	3	2020	Year-3	0	0	Spring
3/5/2020	BL	5M	3	2020	Year-3	0	0	Spring
3/5/2020	BL	6M	3	2020	Year-3	0	0	Spring
3/5/2020	BL	7M	3	2020	Year-3		1	Spring
3/5/2020	BL	10M	3	2020	Year-3	0	0	Spring
3/5/2020	BL	12M	3	2020	Year-3	0	0	Spring
3/5/2020	BL	14M	3	2020	Year-3	0	0	Spring
3/5/2020	BL	8A	3	2020	Year-3		1	Spring
3/5/2020	BL	9A	3	2020	Year-3		1	Spring
3/5/2020	BL	11A	3	2020	Year-3		1	Spring
3/5/2020	BL	13A	3	2020	Year-3		1	Spring
3/6/2020	BL	1M	3	2020	Year-3	0	0	Spring
3/6/2020	BL	2M	3	2020	Year-3	0	0	Spring
3/6/2020	BL	3M	3	2020	Year-3	0	0	Spring
3/6/2020	BL	4M	3	2020	Year-3	0	0	Spring
3/6/2020	BL	5M	3	2020	Year-3	0	0	Spring
3/6/2020	BL	6M	3	2020	Year-3	0	0	Spring
3/6/2020	BL	7M	3	2020	Year-3		1	Spring
3/6/2020	BL	10M	3	2020	Year-3	0	0	Spring
3/6/2020	BL	12M	3	2020	Year-3	0	0	Spring
3/6/2020	BL	14M	3	2020	Year-3	0	0	Spring
3/6/2020	BL	8A	3	2020	Year-3		1	Spring
3/6/2020	BL	9A	3	2020	Year-3		1	Spring
3/6/2020	BL	11A	3	2020	Year-3		1	Spring
3/6/2020	BL	13A	3	2020	Year-3		1	Spring
3/7/2020	BL	1M	3	2020	Year-3	0	0	Spring
3/7/2020	BL	2M	3	2020	Year-3	0	0	Spring
3/7/2020	BL	3M	3	2020	Year-3	0	0	Spring

Date	Species	Site	Month	Year	Survey Year	Presence	Data Gap	Season
3/7/2020	BL	4M	3	2020	Year-3	0	0	Spring
3/7/2020	BL	5M	3	2020	Year-3	0	0	Spring
3/7/2020	BL	6M	3	2020	Year-3	0	0	Spring
3/7/2020	BL	7M	3	2020	Year-3		1	Spring
3/7/2020	BL	10M	3	2020	Year-3	0	0	Spring
3/7/2020	BL	12M	3	2020	Year-3	0	0	Spring
3/7/2020	BL	14M	3	2020	Year-3	0	0	Spring
3/7/2020	BL	8A	3	2020	Year-3		1	Spring
3/7/2020	BL	9A	3	2020	Year-3		1	Spring
3/7/2020	BL	11A	3	2020	Year-3		1	Spring
3/7/2020	BL	13A	3	2020	Year-3		1	Spring
3/8/2020	BL	1M	3	2020	Year-3	0	0	Spring
3/8/2020	BL	2M	3	2020	Year-3	0	0	Spring
3/8/2020	BL	3M	3	2020	Year-3	0	0	Spring
3/8/2020	BL	4M	3	2020	Year-3	0	0	Spring
3/8/2020	BL	5M	3	2020	Year-3	0	0	Spring
3/8/2020	BL	6M	3	2020	Year-3	0	0	Spring
3/8/2020	BL	7M	3	2020	Year-3		1	Spring
3/8/2020	BL	10M	3	2020	Year-3	0	0	Spring
3/8/2020	BL	12M	3	2020	Year-3	0	0	Spring
3/8/2020	BL	14M	3	2020	Year-3	0	0	Spring
3/8/2020	BL	8A	3	2020	Year-3		1	Spring
3/8/2020	BL	9A	3	2020	Year-3		1	Spring
3/8/2020	BL	11A	3	2020	Year-3		1	Spring
3/8/2020	BL	13A	3	2020	Year-3		1	Spring
3/9/2020	BL	1M	3	2020	Year-3	0	0	Spring
3/9/2020	BL	2M	3	2020	Year-3	0	0	Spring
3/9/2020	BL	3M	3	2020	Year-3	0	0	Spring
3/9/2020	BL	4M	3	2020	Year-3	0	0	Spring
3/9/2020	BL	5M	3	2020	Year-3	0	0	Spring
3/9/2020	BL	6M	3	2020	Year-3	0	0	Spring
3/9/2020	BL	7M	3	2020	Year-3		1	Spring
3/9/2020	BL	10M	3	2020	Year-3	0	0	Spring
3/9/2020	BL	12M	3	2020	Year-3	0	0	Spring
3/9/2020	BL	14M	3	2020	Year-3	0	0	Spring
3/9/2020	BL	8A	3	2020	Year-3		1	Spring
3/9/2020	BL	9A	3	2020	Year-3		1	Spring
3/9/2020	BL	11A	3	2020	Year-3		1	Spring
3/9/2020	BL	13A	3	2020	Year-3		1	Spring
3/10/2020	BL	1M	3	2020	Year-3	0	0	Spring
3/10/2020	BL	2M	3	2020	Year-3	0	0	Spring
3/10/2020	BL	3M	3	2020	Year-3	0	0	Spring
3/10/2020	BL	4M	3	2020	Year-3	0	0	Spring
3/10/2020	BL	5M	3	2020	Year-3	0	0	Spring

Date	Species	Site	Month	Year	Survey Year	Presence	Data Gap	Season
3/10/2020	BL	6M	3	2020	Year-3	0	0	Spring
3/10/2020	BL	7M	3	2020	Year-3		1	Spring
3/10/2020	BL	10M	3	2020	Year-3	0	0	Spring
3/10/2020	BL	12M	3	2020	Year-3	0	0	Spring
3/10/2020	BL	14M	3	2020	Year-3	0	0	Spring
3/10/2020	BL	8A	3	2020	Year-3		1	Spring
3/10/2020	BL	9A	3	2020	Year-3		1	Spring
3/10/2020	BL	11A	3	2020	Year-3		1	Spring
3/10/2020	BL	13A	3	2020	Year-3		1	Spring
3/11/2020	BL	1M	3	2020	Year-3	0	0	Spring
3/11/2020	BL	2M	3	2020	Year-3	0	0	Spring
3/11/2020	BL	3M	3	2020	Year-3	0	0	Spring
3/11/2020	BL	4M	3	2020	Year-3	0	0	Spring
3/11/2020	BL	5M	3	2020	Year-3	0	0	Spring
3/11/2020	BL	6M	3	2020	Year-3	0	0	Spring
3/11/2020	BL	7M	3	2020	Year-3		1	Spring
3/11/2020	BL	10M	3	2020	Year-3	0	0	Spring
3/11/2020	BL	12M	3	2020	Year-3	0	0	Spring
3/11/2020	BL	14M	3	2020	Year-3	0	0	Spring
3/11/2020	BL	8A	3	2020	Year-3		1	Spring
3/11/2020	BL	9A	3	2020	Year-3		1	Spring
3/11/2020	BL	11A	3	2020	Year-3		1	Spring
3/11/2020	BL	13A	3	2020	Year-3		1	Spring
3/12/2020	BL	1M	3	2020	Year-3	0	0	Spring
3/12/2020	BL	2M	3	2020	Year-3	0	0	Spring
3/12/2020	BL	3M	3	2020	Year-3	0	0	Spring
3/12/2020	BL	4M	3	2020	Year-3	0	0	Spring
3/12/2020	BL	5M	3	2020	Year-3	0	0	Spring
3/12/2020	BL	6M	3	2020	Year-3	0	0	Spring
3/12/2020	BL	7M	3	2020	Year-3		1	Spring
3/12/2020	BL	10M	3	2020	Year-3	0	0	Spring
3/12/2020	BL	12M	3	2020	Year-3	0	0	Spring
3/12/2020	BL	14M	3	2020	Year-3	0	0	Spring
3/12/2020	BL	8A	3	2020	Year-3		1	Spring
3/12/2020	BL	9A	3	2020	Year-3		1	Spring
3/12/2020	BL	11A	3	2020	Year-3		1	Spring
3/12/2020	BL	13A	3	2020	Year-3		1	Spring
3/13/2020	BL	1M	3	2020	Year-3	0	0	Spring
3/13/2020	BL	2M	3	2020	Year-3	0	0	Spring
3/13/2020	BL	3M	3	2020	Year-3	0	0	Spring
3/13/2020	BL	4M	3	2020	Year-3	0	0	Spring
3/13/2020	BL	5M	3	2020	Year-3	0	0	Spring
3/13/2020	BL	6M	3	2020	Year-3	0	0	Spring
3/13/2020	BL	7M	3	2020	Year-3		1	Spring

Date	Species	Site	Month	Year	Survey Year	Presence	Data Gap	Season
3/13/2020	BL	10M	3	2020	Year-3	0	0	Spring
3/13/2020	BL	12M	3	2020	Year-3	0	0	Spring
3/13/2020	BL	14M	3	2020	Year-3	0	0	Spring
3/13/2020	BL	8A	3	2020	Year-3		1	Spring
3/13/2020	BL	9A	3	2020	Year-3		1	Spring
3/13/2020	BL	11A	3	2020	Year-3		1	Spring
3/13/2020	BL	13A	3	2020	Year-3		1	Spring
3/14/2020	BL	1M	3	2020	Year-3	0	0	Spring
3/14/2020	BL	2M	3	2020	Year-3	0	0	Spring
3/14/2020	BL	3M	3	2020	Year-3	0	0	Spring
3/14/2020	BL	4M	3	2020	Year-3	0	0	Spring
3/14/2020	BL	5M	3	2020	Year-3	0	0	Spring
3/14/2020	BL	6M	3	2020	Year-3	0	0	Spring
3/14/2020	BL	7M	3	2020	Year-3		1	Spring
3/14/2020	BL	10M	3	2020	Year-3	0	0	Spring
3/14/2020	BL	12M	3	2020	Year-3	0	0	Spring
3/14/2020	BL	14M	3	2020	Year-3	0	0	Spring
3/14/2020	BL	8A	3	2020	Year-3		1	Spring
3/14/2020	BL	9A	3	2020	Year-3		1	Spring
3/14/2020	BL	11A	3	2020	Year-3		1	Spring
3/14/2020	BL	13A	3	2020	Year-3		1	Spring
3/15/2020	BL	1M	3	2020	Year-3	0	0	Spring
3/15/2020	BL	2M	3	2020	Year-3	0	0	Spring
3/15/2020	BL	3M	3	2020	Year-3	0	0	Spring
3/15/2020	BL	4M	3	2020	Year-3	0	0	Spring
3/15/2020	BL	5M	3	2020	Year-3	0	0	Spring
3/15/2020	BL	6M	3	2020	Year-3	0	0	Spring
3/15/2020	BL	7M	3	2020	Year-3		1	Spring
3/15/2020	BL	10M	3	2020	Year-3	0	0	Spring
3/15/2020	BL	12M	3	2020	Year-3	0	0	Spring
3/15/2020	BL	14M	3	2020	Year-3	0	0	Spring
3/15/2020	BL	8A	3	2020	Year-3		1	Spring
3/15/2020	BL	9A	3	2020	Year-3		1	Spring
3/15/2020	BL	11A	3	2020	Year-3		1	Spring
3/15/2020	BL	13A	3	2020	Year-3		1	Spring
3/16/2020	BL	1M	3	2020	Year-3	0	0	Spring
3/16/2020	BL	2M	3	2020	Year-3	0	0	Spring
3/16/2020	BL	3M	3	2020	Year-3	0	0	Spring
3/16/2020	BL	4M	3	2020	Year-3	0	0	Spring
3/16/2020	BL	5M	3	2020	Year-3	0	0	Spring
3/16/2020	BL	6M	3	2020	Year-3	0	0	Spring
3/16/2020	BL	7M	3	2020	Year-3		1	Spring
3/16/2020	BL	10M	3	2020	Year-3	0	0	Spring
3/16/2020	BL	12M	3	2020	Year-3	0	0	Spring

Date	Species	Site	Month	Year	Survey Year	Presence	Data Gap	Season
3/16/2020	BL	14M	3	2020	Year-3	0	0	Spring
3/16/2020	BL	8A	3	2020	Year-3		1	Spring
3/16/2020	BL	9A	3	2020	Year-3		1	Spring
3/16/2020	BL	11A	3	2020	Year-3		1	Spring
3/16/2020	BL	13A	3	2020	Year-3		1	Spring
3/17/2020	BL	1M	3	2020	Year-3	0	0	Spring
3/17/2020	BL	2M	3	2020	Year-3	0	0	Spring
3/17/2020	BL	3M	3	2020	Year-3	0	0	Spring
3/17/2020	BL	4M	3	2020	Year-3	0	0	Spring
3/17/2020	BL	5M	3	2020	Year-3	0	0	Spring
3/17/2020	BL	6M	3	2020	Year-3	0	0	Spring
3/17/2020	BL	7M	3	2020	Year-3		1	Spring
3/17/2020	BL	10M	3	2020	Year-3	0	0	Spring
3/17/2020	BL	12M	3	2020	Year-3	0	0	Spring
3/17/2020	BL	14M	3	2020	Year-3	0	0	Spring
3/17/2020	BL	8A	3	2020	Year-3		1	Spring
3/17/2020	BL	9A	3	2020	Year-3		1	Spring
3/17/2020	BL	11A	3	2020	Year-3		1	Spring
3/17/2020	BL	13A	3	2020	Year-3		1	Spring
3/18/2020	BL	1M	3	2020	Year-3	0	0	Spring
3/18/2020	BL	2M	3	2020	Year-3	0	0	Spring
3/18/2020	BL	3M	3	2020	Year-3	0	0	Spring
3/18/2020	BL	4M	3	2020	Year-3	0	0	Spring
3/18/2020	BL	5M	3	2020	Year-3	0	0	Spring
3/18/2020	BL	6M	3	2020	Year-3	0	0	Spring
3/18/2020	BL	7M	3	2020	Year-3		1	Spring
3/18/2020	BL	10M	3	2020	Year-3	0	0	Spring
3/18/2020	BL	12M	3	2020	Year-3	0	0	Spring
3/18/2020	BL	14M	3	2020	Year-3	0	0	Spring
3/18/2020	BL	8A	3	2020	Year-3		1	Spring
3/18/2020	BL	9A	3	2020	Year-3		1	Spring
3/18/2020	BL	11A	3	2020	Year-3		1	Spring
3/18/2020	BL	13A	3	2020	Year-3		1	Spring
3/19/2020	BL	1M	3	2020	Year-3	0	0	Spring
3/19/2020	BL	2M	3	2020	Year-3	0	0	Spring
3/19/2020	BL	3M	3	2020	Year-3	0	0	Spring
3/19/2020	BL	4M	3	2020	Year-3	0	0	Spring
3/19/2020	BL	5M	3	2020	Year-3	0	0	Spring
3/19/2020	BL	6M	3	2020	Year-3	0	0	Spring
3/19/2020	BL	7M	3	2020	Year-3		1	Spring
3/19/2020	BL	10M	3	2020	Year-3	0	0	Spring
3/19/2020	BL	12M	3	2020	Year-3	0	0	Spring
3/19/2020	BL	14M	3	2020	Year-3	0	0	Spring
3/19/2020	BL	8A	3	2020	Year-3		1	Spring

Date	Species	Site	Month	Year	Survey Year	Presence	Data Gap	Season
3/19/2020	BL	9A	3	2020	Year-3		1	Spring
3/19/2020	BL	11A	3	2020	Year-3		1	Spring
3/19/2020	BL	13A	3	2020	Year-3		1	Spring
3/20/2020	BL	1M	3	2020	Year-3	0	0	Spring
3/20/2020	BL	2M	3	2020	Year-3	0	0	Spring
3/20/2020	BL	3M	3	2020	Year-3	0	0	Spring
3/20/2020	BL	4M	3	2020	Year-3	0	0	Spring
3/20/2020	BL	5M	3	2020	Year-3	0	0	Spring
3/20/2020	BL	6M	3	2020	Year-3	0	0	Spring
3/20/2020	BL	7M	3	2020	Year-3		1	Spring
3/20/2020	BL	10M	3	2020	Year-3	0	0	Spring
3/20/2020	BL	12M	3	2020	Year-3	0	0	Spring
3/20/2020	BL	14M	3	2020	Year-3	0	0	Spring
3/20/2020	BL	8A	3	2020	Year-3		1	Spring
3/20/2020	BL	9A	3	2020	Year-3		1	Spring
3/20/2020	BL	11A	3	2020	Year-3		1	Spring
3/20/2020	BL	13A	3	2020	Year-3		1	Spring
3/21/2020	BL	1M	3	2020	Year-3	0	0	Spring
3/21/2020	BL	2M	3	2020	Year-3	0	0	Spring
3/21/2020	BL	3M	3	2020	Year-3	0	0	Spring
3/21/2020	BL	4M	3	2020	Year-3	0	0	Spring
3/21/2020	BL	5M	3	2020	Year-3	0	0	Spring
3/21/2020	BL	6M	3	2020	Year-3	0	0	Spring
3/21/2020	BL	7M	3	2020	Year-3		1	Spring
3/21/2020	BL	10M	3	2020	Year-3	0	0	Spring
3/21/2020	BL	12M	3	2020	Year-3	0	0	Spring
3/21/2020	BL	14M	3	2020	Year-3	0	0	Spring
3/21/2020	BL	8A	3	2020	Year-3		1	Spring
3/21/2020	BL	9A	3	2020	Year-3		1	Spring
3/21/2020	BL	11A	3	2020	Year-3		1	Spring
3/21/2020	BL	13A	3	2020	Year-3		1	Spring
3/22/2020	BL	1M	3	2020	Year-3	0	0	Spring
3/22/2020	BL	2M	3	2020	Year-3	0	0	Spring
3/22/2020	BL	3M	3	2020	Year-3	0	0	Spring
3/22/2020	BL	4M	3	2020	Year-3	0	0	Spring
3/22/2020	BL	5M	3	2020	Year-3	0	0	Spring
3/22/2020	BL	6M	3	2020	Year-3	0	0	Spring
3/22/2020	BL	7M	3	2020	Year-3		1	Spring
3/22/2020	BL	10M	3	2020	Year-3	0	0	Spring
3/22/2020	BL	12M	3	2020	Year-3	0	0	Spring
3/22/2020	BL	14M	3	2020	Year-3	0	0	Spring
3/22/2020	BL	8A	3	2020	Year-3		1	Spring
3/22/2020	BL	9A	3	2020	Year-3		1	Spring
3/22/2020	BL	11A	3	2020	Year-3		1	Spring

Date	Species	Site	Month	Year	Survey Year	Presence	Data Gap	Season
3/22/2020	BL	13A	3	2020	Year-3		1	Spring
3/23/2020	BL	1M	3	2020	Year-3	0	0	Spring
3/23/2020	BL	2M	3	2020	Year-3	0	0	Spring
3/23/2020	BL	3M	3	2020	Year-3	0	0	Spring
3/23/2020	BL	4M	3	2020	Year-3	0	0	Spring
3/23/2020	BL	5M	3	2020	Year-3	0	0	Spring
3/23/2020	BL	6M	3	2020	Year-3	0	0	Spring
3/23/2020	BL	7M	3	2020	Year-3		1	Spring
3/23/2020	BL	10M	3	2020	Year-3	0	0	Spring
3/23/2020	BL	12M	3	2020	Year-3	0	0	Spring
3/23/2020	BL	14M	3	2020	Year-3	0	0	Spring
3/23/2020	BL	8A	3	2020	Year-3		1	Spring
3/23/2020	BL	9A	3	2020	Year-3		1	Spring
3/23/2020	BL	11A	3	2020	Year-3		1	Spring
3/23/2020	BL	13A	3	2020	Year-3		1	Spring
3/24/2020	BL	1M	3	2020	Year-3	0	0	Spring
3/24/2020	BL	2M	3	2020	Year-3	0	0	Spring
3/24/2020	BL	3M	3	2020	Year-3	0	0	Spring
3/24/2020	BL	4M	3	2020	Year-3	0	0	Spring
3/24/2020	BL	5M	3	2020	Year-3	0	0	Spring
3/24/2020	BL	6M	3	2020	Year-3	0	0	Spring
3/24/2020	BL	7M	3	2020	Year-3		1	Spring
3/24/2020	BL	10M	3	2020	Year-3	0	0	Spring
3/24/2020	BL	12M	3	2020	Year-3	0	0	Spring
3/24/2020	BL	14M	3	2020	Year-3	0	0	Spring
3/24/2020	BL	8A	3	2020	Year-3		1	Spring
3/24/2020	BL	9A	3	2020	Year-3		1	Spring
3/24/2020	BL	11A	3	2020	Year-3		1	Spring
3/24/2020	BL	13A	3	2020	Year-3		1	Spring
3/25/2020	BL	1M	3	2020	Year-3	0	0	Spring
3/25/2020	BL	2M	3	2020	Year-3	0	0	Spring
3/25/2020	BL	3M	3	2020	Year-3	0	0	Spring
3/25/2020	BL	4M	3	2020	Year-3	0	0	Spring
3/25/2020	BL	5M	3	2020	Year-3	0	0	Spring
3/25/2020	BL	6M	3	2020	Year-3	0	0	Spring
3/25/2020	BL	7M	3	2020	Year-3		1	Spring
3/25/2020	BL	10M	3	2020	Year-3	0	0	Spring
3/25/2020	BL	12M	3	2020	Year-3	0	0	Spring
3/25/2020	BL	14M	3	2020	Year-3	0	0	Spring
3/25/2020	BL	8A	3	2020	Year-3		1	Spring
3/25/2020	BL	9A	3	2020	Year-3		1	Spring
3/25/2020	BL	11A	3	2020	Year-3		1	Spring
3/25/2020	BL	13A	3	2020	Year-3		1	Spring
3/26/2020	BL	1M	3	2020	Year-3	0	0	Spring

Date	Species	Site	Month	Year	Survey Year	Presence	Data Gap	Season
3/26/2020	BL	2M	3	2020	Year-3	0	0	Spring
3/26/2020	BL	3M	3	2020	Year-3	0	0	Spring
3/26/2020	BL	4M	3	2020	Year-3	0	0	Spring
3/26/2020	BL	5M	3	2020	Year-3	0	0	Spring
3/26/2020	BL	6M	3	2020	Year-3	0	0	Spring
3/26/2020	BL	7M	3	2020	Year-3		1	Spring
3/26/2020	BL	10M	3	2020	Year-3	0	0	Spring
3/26/2020	BL	12M	3	2020	Year-3	0	0	Spring
3/26/2020	BL	14M	3	2020	Year-3	0	0	Spring
3/26/2020	BL	8A	3	2020	Year-3		1	Spring
3/26/2020	BL	9A	3	2020	Year-3		1	Spring
3/26/2020	BL	11A	3	2020	Year-3		1	Spring
3/26/2020	BL	13A	3	2020	Year-3		1	Spring
3/27/2020	BL	1M	3	2020	Year-3	0	0	Spring
3/27/2020	BL	2M	3	2020	Year-3	0	0	Spring
3/27/2020	BL	3M	3	2020	Year-3	0	0	Spring
3/27/2020	BL	4M	3	2020	Year-3	0	0	Spring
3/27/2020	BL	5M	3	2020	Year-3	0	0	Spring
3/27/2020	BL	6M	3	2020	Year-3	0	0	Spring
3/27/2020	BL	7M	3	2020	Year-3		1	Spring
3/27/2020	BL	10M	3	2020	Year-3	0	0	Spring
3/27/2020	BL	12M	3	2020	Year-3	0	0	Spring
3/27/2020	BL	14M	3	2020	Year-3	0	0	Spring
3/27/2020	BL	8A	3	2020	Year-3		1	Spring
3/27/2020	BL	9A	3	2020	Year-3		1	Spring
3/27/2020	BL	11A	3	2020	Year-3		1	Spring
3/27/2020	BL	13A	3	2020	Year-3		1	Spring
3/28/2020	BL	1M	3	2020	Year-3	0	0	Spring
3/28/2020	BL	2M	3	2020	Year-3	0	0	Spring
3/28/2020	BL	3M	3	2020	Year-3	0	0	Spring
3/28/2020	BL	4M	3	2020	Year-3	0	0	Spring
3/28/2020	BL	5M	3	2020	Year-3	0	0	Spring
3/28/2020	BL	6M	3	2020	Year-3	0	0	Spring
3/28/2020	BL	7M	3	2020	Year-3		1	Spring
3/28/2020	BL	10M	3	2020	Year-3	0	0	Spring
3/28/2020	BL	12M	3	2020	Year-3	0	0	Spring
3/28/2020	BL	14M	3	2020	Year-3	0	0	Spring
3/28/2020	BL	8A	3	2020	Year-3		1	Spring
3/28/2020	BL	9A	3	2020	Year-3		1	Spring
3/28/2020	BL	11A	3	2020	Year-3		1	Spring
3/28/2020	BL	13A	3	2020	Year-3		1	Spring
3/29/2020	BL	1M	3	2020	Year-3	0	0	Spring
3/29/2020	BL	2M	3	2020	Year-3	0	0	Spring
3/29/2020	BL	3M	3	2020	Year-3	0	0	Spring

Date	Species	Site	Month	Year	Survey Year	Presence	Data Gap	Season
3/29/2020	BL	4M	3	2020	Year-3	0	0	Spring
3/29/2020	BL	5M	3	2020	Year-3	0	0	Spring
3/29/2020	BL	6M	3	2020	Year-3	0	0	Spring
3/29/2020	BL	7M	3	2020	Year-3		1	Spring
3/29/2020	BL	10M	3	2020	Year-3	0	0	Spring
3/29/2020	BL	12M	3	2020	Year-3	0	0	Spring
3/29/2020	BL	14M	3	2020	Year-3	0	0	Spring
3/29/2020	BL	8A	3	2020	Year-3		1	Spring
3/29/2020	BL	9A	3	2020	Year-3		1	Spring
3/29/2020	BL	11A	3	2020	Year-3		1	Spring
3/29/2020	BL	13A	3	2020	Year-3		1	Spring
3/30/2020	BL	1M	3	2020	Year-3	0	0	Spring
3/30/2020	BL	2M	3	2020	Year-3	0	0	Spring
3/30/2020	BL	3M	3	2020	Year-3	0	0	Spring
3/30/2020	BL	4M	3	2020	Year-3	0	0	Spring
3/30/2020	BL	5M	3	2020	Year-3	0	0	Spring
3/30/2020	BL	6M	3	2020	Year-3	0	0	Spring
3/30/2020	BL	7M	3	2020	Year-3		1	Spring
3/30/2020	BL	10M	3	2020	Year-3	0	0	Spring
3/30/2020	BL	12M	3	2020	Year-3	0	0	Spring
3/30/2020	BL	14M	3	2020	Year-3	0	0	Spring
3/30/2020	BL	8A	3	2020	Year-3		1	Spring
3/30/2020	BL	9A	3	2020	Year-3		1	Spring
3/30/2020	BL	11A	3	2020	Year-3		1	Spring
3/30/2020	BL	13A	3	2020	Year-3		1	Spring
3/31/2020	BL	1M	3	2020	Year-3	0	0	Spring
3/31/2020	BL	2M	3	2020	Year-3	0	0	Spring
3/31/2020	BL	3M	3	2020	Year-3	0	0	Spring
3/31/2020	BL	4M	3	2020	Year-3	0	0	Spring
3/31/2020	BL	5M	3	2020	Year-3	0	0	Spring
3/31/2020	BL	6M	3	2020	Year-3	0	0	Spring
3/31/2020	BL	7M	3	2020	Year-3		1	Spring
3/31/2020	BL	10M	3	2020	Year-3	0	0	Spring
3/31/2020	BL	12M	3	2020	Year-3	0	0	Spring
3/31/2020	BL	14M	3	2020	Year-3	0	0	Spring
3/31/2020	BL	8A	3	2020	Year-3		1	Spring
3/31/2020	BL	9A	3	2020	Year-3		1	Spring
3/31/2020	BL	11A	3	2020	Year-3		1	Spring
3/31/2020	BL	13A	3	2020	Year-3		1	Spring
4/1/2020	BL	1M	4	2020	Year-3	0	0	Spring
4/1/2020	BL	2M	4	2020	Year-3	0	0	Spring
4/1/2020	BL	3M	4	2020	Year-3	0	0	Spring
4/1/2020	BL	4M	4	2020	Year-3	0	0	Spring
4/1/2020	BL	5M	4	2020	Year-3	0	0	Spring

Date	Species	Site	Month	Year	Survey Year	Presence	Data Gap	Season
4/1/2020	BL	6M	4	2020	Year-3	0	0	Spring
4/1/2020	BL	7M	4	2020	Year-3		1	Spring
4/1/2020	BL	10M	4	2020	Year-3	0	0	Spring
4/1/2020	BL	12M	4	2020	Year-3	0	0	Spring
4/1/2020	BL	14M	4	2020	Year-3	0	0	Spring
4/1/2020	BL	8A	4	2020	Year-3		1	Spring
4/1/2020	BL	9A	4	2020	Year-3		1	Spring
4/1/2020	BL	11A	4	2020	Year-3		1	Spring
4/1/2020	BL	13A	4	2020	Year-3		1	Spring
4/2/2020	BL	1M	4	2020	Year-3	0	0	Spring
4/2/2020	BL	2M	4	2020	Year-3	0	0	Spring
4/2/2020	BL	3M	4	2020	Year-3	0	0	Spring
4/2/2020	BL	4M	4	2020	Year-3	0	0	Spring
4/2/2020	BL	5M	4	2020	Year-3	0	0	Spring
4/2/2020	BL	6M	4	2020	Year-3	0	0	Spring
4/2/2020	BL	7M	4	2020	Year-3		1	Spring
4/2/2020	BL	10M	4	2020	Year-3	0	0	Spring
4/2/2020	BL	12M	4	2020	Year-3	0	0	Spring
4/2/2020	BL	14M	4	2020	Year-3	0	0	Spring
4/2/2020	BL	8A	4	2020	Year-3		1	Spring
4/2/2020	BL	9A	4	2020	Year-3		1	Spring
4/2/2020	BL	11A	4	2020	Year-3		1	Spring
4/2/2020	BL	13A	4	2020	Year-3		1	Spring
4/3/2020	BL	1M	4	2020	Year-3	0	0	Spring
4/3/2020	BL	2M	4	2020	Year-3	0	0	Spring
4/3/2020	BL	3M	4	2020	Year-3	0	0	Spring
4/3/2020	BL	4M	4	2020	Year-3	0	0	Spring
4/3/2020	BL	5M	4	2020	Year-3	0	0	Spring
4/3/2020	BL	6M	4	2020	Year-3	0	0	Spring
4/3/2020	BL	7M	4	2020	Year-3		1	Spring
4/3/2020	BL	10M	4	2020	Year-3	0	0	Spring
4/3/2020	BL	12M	4	2020	Year-3	0	0	Spring
4/3/2020	BL	14M	4	2020	Year-3	0	0	Spring
4/3/2020	BL	8A	4	2020	Year-3		1	Spring
4/3/2020	BL	9A	4	2020	Year-3		1	Spring
4/3/2020	BL	11A	4	2020	Year-3		1	Spring
4/3/2020	BL	13A	4	2020	Year-3		1	Spring
4/4/2020	BL	1M	4	2020	Year-3	0	0	Spring
4/4/2020	BL	2M	4	2020	Year-3	0	0	Spring
4/4/2020	BL	3M	4	2020	Year-3	0	0	Spring
4/4/2020	BL	4M	4	2020	Year-3	0	0	Spring
4/4/2020	BL	5M	4	2020	Year-3	0	0	Spring
4/4/2020	BL	6M	4	2020	Year-3	0	0	Spring
4/4/2020	BL	7M	4	2020	Year-3		1	Spring

Date	Species	Site	Month	Year	Survey Year	Presence	Data Gap	Season
4/4/2020	BL	10M	4	2020	Year-3	0	0	Spring
4/4/2020	BL	12M	4	2020	Year-3	0	0	Spring
4/4/2020	BL	14M	4	2020	Year-3	0	0	Spring
4/4/2020	BL	8A	4	2020	Year-3		1	Spring
4/4/2020	BL	9A	4	2020	Year-3		1	Spring
4/4/2020	BL	11A	4	2020	Year-3		1	Spring
4/4/2020	BL	13A	4	2020	Year-3		1	Spring
4/5/2020	BL	1M	4	2020	Year-3	0	0	Spring
4/5/2020	BL	2M	4	2020	Year-3	0	0	Spring
4/5/2020	BL	3M	4	2020	Year-3	0	0	Spring
4/5/2020	BL	4M	4	2020	Year-3	0	0	Spring
4/5/2020	BL	5M	4	2020	Year-3	0	0	Spring
4/5/2020	BL	6M	4	2020	Year-3	0	0	Spring
4/5/2020	BL	7M	4	2020	Year-3		1	Spring
4/5/2020	BL	10M	4	2020	Year-3	0	0	Spring
4/5/2020	BL	12M	4	2020	Year-3	0	0	Spring
4/5/2020	BL	14M	4	2020	Year-3	0	0	Spring
4/5/2020	BL	8A	4	2020	Year-3		1	Spring
4/5/2020	BL	9A	4	2020	Year-3		1	Spring
4/5/2020	BL	11A	4	2020	Year-3		1	Spring
4/5/2020	BL	13A	4	2020	Year-3		1	Spring
4/6/2020	BL	1M	4	2020	Year-3	0	0	Spring
4/6/2020	BL	2M	4	2020	Year-3	0	0	Spring
4/6/2020	BL	3M	4	2020	Year-3	0	0	Spring
4/6/2020	BL	4M	4	2020	Year-3	0	0	Spring
4/6/2020	BL	5M	4	2020	Year-3	0	0	Spring
4/6/2020	BL	6M	4	2020	Year-3	0	0	Spring
4/6/2020	BL	7M	4	2020	Year-3		1	Spring
4/6/2020	BL	10M	4	2020	Year-3	0	0	Spring
4/6/2020	BL	12M	4	2020	Year-3	0	0	Spring
4/6/2020	BL	14M	4	2020	Year-3	0	0	Spring
4/6/2020	BL	8A	4	2020	Year-3		1	Spring
4/6/2020	BL	9A	4	2020	Year-3		1	Spring
4/6/2020	BL	11A	4	2020	Year-3		1	Spring
4/6/2020	BL	13A	4	2020	Year-3		1	Spring
4/7/2020	BL	1M	4	2020	Year-3	0	0	Spring
4/7/2020	BL	2M	4	2020	Year-3	0	0	Spring
4/7/2020	BL	3M	4	2020	Year-3	0	0	Spring
4/7/2020	BL	4M	4	2020	Year-3	0	0	Spring
4/7/2020	BL	5M	4	2020	Year-3	0	0	Spring
4/7/2020	BL	6M	4	2020	Year-3	0	0	Spring
4/7/2020	BL	7M	4	2020	Year-3		1	Spring
4/7/2020	BL	10M	4	2020	Year-3	0	0	Spring
4/7/2020	BL	12M	4	2020	Year-3	0	0	Spring

Date	Species	Site	Month	Year	Survey Year	Presence	Data Gap	Season
4/7/2020	BL	14M	4	2020	Year-3	0	0	Spring
4/7/2020	BL	8A	4	2020	Year-3		1	Spring
4/7/2020	BL	9A	4	2020	Year-3		1	Spring
4/7/2020	BL	11A	4	2020	Year-3		1	Spring
4/7/2020	BL	13A	4	2020	Year-3		1	Spring
4/8/2020	BL	1M	4	2020	Year-3	0	0	Spring
4/8/2020	BL	2M	4	2020	Year-3	0	0	Spring
4/8/2020	BL	3M	4	2020	Year-3	0	0	Spring
4/8/2020	BL	4M	4	2020	Year-3	0	0	Spring
4/8/2020	BL	5M	4	2020	Year-3	0	0	Spring
4/8/2020	BL	6M	4	2020	Year-3	0	0	Spring
4/8/2020	BL	7M	4	2020	Year-3		1	Spring
4/8/2020	BL	10M	4	2020	Year-3	0	0	Spring
4/8/2020	BL	12M	4	2020	Year-3	0	0	Spring
4/8/2020	BL	14M	4	2020	Year-3	0	0	Spring
4/8/2020	BL	8A	4	2020	Year-3		1	Spring
4/8/2020	BL	9A	4	2020	Year-3		1	Spring
4/8/2020	BL	11A	4	2020	Year-3		1	Spring
4/8/2020	BL	13A	4	2020	Year-3		1	Spring
4/9/2020	BL	1M	4	2020	Year-3	0	0	Spring
4/9/2020	BL	2M	4	2020	Year-3	0	0	Spring
4/9/2020	BL	3M	4	2020	Year-3	0	0	Spring
4/9/2020	BL	4M	4	2020	Year-3	0	0	Spring
4/9/2020	BL	5M	4	2020	Year-3	0	0	Spring
4/9/2020	BL	6M	4	2020	Year-3	0	0	Spring
4/9/2020	BL	7M	4	2020	Year-3		1	Spring
4/9/2020	BL	10M	4	2020	Year-3	0	0	Spring
4/9/2020	BL	12M	4	2020	Year-3	0	0	Spring
4/9/2020	BL	14M	4	2020	Year-3	0	0	Spring
4/9/2020	BL	8A	4	2020	Year-3		1	Spring
4/9/2020	BL	9A	4	2020	Year-3		1	Spring
4/9/2020	BL	11A	4	2020	Year-3		1	Spring
4/9/2020	BL	13A	4	2020	Year-3		1	Spring
4/10/2020	BL	1M	4	2020	Year-3	0	0	Spring
4/10/2020	BL	2M	4	2020	Year-3	0	0	Spring
4/10/2020	BL	3M	4	2020	Year-3	0	0	Spring
4/10/2020	BL	4M	4	2020	Year-3	0	0	Spring
4/10/2020	BL	5M	4	2020	Year-3	0	0	Spring
4/10/2020	BL	6M	4	2020	Year-3	0	0	Spring
4/10/2020	BL	7M	4	2020	Year-3		1	Spring
4/10/2020	BL	10M	4	2020	Year-3	0	0	Spring
4/10/2020	BL	12M	4	2020	Year-3	0	0	Spring
4/10/2020	BL	14M	4	2020	Year-3	0	0	Spring
4/10/2020	BL	8A	4	2020	Year-3		1	Spring

Date	Species	Site	Month	Year	Survey Year	Presence	Data Gap	Season
4/10/2020	BL	9A	4	2020	Year-3		1	Spring
4/10/2020	BL	11A	4	2020	Year-3		1	Spring
4/10/2020	BL	13A	4	2020	Year-3		1	Spring
4/11/2020	BL	1M	4	2020	Year-3	0	0	Spring
4/11/2020	BL	2M	4	2020	Year-3	0	0	Spring
4/11/2020	BL	3M	4	2020	Year-3	0	0	Spring
4/11/2020	BL	4M	4	2020	Year-3	0	0	Spring
4/11/2020	BL	5M	4	2020	Year-3	0	0	Spring
4/11/2020	BL	6M	4	2020	Year-3	0	0	Spring
4/11/2020	BL	7M	4	2020	Year-3		1	Spring
4/11/2020	BL	10M	4	2020	Year-3	0	0	Spring
4/11/2020	BL	12M	4	2020	Year-3	0	0	Spring
4/11/2020	BL	14M	4	2020	Year-3	0	0	Spring
4/11/2020	BL	8A	4	2020	Year-3		1	Spring
4/11/2020	BL	9A	4	2020	Year-3		1	Spring
4/11/2020	BL	11A	4	2020	Year-3		1	Spring
4/11/2020	BL	13A	4	2020	Year-3		1	Spring
4/12/2020	BL	1M	4	2020	Year-3	0	0	Spring
4/12/2020	BL	2M	4	2020	Year-3	0	0	Spring
4/12/2020	BL	3M	4	2020	Year-3	0	0	Spring
4/12/2020	BL	4M	4	2020	Year-3	0	0	Spring
4/12/2020	BL	5M	4	2020	Year-3	0	0	Spring
4/12/2020	BL	6M	4	2020	Year-3	0	0	Spring
4/12/2020	BL	7M	4	2020	Year-3		1	Spring
4/12/2020	BL	10M	4	2020	Year-3	0	0	Spring
4/12/2020	BL	12M	4	2020	Year-3	0	0	Spring
4/12/2020	BL	14M	4	2020	Year-3	0	0	Spring
4/12/2020	BL	8A	4	2020	Year-3		1	Spring
4/12/2020	BL	9A	4	2020	Year-3		1	Spring
4/12/2020	BL	11A	4	2020	Year-3		1	Spring
4/12/2020	BL	13A	4	2020	Year-3		1	Spring
4/13/2020	BL	1M	4	2020	Year-3	0	0	Spring
4/13/2020	BL	2M	4	2020	Year-3	0	0	Spring
4/13/2020	BL	3M	4	2020	Year-3	0	0	Spring
4/13/2020	BL	4M	4	2020	Year-3	0	0	Spring
4/13/2020	BL	5M	4	2020	Year-3	0	0	Spring
4/13/2020	BL	6M	4	2020	Year-3	0	0	Spring
4/13/2020	BL	7M	4	2020	Year-3		1	Spring
4/13/2020	BL	10M	4	2020	Year-3	0	0	Spring
4/13/2020	BL	12M	4	2020	Year-3	0	0	Spring
4/13/2020	BL	14M	4	2020	Year-3	0	0	Spring
4/13/2020	BL	8A	4	2020	Year-3		1	Spring
4/13/2020	BL	9A	4	2020	Year-3		1	Spring
4/13/2020	BL	11A	4	2020	Year-3		1	Spring

Date	Species	Site	Month	Year	Survey Year	Presence	Data Gap	Season
4/13/2020	BL	13A	4	2020	Year-3		1	Spring
4/14/2020	BL	1M	4	2020	Year-3	0	0	Spring
4/14/2020	BL	2M	4	2020	Year-3	0	0	Spring
4/14/2020	BL	3M	4	2020	Year-3	0	0	Spring
4/14/2020	BL	4M	4	2020	Year-3	0	0	Spring
4/14/2020	BL	5M	4	2020	Year-3	0	0	Spring
4/14/2020	BL	6M	4	2020	Year-3	0	0	Spring
4/14/2020	BL	7M	4	2020	Year-3		1	Spring
4/14/2020	BL	10M	4	2020	Year-3	0	0	Spring
4/14/2020	BL	12M	4	2020	Year-3	0	0	Spring
4/14/2020	BL	14M	4	2020	Year-3	0	0	Spring
4/14/2020	BL	8A	4	2020	Year-3		1	Spring
4/14/2020	BL	9A	4	2020	Year-3		1	Spring
4/14/2020	BL	11A	4	2020	Year-3		1	Spring
4/14/2020	BL	13A	4	2020	Year-3		1	Spring
4/15/2020	BL	1M	4	2020	Year-3	0	0	Spring
4/15/2020	BL	2M	4	2020	Year-3	0	0	Spring
4/15/2020	BL	3M	4	2020	Year-3	0	0	Spring
4/15/2020	BL	4M	4	2020	Year-3	0	0	Spring
4/15/2020	BL	5M	4	2020	Year-3	0	0	Spring
4/15/2020	BL	6M	4	2020	Year-3	0	0	Spring
4/15/2020	BL	7M	4	2020	Year-3		1	Spring
4/15/2020	BL	10M	4	2020	Year-3	0	0	Spring
4/15/2020	BL	12M	4	2020	Year-3	0	0	Spring
4/15/2020	BL	14M	4	2020	Year-3	0	0	Spring
4/15/2020	BL	8A	4	2020	Year-3		1	Spring
4/15/2020	BL	9A	4	2020	Year-3		1	Spring
4/15/2020	BL	11A	4	2020	Year-3		1	Spring
4/15/2020	BL	13A	4	2020	Year-3		1	Spring
4/16/2020	BL	1M	4	2020	Year-3	0	0	Spring
4/16/2020	BL	2M	4	2020	Year-3	0	0	Spring
4/16/2020	BL	3M	4	2020	Year-3	0	0	Spring
4/16/2020	BL	4M	4	2020	Year-3	0	0	Spring
4/16/2020	BL	5M	4	2020	Year-3	0	0	Spring
4/16/2020	BL	6M	4	2020	Year-3	0	0	Spring
4/16/2020	BL	7M	4	2020	Year-3		1	Spring
4/16/2020	BL	10M	4	2020	Year-3	0	0	Spring
4/16/2020	BL	12M	4	2020	Year-3	0	0	Spring
4/16/2020	BL	14M	4	2020	Year-3	0	0	Spring
4/16/2020	BL	8A	4	2020	Year-3		1	Spring
4/16/2020	BL	9A	4	2020	Year-3		1	Spring
4/16/2020	BL	11A	4	2020	Year-3		1	Spring
4/16/2020	BL	13A	4	2020	Year-3		1	Spring
4/17/2020	BL	1M	4	2020	Year-3	0	0	Spring

Date	Species	Site	Month	Year	Survey Year	Presence	Data Gap	Season
4/17/2020	BL	2M	4	2020	Year-3	0	0	Spring
4/17/2020	BL	3M	4	2020	Year-3	0	0	Spring
4/17/2020	BL	4M	4	2020	Year-3	0	0	Spring
4/17/2020	BL	5M	4	2020	Year-3	0	0	Spring
4/17/2020	BL	6M	4	2020	Year-3	0	0	Spring
4/17/2020	BL	7M	4	2020	Year-3		1	Spring
4/17/2020	BL	10M	4	2020	Year-3	0	0	Spring
4/17/2020	BL	12M	4	2020	Year-3	0	0	Spring
4/17/2020	BL	14M	4	2020	Year-3	0	0	Spring
4/17/2020	BL	8A	4	2020	Year-3		1	Spring
4/17/2020	BL	9A	4	2020	Year-3		1	Spring
4/17/2020	BL	11A	4	2020	Year-3		1	Spring
4/17/2020	BL	13A	4	2020	Year-3		1	Spring
4/18/2020	BL	1M	4	2020	Year-3	0	0	Spring
4/18/2020	BL	2M	4	2020	Year-3	0	0	Spring
4/18/2020	BL	3M	4	2020	Year-3	0	0	Spring
4/18/2020	BL	4M	4	2020	Year-3	0	0	Spring
4/18/2020	BL	5M	4	2020	Year-3	0	0	Spring
4/18/2020	BL	6M	4	2020	Year-3	0	0	Spring
4/18/2020	BL	7M	4	2020	Year-3		1	Spring
4/18/2020	BL	10M	4	2020	Year-3	0	0	Spring
4/18/2020	BL	12M	4	2020	Year-3	0	0	Spring
4/18/2020	BL	14M	4	2020	Year-3	0	0	Spring
4/18/2020	BL	8A	4	2020	Year-3		1	Spring
4/18/2020	BL	9A	4	2020	Year-3		1	Spring
4/18/2020	BL	11A	4	2020	Year-3		1	Spring
4/18/2020	BL	13A	4	2020	Year-3		1	Spring
4/19/2020	BL	1M	4	2020	Year-3	0	0	Spring
4/19/2020	BL	2M	4	2020	Year-3	0	0	Spring
4/19/2020	BL	3M	4	2020	Year-3	0	0	Spring
4/19/2020	BL	4M	4	2020	Year-3	0	0	Spring
4/19/2020	BL	5M	4	2020	Year-3	0	0	Spring
4/19/2020	BL	6M	4	2020	Year-3	0	0	Spring
4/19/2020	BL	7M	4	2020	Year-3		1	Spring
4/19/2020	BL	10M	4	2020	Year-3	0	0	Spring
4/19/2020	BL	12M	4	2020	Year-3	0	0	Spring
4/19/2020	BL	14M	4	2020	Year-3	0	0	Spring
4/19/2020	BL	8A	4	2020	Year-3		1	Spring
4/19/2020	BL	9A	4	2020	Year-3		1	Spring
4/19/2020	BL	11A	4	2020	Year-3		1	Spring
4/19/2020	BL	13A	4	2020	Year-3		1	Spring
4/20/2020	BL	1M	4	2020	Year-3	0	0	Spring
4/20/2020	BL	2M	4	2020	Year-3	0	0	Spring
4/20/2020	BL	3M	4	2020	Year-3	0	0	Spring

Date	Species	Site	Month	Year	Survey Year	Presence	Data Gap	Season
4/20/2020	BL	4M	4	2020	Year-3	0	0	Spring
4/20/2020	BL	5M	4	2020	Year-3	0	0	Spring
4/20/2020	BL	6M	4	2020	Year-3	0	0	Spring
4/20/2020	BL	7M	4	2020	Year-3		1	Spring
4/20/2020	BL	10M	4	2020	Year-3	0	0	Spring
4/20/2020	BL	12M	4	2020	Year-3	0	0	Spring
4/20/2020	BL	14M	4	2020	Year-3	0	0	Spring
4/20/2020	BL	8A	4	2020	Year-3		1	Spring
4/20/2020	BL	9A	4	2020	Year-3		1	Spring
4/20/2020	BL	11A	4	2020	Year-3		1	Spring
4/20/2020	BL	13A	4	2020	Year-3		1	Spring
4/21/2020	BL	1M	4	2020	Year-3	0	0	Spring
4/21/2020	BL	2M	4	2020	Year-3	0	0	Spring
4/21/2020	BL	3M	4	2020	Year-3	0	0	Spring
4/21/2020	BL	4M	4	2020	Year-3	0	0	Spring
4/21/2020	BL	5M	4	2020	Year-3	0	0	Spring
4/21/2020	BL	6M	4	2020	Year-3	0	0	Spring
4/21/2020	BL	7M	4	2020	Year-3		1	Spring
4/21/2020	BL	10M	4	2020	Year-3	0	0	Spring
4/21/2020	BL	12M	4	2020	Year-3	0	0	Spring
4/21/2020	BL	14M	4	2020	Year-3	0	0	Spring
4/21/2020	BL	8A	4	2020	Year-3		1	Spring
4/21/2020	BL	9A	4	2020	Year-3		1	Spring
4/21/2020	BL	11A	4	2020	Year-3		1	Spring
4/21/2020	BL	13A	4	2020	Year-3		1	Spring
4/22/2020	BL	1M	4	2020	Year-3	0	0	Spring
4/22/2020	BL	2M	4	2020	Year-3	0	0	Spring
4/22/2020	BL	3M	4	2020	Year-3	0	0	Spring
4/22/2020	BL	4M	4	2020	Year-3	0	0	Spring
4/22/2020	BL	5M	4	2020	Year-3	0	0	Spring
4/22/2020	BL	6M	4	2020	Year-3	0	0	Spring
4/22/2020	BL	7M	4	2020	Year-3		1	Spring
4/22/2020	BL	10M	4	2020	Year-3	0	0	Spring
4/22/2020	BL	12M	4	2020	Year-3	0	0	Spring
4/22/2020	BL	14M	4	2020	Year-3	0	0	Spring
4/22/2020	BL	8A	4	2020	Year-3		1	Spring
4/22/2020	BL	9A	4	2020	Year-3		1	Spring
4/22/2020	BL	11A	4	2020	Year-3		1	Spring
4/22/2020	BL	13A	4	2020	Year-3		1	Spring
4/23/2020	BL	1M	4	2020	Year-3	0	0	Spring
4/23/2020	BL	2M	4	2020	Year-3	0	0	Spring
4/23/2020	BL	3M	4	2020	Year-3	0	0	Spring
4/23/2020	BL	4M	4	2020	Year-3	0	0	Spring
4/23/2020	BL	5M	4	2020	Year-3	0	0	Spring

Date	Species	Site	Month	Year	Survey Year	Presence	Data Gap	Season
4/23/2020	BL	6M	4	2020	Year-3	0	0	Spring
4/23/2020	BL	7M	4	2020	Year-3		1	Spring
4/23/2020	BL	10M	4	2020	Year-3	0	0	Spring
4/23/2020	BL	12M	4	2020	Year-3	0	0	Spring
4/23/2020	BL	14M	4	2020	Year-3	0	0	Spring
4/23/2020	BL	8A	4	2020	Year-3		1	Spring
4/23/2020	BL	9A	4	2020	Year-3		1	Spring
4/23/2020	BL	11A	4	2020	Year-3		1	Spring
4/23/2020	BL	13A	4	2020	Year-3		1	Spring
4/24/2020	BL	1M	4	2020	Year-3	0	0	Spring
4/24/2020	BL	2M	4	2020	Year-3	0	0	Spring
4/24/2020	BL	3M	4	2020	Year-3	0	0	Spring
4/24/2020	BL	4M	4	2020	Year-3	0	0	Spring
4/24/2020	BL	5M	4	2020	Year-3	0	0	Spring
4/24/2020	BL	6M	4	2020	Year-3	0	0	Spring
4/24/2020	BL	7M	4	2020	Year-3		1	Spring
4/24/2020	BL	10M	4	2020	Year-3	0	0	Spring
4/24/2020	BL	12M	4	2020	Year-3	0	0	Spring
4/24/2020	BL	14M	4	2020	Year-3	0	0	Spring
4/24/2020	BL	8A	4	2020	Year-3		1	Spring
4/24/2020	BL	9A	4	2020	Year-3		1	Spring
4/24/2020	BL	11A	4	2020	Year-3		1	Spring
4/24/2020	BL	13A	4	2020	Year-3		1	Spring
4/25/2020	BL	1M	4	2020	Year-3	0	0	Spring
4/25/2020	BL	2M	4	2020	Year-3	0	0	Spring
4/25/2020	BL	3M	4	2020	Year-3	0	0	Spring
4/25/2020	BL	4M	4	2020	Year-3	0	0	Spring
4/25/2020	BL	5M	4	2020	Year-3	0	0	Spring
4/25/2020	BL	6M	4	2020	Year-3	0	0	Spring
4/25/2020	BL	7M	4	2020	Year-3		1	Spring
4/25/2020	BL	10M	4	2020	Year-3	0	0	Spring
4/25/2020	BL	12M	4	2020	Year-3	0	0	Spring
4/25/2020	BL	14M	4	2020	Year-3	0	0	Spring
4/25/2020	BL	8A	4	2020	Year-3		1	Spring
4/25/2020	BL	9A	4	2020	Year-3		1	Spring
4/25/2020	BL	11A	4	2020	Year-3		1	Spring
4/25/2020	BL	13A	4	2020	Year-3		1	Spring
4/26/2020	BL	1M	4	2020	Year-3	0	0	Spring
4/26/2020	BL	2M	4	2020	Year-3	0	0	Spring
4/26/2020	BL	3M	4	2020	Year-3	0	0	Spring
4/26/2020	BL	4M	4	2020	Year-3	0	0	Spring
4/26/2020	BL	5M	4	2020	Year-3	0	0	Spring
4/26/2020	BL	6M	4	2020	Year-3	0	0	Spring
4/26/2020	BL	7M	4	2020	Year-3		1	Spring

Date	Species	Site	Month	Year	Survey Year	Presence	Data Gap	Season
4/26/2020	BL	10M	4	2020	Year-3	0	0	Spring
4/26/2020	BL	12M	4	2020	Year-3	0	0	Spring
4/26/2020	BL	14M	4	2020	Year-3	0	0	Spring
4/26/2020	BL	8A	4	2020	Year-3		1	Spring
4/26/2020	BL	9A	4	2020	Year-3		1	Spring
4/26/2020	BL	11A	4	2020	Year-3		1	Spring
4/26/2020	BL	13A	4	2020	Year-3		1	Spring
4/27/2020	BL	1M	4	2020	Year-3	0	0	Spring
4/27/2020	BL	2M	4	2020	Year-3	0	0	Spring
4/27/2020	BL	3M	4	2020	Year-3	0	0	Spring
4/27/2020	BL	4M	4	2020	Year-3	0	0	Spring
4/27/2020	BL	5M	4	2020	Year-3	0	0	Spring
4/27/2020	BL	6M	4	2020	Year-3	0	0	Spring
4/27/2020	BL	7M	4	2020	Year-3		1	Spring
4/27/2020	BL	10M	4	2020	Year-3	0	0	Spring
4/27/2020	BL	12M	4	2020	Year-3	0	0	Spring
4/27/2020	BL	14M	4	2020	Year-3	0	0	Spring
4/27/2020	BL	8A	4	2020	Year-3		1	Spring
4/27/2020	BL	9A	4	2020	Year-3		1	Spring
4/27/2020	BL	11A	4	2020	Year-3		1	Spring
4/27/2020	BL	13A	4	2020	Year-3		1	Spring
4/28/2020	BL	1M	4	2020	Year-3	0	0	Spring
4/28/2020	BL	2M	4	2020	Year-3	0	0	Spring
4/28/2020	BL	3M	4	2020	Year-3	0	0	Spring
4/28/2020	BL	4M	4	2020	Year-3	0	0	Spring
4/28/2020	BL	5M	4	2020	Year-3	0	0	Spring
4/28/2020	BL	6M	4	2020	Year-3	0	0	Spring
4/28/2020	BL	7M	4	2020	Year-3		1	Spring
4/28/2020	BL	10M	4	2020	Year-3	0	0	Spring
4/28/2020	BL	12M	4	2020	Year-3	0	0	Spring
4/28/2020	BL	14M	4	2020	Year-3	0	0	Spring
4/28/2020	BL	8A	4	2020	Year-3		1	Spring
4/28/2020	BL	9A	4	2020	Year-3		1	Spring
4/28/2020	BL	11A	4	2020	Year-3		1	Spring
4/28/2020	BL	13A	4	2020	Year-3		1	Spring
4/29/2020	BL	1M	4	2020	Year-3	0	0	Spring
4/29/2020	BL	2M	4	2020	Year-3	0	0	Spring
4/29/2020	BL	3M	4	2020	Year-3	0	0	Spring
4/29/2020	BL	4M	4	2020	Year-3	0	0	Spring
4/29/2020	BL	5M	4	2020	Year-3	0	0	Spring
4/29/2020	BL	6M	4	2020	Year-3	0	0	Spring
4/29/2020	BL	7M	4	2020	Year-3		1	Spring
4/29/2020	BL	10M	4	2020	Year-3	0	0	Spring
4/29/2020	BL	12M	4	2020	Year-3	0	0	Spring

Date	Species	Site	Month	Year	Survey Year	Presence	Data Gap	Season
4/29/2020	BL	14M	4	2020	Year-3	0	0	Spring
4/29/2020	BL	8A	4	2020	Year-3		1	Spring
4/29/2020	BL	9A	4	2020	Year-3		1	Spring
4/29/2020	BL	11A	4	2020	Year-3		1	Spring
4/29/2020	BL	13A	4	2020	Year-3		1	Spring
4/30/2020	BL	1M	4	2020	Year-3	0	0	Spring
4/30/2020	BL	2M	4	2020	Year-3	0	0	Spring
4/30/2020	BL	3M	4	2020	Year-3	0	0	Spring
4/30/2020	BL	4M	4	2020	Year-3	0	0	Spring
4/30/2020	BL	5M	4	2020	Year-3	0	0	Spring
4/30/2020	BL	6M	4	2020	Year-3	0	0	Spring
4/30/2020	BL	7M	4	2020	Year-3		1	Spring
4/30/2020	BL	10M	4	2020	Year-3	0	0	Spring
4/30/2020	BL	12M	4	2020	Year-3	0	0	Spring
4/30/2020	BL	14M	4	2020	Year-3	0	0	Spring
4/30/2020	BL	8A	4	2020	Year-3		1	Spring
4/30/2020	BL	9A	4	2020	Year-3		1	Spring
4/30/2020	BL	11A	4	2020	Year-3		1	Spring
4/30/2020	BL	13A	4	2020	Year-3		1	Spring
5/1/2020	BL	1M	5	2020	Year-3	0	0	Spring
5/1/2020	BL	2M	5	2020	Year-3	0	0	Spring
5/1/2020	BL	3M	5	2020	Year-3	0	0	Spring
5/1/2020	BL	4M	5	2020	Year-3	0	0	Spring
5/1/2020	BL	5M	5	2020	Year-3	0	0	Spring
5/1/2020	BL	6M	5	2020	Year-3	0	0	Spring
5/1/2020	BL	7M	5	2020	Year-3		1	Spring
5/1/2020	BL	10M	5	2020	Year-3	0	0	Spring
5/1/2020	BL	12M	5	2020	Year-3	0	0	Spring
5/1/2020	BL	14M	5	2020	Year-3	0	0	Spring
5/1/2020	BL	8A	5	2020	Year-3		1	Spring
5/1/2020	BL	9A	5	2020	Year-3		1	Spring
5/1/2020	BL	11A	5	2020	Year-3		1	Spring
5/1/2020	BL	13A	5	2020	Year-3		1	Spring
5/2/2020	BL	1M	5	2020	Year-3	0	0	Spring
5/2/2020	BL	2M	5	2020	Year-3	0	0	Spring
5/2/2020	BL	3M	5	2020	Year-3	0	0	Spring
5/2/2020	BL	4M	5	2020	Year-3	0	0	Spring
5/2/2020	BL	5M	5	2020	Year-3	0	0	Spring
5/2/2020	BL	6M	5	2020	Year-3	0	0	Spring
5/2/2020	BL	7M	5	2020	Year-3		1	Spring
5/2/2020	BL	10M	5	2020	Year-3	0	0	Spring
5/2/2020	BL	12M	5	2020	Year-3	0	0	Spring
5/2/2020	BL	14M	5	2020	Year-3	0	0	Spring
5/2/2020	BL	8A	5	2020	Year-3		1	Spring

Date	Species	Site	Month	Year	Survey Year	Presence	Data Gap	Season
5/2/2020	BL	9A	5	2020	Year-3		1	Spring
5/2/2020	BL	11A	5	2020	Year-3		1	Spring
5/2/2020	BL	13A	5	2020	Year-3		1	Spring
5/3/2020	BL	1M	5	2020	Year-3	0	0	Spring
5/3/2020	BL	2M	5	2020	Year-3	0	0	Spring
5/3/2020	BL	3M	5	2020	Year-3	0	0	Spring
5/3/2020	BL	4M	5	2020	Year-3	0	0	Spring
5/3/2020	BL	5M	5	2020	Year-3	0	0	Spring
5/3/2020	BL	6M	5	2020	Year-3	0	0	Spring
5/3/2020	BL	7M	5	2020	Year-3		1	Spring
5/3/2020	BL	10M	5	2020	Year-3	0	0	Spring
5/3/2020	BL	12M	5	2020	Year-3	0	0	Spring
5/3/2020	BL	14M	5	2020	Year-3	0	0	Spring
5/3/2020	BL	8A	5	2020	Year-3		1	Spring
5/3/2020	BL	9A	5	2020	Year-3		1	Spring
5/3/2020	BL	11A	5	2020	Year-3		1	Spring
5/3/2020	BL	13A	5	2020	Year-3		1	Spring
5/4/2020	BL	1M	5	2020	Year-3	0	0	Spring
5/4/2020	BL	2M	5	2020	Year-3	0	0	Spring
5/4/2020	BL	3M	5	2020	Year-3	0	0	Spring
5/4/2020	BL	4M	5	2020	Year-3	0	0	Spring
5/4/2020	BL	5M	5	2020	Year-3	0	0	Spring
5/4/2020	BL	6M	5	2020	Year-3	0	0	Spring
5/4/2020	BL	7M	5	2020	Year-3		1	Spring
5/4/2020	BL	10M	5	2020	Year-3	0	0	Spring
5/4/2020	BL	12M	5	2020	Year-3	0	0	Spring
5/4/2020	BL	14M	5	2020	Year-3	0	0	Spring
5/4/2020	BL	8A	5	2020	Year-3		1	Spring
5/4/2020	BL	9A	5	2020	Year-3		1	Spring
5/4/2020	BL	11A	5	2020	Year-3		1	Spring
5/4/2020	BL	13A	5	2020	Year-3		1	Spring
5/5/2020	BL	1M	5	2020	Year-3	0	0	Spring
5/5/2020	BL	2M	5	2020	Year-3	0	0	Spring
5/5/2020	BL	3M	5	2020	Year-3	0	0	Spring
5/5/2020	BL	4M	5	2020	Year-3	0	0	Spring
5/5/2020	BL	5M	5	2020	Year-3	0	0	Spring
5/5/2020	BL	6M	5	2020	Year-3	0	0	Spring
5/5/2020	BL	7M	5	2020	Year-3		1	Spring
5/5/2020	BL	10M	5	2020	Year-3	0	0	Spring
5/5/2020	BL	12M	5	2020	Year-3	0	0	Spring
5/5/2020	BL	14M	5	2020	Year-3	0	0	Spring
5/5/2020	BL	8A	5	2020	Year-3		1	Spring
5/5/2020	BL	9A	5	2020	Year-3		1	Spring
5/5/2020	BL	11A	5	2020	Year-3		1	Spring

Date	Species	Site	Month	Year	Survey Year	Presence	Data Gap	Season
5/5/2020	BL	13A	5	2020	Year-3		1	Spring
5/6/2020	BL	1M	5	2020	Year-3	0	0	Spring
5/6/2020	BL	2M	5	2020	Year-3	0	0	Spring
5/6/2020	BL	3M	5	2020	Year-3	0	0	Spring
5/6/2020	BL	4M	5	2020	Year-3	0	0	Spring
5/6/2020	BL	5M	5	2020	Year-3	0	0	Spring
5/6/2020	BL	6M	5	2020	Year-3	0	0	Spring
5/6/2020	BL	7M	5	2020	Year-3		1	Spring
5/6/2020	BL	10M	5	2020	Year-3	0	0	Spring
5/6/2020	BL	12M	5	2020	Year-3	0	0	Spring
5/6/2020	BL	14M	5	2020	Year-3	0	0	Spring
5/6/2020	BL	8A	5	2020	Year-3		1	Spring
5/6/2020	BL	9A	5	2020	Year-3		1	Spring
5/6/2020	BL	11A	5	2020	Year-3		1	Spring
5/6/2020	BL	13A	5	2020	Year-3		1	Spring
5/7/2020	BL	1M	5	2020	Year-3	0	0	Spring
5/7/2020	BL	2M	5	2020	Year-3	0	0	Spring
5/7/2020	BL	3M	5	2020	Year-3	0	0	Spring
5/7/2020	BL	4M	5	2020	Year-3	0	0	Spring
5/7/2020	BL	5M	5	2020	Year-3	0	0	Spring
5/7/2020	BL	6M	5	2020	Year-3	0	0	Spring
5/7/2020	BL	7M	5	2020	Year-3		1	Spring
5/7/2020	BL	10M	5	2020	Year-3	0	0	Spring
5/7/2020	BL	12M	5	2020	Year-3	0	0	Spring
5/7/2020	BL	14M	5	2020	Year-3	0	0	Spring
5/7/2020	BL	8A	5	2020	Year-3		1	Spring
5/7/2020	BL	9A	5	2020	Year-3		1	Spring
5/7/2020	BL	11A	5	2020	Year-3		1	Spring
5/7/2020	BL	13A	5	2020	Year-3		1	Spring
5/8/2020	BL	1M	5	2020	Year-3	0	0	Spring
5/8/2020	BL	2M	5	2020	Year-3	0	0	Spring
5/8/2020	BL	3M	5	2020	Year-3	0	0	Spring
5/8/2020	BL	4M	5	2020	Year-3	0	0	Spring
5/8/2020	BL	5M	5	2020	Year-3	0	0	Spring
5/8/2020	BL	6M	5	2020	Year-3	0	0	Spring
5/8/2020	BL	7M	5	2020	Year-3		1	Spring
5/8/2020	BL	10M	5	2020	Year-3	0	0	Spring
5/8/2020	BL	12M	5	2020	Year-3	0	0	Spring
5/8/2020	BL	14M	5	2020	Year-3	0	0	Spring
5/8/2020	BL	8A	5	2020	Year-3		1	Spring
5/8/2020	BL	9A	5	2020	Year-3		1	Spring
5/8/2020	BL	11A	5	2020	Year-3		1	Spring
5/8/2020	BL	13A	5	2020	Year-3		1	Spring
5/9/2020	BL	1M	5	2020	Year-3	0	0	Spring

Date	Species	Site	Month	Year	Survey Year	Presence	Data Gap	Season
5/9/2020	BL	2M	5	2020	Year-3	0	0	Spring
5/9/2020	BL	3M	5	2020	Year-3	0	0	Spring
5/9/2020	BL	4M	5	2020	Year-3	0	0	Spring
5/9/2020	BL	5M	5	2020	Year-3	0	0	Spring
5/9/2020	BL	6M	5	2020	Year-3	0	0	Spring
5/9/2020	BL	7M	5	2020	Year-3		1	Spring
5/9/2020	BL	10M	5	2020	Year-3	0	0	Spring
5/9/2020	BL	12M	5	2020	Year-3	0	0	Spring
5/9/2020	BL	14M	5	2020	Year-3	0	0	Spring
5/9/2020	BL	8A	5	2020	Year-3		1	Spring
5/9/2020	BL	9A	5	2020	Year-3		1	Spring
5/9/2020	BL	11A	5	2020	Year-3		1	Spring
5/9/2020	BL	13A	5	2020	Year-3		1	Spring
5/10/2020	BL	1M	5	2020	Year-3	0	0	Spring
5/10/2020	BL	2M	5	2020	Year-3	0	0	Spring
5/10/2020	BL	3M	5	2020	Year-3	0	0	Spring
5/10/2020	BL	4M	5	2020	Year-3	0	0	Spring
5/10/2020	BL	5M	5	2020	Year-3	0	0	Spring
5/10/2020	BL	6M	5	2020	Year-3	0	0	Spring
5/10/2020	BL	7M	5	2020	Year-3		1	Spring
5/10/2020	BL	10M	5	2020	Year-3	0	0	Spring
5/10/2020	BL	12M	5	2020	Year-3	0	0	Spring
5/10/2020	BL	14M	5	2020	Year-3	0	0	Spring
5/10/2020	BL	8A	5	2020	Year-3		1	Spring
5/10/2020	BL	9A	5	2020	Year-3		1	Spring
5/10/2020	BL	11A	5	2020	Year-3		1	Spring
5/10/2020	BL	13A	5	2020	Year-3		1	Spring
5/11/2020	BL	1M	5	2020	Year-3	0	0	Spring
5/11/2020	BL	2M	5	2020	Year-3	0	0	Spring
5/11/2020	BL	3M	5	2020	Year-3	0	0	Spring
5/11/2020	BL	4M	5	2020	Year-3	0	0	Spring
5/11/2020	BL	5M	5	2020	Year-3	0	0	Spring
5/11/2020	BL	6M	5	2020	Year-3	0	0	Spring
5/11/2020	BL	7M	5	2020	Year-3		1	Spring
5/11/2020	BL	10M	5	2020	Year-3	0	0	Spring
5/11/2020	BL	12M	5	2020	Year-3	0	0	Spring
5/11/2020	BL	14M	5	2020	Year-3	0	0	Spring
5/11/2020	BL	8A	5	2020	Year-3		1	Spring
5/11/2020	BL	9A	5	2020	Year-3		1	Spring
5/11/2020	BL	11A	5	2020	Year-3		1	Spring
5/11/2020	BL	13A	5	2020	Year-3		1	Spring
5/12/2020	BL	1M	5	2020	Year-3	0	0	Spring
5/12/2020	BL	2M	5	2020	Year-3	0	0	Spring
5/12/2020	BL	3M	5	2020	Year-3	0	0	Spring

Date	Species	Site	Month	Year	Survey Year	Presence	Data Gap	Season
5/12/2020	BL	4M	5	2020	Year-3	0	0	Spring
5/12/2020	BL	5M	5	2020	Year-3	0	0	Spring
5/12/2020	BL	6M	5	2020	Year-3	0	0	Spring
5/12/2020	BL	7M	5	2020	Year-3		1	Spring
5/12/2020	BL	10M	5	2020	Year-3	0	0	Spring
5/12/2020	BL	12M	5	2020	Year-3	0	0	Spring
5/12/2020	BL	14M	5	2020	Year-3	0	0	Spring
5/12/2020	BL	8A	5	2020	Year-3		1	Spring
5/12/2020	BL	9A	5	2020	Year-3		1	Spring
5/12/2020	BL	11A	5	2020	Year-3		1	Spring
5/12/2020	BL	13A	5	2020	Year-3		1	Spring
5/13/2020	BL	1M	5	2020	Year-3	0	0	Spring
5/13/2020	BL	2M	5	2020	Year-3	0	0	Spring
5/13/2020	BL	3M	5	2020	Year-3	0	0	Spring
5/13/2020	BL	4M	5	2020	Year-3	0	0	Spring
5/13/2020	BL	5M	5	2020	Year-3	0	0	Spring
5/13/2020	BL	6M	5	2020	Year-3	0	0	Spring
5/13/2020	BL	7M	5	2020	Year-3		1	Spring
5/13/2020	BL	10M	5	2020	Year-3	0	0	Spring
5/13/2020	BL	12M	5	2020	Year-3	0	0	Spring
5/13/2020	BL	14M	5	2020	Year-3	0	0	Spring
5/13/2020	BL	8A	5	2020	Year-3		1	Spring
5/13/2020	BL	9A	5	2020	Year-3		1	Spring
5/13/2020	BL	11A	5	2020	Year-3		1	Spring
5/13/2020	BL	13A	5	2020	Year-3		1	Spring
5/14/2020	BL	1M	5	2020	Year-3	0	0	Spring
5/14/2020	BL	2M	5	2020	Year-3	0	0	Spring
5/14/2020	BL	3M	5	2020	Year-3	0	0	Spring
5/14/2020	BL	4M	5	2020	Year-3		1	Spring
5/14/2020	BL	5M	5	2020	Year-3	0	0	Spring
5/14/2020	BL	6M	5	2020	Year-3	0	0	Spring
5/14/2020	BL	7M	5	2020	Year-3		1	Spring
5/14/2020	BL	10M	5	2020	Year-3	0	0	Spring
5/14/2020	BL	12M	5	2020	Year-3	0	0	Spring
5/14/2020	BL	14M	5	2020	Year-3	0	0	Spring
5/14/2020	BL	8A	5	2020	Year-3		1	Spring
5/14/2020	BL	9A	5	2020	Year-3		1	Spring
5/14/2020	BL	11A	5	2020	Year-3		1	Spring
5/14/2020	BL	13A	5	2020	Year-3		1	Spring
5/15/2020	BL	1M	5	2020	Year-3	0	0	Spring
5/15/2020	BL	2M	5	2020	Year-3	0	0	Spring
5/15/2020	BL	3M	5	2020	Year-3	0	0	Spring
5/15/2020	BL	4M	5	2020	Year-3		1	Spring
5/15/2020	BL	5M	5	2020	Year-3	0	0	Spring

Date	Species	Site	Month	Year	Survey Year	Presence	Data Gap	Season
5/15/2020	BL	6M	5	2020	Year-3	0	0	Spring
5/15/2020	BL	7M	5	2020	Year-3		1	Spring
5/15/2020	BL	10M	5	2020	Year-3	0	0	Spring
5/15/2020	BL	12M	5	2020	Year-3	0	0	Spring
5/15/2020	BL	14M	5	2020	Year-3	0	0	Spring
5/15/2020	BL	8A	5	2020	Year-3		1	Spring
5/15/2020	BL	9A	5	2020	Year-3		1	Spring
5/15/2020	BL	11A	5	2020	Year-3		1	Spring
5/15/2020	BL	13A	5	2020	Year-3		1	Spring
5/16/2020	BL	1M	5	2020	Year-3	0	0	Spring
5/16/2020	BL	2M	5	2020	Year-3	0	0	Spring
5/16/2020	BL	3M	5	2020	Year-3	0	0	Spring
5/16/2020	BL	4M	5	2020	Year-3		1	Spring
5/16/2020	BL	5M	5	2020	Year-3	0	0	Spring
5/16/2020	BL	6M	5	2020	Year-3	0	0	Spring
5/16/2020	BL	7M	5	2020	Year-3		1	Spring
5/16/2020	BL	10M	5	2020	Year-3	0	0	Spring
5/16/2020	BL	12M	5	2020	Year-3	0	0	Spring
5/16/2020	BL	14M	5	2020	Year-3	0	0	Spring
5/16/2020	BL	8A	5	2020	Year-3		1	Spring
5/16/2020	BL	9A	5	2020	Year-3		1	Spring
5/16/2020	BL	11A	5	2020	Year-3		1	Spring
5/16/2020	BL	13A	5	2020	Year-3		1	Spring
5/17/2020	BL	1M	5	2020	Year-3	0	0	Spring
5/17/2020	BL	2M	5	2020	Year-3	0	0	Spring
5/17/2020	BL	3M	5	2020	Year-3	0	0	Spring
5/17/2020	BL	4M	5	2020	Year-3		1	Spring
5/17/2020	BL	5M	5	2020	Year-3	0	0	Spring
5/17/2020	BL	6M	5	2020	Year-3	0	0	Spring
5/17/2020	BL	7M	5	2020	Year-3		1	Spring
5/17/2020	BL	10M	5	2020	Year-3	0	0	Spring
5/17/2020	BL	12M	5	2020	Year-3	0	0	Spring
5/17/2020	BL	14M	5	2020	Year-3	0	0	Spring
5/17/2020	BL	8A	5	2020	Year-3		1	Spring
5/17/2020	BL	9A	5	2020	Year-3		1	Spring
5/17/2020	BL	11A	5	2020	Year-3		1	Spring
5/17/2020	BL	13A	5	2020	Year-3		1	Spring
5/18/2020	BL	1M	5	2020	Year-3	0	0	Spring
5/18/2020	BL	2M	5	2020	Year-3	0	0	Spring
5/18/2020	BL	3M	5	2020	Year-3	0	0	Spring
5/18/2020	BL	4M	5	2020	Year-3		1	Spring
5/18/2020	BL	5M	5	2020	Year-3	0	0	Spring
5/18/2020	BL	6M	5	2020	Year-3	0	0	Spring
5/18/2020	BL	7M	5	2020	Year-3		1	Spring

Date	Species	Site	Month	Year	Survey Year	Presence	Data Gap	Season
5/18/2020	BL	10M	5	2020	Year-3	0	0	Spring
5/18/2020	BL	12M	5	2020	Year-3	0	0	Spring
5/18/2020	BL	14M	5	2020	Year-3	0	0	Spring
5/18/2020	BL	8A	5	2020	Year-3		1	Spring
5/18/2020	BL	9A	5	2020	Year-3		1	Spring
5/18/2020	BL	11A	5	2020	Year-3		1	Spring
5/18/2020	BL	13A	5	2020	Year-3		1	Spring
5/19/2020	BL	1M	5	2020	Year-3	0	0	Spring
5/19/2020	BL	2M	5	2020	Year-3	0	0	Spring
5/19/2020	BL	3M	5	2020	Year-3	0	0	Spring
5/19/2020	BL	4M	5	2020	Year-3		1	Spring
5/19/2020	BL	5M	5	2020	Year-3	0	0	Spring
5/19/2020	BL	6M	5	2020	Year-3	0	0	Spring
5/19/2020	BL	7M	5	2020	Year-3		1	Spring
5/19/2020	BL	10M	5	2020	Year-3	0	0	Spring
5/19/2020	BL	12M	5	2020	Year-3	0	0	Spring
5/19/2020	BL	14M	5	2020	Year-3	0	0	Spring
5/19/2020	BL	8A	5	2020	Year-3		1	Spring
5/19/2020	BL	9A	5	2020	Year-3		1	Spring
5/19/2020	BL	11A	5	2020	Year-3		1	Spring
5/19/2020	BL	13A	5	2020	Year-3		1	Spring
5/20/2020	BL	1M	5	2020	Year-3	0	0	Spring
5/20/2020	BL	2M	5	2020	Year-3	0	0	Spring
5/20/2020	BL	3M	5	2020	Year-3	0	0	Spring
5/20/2020	BL	4M	5	2020	Year-3		1	Spring
5/20/2020	BL	5M	5	2020	Year-3	0	0	Spring
5/20/2020	BL	6M	5	2020	Year-3	0	0	Spring
5/20/2020	BL	7M	5	2020	Year-3		1	Spring
5/20/2020	BL	10M	5	2020	Year-3	0	0	Spring
5/20/2020	BL	12M	5	2020	Year-3	0	0	Spring
5/20/2020	BL	14M	5	2020	Year-3	0	0	Spring
5/20/2020	BL	8A	5	2020	Year-3		1	Spring
5/20/2020	BL	9A	5	2020	Year-3		1	Spring
5/20/2020	BL	11A	5	2020	Year-3		1	Spring
5/20/2020	BL	13A	5	2020	Year-3		1	Spring
5/21/2020	BL	1M	5	2020	Year-3	0	0	Spring
5/21/2020	BL	2M	5	2020	Year-3	0	0	Spring
5/21/2020	BL	3M	5	2020	Year-3	0	0	Spring
5/21/2020	BL	4M	5	2020	Year-3		1	Spring
5/21/2020	BL	5M	5	2020	Year-3	0	0	Spring
5/21/2020	BL	6M	5	2020	Year-3	0	0	Spring
5/21/2020	BL	7M	5	2020	Year-3		1	Spring
5/21/2020	BL	10M	5	2020	Year-3	0	0	Spring
5/21/2020	BL	12M	5	2020	Year-3	0	0	Spring

Date	Species	Site	Month	Year	Survey Year	Presence	Data Gap	Season
5/21/2020	BL	14M	5	2020	Year-3	0	0	Spring
5/21/2020	BL	8A	5	2020	Year-3		1	Spring
5/21/2020	BL	9A	5	2020	Year-3		1	Spring
5/21/2020	BL	11A	5	2020	Year-3		1	Spring
5/21/2020	BL	13A	5	2020	Year-3		1	Spring
5/22/2020	BL	1M	5	2020	Year-3	0	0	Spring
5/22/2020	BL	2M	5	2020	Year-3	0	0	Spring
5/22/2020	BL	3M	5	2020	Year-3	0	0	Spring
5/22/2020	BL	4M	5	2020	Year-3		1	Spring
5/22/2020	BL	5M	5	2020	Year-3	0	0	Spring
5/22/2020	BL	6M	5	2020	Year-3	0	0	Spring
5/22/2020	BL	7M	5	2020	Year-3		1	Spring
5/22/2020	BL	10M	5	2020	Year-3	0	0	Spring
5/22/2020	BL	12M	5	2020	Year-3	0	0	Spring
5/22/2020	BL	14M	5	2020	Year-3	0	0	Spring
5/22/2020	BL	8A	5	2020	Year-3		1	Spring
5/22/2020	BL	9A	5	2020	Year-3		1	Spring
5/22/2020	BL	11A	5	2020	Year-3		1	Spring
5/22/2020	BL	13A	5	2020	Year-3		1	Spring
5/23/2020	BL	1M	5	2020	Year-3	0	0	Spring
5/23/2020	BL	2M	5	2020	Year-3	0	0	Spring
5/23/2020	BL	3M	5	2020	Year-3	0	0	Spring
5/23/2020	BL	4M	5	2020	Year-3		1	Spring
5/23/2020	BL	5M	5	2020	Year-3	0	0	Spring
5/23/2020	BL	6M	5	2020	Year-3	0	0	Spring
5/23/2020	BL	7M	5	2020	Year-3		1	Spring
5/23/2020	BL	10M	5	2020	Year-3	0	0	Spring
5/23/2020	BL	12M	5	2020	Year-3	0	0	Spring
5/23/2020	BL	14M	5	2020	Year-3	0	0	Spring
5/23/2020	BL	8A	5	2020	Year-3		1	Spring
5/23/2020	BL	9A	5	2020	Year-3		1	Spring
5/23/2020	BL	11A	5	2020	Year-3		1	Spring
5/23/2020	BL	13A	5	2020	Year-3		1	Spring
5/24/2020	BL	1M	5	2020	Year-3	0	0	Spring
5/24/2020	BL	2M	5	2020	Year-3	0	0	Spring
5/24/2020	BL	3M	5	2020	Year-3	0	0	Spring
5/24/2020	BL	4M	5	2020	Year-3		1	Spring
5/24/2020	BL	5M	5	2020	Year-3	0	0	Spring
5/24/2020	BL	6M	5	2020	Year-3	0	0	Spring
5/24/2020	BL	7M	5	2020	Year-3		1	Spring
5/24/2020	BL	10M	5	2020	Year-3	0	0	Spring
5/24/2020	BL	12M	5	2020	Year-3	0	0	Spring
5/24/2020	BL	14M	5	2020	Year-3	0	0	Spring
5/24/2020	BL	8A	5	2020	Year-3		1	Spring

Date	Species	Site	Month	Year	Survey Year	Presence	Data Gap	Season
5/24/2020	BL	9A	5	2020	Year-3		1	Spring
5/24/2020	BL	11A	5	2020	Year-3		1	Spring
5/24/2020	BL	13A	5	2020	Year-3		1	Spring
5/25/2020	BL	1M	5	2020	Year-3	0	0	Spring
5/25/2020	BL	2M	5	2020	Year-3	0	0	Spring
5/25/2020	BL	3M	5	2020	Year-3	0	0	Spring
5/25/2020	BL	4M	5	2020	Year-3		1	Spring
5/25/2020	BL	5M	5	2020	Year-3	0	0	Spring
5/25/2020	BL	6M	5	2020	Year-3	0	0	Spring
5/25/2020	BL	7M	5	2020	Year-3		1	Spring
5/25/2020	BL	10M	5	2020	Year-3	0	0	Spring
5/25/2020	BL	12M	5	2020	Year-3	0	0	Spring
5/25/2020	BL	14M	5	2020	Year-3	0	0	Spring
5/25/2020	BL	8A	5	2020	Year-3		1	Spring
5/25/2020	BL	9A	5	2020	Year-3		1	Spring
5/25/2020	BL	11A	5	2020	Year-3		1	Spring
5/25/2020	BL	13A	5	2020	Year-3		1	Spring
5/26/2020	BL	1M	5	2020	Year-3	0	0	Spring
5/26/2020	BL	2M	5	2020	Year-3	0	0	Spring
5/26/2020	BL	3M	5	2020	Year-3	0	0	Spring
5/26/2020	BL	4M	5	2020	Year-3		1	Spring
5/26/2020	BL	5M	5	2020	Year-3	0	0	Spring
5/26/2020	BL	6M	5	2020	Year-3	0	0	Spring
5/26/2020	BL	7M	5	2020	Year-3		1	Spring
5/26/2020	BL	10M	5	2020	Year-3	0	0	Spring
5/26/2020	BL	12M	5	2020	Year-3	0	0	Spring
5/26/2020	BL	14M	5	2020	Year-3	0	0	Spring
5/26/2020	BL	8A	5	2020	Year-3		1	Spring
5/26/2020	BL	9A	5	2020	Year-3		1	Spring
5/26/2020	BL	11A	5	2020	Year-3		1	Spring
5/26/2020	BL	13A	5	2020	Year-3		1	Spring
5/27/2020	BL	1M	5	2020	Year-3	0	0	Spring
5/27/2020	BL	2M	5	2020	Year-3	0	0	Spring
5/27/2020	BL	3M	5	2020	Year-3	0	0	Spring
5/27/2020	BL	4M	5	2020	Year-3		1	Spring
5/27/2020	BL	5M	5	2020	Year-3	0	0	Spring
5/27/2020	BL	6M	5	2020	Year-3	0	0	Spring
5/27/2020	BL	7M	5	2020	Year-3		1	Spring
5/27/2020	BL	10M	5	2020	Year-3	0	0	Spring
5/27/2020	BL	12M	5	2020	Year-3	0	0	Spring
5/27/2020	BL	14M	5	2020	Year-3	0	0	Spring
5/27/2020	BL	8A	5	2020	Year-3		1	Spring
5/27/2020	BL	9A	5	2020	Year-3		1	Spring
5/27/2020	BL	11A	5	2020	Year-3		1	Spring

Date	Species	Site	Month	Year	Survey Year	Presence	Data Gap	Season
5/27/2020	BL	13A	5	2020	Year-3		1	Spring
5/28/2020	BL	1M	5	2020	Year-3	0	0	Spring
5/28/2020	BL	2M	5	2020	Year-3	0	0	Spring
5/28/2020	BL	3M	5	2020	Year-3	0	0	Spring
5/28/2020	BL	4M	5	2020	Year-3		1	Spring
5/28/2020	BL	5M	5	2020	Year-3	0	0	Spring
5/28/2020	BL	6M	5	2020	Year-3	0	0	Spring
5/28/2020	BL	7M	5	2020	Year-3		1	Spring
5/28/2020	BL	10M	5	2020	Year-3	0	0	Spring
5/28/2020	BL	12M	5	2020	Year-3	0	0	Spring
5/28/2020	BL	14M	5	2020	Year-3	0	0	Spring
5/28/2020	BL	8A	5	2020	Year-3		1	Spring
5/28/2020	BL	9A	5	2020	Year-3		1	Spring
5/28/2020	BL	11A	5	2020	Year-3		1	Spring
5/28/2020	BL	13A	5	2020	Year-3		1	Spring
5/29/2020	BL	1M	5	2020	Year-3	0	0	Spring
5/29/2020	BL	2M	5	2020	Year-3	0	0	Spring
5/29/2020	BL	3M	5	2020	Year-3	0	0	Spring
5/29/2020	BL	4M	5	2020	Year-3		1	Spring
5/29/2020	BL	5M	5	2020	Year-3	0	0	Spring
5/29/2020	BL	6M	5	2020	Year-3	0	0	Spring
5/29/2020	BL	7M	5	2020	Year-3		1	Spring
5/29/2020	BL	10M	5	2020	Year-3	0	0	Spring
5/29/2020	BL	12M	5	2020	Year-3	0	0	Spring
5/29/2020	BL	14M	5	2020	Year-3	0	0	Spring
5/29/2020	BL	8A	5	2020	Year-3		1	Spring
5/29/2020	BL	9A	5	2020	Year-3		1	Spring
5/29/2020	BL	11A	5	2020	Year-3		1	Spring
5/29/2020	BL	13A	5	2020	Year-3		1	Spring
5/30/2020	BL	1M	5	2020	Year-3	0	0	Spring
5/30/2020	BL	2M	5	2020	Year-3	0	0	Spring
5/30/2020	BL	3M	5	2020	Year-3	0	0	Spring
5/30/2020	BL	4M	5	2020	Year-3		1	Spring
5/30/2020	BL	5M	5	2020	Year-3	0	0	Spring
5/30/2020	BL	6M	5	2020	Year-3	0	0	Spring
5/30/2020	BL	7M	5	2020	Year-3		1	Spring
5/30/2020	BL	10M	5	2020	Year-3	0	0	Spring
5/30/2020	BL	12M	5	2020	Year-3	0	0	Spring
5/30/2020	BL	14M	5	2020	Year-3	0	0	Spring
5/30/2020	BL	8A	5	2020	Year-3		1	Spring
5/30/2020	BL	9A	5	2020	Year-3		1	Spring
5/30/2020	BL	11A	5	2020	Year-3		1	Spring
5/30/2020	BL	13A	5	2020	Year-3		1	Spring
5/31/2020	BL	1M	5	2020	Year-3	0	0	Spring

Date	Species	Site	Month	Year	Survey Year	Presence	Data Gap	Season
5/31/2020	BL	2M	5	2020	Year-3	0	0	Spring
5/31/2020	BL	3M	5	2020	Year-3	0	0	Spring
5/31/2020	BL	4M	5	2020	Year-3		1	Spring
5/31/2020	BL	5M	5	2020	Year-3	0	0	Spring
5/31/2020	BL	6M	5	2020	Year-3	0	0	Spring
5/31/2020	BL	7M	5	2020	Year-3		1	Spring
5/31/2020	BL	10M	5	2020	Year-3	0	0	Spring
5/31/2020	BL	12M	5	2020	Year-3	0	0	Spring
5/31/2020	BL	14M	5	2020	Year-3	0	0	Spring
5/31/2020	BL	8A	5	2020	Year-3		1	Spring
5/31/2020	BL	9A	5	2020	Year-3		1	Spring
5/31/2020	BL	11A	5	2020	Year-3		1	Spring
5/31/2020	BL	13A	5	2020	Year-3		1	Spring
6/1/2020	BL	1M	6	2020	Year-3	0	0	Summer
6/1/2020	BL	2M	6	2020	Year-3	0	0	Summer
6/1/2020	BL	3M	6	2020	Year-3	0	0	Summer
6/1/2020	BL	4M	6	2020	Year-3		1	Summer
6/1/2020	BL	5M	6	2020	Year-3	0	0	Summer
6/1/2020	BL	6M	6	2020	Year-3	0	0	Summer
6/1/2020	BL	7M	6	2020	Year-3		1	Summer
6/1/2020	BL	10M	6	2020	Year-3	0	0	Summer
6/1/2020	BL	12M	6	2020	Year-3	0	0	Summer
6/1/2020	BL	14M	6	2020	Year-3	0	0	Summer
6/1/2020	BL	8A	6	2020	Year-3		1	Summer
6/1/2020	BL	9A	6	2020	Year-3		1	Summer
6/1/2020	BL	11A	6	2020	Year-3		1	Summer
6/1/2020	BL	13A	6	2020	Year-3		1	Summer
6/2/2020	BL	1M	6	2020	Year-3	0	0	Summer
6/2/2020	BL	2M	6	2020	Year-3	0	0	Summer
6/2/2020	BL	3M	6	2020	Year-3	0	0	Summer
6/2/2020	BL	4M	6	2020	Year-3		1	Summer
6/2/2020	BL	5M	6	2020	Year-3	0	0	Summer
6/2/2020	BL	6M	6	2020	Year-3	0	0	Summer
6/2/2020	BL	7M	6	2020	Year-3		1	Summer
6/2/2020	BL	10M	6	2020	Year-3	0	0	Summer
6/2/2020	BL	12M	6	2020	Year-3	0	0	Summer
6/2/2020	BL	14M	6	2020	Year-3	0	0	Summer
6/2/2020	BL	8A	6	2020	Year-3		1	Summer
6/2/2020	BL	9A	6	2020	Year-3		1	Summer
6/2/2020	BL	11A	6	2020	Year-3		1	Summer
6/2/2020	BL	13A	6	2020	Year-3		1	Summer
6/3/2020	BL	1M	6	2020	Year-3	0	0	Summer
6/3/2020	BL	2M	6	2020	Year-3	0	0	Summer
6/3/2020	BL	3M	6	2020	Year-3	0	0	Summer

Date	Species	Site	Month	Year	Survey Year	Presence	Data Gap	Season
6/3/2020	BL	4M	6	2020	Year-3		1	Summer
6/3/2020	BL	5M	6	2020	Year-3	0	0	Summer
6/3/2020	BL	6M	6	2020	Year-3	0	0	Summer
6/3/2020	BL	7M	6	2020	Year-3		1	Summer
6/3/2020	BL	10M	6	2020	Year-3	0	0	Summer
6/3/2020	BL	12M	6	2020	Year-3	0	0	Summer
6/3/2020	BL	14M	6	2020	Year-3	0	0	Summer
6/3/2020	BL	8A	6	2020	Year-3		1	Summer
6/3/2020	BL	9A	6	2020	Year-3		1	Summer
6/3/2020	BL	11A	6	2020	Year-3		1	Summer
6/3/2020	BL	13A	6	2020	Year-3		1	Summer
6/4/2020	BL	1M	6	2020	Year-3	0	0	Summer
6/4/2020	BL	2M	6	2020	Year-3	0	0	Summer
6/4/2020	BL	3M	6	2020	Year-3	0	0	Summer
6/4/2020	BL	4M	6	2020	Year-3		1	Summer
6/4/2020	BL	5M	6	2020	Year-3	0	0	Summer
6/4/2020	BL	6M	6	2020	Year-3	0	0	Summer
6/4/2020	BL	7M	6	2020	Year-3		1	Summer
6/4/2020	BL	10M	6	2020	Year-3	0	0	Summer
6/4/2020	BL	12M	6	2020	Year-3	0	0	Summer
6/4/2020	BL	14M	6	2020	Year-3	0	0	Summer
6/4/2020	BL	8A	6	2020	Year-3		1	Summer
6/4/2020	BL	9A	6	2020	Year-3		1	Summer
6/4/2020	BL	11A	6	2020	Year-3		1	Summer
6/4/2020	BL	13A	6	2020	Year-3		1	Summer
6/5/2020	BL	1M	6	2020	Year-3	0	0	Summer
6/5/2020	BL	2M	6	2020	Year-3	0	0	Summer
6/5/2020	BL	3M	6	2020	Year-3	0	0	Summer
6/5/2020	BL	4M	6	2020	Year-3		1	Summer
6/5/2020	BL	5M	6	2020	Year-3	0	0	Summer
6/5/2020	BL	6M	6	2020	Year-3	0	0	Summer
6/5/2020	BL	7M	6	2020	Year-3		1	Summer
6/5/2020	BL	10M	6	2020	Year-3	0	0	Summer
6/5/2020	BL	12M	6	2020	Year-3	0	0	Summer
6/5/2020	BL	14M	6	2020	Year-3	0	0	Summer
6/5/2020	BL	8A	6	2020	Year-3		1	Summer
6/5/2020	BL	9A	6	2020	Year-3		1	Summer
6/5/2020	BL	11A	6	2020	Year-3		1	Summer
6/5/2020	BL	13A	6	2020	Year-3		1	Summer
6/6/2020	BL	1M	6	2020	Year-3	0	0	Summer
6/6/2020	BL	2M	6	2020	Year-3	0	0	Summer
6/6/2020	BL	3M	6	2020	Year-3	0	0	Summer
6/6/2020	BL	4M	6	2020	Year-3		1	Summer
6/6/2020	BL	5M	6	2020	Year-3	0	0	Summer

Date	Species	Site	Month	Year	Survey Year	Presence	Data Gap	Season
6/6/2020	BL	6M	6	2020	Year-3	0	0	Summer
6/6/2020	BL	7M	6	2020	Year-3		1	Summer
6/6/2020	BL	10M	6	2020	Year-3	0	0	Summer
6/6/2020	BL	12M	6	2020	Year-3	0	0	Summer
6/6/2020	BL	14M	6	2020	Year-3	0	0	Summer
6/6/2020	BL	8A	6	2020	Year-3		1	Summer
6/6/2020	BL	9A	6	2020	Year-3		1	Summer
6/6/2020	BL	11A	6	2020	Year-3		1	Summer
6/6/2020	BL	13A	6	2020	Year-3		1	Summer
6/7/2020	BL	1M	6	2020	Year-3	0	0	Summer
6/7/2020	BL	2M	6	2020	Year-3	0	0	Summer
6/7/2020	BL	3M	6	2020	Year-3	0	0	Summer
6/7/2020	BL	4M	6	2020	Year-3		1	Summer
6/7/2020	BL	5M	6	2020	Year-3	0	0	Summer
6/7/2020	BL	6M	6	2020	Year-3	0	0	Summer
6/7/2020	BL	7M	6	2020	Year-3		1	Summer
6/7/2020	BL	10M	6	2020	Year-3	0	0	Summer
6/7/2020	BL	12M	6	2020	Year-3	0	0	Summer
6/7/2020	BL	14M	6	2020	Year-3	0	0	Summer
6/7/2020	BL	8A	6	2020	Year-3		1	Summer
6/7/2020	BL	9A	6	2020	Year-3		1	Summer
6/7/2020	BL	11A	6	2020	Year-3		1	Summer
6/7/2020	BL	13A	6	2020	Year-3		1	Summer
6/8/2020	BL	1M	6	2020	Year-3	0	0	Summer
6/8/2020	BL	2M	6	2020	Year-3	0	0	Summer
6/8/2020	BL	3M	6	2020	Year-3	0	0	Summer
6/8/2020	BL	4M	6	2020	Year-3		1	Summer
6/8/2020	BL	5M	6	2020	Year-3	0	0	Summer
6/8/2020	BL	6M	6	2020	Year-3	0	0	Summer
6/8/2020	BL	7M	6	2020	Year-3		1	Summer
6/8/2020	BL	10M	6	2020	Year-3	0	0	Summer
6/8/2020	BL	12M	6	2020	Year-3	0	0	Summer
6/8/2020	BL	14M	6	2020	Year-3	0	0	Summer
6/8/2020	BL	8A	6	2020	Year-3		1	Summer
6/8/2020	BL	9A	6	2020	Year-3		1	Summer
6/8/2020	BL	11A	6	2020	Year-3		1	Summer
6/8/2020	BL	13A	6	2020	Year-3		1	Summer
6/9/2020	BL	1M	6	2020	Year-3	0	0	Summer
6/9/2020	BL	2M	6	2020	Year-3	0	0	Summer
6/9/2020	BL	3M	6	2020	Year-3	0	0	Summer
6/9/2020	BL	4M	6	2020	Year-3		1	Summer
6/9/2020	BL	5M	6	2020	Year-3	0	0	Summer
6/9/2020	BL	6M	6	2020	Year-3	0	0	Summer
6/9/2020	BL	7M	6	2020	Year-3		1	Summer

Date	Species	Site	Month	Year	Survey Year	Presence	Data Gap	Season
6/9/2020	BL	10M	6	2020	Year-3	0	0	Summer
6/9/2020	BL	12M	6	2020	Year-3	0	0	Summer
6/9/2020	BL	14M	6	2020	Year-3	0	0	Summer
6/9/2020	BL	8A	6	2020	Year-3		1	Summer
6/9/2020	BL	9A	6	2020	Year-3		1	Summer
6/9/2020	BL	11A	6	2020	Year-3		1	Summer
6/9/2020	BL	13A	6	2020	Year-3		1	Summer
6/10/2020	BL	1M	6	2020	Year-3	0	0	Summer
6/10/2020	BL	2M	6	2020	Year-3	0	0	Summer
6/10/2020	BL	3M	6	2020	Year-3	0	0	Summer
6/10/2020	BL	4M	6	2020	Year-3		1	Summer
6/10/2020	BL	5M	6	2020	Year-3	0	0	Summer
6/10/2020	BL	6M	6	2020	Year-3	0	0	Summer
6/10/2020	BL	7M	6	2020	Year-3		1	Summer
6/10/2020	BL	10M	6	2020	Year-3	0	0	Summer
6/10/2020	BL	12M	6	2020	Year-3	0	0	Summer
6/10/2020	BL	14M	6	2020	Year-3	0	0	Summer
6/10/2020	BL	8A	6	2020	Year-3		1	Summer
6/10/2020	BL	9A	6	2020	Year-3		1	Summer
6/10/2020	BL	11A	6	2020	Year-3		1	Summer
6/10/2020	BL	13A	6	2020	Year-3		1	Summer
6/11/2020	BL	1M	6	2020	Year-3	0	0	Summer
6/11/2020	BL	2M	6	2020	Year-3	0	0	Summer
6/11/2020	BL	3M	6	2020	Year-3	0	0	Summer
6/11/2020	BL	4M	6	2020	Year-3		1	Summer
6/11/2020	BL	5M	6	2020	Year-3	0	0	Summer
6/11/2020	BL	6M	6	2020	Year-3	0	0	Summer
6/11/2020	BL	7M	6	2020	Year-3		1	Summer
6/11/2020	BL	10M	6	2020	Year-3	0	0	Summer
6/11/2020	BL	12M	6	2020	Year-3	0	0	Summer
6/11/2020	BL	14M	6	2020	Year-3	0	0	Summer
6/11/2020	BL	8A	6	2020	Year-3		1	Summer
6/11/2020	BL	9A	6	2020	Year-3		1	Summer
6/11/2020	BL	11A	6	2020	Year-3		1	Summer
6/11/2020	BL	13A	6	2020	Year-3		1	Summer
6/12/2020	BL	1M	6	2020	Year-3	0	0	Summer
6/12/2020	BL	2M	6	2020	Year-3	0	0	Summer
6/12/2020	BL	3M	6	2020	Year-3	0	0	Summer
6/12/2020	BL	4M	6	2020	Year-3		1	Summer
6/12/2020	BL	5M	6	2020	Year-3	0	0	Summer
6/12/2020	BL	6M	6	2020	Year-3	0	0	Summer
6/12/2020	BL	7M	6	2020	Year-3		1	Summer
6/12/2020	BL	10M	6	2020	Year-3	0	0	Summer
6/12/2020	BL	12M	6	2020	Year-3	0	0	Summer

Date	Species	Site	Month	Year	Survey Year	Presence	Data Gap	Season
6/12/2020	BL	14M	6	2020	Year-3	0	0	Summer
6/12/2020	BL	8A	6	2020	Year-3		1	Summer
6/12/2020	BL	9A	6	2020	Year-3		1	Summer
6/12/2020	BL	11A	6	2020	Year-3		1	Summer
6/12/2020	BL	13A	6	2020	Year-3		1	Summer
6/13/2020	BL	1M	6	2020	Year-3	0	0	Summer
6/13/2020	BL	2M	6	2020	Year-3	0	0	Summer
6/13/2020	BL	3M	6	2020	Year-3	0	0	Summer
6/13/2020	BL	4M	6	2020	Year-3		1	Summer
6/13/2020	BL	5M	6	2020	Year-3	0	0	Summer
6/13/2020	BL	6M	6	2020	Year-3	0	0	Summer
6/13/2020	BL	7M	6	2020	Year-3		1	Summer
6/13/2020	BL	10M	6	2020	Year-3	0	0	Summer
6/13/2020	BL	12M	6	2020	Year-3	0	0	Summer
6/13/2020	BL	14M	6	2020	Year-3	0	0	Summer
6/13/2020	BL	8A	6	2020	Year-3		1	Summer
6/13/2020	BL	9A	6	2020	Year-3		1	Summer
6/13/2020	BL	11A	6	2020	Year-3		1	Summer
6/13/2020	BL	13A	6	2020	Year-3		1	Summer
6/14/2020	BL	1M	6	2020	Year-3	0	0	Summer
6/14/2020	BL	2M	6	2020	Year-3	0	0	Summer
6/14/2020	BL	3M	6	2020	Year-3	0	0	Summer
6/14/2020	BL	4M	6	2020	Year-3		1	Summer
6/14/2020	BL	5M	6	2020	Year-3	0	0	Summer
6/14/2020	BL	6M	6	2020	Year-3	0	0	Summer
6/14/2020	BL	7M	6	2020	Year-3		1	Summer
6/14/2020	BL	10M	6	2020	Year-3	0	0	Summer
6/14/2020	BL	12M	6	2020	Year-3	0	0	Summer
6/14/2020	BL	14M	6	2020	Year-3	0	0	Summer
6/14/2020	BL	8A	6	2020	Year-3		1	Summer
6/14/2020	BL	9A	6	2020	Year-3		1	Summer
6/14/2020	BL	11A	6	2020	Year-3		1	Summer
6/14/2020	BL	13A	6	2020	Year-3		1	Summer
6/15/2020	BL	1M	6	2020	Year-3	0	0	Summer
6/15/2020	BL	2M	6	2020	Year-3	0	0	Summer
6/15/2020	BL	3M	6	2020	Year-3	0	0	Summer
6/15/2020	BL	4M	6	2020	Year-3		1	Summer
6/15/2020	BL	5M	6	2020	Year-3	0	0	Summer
6/15/2020	BL	6M	6	2020	Year-3	0	0	Summer
6/15/2020	BL	7M	6	2020	Year-3		1	Summer
6/15/2020	BL	10M	6	2020	Year-3	0	0	Summer
6/15/2020	BL	12M	6	2020	Year-3	0	0	Summer
6/15/2020	BL	14M	6	2020	Year-3	0	0	Summer
6/15/2020	BL	8A	6	2020	Year-3		1	Summer

Date	Species	Site	Month	Year	Survey Year	Presence	Data Gap	Season
6/15/2020	BL	9A	6	2020	Year-3		1	Summer
6/15/2020	BL	11A	6	2020	Year-3		1	Summer
6/15/2020	BL	13A	6	2020	Year-3		1	Summer
6/16/2020	BL	1M	6	2020	Year-3	0	0	Summer
6/16/2020	BL	2M	6	2020	Year-3	0	0	Summer
6/16/2020	BL	3M	6	2020	Year-3	0	0	Summer
6/16/2020	BL	4M	6	2020	Year-3		1	Summer
6/16/2020	BL	5M	6	2020	Year-3	0	0	Summer
6/16/2020	BL	6M	6	2020	Year-3	0	0	Summer
6/16/2020	BL	7M	6	2020	Year-3		1	Summer
6/16/2020	BL	10M	6	2020	Year-3	0	0	Summer
6/16/2020	BL	12M	6	2020	Year-3	0	0	Summer
6/16/2020	BL	14M	6	2020	Year-3	0	0	Summer
6/16/2020	BL	8A	6	2020	Year-3		1	Summer
6/16/2020	BL	9A	6	2020	Year-3		1	Summer
6/16/2020	BL	11A	6	2020	Year-3		1	Summer
6/16/2020	BL	13A	6	2020	Year-3		1	Summer
6/17/2020	BL	1M	6	2020	Year-3	0	0	Summer
6/17/2020	BL	2M	6	2020	Year-3	0	0	Summer
6/17/2020	BL	3M	6	2020	Year-3	0	0	Summer
6/17/2020	BL	4M	6	2020	Year-3		1	Summer
6/17/2020	BL	5M	6	2020	Year-3	0	0	Summer
6/17/2020	BL	6M	6	2020	Year-3	0	0	Summer
6/17/2020	BL	7M	6	2020	Year-3		1	Summer
6/17/2020	BL	10M	6	2020	Year-3	0	0	Summer
6/17/2020	BL	12M	6	2020	Year-3	0	0	Summer
6/17/2020	BL	14M	6	2020	Year-3	0	0	Summer
6/17/2020	BL	8A	6	2020	Year-3		1	Summer
6/17/2020	BL	9A	6	2020	Year-3		1	Summer
6/17/2020	BL	11A	6	2020	Year-3		1	Summer
6/17/2020	BL	13A	6	2020	Year-3		1	Summer
6/18/2020	BL	1M	6	2020	Year-3	0	0	Summer
6/18/2020	BL	2M	6	2020	Year-3	0	0	Summer
6/18/2020	BL	3M	6	2020	Year-3	0	0	Summer
6/18/2020	BL	4M	6	2020	Year-3		1	Summer
6/18/2020	BL	5M	6	2020	Year-3	0	0	Summer
6/18/2020	BL	6M	6	2020	Year-3	0	0	Summer
6/18/2020	BL	7M	6	2020	Year-3		1	Summer
6/18/2020	BL	10M	6	2020	Year-3	0	0	Summer
6/18/2020	BL	12M	6	2020	Year-3	0	0	Summer
6/18/2020	BL	14M	6	2020	Year-3	0	0	Summer
6/18/2020	BL	8A	6	2020	Year-3		1	Summer
6/18/2020	BL	9A	6	2020	Year-3		1	Summer
6/18/2020	BL	11A	6	2020	Year-3		1	Summer

Date	Species	Site	Month	Year	Survey Year	Presence	Data Gap	Season
6/18/2020	BL	13A	6	2020	Year-3		1	Summer
6/19/2020	BL	1M	6	2020	Year-3	0	0	Summer
6/19/2020	BL	2M	6	2020	Year-3	0	0	Summer
6/19/2020	BL	3M	6	2020	Year-3	0	0	Summer
6/19/2020	BL	4M	6	2020	Year-3		1	Summer
6/19/2020	BL	5M	6	2020	Year-3	0	0	Summer
6/19/2020	BL	6M	6	2020	Year-3	0	0	Summer
6/19/2020	BL	7M	6	2020	Year-3		1	Summer
6/19/2020	BL	10M	6	2020	Year-3	0	0	Summer
6/19/2020	BL	12M	6	2020	Year-3	0	0	Summer
6/19/2020	BL	14M	6	2020	Year-3	0	0	Summer
6/19/2020	BL	8A	6	2020	Year-3		1	Summer
6/19/2020	BL	9A	6	2020	Year-3		1	Summer
6/19/2020	BL	11A	6	2020	Year-3		1	Summer
6/19/2020	BL	13A	6	2020	Year-3		1	Summer
6/20/2020	BL	1M	6	2020	Year-3	0	0	Summer
6/20/2020	BL	2M	6	2020	Year-3	0	0	Summer
6/20/2020	BL	3M	6	2020	Year-3	0	0	Summer
6/20/2020	BL	4M	6	2020	Year-3		1	Summer
6/20/2020	BL	5M	6	2020	Year-3	0	0	Summer
6/20/2020	BL	6M	6	2020	Year-3	0	0	Summer
6/20/2020	BL	7M	6	2020	Year-3		1	Summer
6/20/2020	BL	10M	6	2020	Year-3		1	Summer
6/20/2020	BL	12M	6	2020	Year-3		1	Summer
6/20/2020	BL	14M	6	2020	Year-3		1	Summer
6/20/2020	BL	8A	6	2020	Year-3		1	Summer
6/20/2020	BL	9A	6	2020	Year-3		1	Summer
6/20/2020	BL	11A	6	2020	Year-3		1	Summer
6/20/2020	BL	13A	6	2020	Year-3		1	Summer
6/21/2020	BL	1M	6	2020	Year-3	0	0	Summer
6/21/2020	BL	2M	6	2020	Year-3	0	0	Summer
6/21/2020	BL	3M	6	2020	Year-3	0	0	Summer
6/21/2020	BL	4M	6	2020	Year-3		1	Summer
6/21/2020	BL	5M	6	2020	Year-3		1	Summer
6/21/2020	BL	6M	6	2020	Year-3		1	Summer
6/21/2020	BL	7M	6	2020	Year-3		1	Summer
6/21/2020	BL	10M	6	2020	Year-3	0	0	Summer
6/21/2020	BL	12M	6	2020	Year-3	0	0	Summer
6/21/2020	BL	14M	6	2020	Year-3	0	0	Summer
6/21/2020	BL	8A	6	2020	Year-3		1	Summer
6/21/2020	BL	9A	6	2020	Year-3		1	Summer
6/21/2020	BL	11A	6	2020	Year-3		1	Summer
6/21/2020	BL	13A	6	2020	Year-3		1	Summer
6/22/2020	BL	1M	6	2020	Year-3	0	0	Summer

Date	Species	Site	Month	Year	Survey Year	Presence	Data Gap	Season
6/22/2020	BL	2M	6	2020	Year-3	0	0	Summer
6/22/2020	BL	3M	6	2020	Year-3	0	0	Summer
6/22/2020	BL	4M	6	2020	Year-3		1	Summer
6/22/2020	BL	5M	6	2020	Year-3		1	Summer
6/22/2020	BL	6M	6	2020	Year-3		1	Summer
6/22/2020	BL	7M	6	2020	Year-3		1	Summer
6/22/2020	BL	10M	6	2020	Year-3	0	0	Summer
6/22/2020	BL	12M	6	2020	Year-3	0	0	Summer
6/22/2020	BL	14M	6	2020	Year-3	0	0	Summer
6/22/2020	BL	8A	6	2020	Year-3		1	Summer
6/22/2020	BL	9A	6	2020	Year-3		1	Summer
6/22/2020	BL	11A	6	2020	Year-3		1	Summer
6/22/2020	BL	13A	6	2020	Year-3		1	Summer
6/23/2020	BL	1M	6	2020	Year-3	0	0	Summer
6/23/2020	BL	2M	6	2020	Year-3	0	0	Summer
6/23/2020	BL	3M	6	2020	Year-3	0	0	Summer
6/23/2020	BL	4M	6	2020	Year-3		1	Summer
6/23/2020	BL	5M	6	2020	Year-3		1	Summer
6/23/2020	BL	6M	6	2020	Year-3		1	Summer
6/23/2020	BL	7M	6	2020	Year-3		1	Summer
6/23/2020	BL	10M	6	2020	Year-3	0	0	Summer
6/23/2020	BL	12M	6	2020	Year-3	0	0	Summer
6/23/2020	BL	14M	6	2020	Year-3	0	0	Summer
6/23/2020	BL	8A	6	2020	Year-3		1	Summer
6/23/2020	BL	9A	6	2020	Year-3		1	Summer
6/23/2020	BL	11A	6	2020	Year-3		1	Summer
6/23/2020	BL	13A	6	2020	Year-3		1	Summer
6/24/2020	BL	1M	6	2020	Year-3	0	0	Summer
6/24/2020	BL	2M	6	2020	Year-3	0	0	Summer
6/24/2020	BL	3M	6	2020	Year-3	0	0	Summer
6/24/2020	BL	4M	6	2020	Year-3		1	Summer
6/24/2020	BL	5M	6	2020	Year-3		1	Summer
6/24/2020	BL	6M	6	2020	Year-3		1	Summer
6/24/2020	BL	7M	6	2020	Year-3		1	Summer
6/24/2020	BL	10M	6	2020	Year-3	0	0	Summer
6/24/2020	BL	12M	6	2020	Year-3	0	0	Summer
6/24/2020	BL	14M	6	2020	Year-3	0	0	Summer
6/24/2020	BL	8A	6	2020	Year-3		1	Summer
6/24/2020	BL	9A	6	2020	Year-3		1	Summer
6/24/2020	BL	11A	6	2020	Year-3		1	Summer
6/24/2020	BL	13A	6	2020	Year-3		1	Summer
6/25/2020	BL	1M	6	2020	Year-3	0	0	Summer
6/25/2020	BL	2M	6	2020	Year-3	0	0	Summer
6/25/2020	BL	3M	6	2020	Year-3	0	0	Summer

Date	Species	Site	Month	Year	Survey Year	Presence	Data Gap	Season
6/25/2020	BL	4M	6	2020	Year-3		1	Summer
6/25/2020	BL	5M	6	2020	Year-3		1	Summer
6/25/2020	BL	6M	6	2020	Year-3		1	Summer
6/25/2020	BL	7M	6	2020	Year-3		1	Summer
6/25/2020	BL	10M	6	2020	Year-3	0	0	Summer
6/25/2020	BL	12M	6	2020	Year-3	0	0	Summer
6/25/2020	BL	14M	6	2020	Year-3	0	0	Summer
6/25/2020	BL	8A	6	2020	Year-3		1	Summer
6/25/2020	BL	9A	6	2020	Year-3		1	Summer
6/25/2020	BL	11A	6	2020	Year-3		1	Summer
6/25/2020	BL	13A	6	2020	Year-3		1	Summer
6/26/2020	BL	1M	6	2020	Year-3	0	0	Summer
6/26/2020	BL	2M	6	2020	Year-3		1	Summer
6/26/2020	BL	3M	6	2020	Year-3	0	0	Summer
6/26/2020	BL	4M	6	2020	Year-3		1	Summer
6/26/2020	BL	5M	6	2020	Year-3		1	Summer
6/26/2020	BL	6M	6	2020	Year-3		1	Summer
6/26/2020	BL	7M	6	2020	Year-3		1	Summer
6/26/2020	BL	10M	6	2020	Year-3	0	0	Summer
6/26/2020	BL	12M	6	2020	Year-3	0	0	Summer
6/26/2020	BL	14M	6	2020	Year-3	0	0	Summer
6/26/2020	BL	8A	6	2020	Year-3		1	Summer
6/26/2020	BL	9A	6	2020	Year-3		1	Summer
6/26/2020	BL	11A	6	2020	Year-3		1	Summer
6/26/2020	BL	13A	6	2020	Year-3		1	Summer
6/27/2020	BL	1M	6	2020	Year-3	0	0	Summer
6/27/2020	BL	2M	6	2020	Year-3		1	Summer
6/27/2020	BL	3M	6	2020	Year-3	0	0	Summer
6/27/2020	BL	4M	6	2020	Year-3		1	Summer
6/27/2020	BL	5M	6	2020	Year-3		1	Summer
6/27/2020	BL	6M	6	2020	Year-3		1	Summer
6/27/2020	BL	7M	6	2020	Year-3		1	Summer
6/27/2020	BL	10M	6	2020	Year-3	0	0	Summer
6/27/2020	BL	12M	6	2020	Year-3	0	0	Summer
6/27/2020	BL	14M	6	2020	Year-3	0	0	Summer
6/27/2020	BL	8A	6	2020	Year-3		1	Summer
6/27/2020	BL	9A	6	2020	Year-3		1	Summer
6/27/2020	BL	11A	6	2020	Year-3		1	Summer
6/27/2020	BL	13A	6	2020	Year-3		1	Summer
6/28/2020	BL	1M	6	2020	Year-3		1	Summer
6/28/2020	BL	2M	6	2020	Year-3		1	Summer
6/28/2020	BL	3M	6	2020	Year-3	0	0	Summer
6/28/2020	BL	4M	6	2020	Year-3		1	Summer
6/28/2020	BL	5M	6	2020	Year-3		1	Summer

Date	Species	Site	Month	Year	Survey Year	Presence	Data Gap	Season
6/28/2020	BL	6M	6	2020	Year-3		1	Summer
6/28/2020	BL	7M	6	2020	Year-3		1	Summer
6/28/2020	BL	10M	6	2020	Year-3	0	0	Summer
6/28/2020	BL	12M	6	2020	Year-3	0	0	Summer
6/28/2020	BL	14M	6	2020	Year-3	0	0	Summer
6/28/2020	BL	8A	6	2020	Year-3		1	Summer
6/28/2020	BL	9A	6	2020	Year-3		1	Summer
6/28/2020	BL	11A	6	2020	Year-3		1	Summer
6/28/2020	BL	13A	6	2020	Year-3		1	Summer
6/29/2020	BL	1M	6	2020	Year-3		1	Summer
6/29/2020	BL	2M	6	2020	Year-3		1	Summer
6/29/2020	BL	3M	6	2020	Year-3		1	Summer
6/29/2020	BL	4M	6	2020	Year-3		1	Summer
6/29/2020	BL	5M	6	2020	Year-3		1	Summer
6/29/2020	BL	6M	6	2020	Year-3		1	Summer
6/29/2020	BL	7M	6	2020	Year-3		1	Summer
6/29/2020	BL	10M	6	2020	Year-3	0	0	Summer
6/29/2020	BL	12M	6	2020	Year-3	0	0	Summer
6/29/2020	BL	14M	6	2020	Year-3	0	0	Summer
6/29/2020	BL	8A	6	2020	Year-3		1	Summer
6/29/2020	BL	9A	6	2020	Year-3		1	Summer
6/29/2020	BL	11A	6	2020	Year-3		1	Summer
6/29/2020	BL	13A	6	2020	Year-3		1	Summer
6/30/2020	BL	1M	6	2020	Year-3		1	Summer
6/30/2020	BL	2M	6	2020	Year-3		1	Summer
6/30/2020	BL	3M	6	2020	Year-3		1	Summer
6/30/2020	BL	4M	6	2020	Year-3		1	Summer
6/30/2020	BL	5M	6	2020	Year-3		1	Summer
6/30/2020	BL	6M	6	2020	Year-3		1	Summer
6/30/2020	BL	7M	6	2020	Year-3		1	Summer
6/30/2020	BL	10M	6	2020	Year-3	0	0	Summer
6/30/2020	BL	12M	6	2020	Year-3	0	0	Summer
6/30/2020	BL	14M	6	2020	Year-3	0	0	Summer
6/30/2020	BL	8A	6	2020	Year-3		1	Summer
6/30/2020	BL	9A	6	2020	Year-3		1	Summer
6/30/2020	BL	11A	6	2020	Year-3		1	Summer
6/30/2020	BL	13A	6	2020	Year-3		1	Summer
7/1/2020	BL	1M	7	2020	Year-3		1	Summer
7/1/2020	BL	2M	7	2020	Year-3		1	Summer
7/1/2020	BL	3M	7	2020	Year-3		1	Summer
7/1/2020	BL	4M	7	2020	Year-3		1	Summer
7/1/2020	BL	5M	7	2020	Year-3		1	Summer
7/1/2020	BL	6M	7	2020	Year-3		1	Summer
7/1/2020	BL	7M	7	2020	Year-3		1	Summer

Date	Species	Site	Month	Year	Survey Year	Presence	Data Gap	Season
7/1/2020	BL	10M	7	2020	Year-3	0	0	Summer
7/1/2020	BL	12M	7	2020	Year-3	0	0	Summer
7/1/2020	BL	14M	7	2020	Year-3	0	0	Summer
7/1/2020	BL	8A	7	2020	Year-3		1	Summer
7/1/2020	BL	9A	7	2020	Year-3		1	Summer
7/1/2020	BL	11A	7	2020	Year-3		1	Summer
7/1/2020	BL	13A	7	2020	Year-3		1	Summer
7/2/2020	BL	1M	7	2020	Year-3		1	Summer
7/2/2020	BL	2M	7	2020	Year-3		1	Summer
7/2/2020	BL	3M	7	2020	Year-3		1	Summer
7/2/2020	BL	4M	7	2020	Year-3		1	Summer
7/2/2020	BL	5M	7	2020	Year-3		1	Summer
7/2/2020	BL	6M	7	2020	Year-3		1	Summer
7/2/2020	BL	7M	7	2020	Year-3		1	Summer
7/2/2020	BL	10M	7	2020	Year-3	0	0	Summer
7/2/2020	BL	12M	7	2020	Year-3	0	0	Summer
7/2/2020	BL	14M	7	2020	Year-3	0	0	Summer
7/2/2020	BL	8A	7	2020	Year-3		1	Summer
7/2/2020	BL	9A	7	2020	Year-3		1	Summer
7/2/2020	BL	11A	7	2020	Year-3		1	Summer
7/2/2020	BL	13A	7	2020	Year-3		1	Summer
7/3/2020	BL	1M	7	2020	Year-3		1	Summer
7/3/2020	BL	2M	7	2020	Year-3		1	Summer
7/3/2020	BL	3M	7	2020	Year-3		1	Summer
7/3/2020	BL	4M	7	2020	Year-3		1	Summer
7/3/2020	BL	5M	7	2020	Year-3		1	Summer
7/3/2020	BL	6M	7	2020	Year-3		1	Summer
7/3/2020	BL	7M	7	2020	Year-3		1	Summer
7/3/2020	BL	10M	7	2020	Year-3	0	0	Summer
7/3/2020	BL	12M	7	2020	Year-3	0	0	Summer
7/3/2020	BL	14M	7	2020	Year-3	0	0	Summer
7/3/2020	BL	8A	7	2020	Year-3		1	Summer
7/3/2020	BL	9A	7	2020	Year-3		1	Summer
7/3/2020	BL	11A	7	2020	Year-3		1	Summer
7/3/2020	BL	13A	7	2020	Year-3		1	Summer
7/4/2020	BL	1M	7	2020	Year-3		1	Summer
7/4/2020	BL	2M	7	2020	Year-3		1	Summer
7/4/2020	BL	3M	7	2020	Year-3		1	Summer
7/4/2020	BL	4M	7	2020	Year-3		1	Summer
7/4/2020	BL	5M	7	2020	Year-3		1	Summer
7/4/2020	BL	6M	7	2020	Year-3		1	Summer
7/4/2020	BL	7M	7	2020	Year-3		1	Summer
7/4/2020	BL	10M	7	2020	Year-3	0	0	Summer
7/4/2020	BL	12M	7	2020	Year-3	0	0	Summer

Date	Species	Site	Month	Year	Survey Year	Presence	Data Gap	Season
7/4/2020	BL	14M	7	2020	Year-3	0	0	Summer
7/4/2020	BL	8A	7	2020	Year-3		1	Summer
7/4/2020	BL	9A	7	2020	Year-3		1	Summer
7/4/2020	BL	11A	7	2020	Year-3		1	Summer
7/4/2020	BL	13A	7	2020	Year-3		1	Summer
7/5/2020	BL	1M	7	2020	Year-3		1	Summer
7/5/2020	BL	2M	7	2020	Year-3		1	Summer
7/5/2020	BL	3M	7	2020	Year-3		1	Summer
7/5/2020	BL	4M	7	2020	Year-3		1	Summer
7/5/2020	BL	5M	7	2020	Year-3		1	Summer
7/5/2020	BL	6M	7	2020	Year-3		1	Summer
7/5/2020	BL	7M	7	2020	Year-3		1	Summer
7/5/2020	BL	10M	7	2020	Year-3	0	0	Summer
7/5/2020	BL	12M	7	2020	Year-3	0	0	Summer
7/5/2020	BL	14M	7	2020	Year-3	0	0	Summer
7/5/2020	BL	8A	7	2020	Year-3		1	Summer
7/5/2020	BL	9A	7	2020	Year-3		1	Summer
7/5/2020	BL	11A	7	2020	Year-3		1	Summer
7/5/2020	BL	13A	7	2020	Year-3		1	Summer
7/6/2020	BL	1M	7	2020	Year-3		1	Summer
7/6/2020	BL	2M	7	2020	Year-3		1	Summer
7/6/2020	BL	3M	7	2020	Year-3		1	Summer
7/6/2020	BL	4M	7	2020	Year-3		1	Summer
7/6/2020	BL	5M	7	2020	Year-3		1	Summer
7/6/2020	BL	6M	7	2020	Year-3		1	Summer
7/6/2020	BL	7M	7	2020	Year-3		1	Summer
7/6/2020	BL	10M	7	2020	Year-3	0	0	Summer
7/6/2020	BL	12M	7	2020	Year-3	0	0	Summer
7/6/2020	BL	14M	7	2020	Year-3	0	0	Summer
7/6/2020	BL	8A	7	2020	Year-3		1	Summer
7/6/2020	BL	9A	7	2020	Year-3		1	Summer
7/6/2020	BL	11A	7	2020	Year-3		1	Summer
7/6/2020	BL	13A	7	2020	Year-3		1	Summer
7/7/2020	BL	1M	7	2020	Year-3		1	Summer
7/7/2020	BL	2M	7	2020	Year-3		1	Summer
7/7/2020	BL	3M	7	2020	Year-3		1	Summer
7/7/2020	BL	4M	7	2020	Year-3		1	Summer
7/7/2020	BL	5M	7	2020	Year-3		1	Summer
7/7/2020	BL	6M	7	2020	Year-3		1	Summer
7/7/2020	BL	7M	7	2020	Year-3		1	Summer
7/7/2020	BL	10M	7	2020	Year-3	0	0	Summer
7/7/2020	BL	12M	7	2020	Year-3	0	0	Summer
7/7/2020	BL	14M	7	2020	Year-3	0	0	Summer
7/7/2020	BL	8A	7	2020	Year-3		1	Summer

Date	Species	Site	Month	Year	Survey Year	Presence	Data Gap	Season
7/7/2020	BL	9A	7	2020	Year-3		1	Summer
7/7/2020	BL	11A	7	2020	Year-3		1	Summer
7/7/2020	BL	13A	7	2020	Year-3		1	Summer
7/8/2020	BL	1M	7	2020	Year-3		1	Summer
7/8/2020	BL	2M	7	2020	Year-3		1	Summer
7/8/2020	BL	3M	7	2020	Year-3		1	Summer
7/8/2020	BL	4M	7	2020	Year-3		1	Summer
7/8/2020	BL	5M	7	2020	Year-3		1	Summer
7/8/2020	BL	6M	7	2020	Year-3		1	Summer
7/8/2020	BL	7M	7	2020	Year-3		1	Summer
7/8/2020	BL	10M	7	2020	Year-3	0	0	Summer
7/8/2020	BL	12M	7	2020	Year-3	0	0	Summer
7/8/2020	BL	14M	7	2020	Year-3	0	0	Summer
7/8/2020	BL	8A	7	2020	Year-3		1	Summer
7/8/2020	BL	9A	7	2020	Year-3		1	Summer
7/8/2020	BL	11A	7	2020	Year-3		1	Summer
7/8/2020	BL	13A	7	2020	Year-3		1	Summer
7/9/2020	BL	1M	7	2020	Year-3		1	Summer
7/9/2020	BL	2M	7	2020	Year-3	0	0	Summer
7/9/2020	BL	3M	7	2020	Year-3	0	0	Summer
7/9/2020	BL	4M	7	2020	Year-3	0	0	Summer
7/9/2020	BL	5M	7	2020	Year-3		1	Summer
7/9/2020	BL	6M	7	2020	Year-3		1	Summer
7/9/2020	BL	7M	7	2020	Year-3		1	Summer
7/9/2020	BL	10M	7	2020	Year-3	0	0	Summer
7/9/2020	BL	12M	7	2020	Year-3	0	0	Summer
7/9/2020	BL	14M	7	2020	Year-3	0	0	Summer
7/9/2020	BL	8A	7	2020	Year-3		1	Summer
7/9/2020	BL	9A	7	2020	Year-3		1	Summer
7/9/2020	BL	11A	7	2020	Year-3		1	Summer
7/9/2020	BL	13A	7	2020	Year-3		1	Summer
7/10/2020	BL	1M	7	2020	Year-3	0	0	Summer
7/10/2020	BL	2M	7	2020	Year-3		1	Summer
7/10/2020	BL	3M	7	2020	Year-3	0	0	Summer
7/10/2020	BL	4M	7	2020	Year-3	0	0	Summer
7/10/2020	BL	5M	7	2020	Year-3		1	Summer
7/10/2020	BL	6M	7	2020	Year-3		1	Summer
7/10/2020	BL	7M	7	2020	Year-3		1	Summer
7/10/2020	BL	10M	7	2020	Year-3	0	0	Summer
7/10/2020	BL	12M	7	2020	Year-3	0	0	Summer
7/10/2020	BL	14M	7	2020	Year-3	0	0	Summer
7/10/2020	BL	8A	7	2020	Year-3		1	Summer
7/10/2020	BL	9A	7	2020	Year-3		1	Summer
7/10/2020	BL	11A	7	2020	Year-3		1	Summer

Date	Species	Site	Month	Year	Survey Year	Presence	Data Gap	Season
7/10/2020	BL	13A	7	2020	Year-3		1	Summer
7/11/2020	BL	1M	7	2020	Year-3	0	0	Summer
7/11/2020	BL	2M	7	2020	Year-3		1	Summer
7/11/2020	BL	3M	7	2020	Year-3	0	0	Summer
7/11/2020	BL	4M	7	2020	Year-3	0	0	Summer
7/11/2020	BL	5M	7	2020	Year-3		1	Summer
7/11/2020	BL	6M	7	2020	Year-3		1	Summer
7/11/2020	BL	7M	7	2020	Year-3		1	Summer
7/11/2020	BL	10M	7	2020	Year-3	0	0	Summer
7/11/2020	BL	12M	7	2020	Year-3	0	0	Summer
7/11/2020	BL	14M	7	2020	Year-3	0	0	Summer
7/11/2020	BL	8A	7	2020	Year-3		1	Summer
7/11/2020	BL	9A	7	2020	Year-3		1	Summer
7/11/2020	BL	11A	7	2020	Year-3		1	Summer
7/11/2020	BL	13A	7	2020	Year-3		1	Summer
7/12/2020	BL	1M	7	2020	Year-3	0	0	Summer
7/12/2020	BL	2M	7	2020	Year-3		1	Summer
7/12/2020	BL	3M	7	2020	Year-3	0	0	Summer
7/12/2020	BL	4M	7	2020	Year-3	0	0	Summer
7/12/2020	BL	5M	7	2020	Year-3		1	Summer
7/12/2020	BL	6M	7	2020	Year-3		1	Summer
7/12/2020	BL	7M	7	2020	Year-3		1	Summer
7/12/2020	BL	10M	7	2020	Year-3	0	0	Summer
7/12/2020	BL	12M	7	2020	Year-3	0	0	Summer
7/12/2020	BL	14M	7	2020	Year-3	0	0	Summer
7/12/2020	BL	8A	7	2020	Year-3		1	Summer
7/12/2020	BL	9A	7	2020	Year-3		1	Summer
7/12/2020	BL	11A	7	2020	Year-3		1	Summer
7/12/2020	BL	13A	7	2020	Year-3		1	Summer
7/13/2020	BL	1M	7	2020	Year-3	0	0	Summer
7/13/2020	BL	2M	7	2020	Year-3		1	Summer
7/13/2020	BL	3M	7	2020	Year-3	0	0	Summer
7/13/2020	BL	4M	7	2020	Year-3	0	0	Summer
7/13/2020	BL	5M	7	2020	Year-3		1	Summer
7/13/2020	BL	6M	7	2020	Year-3		1	Summer
7/13/2020	BL	7M	7	2020	Year-3		1	Summer
7/13/2020	BL	10M	7	2020	Year-3	0	0	Summer
7/13/2020	BL	12M	7	2020	Year-3	0	0	Summer
7/13/2020	BL	14M	7	2020	Year-3	0	0	Summer
7/13/2020	BL	8A	7	2020	Year-3		1	Summer
7/13/2020	BL	9A	7	2020	Year-3		1	Summer
7/13/2020	BL	11A	7	2020	Year-3		1	Summer
7/13/2020	BL	13A	7	2020	Year-3		1	Summer
7/14/2020	BL	1M	7	2020	Year-3	0	0	Summer

Date	Species	Site	Month	Year	Survey Year	Presence	Data Gap	Season
7/14/2020	BL	2M	7	2020	Year-3		1	Summer
7/14/2020	BL	3M	7	2020	Year-3	0	0	Summer
7/14/2020	BL	4M	7	2020	Year-3	0	0	Summer
7/14/2020	BL	5M	7	2020	Year-3		1	Summer
7/14/2020	BL	6M	7	2020	Year-3		1	Summer
7/14/2020	BL	7M	7	2020	Year-3		1	Summer
7/14/2020	BL	10M	7	2020	Year-3	0	0	Summer
7/14/2020	BL	12M	7	2020	Year-3	0	0	Summer
7/14/2020	BL	14M	7	2020	Year-3	0	0	Summer
7/14/2020	BL	8A	7	2020	Year-3		1	Summer
7/14/2020	BL	9A	7	2020	Year-3		1	Summer
7/14/2020	BL	11A	7	2020	Year-3		1	Summer
7/14/2020	BL	13A	7	2020	Year-3		1	Summer
7/15/2020	BL	1M	7	2020	Year-3	0	0	Summer
7/15/2020	BL	2M	7	2020	Year-3		1	Summer
7/15/2020	BL	3M	7	2020	Year-3	0	0	Summer
7/15/2020	BL	4M	7	2020	Year-3	0	0	Summer
7/15/2020	BL	5M	7	2020	Year-3		1	Summer
7/15/2020	BL	6M	7	2020	Year-3		1	Summer
7/15/2020	BL	7M	7	2020	Year-3		1	Summer
7/15/2020	BL	10M	7	2020	Year-3	0	0	Summer
7/15/2020	BL	12M	7	2020	Year-3	0	0	Summer
7/15/2020	BL	14M	7	2020	Year-3	0	0	Summer
7/15/2020	BL	8A	7	2020	Year-3		1	Summer
7/15/2020	BL	9A	7	2020	Year-3		1	Summer
7/15/2020	BL	11A	7	2020	Year-3		1	Summer
7/15/2020	BL	13A	7	2020	Year-3		1	Summer
7/16/2020	BL	1M	7	2020	Year-3	0	0	Summer
7/16/2020	BL	2M	7	2020	Year-3		1	Summer
7/16/2020	BL	3M	7	2020	Year-3	0	0	Summer
7/16/2020	BL	4M	7	2020	Year-3	0	0	Summer
7/16/2020	BL	5M	7	2020	Year-3		1	Summer
7/16/2020	BL	6M	7	2020	Year-3		1	Summer
7/16/2020	BL	7M	7	2020	Year-3		1	Summer
7/16/2020	BL	10M	7	2020	Year-3	0	0	Summer
7/16/2020	BL	12M	7	2020	Year-3	0	0	Summer
7/16/2020	BL	14M	7	2020	Year-3	0	0	Summer
7/16/2020	BL	8A	7	2020	Year-3		1	Summer
7/16/2020	BL	9A	7	2020	Year-3		1	Summer
7/16/2020	BL	11A	7	2020	Year-3		1	Summer
7/16/2020	BL	13A	7	2020	Year-3		1	Summer
7/17/2020	BL	1M	7	2020	Year-3	0	0	Summer
7/17/2020	BL	2M	7	2020	Year-3		1	Summer
7/17/2020	BL	3M	7	2020	Year-3	0	0	Summer

Date	Species	Site	Month	Year	Survey Year	Presence	Data Gap	Season
7/17/2020	BL	4M	7	2020	Year-3	0	0	Summer
7/17/2020	BL	5M	7	2020	Year-3		1	Summer
7/17/2020	BL	6M	7	2020	Year-3		1	Summer
7/17/2020	BL	7M	7	2020	Year-3		1	Summer
7/17/2020	BL	10M	7	2020	Year-3	0	0	Summer
7/17/2020	BL	12M	7	2020	Year-3	0	0	Summer
7/17/2020	BL	14M	7	2020	Year-3	0	0	Summer
7/17/2020	BL	8A	7	2020	Year-3		1	Summer
7/17/2020	BL	9A	7	2020	Year-3		1	Summer
7/17/2020	BL	11A	7	2020	Year-3		1	Summer
7/17/2020	BL	13A	7	2020	Year-3		1	Summer
7/18/2020	BL	1M	7	2020	Year-3	0	0	Summer
7/18/2020	BL	2M	7	2020	Year-3		1	Summer
7/18/2020	BL	3M	7	2020	Year-3	0	0	Summer
7/18/2020	BL	4M	7	2020	Year-3	0	0	Summer
7/18/2020	BL	5M	7	2020	Year-3		1	Summer
7/18/2020	BL	6M	7	2020	Year-3		1	Summer
7/18/2020	BL	7M	7	2020	Year-3		1	Summer
7/18/2020	BL	10M	7	2020	Year-3	0	0	Summer
7/18/2020	BL	12M	7	2020	Year-3	0	0	Summer
7/18/2020	BL	14M	7	2020	Year-3	0	0	Summer
7/18/2020	BL	8A	7	2020	Year-3		1	Summer
7/18/2020	BL	9A	7	2020	Year-3		1	Summer
7/18/2020	BL	11A	7	2020	Year-3		1	Summer
7/18/2020	BL	13A	7	2020	Year-3		1	Summer
7/19/2020	BL	1M	7	2020	Year-3	0	0	Summer
7/19/2020	BL	2M	7	2020	Year-3		1	Summer
7/19/2020	BL	3M	7	2020	Year-3	0	0	Summer
7/19/2020	BL	4M	7	2020	Year-3	0	0	Summer
7/19/2020	BL	5M	7	2020	Year-3		1	Summer
7/19/2020	BL	6M	7	2020	Year-3		1	Summer
7/19/2020	BL	7M	7	2020	Year-3		1	Summer
7/19/2020	BL	10M	7	2020	Year-3	0	0	Summer
7/19/2020	BL	12M	7	2020	Year-3	0	0	Summer
7/19/2020	BL	14M	7	2020	Year-3	0	0	Summer
7/19/2020	BL	8A	7	2020	Year-3		1	Summer
7/19/2020	BL	9A	7	2020	Year-3		1	Summer
7/19/2020	BL	11A	7	2020	Year-3		1	Summer
7/19/2020	BL	13A	7	2020	Year-3		1	Summer
7/20/2020	BL	1M	7	2020	Year-3	0	0	Summer
7/20/2020	BL	2M	7	2020	Year-3		1	Summer
7/20/2020	BL	3M	7	2020	Year-3	0	0	Summer
7/20/2020	BL	4M	7	2020	Year-3	0	0	Summer
7/20/2020	BL	5M	7	2020	Year-3		1	Summer

Date	Species	Site	Month	Year	Survey Year	Presence	Data Gap	Season
7/20/2020	BL	6M	7	2020	Year-3		1	Summer
7/20/2020	BL	7M	7	2020	Year-3		1	Summer
7/20/2020	BL	10M	7	2020	Year-3	0	0	Summer
7/20/2020	BL	12M	7	2020	Year-3	0	0	Summer
7/20/2020	BL	14M	7	2020	Year-3	0	0	Summer
7/20/2020	BL	8A	7	2020	Year-3		1	Summer
7/20/2020	BL	9A	7	2020	Year-3		1	Summer
7/20/2020	BL	11A	7	2020	Year-3		1	Summer
7/20/2020	BL	13A	7	2020	Year-3		1	Summer
7/21/2020	BL	1M	7	2020	Year-3	0	0	Summer
7/21/2020	BL	2M	7	2020	Year-3		1	Summer
7/21/2020	BL	3M	7	2020	Year-3	0	0	Summer
7/21/2020	BL	4M	7	2020	Year-3	0	0	Summer
7/21/2020	BL	5M	7	2020	Year-3		1	Summer
7/21/2020	BL	6M	7	2020	Year-3		1	Summer
7/21/2020	BL	7M	7	2020	Year-3		1	Summer
7/21/2020	BL	10M	7	2020	Year-3	0	0	Summer
7/21/2020	BL	12M	7	2020	Year-3	0	0	Summer
7/21/2020	BL	14M	7	2020	Year-3	0	0	Summer
7/21/2020	BL	8A	7	2020	Year-3		1	Summer
7/21/2020	BL	9A	7	2020	Year-3		1	Summer
7/21/2020	BL	11A	7	2020	Year-3		1	Summer
7/21/2020	BL	13A	7	2020	Year-3		1	Summer
7/22/2020	BL	1M	7	2020	Year-3	0	0	Summer
7/22/2020	BL	2M	7	2020	Year-3		1	Summer
7/22/2020	BL	3M	7	2020	Year-3	0	0	Summer
7/22/2020	BL	4M	7	2020	Year-3	0	0	Summer
7/22/2020	BL	5M	7	2020	Year-3		1	Summer
7/22/2020	BL	6M	7	2020	Year-3		1	Summer
7/22/2020	BL	7M	7	2020	Year-3		1	Summer
7/22/2020	BL	10M	7	2020	Year-3	0	0	Summer
7/22/2020	BL	12M	7	2020	Year-3	0	0	Summer
7/22/2020	BL	14M	7	2020	Year-3	0	0	Summer
7/22/2020	BL	8A	7	2020	Year-3		1	Summer
7/22/2020	BL	9A	7	2020	Year-3		1	Summer
7/22/2020	BL	11A	7	2020	Year-3		1	Summer
7/22/2020	BL	13A	7	2020	Year-3		1	Summer
7/23/2020	BL	1M	7	2020	Year-3	0	0	Summer
7/23/2020	BL	2M	7	2020	Year-3		1	Summer
7/23/2020	BL	3M	7	2020	Year-3	0	0	Summer
7/23/2020	BL	4M	7	2020	Year-3	0	0	Summer
7/23/2020	BL	5M	7	2020	Year-3		1	Summer
7/23/2020	BL	6M	7	2020	Year-3		1	Summer
7/23/2020	BL	7M	7	2020	Year-3		1	Summer

Date	Species	Site	Month	Year	Survey Year	Presence	Data Gap	Season
7/23/2020	BL	10M	7	2020	Year-3	0	0	Summer
7/23/2020	BL	12M	7	2020	Year-3	0	0	Summer
7/23/2020	BL	14M	7	2020	Year-3	0	0	Summer
7/23/2020	BL	8A	7	2020	Year-3		1	Summer
7/23/2020	BL	9A	7	2020	Year-3		1	Summer
7/23/2020	BL	11A	7	2020	Year-3		1	Summer
7/23/2020	BL	13A	7	2020	Year-3		1	Summer
7/24/2020	BL	1M	7	2020	Year-3	0	0	Summer
7/24/2020	BL	2M	7	2020	Year-3		1	Summer
7/24/2020	BL	3M	7	2020	Year-3	0	0	Summer
7/24/2020	BL	4M	7	2020	Year-3	0	0	Summer
7/24/2020	BL	5M	7	2020	Year-3		1	Summer
7/24/2020	BL	6M	7	2020	Year-3		1	Summer
7/24/2020	BL	7M	7	2020	Year-3		1	Summer
7/24/2020	BL	10M	7	2020	Year-3	0	0	Summer
7/24/2020	BL	12M	7	2020	Year-3	0	0	Summer
7/24/2020	BL	14M	7	2020	Year-3	0	0	Summer
7/24/2020	BL	8A	7	2020	Year-3		1	Summer
7/24/2020	BL	9A	7	2020	Year-3		1	Summer
7/24/2020	BL	11A	7	2020	Year-3		1	Summer
7/24/2020	BL	13A	7	2020	Year-3		1	Summer
7/25/2020	BL	1M	7	2020	Year-3	0	0	Summer
7/25/2020	BL	2M	7	2020	Year-3		1	Summer
7/25/2020	BL	3M	7	2020	Year-3	0	0	Summer
7/25/2020	BL	4M	7	2020	Year-3	0	0	Summer
7/25/2020	BL	5M	7	2020	Year-3		1	Summer
7/25/2020	BL	6M	7	2020	Year-3		1	Summer
7/25/2020	BL	7M	7	2020	Year-3		1	Summer
7/25/2020	BL	10M	7	2020	Year-3	0	0	Summer
7/25/2020	BL	12M	7	2020	Year-3	0	0	Summer
7/25/2020	BL	14M	7	2020	Year-3	0	0	Summer
7/25/2020	BL	8A	7	2020	Year-3		1	Summer
7/25/2020	BL	9A	7	2020	Year-3		1	Summer
7/25/2020	BL	11A	7	2020	Year-3		1	Summer
7/25/2020	BL	13A	7	2020	Year-3		1	Summer
7/26/2020	BL	1M	7	2020	Year-3	0	0	Summer
7/26/2020	BL	2M	7	2020	Year-3		1	Summer
7/26/2020	BL	3M	7	2020	Year-3	0	0	Summer
7/26/2020	BL	4M	7	2020	Year-3	0	0	Summer
7/26/2020	BL	5M	7	2020	Year-3		1	Summer
7/26/2020	BL	6M	7	2020	Year-3		1	Summer
7/26/2020	BL	7M	7	2020	Year-3		1	Summer
7/26/2020	BL	10M	7	2020	Year-3	0	0	Summer
7/26/2020	BL	12M	7	2020	Year-3	0	0	Summer

Date	Species	Site	Month	Year	Survey Year	Presence	Data Gap	Season
7/26/2020	BL	14M	7	2020	Year-3	0	0	Summer
7/26/2020	BL	8A	7	2020	Year-3		1	Summer
7/26/2020	BL	9A	7	2020	Year-3		1	Summer
7/26/2020	BL	11A	7	2020	Year-3		1	Summer
7/26/2020	BL	13A	7	2020	Year-3		1	Summer
7/27/2020	BL	1M	7	2020	Year-3	0	0	Summer
7/27/2020	BL	2M	7	2020	Year-3		1	Summer
7/27/2020	BL	3M	7	2020	Year-3	0	0	Summer
7/27/2020	BL	4M	7	2020	Year-3	0	0	Summer
7/27/2020	BL	5M	7	2020	Year-3		1	Summer
7/27/2020	BL	6M	7	2020	Year-3		1	Summer
7/27/2020	BL	7M	7	2020	Year-3		1	Summer
7/27/2020	BL	10M	7	2020	Year-3	0	0	Summer
7/27/2020	BL	12M	7	2020	Year-3	0	0	Summer
7/27/2020	BL	14M	7	2020	Year-3	0	0	Summer
7/27/2020	BL	8A	7	2020	Year-3		1	Summer
7/27/2020	BL	9A	7	2020	Year-3		1	Summer
7/27/2020	BL	11A	7	2020	Year-3		1	Summer
7/27/2020	BL	13A	7	2020	Year-3		1	Summer
7/28/2020	BL	1M	7	2020	Year-3	0	0	Summer
7/28/2020	BL	2M	7	2020	Year-3		1	Summer
7/28/2020	BL	3M	7	2020	Year-3	0	0	Summer
7/28/2020	BL	4M	7	2020	Year-3	0	0	Summer
7/28/2020	BL	5M	7	2020	Year-3		1	Summer
7/28/2020	BL	6M	7	2020	Year-3		1	Summer
7/28/2020	BL	7M	7	2020	Year-3		1	Summer
7/28/2020	BL	10M	7	2020	Year-3	0	0	Summer
7/28/2020	BL	12M	7	2020	Year-3	0	0	Summer
7/28/2020	BL	14M	7	2020	Year-3	0	0	Summer
7/28/2020	BL	8A	7	2020	Year-3		1	Summer
7/28/2020	BL	9A	7	2020	Year-3		1	Summer
7/28/2020	BL	11A	7	2020	Year-3		1	Summer
7/28/2020	BL	13A	7	2020	Year-3		1	Summer
7/29/2020	BL	1M	7	2020	Year-3	0	0	Summer
7/29/2020	BL	2M	7	2020	Year-3		1	Summer
7/29/2020	BL	3M	7	2020	Year-3	0	0	Summer
7/29/2020	BL	4M	7	2020	Year-3	0	0	Summer
7/29/2020	BL	5M	7	2020	Year-3		1	Summer
7/29/2020	BL	6M	7	2020	Year-3		1	Summer
7/29/2020	BL	7M	7	2020	Year-3		1	Summer
7/29/2020	BL	10M	7	2020	Year-3	0	0	Summer
7/29/2020	BL	12M	7	2020	Year-3	0	0	Summer
7/29/2020	BL	14M	7	2020	Year-3	0	0	Summer
7/29/2020	BL	8A	7	2020	Year-3		1	Summer

Date	Species	Site	Month	Year	Survey Year	Presence	Data Gap	Season
7/29/2020	BL	9A	7	2020	Year-3		1	Summer
7/29/2020	BL	11A	7	2020	Year-3		1	Summer
7/29/2020	BL	13A	7	2020	Year-3		1	Summer
7/30/2020	BL	1M	7	2020	Year-3	0	0	Summer
7/30/2020	BL	2M	7	2020	Year-3		1	Summer
7/30/2020	BL	3M	7	2020	Year-3	0	0	Summer
7/30/2020	BL	4M	7	2020	Year-3	0	0	Summer
7/30/2020	BL	5M	7	2020	Year-3		1	Summer
7/30/2020	BL	6M	7	2020	Year-3		1	Summer
7/30/2020	BL	7M	7	2020	Year-3		1	Summer
7/30/2020	BL	10M	7	2020	Year-3	0	0	Summer
7/30/2020	BL	12M	7	2020	Year-3	0	0	Summer
7/30/2020	BL	14M	7	2020	Year-3	0	0	Summer
7/30/2020	BL	8A	7	2020	Year-3		1	Summer
7/30/2020	BL	9A	7	2020	Year-3		1	Summer
7/30/2020	BL	11A	7	2020	Year-3		1	Summer
7/30/2020	BL	13A	7	2020	Year-3		1	Summer
7/31/2020	BL	1M	7	2020	Year-3	0	0	Summer
7/31/2020	BL	2M	7	2020	Year-3		1	Summer
7/31/2020	BL	3M	7	2020	Year-3	0	0	Summer
7/31/2020	BL	4M	7	2020	Year-3	0	0	Summer
7/31/2020	BL	5M	7	2020	Year-3		1	Summer
7/31/2020	BL	6M	7	2020	Year-3		1	Summer
7/31/2020	BL	7M	7	2020	Year-3		1	Summer
7/31/2020	BL	10M	7	2020	Year-3	0	0	Summer
7/31/2020	BL	12M	7	2020	Year-3	0	0	Summer
7/31/2020	BL	14M	7	2020	Year-3	0	0	Summer
7/31/2020	BL	8A	7	2020	Year-3		1	Summer
7/31/2020	BL	9A	7	2020	Year-3		1	Summer
7/31/2020	BL	11A	7	2020	Year-3		1	Summer
7/31/2020	BL	13A	7	2020	Year-3		1	Summer
8/1/2020	BL	1M	8	2020	Year-3	0	0	Summer
8/1/2020	BL	2M	8	2020	Year-3		1	Summer
8/1/2020	BL	3M	8	2020	Year-3	0	0	Summer
8/1/2020	BL	4M	8	2020	Year-3	0	0	Summer
8/1/2020	BL	5M	8	2020	Year-3		1	Summer
8/1/2020	BL	6M	8	2020	Year-3		1	Summer
8/1/2020	BL	7M	8	2020	Year-3		1	Summer
8/1/2020	BL	10M	8	2020	Year-3	0	0	Summer
8/1/2020	BL	12M	8	2020	Year-3	0	0	Summer
8/1/2020	BL	14M	8	2020	Year-3	0	0	Summer
8/1/2020	BL	8A	8	2020	Year-3		1	Summer
8/1/2020	BL	9A	8	2020	Year-3		1	Summer
8/1/2020	BL	11A	8	2020	Year-3		1	Summer

Date	Species	Site	Month	Year	Survey Year	Presence	Data Gap	Season
8/1/2020	BL	13A	8	2020	Year-3		1	Summer
8/2/2020	BL	1M	8	2020	Year-3	0	0	Summer
8/2/2020	BL	2M	8	2020	Year-3		1	Summer
8/2/2020	BL	3M	8	2020	Year-3	0	0	Summer
8/2/2020	BL	4M	8	2020	Year-3	0	0	Summer
8/2/2020	BL	5M	8	2020	Year-3		1	Summer
8/2/2020	BL	6M	8	2020	Year-3		1	Summer
8/2/2020	BL	7M	8	2020	Year-3		1	Summer
8/2/2020	BL	10M	8	2020	Year-3	0	0	Summer
8/2/2020	BL	12M	8	2020	Year-3	0	0	Summer
8/2/2020	BL	14M	8	2020	Year-3	0	0	Summer
8/2/2020	BL	8A	8	2020	Year-3		1	Summer
8/2/2020	BL	9A	8	2020	Year-3		1	Summer
8/2/2020	BL	11A	8	2020	Year-3		1	Summer
8/2/2020	BL	13A	8	2020	Year-3		1	Summer
8/3/2020	BL	1M	8	2020	Year-3	0	0	Summer
8/3/2020	BL	2M	8	2020	Year-3		1	Summer
8/3/2020	BL	3M	8	2020	Year-3	0	0	Summer
8/3/2020	BL	4M	8	2020	Year-3	0	0	Summer
8/3/2020	BL	5M	8	2020	Year-3		1	Summer
8/3/2020	BL	6M	8	2020	Year-3		1	Summer
8/3/2020	BL	7M	8	2020	Year-3		1	Summer
8/3/2020	BL	10M	8	2020	Year-3	0	0	Summer
8/3/2020	BL	12M	8	2020	Year-3	0	0	Summer
8/3/2020	BL	14M	8	2020	Year-3	0	0	Summer
8/3/2020	BL	8A	8	2020	Year-3		1	Summer
8/3/2020	BL	9A	8	2020	Year-3		1	Summer
8/3/2020	BL	11A	8	2020	Year-3		1	Summer
8/3/2020	BL	13A	8	2020	Year-3		1	Summer
8/4/2020	BL	1M	8	2020	Year-3	0	0	Summer
8/4/2020	BL	2M	8	2020	Year-3		1	Summer
8/4/2020	BL	3M	8	2020	Year-3	0	0	Summer
8/4/2020	BL	4M	8	2020	Year-3	0	0	Summer
8/4/2020	BL	5M	8	2020	Year-3		1	Summer
8/4/2020	BL	6M	8	2020	Year-3		1	Summer
8/4/2020	BL	7M	8	2020	Year-3		1	Summer
8/4/2020	BL	10M	8	2020	Year-3	0	0	Summer
8/4/2020	BL	12M	8	2020	Year-3	0	0	Summer
8/4/2020	BL	14M	8	2020	Year-3	0	0	Summer
8/4/2020	BL	8A	8	2020	Year-3		1	Summer
8/4/2020	BL	9A	8	2020	Year-3		1	Summer
8/4/2020	BL	11A	8	2020	Year-3		1	Summer
8/4/2020	BL	13A	8	2020	Year-3		1	Summer
8/5/2020	BL	1M	8	2020	Year-3	0	0	Summer

Date	Species	Site	Month	Year	Survey Year	Presence	Data Gap	Season
8/5/2020	BL	2M	8	2020	Year-3		1	Summer
8/5/2020	BL	3M	8	2020	Year-3	0	0	Summer
8/5/2020	BL	4M	8	2020	Year-3	0	0	Summer
8/5/2020	BL	5M	8	2020	Year-3		1	Summer
8/5/2020	BL	6M	8	2020	Year-3		1	Summer
8/5/2020	BL	7M	8	2020	Year-3		1	Summer
8/5/2020	BL	10M	8	2020	Year-3	0	0	Summer
8/5/2020	BL	12M	8	2020	Year-3	0	0	Summer
8/5/2020	BL	14M	8	2020	Year-3	0	0	Summer
8/5/2020	BL	8A	8	2020	Year-3		1	Summer
8/5/2020	BL	9A	8	2020	Year-3		1	Summer
8/5/2020	BL	11A	8	2020	Year-3		1	Summer
8/5/2020	BL	13A	8	2020	Year-3		1	Summer
8/6/2020	BL	1M	8	2020	Year-3	0	0	Summer
8/6/2020	BL	2M	8	2020	Year-3		1	Summer
8/6/2020	BL	3M	8	2020	Year-3	0	0	Summer
8/6/2020	BL	4M	8	2020	Year-3	0	0	Summer
8/6/2020	BL	5M	8	2020	Year-3		1	Summer
8/6/2020	BL	6M	8	2020	Year-3		1	Summer
8/6/2020	BL	7M	8	2020	Year-3		1	Summer
8/6/2020	BL	10M	8	2020	Year-3	0	0	Summer
8/6/2020	BL	12M	8	2020	Year-3	0	0	Summer
8/6/2020	BL	14M	8	2020	Year-3	0	0	Summer
8/6/2020	BL	8A	8	2020	Year-3		1	Summer
8/6/2020	BL	9A	8	2020	Year-3		1	Summer
8/6/2020	BL	11A	8	2020	Year-3		1	Summer
8/6/2020	BL	13A	8	2020	Year-3		1	Summer
8/7/2020	BL	1M	8	2020	Year-3	0	0	Summer
8/7/2020	BL	2M	8	2020	Year-3		1	Summer
8/7/2020	BL	3M	8	2020	Year-3	0	0	Summer
8/7/2020	BL	4M	8	2020	Year-3	0	0	Summer
8/7/2020	BL	5M	8	2020	Year-3		1	Summer
8/7/2020	BL	6M	8	2020	Year-3		1	Summer
8/7/2020	BL	7M	8	2020	Year-3		1	Summer
8/7/2020	BL	10M	8	2020	Year-3	0	0	Summer
8/7/2020	BL	12M	8	2020	Year-3	0	0	Summer
8/7/2020	BL	14M	8	2020	Year-3	0	0	Summer
8/7/2020	BL	8A	8	2020	Year-3		1	Summer
8/7/2020	BL	9A	8	2020	Year-3		1	Summer
8/7/2020	BL	11A	8	2020	Year-3		1	Summer
8/7/2020	BL	13A	8	2020	Year-3		1	Summer
8/8/2020	BL	1M	8	2020	Year-3	0	0	Summer
8/8/2020	BL	2M	8	2020	Year-3		1	Summer
8/8/2020	BL	3M	8	2020	Year-3	0	0	Summer

Date	Species	Site	Month	Year	Survey Year	Presence	Data Gap	Season
8/8/2020	BL	4M	8	2020	Year-3	0	0	Summer
8/8/2020	BL	5M	8	2020	Year-3		1	Summer
8/8/2020	BL	6M	8	2020	Year-3		1	Summer
8/8/2020	BL	7M	8	2020	Year-3		1	Summer
8/8/2020	BL	10M	8	2020	Year-3	0	0	Summer
8/8/2020	BL	12M	8	2020	Year-3	0	0	Summer
8/8/2020	BL	14M	8	2020	Year-3	0	0	Summer
8/8/2020	BL	8A	8	2020	Year-3		1	Summer
8/8/2020	BL	9A	8	2020	Year-3		1	Summer
8/8/2020	BL	11A	8	2020	Year-3		1	Summer
8/8/2020	BL	13A	8	2020	Year-3		1	Summer
8/9/2020	BL	1M	8	2020	Year-3	0	0	Summer
8/9/2020	BL	2M	8	2020	Year-3		1	Summer
8/9/2020	BL	3M	8	2020	Year-3	0	0	Summer
8/9/2020	BL	4M	8	2020	Year-3	0	0	Summer
8/9/2020	BL	5M	8	2020	Year-3		1	Summer
8/9/2020	BL	6M	8	2020	Year-3		1	Summer
8/9/2020	BL	7M	8	2020	Year-3		1	Summer
8/9/2020	BL	10M	8	2020	Year-3	0	0	Summer
8/9/2020	BL	12M	8	2020	Year-3	0	0	Summer
8/9/2020	BL	14M	8	2020	Year-3	0	0	Summer
8/9/2020	BL	8A	8	2020	Year-3		1	Summer
8/9/2020	BL	9A	8	2020	Year-3		1	Summer
8/9/2020	BL	11A	8	2020	Year-3		1	Summer
8/9/2020	BL	13A	8	2020	Year-3		1	Summer
8/10/2020	BL	1M	8	2020	Year-3	0	0	Summer
8/10/2020	BL	2M	8	2020	Year-3		1	Summer
8/10/2020	BL	3M	8	2020	Year-3	0	0	Summer
8/10/2020	BL	4M	8	2020	Year-3	0	0	Summer
8/10/2020	BL	5M	8	2020	Year-3		1	Summer
8/10/2020	BL	6M	8	2020	Year-3		1	Summer
8/10/2020	BL	7M	8	2020	Year-3		1	Summer
8/10/2020	BL	10M	8	2020	Year-3	0	0	Summer
8/10/2020	BL	12M	8	2020	Year-3	0	0	Summer
8/10/2020	BL	14M	8	2020	Year-3	0	0	Summer
8/10/2020	BL	8A	8	2020	Year-3		1	Summer
8/10/2020	BL	9A	8	2020	Year-3		1	Summer
8/10/2020	BL	11A	8	2020	Year-3		1	Summer
8/10/2020	BL	13A	8	2020	Year-3		1	Summer
8/11/2020	BL	1M	8	2020	Year-3	0	0	Summer
8/11/2020	BL	2M	8	2020	Year-3		1	Summer
8/11/2020	BL	3M	8	2020	Year-3	0	0	Summer
8/11/2020	BL	4M	8	2020	Year-3	0	0	Summer
8/11/2020	BL	5M	8	2020	Year-3		1	Summer

Date	Species	Site	Month	Year	Survey Year	Presence	Data Gap	Season
8/11/2020	BL	6M	8	2020	Year-3		1	Summer
8/11/2020	BL	7M	8	2020	Year-3		1	Summer
8/11/2020	BL	10M	8	2020	Year-3	0	0	Summer
8/11/2020	BL	12M	8	2020	Year-3	0	0	Summer
8/11/2020	BL	14M	8	2020	Year-3	0	0	Summer
8/11/2020	BL	8A	8	2020	Year-3		1	Summer
8/11/2020	BL	9A	8	2020	Year-3		1	Summer
8/11/2020	BL	11A	8	2020	Year-3		1	Summer
8/11/2020	BL	13A	8	2020	Year-3		1	Summer
8/12/2020	BL	1M	8	2020	Year-3	0	0	Summer
8/12/2020	BL	2M	8	2020	Year-3		1	Summer
8/12/2020	BL	3M	8	2020	Year-3	0	0	Summer
8/12/2020	BL	4M	8	2020	Year-3	0	0	Summer
8/12/2020	BL	5M	8	2020	Year-3		1	Summer
8/12/2020	BL	6M	8	2020	Year-3		1	Summer
8/12/2020	BL	7M	8	2020	Year-3		1	Summer
8/12/2020	BL	10M	8	2020	Year-3	0	0	Summer
8/12/2020	BL	12M	8	2020	Year-3	0	0	Summer
8/12/2020	BL	14M	8	2020	Year-3	0	0	Summer
8/12/2020	BL	8A	8	2020	Year-3		1	Summer
8/12/2020	BL	9A	8	2020	Year-3		1	Summer
8/12/2020	BL	11A	8	2020	Year-3		1	Summer
8/12/2020	BL	13A	8	2020	Year-3		1	Summer
8/13/2020	BL	1M	8	2020	Year-3	0	0	Summer
8/13/2020	BL	2M	8	2020	Year-3		1	Summer
8/13/2020	BL	3M	8	2020	Year-3	0	0	Summer
8/13/2020	BL	4M	8	2020	Year-3	0	0	Summer
8/13/2020	BL	5M	8	2020	Year-3		1	Summer
8/13/2020	BL	6M	8	2020	Year-3		1	Summer
8/13/2020	BL	7M	8	2020	Year-3		1	Summer
8/13/2020	BL	10M	8	2020	Year-3	0	0	Summer
8/13/2020	BL	12M	8	2020	Year-3	0	0	Summer
8/13/2020	BL	14M	8	2020	Year-3	0	0	Summer
8/13/2020	BL	8A	8	2020	Year-3		1	Summer
8/13/2020	BL	9A	8	2020	Year-3		1	Summer
8/13/2020	BL	11A	8	2020	Year-3		1	Summer
8/13/2020	BL	13A	8	2020	Year-3		1	Summer
8/14/2020	BL	1M	8	2020	Year-3	0	0	Summer
8/14/2020	BL	2M	8	2020	Year-3		1	Summer
8/14/2020	BL	3M	8	2020	Year-3	0	0	Summer
8/14/2020	BL	4M	8	2020	Year-3	0	0	Summer
8/14/2020	BL	5M	8	2020	Year-3		1	Summer
8/14/2020	BL	6M	8	2020	Year-3		1	Summer
8/14/2020	BL	7M	8	2020	Year-3		1	Summer

Date	Species	Site	Month	Year	Survey Year	Presence	Data Gap	Season
8/14/2020	BL	10M	8	2020	Year-3	0	0	Summer
8/14/2020	BL	12M	8	2020	Year-3	0	0	Summer
8/14/2020	BL	14M	8	2020	Year-3	0	0	Summer
8/14/2020	BL	8A	8	2020	Year-3		1	Summer
8/14/2020	BL	9A	8	2020	Year-3		1	Summer
8/14/2020	BL	11A	8	2020	Year-3		1	Summer
8/14/2020	BL	13A	8	2020	Year-3		1	Summer
8/15/2020	BL	1M	8	2020	Year-3	0	0	Summer
8/15/2020	BL	2M	8	2020	Year-3		1	Summer
8/15/2020	BL	3M	8	2020	Year-3	0	0	Summer
8/15/2020	BL	4M	8	2020	Year-3	0	0	Summer
8/15/2020	BL	5M	8	2020	Year-3		1	Summer
8/15/2020	BL	6M	8	2020	Year-3		1	Summer
8/15/2020	BL	7M	8	2020	Year-3		1	Summer
8/15/2020	BL	10M	8	2020	Year-3	0	0	Summer
8/15/2020	BL	12M	8	2020	Year-3	0	0	Summer
8/15/2020	BL	14M	8	2020	Year-3	0	0	Summer
8/15/2020	BL	8A	8	2020	Year-3		1	Summer
8/15/2020	BL	9A	8	2020	Year-3		1	Summer
8/15/2020	BL	11A	8	2020	Year-3		1	Summer
8/15/2020	BL	13A	8	2020	Year-3		1	Summer
8/16/2020	BL	1M	8	2020	Year-3	0	0	Summer
8/16/2020	BL	2M	8	2020	Year-3		1	Summer
8/16/2020	BL	3M	8	2020	Year-3	0	0	Summer
8/16/2020	BL	4M	8	2020	Year-3	0	0	Summer
8/16/2020	BL	5M	8	2020	Year-3		1	Summer
8/16/2020	BL	6M	8	2020	Year-3		1	Summer
8/16/2020	BL	7M	8	2020	Year-3		1	Summer
8/16/2020	BL	10M	8	2020	Year-3	0	0	Summer
8/16/2020	BL	12M	8	2020	Year-3	0	0	Summer
8/16/2020	BL	14M	8	2020	Year-3	0	0	Summer
8/16/2020	BL	8A	8	2020	Year-3		1	Summer
8/16/2020	BL	9A	8	2020	Year-3		1	Summer
8/16/2020	BL	11A	8	2020	Year-3		1	Summer
8/16/2020	BL	13A	8	2020	Year-3		1	Summer
8/17/2020	BL	1M	8	2020	Year-3	0	0	Summer
8/17/2020	BL	2M	8	2020	Year-3		1	Summer
8/17/2020	BL	3M	8	2020	Year-3	0	0	Summer
8/17/2020	BL	4M	8	2020	Year-3	0	0	Summer
8/17/2020	BL	5M	8	2020	Year-3		1	Summer
8/17/2020	BL	6M	8	2020	Year-3		1	Summer
8/17/2020	BL	7M	8	2020	Year-3		1	Summer
8/17/2020	BL	10M	8	2020	Year-3	0	0	Summer
8/17/2020	BL	12M	8	2020	Year-3	0	0	Summer

Date	Species	Site	Month	Year	Survey Year	Presence	Data Gap	Season
8/17/2020	BL	14M	8	2020	Year-3	0	0	Summer
8/17/2020	BL	8A	8	2020	Year-3		1	Summer
8/17/2020	BL	9A	8	2020	Year-3		1	Summer
8/17/2020	BL	11A	8	2020	Year-3		1	Summer
8/17/2020	BL	13A	8	2020	Year-3		1	Summer
8/18/2020	BL	1M	8	2020	Year-3	0	0	Summer
8/18/2020	BL	2M	8	2020	Year-3		1	Summer
8/18/2020	BL	3M	8	2020	Year-3	0	0	Summer
8/18/2020	BL	4M	8	2020	Year-3	0	0	Summer
8/18/2020	BL	5M	8	2020	Year-3		1	Summer
8/18/2020	BL	6M	8	2020	Year-3		1	Summer
8/18/2020	BL	7M	8	2020	Year-3		1	Summer
8/18/2020	BL	10M	8	2020	Year-3	0	0	Summer
8/18/2020	BL	12M	8	2020	Year-3	0	0	Summer
8/18/2020	BL	14M	8	2020	Year-3	0	0	Summer
8/18/2020	BL	8A	8	2020	Year-3		1	Summer
8/18/2020	BL	9A	8	2020	Year-3		1	Summer
8/18/2020	BL	11A	8	2020	Year-3		1	Summer
8/18/2020	BL	13A	8	2020	Year-3		1	Summer
8/19/2020	BL	1M	8	2020	Year-3	0	0	Summer
8/19/2020	BL	2M	8	2020	Year-3		1	Summer
8/19/2020	BL	3M	8	2020	Year-3	0	0	Summer
8/19/2020	BL	4M	8	2020	Year-3	0	0	Summer
8/19/2020	BL	5M	8	2020	Year-3		1	Summer
8/19/2020	BL	6M	8	2020	Year-3		1	Summer
8/19/2020	BL	7M	8	2020	Year-3		1	Summer
8/19/2020	BL	10M	8	2020	Year-3	0	0	Summer
8/19/2020	BL	12M	8	2020	Year-3	0	0	Summer
8/19/2020	BL	14M	8	2020	Year-3	0	0	Summer
8/19/2020	BL	8A	8	2020	Year-3		1	Summer
8/19/2020	BL	9A	8	2020	Year-3		1	Summer
8/19/2020	BL	11A	8	2020	Year-3		1	Summer
8/19/2020	BL	13A	8	2020	Year-3		1	Summer
8/20/2020	BL	1M	8	2020	Year-3	0	0	Summer
8/20/2020	BL	2M	8	2020	Year-3		1	Summer
8/20/2020	BL	3M	8	2020	Year-3	0	0	Summer
8/20/2020	BL	4M	8	2020	Year-3	0	0	Summer
8/20/2020	BL	5M	8	2020	Year-3		1	Summer
8/20/2020	BL	6M	8	2020	Year-3		1	Summer
8/20/2020	BL	7M	8	2020	Year-3		1	Summer
8/20/2020	BL	10M	8	2020	Year-3	0	0	Summer
8/20/2020	BL	12M	8	2020	Year-3	0	0	Summer
8/20/2020	BL	14M	8	2020	Year-3	0	0	Summer
8/20/2020	BL	8A	8	2020	Year-3		1	Summer

Date	Species	Site	Month	Year	Survey Year	Presence	Data Gap	Season
8/20/2020	BL	9A	8	2020	Year-3		1	Summer
8/20/2020	BL	11A	8	2020	Year-3		1	Summer
8/20/2020	BL	13A	8	2020	Year-3		1	Summer
8/21/2020	BL	1M	8	2020	Year-3	0	0	Summer
8/21/2020	BL	2M	8	2020	Year-3		1	Summer
8/21/2020	BL	3M	8	2020	Year-3	0	0	Summer
8/21/2020	BL	4M	8	2020	Year-3	0	0	Summer
8/21/2020	BL	5M	8	2020	Year-3		1	Summer
8/21/2020	BL	6M	8	2020	Year-3		1	Summer
8/21/2020	BL	7M	8	2020	Year-3		1	Summer
8/21/2020	BL	10M	8	2020	Year-3	0	0	Summer
8/21/2020	BL	12M	8	2020	Year-3	0	0	Summer
8/21/2020	BL	14M	8	2020	Year-3	0	0	Summer
8/21/2020	BL	8A	8	2020	Year-3		1	Summer
8/21/2020	BL	9A	8	2020	Year-3		1	Summer
8/21/2020	BL	11A	8	2020	Year-3		1	Summer
8/21/2020	BL	13A	8	2020	Year-3		1	Summer
8/22/2020	BL	1M	8	2020	Year-3	0	0	Summer
8/22/2020	BL	2M	8	2020	Year-3		1	Summer
8/22/2020	BL	3M	8	2020	Year-3	0	0	Summer
8/22/2020	BL	4M	8	2020	Year-3	0	0	Summer
8/22/2020	BL	5M	8	2020	Year-3		1	Summer
8/22/2020	BL	6M	8	2020	Year-3		1	Summer
8/22/2020	BL	7M	8	2020	Year-3		1	Summer
8/22/2020	BL	10M	8	2020	Year-3	0	0	Summer
8/22/2020	BL	12M	8	2020	Year-3	0	0	Summer
8/22/2020	BL	14M	8	2020	Year-3	0	0	Summer
8/22/2020	BL	8A	8	2020	Year-3		1	Summer
8/22/2020	BL	9A	8	2020	Year-3		1	Summer
8/22/2020	BL	11A	8	2020	Year-3		1	Summer
8/22/2020	BL	13A	8	2020	Year-3		1	Summer
8/23/2020	BL	1M	8	2020	Year-3	0	0	Summer
8/23/2020	BL	2M	8	2020	Year-3		1	Summer
8/23/2020	BL	3M	8	2020	Year-3	0	0	Summer
8/23/2020	BL	4M	8	2020	Year-3	0	0	Summer
8/23/2020	BL	5M	8	2020	Year-3		1	Summer
8/23/2020	BL	6M	8	2020	Year-3		1	Summer
8/23/2020	BL	7M	8	2020	Year-3		1	Summer
8/23/2020	BL	10M	8	2020	Year-3	0	0	Summer
8/23/2020	BL	12M	8	2020	Year-3	0	0	Summer
8/23/2020	BL	14M	8	2020	Year-3	0	0	Summer
8/23/2020	BL	8A	8	2020	Year-3		1	Summer
8/23/2020	BL	9A	8	2020	Year-3		1	Summer
8/23/2020	BL	11A	8	2020	Year-3		1	Summer

Date	Species	Site	Month	Year	Survey Year	Presence	Data Gap	Season
8/23/2020	BL	13A	8	2020	Year-3		1	Summer
8/24/2020	BL	1M	8	2020	Year-3	0	0	Summer
8/24/2020	BL	2M	8	2020	Year-3		1	Summer
8/24/2020	BL	3M	8	2020	Year-3	0	0	Summer
8/24/2020	BL	4M	8	2020	Year-3	0	0	Summer
8/24/2020	BL	5M	8	2020	Year-3		1	Summer
8/24/2020	BL	6M	8	2020	Year-3		1	Summer
8/24/2020	BL	7M	8	2020	Year-3		1	Summer
8/24/2020	BL	10M	8	2020	Year-3	0	0	Summer
8/24/2020	BL	12M	8	2020	Year-3	0	0	Summer
8/24/2020	BL	14M	8	2020	Year-3	0	0	Summer
8/24/2020	BL	8A	8	2020	Year-3		1	Summer
8/24/2020	BL	9A	8	2020	Year-3		1	Summer
8/24/2020	BL	11A	8	2020	Year-3		1	Summer
8/24/2020	BL	13A	8	2020	Year-3		1	Summer
8/25/2020	BL	1M	8	2020	Year-3	0	0	Summer
8/25/2020	BL	2M	8	2020	Year-3		1	Summer
8/25/2020	BL	3M	8	2020	Year-3	0	0	Summer
8/25/2020	BL	4M	8	2020	Year-3	0	0	Summer
8/25/2020	BL	5M	8	2020	Year-3		1	Summer
8/25/2020	BL	6M	8	2020	Year-3		1	Summer
8/25/2020	BL	7M	8	2020	Year-3		1	Summer
8/25/2020	BL	10M	8	2020	Year-3	0	0	Summer
8/25/2020	BL	12M	8	2020	Year-3	0	0	Summer
8/25/2020	BL	14M	8	2020	Year-3	0	0	Summer
8/25/2020	BL	8A	8	2020	Year-3		1	Summer
8/25/2020	BL	9A	8	2020	Year-3		1	Summer
8/25/2020	BL	11A	8	2020	Year-3		1	Summer
8/25/2020	BL	13A	8	2020	Year-3		1	Summer
8/26/2020	BL	1M	8	2020	Year-3	0	0	Summer
8/26/2020	BL	2M	8	2020	Year-3		1	Summer
8/26/2020	BL	3M	8	2020	Year-3	0	0	Summer
8/26/2020	BL	4M	8	2020	Year-3	0	0	Summer
8/26/2020	BL	5M	8	2020	Year-3		1	Summer
8/26/2020	BL	6M	8	2020	Year-3		1	Summer
8/26/2020	BL	7M	8	2020	Year-3		1	Summer
8/26/2020	BL	10M	8	2020	Year-3	0	0	Summer
8/26/2020	BL	12M	8	2020	Year-3	0	0	Summer
8/26/2020	BL	14M	8	2020	Year-3	0	0	Summer
8/26/2020	BL	8A	8	2020	Year-3		1	Summer
8/26/2020	BL	9A	8	2020	Year-3		1	Summer
8/26/2020	BL	11A	8	2020	Year-3		1	Summer
8/26/2020	BL	13A	8	2020	Year-3		1	Summer
8/27/2020	BL	1M	8	2020	Year-3	0	0	Summer

Date	Species	Site	Month	Year	Survey Year	Presence	Data Gap	Season
8/27/2020	BL	2M	8	2020	Year-3		1	Summer
8/27/2020	BL	3M	8	2020	Year-3	0	0	Summer
8/27/2020	BL	4M	8	2020	Year-3	0	0	Summer
8/27/2020	BL	5M	8	2020	Year-3		1	Summer
8/27/2020	BL	6M	8	2020	Year-3		1	Summer
8/27/2020	BL	7M	8	2020	Year-3		1	Summer
8/27/2020	BL	10M	8	2020	Year-3	0	0	Summer
8/27/2020	BL	12M	8	2020	Year-3	0	0	Summer
8/27/2020	BL	14M	8	2020	Year-3	0	0	Summer
8/27/2020	BL	8A	8	2020	Year-3		1	Summer
8/27/2020	BL	9A	8	2020	Year-3		1	Summer
8/27/2020	BL	11A	8	2020	Year-3		1	Summer
8/27/2020	BL	13A	8	2020	Year-3		1	Summer
8/28/2020	BL	1M	8	2020	Year-3	0	0	Summer
8/28/2020	BL	2M	8	2020	Year-3		1	Summer
8/28/2020	BL	3M	8	2020	Year-3	0	0	Summer
8/28/2020	BL	4M	8	2020	Year-3	0	0	Summer
8/28/2020	BL	5M	8	2020	Year-3		1	Summer
8/28/2020	BL	6M	8	2020	Year-3		1	Summer
8/28/2020	BL	7M	8	2020	Year-3		1	Summer
8/28/2020	BL	10M	8	2020	Year-3	0	0	Summer
8/28/2020	BL	12M	8	2020	Year-3	0	0	Summer
8/28/2020	BL	14M	8	2020	Year-3	0	0	Summer
8/28/2020	BL	8A	8	2020	Year-3		1	Summer
8/28/2020	BL	9A	8	2020	Year-3		1	Summer
8/28/2020	BL	11A	8	2020	Year-3		1	Summer
8/28/2020	BL	13A	8	2020	Year-3		1	Summer
8/29/2020	BL	1M	8	2020	Year-3	0	0	Summer
8/29/2020	BL	2M	8	2020	Year-3		1	Summer
8/29/2020	BL	3M	8	2020	Year-3	0	0	Summer
8/29/2020	BL	4M	8	2020	Year-3	0	0	Summer
8/29/2020	BL	5M	8	2020	Year-3		1	Summer
8/29/2020	BL	6M	8	2020	Year-3		1	Summer
8/29/2020	BL	7M	8	2020	Year-3		1	Summer
8/29/2020	BL	10M	8	2020	Year-3	0	0	Summer
8/29/2020	BL	12M	8	2020	Year-3	0	0	Summer
8/29/2020	BL	14M	8	2020	Year-3	0	0	Summer
8/29/2020	BL	8A	8	2020	Year-3		1	Summer
8/29/2020	BL	9A	8	2020	Year-3		1	Summer
8/29/2020	BL	11A	8	2020	Year-3		1	Summer
8/29/2020	BL	13A	8	2020	Year-3		1	Summer
8/30/2020	BL	1M	8	2020	Year-3	0	0	Summer
8/30/2020	BL	2M	8	2020	Year-3		1	Summer
8/30/2020	BL	3M	8	2020	Year-3	0	0	Summer

Date	Species	Site	Month	Year	Survey Year	Presence	Data Gap	Season
8/30/2020	BL	4M	8	2020	Year-3	0	0	Summer
8/30/2020	BL	5M	8	2020	Year-3		1	Summer
8/30/2020	BL	6M	8	2020	Year-3		1	Summer
8/30/2020	BL	7M	8	2020	Year-3		1	Summer
8/30/2020	BL	10M	8	2020	Year-3	0	0	Summer
8/30/2020	BL	12M	8	2020	Year-3	0	0	Summer
8/30/2020	BL	14M	8	2020	Year-3	0	0	Summer
8/30/2020	BL	8A	8	2020	Year-3		1	Summer
8/30/2020	BL	9A	8	2020	Year-3		1	Summer
8/30/2020	BL	11A	8	2020	Year-3		1	Summer
8/30/2020	BL	13A	8	2020	Year-3		1	Summer
8/31/2020	BL	1M	8	2020	Year-3	0	0	Summer
8/31/2020	BL	2M	8	2020	Year-3		1	Summer
8/31/2020	BL	3M	8	2020	Year-3	0	0	Summer
8/31/2020	BL	4M	8	2020	Year-3	0	0	Summer
8/31/2020	BL	5M	8	2020	Year-3		1	Summer
8/31/2020	BL	6M	8	2020	Year-3		1	Summer
8/31/2020	BL	7M	8	2020	Year-3		1	Summer
8/31/2020	BL	10M	8	2020	Year-3	0	0	Summer
8/31/2020	BL	12M	8	2020	Year-3	0	0	Summer
8/31/2020	BL	14M	8	2020	Year-3	0	0	Summer
8/31/2020	BL	8A	8	2020	Year-3		1	Summer
8/31/2020	BL	9A	8	2020	Year-3		1	Summer
8/31/2020	BL	11A	8	2020	Year-3		1	Summer
8/31/2020	BL	13A	8	2020	Year-3		1	Summer
9/1/2020	BL	1M	9	2020	Year-3	0	0	Fall
9/1/2020	BL	2M	9	2020	Year-3		1	Fall
9/1/2020	BL	3M	9	2020	Year-3	0	0	Fall
9/1/2020	BL	4M	9	2020	Year-3	0	0	Fall
9/1/2020	BL	5M	9	2020	Year-3		1	Fall
9/1/2020	BL	6M	9	2020	Year-3		1	Fall
9/1/2020	BL	7M	9	2020	Year-3		1	Fall
9/1/2020	BL	10M	9	2020	Year-3	0	0	Fall
9/1/2020	BL	12M	9	2020	Year-3	0	0	Fall
9/1/2020	BL	14M	9	2020	Year-3	0	0	Fall
9/1/2020	BL	8A	9	2020	Year-3		1	Fall
9/1/2020	BL	9A	9	2020	Year-3		1	Fall
9/1/2020	BL	11A	9	2020	Year-3		1	Fall
9/1/2020	BL	13A	9	2020	Year-3		1	Fall
9/2/2020	BL	1M	9	2020	Year-3	0	0	Fall
9/2/2020	BL	2M	9	2020	Year-3		1	Fall
9/2/2020	BL	3M	9	2020	Year-3	0	0	Fall
9/2/2020	BL	4M	9	2020	Year-3	0	0	Fall
9/2/2020	BL	5M	9	2020	Year-3		1	Fall

Date	Species	Site	Month	Year	Survey Year	Presence	Data Gap	Season
9/2/2020	BL	6M	9	2020	Year-3		1	Fall
9/2/2020	BL	7M	9	2020	Year-3		1	Fall
9/2/2020	BL	10M	9	2020	Year-3	0	0	Fall
9/2/2020	BL	12M	9	2020	Year-3	0	0	Fall
9/2/2020	BL	14M	9	2020	Year-3	0	0	Fall
9/2/2020	BL	8A	9	2020	Year-3		1	Fall
9/2/2020	BL	9A	9	2020	Year-3		1	Fall
9/2/2020	BL	11A	9	2020	Year-3		1	Fall
9/2/2020	BL	13A	9	2020	Year-3		1	Fall
9/3/2020	BL	1M	9	2020	Year-3	0	0	Fall
9/3/2020	BL	2M	9	2020	Year-3		1	Fall
9/3/2020	BL	3M	9	2020	Year-3	0	0	Fall
9/3/2020	BL	4M	9	2020	Year-3	0	0	Fall
9/3/2020	BL	5M	9	2020	Year-3		1	Fall
9/3/2020	BL	6M	9	2020	Year-3		1	Fall
9/3/2020	BL	7M	9	2020	Year-3		1	Fall
9/3/2020	BL	10M	9	2020	Year-3	0	0	Fall
9/3/2020	BL	12M	9	2020	Year-3	0	0	Fall
9/3/2020	BL	14M	9	2020	Year-3	0	0	Fall
9/3/2020	BL	8A	9	2020	Year-3		1	Fall
9/3/2020	BL	9A	9	2020	Year-3		1	Fall
9/3/2020	BL	11A	9	2020	Year-3		1	Fall
9/3/2020	BL	13A	9	2020	Year-3		1	Fall
9/4/2020	BL	1M	9	2020	Year-3	0	0	Fall
9/4/2020	BL	2M	9	2020	Year-3		1	Fall
9/4/2020	BL	3M	9	2020	Year-3	0	0	Fall
9/4/2020	BL	4M	9	2020	Year-3	0	0	Fall
9/4/2020	BL	5M	9	2020	Year-3		1	Fall
9/4/2020	BL	6M	9	2020	Year-3		1	Fall
9/4/2020	BL	7M	9	2020	Year-3		1	Fall
9/4/2020	BL	10M	9	2020	Year-3	0	0	Fall
9/4/2020	BL	12M	9	2020	Year-3	0	0	Fall
9/4/2020	BL	14M	9	2020	Year-3	0	0	Fall
9/4/2020	BL	8A	9	2020	Year-3		1	Fall
9/4/2020	BL	9A	9	2020	Year-3		1	Fall
9/4/2020	BL	11A	9	2020	Year-3		1	Fall
9/4/2020	BL	13A	9	2020	Year-3		1	Fall
9/5/2020	BL	1M	9	2020	Year-3	0	0	Fall
9/5/2020	BL	2M	9	2020	Year-3		1	Fall
9/5/2020	BL	3M	9	2020	Year-3	0	0	Fall
9/5/2020	BL	4M	9	2020	Year-3	0	0	Fall
9/5/2020	BL	5M	9	2020	Year-3		1	Fall
9/5/2020	BL	6M	9	2020	Year-3		1	Fall
9/5/2020	BL	7M	9	2020	Year-3		1	Fall

Date	Species	Site	Month	Year	Survey Year	Presence	Data Gap	Season
9/5/2020	BL	10M	9	2020	Year-3	0	0	Fall
9/5/2020	BL	12M	9	2020	Year-3	0	0	Fall
9/5/2020	BL	14M	9	2020	Year-3	0	0	Fall
9/5/2020	BL	8A	9	2020	Year-3		1	Fall
9/5/2020	BL	9A	9	2020	Year-3		1	Fall
9/5/2020	BL	11A	9	2020	Year-3		1	Fall
9/5/2020	BL	13A	9	2020	Year-3		1	Fall
9/6/2020	BL	1M	9	2020	Year-3	0	0	Fall
9/6/2020	BL	2M	9	2020	Year-3		1	Fall
9/6/2020	BL	3M	9	2020	Year-3	0	0	Fall
9/6/2020	BL	4M	9	2020	Year-3	0	0	Fall
9/6/2020	BL	5M	9	2020	Year-3		1	Fall
9/6/2020	BL	6M	9	2020	Year-3		1	Fall
9/6/2020	BL	7M	9	2020	Year-3		1	Fall
9/6/2020	BL	10M	9	2020	Year-3	0	0	Fall
9/6/2020	BL	12M	9	2020	Year-3	0	0	Fall
9/6/2020	BL	14M	9	2020	Year-3	0	0	Fall
9/6/2020	BL	8A	9	2020	Year-3		1	Fall
9/6/2020	BL	9A	9	2020	Year-3		1	Fall
9/6/2020	BL	11A	9	2020	Year-3		1	Fall
9/6/2020	BL	13A	9	2020	Year-3		1	Fall
9/7/2020	BL	1M	9	2020	Year-3	0	0	Fall
9/7/2020	BL	2M	9	2020	Year-3		1	Fall
9/7/2020	BL	3M	9	2020	Year-3	0	0	Fall
9/7/2020	BL	4M	9	2020	Year-3	0	0	Fall
9/7/2020	BL	5M	9	2020	Year-3		1	Fall
9/7/2020	BL	6M	9	2020	Year-3		1	Fall
9/7/2020	BL	7M	9	2020	Year-3		1	Fall
9/7/2020	BL	10M	9	2020	Year-3	0	0	Fall
9/7/2020	BL	12M	9	2020	Year-3	0	0	Fall
9/7/2020	BL	14M	9	2020	Year-3	0	0	Fall
9/7/2020	BL	8A	9	2020	Year-3		1	Fall
9/7/2020	BL	9A	9	2020	Year-3		1	Fall
9/7/2020	BL	11A	9	2020	Year-3		1	Fall
9/7/2020	BL	13A	9	2020	Year-3		1	Fall
9/8/2020	BL	1M	9	2020	Year-3	0	0	Fall
9/8/2020	BL	2M	9	2020	Year-3		1	Fall
9/8/2020	BL	3M	9	2020	Year-3	0	0	Fall
9/8/2020	BL	4M	9	2020	Year-3	0	0	Fall
9/8/2020	BL	5M	9	2020	Year-3		1	Fall
9/8/2020	BL	6M	9	2020	Year-3		1	Fall
9/8/2020	BL	7M	9	2020	Year-3		1	Fall
9/8/2020	BL	10M	9	2020	Year-3	0	0	Fall
9/8/2020	BL	12M	9	2020	Year-3	0	0	Fall

Date	Species	Site	Month	Year	Survey Year	Presence	Data Gap	Season
9/8/2020	BL	14M	9	2020	Year-3	0	0	Fall
9/8/2020	BL	8A	9	2020	Year-3		1	Fall
9/8/2020	BL	9A	9	2020	Year-3		1	Fall
9/8/2020	BL	11A	9	2020	Year-3		1	Fall
9/8/2020	BL	13A	9	2020	Year-3		1	Fall
9/9/2020	BL	1M	9	2020	Year-3	0	0	Fall
9/9/2020	BL	2M	9	2020	Year-3		1	Fall
9/9/2020	BL	3M	9	2020	Year-3	0	0	Fall
9/9/2020	BL	4M	9	2020	Year-3	0	0	Fall
9/9/2020	BL	5M	9	2020	Year-3		1	Fall
9/9/2020	BL	6M	9	2020	Year-3		1	Fall
9/9/2020	BL	7M	9	2020	Year-3		1	Fall
9/9/2020	BL	10M	9	2020	Year-3	0	0	Fall
9/9/2020	BL	12M	9	2020	Year-3	0	0	Fall
9/9/2020	BL	14M	9	2020	Year-3	0	0	Fall
9/9/2020	BL	8A	9	2020	Year-3		1	Fall
9/9/2020	BL	9A	9	2020	Year-3		1	Fall
9/9/2020	BL	11A	9	2020	Year-3		1	Fall
9/9/2020	BL	13A	9	2020	Year-3		1	Fall
9/10/2020	BL	1M	9	2020	Year-3	0	0	Fall
9/10/2020	BL	2M	9	2020	Year-3		1	Fall
9/10/2020	BL	3M	9	2020	Year-3	0	0	Fall
9/10/2020	BL	4M	9	2020	Year-3	0	0	Fall
9/10/2020	BL	5M	9	2020	Year-3		1	Fall
9/10/2020	BL	6M	9	2020	Year-3		1	Fall
9/10/2020	BL	7M	9	2020	Year-3		1	Fall
9/10/2020	BL	10M	9	2020	Year-3	0	0	Fall
9/10/2020	BL	12M	9	2020	Year-3	0	0	Fall
9/10/2020	BL	14M	9	2020	Year-3	0	0	Fall
9/10/2020	BL	8A	9	2020	Year-3		1	Fall
9/10/2020	BL	9A	9	2020	Year-3		1	Fall
9/10/2020	BL	11A	9	2020	Year-3		1	Fall
9/10/2020	BL	13A	9	2020	Year-3		1	Fall
9/11/2020	BL	1M	9	2020	Year-3	0	0	Fall
9/11/2020	BL	2M	9	2020	Year-3		1	Fall
9/11/2020	BL	3M	9	2020	Year-3	0	0	Fall
9/11/2020	BL	4M	9	2020	Year-3	0	0	Fall
9/11/2020	BL	5M	9	2020	Year-3		1	Fall
9/11/2020	BL	6M	9	2020	Year-3		1	Fall
9/11/2020	BL	7M	9	2020	Year-3		1	Fall
9/11/2020	BL	10M	9	2020	Year-3	0	0	Fall
9/11/2020	BL	12M	9	2020	Year-3	0	0	Fall
9/11/2020	BL	14M	9	2020	Year-3	0	0	Fall
9/11/2020	BL	8A	9	2020	Year-3		1	Fall

Date	Species	Site	Month	Year	Survey Year	Presence	Data Gap	Season
9/11/2020	BL	9A	9	2020	Year-3		1	Fall
9/11/2020	BL	11A	9	2020	Year-3		1	Fall
9/11/2020	BL	13A	9	2020	Year-3		1	Fall
9/12/2020	BL	1M	9	2020	Year-3	0	0	Fall
9/12/2020	BL	2M	9	2020	Year-3		1	Fall
9/12/2020	BL	3M	9	2020	Year-3	0	0	Fall
9/12/2020	BL	4M	9	2020	Year-3	0	0	Fall
9/12/2020	BL	5M	9	2020	Year-3		1	Fall
9/12/2020	BL	6M	9	2020	Year-3		1	Fall
9/12/2020	BL	7M	9	2020	Year-3		1	Fall
9/12/2020	BL	10M	9	2020	Year-3	0	0	Fall
9/12/2020	BL	12M	9	2020	Year-3	0	0	Fall
9/12/2020	BL	14M	9	2020	Year-3	0	0	Fall
9/12/2020	BL	8A	9	2020	Year-3		1	Fall
9/12/2020	BL	9A	9	2020	Year-3		1	Fall
9/12/2020	BL	11A	9	2020	Year-3		1	Fall
9/12/2020	BL	13A	9	2020	Year-3		1	Fall
9/13/2020	BL	1M	9	2020	Year-3	0	0	Fall
9/13/2020	BL	2M	9	2020	Year-3		1	Fall
9/13/2020	BL	3M	9	2020	Year-3	0	0	Fall
9/13/2020	BL	4M	9	2020	Year-3	0	0	Fall
9/13/2020	BL	5M	9	2020	Year-3		1	Fall
9/13/2020	BL	6M	9	2020	Year-3		1	Fall
9/13/2020	BL	7M	9	2020	Year-3		1	Fall
9/13/2020	BL	10M	9	2020	Year-3	0	0	Fall
9/13/2020	BL	12M	9	2020	Year-3	0	0	Fall
9/13/2020	BL	14M	9	2020	Year-3	0	0	Fall
9/13/2020	BL	8A	9	2020	Year-3		1	Fall
9/13/2020	BL	9A	9	2020	Year-3		1	Fall
9/13/2020	BL	11A	9	2020	Year-3		1	Fall
9/13/2020	BL	13A	9	2020	Year-3		1	Fall
9/14/2020	BL	1M	9	2020	Year-3	0	0	Fall
9/14/2020	BL	2M	9	2020	Year-3		1	Fall
9/14/2020	BL	3M	9	2020	Year-3	0	0	Fall
9/14/2020	BL	4M	9	2020	Year-3	0	0	Fall
9/14/2020	BL	5M	9	2020	Year-3		1	Fall
9/14/2020	BL	6M	9	2020	Year-3		1	Fall
9/14/2020	BL	7M	9	2020	Year-3		1	Fall
9/14/2020	BL	10M	9	2020	Year-3	0	0	Fall
9/14/2020	BL	12M	9	2020	Year-3	0	0	Fall
9/14/2020	BL	14M	9	2020	Year-3	0	0	Fall
9/14/2020	BL	8A	9	2020	Year-3		1	Fall
9/14/2020	BL	9A	9	2020	Year-3		1	Fall
9/14/2020	BL	11A	9	2020	Year-3		1	Fall

Date	Species	Site	Month	Year	Survey Year	Presence	Data Gap	Season
9/14/2020	BL	13A	9	2020	Year-3		1	Fall
9/15/2020	BL	1M	9	2020	Year-3	0	0	Fall
9/15/2020	BL	2M	9	2020	Year-3		1	Fall
9/15/2020	BL	3M	9	2020	Year-3	0	0	Fall
9/15/2020	BL	4M	9	2020	Year-3	0	0	Fall
9/15/2020	BL	5M	9	2020	Year-3		1	Fall
9/15/2020	BL	6M	9	2020	Year-3		1	Fall
9/15/2020	BL	7M	9	2020	Year-3		1	Fall
9/15/2020	BL	10M	9	2020	Year-3	0	0	Fall
9/15/2020	BL	12M	9	2020	Year-3	0	0	Fall
9/15/2020	BL	14M	9	2020	Year-3	0	0	Fall
9/15/2020	BL	8A	9	2020	Year-3		1	Fall
9/15/2020	BL	9A	9	2020	Year-3		1	Fall
9/15/2020	BL	11A	9	2020	Year-3		1	Fall
9/15/2020	BL	13A	9	2020	Year-3		1	Fall
9/16/2020	BL	1M	9	2020	Year-3	0	0	Fall
9/16/2020	BL	2M	9	2020	Year-3		1	Fall
9/16/2020	BL	3M	9	2020	Year-3	0	0	Fall
9/16/2020	BL	4M	9	2020	Year-3	0	0	Fall
9/16/2020	BL	5M	9	2020	Year-3		1	Fall
9/16/2020	BL	6M	9	2020	Year-3		1	Fall
9/16/2020	BL	7M	9	2020	Year-3		1	Fall
9/16/2020	BL	10M	9	2020	Year-3	0	0	Fall
9/16/2020	BL	12M	9	2020	Year-3	0	0	Fall
9/16/2020	BL	14M	9	2020	Year-3	0	0	Fall
9/16/2020	BL	8A	9	2020	Year-3		1	Fall
9/16/2020	BL	9A	9	2020	Year-3		1	Fall
9/16/2020	BL	11A	9	2020	Year-3		1	Fall
9/16/2020	BL	13A	9	2020	Year-3		1	Fall
9/17/2020	BL	1M	9	2020	Year-3	0	0	Fall
9/17/2020	BL	2M	9	2020	Year-3		1	Fall
9/17/2020	BL	3M	9	2020	Year-3	0	0	Fall
9/17/2020	BL	4M	9	2020	Year-3	0	0	Fall
9/17/2020	BL	5M	9	2020	Year-3		1	Fall
9/17/2020	BL	6M	9	2020	Year-3		1	Fall
9/17/2020	BL	7M	9	2020	Year-3		1	Fall
9/17/2020	BL	10M	9	2020	Year-3	0	0	Fall
9/17/2020	BL	12M	9	2020	Year-3	0	0	Fall
9/17/2020	BL	14M	9	2020	Year-3	0	0	Fall
9/17/2020	BL	8A	9	2020	Year-3		1	Fall
9/17/2020	BL	9A	9	2020	Year-3		1	Fall
9/17/2020	BL	11A	9	2020	Year-3		1	Fall
9/17/2020	BL	13A	9	2020	Year-3		1	Fall
9/18/2020	BL	1M	9	2020	Year-3	0	0	Fall

Date	Species	Site	Month	Year	Survey Year	Presence	Data Gap	Season
9/18/2020	BL	2M	9	2020	Year-3		1	Fall
9/18/2020	BL	3M	9	2020	Year-3	0	0	Fall
9/18/2020	BL	4M	9	2020	Year-3	0	0	Fall
9/18/2020	BL	5M	9	2020	Year-3		1	Fall
9/18/2020	BL	6M	9	2020	Year-3		1	Fall
9/18/2020	BL	7M	9	2020	Year-3		1	Fall
9/18/2020	BL	10M	9	2020	Year-3	0	0	Fall
9/18/2020	BL	12M	9	2020	Year-3	0	0	Fall
9/18/2020	BL	14M	9	2020	Year-3	0	0	Fall
9/18/2020	BL	8A	9	2020	Year-3		1	Fall
9/18/2020	BL	9A	9	2020	Year-3		1	Fall
9/18/2020	BL	11A	9	2020	Year-3		1	Fall
9/18/2020	BL	13A	9	2020	Year-3		1	Fall
9/19/2020	BL	1M	9	2020	Year-3	0	0	Fall
9/19/2020	BL	2M	9	2020	Year-3		1	Fall
9/19/2020	BL	3M	9	2020	Year-3	0	0	Fall
9/19/2020	BL	4M	9	2020	Year-3	0	0	Fall
9/19/2020	BL	5M	9	2020	Year-3		1	Fall
9/19/2020	BL	6M	9	2020	Year-3		1	Fall
9/19/2020	BL	7M	9	2020	Year-3		1	Fall
9/19/2020	BL	10M	9	2020	Year-3	0	0	Fall
9/19/2020	BL	12M	9	2020	Year-3	0	0	Fall
9/19/2020	BL	14M	9	2020	Year-3	0	0	Fall
9/19/2020	BL	8A	9	2020	Year-3		1	Fall
9/19/2020	BL	9A	9	2020	Year-3		1	Fall
9/19/2020	BL	11A	9	2020	Year-3		1	Fall
9/19/2020	BL	13A	9	2020	Year-3		1	Fall
9/20/2020	BL	1M	9	2020	Year-3	0	0	Fall
9/20/2020	BL	2M	9	2020	Year-3		1	Fall
9/20/2020	BL	3M	9	2020	Year-3	0	0	Fall
9/20/2020	BL	4M	9	2020	Year-3	0	0	Fall
9/20/2020	BL	5M	9	2020	Year-3		1	Fall
9/20/2020	BL	6M	9	2020	Year-3		1	Fall
9/20/2020	BL	7M	9	2020	Year-3		1	Fall
9/20/2020	BL	10M	9	2020	Year-3	0	0	Fall
9/20/2020	BL	12M	9	2020	Year-3	0	0	Fall
9/20/2020	BL	14M	9	2020	Year-3	0	0	Fall
9/20/2020	BL	8A	9	2020	Year-3		1	Fall
9/20/2020	BL	9A	9	2020	Year-3		1	Fall
9/20/2020	BL	11A	9	2020	Year-3		1	Fall
9/20/2020	BL	13A	9	2020	Year-3		1	Fall
9/21/2020	BL	1M	9	2020	Year-3	0	0	Fall
9/21/2020	BL	2M	9	2020	Year-3		1	Fall
9/21/2020	BL	3M	9	2020	Year-3	0	0	Fall

Date	Species	Site	Month	Year	Survey Year	Presence	Data Gap	Season
9/21/2020	BL	4M	9	2020	Year-3	0	0	Fall
9/21/2020	BL	5M	9	2020	Year-3		1	Fall
9/21/2020	BL	6M	9	2020	Year-3		1	Fall
9/21/2020	BL	7M	9	2020	Year-3		1	Fall
9/21/2020	BL	10M	9	2020	Year-3	0	0	Fall
9/21/2020	BL	12M	9	2020	Year-3	0	0	Fall
9/21/2020	BL	14M	9	2020	Year-3	0	0	Fall
9/21/2020	BL	8A	9	2020	Year-3		1	Fall
9/21/2020	BL	9A	9	2020	Year-3		1	Fall
9/21/2020	BL	11A	9	2020	Year-3		1	Fall
9/21/2020	BL	13A	9	2020	Year-3		1	Fall
9/22/2020	BL	1M	9	2020	Year-3	0	0	Fall
9/22/2020	BL	2M	9	2020	Year-3		1	Fall
9/22/2020	BL	3M	9	2020	Year-3	0	0	Fall
9/22/2020	BL	4M	9	2020	Year-3	0	0	Fall
9/22/2020	BL	5M	9	2020	Year-3		1	Fall
9/22/2020	BL	6M	9	2020	Year-3		1	Fall
9/22/2020	BL	7M	9	2020	Year-3		1	Fall
9/22/2020	BL	10M	9	2020	Year-3	0	0	Fall
9/22/2020	BL	12M	9	2020	Year-3	0	0	Fall
9/22/2020	BL	14M	9	2020	Year-3	0	0	Fall
9/22/2020	BL	8A	9	2020	Year-3		1	Fall
9/22/2020	BL	9A	9	2020	Year-3		1	Fall
9/22/2020	BL	11A	9	2020	Year-3		1	Fall
9/22/2020	BL	13A	9	2020	Year-3		1	Fall
9/23/2020	BL	1M	9	2020	Year-3	0	0	Fall
9/23/2020	BL	2M	9	2020	Year-3		1	Fall
9/23/2020	BL	3M	9	2020	Year-3	0	0	Fall
9/23/2020	BL	4M	9	2020	Year-3	0	0	Fall
9/23/2020	BL	5M	9	2020	Year-3		1	Fall
9/23/2020	BL	6M	9	2020	Year-3		1	Fall
9/23/2020	BL	7M	9	2020	Year-3		1	Fall
9/23/2020	BL	10M	9	2020	Year-3	0	0	Fall
9/23/2020	BL	12M	9	2020	Year-3	0	0	Fall
9/23/2020	BL	14M	9	2020	Year-3	0	0	Fall
9/23/2020	BL	8A	9	2020	Year-3		1	Fall
9/23/2020	BL	9A	9	2020	Year-3		1	Fall
9/23/2020	BL	11A	9	2020	Year-3		1	Fall
9/23/2020	BL	13A	9	2020	Year-3		1	Fall
9/24/2020	BL	1M	9	2020	Year-3	0	0	Fall
9/24/2020	BL	2M	9	2020	Year-3		1	Fall
9/24/2020	BL	3M	9	2020	Year-3	0	0	Fall
9/24/2020	BL	4M	9	2020	Year-3	0	0	Fall
9/24/2020	BL	5M	9	2020	Year-3		1	Fall

Date	Species	Site	Month	Year	Survey Year	Presence	Data Gap	Season
9/24/2020	BL	6M	9	2020	Year-3		1	Fall
9/24/2020	BL	7M	9	2020	Year-3		1	Fall
9/24/2020	BL	10M	9	2020	Year-3	0	0	Fall
9/24/2020	BL	12M	9	2020	Year-3	0	0	Fall
9/24/2020	BL	14M	9	2020	Year-3	0	0	Fall
9/24/2020	BL	8A	9	2020	Year-3		1	Fall
9/24/2020	BL	9A	9	2020	Year-3		1	Fall
9/24/2020	BL	11A	9	2020	Year-3		1	Fall
9/24/2020	BL	13A	9	2020	Year-3		1	Fall
9/25/2020	BL	1M	9	2020	Year-3	0	0	Fall
9/25/2020	BL	2M	9	2020	Year-3		1	Fall
9/25/2020	BL	3M	9	2020	Year-3	0	0	Fall
9/25/2020	BL	4M	9	2020	Year-3	0	0	Fall
9/25/2020	BL	5M	9	2020	Year-3		1	Fall
9/25/2020	BL	6M	9	2020	Year-3		1	Fall
9/25/2020	BL	7M	9	2020	Year-3		1	Fall
9/25/2020	BL	10M	9	2020	Year-3	0	0	Fall
9/25/2020	BL	12M	9	2020	Year-3	0	0	Fall
9/25/2020	BL	14M	9	2020	Year-3	0	0	Fall
9/25/2020	BL	8A	9	2020	Year-3		1	Fall
9/25/2020	BL	9A	9	2020	Year-3		1	Fall
9/25/2020	BL	11A	9	2020	Year-3		1	Fall
9/25/2020	BL	13A	9	2020	Year-3		1	Fall
9/26/2020	BL	1M	9	2020	Year-3	0	0	Fall
9/26/2020	BL	2M	9	2020	Year-3		1	Fall
9/26/2020	BL	3M	9	2020	Year-3	0	0	Fall
9/26/2020	BL	4M	9	2020	Year-3	0	0	Fall
9/26/2020	BL	5M	9	2020	Year-3		1	Fall
9/26/2020	BL	6M	9	2020	Year-3		1	Fall
9/26/2020	BL	7M	9	2020	Year-3		1	Fall
9/26/2020	BL	10M	9	2020	Year-3	0	0	Fall
9/26/2020	BL	12M	9	2020	Year-3	0	0	Fall
9/26/2020	BL	14M	9	2020	Year-3	0	0	Fall
9/26/2020	BL	8A	9	2020	Year-3		1	Fall
9/26/2020	BL	9A	9	2020	Year-3		1	Fall
9/26/2020	BL	11A	9	2020	Year-3		1	Fall
9/26/2020	BL	13A	9	2020	Year-3		1	Fall
9/27/2020	BL	1M	9	2020	Year-3	0	0	Fall
9/27/2020	BL	2M	9	2020	Year-3		1	Fall
9/27/2020	BL	3M	9	2020	Year-3	0	0	Fall
9/27/2020	BL	4M	9	2020	Year-3	0	0	Fall
9/27/2020	BL	5M	9	2020	Year-3		1	Fall
9/27/2020	BL	6M	9	2020	Year-3		1	Fall
9/27/2020	BL	7M	9	2020	Year-3		1	Fall

Date	Species	Site	Month	Year	Survey Year	Presence	Data Gap	Season
9/27/2020	BL	10M	9	2020	Year-3	0	0	Fall
9/27/2020	BL	12M	9	2020	Year-3	0	0	Fall
9/27/2020	BL	14M	9	2020	Year-3	0	0	Fall
9/27/2020	BL	8A	9	2020	Year-3		1	Fall
9/27/2020	BL	9A	9	2020	Year-3		1	Fall
9/27/2020	BL	11A	9	2020	Year-3		1	Fall
9/27/2020	BL	13A	9	2020	Year-3		1	Fall
9/28/2020	BL	1M	9	2020	Year-3	0	0	Fall
9/28/2020	BL	2M	9	2020	Year-3		1	Fall
9/28/2020	BL	3M	9	2020	Year-3	0	0	Fall
9/28/2020	BL	4M	9	2020	Year-3	0	0	Fall
9/28/2020	BL	5M	9	2020	Year-3		1	Fall
9/28/2020	BL	6M	9	2020	Year-3		1	Fall
9/28/2020	BL	7M	9	2020	Year-3		1	Fall
9/28/2020	BL	10M	9	2020	Year-3	0	0	Fall
9/28/2020	BL	12M	9	2020	Year-3	0	0	Fall
9/28/2020	BL	14M	9	2020	Year-3	0	0	Fall
9/28/2020	BL	8A	9	2020	Year-3		1	Fall
9/28/2020	BL	9A	9	2020	Year-3		1	Fall
9/28/2020	BL	11A	9	2020	Year-3		1	Fall
9/28/2020	BL	13A	9	2020	Year-3		1	Fall
9/29/2020	BL	1M	9	2020	Year-3	0	0	Fall
9/29/2020	BL	2M	9	2020	Year-3		1	Fall
9/29/2020	BL	3M	9	2020	Year-3	0	0	Fall
9/29/2020	BL	4M	9	2020	Year-3	0	0	Fall
9/29/2020	BL	5M	9	2020	Year-3		1	Fall
9/29/2020	BL	6M	9	2020	Year-3		1	Fall
9/29/2020	BL	7M	9	2020	Year-3		1	Fall
9/29/2020	BL	10M	9	2020	Year-3	0	0	Fall
9/29/2020	BL	12M	9	2020	Year-3	0	0	Fall
9/29/2020	BL	14M	9	2020	Year-3	0	0	Fall
9/29/2020	BL	8A	9	2020	Year-3		1	Fall
9/29/2020	BL	9A	9	2020	Year-3		1	Fall
9/29/2020	BL	11A	9	2020	Year-3		1	Fall
9/29/2020	BL	13A	9	2020	Year-3		1	Fall
9/30/2020	BL	1M	9	2020	Year-3	0	0	Fall
9/30/2020	BL	2M	9	2020	Year-3		1	Fall
9/30/2020	BL	3M	9	2020	Year-3	0	0	Fall
9/30/2020	BL	4M	9	2020	Year-3	0	0	Fall
9/30/2020	BL	5M	9	2020	Year-3		1	Fall
9/30/2020	BL	6M	9	2020	Year-3		1	Fall
9/30/2020	BL	7M	9	2020	Year-3		1	Fall
9/30/2020	BL	10M	9	2020	Year-3	0	0	Fall
9/30/2020	BL	12M	9	2020	Year-3	0	0	Fall

Date	Species	Site	Month	Year	Survey Year	Presence	Data Gap	Season
9/30/2020	BL	14M	9	2020	Year-3	0	0	Fall
9/30/2020	BL	8A	9	2020	Year-3		1	Fall
9/30/2020	BL	9A	9	2020	Year-3		1	Fall
9/30/2020	BL	11A	9	2020	Year-3		1	Fall
9/30/2020	BL	13A	9	2020	Year-3		1	Fall
10/1/2020	BL	1M	10	2020	Year-3	0	0	Fall
10/1/2020	BL	2M	10	2020	Year-3		1	Fall
10/1/2020	BL	3M	10	2020	Year-3	0	0	Fall
10/1/2020	BL	4M	10	2020	Year-3	0	0	Fall
10/1/2020	BL	5M	10	2020	Year-3		1	Fall
10/1/2020	BL	6M	10	2020	Year-3		1	Fall
10/1/2020	BL	7M	10	2020	Year-3		1	Fall
10/1/2020	BL	10M	10	2020	Year-3	0	0	Fall
10/1/2020	BL	12M	10	2020	Year-3	0	0	Fall
10/1/2020	BL	14M	10	2020	Year-3	0	0	Fall
10/1/2020	BL	8A	10	2020	Year-3		1	Fall
10/1/2020	BL	9A	10	2020	Year-3		1	Fall
10/1/2020	BL	11A	10	2020	Year-3		1	Fall
10/1/2020	BL	13A	10	2020	Year-3		1	Fall
10/2/2020	BL	1M	10	2020	Year-3	0	0	Fall
10/2/2020	BL	2M	10	2020	Year-3		1	Fall
10/2/2020	BL	3M	10	2020	Year-3	0	0	Fall
10/2/2020	BL	4M	10	2020	Year-3	0	0	Fall
10/2/2020	BL	5M	10	2020	Year-3		1	Fall
10/2/2020	BL	6M	10	2020	Year-3		1	Fall
10/2/2020	BL	7M	10	2020	Year-3		1	Fall
10/2/2020	BL	10M	10	2020	Year-3	0	0	Fall
10/2/2020	BL	12M	10	2020	Year-3	0	0	Fall
10/2/2020	BL	14M	10	2020	Year-3	0	0	Fall
10/2/2020	BL	8A	10	2020	Year-3		1	Fall
10/2/2020	BL	9A	10	2020	Year-3		1	Fall
10/2/2020	BL	11A	10	2020	Year-3		1	Fall
10/2/2020	BL	13A	10	2020	Year-3		1	Fall
10/3/2020	BL	1M	10	2020	Year-3	0	0	Fall
10/3/2020	BL	2M	10	2020	Year-3		1	Fall
10/3/2020	BL	3M	10	2020	Year-3	0	0	Fall
10/3/2020	BL	4M	10	2020	Year-3	0	0	Fall
10/3/2020	BL	5M	10	2020	Year-3		1	Fall
10/3/2020	BL	6M	10	2020	Year-3		1	Fall
10/3/2020	BL	7M	10	2020	Year-3		1	Fall
10/3/2020	BL	10M	10	2020	Year-3	0	0	Fall
10/3/2020	BL	12M	10	2020	Year-3	0	0	Fall
10/3/2020	BL	14M	10	2020	Year-3	0	0	Fall
10/3/2020	BL	8A	10	2020	Year-3		1	Fall

Date	Species	Site	Month	Year	Survey Year	Presence	Data Gap	Season
10/3/2020	BL	9A	10	2020	Year-3		1	Fall
10/3/2020	BL	11A	10	2020	Year-3		1	Fall
10/3/2020	BL	13A	10	2020	Year-3		1	Fall
10/4/2020	BL	1M	10	2020	Year-3	0	0	Fall
10/4/2020	BL	2M	10	2020	Year-3		1	Fall
10/4/2020	BL	3M	10	2020	Year-3	0	0	Fall
10/4/2020	BL	4M	10	2020	Year-3	0	0	Fall
10/4/2020	BL	5M	10	2020	Year-3		1	Fall
10/4/2020	BL	6M	10	2020	Year-3		1	Fall
10/4/2020	BL	7M	10	2020	Year-3		1	Fall
10/4/2020	BL	10M	10	2020	Year-3	0	0	Fall
10/4/2020	BL	12M	10	2020	Year-3	0	0	Fall
10/4/2020	BL	14M	10	2020	Year-3	0	0	Fall
10/4/2020	BL	8A	10	2020	Year-3		1	Fall
10/4/2020	BL	9A	10	2020	Year-3		1	Fall
10/4/2020	BL	11A	10	2020	Year-3		1	Fall
10/4/2020	BL	13A	10	2020	Year-3		1	Fall
10/5/2020	BL	1M	10	2020	Year-3	0	0	Fall
10/5/2020	BL	2M	10	2020	Year-3		1	Fall
10/5/2020	BL	3M	10	2020	Year-3	0	0	Fall
10/5/2020	BL	4M	10	2020	Year-3	0	0	Fall
10/5/2020	BL	5M	10	2020	Year-3		1	Fall
10/5/2020	BL	6M	10	2020	Year-3		1	Fall
10/5/2020	BL	7M	10	2020	Year-3		1	Fall
10/5/2020	BL	10M	10	2020	Year-3	0	0	Fall
10/5/2020	BL	12M	10	2020	Year-3	0	0	Fall
10/5/2020	BL	14M	10	2020	Year-3	0	0	Fall
10/5/2020	BL	8A	10	2020	Year-3		1	Fall
10/5/2020	BL	9A	10	2020	Year-3		1	Fall
10/5/2020	BL	11A	10	2020	Year-3		1	Fall
10/5/2020	BL	13A	10	2020	Year-3		1	Fall
10/6/2020	BL	1M	10	2020	Year-3	0	0	Fall
10/6/2020	BL	2M	10	2020	Year-3		1	Fall
10/6/2020	BL	3M	10	2020	Year-3	0	0	Fall
10/6/2020	BL	4M	10	2020	Year-3		1	Fall
10/6/2020	BL	5M	10	2020	Year-3		1	Fall
10/6/2020	BL	6M	10	2020	Year-3		1	Fall
10/6/2020	BL	7M	10	2020	Year-3		1	Fall
10/6/2020	BL	10M	10	2020	Year-3	0	0	Fall
10/6/2020	BL	12M	10	2020	Year-3	0	0	Fall
10/6/2020	BL	14M	10	2020	Year-3	0	0	Fall
10/6/2020	BL	8A	10	2020	Year-3		1	Fall
10/6/2020	BL	9A	10	2020	Year-3		1	Fall
10/6/2020	BL	11A	10	2020	Year-3		1	Fall

Date	Species	Site	Month	Year	Survey Year	Presence	Data Gap	Season
10/6/2020	BL	13A	10	2020	Year-3		1	Fall
10/7/2020	BL	1M	10	2020	Year-3	0	0	Fall
10/7/2020	BL	2M	10	2020	Year-3		1	Fall
10/7/2020	BL	3M	10	2020	Year-3	0	0	Fall
10/7/2020	BL	4M	10	2020	Year-3		1	Fall
10/7/2020	BL	5M	10	2020	Year-3		1	Fall
10/7/2020	BL	6M	10	2020	Year-3		1	Fall
10/7/2020	BL	7M	10	2020	Year-3		1	Fall
10/7/2020	BL	10M	10	2020	Year-3	0	0	Fall
10/7/2020	BL	12M	10	2020	Year-3	0	0	Fall
10/7/2020	BL	14M	10	2020	Year-3	0	0	Fall
10/7/2020	BL	8A	10	2020	Year-3		1	Fall
10/7/2020	BL	9A	10	2020	Year-3		1	Fall
10/7/2020	BL	11A	10	2020	Year-3		1	Fall
10/7/2020	BL	13A	10	2020	Year-3		1	Fall
10/8/2020	BL	1M	10	2020	Year-3	0	0	Fall
10/8/2020	BL	2M	10	2020	Year-3		1	Fall
10/8/2020	BL	3M	10	2020	Year-3	0	0	Fall
10/8/2020	BL	4M	10	2020	Year-3		1	Fall
10/8/2020	BL	5M	10	2020	Year-3		1	Fall
10/8/2020	BL	6M	10	2020	Year-3		1	Fall
10/8/2020	BL	7M	10	2020	Year-3		1	Fall
10/8/2020	BL	10M	10	2020	Year-3	0	0	Fall
10/8/2020	BL	12M	10	2020	Year-3	0	0	Fall
10/8/2020	BL	14M	10	2020	Year-3	0	0	Fall
10/8/2020	BL	8A	10	2020	Year-3		1	Fall
10/8/2020	BL	9A	10	2020	Year-3		1	Fall
10/8/2020	BL	11A	10	2020	Year-3		1	Fall
10/8/2020	BL	13A	10	2020	Year-3		1	Fall
10/9/2020	BL	1M	10	2020	Year-3	0	0	Fall
10/9/2020	BL	2M	10	2020	Year-3		1	Fall
10/9/2020	BL	3M	10	2020	Year-3	0	0	Fall
10/9/2020	BL	4M	10	2020	Year-3		1	Fall
10/9/2020	BL	5M	10	2020	Year-3		1	Fall
10/9/2020	BL	6M	10	2020	Year-3		1	Fall
10/9/2020	BL	7M	10	2020	Year-3		1	Fall
10/9/2020	BL	10M	10	2020	Year-3	0	0	Fall
10/9/2020	BL	12M	10	2020	Year-3	0	0	Fall
10/9/2020	BL	14M	10	2020	Year-3	0	0	Fall
10/9/2020	BL	8A	10	2020	Year-3		1	Fall
10/9/2020	BL	9A	10	2020	Year-3		1	Fall
10/9/2020	BL	11A	10	2020	Year-3		1	Fall
10/9/2020	BL	13A	10	2020	Year-3		1	Fall
10/10/2020	BL	1M	10	2020	Year-3	0	0	Fall

Date	Species	Site	Month	Year	Survey Year	Presence	Data Gap	Season
10/10/2020	BL	2M	10	2020	Year-3		1	Fall
10/10/2020	BL	3M	10	2020	Year-3	0	0	Fall
10/10/2020	BL	4M	10	2020	Year-3		1	Fall
10/10/2020	BL	5M	10	2020	Year-3		1	Fall
10/10/2020	BL	6M	10	2020	Year-3		1	Fall
10/10/2020	BL	7M	10	2020	Year-3		1	Fall
10/10/2020	BL	10M	10	2020	Year-3	0	0	Fall
10/10/2020	BL	12M	10	2020	Year-3	0	0	Fall
10/10/2020	BL	14M	10	2020	Year-3	0	0	Fall
10/10/2020	BL	8A	10	2020	Year-3		1	Fall
10/10/2020	BL	9A	10	2020	Year-3		1	Fall
10/10/2020	BL	11A	10	2020	Year-3		1	Fall
10/10/2020	BL	13A	10	2020	Year-3		1	Fall
10/11/2020	BL	1M	10	2020	Year-3	0	0	Fall
10/11/2020	BL	2M	10	2020	Year-3		1	Fall
10/11/2020	BL	3M	10	2020	Year-3	0	0	Fall
10/11/2020	BL	4M	10	2020	Year-3		1	Fall
10/11/2020	BL	5M	10	2020	Year-3		1	Fall
10/11/2020	BL	6M	10	2020	Year-3		1	Fall
10/11/2020	BL	7M	10	2020	Year-3		1	Fall
10/11/2020	BL	10M	10	2020	Year-3	0	0	Fall
10/11/2020	BL	12M	10	2020	Year-3	0	0	Fall
10/11/2020	BL	14M	10	2020	Year-3	0	0	Fall
10/11/2020	BL	8A	10	2020	Year-3		1	Fall
10/11/2020	BL	9A	10	2020	Year-3		1	Fall
10/11/2020	BL	11A	10	2020	Year-3		1	Fall
10/11/2020	BL	13A	10	2020	Year-3		1	Fall
10/12/2020	BL	1M	10	2020	Year-3	0	0	Fall
10/12/2020	BL	2M	10	2020	Year-3		1	Fall
10/12/2020	BL	3M	10	2020	Year-3	0	0	Fall
10/12/2020	BL	4M	10	2020	Year-3		1	Fall
10/12/2020	BL	5M	10	2020	Year-3		1	Fall
10/12/2020	BL	6M	10	2020	Year-3		1	Fall
10/12/2020	BL	7M	10	2020	Year-3		1	Fall
10/12/2020	BL	10M	10	2020	Year-3	0	0	Fall
10/12/2020	BL	12M	10	2020	Year-3	0	0	Fall
10/12/2020	BL	14M	10	2020	Year-3	0	0	Fall
10/12/2020	BL	8A	10	2020	Year-3		1	Fall
10/12/2020	BL	9A	10	2020	Year-3		1	Fall
10/12/2020	BL	11A	10	2020	Year-3		1	Fall
10/12/2020	BL	13A	10	2020	Year-3		1	Fall
10/13/2020	BL	1M	10	2020	Year-3	0	0	Fall
10/13/2020	BL	2M	10	2020	Year-3		1	Fall
10/13/2020	BL	3M	10	2020	Year-3	0	0	Fall

Date	Species	Site	Month	Year	Survey Year	Presence	Data Gap	Season
10/13/2020	BL	4M	10	2020	Year-3		1	Fall
10/13/2020	BL	5M	10	2020	Year-3		1	Fall
10/13/2020	BL	6M	10	2020	Year-3		1	Fall
10/13/2020	BL	7M	10	2020	Year-3		1	Fall
10/13/2020	BL	10M	10	2020	Year-3	0	0	Fall
10/13/2020	BL	12M	10	2020	Year-3	0	0	Fall
10/13/2020	BL	14M	10	2020	Year-3	0	0	Fall
10/13/2020	BL	8A	10	2020	Year-3		1	Fall
10/13/2020	BL	9A	10	2020	Year-3		1	Fall
10/13/2020	BL	11A	10	2020	Year-3		1	Fall
10/13/2020	BL	13A	10	2020	Year-3		1	Fall
10/14/2020	BL	1M	10	2020	Year-3	0	0	Fall
10/14/2020	BL	2M	10	2020	Year-3		1	Fall
10/14/2020	BL	3M	10	2020	Year-3	0	0	Fall
10/14/2020	BL	4M	10	2020	Year-3		1	Fall
10/14/2020	BL	5M	10	2020	Year-3		1	Fall
10/14/2020	BL	6M	10	2020	Year-3		1	Fall
10/14/2020	BL	7M	10	2020	Year-3		1	Fall
10/14/2020	BL	10M	10	2020	Year-3	0	0	Fall
10/14/2020	BL	12M	10	2020	Year-3	0	0	Fall
10/14/2020	BL	14M	10	2020	Year-3	0	0	Fall
10/14/2020	BL	8A	10	2020	Year-3		1	Fall
10/14/2020	BL	9A	10	2020	Year-3		1	Fall
10/14/2020	BL	11A	10	2020	Year-3		1	Fall
10/14/2020	BL	13A	10	2020	Year-3		1	Fall
10/15/2020	BL	1M	10	2020	Year-3	0	0	Fall
10/15/2020	BL	2M	10	2020	Year-3		1	Fall
10/15/2020	BL	3M	10	2020	Year-3	0	0	Fall
10/15/2020	BL	4M	10	2020	Year-3		1	Fall
10/15/2020	BL	5M	10	2020	Year-3		1	Fall
10/15/2020	BL	6M	10	2020	Year-3		1	Fall
10/15/2020	BL	7M	10	2020	Year-3		1	Fall
10/15/2020	BL	10M	10	2020	Year-3	0	0	Fall
10/15/2020	BL	12M	10	2020	Year-3	0	0	Fall
10/15/2020	BL	14M	10	2020	Year-3	0	0	Fall
10/15/2020	BL	8A	10	2020	Year-3		1	Fall
10/15/2020	BL	9A	10	2020	Year-3		1	Fall
10/15/2020	BL	11A	10	2020	Year-3		1	Fall
10/15/2020	BL	13A	10	2020	Year-3		1	Fall
10/16/2020	BL	1M	10	2020	Year-3	0	0	Fall
10/16/2020	BL	2M	10	2020	Year-3		1	Fall
10/16/2020	BL	3M	10	2020	Year-3	0	0	Fall
10/16/2020	BL	4M	10	2020	Year-3		1	Fall
10/16/2020	BL	5M	10	2020	Year-3		1	Fall

Date	Species	Site	Month	Year	Survey Year	Presence	Data Gap	Season
10/16/2020	BL	6M	10	2020	Year-3		1	Fall
10/16/2020	BL	7M	10	2020	Year-3		1	Fall
10/16/2020	BL	10M	10	2020	Year-3	0	0	Fall
10/16/2020	BL	12M	10	2020	Year-3	0	0	Fall
10/16/2020	BL	14M	10	2020	Year-3	0	0	Fall
10/16/2020	BL	8A	10	2020	Year-3		1	Fall
10/16/2020	BL	9A	10	2020	Year-3		1	Fall
10/16/2020	BL	11A	10	2020	Year-3		1	Fall
10/16/2020	BL	13A	10	2020	Year-3		1	Fall
10/17/2020	BL	1M	10	2020	Year-3	0	0	Fall
10/17/2020	BL	2M	10	2020	Year-3		1	Fall
10/17/2020	BL	3M	10	2020	Year-3	0	0	Fall
10/17/2020	BL	4M	10	2020	Year-3		1	Fall
10/17/2020	BL	5M	10	2020	Year-3		1	Fall
10/17/2020	BL	6M	10	2020	Year-3		1	Fall
10/17/2020	BL	7M	10	2020	Year-3		1	Fall
10/17/2020	BL	10M	10	2020	Year-3	0	0	Fall
10/17/2020	BL	12M	10	2020	Year-3	0	0	Fall
10/17/2020	BL	14M	10	2020	Year-3	0	0	Fall
10/17/2020	BL	8A	10	2020	Year-3		1	Fall
10/17/2020	BL	9A	10	2020	Year-3		1	Fall
10/17/2020	BL	11A	10	2020	Year-3		1	Fall
10/17/2020	BL	13A	10	2020	Year-3		1	Fall
10/18/2020	BL	1M	10	2020	Year-3	0	0	Fall
10/18/2020	BL	2M	10	2020	Year-3		1	Fall
10/18/2020	BL	3M	10	2020	Year-3	0	0	Fall
10/18/2020	BL	4M	10	2020	Year-3		1	Fall
10/18/2020	BL	5M	10	2020	Year-3		1	Fall
10/18/2020	BL	6M	10	2020	Year-3		1	Fall
10/18/2020	BL	7M	10	2020	Year-3		1	Fall
10/18/2020	BL	10M	10	2020	Year-3	0	0	Fall
10/18/2020	BL	12M	10	2020	Year-3	0	0	Fall
10/18/2020	BL	14M	10	2020	Year-3	0	0	Fall
10/18/2020	BL	8A	10	2020	Year-3		1	Fall
10/18/2020	BL	9A	10	2020	Year-3		1	Fall
10/18/2020	BL	11A	10	2020	Year-3		1	Fall
10/18/2020	BL	13A	10	2020	Year-3		1	Fall
10/19/2020	BL	1M	10	2020	Year-3	0	0	Fall
10/19/2020	BL	2M	10	2020	Year-3		1	Fall
10/19/2020	BL	3M	10	2020	Year-3	0	0	Fall
10/19/2020	BL	4M	10	2020	Year-3		1	Fall
10/19/2020	BL	5M	10	2020	Year-3		1	Fall
10/19/2020	BL	6M	10	2020	Year-3		1	Fall
10/19/2020	BL	7M	10	2020	Year-3		1	Fall

Date	Species	Site	Month	Year	Survey Year	Presence	Data Gap	Season
10/19/2020	BL	10M	10	2020	Year-3	0	0	Fall
10/19/2020	BL	12M	10	2020	Year-3	0	0	Fall
10/19/2020	BL	14M	10	2020	Year-3	0	0	Fall
10/19/2020	BL	8A	10	2020	Year-3		1	Fall
10/19/2020	BL	9A	10	2020	Year-3		1	Fall
10/19/2020	BL	11A	10	2020	Year-3		1	Fall
10/19/2020	BL	13A	10	2020	Year-3		1	Fall
10/20/2020	BL	1M	10	2020	Year-3	0	0	Fall
10/20/2020	BL	2M	10	2020	Year-3		1	Fall
10/20/2020	BL	3M	10	2020	Year-3	0	0	Fall
10/20/2020	BL	4M	10	2020	Year-3		1	Fall
10/20/2020	BL	5M	10	2020	Year-3		1	Fall
10/20/2020	BL	6M	10	2020	Year-3		1	Fall
10/20/2020	BL	7M	10	2020	Year-3		1	Fall
10/20/2020	BL	10M	10	2020	Year-3	0	0	Fall
10/20/2020	BL	12M	10	2020	Year-3	0	0	Fall
10/20/2020	BL	14M	10	2020	Year-3	0	0	Fall
10/20/2020	BL	8A	10	2020	Year-3		1	Fall
10/20/2020	BL	9A	10	2020	Year-3		1	Fall
10/20/2020	BL	11A	10	2020	Year-3		1	Fall
10/20/2020	BL	13A	10	2020	Year-3		1	Fall
10/21/2020	BL	1M	10	2020	Year-3	0	0	Fall
10/21/2020	BL	2M	10	2020	Year-3		1	Fall
10/21/2020	BL	3M	10	2020	Year-3	0	0	Fall
10/21/2020	BL	4M	10	2020	Year-3		1	Fall
10/21/2020	BL	5M	10	2020	Year-3		1	Fall
10/21/2020	BL	6M	10	2020	Year-3		1	Fall
10/21/2020	BL	7M	10	2020	Year-3		1	Fall
10/21/2020	BL	10M	10	2020	Year-3	0	0	Fall
10/21/2020	BL	12M	10	2020	Year-3	0	0	Fall
10/21/2020	BL	14M	10	2020	Year-3	0	0	Fall
10/21/2020	BL	8A	10	2020	Year-3		1	Fall
10/21/2020	BL	9A	10	2020	Year-3		1	Fall
10/21/2020	BL	11A	10	2020	Year-3		1	Fall
10/21/2020	BL	13A	10	2020	Year-3		1	Fall
10/22/2020	BL	1M	10	2020	Year-3	0	0	Fall
10/22/2020	BL	2M	10	2020	Year-3		1	Fall
10/22/2020	BL	3M	10	2020	Year-3	0	0	Fall
10/22/2020	BL	4M	10	2020	Year-3		1	Fall
10/22/2020	BL	5M	10	2020	Year-3		1	Fall
10/22/2020	BL	6M	10	2020	Year-3		1	Fall
10/22/2020	BL	7M	10	2020	Year-3		1	Fall
10/22/2020	BL	10M	10	2020	Year-3	0	0	Fall
10/22/2020	BL	12M	10	2020	Year-3	0	0	Fall

Date	Species	Site	Month	Year	Survey Year	Presence	Data Gap	Season
10/22/2020	BL	14M	10	2020	Year-3	0	0	Fall
10/22/2020	BL	8A	10	2020	Year-3		1	Fall
10/22/2020	BL	9A	10	2020	Year-3		1	Fall
10/22/2020	BL	11A	10	2020	Year-3		1	Fall
10/22/2020	BL	13A	10	2020	Year-3		1	Fall
10/23/2020	BL	1M	10	2020	Year-3	0	0	Fall
10/23/2020	BL	2M	10	2020	Year-3		1	Fall
10/23/2020	BL	3M	10	2020	Year-3	0	0	Fall
10/23/2020	BL	4M	10	2020	Year-3		1	Fall
10/23/2020	BL	5M	10	2020	Year-3		1	Fall
10/23/2020	BL	6M	10	2020	Year-3		1	Fall
10/23/2020	BL	7M	10	2020	Year-3		1	Fall
10/23/2020	BL	10M	10	2020	Year-3	0	0	Fall
10/23/2020	BL	12M	10	2020	Year-3	0	0	Fall
10/23/2020	BL	14M	10	2020	Year-3	0	0	Fall
10/23/2020	BL	8A	10	2020	Year-3		1	Fall
10/23/2020	BL	9A	10	2020	Year-3		1	Fall
10/23/2020	BL	11A	10	2020	Year-3		1	Fall
10/23/2020	BL	13A	10	2020	Year-3		1	Fall
10/24/2020	BL	1M	10	2020	Year-3	0	0	Fall
10/24/2020	BL	2M	10	2020	Year-3		1	Fall
10/24/2020	BL	3M	10	2020	Year-3	0	0	Fall
10/24/2020	BL	4M	10	2020	Year-3		1	Fall
10/24/2020	BL	5M	10	2020	Year-3		1	Fall
10/24/2020	BL	6M	10	2020	Year-3		1	Fall
10/24/2020	BL	7M	10	2020	Year-3		1	Fall
10/24/2020	BL	10M	10	2020	Year-3	0	0	Fall
10/24/2020	BL	12M	10	2020	Year-3	0	0	Fall
10/24/2020	BL	14M	10	2020	Year-3	0	0	Fall
10/24/2020	BL	8A	10	2020	Year-3		1	Fall
10/24/2020	BL	9A	10	2020	Year-3		1	Fall
10/24/2020	BL	11A	10	2020	Year-3		1	Fall
10/24/2020	BL	13A	10	2020	Year-3		1	Fall
10/25/2020	BL	1M	10	2020	Year-3	0	0	Fall
10/25/2020	BL	2M	10	2020	Year-3		1	Fall
10/25/2020	BL	3M	10	2020	Year-3	0	0	Fall
10/25/2020	BL	4M	10	2020	Year-3		1	Fall
10/25/2020	BL	5M	10	2020	Year-3		1	Fall
10/25/2020	BL	6M	10	2020	Year-3		1	Fall
10/25/2020	BL	7M	10	2020	Year-3		1	Fall
10/25/2020	BL	10M	10	2020	Year-3	0	0	Fall
10/25/2020	BL	12M	10	2020	Year-3	0	0	Fall
10/25/2020	BL	14M	10	2020	Year-3	0	0	Fall
10/25/2020	BL	8A	10	2020	Year-3		1	Fall

Date	Species	Site	Month	Year	Survey Year	Presence	Data Gap	Season
10/25/2020	BL	9A	10	2020	Year-3		1	Fall
10/25/2020	BL	11A	10	2020	Year-3		1	Fall
10/25/2020	BL	13A	10	2020	Year-3		1	Fall
10/26/2020	BL	1M	10	2020	Year-3	0	0	Fall
10/26/2020	BL	2M	10	2020	Year-3		1	Fall
10/26/2020	BL	3M	10	2020	Year-3	0	0	Fall
10/26/2020	BL	4M	10	2020	Year-3		1	Fall
10/26/2020	BL	5M	10	2020	Year-3		1	Fall
10/26/2020	BL	6M	10	2020	Year-3		1	Fall
10/26/2020	BL	7M	10	2020	Year-3		1	Fall
10/26/2020	BL	10M	10	2020	Year-3	0	0	Fall
10/26/2020	BL	12M	10	2020	Year-3	0	0	Fall
10/26/2020	BL	14M	10	2020	Year-3	0	0	Fall
10/26/2020	BL	8A	10	2020	Year-3		1	Fall
10/26/2020	BL	9A	10	2020	Year-3		1	Fall
10/26/2020	BL	11A	10	2020	Year-3		1	Fall
10/26/2020	BL	13A	10	2020	Year-3		1	Fall
10/27/2020	BL	1M	10	2020	Year-3	0	0	Fall
10/27/2020	BL	2M	10	2020	Year-3		1	Fall
10/27/2020	BL	3M	10	2020	Year-3	0	0	Fall
10/27/2020	BL	4M	10	2020	Year-3		1	Fall
10/27/2020	BL	5M	10	2020	Year-3		1	Fall
10/27/2020	BL	6M	10	2020	Year-3		1	Fall
10/27/2020	BL	7M	10	2020	Year-3		1	Fall
10/27/2020	BL	10M	10	2020	Year-3	0	0	Fall
10/27/2020	BL	12M	10	2020	Year-3	0	0	Fall
10/27/2020	BL	14M	10	2020	Year-3	0	0	Fall
10/27/2020	BL	8A	10	2020	Year-3		1	Fall
10/27/2020	BL	9A	10	2020	Year-3		1	Fall
10/27/2020	BL	11A	10	2020	Year-3		1	Fall
10/27/2020	BL	13A	10	2020	Year-3		1	Fall
10/28/2020	BL	1M	10	2020	Year-3	0	0	Fall
10/28/2020	BL	2M	10	2020	Year-3		1	Fall
10/28/2020	BL	3M	10	2020	Year-3	0	0	Fall
10/28/2020	BL	4M	10	2020	Year-3		1	Fall
10/28/2020	BL	5M	10	2020	Year-3		1	Fall
10/28/2020	BL	6M	10	2020	Year-3		1	Fall
10/28/2020	BL	7M	10	2020	Year-3		1	Fall
10/28/2020	BL	10M	10	2020	Year-3	0	0	Fall
10/28/2020	BL	12M	10	2020	Year-3	0	0	Fall
10/28/2020	BL	14M	10	2020	Year-3	0	0	Fall
10/28/2020	BL	8A	10	2020	Year-3		1	Fall
10/28/2020	BL	9A	10	2020	Year-3		1	Fall
10/28/2020	BL	11A	10	2020	Year-3		1	Fall

Date	Species	Site	Month	Year	Survey Year	Presence	Data Gap	Season
10/28/2020	BL	13A	10	2020	Year-3		1	Fall
10/29/2020	BL	1M	10	2020	Year-3	0	0	Fall
10/29/2020	BL	2M	10	2020	Year-3		1	Fall
10/29/2020	BL	3M	10	2020	Year-3	0	0	Fall
10/29/2020	BL	4M	10	2020	Year-3		1	Fall
10/29/2020	BL	5M	10	2020	Year-3		1	Fall
10/29/2020	BL	6M	10	2020	Year-3		1	Fall
10/29/2020	BL	7M	10	2020	Year-3		1	Fall
10/29/2020	BL	10M	10	2020	Year-3	0	0	Fall
10/29/2020	BL	12M	10	2020	Year-3	0	0	Fall
10/29/2020	BL	14M	10	2020	Year-3	0	0	Fall
10/29/2020	BL	8A	10	2020	Year-3		1	Fall
10/29/2020	BL	9A	10	2020	Year-3		1	Fall
10/29/2020	BL	11A	10	2020	Year-3		1	Fall
10/29/2020	BL	13A	10	2020	Year-3		1	Fall
10/30/2020	BL	1M	10	2020	Year-3	0	0	Fall
10/30/2020	BL	2M	10	2020	Year-3		1	Fall
10/30/2020	BL	3M	10	2020	Year-3	0	0	Fall
10/30/2020	BL	4M	10	2020	Year-3		1	Fall
10/30/2020	BL	5M	10	2020	Year-3		1	Fall
10/30/2020	BL	6M	10	2020	Year-3		1	Fall
10/30/2020	BL	7M	10	2020	Year-3		1	Fall
10/30/2020	BL	10M	10	2020	Year-3	0	0	Fall
10/30/2020	BL	12M	10	2020	Year-3	0	0	Fall
10/30/2020	BL	14M	10	2020	Year-3	0	0	Fall
10/30/2020	BL	8A	10	2020	Year-3		1	Fall
10/30/2020	BL	9A	10	2020	Year-3		1	Fall
10/30/2020	BL	11A	10	2020	Year-3		1	Fall
10/30/2020	BL	13A	10	2020	Year-3		1	Fall
10/31/2020	BL	1M	10	2020	Year-3	0	0	Fall
10/31/2020	BL	2M	10	2020	Year-3		1	Fall
10/31/2020	BL	3M	10	2020	Year-3	0	0	Fall
10/31/2020	BL	4M	10	2020	Year-3		1	Fall
10/31/2020	BL	5M	10	2020	Year-3		1	Fall
10/31/2020	BL	6M	10	2020	Year-3		1	Fall
10/31/2020	BL	7M	10	2020	Year-3		1	Fall
10/31/2020	BL	10M	10	2020	Year-3	0	0	Fall
10/31/2020	BL	12M	10	2020	Year-3	0	0	Fall
10/31/2020	BL	14M	10	2020	Year-3	0	0	Fall
10/31/2020	BL	8A	10	2020	Year-3		1	Fall
10/31/2020	BL	9A	10	2020	Year-3		1	Fall
10/31/2020	BL	11A	10	2020	Year-3		1	Fall
10/31/2020	BL	13A	10	2020	Year-3		1	Fall
10/16/2017	FP	1M	10	2017	Year-1	1	0	Fall

Date	Species	Site	Month	Year	Survey Year	Presence	Data Gap	Season
10/16/2017	FP	2M	10	2017	Year-1	1	0	Fall
10/16/2017	FP	3M	10	2017	Year-1	1	0	Fall
10/16/2017	FP	4M	10	2017	Year-1	1	0	Fall
10/16/2017	FP	5M	10	2017	Year-1	0	0	Fall
10/16/2017	FP	6M	10	2017	Year-1	0	0	Fall
10/16/2017	FP	7M	10	2017	Year-1	1	0	Fall
10/16/2017	FP	10M	10	2017	Year-1	0	0	Fall
10/16/2017	FP	12M	10	2017	Year-1	1	0	Fall
10/16/2017	FP	14M	10	2017	Year-1		1	Fall
10/16/2017	FP	8A	10	2017	Year-1	1	0	Fall
10/16/2017	FP	9A	10	2017	Year-1	0	0	Fall
10/16/2017	FP	11A	10	2017	Year-1	1	0	Fall
10/16/2017	FP	13A	10	2017	Year-1	1	0	Fall
10/17/2017	FP	1M	10	2017	Year-1	1	0	Fall
10/17/2017	FP	2M	10	2017	Year-1	1	0	Fall
10/17/2017	FP	3M	10	2017	Year-1	1	0	Fall
10/17/2017	FP	4M	10	2017	Year-1	1	0	Fall
10/17/2017	FP	5M	10	2017	Year-1	1	0	Fall
10/17/2017	FP	6M	10	2017	Year-1	0	0	Fall
10/17/2017	FP	7M	10	2017	Year-1	0	0	Fall
10/17/2017	FP	10M	10	2017	Year-1	0	0	Fall
10/17/2017	FP	12M	10	2017	Year-1	1	0	Fall
10/17/2017	FP	14M	10	2017	Year-1		1	Fall
10/17/2017	FP	8A	10	2017	Year-1	0	0	Fall
10/17/2017	FP	9A	10	2017	Year-1	0	0	Fall
10/17/2017	FP	11A	10	2017	Year-1	1	0	Fall
10/17/2017	FP	13A	10	2017	Year-1	1	0	Fall
10/18/2017	FP	1M	10	2017	Year-1	1	0	Fall
10/18/2017	FP	2M	10	2017	Year-1	1	0	Fall
10/18/2017	FP	3M	10	2017	Year-1	1	0	Fall
10/18/2017	FP	4M	10	2017	Year-1	1	0	Fall
10/18/2017	FP	5M	10	2017	Year-1	0	0	Fall
10/18/2017	FP	6M	10	2017	Year-1	0	0	Fall
10/18/2017	FP	7M	10	2017	Year-1	0	0	Fall
10/18/2017	FP	10M	10	2017	Year-1	1	0	Fall
10/18/2017	FP	12M	10	2017	Year-1	1	0	Fall
10/18/2017	FP	14M	10	2017	Year-1		1	Fall
10/18/2017	FP	8A	10	2017	Year-1	0	0	Fall
10/18/2017	FP	9A	10	2017	Year-1	0	0	Fall
10/18/2017	FP	11A	10	2017	Year-1	1	0	Fall
10/18/2017	FP	13A	10	2017	Year-1	1	0	Fall
10/19/2017	FP	1M	10	2017	Year-1	1	0	Fall
10/19/2017	FP	2M	10	2017	Year-1	1	0	Fall
10/19/2017	FP	3M	10	2017	Year-1	1	0	Fall

Date	Species	Site	Month	Year	Survey Year	Presence	Data Gap	Season
10/19/2017	FP	4M	10	2017	Year-1	1	0	Fall
10/19/2017	FP	5M	10	2017	Year-1	0	0	Fall
10/19/2017	FP	6M	10	2017	Year-1	0	0	Fall
10/19/2017	FP	7M	10	2017	Year-1	0	0	Fall
10/19/2017	FP	10M	10	2017	Year-1	1	0	Fall
10/19/2017	FP	12M	10	2017	Year-1	1	0	Fall
10/19/2017	FP	14M	10	2017	Year-1		1	Fall
10/19/2017	FP	8A	10	2017	Year-1	0	0	Fall
10/19/2017	FP	9A	10	2017	Year-1	0	0	Fall
10/19/2017	FP	11A	10	2017	Year-1	1	0	Fall
10/19/2017	FP	13A	10	2017	Year-1	1	0	Fall
10/20/2017	FP	1M	10	2017	Year-1	1	0	Fall
10/20/2017	FP	2M	10	2017	Year-1	1	0	Fall
10/20/2017	FP	3M	10	2017	Year-1	1	0	Fall
10/20/2017	FP	4M	10	2017	Year-1	1	0	Fall
10/20/2017	FP	5M	10	2017	Year-1	1	0	Fall
10/20/2017	FP	6M	10	2017	Year-1	0	0	Fall
10/20/2017	FP	7M	10	2017	Year-1	0	0	Fall
10/20/2017	FP	10M	10	2017	Year-1	1	0	Fall
10/20/2017	FP	12M	10	2017	Year-1	1	0	Fall
10/20/2017	FP	14M	10	2017	Year-1		1	Fall
10/20/2017	FP	8A	10	2017	Year-1	0	0	Fall
10/20/2017	FP	9A	10	2017	Year-1	0	0	Fall
10/20/2017	FP	11A	10	2017	Year-1	1	0	Fall
10/20/2017	FP	13A	10	2017	Year-1	1	0	Fall
10/21/2017	FP	1M	10	2017	Year-1	1	0	Fall
10/21/2017	FP	2M	10	2017	Year-1	1	0	Fall
10/21/2017	FP	3M	10	2017	Year-1	1	0	Fall
10/21/2017	FP	4M	10	2017	Year-1	1	0	Fall
10/21/2017	FP	5M	10	2017	Year-1	1	0	Fall
10/21/2017	FP	6M	10	2017	Year-1	0	0	Fall
10/21/2017	FP	7M	10	2017	Year-1	0	0	Fall
10/21/2017	FP	10M	10	2017	Year-1	1	0	Fall
10/21/2017	FP	12M	10	2017	Year-1	1	0	Fall
10/21/2017	FP	14M	10	2017	Year-1		1	Fall
10/21/2017	FP	8A	10	2017	Year-1	0	0	Fall
10/21/2017	FP	9A	10	2017	Year-1	1	0	Fall
10/21/2017	FP	11A	10	2017	Year-1	1	0	Fall
10/21/2017	FP	13A	10	2017	Year-1	1	0	Fall
10/22/2017	FP	1M	10	2017	Year-1	1	0	Fall
10/22/2017	FP	2M	10	2017	Year-1	1	0	Fall
10/22/2017	FP	3M	10	2017	Year-1	1	0	Fall
10/22/2017	FP	4M	10	2017	Year-1	1	0	Fall
10/22/2017	FP	5M	10	2017	Year-1	1	0	Fall

Date	Species	Site	Month	Year	Survey Year	Presence	Data Gap	Season
10/22/2017	FP	6M	10	2017	Year-1	0	0	Fall
10/22/2017	FP	7M	10	2017	Year-1	0	0	Fall
10/22/2017	FP	10M	10	2017	Year-1	1	0	Fall
10/22/2017	FP	12M	10	2017	Year-1	1	0	Fall
10/22/2017	FP	14M	10	2017	Year-1		1	Fall
10/22/2017	FP	8A	10	2017	Year-1	0	0	Fall
10/22/2017	FP	9A	10	2017	Year-1	1	0	Fall
10/22/2017	FP	11A	10	2017	Year-1	1	0	Fall
10/22/2017	FP	13A	10	2017	Year-1	1	0	Fall
10/23/2017	FP	1M	10	2017	Year-1	1	0	Fall
10/23/2017	FP	2M	10	2017	Year-1	1	0	Fall
10/23/2017	FP	3M	10	2017	Year-1	1	0	Fall
10/23/2017	FP	4M	10	2017	Year-1	1	0	Fall
10/23/2017	FP	5M	10	2017	Year-1	0	0	Fall
10/23/2017	FP	6M	10	2017	Year-1	0	0	Fall
10/23/2017	FP	7M	10	2017	Year-1	0	0	Fall
10/23/2017	FP	10M	10	2017	Year-1	1	0	Fall
10/23/2017	FP	12M	10	2017	Year-1	1	0	Fall
10/23/2017	FP	14M	10	2017	Year-1		1	Fall
10/23/2017	FP	8A	10	2017	Year-1	0	0	Fall
10/23/2017	FP	9A	10	2017	Year-1	1	0	Fall
10/23/2017	FP	11A	10	2017	Year-1	1	0	Fall
10/23/2017	FP	13A	10	2017	Year-1	1	0	Fall
10/24/2017	FP	1M	10	2017	Year-1	1	0	Fall
10/24/2017	FP	2M	10	2017	Year-1	1	0	Fall
10/24/2017	FP	3M	10	2017	Year-1	1	0	Fall
10/24/2017	FP	4M	10	2017	Year-1	1	0	Fall
10/24/2017	FP	5M	10	2017	Year-1	0	0	Fall
10/24/2017	FP	6M	10	2017	Year-1	0	0	Fall
10/24/2017	FP	7M	10	2017	Year-1	0	0	Fall
10/24/2017	FP	10M	10	2017	Year-1	1	0	Fall
10/24/2017	FP	12M	10	2017	Year-1	1	0	Fall
10/24/2017	FP	14M	10	2017	Year-1		1	Fall
10/24/2017	FP	8A	10	2017	Year-1	0	0	Fall
10/24/2017	FP	9A	10	2017	Year-1	0	0	Fall
10/24/2017	FP	11A	10	2017	Year-1	1	0	Fall
10/24/2017	FP	13A	10	2017	Year-1	1	0	Fall
10/25/2017	FP	1M	10	2017	Year-1	1	0	Fall
10/25/2017	FP	2M	10	2017	Year-1	1	0	Fall
10/25/2017	FP	3M	10	2017	Year-1	1	0	Fall
10/25/2017	FP	4M	10	2017	Year-1	1	0	Fall
10/25/2017	FP	5M	10	2017	Year-1	1	0	Fall
10/25/2017	FP	6M	10	2017	Year-1	0	0	Fall
10/25/2017	FP	7M	10	2017	Year-1	0	0	Fall

Date	Species	Site	Month	Year	Survey Year	Presence	Data Gap	Season
10/25/2017	FP	10M	10	2017	Year-1	1	0	Fall
10/25/2017	FP	12M	10	2017	Year-1	1	0	Fall
10/25/2017	FP	14M	10	2017	Year-1		1	Fall
10/25/2017	FP	8A	10	2017	Year-1	0	0	Fall
10/25/2017	FP	9A	10	2017	Year-1	0	0	Fall
10/25/2017	FP	11A	10	2017	Year-1	1	0	Fall
10/25/2017	FP	13A	10	2017	Year-1	1	0	Fall
10/26/2017	FP	1M	10	2017	Year-1	1	0	Fall
10/26/2017	FP	2M	10	2017	Year-1	1	0	Fall
10/26/2017	FP	3M	10	2017	Year-1	1	0	Fall
10/26/2017	FP	4M	10	2017	Year-1	1	0	Fall
10/26/2017	FP	5M	10	2017	Year-1	1	0	Fall
10/26/2017	FP	6M	10	2017	Year-1	0	0	Fall
10/26/2017	FP	7M	10	2017	Year-1	0	0	Fall
10/26/2017	FP	10M	10	2017	Year-1	1	0	Fall
10/26/2017	FP	12M	10	2017	Year-1	1	0	Fall
10/26/2017	FP	14M	10	2017	Year-1		1	Fall
10/26/2017	FP	8A	10	2017	Year-1	0	0	Fall
10/26/2017	FP	9A	10	2017	Year-1	0	0	Fall
10/26/2017	FP	11A	10	2017	Year-1	1	0	Fall
10/26/2017	FP	13A	10	2017	Year-1	1	0	Fall
10/27/2017	FP	1M	10	2017	Year-1	1	0	Fall
10/27/2017	FP	2M	10	2017	Year-1	1	0	Fall
10/27/2017	FP	3M	10	2017	Year-1	1	0	Fall
10/27/2017	FP	4M	10	2017	Year-1	1	0	Fall
10/27/2017	FP	5M	10	2017	Year-1	1	0	Fall
10/27/2017	FP	6M	10	2017	Year-1	0	0	Fall
10/27/2017	FP	7M	10	2017	Year-1	0	0	Fall
10/27/2017	FP	10M	10	2017	Year-1	1	0	Fall
10/27/2017	FP	12M	10	2017	Year-1	1	0	Fall
10/27/2017	FP	14M	10	2017	Year-1		1	Fall
10/27/2017	FP	8A	10	2017	Year-1	0	0	Fall
10/27/2017	FP	9A	10	2017	Year-1	0	0	Fall
10/27/2017	FP	11A	10	2017	Year-1	1	0	Fall
10/27/2017	FP	13A	10	2017	Year-1	1	0	Fall
10/28/2017	FP	1M	10	2017	Year-1	1	0	Fall
10/28/2017	FP	2M	10	2017	Year-1	1	0	Fall
10/28/2017	FP	3M	10	2017	Year-1	1	0	Fall
10/28/2017	FP	4M	10	2017	Year-1	1	0	Fall
10/28/2017	FP	5M	10	2017	Year-1	0	0	Fall
10/28/2017	FP	6M	10	2017	Year-1	0	0	Fall
10/28/2017	FP	7M	10	2017	Year-1	0	0	Fall
10/28/2017	FP	10M	10	2017	Year-1	1	0	Fall
10/28/2017	FP	12M	10	2017	Year-1	1	0	Fall

Date	Species	Site	Month	Year	Survey Year	Presence	Data Gap	Season
10/28/2017	FP	14M	10	2017	Year-1		1	Fall
10/28/2017	FP	8A	10	2017	Year-1	0	0	Fall
10/28/2017	FP	9A	10	2017	Year-1	1	0	Fall
10/28/2017	FP	11A	10	2017	Year-1	1	0	Fall
10/28/2017	FP	13A	10	2017	Year-1	1	0	Fall
10/29/2017	FP	1M	10	2017	Year-1	1	0	Fall
10/29/2017	FP	2M	10	2017	Year-1	1	0	Fall
10/29/2017	FP	3M	10	2017	Year-1	1	0	Fall
10/29/2017	FP	4M	10	2017	Year-1	1	0	Fall
10/29/2017	FP	5M	10	2017	Year-1	0	0	Fall
10/29/2017	FP	6M	10	2017	Year-1	0	0	Fall
10/29/2017	FP	7M	10	2017	Year-1	0	0	Fall
10/29/2017	FP	10M	10	2017	Year-1	1	0	Fall
10/29/2017	FP	12M	10	2017	Year-1	1	0	Fall
10/29/2017	FP	14M	10	2017	Year-1		1	Fall
10/29/2017	FP	8A	10	2017	Year-1	0	0	Fall
10/29/2017	FP	9A	10	2017	Year-1	0	0	Fall
10/29/2017	FP	11A	10	2017	Year-1	1	0	Fall
10/29/2017	FP	13A	10	2017	Year-1	1	0	Fall
10/30/2017	FP	1M	10	2017	Year-1	1	0	Fall
10/30/2017	FP	2M	10	2017	Year-1	1	0	Fall
10/30/2017	FP	3M	10	2017	Year-1	1	0	Fall
10/30/2017	FP	4M	10	2017	Year-1	1	0	Fall
10/30/2017	FP	5M	10	2017	Year-1	1	0	Fall
10/30/2017	FP	6M	10	2017	Year-1	0	0	Fall
10/30/2017	FP	7M	10	2017	Year-1	1	0	Fall
10/30/2017	FP	10M	10	2017	Year-1	1	0	Fall
10/30/2017	FP	12M	10	2017	Year-1	1	0	Fall
10/30/2017	FP	14M	10	2017	Year-1		1	Fall
10/30/2017	FP	8A	10	2017	Year-1	0	0	Fall
10/30/2017	FP	9A	10	2017	Year-1	0	0	Fall
10/30/2017	FP	11A	10	2017	Year-1	1	0	Fall
10/30/2017	FP	13A	10	2017	Year-1	1	0	Fall
10/31/2017	FP	1M	10	2017	Year-1	1	0	Fall
10/31/2017	FP	2M	10	2017	Year-1	1	0	Fall
10/31/2017	FP	3M	10	2017	Year-1	1	0	Fall
10/31/2017	FP	4M	10	2017	Year-1	1	0	Fall
10/31/2017	FP	5M	10	2017	Year-1	1	0	Fall
10/31/2017	FP	6M	10	2017	Year-1	0	0	Fall
10/31/2017	FP	7M	10	2017	Year-1	1	0	Fall
10/31/2017	FP	10M	10	2017	Year-1	1	0	Fall
10/31/2017	FP	12M	10	2017	Year-1	1	0	Fall
10/31/2017	FP	14M	10	2017	Year-1		1	Fall
10/31/2017	FP	8A	10	2017	Year-1	0	0	Fall

Date	Species	Site	Month	Year	Survey Year	Presence	Data Gap	Season
10/31/2017	FP	9A	10	2017	Year-1	1	0	Fall
10/31/2017	FP	11A	10	2017	Year-1	1	0	Fall
10/31/2017	FP	13A	10	2017	Year-1	1	0	Fall
11/1/2017	FP	1M	11	2017	Year-1	1	0	Fall
11/1/2017	FP	2M	11	2017	Year-1	1	0	Fall
11/1/2017	FP	3M	11	2017	Year-1	1	0	Fall
11/1/2017	FP	4M	11	2017	Year-1	1	0	Fall
11/1/2017	FP	5M	11	2017	Year-1	1	0	Fall
11/1/2017	FP	6M	11	2017	Year-1	1	0	Fall
11/1/2017	FP	7M	11	2017	Year-1	1	0	Fall
11/1/2017	FP	10M	11	2017	Year-1	1	0	Fall
11/1/2017	FP	12M	11	2017	Year-1	1	0	Fall
11/1/2017	FP	14M	11	2017	Year-1		1	Fall
11/1/2017	FP	8A	11	2017	Year-1	0	0	Fall
11/1/2017	FP	9A	11	2017	Year-1	1	0	Fall
11/1/2017	FP	11A	11	2017	Year-1	1	0	Fall
11/1/2017	FP	13A	11	2017	Year-1	1	0	Fall
11/2/2017	FP	1M	11	2017	Year-1	1	0	Fall
11/2/2017	FP	2M	11	2017	Year-1	1	0	Fall
11/2/2017	FP	3M	11	2017	Year-1	1	0	Fall
11/2/2017	FP	4M	11	2017	Year-1	1	0	Fall
11/2/2017	FP	5M	11	2017	Year-1	1	0	Fall
11/2/2017	FP	6M	11	2017	Year-1	0	0	Fall
11/2/2017	FP	7M	11	2017	Year-1	1	0	Fall
11/2/2017	FP	10M	11	2017	Year-1	1	0	Fall
11/2/2017	FP	12M	11	2017	Year-1	1	0	Fall
11/2/2017	FP	14M	11	2017	Year-1		1	Fall
11/2/2017	FP	8A	11	2017	Year-1	0	0	Fall
11/2/2017	FP	9A	11	2017	Year-1	1	0	Fall
11/2/2017	FP	11A	11	2017	Year-1	1	0	Fall
11/2/2017	FP	13A	11	2017	Year-1	1	0	Fall
11/3/2017	FP	1M	11	2017	Year-1	1	0	Fall
11/3/2017	FP	2M	11	2017	Year-1	1	0	Fall
11/3/2017	FP	3M	11	2017	Year-1	1	0	Fall
11/3/2017	FP	4M	11	2017	Year-1	1	0	Fall
11/3/2017	FP	5M	11	2017	Year-1	0	0	Fall
11/3/2017	FP	6M	11	2017	Year-1	0	0	Fall
11/3/2017	FP	7M	11	2017	Year-1	1	0	Fall
11/3/2017	FP	10M	11	2017	Year-1	1	0	Fall
11/3/2017	FP	12M	11	2017	Year-1	1	0	Fall
11/3/2017	FP	14M	11	2017	Year-1		1	Fall
11/3/2017	FP	8A	11	2017	Year-1	1	0	Fall
11/3/2017	FP	9A	11	2017	Year-1	1	0	Fall
11/3/2017	FP	11A	11	2017	Year-1	1	0	Fall

Date	Species	Site	Month	Year	Survey Year	Presence	Data Gap	Season
11/3/2017	FP	13A	11	2017	Year-1	1	0	Fall
11/4/2017	FP	1M	11	2017	Year-1	1	0	Fall
11/4/2017	FP	2M	11	2017	Year-1	1	0	Fall
11/4/2017	FP	3M	11	2017	Year-1	1	0	Fall
11/4/2017	FP	4M	11	2017	Year-1	1	0	Fall
11/4/2017	FP	5M	11	2017	Year-1	0	0	Fall
11/4/2017	FP	6M	11	2017	Year-1	0	0	Fall
11/4/2017	FP	7M	11	2017	Year-1	0	0	Fall
11/4/2017	FP	10M	11	2017	Year-1	1	0	Fall
11/4/2017	FP	12M	11	2017	Year-1	1	0	Fall
11/4/2017	FP	14M	11	2017	Year-1		1	Fall
11/4/2017	FP	8A	11	2017	Year-1	0	0	Fall
11/4/2017	FP	9A	11	2017	Year-1	0	0	Fall
11/4/2017	FP	11A	11	2017	Year-1	1	0	Fall
11/4/2017	FP	13A	11	2017	Year-1	1	0	Fall
11/5/2017	FP	1M	11	2017	Year-1	1	0	Fall
11/5/2017	FP	2M	11	2017	Year-1	1	0	Fall
11/5/2017	FP	3M	11	2017	Year-1	1	0	Fall
11/5/2017	FP	4M	11	2017	Year-1	1	0	Fall
11/5/2017	FP	5M	11	2017	Year-1	1	0	Fall
11/5/2017	FP	6M	11	2017	Year-1	0	0	Fall
11/5/2017	FP	7M	11	2017	Year-1	0	0	Fall
11/5/2017	FP	10M	11	2017	Year-1	1	0	Fall
11/5/2017	FP	12M	11	2017	Year-1	1	0	Fall
11/5/2017	FP	14M	11	2017	Year-1		1	Fall
11/5/2017	FP	8A	11	2017	Year-1	0	0	Fall
11/5/2017	FP	9A	11	2017	Year-1	0	0	Fall
11/5/2017	FP	11A	11	2017	Year-1	1	0	Fall
11/5/2017	FP	13A	11	2017	Year-1	1	0	Fall
11/6/2017	FP	1M	11	2017	Year-1	1	0	Fall
11/6/2017	FP	2M	11	2017	Year-1	1	0	Fall
11/6/2017	FP	3M	11	2017	Year-1	1	0	Fall
11/6/2017	FP	4M	11	2017	Year-1	1	0	Fall
11/6/2017	FP	5M	11	2017	Year-1	1	0	Fall
11/6/2017	FP	6M	11	2017	Year-1	0	0	Fall
11/6/2017	FP	7M	11	2017	Year-1	0	0	Fall
11/6/2017	FP	10M	11	2017	Year-1	1	0	Fall
11/6/2017	FP	12M	11	2017	Year-1	1	0	Fall
11/6/2017	FP	14M	11	2017	Year-1		1	Fall
11/6/2017	FP	8A	11	2017	Year-1	0	0	Fall
11/6/2017	FP	9A	11	2017	Year-1	1	0	Fall
11/6/2017	FP	11A	11	2017	Year-1	1	0	Fall
11/6/2017	FP	13A	11	2017	Year-1	1	0	Fall
11/7/2017	FP	1M	11	2017	Year-1	1	0	Fall

Date	Species	Site	Month	Year	Survey Year	Presence	Data Gap	Season
11/7/2017	FP	2M	11	2017	Year-1	1	0	Fall
11/7/2017	FP	3M	11	2017	Year-1	1	0	Fall
11/7/2017	FP	4M	11	2017	Year-1	1	0	Fall
11/7/2017	FP	5M	11	2017	Year-1	0	0	Fall
11/7/2017	FP	6M	11	2017	Year-1	0	0	Fall
11/7/2017	FP	7M	11	2017	Year-1	0	0	Fall
11/7/2017	FP	10M	11	2017	Year-1	1	0	Fall
11/7/2017	FP	12M	11	2017	Year-1	1	0	Fall
11/7/2017	FP	14M	11	2017	Year-1		1	Fall
11/7/2017	FP	8A	11	2017	Year-1	0	0	Fall
11/7/2017	FP	9A	11	2017	Year-1	0	0	Fall
11/7/2017	FP	11A	11	2017	Year-1	1	0	Fall
11/7/2017	FP	13A	11	2017	Year-1	1	0	Fall
11/8/2017	FP	1M	11	2017	Year-1	1	0	Fall
11/8/2017	FP	2M	11	2017	Year-1	1	0	Fall
11/8/2017	FP	3M	11	2017	Year-1	1	0	Fall
11/8/2017	FP	4M	11	2017	Year-1	1	0	Fall
11/8/2017	FP	5M	11	2017	Year-1	0	0	Fall
11/8/2017	FP	6M	11	2017	Year-1	0	0	Fall
11/8/2017	FP	7M	11	2017	Year-1	0	0	Fall
11/8/2017	FP	10M	11	2017	Year-1	1	0	Fall
11/8/2017	FP	12M	11	2017	Year-1	1	0	Fall
11/8/2017	FP	14M	11	2017	Year-1		1	Fall
11/8/2017	FP	8A	11	2017	Year-1	0	0	Fall
11/8/2017	FP	9A	11	2017	Year-1	0	0	Fall
11/8/2017	FP	11A	11	2017	Year-1	1	0	Fall
11/8/2017	FP	13A	11	2017	Year-1	1	0	Fall
11/9/2017	FP	1M	11	2017	Year-1	1	0	Fall
11/9/2017	FP	2M	11	2017	Year-1	1	0	Fall
11/9/2017	FP	3M	11	2017	Year-1	1	0	Fall
11/9/2017	FP	4M	11	2017	Year-1	1	0	Fall
11/9/2017	FP	5M	11	2017	Year-1	1	0	Fall
11/9/2017	FP	6M	11	2017	Year-1	0	0	Fall
11/9/2017	FP	7M	11	2017	Year-1	0	0	Fall
11/9/2017	FP	10M	11	2017	Year-1	1	0	Fall
11/9/2017	FP	12M	11	2017	Year-1	1	0	Fall
11/9/2017	FP	14M	11	2017	Year-1		1	Fall
11/9/2017	FP	8A	11	2017	Year-1	0	0	Fall
11/9/2017	FP	9A	11	2017	Year-1	0	0	Fall
11/9/2017	FP	11A	11	2017	Year-1	1	0	Fall
11/9/2017	FP	13A	11	2017	Year-1	1	0	Fall
11/10/2017	FP	1M	11	2017	Year-1	0	0	Fall
11/10/2017	FP	2M	11	2017	Year-1	0	0	Fall
11/10/2017	FP	3M	11	2017	Year-1	0	0	Fall

Date	Species	Site	Month	Year	Survey Year	Presence	Data Gap	Season
11/10/2017	FP	4M	11	2017	Year-1	0	0	Fall
11/10/2017	FP	5M	11	2017	Year-1	0	0	Fall
11/10/2017	FP	6M	11	2017	Year-1	0	0	Fall
11/10/2017	FP	7M	11	2017	Year-1	0	0	Fall
11/10/2017	FP	10M	11	2017	Year-1	0	0	Fall
11/10/2017	FP	12M	11	2017	Year-1	0	0	Fall
11/10/2017	FP	14M	11	2017	Year-1		1	Fall
11/10/2017	FP	8A	11	2017	Year-1	0	0	Fall
11/10/2017	FP	9A	11	2017	Year-1	0	0	Fall
11/10/2017	FP	11A	11	2017	Year-1	1	0	Fall
11/10/2017	FP	13A	11	2017	Year-1	1	0	Fall
11/11/2017	FP	1M	11	2017	Year-1	0	0	Fall
11/11/2017	FP	2M	11	2017	Year-1	0	0	Fall
11/11/2017	FP	3M	11	2017	Year-1	0	0	Fall
11/11/2017	FP	4M	11	2017	Year-1	0	0	Fall
11/11/2017	FP	5M	11	2017	Year-1	0	0	Fall
11/11/2017	FP	6M	11	2017	Year-1	0	0	Fall
11/11/2017	FP	7M	11	2017	Year-1	0	0	Fall
11/11/2017	FP	10M	11	2017	Year-1	0	0	Fall
11/11/2017	FP	12M	11	2017	Year-1	0	0	Fall
11/11/2017	FP	14M	11	2017	Year-1		1	Fall
11/11/2017	FP	8A	11	2017	Year-1	1	0	Fall
11/11/2017	FP	9A	11	2017	Year-1	0	0	Fall
11/11/2017	FP	11A	11	2017	Year-1	1	0	Fall
11/11/2017	FP	13A	11	2017	Year-1	1	0	Fall
11/12/2017	FP	1M	11	2017	Year-1	0	0	Fall
11/12/2017	FP	2M	11	2017	Year-1	0	0	Fall
11/12/2017	FP	3M	11	2017	Year-1	0	0	Fall
11/12/2017	FP	4M	11	2017	Year-1	0	0	Fall
11/12/2017	FP	5M	11	2017	Year-1	0	0	Fall
11/12/2017	FP	6M	11	2017	Year-1	0	0	Fall
11/12/2017	FP	7M	11	2017	Year-1	0	0	Fall
11/12/2017	FP	10M	11	2017	Year-1	0	0	Fall
11/12/2017	FP	12M	11	2017	Year-1	0	0	Fall
11/12/2017	FP	14M	11	2017	Year-1		1	Fall
11/12/2017	FP	8A	11	2017	Year-1	0	0	Fall
11/12/2017	FP	9A	11	2017	Year-1	0	0	Fall
11/12/2017	FP	11A	11	2017	Year-1	1	0	Fall
11/12/2017	FP	13A	11	2017	Year-1	1	0	Fall
11/13/2017	FP	1M	11	2017	Year-1	0	0	Fall
11/13/2017	FP	2M	11	2017	Year-1	0	0	Fall
11/13/2017	FP	3M	11	2017	Year-1	0	0	Fall
11/13/2017	FP	4M	11	2017	Year-1	0	0	Fall
11/13/2017	FP	5M	11	2017	Year-1	0	0	Fall

Date	Species	Site	Month	Year	Survey Year	Presence	Data Gap	Season
11/13/2017	FP	6M	11	2017	Year-1	0	0	Fall
11/13/2017	FP	7M	11	2017	Year-1	0	0	Fall
11/13/2017	FP	10M	11	2017	Year-1	0	0	Fall
11/13/2017	FP	12M	11	2017	Year-1	0	0	Fall
11/13/2017	FP	14M	11	2017	Year-1		1	Fall
11/13/2017	FP	8A	11	2017	Year-1	0	0	Fall
11/13/2017	FP	9A	11	2017	Year-1	0	0	Fall
11/13/2017	FP	11A	11	2017	Year-1	1	0	Fall
11/13/2017	FP	13A	11	2017	Year-1	1	0	Fall
11/14/2017	FP	1M	11	2017	Year-1	0	0	Fall
11/14/2017	FP	2M	11	2017	Year-1	0	0	Fall
11/14/2017	FP	3M	11	2017	Year-1	0	0	Fall
11/14/2017	FP	4M	11	2017	Year-1	0	0	Fall
11/14/2017	FP	5M	11	2017	Year-1	0	0	Fall
11/14/2017	FP	6M	11	2017	Year-1	0	0	Fall
11/14/2017	FP	7M	11	2017	Year-1	0	0	Fall
11/14/2017	FP	10M	11	2017	Year-1	0	0	Fall
11/14/2017	FP	12M	11	2017	Year-1	0	0	Fall
11/14/2017	FP	14M	11	2017	Year-1		1	Fall
11/14/2017	FP	8A	11	2017	Year-1	0	0	Fall
11/14/2017	FP	9A	11	2017	Year-1	0	0	Fall
11/14/2017	FP	11A	11	2017	Year-1	1	0	Fall
11/14/2017	FP	13A	11	2017	Year-1	1	0	Fall
11/15/2017	FP	1M	11	2017	Year-1	0	0	Fall
11/15/2017	FP	2M	11	2017	Year-1	0	0	Fall
11/15/2017	FP	3M	11	2017	Year-1	0	0	Fall
11/15/2017	FP	4M	11	2017	Year-1	0	0	Fall
11/15/2017	FP	5M	11	2017	Year-1	0	0	Fall
11/15/2017	FP	6M	11	2017	Year-1	0	0	Fall
11/15/2017	FP	7M	11	2017	Year-1	0	0	Fall
11/15/2017	FP	10M	11	2017	Year-1	0	0	Fall
11/15/2017	FP	12M	11	2017	Year-1	0	0	Fall
11/15/2017	FP	14M	11	2017	Year-1		1	Fall
11/15/2017	FP	8A	11	2017	Year-1	0	0	Fall
11/15/2017	FP	9A	11	2017	Year-1	1	0	Fall
11/15/2017	FP	11A	11	2017	Year-1	1	0	Fall
11/15/2017	FP	13A	11	2017	Year-1	1	0	Fall
11/16/2017	FP	1M	11	2017	Year-1	0	0	Fall
11/16/2017	FP	2M	11	2017	Year-1	0	0	Fall
11/16/2017	FP	3M	11	2017	Year-1	0	0	Fall
11/16/2017	FP	4M	11	2017	Year-1	0	0	Fall
11/16/2017	FP	5M	11	2017	Year-1	0	0	Fall
11/16/2017	FP	6M	11	2017	Year-1	0	0	Fall
11/16/2017	FP	7M	11	2017	Year-1	0	0	Fall

Date	Species	Site	Month	Year	Survey Year	Presence	Data Gap	Season
11/16/2017	FP	10M	11	2017	Year-1	0	0	Fall
11/16/2017	FP	12M	11	2017	Year-1	0	0	Fall
11/16/2017	FP	14M	11	2017	Year-1		1	Fall
11/16/2017	FP	8A	11	2017	Year-1	0	0	Fall
11/16/2017	FP	9A	11	2017	Year-1	1	0	Fall
11/16/2017	FP	11A	11	2017	Year-1	1	0	Fall
11/16/2017	FP	13A	11	2017	Year-1	1	0	Fall
11/17/2017	FP	1M	11	2017	Year-1	0	0	Fall
11/17/2017	FP	2M	11	2017	Year-1	0	0	Fall
11/17/2017	FP	3M	11	2017	Year-1	0	0	Fall
11/17/2017	FP	4M	11	2017	Year-1	0	0	Fall
11/17/2017	FP	5M	11	2017	Year-1	0	0	Fall
11/17/2017	FP	6M	11	2017	Year-1	0	0	Fall
11/17/2017	FP	7M	11	2017	Year-1	0	0	Fall
11/17/2017	FP	10M	11	2017	Year-1	0	0	Fall
11/17/2017	FP	12M	11	2017	Year-1	0	0	Fall
11/17/2017	FP	14M	11	2017	Year-1		1	Fall
11/17/2017	FP	8A	11	2017	Year-1	0	0	Fall
11/17/2017	FP	9A	11	2017	Year-1	1	0	Fall
11/17/2017	FP	11A	11	2017	Year-1	1	0	Fall
11/17/2017	FP	13A	11	2017	Year-1	1	0	Fall
11/18/2017	FP	1M	11	2017	Year-1	0	0	Fall
11/18/2017	FP	2M	11	2017	Year-1	0	0	Fall
11/18/2017	FP	3M	11	2017	Year-1	0	0	Fall
11/18/2017	FP	4M	11	2017	Year-1	0	0	Fall
11/18/2017	FP	5M	11	2017	Year-1	0	0	Fall
11/18/2017	FP	6M	11	2017	Year-1	0	0	Fall
11/18/2017	FP	7M	11	2017	Year-1	0	0	Fall
11/18/2017	FP	10M	11	2017	Year-1	0	0	Fall
11/18/2017	FP	12M	11	2017	Year-1	0	0	Fall
11/18/2017	FP	14M	11	2017	Year-1		1	Fall
11/18/2017	FP	8A	11	2017	Year-1	1	0	Fall
11/18/2017	FP	9A	11	2017	Year-1	1	0	Fall
11/18/2017	FP	11A	11	2017	Year-1	1	0	Fall
11/18/2017	FP	13A	11	2017	Year-1	1	0	Fall
11/19/2017	FP	1M	11	2017	Year-1	1	0	Fall
11/19/2017	FP	2M	11	2017	Year-1	1	0	Fall
11/19/2017	FP	3M	11	2017	Year-1	1	0	Fall
11/19/2017	FP	4M	11	2017	Year-1	1	0	Fall
11/19/2017	FP	5M	11	2017	Year-1	0	0	Fall
11/19/2017	FP	6M	11	2017	Year-1	1	0	Fall
11/19/2017	FP	7M	11	2017	Year-1	1	0	Fall
11/19/2017	FP	10M	11	2017	Year-1	1	0	Fall
11/19/2017	FP	12M	11	2017	Year-1	0	0	Fall

Date	Species	Site	Month	Year	Survey Year	Presence	Data Gap	Season
11/19/2017	FP	14M	11	2017	Year-1		1	Fall
11/19/2017	FP	8A	11	2017	Year-1	1	0	Fall
11/19/2017	FP	9A	11	2017	Year-1	1	0	Fall
11/19/2017	FP	11A	11	2017	Year-1	1	0	Fall
11/19/2017	FP	13A	11	2017	Year-1	1	0	Fall
11/20/2017	FP	1M	11	2017	Year-1	1	0	Fall
11/20/2017	FP	2M	11	2017	Year-1	1	0	Fall
11/20/2017	FP	3M	11	2017	Year-1	1	0	Fall
11/20/2017	FP	4M	11	2017	Year-1	1	0	Fall
11/20/2017	FP	5M	11	2017	Year-1	0	0	Fall
11/20/2017	FP	6M	11	2017	Year-1	0	0	Fall
11/20/2017	FP	7M	11	2017	Year-1	0	0	Fall
11/20/2017	FP	10M	11	2017	Year-1	1	0	Fall
11/20/2017	FP	12M	11	2017	Year-1	1	0	Fall
11/20/2017	FP	14M	11	2017	Year-1		1	Fall
11/20/2017	FP	8A	11	2017	Year-1	0	0	Fall
11/20/2017	FP	9A	11	2017	Year-1	1	0	Fall
11/20/2017	FP	11A	11	2017	Year-1	1	0	Fall
11/20/2017	FP	13A	11	2017	Year-1	1	0	Fall
11/21/2017	FP	1M	11	2017	Year-1	1	0	Fall
11/21/2017	FP	2M	11	2017	Year-1	1	0	Fall
11/21/2017	FP	3M	11	2017	Year-1	1	0	Fall
11/21/2017	FP	4M	11	2017	Year-1	1	0	Fall
11/21/2017	FP	5M	11	2017	Year-1	0	0	Fall
11/21/2017	FP	6M	11	2017	Year-1	0	0	Fall
11/21/2017	FP	7M	11	2017	Year-1	0	0	Fall
11/21/2017	FP	10M	11	2017	Year-1	1	0	Fall
11/21/2017	FP	12M	11	2017	Year-1	1	0	Fall
11/21/2017	FP	14M	11	2017	Year-1		1	Fall
11/21/2017	FP	8A	11	2017	Year-1	0	0	Fall
11/21/2017	FP	9A	11	2017	Year-1	1	0	Fall
11/21/2017	FP	11A	11	2017	Year-1	1	0	Fall
11/21/2017	FP	13A	11	2017	Year-1	1	0	Fall
11/22/2017	FP	1M	11	2017	Year-1	1	0	Fall
11/22/2017	FP	2M	11	2017	Year-1	1	0	Fall
11/22/2017	FP	3M	11	2017	Year-1	1	0	Fall
11/22/2017	FP	4M	11	2017	Year-1	1	0	Fall
11/22/2017	FP	5M	11	2017	Year-1	0	0	Fall
11/22/2017	FP	6M	11	2017	Year-1	0	0	Fall
11/22/2017	FP	7M	11	2017	Year-1	0	0	Fall
11/22/2017	FP	10M	11	2017	Year-1	1	0	Fall
11/22/2017	FP	12M	11	2017	Year-1	1	0	Fall
11/22/2017	FP	14M	11	2017	Year-1		1	Fall
11/22/2017	FP	8A	11	2017	Year-1	0	0	Fall

Date	Species	Site	Month	Year	Survey Year	Presence	Data Gap	Season
11/22/2017	FP	9A	11	2017	Year-1	1	0	Fall
11/22/2017	FP	11A	11	2017	Year-1	1	0	Fall
11/22/2017	FP	13A	11	2017	Year-1	1	0	Fall
11/23/2017	FP	1M	11	2017	Year-1	1	0	Fall
11/23/2017	FP	2M	11	2017	Year-1	1	0	Fall
11/23/2017	FP	3M	11	2017	Year-1	1	0	Fall
11/23/2017	FP	4M	11	2017	Year-1	1	0	Fall
11/23/2017	FP	5M	11	2017	Year-1	0	0	Fall
11/23/2017	FP	6M	11	2017	Year-1	0	0	Fall
11/23/2017	FP	7M	11	2017	Year-1	0	0	Fall
11/23/2017	FP	10M	11	2017	Year-1	1	0	Fall
11/23/2017	FP	12M	11	2017	Year-1	1	0	Fall
11/23/2017	FP	14M	11	2017	Year-1		1	Fall
11/23/2017	FP	8A	11	2017	Year-1	0	0	Fall
11/23/2017	FP	9A	11	2017	Year-1	1	0	Fall
11/23/2017	FP	11A	11	2017	Year-1	1	0	Fall
11/23/2017	FP	13A	11	2017	Year-1	1	0	Fall
11/24/2017	FP	1M	11	2017	Year-1	1	0	Fall
11/24/2017	FP	2M	11	2017	Year-1	1	0	Fall
11/24/2017	FP	3M	11	2017	Year-1	0	0	Fall
11/24/2017	FP	4M	11	2017	Year-1	1	0	Fall
11/24/2017	FP	5M	11	2017	Year-1	0	0	Fall
11/24/2017	FP	6M	11	2017	Year-1	0	0	Fall
11/24/2017	FP	7M	11	2017	Year-1	0	0	Fall
11/24/2017	FP	10M	11	2017	Year-1	1	0	Fall
11/24/2017	FP	12M	11	2017	Year-1	1	0	Fall
11/24/2017	FP	14M	11	2017	Year-1		1	Fall
11/24/2017	FP	8A	11	2017	Year-1	0	0	Fall
11/24/2017	FP	9A	11	2017	Year-1	1	0	Fall
11/24/2017	FP	11A	11	2017	Year-1	1	0	Fall
11/24/2017	FP	13A	11	2017	Year-1	1	0	Fall
11/25/2017	FP	1M	11	2017	Year-1	1	0	Fall
11/25/2017	FP	2M	11	2017	Year-1	1	0	Fall
11/25/2017	FP	3M	11	2017	Year-1	1	0	Fall
11/25/2017	FP	4M	11	2017	Year-1	1	0	Fall
11/25/2017	FP	5M	11	2017	Year-1	0	0	Fall
11/25/2017	FP	6M	11	2017	Year-1	0	0	Fall
11/25/2017	FP	7M	11	2017	Year-1	0	0	Fall
11/25/2017	FP	10M	11	2017	Year-1	1	0	Fall
11/25/2017	FP	12M	11	2017	Year-1	1	0	Fall
11/25/2017	FP	14M	11	2017	Year-1		1	Fall
11/25/2017	FP	8A	11	2017	Year-1	0	0	Fall
11/25/2017	FP	9A	11	2017	Year-1	1	0	Fall
11/25/2017	FP	11A	11	2017	Year-1	1	0	Fall

Date	Species	Site	Month	Year	Survey Year	Presence	Data Gap	Season
11/25/2017	FP	13A	11	2017	Year-1	1	0	Fall
11/26/2017	FP	1M	11	2017	Year-1	1	0	Fall
11/26/2017	FP	2M	11	2017	Year-1	1	0	Fall
11/26/2017	FP	3M	11	2017	Year-1	1	0	Fall
11/26/2017	FP	4M	11	2017	Year-1	0	0	Fall
11/26/2017	FP	5M	11	2017	Year-1	1	0	Fall
11/26/2017	FP	6M	11	2017	Year-1	0	0	Fall
11/26/2017	FP	7M	11	2017	Year-1	0	0	Fall
11/26/2017	FP	10M	11	2017	Year-1	1	0	Fall
11/26/2017	FP	12M	11	2017	Year-1	1	0	Fall
11/26/2017	FP	14M	11	2017	Year-1		1	Fall
11/26/2017	FP	8A	11	2017	Year-1	0	0	Fall
11/26/2017	FP	9A	11	2017	Year-1	1	0	Fall
11/26/2017	FP	11A	11	2017	Year-1	1	0	Fall
11/26/2017	FP	13A	11	2017	Year-1	0	0	Fall
11/27/2017	FP	1M	11	2017	Year-1	1	0	Fall
11/27/2017	FP	2M	11	2017	Year-1	1	0	Fall
11/27/2017	FP	3M	11	2017	Year-1	1	0	Fall
11/27/2017	FP	4M	11	2017	Year-1	1	0	Fall
11/27/2017	FP	5M	11	2017	Year-1	0	0	Fall
11/27/2017	FP	6M	11	2017	Year-1	0	0	Fall
11/27/2017	FP	7M	11	2017	Year-1	0	0	Fall
11/27/2017	FP	10M	11	2017	Year-1	1	0	Fall
11/27/2017	FP	12M	11	2017	Year-1	1	0	Fall
11/27/2017	FP	14M	11	2017	Year-1		1	Fall
11/27/2017	FP	8A	11	2017	Year-1	0	0	Fall
11/27/2017	FP	9A	11	2017	Year-1	1	0	Fall
11/27/2017	FP	11A	11	2017	Year-1	1	0	Fall
11/27/2017	FP	13A	11	2017	Year-1	1	0	Fall
11/28/2017	FP	1M	11	2017	Year-1	1	0	Fall
11/28/2017	FP	2M	11	2017	Year-1	1	0	Fall
11/28/2017	FP	3M	11	2017	Year-1	1	0	Fall
11/28/2017	FP	4M	11	2017	Year-1	1	0	Fall
11/28/2017	FP	5M	11	2017	Year-1	0	0	Fall
11/28/2017	FP	6M	11	2017	Year-1	0	0	Fall
11/28/2017	FP	7M	11	2017	Year-1	0	0	Fall
11/28/2017	FP	10M	11	2017	Year-1	1	0	Fall
11/28/2017	FP	12M	11	2017	Year-1	1	0	Fall
11/28/2017	FP	14M	11	2017	Year-1		1	Fall
11/28/2017	FP	8A	11	2017	Year-1	0	0	Fall
11/28/2017	FP	9A	11	2017	Year-1	1	0	Fall
11/28/2017	FP	11A	11	2017	Year-1	1	0	Fall
11/28/2017	FP	13A	11	2017	Year-1	1	0	Fall
11/29/2017	FP	1M	11	2017	Year-1	1	0	Fall

Date	Species	Site	Month	Year	Survey Year	Presence	Data Gap	Season
11/29/2017	FP	2M	11	2017	Year-1	1	0	Fall
11/29/2017	FP	3M	11	2017	Year-1	1	0	Fall
11/29/2017	FP	4M	11	2017	Year-1	1	0	Fall
11/29/2017	FP	5M	11	2017	Year-1	1	0	Fall
11/29/2017	FP	6M	11	2017	Year-1	0	0	Fall
11/29/2017	FP	7M	11	2017	Year-1	0	0	Fall
11/29/2017	FP	10M	11	2017	Year-1	1	0	Fall
11/29/2017	FP	12M	11	2017	Year-1	1	0	Fall
11/29/2017	FP	14M	11	2017	Year-1		1	Fall
11/29/2017	FP	8A	11	2017	Year-1	0	0	Fall
11/29/2017	FP	9A	11	2017	Year-1	1	0	Fall
11/29/2017	FP	11A	11	2017	Year-1	1	0	Fall
11/29/2017	FP	13A	11	2017	Year-1	1	0	Fall
11/30/2017	FP	1M	11	2017	Year-1	1	0	Fall
11/30/2017	FP	2M	11	2017	Year-1	1	0	Fall
11/30/2017	FP	3M	11	2017	Year-1	1	0	Fall
11/30/2017	FP	4M	11	2017	Year-1	1	0	Fall
11/30/2017	FP	5M	11	2017	Year-1	0	0	Fall
11/30/2017	FP	6M	11	2017	Year-1	0	0	Fall
11/30/2017	FP	7M	11	2017	Year-1	0	0	Fall
11/30/2017	FP	10M	11	2017	Year-1	1	0	Fall
11/30/2017	FP	12M	11	2017	Year-1	1	0	Fall
11/30/2017	FP	14M	11	2017	Year-1		1	Fall
11/30/2017	FP	8A	11	2017	Year-1	0	0	Fall
11/30/2017	FP	9A	11	2017	Year-1	1	0	Fall
11/30/2017	FP	11A	11	2017	Year-1	1	0	Fall
11/30/2017	FP	13A	11	2017	Year-1	1	0	Fall
12/1/2017	FP	1M	12	2017	Year-1	1	0	Winter
12/1/2017	FP	2M	12	2017	Year-1	1	0	Winter
12/1/2017	FP	3M	12	2017	Year-1	1	0	Winter
12/1/2017	FP	4M	12	2017	Year-1	1	0	Winter
12/1/2017	FP	5M	12	2017	Year-1	0	0	Winter
12/1/2017	FP	6M	12	2017	Year-1	0	0	Winter
12/1/2017	FP	7M	12	2017	Year-1	0	0	Winter
12/1/2017	FP	10M	12	2017	Year-1	1	0	Winter
12/1/2017	FP	12M	12	2017	Year-1	1	0	Winter
12/1/2017	FP	14M	12	2017	Year-1		1	Winter
12/1/2017	FP	8A	12	2017	Year-1	0	0	Winter
12/1/2017	FP	9A	12	2017	Year-1	1	0	Winter
12/1/2017	FP	11A	12	2017	Year-1	1	0	Winter
12/1/2017	FP	13A	12	2017	Year-1	1	0	Winter
12/2/2017	FP	1M	12	2017	Year-1	1	0	Winter
12/2/2017	FP	2M	12	2017	Year-1	1	0	Winter
12/2/2017	FP	3M	12	2017	Year-1	1	0	Winter

Date	Species	Site	Month	Year	Survey Year	Presence	Data Gap	Season
12/2/2017	FP	4M	12	2017	Year-1	1	0	Winter
12/2/2017	FP	5M	12	2017	Year-1	0	0	Winter
12/2/2017	FP	6M	12	2017	Year-1	0	0	Winter
12/2/2017	FP	7M	12	2017	Year-1	1	0	Winter
12/2/2017	FP	10M	12	2017	Year-1	1	0	Winter
12/2/2017	FP	12M	12	2017	Year-1	1	0	Winter
12/2/2017	FP	14M	12	2017	Year-1		1	Winter
12/2/2017	FP	8A	12	2017	Year-1	0	0	Winter
12/2/2017	FP	9A	12	2017	Year-1	1	0	Winter
12/2/2017	FP	11A	12	2017	Year-1	1	0	Winter
12/2/2017	FP	13A	12	2017	Year-1	1	0	Winter
12/3/2017	FP	1M	12	2017	Year-1	1	0	Winter
12/3/2017	FP	2M	12	2017	Year-1	1	0	Winter
12/3/2017	FP	3M	12	2017	Year-1	1	0	Winter
12/3/2017	FP	4M	12	2017	Year-1	1	0	Winter
12/3/2017	FP	5M	12	2017	Year-1	0	0	Winter
12/3/2017	FP	6M	12	2017	Year-1	0	0	Winter
12/3/2017	FP	7M	12	2017	Year-1	0	0	Winter
12/3/2017	FP	10M	12	2017	Year-1	1	0	Winter
12/3/2017	FP	12M	12	2017	Year-1	1	0	Winter
12/3/2017	FP	14M	12	2017	Year-1		1	Winter
12/3/2017	FP	8A	12	2017	Year-1	1	0	Winter
12/3/2017	FP	9A	12	2017	Year-1	1	0	Winter
12/3/2017	FP	11A	12	2017	Year-1	1	0	Winter
12/3/2017	FP	13A	12	2017	Year-1	1	0	Winter
12/4/2017	FP	1M	12	2017	Year-1	1	0	Winter
12/4/2017	FP	2M	12	2017	Year-1	0	0	Winter
12/4/2017	FP	3M	12	2017	Year-1	1	0	Winter
12/4/2017	FP	4M	12	2017	Year-1	0	0	Winter
12/4/2017	FP	5M	12	2017	Year-1	0	0	Winter
12/4/2017	FP	6M	12	2017	Year-1	0	0	Winter
12/4/2017	FP	7M	12	2017	Year-1	0	0	Winter
12/4/2017	FP	10M	12	2017	Year-1	1	0	Winter
12/4/2017	FP	12M	12	2017	Year-1	0	0	Winter
12/4/2017	FP	14M	12	2017	Year-1		1	Winter
12/4/2017	FP	8A	12	2017	Year-1	0	0	Winter
12/4/2017	FP	9A	12	2017	Year-1	1	0	Winter
12/4/2017	FP	11A	12	2017	Year-1	1	0	Winter
12/4/2017	FP	13A	12	2017	Year-1	1	0	Winter
12/5/2017	FP	1M	12	2017	Year-1	0	0	Winter
12/5/2017	FP	2M	12	2017	Year-1	1	0	Winter
12/5/2017	FP	3M	12	2017	Year-1	0	0	Winter
12/5/2017	FP	4M	12	2017	Year-1	1	0	Winter
12/5/2017	FP	5M	12	2017	Year-1	0	0	Winter

Date	Species	Site	Month	Year	Survey Year	Presence	Data Gap	Season
12/5/2017	FP	6M	12	2017	Year-1	0	0	Winter
12/5/2017	FP	7M	12	2017	Year-1	0	0	Winter
12/5/2017	FP	10M	12	2017	Year-1	0	0	Winter
12/5/2017	FP	12M	12	2017	Year-1	0	0	Winter
12/5/2017	FP	14M	12	2017	Year-1		1	Winter
12/5/2017	FP	8A	12	2017	Year-1	1	0	Winter
12/5/2017	FP	9A	12	2017	Year-1	1	0	Winter
12/5/2017	FP	11A	12	2017	Year-1	1	0	Winter
12/5/2017	FP	13A	12	2017	Year-1	1	0	Winter
12/6/2017	FP	1M	12	2017	Year-1	1	0	Winter
12/6/2017	FP	2M	12	2017	Year-1	1	0	Winter
12/6/2017	FP	3M	12	2017	Year-1	1	0	Winter
12/6/2017	FP	4M	12	2017	Year-1	1	0	Winter
12/6/2017	FP	5M	12	2017	Year-1	1	0	Winter
12/6/2017	FP	6M	12	2017	Year-1	0	0	Winter
12/6/2017	FP	7M	12	2017	Year-1	1	0	Winter
12/6/2017	FP	10M	12	2017	Year-1	1	0	Winter
12/6/2017	FP	12M	12	2017	Year-1	1	0	Winter
12/6/2017	FP	14M	12	2017	Year-1		1	Winter
12/6/2017	FP	8A	12	2017	Year-1	1	0	Winter
12/6/2017	FP	9A	12	2017	Year-1	1	0	Winter
12/6/2017	FP	11A	12	2017	Year-1	1	0	Winter
12/6/2017	FP	13A	12	2017	Year-1	1	0	Winter
12/7/2017	FP	1M	12	2017	Year-1	1	0	Winter
12/7/2017	FP	2M	12	2017	Year-1	1	0	Winter
12/7/2017	FP	3M	12	2017	Year-1	1	0	Winter
12/7/2017	FP	4M	12	2017	Year-1	1	0	Winter
12/7/2017	FP	5M	12	2017	Year-1	1	0	Winter
12/7/2017	FP	6M	12	2017	Year-1	0	0	Winter
12/7/2017	FP	7M	12	2017	Year-1	1	0	Winter
12/7/2017	FP	10M	12	2017	Year-1	1	0	Winter
12/7/2017	FP	12M	12	2017	Year-1	1	0	Winter
12/7/2017	FP	14M	12	2017	Year-1		1	Winter
12/7/2017	FP	8A	12	2017	Year-1	1	0	Winter
12/7/2017	FP	9A	12	2017	Year-1	1	0	Winter
12/7/2017	FP	11A	12	2017	Year-1	1	0	Winter
12/7/2017	FP	13A	12	2017	Year-1	1	0	Winter
12/8/2017	FP	1M	12	2017	Year-1	1	0	Winter
12/8/2017	FP	2M	12	2017	Year-1	1	0	Winter
12/8/2017	FP	3M	12	2017	Year-1	1	0	Winter
12/8/2017	FP	4M	12	2017	Year-1	1	0	Winter
12/8/2017	FP	5M	12	2017	Year-1	0	0	Winter
12/8/2017	FP	6M	12	2017	Year-1	0	0	Winter
12/8/2017	FP	7M	12	2017	Year-1	0	0	Winter

Date	Species	Site	Month	Year	Survey Year	Presence	Data Gap	Season
12/8/2017	FP	10M	12	2017	Year-1	1	0	Winter
12/8/2017	FP	12M	12	2017	Year-1	1	0	Winter
12/8/2017	FP	14M	12	2017	Year-1		1	Winter
12/8/2017	FP	8A	12	2017	Year-1	0	0	Winter
12/8/2017	FP	9A	12	2017	Year-1	1	0	Winter
12/8/2017	FP	11A	12	2017	Year-1	1	0	Winter
12/8/2017	FP	13A	12	2017	Year-1	1	0	Winter
12/9/2017	FP	1M	12	2017	Year-1	1	0	Winter
12/9/2017	FP	2M	12	2017	Year-1	1	0	Winter
12/9/2017	FP	3M	12	2017	Year-1	1	0	Winter
12/9/2017	FP	4M	12	2017	Year-1	1	0	Winter
12/9/2017	FP	5M	12	2017	Year-1	0	0	Winter
12/9/2017	FP	6M	12	2017	Year-1	0	0	Winter
12/9/2017	FP	7M	12	2017	Year-1	1	0	Winter
12/9/2017	FP	10M	12	2017	Year-1	1	0	Winter
12/9/2017	FP	12M	12	2017	Year-1	0	0	Winter
12/9/2017	FP	14M	12	2017	Year-1		1	Winter
12/9/2017	FP	8A	12	2017	Year-1	1	0	Winter
12/9/2017	FP	9A	12	2017	Year-1	1	0	Winter
12/9/2017	FP	11A	12	2017	Year-1	1	0	Winter
12/9/2017	FP	13A	12	2017	Year-1	1	0	Winter
12/10/2017	FP	1M	12	2017	Year-1	1	0	Winter
12/10/2017	FP	2M	12	2017	Year-1	1	0	Winter
12/10/2017	FP	3M	12	2017	Year-1	1	0	Winter
12/10/2017	FP	4M	12	2017	Year-1	1	0	Winter
12/10/2017	FP	5M	12	2017	Year-1	1	0	Winter
12/10/2017	FP	6M	12	2017	Year-1	0	0	Winter
12/10/2017	FP	7M	12	2017	Year-1	1	0	Winter
12/10/2017	FP	10M	12	2017	Year-1	1	0	Winter
12/10/2017	FP	12M	12	2017	Year-1	1	0	Winter
12/10/2017	FP	14M	12	2017	Year-1		1	Winter
12/10/2017	FP	8A	12	2017	Year-1	1	0	Winter
12/10/2017	FP	9A	12	2017	Year-1	1	0	Winter
12/10/2017	FP	11A	12	2017	Year-1	1	0	Winter
12/10/2017	FP	13A	12	2017	Year-1	1	0	Winter
12/11/2017	FP	1M	12	2017	Year-1	1	0	Winter
12/11/2017	FP	2M	12	2017	Year-1	1	0	Winter
12/11/2017	FP	3M	12	2017	Year-1	1	0	Winter
12/11/2017	FP	4M	12	2017	Year-1	1	0	Winter
12/11/2017	FP	5M	12	2017	Year-1	1	0	Winter
12/11/2017	FP	6M	12	2017	Year-1	0	0	Winter
12/11/2017	FP	7M	12	2017	Year-1	1	0	Winter
12/11/2017	FP	10M	12	2017	Year-1	1	0	Winter
12/11/2017	FP	12M	12	2017	Year-1	1	0	Winter

Date	Species	Site	Month	Year	Survey Year	Presence	Data Gap	Season
12/11/2017	FP	14M	12	2017	Year-1		1	Winter
12/11/2017	FP	8A	12	2017	Year-1	1	0	Winter
12/11/2017	FP	9A	12	2017	Year-1	1	0	Winter
12/11/2017	FP	11A	12	2017	Year-1	1	0	Winter
12/11/2017	FP	13A	12	2017	Year-1	1	0	Winter
12/12/2017	FP	1M	12	2017	Year-1	1	0	Winter
12/12/2017	FP	2M	12	2017	Year-1	1	0	Winter
12/12/2017	FP	3M	12	2017	Year-1	1	0	Winter
12/12/2017	FP	4M	12	2017	Year-1	1	0	Winter
12/12/2017	FP	5M	12	2017	Year-1	1	0	Winter
12/12/2017	FP	6M	12	2017	Year-1	0	0	Winter
12/12/2017	FP	7M	12	2017	Year-1	1	0	Winter
12/12/2017	FP	10M	12	2017	Year-1	1	0	Winter
12/12/2017	FP	12M	12	2017	Year-1	1	0	Winter
12/12/2017	FP	14M	12	2017	Year-1		1	Winter
12/12/2017	FP	8A	12	2017	Year-1	1	0	Winter
12/12/2017	FP	9A	12	2017	Year-1	1	0	Winter
12/12/2017	FP	11A	12	2017	Year-1	1	0	Winter
12/12/2017	FP	13A	12	2017	Year-1	1	0	Winter
12/13/2017	FP	1M	12	2017	Year-1	1	0	Winter
12/13/2017	FP	2M	12	2017	Year-1	1	0	Winter
12/13/2017	FP	3M	12	2017	Year-1	0	0	Winter
12/13/2017	FP	4M	12	2017	Year-1	1	0	Winter
12/13/2017	FP	5M	12	2017	Year-1	1	0	Winter
12/13/2017	FP	6M	12	2017	Year-1	0	0	Winter
12/13/2017	FP	7M	12	2017	Year-1	1	0	Winter
12/13/2017	FP	10M	12	2017	Year-1	1	0	Winter
12/13/2017	FP	12M	12	2017	Year-1	1	0	Winter
12/13/2017	FP	14M	12	2017	Year-1		1	Winter
12/13/2017	FP	8A	12	2017	Year-1	1	0	Winter
12/13/2017	FP	9A	12	2017	Year-1	0	0	Winter
12/13/2017	FP	11A	12	2017	Year-1	1	0	Winter
12/13/2017	FP	13A	12	2017	Year-1	1	0	Winter
12/14/2017	FP	1M	12	2017	Year-1	1	0	Winter
12/14/2017	FP	2M	12	2017	Year-1	1	0	Winter
12/14/2017	FP	3M	12	2017	Year-1	1	0	Winter
12/14/2017	FP	4M	12	2017	Year-1	1	0	Winter
12/14/2017	FP	5M	12	2017	Year-1	1	0	Winter
12/14/2017	FP	6M	12	2017	Year-1	1	0	Winter
12/14/2017	FP	7M	12	2017	Year-1	1	0	Winter
12/14/2017	FP	10M	12	2017	Year-1	1	0	Winter
12/14/2017	FP	12M	12	2017	Year-1	1	0	Winter
12/14/2017	FP	14M	12	2017	Year-1		1	Winter
12/14/2017	FP	8A	12	2017	Year-1	1	0	Winter

Date	Species	Site	Month	Year	Survey Year	Presence	Data Gap	Season
12/14/2017	FP	9A	12	2017	Year-1	1	0	Winter
12/14/2017	FP	11A	12	2017	Year-1	1	0	Winter
12/14/2017	FP	13A	12	2017	Year-1	0	0	Winter
12/15/2017	FP	1M	12	2017	Year-1	1	0	Winter
12/15/2017	FP	2M	12	2017	Year-1	1	0	Winter
12/15/2017	FP	3M	12	2017	Year-1	1	0	Winter
12/15/2017	FP	4M	12	2017	Year-1	1	0	Winter
12/15/2017	FP	5M	12	2017	Year-1	1	0	Winter
12/15/2017	FP	6M	12	2017	Year-1	0	0	Winter
12/15/2017	FP	7M	12	2017	Year-1	1	0	Winter
12/15/2017	FP	10M	12	2017	Year-1	1	0	Winter
12/15/2017	FP	12M	12	2017	Year-1	1	0	Winter
12/15/2017	FP	14M	12	2017	Year-1		1	Winter
12/15/2017	FP	8A	12	2017	Year-1	0	0	Winter
12/15/2017	FP	9A	12	2017	Year-1	1	0	Winter
12/15/2017	FP	11A	12	2017	Year-1	1	0	Winter
12/15/2017	FP	13A	12	2017	Year-1	1	0	Winter
12/16/2017	FP	1M	12	2017	Year-1	1	0	Winter
12/16/2017	FP	2M	12	2017	Year-1	1	0	Winter
12/16/2017	FP	3M	12	2017	Year-1	1	0	Winter
12/16/2017	FP	4M	12	2017	Year-1	1	0	Winter
12/16/2017	FP	5M	12	2017	Year-1	1	0	Winter
12/16/2017	FP	6M	12	2017	Year-1	1	0	Winter
12/16/2017	FP	7M	12	2017	Year-1	0	0	Winter
12/16/2017	FP	10M	12	2017	Year-1	0	0	Winter
12/16/2017	FP	12M	12	2017	Year-1	1	0	Winter
12/16/2017	FP	14M	12	2017	Year-1		1	Winter
12/16/2017	FP	8A	12	2017	Year-1	0	0	Winter
12/16/2017	FP	9A	12	2017	Year-1	1	0	Winter
12/16/2017	FP	11A	12	2017	Year-1	1	0	Winter
12/16/2017	FP	13A	12	2017	Year-1	1	0	Winter
12/17/2017	FP	1M	12	2017	Year-1	1	0	Winter
12/17/2017	FP	2M	12	2017	Year-1	1	0	Winter
12/17/2017	FP	3M	12	2017	Year-1	1	0	Winter
12/17/2017	FP	4M	12	2017	Year-1	1	0	Winter
12/17/2017	FP	5M	12	2017	Year-1	1	0	Winter
12/17/2017	FP	6M	12	2017	Year-1	1	0	Winter
12/17/2017	FP	7M	12	2017	Year-1	1	0	Winter
12/17/2017	FP	10M	12	2017	Year-1	1	0	Winter
12/17/2017	FP	12M	12	2017	Year-1	1	0	Winter
12/17/2017	FP	14M	12	2017	Year-1		1	Winter
12/17/2017	FP	8A	12	2017	Year-1	0	0	Winter
12/17/2017	FP	9A	12	2017	Year-1	1	0	Winter
12/17/2017	FP	11A	12	2017	Year-1	1	0	Winter

Date	Species	Site	Month	Year	Survey Year	Presence	Data Gap	Season
12/17/2017	FP	13A	12	2017	Year-1	1	0	Winter
12/18/2017	FP	1M	12	2017	Year-1	1	0	Winter
12/18/2017	FP	2M	12	2017	Year-1	1	0	Winter
12/18/2017	FP	3M	12	2017	Year-1	1	0	Winter
12/18/2017	FP	4M	12	2017	Year-1		1	Winter
12/18/2017	FP	5M	12	2017	Year-1	1	0	Winter
12/18/2017	FP	6M	12	2017	Year-1	0	0	Winter
12/18/2017	FP	7M	12	2017	Year-1	0	0	Winter
12/18/2017	FP	10M	12	2017	Year-1	1	0	Winter
12/18/2017	FP	12M	12	2017	Year-1	1	0	Winter
12/18/2017	FP	14M	12	2017	Year-1		1	Winter
12/18/2017	FP	8A	12	2017	Year-1	1	0	Winter
12/18/2017	FP	9A	12	2017	Year-1	1	0	Winter
12/18/2017	FP	11A	12	2017	Year-1	1	0	Winter
12/18/2017	FP	13A	12	2017	Year-1	1	0	Winter
12/19/2017	FP	1M	12	2017	Year-1	1	0	Winter
12/19/2017	FP	2M	12	2017	Year-1	1	0	Winter
12/19/2017	FP	3M	12	2017	Year-1	1	0	Winter
12/19/2017	FP	4M	12	2017	Year-1		1	Winter
12/19/2017	FP	5M	12	2017	Year-1	0	0	Winter
12/19/2017	FP	6M	12	2017	Year-1	0	0	Winter
12/19/2017	FP	7M	12	2017	Year-1	1	0	Winter
12/19/2017	FP	10M	12	2017	Year-1	1	0	Winter
12/19/2017	FP	12M	12	2017	Year-1	1	0	Winter
12/19/2017	FP	14M	12	2017	Year-1		1	Winter
12/19/2017	FP	8A	12	2017	Year-1	1	0	Winter
12/19/2017	FP	9A	12	2017	Year-1	1	0	Winter
12/19/2017	FP	11A	12	2017	Year-1	1	0	Winter
12/19/2017	FP	13A	12	2017	Year-1	1	0	Winter
12/20/2017	FP	1M	12	2017	Year-1	1	0	Winter
12/20/2017	FP	2M	12	2017	Year-1	1	0	Winter
12/20/2017	FP	3M	12	2017	Year-1	1	0	Winter
12/20/2017	FP	4M	12	2017	Year-1		1	Winter
12/20/2017	FP	5M	12	2017	Year-1	1	0	Winter
12/20/2017	FP	6M	12	2017	Year-1	0	0	Winter
12/20/2017	FP	7M	12	2017	Year-1	1	0	Winter
12/20/2017	FP	10M	12	2017	Year-1	1	0	Winter
12/20/2017	FP	12M	12	2017	Year-1	1	0	Winter
12/20/2017	FP	14M	12	2017	Year-1		1	Winter
12/20/2017	FP	8A	12	2017	Year-1	1	0	Winter
12/20/2017	FP	9A	12	2017	Year-1	1	0	Winter
12/20/2017	FP	11A	12	2017	Year-1	1	0	Winter
12/20/2017	FP	13A	12	2017	Year-1	1	0	Winter
12/21/2017	FP	1M	12	2017	Year-1	1	0	Winter

Date	Species	Site	Month	Year	Survey Year	Presence	Data Gap	Season
12/21/2017	FP	2M	12	2017	Year-1	1	0	Winter
12/21/2017	FP	3M	12	2017	Year-1	1	0	Winter
12/21/2017	FP	4M	12	2017	Year-1		1	Winter
12/21/2017	FP	5M	12	2017	Year-1	1	0	Winter
12/21/2017	FP	6M	12	2017	Year-1	0	0	Winter
12/21/2017	FP	7M	12	2017	Year-1	1	0	Winter
12/21/2017	FP	10M	12	2017	Year-1	1	0	Winter
12/21/2017	FP	12M	12	2017	Year-1	1	0	Winter
12/21/2017	FP	14M	12	2017	Year-1		1	Winter
12/21/2017	FP	8A	12	2017	Year-1	1	0	Winter
12/21/2017	FP	9A	12	2017	Year-1	1	0	Winter
12/21/2017	FP	11A	12	2017	Year-1	1	0	Winter
12/21/2017	FP	13A	12	2017	Year-1	1	0	Winter
12/22/2017	FP	1M	12	2017	Year-1	1	0	Winter
12/22/2017	FP	2M	12	2017	Year-1	1	0	Winter
12/22/2017	FP	3M	12	2017	Year-1	1	0	Winter
12/22/2017	FP	4M	12	2017	Year-1		1	Winter
12/22/2017	FP	5M	12	2017	Year-1	1	0	Winter
12/22/2017	FP	6M	12	2017	Year-1	0	0	Winter
12/22/2017	FP	7M	12	2017	Year-1	1	0	Winter
12/22/2017	FP	10M	12	2017	Year-1	1	0	Winter
12/22/2017	FP	12M	12	2017	Year-1	1	0	Winter
12/22/2017	FP	14M	12	2017	Year-1		1	Winter
12/22/2017	FP	8A	12	2017	Year-1	1	0	Winter
12/22/2017	FP	9A	12	2017	Year-1	1	0	Winter
12/22/2017	FP	11A	12	2017	Year-1	1	0	Winter
12/22/2017	FP	13A	12	2017	Year-1	1	0	Winter
12/23/2017	FP	1M	12	2017	Year-1	1	0	Winter
12/23/2017	FP	2M	12	2017	Year-1	1	0	Winter
12/23/2017	FP	3M	12	2017	Year-1	1	0	Winter
12/23/2017	FP	4M	12	2017	Year-1		1	Winter
12/23/2017	FP	5M	12	2017	Year-1	0	0	Winter
12/23/2017	FP	6M	12	2017	Year-1	0	0	Winter
12/23/2017	FP	7M	12	2017	Year-1	1	0	Winter
12/23/2017	FP	10M	12	2017	Year-1	1	0	Winter
12/23/2017	FP	12M	12	2017	Year-1	1	0	Winter
12/23/2017	FP	14M	12	2017	Year-1		1	Winter
12/23/2017	FP	8A	12	2017	Year-1	1	0	Winter
12/23/2017	FP	9A	12	2017	Year-1	1	0	Winter
12/23/2017	FP	11A	12	2017	Year-1	1	0	Winter
12/23/2017	FP	13A	12	2017	Year-1	1	0	Winter
12/24/2017	FP	1M	12	2017	Year-1	1	0	Winter
12/24/2017	FP	2M	12	2017	Year-1	1	0	Winter
12/24/2017	FP	3M	12	2017	Year-1	1	0	Winter

Date	Species	Site	Month	Year	Survey Year	Presence	Data Gap	Season
12/24/2017	FP	4M	12	2017	Year-1		1	Winter
12/24/2017	FP	5M	12	2017	Year-1	1	0	Winter
12/24/2017	FP	6M	12	2017	Year-1	0	0	Winter
12/24/2017	FP	7M	12	2017	Year-1	0	0	Winter
12/24/2017	FP	10M	12	2017	Year-1	1	0	Winter
12/24/2017	FP	12M	12	2017	Year-1	1	0	Winter
12/24/2017	FP	14M	12	2017	Year-1		1	Winter
12/24/2017	FP	8A	12	2017	Year-1	1	0	Winter
12/24/2017	FP	9A	12	2017	Year-1	1	0	Winter
12/24/2017	FP	11A	12	2017	Year-1	1	0	Winter
12/24/2017	FP	13A	12	2017	Year-1	1	0	Winter
12/25/2017	FP	1M	12	2017	Year-1	1	0	Winter
12/25/2017	FP	2M	12	2017	Year-1	1	0	Winter
12/25/2017	FP	3M	12	2017	Year-1	1	0	Winter
12/25/2017	FP	4M	12	2017	Year-1		1	Winter
12/25/2017	FP	5M	12	2017	Year-1	1	0	Winter
12/25/2017	FP	6M	12	2017	Year-1	1	0	Winter
12/25/2017	FP	7M	12	2017	Year-1	1	0	Winter
12/25/2017	FP	10M	12	2017	Year-1	1	0	Winter
12/25/2017	FP	12M	12	2017	Year-1	1	0	Winter
12/25/2017	FP	14M	12	2017	Year-1		1	Winter
12/25/2017	FP	8A	12	2017	Year-1	1	0	Winter
12/25/2017	FP	9A	12	2017	Year-1	1	0	Winter
12/25/2017	FP	11A	12	2017	Year-1	1	0	Winter
12/25/2017	FP	13A	12	2017	Year-1	1	0	Winter
12/26/2017	FP	1M	12	2017	Year-1	1	0	Winter
12/26/2017	FP	2M	12	2017	Year-1	1	0	Winter
12/26/2017	FP	3M	12	2017	Year-1	1	0	Winter
12/26/2017	FP	4M	12	2017	Year-1		1	Winter
12/26/2017	FP	5M	12	2017	Year-1	1	0	Winter
12/26/2017	FP	6M	12	2017	Year-1	0	0	Winter
12/26/2017	FP	7M	12	2017	Year-1	1	0	Winter
12/26/2017	FP	10M	12	2017	Year-1	1	0	Winter
12/26/2017	FP	12M	12	2017	Year-1	1	0	Winter
12/26/2017	FP	14M	12	2017	Year-1		1	Winter
12/26/2017	FP	8A	12	2017	Year-1	1	0	Winter
12/26/2017	FP	9A	12	2017	Year-1	1	0	Winter
12/26/2017	FP	11A	12	2017	Year-1	1	0	Winter
12/26/2017	FP	13A	12	2017	Year-1	1	0	Winter
12/27/2017	FP	1M	12	2017	Year-1	1	0	Winter
12/27/2017	FP	2M	12	2017	Year-1	1	0	Winter
12/27/2017	FP	3M	12	2017	Year-1	1	0	Winter
12/27/2017	FP	4M	12	2017	Year-1		1	Winter
12/27/2017	FP	5M	12	2017	Year-1	0	0	Winter

Date	Species	Site	Month	Year	Survey Year	Presence	Data Gap	Season
12/27/2017	FP	6M	12	2017	Year-1	0	0	Winter
12/27/2017	FP	7M	12	2017	Year-1	1	0	Winter
12/27/2017	FP	10M	12	2017	Year-1	1	0	Winter
12/27/2017	FP	12M	12	2017	Year-1	1	0	Winter
12/27/2017	FP	14M	12	2017	Year-1		1	Winter
12/27/2017	FP	8A	12	2017	Year-1	1	0	Winter
12/27/2017	FP	9A	12	2017	Year-1	1	0	Winter
12/27/2017	FP	11A	12	2017	Year-1	1	0	Winter
12/27/2017	FP	13A	12	2017	Year-1	1	0	Winter
12/28/2017	FP	1M	12	2017	Year-1	1	0	Winter
12/28/2017	FP	2M	12	2017	Year-1	1	0	Winter
12/28/2017	FP	3M	12	2017	Year-1	1	0	Winter
12/28/2017	FP	4M	12	2017	Year-1		1	Winter
12/28/2017	FP	5M	12	2017	Year-1	1	0	Winter
12/28/2017	FP	6M	12	2017	Year-1	1	0	Winter
12/28/2017	FP	7M	12	2017	Year-1	1	0	Winter
12/28/2017	FP	10M	12	2017	Year-1	1	0	Winter
12/28/2017	FP	12M	12	2017	Year-1	1	0	Winter
12/28/2017	FP	14M	12	2017	Year-1		1	Winter
12/28/2017	FP	8A	12	2017	Year-1	1	0	Winter
12/28/2017	FP	9A	12	2017	Year-1	1	0	Winter
12/28/2017	FP	11A	12	2017	Year-1	1	0	Winter
12/28/2017	FP	13A	12	2017	Year-1	1	0	Winter
12/29/2017	FP	1M	12	2017	Year-1	1	0	Winter
12/29/2017	FP	2M	12	2017	Year-1	1	0	Winter
12/29/2017	FP	3M	12	2017	Year-1	1	0	Winter
12/29/2017	FP	4M	12	2017	Year-1		1	Winter
12/29/2017	FP	5M	12	2017	Year-1	1	0	Winter
12/29/2017	FP	6M	12	2017	Year-1	0	0	Winter
12/29/2017	FP	7M	12	2017	Year-1	1	0	Winter
12/29/2017	FP	10M	12	2017	Year-1	1	0	Winter
12/29/2017	FP	12M	12	2017	Year-1	1	0	Winter
12/29/2017	FP	14M	12	2017	Year-1		1	Winter
12/29/2017	FP	8A	12	2017	Year-1	1	0	Winter
12/29/2017	FP	9A	12	2017	Year-1	1	0	Winter
12/29/2017	FP	11A	12	2017	Year-1	1	0	Winter
12/29/2017	FP	13A	12	2017	Year-1	1	0	Winter
12/30/2017	FP	1M	12	2017	Year-1	1	0	Winter
12/30/2017	FP	2M	12	2017	Year-1	1	0	Winter
12/30/2017	FP	3M	12	2017	Year-1	1	0	Winter
12/30/2017	FP	4M	12	2017	Year-1		1	Winter
12/30/2017	FP	5M	12	2017	Year-1	1	0	Winter
12/30/2017	FP	6M	12	2017	Year-1	0	0	Winter
12/30/2017	FP	7M	12	2017	Year-1	1	0	Winter

Date	Species	Site	Month	Year	Survey Year	Presence	Data Gap	Season
12/30/2017	FP	10M	12	2017	Year-1	1	0	Winter
12/30/2017	FP	12M	12	2017	Year-1	1	0	Winter
12/30/2017	FP	14M	12	2017	Year-1		1	Winter
12/30/2017	FP	8A	12	2017	Year-1	1	0	Winter
12/30/2017	FP	9A	12	2017	Year-1	1	0	Winter
12/30/2017	FP	11A	12	2017	Year-1	1	0	Winter
12/30/2017	FP	13A	12	2017	Year-1	1	0	Winter
12/31/2017	FP	1M	12	2017	Year-1	1	0	Winter
12/31/2017	FP	2M	12	2017	Year-1	1	0	Winter
12/31/2017	FP	3M	12	2017	Year-1	1	0	Winter
12/31/2017	FP	4M	12	2017	Year-1		1	Winter
12/31/2017	FP	5M	12	2017	Year-1	1	0	Winter
12/31/2017	FP	6M	12	2017	Year-1	1	0	Winter
12/31/2017	FP	7M	12	2017	Year-1	1	0	Winter
12/31/2017	FP	10M	12	2017	Year-1	1	0	Winter
12/31/2017	FP	12M	12	2017	Year-1	1	0	Winter
12/31/2017	FP	14M	12	2017	Year-1		1	Winter
12/31/2017	FP	8A	12	2017	Year-1	1	0	Winter
12/31/2017	FP	9A	12	2017	Year-1	1	0	Winter
12/31/2017	FP	11A	12	2017	Year-1	1	0	Winter
12/31/2017	FP	13A	12	2017	Year-1	1	0	Winter
1/1/2018	FP	1M	1	2018	Year-1	1	0	Winter
1/1/2018	FP	2M	1	2018	Year-1	1	0	Winter
1/1/2018	FP	3M	1	2018	Year-1	1	0	Winter
1/1/2018	FP	4M	1	2018	Year-1		1	Winter
1/1/2018	FP	5M	1	2018	Year-1	1	0	Winter
1/1/2018	FP	6M	1	2018	Year-1	1	0	Winter
1/1/2018	FP	7M	1	2018	Year-1	1	0	Winter
1/1/2018	FP	10M	1	2018	Year-1	1	0	Winter
1/1/2018	FP	12M	1	2018	Year-1	1	0	Winter
1/1/2018	FP	14M	1	2018	Year-1		1	Winter
1/1/2018	FP	8A	1	2018	Year-1	1	0	Winter
1/1/2018	FP	9A	1	2018	Year-1	1	0	Winter
1/1/2018	FP	11A	1	2018	Year-1	1	0	Winter
1/1/2018	FP	13A	1	2018	Year-1	1	0	Winter
1/2/2018	FP	1M	1	2018	Year-1	1	0	Winter
1/2/2018	FP	2M	1	2018	Year-1	1	0	Winter
1/2/2018	FP	3M	1	2018	Year-1	1	0	Winter
1/2/2018	FP	4M	1	2018	Year-1		1	Winter
1/2/2018	FP	5M	1	2018	Year-1	1	0	Winter
1/2/2018	FP	6M	1	2018	Year-1	1	0	Winter
1/2/2018	FP	7M	1	2018	Year-1	1	0	Winter
1/2/2018	FP	10M	1	2018	Year-1	1	0	Winter
1/2/2018	FP	12M	1	2018	Year-1	1	0	Winter

Date	Species	Site	Month	Year	Survey Year	Presence	Data Gap	Season
1/2/2018	FP	14M	1	2018	Year-1		1	Winter
1/2/2018	FP	8A	1	2018	Year-1	1	0	Winter
1/2/2018	FP	9A	1	2018	Year-1	1	0	Winter
1/2/2018	FP	11A	1	2018	Year-1	1	0	Winter
1/2/2018	FP	13A	1	2018	Year-1	1	0	Winter
1/3/2018	FP	1M	1	2018	Year-1	1	0	Winter
1/3/2018	FP	2M	1	2018	Year-1	1	0	Winter
1/3/2018	FP	3M	1	2018	Year-1	1	0	Winter
1/3/2018	FP	4M	1	2018	Year-1		1	Winter
1/3/2018	FP	5M	1	2018	Year-1	1	0	Winter
1/3/2018	FP	6M	1	2018	Year-1	0	0	Winter
1/3/2018	FP	7M	1	2018	Year-1	1	0	Winter
1/3/2018	FP	10M	1	2018	Year-1	1	0	Winter
1/3/2018	FP	12M	1	2018	Year-1	1	0	Winter
1/3/2018	FP	14M	1	2018	Year-1		1	Winter
1/3/2018	FP	8A	1	2018	Year-1	1	0	Winter
1/3/2018	FP	9A	1	2018	Year-1	1	0	Winter
1/3/2018	FP	11A	1	2018	Year-1	1	0	Winter
1/3/2018	FP	13A	1	2018	Year-1	1	0	Winter
1/4/2018	FP	1M	1	2018	Year-1	1	0	Winter
1/4/2018	FP	2M	1	2018	Year-1	1	0	Winter
1/4/2018	FP	3M	1	2018	Year-1	1	0	Winter
1/4/2018	FP	4M	1	2018	Year-1		1	Winter
1/4/2018	FP	5M	1	2018	Year-1	1	0	Winter
1/4/2018	FP	6M	1	2018	Year-1	1	0	Winter
1/4/2018	FP	7M	1	2018	Year-1	1	0	Winter
1/4/2018	FP	10M	1	2018	Year-1	1	0	Winter
1/4/2018	FP	12M	1	2018	Year-1	0	0	Winter
1/4/2018	FP	14M	1	2018	Year-1		1	Winter
1/4/2018	FP	8A	1	2018	Year-1	1	0	Winter
1/4/2018	FP	9A	1	2018	Year-1	1	0	Winter
1/4/2018	FP	11A	1	2018	Year-1	1	0	Winter
1/4/2018	FP	13A	1	2018	Year-1	1	0	Winter
1/5/2018	FP	1M	1	2018	Year-1	1	0	Winter
1/5/2018	FP	2M	1	2018	Year-1	1	0	Winter
1/5/2018	FP	3M	1	2018	Year-1	0	0	Winter
1/5/2018	FP	4M	1	2018	Year-1		1	Winter
1/5/2018	FP	5M	1	2018	Year-1	1	0	Winter
1/5/2018	FP	6M	1	2018	Year-1	0	0	Winter
1/5/2018	FP	7M	1	2018	Year-1	1	0	Winter
1/5/2018	FP	10M	1	2018	Year-1	1	0	Winter
1/5/2018	FP	12M	1	2018	Year-1	1	0	Winter
1/5/2018	FP	14M	1	2018	Year-1		1	Winter
1/5/2018	FP	8A	1	2018	Year-1	1	0	Winter

Date	Species	Site	Month	Year	Survey Year	Presence	Data Gap	Season
1/5/2018	FP	9A	1	2018	Year-1	1	0	Winter
1/5/2018	FP	11A	1	2018	Year-1	1	0	Winter
1/5/2018	FP	13A	1	2018	Year-1	0	0	Winter
1/6/2018	FP	1M	1	2018	Year-1	1	0	Winter
1/6/2018	FP	2M	1	2018	Year-1	1	0	Winter
1/6/2018	FP	3M	1	2018	Year-1	1	0	Winter
1/6/2018	FP	4M	1	2018	Year-1		1	Winter
1/6/2018	FP	5M	1	2018	Year-1	1	0	Winter
1/6/2018	FP	6M	1	2018	Year-1	0	0	Winter
1/6/2018	FP	7M	1	2018	Year-1	1	0	Winter
1/6/2018	FP	10M	1	2018	Year-1	0	0	Winter
1/6/2018	FP	12M	1	2018	Year-1	1	0	Winter
1/6/2018	FP	14M	1	2018	Year-1		1	Winter
1/6/2018	FP	8A	1	2018	Year-1	1	0	Winter
1/6/2018	FP	9A	1	2018	Year-1	1	0	Winter
1/6/2018	FP	11A	1	2018	Year-1	1	0	Winter
1/6/2018	FP	13A	1	2018	Year-1	0	0	Winter
1/7/2018	FP	1M	1	2018	Year-1	1	0	Winter
1/7/2018	FP	2M	1	2018	Year-1	1	0	Winter
1/7/2018	FP	3M	1	2018	Year-1	1	0	Winter
1/7/2018	FP	4M	1	2018	Year-1		1	Winter
1/7/2018	FP	5M	1	2018	Year-1	1	0	Winter
1/7/2018	FP	6M	1	2018	Year-1	0	0	Winter
1/7/2018	FP	7M	1	2018	Year-1	1	0	Winter
1/7/2018	FP	10M	1	2018	Year-1	1	0	Winter
1/7/2018	FP	12M	1	2018	Year-1	1	0	Winter
1/7/2018	FP	14M	1	2018	Year-1		1	Winter
1/7/2018	FP	8A	1	2018	Year-1	1	0	Winter
1/7/2018	FP	9A	1	2018	Year-1	1	0	Winter
1/7/2018	FP	11A	1	2018	Year-1	1	0	Winter
1/7/2018	FP	13A	1	2018	Year-1	1	0	Winter
1/8/2018	FP	1M	1	2018	Year-1	1	0	Winter
1/8/2018	FP	2M	1	2018	Year-1	1	0	Winter
1/8/2018	FP	3M	1	2018	Year-1	1	0	Winter
1/8/2018	FP	4M	1	2018	Year-1		1	Winter
1/8/2018	FP	5M	1	2018	Year-1	1	0	Winter
1/8/2018	FP	6M	1	2018	Year-1	0	0	Winter
1/8/2018	FP	7M	1	2018	Year-1	1	0	Winter
1/8/2018	FP	10M	1	2018	Year-1	1	0	Winter
1/8/2018	FP	12M	1	2018	Year-1	1	0	Winter
1/8/2018	FP	14M	1	2018	Year-1		1	Winter
1/8/2018	FP	8A	1	2018	Year-1	1	0	Winter
1/8/2018	FP	9A	1	2018	Year-1	1	0	Winter
1/8/2018	FP	11A	1	2018	Year-1	1	0	Winter

Date	Species	Site	Month	Year	Survey Year	Presence	Data Gap	Season
1/8/2018	FP	13A	1	2018	Year-1	1	0	Winter
1/9/2018	FP	1M	1	2018	Year-1	1	0	Winter
1/9/2018	FP	2M	1	2018	Year-1	1	0	Winter
1/9/2018	FP	3M	1	2018	Year-1	1	0	Winter
1/9/2018	FP	4M	1	2018	Year-1		1	Winter
1/9/2018	FP	5M	1	2018	Year-1	1	0	Winter
1/9/2018	FP	6M	1	2018	Year-1	0	0	Winter
1/9/2018	FP	7M	1	2018	Year-1	1	0	Winter
1/9/2018	FP	10M	1	2018	Year-1	1	0	Winter
1/9/2018	FP	12M	1	2018	Year-1	1	0	Winter
1/9/2018	FP	14M	1	2018	Year-1		1	Winter
1/9/2018	FP	8A	1	2018	Year-1	0	0	Winter
1/9/2018	FP	9A	1	2018	Year-1	1	0	Winter
1/9/2018	FP	11A	1	2018	Year-1	1	0	Winter
1/9/2018	FP	13A	1	2018	Year-1	1	0	Winter
1/10/2018	FP	1M	1	2018	Year-1	1	0	Winter
1/10/2018	FP	2M	1	2018	Year-1	1	0	Winter
1/10/2018	FP	3M	1	2018	Year-1	1	0	Winter
1/10/2018	FP	4M	1	2018	Year-1		1	Winter
1/10/2018	FP	5M	1	2018	Year-1	1	0	Winter
1/10/2018	FP	6M	1	2018	Year-1	0	0	Winter
1/10/2018	FP	7M	1	2018	Year-1	1	0	Winter
1/10/2018	FP	10M	1	2018	Year-1	1	0	Winter
1/10/2018	FP	12M	1	2018	Year-1	1	0	Winter
1/10/2018	FP	14M	1	2018	Year-1		1	Winter
1/10/2018	FP	8A	1	2018	Year-1	0	0	Winter
1/10/2018	FP	9A	1	2018	Year-1	1	0	Winter
1/10/2018	FP	11A	1	2018	Year-1	1	0	Winter
1/10/2018	FP	13A	1	2018	Year-1	1	0	Winter
1/11/2018	FP	1M	1	2018	Year-1	1	0	Winter
1/11/2018	FP	2M	1	2018	Year-1	1	0	Winter
1/11/2018	FP	3M	1	2018	Year-1	1	0	Winter
1/11/2018	FP	4M	1	2018	Year-1		1	Winter
1/11/2018	FP	5M	1	2018	Year-1	1	0	Winter
1/11/2018	FP	6M	1	2018	Year-1	0	0	Winter
1/11/2018	FP	7M	1	2018	Year-1	1	0	Winter
1/11/2018	FP	10M	1	2018	Year-1	1	0	Winter
1/11/2018	FP	12M	1	2018	Year-1	1	0	Winter
1/11/2018	FP	14M	1	2018	Year-1		1	Winter
1/11/2018	FP	8A	1	2018	Year-1	0	0	Winter
1/11/2018	FP	9A	1	2018	Year-1	1	0	Winter
1/11/2018	FP	11A	1	2018	Year-1	1	0	Winter
1/11/2018	FP	13A	1	2018	Year-1	1	0	Winter
1/12/2018	FP	1M	1	2018	Year-1	1	0	Winter

Date	Species	Site	Month	Year	Survey Year	Presence	Data Gap	Season
1/12/2018	FP	2M	1	2018	Year-1	1	0	Winter
1/12/2018	FP	3M	1	2018	Year-1	1	0	Winter
1/12/2018	FP	4M	1	2018	Year-1		1	Winter
1/12/2018	FP	5M	1	2018	Year-1	0	0	Winter
1/12/2018	FP	6M	1	2018	Year-1	1	0	Winter
1/12/2018	FP	7M	1	2018	Year-1	1	0	Winter
1/12/2018	FP	10M	1	2018	Year-1	1	0	Winter
1/12/2018	FP	12M	1	2018	Year-1	1	0	Winter
1/12/2018	FP	14M	1	2018	Year-1		1	Winter
1/12/2018	FP	8A	1	2018	Year-1	1	0	Winter
1/12/2018	FP	9A	1	2018	Year-1	1	0	Winter
1/12/2018	FP	11A	1	2018	Year-1	1	0	Winter
1/12/2018	FP	13A	1	2018	Year-1	1	0	Winter
1/13/2018	FP	1M	1	2018	Year-1	1	0	Winter
1/13/2018	FP	2M	1	2018	Year-1	1	0	Winter
1/13/2018	FP	3M	1	2018	Year-1	1	0	Winter
1/13/2018	FP	4M	1	2018	Year-1		1	Winter
1/13/2018	FP	5M	1	2018	Year-1	1	0	Winter
1/13/2018	FP	6M	1	2018	Year-1	1	0	Winter
1/13/2018	FP	7M	1	2018	Year-1	1	0	Winter
1/13/2018	FP	10M	1	2018	Year-1	1	0	Winter
1/13/2018	FP	12M	1	2018	Year-1	1	0	Winter
1/13/2018	FP	14M	1	2018	Year-1		1	Winter
1/13/2018	FP	8A	1	2018	Year-1	1	0	Winter
1/13/2018	FP	9A	1	2018	Year-1	1	0	Winter
1/13/2018	FP	11A	1	2018	Year-1	1	0	Winter
1/13/2018	FP	13A	1	2018	Year-1	1	0	Winter
1/14/2018	FP	1M	1	2018	Year-1	1	0	Winter
1/14/2018	FP	2M	1	2018	Year-1	1	0	Winter
1/14/2018	FP	3M	1	2018	Year-1	1	0	Winter
1/14/2018	FP	4M	1	2018	Year-1		1	Winter
1/14/2018	FP	5M	1	2018	Year-1	1	0	Winter
1/14/2018	FP	6M	1	2018	Year-1	0	0	Winter
1/14/2018	FP	7M	1	2018	Year-1	1	0	Winter
1/14/2018	FP	10M	1	2018	Year-1	1	0	Winter
1/14/2018	FP	12M	1	2018	Year-1	1	0	Winter
1/14/2018	FP	14M	1	2018	Year-1		1	Winter
1/14/2018	FP	8A	1	2018	Year-1	1	0	Winter
1/14/2018	FP	9A	1	2018	Year-1	1	0	Winter
1/14/2018	FP	11A	1	2018	Year-1	1	0	Winter
1/14/2018	FP	13A	1	2018	Year-1	1	0	Winter
1/15/2018	FP	1M	1	2018	Year-1	1	0	Winter
1/15/2018	FP	2M	1	2018	Year-1	1	0	Winter
1/15/2018	FP	3M	1	2018	Year-1	1	0	Winter

Date	Species	Site	Month	Year	Survey Year	Presence	Data Gap	Season
1/15/2018	FP	4M	1	2018	Year-1		1	Winter
1/15/2018	FP	5M	1	2018	Year-1	1	0	Winter
1/15/2018	FP	6M	1	2018	Year-1	0	0	Winter
1/15/2018	FP	7M	1	2018	Year-1	1	0	Winter
1/15/2018	FP	10M	1	2018	Year-1	1	0	Winter
1/15/2018	FP	12M	1	2018	Year-1	1	0	Winter
1/15/2018	FP	14M	1	2018	Year-1		1	Winter
1/15/2018	FP	8A	1	2018	Year-1	1	0	Winter
1/15/2018	FP	9A	1	2018	Year-1	1	0	Winter
1/15/2018	FP	11A	1	2018	Year-1	1	0	Winter
1/15/2018	FP	13A	1	2018	Year-1	1	0	Winter
1/16/2018	FP	1M	1	2018	Year-1	1	0	Winter
1/16/2018	FP	2M	1	2018	Year-1	1	0	Winter
1/16/2018	FP	3M	1	2018	Year-1	1	0	Winter
1/16/2018	FP	4M	1	2018	Year-1		1	Winter
1/16/2018	FP	5M	1	2018	Year-1	1	0	Winter
1/16/2018	FP	6M	1	2018	Year-1	1	0	Winter
1/16/2018	FP	7M	1	2018	Year-1	1	0	Winter
1/16/2018	FP	10M	1	2018	Year-1	1	0	Winter
1/16/2018	FP	12M	1	2018	Year-1	1	0	Winter
1/16/2018	FP	14M	1	2018	Year-1		1	Winter
1/16/2018	FP	8A	1	2018	Year-1	1	0	Winter
1/16/2018	FP	9A	1	2018	Year-1	1	0	Winter
1/16/2018	FP	11A	1	2018	Year-1	1	0	Winter
1/16/2018	FP	13A	1	2018	Year-1	1	0	Winter
1/17/2018	FP	1M	1	2018	Year-1	1	0	Winter
1/17/2018	FP	2M	1	2018	Year-1	1	0	Winter
1/17/2018	FP	3M	1	2018	Year-1	1	0	Winter
1/17/2018	FP	4M	1	2018	Year-1		1	Winter
1/17/2018	FP	5M	1	2018	Year-1	1	0	Winter
1/17/2018	FP	6M	1	2018	Year-1	0	0	Winter
1/17/2018	FP	7M	1	2018	Year-1	1	0	Winter
1/17/2018	FP	10M	1	2018	Year-1	1	0	Winter
1/17/2018	FP	12M	1	2018	Year-1	1	0	Winter
1/17/2018	FP	14M	1	2018	Year-1		1	Winter
1/17/2018	FP	8A	1	2018	Year-1	1	0	Winter
1/17/2018	FP	9A	1	2018	Year-1	1	0	Winter
1/17/2018	FP	11A	1	2018	Year-1	1	0	Winter
1/17/2018	FP	13A	1	2018	Year-1	1	0	Winter
1/18/2018	FP	1M	1	2018	Year-1	1	0	Winter
1/18/2018	FP	2M	1	2018	Year-1	1	0	Winter
1/18/2018	FP	3M	1	2018	Year-1	1	0	Winter
1/18/2018	FP	4M	1	2018	Year-1		1	Winter
1/18/2018	FP	5M	1	2018	Year-1	1	0	Winter

Date	Species	Site	Month	Year	Survey Year	Presence	Data Gap	Season
1/18/2018	FP	6M	1	2018	Year-1	1	0	Winter
1/18/2018	FP	7M	1	2018	Year-1	1	0	Winter
1/18/2018	FP	10M	1	2018	Year-1	1	0	Winter
1/18/2018	FP	12M	1	2018	Year-1	1	0	Winter
1/18/2018	FP	14M	1	2018	Year-1		1	Winter
1/18/2018	FP	8A	1	2018	Year-1	1	0	Winter
1/18/2018	FP	9A	1	2018	Year-1	1	0	Winter
1/18/2018	FP	11A	1	2018	Year-1	1	0	Winter
1/18/2018	FP	13A	1	2018	Year-1	1	0	Winter
1/19/2018	FP	1M	1	2018	Year-1	1	0	Winter
1/19/2018	FP	2M	1	2018	Year-1	1	0	Winter
1/19/2018	FP	3M	1	2018	Year-1	1	0	Winter
1/19/2018	FP	4M	1	2018	Year-1		1	Winter
1/19/2018	FP	5M	1	2018	Year-1	1	0	Winter
1/19/2018	FP	6M	1	2018	Year-1	0	0	Winter
1/19/2018	FP	7M	1	2018	Year-1	1	0	Winter
1/19/2018	FP	10M	1	2018	Year-1	1	0	Winter
1/19/2018	FP	12M	1	2018	Year-1	1	0	Winter
1/19/2018	FP	14M	1	2018	Year-1		1	Winter
1/19/2018	FP	8A	1	2018	Year-1	1	0	Winter
1/19/2018	FP	9A	1	2018	Year-1	1	0	Winter
1/19/2018	FP	11A	1	2018	Year-1	1	0	Winter
1/19/2018	FP	13A	1	2018	Year-1	1	0	Winter
1/20/2018	FP	1M	1	2018	Year-1	1	0	Winter
1/20/2018	FP	2M	1	2018	Year-1	1	0	Winter
1/20/2018	FP	3M	1	2018	Year-1	1	0	Winter
1/20/2018	FP	4M	1	2018	Year-1		1	Winter
1/20/2018	FP	5M	1	2018	Year-1	1	0	Winter
1/20/2018	FP	6M	1	2018	Year-1	0	0	Winter
1/20/2018	FP	7M	1	2018	Year-1	0	0	Winter
1/20/2018	FP	10M	1	2018	Year-1	1	0	Winter
1/20/2018	FP	12M	1	2018	Year-1	1	0	Winter
1/20/2018	FP	14M	1	2018	Year-1		1	Winter
1/20/2018	FP	8A	1	2018	Year-1	1	0	Winter
1/20/2018	FP	9A	1	2018	Year-1	1	0	Winter
1/20/2018	FP	11A	1	2018	Year-1	1	0	Winter
1/20/2018	FP	13A	1	2018	Year-1	1	0	Winter
1/21/2018	FP	1M	1	2018	Year-1	1	0	Winter
1/21/2018	FP	2M	1	2018	Year-1	1	0	Winter
1/21/2018	FP	3M	1	2018	Year-1	1	0	Winter
1/21/2018	FP	4M	1	2018	Year-1		1	Winter
1/21/2018	FP	5M	1	2018	Year-1	1	0	Winter
1/21/2018	FP	6M	1	2018	Year-1	1	0	Winter
1/21/2018	FP	7M	1	2018	Year-1	1	0	Winter

Date	Species	Site	Month	Year	Survey Year	Presence	Data Gap	Season
1/21/2018	FP	10M	1	2018	Year-1	1	0	Winter
1/21/2018	FP	12M	1	2018	Year-1	1	0	Winter
1/21/2018	FP	14M	1	2018	Year-1		1	Winter
1/21/2018	FP	8A	1	2018	Year-1	1	0	Winter
1/21/2018	FP	9A	1	2018	Year-1	1	0	Winter
1/21/2018	FP	11A	1	2018	Year-1	1	0	Winter
1/21/2018	FP	13A	1	2018	Year-1	1	0	Winter
1/22/2018	FP	1M	1	2018	Year-1	1	0	Winter
1/22/2018	FP	2M	1	2018	Year-1	1	0	Winter
1/22/2018	FP	3M	1	2018	Year-1	1	0	Winter
1/22/2018	FP	4M	1	2018	Year-1		1	Winter
1/22/2018	FP	5M	1	2018	Year-1	1	0	Winter
1/22/2018	FP	6M	1	2018	Year-1	1	0	Winter
1/22/2018	FP	7M	1	2018	Year-1	1	0	Winter
1/22/2018	FP	10M	1	2018	Year-1	1	0	Winter
1/22/2018	FP	12M	1	2018	Year-1	1	0	Winter
1/22/2018	FP	14M	1	2018	Year-1		1	Winter
1/22/2018	FP	8A	1	2018	Year-1	1	0	Winter
1/22/2018	FP	9A	1	2018	Year-1	1	0	Winter
1/22/2018	FP	11A	1	2018	Year-1	1	0	Winter
1/22/2018	FP	13A	1	2018	Year-1	1	0	Winter
1/23/2018	FP	1M	1	2018	Year-1	1	0	Winter
1/23/2018	FP	2M	1	2018	Year-1	1	0	Winter
1/23/2018	FP	3M	1	2018	Year-1	1	0	Winter
1/23/2018	FP	4M	1	2018	Year-1		1	Winter
1/23/2018	FP	5M	1	2018	Year-1	1	0	Winter
1/23/2018	FP	6M	1	2018	Year-1	1	0	Winter
1/23/2018	FP	7M	1	2018	Year-1	1	0	Winter
1/23/2018	FP	10M	1	2018	Year-1	1	0	Winter
1/23/2018	FP	12M	1	2018	Year-1	1	0	Winter
1/23/2018	FP	14M	1	2018	Year-1		1	Winter
1/23/2018	FP	8A	1	2018	Year-1	1	0	Winter
1/23/2018	FP	9A	1	2018	Year-1	1	0	Winter
1/23/2018	FP	11A	1	2018	Year-1	1	0	Winter
1/23/2018	FP	13A	1	2018	Year-1	1	0	Winter
1/24/2018	FP	1M	1	2018	Year-1	1	0	Winter
1/24/2018	FP	2M	1	2018	Year-1	1	0	Winter
1/24/2018	FP	3M	1	2018	Year-1	1	0	Winter
1/24/2018	FP	4M	1	2018	Year-1		1	Winter
1/24/2018	FP	5M	1	2018	Year-1	1	0	Winter
1/24/2018	FP	6M	1	2018	Year-1	0	0	Winter
1/24/2018	FP	7M	1	2018	Year-1	1	0	Winter
1/24/2018	FP	10M	1	2018	Year-1	1	0	Winter
1/24/2018	FP	12M	1	2018	Year-1	1	0	Winter

Date	Species	Site	Month	Year	Survey Year	Presence	Data Gap	Season
1/24/2018	FP	14M	1	2018	Year-1		1	Winter
1/24/2018	FP	8A	1	2018	Year-1	0	0	Winter
1/24/2018	FP	9A	1	2018	Year-1	1	0	Winter
1/24/2018	FP	11A	1	2018	Year-1	1	0	Winter
1/24/2018	FP	13A	1	2018	Year-1	1	0	Winter
1/25/2018	FP	1M	1	2018	Year-1	1	0	Winter
1/25/2018	FP	2M	1	2018	Year-1	1	0	Winter
1/25/2018	FP	3M	1	2018	Year-1	1	0	Winter
1/25/2018	FP	4M	1	2018	Year-1		1	Winter
1/25/2018	FP	5M	1	2018	Year-1	1	0	Winter
1/25/2018	FP	6M	1	2018	Year-1	1	0	Winter
1/25/2018	FP	7M	1	2018	Year-1	1	0	Winter
1/25/2018	FP	10M	1	2018	Year-1	1	0	Winter
1/25/2018	FP	12M	1	2018	Year-1	1	0	Winter
1/25/2018	FP	14M	1	2018	Year-1		1	Winter
1/25/2018	FP	8A	1	2018	Year-1	0	0	Winter
1/25/2018	FP	9A	1	2018	Year-1	1	0	Winter
1/25/2018	FP	11A	1	2018	Year-1	1	0	Winter
1/25/2018	FP	13A	1	2018	Year-1	1	0	Winter
1/26/2018	FP	1M	1	2018	Year-1	1	0	Winter
1/26/2018	FP	2M	1	2018	Year-1	1	0	Winter
1/26/2018	FP	3M	1	2018	Year-1	1	0	Winter
1/26/2018	FP	4M	1	2018	Year-1		1	Winter
1/26/2018	FP	5M	1	2018	Year-1	1	0	Winter
1/26/2018	FP	6M	1	2018	Year-1	0	0	Winter
1/26/2018	FP	7M	1	2018	Year-1	1	0	Winter
1/26/2018	FP	10M	1	2018	Year-1	1	0	Winter
1/26/2018	FP	12M	1	2018	Year-1	1	0	Winter
1/26/2018	FP	14M	1	2018	Year-1		1	Winter
1/26/2018	FP	8A	1	2018	Year-1	0	0	Winter
1/26/2018	FP	9A	1	2018	Year-1	1	0	Winter
1/26/2018	FP	11A	1	2018	Year-1	1	0	Winter
1/26/2018	FP	13A	1	2018	Year-1	1	0	Winter
1/27/2018	FP	1M	1	2018	Year-1	1	0	Winter
1/27/2018	FP	2M	1	2018	Year-1	1	0	Winter
1/27/2018	FP	3M	1	2018	Year-1	1	0	Winter
1/27/2018	FP	4M	1	2018	Year-1		1	Winter
1/27/2018	FP	5M	1	2018	Year-1	1	0	Winter
1/27/2018	FP	6M	1	2018	Year-1	1	0	Winter
1/27/2018	FP	7M	1	2018	Year-1	1	0	Winter
1/27/2018	FP	10M	1	2018	Year-1	1	0	Winter
1/27/2018	FP	12M	1	2018	Year-1	1	0	Winter
1/27/2018	FP	14M	1	2018	Year-1		1	Winter
1/27/2018	FP	8A	1	2018	Year-1	0	0	Winter

Date	Species	Site	Month	Year	Survey Year	Presence	Data Gap	Season
1/27/2018	FP	9A	1	2018	Year-1	1	0	Winter
1/27/2018	FP	11A	1	2018	Year-1	1	0	Winter
1/27/2018	FP	13A	1	2018	Year-1	1	0	Winter
1/28/2018	FP	1M	1	2018	Year-1	1	0	Winter
1/28/2018	FP	2M	1	2018	Year-1	1	0	Winter
1/28/2018	FP	3M	1	2018	Year-1	1	0	Winter
1/28/2018	FP	4M	1	2018	Year-1		1	Winter
1/28/2018	FP	5M	1	2018	Year-1	1	0	Winter
1/28/2018	FP	6M	1	2018	Year-1	1	0	Winter
1/28/2018	FP	7M	1	2018	Year-1	0	0	Winter
1/28/2018	FP	10M	1	2018	Year-1	1	0	Winter
1/28/2018	FP	12M	1	2018	Year-1	1	0	Winter
1/28/2018	FP	14M	1	2018	Year-1		1	Winter
1/28/2018	FP	8A	1	2018	Year-1	0	0	Winter
1/28/2018	FP	9A	1	2018	Year-1	1	0	Winter
1/28/2018	FP	11A	1	2018	Year-1	1	0	Winter
1/28/2018	FP	13A	1	2018	Year-1	1	0	Winter
1/29/2018	FP	1M	1	2018	Year-1	0	0	Winter
1/29/2018	FP	2M	1	2018	Year-1	1	0	Winter
1/29/2018	FP	3M	1	2018	Year-1	1	0	Winter
1/29/2018	FP	4M	1	2018	Year-1		1	Winter
1/29/2018	FP	5M	1	2018	Year-1	1	0	Winter
1/29/2018	FP	6M	1	2018	Year-1	1	0	Winter
1/29/2018	FP	7M	1	2018	Year-1	1	0	Winter
1/29/2018	FP	10M	1	2018	Year-1	1	0	Winter
1/29/2018	FP	12M	1	2018	Year-1	1	0	Winter
1/29/2018	FP	14M	1	2018	Year-1		1	Winter
1/29/2018	FP	8A	1	2018	Year-1	0	0	Winter
1/29/2018	FP	9A	1	2018	Year-1	1	0	Winter
1/29/2018	FP	11A	1	2018	Year-1	1	0	Winter
1/29/2018	FP	13A	1	2018	Year-1	1	0	Winter
1/30/2018	FP	1M	1	2018	Year-1	1	0	Winter
1/30/2018	FP	2M	1	2018	Year-1	1	0	Winter
1/30/2018	FP	3M	1	2018	Year-1	1	0	Winter
1/30/2018	FP	4M	1	2018	Year-1		1	Winter
1/30/2018	FP	5M	1	2018	Year-1	1	0	Winter
1/30/2018	FP	6M	1	2018	Year-1	0	0	Winter
1/30/2018	FP	7M	1	2018	Year-1	1	0	Winter
1/30/2018	FP	10M	1	2018	Year-1	1	0	Winter
1/30/2018	FP	12M	1	2018	Year-1	1	0	Winter
1/30/2018	FP	14M	1	2018	Year-1		1	Winter
1/30/2018	FP	8A	1	2018	Year-1	0	0	Winter
1/30/2018	FP	9A	1	2018	Year-1	1	0	Winter
1/30/2018	FP	11A	1	2018	Year-1	1	0	Winter

Date	Species	Site	Month	Year	Survey Year	Presence	Data Gap	Season
1/30/2018	FP	13A	1	2018	Year-1	1	0	Winter
1/31/2018	FP	1M	1	2018	Year-1	1	0	Winter
1/31/2018	FP	2M	1	2018	Year-1	1	0	Winter
1/31/2018	FP	3M	1	2018	Year-1	1	0	Winter
1/31/2018	FP	4M	1	2018	Year-1		1	Winter
1/31/2018	FP	5M	1	2018	Year-1	1	0	Winter
1/31/2018	FP	6M	1	2018	Year-1	0	0	Winter
1/31/2018	FP	7M	1	2018	Year-1	0	0	Winter
1/31/2018	FP	10M	1	2018	Year-1	1	0	Winter
1/31/2018	FP	12M	1	2018	Year-1	1	0	Winter
1/31/2018	FP	14M	1	2018	Year-1		1	Winter
1/31/2018	FP	8A	1	2018	Year-1	0	0	Winter
1/31/2018	FP	9A	1	2018	Year-1	1	0	Winter
1/31/2018	FP	11A	1	2018	Year-1	1	0	Winter
1/31/2018	FP	13A	1	2018	Year-1	1	0	Winter
2/1/2018	FP	1M	2	2018	Year-1	1	0	Winter
2/1/2018	FP	2M	2	2018	Year-1	1	0	Winter
2/1/2018	FP	3M	2	2018	Year-1	1	0	Winter
2/1/2018	FP	4M	2	2018	Year-1		1	Winter
2/1/2018	FP	5M	2	2018	Year-1	0	0	Winter
2/1/2018	FP	6M	2	2018	Year-1	0	0	Winter
2/1/2018	FP	7M	2	2018	Year-1	0	0	Winter
2/1/2018	FP	10M	2	2018	Year-1	1	0	Winter
2/1/2018	FP	12M	2	2018	Year-1	1	0	Winter
2/1/2018	FP	14M	2	2018	Year-1		1	Winter
2/1/2018	FP	8A	2	2018	Year-1	0	0	Winter
2/1/2018	FP	9A	2	2018	Year-1	1	0	Winter
2/1/2018	FP	11A	2	2018	Year-1	1	0	Winter
2/1/2018	FP	13A	2	2018	Year-1	1	0	Winter
2/2/2018	FP	1M	2	2018	Year-1	1	0	Winter
2/2/2018	FP	2M	2	2018	Year-1	1	0	Winter
2/2/2018	FP	3M	2	2018	Year-1	1	0	Winter
2/2/2018	FP	4M	2	2018	Year-1		1	Winter
2/2/2018	FP	5M	2	2018	Year-1	1	0	Winter
2/2/2018	FP	6M	2	2018	Year-1	1	0	Winter
2/2/2018	FP	7M	2	2018	Year-1	0	0	Winter
2/2/2018	FP	10M	2	2018	Year-1	1	0	Winter
2/2/2018	FP	12M	2	2018	Year-1	1	0	Winter
2/2/2018	FP	14M	2	2018	Year-1		1	Winter
2/2/2018	FP	8A	2	2018	Year-1	0	0	Winter
2/2/2018	FP	9A	2	2018	Year-1	1	0	Winter
2/2/2018	FP	11A	2	2018	Year-1	1	0	Winter
2/2/2018	FP	13A	2	2018	Year-1	1	0	Winter
2/3/2018	FP	1M	2	2018	Year-1	1	0	Winter

Date	Species	Site	Month	Year	Survey Year	Presence	Data Gap	Season
2/3/2018	FP	2M	2	2018	Year-1	1	0	Winter
2/3/2018	FP	3M	2	2018	Year-1	1	0	Winter
2/3/2018	FP	4M	2	2018	Year-1		1	Winter
2/3/2018	FP	5M	2	2018	Year-1	1	0	Winter
2/3/2018	FP	6M	2	2018	Year-1	0	0	Winter
2/3/2018	FP	7M	2	2018	Year-1	1	0	Winter
2/3/2018	FP	10M	2	2018	Year-1	1	0	Winter
2/3/2018	FP	12M	2	2018	Year-1	1	0	Winter
2/3/2018	FP	14M	2	2018	Year-1		1	Winter
2/3/2018	FP	8A	2	2018	Year-1	0	0	Winter
2/3/2018	FP	9A	2	2018	Year-1	1	0	Winter
2/3/2018	FP	11A	2	2018	Year-1	1	0	Winter
2/3/2018	FP	13A	2	2018	Year-1	1	0	Winter
2/4/2018	FP	1M	2	2018	Year-1	1	0	Winter
2/4/2018	FP	2M	2	2018	Year-1	1	0	Winter
2/4/2018	FP	3M	2	2018	Year-1	1	0	Winter
2/4/2018	FP	4M	2	2018	Year-1		1	Winter
2/4/2018	FP	5M	2	2018	Year-1	0	0	Winter
2/4/2018	FP	6M	2	2018	Year-1	0	0	Winter
2/4/2018	FP	7M	2	2018	Year-1	1	0	Winter
2/4/2018	FP	10M	2	2018	Year-1	0	0	Winter
2/4/2018	FP	12M	2	2018	Year-1	0	0	Winter
2/4/2018	FP	14M	2	2018	Year-1		1	Winter
2/4/2018	FP	8A	2	2018	Year-1	0	0	Winter
2/4/2018	FP	9A	2	2018	Year-1	1	0	Winter
2/4/2018	FP	11A	2	2018	Year-1	1	0	Winter
2/4/2018	FP	13A	2	2018	Year-1	1	0	Winter
2/5/2018	FP	1M	2	2018	Year-1	1	0	Winter
2/5/2018	FP	2M	2	2018	Year-1	1	0	Winter
2/5/2018	FP	3M	2	2018	Year-1	1	0	Winter
2/5/2018	FP	4M	2	2018	Year-1		1	Winter
2/5/2018	FP	5M	2	2018	Year-1	1	0	Winter
2/5/2018	FP	6M	2	2018	Year-1	0	0	Winter
2/5/2018	FP	7M	2	2018	Year-1	0	0	Winter
2/5/2018	FP	10M	2	2018	Year-1	1	0	Winter
2/5/2018	FP	12M	2	2018	Year-1	1	0	Winter
2/5/2018	FP	14M	2	2018	Year-1		1	Winter
2/5/2018	FP	8A	2	2018	Year-1	0	0	Winter
2/5/2018	FP	9A	2	2018	Year-1	1	0	Winter
2/5/2018	FP	11A	2	2018	Year-1	1	0	Winter
2/5/2018	FP	13A	2	2018	Year-1	1	0	Winter
2/6/2018	FP	1M	2	2018	Year-1	1	0	Winter
2/6/2018	FP	2M	2	2018	Year-1	1	0	Winter
2/6/2018	FP	3M	2	2018	Year-1	1	0	Winter

Date	Species	Site	Month	Year	Survey Year	Presence	Data Gap	Season
2/6/2018	FP	4M	2	2018	Year-1		1	Winter
2/6/2018	FP	5M	2	2018	Year-1	1	0	Winter
2/6/2018	FP	6M	2	2018	Year-1	1	0	Winter
2/6/2018	FP	7M	2	2018	Year-1	1	0	Winter
2/6/2018	FP	10M	2	2018	Year-1	1	0	Winter
2/6/2018	FP	12M	2	2018	Year-1	1	0	Winter
2/6/2018	FP	14M	2	2018	Year-1		1	Winter
2/6/2018	FP	8A	2	2018	Year-1	1	0	Winter
2/6/2018	FP	9A	2	2018	Year-1	1	0	Winter
2/6/2018	FP	11A	2	2018	Year-1	1	0	Winter
2/6/2018	FP	13A	2	2018	Year-1	1	0	Winter
2/7/2018	FP	1M	2	2018	Year-1	1	0	Winter
2/7/2018	FP	2M	2	2018	Year-1	1	0	Winter
2/7/2018	FP	3M	2	2018	Year-1	1	0	Winter
2/7/2018	FP	4M	2	2018	Year-1		1	Winter
2/7/2018	FP	5M	2	2018	Year-1	1	0	Winter
2/7/2018	FP	6M	2	2018	Year-1	0	0	Winter
2/7/2018	FP	7M	2	2018	Year-1	1	0	Winter
2/7/2018	FP	10M	2	2018	Year-1	1	0	Winter
2/7/2018	FP	12M	2	2018	Year-1	1	0	Winter
2/7/2018	FP	14M	2	2018	Year-1		1	Winter
2/7/2018	FP	8A	2	2018	Year-1	1	0	Winter
2/7/2018	FP	9A	2	2018	Year-1	1	0	Winter
2/7/2018	FP	11A	2	2018	Year-1	1	0	Winter
2/7/2018	FP	13A	2	2018	Year-1	1	0	Winter
2/8/2018	FP	1M	2	2018	Year-1	1	0	Winter
2/8/2018	FP	2M	2	2018	Year-1	1	0	Winter
2/8/2018	FP	3M	2	2018	Year-1	1	0	Winter
2/8/2018	FP	4M	2	2018	Year-1		1	Winter
2/8/2018	FP	5M	2	2018	Year-1	1	0	Winter
2/8/2018	FP	6M	2	2018	Year-1	0	0	Winter
2/8/2018	FP	7M	2	2018	Year-1	1	0	Winter
2/8/2018	FP	10M	2	2018	Year-1	1	0	Winter
2/8/2018	FP	12M	2	2018	Year-1	1	0	Winter
2/8/2018	FP	14M	2	2018	Year-1		1	Winter
2/8/2018	FP	8A	2	2018	Year-1	1	0	Winter
2/8/2018	FP	9A	2	2018	Year-1	1	0	Winter
2/8/2018	FP	11A	2	2018	Year-1	1	0	Winter
2/8/2018	FP	13A	2	2018	Year-1	1	0	Winter
2/9/2018	FP	1M	2	2018	Year-1	0	0	Winter
2/9/2018	FP	2M	2	2018	Year-1	0	0	Winter
2/9/2018	FP	3M	2	2018	Year-1	0	0	Winter
2/9/2018	FP	4M	2	2018	Year-1		1	Winter
2/9/2018	FP	5M	2	2018	Year-1	0	0	Winter

Date	Species	Site	Month	Year	Survey Year	Presence	Data Gap	Season
2/9/2018	FP	6M	2	2018	Year-1	0	0	Winter
2/9/2018	FP	7M	2	2018	Year-1	0	0	Winter
2/9/2018	FP	10M	2	2018	Year-1	0	0	Winter
2/9/2018	FP	12M	2	2018	Year-1	0	0	Winter
2/9/2018	FP	14M	2	2018	Year-1		1	Winter
2/9/2018	FP	8A	2	2018	Year-1	1	0	Winter
2/9/2018	FP	9A	2	2018	Year-1	1	0	Winter
2/9/2018	FP	11A	2	2018	Year-1	1	0	Winter
2/9/2018	FP	13A	2	2018	Year-1	1	0	Winter
2/10/2018	FP	1M	2	2018	Year-1	1	0	Winter
2/10/2018	FP	2M	2	2018	Year-1	1	0	Winter
2/10/2018	FP	3M	2	2018	Year-1	1	0	Winter
2/10/2018	FP	4M	2	2018	Year-1		1	Winter
2/10/2018	FP	5M	2	2018	Year-1	1	0	Winter
2/10/2018	FP	6M	2	2018	Year-1	0	0	Winter
2/10/2018	FP	7M	2	2018	Year-1	1	0	Winter
2/10/2018	FP	10M	2	2018	Year-1	1	0	Winter
2/10/2018	FP	12M	2	2018	Year-1	1	0	Winter
2/10/2018	FP	14M	2	2018	Year-1		1	Winter
2/10/2018	FP	8A	2	2018	Year-1	1	0	Winter
2/10/2018	FP	9A	2	2018	Year-1	1	0	Winter
2/10/2018	FP	11A	2	2018	Year-1	1	0	Winter
2/10/2018	FP	13A	2	2018	Year-1	1	0	Winter
2/11/2018	FP	1M	2	2018	Year-1	1	0	Winter
2/11/2018	FP	2M	2	2018	Year-1	1	0	Winter
2/11/2018	FP	3M	2	2018	Year-1	1	0	Winter
2/11/2018	FP	4M	2	2018	Year-1		1	Winter
2/11/2018	FP	5M	2	2018	Year-1	1	0	Winter
2/11/2018	FP	6M	2	2018	Year-1	0	0	Winter
2/11/2018	FP	7M	2	2018	Year-1	1	0	Winter
2/11/2018	FP	10M	2	2018	Year-1	1	0	Winter
2/11/2018	FP	12M	2	2018	Year-1	1	0	Winter
2/11/2018	FP	14M	2	2018	Year-1		1	Winter
2/11/2018	FP	8A	2	2018	Year-1	0	0	Winter
2/11/2018	FP	9A	2	2018	Year-1	1	0	Winter
2/11/2018	FP	11A	2	2018	Year-1	1	0	Winter
2/11/2018	FP	13A	2	2018	Year-1	1	0	Winter
2/12/2018	FP	1M	2	2018	Year-1	1	0	Winter
2/12/2018	FP	2M	2	2018	Year-1	1	0	Winter
2/12/2018	FP	3M	2	2018	Year-1	1	0	Winter
2/12/2018	FP	4M	2	2018	Year-1		1	Winter
2/12/2018	FP	5M	2	2018	Year-1	1	0	Winter
2/12/2018	FP	6M	2	2018	Year-1	0	0	Winter
2/12/2018	FP	7M	2	2018	Year-1	1	0	Winter

Date	Species	Site	Month	Year	Survey Year	Presence	Data Gap	Season
2/12/2018	FP	10M	2	2018	Year-1	1	0	Winter
2/12/2018	FP	12M	2	2018	Year-1	1	0	Winter
2/12/2018	FP	14M	2	2018	Year-1		1	Winter
2/12/2018	FP	8A	2	2018	Year-1	1	0	Winter
2/12/2018	FP	9A	2	2018	Year-1	1	0	Winter
2/12/2018	FP	11A	2	2018	Year-1	1	0	Winter
2/12/2018	FP	13A	2	2018	Year-1	1	0	Winter
2/13/2018	FP	1M	2	2018	Year-1	1	0	Winter
2/13/2018	FP	2M	2	2018	Year-1	1	0	Winter
2/13/2018	FP	3M	2	2018	Year-1	1	0	Winter
2/13/2018	FP	4M	2	2018	Year-1		1	Winter
2/13/2018	FP	5M	2	2018	Year-1	1	0	Winter
2/13/2018	FP	6M	2	2018	Year-1	0	0	Winter
2/13/2018	FP	7M	2	2018	Year-1	1	0	Winter
2/13/2018	FP	10M	2	2018	Year-1	1	0	Winter
2/13/2018	FP	12M	2	2018	Year-1	1	0	Winter
2/13/2018	FP	14M	2	2018	Year-1		1	Winter
2/13/2018	FP	8A	2	2018	Year-1	1	0	Winter
2/13/2018	FP	9A	2	2018	Year-1	1	0	Winter
2/13/2018	FP	11A	2	2018	Year-1	1	0	Winter
2/13/2018	FP	13A	2	2018	Year-1	1	0	Winter
2/14/2018	FP	1M	2	2018	Year-1	1	0	Winter
2/14/2018	FP	2M	2	2018	Year-1	1	0	Winter
2/14/2018	FP	3M	2	2018	Year-1	1	0	Winter
2/14/2018	FP	4M	2	2018	Year-1		1	Winter
2/14/2018	FP	5M	2	2018	Year-1	1	0	Winter
2/14/2018	FP	6M	2	2018	Year-1	0	0	Winter
2/14/2018	FP	7M	2	2018	Year-1	1	0	Winter
2/14/2018	FP	10M	2	2018	Year-1	1	0	Winter
2/14/2018	FP	12M	2	2018	Year-1	1	0	Winter
2/14/2018	FP	14M	2	2018	Year-1		1	Winter
2/14/2018	FP	8A	2	2018	Year-1	1	0	Winter
2/14/2018	FP	9A	2	2018	Year-1	1	0	Winter
2/14/2018	FP	11A	2	2018	Year-1	1	0	Winter
2/14/2018	FP	13A	2	2018	Year-1	1	0	Winter
2/15/2018	FP	1M	2	2018	Year-1	1	0	Winter
2/15/2018	FP	2M	2	2018	Year-1	1	0	Winter
2/15/2018	FP	3M	2	2018	Year-1	1	0	Winter
2/15/2018	FP	4M	2	2018	Year-1		1	Winter
2/15/2018	FP	5M	2	2018	Year-1	1	0	Winter
2/15/2018	FP	6M	2	2018	Year-1	0	0	Winter
2/15/2018	FP	7M	2	2018	Year-1	1	0	Winter
2/15/2018	FP	10M	2	2018	Year-1	1	0	Winter
2/15/2018	FP	12M	2	2018	Year-1	1	0	Winter

Date	Species	Site	Month	Year	Survey Year	Presence	Data Gap	Season
2/15/2018	FP	14M	2	2018	Year-1		1	Winter
2/15/2018	FP	8A	2	2018	Year-1	1	0	Winter
2/15/2018	FP	9A	2	2018	Year-1	1	0	Winter
2/15/2018	FP	11A	2	2018	Year-1	1	0	Winter
2/15/2018	FP	13A	2	2018	Year-1	1	0	Winter
2/16/2018	FP	1M	2	2018	Year-1	1	0	Winter
2/16/2018	FP	2M	2	2018	Year-1	1	0	Winter
2/16/2018	FP	3M	2	2018	Year-1	1	0	Winter
2/16/2018	FP	4M	2	2018	Year-1		1	Winter
2/16/2018	FP	5M	2	2018	Year-1	1	0	Winter
2/16/2018	FP	6M	2	2018	Year-1	0	0	Winter
2/16/2018	FP	7M	2	2018	Year-1	1	0	Winter
2/16/2018	FP	10M	2	2018	Year-1	1	0	Winter
2/16/2018	FP	12M	2	2018	Year-1	1	0	Winter
2/16/2018	FP	14M	2	2018	Year-1		1	Winter
2/16/2018	FP	8A	2	2018	Year-1	1	0	Winter
2/16/2018	FP	9A	2	2018	Year-1	1	0	Winter
2/16/2018	FP	11A	2	2018	Year-1	1	0	Winter
2/16/2018	FP	13A	2	2018	Year-1	1	0	Winter
2/17/2018	FP	1M	2	2018	Year-1	1	0	Winter
2/17/2018	FP	2M	2	2018	Year-1	1	0	Winter
2/17/2018	FP	3M	2	2018	Year-1	1	0	Winter
2/17/2018	FP	4M	2	2018	Year-1		1	Winter
2/17/2018	FP	5M	2	2018	Year-1	1	0	Winter
2/17/2018	FP	6M	2	2018	Year-1	1	0	Winter
2/17/2018	FP	7M	2	2018	Year-1	0	0	Winter
2/17/2018	FP	10M	2	2018	Year-1	1	0	Winter
2/17/2018	FP	12M	2	2018	Year-1	1	0	Winter
2/17/2018	FP	14M	2	2018	Year-1		1	Winter
2/17/2018	FP	8A	2	2018	Year-1	1	0	Winter
2/17/2018	FP	9A	2	2018	Year-1	1	0	Winter
2/17/2018	FP	11A	2	2018	Year-1	1	0	Winter
2/17/2018	FP	13A	2	2018	Year-1	1	0	Winter
2/18/2018	FP	1M	2	2018	Year-1	1	0	Winter
2/18/2018	FP	2M	2	2018	Year-1	1	0	Winter
2/18/2018	FP	3M	2	2018	Year-1	1	0	Winter
2/18/2018	FP	4M	2	2018	Year-1		1	Winter
2/18/2018	FP	5M	2	2018	Year-1	1	0	Winter
2/18/2018	FP	6M	2	2018	Year-1	0	0	Winter
2/18/2018	FP	7M	2	2018	Year-1	0	0	Winter
2/18/2018	FP	10M	2	2018	Year-1	1	0	Winter
2/18/2018	FP	12M	2	2018	Year-1	1	0	Winter
2/18/2018	FP	14M	2	2018	Year-1		1	Winter
2/18/2018	FP	8A	2	2018	Year-1	1	0	Winter

Date	Species	Site	Month	Year	Survey Year	Presence	Data Gap	Season
2/18/2018	FP	9A	2	2018	Year-1	1	0	Winter
2/18/2018	FP	11A	2	2018	Year-1	1	0	Winter
2/18/2018	FP	13A	2	2018	Year-1	1	0	Winter
2/19/2018	FP	1M	2	2018	Year-1	1	0	Winter
2/19/2018	FP	2M	2	2018	Year-1	1	0	Winter
2/19/2018	FP	3M	2	2018	Year-1	1	0	Winter
2/19/2018	FP	4M	2	2018	Year-1		1	Winter
2/19/2018	FP	5M	2	2018	Year-1	1	0	Winter
2/19/2018	FP	6M	2	2018	Year-1	0	0	Winter
2/19/2018	FP	7M	2	2018	Year-1	1	0	Winter
2/19/2018	FP	10M	2	2018	Year-1	1	0	Winter
2/19/2018	FP	12M	2	2018	Year-1	1	0	Winter
2/19/2018	FP	14M	2	2018	Year-1		1	Winter
2/19/2018	FP	8A	2	2018	Year-1	1	0	Winter
2/19/2018	FP	9A	2	2018	Year-1	1	0	Winter
2/19/2018	FP	11A	2	2018	Year-1	1	0	Winter
2/19/2018	FP	13A	2	2018	Year-1	1	0	Winter
2/20/2018	FP	1M	2	2018	Year-1	1	0	Winter
2/20/2018	FP	2M	2	2018	Year-1	1	0	Winter
2/20/2018	FP	3M	2	2018	Year-1	1	0	Winter
2/20/2018	FP	4M	2	2018	Year-1		1	Winter
2/20/2018	FP	5M	2	2018	Year-1	1	0	Winter
2/20/2018	FP	6M	2	2018	Year-1	0	0	Winter
2/20/2018	FP	7M	2	2018	Year-1	1	0	Winter
2/20/2018	FP	10M	2	2018	Year-1	1	0	Winter
2/20/2018	FP	12M	2	2018	Year-1	1	0	Winter
2/20/2018	FP	14M	2	2018	Year-1		1	Winter
2/20/2018	FP	8A	2	2018	Year-1	1	0	Winter
2/20/2018	FP	9A	2	2018	Year-1	1	0	Winter
2/20/2018	FP	11A	2	2018	Year-1	1	0	Winter
2/20/2018	FP	13A	2	2018	Year-1	1	0	Winter
2/21/2018	FP	1M	2	2018	Year-1	1	0	Winter
2/21/2018	FP	2M	2	2018	Year-1	1	0	Winter
2/21/2018	FP	3M	2	2018	Year-1	1	0	Winter
2/21/2018	FP	4M	2	2018	Year-1		1	Winter
2/21/2018	FP	5M	2	2018	Year-1	1	0	Winter
2/21/2018	FP	6M	2	2018	Year-1	0	0	Winter
2/21/2018	FP	7M	2	2018	Year-1	0	0	Winter
2/21/2018	FP	10M	2	2018	Year-1	1	0	Winter
2/21/2018	FP	12M	2	2018	Year-1	1	0	Winter
2/21/2018	FP	14M	2	2018	Year-1		1	Winter
2/21/2018	FP	8A	2	2018	Year-1	1	0	Winter
2/21/2018	FP	9A	2	2018	Year-1	1	0	Winter
2/21/2018	FP	11A	2	2018	Year-1	1	0	Winter

Date	Species	Site	Month	Year	Survey Year	Presence	Data Gap	Season
2/21/2018	FP	13A	2	2018	Year-1	1	0	Winter
2/22/2018	FP	1M	2	2018	Year-1	1	0	Winter
2/22/2018	FP	2M	2	2018	Year-1	1	0	Winter
2/22/2018	FP	3M	2	2018	Year-1	1	0	Winter
2/22/2018	FP	4M	2	2018	Year-1		1	Winter
2/22/2018	FP	5M	2	2018	Year-1	1	0	Winter
2/22/2018	FP	6M	2	2018	Year-1	1	0	Winter
2/22/2018	FP	7M	2	2018	Year-1	0	0	Winter
2/22/2018	FP	10M	2	2018	Year-1	1	0	Winter
2/22/2018	FP	12M	2	2018	Year-1	1	0	Winter
2/22/2018	FP	14M	2	2018	Year-1		1	Winter
2/22/2018	FP	8A	2	2018	Year-1	1	0	Winter
2/22/2018	FP	9A	2	2018	Year-1	1	0	Winter
2/22/2018	FP	11A	2	2018	Year-1	1	0	Winter
2/22/2018	FP	13A	2	2018	Year-1	1	0	Winter
2/23/2018	FP	1M	2	2018	Year-1	1	0	Winter
2/23/2018	FP	2M	2	2018	Year-1	1	0	Winter
2/23/2018	FP	3M	2	2018	Year-1	1	0	Winter
2/23/2018	FP	4M	2	2018	Year-1		1	Winter
2/23/2018	FP	5M	2	2018	Year-1	0	0	Winter
2/23/2018	FP	6M	2	2018	Year-1	0	0	Winter
2/23/2018	FP	7M	2	2018	Year-1	0	0	Winter
2/23/2018	FP	10M	2	2018	Year-1	1	0	Winter
2/23/2018	FP	12M	2	2018	Year-1	1	0	Winter
2/23/2018	FP	14M	2	2018	Year-1		1	Winter
2/23/2018	FP	8A	2	2018	Year-1	1	0	Winter
2/23/2018	FP	9A	2	2018	Year-1	1	0	Winter
2/23/2018	FP	11A	2	2018	Year-1	1	0	Winter
2/23/2018	FP	13A	2	2018	Year-1	1	0	Winter
2/24/2018	FP	1M	2	2018	Year-1	1	0	Winter
2/24/2018	FP	2M	2	2018	Year-1	1	0	Winter
2/24/2018	FP	3M	2	2018	Year-1	1	0	Winter
2/24/2018	FP	4M	2	2018	Year-1		1	Winter
2/24/2018	FP	5M	2	2018	Year-1		1	Winter
2/24/2018	FP	6M	2	2018	Year-1	1	0	Winter
2/24/2018	FP	7M	2	2018	Year-1	1	0	Winter
2/24/2018	FP	10M	2	2018	Year-1		1	Winter
2/24/2018	FP	12M	2	2018	Year-1	1	0	Winter
2/24/2018	FP	14M	2	2018	Year-1		1	Winter
2/24/2018	FP	8A	2	2018	Year-1	1	0	Winter
2/24/2018	FP	9A	2	2018	Year-1	1	0	Winter
2/24/2018	FP	11A	2	2018	Year-1	1	0	Winter
2/24/2018	FP	13A	2	2018	Year-1	1	0	Winter
2/25/2018	FP	1M	2	2018	Year-1	1	0	Winter

Date	Species	Site	Month	Year	Survey Year	Presence	Data Gap	Season
2/25/2018	FP	2M	2	2018	Year-1	1	0	Winter
2/25/2018	FP	3M	2	2018	Year-1	1	0	Winter
2/25/2018	FP	4M	2	2018	Year-1		1	Winter
2/25/2018	FP	5M	2	2018	Year-1		1	Winter
2/25/2018	FP	6M	2	2018	Year-1	1	0	Winter
2/25/2018	FP	7M	2	2018	Year-1	0	0	Winter
2/25/2018	FP	10M	2	2018	Year-1		1	Winter
2/25/2018	FP	12M	2	2018	Year-1	1	0	Winter
2/25/2018	FP	14M	2	2018	Year-1		1	Winter
2/25/2018	FP	8A	2	2018	Year-1	1	0	Winter
2/25/2018	FP	9A	2	2018	Year-1	1	0	Winter
2/25/2018	FP	11A	2	2018	Year-1	1	0	Winter
2/25/2018	FP	13A	2	2018	Year-1	1	0	Winter
2/26/2018	FP	1M	2	2018	Year-1	1	0	Winter
2/26/2018	FP	2M	2	2018	Year-1	1	0	Winter
2/26/2018	FP	3M	2	2018	Year-1	1	0	Winter
2/26/2018	FP	4M	2	2018	Year-1		1	Winter
2/26/2018	FP	5M	2	2018	Year-1		1	Winter
2/26/2018	FP	6M	2	2018	Year-1	0	0	Winter
2/26/2018	FP	7M	2	2018	Year-1	0	0	Winter
2/26/2018	FP	10M	2	2018	Year-1		1	Winter
2/26/2018	FP	12M	2	2018	Year-1	1	0	Winter
2/26/2018	FP	14M	2	2018	Year-1		1	Winter
2/26/2018	FP	8A	2	2018	Year-1	0	0	Winter
2/26/2018	FP	9A	2	2018	Year-1	1	0	Winter
2/26/2018	FP	11A	2	2018	Year-1	1	0	Winter
2/26/2018	FP	13A	2	2018	Year-1	1	0	Winter
2/27/2018	FP	1M	2	2018	Year-1	1	0	Winter
2/27/2018	FP	2M	2	2018	Year-1	1	0	Winter
2/27/2018	FP	3M	2	2018	Year-1	1	0	Winter
2/27/2018	FP	4M	2	2018	Year-1		1	Winter
2/27/2018	FP	5M	2	2018	Year-1		1	Winter
2/27/2018	FP	6M	2	2018	Year-1	0	0	Winter
2/27/2018	FP	7M	2	2018	Year-1	0	0	Winter
2/27/2018	FP	10M	2	2018	Year-1		1	Winter
2/27/2018	FP	12M	2	2018	Year-1	1	0	Winter
2/27/2018	FP	14M	2	2018	Year-1		1	Winter
2/27/2018	FP	8A	2	2018	Year-1	0	0	Winter
2/27/2018	FP	9A	2	2018	Year-1	1	0	Winter
2/27/2018	FP	11A	2	2018	Year-1	1	0	Winter
2/27/2018	FP	13A	2	2018	Year-1	1	0	Winter
2/28/2018	FP	1M	2	2018	Year-1	0	0	Winter
2/28/2018	FP	2M	2	2018	Year-1	0	0	Winter
2/28/2018	FP	3M	2	2018	Year-1	0	0	Winter

Date	Species	Site	Month	Year	Survey Year	Presence	Data Gap	Season
2/28/2018	FP	4M	2	2018	Year-1		1	Winter
2/28/2018	FP	5M	2	2018	Year-1		1	Winter
2/28/2018	FP	6M	2	2018	Year-1	0	0	Winter
2/28/2018	FP	7M	2	2018	Year-1	0	0	Winter
2/28/2018	FP	10M	2	2018	Year-1		1	Winter
2/28/2018	FP	12M	2	2018	Year-1	0	0	Winter
2/28/2018	FP	14M	2	2018	Year-1		1	Winter
2/28/2018	FP	8A	2	2018	Year-1	0	0	Winter
2/28/2018	FP	9A	2	2018	Year-1	0	0	Winter
2/28/2018	FP	11A	2	2018	Year-1	1	0	Winter
2/28/2018	FP	13A	2	2018	Year-1	1	0	Winter
3/1/2018	FP	1M	3	2018	Year-1	0	0	Spring
3/1/2018	FP	2M	3	2018	Year-1	0	0	Spring
3/1/2018	FP	3M	3	2018	Year-1	0	0	Spring
3/1/2018	FP	4M	3	2018	Year-1		1	Spring
3/1/2018	FP	5M	3	2018	Year-1		1	Spring
3/1/2018	FP	6M	3	2018	Year-1	1	0	Spring
3/1/2018	FP	7M	3	2018	Year-1	0	0	Spring
3/1/2018	FP	10M	3	2018	Year-1		1	Spring
3/1/2018	FP	12M	3	2018	Year-1	0	0	Spring
3/1/2018	FP	14M	3	2018	Year-1		1	Spring
3/1/2018	FP	8A	3	2018	Year-1	1	0	Spring
3/1/2018	FP	9A	3	2018	Year-1	1	0	Spring
3/1/2018	FP	11A	3	2018	Year-1	1	0	Spring
3/1/2018	FP	13A	3	2018	Year-1	1	0	Spring
3/2/2018	FP	1M	3	2018	Year-1	1	0	Spring
3/2/2018	FP	2M	3	2018	Year-1	1	0	Spring
3/2/2018	FP	3M	3	2018	Year-1	1	0	Spring
3/2/2018	FP	4M	3	2018	Year-1		1	Spring
3/2/2018	FP	5M	3	2018	Year-1		1	Spring
3/2/2018	FP	6M	3	2018	Year-1	0	0	Spring
3/2/2018	FP	7M	3	2018	Year-1	0	0	Spring
3/2/2018	FP	10M	3	2018	Year-1		1	Spring
3/2/2018	FP	12M	3	2018	Year-1	1	0	Spring
3/2/2018	FP	14M	3	2018	Year-1		1	Spring
3/2/2018	FP	8A	3	2018	Year-1	0	0	Spring
3/2/2018	FP	9A	3	2018	Year-1	1	0	Spring
3/2/2018	FP	11A	3	2018	Year-1	1	0	Spring
3/2/2018	FP	13A	3	2018	Year-1	1	0	Spring
3/3/2018	FP	1M	3	2018	Year-1	0	0	Spring
3/3/2018	FP	2M	3	2018	Year-1	0	0	Spring
3/3/2018	FP	3M	3	2018	Year-1	0	0	Spring
3/3/2018	FP	4M	3	2018	Year-1		1	Spring
3/3/2018	FP	5M	3	2018	Year-1		1	Spring

Date	Species	Site	Month	Year	Survey Year	Presence	Data Gap	Season
3/3/2018	FP	6M	3	2018	Year-1	0	0	Spring
3/3/2018	FP	7M	3	2018	Year-1	0	0	Spring
3/3/2018	FP	10M	3	2018	Year-1		1	Spring
3/3/2018	FP	12M	3	2018	Year-1	1	0	Spring
3/3/2018	FP	14M	3	2018	Year-1		1	Spring
3/3/2018	FP	8A	3	2018	Year-1	0	0	Spring
3/3/2018	FP	9A	3	2018	Year-1	0	0	Spring
3/3/2018	FP	11A	3	2018	Year-1	1	0	Spring
3/3/2018	FP	13A	3	2018	Year-1	1	0	Spring
3/4/2018	FP	1M	3	2018	Year-1	0	0	Spring
3/4/2018	FP	2M	3	2018	Year-1	1	0	Spring
3/4/2018	FP	3M	3	2018	Year-1	1	0	Spring
3/4/2018	FP	4M	3	2018	Year-1		1	Spring
3/4/2018	FP	5M	3	2018	Year-1		1	Spring
3/4/2018	FP	6M	3	2018	Year-1	0	0	Spring
3/4/2018	FP	7M	3	2018	Year-1	0	0	Spring
3/4/2018	FP	10M	3	2018	Year-1		1	Spring
3/4/2018	FP	12M	3	2018	Year-1	1	0	Spring
3/4/2018	FP	14M	3	2018	Year-1		1	Spring
3/4/2018	FP	8A	3	2018	Year-1	0	0	Spring
3/4/2018	FP	9A	3	2018	Year-1	0	0	Spring
3/4/2018	FP	11A	3	2018	Year-1	1	0	Spring
3/4/2018	FP	13A	3	2018	Year-1	1	0	Spring
3/5/2018	FP	1M	3	2018	Year-1	0	0	Spring
3/5/2018	FP	2M	3	2018	Year-1	1	0	Spring
3/5/2018	FP	3M	3	2018	Year-1	1	0	Spring
3/5/2018	FP	4M	3	2018	Year-1		1	Spring
3/5/2018	FP	5M	3	2018	Year-1		1	Spring
3/5/2018	FP	6M	3	2018	Year-1	0	0	Spring
3/5/2018	FP	7M	3	2018	Year-1	0	0	Spring
3/5/2018	FP	10M	3	2018	Year-1		1	Spring
3/5/2018	FP	12M	3	2018	Year-1	1	0	Spring
3/5/2018	FP	14M	3	2018	Year-1		1	Spring
3/5/2018	FP	8A	3	2018	Year-1	0	0	Spring
3/5/2018	FP	9A	3	2018	Year-1	0	0	Spring
3/5/2018	FP	11A	3	2018	Year-1	1	0	Spring
3/5/2018	FP	13A	3	2018	Year-1	1	0	Spring
3/6/2018	FP	1M	3	2018	Year-1	0	0	Spring
3/6/2018	FP	2M	3	2018	Year-1	0	0	Spring
3/6/2018	FP	3M	3	2018	Year-1	0	0	Spring
3/6/2018	FP	4M	3	2018	Year-1		1	Spring
3/6/2018	FP	5M	3	2018	Year-1		1	Spring
3/6/2018	FP	6M	3	2018	Year-1	0	0	Spring
3/6/2018	FP	7M	3	2018	Year-1	0	0	Spring

Date	Species	Site	Month	Year	Survey Year	Presence	Data Gap	Season
3/6/2018	FP	10M	3	2018	Year-1		1	Spring
3/6/2018	FP	12M	3	2018	Year-1	1	0	Spring
3/6/2018	FP	14M	3	2018	Year-1		1	Spring
3/6/2018	FP	8A	3	2018	Year-1	0	0	Spring
3/6/2018	FP	9A	3	2018	Year-1	0	0	Spring
3/6/2018	FP	11A	3	2018	Year-1	1	0	Spring
3/6/2018	FP	13A	3	2018	Year-1	1	0	Spring
3/7/2018	FP	1M	3	2018	Year-1	0	0	Spring
3/7/2018	FP	2M	3	2018	Year-1	0	0	Spring
3/7/2018	FP	3M	3	2018	Year-1	1	0	Spring
3/7/2018	FP	4M	3	2018	Year-1		1	Spring
3/7/2018	FP	5M	3	2018	Year-1		1	Spring
3/7/2018	FP	6M	3	2018	Year-1	0	0	Spring
3/7/2018	FP	7M	3	2018	Year-1	0	0	Spring
3/7/2018	FP	10M	3	2018	Year-1		1	Spring
3/7/2018	FP	12M	3	2018	Year-1	1	0	Spring
3/7/2018	FP	14M	3	2018	Year-1		1	Spring
3/7/2018	FP	8A	3	2018	Year-1	0	0	Spring
3/7/2018	FP	9A	3	2018	Year-1	1	0	Spring
3/7/2018	FP	11A	3	2018	Year-1	1	0	Spring
3/7/2018	FP	13A	3	2018	Year-1	1	0	Spring
3/8/2018	FP	1M	3	2018	Year-1	0	0	Spring
3/8/2018	FP	2M	3	2018	Year-1	1	0	Spring
3/8/2018	FP	3M	3	2018	Year-1	1	0	Spring
3/8/2018	FP	4M	3	2018	Year-1		1	Spring
3/8/2018	FP	5M	3	2018	Year-1		1	Spring
3/8/2018	FP	6M	3	2018	Year-1	0	0	Spring
3/8/2018	FP	7M	3	2018	Year-1	0	0	Spring
3/8/2018	FP	10M	3	2018	Year-1		1	Spring
3/8/2018	FP	12M	3	2018	Year-1	1	0	Spring
3/8/2018	FP	14M	3	2018	Year-1		1	Spring
3/8/2018	FP	8A	3	2018	Year-1	0	0	Spring
3/8/2018	FP	9A	3	2018	Year-1	0	0	Spring
3/8/2018	FP	11A	3	2018	Year-1	1	0	Spring
3/8/2018	FP	13A	3	2018	Year-1	1	0	Spring
3/9/2018	FP	1M	3	2018	Year-1	0	0	Spring
3/9/2018	FP	2M	3	2018	Year-1	1	0	Spring
3/9/2018	FP	3M	3	2018	Year-1	1	0	Spring
3/9/2018	FP	4M	3	2018	Year-1		1	Spring
3/9/2018	FP	5M	3	2018	Year-1		1	Spring
3/9/2018	FP	6M	3	2018	Year-1	0	0	Spring
3/9/2018	FP	7M	3	2018	Year-1	0	0	Spring
3/9/2018	FP	10M	3	2018	Year-1		1	Spring
3/9/2018	FP	12M	3	2018	Year-1	1	0	Spring

Date	Species	Site	Month	Year	Survey Year	Presence	Data Gap	Season
3/9/2018	FP	14M	3	2018	Year-1		1	Spring
3/9/2018	FP	8A	3	2018	Year-1	0	0	Spring
3/9/2018	FP	9A	3	2018	Year-1	0	0	Spring
3/9/2018	FP	11A	3	2018	Year-1	1	0	Spring
3/9/2018	FP	13A	3	2018	Year-1	1	0	Spring
3/10/2018	FP	1M	3	2018	Year-1	1	0	Spring
3/10/2018	FP	2M	3	2018	Year-1	0	0	Spring
3/10/2018	FP	3M	3	2018	Year-1	1	0	Spring
3/10/2018	FP	4M	3	2018	Year-1		1	Spring
3/10/2018	FP	5M	3	2018	Year-1		1	Spring
3/10/2018	FP	6M	3	2018	Year-1	0	0	Spring
3/10/2018	FP	7M	3	2018	Year-1	0	0	Spring
3/10/2018	FP	10M	3	2018	Year-1		1	Spring
3/10/2018	FP	12M	3	2018	Year-1	1	0	Spring
3/10/2018	FP	14M	3	2018	Year-1		1	Spring
3/10/2018	FP	8A	3	2018	Year-1	0	0	Spring
3/10/2018	FP	9A	3	2018	Year-1	0	0	Spring
3/10/2018	FP	11A	3	2018	Year-1	1	0	Spring
3/10/2018	FP	13A	3	2018	Year-1	1	0	Spring
3/11/2018	FP	1M	3	2018	Year-1	1	0	Spring
3/11/2018	FP	2M	3	2018	Year-1	1	0	Spring
3/11/2018	FP	3M	3	2018	Year-1	1	0	Spring
3/11/2018	FP	4M	3	2018	Year-1		1	Spring
3/11/2018	FP	5M	3	2018	Year-1		1	Spring
3/11/2018	FP	6M	3	2018	Year-1	0	0	Spring
3/11/2018	FP	7M	3	2018	Year-1	0	0	Spring
3/11/2018	FP	10M	3	2018	Year-1		1	Spring
3/11/2018	FP	12M	3	2018	Year-1	0	0	Spring
3/11/2018	FP	14M	3	2018	Year-1		1	Spring
3/11/2018	FP	8A	3	2018	Year-1	0	0	Spring
3/11/2018	FP	9A	3	2018	Year-1	0	0	Spring
3/11/2018	FP	11A	3	2018	Year-1	1	0	Spring
3/11/2018	FP	13A	3	2018	Year-1	1	0	Spring
3/12/2018	FP	1M	3	2018	Year-1	0	0	Spring
3/12/2018	FP	2M	3	2018	Year-1	0	0	Spring
3/12/2018	FP	3M	3	2018	Year-1	0	0	Spring
3/12/2018	FP	4M	3	2018	Year-1		1	Spring
3/12/2018	FP	5M	3	2018	Year-1		1	Spring
3/12/2018	FP	6M	3	2018	Year-1	0	0	Spring
3/12/2018	FP	7M	3	2018	Year-1	0	0	Spring
3/12/2018	FP	10M	3	2018	Year-1		1	Spring
3/12/2018	FP	12M	3	2018	Year-1	1	0	Spring
3/12/2018	FP	14M	3	2018	Year-1		1	Spring
3/12/2018	FP	8A	3	2018	Year-1	0	0	Spring

Date	Species	Site	Month	Year	Survey Year	Presence	Data Gap	Season
3/12/2018	FP	9A	3	2018	Year-1	0	0	Spring
3/12/2018	FP	11A	3	2018	Year-1	1	0	Spring
3/12/2018	FP	13A	3	2018	Year-1	1	0	Spring
3/13/2018	FP	1M	3	2018	Year-1	0	0	Spring
3/13/2018	FP	2M	3	2018	Year-1	0	0	Spring
3/13/2018	FP	3M	3	2018	Year-1	0	0	Spring
3/13/2018	FP	4M	3	2018	Year-1		1	Spring
3/13/2018	FP	5M	3	2018	Year-1		1	Spring
3/13/2018	FP	6M	3	2018	Year-1	0	0	Spring
3/13/2018	FP	7M	3	2018	Year-1	0	0	Spring
3/13/2018	FP	10M	3	2018	Year-1		1	Spring
3/13/2018	FP	12M	3	2018	Year-1		1	Spring
3/13/2018	FP	14M	3	2018	Year-1		1	Spring
3/13/2018	FP	8A	3	2018	Year-1	0	0	Spring
3/13/2018	FP	9A	3	2018	Year-1	0	0	Spring
3/13/2018	FP	11A	3	2018	Year-1	1	0	Spring
3/13/2018	FP	13A	3	2018	Year-1	1	0	Spring
3/14/2018	FP	1M	3	2018	Year-1	0	0	Spring
3/14/2018	FP	2M	3	2018	Year-1	1	0	Spring
3/14/2018	FP	3M	3	2018	Year-1	1	0	Spring
3/14/2018	FP	4M	3	2018	Year-1		1	Spring
3/14/2018	FP	5M	3	2018	Year-1		1	Spring
3/14/2018	FP	6M	3	2018	Year-1	0	0	Spring
3/14/2018	FP	7M	3	2018	Year-1	0	0	Spring
3/14/2018	FP	10M	3	2018	Year-1		1	Spring
3/14/2018	FP	12M	3	2018	Year-1		1	Spring
3/14/2018	FP	14M	3	2018	Year-1		1	Spring
3/14/2018	FP	8A	3	2018	Year-1	0	0	Spring
3/14/2018	FP	9A	3	2018	Year-1	0	0	Spring
3/14/2018	FP	11A	3	2018	Year-1	1	0	Spring
3/14/2018	FP	13A	3	2018	Year-1	1	0	Spring
3/15/2018	FP	1M	3	2018	Year-1	0	0	Spring
3/15/2018	FP	2M	3	2018	Year-1	1	0	Spring
3/15/2018	FP	3M	3	2018	Year-1	1	0	Spring
3/15/2018	FP	4M	3	2018	Year-1		1	Spring
3/15/2018	FP	5M	3	2018	Year-1		1	Spring
3/15/2018	FP	6M	3	2018	Year-1	0	0	Spring
3/15/2018	FP	7M	3	2018	Year-1	0	0	Spring
3/15/2018	FP	10M	3	2018	Year-1		1	Spring
3/15/2018	FP	12M	3	2018	Year-1		1	Spring
3/15/2018	FP	14M	3	2018	Year-1		1	Spring
3/15/2018	FP	8A	3	2018	Year-1	0	0	Spring
3/15/2018	FP	9A	3	2018	Year-1	0	0	Spring
3/15/2018	FP	11A	3	2018	Year-1	1	0	Spring

Date	Species	Site	Month	Year	Survey Year	Presence	Data Gap	Season
3/15/2018	FP	13A	3	2018	Year-1	1	0	Spring
3/16/2018	FP	1M	3	2018	Year-1	0	0	Spring
3/16/2018	FP	2M	3	2018	Year-1	1	0	Spring
3/16/2018	FP	3M	3	2018	Year-1	1	0	Spring
3/16/2018	FP	4M	3	2018	Year-1		1	Spring
3/16/2018	FP	5M	3	2018	Year-1		1	Spring
3/16/2018	FP	6M	3	2018	Year-1	1	0	Spring
3/16/2018	FP	7M	3	2018	Year-1	0	0	Spring
3/16/2018	FP	10M	3	2018	Year-1		1	Spring
3/16/2018	FP	12M	3	2018	Year-1		1	Spring
3/16/2018	FP	14M	3	2018	Year-1		1	Spring
3/16/2018	FP	8A	3	2018	Year-1	0	0	Spring
3/16/2018	FP	9A	3	2018	Year-1	0	0	Spring
3/16/2018	FP	11A	3	2018	Year-1	1	0	Spring
3/16/2018	FP	13A	3	2018	Year-1	1	0	Spring
3/17/2018	FP	1M	3	2018	Year-1	0	0	Spring
3/17/2018	FP	2M	3	2018	Year-1	1	0	Spring
3/17/2018	FP	3M	3	2018	Year-1	1	0	Spring
3/17/2018	FP	4M	3	2018	Year-1		1	Spring
3/17/2018	FP	5M	3	2018	Year-1		1	Spring
3/17/2018	FP	6M	3	2018	Year-1	0	0	Spring
3/17/2018	FP	7M	3	2018	Year-1	0	0	Spring
3/17/2018	FP	10M	3	2018	Year-1		1	Spring
3/17/2018	FP	12M	3	2018	Year-1		1	Spring
3/17/2018	FP	14M	3	2018	Year-1		1	Spring
3/17/2018	FP	8A	3	2018	Year-1	0	0	Spring
3/17/2018	FP	9A	3	2018	Year-1	0	0	Spring
3/17/2018	FP	11A	3	2018	Year-1	1	0	Spring
3/17/2018	FP	13A	3	2018	Year-1	1	0	Spring
3/18/2018	FP	1M	3	2018	Year-1	0	0	Spring
3/18/2018	FP	2M	3	2018	Year-1		1	Spring
3/18/2018	FP	3M	3	2018	Year-1	0	0	Spring
3/18/2018	FP	4M	3	2018	Year-1		1	Spring
3/18/2018	FP	5M	3	2018	Year-1		1	Spring
3/18/2018	FP	6M	3	2018	Year-1	0	0	Spring
3/18/2018	FP	7M	3	2018	Year-1	0	0	Spring
3/18/2018	FP	10M	3	2018	Year-1		1	Spring
3/18/2018	FP	12M	3	2018	Year-1		1	Spring
3/18/2018	FP	14M	3	2018	Year-1		1	Spring
3/18/2018	FP	8A	3	2018	Year-1	0	0	Spring
3/18/2018	FP	9A	3	2018	Year-1	0	0	Spring
3/18/2018	FP	11A	3	2018	Year-1	1	0	Spring
3/18/2018	FP	13A	3	2018	Year-1	1	0	Spring
3/19/2018	FP	1M	3	2018	Year-1		1	Spring

Date	Species	Site	Month	Year	Survey Year	Presence	Data Gap	Season
3/19/2018	FP	2M	3	2018	Year-1		1	Spring
3/19/2018	FP	3M	3	2018	Year-1		1	Spring
3/19/2018	FP	4M	3	2018	Year-1		1	Spring
3/19/2018	FP	5M	3	2018	Year-1		1	Spring
3/19/2018	FP	6M	3	2018	Year-1		1	Spring
3/19/2018	FP	7M	3	2018	Year-1		1	Spring
3/19/2018	FP	10M	3	2018	Year-1		1	Spring
3/19/2018	FP	12M	3	2018	Year-1		1	Spring
3/19/2018	FP	14M	3	2018	Year-1		1	Spring
3/19/2018	FP	8A	3	2018	Year-1	0	0	Spring
3/19/2018	FP	9A	3	2018	Year-1	0	0	Spring
3/19/2018	FP	11A	3	2018	Year-1	1	0	Spring
3/19/2018	FP	13A	3	2018	Year-1	1	0	Spring
3/20/2018	FP	1M	3	2018	Year-1		1	Spring
3/20/2018	FP	2M	3	2018	Year-1		1	Spring
3/20/2018	FP	3M	3	2018	Year-1		1	Spring
3/20/2018	FP	4M	3	2018	Year-1		1	Spring
3/20/2018	FP	5M	3	2018	Year-1		1	Spring
3/20/2018	FP	6M	3	2018	Year-1		1	Spring
3/20/2018	FP	7M	3	2018	Year-1		1	Spring
3/20/2018	FP	10M	3	2018	Year-1		1	Spring
3/20/2018	FP	12M	3	2018	Year-1		1	Spring
3/20/2018	FP	14M	3	2018	Year-1		1	Spring
3/20/2018	FP	8A	3	2018	Year-1	0	0	Spring
3/20/2018	FP	9A	3	2018	Year-1	0	0	Spring
3/20/2018	FP	11A	3	2018	Year-1	1	0	Spring
3/20/2018	FP	13A	3	2018	Year-1	1	0	Spring
3/21/2018	FP	1M	3	2018	Year-1		1	Spring
3/21/2018	FP	2M	3	2018	Year-1		1	Spring
3/21/2018	FP	3M	3	2018	Year-1		1	Spring
3/21/2018	FP	4M	3	2018	Year-1		1	Spring
3/21/2018	FP	5M	3	2018	Year-1		1	Spring
3/21/2018	FP	6M	3	2018	Year-1		1	Spring
3/21/2018	FP	7M	3	2018	Year-1		1	Spring
3/21/2018	FP	10M	3	2018	Year-1		1	Spring
3/21/2018	FP	12M	3	2018	Year-1		1	Spring
3/21/2018	FP	14M	3	2018	Year-1		1	Spring
3/21/2018	FP	8A	3	2018	Year-1	0	0	Spring
3/21/2018	FP	9A	3	2018	Year-1	0	0	Spring
3/21/2018	FP	11A	3	2018	Year-1	1	0	Spring
3/21/2018	FP	13A	3	2018	Year-1	1	0	Spring
3/22/2018	FP	1M	3	2018	Year-1		1	Spring
3/22/2018	FP	2M	3	2018	Year-1		1	Spring
3/22/2018	FP	3M	3	2018	Year-1		1	Spring

Date	Species	Site	Month	Year	Survey Year	Presence	Data Gap	Season
3/22/2018	FP	4M	3	2018	Year-1		1	Spring
3/22/2018	FP	5M	3	2018	Year-1		1	Spring
3/22/2018	FP	6M	3	2018	Year-1		1	Spring
3/22/2018	FP	7M	3	2018	Year-1		1	Spring
3/22/2018	FP	10M	3	2018	Year-1		1	Spring
3/22/2018	FP	12M	3	2018	Year-1		1	Spring
3/22/2018	FP	14M	3	2018	Year-1		1	Spring
3/22/2018	FP	8A	3	2018	Year-1	0	0	Spring
3/22/2018	FP	9A	3	2018	Year-1	0	0	Spring
3/22/2018	FP	11A	3	2018	Year-1	1	0	Spring
3/22/2018	FP	13A	3	2018	Year-1	1	0	Spring
3/23/2018	FP	1M	3	2018	Year-1		1	Spring
3/23/2018	FP	2M	3	2018	Year-1		1	Spring
3/23/2018	FP	3M	3	2018	Year-1		1	Spring
3/23/2018	FP	4M	3	2018	Year-1		1	Spring
3/23/2018	FP	5M	3	2018	Year-1		1	Spring
3/23/2018	FP	6M	3	2018	Year-1		1	Spring
3/23/2018	FP	7M	3	2018	Year-1		1	Spring
3/23/2018	FP	10M	3	2018	Year-1		1	Spring
3/23/2018	FP	12M	3	2018	Year-1		1	Spring
3/23/2018	FP	14M	3	2018	Year-1		1	Spring
3/23/2018	FP	8A	3	2018	Year-1	0	0	Spring
3/23/2018	FP	9A	3	2018	Year-1	0	0	Spring
3/23/2018	FP	11A	3	2018	Year-1	1	0	Spring
3/23/2018	FP	13A	3	2018	Year-1	1	0	Spring
3/24/2018	FP	1M	3	2018	Year-1		1	Spring
3/24/2018	FP	2M	3	2018	Year-1		1	Spring
3/24/2018	FP	3M	3	2018	Year-1		1	Spring
3/24/2018	FP	4M	3	2018	Year-1		1	Spring
3/24/2018	FP	5M	3	2018	Year-1		1	Spring
3/24/2018	FP	6M	3	2018	Year-1		1	Spring
3/24/2018	FP	7M	3	2018	Year-1		1	Spring
3/24/2018	FP	10M	3	2018	Year-1		1	Spring
3/24/2018	FP	12M	3	2018	Year-1		1	Spring
3/24/2018	FP	14M	3	2018	Year-1		1	Spring
3/24/2018	FP	8A	3	2018	Year-1	0	0	Spring
3/24/2018	FP	9A	3	2018	Year-1	0	0	Spring
3/24/2018	FP	11A	3	2018	Year-1	1	0	Spring
3/24/2018	FP	13A	3	2018	Year-1	1	0	Spring
3/25/2018	FP	1M	3	2018	Year-1		1	Spring
3/25/2018	FP	2M	3	2018	Year-1		1	Spring
3/25/2018	FP	3M	3	2018	Year-1		1	Spring
3/25/2018	FP	4M	3	2018	Year-1		1	Spring
3/25/2018	FP	5M	3	2018	Year-1		1	Spring

Date	Species	Site	Month	Year	Survey Year	Presence	Data Gap	Season
3/25/2018	FP	6M	3	2018	Year-1		1	Spring
3/25/2018	FP	7M	3	2018	Year-1		1	Spring
3/25/2018	FP	10M	3	2018	Year-1		1	Spring
3/25/2018	FP	12M	3	2018	Year-1		1	Spring
3/25/2018	FP	14M	3	2018	Year-1		1	Spring
3/25/2018	FP	8A	3	2018	Year-1	0	0	Spring
3/25/2018	FP	9A	3	2018	Year-1	1	0	Spring
3/25/2018	FP	11A	3	2018	Year-1	1	0	Spring
3/25/2018	FP	13A	3	2018	Year-1	1	0	Spring
3/26/2018	FP	1M	3	2018	Year-1		1	Spring
3/26/2018	FP	2M	3	2018	Year-1		1	Spring
3/26/2018	FP	3M	3	2018	Year-1		1	Spring
3/26/2018	FP	4M	3	2018	Year-1		1	Spring
3/26/2018	FP	5M	3	2018	Year-1		1	Spring
3/26/2018	FP	6M	3	2018	Year-1		1	Spring
3/26/2018	FP	7M	3	2018	Year-1		1	Spring
3/26/2018	FP	10M	3	2018	Year-1		1	Spring
3/26/2018	FP	12M	3	2018	Year-1		1	Spring
3/26/2018	FP	14M	3	2018	Year-1		1	Spring
3/26/2018	FP	8A	3	2018	Year-1	0	0	Spring
3/26/2018	FP	9A	3	2018	Year-1	0	0	Spring
3/26/2018	FP	11A	3	2018	Year-1	1	0	Spring
3/26/2018	FP	13A	3	2018	Year-1	1	0	Spring
3/27/2018	FP	1M	3	2018	Year-1		1	Spring
3/27/2018	FP	2M	3	2018	Year-1		1	Spring
3/27/2018	FP	3M	3	2018	Year-1		1	Spring
3/27/2018	FP	4M	3	2018	Year-1		1	Spring
3/27/2018	FP	5M	3	2018	Year-1		1	Spring
3/27/2018	FP	6M	3	2018	Year-1		1	Spring
3/27/2018	FP	7M	3	2018	Year-1		1	Spring
3/27/2018	FP	10M	3	2018	Year-1		1	Spring
3/27/2018	FP	12M	3	2018	Year-1		1	Spring
3/27/2018	FP	14M	3	2018	Year-1		1	Spring
3/27/2018	FP	8A	3	2018	Year-1	0	0	Spring
3/27/2018	FP	9A	3	2018	Year-1	0	0	Spring
3/27/2018	FP	11A	3	2018	Year-1	1	0	Spring
3/27/2018	FP	13A	3	2018	Year-1	1	0	Spring
3/28/2018	FP	1M	3	2018	Year-1		1	Spring
3/28/2018	FP	2M	3	2018	Year-1		1	Spring
3/28/2018	FP	3M	3	2018	Year-1		1	Spring
3/28/2018	FP	4M	3	2018	Year-1		1	Spring
3/28/2018	FP	5M	3	2018	Year-1		1	Spring
3/28/2018	FP	6M	3	2018	Year-1		1	Spring
3/28/2018	FP	7M	3	2018	Year-1		1	Spring

Date	Species	Site	Month	Year	Survey Year	Presence	Data Gap	Season
3/28/2018	FP	10M	3	2018	Year-1		1	Spring
3/28/2018	FP	12M	3	2018	Year-1		1	Spring
3/28/2018	FP	14M	3	2018	Year-1		1	Spring
3/28/2018	FP	8A	3	2018	Year-1	0	0	Spring
3/28/2018	FP	9A	3	2018	Year-1	0	0	Spring
3/28/2018	FP	11A	3	2018	Year-1	1	0	Spring
3/28/2018	FP	13A	3	2018	Year-1	1	0	Spring
3/29/2018	FP	1M	3	2018	Year-1		1	Spring
3/29/2018	FP	2M	3	2018	Year-1		1	Spring
3/29/2018	FP	3M	3	2018	Year-1		1	Spring
3/29/2018	FP	4M	3	2018	Year-1		1	Spring
3/29/2018	FP	5M	3	2018	Year-1		1	Spring
3/29/2018	FP	6M	3	2018	Year-1		1	Spring
3/29/2018	FP	7M	3	2018	Year-1		1	Spring
3/29/2018	FP	10M	3	2018	Year-1		1	Spring
3/29/2018	FP	12M	3	2018	Year-1		1	Spring
3/29/2018	FP	14M	3	2018	Year-1		1	Spring
3/29/2018	FP	8A	3	2018	Year-1	0	0	Spring
3/29/2018	FP	9A	3	2018	Year-1	0	0	Spring
3/29/2018	FP	11A	3	2018	Year-1	1	0	Spring
3/29/2018	FP	13A	3	2018	Year-1	1	0	Spring
3/30/2018	FP	1M	3	2018	Year-1		1	Spring
3/30/2018	FP	2M	3	2018	Year-1		1	Spring
3/30/2018	FP	3M	3	2018	Year-1		1	Spring
3/30/2018	FP	4M	3	2018	Year-1		1	Spring
3/30/2018	FP	5M	3	2018	Year-1		1	Spring
3/30/2018	FP	6M	3	2018	Year-1		1	Spring
3/30/2018	FP	7M	3	2018	Year-1		1	Spring
3/30/2018	FP	10M	3	2018	Year-1		1	Spring
3/30/2018	FP	12M	3	2018	Year-1		1	Spring
3/30/2018	FP	14M	3	2018	Year-1		1	Spring
3/30/2018	FP	8A	3	2018	Year-1	0	0	Spring
3/30/2018	FP	9A	3	2018	Year-1	0	0	Spring
3/30/2018	FP	11A	3	2018	Year-1	1	0	Spring
3/30/2018	FP	13A	3	2018	Year-1	1	0	Spring
3/31/2018	FP	1M	3	2018	Year-1		1	Spring
3/31/2018	FP	2M	3	2018	Year-1		1	Spring
3/31/2018	FP	3M	3	2018	Year-1		1	Spring
3/31/2018	FP	4M	3	2018	Year-1		1	Spring
3/31/2018	FP	5M	3	2018	Year-1		1	Spring
3/31/2018	FP	6M	3	2018	Year-1		1	Spring
3/31/2018	FP	7M	3	2018	Year-1		1	Spring
3/31/2018	FP	10M	3	2018	Year-1		1	Spring
3/31/2018	FP	12M	3	2018	Year-1		1	Spring

Date	Species	Site	Month	Year	Survey Year	Presence	Data Gap	Season
3/31/2018	FP	14M	3	2018	Year-1		1	Spring
3/31/2018	FP	8A	3	2018	Year-1	0	0	Spring
3/31/2018	FP	9A	3	2018	Year-1	0	0	Spring
3/31/2018	FP	11A	3	2018	Year-1	1	0	Spring
3/31/2018	FP	13A	3	2018	Year-1	1	0	Spring
4/1/2018	FP	1M	4	2018	Year-1		1	Spring
4/1/2018	FP	2M	4	2018	Year-1		1	Spring
4/1/2018	FP	3M	4	2018	Year-1		1	Spring
4/1/2018	FP	4M	4	2018	Year-1		1	Spring
4/1/2018	FP	5M	4	2018	Year-1		1	Spring
4/1/2018	FP	6M	4	2018	Year-1		1	Spring
4/1/2018	FP	7M	4	2018	Year-1		1	Spring
4/1/2018	FP	10M	4	2018	Year-1		1	Spring
4/1/2018	FP	12M	4	2018	Year-1		1	Spring
4/1/2018	FP	14M	4	2018	Year-1		1	Spring
4/1/2018	FP	8A	4	2018	Year-1	0	0	Spring
4/1/2018	FP	9A	4	2018	Year-1	0	0	Spring
4/1/2018	FP	11A	4	2018	Year-1	1	0	Spring
4/1/2018	FP	13A	4	2018	Year-1	1	0	Spring
4/2/2018	FP	1M	4	2018	Year-1		1	Spring
4/2/2018	FP	2M	4	2018	Year-1		1	Spring
4/2/2018	FP	3M	4	2018	Year-1		1	Spring
4/2/2018	FP	4M	4	2018	Year-1		1	Spring
4/2/2018	FP	5M	4	2018	Year-1		1	Spring
4/2/2018	FP	6M	4	2018	Year-1		1	Spring
4/2/2018	FP	7M	4	2018	Year-1		1	Spring
4/2/2018	FP	10M	4	2018	Year-1		1	Spring
4/2/2018	FP	12M	4	2018	Year-1		1	Spring
4/2/2018	FP	14M	4	2018	Year-1		1	Spring
4/2/2018	FP	8A	4	2018	Year-1	0	0	Spring
4/2/2018	FP	9A	4	2018	Year-1	0	0	Spring
4/2/2018	FP	11A	4	2018	Year-1	1	0	Spring
4/2/2018	FP	13A	4	2018	Year-1	1	0	Spring
4/3/2018	FP	1M	4	2018	Year-1		1	Spring
4/3/2018	FP	2M	4	2018	Year-1		1	Spring
4/3/2018	FP	3M	4	2018	Year-1		1	Spring
4/3/2018	FP	4M	4	2018	Year-1		1	Spring
4/3/2018	FP	5M	4	2018	Year-1		1	Spring
4/3/2018	FP	6M	4	2018	Year-1		1	Spring
4/3/2018	FP	7M	4	2018	Year-1		1	Spring
4/3/2018	FP	10M	4	2018	Year-1		1	Spring
4/3/2018	FP	12M	4	2018	Year-1		1	Spring
4/3/2018	FP	14M	4	2018	Year-1		1	Spring
4/3/2018	FP	8A	4	2018	Year-1	0	0	Spring

Date	Species	Site	Month	Year	Survey Year	Presence	Data Gap	Season
4/3/2018	FP	9A	4	2018	Year-1	1	0	Spring
4/3/2018	FP	11A	4	2018	Year-1	0	0	Spring
4/3/2018	FP	13A	4	2018	Year-1	1	0	Spring
4/4/2018	FP	1M	4	2018	Year-1		1	Spring
4/4/2018	FP	2M	4	2018	Year-1		1	Spring
4/4/2018	FP	3M	4	2018	Year-1		1	Spring
4/4/2018	FP	4M	4	2018	Year-1		1	Spring
4/4/2018	FP	5M	4	2018	Year-1		1	Spring
4/4/2018	FP	6M	4	2018	Year-1		1	Spring
4/4/2018	FP	7M	4	2018	Year-1		1	Spring
4/4/2018	FP	10M	4	2018	Year-1		1	Spring
4/4/2018	FP	12M	4	2018	Year-1		1	Spring
4/4/2018	FP	14M	4	2018	Year-1		1	Spring
4/4/2018	FP	8A	4	2018	Year-1	0	0	Spring
4/4/2018	FP	9A	4	2018	Year-1	1	0	Spring
4/4/2018	FP	11A	4	2018	Year-1	1	0	Spring
4/4/2018	FP	13A	4	2018	Year-1	1	0	Spring
4/5/2018	FP	1M	4	2018	Year-1		1	Spring
4/5/2018	FP	2M	4	2018	Year-1		1	Spring
4/5/2018	FP	3M	4	2018	Year-1		1	Spring
4/5/2018	FP	4M	4	2018	Year-1		1	Spring
4/5/2018	FP	5M	4	2018	Year-1		1	Spring
4/5/2018	FP	6M	4	2018	Year-1		1	Spring
4/5/2018	FP	7M	4	2018	Year-1		1	Spring
4/5/2018	FP	10M	4	2018	Year-1		1	Spring
4/5/2018	FP	12M	4	2018	Year-1		1	Spring
4/5/2018	FP	14M	4	2018	Year-1		1	Spring
4/5/2018	FP	8A	4	2018	Year-1	0	0	Spring
4/5/2018	FP	9A	4	2018	Year-1	0	0	Spring
4/5/2018	FP	11A	4	2018	Year-1	1	0	Spring
4/5/2018	FP	13A	4	2018	Year-1	1	0	Spring
4/6/2018	FP	1M	4	2018	Year-1		1	Spring
4/6/2018	FP	2M	4	2018	Year-1		1	Spring
4/6/2018	FP	3M	4	2018	Year-1		1	Spring
4/6/2018	FP	4M	4	2018	Year-1		1	Spring
4/6/2018	FP	5M	4	2018	Year-1		1	Spring
4/6/2018	FP	6M	4	2018	Year-1		1	Spring
4/6/2018	FP	7M	4	2018	Year-1		1	Spring
4/6/2018	FP	10M	4	2018	Year-1		1	Spring
4/6/2018	FP	12M	4	2018	Year-1		1	Spring
4/6/2018	FP	14M	4	2018	Year-1		1	Spring
4/6/2018	FP	8A	4	2018	Year-1	0	0	Spring
4/6/2018	FP	9A	4	2018	Year-1	1	0	Spring
4/6/2018	FP	11A	4	2018	Year-1	0	0	Spring

Date	Species	Site	Month	Year	Survey Year	Presence	Data Gap	Season
4/6/2018	FP	13A	4	2018	Year-1	1	0	Spring
4/7/2018	FP	1M	4	2018	Year-1		1	Spring
4/7/2018	FP	2M	4	2018	Year-1		1	Spring
4/7/2018	FP	3M	4	2018	Year-1		1	Spring
4/7/2018	FP	4M	4	2018	Year-1		1	Spring
4/7/2018	FP	5M	4	2018	Year-1		1	Spring
4/7/2018	FP	6M	4	2018	Year-1		1	Spring
4/7/2018	FP	7M	4	2018	Year-1		1	Spring
4/7/2018	FP	10M	4	2018	Year-1		1	Spring
4/7/2018	FP	12M	4	2018	Year-1		1	Spring
4/7/2018	FP	14M	4	2018	Year-1		1	Spring
4/7/2018	FP	8A	4	2018	Year-1	0	0	Spring
4/7/2018	FP	9A	4	2018	Year-1	1	0	Spring
4/7/2018	FP	11A	4	2018	Year-1	1	0	Spring
4/7/2018	FP	13A	4	2018	Year-1	1	0	Spring
4/8/2018	FP	1M	4	2018	Year-1		1	Spring
4/8/2018	FP	2M	4	2018	Year-1		1	Spring
4/8/2018	FP	3M	4	2018	Year-1		1	Spring
4/8/2018	FP	4M	4	2018	Year-1		1	Spring
4/8/2018	FP	5M	4	2018	Year-1		1	Spring
4/8/2018	FP	6M	4	2018	Year-1		1	Spring
4/8/2018	FP	7M	4	2018	Year-1		1	Spring
4/8/2018	FP	10M	4	2018	Year-1		1	Spring
4/8/2018	FP	12M	4	2018	Year-1		1	Spring
4/8/2018	FP	14M	4	2018	Year-1		1	Spring
4/8/2018	FP	8A	4	2018	Year-1	0	0	Spring
4/8/2018	FP	9A	4	2018	Year-1	0	0	Spring
4/8/2018	FP	11A	4	2018	Year-1	1	0	Spring
4/8/2018	FP	13A	4	2018	Year-1	1	0	Spring
4/9/2018	FP	1M	4	2018	Year-1		1	Spring
4/9/2018	FP	2M	4	2018	Year-1		1	Spring
4/9/2018	FP	3M	4	2018	Year-1		1	Spring
4/9/2018	FP	4M	4	2018	Year-1		1	Spring
4/9/2018	FP	5M	4	2018	Year-1		1	Spring
4/9/2018	FP	6M	4	2018	Year-1		1	Spring
4/9/2018	FP	7M	4	2018	Year-1		1	Spring
4/9/2018	FP	10M	4	2018	Year-1		1	Spring
4/9/2018	FP	12M	4	2018	Year-1		1	Spring
4/9/2018	FP	14M	4	2018	Year-1		1	Spring
4/9/2018	FP	8A	4	2018	Year-1	0	0	Spring
4/9/2018	FP	9A	4	2018	Year-1	0	0	Spring
4/9/2018	FP	11A	4	2018	Year-1	1	0	Spring
4/9/2018	FP	13A	4	2018	Year-1	1	0	Spring
4/10/2018	FP	1M	4	2018	Year-1		1	Spring

Date	Species	Site	Month	Year	Survey Year	Presence	Data Gap	Season
4/10/2018	FP	2M	4	2018	Year-1		1	Spring
4/10/2018	FP	3M	4	2018	Year-1		1	Spring
4/10/2018	FP	4M	4	2018	Year-1		1	Spring
4/10/2018	FP	5M	4	2018	Year-1		1	Spring
4/10/2018	FP	6M	4	2018	Year-1		1	Spring
4/10/2018	FP	7M	4	2018	Year-1		1	Spring
4/10/2018	FP	10M	4	2018	Year-1		1	Spring
4/10/2018	FP	12M	4	2018	Year-1		1	Spring
4/10/2018	FP	14M	4	2018	Year-1		1	Spring
4/10/2018	FP	8A	4	2018	Year-1	0	0	Spring
4/10/2018	FP	9A	4	2018	Year-1	0	0	Spring
4/10/2018	FP	11A	4	2018	Year-1	1	0	Spring
4/10/2018	FP	13A	4	2018	Year-1	1	0	Spring
4/11/2018	FP	1M	4	2018	Year-1		1	Spring
4/11/2018	FP	2M	4	2018	Year-1		1	Spring
4/11/2018	FP	3M	4	2018	Year-1		1	Spring
4/11/2018	FP	4M	4	2018	Year-1		1	Spring
4/11/2018	FP	5M	4	2018	Year-1		1	Spring
4/11/2018	FP	6M	4	2018	Year-1		1	Spring
4/11/2018	FP	7M	4	2018	Year-1		1	Spring
4/11/2018	FP	10M	4	2018	Year-1		1	Spring
4/11/2018	FP	12M	4	2018	Year-1		1	Spring
4/11/2018	FP	14M	4	2018	Year-1		1	Spring
4/11/2018	FP	8A	4	2018	Year-1	0	0	Spring
4/11/2018	FP	9A	4	2018	Year-1	0	0	Spring
4/11/2018	FP	11A	4	2018	Year-1	0	0	Spring
4/11/2018	FP	13A	4	2018	Year-1	1	0	Spring
4/12/2018	FP	1M	4	2018	Year-1	1	0	Spring
4/12/2018	FP	2M	4	2018	Year-1		1	Spring
4/12/2018	FP	3M	4	2018	Year-1		1	Spring
4/12/2018	FP	4M	4	2018	Year-1		1	Spring
4/12/2018	FP	5M	4	2018	Year-1	1	0	Spring
4/12/2018	FP	6M	4	2018	Year-1	1	0	Spring
4/12/2018	FP	7M	4	2018	Year-1	0	0	Spring
4/12/2018	FP	10M	4	2018	Year-1	1	0	Spring
4/12/2018	FP	12M	4	2018	Year-1	1	0	Spring
4/12/2018	FP	14M	4	2018	Year-1	1	0	Spring
4/12/2018	FP	8A	4	2018	Year-1	0	0	Spring
4/12/2018	FP	9A	4	2018	Year-1	0	0	Spring
4/12/2018	FP	11A	4	2018	Year-1	1	0	Spring
4/12/2018	FP	13A	4	2018	Year-1	1	0	Spring
4/13/2018	FP	1M	4	2018	Year-1	1	0	Spring
4/13/2018	FP	2M	4	2018	Year-1		1	Spring
4/13/2018	FP	3M	4	2018	Year-1		1	Spring

Date	Species	Site	Month	Year	Survey Year	Presence	Data Gap	Season
4/13/2018	FP	4M	4	2018	Year-1		1	Spring
4/13/2018	FP	5M	4	2018	Year-1	1	0	Spring
4/13/2018	FP	6M	4	2018	Year-1	1	0	Spring
4/13/2018	FP	7M	4	2018	Year-1	0	0	Spring
4/13/2018	FP	10M	4	2018	Year-1	1	0	Spring
4/13/2018	FP	12M	4	2018	Year-1	1	0	Spring
4/13/2018	FP	14M	4	2018	Year-1	1	0	Spring
4/13/2018	FP	8A	4	2018	Year-1	0	0	Spring
4/13/2018	FP	9A	4	2018	Year-1	0	0	Spring
4/13/2018	FP	11A	4	2018	Year-1	1	0	Spring
4/13/2018	FP	13A	4	2018	Year-1	1	0	Spring
4/14/2018	FP	1M	4	2018	Year-1	1	0	Spring
4/14/2018	FP	2M	4	2018	Year-1		1	Spring
4/14/2018	FP	3M	4	2018	Year-1		1	Spring
4/14/2018	FP	4M	4	2018	Year-1		1	Spring
4/14/2018	FP	5M	4	2018	Year-1	0	0	Spring
4/14/2018	FP	6M	4	2018	Year-1	0	0	Spring
4/14/2018	FP	7M	4	2018	Year-1	0	0	Spring
4/14/2018	FP	10M	4	2018	Year-1	0	0	Spring
4/14/2018	FP	12M	4	2018	Year-1	1	0	Spring
4/14/2018	FP	14M	4	2018	Year-1	1	0	Spring
4/14/2018	FP	8A	4	2018	Year-1	0	0	Spring
4/14/2018	FP	9A	4	2018	Year-1	0	0	Spring
4/14/2018	FP	11A	4	2018	Year-1	1	0	Spring
4/14/2018	FP	13A	4	2018	Year-1	1	0	Spring
4/15/2018	FP	1M	4	2018	Year-1	1	0	Spring
4/15/2018	FP	2M	4	2018	Year-1		1	Spring
4/15/2018	FP	3M	4	2018	Year-1		1	Spring
4/15/2018	FP	4M	4	2018	Year-1		1	Spring
4/15/2018	FP	5M	4	2018	Year-1		1	Spring
4/15/2018	FP	6M	4	2018	Year-1	1	0	Spring
4/15/2018	FP	7M	4	2018	Year-1	0	0	Spring
4/15/2018	FP	10M	4	2018	Year-1	1	0	Spring
4/15/2018	FP	12M	4	2018	Year-1	1	0	Spring
4/15/2018	FP	14M	4	2018	Year-1	0	0	Spring
4/15/2018	FP	8A	4	2018	Year-1	0	0	Spring
4/15/2018	FP	9A	4	2018	Year-1	0	0	Spring
4/15/2018	FP	11A	4	2018	Year-1	1	0	Spring
4/15/2018	FP	13A	4	2018	Year-1	1	0	Spring
4/16/2018	FP	1M	4	2018	Year-1	1	0	Spring
4/16/2018	FP	2M	4	2018	Year-1		1	Spring
4/16/2018	FP	3M	4	2018	Year-1		1	Spring
4/16/2018	FP	4M	4	2018	Year-1		1	Spring
4/16/2018	FP	5M	4	2018	Year-1		1	Spring

Date	Species	Site	Month	Year	Survey Year	Presence	Data Gap	Season
4/16/2018	FP	6M	4	2018	Year-1	1	0	Spring
4/16/2018	FP	7M	4	2018	Year-1	0	0	Spring
4/16/2018	FP	10M	4	2018	Year-1	1	0	Spring
4/16/2018	FP	12M	4	2018	Year-1	0	0	Spring
4/16/2018	FP	14M	4	2018	Year-1	1	0	Spring
4/16/2018	FP	8A	4	2018	Year-1	0	0	Spring
4/16/2018	FP	9A	4	2018	Year-1	0	0	Spring
4/16/2018	FP	11A	4	2018	Year-1	1	0	Spring
4/16/2018	FP	13A	4	2018	Year-1	1	0	Spring
4/17/2018	FP	1M	4	2018	Year-1	1	0	Spring
4/17/2018	FP	2M	4	2018	Year-1		1	Spring
4/17/2018	FP	3M	4	2018	Year-1		1	Spring
4/17/2018	FP	4M	4	2018	Year-1		1	Spring
4/17/2018	FP	5M	4	2018	Year-1		1	Spring
4/17/2018	FP	6M	4	2018	Year-1	0	0	Spring
4/17/2018	FP	7M	4	2018	Year-1	0	0	Spring
4/17/2018	FP	10M	4	2018	Year-1	1	0	Spring
4/17/2018	FP	12M	4	2018	Year-1	1	0	Spring
4/17/2018	FP	14M	4	2018	Year-1	1	0	Spring
4/17/2018	FP	8A	4	2018	Year-1	0	0	Spring
4/17/2018	FP	9A	4	2018	Year-1	0	0	Spring
4/17/2018	FP	11A	4	2018	Year-1	0	0	Spring
4/17/2018	FP	13A	4	2018	Year-1	1	0	Spring
4/18/2018	FP	1M	4	2018	Year-1	0	0	Spring
4/18/2018	FP	2M	4	2018	Year-1		1	Spring
4/18/2018	FP	3M	4	2018	Year-1		1	Spring
4/18/2018	FP	4M	4	2018	Year-1		1	Spring
4/18/2018	FP	5M	4	2018	Year-1		1	Spring
4/18/2018	FP	6M	4	2018	Year-1	0	0	Spring
4/18/2018	FP	7M	4	2018	Year-1	0	0	Spring
4/18/2018	FP	10M	4	2018	Year-1	0	0	Spring
4/18/2018	FP	12M	4	2018	Year-1	0	0	Spring
4/18/2018	FP	14M	4	2018	Year-1	1	0	Spring
4/18/2018	FP	8A	4	2018	Year-1	0	0	Spring
4/18/2018	FP	9A	4	2018	Year-1	0	0	Spring
4/18/2018	FP	11A	4	2018	Year-1	0	0	Spring
4/18/2018	FP	13A	4	2018	Year-1	1	0	Spring
4/19/2018	FP	1M	4	2018	Year-1	1	0	Spring
4/19/2018	FP	2M	4	2018	Year-1		1	Spring
4/19/2018	FP	3M	4	2018	Year-1		1	Spring
4/19/2018	FP	4M	4	2018	Year-1		1	Spring
4/19/2018	FP	5M	4	2018	Year-1		1	Spring
4/19/2018	FP	6M	4	2018	Year-1	0	0	Spring
4/19/2018	FP	7M	4	2018	Year-1	0	0	Spring

Date	Species	Site	Month	Year	Survey Year	Presence	Data Gap	Season
4/19/2018	FP	10M	4	2018	Year-1	1	0	Spring
4/19/2018	FP	12M	4	2018	Year-1	1	0	Spring
4/19/2018	FP	14M	4	2018	Year-1	0	0	Spring
4/19/2018	FP	8A	4	2018	Year-1	0	0	Spring
4/19/2018	FP	9A	4	2018	Year-1	1	0	Spring
4/19/2018	FP	11A	4	2018	Year-1	1	0	Spring
4/19/2018	FP	13A	4	2018	Year-1	1	0	Spring
4/20/2018	FP	1M	4	2018	Year-1	0	0	Spring
4/20/2018	FP	2M	4	2018	Year-1		1	Spring
4/20/2018	FP	3M	4	2018	Year-1		1	Spring
4/20/2018	FP	4M	4	2018	Year-1		1	Spring
4/20/2018	FP	5M	4	2018	Year-1		1	Spring
4/20/2018	FP	6M	4	2018	Year-1	0	0	Spring
4/20/2018	FP	7M	4	2018	Year-1	0	0	Spring
4/20/2018	FP	10M	4	2018	Year-1	1	0	Spring
4/20/2018	FP	12M	4	2018	Year-1	1	0	Spring
4/20/2018	FP	14M	4	2018	Year-1	1	0	Spring
4/20/2018	FP	8A	4	2018	Year-1	0	0	Spring
4/20/2018	FP	9A	4	2018	Year-1	0	0	Spring
4/20/2018	FP	11A	4	2018	Year-1	1	0	Spring
4/20/2018	FP	13A	4	2018	Year-1	1	0	Spring
4/21/2018	FP	1M	4	2018	Year-1	1	0	Spring
4/21/2018	FP	2M	4	2018	Year-1		1	Spring
4/21/2018	FP	3M	4	2018	Year-1		1	Spring
4/21/2018	FP	4M	4	2018	Year-1		1	Spring
4/21/2018	FP	5M	4	2018	Year-1		1	Spring
4/21/2018	FP	6M	4	2018	Year-1	0	0	Spring
4/21/2018	FP	7M	4	2018	Year-1	0	0	Spring
4/21/2018	FP	10M	4	2018	Year-1	1	0	Spring
4/21/2018	FP	12M	4	2018	Year-1	1	0	Spring
4/21/2018	FP	14M	4	2018	Year-1	1	0	Spring
4/21/2018	FP	8A	4	2018	Year-1	0	0	Spring
4/21/2018	FP	9A	4	2018	Year-1	1	0	Spring
4/21/2018	FP	11A	4	2018	Year-1	1	0	Spring
4/21/2018	FP	13A	4	2018	Year-1	1	0	Spring
4/22/2018	FP	1M	4	2018	Year-1	1	0	Spring
4/22/2018	FP	2M	4	2018	Year-1		1	Spring
4/22/2018	FP	3M	4	2018	Year-1		1	Spring
4/22/2018	FP	4M	4	2018	Year-1		1	Spring
4/22/2018	FP	5M	4	2018	Year-1		1	Spring
4/22/2018	FP	6M	4	2018	Year-1	0	0	Spring
4/22/2018	FP	7M	4	2018	Year-1	0	0	Spring
4/22/2018	FP	10M	4	2018	Year-1	1	0	Spring
4/22/2018	FP	12M	4	2018	Year-1	0	0	Spring

Date	Species	Site	Month	Year	Survey Year	Presence	Data Gap	Season
4/22/2018	FP	14M	4	2018	Year-1	1	0	Spring
4/22/2018	FP	8A	4	2018	Year-1	0	0	Spring
4/22/2018	FP	9A	4	2018	Year-1	0	0	Spring
4/22/2018	FP	11A	4	2018	Year-1	1	0	Spring
4/22/2018	FP	13A	4	2018	Year-1	1	0	Spring
4/23/2018	FP	1M	4	2018	Year-1	1	0	Spring
4/23/2018	FP	2M	4	2018	Year-1		1	Spring
4/23/2018	FP	3M	4	2018	Year-1		1	Spring
4/23/2018	FP	4M	4	2018	Year-1		1	Spring
4/23/2018	FP	5M	4	2018	Year-1		1	Spring
4/23/2018	FP	6M	4	2018	Year-1	1	0	Spring
4/23/2018	FP	7M	4	2018	Year-1	0	0	Spring
4/23/2018	FP	10M	4	2018	Year-1	0	0	Spring
4/23/2018	FP	12M	4	2018	Year-1	0	0	Spring
4/23/2018	FP	14M	4	2018	Year-1	1	0	Spring
4/23/2018	FP	8A	4	2018	Year-1	0	0	Spring
4/23/2018	FP	9A	4	2018	Year-1	0	0	Spring
4/23/2018	FP	11A	4	2018	Year-1	0	0	Spring
4/23/2018	FP	13A	4	2018	Year-1	0	0	Spring
4/24/2018	FP	1M	4	2018	Year-1	1	0	Spring
4/24/2018	FP	2M	4	2018	Year-1		1	Spring
4/24/2018	FP	3M	4	2018	Year-1		1	Spring
4/24/2018	FP	4M	4	2018	Year-1		1	Spring
4/24/2018	FP	5M	4	2018	Year-1		1	Spring
4/24/2018	FP	6M	4	2018	Year-1	0	0	Spring
4/24/2018	FP	7M	4	2018	Year-1	0	0	Spring
4/24/2018	FP	10M	4	2018	Year-1	0	0	Spring
4/24/2018	FP	12M	4	2018	Year-1	1	0	Spring
4/24/2018	FP	14M	4	2018	Year-1	1	0	Spring
4/24/2018	FP	8A	4	2018	Year-1	0	0	Spring
4/24/2018	FP	9A	4	2018	Year-1	0	0	Spring
4/24/2018	FP	11A	4	2018	Year-1	1	0	Spring
4/24/2018	FP	13A	4	2018	Year-1	0	0	Spring
4/25/2018	FP	1M	4	2018	Year-1	1	0	Spring
4/25/2018	FP	2M	4	2018	Year-1		1	Spring
4/25/2018	FP	3M	4	2018	Year-1		1	Spring
4/25/2018	FP	4M	4	2018	Year-1		1	Spring
4/25/2018	FP	5M	4	2018	Year-1		1	Spring
4/25/2018	FP	6M	4	2018	Year-1	0	0	Spring
4/25/2018	FP	7M	4	2018	Year-1	0	0	Spring
4/25/2018	FP	10M	4	2018	Year-1	1	0	Spring
4/25/2018	FP	12M	4	2018	Year-1	1	0	Spring
4/25/2018	FP	14M	4	2018	Year-1	1	0	Spring
4/25/2018	FP	8A	4	2018	Year-1	0	0	Spring

Date	Species	Site	Month	Year	Survey Year	Presence	Data Gap	Season
4/25/2018	FP	9A	4	2018	Year-1	0	0	Spring
4/25/2018	FP	11A	4	2018	Year-1	0	0	Spring
4/25/2018	FP	13A	4	2018	Year-1	1	0	Spring
4/26/2018	FP	1M	4	2018	Year-1	1	0	Spring
4/26/2018	FP	2M	4	2018	Year-1		1	Spring
4/26/2018	FP	3M	4	2018	Year-1		1	Spring
4/26/2018	FP	4M	4	2018	Year-1		1	Spring
4/26/2018	FP	5M	4	2018	Year-1		1	Spring
4/26/2018	FP	6M	4	2018	Year-1	0	0	Spring
4/26/2018	FP	7M	4	2018	Year-1	0	0	Spring
4/26/2018	FP	10M	4	2018	Year-1	0	0	Spring
4/26/2018	FP	12M	4	2018	Year-1	1	0	Spring
4/26/2018	FP	14M	4	2018	Year-1	1	0	Spring
4/26/2018	FP	8A	4	2018	Year-1	0	0	Spring
4/26/2018	FP	9A	4	2018	Year-1	0	0	Spring
4/26/2018	FP	11A	4	2018	Year-1	1	0	Spring
4/26/2018	FP	13A	4	2018	Year-1	1	0	Spring
4/27/2018	FP	1M	4	2018	Year-1	1	0	Spring
4/27/2018	FP	2M	4	2018	Year-1		1	Spring
4/27/2018	FP	3M	4	2018	Year-1		1	Spring
4/27/2018	FP	4M	4	2018	Year-1		1	Spring
4/27/2018	FP	5M	4	2018	Year-1		1	Spring
4/27/2018	FP	6M	4	2018	Year-1	0	0	Spring
4/27/2018	FP	7M	4	2018	Year-1	0	0	Spring
4/27/2018	FP	10M	4	2018	Year-1	0	0	Spring
4/27/2018	FP	12M	4	2018	Year-1	1	0	Spring
4/27/2018	FP	14M	4	2018	Year-1	1	0	Spring
4/27/2018	FP	8A	4	2018	Year-1	0	0	Spring
4/27/2018	FP	9A	4	2018	Year-1	0	0	Spring
4/27/2018	FP	11A	4	2018	Year-1	0	0	Spring
4/27/2018	FP	13A	4	2018	Year-1	1	0	Spring
4/28/2018	FP	1M	4	2018	Year-1	1	0	Spring
4/28/2018	FP	2M	4	2018	Year-1		1	Spring
4/28/2018	FP	3M	4	2018	Year-1		1	Spring
4/28/2018	FP	4M	4	2018	Year-1		1	Spring
4/28/2018	FP	5M	4	2018	Year-1		1	Spring
4/28/2018	FP	6M	4	2018	Year-1	0	0	Spring
4/28/2018	FP	7M	4	2018	Year-1	0	0	Spring
4/28/2018	FP	10M	4	2018	Year-1	1	0	Spring
4/28/2018	FP	12M	4	2018	Year-1	1	0	Spring
4/28/2018	FP	14M	4	2018	Year-1	1	0	Spring
4/28/2018	FP	8A	4	2018	Year-1	0	0	Spring
4/28/2018	FP	9A	4	2018	Year-1	0	0	Spring
4/28/2018	FP	11A	4	2018	Year-1	0	0	Spring

Date	Species	Site	Month	Year	Survey Year	Presence	Data Gap	Season
4/28/2018	FP	13A	4	2018	Year-1	1	0	Spring
4/29/2018	FP	1M	4	2018	Year-1	1	0	Spring
4/29/2018	FP	2M	4	2018	Year-1		1	Spring
4/29/2018	FP	3M	4	2018	Year-1		1	Spring
4/29/2018	FP	4M	4	2018	Year-1		1	Spring
4/29/2018	FP	5M	4	2018	Year-1		1	Spring
4/29/2018	FP	6M	4	2018	Year-1	0	0	Spring
4/29/2018	FP	7M	4	2018	Year-1	0	0	Spring
4/29/2018	FP	10M	4	2018	Year-1	0	0	Spring
4/29/2018	FP	12M	4	2018	Year-1	1	0	Spring
4/29/2018	FP	14M	4	2018	Year-1	1	0	Spring
4/29/2018	FP	8A	4	2018	Year-1	0	0	Spring
4/29/2018	FP	9A	4	2018	Year-1	0	0	Spring
4/29/2018	FP	11A	4	2018	Year-1	0	0	Spring
4/29/2018	FP	13A	4	2018	Year-1	1	0	Spring
4/30/2018	FP	1M	4	2018	Year-1	1	0	Spring
4/30/2018	FP	2M	4	2018	Year-1		1	Spring
4/30/2018	FP	3M	4	2018	Year-1		1	Spring
4/30/2018	FP	4M	4	2018	Year-1		1	Spring
4/30/2018	FP	5M	4	2018	Year-1		1	Spring
4/30/2018	FP	6M	4	2018	Year-1	0	0	Spring
4/30/2018	FP	7M	4	2018	Year-1	0	0	Spring
4/30/2018	FP	10M	4	2018	Year-1	0	0	Spring
4/30/2018	FP	12M	4	2018	Year-1	1	0	Spring
4/30/2018	FP	14M	4	2018	Year-1	1	0	Spring
4/30/2018	FP	8A	4	2018	Year-1	0	0	Spring
4/30/2018	FP	9A	4	2018	Year-1	0	0	Spring
4/30/2018	FP	11A	4	2018	Year-1	0	0	Spring
4/30/2018	FP	13A	4	2018	Year-1	1	0	Spring
5/1/2018	FP	1M	5	2018	Year-1	1	0	Spring
5/1/2018	FP	2M	5	2018	Year-1		1	Spring
5/1/2018	FP	3M	5	2018	Year-1		1	Spring
5/1/2018	FP	4M	5	2018	Year-1		1	Spring
5/1/2018	FP	5M	5	2018	Year-1		1	Spring
5/1/2018	FP	6M	5	2018	Year-1	0	0	Spring
5/1/2018	FP	7M	5	2018	Year-1	0	0	Spring
5/1/2018	FP	10M	5	2018	Year-1	0	0	Spring
5/1/2018	FP	12M	5	2018	Year-1	1	0	Spring
5/1/2018	FP	14M	5	2018	Year-1	1	0	Spring
5/1/2018	FP	8A	5	2018	Year-1	0	0	Spring
5/1/2018	FP	9A	5	2018	Year-1	0	0	Spring
5/1/2018	FP	11A	5	2018	Year-1	0	0	Spring
5/1/2018	FP	13A	5	2018	Year-1	1	0	Spring
5/2/2018	FP	1M	5	2018	Year-1	1	0	Spring

Date	Species	Site	Month	Year	Survey Year	Presence	Data Gap	Season
5/2/2018	FP	2M	5	2018	Year-1		1	Spring
5/2/2018	FP	3M	5	2018	Year-1		1	Spring
5/2/2018	FP	4M	5	2018	Year-1		1	Spring
5/2/2018	FP	5M	5	2018	Year-1		1	Spring
5/2/2018	FP	6M	5	2018	Year-1	0	0	Spring
5/2/2018	FP	7M	5	2018	Year-1	0	0	Spring
5/2/2018	FP	10M	5	2018	Year-1	0	0	Spring
5/2/2018	FP	12M	5	2018	Year-1	1	0	Spring
5/2/2018	FP	14M	5	2018	Year-1	1	0	Spring
5/2/2018	FP	8A	5	2018	Year-1	0	0	Spring
5/2/2018	FP	9A	5	2018	Year-1	0	0	Spring
5/2/2018	FP	11A	5	2018	Year-1	1	0	Spring
5/2/2018	FP	13A	5	2018	Year-1	1	0	Spring
5/3/2018	FP	1M	5	2018	Year-1	1	0	Spring
5/3/2018	FP	2M	5	2018	Year-1		1	Spring
5/3/2018	FP	3M	5	2018	Year-1		1	Spring
5/3/2018	FP	4M	5	2018	Year-1		1	Spring
5/3/2018	FP	5M	5	2018	Year-1		1	Spring
5/3/2018	FP	6M	5	2018	Year-1	0	0	Spring
5/3/2018	FP	7M	5	2018	Year-1	0	0	Spring
5/3/2018	FP	10M	5	2018	Year-1	0	0	Spring
5/3/2018	FP	12M	5	2018	Year-1	0	0	Spring
5/3/2018	FP	14M	5	2018	Year-1	1	0	Spring
5/3/2018	FP	8A	5	2018	Year-1	0	0	Spring
5/3/2018	FP	9A	5	2018	Year-1	0	0	Spring
5/3/2018	FP	11A	5	2018	Year-1	0	0	Spring
5/3/2018	FP	13A	5	2018	Year-1	1	0	Spring
5/4/2018	FP	1M	5	2018	Year-1	1	0	Spring
5/4/2018	FP	2M	5	2018	Year-1		1	Spring
5/4/2018	FP	3M	5	2018	Year-1		1	Spring
5/4/2018	FP	4M	5	2018	Year-1		1	Spring
5/4/2018	FP	5M	5	2018	Year-1		1	Spring
5/4/2018	FP	6M	5	2018	Year-1	0	0	Spring
5/4/2018	FP	7M	5	2018	Year-1	0	0	Spring
5/4/2018	FP	10M	5	2018	Year-1	0	0	Spring
5/4/2018	FP	12M	5	2018	Year-1	0	0	Spring
5/4/2018	FP	14M	5	2018	Year-1	0	0	Spring
5/4/2018	FP	8A	5	2018	Year-1	0	0	Spring
5/4/2018	FP	9A	5	2018	Year-1	0	0	Spring
5/4/2018	FP	11A	5	2018	Year-1	0	0	Spring
5/4/2018	FP	13A	5	2018	Year-1	0	0	Spring
5/5/2018	FP	1M	5	2018	Year-1	1	0	Spring
5/5/2018	FP	2M	5	2018	Year-1		1	Spring
5/5/2018	FP	3M	5	2018	Year-1		1	Spring

Date	Species	Site	Month	Year	Survey Year	Presence	Data Gap	Season
5/5/2018	FP	4M	5	2018	Year-1		1	Spring
5/5/2018	FP	5M	5	2018	Year-1		1	Spring
5/5/2018	FP	6M	5	2018	Year-1	0	0	Spring
5/5/2018	FP	7M	5	2018	Year-1	0	0	Spring
5/5/2018	FP	10M	5	2018	Year-1	0	0	Spring
5/5/2018	FP	12M	5	2018	Year-1	0	0	Spring
5/5/2018	FP	14M	5	2018	Year-1	1	0	Spring
5/5/2018	FP	8A	5	2018	Year-1	0	0	Spring
5/5/2018	FP	9A	5	2018	Year-1	0	0	Spring
5/5/2018	FP	11A	5	2018	Year-1	0	0	Spring
5/5/2018	FP	13A	5	2018	Year-1	1	0	Spring
5/6/2018	FP	1M	5	2018	Year-1	0	0	Spring
5/6/2018	FP	2M	5	2018	Year-1		1	Spring
5/6/2018	FP	3M	5	2018	Year-1		1	Spring
5/6/2018	FP	4M	5	2018	Year-1		1	Spring
5/6/2018	FP	5M	5	2018	Year-1		1	Spring
5/6/2018	FP	6M	5	2018	Year-1	0	0	Spring
5/6/2018	FP	7M	5	2018	Year-1	0	0	Spring
5/6/2018	FP	10M	5	2018	Year-1	0	0	Spring
5/6/2018	FP	12M	5	2018	Year-1	0	0	Spring
5/6/2018	FP	14M	5	2018	Year-1	0	0	Spring
5/6/2018	FP	8A	5	2018	Year-1	0	0	Spring
5/6/2018	FP	9A	5	2018	Year-1	0	0	Spring
5/6/2018	FP	11A	5	2018	Year-1	0	0	Spring
5/6/2018	FP	13A	5	2018	Year-1	0	0	Spring
5/7/2018	FP	1M	5	2018	Year-1	0	0	Spring
5/7/2018	FP	2M	5	2018	Year-1		1	Spring
5/7/2018	FP	3M	5	2018	Year-1		1	Spring
5/7/2018	FP	4M	5	2018	Year-1		1	Spring
5/7/2018	FP	5M	5	2018	Year-1		1	Spring
5/7/2018	FP	6M	5	2018	Year-1	0	0	Spring
5/7/2018	FP	7M	5	2018	Year-1	0	0	Spring
5/7/2018	FP	10M	5	2018	Year-1	0	0	Spring
5/7/2018	FP	12M	5	2018	Year-1	0	0	Spring
5/7/2018	FP	14M	5	2018	Year-1	0	0	Spring
5/7/2018	FP	8A	5	2018	Year-1	0	0	Spring
5/7/2018	FP	9A	5	2018	Year-1	0	0	Spring
5/7/2018	FP	11A	5	2018	Year-1	0	0	Spring
5/7/2018	FP	13A	5	2018	Year-1	0	0	Spring
5/8/2018	FP	1M	5	2018	Year-1	1	0	Spring
5/8/2018	FP	2M	5	2018	Year-1		1	Spring
5/8/2018	FP	3M	5	2018	Year-1		1	Spring
5/8/2018	FP	4M	5	2018	Year-1		1	Spring
5/8/2018	FP	5M	5	2018	Year-1		1	Spring

Date	Species	Site	Month	Year	Survey Year	Presence	Data Gap	Season
5/8/2018	FP	6M	5	2018	Year-1	0	0	Spring
5/8/2018	FP	7M	5	2018	Year-1	0	0	Spring
5/8/2018	FP	10M	5	2018	Year-1	0	0	Spring
5/8/2018	FP	12M	5	2018	Year-1	0	0	Spring
5/8/2018	FP	14M	5	2018	Year-1	1	0	Spring
5/8/2018	FP	8A	5	2018	Year-1	0	0	Spring
5/8/2018	FP	9A	5	2018	Year-1	0	0	Spring
5/8/2018	FP	11A	5	2018	Year-1	0	0	Spring
5/8/2018	FP	13A	5	2018	Year-1	1	0	Spring
5/9/2018	FP	1M	5	2018	Year-1	0	0	Spring
5/9/2018	FP	2M	5	2018	Year-1		1	Spring
5/9/2018	FP	3M	5	2018	Year-1		1	Spring
5/9/2018	FP	4M	5	2018	Year-1		1	Spring
5/9/2018	FP	5M	5	2018	Year-1		1	Spring
5/9/2018	FP	6M	5	2018	Year-1	0	0	Spring
5/9/2018	FP	7M	5	2018	Year-1	0	0	Spring
5/9/2018	FP	10M	5	2018	Year-1	0	0	Spring
5/9/2018	FP	12M	5	2018	Year-1	1	0	Spring
5/9/2018	FP	14M	5	2018	Year-1	1	0	Spring
5/9/2018	FP	8A	5	2018	Year-1	0	0	Spring
5/9/2018	FP	9A	5	2018	Year-1	0	0	Spring
5/9/2018	FP	11A	5	2018	Year-1	0	0	Spring
5/9/2018	FP	13A	5	2018	Year-1	1	0	Spring
5/10/2018	FP	1M	5	2018	Year-1	1	0	Spring
5/10/2018	FP	2M	5	2018	Year-1		1	Spring
5/10/2018	FP	3M	5	2018	Year-1		1	Spring
5/10/2018	FP	4M	5	2018	Year-1		1	Spring
5/10/2018	FP	5M	5	2018	Year-1		1	Spring
5/10/2018	FP	6M	5	2018	Year-1	0	0	Spring
5/10/2018	FP	7M	5	2018	Year-1	0	0	Spring
5/10/2018	FP	10M	5	2018	Year-1	0	0	Spring
5/10/2018	FP	12M	5	2018	Year-1	1	0	Spring
5/10/2018	FP	14M	5	2018	Year-1	1	0	Spring
5/10/2018	FP	8A	5	2018	Year-1	0	0	Spring
5/10/2018	FP	9A	5	2018	Year-1	0	0	Spring
5/10/2018	FP	11A	5	2018	Year-1	0	0	Spring
5/10/2018	FP	13A	5	2018	Year-1	1	0	Spring
5/11/2018	FP	1M	5	2018	Year-1	0	0	Spring
5/11/2018	FP	2M	5	2018	Year-1		1	Spring
5/11/2018	FP	3M	5	2018	Year-1		1	Spring
5/11/2018	FP	4M	5	2018	Year-1		1	Spring
5/11/2018	FP	5M	5	2018	Year-1		1	Spring
5/11/2018	FP	6M	5	2018	Year-1	0	0	Spring
5/11/2018	FP	7M	5	2018	Year-1	0	0	Spring

Date	Species	Site	Month	Year	Survey Year	Presence	Data Gap	Season
5/11/2018	FP	10M	5	2018	Year-1	0	0	Spring
5/11/2018	FP	12M	5	2018	Year-1	0	0	Spring
5/11/2018	FP	14M	5	2018	Year-1	1	0	Spring
5/11/2018	FP	8A	5	2018	Year-1	0	0	Spring
5/11/2018	FP	9A	5	2018	Year-1	0	0	Spring
5/11/2018	FP	11A	5	2018	Year-1	0	0	Spring
5/11/2018	FP	13A	5	2018	Year-1	0	0	Spring
5/12/2018	FP	1M	5	2018	Year-1	1	0	Spring
5/12/2018	FP	2M	5	2018	Year-1		1	Spring
5/12/2018	FP	3M	5	2018	Year-1		1	Spring
5/12/2018	FP	4M	5	2018	Year-1		1	Spring
5/12/2018	FP	5M	5	2018	Year-1		1	Spring
5/12/2018	FP	6M	5	2018	Year-1	0	0	Spring
5/12/2018	FP	7M	5	2018	Year-1	0	0	Spring
5/12/2018	FP	10M	5	2018	Year-1	0	0	Spring
5/12/2018	FP	12M	5	2018	Year-1	0	0	Spring
5/12/2018	FP	14M	5	2018	Year-1	1	0	Spring
5/12/2018	FP	8A	5	2018	Year-1	0	0	Spring
5/12/2018	FP	9A	5	2018	Year-1	0	0	Spring
5/12/2018	FP	11A	5	2018	Year-1	0	0	Spring
5/12/2018	FP	13A	5	2018	Year-1	0	0	Spring
5/13/2018	FP	1M	5	2018	Year-1	1	0	Spring
5/13/2018	FP	2M	5	2018	Year-1		1	Spring
5/13/2018	FP	3M	5	2018	Year-1		1	Spring
5/13/2018	FP	4M	5	2018	Year-1		1	Spring
5/13/2018	FP	5M	5	2018	Year-1		1	Spring
5/13/2018	FP	6M	5	2018	Year-1	0	0	Spring
5/13/2018	FP	7M	5	2018	Year-1	0	0	Spring
5/13/2018	FP	10M	5	2018	Year-1	0	0	Spring
5/13/2018	FP	12M	5	2018	Year-1	0	0	Spring
5/13/2018	FP	14M	5	2018	Year-1	1	0	Spring
5/13/2018	FP	8A	5	2018	Year-1	0	0	Spring
5/13/2018	FP	9A	5	2018	Year-1	0	0	Spring
5/13/2018	FP	11A	5	2018	Year-1	0	0	Spring
5/13/2018	FP	13A	5	2018	Year-1	0	0	Spring
5/14/2018	FP	1M	5	2018	Year-1	0	0	Spring
5/14/2018	FP	2M	5	2018	Year-1		1	Spring
5/14/2018	FP	3M	5	2018	Year-1		1	Spring
5/14/2018	FP	4M	5	2018	Year-1		1	Spring
5/14/2018	FP	5M	5	2018	Year-1		1	Spring
5/14/2018	FP	6M	5	2018	Year-1	0	0	Spring
5/14/2018	FP	7M	5	2018	Year-1	0	0	Spring
5/14/2018	FP	10M	5	2018	Year-1	0	0	Spring
5/14/2018	FP	12M	5	2018	Year-1	0	0	Spring

Date	Species	Site	Month	Year	Survey Year	Presence	Data Gap	Season
5/14/2018	FP	14M	5	2018	Year-1	1	0	Spring
5/14/2018	FP	8A	5	2018	Year-1	0	0	Spring
5/14/2018	FP	9A	5	2018	Year-1	0	0	Spring
5/14/2018	FP	11A	5	2018	Year-1	0	0	Spring
5/14/2018	FP	13A	5	2018	Year-1	1	0	Spring
5/15/2018	FP	1M	5	2018	Year-1	0	0	Spring
5/15/2018	FP	2M	5	2018	Year-1		1	Spring
5/15/2018	FP	3M	5	2018	Year-1		1	Spring
5/15/2018	FP	4M	5	2018	Year-1		1	Spring
5/15/2018	FP	5M	5	2018	Year-1		1	Spring
5/15/2018	FP	6M	5	2018	Year-1	0	0	Spring
5/15/2018	FP	7M	5	2018	Year-1	0	0	Spring
5/15/2018	FP	10M	5	2018	Year-1	0	0	Spring
5/15/2018	FP	12M	5	2018	Year-1	0	0	Spring
5/15/2018	FP	14M	5	2018	Year-1	1	0	Spring
5/15/2018	FP	8A	5	2018	Year-1	0	0	Spring
5/15/2018	FP	9A	5	2018	Year-1	0	0	Spring
5/15/2018	FP	11A	5	2018	Year-1	0	0	Spring
5/15/2018	FP	13A	5	2018	Year-1	0	0	Spring
5/16/2018	FP	1M	5	2018	Year-1	0	0	Spring
5/16/2018	FP	2M	5	2018	Year-1		1	Spring
5/16/2018	FP	3M	5	2018	Year-1		1	Spring
5/16/2018	FP	4M	5	2018	Year-1		1	Spring
5/16/2018	FP	5M	5	2018	Year-1		1	Spring
5/16/2018	FP	6M	5	2018	Year-1	0	0	Spring
5/16/2018	FP	7M	5	2018	Year-1	0	0	Spring
5/16/2018	FP	10M	5	2018	Year-1	0	0	Spring
5/16/2018	FP	12M	5	2018	Year-1	1	0	Spring
5/16/2018	FP	14M	5	2018	Year-1	1	0	Spring
5/16/2018	FP	8A	5	2018	Year-1	0	0	Spring
5/16/2018	FP	9A	5	2018	Year-1	0	0	Spring
5/16/2018	FP	11A	5	2018	Year-1	0	0	Spring
5/16/2018	FP	13A	5	2018	Year-1	1	0	Spring
5/17/2018	FP	1M	5	2018	Year-1	0	0	Spring
5/17/2018	FP	2M	5	2018	Year-1		1	Spring
5/17/2018	FP	3M	5	2018	Year-1		1	Spring
5/17/2018	FP	4M	5	2018	Year-1		1	Spring
5/17/2018	FP	5M	5	2018	Year-1		1	Spring
5/17/2018	FP	6M	5	2018	Year-1	0	0	Spring
5/17/2018	FP	7M	5	2018	Year-1	0	0	Spring
5/17/2018	FP	10M	5	2018	Year-1	0	0	Spring
5/17/2018	FP	12M	5	2018	Year-1	1	0	Spring
5/17/2018	FP	14M	5	2018	Year-1	1	0	Spring
5/17/2018	FP	8A	5	2018	Year-1	0	0	Spring

Date	Species	Site	Month	Year	Survey Year	Presence	Data Gap	Season
5/17/2018	FP	9A	5	2018	Year-1	0	0	Spring
5/17/2018	FP	11A	5	2018	Year-1	0	0	Spring
5/17/2018	FP	13A	5	2018	Year-1	1	0	Spring
5/18/2018	FP	1M	5	2018	Year-1	0	0	Spring
5/18/2018	FP	2M	5	2018	Year-1		1	Spring
5/18/2018	FP	3M	5	2018	Year-1		1	Spring
5/18/2018	FP	4M	5	2018	Year-1		1	Spring
5/18/2018	FP	5M	5	2018	Year-1		1	Spring
5/18/2018	FP	6M	5	2018	Year-1	0	0	Spring
5/18/2018	FP	7M	5	2018	Year-1	0	0	Spring
5/18/2018	FP	10M	5	2018	Year-1	0	0	Spring
5/18/2018	FP	12M	5	2018	Year-1	0	0	Spring
5/18/2018	FP	14M	5	2018	Year-1	1	0	Spring
5/18/2018	FP	8A	5	2018	Year-1	0	0	Spring
5/18/2018	FP	9A	5	2018	Year-1	0	0	Spring
5/18/2018	FP	11A	5	2018	Year-1	0	0	Spring
5/18/2018	FP	13A	5	2018	Year-1	1	0	Spring
5/19/2018	FP	1M	5	2018	Year-1	0	0	Spring
5/19/2018	FP	2M	5	2018	Year-1		1	Spring
5/19/2018	FP	3M	5	2018	Year-1		1	Spring
5/19/2018	FP	4M	5	2018	Year-1		1	Spring
5/19/2018	FP	5M	5	2018	Year-1		1	Spring
5/19/2018	FP	6M	5	2018	Year-1	0	0	Spring
5/19/2018	FP	7M	5	2018	Year-1	0	0	Spring
5/19/2018	FP	10M	5	2018	Year-1	0	0	Spring
5/19/2018	FP	12M	5	2018	Year-1	1	0	Spring
5/19/2018	FP	14M	5	2018	Year-1	1	0	Spring
5/19/2018	FP	8A	5	2018	Year-1	0	0	Spring
5/19/2018	FP	9A	5	2018	Year-1	0	0	Spring
5/19/2018	FP	11A	5	2018	Year-1	0	0	Spring
5/19/2018	FP	13A	5	2018	Year-1	1	0	Spring
5/20/2018	FP	1M	5	2018	Year-1	0	0	Spring
5/20/2018	FP	2M	5	2018	Year-1		1	Spring
5/20/2018	FP	3M	5	2018	Year-1		1	Spring
5/20/2018	FP	4M	5	2018	Year-1		1	Spring
5/20/2018	FP	5M	5	2018	Year-1		1	Spring
5/20/2018	FP	6M	5	2018	Year-1	0	0	Spring
5/20/2018	FP	7M	5	2018	Year-1	0	0	Spring
5/20/2018	FP	10M	5	2018	Year-1	0	0	Spring
5/20/2018	FP	12M	5	2018	Year-1	1	0	Spring
5/20/2018	FP	14M	5	2018	Year-1	1	0	Spring
5/20/2018	FP	8A	5	2018	Year-1	0	0	Spring
5/20/2018	FP	9A	5	2018	Year-1	0	0	Spring
5/20/2018	FP	11A	5	2018	Year-1	1	0	Spring

Date	Species	Site	Month	Year	Survey Year	Presence	Data Gap	Season
5/20/2018	FP	13A	5	2018	Year-1	1	0	Spring
5/21/2018	FP	1M	5	2018	Year-1	0	0	Spring
5/21/2018	FP	2M	5	2018	Year-1		1	Spring
5/21/2018	FP	3M	5	2018	Year-1		1	Spring
5/21/2018	FP	4M	5	2018	Year-1		1	Spring
5/21/2018	FP	5M	5	2018	Year-1		1	Spring
5/21/2018	FP	6M	5	2018	Year-1	0	0	Spring
5/21/2018	FP	7M	5	2018	Year-1	0	0	Spring
5/21/2018	FP	10M	5	2018	Year-1	0	0	Spring
5/21/2018	FP	12M	5	2018	Year-1	1	0	Spring
5/21/2018	FP	14M	5	2018	Year-1	1	0	Spring
5/21/2018	FP	8A	5	2018	Year-1	0	0	Spring
5/21/2018	FP	9A	5	2018	Year-1	0	0	Spring
5/21/2018	FP	11A	5	2018	Year-1	1	0	Spring
5/21/2018	FP	13A	5	2018	Year-1	1	0	Spring
5/22/2018	FP	1M	5	2018	Year-1	0	0	Spring
5/22/2018	FP	2M	5	2018	Year-1		1	Spring
5/22/2018	FP	3M	5	2018	Year-1		1	Spring
5/22/2018	FP	4M	5	2018	Year-1		1	Spring
5/22/2018	FP	5M	5	2018	Year-1		1	Spring
5/22/2018	FP	6M	5	2018	Year-1	0	0	Spring
5/22/2018	FP	7M	5	2018	Year-1	0	0	Spring
5/22/2018	FP	10M	5	2018	Year-1	1	0	Spring
5/22/2018	FP	12M	5	2018	Year-1	1	0	Spring
5/22/2018	FP	14M	5	2018	Year-1	1	0	Spring
5/22/2018	FP	8A	5	2018	Year-1	0	0	Spring
5/22/2018	FP	9A	5	2018	Year-1	0	0	Spring
5/22/2018	FP	11A	5	2018	Year-1	1	0	Spring
5/22/2018	FP	13A	5	2018	Year-1	1	0	Spring
5/23/2018	FP	1M	5	2018	Year-1	1	0	Spring
5/23/2018	FP	2M	5	2018	Year-1		1	Spring
5/23/2018	FP	3M	5	2018	Year-1		1	Spring
5/23/2018	FP	4M	5	2018	Year-1		1	Spring
5/23/2018	FP	5M	5	2018	Year-1		1	Spring
5/23/2018	FP	6M	5	2018	Year-1	0	0	Spring
5/23/2018	FP	7M	5	2018	Year-1	0	0	Spring
5/23/2018	FP	10M	5	2018	Year-1	0	0	Spring
5/23/2018	FP	12M	5	2018	Year-1	1	0	Spring
5/23/2018	FP	14M	5	2018	Year-1	1	0	Spring
5/23/2018	FP	8A	5	2018	Year-1	0	0	Spring
5/23/2018	FP	9A	5	2018	Year-1	0	0	Spring
5/23/2018	FP	11A	5	2018	Year-1	1	0	Spring
5/23/2018	FP	13A	5	2018	Year-1	0	0	Spring
5/24/2018	FP	1M	5	2018	Year-1	0	0	Spring

Date	Species	Site	Month	Year	Survey Year	Presence	Data Gap	Season
5/24/2018	FP	2M	5	2018	Year-1		1	Spring
5/24/2018	FP	3M	5	2018	Year-1		1	Spring
5/24/2018	FP	4M	5	2018	Year-1		1	Spring
5/24/2018	FP	5M	5	2018	Year-1		1	Spring
5/24/2018	FP	6M	5	2018	Year-1	0	0	Spring
5/24/2018	FP	7M	5	2018	Year-1	0	0	Spring
5/24/2018	FP	10M	5	2018	Year-1	0	0	Spring
5/24/2018	FP	12M	5	2018	Year-1	1	0	Spring
5/24/2018	FP	14M	5	2018	Year-1	1	0	Spring
5/24/2018	FP	8A	5	2018	Year-1	0	0	Spring
5/24/2018	FP	9A	5	2018	Year-1	0	0	Spring
5/24/2018	FP	11A	5	2018	Year-1	1	0	Spring
5/24/2018	FP	13A	5	2018	Year-1	0	0	Spring
5/25/2018	FP	1M	5	2018	Year-1	1	0	Spring
5/25/2018	FP	2M	5	2018	Year-1		1	Spring
5/25/2018	FP	3M	5	2018	Year-1		1	Spring
5/25/2018	FP	4M	5	2018	Year-1		1	Spring
5/25/2018	FP	5M	5	2018	Year-1		1	Spring
5/25/2018	FP	6M	5	2018	Year-1	0	0	Spring
5/25/2018	FP	7M	5	2018	Year-1	0	0	Spring
5/25/2018	FP	10M	5	2018	Year-1	0	0	Spring
5/25/2018	FP	12M	5	2018	Year-1	1	0	Spring
5/25/2018	FP	14M	5	2018	Year-1	1	0	Spring
5/25/2018	FP	8A	5	2018	Year-1	0	0	Spring
5/25/2018	FP	9A	5	2018	Year-1	0	0	Spring
5/25/2018	FP	11A	5	2018	Year-1	1	0	Spring
5/25/2018	FP	13A	5	2018	Year-1	1	0	Spring
5/26/2018	FP	1M	5	2018	Year-1	0	0	Spring
5/26/2018	FP	2M	5	2018	Year-1		1	Spring
5/26/2018	FP	3M	5	2018	Year-1		1	Spring
5/26/2018	FP	4M	5	2018	Year-1		1	Spring
5/26/2018	FP	5M	5	2018	Year-1		1	Spring
5/26/2018	FP	6M	5	2018	Year-1	0	0	Spring
5/26/2018	FP	7M	5	2018	Year-1	0	0	Spring
5/26/2018	FP	10M	5	2018	Year-1	0	0	Spring
5/26/2018	FP	12M	5	2018	Year-1	1	0	Spring
5/26/2018	FP	14M	5	2018	Year-1	1	0	Spring
5/26/2018	FP	8A	5	2018	Year-1	0	0	Spring
5/26/2018	FP	9A	5	2018	Year-1	0	0	Spring
5/26/2018	FP	11A	5	2018	Year-1	0	0	Spring
5/26/2018	FP	13A	5	2018	Year-1	1	0	Spring
5/27/2018	FP	1M	5	2018	Year-1	0	0	Spring
5/27/2018	FP	2M	5	2018	Year-1		1	Spring
5/27/2018	FP	3M	5	2018	Year-1		1	Spring

Date	Species	Site	Month	Year	Survey Year	Presence	Data Gap	Season
5/27/2018	FP	4M	5	2018	Year-1		1	Spring
5/27/2018	FP	5M	5	2018	Year-1		1	Spring
5/27/2018	FP	6M	5	2018	Year-1	0	0	Spring
5/27/2018	FP	7M	5	2018	Year-1	0	0	Spring
5/27/2018	FP	10M	5	2018	Year-1	0	0	Spring
5/27/2018	FP	12M	5	2018	Year-1	1	0	Spring
5/27/2018	FP	14M	5	2018	Year-1	1	0	Spring
5/27/2018	FP	8A	5	2018	Year-1	0	0	Spring
5/27/2018	FP	9A	5	2018	Year-1	0	0	Spring
5/27/2018	FP	11A	5	2018	Year-1	0	0	Spring
5/27/2018	FP	13A	5	2018	Year-1	1	0	Spring
5/28/2018	FP	1M	5	2018	Year-1	0	0	Spring
5/28/2018	FP	2M	5	2018	Year-1		1	Spring
5/28/2018	FP	3M	5	2018	Year-1		1	Spring
5/28/2018	FP	4M	5	2018	Year-1		1	Spring
5/28/2018	FP	5M	5	2018	Year-1		1	Spring
5/28/2018	FP	6M	5	2018	Year-1	0	0	Spring
5/28/2018	FP	7M	5	2018	Year-1	0	0	Spring
5/28/2018	FP	10M	5	2018	Year-1	0	0	Spring
5/28/2018	FP	12M	5	2018	Year-1	0	0	Spring
5/28/2018	FP	14M	5	2018	Year-1	1	0	Spring
5/28/2018	FP	8A	5	2018	Year-1	0	0	Spring
5/28/2018	FP	9A	5	2018	Year-1	0	0	Spring
5/28/2018	FP	11A	5	2018	Year-1	1	0	Spring
5/28/2018	FP	13A	5	2018	Year-1	1	0	Spring
5/29/2018	FP	1M	5	2018	Year-1	0	0	Spring
5/29/2018	FP	2M	5	2018	Year-1		1	Spring
5/29/2018	FP	3M	5	2018	Year-1		1	Spring
5/29/2018	FP	4M	5	2018	Year-1		1	Spring
5/29/2018	FP	5M	5	2018	Year-1		1	Spring
5/29/2018	FP	6M	5	2018	Year-1	0	0	Spring
5/29/2018	FP	7M	5	2018	Year-1	0	0	Spring
5/29/2018	FP	10M	5	2018	Year-1	0	0	Spring
5/29/2018	FP	12M	5	2018	Year-1	1	0	Spring
5/29/2018	FP	14M	5	2018	Year-1	1	0	Spring
5/29/2018	FP	8A	5	2018	Year-1	0	0	Spring
5/29/2018	FP	9A	5	2018	Year-1	0	0	Spring
5/29/2018	FP	11A	5	2018	Year-1	0	0	Spring
5/29/2018	FP	13A	5	2018	Year-1	1	0	Spring
5/30/2018	FP	1M	5	2018	Year-1	1	0	Spring
5/30/2018	FP	2M	5	2018	Year-1		1	Spring
5/30/2018	FP	3M	5	2018	Year-1		1	Spring
5/30/2018	FP	4M	5	2018	Year-1		1	Spring
5/30/2018	FP	5M	5	2018	Year-1		1	Spring

Date	Species	Site	Month	Year	Survey Year	Presence	Data Gap	Season
5/30/2018	FP	6M	5	2018	Year-1	0	0	Spring
5/30/2018	FP	7M	5	2018	Year-1	0	0	Spring
5/30/2018	FP	10M	5	2018	Year-1	0	0	Spring
5/30/2018	FP	12M	5	2018	Year-1	1	0	Spring
5/30/2018	FP	14M	5	2018	Year-1	1	0	Spring
5/30/2018	FP	8A	5	2018	Year-1	0	0	Spring
5/30/2018	FP	9A	5	2018	Year-1	0	0	Spring
5/30/2018	FP	11A	5	2018	Year-1	0	0	Spring
5/30/2018	FP	13A	5	2018	Year-1	1	0	Spring
5/31/2018	FP	1M	5	2018	Year-1	0	0	Spring
5/31/2018	FP	2M	5	2018	Year-1		1	Spring
5/31/2018	FP	3M	5	2018	Year-1		1	Spring
5/31/2018	FP	4M	5	2018	Year-1		1	Spring
5/31/2018	FP	5M	5	2018	Year-1		1	Spring
5/31/2018	FP	6M	5	2018	Year-1	1	0	Spring
5/31/2018	FP	7M	5	2018	Year-1	0	0	Spring
5/31/2018	FP	10M	5	2018	Year-1	1	0	Spring
5/31/2018	FP	12M	5	2018	Year-1	1	0	Spring
5/31/2018	FP	14M	5	2018	Year-1	1	0	Spring
5/31/2018	FP	8A	5	2018	Year-1	0	0	Spring
5/31/2018	FP	9A	5	2018	Year-1	0	0	Spring
5/31/2018	FP	11A	5	2018	Year-1	1	0	Spring
5/31/2018	FP	13A	5	2018	Year-1	1	0	Spring
6/1/2018	FP	1M	6	2018	Year-1	1	0	Summer
6/1/2018	FP	2M	6	2018	Year-1		1	Summer
6/1/2018	FP	3M	6	2018	Year-1		1	Summer
6/1/2018	FP	4M	6	2018	Year-1		1	Summer
6/1/2018	FP	5M	6	2018	Year-1		1	Summer
6/1/2018	FP	6M	6	2018	Year-1	0	0	Summer
6/1/2018	FP	7M	6	2018	Year-1	0	0	Summer
6/1/2018	FP	10M	6	2018	Year-1	1	0	Summer
6/1/2018	FP	12M	6	2018	Year-1	1	0	Summer
6/1/2018	FP	14M	6	2018	Year-1	1	0	Summer
6/1/2018	FP	8A	6	2018	Year-1	0	0	Summer
6/1/2018	FP	9A	6	2018	Year-1	0	0	Summer
6/1/2018	FP	11A	6	2018	Year-1	1	0	Summer
6/1/2018	FP	13A	6	2018	Year-1	1	0	Summer
6/2/2018	FP	1M	6	2018	Year-1	0	0	Summer
6/2/2018	FP	2M	6	2018	Year-1		1	Summer
6/2/2018	FP	3M	6	2018	Year-1		1	Summer
6/2/2018	FP	4M	6	2018	Year-1		1	Summer
6/2/2018	FP	5M	6	2018	Year-1		1	Summer
6/2/2018	FP	6M	6	2018	Year-1	1	0	Summer
6/2/2018	FP	7M	6	2018	Year-1	0	0	Summer

Date	Species	Site	Month	Year	Survey Year	Presence	Data Gap	Season
6/2/2018	FP	10M	6	2018	Year-1	1	0	Summer
6/2/2018	FP	12M	6	2018	Year-1	1	0	Summer
6/2/2018	FP	14M	6	2018	Year-1	1	0	Summer
6/2/2018	FP	8A	6	2018	Year-1	0	0	Summer
6/2/2018	FP	9A	6	2018	Year-1	0	0	Summer
6/2/2018	FP	11A	6	2018	Year-1	1	0	Summer
6/2/2018	FP	13A	6	2018	Year-1	1	0	Summer
6/3/2018	FP	1M	6	2018	Year-1	1	0	Summer
6/3/2018	FP	2M	6	2018	Year-1		1	Summer
6/3/2018	FP	3M	6	2018	Year-1		1	Summer
6/3/2018	FP	4M	6	2018	Year-1		1	Summer
6/3/2018	FP	5M	6	2018	Year-1		1	Summer
6/3/2018	FP	6M	6	2018	Year-1	1	0	Summer
6/3/2018	FP	7M	6	2018	Year-1	0	0	Summer
6/3/2018	FP	10M	6	2018	Year-1	1	0	Summer
6/3/2018	FP	12M	6	2018	Year-1	1	0	Summer
6/3/2018	FP	14M	6	2018	Year-1	1	0	Summer
6/3/2018	FP	8A	6	2018	Year-1	0	0	Summer
6/3/2018	FP	9A	6	2018	Year-1	0	0	Summer
6/3/2018	FP	11A	6	2018	Year-1	1	0	Summer
6/3/2018	FP	13A	6	2018	Year-1	1	0	Summer
6/4/2018	FP	1M	6	2018	Year-1	0	0	Summer
6/4/2018	FP	2M	6	2018	Year-1		1	Summer
6/4/2018	FP	3M	6	2018	Year-1		1	Summer
6/4/2018	FP	4M	6	2018	Year-1		1	Summer
6/4/2018	FP	5M	6	2018	Year-1		1	Summer
6/4/2018	FP	6M	6	2018	Year-1	1	0	Summer
6/4/2018	FP	7M	6	2018	Year-1	0	0	Summer
6/4/2018	FP	10M	6	2018	Year-1	1	0	Summer
6/4/2018	FP	12M	6	2018	Year-1	1	0	Summer
6/4/2018	FP	14M	6	2018	Year-1	1	0	Summer
6/4/2018	FP	8A	6	2018	Year-1	0	0	Summer
6/4/2018	FP	9A	6	2018	Year-1	0	0	Summer
6/4/2018	FP	11A	6	2018	Year-1	1	0	Summer
6/4/2018	FP	13A	6	2018	Year-1	1	0	Summer
6/5/2018	FP	1M	6	2018	Year-1	0	0	Summer
6/5/2018	FP	2M	6	2018	Year-1		1	Summer
6/5/2018	FP	3M	6	2018	Year-1		1	Summer
6/5/2018	FP	4M	6	2018	Year-1		1	Summer
6/5/2018	FP	5M	6	2018	Year-1		1	Summer
6/5/2018	FP	6M	6	2018	Year-1	1	0	Summer
6/5/2018	FP	7M	6	2018	Year-1	0	0	Summer
6/5/2018	FP	10M	6	2018	Year-1	1	0	Summer
6/5/2018	FP	12M	6	2018	Year-1	1	0	Summer

Date	Species	Site	Month	Year	Survey Year	Presence	Data Gap	Season
6/5/2018	FP	14M	6	2018	Year-1	1	0	Summer
6/5/2018	FP	8A	6	2018	Year-1	0	0	Summer
6/5/2018	FP	9A	6	2018	Year-1	0	0	Summer
6/5/2018	FP	11A	6	2018	Year-1	1	0	Summer
6/5/2018	FP	13A	6	2018	Year-1	1	0	Summer
6/6/2018	FP	1M	6	2018	Year-1	1	0	Summer
6/6/2018	FP	2M	6	2018	Year-1		1	Summer
6/6/2018	FP	3M	6	2018	Year-1		1	Summer
6/6/2018	FP	4M	6	2018	Year-1		1	Summer
6/6/2018	FP	5M	6	2018	Year-1		1	Summer
6/6/2018	FP	6M	6	2018	Year-1	0	0	Summer
6/6/2018	FP	7M	6	2018	Year-1	0	0	Summer
6/6/2018	FP	10M	6	2018	Year-1	1	0	Summer
6/6/2018	FP	12M	6	2018	Year-1	1	0	Summer
6/6/2018	FP	14M	6	2018	Year-1	1	0	Summer
6/6/2018	FP	8A	6	2018	Year-1	0	0	Summer
6/6/2018	FP	9A	6	2018	Year-1	0	0	Summer
6/6/2018	FP	11A	6	2018	Year-1	1	0	Summer
6/6/2018	FP	13A	6	2018	Year-1	1	0	Summer
6/7/2018	FP	1M	6	2018	Year-1	0	0	Summer
6/7/2018	FP	2M	6	2018	Year-1		1	Summer
6/7/2018	FP	3M	6	2018	Year-1		1	Summer
6/7/2018	FP	4M	6	2018	Year-1		1	Summer
6/7/2018	FP	5M	6	2018	Year-1		1	Summer
6/7/2018	FP	6M	6	2018	Year-1	1	0	Summer
6/7/2018	FP	7M	6	2018	Year-1	1	0	Summer
6/7/2018	FP	10M	6	2018	Year-1	1	0	Summer
6/7/2018	FP	12M	6	2018	Year-1	1	0	Summer
6/7/2018	FP	14M	6	2018	Year-1	1	0	Summer
6/7/2018	FP	8A	6	2018	Year-1	0	0	Summer
6/7/2018	FP	9A	6	2018	Year-1	1	0	Summer
6/7/2018	FP	11A	6	2018	Year-1	1	0	Summer
6/7/2018	FP	13A	6	2018	Year-1	1	0	Summer
6/8/2018	FP	1M	6	2018	Year-1	0	0	Summer
6/8/2018	FP	2M	6	2018	Year-1		1	Summer
6/8/2018	FP	3M	6	2018	Year-1		1	Summer
6/8/2018	FP	4M	6	2018	Year-1		1	Summer
6/8/2018	FP	5M	6	2018	Year-1		1	Summer
6/8/2018	FP	6M	6	2018	Year-1	1	0	Summer
6/8/2018	FP	7M	6	2018	Year-1	0	0	Summer
6/8/2018	FP	10M	6	2018	Year-1	1	0	Summer
6/8/2018	FP	12M	6	2018	Year-1	1	0	Summer
6/8/2018	FP	14M	6	2018	Year-1	1	0	Summer
6/8/2018	FP	8A	6	2018	Year-1	0	0	Summer

Date	Species	Site	Month	Year	Survey Year	Presence	Data Gap	Season
6/8/2018	FP	9A	6	2018	Year-1	0	0	Summer
6/8/2018	FP	11A	6	2018	Year-1	1	0	Summer
6/8/2018	FP	13A	6	2018	Year-1	1	0	Summer
6/9/2018	FP	1M	6	2018	Year-1	0	0	Summer
6/9/2018	FP	2M	6	2018	Year-1		1	Summer
6/9/2018	FP	3M	6	2018	Year-1		1	Summer
6/9/2018	FP	4M	6	2018	Year-1		1	Summer
6/9/2018	FP	5M	6	2018	Year-1		1	Summer
6/9/2018	FP	6M	6	2018	Year-1	0	0	Summer
6/9/2018	FP	7M	6	2018	Year-1	0	0	Summer
6/9/2018	FP	10M	6	2018	Year-1	1	0	Summer
6/9/2018	FP	12M	6	2018	Year-1	1	0	Summer
6/9/2018	FP	14M	6	2018	Year-1	1	0	Summer
6/9/2018	FP	8A	6	2018	Year-1	0	0	Summer
6/9/2018	FP	9A	6	2018	Year-1	0	0	Summer
6/9/2018	FP	11A	6	2018	Year-1	1	0	Summer
6/9/2018	FP	13A	6	2018	Year-1	1	0	Summer
6/10/2018	FP	1M	6	2018	Year-1	1	0	Summer
6/10/2018	FP	2M	6	2018	Year-1		1	Summer
6/10/2018	FP	3M	6	2018	Year-1		1	Summer
6/10/2018	FP	4M	6	2018	Year-1		1	Summer
6/10/2018	FP	5M	6	2018	Year-1		1	Summer
6/10/2018	FP	6M	6	2018	Year-1	1	0	Summer
6/10/2018	FP	7M	6	2018	Year-1	0	0	Summer
6/10/2018	FP	10M	6	2018	Year-1	1	0	Summer
6/10/2018	FP	12M	6	2018	Year-1	1	0	Summer
6/10/2018	FP	14M	6	2018	Year-1	1	0	Summer
6/10/2018	FP	8A	6	2018	Year-1	0	0	Summer
6/10/2018	FP	9A	6	2018	Year-1	1	0	Summer
6/10/2018	FP	11A	6	2018	Year-1	1	0	Summer
6/10/2018	FP	13A	6	2018	Year-1	1	0	Summer
6/11/2018	FP	1M	6	2018	Year-1	0	0	Summer
6/11/2018	FP	2M	6	2018	Year-1		1	Summer
6/11/2018	FP	3M	6	2018	Year-1		1	Summer
6/11/2018	FP	4M	6	2018	Year-1		1	Summer
6/11/2018	FP	5M	6	2018	Year-1		1	Summer
6/11/2018	FP	6M	6	2018	Year-1	1	0	Summer
6/11/2018	FP	7M	6	2018	Year-1	0	0	Summer
6/11/2018	FP	10M	6	2018	Year-1	1	0	Summer
6/11/2018	FP	12M	6	2018	Year-1	1	0	Summer
6/11/2018	FP	14M	6	2018	Year-1	1	0	Summer
6/11/2018	FP	8A	6	2018	Year-1	0	0	Summer
6/11/2018	FP	9A	6	2018	Year-1	0	0	Summer
6/11/2018	FP	11A	6	2018	Year-1	1	0	Summer

Date	Species	Site	Month	Year	Survey Year	Presence	Data Gap	Season
6/11/2018	FP	13A	6	2018	Year-1	1	0	Summer
6/12/2018	FP	1M	6	2018	Year-1	0	0	Summer
6/12/2018	FP	2M	6	2018	Year-1		1	Summer
6/12/2018	FP	3M	6	2018	Year-1		1	Summer
6/12/2018	FP	4M	6	2018	Year-1		1	Summer
6/12/2018	FP	5M	6	2018	Year-1		1	Summer
6/12/2018	FP	6M	6	2018	Year-1	0	0	Summer
6/12/2018	FP	7M	6	2018	Year-1	0	0	Summer
6/12/2018	FP	10M	6	2018	Year-1	1	0	Summer
6/12/2018	FP	12M	6	2018	Year-1	1	0	Summer
6/12/2018	FP	14M	6	2018	Year-1	1	0	Summer
6/12/2018	FP	8A	6	2018	Year-1	0	0	Summer
6/12/2018	FP	9A	6	2018	Year-1	0	0	Summer
6/12/2018	FP	11A	6	2018	Year-1	1	0	Summer
6/12/2018	FP	13A	6	2018	Year-1	1	0	Summer
6/13/2018	FP	1M	6	2018	Year-1	0	0	Summer
6/13/2018	FP	2M	6	2018	Year-1		1	Summer
6/13/2018	FP	3M	6	2018	Year-1		1	Summer
6/13/2018	FP	4M	6	2018	Year-1		1	Summer
6/13/2018	FP	5M	6	2018	Year-1		1	Summer
6/13/2018	FP	6M	6	2018	Year-1	0	0	Summer
6/13/2018	FP	7M	6	2018	Year-1	0	0	Summer
6/13/2018	FP	10M	6	2018	Year-1	1	0	Summer
6/13/2018	FP	12M	6	2018	Year-1	1	0	Summer
6/13/2018	FP	14M	6	2018	Year-1	1	0	Summer
6/13/2018	FP	8A	6	2018	Year-1	0	0	Summer
6/13/2018	FP	9A	6	2018	Year-1	0	0	Summer
6/13/2018	FP	11A	6	2018	Year-1	1	0	Summer
6/13/2018	FP	13A	6	2018	Year-1	1	0	Summer
6/14/2018	FP	1M	6	2018	Year-1	0	0	Summer
6/14/2018	FP	2M	6	2018	Year-1		1	Summer
6/14/2018	FP	3M	6	2018	Year-1		1	Summer
6/14/2018	FP	4M	6	2018	Year-1		1	Summer
6/14/2018	FP	5M	6	2018	Year-1		1	Summer
6/14/2018	FP	6M	6	2018	Year-1	1	0	Summer
6/14/2018	FP	7M	6	2018	Year-1	0	0	Summer
6/14/2018	FP	10M	6	2018	Year-1	1	0	Summer
6/14/2018	FP	12M	6	2018	Year-1	1	0	Summer
6/14/2018	FP	14M	6	2018	Year-1	1	0	Summer
6/14/2018	FP	8A	6	2018	Year-1	0	0	Summer
6/14/2018	FP	9A	6	2018	Year-1	0	0	Summer
6/14/2018	FP	11A	6	2018	Year-1	1	0	Summer
6/14/2018	FP	13A	6	2018	Year-1	1	0	Summer
6/15/2018	FP	1M	6	2018	Year-1	0	0	Summer

Date	Species	Site	Month	Year	Survey Year	Presence	Data Gap	Season
6/15/2018	FP	2M	6	2018	Year-1		1	Summer
6/15/2018	FP	3M	6	2018	Year-1		1	Summer
6/15/2018	FP	4M	6	2018	Year-1		1	Summer
6/15/2018	FP	5M	6	2018	Year-1		1	Summer
6/15/2018	FP	6M	6	2018	Year-1	0	0	Summer
6/15/2018	FP	7M	6	2018	Year-1	0	0	Summer
6/15/2018	FP	10M	6	2018	Year-1	1	0	Summer
6/15/2018	FP	12M	6	2018	Year-1	1	0	Summer
6/15/2018	FP	14M	6	2018	Year-1	1	0	Summer
6/15/2018	FP	8A	6	2018	Year-1	0	0	Summer
6/15/2018	FP	9A	6	2018	Year-1	0	0	Summer
6/15/2018	FP	11A	6	2018	Year-1	1	0	Summer
6/15/2018	FP	13A	6	2018	Year-1	1	0	Summer
6/16/2018	FP	1M	6	2018	Year-1	0	0	Summer
6/16/2018	FP	2M	6	2018	Year-1		1	Summer
6/16/2018	FP	3M	6	2018	Year-1		1	Summer
6/16/2018	FP	4M	6	2018	Year-1		1	Summer
6/16/2018	FP	5M	6	2018	Year-1		1	Summer
6/16/2018	FP	6M	6	2018	Year-1	0	0	Summer
6/16/2018	FP	7M	6	2018	Year-1	0	0	Summer
6/16/2018	FP	10M	6	2018	Year-1	0	0	Summer
6/16/2018	FP	12M	6	2018	Year-1	0	0	Summer
6/16/2018	FP	14M	6	2018	Year-1	0	0	Summer
6/16/2018	FP	8A	6	2018	Year-1	0	0	Summer
6/16/2018	FP	9A	6	2018	Year-1	0	0	Summer
6/16/2018	FP	11A	6	2018	Year-1	1	0	Summer
6/16/2018	FP	13A	6	2018	Year-1	1	0	Summer
6/17/2018	FP	1M	6	2018	Year-1	0	0	Summer
6/17/2018	FP	2M	6	2018	Year-1		1	Summer
6/17/2018	FP	3M	6	2018	Year-1		1	Summer
6/17/2018	FP	4M	6	2018	Year-1		1	Summer
6/17/2018	FP	5M	6	2018	Year-1		1	Summer
6/17/2018	FP	6M	6	2018	Year-1	0	0	Summer
6/17/2018	FP	7M	6	2018	Year-1	0	0	Summer
6/17/2018	FP	10M	6	2018	Year-1	0	0	Summer
6/17/2018	FP	12M	6	2018	Year-1	1	0	Summer
6/17/2018	FP	14M	6	2018	Year-1	1	0	Summer
6/17/2018	FP	8A	6	2018	Year-1	0	0	Summer
6/17/2018	FP	9A	6	2018	Year-1	0	0	Summer
6/17/2018	FP	11A	6	2018	Year-1	1	0	Summer
6/17/2018	FP	13A	6	2018	Year-1	1	0	Summer
6/18/2018	FP	1M	6	2018	Year-1	0	0	Summer
6/18/2018	FP	2M	6	2018	Year-1		1	Summer
6/18/2018	FP	3M	6	2018	Year-1		1	Summer

Date	Species	Site	Month	Year	Survey Year	Presence	Data Gap	Season
6/18/2018	FP	4M	6	2018	Year-1		1	Summer
6/18/2018	FP	5M	6	2018	Year-1		1	Summer
6/18/2018	FP	6M	6	2018	Year-1	0	0	Summer
6/18/2018	FP	7M	6	2018	Year-1	0	0	Summer
6/18/2018	FP	10M	6	2018	Year-1	1	0	Summer
6/18/2018	FP	12M	6	2018	Year-1	1	0	Summer
6/18/2018	FP	14M	6	2018	Year-1	1	0	Summer
6/18/2018	FP	8A	6	2018	Year-1	0	0	Summer
6/18/2018	FP	9A	6	2018	Year-1	0	0	Summer
6/18/2018	FP	11A	6	2018	Year-1	1	0	Summer
6/18/2018	FP	13A	6	2018	Year-1	1	0	Summer
6/19/2018	FP	1M	6	2018	Year-1	0	0	Summer
6/19/2018	FP	2M	6	2018	Year-1		1	Summer
6/19/2018	FP	3M	6	2018	Year-1		1	Summer
6/19/2018	FP	4M	6	2018	Year-1		1	Summer
6/19/2018	FP	5M	6	2018	Year-1		1	Summer
6/19/2018	FP	6M	6	2018	Year-1	1	0	Summer
6/19/2018	FP	7M	6	2018	Year-1	0	0	Summer
6/19/2018	FP	10M	6	2018	Year-1	1	0	Summer
6/19/2018	FP	12M	6	2018	Year-1	1	0	Summer
6/19/2018	FP	14M	6	2018	Year-1	1	0	Summer
6/19/2018	FP	8A	6	2018	Year-1	0	0	Summer
6/19/2018	FP	9A	6	2018	Year-1	0	0	Summer
6/19/2018	FP	11A	6	2018	Year-1	1	0	Summer
6/19/2018	FP	13A	6	2018	Year-1	1	0	Summer
6/20/2018	FP	1M	6	2018	Year-1	0	0	Summer
6/20/2018	FP	2M	6	2018	Year-1		1	Summer
6/20/2018	FP	3M	6	2018	Year-1		1	Summer
6/20/2018	FP	4M	6	2018	Year-1		1	Summer
6/20/2018	FP	5M	6	2018	Year-1		1	Summer
6/20/2018	FP	6M	6	2018	Year-1	1	0	Summer
6/20/2018	FP	7M	6	2018	Year-1	0	0	Summer
6/20/2018	FP	10M	6	2018	Year-1	1	0	Summer
6/20/2018	FP	12M	6	2018	Year-1	1	0	Summer
6/20/2018	FP	14M	6	2018	Year-1	1	0	Summer
6/20/2018	FP	8A	6	2018	Year-1	0	0	Summer
6/20/2018	FP	9A	6	2018	Year-1	0	0	Summer
6/20/2018	FP	11A	6	2018	Year-1	1	0	Summer
6/20/2018	FP	13A	6	2018	Year-1	1	0	Summer
6/21/2018	FP	1M	6	2018	Year-1	0	0	Summer
6/21/2018	FP	2M	6	2018	Year-1		1	Summer
6/21/2018	FP	3M	6	2018	Year-1		1	Summer
6/21/2018	FP	4M	6	2018	Year-1		1	Summer
6/21/2018	FP	5M	6	2018	Year-1		1	Summer

Date	Species	Site	Month	Year	Survey Year	Presence	Data Gap	Season
6/21/2018	FP	6M	6	2018	Year-1	1	0	Summer
6/21/2018	FP	7M	6	2018	Year-1	0	0	Summer
6/21/2018	FP	10M	6	2018	Year-1	1	0	Summer
6/21/2018	FP	12M	6	2018	Year-1	1	0	Summer
6/21/2018	FP	14M	6	2018	Year-1	1	0	Summer
6/21/2018	FP	8A	6	2018	Year-1	0	0	Summer
6/21/2018	FP	9A	6	2018	Year-1	0	0	Summer
6/21/2018	FP	11A	6	2018	Year-1	1	0	Summer
6/21/2018	FP	13A	6	2018	Year-1	1	0	Summer
6/22/2018	FP	1M	6	2018	Year-1	0	0	Summer
6/22/2018	FP	2M	6	2018	Year-1		1	Summer
6/22/2018	FP	3M	6	2018	Year-1		1	Summer
6/22/2018	FP	4M	6	2018	Year-1		1	Summer
6/22/2018	FP	5M	6	2018	Year-1		1	Summer
6/22/2018	FP	6M	6	2018	Year-1	1	0	Summer
6/22/2018	FP	7M	6	2018	Year-1	0	0	Summer
6/22/2018	FP	10M	6	2018	Year-1	0	0	Summer
6/22/2018	FP	12M	6	2018	Year-1	1	0	Summer
6/22/2018	FP	14M	6	2018	Year-1	1	0	Summer
6/22/2018	FP	8A	6	2018	Year-1	0	0	Summer
6/22/2018	FP	9A	6	2018	Year-1	0	0	Summer
6/22/2018	FP	11A	6	2018	Year-1	1	0	Summer
6/22/2018	FP	13A	6	2018	Year-1	1	0	Summer
6/23/2018	FP	1M	6	2018	Year-1	1	0	Summer
6/23/2018	FP	2M	6	2018	Year-1		1	Summer
6/23/2018	FP	3M	6	2018	Year-1		1	Summer
6/23/2018	FP	4M	6	2018	Year-1		1	Summer
6/23/2018	FP	5M	6	2018	Year-1		1	Summer
6/23/2018	FP	6M	6	2018	Year-1	1	0	Summer
6/23/2018	FP	7M	6	2018	Year-1	0	0	Summer
6/23/2018	FP	10M	6	2018	Year-1	1	0	Summer
6/23/2018	FP	12M	6	2018	Year-1	0	0	Summer
6/23/2018	FP	14M	6	2018	Year-1	1	0	Summer
6/23/2018	FP	8A	6	2018	Year-1	0	0	Summer
6/23/2018	FP	9A	6	2018	Year-1	0	0	Summer
6/23/2018	FP	11A	6	2018	Year-1	1	0	Summer
6/23/2018	FP	13A	6	2018	Year-1	1	0	Summer
6/24/2018	FP	1M	6	2018	Year-1	0	0	Summer
6/24/2018	FP	2M	6	2018	Year-1		1	Summer
6/24/2018	FP	3M	6	2018	Year-1		1	Summer
6/24/2018	FP	4M	6	2018	Year-1		1	Summer
6/24/2018	FP	5M	6	2018	Year-1		1	Summer
6/24/2018	FP	6M	6	2018	Year-1	1	0	Summer
6/24/2018	FP	7M	6	2018	Year-1	0	0	Summer

Date	Species	Site	Month	Year	Survey Year	Presence	Data Gap	Season
6/24/2018	FP	10M	6	2018	Year-1	1	0	Summer
6/24/2018	FP	12M	6	2018	Year-1	1	0	Summer
6/24/2018	FP	14M	6	2018	Year-1	1	0	Summer
6/24/2018	FP	8A	6	2018	Year-1	0	0	Summer
6/24/2018	FP	9A	6	2018	Year-1	0	0	Summer
6/24/2018	FP	11A	6	2018	Year-1	1	0	Summer
6/24/2018	FP	13A	6	2018	Year-1	1	0	Summer
6/25/2018	FP	1M	6	2018	Year-1	0	0	Summer
6/25/2018	FP	2M	6	2018	Year-1		1	Summer
6/25/2018	FP	3M	6	2018	Year-1		1	Summer
6/25/2018	FP	4M	6	2018	Year-1		1	Summer
6/25/2018	FP	5M	6	2018	Year-1		1	Summer
6/25/2018	FP	6M	6	2018	Year-1	0	0	Summer
6/25/2018	FP	7M	6	2018	Year-1	0	0	Summer
6/25/2018	FP	10M	6	2018	Year-1	0	0	Summer
6/25/2018	FP	12M	6	2018	Year-1	1	0	Summer
6/25/2018	FP	14M	6	2018	Year-1	1	0	Summer
6/25/2018	FP	8A	6	2018	Year-1	0	0	Summer
6/25/2018	FP	9A	6	2018	Year-1	0	0	Summer
6/25/2018	FP	11A	6	2018	Year-1	1	0	Summer
6/25/2018	FP	13A	6	2018	Year-1	1	0	Summer
6/26/2018	FP	1M	6	2018	Year-1	0	0	Summer
6/26/2018	FP	2M	6	2018	Year-1		1	Summer
6/26/2018	FP	3M	6	2018	Year-1		1	Summer
6/26/2018	FP	4M	6	2018	Year-1		1	Summer
6/26/2018	FP	5M	6	2018	Year-1		1	Summer
6/26/2018	FP	6M	6	2018	Year-1	0	0	Summer
6/26/2018	FP	7M	6	2018	Year-1	0	0	Summer
6/26/2018	FP	10M	6	2018	Year-1	0	0	Summer
6/26/2018	FP	12M	6	2018	Year-1	1	0	Summer
6/26/2018	FP	14M	6	2018	Year-1	1	0	Summer
6/26/2018	FP	8A	6	2018	Year-1	0	0	Summer
6/26/2018	FP	9A	6	2018	Year-1	0	0	Summer
6/26/2018	FP	11A	6	2018	Year-1	1	0	Summer
6/26/2018	FP	13A	6	2018	Year-1	1	0	Summer
6/27/2018	FP	1M	6	2018	Year-1	0	0	Summer
6/27/2018	FP	2M	6	2018	Year-1		1	Summer
6/27/2018	FP	3M	6	2018	Year-1		1	Summer
6/27/2018	FP	4M	6	2018	Year-1		1	Summer
6/27/2018	FP	5M	6	2018	Year-1		1	Summer
6/27/2018	FP	6M	6	2018	Year-1	0	0	Summer
6/27/2018	FP	7M	6	2018	Year-1		1	Summer
6/27/2018	FP	10M	6	2018	Year-1	1	0	Summer
6/27/2018	FP	12M	6	2018	Year-1	1	0	Summer

Date	Species	Site	Month	Year	Survey Year	Presence	Data Gap	Season
6/27/2018	FP	14M	6	2018	Year-1	1	0	Summer
6/27/2018	FP	8A	6	2018	Year-1	0	0	Summer
6/27/2018	FP	9A	6	2018	Year-1	0	0	Summer
6/27/2018	FP	11A	6	2018	Year-1	1	0	Summer
6/27/2018	FP	13A	6	2018	Year-1	1	0	Summer
6/28/2018	FP	1M	6	2018	Year-1	0	0	Summer
6/28/2018	FP	2M	6	2018	Year-1		1	Summer
6/28/2018	FP	3M	6	2018	Year-1		1	Summer
6/28/2018	FP	4M	6	2018	Year-1		1	Summer
6/28/2018	FP	5M	6	2018	Year-1		1	Summer
6/28/2018	FP	6M	6	2018	Year-1	1	0	Summer
6/28/2018	FP	7M	6	2018	Year-1		1	Summer
6/28/2018	FP	10M	6	2018	Year-1	1	0	Summer
6/28/2018	FP	12M	6	2018	Year-1	1	0	Summer
6/28/2018	FP	14M	6	2018	Year-1	1	0	Summer
6/28/2018	FP	8A	6	2018	Year-1	0	0	Summer
6/28/2018	FP	9A	6	2018	Year-1	0	0	Summer
6/28/2018	FP	11A	6	2018	Year-1	1	0	Summer
6/28/2018	FP	13A	6	2018	Year-1	1	0	Summer
6/29/2018	FP	1M	6	2018	Year-1	0	0	Summer
6/29/2018	FP	2M	6	2018	Year-1		1	Summer
6/29/2018	FP	3M	6	2018	Year-1		1	Summer
6/29/2018	FP	4M	6	2018	Year-1		1	Summer
6/29/2018	FP	5M	6	2018	Year-1		1	Summer
6/29/2018	FP	6M	6	2018	Year-1	0	0	Summer
6/29/2018	FP	7M	6	2018	Year-1		1	Summer
6/29/2018	FP	10M	6	2018	Year-1	1	0	Summer
6/29/2018	FP	12M	6	2018	Year-1	1	0	Summer
6/29/2018	FP	14M	6	2018	Year-1	1	0	Summer
6/29/2018	FP	8A	6	2018	Year-1	0	0	Summer
6/29/2018	FP	9A	6	2018	Year-1	0	0	Summer
6/29/2018	FP	11A	6	2018	Year-1	1	0	Summer
6/29/2018	FP	13A	6	2018	Year-1	1	0	Summer
6/30/2018	FP	1M	6	2018	Year-1	0	0	Summer
6/30/2018	FP	2M	6	2018	Year-1		1	Summer
6/30/2018	FP	3M	6	2018	Year-1		1	Summer
6/30/2018	FP	4M	6	2018	Year-1		1	Summer
6/30/2018	FP	5M	6	2018	Year-1		1	Summer
6/30/2018	FP	6M	6	2018	Year-1	0	0	Summer
6/30/2018	FP	7M	6	2018	Year-1		1	Summer
6/30/2018	FP	10M	6	2018	Year-1	1	0	Summer
6/30/2018	FP	12M	6	2018	Year-1	1	0	Summer
6/30/2018	FP	14M	6	2018	Year-1	1	0	Summer
6/30/2018	FP	8A	6	2018	Year-1	0	0	Summer

Date	Species	Site	Month	Year	Survey Year	Presence	Data Gap	Season
6/30/2018	FP	9A	6	2018	Year-1	0	0	Summer
6/30/2018	FP	11A	6	2018	Year-1	1	0	Summer
6/30/2018	FP	13A	6	2018	Year-1	1	0	Summer
7/1/2018	FP	1M	7	2018	Year-1	0	0	Summer
7/1/2018	FP	2M	7	2018	Year-1		1	Summer
7/1/2018	FP	3M	7	2018	Year-1		1	Summer
7/1/2018	FP	4M	7	2018	Year-1		1	Summer
7/1/2018	FP	5M	7	2018	Year-1		1	Summer
7/1/2018	FP	6M	7	2018	Year-1	1	0	Summer
7/1/2018	FP	7M	7	2018	Year-1		1	Summer
7/1/2018	FP	10M	7	2018	Year-1	1	0	Summer
7/1/2018	FP	12M	7	2018	Year-1	1	0	Summer
7/1/2018	FP	14M	7	2018	Year-1	1	0	Summer
7/1/2018	FP	8A	7	2018	Year-1	0	0	Summer
7/1/2018	FP	9A	7	2018	Year-1	0	0	Summer
7/1/2018	FP	11A	7	2018	Year-1	1	0	Summer
7/1/2018	FP	13A	7	2018	Year-1	1	0	Summer
7/2/2018	FP	1M	7	2018	Year-1	0	0	Summer
7/2/2018	FP	2M	7	2018	Year-1		1	Summer
7/2/2018	FP	3M	7	2018	Year-1		1	Summer
7/2/2018	FP	4M	7	2018	Year-1		1	Summer
7/2/2018	FP	5M	7	2018	Year-1		1	Summer
7/2/2018	FP	6M	7	2018	Year-1	1	0	Summer
7/2/2018	FP	7M	7	2018	Year-1		1	Summer
7/2/2018	FP	10M	7	2018	Year-1	0	0	Summer
7/2/2018	FP	12M	7	2018	Year-1	1	0	Summer
7/2/2018	FP	14M	7	2018	Year-1	1	0	Summer
7/2/2018	FP	8A	7	2018	Year-1	0	0	Summer
7/2/2018	FP	9A	7	2018	Year-1	0	0	Summer
7/2/2018	FP	11A	7	2018	Year-1	1	0	Summer
7/2/2018	FP	13A	7	2018	Year-1	1	0	Summer
7/3/2018	FP	1M	7	2018	Year-1	1	0	Summer
7/3/2018	FP	2M	7	2018	Year-1		1	Summer
7/3/2018	FP	3M	7	2018	Year-1		1	Summer
7/3/2018	FP	4M	7	2018	Year-1		1	Summer
7/3/2018	FP	5M	7	2018	Year-1		1	Summer
7/3/2018	FP	6M	7	2018	Year-1	1	0	Summer
7/3/2018	FP	7M	7	2018	Year-1		1	Summer
7/3/2018	FP	10M	7	2018	Year-1	0	0	Summer
7/3/2018	FP	12M	7	2018	Year-1	1	0	Summer
7/3/2018	FP	14M	7	2018	Year-1	1	0	Summer
7/3/2018	FP	8A	7	2018	Year-1	0	0	Summer
7/3/2018	FP	9A	7	2018	Year-1	0	0	Summer
7/3/2018	FP	11A	7	2018	Year-1	0	0	Summer

Date	Species	Site	Month	Year	Survey Year	Presence	Data Gap	Season
7/3/2018	FP	13A	7	2018	Year-1	1	0	Summer
7/4/2018	FP	1M	7	2018	Year-1	1	0	Summer
7/4/2018	FP	2M	7	2018	Year-1		1	Summer
7/4/2018	FP	3M	7	2018	Year-1		1	Summer
7/4/2018	FP	4M	7	2018	Year-1		1	Summer
7/4/2018	FP	5M	7	2018	Year-1		1	Summer
7/4/2018	FP	6M	7	2018	Year-1	1	0	Summer
7/4/2018	FP	7M	7	2018	Year-1		1	Summer
7/4/2018	FP	10M	7	2018	Year-1	0	0	Summer
7/4/2018	FP	12M	7	2018	Year-1	1	0	Summer
7/4/2018	FP	14M	7	2018	Year-1	1	0	Summer
7/4/2018	FP	8A	7	2018	Year-1	0	0	Summer
7/4/2018	FP	9A	7	2018	Year-1	0	0	Summer
7/4/2018	FP	11A	7	2018	Year-1	1	0	Summer
7/4/2018	FP	13A	7	2018	Year-1	1	0	Summer
7/5/2018	FP	1M	7	2018	Year-1	0	0	Summer
7/5/2018	FP	2M	7	2018	Year-1		1	Summer
7/5/2018	FP	3M	7	2018	Year-1		1	Summer
7/5/2018	FP	4M	7	2018	Year-1		1	Summer
7/5/2018	FP	5M	7	2018	Year-1		1	Summer
7/5/2018	FP	6M	7	2018	Year-1	1	0	Summer
7/5/2018	FP	7M	7	2018	Year-1		1	Summer
7/5/2018	FP	10M	7	2018	Year-1	1	0	Summer
7/5/2018	FP	12M	7	2018	Year-1	1	0	Summer
7/5/2018	FP	14M	7	2018	Year-1	1	0	Summer
7/5/2018	FP	8A	7	2018	Year-1	0	0	Summer
7/5/2018	FP	9A	7	2018	Year-1	0	0	Summer
7/5/2018	FP	11A	7	2018	Year-1	1	0	Summer
7/5/2018	FP	13A	7	2018	Year-1	1	0	Summer
7/6/2018	FP	1M	7	2018	Year-1	0	0	Summer
7/6/2018	FP	2M	7	2018	Year-1		1	Summer
7/6/2018	FP	3M	7	2018	Year-1		1	Summer
7/6/2018	FP	4M	7	2018	Year-1		1	Summer
7/6/2018	FP	5M	7	2018	Year-1		1	Summer
7/6/2018	FP	6M	7	2018	Year-1	1	0	Summer
7/6/2018	FP	7M	7	2018	Year-1		1	Summer
7/6/2018	FP	10M	7	2018	Year-1	1	0	Summer
7/6/2018	FP	12M	7	2018	Year-1	1	0	Summer
7/6/2018	FP	14M	7	2018	Year-1	1	0	Summer
7/6/2018	FP	8A	7	2018	Year-1	0	0	Summer
7/6/2018	FP	9A	7	2018	Year-1	0	0	Summer
7/6/2018	FP	11A	7	2018	Year-1	1	0	Summer
7/6/2018	FP	13A	7	2018	Year-1	1	0	Summer
7/7/2018	FP	1M	7	2018	Year-1	0	0	Summer

Date	Species	Site	Month	Year	Survey Year	Presence	Data Gap	Season
7/7/2018	FP	2M	7	2018	Year-1		1	Summer
7/7/2018	FP	3M	7	2018	Year-1		1	Summer
7/7/2018	FP	4M	7	2018	Year-1		1	Summer
7/7/2018	FP	5M	7	2018	Year-1		1	Summer
7/7/2018	FP	6M	7	2018	Year-1	1	0	Summer
7/7/2018	FP	7M	7	2018	Year-1		1	Summer
7/7/2018	FP	10M	7	2018	Year-1	1	0	Summer
7/7/2018	FP	12M	7	2018	Year-1	1	0	Summer
7/7/2018	FP	14M	7	2018	Year-1	1	0	Summer
7/7/2018	FP	8A	7	2018	Year-1	0	0	Summer
7/7/2018	FP	9A	7	2018	Year-1	0	0	Summer
7/7/2018	FP	11A	7	2018	Year-1	1	0	Summer
7/7/2018	FP	13A	7	2018	Year-1	1	0	Summer
7/8/2018	FP	1M	7	2018	Year-1	0	0	Summer
7/8/2018	FP	2M	7	2018	Year-1		1	Summer
7/8/2018	FP	3M	7	2018	Year-1		1	Summer
7/8/2018	FP	4M	7	2018	Year-1		1	Summer
7/8/2018	FP	5M	7	2018	Year-1		1	Summer
7/8/2018	FP	6M	7	2018	Year-1	0	0	Summer
7/8/2018	FP	7M	7	2018	Year-1		1	Summer
7/8/2018	FP	10M	7	2018	Year-1	1	0	Summer
7/8/2018	FP	12M	7	2018	Year-1	1	0	Summer
7/8/2018	FP	14M	7	2018	Year-1	1	0	Summer
7/8/2018	FP	8A	7	2018	Year-1	0	0	Summer
7/8/2018	FP	9A	7	2018	Year-1	0	0	Summer
7/8/2018	FP	11A	7	2018	Year-1	0	0	Summer
7/8/2018	FP	13A	7	2018	Year-1	1	0	Summer
7/9/2018	FP	1M	7	2018	Year-1	0	0	Summer
7/9/2018	FP	2M	7	2018	Year-1		1	Summer
7/9/2018	FP	3M	7	2018	Year-1		1	Summer
7/9/2018	FP	4M	7	2018	Year-1		1	Summer
7/9/2018	FP	5M	7	2018	Year-1		1	Summer
7/9/2018	FP	6M	7	2018	Year-1	0	0	Summer
7/9/2018	FP	7M	7	2018	Year-1		1	Summer
7/9/2018	FP	10M	7	2018	Year-1	1	0	Summer
7/9/2018	FP	12M	7	2018	Year-1	1	0	Summer
7/9/2018	FP	14M	7	2018	Year-1	1	0	Summer
7/9/2018	FP	8A	7	2018	Year-1	0	0	Summer
7/9/2018	FP	9A	7	2018	Year-1	1	0	Summer
7/9/2018	FP	11A	7	2018	Year-1	1	0	Summer
7/9/2018	FP	13A	7	2018	Year-1	1	0	Summer
7/10/2018	FP	1M	7	2018	Year-1	1	0	Summer
7/10/2018	FP	2M	7	2018	Year-1		1	Summer
7/10/2018	FP	3M	7	2018	Year-1		1	Summer

Date	Species	Site	Month	Year	Survey Year	Presence	Data Gap	Season
7/10/2018	FP	4M	7	2018	Year-1		1	Summer
7/10/2018	FP	5M	7	2018	Year-1		1	Summer
7/10/2018	FP	6M	7	2018	Year-1	1	0	Summer
7/10/2018	FP	7M	7	2018	Year-1		1	Summer
7/10/2018	FP	10M	7	2018	Year-1	1	0	Summer
7/10/2018	FP	12M	7	2018	Year-1	1	0	Summer
7/10/2018	FP	14M	7	2018	Year-1	1	0	Summer
7/10/2018	FP	8A	7	2018	Year-1	0	0	Summer
7/10/2018	FP	9A	7	2018	Year-1	1	0	Summer
7/10/2018	FP	11A	7	2018	Year-1	1	0	Summer
7/10/2018	FP	13A	7	2018	Year-1	1	0	Summer
7/11/2018	FP	1M	7	2018	Year-1	1	0	Summer
7/11/2018	FP	2M	7	2018	Year-1		1	Summer
7/11/2018	FP	3M	7	2018	Year-1		1	Summer
7/11/2018	FP	4M	7	2018	Year-1		1	Summer
7/11/2018	FP	5M	7	2018	Year-1		1	Summer
7/11/2018	FP	6M	7	2018	Year-1	1	0	Summer
7/11/2018	FP	7M	7	2018	Year-1		1	Summer
7/11/2018	FP	10M	7	2018	Year-1	1	0	Summer
7/11/2018	FP	12M	7	2018	Year-1	1	0	Summer
7/11/2018	FP	14M	7	2018	Year-1	1	0	Summer
7/11/2018	FP	8A	7	2018	Year-1	0	0	Summer
7/11/2018	FP	9A	7	2018	Year-1	1	0	Summer
7/11/2018	FP	11A	7	2018	Year-1	1	0	Summer
7/11/2018	FP	13A	7	2018	Year-1	1	0	Summer
7/12/2018	FP	1M	7	2018	Year-1	1	0	Summer
7/12/2018	FP	2M	7	2018	Year-1		1	Summer
7/12/2018	FP	3M	7	2018	Year-1		1	Summer
7/12/2018	FP	4M	7	2018	Year-1		1	Summer
7/12/2018	FP	5M	7	2018	Year-1		1	Summer
7/12/2018	FP	6M	7	2018	Year-1	1	0	Summer
7/12/2018	FP	7M	7	2018	Year-1		1	Summer
7/12/2018	FP	10M	7	2018	Year-1	0	0	Summer
7/12/2018	FP	12M	7	2018	Year-1	1	0	Summer
7/12/2018	FP	14M	7	2018	Year-1	1	0	Summer
7/12/2018	FP	8A	7	2018	Year-1	0	0	Summer
7/12/2018	FP	9A	7	2018	Year-1	0	0	Summer
7/12/2018	FP	11A	7	2018	Year-1	1	0	Summer
7/12/2018	FP	13A	7	2018	Year-1	1	0	Summer
7/13/2018	FP	1M	7	2018	Year-1	0	0	Summer
7/13/2018	FP	2M	7	2018	Year-1		1	Summer
7/13/2018	FP	3M	7	2018	Year-1		1	Summer
7/13/2018	FP	4M	7	2018	Year-1		1	Summer
7/13/2018	FP	5M	7	2018	Year-1		1	Summer

Date	Species	Site	Month	Year	Survey Year	Presence	Data Gap	Season
7/13/2018	FP	6M	7	2018	Year-1	1	0	Summer
7/13/2018	FP	7M	7	2018	Year-1		1	Summer
7/13/2018	FP	10M	7	2018	Year-1	0	0	Summer
7/13/2018	FP	12M	7	2018	Year-1	1	0	Summer
7/13/2018	FP	14M	7	2018	Year-1	1	0	Summer
7/13/2018	FP	8A	7	2018	Year-1	0	0	Summer
7/13/2018	FP	9A	7	2018	Year-1	0	0	Summer
7/13/2018	FP	11A	7	2018	Year-1	1	0	Summer
7/13/2018	FP	13A	7	2018	Year-1	1	0	Summer
7/14/2018	FP	1M	7	2018	Year-1	1	0	Summer
7/14/2018	FP	2M	7	2018	Year-1		1	Summer
7/14/2018	FP	3M	7	2018	Year-1		1	Summer
7/14/2018	FP	4M	7	2018	Year-1		1	Summer
7/14/2018	FP	5M	7	2018	Year-1		1	Summer
7/14/2018	FP	6M	7	2018	Year-1	1	0	Summer
7/14/2018	FP	7M	7	2018	Year-1		1	Summer
7/14/2018	FP	10M	7	2018	Year-1	0	0	Summer
7/14/2018	FP	12M	7	2018	Year-1	1	0	Summer
7/14/2018	FP	14M	7	2018	Year-1	1	0	Summer
7/14/2018	FP	8A	7	2018	Year-1	0	0	Summer
7/14/2018	FP	9A	7	2018	Year-1	0	0	Summer
7/14/2018	FP	11A	7	2018	Year-1	1	0	Summer
7/14/2018	FP	13A	7	2018	Year-1	1	0	Summer
7/15/2018	FP	1M	7	2018	Year-1		1	Summer
7/15/2018	FP	2M	7	2018	Year-1		1	Summer
7/15/2018	FP	3M	7	2018	Year-1		1	Summer
7/15/2018	FP	4M	7	2018	Year-1		1	Summer
7/15/2018	FP	5M	7	2018	Year-1		1	Summer
7/15/2018	FP	6M	7	2018	Year-1		1	Summer
7/15/2018	FP	7M	7	2018	Year-1		1	Summer
7/15/2018	FP	10M	7	2018	Year-1		1	Summer
7/15/2018	FP	12M	7	2018	Year-1		1	Summer
7/15/2018	FP	14M	7	2018	Year-1		1	Summer
7/15/2018	FP	8A	7	2018	Year-1		1	Summer
7/15/2018	FP	9A	7	2018	Year-1		1	Summer
7/15/2018	FP	11A	7	2018	Year-1		1	Summer
7/15/2018	FP	13A	7	2018	Year-1		1	Summer
7/16/2018	FP	1M	7	2018	Year-1		1	Summer
7/16/2018	FP	2M	7	2018	Year-1		1	Summer
7/16/2018	FP	3M	7	2018	Year-1		1	Summer
7/16/2018	FP	4M	7	2018	Year-1		1	Summer
7/16/2018	FP	5M	7	2018	Year-1		1	Summer
7/16/2018	FP	6M	7	2018	Year-1		1	Summer
7/16/2018	FP	7M	7	2018	Year-1		1	Summer

Date	Species	Site	Month	Year	Survey Year	Presence	Data Gap	Season
7/16/2018	FP	10M	7	2018	Year-1		1	Summer
7/16/2018	FP	12M	7	2018	Year-1	0	0	Summer
7/16/2018	FP	14M	7	2018	Year-1		1	Summer
7/16/2018	FP	8A	7	2018	Year-1		1	Summer
7/16/2018	FP	9A	7	2018	Year-1	0	0	Summer
7/16/2018	FP	11A	7	2018	Year-1	1	0	Summer
7/16/2018	FP	13A	7	2018	Year-1	1	0	Summer
7/17/2018	FP	1M	7	2018	Year-1		1	Summer
7/17/2018	FP	2M	7	2018	Year-1		1	Summer
7/17/2018	FP	3M	7	2018	Year-1		1	Summer
7/17/2018	FP	4M	7	2018	Year-1		1	Summer
7/17/2018	FP	5M	7	2018	Year-1		1	Summer
7/17/2018	FP	6M	7	2018	Year-1		1	Summer
7/17/2018	FP	7M	7	2018	Year-1		1	Summer
7/17/2018	FP	10M	7	2018	Year-1		1	Summer
7/17/2018	FP	12M	7	2018	Year-1	0	0	Summer
7/17/2018	FP	14M	7	2018	Year-1		1	Summer
7/17/2018	FP	8A	7	2018	Year-1		1	Summer
7/17/2018	FP	9A	7	2018	Year-1	0	0	Summer
7/17/2018	FP	11A	7	2018	Year-1	1	0	Summer
7/17/2018	FP	13A	7	2018	Year-1	1	0	Summer
7/18/2018	FP	1M	7	2018	Year-1		1	Summer
7/18/2018	FP	2M	7	2018	Year-1		1	Summer
7/18/2018	FP	3M	7	2018	Year-1		1	Summer
7/18/2018	FP	4M	7	2018	Year-1		1	Summer
7/18/2018	FP	5M	7	2018	Year-1		1	Summer
7/18/2018	FP	6M	7	2018	Year-1		1	Summer
7/18/2018	FP	7M	7	2018	Year-1		1	Summer
7/18/2018	FP	10M	7	2018	Year-1		1	Summer
7/18/2018	FP	12M	7	2018	Year-1	0	0	Summer
7/18/2018	FP	14M	7	2018	Year-1		1	Summer
7/18/2018	FP	8A	7	2018	Year-1		1	Summer
7/18/2018	FP	9A	7	2018	Year-1	0	0	Summer
7/18/2018	FP	11A	7	2018	Year-1	1	0	Summer
7/18/2018	FP	13A	7	2018	Year-1	1	0	Summer
7/19/2018	FP	1M	7	2018	Year-1		1	Summer
7/19/2018	FP	2M	7	2018	Year-1		1	Summer
7/19/2018	FP	3M	7	2018	Year-1		1	Summer
7/19/2018	FP	4M	7	2018	Year-1		1	Summer
7/19/2018	FP	5M	7	2018	Year-1		1	Summer
7/19/2018	FP	6M	7	2018	Year-1		1	Summer
7/19/2018	FP	7M	7	2018	Year-1		1	Summer
7/19/2018	FP	10M	7	2018	Year-1		1	Summer
7/19/2018	FP	12M	7	2018	Year-1	0	0	Summer

Date	Species	Site	Month	Year	Survey Year	Presence	Data Gap	Season
7/19/2018	FP	14M	7	2018	Year-1		1	Summer
7/19/2018	FP	8A	7	2018	Year-1		1	Summer
7/19/2018	FP	9A	7	2018	Year-1	0	0	Summer
7/19/2018	FP	11A	7	2018	Year-1	1	0	Summer
7/19/2018	FP	13A	7	2018	Year-1	1	0	Summer
7/20/2018	FP	1M	7	2018	Year-1	1	0	Summer
7/20/2018	FP	2M	7	2018	Year-1		1	Summer
7/20/2018	FP	3M	7	2018	Year-1	1	0	Summer
7/20/2018	FP	4M	7	2018	Year-1	1	0	Summer
7/20/2018	FP	5M	7	2018	Year-1		1	Summer
7/20/2018	FP	6M	7	2018	Year-1	1	0	Summer
7/20/2018	FP	7M	7	2018	Year-1	0	0	Summer
7/20/2018	FP	10M	7	2018	Year-1		1	Summer
7/20/2018	FP	12M	7	2018	Year-1	0	0	Summer
7/20/2018	FP	14M	7	2018	Year-1	0	0	Summer
7/20/2018	FP	8A	7	2018	Year-1		1	Summer
7/20/2018	FP	9A	7	2018	Year-1	0	0	Summer
7/20/2018	FP	11A	7	2018	Year-1	1	0	Summer
7/20/2018	FP	13A	7	2018	Year-1	1	0	Summer
7/21/2018	FP	1M	7	2018	Year-1	1	0	Summer
7/21/2018	FP	2M	7	2018	Year-1		1	Summer
7/21/2018	FP	3M	7	2018	Year-1	1	0	Summer
7/21/2018	FP	4M	7	2018	Year-1	1	0	Summer
7/21/2018	FP	5M	7	2018	Year-1		1	Summer
7/21/2018	FP	6M	7	2018	Year-1	1	0	Summer
7/21/2018	FP	7M	7	2018	Year-1	0	0	Summer
7/21/2018	FP	10M	7	2018	Year-1		1	Summer
7/21/2018	FP	12M	7	2018	Year-1	0	0	Summer
7/21/2018	FP	14M	7	2018	Year-1	0	0	Summer
7/21/2018	FP	8A	7	2018	Year-1		1	Summer
7/21/2018	FP	9A	7	2018	Year-1	1	0	Summer
7/21/2018	FP	11A	7	2018	Year-1	1	0	Summer
7/21/2018	FP	13A	7	2018	Year-1	1	0	Summer
7/22/2018	FP	1M	7	2018	Year-1	1	0	Summer
7/22/2018	FP	2M	7	2018	Year-1		1	Summer
7/22/2018	FP	3M	7	2018	Year-1	1	0	Summer
7/22/2018	FP	4M	7	2018	Year-1	1	0	Summer
7/22/2018	FP	5M	7	2018	Year-1		1	Summer
7/22/2018	FP	6M	7	2018	Year-1	1	0	Summer
7/22/2018	FP	7M	7	2018	Year-1	0	0	Summer
7/22/2018	FP	10M	7	2018	Year-1		1	Summer
7/22/2018	FP	12M	7	2018	Year-1	0	0	Summer
7/22/2018	FP	14M	7	2018	Year-1	0	0	Summer
7/22/2018	FP	8A	7	2018	Year-1		1	Summer

Date	Species	Site	Month	Year	Survey Year	Presence	Data Gap	Season
7/22/2018	FP	9A	7	2018	Year-1	0	0	Summer
7/22/2018	FP	11A	7	2018	Year-1	1	0	Summer
7/22/2018	FP	13A	7	2018	Year-1	1	0	Summer
7/23/2018	FP	1M	7	2018	Year-1	0	0	Summer
7/23/2018	FP	2M	7	2018	Year-1		1	Summer
7/23/2018	FP	3M	7	2018	Year-1	1	0	Summer
7/23/2018	FP	4M	7	2018	Year-1	1	0	Summer
7/23/2018	FP	5M	7	2018	Year-1		1	Summer
7/23/2018	FP	6M	7	2018	Year-1	0	0	Summer
7/23/2018	FP	7M	7	2018	Year-1	0	0	Summer
7/23/2018	FP	10M	7	2018	Year-1		1	Summer
7/23/2018	FP	12M	7	2018	Year-1	0	0	Summer
7/23/2018	FP	14M	7	2018	Year-1	0	0	Summer
7/23/2018	FP	8A	7	2018	Year-1		1	Summer
7/23/2018	FP	9A	7	2018	Year-1	0	0	Summer
7/23/2018	FP	11A	7	2018	Year-1	1	0	Summer
7/23/2018	FP	13A	7	2018	Year-1	1	0	Summer
7/24/2018	FP	1M	7	2018	Year-1	1	0	Summer
7/24/2018	FP	2M	7	2018	Year-1		1	Summer
7/24/2018	FP	3M	7	2018	Year-1	1	0	Summer
7/24/2018	FP	4M	7	2018	Year-1	1	0	Summer
7/24/2018	FP	5M	7	2018	Year-1		1	Summer
7/24/2018	FP	6M	7	2018	Year-1	1	0	Summer
7/24/2018	FP	7M	7	2018	Year-1	0	0	Summer
7/24/2018	FP	10M	7	2018	Year-1		1	Summer
7/24/2018	FP	12M	7	2018	Year-1	0	0	Summer
7/24/2018	FP	14M	7	2018	Year-1	0	0	Summer
7/24/2018	FP	8A	7	2018	Year-1		1	Summer
7/24/2018	FP	9A	7	2018	Year-1	1	0	Summer
7/24/2018	FP	11A	7	2018	Year-1	1	0	Summer
7/24/2018	FP	13A	7	2018	Year-1	1	0	Summer
7/25/2018	FP	1M	7	2018	Year-1	1	0	Summer
7/25/2018	FP	2M	7	2018	Year-1		1	Summer
7/25/2018	FP	3M	7	2018	Year-1	1	0	Summer
7/25/2018	FP	4M	7	2018	Year-1	1	0	Summer
7/25/2018	FP	5M	7	2018	Year-1		1	Summer
7/25/2018	FP	6M	7	2018	Year-1	1	0	Summer
7/25/2018	FP	7M	7	2018	Year-1	1	0	Summer
7/25/2018	FP	10M	7	2018	Year-1		1	Summer
7/25/2018	FP	12M	7	2018	Year-1	0	0	Summer
7/25/2018	FP	14M	7	2018	Year-1	0	0	Summer
7/25/2018	FP	8A	7	2018	Year-1		1	Summer
7/25/2018	FP	9A	7	2018	Year-1	1	0	Summer
7/25/2018	FP	11A	7	2018	Year-1	1	0	Summer

Date	Species	Site	Month	Year	Survey Year	Presence	Data Gap	Season
7/25/2018	FP	13A	7	2018	Year-1	1	0	Summer
7/26/2018	FP	1M	7	2018	Year-1	1	0	Summer
7/26/2018	FP	2M	7	2018	Year-1		1	Summer
7/26/2018	FP	3M	7	2018	Year-1	1	0	Summer
7/26/2018	FP	4M	7	2018	Year-1	1	0	Summer
7/26/2018	FP	5M	7	2018	Year-1		1	Summer
7/26/2018	FP	6M	7	2018	Year-1	1	0	Summer
7/26/2018	FP	7M	7	2018	Year-1	0	0	Summer
7/26/2018	FP	10M	7	2018	Year-1		1	Summer
7/26/2018	FP	12M	7	2018	Year-1	0	0	Summer
7/26/2018	FP	14M	7	2018	Year-1	0	0	Summer
7/26/2018	FP	8A	7	2018	Year-1		1	Summer
7/26/2018	FP	9A	7	2018	Year-1	1	0	Summer
7/26/2018	FP	11A	7	2018	Year-1	1	0	Summer
7/26/2018	FP	13A	7	2018	Year-1	1	0	Summer
7/27/2018	FP	1M	7	2018	Year-1	1	0	Summer
7/27/2018	FP	2M	7	2018	Year-1		1	Summer
7/27/2018	FP	3M	7	2018	Year-1	1	0	Summer
7/27/2018	FP	4M	7	2018	Year-1	1	0	Summer
7/27/2018	FP	5M	7	2018	Year-1		1	Summer
7/27/2018	FP	6M	7	2018	Year-1	1	0	Summer
7/27/2018	FP	7M	7	2018	Year-1	1	0	Summer
7/27/2018	FP	10M	7	2018	Year-1		1	Summer
7/27/2018	FP	12M	7	2018	Year-1	0	0	Summer
7/27/2018	FP	14M	7	2018	Year-1	0	0	Summer
7/27/2018	FP	8A	7	2018	Year-1		1	Summer
7/27/2018	FP	9A	7	2018	Year-1	1	0	Summer
7/27/2018	FP	11A	7	2018	Year-1	1	0	Summer
7/27/2018	FP	13A	7	2018	Year-1	1	0	Summer
7/28/2018	FP	1M	7	2018	Year-1	1	0	Summer
7/28/2018	FP	2M	7	2018	Year-1		1	Summer
7/28/2018	FP	3M	7	2018	Year-1	1	0	Summer
7/28/2018	FP	4M	7	2018	Year-1	1	0	Summer
7/28/2018	FP	5M	7	2018	Year-1		1	Summer
7/28/2018	FP	6M	7	2018	Year-1	1	0	Summer
7/28/2018	FP	7M	7	2018	Year-1	0	0	Summer
7/28/2018	FP	10M	7	2018	Year-1		1	Summer
7/28/2018	FP	12M	7	2018	Year-1	0	0	Summer
7/28/2018	FP	14M	7	2018	Year-1	0	0	Summer
7/28/2018	FP	8A	7	2018	Year-1		1	Summer
7/28/2018	FP	9A	7	2018	Year-1	0	0	Summer
7/28/2018	FP	11A	7	2018	Year-1	1	0	Summer
7/28/2018	FP	13A	7	2018	Year-1	1	0	Summer
7/29/2018	FP	1M	7	2018	Year-1	0	0	Summer

Date	Species	Site	Month	Year	Survey Year	Presence	Data Gap	Season
7/29/2018	FP	2M	7	2018	Year-1		1	Summer
7/29/2018	FP	3M	7	2018	Year-1	1	0	Summer
7/29/2018	FP	4M	7	2018	Year-1	1	0	Summer
7/29/2018	FP	5M	7	2018	Year-1		1	Summer
7/29/2018	FP	6M	7	2018	Year-1	1	0	Summer
7/29/2018	FP	7M	7	2018	Year-1	0	0	Summer
7/29/2018	FP	10M	7	2018	Year-1		1	Summer
7/29/2018	FP	12M	7	2018	Year-1	0	0	Summer
7/29/2018	FP	14M	7	2018	Year-1	0	0	Summer
7/29/2018	FP	8A	7	2018	Year-1		1	Summer
7/29/2018	FP	9A	7	2018	Year-1	0	0	Summer
7/29/2018	FP	11A	7	2018	Year-1	1	0	Summer
7/29/2018	FP	13A	7	2018	Year-1	1	0	Summer
7/30/2018	FP	1M	7	2018	Year-1	1	0	Summer
7/30/2018	FP	2M	7	2018	Year-1		1	Summer
7/30/2018	FP	3M	7	2018	Year-1	1	0	Summer
7/30/2018	FP	4M	7	2018	Year-1	1	0	Summer
7/30/2018	FP	5M	7	2018	Year-1		1	Summer
7/30/2018	FP	6M	7	2018	Year-1	1	0	Summer
7/30/2018	FP	7M	7	2018	Year-1	0	0	Summer
7/30/2018	FP	10M	7	2018	Year-1		1	Summer
7/30/2018	FP	12M	7	2018	Year-1	0	0	Summer
7/30/2018	FP	14M	7	2018	Year-1	0	0	Summer
7/30/2018	FP	8A	7	2018	Year-1		1	Summer
7/30/2018	FP	9A	7	2018	Year-1	1	0	Summer
7/30/2018	FP	11A	7	2018	Year-1	1	0	Summer
7/30/2018	FP	13A	7	2018	Year-1	1	0	Summer
7/31/2018	FP	1M	7	2018	Year-1	0	0	Summer
7/31/2018	FP	2M	7	2018	Year-1		1	Summer
7/31/2018	FP	3M	7	2018	Year-1	1	0	Summer
7/31/2018	FP	4M	7	2018	Year-1	1	0	Summer
7/31/2018	FP	5M	7	2018	Year-1		1	Summer
7/31/2018	FP	6M	7	2018	Year-1	1	0	Summer
7/31/2018	FP	7M	7	2018	Year-1	0	0	Summer
7/31/2018	FP	10M	7	2018	Year-1		1	Summer
7/31/2018	FP	12M	7	2018	Year-1	0	0	Summer
7/31/2018	FP	14M	7	2018	Year-1	0	0	Summer
7/31/2018	FP	8A	7	2018	Year-1		1	Summer
7/31/2018	FP	9A	7	2018	Year-1	0	0	Summer
7/31/2018	FP	11A	7	2018	Year-1	1	0	Summer
7/31/2018	FP	13A	7	2018	Year-1	1	0	Summer
8/1/2018	FP	1M	8	2018	Year-1	0	0	Summer
8/1/2018	FP	2M	8	2018	Year-1		1	Summer
8/1/2018	FP	3M	8	2018	Year-1	1	0	Summer

Date	Species	Site	Month	Year	Survey Year	Presence	Data Gap	Season
8/1/2018	FP	4M	8	2018	Year-1	1	0	Summer
8/1/2018	FP	5M	8	2018	Year-1		1	Summer
8/1/2018	FP	6M	8	2018	Year-1	1	0	Summer
8/1/2018	FP	7M	8	2018	Year-1	0	0	Summer
8/1/2018	FP	10M	8	2018	Year-1		1	Summer
8/1/2018	FP	12M	8	2018	Year-1	0	0	Summer
8/1/2018	FP	14M	8	2018	Year-1	0	0	Summer
8/1/2018	FP	8A	8	2018	Year-1		1	Summer
8/1/2018	FP	9A	8	2018	Year-1	0	0	Summer
8/1/2018	FP	11A	8	2018	Year-1	1	0	Summer
8/1/2018	FP	13A	8	2018	Year-1	1	0	Summer
8/2/2018	FP	1M	8	2018	Year-1	0	0	Summer
8/2/2018	FP	2M	8	2018	Year-1		1	Summer
8/2/2018	FP	3M	8	2018	Year-1	1	0	Summer
8/2/2018	FP	4M	8	2018	Year-1	1	0	Summer
8/2/2018	FP	5M	8	2018	Year-1		1	Summer
8/2/2018	FP	6M	8	2018	Year-1	1	0	Summer
8/2/2018	FP	7M	8	2018	Year-1	0	0	Summer
8/2/2018	FP	10M	8	2018	Year-1		1	Summer
8/2/2018	FP	12M	8	2018	Year-1	0	0	Summer
8/2/2018	FP	14M	8	2018	Year-1	0	0	Summer
8/2/2018	FP	8A	8	2018	Year-1		1	Summer
8/2/2018	FP	9A	8	2018	Year-1	0	0	Summer
8/2/2018	FP	11A	8	2018	Year-1	1	0	Summer
8/2/2018	FP	13A	8	2018	Year-1	1	0	Summer
8/3/2018	FP	1M	8	2018	Year-1	0	0	Summer
8/3/2018	FP	2M	8	2018	Year-1		1	Summer
8/3/2018	FP	3M	8	2018	Year-1	1	0	Summer
8/3/2018	FP	4M	8	2018	Year-1	1	0	Summer
8/3/2018	FP	5M	8	2018	Year-1		1	Summer
8/3/2018	FP	6M	8	2018	Year-1	1	0	Summer
8/3/2018	FP	7M	8	2018	Year-1	0	0	Summer
8/3/2018	FP	10M	8	2018	Year-1		1	Summer
8/3/2018	FP	12M	8	2018	Year-1	0	0	Summer
8/3/2018	FP	14M	8	2018	Year-1	0	0	Summer
8/3/2018	FP	8A	8	2018	Year-1		1	Summer
8/3/2018	FP	9A	8	2018	Year-1	0	0	Summer
8/3/2018	FP	11A	8	2018	Year-1	1	0	Summer
8/3/2018	FP	13A	8	2018	Year-1	1	0	Summer
8/4/2018	FP	1M	8	2018	Year-1	1	0	Summer
8/4/2018	FP	2M	8	2018	Year-1		1	Summer
8/4/2018	FP	3M	8	2018	Year-1	1	0	Summer
8/4/2018	FP	4M	8	2018	Year-1	1	0	Summer
8/4/2018	FP	5M	8	2018	Year-1		1	Summer

Date	Species	Site	Month	Year	Survey Year	Presence	Data Gap	Season
8/4/2018	FP	6M	8	2018	Year-1	1	0	Summer
8/4/2018	FP	7M	8	2018	Year-1	0	0	Summer
8/4/2018	FP	10M	8	2018	Year-1		1	Summer
8/4/2018	FP	12M	8	2018	Year-1	0	0	Summer
8/4/2018	FP	14M	8	2018	Year-1	0	0	Summer
8/4/2018	FP	8A	8	2018	Year-1		1	Summer
8/4/2018	FP	9A	8	2018	Year-1	0	0	Summer
8/4/2018	FP	11A	8	2018	Year-1	1	0	Summer
8/4/2018	FP	13A	8	2018	Year-1	1	0	Summer
8/5/2018	FP	1M	8	2018	Year-1	0	0	Summer
8/5/2018	FP	2M	8	2018	Year-1		1	Summer
8/5/2018	FP	3M	8	2018	Year-1	1	0	Summer
8/5/2018	FP	4M	8	2018	Year-1	1	0	Summer
8/5/2018	FP	5M	8	2018	Year-1		1	Summer
8/5/2018	FP	6M	8	2018	Year-1	1	0	Summer
8/5/2018	FP	7M	8	2018	Year-1	0	0	Summer
8/5/2018	FP	10M	8	2018	Year-1		1	Summer
8/5/2018	FP	12M	8	2018	Year-1	0	0	Summer
8/5/2018	FP	14M	8	2018	Year-1	0	0	Summer
8/5/2018	FP	8A	8	2018	Year-1		1	Summer
8/5/2018	FP	9A	8	2018	Year-1	0	0	Summer
8/5/2018	FP	11A	8	2018	Year-1	1	0	Summer
8/5/2018	FP	13A	8	2018	Year-1	1	0	Summer
8/6/2018	FP	1M	8	2018	Year-1	1	0	Summer
8/6/2018	FP	2M	8	2018	Year-1		1	Summer
8/6/2018	FP	3M	8	2018	Year-1	1	0	Summer
8/6/2018	FP	4M	8	2018	Year-1	1	0	Summer
8/6/2018	FP	5M	8	2018	Year-1		1	Summer
8/6/2018	FP	6M	8	2018	Year-1	0	0	Summer
8/6/2018	FP	7M	8	2018	Year-1	0	0	Summer
8/6/2018	FP	10M	8	2018	Year-1		1	Summer
8/6/2018	FP	12M	8	2018	Year-1	0	0	Summer
8/6/2018	FP	14M	8	2018	Year-1	0	0	Summer
8/6/2018	FP	8A	8	2018	Year-1		1	Summer
8/6/2018	FP	9A	8	2018	Year-1	1	0	Summer
8/6/2018	FP	11A	8	2018	Year-1	1	0	Summer
8/6/2018	FP	13A	8	2018	Year-1	1	0	Summer
8/7/2018	FP	1M	8	2018	Year-1	1	0	Summer
8/7/2018	FP	2M	8	2018	Year-1		1	Summer
8/7/2018	FP	3M	8	2018	Year-1	1	0	Summer
8/7/2018	FP	4M	8	2018	Year-1	1	0	Summer
8/7/2018	FP	5M	8	2018	Year-1		1	Summer
8/7/2018	FP	6M	8	2018	Year-1	1	0	Summer
8/7/2018	FP	7M	8	2018	Year-1	0	0	Summer

Date	Species	Site	Month	Year	Survey Year	Presence	Data Gap	Season
8/7/2018	FP	10M	8	2018	Year-1		1	Summer
8/7/2018	FP	12M	8	2018	Year-1	0	0	Summer
8/7/2018	FP	14M	8	2018	Year-1	0	0	Summer
8/7/2018	FP	8A	8	2018	Year-1		1	Summer
8/7/2018	FP	9A	8	2018	Year-1	1	0	Summer
8/7/2018	FP	11A	8	2018	Year-1	1	0	Summer
8/7/2018	FP	13A	8	2018	Year-1	1	0	Summer
8/8/2018	FP	1M	8	2018	Year-1	1	0	Summer
8/8/2018	FP	2M	8	2018	Year-1		1	Summer
8/8/2018	FP	3M	8	2018	Year-1	1	0	Summer
8/8/2018	FP	4M	8	2018	Year-1	1	0	Summer
8/8/2018	FP	5M	8	2018	Year-1		1	Summer
8/8/2018	FP	6M	8	2018	Year-1	0	0	Summer
8/8/2018	FP	7M	8	2018	Year-1	0	0	Summer
8/8/2018	FP	10M	8	2018	Year-1		1	Summer
8/8/2018	FP	12M	8	2018	Year-1		1	Summer
8/8/2018	FP	14M	8	2018	Year-1	0	0	Summer
8/8/2018	FP	8A	8	2018	Year-1		1	Summer
8/8/2018	FP	9A	8	2018	Year-1	1	0	Summer
8/8/2018	FP	11A	8	2018	Year-1	1	0	Summer
8/8/2018	FP	13A	8	2018	Year-1	1	0	Summer
8/9/2018	FP	1M	8	2018	Year-1	1	0	Summer
8/9/2018	FP	2M	8	2018	Year-1		1	Summer
8/9/2018	FP	3M	8	2018	Year-1	1	0	Summer
8/9/2018	FP	4M	8	2018	Year-1	1	0	Summer
8/9/2018	FP	5M	8	2018	Year-1		1	Summer
8/9/2018	FP	6M	8	2018	Year-1	1	0	Summer
8/9/2018	FP	7M	8	2018	Year-1	0	0	Summer
8/9/2018	FP	10M	8	2018	Year-1		1	Summer
8/9/2018	FP	12M	8	2018	Year-1		1	Summer
8/9/2018	FP	14M	8	2018	Year-1	0	0	Summer
8/9/2018	FP	8A	8	2018	Year-1		1	Summer
8/9/2018	FP	9A	8	2018	Year-1	1	0	Summer
8/9/2018	FP	11A	8	2018	Year-1	1	0	Summer
8/9/2018	FP	13A	8	2018	Year-1	1	0	Summer
8/10/2018	FP	1M	8	2018	Year-1	1	0	Summer
8/10/2018	FP	2M	8	2018	Year-1		1	Summer
8/10/2018	FP	3M	8	2018	Year-1	1	0	Summer
8/10/2018	FP	4M	8	2018	Year-1	1	0	Summer
8/10/2018	FP	5M	8	2018	Year-1		1	Summer
8/10/2018	FP	6M	8	2018	Year-1	1	0	Summer
8/10/2018	FP	7M	8	2018	Year-1	0	0	Summer
8/10/2018	FP	10M	8	2018	Year-1		1	Summer
8/10/2018	FP	12M	8	2018	Year-1		1	Summer

Date	Species	Site	Month	Year	Survey Year	Presence	Data Gap	Season
8/10/2018	FP	14M	8	2018	Year-1	0	0	Summer
8/10/2018	FP	8A	8	2018	Year-1		1	Summer
8/10/2018	FP	9A	8	2018	Year-1	1	0	Summer
8/10/2018	FP	11A	8	2018	Year-1	1	0	Summer
8/10/2018	FP	13A	8	2018	Year-1	1	0	Summer
8/11/2018	FP	1M	8	2018	Year-1	1	0	Summer
8/11/2018	FP	2M	8	2018	Year-1		1	Summer
8/11/2018	FP	3M	8	2018	Year-1	1	0	Summer
8/11/2018	FP	4M	8	2018	Year-1	1	0	Summer
8/11/2018	FP	5M	8	2018	Year-1		1	Summer
8/11/2018	FP	6M	8	2018	Year-1	1	0	Summer
8/11/2018	FP	7M	8	2018	Year-1	0	0	Summer
8/11/2018	FP	10M	8	2018	Year-1		1	Summer
8/11/2018	FP	12M	8	2018	Year-1		1	Summer
8/11/2018	FP	14M	8	2018	Year-1	0	0	Summer
8/11/2018	FP	8A	8	2018	Year-1		1	Summer
8/11/2018	FP	9A	8	2018	Year-1	1	0	Summer
8/11/2018	FP	11A	8	2018	Year-1	1	0	Summer
8/11/2018	FP	13A	8	2018	Year-1	1	0	Summer
8/12/2018	FP	1M	8	2018	Year-1	1	0	Summer
8/12/2018	FP	2M	8	2018	Year-1		1	Summer
8/12/2018	FP	3M	8	2018	Year-1	1	0	Summer
8/12/2018	FP	4M	8	2018	Year-1	1	0	Summer
8/12/2018	FP	5M	8	2018	Year-1		1	Summer
8/12/2018	FP	6M	8	2018	Year-1	1	0	Summer
8/12/2018	FP	7M	8	2018	Year-1	0	0	Summer
8/12/2018	FP	10M	8	2018	Year-1		1	Summer
8/12/2018	FP	12M	8	2018	Year-1		1	Summer
8/12/2018	FP	14M	8	2018	Year-1	0	0	Summer
8/12/2018	FP	8A	8	2018	Year-1		1	Summer
8/12/2018	FP	9A	8	2018	Year-1	0	0	Summer
8/12/2018	FP	11A	8	2018	Year-1	1	0	Summer
8/12/2018	FP	13A	8	2018	Year-1	1	0	Summer
8/13/2018	FP	1M	8	2018	Year-1	1	0	Summer
8/13/2018	FP	2M	8	2018	Year-1		1	Summer
8/13/2018	FP	3M	8	2018	Year-1	1	0	Summer
8/13/2018	FP	4M	8	2018	Year-1	1	0	Summer
8/13/2018	FP	5M	8	2018	Year-1		1	Summer
8/13/2018	FP	6M	8	2018	Year-1	1	0	Summer
8/13/2018	FP	7M	8	2018	Year-1	0	0	Summer
8/13/2018	FP	10M	8	2018	Year-1		1	Summer
8/13/2018	FP	12M	8	2018	Year-1		1	Summer
8/13/2018	FP	14M	8	2018	Year-1	0	0	Summer
8/13/2018	FP	8A	8	2018	Year-1		1	Summer

Date	Species	Site	Month	Year	Survey Year	Presence	Data Gap	Season
8/13/2018	FP	9A	8	2018	Year-1	1	0	Summer
8/13/2018	FP	11A	8	2018	Year-1	1	0	Summer
8/13/2018	FP	13A	8	2018	Year-1	1	0	Summer
8/14/2018	FP	1M	8	2018	Year-1	1	0	Summer
8/14/2018	FP	2M	8	2018	Year-1		1	Summer
8/14/2018	FP	3M	8	2018	Year-1	1	0	Summer
8/14/2018	FP	4M	8	2018	Year-1	0	0	Summer
8/14/2018	FP	5M	8	2018	Year-1		1	Summer
8/14/2018	FP	6M	8	2018	Year-1	1	0	Summer
8/14/2018	FP	7M	8	2018	Year-1	0	0	Summer
8/14/2018	FP	10M	8	2018	Year-1		1	Summer
8/14/2018	FP	12M	8	2018	Year-1		1	Summer
8/14/2018	FP	14M	8	2018	Year-1	0	0	Summer
8/14/2018	FP	8A	8	2018	Year-1		1	Summer
8/14/2018	FP	9A	8	2018	Year-1	1	0	Summer
8/14/2018	FP	11A	8	2018	Year-1	1	0	Summer
8/14/2018	FP	13A	8	2018	Year-1	1	0	Summer
8/15/2018	FP	1M	8	2018	Year-1	1	0	Summer
8/15/2018	FP	2M	8	2018	Year-1		1	Summer
8/15/2018	FP	3M	8	2018	Year-1	1	0	Summer
8/15/2018	FP	4M	8	2018	Year-1	1	0	Summer
8/15/2018	FP	5M	8	2018	Year-1		1	Summer
8/15/2018	FP	6M	8	2018	Year-1	1	0	Summer
8/15/2018	FP	7M	8	2018	Year-1	0	0	Summer
8/15/2018	FP	10M	8	2018	Year-1		1	Summer
8/15/2018	FP	12M	8	2018	Year-1		1	Summer
8/15/2018	FP	14M	8	2018	Year-1	0	0	Summer
8/15/2018	FP	8A	8	2018	Year-1		1	Summer
8/15/2018	FP	9A	8	2018	Year-1	1	0	Summer
8/15/2018	FP	11A	8	2018	Year-1	1	0	Summer
8/15/2018	FP	13A	8	2018	Year-1	1	0	Summer
8/16/2018	FP	1M	8	2018	Year-1	1	0	Summer
8/16/2018	FP	2M	8	2018	Year-1		1	Summer
8/16/2018	FP	3M	8	2018	Year-1	1	0	Summer
8/16/2018	FP	4M	8	2018	Year-1	1	0	Summer
8/16/2018	FP	5M	8	2018	Year-1		1	Summer
8/16/2018	FP	6M	8	2018	Year-1	1	0	Summer
8/16/2018	FP	7M	8	2018	Year-1	0	0	Summer
8/16/2018	FP	10M	8	2018	Year-1		1	Summer
8/16/2018	FP	12M	8	2018	Year-1		1	Summer
8/16/2018	FP	14M	8	2018	Year-1	0	0	Summer
8/16/2018	FP	8A	8	2018	Year-1		1	Summer
8/16/2018	FP	9A	8	2018	Year-1	1	0	Summer
8/16/2018	FP	11A	8	2018	Year-1	1	0	Summer

Date	Species	Site	Month	Year	Survey Year	Presence	Data Gap	Season
8/16/2018	FP	13A	8	2018	Year-1	1	0	Summer
8/17/2018	FP	1M	8	2018	Year-1	1	0	Summer
8/17/2018	FP	2M	8	2018	Year-1		1	Summer
8/17/2018	FP	3M	8	2018	Year-1	1	0	Summer
8/17/2018	FP	4M	8	2018	Year-1	1	0	Summer
8/17/2018	FP	5M	8	2018	Year-1		1	Summer
8/17/2018	FP	6M	8	2018	Year-1	1	0	Summer
8/17/2018	FP	7M	8	2018	Year-1	0	0	Summer
8/17/2018	FP	10M	8	2018	Year-1		1	Summer
8/17/2018	FP	12M	8	2018	Year-1		1	Summer
8/17/2018	FP	14M	8	2018	Year-1	0	0	Summer
8/17/2018	FP	8A	8	2018	Year-1		1	Summer
8/17/2018	FP	9A	8	2018	Year-1	1	0	Summer
8/17/2018	FP	11A	8	2018	Year-1	1	0	Summer
8/17/2018	FP	13A	8	2018	Year-1	1	0	Summer
8/18/2018	FP	1M	8	2018	Year-1	1	0	Summer
8/18/2018	FP	2M	8	2018	Year-1		1	Summer
8/18/2018	FP	3M	8	2018	Year-1	1	0	Summer
8/18/2018	FP	4M	8	2018	Year-1	1	0	Summer
8/18/2018	FP	5M	8	2018	Year-1		1	Summer
8/18/2018	FP	6M	8	2018	Year-1	1	0	Summer
8/18/2018	FP	7M	8	2018	Year-1	0	0	Summer
8/18/2018	FP	10M	8	2018	Year-1		1	Summer
8/18/2018	FP	12M	8	2018	Year-1		1	Summer
8/18/2018	FP	14M	8	2018	Year-1	0	0	Summer
8/18/2018	FP	8A	8	2018	Year-1		1	Summer
8/18/2018	FP	9A	8	2018	Year-1	1	0	Summer
8/18/2018	FP	11A	8	2018	Year-1	1	0	Summer
8/18/2018	FP	13A	8	2018	Year-1	1	0	Summer
8/19/2018	FP	1M	8	2018	Year-1	1	0	Summer
8/19/2018	FP	2M	8	2018	Year-1		1	Summer
8/19/2018	FP	3M	8	2018	Year-1	1	0	Summer
8/19/2018	FP	4M	8	2018	Year-1	1	0	Summer
8/19/2018	FP	5M	8	2018	Year-1		1	Summer
8/19/2018	FP	6M	8	2018	Year-1	1	0	Summer
8/19/2018	FP	7M	8	2018	Year-1	0	0	Summer
8/19/2018	FP	10M	8	2018	Year-1		1	Summer
8/19/2018	FP	12M	8	2018	Year-1		1	Summer
8/19/2018	FP	14M	8	2018	Year-1	0	0	Summer
8/19/2018	FP	8A	8	2018	Year-1		1	Summer
8/19/2018	FP	9A	8	2018	Year-1	1	0	Summer
8/19/2018	FP	11A	8	2018	Year-1	1	0	Summer
8/19/2018	FP	13A	8	2018	Year-1	1	0	Summer
8/20/2018	FP	1M	8	2018	Year-1	1	0	Summer

Date	Species	Site	Month	Year	Survey Year	Presence	Data Gap	Season
8/20/2018	FP	2M	8	2018	Year-1		1	Summer
8/20/2018	FP	3M	8	2018	Year-1	1	0	Summer
8/20/2018	FP	4M	8	2018	Year-1	1	0	Summer
8/20/2018	FP	5M	8	2018	Year-1		1	Summer
8/20/2018	FP	6M	8	2018	Year-1	1	0	Summer
8/20/2018	FP	7M	8	2018	Year-1	0	0	Summer
8/20/2018	FP	10M	8	2018	Year-1		1	Summer
8/20/2018	FP	12M	8	2018	Year-1		1	Summer
8/20/2018	FP	14M	8	2018	Year-1	0	0	Summer
8/20/2018	FP	8A	8	2018	Year-1		1	Summer
8/20/2018	FP	9A	8	2018	Year-1	1	0	Summer
8/20/2018	FP	11A	8	2018	Year-1	1	0	Summer
8/20/2018	FP	13A	8	2018	Year-1	1	0	Summer
8/21/2018	FP	1M	8	2018	Year-1	1	0	Summer
8/21/2018	FP	2M	8	2018	Year-1		1	Summer
8/21/2018	FP	3M	8	2018	Year-1	1	0	Summer
8/21/2018	FP	4M	8	2018	Year-1	1	0	Summer
8/21/2018	FP	5M	8	2018	Year-1		1	Summer
8/21/2018	FP	6M	8	2018	Year-1	1	0	Summer
8/21/2018	FP	7M	8	2018	Year-1	1	0	Summer
8/21/2018	FP	10M	8	2018	Year-1		1	Summer
8/21/2018	FP	12M	8	2018	Year-1		1	Summer
8/21/2018	FP	14M	8	2018	Year-1	0	0	Summer
8/21/2018	FP	8A	8	2018	Year-1		1	Summer
8/21/2018	FP	9A	8	2018	Year-1	1	0	Summer
8/21/2018	FP	11A	8	2018	Year-1	1	0	Summer
8/21/2018	FP	13A	8	2018	Year-1	1	0	Summer
8/22/2018	FP	1M	8	2018	Year-1	0	0	Summer
8/22/2018	FP	2M	8	2018	Year-1		1	Summer
8/22/2018	FP	3M	8	2018	Year-1	1	0	Summer
8/22/2018	FP	4M	8	2018	Year-1	1	0	Summer
8/22/2018	FP	5M	8	2018	Year-1		1	Summer
8/22/2018	FP	6M	8	2018	Year-1	1	0	Summer
8/22/2018	FP	7M	8	2018	Year-1	1	0	Summer
8/22/2018	FP	10M	8	2018	Year-1		1	Summer
8/22/2018	FP	12M	8	2018	Year-1		1	Summer
8/22/2018	FP	14M	8	2018	Year-1	0	0	Summer
8/22/2018	FP	8A	8	2018	Year-1		1	Summer
8/22/2018	FP	9A	8	2018	Year-1	1	0	Summer
8/22/2018	FP	11A	8	2018	Year-1	1	0	Summer
8/22/2018	FP	13A	8	2018	Year-1	1	0	Summer
8/23/2018	FP	1M	8	2018	Year-1	1	0	Summer
8/23/2018	FP	2M	8	2018	Year-1		1	Summer
8/23/2018	FP	3M	8	2018	Year-1	1	0	Summer

Date	Species	Site	Month	Year	Survey Year	Presence	Data Gap	Season
8/23/2018	FP	4M	8	2018	Year-1	1	0	Summer
8/23/2018	FP	5M	8	2018	Year-1		1	Summer
8/23/2018	FP	6M	8	2018	Year-1	1	0	Summer
8/23/2018	FP	7M	8	2018	Year-1	1	0	Summer
8/23/2018	FP	10M	8	2018	Year-1		1	Summer
8/23/2018	FP	12M	8	2018	Year-1		1	Summer
8/23/2018	FP	14M	8	2018	Year-1	0	0	Summer
8/23/2018	FP	8A	8	2018	Year-1		1	Summer
8/23/2018	FP	9A	8	2018	Year-1	1	0	Summer
8/23/2018	FP	11A	8	2018	Year-1	1	0	Summer
8/23/2018	FP	13A	8	2018	Year-1	1	0	Summer
8/24/2018	FP	1M	8	2018	Year-1	1	0	Summer
8/24/2018	FP	2M	8	2018	Year-1		1	Summer
8/24/2018	FP	3M	8	2018	Year-1	1	0	Summer
8/24/2018	FP	4M	8	2018	Year-1	1	0	Summer
8/24/2018	FP	5M	8	2018	Year-1		1	Summer
8/24/2018	FP	6M	8	2018	Year-1	1	0	Summer
8/24/2018	FP	7M	8	2018	Year-1	1	0	Summer
8/24/2018	FP	10M	8	2018	Year-1		1	Summer
8/24/2018	FP	12M	8	2018	Year-1		1	Summer
8/24/2018	FP	14M	8	2018	Year-1	0	0	Summer
8/24/2018	FP	8A	8	2018	Year-1		1	Summer
8/24/2018	FP	9A	8	2018	Year-1	1	0	Summer
8/24/2018	FP	11A	8	2018	Year-1	1	0	Summer
8/24/2018	FP	13A	8	2018	Year-1	1	0	Summer
8/25/2018	FP	1M	8	2018	Year-1	1	0	Summer
8/25/2018	FP	2M	8	2018	Year-1		1	Summer
8/25/2018	FP	3M	8	2018	Year-1	1	0	Summer
8/25/2018	FP	4M	8	2018	Year-1	1	0	Summer
8/25/2018	FP	5M	8	2018	Year-1		1	Summer
8/25/2018	FP	6M	8	2018	Year-1	1	0	Summer
8/25/2018	FP	7M	8	2018	Year-1	0	0	Summer
8/25/2018	FP	10M	8	2018	Year-1		1	Summer
8/25/2018	FP	12M	8	2018	Year-1		1	Summer
8/25/2018	FP	14M	8	2018	Year-1	0	0	Summer
8/25/2018	FP	8A	8	2018	Year-1		1	Summer
8/25/2018	FP	9A	8	2018	Year-1	1	0	Summer
8/25/2018	FP	11A	8	2018	Year-1	1	0	Summer
8/25/2018	FP	13A	8	2018	Year-1	1	0	Summer
8/26/2018	FP	1M	8	2018	Year-1	1	0	Summer
8/26/2018	FP	2M	8	2018	Year-1		1	Summer
8/26/2018	FP	3M	8	2018	Year-1	1	0	Summer
8/26/2018	FP	4M	8	2018	Year-1	1	0	Summer
8/26/2018	FP	5M	8	2018	Year-1		1	Summer

Date	Species	Site	Month	Year	Survey Year	Presence	Data Gap	Season
8/26/2018	FP	6M	8	2018	Year-1	1	0	Summer
8/26/2018	FP	7M	8	2018	Year-1	0	0	Summer
8/26/2018	FP	10M	8	2018	Year-1		1	Summer
8/26/2018	FP	12M	8	2018	Year-1		1	Summer
8/26/2018	FP	14M	8	2018	Year-1	0	0	Summer
8/26/2018	FP	8A	8	2018	Year-1		1	Summer
8/26/2018	FP	9A	8	2018	Year-1	1	0	Summer
8/26/2018	FP	11A	8	2018	Year-1	1	0	Summer
8/26/2018	FP	13A	8	2018	Year-1	1	0	Summer
8/27/2018	FP	1M	8	2018	Year-1	1	0	Summer
8/27/2018	FP	2M	8	2018	Year-1		1	Summer
8/27/2018	FP	3M	8	2018	Year-1	1	0	Summer
8/27/2018	FP	4M	8	2018	Year-1	1	0	Summer
8/27/2018	FP	5M	8	2018	Year-1		1	Summer
8/27/2018	FP	6M	8	2018	Year-1	1	0	Summer
8/27/2018	FP	7M	8	2018	Year-1	0	0	Summer
8/27/2018	FP	10M	8	2018	Year-1		1	Summer
8/27/2018	FP	12M	8	2018	Year-1		1	Summer
8/27/2018	FP	14M	8	2018	Year-1	0	0	Summer
8/27/2018	FP	8A	8	2018	Year-1		1	Summer
8/27/2018	FP	9A	8	2018	Year-1	1	0	Summer
8/27/2018	FP	11A	8	2018	Year-1	1	0	Summer
8/27/2018	FP	13A	8	2018	Year-1	1	0	Summer
8/28/2018	FP	1M	8	2018	Year-1	1	0	Summer
8/28/2018	FP	2M	8	2018	Year-1		1	Summer
8/28/2018	FP	3M	8	2018	Year-1	1	0	Summer
8/28/2018	FP	4M	8	2018	Year-1	1	0	Summer
8/28/2018	FP	5M	8	2018	Year-1		1	Summer
8/28/2018	FP	6M	8	2018	Year-1	1	0	Summer
8/28/2018	FP	7M	8	2018	Year-1	0	0	Summer
8/28/2018	FP	10M	8	2018	Year-1		1	Summer
8/28/2018	FP	12M	8	2018	Year-1		1	Summer
8/28/2018	FP	14M	8	2018	Year-1	0	0	Summer
8/28/2018	FP	8A	8	2018	Year-1		1	Summer
8/28/2018	FP	9A	8	2018	Year-1	1	0	Summer
8/28/2018	FP	11A	8	2018	Year-1	1	0	Summer
8/28/2018	FP	13A	8	2018	Year-1	1	0	Summer
8/29/2018	FP	1M	8	2018	Year-1	1	0	Summer
8/29/2018	FP	2M	8	2018	Year-1		1	Summer
8/29/2018	FP	3M	8	2018	Year-1	1	0	Summer
8/29/2018	FP	4M	8	2018	Year-1	1	0	Summer
8/29/2018	FP	5M	8	2018	Year-1		1	Summer
8/29/2018	FP	6M	8	2018	Year-1	1	0	Summer
8/29/2018	FP	7M	8	2018	Year-1	0	0	Summer

Date	Species	Site	Month	Year	Survey Year	Presence	Data Gap	Season
8/29/2018	FP	10M	8	2018	Year-1		1	Summer
8/29/2018	FP	12M	8	2018	Year-1		1	Summer
8/29/2018	FP	14M	8	2018	Year-1	0	0	Summer
8/29/2018	FP	8A	8	2018	Year-1		1	Summer
8/29/2018	FP	9A	8	2018	Year-1	1	0	Summer
8/29/2018	FP	11A	8	2018	Year-1	1	0	Summer
8/29/2018	FP	13A	8	2018	Year-1	1	0	Summer
8/30/2018	FP	1M	8	2018	Year-1	1	0	Summer
8/30/2018	FP	2M	8	2018	Year-1		1	Summer
8/30/2018	FP	3M	8	2018	Year-1	1	0	Summer
8/30/2018	FP	4M	8	2018	Year-1	1	0	Summer
8/30/2018	FP	5M	8	2018	Year-1		1	Summer
8/30/2018	FP	6M	8	2018	Year-1	1	0	Summer
8/30/2018	FP	7M	8	2018	Year-1	0	0	Summer
8/30/2018	FP	10M	8	2018	Year-1		1	Summer
8/30/2018	FP	12M	8	2018	Year-1		1	Summer
8/30/2018	FP	14M	8	2018	Year-1	0	0	Summer
8/30/2018	FP	8A	8	2018	Year-1		1	Summer
8/30/2018	FP	9A	8	2018	Year-1	0	0	Summer
8/30/2018	FP	11A	8	2018	Year-1	1	0	Summer
8/30/2018	FP	13A	8	2018	Year-1	1	0	Summer
8/31/2018	FP	1M	8	2018	Year-1	0	0	Summer
8/31/2018	FP	2M	8	2018	Year-1		1	Summer
8/31/2018	FP	3M	8	2018	Year-1	0	0	Summer
8/31/2018	FP	4M	8	2018	Year-1	0	0	Summer
8/31/2018	FP	5M	8	2018	Year-1		1	Summer
8/31/2018	FP	6M	8	2018	Year-1	0	0	Summer
8/31/2018	FP	7M	8	2018	Year-1	0	0	Summer
8/31/2018	FP	10M	8	2018	Year-1		1	Summer
8/31/2018	FP	12M	8	2018	Year-1		1	Summer
8/31/2018	FP	14M	8	2018	Year-1	0	0	Summer
8/31/2018	FP	8A	8	2018	Year-1		1	Summer
8/31/2018	FP	9A	8	2018	Year-1	0	0	Summer
8/31/2018	FP	11A	8	2018	Year-1	1	0	Summer
8/31/2018	FP	13A	8	2018	Year-1	1	0	Summer
9/1/2018	FP	1M	9	2018	Year-1	1	0	Fall
9/1/2018	FP	2M	9	2018	Year-1		1	Fall
9/1/2018	FP	3M	9	2018	Year-1	1	0	Fall
9/1/2018	FP	4M	9	2018	Year-1	1	0	Fall
9/1/2018	FP	5M	9	2018	Year-1		1	Fall
9/1/2018	FP	6M	9	2018	Year-1	1	0	Fall
9/1/2018	FP	7M	9	2018	Year-1	1	0	Fall
9/1/2018	FP	10M	9	2018	Year-1		1	Fall
9/1/2018	FP	12M	9	2018	Year-1		1	Fall

Date	Species	Site	Month	Year	Survey Year	Presence	Data Gap	Season
9/1/2018	FP	14M	9	2018	Year-1	0	0	Fall
9/1/2018	FP	8A	9	2018	Year-1		1	Fall
9/1/2018	FP	9A	9	2018	Year-1	0	0	Fall
9/1/2018	FP	11A	9	2018	Year-1	1	0	Fall
9/1/2018	FP	13A	9	2018	Year-1	1	0	Fall
9/2/2018	FP	1M	9	2018	Year-1	1	0	Fall
9/2/2018	FP	2M	9	2018	Year-1		1	Fall
9/2/2018	FP	3M	9	2018	Year-1	1	0	Fall
9/2/2018	FP	4M	9	2018	Year-1	1	0	Fall
9/2/2018	FP	5M	9	2018	Year-1		1	Fall
9/2/2018	FP	6M	9	2018	Year-1	1	0	Fall
9/2/2018	FP	7M	9	2018	Year-1	1	0	Fall
9/2/2018	FP	10M	9	2018	Year-1		1	Fall
9/2/2018	FP	12M	9	2018	Year-1		1	Fall
9/2/2018	FP	14M	9	2018	Year-1	0	0	Fall
9/2/2018	FP	8A	9	2018	Year-1		1	Fall
9/2/2018	FP	9A	9	2018	Year-1	1	0	Fall
9/2/2018	FP	11A	9	2018	Year-1	1	0	Fall
9/2/2018	FP	13A	9	2018	Year-1	1	0	Fall
9/3/2018	FP	1M	9	2018	Year-1	1	0	Fall
9/3/2018	FP	2M	9	2018	Year-1		1	Fall
9/3/2018	FP	3M	9	2018	Year-1	1	0	Fall
9/3/2018	FP	4M	9	2018	Year-1	1	0	Fall
9/3/2018	FP	5M	9	2018	Year-1		1	Fall
9/3/2018	FP	6M	9	2018	Year-1	1	0	Fall
9/3/2018	FP	7M	9	2018	Year-1	0	0	Fall
9/3/2018	FP	10M	9	2018	Year-1		1	Fall
9/3/2018	FP	12M	9	2018	Year-1		1	Fall
9/3/2018	FP	14M	9	2018	Year-1	0	0	Fall
9/3/2018	FP	8A	9	2018	Year-1		1	Fall
9/3/2018	FP	9A	9	2018	Year-1	1	0	Fall
9/3/2018	FP	11A	9	2018	Year-1	1	0	Fall
9/3/2018	FP	13A	9	2018	Year-1	1	0	Fall
9/4/2018	FP	1M	9	2018	Year-1	1	0	Fall
9/4/2018	FP	2M	9	2018	Year-1		1	Fall
9/4/2018	FP	3M	9	2018	Year-1	1	0	Fall
9/4/2018	FP	4M	9	2018	Year-1	1	0	Fall
9/4/2018	FP	5M	9	2018	Year-1		1	Fall
9/4/2018	FP	6M	9	2018	Year-1	1	0	Fall
9/4/2018	FP	7M	9	2018	Year-1	1	0	Fall
9/4/2018	FP	10M	9	2018	Year-1		1	Fall
9/4/2018	FP	12M	9	2018	Year-1		1	Fall
9/4/2018	FP	14M	9	2018	Year-1	0	0	Fall
9/4/2018	FP	8A	9	2018	Year-1		1	Fall

Date	Species	Site	Month	Year	Survey Year	Presence	Data Gap	Season
9/4/2018	FP	9A	9	2018	Year-1	1	0	Fall
9/4/2018	FP	11A	9	2018	Year-1	1	0	Fall
9/4/2018	FP	13A	9	2018	Year-1	1	0	Fall
9/5/2018	FP	1M	9	2018	Year-1	1	0	Fall
9/5/2018	FP	2M	9	2018	Year-1		1	Fall
9/5/2018	FP	3M	9	2018	Year-1	1	0	Fall
9/5/2018	FP	4M	9	2018	Year-1	1	0	Fall
9/5/2018	FP	5M	9	2018	Year-1		1	Fall
9/5/2018	FP	6M	9	2018	Year-1	1	0	Fall
9/5/2018	FP	7M	9	2018	Year-1	0	0	Fall
9/5/2018	FP	10M	9	2018	Year-1		1	Fall
9/5/2018	FP	12M	9	2018	Year-1		1	Fall
9/5/2018	FP	14M	9	2018	Year-1	0	0	Fall
9/5/2018	FP	8A	9	2018	Year-1		1	Fall
9/5/2018	FP	9A	9	2018	Year-1	0	0	Fall
9/5/2018	FP	11A	9	2018	Year-1	1	0	Fall
9/5/2018	FP	13A	9	2018	Year-1	1	0	Fall
9/6/2018	FP	1M	9	2018	Year-1	1	0	Fall
9/6/2018	FP	2M	9	2018	Year-1		1	Fall
9/6/2018	FP	3M	9	2018	Year-1	1	0	Fall
9/6/2018	FP	4M	9	2018	Year-1	1	0	Fall
9/6/2018	FP	5M	9	2018	Year-1		1	Fall
9/6/2018	FP	6M	9	2018	Year-1	1	0	Fall
9/6/2018	FP	7M	9	2018	Year-1	0	0	Fall
9/6/2018	FP	10M	9	2018	Year-1		1	Fall
9/6/2018	FP	12M	9	2018	Year-1		1	Fall
9/6/2018	FP	14M	9	2018	Year-1	0	0	Fall
9/6/2018	FP	8A	9	2018	Year-1		1	Fall
9/6/2018	FP	9A	9	2018	Year-1	0	0	Fall
9/6/2018	FP	11A	9	2018	Year-1	1	0	Fall
9/6/2018	FP	13A	9	2018	Year-1	1	0	Fall
9/7/2018	FP	1M	9	2018	Year-1	1	0	Fall
9/7/2018	FP	2M	9	2018	Year-1		1	Fall
9/7/2018	FP	3M	9	2018	Year-1	1	0	Fall
9/7/2018	FP	4M	9	2018	Year-1	1	0	Fall
9/7/2018	FP	5M	9	2018	Year-1		1	Fall
9/7/2018	FP	6M	9	2018	Year-1	1	0	Fall
9/7/2018	FP	7M	9	2018	Year-1	0	0	Fall
9/7/2018	FP	10M	9	2018	Year-1		1	Fall
9/7/2018	FP	12M	9	2018	Year-1		1	Fall
9/7/2018	FP	14M	9	2018	Year-1	0	0	Fall
9/7/2018	FP	8A	9	2018	Year-1		1	Fall
9/7/2018	FP	9A	9	2018	Year-1	1	0	Fall
9/7/2018	FP	11A	9	2018	Year-1	1	0	Fall

Date	Species	Site	Month	Year	Survey Year	Presence	Data Gap	Season
9/7/2018	FP	13A	9	2018	Year-1	1	0	Fall
9/8/2018	FP	1M	9	2018	Year-1	1	0	Fall
9/8/2018	FP	2M	9	2018	Year-1		1	Fall
9/8/2018	FP	3M	9	2018	Year-1	1	0	Fall
9/8/2018	FP	4M	9	2018	Year-1	1	0	Fall
9/8/2018	FP	5M	9	2018	Year-1		1	Fall
9/8/2018	FP	6M	9	2018	Year-1	1	0	Fall
9/8/2018	FP	7M	9	2018	Year-1	0	0	Fall
9/8/2018	FP	10M	9	2018	Year-1		1	Fall
9/8/2018	FP	12M	9	2018	Year-1		1	Fall
9/8/2018	FP	14M	9	2018	Year-1	0	0	Fall
9/8/2018	FP	8A	9	2018	Year-1		1	Fall
9/8/2018	FP	9A	9	2018	Year-1	0	0	Fall
9/8/2018	FP	11A	9	2018	Year-1	1	0	Fall
9/8/2018	FP	13A	9	2018	Year-1	1	0	Fall
9/9/2018	FP	1M	9	2018	Year-1	1	0	Fall
9/9/2018	FP	2M	9	2018	Year-1		1	Fall
9/9/2018	FP	3M	9	2018	Year-1	1	0	Fall
9/9/2018	FP	4M	9	2018	Year-1	1	0	Fall
9/9/2018	FP	5M	9	2018	Year-1		1	Fall
9/9/2018	FP	6M	9	2018	Year-1	1	0	Fall
9/9/2018	FP	7M	9	2018	Year-1	0	0	Fall
9/9/2018	FP	10M	9	2018	Year-1		1	Fall
9/9/2018	FP	12M	9	2018	Year-1		1	Fall
9/9/2018	FP	14M	9	2018	Year-1	0	0	Fall
9/9/2018	FP	8A	9	2018	Year-1		1	Fall
9/9/2018	FP	9A	9	2018	Year-1	1	0	Fall
9/9/2018	FP	11A	9	2018	Year-1	1	0	Fall
9/9/2018	FP	13A	9	2018	Year-1	1	0	Fall
9/10/2018	FP	1M	9	2018	Year-1	1	0	Fall
9/10/2018	FP	2M	9	2018	Year-1		1	Fall
9/10/2018	FP	3M	9	2018	Year-1	1	0	Fall
9/10/2018	FP	4M	9	2018	Year-1	1	0	Fall
9/10/2018	FP	5M	9	2018	Year-1		1	Fall
9/10/2018	FP	6M	9	2018	Year-1	1	0	Fall
9/10/2018	FP	7M	9	2018	Year-1	0	0	Fall
9/10/2018	FP	10M	9	2018	Year-1		1	Fall
9/10/2018	FP	12M	9	2018	Year-1		1	Fall
9/10/2018	FP	14M	9	2018	Year-1	0	0	Fall
9/10/2018	FP	8A	9	2018	Year-1		1	Fall
9/10/2018	FP	9A	9	2018	Year-1	0	0	Fall
9/10/2018	FP	11A	9	2018	Year-1	1	0	Fall
9/10/2018	FP	13A	9	2018	Year-1	1	0	Fall
9/11/2018	FP	1M	9	2018	Year-1	1	0	Fall

Date	Species	Site	Month	Year	Survey Year	Presence	Data Gap	Season
9/11/2018	FP	2M	9	2018	Year-1		1	Fall
9/11/2018	FP	3M	9	2018	Year-1	1	0	Fall
9/11/2018	FP	4M	9	2018	Year-1	1	0	Fall
9/11/2018	FP	5M	9	2018	Year-1		1	Fall
9/11/2018	FP	6M	9	2018	Year-1	1	0	Fall
9/11/2018	FP	7M	9	2018	Year-1	1	0	Fall
9/11/2018	FP	10M	9	2018	Year-1		1	Fall
9/11/2018	FP	12M	9	2018	Year-1		1	Fall
9/11/2018	FP	14M	9	2018	Year-1	0	0	Fall
9/11/2018	FP	8A	9	2018	Year-1		1	Fall
9/11/2018	FP	9A	9	2018	Year-1	1	0	Fall
9/11/2018	FP	11A	9	2018	Year-1	1	0	Fall
9/11/2018	FP	13A	9	2018	Year-1	1	0	Fall
9/12/2018	FP	1M	9	2018	Year-1	1	0	Fall
9/12/2018	FP	2M	9	2018	Year-1		1	Fall
9/12/2018	FP	3M	9	2018	Year-1	1	0	Fall
9/12/2018	FP	4M	9	2018	Year-1	1	0	Fall
9/12/2018	FP	5M	9	2018	Year-1		1	Fall
9/12/2018	FP	6M	9	2018	Year-1	1	0	Fall
9/12/2018	FP	7M	9	2018	Year-1	1	0	Fall
9/12/2018	FP	10M	9	2018	Year-1		1	Fall
9/12/2018	FP	12M	9	2018	Year-1		1	Fall
9/12/2018	FP	14M	9	2018	Year-1	0	0	Fall
9/12/2018	FP	8A	9	2018	Year-1		1	Fall
9/12/2018	FP	9A	9	2018	Year-1	1	0	Fall
9/12/2018	FP	11A	9	2018	Year-1	1	0	Fall
9/12/2018	FP	13A	9	2018	Year-1	1	0	Fall
9/13/2018	FP	1M	9	2018	Year-1	1	0	Fall
9/13/2018	FP	2M	9	2018	Year-1		1	Fall
9/13/2018	FP	3M	9	2018	Year-1	1	0	Fall
9/13/2018	FP	4M	9	2018	Year-1	1	0	Fall
9/13/2018	FP	5M	9	2018	Year-1		1	Fall
9/13/2018	FP	6M	9	2018	Year-1	1	0	Fall
9/13/2018	FP	7M	9	2018	Year-1	0	0	Fall
9/13/2018	FP	10M	9	2018	Year-1		1	Fall
9/13/2018	FP	12M	9	2018	Year-1		1	Fall
9/13/2018	FP	14M	9	2018	Year-1	0	0	Fall
9/13/2018	FP	8A	9	2018	Year-1		1	Fall
9/13/2018	FP	9A	9	2018	Year-1	0	0	Fall
9/13/2018	FP	11A	9	2018	Year-1	1	0	Fall
9/13/2018	FP	13A	9	2018	Year-1	1	0	Fall
9/14/2018	FP	1M	9	2018	Year-1	1	0	Fall
9/14/2018	FP	2M	9	2018	Year-1		1	Fall
9/14/2018	FP	3M	9	2018	Year-1	1	0	Fall

Date	Species	Site	Month	Year	Survey Year	Presence	Data Gap	Season
9/14/2018	FP	4M	9	2018	Year-1	1	0	Fall
9/14/2018	FP	5M	9	2018	Year-1		1	Fall
9/14/2018	FP	6M	9	2018	Year-1	1	0	Fall
9/14/2018	FP	7M	9	2018	Year-1	0	0	Fall
9/14/2018	FP	10M	9	2018	Year-1		1	Fall
9/14/2018	FP	12M	9	2018	Year-1		1	Fall
9/14/2018	FP	14M	9	2018	Year-1	0	0	Fall
9/14/2018	FP	8A	9	2018	Year-1		1	Fall
9/14/2018	FP	9A	9	2018	Year-1	0	0	Fall
9/14/2018	FP	11A	9	2018	Year-1	1	0	Fall
9/14/2018	FP	13A	9	2018	Year-1	1	0	Fall
9/15/2018	FP	1M	9	2018	Year-1	1	0	Fall
9/15/2018	FP	2M	9	2018	Year-1		1	Fall
9/15/2018	FP	3M	9	2018	Year-1	1	0	Fall
9/15/2018	FP	4M	9	2018	Year-1	1	0	Fall
9/15/2018	FP	5M	9	2018	Year-1		1	Fall
9/15/2018	FP	6M	9	2018	Year-1	1	0	Fall
9/15/2018	FP	7M	9	2018	Year-1	0	0	Fall
9/15/2018	FP	10M	9	2018	Year-1		1	Fall
9/15/2018	FP	12M	9	2018	Year-1		1	Fall
9/15/2018	FP	14M	9	2018	Year-1	0	0	Fall
9/15/2018	FP	8A	9	2018	Year-1		1	Fall
9/15/2018	FP	9A	9	2018	Year-1	0	0	Fall
9/15/2018	FP	11A	9	2018	Year-1	1	0	Fall
9/15/2018	FP	13A	9	2018	Year-1	1	0	Fall
9/16/2018	FP	1M	9	2018	Year-1	1	0	Fall
9/16/2018	FP	2M	9	2018	Year-1		1	Fall
9/16/2018	FP	3M	9	2018	Year-1	1	0	Fall
9/16/2018	FP	4M	9	2018	Year-1		1	Fall
9/16/2018	FP	5M	9	2018	Year-1		1	Fall
9/16/2018	FP	6M	9	2018	Year-1	1	0	Fall
9/16/2018	FP	7M	9	2018	Year-1	0	0	Fall
9/16/2018	FP	10M	9	2018	Year-1		1	Fall
9/16/2018	FP	12M	9	2018	Year-1		1	Fall
9/16/2018	FP	14M	9	2018	Year-1	0	0	Fall
9/16/2018	FP	8A	9	2018	Year-1		1	Fall
9/16/2018	FP	9A	9	2018	Year-1	0	0	Fall
9/16/2018	FP	11A	9	2018	Year-1	1	0	Fall
9/16/2018	FP	13A	9	2018	Year-1	1	0	Fall
9/17/2018	FP	1M	9	2018	Year-1	1	0	Fall
9/17/2018	FP	2M	9	2018	Year-1		1	Fall
9/17/2018	FP	3M	9	2018	Year-1	1	0	Fall
9/17/2018	FP	4M	9	2018	Year-1		1	Fall
9/17/2018	FP	5M	9	2018	Year-1		1	Fall

Date	Species	Site	Month	Year	Survey Year	Presence	Data Gap	Season
9/17/2018	FP	6M	9	2018	Year-1	1	0	Fall
9/17/2018	FP	7M	9	2018	Year-1	0	0	Fall
9/17/2018	FP	10M	9	2018	Year-1		1	Fall
9/17/2018	FP	12M	9	2018	Year-1		1	Fall
9/17/2018	FP	14M	9	2018	Year-1	0	0	Fall
9/17/2018	FP	8A	9	2018	Year-1		1	Fall
9/17/2018	FP	9A	9	2018	Year-1	1	0	Fall
9/17/2018	FP	11A	9	2018	Year-1	1	0	Fall
9/17/2018	FP	13A	9	2018	Year-1	1	0	Fall
9/18/2018	FP	1M	9	2018	Year-1	1	0	Fall
9/18/2018	FP	2M	9	2018	Year-1		1	Fall
9/18/2018	FP	3M	9	2018	Year-1	1	0	Fall
9/18/2018	FP	4M	9	2018	Year-1		1	Fall
9/18/2018	FP	5M	9	2018	Year-1		1	Fall
9/18/2018	FP	6M	9	2018	Year-1	1	0	Fall
9/18/2018	FP	7M	9	2018	Year-1	0	0	Fall
9/18/2018	FP	10M	9	2018	Year-1		1	Fall
9/18/2018	FP	12M	9	2018	Year-1		1	Fall
9/18/2018	FP	14M	9	2018	Year-1	0	0	Fall
9/18/2018	FP	8A	9	2018	Year-1		1	Fall
9/18/2018	FP	9A	9	2018	Year-1	1	0	Fall
9/18/2018	FP	11A	9	2018	Year-1	1	0	Fall
9/18/2018	FP	13A	9	2018	Year-1	1	0	Fall
9/19/2018	FP	1M	9	2018	Year-1	1	0	Fall
9/19/2018	FP	2M	9	2018	Year-1		1	Fall
9/19/2018	FP	3M	9	2018	Year-1	1	0	Fall
9/19/2018	FP	4M	9	2018	Year-1		1	Fall
9/19/2018	FP	5M	9	2018	Year-1		1	Fall
9/19/2018	FP	6M	9	2018	Year-1	1	0	Fall
9/19/2018	FP	7M	9	2018	Year-1	1	0	Fall
9/19/2018	FP	10M	9	2018	Year-1		1	Fall
9/19/2018	FP	12M	9	2018	Year-1		1	Fall
9/19/2018	FP	14M	9	2018	Year-1	0	0	Fall
9/19/2018	FP	8A	9	2018	Year-1		1	Fall
9/19/2018	FP	9A	9	2018	Year-1	1	0	Fall
9/19/2018	FP	11A	9	2018	Year-1	1	0	Fall
9/19/2018	FP	13A	9	2018	Year-1	1	0	Fall
9/20/2018	FP	1M	9	2018	Year-1	1	0	Fall
9/20/2018	FP	2M	9	2018	Year-1		1	Fall
9/20/2018	FP	3M	9	2018	Year-1	1	0	Fall
9/20/2018	FP	4M	9	2018	Year-1		1	Fall
9/20/2018	FP	5M	9	2018	Year-1		1	Fall
9/20/2018	FP	6M	9	2018	Year-1	1	0	Fall
9/20/2018	FP	7M	9	2018	Year-1	0	0	Fall

Date	Species	Site	Month	Year	Survey Year	Presence	Data Gap	Season
9/20/2018	FP	10M	9	2018	Year-1		1	Fall
9/20/2018	FP	12M	9	2018	Year-1		1	Fall
9/20/2018	FP	14M	9	2018	Year-1	0	0	Fall
9/20/2018	FP	8A	9	2018	Year-1		1	Fall
9/20/2018	FP	9A	9	2018	Year-1	1	0	Fall
9/20/2018	FP	11A	9	2018	Year-1	1	0	Fall
9/20/2018	FP	13A	9	2018	Year-1	1	0	Fall
9/21/2018	FP	1M	9	2018	Year-1	1	0	Fall
9/21/2018	FP	2M	9	2018	Year-1		1	Fall
9/21/2018	FP	3M	9	2018	Year-1	1	0	Fall
9/21/2018	FP	4M	9	2018	Year-1		1	Fall
9/21/2018	FP	5M	9	2018	Year-1		1	Fall
9/21/2018	FP	6M	9	2018	Year-1	1	0	Fall
9/21/2018	FP	7M	9	2018	Year-1	0	0	Fall
9/21/2018	FP	10M	9	2018	Year-1		1	Fall
9/21/2018	FP	12M	9	2018	Year-1		1	Fall
9/21/2018	FP	14M	9	2018	Year-1	0	0	Fall
9/21/2018	FP	8A	9	2018	Year-1		1	Fall
9/21/2018	FP	9A	9	2018	Year-1	1	0	Fall
9/21/2018	FP	11A	9	2018	Year-1	1	0	Fall
9/21/2018	FP	13A	9	2018	Year-1	1	0	Fall
9/22/2018	FP	1M	9	2018	Year-1	1	0	Fall
9/22/2018	FP	2M	9	2018	Year-1		1	Fall
9/22/2018	FP	3M	9	2018	Year-1	1	0	Fall
9/22/2018	FP	4M	9	2018	Year-1		1	Fall
9/22/2018	FP	5M	9	2018	Year-1		1	Fall
9/22/2018	FP	6M	9	2018	Year-1	1	0	Fall
9/22/2018	FP	7M	9	2018	Year-1	1	0	Fall
9/22/2018	FP	10M	9	2018	Year-1		1	Fall
9/22/2018	FP	12M	9	2018	Year-1		1	Fall
9/22/2018	FP	14M	9	2018	Year-1	0	0	Fall
9/22/2018	FP	8A	9	2018	Year-1		1	Fall
9/22/2018	FP	9A	9	2018	Year-1	1	0	Fall
9/22/2018	FP	11A	9	2018	Year-1	1	0	Fall
9/22/2018	FP	13A	9	2018	Year-1	1	0	Fall
9/23/2018	FP	1M	9	2018	Year-1	1	0	Fall
9/23/2018	FP	2M	9	2018	Year-1		1	Fall
9/23/2018	FP	3M	9	2018	Year-1	1	0	Fall
9/23/2018	FP	4M	9	2018	Year-1		1	Fall
9/23/2018	FP	5M	9	2018	Year-1		1	Fall
9/23/2018	FP	6M	9	2018	Year-1	1	0	Fall
9/23/2018	FP	7M	9	2018	Year-1	1	0	Fall
9/23/2018	FP	10M	9	2018	Year-1		1	Fall
9/23/2018	FP	12M	9	2018	Year-1		1	Fall

Date	Species	Site	Month	Year	Survey Year	Presence	Data Gap	Season
9/23/2018	FP	14M	9	2018	Year-1	0	0	Fall
9/23/2018	FP	8A	9	2018	Year-1		1	Fall
9/23/2018	FP	9A	9	2018	Year-1	1	0	Fall
9/23/2018	FP	11A	9	2018	Year-1	1	0	Fall
9/23/2018	FP	13A	9	2018	Year-1	1	0	Fall
9/24/2018	FP	1M	9	2018	Year-1	1	0	Fall
9/24/2018	FP	2M	9	2018	Year-1		1	Fall
9/24/2018	FP	3M	9	2018	Year-1	1	0	Fall
9/24/2018	FP	4M	9	2018	Year-1		1	Fall
9/24/2018	FP	5M	9	2018	Year-1		1	Fall
9/24/2018	FP	6M	9	2018	Year-1	1	0	Fall
9/24/2018	FP	7M	9	2018	Year-1	1	0	Fall
9/24/2018	FP	10M	9	2018	Year-1		1	Fall
9/24/2018	FP	12M	9	2018	Year-1		1	Fall
9/24/2018	FP	14M	9	2018	Year-1	0	0	Fall
9/24/2018	FP	8A	9	2018	Year-1		1	Fall
9/24/2018	FP	9A	9	2018	Year-1	0	0	Fall
9/24/2018	FP	11A	9	2018	Year-1	1	0	Fall
9/24/2018	FP	13A	9	2018	Year-1	1	0	Fall
9/25/2018	FP	1M	9	2018	Year-1	1	0	Fall
9/25/2018	FP	2M	9	2018	Year-1		1	Fall
9/25/2018	FP	3M	9	2018	Year-1	1	0	Fall
9/25/2018	FP	4M	9	2018	Year-1		1	Fall
9/25/2018	FP	5M	9	2018	Year-1		1	Fall
9/25/2018	FP	6M	9	2018	Year-1	1	0	Fall
9/25/2018	FP	7M	9	2018	Year-1	1	0	Fall
9/25/2018	FP	10M	9	2018	Year-1		1	Fall
9/25/2018	FP	12M	9	2018	Year-1		1	Fall
9/25/2018	FP	14M	9	2018	Year-1	0	0	Fall
9/25/2018	FP	8A	9	2018	Year-1		1	Fall
9/25/2018	FP	9A	9	2018	Year-1	1	0	Fall
9/25/2018	FP	11A	9	2018	Year-1	1	0	Fall
9/25/2018	FP	13A	9	2018	Year-1	1	0	Fall
9/26/2018	FP	1M	9	2018	Year-1	1	0	Fall
9/26/2018	FP	2M	9	2018	Year-1		1	Fall
9/26/2018	FP	3M	9	2018	Year-1	1	0	Fall
9/26/2018	FP	4M	9	2018	Year-1		1	Fall
9/26/2018	FP	5M	9	2018	Year-1		1	Fall
9/26/2018	FP	6M	9	2018	Year-1	1	0	Fall
9/26/2018	FP	7M	9	2018	Year-1	0	0	Fall
9/26/2018	FP	10M	9	2018	Year-1		1	Fall
9/26/2018	FP	12M	9	2018	Year-1		1	Fall
9/26/2018	FP	14M	9	2018	Year-1	0	0	Fall
9/26/2018	FP	8A	9	2018	Year-1		1	Fall

Date	Species	Site	Month	Year	Survey Year	Presence	Data Gap	Season
9/26/2018	FP	9A	9	2018	Year-1	1	0	Fall
9/26/2018	FP	11A	9	2018	Year-1	1	0	Fall
9/26/2018	FP	13A	9	2018	Year-1	1	0	Fall
9/27/2018	FP	1M	9	2018	Year-1	1	0	Fall
9/27/2018	FP	2M	9	2018	Year-1		1	Fall
9/27/2018	FP	3M	9	2018	Year-1	1	0	Fall
9/27/2018	FP	4M	9	2018	Year-1		1	Fall
9/27/2018	FP	5M	9	2018	Year-1		1	Fall
9/27/2018	FP	6M	9	2018	Year-1	1	0	Fall
9/27/2018	FP	7M	9	2018	Year-1	1	0	Fall
9/27/2018	FP	10M	9	2018	Year-1		1	Fall
9/27/2018	FP	12M	9	2018	Year-1		1	Fall
9/27/2018	FP	14M	9	2018	Year-1	0	0	Fall
9/27/2018	FP	8A	9	2018	Year-1		1	Fall
9/27/2018	FP	9A	9	2018	Year-1	1	0	Fall
9/27/2018	FP	11A	9	2018	Year-1	1	0	Fall
9/27/2018	FP	13A	9	2018	Year-1	1	0	Fall
9/28/2018	FP	1M	9	2018	Year-1	1	0	Fall
9/28/2018	FP	2M	9	2018	Year-1		1	Fall
9/28/2018	FP	3M	9	2018	Year-1	1	0	Fall
9/28/2018	FP	4M	9	2018	Year-1		1	Fall
9/28/2018	FP	5M	9	2018	Year-1		1	Fall
9/28/2018	FP	6M	9	2018	Year-1	1	0	Fall
9/28/2018	FP	7M	9	2018	Year-1	1	0	Fall
9/28/2018	FP	10M	9	2018	Year-1		1	Fall
9/28/2018	FP	12M	9	2018	Year-1		1	Fall
9/28/2018	FP	14M	9	2018	Year-1	0	0	Fall
9/28/2018	FP	8A	9	2018	Year-1		1	Fall
9/28/2018	FP	9A	9	2018	Year-1	1	0	Fall
9/28/2018	FP	11A	9	2018	Year-1	1	0	Fall
9/28/2018	FP	13A	9	2018	Year-1	1	0	Fall
9/29/2018	FP	1M	9	2018	Year-1	1	0	Fall
9/29/2018	FP	2M	9	2018	Year-1		1	Fall
9/29/2018	FP	3M	9	2018	Year-1	1	0	Fall
9/29/2018	FP	4M	9	2018	Year-1		1	Fall
9/29/2018	FP	5M	9	2018	Year-1		1	Fall
9/29/2018	FP	6M	9	2018	Year-1	1	0	Fall
9/29/2018	FP	7M	9	2018	Year-1	1	0	Fall
9/29/2018	FP	10M	9	2018	Year-1		1	Fall
9/29/2018	FP	12M	9	2018	Year-1		1	Fall
9/29/2018	FP	14M	9	2018	Year-1	0	0	Fall
9/29/2018	FP	8A	9	2018	Year-1		1	Fall
9/29/2018	FP	9A	9	2018	Year-1	0	0	Fall
9/29/2018	FP	11A	9	2018	Year-1	1	0	Fall

Date	Species	Site	Month	Year	Survey Year	Presence	Data Gap	Season
9/29/2018	FP	13A	9	2018	Year-1	1	0	Fall
9/30/2018	FP	1M	9	2018	Year-1	1	0	Fall
9/30/2018	FP	2M	9	2018	Year-1		1	Fall
9/30/2018	FP	3M	9	2018	Year-1	1	0	Fall
9/30/2018	FP	4M	9	2018	Year-1		1	Fall
9/30/2018	FP	5M	9	2018	Year-1		1	Fall
9/30/2018	FP	6M	9	2018	Year-1	1	0	Fall
9/30/2018	FP	7M	9	2018	Year-1	0	0	Fall
9/30/2018	FP	10M	9	2018	Year-1		1	Fall
9/30/2018	FP	12M	9	2018	Year-1		1	Fall
9/30/2018	FP	14M	9	2018	Year-1	0	0	Fall
9/30/2018	FP	8A	9	2018	Year-1		1	Fall
9/30/2018	FP	9A	9	2018	Year-1	0	0	Fall
9/30/2018	FP	11A	9	2018	Year-1	1	0	Fall
9/30/2018	FP	13A	9	2018	Year-1	1	0	Fall
10/1/2018	FP	1M	10	2018	Year-1	1	0	Fall
10/1/2018	FP	2M	10	2018	Year-1		1	Fall
10/1/2018	FP	3M	10	2018	Year-1	1	0	Fall
10/1/2018	FP	4M	10	2018	Year-1		1	Fall
10/1/2018	FP	5M	10	2018	Year-1		1	Fall
10/1/2018	FP	6M	10	2018	Year-1		1	Fall
10/1/2018	FP	7M	10	2018	Year-1	0	0	Fall
10/1/2018	FP	10M	10	2018	Year-1		1	Fall
10/1/2018	FP	12M	10	2018	Year-1		1	Fall
10/1/2018	FP	14M	10	2018	Year-1	0	0	Fall
10/1/2018	FP	8A	10	2018	Year-1		1	Fall
10/1/2018	FP	9A	10	2018	Year-1	1	0	Fall
10/1/2018	FP	11A	10	2018	Year-1	1	0	Fall
10/1/2018	FP	13A	10	2018	Year-1	1	0	Fall
10/2/2018	FP	1M	10	2018	Year-1	1	0	Fall
10/2/2018	FP	2M	10	2018	Year-1		1	Fall
10/2/2018	FP	3M	10	2018	Year-1	1	0	Fall
10/2/2018	FP	4M	10	2018	Year-1		1	Fall
10/2/2018	FP	5M	10	2018	Year-1		1	Fall
10/2/2018	FP	6M	10	2018	Year-1		1	Fall
10/2/2018	FP	7M	10	2018	Year-1	0	0	Fall
10/2/2018	FP	10M	10	2018	Year-1		1	Fall
10/2/2018	FP	12M	10	2018	Year-1		1	Fall
10/2/2018	FP	14M	10	2018	Year-1	0	0	Fall
10/2/2018	FP	8A	10	2018	Year-1		1	Fall
10/2/2018	FP	9A	10	2018	Year-1	1	0	Fall
10/2/2018	FP	11A	10	2018	Year-1	1	0	Fall
10/2/2018	FP	13A	10	2018	Year-1	1	0	Fall
10/3/2018	FP	1M	10	2018	Year-1	1	0	Fall

Date	Species	Site	Month	Year	Survey Year	Presence	Data Gap	Season
10/3/2018	FP	2M	10	2018	Year-1		1	Fall
10/3/2018	FP	3M	10	2018	Year-1	1	0	Fall
10/3/2018	FP	4M	10	2018	Year-1		1	Fall
10/3/2018	FP	5M	10	2018	Year-1		1	Fall
10/3/2018	FP	6M	10	2018	Year-1		1	Fall
10/3/2018	FP	7M	10	2018	Year-1	0	0	Fall
10/3/2018	FP	10M	10	2018	Year-1		1	Fall
10/3/2018	FP	12M	10	2018	Year-1		1	Fall
10/3/2018	FP	14M	10	2018	Year-1	0	0	Fall
10/3/2018	FP	8A	10	2018	Year-1		1	Fall
10/3/2018	FP	9A	10	2018	Year-1	1	0	Fall
10/3/2018	FP	11A	10	2018	Year-1	1	0	Fall
10/3/2018	FP	13A	10	2018	Year-1	1	0	Fall
10/4/2018	FP	1M	10	2018	Year-1	1	0	Fall
10/4/2018	FP	2M	10	2018	Year-1		1	Fall
10/4/2018	FP	3M	10	2018	Year-1	1	0	Fall
10/4/2018	FP	4M	10	2018	Year-1		1	Fall
10/4/2018	FP	5M	10	2018	Year-1		1	Fall
10/4/2018	FP	6M	10	2018	Year-1		1	Fall
10/4/2018	FP	7M	10	2018	Year-1	0	0	Fall
10/4/2018	FP	10M	10	2018	Year-1		1	Fall
10/4/2018	FP	12M	10	2018	Year-1		1	Fall
10/4/2018	FP	14M	10	2018	Year-1	0	0	Fall
10/4/2018	FP	8A	10	2018	Year-1		1	Fall
10/4/2018	FP	9A	10	2018	Year-1	1	0	Fall
10/4/2018	FP	11A	10	2018	Year-1	1	0	Fall
10/4/2018	FP	13A	10	2018	Year-1	1	0	Fall
10/5/2018	FP	1M	10	2018	Year-1	1	0	Fall
10/5/2018	FP	2M	10	2018	Year-1		1	Fall
10/5/2018	FP	3M	10	2018	Year-1	1	0	Fall
10/5/2018	FP	4M	10	2018	Year-1		1	Fall
10/5/2018	FP	5M	10	2018	Year-1		1	Fall
10/5/2018	FP	6M	10	2018	Year-1		1	Fall
10/5/2018	FP	7M	10	2018	Year-1	0	0	Fall
10/5/2018	FP	10M	10	2018	Year-1		1	Fall
10/5/2018	FP	12M	10	2018	Year-1		1	Fall
10/5/2018	FP	14M	10	2018	Year-1	0	0	Fall
10/5/2018	FP	8A	10	2018	Year-1		1	Fall
10/5/2018	FP	9A	10	2018	Year-1	1	0	Fall
10/5/2018	FP	11A	10	2018	Year-1	1	0	Fall
10/5/2018	FP	13A	10	2018	Year-1	1	0	Fall
10/6/2018	FP	1M	10	2018	Year-1	1	0	Fall
10/6/2018	FP	2M	10	2018	Year-1		1	Fall
10/6/2018	FP	3M	10	2018	Year-1	1	0	Fall

Date	Species	Site	Month	Year	Survey Year	Presence	Data Gap	Season
10/6/2018	FP	4M	10	2018	Year-1		1	Fall
10/6/2018	FP	5M	10	2018	Year-1		1	Fall
10/6/2018	FP	6M	10	2018	Year-1		1	Fall
10/6/2018	FP	7M	10	2018	Year-1	0	0	Fall
10/6/2018	FP	10M	10	2018	Year-1		1	Fall
10/6/2018	FP	12M	10	2018	Year-1		1	Fall
10/6/2018	FP	14M	10	2018	Year-1	0	0	Fall
10/6/2018	FP	8A	10	2018	Year-1		1	Fall
10/6/2018	FP	9A	10	2018	Year-1	1	0	Fall
10/6/2018	FP	11A	10	2018	Year-1	1	0	Fall
10/6/2018	FP	13A	10	2018	Year-1	1	0	Fall
10/7/2018	FP	1M	10	2018	Year-1	1	0	Fall
10/7/2018	FP	2M	10	2018	Year-1		1	Fall
10/7/2018	FP	3M	10	2018	Year-1	1	0	Fall
10/7/2018	FP	4M	10	2018	Year-1		1	Fall
10/7/2018	FP	5M	10	2018	Year-1		1	Fall
10/7/2018	FP	6M	10	2018	Year-1		1	Fall
10/7/2018	FP	7M	10	2018	Year-1	1	0	Fall
10/7/2018	FP	10M	10	2018	Year-1		1	Fall
10/7/2018	FP	12M	10	2018	Year-1		1	Fall
10/7/2018	FP	14M	10	2018	Year-1	0	0	Fall
10/7/2018	FP	8A	10	2018	Year-1		1	Fall
10/7/2018	FP	9A	10	2018	Year-1	1	0	Fall
10/7/2018	FP	11A	10	2018	Year-1	1	0	Fall
10/7/2018	FP	13A	10	2018	Year-1	1	0	Fall
10/8/2018	FP	1M	10	2018	Year-1	1	0	Fall
10/8/2018	FP	2M	10	2018	Year-1		1	Fall
10/8/2018	FP	3M	10	2018	Year-1	1	0	Fall
10/8/2018	FP	4M	10	2018	Year-1		1	Fall
10/8/2018	FP	5M	10	2018	Year-1		1	Fall
10/8/2018	FP	6M	10	2018	Year-1		1	Fall
10/8/2018	FP	7M	10	2018	Year-1	0	0	Fall
10/8/2018	FP	10M	10	2018	Year-1		1	Fall
10/8/2018	FP	12M	10	2018	Year-1		1	Fall
10/8/2018	FP	14M	10	2018	Year-1	0	0	Fall
10/8/2018	FP	8A	10	2018	Year-1		1	Fall
10/8/2018	FP	9A	10	2018	Year-1	1	0	Fall
10/8/2018	FP	11A	10	2018	Year-1	1	0	Fall
10/8/2018	FP	13A	10	2018	Year-1	1	0	Fall
10/9/2018	FP	1M	10	2018	Year-1	1	0	Fall
10/9/2018	FP	2M	10	2018	Year-1		1	Fall
10/9/2018	FP	3M	10	2018	Year-1	1	0	Fall
10/9/2018	FP	4M	10	2018	Year-1		1	Fall
10/9/2018	FP	5M	10	2018	Year-1		1	Fall

Date	Species	Site	Month	Year	Survey Year	Presence	Data Gap	Season
10/9/2018	FP	6M	10	2018	Year-1		1	Fall
10/9/2018	FP	7M	10	2018	Year-1	0	0	Fall
10/9/2018	FP	10M	10	2018	Year-1		1	Fall
10/9/2018	FP	12M	10	2018	Year-1		1	Fall
10/9/2018	FP	14M	10	2018	Year-1	0	0	Fall
10/9/2018	FP	8A	10	2018	Year-1		1	Fall
10/9/2018	FP	9A	10	2018	Year-1	0	0	Fall
10/9/2018	FP	11A	10	2018	Year-1	1	0	Fall
10/9/2018	FP	13A	10	2018	Year-1	1	0	Fall
10/10/2018	FP	1M	10	2018	Year-1	1	0	Fall
10/10/2018	FP	2M	10	2018	Year-1		1	Fall
10/10/2018	FP	3M	10	2018	Year-1	1	0	Fall
10/10/2018	FP	4M	10	2018	Year-1		1	Fall
10/10/2018	FP	5M	10	2018	Year-1		1	Fall
10/10/2018	FP	6M	10	2018	Year-1		1	Fall
10/10/2018	FP	7M	10	2018	Year-1	0	0	Fall
10/10/2018	FP	10M	10	2018	Year-1		1	Fall
10/10/2018	FP	12M	10	2018	Year-1		1	Fall
10/10/2018	FP	14M	10	2018	Year-1	0	0	Fall
10/10/2018	FP	8A	10	2018	Year-1		1	Fall
10/10/2018	FP	9A	10	2018	Year-1	0	0	Fall
10/10/2018	FP	11A	10	2018	Year-1	1	0	Fall
10/10/2018	FP	13A	10	2018	Year-1	1	0	Fall
10/11/2018	FP	1M	10	2018	Year-1	1	0	Fall
10/11/2018	FP	2M	10	2018	Year-1		1	Fall
10/11/2018	FP	3M	10	2018	Year-1	1	0	Fall
10/11/2018	FP	4M	10	2018	Year-1		1	Fall
10/11/2018	FP	5M	10	2018	Year-1		1	Fall
10/11/2018	FP	6M	10	2018	Year-1		1	Fall
10/11/2018	FP	7M	10	2018	Year-1	1	0	Fall
10/11/2018	FP	10M	10	2018	Year-1		1	Fall
10/11/2018	FP	12M	10	2018	Year-1		1	Fall
10/11/2018	FP	14M	10	2018	Year-1	0	0	Fall
10/11/2018	FP	8A	10	2018	Year-1		1	Fall
10/11/2018	FP	9A	10	2018	Year-1	0	0	Fall
10/11/2018	FP	11A	10	2018	Year-1	1	0	Fall
10/11/2018	FP	13A	10	2018	Year-1	1	0	Fall
10/12/2018	FP	1M	10	2018	Year-1	1	0	Fall
10/12/2018	FP	2M	10	2018	Year-1		1	Fall
10/12/2018	FP	3M	10	2018	Year-1	1	0	Fall
10/12/2018	FP	4M	10	2018	Year-1		1	Fall
10/12/2018	FP	5M	10	2018	Year-1		1	Fall
10/12/2018	FP	6M	10	2018	Year-1		1	Fall
10/12/2018	FP	7M	10	2018	Year-1	1	0	Fall

Date	Species	Site	Month	Year	Survey Year	Presence	Data Gap	Season
10/12/2018	FP	10M	10	2018	Year-1		1	Fall
10/12/2018	FP	12M	10	2018	Year-1		1	Fall
10/12/2018	FP	14M	10	2018	Year-1	0	0	Fall
10/12/2018	FP	8A	10	2018	Year-1		1	Fall
10/12/2018	FP	9A	10	2018	Year-1	0	0	Fall
10/12/2018	FP	11A	10	2018	Year-1	1	0	Fall
10/12/2018	FP	13A	10	2018	Year-1	1	0	Fall
10/13/2018	FP	1M	10	2018	Year-1	1	0	Fall
10/13/2018	FP	2M	10	2018	Year-1		1	Fall
10/13/2018	FP	3M	10	2018	Year-1	1	0	Fall
10/13/2018	FP	4M	10	2018	Year-1		1	Fall
10/13/2018	FP	5M	10	2018	Year-1		1	Fall
10/13/2018	FP	6M	10	2018	Year-1		1	Fall
10/13/2018	FP	7M	10	2018	Year-1	0	0	Fall
10/13/2018	FP	10M	10	2018	Year-1		1	Fall
10/13/2018	FP	12M	10	2018	Year-1		1	Fall
10/13/2018	FP	14M	10	2018	Year-1	0	0	Fall
10/13/2018	FP	8A	10	2018	Year-1		1	Fall
10/13/2018	FP	9A	10	2018	Year-1	0	0	Fall
10/13/2018	FP	11A	10	2018	Year-1	1	0	Fall
10/13/2018	FP	13A	10	2018	Year-1	1	0	Fall
10/14/2018	FP	1M	10	2018	Year-1	1	0	Fall
10/14/2018	FP	2M	10	2018	Year-1		1	Fall
10/14/2018	FP	3M	10	2018	Year-1	1	0	Fall
10/14/2018	FP	4M	10	2018	Year-1		1	Fall
10/14/2018	FP	5M	10	2018	Year-1		1	Fall
10/14/2018	FP	6M	10	2018	Year-1		1	Fall
10/14/2018	FP	7M	10	2018	Year-1	0	0	Fall
10/14/2018	FP	10M	10	2018	Year-1		1	Fall
10/14/2018	FP	12M	10	2018	Year-1		1	Fall
10/14/2018	FP	14M	10	2018	Year-1	0	0	Fall
10/14/2018	FP	8A	10	2018	Year-1		1	Fall
10/14/2018	FP	9A	10	2018	Year-1	0	0	Fall
10/14/2018	FP	11A	10	2018	Year-1	1	0	Fall
10/14/2018	FP	13A	10	2018	Year-1	1	0	Fall
10/15/2018	FP	1M	10	2018	Year-1	1	0	Fall
10/15/2018	FP	2M	10	2018	Year-1		1	Fall
10/15/2018	FP	3M	10	2018	Year-1	1	0	Fall
10/15/2018	FP	4M	10	2018	Year-1		1	Fall
10/15/2018	FP	5M	10	2018	Year-1		1	Fall
10/15/2018	FP	6M	10	2018	Year-1		1	Fall
10/15/2018	FP	7M	10	2018	Year-1	0	0	Fall
10/15/2018	FP	10M	10	2018	Year-1		1	Fall
10/15/2018	FP	12M	10	2018	Year-1		1	Fall

Date	Species	Site	Month	Year	Survey Year	Presence	Data Gap	Season
10/15/2018	FP	14M	10	2018	Year-1	0	0	Fall
10/15/2018	FP	8A	10	2018	Year-1		1	Fall
10/15/2018	FP	9A	10	2018	Year-1	0	0	Fall
10/15/2018	FP	11A	10	2018	Year-1	1	0	Fall
10/15/2018	FP	13A	10	2018	Year-1	1	0	Fall
10/16/2018	FP	1M	10	2018	Year-2	0	0	Fall
10/16/2018	FP	2M	10	2018	Year-2		1	Fall
10/16/2018	FP	3M	10	2018	Year-2	0	0	Fall
10/16/2018	FP	4M	10	2018	Year-2		1	Fall
10/16/2018	FP	5M	10	2018	Year-2		1	Fall
10/16/2018	FP	6M	10	2018	Year-2		1	Fall
10/16/2018	FP	7M	10	2018	Year-2	0	0	Fall
10/16/2018	FP	10M	10	2018	Year-2		1	Fall
10/16/2018	FP	12M	10	2018	Year-2		1	Fall
10/16/2018	FP	14M	10	2018	Year-2	0	0	Fall
10/16/2018	FP	8A	10	2018	Year-2		1	Fall
10/16/2018	FP	9A	10	2018	Year-2	0	0	Fall
10/16/2018	FP	11A	10	2018	Year-2	1	0	Fall
10/16/2018	FP	13A	10	2018	Year-2	1	0	Fall
10/17/2018	FP	1M	10	2018	Year-2	1	0	Fall
10/17/2018	FP	2M	10	2018	Year-2		1	Fall
10/17/2018	FP	3M	10	2018	Year-2	1	0	Fall
10/17/2018	FP	4M	10	2018	Year-2		1	Fall
10/17/2018	FP	5M	10	2018	Year-2		1	Fall
10/17/2018	FP	6M	10	2018	Year-2		1	Fall
10/17/2018	FP	7M	10	2018	Year-2	1	0	Fall
10/17/2018	FP	10M	10	2018	Year-2		1	Fall
10/17/2018	FP	12M	10	2018	Year-2		1	Fall
10/17/2018	FP	14M	10	2018	Year-2	1	0	Fall
10/17/2018	FP	8A	10	2018	Year-2		1	Fall
10/17/2018	FP	9A	10	2018	Year-2	0	0	Fall
10/17/2018	FP	11A	10	2018	Year-2	1	0	Fall
10/17/2018	FP	13A	10	2018	Year-2	1	0	Fall
10/18/2018	FP	1M	10	2018	Year-2	1	0	Fall
10/18/2018	FP	2M	10	2018	Year-2		1	Fall
10/18/2018	FP	3M	10	2018	Year-2	1	0	Fall
10/18/2018	FP	4M	10	2018	Year-2		1	Fall
10/18/2018	FP	5M	10	2018	Year-2		1	Fall
10/18/2018	FP	6M	10	2018	Year-2		1	Fall
10/18/2018	FP	7M	10	2018	Year-2	0	0	Fall
10/18/2018	FP	10M	10	2018	Year-2		1	Fall
10/18/2018	FP	12M	10	2018	Year-2		1	Fall
10/18/2018	FP	14M	10	2018	Year-2	0	0	Fall
10/18/2018	FP	8A	10	2018	Year-2		1	Fall

Date	Species	Site	Month	Year	Survey Year	Presence	Data Gap	Season
10/18/2018	FP	9A	10	2018	Year-2	0	0	Fall
10/18/2018	FP	11A	10	2018	Year-2	1	0	Fall
10/18/2018	FP	13A	10	2018	Year-2	1	0	Fall
10/19/2018	FP	1M	10	2018	Year-2	1	0	Fall
10/19/2018	FP	2M	10	2018	Year-2		1	Fall
10/19/2018	FP	3M	10	2018	Year-2	1	0	Fall
10/19/2018	FP	4M	10	2018	Year-2		1	Fall
10/19/2018	FP	5M	10	2018	Year-2		1	Fall
10/19/2018	FP	6M	10	2018	Year-2		1	Fall
10/19/2018	FP	7M	10	2018	Year-2	0	0	Fall
10/19/2018	FP	10M	10	2018	Year-2		1	Fall
10/19/2018	FP	12M	10	2018	Year-2		1	Fall
10/19/2018	FP	14M	10	2018	Year-2	0	0	Fall
10/19/2018	FP	8A	10	2018	Year-2		1	Fall
10/19/2018	FP	9A	10	2018	Year-2	0	0	Fall
10/19/2018	FP	11A	10	2018	Year-2	1	0	Fall
10/19/2018	FP	13A	10	2018	Year-2	1	0	Fall
10/20/2018	FP	1M	10	2018	Year-2	1	0	Fall
10/20/2018	FP	2M	10	2018	Year-2		1	Fall
10/20/2018	FP	3M	10	2018	Year-2	1	0	Fall
10/20/2018	FP	4M	10	2018	Year-2		1	Fall
10/20/2018	FP	5M	10	2018	Year-2		1	Fall
10/20/2018	FP	6M	10	2018	Year-2		1	Fall
10/20/2018	FP	7M	10	2018	Year-2	0	0	Fall
10/20/2018	FP	10M	10	2018	Year-2		1	Fall
10/20/2018	FP	12M	10	2018	Year-2		1	Fall
10/20/2018	FP	14M	10	2018	Year-2	0	0	Fall
10/20/2018	FP	8A	10	2018	Year-2		1	Fall
10/20/2018	FP	9A	10	2018	Year-2	0	0	Fall
10/20/2018	FP	11A	10	2018	Year-2	1	0	Fall
10/20/2018	FP	13A	10	2018	Year-2	1	0	Fall
10/21/2018	FP	1M	10	2018	Year-2	1	0	Fall
10/21/2018	FP	2M	10	2018	Year-2		1	Fall
10/21/2018	FP	3M	10	2018	Year-2	1	0	Fall
10/21/2018	FP	4M	10	2018	Year-2		1	Fall
10/21/2018	FP	5M	10	2018	Year-2		1	Fall
10/21/2018	FP	6M	10	2018	Year-2		1	Fall
10/21/2018	FP	7M	10	2018	Year-2	1	0	Fall
10/21/2018	FP	10M	10	2018	Year-2		1	Fall
10/21/2018	FP	12M	10	2018	Year-2		1	Fall
10/21/2018	FP	14M	10	2018	Year-2	0	0	Fall
10/21/2018	FP	8A	10	2018	Year-2		1	Fall
10/21/2018	FP	9A	10	2018	Year-2	0	0	Fall
10/21/2018	FP	11A	10	2018	Year-2	1	0	Fall

Date	Species	Site	Month	Year	Survey Year	Presence	Data Gap	Season
10/21/2018	FP	13A	10	2018	Year-2	1	0	Fall
10/22/2018	FP	1M	10	2018	Year-2	1	0	Fall
10/22/2018	FP	2M	10	2018	Year-2		1	Fall
10/22/2018	FP	3M	10	2018	Year-2	1	0	Fall
10/22/2018	FP	4M	10	2018	Year-2		1	Fall
10/22/2018	FP	5M	10	2018	Year-2		1	Fall
10/22/2018	FP	6M	10	2018	Year-2		1	Fall
10/22/2018	FP	7M	10	2018	Year-2	1	0	Fall
10/22/2018	FP	10M	10	2018	Year-2		1	Fall
10/22/2018	FP	12M	10	2018	Year-2		1	Fall
10/22/2018	FP	14M	10	2018	Year-2	0	0	Fall
10/22/2018	FP	8A	10	2018	Year-2		1	Fall
10/22/2018	FP	9A	10	2018	Year-2	0	0	Fall
10/22/2018	FP	11A	10	2018	Year-2	1	0	Fall
10/22/2018	FP	13A	10	2018	Year-2	1	0	Fall
10/23/2018	FP	1M	10	2018	Year-2	1	0	Fall
10/23/2018	FP	2M	10	2018	Year-2		1	Fall
10/23/2018	FP	3M	10	2018	Year-2	1	0	Fall
10/23/2018	FP	4M	10	2018	Year-2		1	Fall
10/23/2018	FP	5M	10	2018	Year-2		1	Fall
10/23/2018	FP	6M	10	2018	Year-2		1	Fall
10/23/2018	FP	7M	10	2018	Year-2	0	0	Fall
10/23/2018	FP	10M	10	2018	Year-2		1	Fall
10/23/2018	FP	12M	10	2018	Year-2		1	Fall
10/23/2018	FP	14M	10	2018	Year-2	0	0	Fall
10/23/2018	FP	8A	10	2018	Year-2		1	Fall
10/23/2018	FP	9A	10	2018	Year-2	0	0	Fall
10/23/2018	FP	11A	10	2018	Year-2	1	0	Fall
10/23/2018	FP	13A	10	2018	Year-2	1	0	Fall
10/24/2018	FP	1M	10	2018	Year-2	1	0	Fall
10/24/2018	FP	2M	10	2018	Year-2		1	Fall
10/24/2018	FP	3M	10	2018	Year-2	1	0	Fall
10/24/2018	FP	4M	10	2018	Year-2		1	Fall
10/24/2018	FP	5M	10	2018	Year-2		1	Fall
10/24/2018	FP	6M	10	2018	Year-2		1	Fall
10/24/2018	FP	7M	10	2018	Year-2	0	0	Fall
10/24/2018	FP	10M	10	2018	Year-2		1	Fall
10/24/2018	FP	12M	10	2018	Year-2		1	Fall
10/24/2018	FP	14M	10	2018	Year-2	0	0	Fall
10/24/2018	FP	8A	10	2018	Year-2		1	Fall
10/24/2018	FP	9A	10	2018	Year-2	0	0	Fall
10/24/2018	FP	11A	10	2018	Year-2	0	0	Fall
10/24/2018	FP	13A	10	2018	Year-2	1	0	Fall
10/25/2018	FP	1M	10	2018	Year-2	1	0	Fall

Date	Species	Site	Month	Year	Survey Year	Presence	Data Gap	Season
10/25/2018	FP	2M	10	2018	Year-2		1	Fall
10/25/2018	FP	3M	10	2018	Year-2	1	0	Fall
10/25/2018	FP	4M	10	2018	Year-2		1	Fall
10/25/2018	FP	5M	10	2018	Year-2		1	Fall
10/25/2018	FP	6M	10	2018	Year-2		1	Fall
10/25/2018	FP	7M	10	2018	Year-2	0	0	Fall
10/25/2018	FP	10M	10	2018	Year-2		1	Fall
10/25/2018	FP	12M	10	2018	Year-2		1	Fall
10/25/2018	FP	14M	10	2018	Year-2	0	0	Fall
10/25/2018	FP	8A	10	2018	Year-2		1	Fall
10/25/2018	FP	9A	10	2018	Year-2	0	0	Fall
10/25/2018	FP	11A	10	2018	Year-2	1	0	Fall
10/25/2018	FP	13A	10	2018	Year-2	1	0	Fall
10/26/2018	FP	1M	10	2018	Year-2	1	0	Fall
10/26/2018	FP	2M	10	2018	Year-2		1	Fall
10/26/2018	FP	3M	10	2018	Year-2	1	0	Fall
10/26/2018	FP	4M	10	2018	Year-2		1	Fall
10/26/2018	FP	5M	10	2018	Year-2		1	Fall
10/26/2018	FP	6M	10	2018	Year-2		1	Fall
10/26/2018	FP	7M	10	2018	Year-2	0	0	Fall
10/26/2018	FP	10M	10	2018	Year-2		1	Fall
10/26/2018	FP	12M	10	2018	Year-2		1	Fall
10/26/2018	FP	14M	10	2018	Year-2	0	0	Fall
10/26/2018	FP	8A	10	2018	Year-2		1	Fall
10/26/2018	FP	9A	10	2018	Year-2	0	0	Fall
10/26/2018	FP	11A	10	2018	Year-2	1	0	Fall
10/26/2018	FP	13A	10	2018	Year-2	1	0	Fall
10/27/2018	FP	1M	10	2018	Year-2	1	0	Fall
10/27/2018	FP	2M	10	2018	Year-2		1	Fall
10/27/2018	FP	3M	10	2018	Year-2	1	0	Fall
10/27/2018	FP	4M	10	2018	Year-2		1	Fall
10/27/2018	FP	5M	10	2018	Year-2		1	Fall
10/27/2018	FP	6M	10	2018	Year-2		1	Fall
10/27/2018	FP	7M	10	2018	Year-2	0	0	Fall
10/27/2018	FP	10M	10	2018	Year-2		1	Fall
10/27/2018	FP	12M	10	2018	Year-2		1	Fall
10/27/2018	FP	14M	10	2018	Year-2	0	0	Fall
10/27/2018	FP	8A	10	2018	Year-2		1	Fall
10/27/2018	FP	9A	10	2018	Year-2	0	0	Fall
10/27/2018	FP	11A	10	2018	Year-2	1	0	Fall
10/27/2018	FP	13A	10	2018	Year-2	1	0	Fall
10/28/2018	FP	1M	10	2018	Year-2	1	0	Fall
10/28/2018	FP	2M	10	2018	Year-2		1	Fall
10/28/2018	FP	3M	10	2018	Year-2	1	0	Fall

Date	Species	Site	Month	Year	Survey Year	Presence	Data Gap	Season
10/28/2018	FP	4M	10	2018	Year-2		1	Fall
10/28/2018	FP	5M	10	2018	Year-2		1	Fall
10/28/2018	FP	6M	10	2018	Year-2		1	Fall
10/28/2018	FP	7M	10	2018	Year-2	0	0	Fall
10/28/2018	FP	10M	10	2018	Year-2		1	Fall
10/28/2018	FP	12M	10	2018	Year-2		1	Fall
10/28/2018	FP	14M	10	2018	Year-2	0	0	Fall
10/28/2018	FP	8A	10	2018	Year-2		1	Fall
10/28/2018	FP	9A	10	2018	Year-2	0	0	Fall
10/28/2018	FP	11A	10	2018	Year-2	1	0	Fall
10/28/2018	FP	13A	10	2018	Year-2	1	0	Fall
10/29/2018	FP	1M	10	2018	Year-2	1	0	Fall
10/29/2018	FP	2M	10	2018	Year-2		1	Fall
10/29/2018	FP	3M	10	2018	Year-2	1	0	Fall
10/29/2018	FP	4M	10	2018	Year-2		1	Fall
10/29/2018	FP	5M	10	2018	Year-2		1	Fall
10/29/2018	FP	6M	10	2018	Year-2		1	Fall
10/29/2018	FP	7M	10	2018	Year-2	1	0	Fall
10/29/2018	FP	10M	10	2018	Year-2		1	Fall
10/29/2018	FP	12M	10	2018	Year-2		1	Fall
10/29/2018	FP	14M	10	2018	Year-2	0	0	Fall
10/29/2018	FP	8A	10	2018	Year-2		1	Fall
10/29/2018	FP	9A	10	2018	Year-2	0	0	Fall
10/29/2018	FP	11A	10	2018	Year-2	1	0	Fall
10/29/2018	FP	13A	10	2018	Year-2	1	0	Fall
10/30/2018	FP	1M	10	2018	Year-2	1	0	Fall
10/30/2018	FP	2M	10	2018	Year-2		1	Fall
10/30/2018	FP	3M	10	2018	Year-2	1	0	Fall
10/30/2018	FP	4M	10	2018	Year-2		1	Fall
10/30/2018	FP	5M	10	2018	Year-2		1	Fall
10/30/2018	FP	6M	10	2018	Year-2		1	Fall
10/30/2018	FP	7M	10	2018	Year-2	1	0	Fall
10/30/2018	FP	10M	10	2018	Year-2		1	Fall
10/30/2018	FP	12M	10	2018	Year-2		1	Fall
10/30/2018	FP	14M	10	2018	Year-2	1	0	Fall
10/30/2018	FP	8A	10	2018	Year-2		1	Fall
10/30/2018	FP	9A	10	2018	Year-2	0	0	Fall
10/30/2018	FP	11A	10	2018	Year-2	1	0	Fall
10/30/2018	FP	13A	10	2018	Year-2	1	0	Fall
10/31/2018	FP	1M	10	2018	Year-2	1	0	Fall
10/31/2018	FP	2M	10	2018	Year-2		1	Fall
10/31/2018	FP	3M	10	2018	Year-2	1	0	Fall
10/31/2018	FP	4M	10	2018	Year-2		1	Fall
10/31/2018	FP	5M	10	2018	Year-2		1	Fall

Date	Species	Site	Month	Year	Survey Year	Presence	Data Gap	Season
10/31/2018	FP	6M	10	2018	Year-2		1	Fall
10/31/2018	FP	7M	10	2018	Year-2	1	0	Fall
10/31/2018	FP	10M	10	2018	Year-2		1	Fall
10/31/2018	FP	12M	10	2018	Year-2		1	Fall
10/31/2018	FP	14M	10	2018	Year-2	0	0	Fall
10/31/2018	FP	8A	10	2018	Year-2		1	Fall
10/31/2018	FP	9A	10	2018	Year-2	0	0	Fall
10/31/2018	FP	11A	10	2018	Year-2	1	0	Fall
10/31/2018	FP	13A	10	2018	Year-2	1	0	Fall
11/1/2018	FP	1M	11	2018	Year-2	1	0	Fall
11/1/2018	FP	2M	11	2018	Year-2		1	Fall
11/1/2018	FP	3M	11	2018	Year-2	1	0	Fall
11/1/2018	FP	4M	11	2018	Year-2		1	Fall
11/1/2018	FP	5M	11	2018	Year-2		1	Fall
11/1/2018	FP	6M	11	2018	Year-2		1	Fall
11/1/2018	FP	7M	11	2018	Year-2	0	0	Fall
11/1/2018	FP	10M	11	2018	Year-2		1	Fall
11/1/2018	FP	12M	11	2018	Year-2		1	Fall
11/1/2018	FP	14M	11	2018	Year-2	0	0	Fall
11/1/2018	FP	8A	11	2018	Year-2		1	Fall
11/1/2018	FP	9A	11	2018	Year-2	0	0	Fall
11/1/2018	FP	11A	11	2018	Year-2	1	0	Fall
11/1/2018	FP	13A	11	2018	Year-2	1	0	Fall
11/2/2018	FP	1M	11	2018	Year-2	1	0	Fall
11/2/2018	FP	2M	11	2018	Year-2		1	Fall
11/2/2018	FP	3M	11	2018	Year-2	1	0	Fall
11/2/2018	FP	4M	11	2018	Year-2		1	Fall
11/2/2018	FP	5M	11	2018	Year-2		1	Fall
11/2/2018	FP	6M	11	2018	Year-2		1	Fall
11/2/2018	FP	7M	11	2018	Year-2	1	0	Fall
11/2/2018	FP	10M	11	2018	Year-2		1	Fall
11/2/2018	FP	12M	11	2018	Year-2		1	Fall
11/2/2018	FP	14M	11	2018	Year-2	0	0	Fall
11/2/2018	FP	8A	11	2018	Year-2		1	Fall
11/2/2018	FP	9A	11	2018	Year-2	0	0	Fall
11/2/2018	FP	11A	11	2018	Year-2	1	0	Fall
11/2/2018	FP	13A	11	2018	Year-2	1	0	Fall
11/3/2018	FP	1M	11	2018	Year-2	1	0	Fall
11/3/2018	FP	2M	11	2018	Year-2		1	Fall
11/3/2018	FP	3M	11	2018	Year-2	1	0	Fall
11/3/2018	FP	4M	11	2018	Year-2		1	Fall
11/3/2018	FP	5M	11	2018	Year-2		1	Fall
11/3/2018	FP	6M	11	2018	Year-2		1	Fall
11/3/2018	FP	7M	11	2018	Year-2	1	0	Fall

Date	Species	Site	Month	Year	Survey Year	Presence	Data Gap	Season
11/3/2018	FP	10M	11	2018	Year-2		1	Fall
11/3/2018	FP	12M	11	2018	Year-2		1	Fall
11/3/2018	FP	14M	11	2018	Year-2	0	0	Fall
11/3/2018	FP	8A	11	2018	Year-2		1	Fall
11/3/2018	FP	9A	11	2018	Year-2	0	0	Fall
11/3/2018	FP	11A	11	2018	Year-2	1	0	Fall
11/3/2018	FP	13A	11	2018	Year-2	1	0	Fall
11/4/2018	FP	1M	11	2018	Year-2	1	0	Fall
11/4/2018	FP	2M	11	2018	Year-2		1	Fall
11/4/2018	FP	3M	11	2018	Year-2	1	0	Fall
11/4/2018	FP	4M	11	2018	Year-2		1	Fall
11/4/2018	FP	5M	11	2018	Year-2		1	Fall
11/4/2018	FP	6M	11	2018	Year-2		1	Fall
11/4/2018	FP	7M	11	2018	Year-2	0	0	Fall
11/4/2018	FP	10M	11	2018	Year-2		1	Fall
11/4/2018	FP	12M	11	2018	Year-2		1	Fall
11/4/2018	FP	14M	11	2018	Year-2	0	0	Fall
11/4/2018	FP	8A	11	2018	Year-2		1	Fall
11/4/2018	FP	9A	11	2018	Year-2	0	0	Fall
11/4/2018	FP	11A	11	2018	Year-2	1	0	Fall
11/4/2018	FP	13A	11	2018	Year-2	1	0	Fall
11/5/2018	FP	1M	11	2018	Year-2	1	0	Fall
11/5/2018	FP	2M	11	2018	Year-2		1	Fall
11/5/2018	FP	3M	11	2018	Year-2	1	0	Fall
11/5/2018	FP	4M	11	2018	Year-2		1	Fall
11/5/2018	FP	5M	11	2018	Year-2		1	Fall
11/5/2018	FP	6M	11	2018	Year-2		1	Fall
11/5/2018	FP	7M	11	2018	Year-2	0	0	Fall
11/5/2018	FP	10M	11	2018	Year-2		1	Fall
11/5/2018	FP	12M	11	2018	Year-2		1	Fall
11/5/2018	FP	14M	11	2018	Year-2	0	0	Fall
11/5/2018	FP	8A	11	2018	Year-2		1	Fall
11/5/2018	FP	9A	11	2018	Year-2	0	0	Fall
11/5/2018	FP	11A	11	2018	Year-2	1	0	Fall
11/5/2018	FP	13A	11	2018	Year-2	1	0	Fall
11/6/2018	FP	1M	11	2018	Year-2	1	0	Fall
11/6/2018	FP	2M	11	2018	Year-2		1	Fall
11/6/2018	FP	3M	11	2018	Year-2	1	0	Fall
11/6/2018	FP	4M	11	2018	Year-2		1	Fall
11/6/2018	FP	5M	11	2018	Year-2		1	Fall
11/6/2018	FP	6M	11	2018	Year-2		1	Fall
11/6/2018	FP	7M	11	2018	Year-2	1	0	Fall
11/6/2018	FP	10M	11	2018	Year-2		1	Fall
11/6/2018	FP	12M	11	2018	Year-2		1	Fall

Date	Species	Site	Month	Year	Survey Year	Presence	Data Gap	Season
11/6/2018	FP	14M	11	2018	Year-2	0	0	Fall
11/6/2018	FP	8A	11	2018	Year-2		1	Fall
11/6/2018	FP	9A	11	2018	Year-2	0	0	Fall
11/6/2018	FP	11A	11	2018	Year-2	1	0	Fall
11/6/2018	FP	13A	11	2018	Year-2	1	0	Fall
11/7/2018	FP	1M	11	2018	Year-2	1	0	Fall
11/7/2018	FP	2M	11	2018	Year-2		1	Fall
11/7/2018	FP	3M	11	2018	Year-2	1	0	Fall
11/7/2018	FP	4M	11	2018	Year-2		1	Fall
11/7/2018	FP	5M	11	2018	Year-2		1	Fall
11/7/2018	FP	6M	11	2018	Year-2		1	Fall
11/7/2018	FP	7M	11	2018	Year-2	0	0	Fall
11/7/2018	FP	10M	11	2018	Year-2		1	Fall
11/7/2018	FP	12M	11	2018	Year-2		1	Fall
11/7/2018	FP	14M	11	2018	Year-2	0	0	Fall
11/7/2018	FP	8A	11	2018	Year-2		1	Fall
11/7/2018	FP	9A	11	2018	Year-2	0	0	Fall
11/7/2018	FP	11A	11	2018	Year-2	1	0	Fall
11/7/2018	FP	13A	11	2018	Year-2	1	0	Fall
11/8/2018	FP	1M	11	2018	Year-2	1	0	Fall
11/8/2018	FP	2M	11	2018	Year-2		1	Fall
11/8/2018	FP	3M	11	2018	Year-2	1	0	Fall
11/8/2018	FP	4M	11	2018	Year-2		1	Fall
11/8/2018	FP	5M	11	2018	Year-2		1	Fall
11/8/2018	FP	6M	11	2018	Year-2		1	Fall
11/8/2018	FP	7M	11	2018	Year-2	0	0	Fall
11/8/2018	FP	10M	11	2018	Year-2		1	Fall
11/8/2018	FP	12M	11	2018	Year-2		1	Fall
11/8/2018	FP	14M	11	2018	Year-2	0	0	Fall
11/8/2018	FP	8A	11	2018	Year-2		1	Fall
11/8/2018	FP	9A	11	2018	Year-2	0	0	Fall
11/8/2018	FP	11A	11	2018	Year-2	1	0	Fall
11/8/2018	FP	13A	11	2018	Year-2	1	0	Fall
11/9/2018	FP	1M	11	2018	Year-2	1	0	Fall
11/9/2018	FP	2M	11	2018	Year-2		1	Fall
11/9/2018	FP	3M	11	2018	Year-2	1	0	Fall
11/9/2018	FP	4M	11	2018	Year-2		1	Fall
11/9/2018	FP	5M	11	2018	Year-2		1	Fall
11/9/2018	FP	6M	11	2018	Year-2		1	Fall
11/9/2018	FP	7M	11	2018	Year-2	1	0	Fall
11/9/2018	FP	10M	11	2018	Year-2		1	Fall
11/9/2018	FP	12M	11	2018	Year-2		1	Fall
11/9/2018	FP	14M	11	2018	Year-2	0	0	Fall
11/9/2018	FP	8A	11	2018	Year-2		1	Fall

Date	Species	Site	Month	Year	Survey Year	Presence	Data Gap	Season
11/9/2018	FP	9A	11	2018	Year-2	0	0	Fall
11/9/2018	FP	11A	11	2018	Year-2	1	0	Fall
11/9/2018	FP	13A	11	2018	Year-2	1	0	Fall
11/10/2018	FP	1M	11	2018	Year-2	1	0	Fall
11/10/2018	FP	2M	11	2018	Year-2		1	Fall
11/10/2018	FP	3M	11	2018	Year-2	1	0	Fall
11/10/2018	FP	4M	11	2018	Year-2		1	Fall
11/10/2018	FP	5M	11	2018	Year-2		1	Fall
11/10/2018	FP	6M	11	2018	Year-2		1	Fall
11/10/2018	FP	7M	11	2018	Year-2	1	0	Fall
11/10/2018	FP	10M	11	2018	Year-2		1	Fall
11/10/2018	FP	12M	11	2018	Year-2		1	Fall
11/10/2018	FP	14M	11	2018	Year-2	0	0	Fall
11/10/2018	FP	8A	11	2018	Year-2		1	Fall
11/10/2018	FP	9A	11	2018	Year-2	0	0	Fall
11/10/2018	FP	11A	11	2018	Year-2	1	0	Fall
11/10/2018	FP	13A	11	2018	Year-2	1	0	Fall
11/11/2018	FP	1M	11	2018	Year-2	1	0	Fall
11/11/2018	FP	2M	11	2018	Year-2		1	Fall
11/11/2018	FP	3M	11	2018	Year-2	1	0	Fall
11/11/2018	FP	4M	11	2018	Year-2		1	Fall
11/11/2018	FP	5M	11	2018	Year-2		1	Fall
11/11/2018	FP	6M	11	2018	Year-2		1	Fall
11/11/2018	FP	7M	11	2018	Year-2	0	0	Fall
11/11/2018	FP	10M	11	2018	Year-2		1	Fall
11/11/2018	FP	12M	11	2018	Year-2		1	Fall
11/11/2018	FP	14M	11	2018	Year-2	0	0	Fall
11/11/2018	FP	8A	11	2018	Year-2		1	Fall
11/11/2018	FP	9A	11	2018	Year-2	0	0	Fall
11/11/2018	FP	11A	11	2018	Year-2	1	0	Fall
11/11/2018	FP	13A	11	2018	Year-2	1	0	Fall
11/12/2018	FP	1M	11	2018	Year-2	1	0	Fall
11/12/2018	FP	2M	11	2018	Year-2		1	Fall
11/12/2018	FP	3M	11	2018	Year-2	1	0	Fall
11/12/2018	FP	4M	11	2018	Year-2		1	Fall
11/12/2018	FP	5M	11	2018	Year-2		1	Fall
11/12/2018	FP	6M	11	2018	Year-2		1	Fall
11/12/2018	FP	7M	11	2018	Year-2	1	0	Fall
11/12/2018	FP	10M	11	2018	Year-2		1	Fall
11/12/2018	FP	12M	11	2018	Year-2		1	Fall
11/12/2018	FP	14M	11	2018	Year-2	0	0	Fall
11/12/2018	FP	8A	11	2018	Year-2		1	Fall
11/12/2018	FP	9A	11	2018	Year-2	0	0	Fall
11/12/2018	FP	11A	11	2018	Year-2	1	0	Fall

Date	Species	Site	Month	Year	Survey Year	Presence	Data Gap	Season
11/12/2018	FP	13A	11	2018	Year-2	1	0	Fall
11/13/2018	FP	1M	11	2018	Year-2	1	0	Fall
11/13/2018	FP	2M	11	2018	Year-2		1	Fall
11/13/2018	FP	3M	11	2018	Year-2	1	0	Fall
11/13/2018	FP	4M	11	2018	Year-2		1	Fall
11/13/2018	FP	5M	11	2018	Year-2		1	Fall
11/13/2018	FP	6M	11	2018	Year-2		1	Fall
11/13/2018	FP	7M	11	2018	Year-2	1	0	Fall
11/13/2018	FP	10M	11	2018	Year-2		1	Fall
11/13/2018	FP	12M	11	2018	Year-2		1	Fall
11/13/2018	FP	14M	11	2018	Year-2	0	0	Fall
11/13/2018	FP	8A	11	2018	Year-2		1	Fall
11/13/2018	FP	9A	11	2018	Year-2	0	0	Fall
11/13/2018	FP	11A	11	2018	Year-2	1	0	Fall
11/13/2018	FP	13A	11	2018	Year-2	1	0	Fall
11/14/2018	FP	1M	11	2018	Year-2	1	0	Fall
11/14/2018	FP	2M	11	2018	Year-2		1	Fall
11/14/2018	FP	3M	11	2018	Year-2	1	0	Fall
11/14/2018	FP	4M	11	2018	Year-2		1	Fall
11/14/2018	FP	5M	11	2018	Year-2		1	Fall
11/14/2018	FP	6M	11	2018	Year-2		1	Fall
11/14/2018	FP	7M	11	2018	Year-2	0	0	Fall
11/14/2018	FP	10M	11	2018	Year-2		1	Fall
11/14/2018	FP	12M	11	2018	Year-2		1	Fall
11/14/2018	FP	14M	11	2018	Year-2	0	0	Fall
11/14/2018	FP	8A	11	2018	Year-2		1	Fall
11/14/2018	FP	9A	11	2018	Year-2	0	0	Fall
11/14/2018	FP	11A	11	2018	Year-2	1	0	Fall
11/14/2018	FP	13A	11	2018	Year-2	1	0	Fall
11/15/2018	FP	1M	11	2018	Year-2	1	0	Fall
11/15/2018	FP	2M	11	2018	Year-2		1	Fall
11/15/2018	FP	3M	11	2018	Year-2	1	0	Fall
11/15/2018	FP	4M	11	2018	Year-2		1	Fall
11/15/2018	FP	5M	11	2018	Year-2		1	Fall
11/15/2018	FP	6M	11	2018	Year-2		1	Fall
11/15/2018	FP	7M	11	2018	Year-2	0	0	Fall
11/15/2018	FP	10M	11	2018	Year-2		1	Fall
11/15/2018	FP	12M	11	2018	Year-2		1	Fall
11/15/2018	FP	14M	11	2018	Year-2	0	0	Fall
11/15/2018	FP	8A	11	2018	Year-2		1	Fall
11/15/2018	FP	9A	11	2018	Year-2	0	0	Fall
11/15/2018	FP	11A	11	2018	Year-2	1	0	Fall
11/15/2018	FP	13A	11	2018	Year-2	1	0	Fall
11/16/2018	FP	1M	11	2018	Year-2	1	0	Fall

Date	Species	Site	Month	Year	Survey Year	Presence	Data Gap	Season
11/16/2018	FP	2M	11	2018	Year-2		1	Fall
11/16/2018	FP	3M	11	2018	Year-2	1	0	Fall
11/16/2018	FP	4M	11	2018	Year-2		1	Fall
11/16/2018	FP	5M	11	2018	Year-2		1	Fall
11/16/2018	FP	6M	11	2018	Year-2		1	Fall
11/16/2018	FP	7M	11	2018	Year-2	0	0	Fall
11/16/2018	FP	10M	11	2018	Year-2		1	Fall
11/16/2018	FP	12M	11	2018	Year-2		1	Fall
11/16/2018	FP	14M	11	2018	Year-2	0	0	Fall
11/16/2018	FP	8A	11	2018	Year-2		1	Fall
11/16/2018	FP	9A	11	2018	Year-2	0	0	Fall
11/16/2018	FP	11A	11	2018	Year-2	1	0	Fall
11/16/2018	FP	13A	11	2018	Year-2	1	0	Fall
11/17/2018	FP	1M	11	2018	Year-2	1	0	Fall
11/17/2018	FP	2M	11	2018	Year-2		1	Fall
11/17/2018	FP	3M	11	2018	Year-2	1	0	Fall
11/17/2018	FP	4M	11	2018	Year-2		1	Fall
11/17/2018	FP	5M	11	2018	Year-2		1	Fall
11/17/2018	FP	6M	11	2018	Year-2		1	Fall
11/17/2018	FP	7M	11	2018	Year-2	1	0	Fall
11/17/2018	FP	10M	11	2018	Year-2		1	Fall
11/17/2018	FP	12M	11	2018	Year-2		1	Fall
11/17/2018	FP	14M	11	2018	Year-2	0	0	Fall
11/17/2018	FP	8A	11	2018	Year-2		1	Fall
11/17/2018	FP	9A	11	2018	Year-2	1	0	Fall
11/17/2018	FP	11A	11	2018	Year-2	1	0	Fall
11/17/2018	FP	13A	11	2018	Year-2	1	0	Fall
11/18/2018	FP	1M	11	2018	Year-2	1	0	Fall
11/18/2018	FP	2M	11	2018	Year-2		1	Fall
11/18/2018	FP	3M	11	2018	Year-2	1	0	Fall
11/18/2018	FP	4M	11	2018	Year-2		1	Fall
11/18/2018	FP	5M	11	2018	Year-2		1	Fall
11/18/2018	FP	6M	11	2018	Year-2		1	Fall
11/18/2018	FP	7M	11	2018	Year-2	1	0	Fall
11/18/2018	FP	10M	11	2018	Year-2		1	Fall
11/18/2018	FP	12M	11	2018	Year-2		1	Fall
11/18/2018	FP	14M	11	2018	Year-2	0	0	Fall
11/18/2018	FP	8A	11	2018	Year-2		1	Fall
11/18/2018	FP	9A	11	2018	Year-2	1	0	Fall
11/18/2018	FP	11A	11	2018	Year-2	1	0	Fall
11/18/2018	FP	13A	11	2018	Year-2	1	0	Fall
11/19/2018	FP	1M	11	2018	Year-2	1	0	Fall
11/19/2018	FP	2M	11	2018	Year-2		1	Fall
11/19/2018	FP	3M	11	2018	Year-2	1	0	Fall

Date	Species	Site	Month	Year	Survey Year	Presence	Data Gap	Season
11/19/2018	FP	4M	11	2018	Year-2		1	Fall
11/19/2018	FP	5M	11	2018	Year-2		1	Fall
11/19/2018	FP	6M	11	2018	Year-2		1	Fall
11/19/2018	FP	7M	11	2018	Year-2	0	0	Fall
11/19/2018	FP	10M	11	2018	Year-2		1	Fall
11/19/2018	FP	12M	11	2018	Year-2		1	Fall
11/19/2018	FP	14M	11	2018	Year-2	0	0	Fall
11/19/2018	FP	8A	11	2018	Year-2		1	Fall
11/19/2018	FP	9A	11	2018	Year-2	1	0	Fall
11/19/2018	FP	11A	11	2018	Year-2	1	0	Fall
11/19/2018	FP	13A	11	2018	Year-2	1	0	Fall
11/20/2018	FP	1M	11	2018	Year-2	1	0	Fall
11/20/2018	FP	2M	11	2018	Year-2		1	Fall
11/20/2018	FP	3M	11	2018	Year-2	1	0	Fall
11/20/2018	FP	4M	11	2018	Year-2		1	Fall
11/20/2018	FP	5M	11	2018	Year-2		1	Fall
11/20/2018	FP	6M	11	2018	Year-2		1	Fall
11/20/2018	FP	7M	11	2018	Year-2	1	0	Fall
11/20/2018	FP	10M	11	2018	Year-2		1	Fall
11/20/2018	FP	12M	11	2018	Year-2		1	Fall
11/20/2018	FP	14M	11	2018	Year-2	0	0	Fall
11/20/2018	FP	8A	11	2018	Year-2		1	Fall
11/20/2018	FP	9A	11	2018	Year-2	1	0	Fall
11/20/2018	FP	11A	11	2018	Year-2	1	0	Fall
11/20/2018	FP	13A	11	2018	Year-2	1	0	Fall
11/21/2018	FP	1M	11	2018	Year-2	1	0	Fall
11/21/2018	FP	2M	11	2018	Year-2		1	Fall
11/21/2018	FP	3M	11	2018	Year-2	1	0	Fall
11/21/2018	FP	4M	11	2018	Year-2		1	Fall
11/21/2018	FP	5M	11	2018	Year-2		1	Fall
11/21/2018	FP	6M	11	2018	Year-2		1	Fall
11/21/2018	FP	7M	11	2018	Year-2	1	0	Fall
11/21/2018	FP	10M	11	2018	Year-2		1	Fall
11/21/2018	FP	12M	11	2018	Year-2		1	Fall
11/21/2018	FP	14M	11	2018	Year-2	0	0	Fall
11/21/2018	FP	8A	11	2018	Year-2		1	Fall
11/21/2018	FP	9A	11	2018	Year-2	1	0	Fall
11/21/2018	FP	11A	11	2018	Year-2	1	0	Fall
11/21/2018	FP	13A	11	2018	Year-2	1	0	Fall
11/22/2018	FP	1M	11	2018	Year-2	1	0	Fall
11/22/2018	FP	2M	11	2018	Year-2		1	Fall
11/22/2018	FP	3M	11	2018	Year-2	1	0	Fall
11/22/2018	FP	4M	11	2018	Year-2		1	Fall
11/22/2018	FP	5M	11	2018	Year-2		1	Fall

Date	Species	Site	Month	Year	Survey Year	Presence	Data Gap	Season
11/22/2018	FP	6M	11	2018	Year-2		1	Fall
11/22/2018	FP	7M	11	2018	Year-2	1	0	Fall
11/22/2018	FP	10M	11	2018	Year-2		1	Fall
11/22/2018	FP	12M	11	2018	Year-2		1	Fall
11/22/2018	FP	14M	11	2018	Year-2	0	0	Fall
11/22/2018	FP	8A	11	2018	Year-2		1	Fall
11/22/2018	FP	9A	11	2018	Year-2	1	0	Fall
11/22/2018	FP	11A	11	2018	Year-2	1	0	Fall
11/22/2018	FP	13A	11	2018	Year-2	1	0	Fall
11/23/2018	FP	1M	11	2018	Year-2	1	0	Fall
11/23/2018	FP	2M	11	2018	Year-2		1	Fall
11/23/2018	FP	3M	11	2018	Year-2	1	0	Fall
11/23/2018	FP	4M	11	2018	Year-2		1	Fall
11/23/2018	FP	5M	11	2018	Year-2		1	Fall
11/23/2018	FP	6M	11	2018	Year-2		1	Fall
11/23/2018	FP	7M	11	2018	Year-2	1	0	Fall
11/23/2018	FP	10M	11	2018	Year-2		1	Fall
11/23/2018	FP	12M	11	2018	Year-2		1	Fall
11/23/2018	FP	14M	11	2018	Year-2	0	0	Fall
11/23/2018	FP	8A	11	2018	Year-2		1	Fall
11/23/2018	FP	9A	11	2018	Year-2	1	0	Fall
11/23/2018	FP	11A	11	2018	Year-2	1	0	Fall
11/23/2018	FP	13A	11	2018	Year-2	1	0	Fall
11/24/2018	FP	1M	11	2018	Year-2	1	0	Fall
11/24/2018	FP	2M	11	2018	Year-2		1	Fall
11/24/2018	FP	3M	11	2018	Year-2	1	0	Fall
11/24/2018	FP	4M	11	2018	Year-2		1	Fall
11/24/2018	FP	5M	11	2018	Year-2		1	Fall
11/24/2018	FP	6M	11	2018	Year-2		1	Fall
11/24/2018	FP	7M	11	2018	Year-2	1	0	Fall
11/24/2018	FP	10M	11	2018	Year-2		1	Fall
11/24/2018	FP	12M	11	2018	Year-2		1	Fall
11/24/2018	FP	14M	11	2018	Year-2	0	0	Fall
11/24/2018	FP	8A	11	2018	Year-2		1	Fall
11/24/2018	FP	9A	11	2018	Year-2	1	0	Fall
11/24/2018	FP	11A	11	2018	Year-2	1	0	Fall
11/24/2018	FP	13A	11	2018	Year-2	1	0	Fall
11/25/2018	FP	1M	11	2018	Year-2	0	0	Fall
11/25/2018	FP	2M	11	2018	Year-2		1	Fall
11/25/2018	FP	3M	11	2018	Year-2	0	0	Fall
11/25/2018	FP	4M	11	2018	Year-2		1	Fall
11/25/2018	FP	5M	11	2018	Year-2		1	Fall
11/25/2018	FP	6M	11	2018	Year-2		1	Fall
11/25/2018	FP	7M	11	2018	Year-2	1	0	Fall

Date	Species	Site	Month	Year	Survey Year	Presence	Data Gap	Season
11/25/2018	FP	10M	11	2018	Year-2		1	Fall
11/25/2018	FP	12M	11	2018	Year-2		1	Fall
11/25/2018	FP	14M	11	2018	Year-2	0	0	Fall
11/25/2018	FP	8A	11	2018	Year-2		1	Fall
11/25/2018	FP	9A	11	2018	Year-2	1	0	Fall
11/25/2018	FP	11A	11	2018	Year-2	1	0	Fall
11/25/2018	FP	13A	11	2018	Year-2	1	0	Fall
11/26/2018	FP	1M	11	2018	Year-2	1	0	Fall
11/26/2018	FP	2M	11	2018	Year-2		1	Fall
11/26/2018	FP	3M	11	2018	Year-2	1	0	Fall
11/26/2018	FP	4M	11	2018	Year-2		1	Fall
11/26/2018	FP	5M	11	2018	Year-2		1	Fall
11/26/2018	FP	6M	11	2018	Year-2		1	Fall
11/26/2018	FP	7M	11	2018	Year-2	1	0	Fall
11/26/2018	FP	10M	11	2018	Year-2		1	Fall
11/26/2018	FP	12M	11	2018	Year-2		1	Fall
11/26/2018	FP	14M	11	2018	Year-2	0	0	Fall
11/26/2018	FP	8A	11	2018	Year-2		1	Fall
11/26/2018	FP	9A	11	2018	Year-2	1	0	Fall
11/26/2018	FP	11A	11	2018	Year-2	1	0	Fall
11/26/2018	FP	13A	11	2018	Year-2	1	0	Fall
11/27/2018	FP	1M	11	2018	Year-2	1	0	Fall
11/27/2018	FP	2M	11	2018	Year-2		1	Fall
11/27/2018	FP	3M	11	2018	Year-2	1	0	Fall
11/27/2018	FP	4M	11	2018	Year-2		1	Fall
11/27/2018	FP	5M	11	2018	Year-2		1	Fall
11/27/2018	FP	6M	11	2018	Year-2		1	Fall
11/27/2018	FP	7M	11	2018	Year-2	0	0	Fall
11/27/2018	FP	10M	11	2018	Year-2		1	Fall
11/27/2018	FP	12M	11	2018	Year-2		1	Fall
11/27/2018	FP	14M	11	2018	Year-2	0	0	Fall
11/27/2018	FP	8A	11	2018	Year-2		1	Fall
11/27/2018	FP	9A	11	2018	Year-2	1	0	Fall
11/27/2018	FP	11A	11	2018	Year-2	1	0	Fall
11/27/2018	FP	13A	11	2018	Year-2	1	0	Fall
11/28/2018	FP	1M	11	2018	Year-2	1	0	Fall
11/28/2018	FP	2M	11	2018	Year-2		1	Fall
11/28/2018	FP	3M	11	2018	Year-2	1	0	Fall
11/28/2018	FP	4M	11	2018	Year-2		1	Fall
11/28/2018	FP	5M	11	2018	Year-2		1	Fall
11/28/2018	FP	6M	11	2018	Year-2		1	Fall
11/28/2018	FP	7M	11	2018	Year-2	0	0	Fall
11/28/2018	FP	10M	11	2018	Year-2		1	Fall
11/28/2018	FP	12M	11	2018	Year-2		1	Fall

Date	Species	Site	Month	Year	Survey Year	Presence	Data Gap	Season
11/28/2018	FP	14M	11	2018	Year-2	0	0	Fall
11/28/2018	FP	8A	11	2018	Year-2		1	Fall
11/28/2018	FP	9A	11	2018	Year-2	1	0	Fall
11/28/2018	FP	11A	11	2018	Year-2	1	0	Fall
11/28/2018	FP	13A	11	2018	Year-2	1	0	Fall
11/29/2018	FP	1M	11	2018	Year-2	1	0	Fall
11/29/2018	FP	2M	11	2018	Year-2		1	Fall
11/29/2018	FP	3M	11	2018	Year-2	1	0	Fall
11/29/2018	FP	4M	11	2018	Year-2		1	Fall
11/29/2018	FP	5M	11	2018	Year-2		1	Fall
11/29/2018	FP	6M	11	2018	Year-2		1	Fall
11/29/2018	FP	7M	11	2018	Year-2	1	0	Fall
11/29/2018	FP	10M	11	2018	Year-2		1	Fall
11/29/2018	FP	12M	11	2018	Year-2		1	Fall
11/29/2018	FP	14M	11	2018	Year-2	0	0	Fall
11/29/2018	FP	8A	11	2018	Year-2		1	Fall
11/29/2018	FP	9A	11	2018	Year-2	0	0	Fall
11/29/2018	FP	11A	11	2018	Year-2	1	0	Fall
11/29/2018	FP	13A	11	2018	Year-2	1	0	Fall
11/30/2018	FP	1M	11	2018	Year-2	0	0	Fall
11/30/2018	FP	2M	11	2018	Year-2		1	Fall
11/30/2018	FP	3M	11	2018	Year-2	0	0	Fall
11/30/2018	FP	4M	11	2018	Year-2		1	Fall
11/30/2018	FP	5M	11	2018	Year-2		1	Fall
11/30/2018	FP	6M	11	2018	Year-2		1	Fall
11/30/2018	FP	7M	11	2018	Year-2	1	0	Fall
11/30/2018	FP	10M	11	2018	Year-2		1	Fall
11/30/2018	FP	12M	11	2018	Year-2		1	Fall
11/30/2018	FP	14M	11	2018	Year-2	0	0	Fall
11/30/2018	FP	8A	11	2018	Year-2		1	Fall
11/30/2018	FP	9A	11	2018	Year-2	1	0	Fall
11/30/2018	FP	11A	11	2018	Year-2	1	0	Fall
11/30/2018	FP	13A	11	2018	Year-2	1	0	Fall
12/1/2018	FP	1M	12	2018	Year-2	1	0	Winter
12/1/2018	FP	2M	12	2018	Year-2		1	Winter
12/1/2018	FP	3M	12	2018	Year-2	1	0	Winter
12/1/2018	FP	4M	12	2018	Year-2		1	Winter
12/1/2018	FP	5M	12	2018	Year-2		1	Winter
12/1/2018	FP	6M	12	2018	Year-2		1	Winter
12/1/2018	FP	7M	12	2018	Year-2	1	0	Winter
12/1/2018	FP	10M	12	2018	Year-2		1	Winter
12/1/2018	FP	12M	12	2018	Year-2		1	Winter
12/1/2018	FP	14M	12	2018	Year-2	0	0	Winter
12/1/2018	FP	8A	12	2018	Year-2		1	Winter

Date	Species	Site	Month	Year	Survey Year	Presence	Data Gap	Season
12/1/2018	FP	9A	12	2018	Year-2	1	0	Winter
12/1/2018	FP	11A	12	2018	Year-2	1	0	Winter
12/1/2018	FP	13A	12	2018	Year-2	0	0	Winter
12/2/2018	FP	1M	12	2018	Year-2	1	0	Winter
12/2/2018	FP	2M	12	2018	Year-2		1	Winter
12/2/2018	FP	3M	12	2018	Year-2	1	0	Winter
12/2/2018	FP	4M	12	2018	Year-2		1	Winter
12/2/2018	FP	5M	12	2018	Year-2		1	Winter
12/2/2018	FP	6M	12	2018	Year-2		1	Winter
12/2/2018	FP	7M	12	2018	Year-2	1	0	Winter
12/2/2018	FP	10M	12	2018	Year-2		1	Winter
12/2/2018	FP	12M	12	2018	Year-2		1	Winter
12/2/2018	FP	14M	12	2018	Year-2	0	0	Winter
12/2/2018	FP	8A	12	2018	Year-2		1	Winter
12/2/2018	FP	9A	12	2018	Year-2	1	0	Winter
12/2/2018	FP	11A	12	2018	Year-2	1	0	Winter
12/2/2018	FP	13A	12	2018	Year-2	1	0	Winter
12/3/2018	FP	1M	12	2018	Year-2	1	0	Winter
12/3/2018	FP	2M	12	2018	Year-2		1	Winter
12/3/2018	FP	3M	12	2018	Year-2	1	0	Winter
12/3/2018	FP	4M	12	2018	Year-2		1	Winter
12/3/2018	FP	5M	12	2018	Year-2		1	Winter
12/3/2018	FP	6M	12	2018	Year-2		1	Winter
12/3/2018	FP	7M	12	2018	Year-2	0	0	Winter
12/3/2018	FP	10M	12	2018	Year-2		1	Winter
12/3/2018	FP	12M	12	2018	Year-2		1	Winter
12/3/2018	FP	14M	12	2018	Year-2	0	0	Winter
12/3/2018	FP	8A	12	2018	Year-2		1	Winter
12/3/2018	FP	9A	12	2018	Year-2	1	0	Winter
12/3/2018	FP	11A	12	2018	Year-2	1	0	Winter
12/3/2018	FP	13A	12	2018	Year-2	1	0	Winter
12/4/2018	FP	1M	12	2018	Year-2	1	0	Winter
12/4/2018	FP	2M	12	2018	Year-2		1	Winter
12/4/2018	FP	3M	12	2018	Year-2	1	0	Winter
12/4/2018	FP	4M	12	2018	Year-2		1	Winter
12/4/2018	FP	5M	12	2018	Year-2		1	Winter
12/4/2018	FP	6M	12	2018	Year-2		1	Winter
12/4/2018	FP	7M	12	2018	Year-2	1	0	Winter
12/4/2018	FP	10M	12	2018	Year-2		1	Winter
12/4/2018	FP	12M	12	2018	Year-2		1	Winter
12/4/2018	FP	14M	12	2018	Year-2	0	0	Winter
12/4/2018	FP	8A	12	2018	Year-2		1	Winter
12/4/2018	FP	9A	12	2018	Year-2	1	0	Winter
12/4/2018	FP	11A	12	2018	Year-2	1	0	Winter

Date	Species	Site	Month	Year	Survey Year	Presence	Data Gap	Season
12/4/2018	FP	13A	12	2018	Year-2	1	0	Winter
12/5/2018	FP	1M	12	2018	Year-2	1	0	Winter
12/5/2018	FP	2M	12	2018	Year-2		1	Winter
12/5/2018	FP	3M	12	2018	Year-2	1	0	Winter
12/5/2018	FP	4M	12	2018	Year-2		1	Winter
12/5/2018	FP	5M	12	2018	Year-2		1	Winter
12/5/2018	FP	6M	12	2018	Year-2		1	Winter
12/5/2018	FP	7M	12	2018	Year-2	1	0	Winter
12/5/2018	FP	10M	12	2018	Year-2		1	Winter
12/5/2018	FP	12M	12	2018	Year-2		1	Winter
12/5/2018	FP	14M	12	2018	Year-2	0	0	Winter
12/5/2018	FP	8A	12	2018	Year-2		1	Winter
12/5/2018	FP	9A	12	2018	Year-2	1	0	Winter
12/5/2018	FP	11A	12	2018	Year-2	0	0	Winter
12/5/2018	FP	13A	12	2018	Year-2	1	0	Winter
12/6/2018	FP	1M	12	2018	Year-2	1	0	Winter
12/6/2018	FP	2M	12	2018	Year-2		1	Winter
12/6/2018	FP	3M	12	2018	Year-2	0	0	Winter
12/6/2018	FP	4M	12	2018	Year-2		1	Winter
12/6/2018	FP	5M	12	2018	Year-2		1	Winter
12/6/2018	FP	6M	12	2018	Year-2		1	Winter
12/6/2018	FP	7M	12	2018	Year-2	1	0	Winter
12/6/2018	FP	10M	12	2018	Year-2		1	Winter
12/6/2018	FP	12M	12	2018	Year-2		1	Winter
12/6/2018	FP	14M	12	2018	Year-2	0	0	Winter
12/6/2018	FP	8A	12	2018	Year-2		1	Winter
12/6/2018	FP	9A	12	2018	Year-2	1	0	Winter
12/6/2018	FP	11A	12	2018	Year-2	1	0	Winter
12/6/2018	FP	13A	12	2018	Year-2	1	0	Winter
12/7/2018	FP	1M	12	2018	Year-2	1	0	Winter
12/7/2018	FP	2M	12	2018	Year-2		1	Winter
12/7/2018	FP	3M	12	2018	Year-2	1	0	Winter
12/7/2018	FP	4M	12	2018	Year-2		1	Winter
12/7/2018	FP	5M	12	2018	Year-2		1	Winter
12/7/2018	FP	6M	12	2018	Year-2		1	Winter
12/7/2018	FP	7M	12	2018	Year-2	1	0	Winter
12/7/2018	FP	10M	12	2018	Year-2		1	Winter
12/7/2018	FP	12M	12	2018	Year-2		1	Winter
12/7/2018	FP	14M	12	2018	Year-2	0	0	Winter
12/7/2018	FP	8A	12	2018	Year-2		1	Winter
12/7/2018	FP	9A	12	2018	Year-2	1	0	Winter
12/7/2018	FP	11A	12	2018	Year-2	1	0	Winter
12/7/2018	FP	13A	12	2018	Year-2	0	0	Winter
12/8/2018	FP	1M	12	2018	Year-2	1	0	Winter

Date	Species	Site	Month	Year	Survey Year	Presence	Data Gap	Season
12/8/2018	FP	2M	12	2018	Year-2		1	Winter
12/8/2018	FP	3M	12	2018	Year-2	1	0	Winter
12/8/2018	FP	4M	12	2018	Year-2		1	Winter
12/8/2018	FP	5M	12	2018	Year-2		1	Winter
12/8/2018	FP	6M	12	2018	Year-2		1	Winter
12/8/2018	FP	7M	12	2018	Year-2	1	0	Winter
12/8/2018	FP	10M	12	2018	Year-2		1	Winter
12/8/2018	FP	12M	12	2018	Year-2		1	Winter
12/8/2018	FP	14M	12	2018	Year-2	0	0	Winter
12/8/2018	FP	8A	12	2018	Year-2		1	Winter
12/8/2018	FP	9A	12	2018	Year-2	1	0	Winter
12/8/2018	FP	11A	12	2018	Year-2	1	0	Winter
12/8/2018	FP	13A	12	2018	Year-2	1	0	Winter
12/9/2018	FP	1M	12	2018	Year-2	1	0	Winter
12/9/2018	FP	2M	12	2018	Year-2		1	Winter
12/9/2018	FP	3M	12	2018	Year-2	0	0	Winter
12/9/2018	FP	4M	12	2018	Year-2		1	Winter
12/9/2018	FP	5M	12	2018	Year-2		1	Winter
12/9/2018	FP	6M	12	2018	Year-2		1	Winter
12/9/2018	FP	7M	12	2018	Year-2	1	0	Winter
12/9/2018	FP	10M	12	2018	Year-2		1	Winter
12/9/2018	FP	12M	12	2018	Year-2		1	Winter
12/9/2018	FP	14M	12	2018	Year-2	0	0	Winter
12/9/2018	FP	8A	12	2018	Year-2		1	Winter
12/9/2018	FP	9A	12	2018	Year-2	0	0	Winter
12/9/2018	FP	11A	12	2018	Year-2	1	0	Winter
12/9/2018	FP	13A	12	2018	Year-2	1	0	Winter
12/10/2018	FP	1M	12	2018	Year-2	0	0	Winter
12/10/2018	FP	2M	12	2018	Year-2		1	Winter
12/10/2018	FP	3M	12	2018	Year-2	0	0	Winter
12/10/2018	FP	4M	12	2018	Year-2		1	Winter
12/10/2018	FP	5M	12	2018	Year-2		1	Winter
12/10/2018	FP	6M	12	2018	Year-2		1	Winter
12/10/2018	FP	7M	12	2018	Year-2	1	0	Winter
12/10/2018	FP	10M	12	2018	Year-2		1	Winter
12/10/2018	FP	12M	12	2018	Year-2		1	Winter
12/10/2018	FP	14M	12	2018	Year-2	0	0	Winter
12/10/2018	FP	8A	12	2018	Year-2		1	Winter
12/10/2018	FP	9A	12	2018	Year-2	0	0	Winter
12/10/2018	FP	11A	12	2018	Year-2	0	0	Winter
12/10/2018	FP	13A	12	2018	Year-2	1	0	Winter
12/11/2018	FP	1M	12	2018	Year-2	1	0	Winter
12/11/2018	FP	2M	12	2018	Year-2		1	Winter
12/11/2018	FP	3M	12	2018	Year-2	1	0	Winter

Date	Species	Site	Month	Year	Survey Year	Presence	Data Gap	Season
12/11/2018	FP	4M	12	2018	Year-2		1	Winter
12/11/2018	FP	5M	12	2018	Year-2		1	Winter
12/11/2018	FP	6M	12	2018	Year-2		1	Winter
12/11/2018	FP	7M	12	2018	Year-2	1	0	Winter
12/11/2018	FP	10M	12	2018	Year-2		1	Winter
12/11/2018	FP	12M	12	2018	Year-2		1	Winter
12/11/2018	FP	14M	12	2018	Year-2	0	0	Winter
12/11/2018	FP	8A	12	2018	Year-2		1	Winter
12/11/2018	FP	9A	12	2018	Year-2	1	0	Winter
12/11/2018	FP	11A	12	2018	Year-2	1	0	Winter
12/11/2018	FP	13A	12	2018	Year-2	1	0	Winter
12/12/2018	FP	1M	12	2018	Year-2	1	0	Winter
12/12/2018	FP	2M	12	2018	Year-2		1	Winter
12/12/2018	FP	3M	12	2018	Year-2	1	0	Winter
12/12/2018	FP	4M	12	2018	Year-2		1	Winter
12/12/2018	FP	5M	12	2018	Year-2		1	Winter
12/12/2018	FP	6M	12	2018	Year-2		1	Winter
12/12/2018	FP	7M	12	2018	Year-2	1	0	Winter
12/12/2018	FP	10M	12	2018	Year-2		1	Winter
12/12/2018	FP	12M	12	2018	Year-2		1	Winter
12/12/2018	FP	14M	12	2018	Year-2	0	0	Winter
12/12/2018	FP	8A	12	2018	Year-2		1	Winter
12/12/2018	FP	9A	12	2018	Year-2	1	0	Winter
12/12/2018	FP	11A	12	2018	Year-2	1	0	Winter
12/12/2018	FP	13A	12	2018	Year-2	1	0	Winter
12/13/2018	FP	1M	12	2018	Year-2	1	0	Winter
12/13/2018	FP	2M	12	2018	Year-2		1	Winter
12/13/2018	FP	3M	12	2018	Year-2	1	0	Winter
12/13/2018	FP	4M	12	2018	Year-2		1	Winter
12/13/2018	FP	5M	12	2018	Year-2		1	Winter
12/13/2018	FP	6M	12	2018	Year-2		1	Winter
12/13/2018	FP	7M	12	2018	Year-2	1	0	Winter
12/13/2018	FP	10M	12	2018	Year-2		1	Winter
12/13/2018	FP	12M	12	2018	Year-2		1	Winter
12/13/2018	FP	14M	12	2018	Year-2	0	0	Winter
12/13/2018	FP	8A	12	2018	Year-2		1	Winter
12/13/2018	FP	9A	12	2018	Year-2	1	0	Winter
12/13/2018	FP	11A	12	2018	Year-2	1	0	Winter
12/13/2018	FP	13A	12	2018	Year-2	1	0	Winter
12/14/2018	FP	1M	12	2018	Year-2	1	0	Winter
12/14/2018	FP	2M	12	2018	Year-2		1	Winter
12/14/2018	FP	3M	12	2018	Year-2	1	0	Winter
12/14/2018	FP	4M	12	2018	Year-2		1	Winter
12/14/2018	FP	5M	12	2018	Year-2		1	Winter

Date	Species	Site	Month	Year	Survey Year	Presence	Data Gap	Season
12/14/2018	FP	6M	12	2018	Year-2		1	Winter
12/14/2018	FP	7M	12	2018	Year-2	1	0	Winter
12/14/2018	FP	10M	12	2018	Year-2		1	Winter
12/14/2018	FP	12M	12	2018	Year-2		1	Winter
12/14/2018	FP	14M	12	2018	Year-2	0	0	Winter
12/14/2018	FP	8A	12	2018	Year-2		1	Winter
12/14/2018	FP	9A	12	2018	Year-2	1	0	Winter
12/14/2018	FP	11A	12	2018	Year-2	1	0	Winter
12/14/2018	FP	13A	12	2018	Year-2	1	0	Winter
12/15/2018	FP	1M	12	2018	Year-2	1	0	Winter
12/15/2018	FP	2M	12	2018	Year-2		1	Winter
12/15/2018	FP	3M	12	2018	Year-2	0	0	Winter
12/15/2018	FP	4M	12	2018	Year-2		1	Winter
12/15/2018	FP	5M	12	2018	Year-2		1	Winter
12/15/2018	FP	6M	12	2018	Year-2		1	Winter
12/15/2018	FP	7M	12	2018	Year-2	1	0	Winter
12/15/2018	FP	10M	12	2018	Year-2		1	Winter
12/15/2018	FP	12M	12	2018	Year-2		1	Winter
12/15/2018	FP	14M	12	2018	Year-2	0	0	Winter
12/15/2018	FP	8A	12	2018	Year-2		1	Winter
12/15/2018	FP	9A	12	2018	Year-2	1	0	Winter
12/15/2018	FP	11A	12	2018	Year-2	1	0	Winter
12/15/2018	FP	13A	12	2018	Year-2	1	0	Winter
12/16/2018	FP	1M	12	2018	Year-2	1	0	Winter
12/16/2018	FP	2M	12	2018	Year-2		1	Winter
12/16/2018	FP	3M	12	2018	Year-2	0	0	Winter
12/16/2018	FP	4M	12	2018	Year-2		1	Winter
12/16/2018	FP	5M	12	2018	Year-2		1	Winter
12/16/2018	FP	6M	12	2018	Year-2		1	Winter
12/16/2018	FP	7M	12	2018	Year-2	1	0	Winter
12/16/2018	FP	10M	12	2018	Year-2		1	Winter
12/16/2018	FP	12M	12	2018	Year-2		1	Winter
12/16/2018	FP	14M	12	2018	Year-2	0	0	Winter
12/16/2018	FP	8A	12	2018	Year-2		1	Winter
12/16/2018	FP	9A	12	2018	Year-2	1	0	Winter
12/16/2018	FP	11A	12	2018	Year-2	1	0	Winter
12/16/2018	FP	13A	12	2018	Year-2	0	0	Winter
12/17/2018	FP	1M	12	2018	Year-2	1	0	Winter
12/17/2018	FP	2M	12	2018	Year-2		1	Winter
12/17/2018	FP	3M	12	2018	Year-2	1	0	Winter
12/17/2018	FP	4M	12	2018	Year-2		1	Winter
12/17/2018	FP	5M	12	2018	Year-2		1	Winter
12/17/2018	FP	6M	12	2018	Year-2		1	Winter
12/17/2018	FP	7M	12	2018	Year-2	1	0	Winter

Date	Species	Site	Month	Year	Survey Year	Presence	Data Gap	Season
12/17/2018	FP	10M	12	2018	Year-2		1	Winter
12/17/2018	FP	12M	12	2018	Year-2		1	Winter
12/17/2018	FP	14M	12	2018	Year-2	0	0	Winter
12/17/2018	FP	8A	12	2018	Year-2		1	Winter
12/17/2018	FP	9A	12	2018	Year-2	1	0	Winter
12/17/2018	FP	11A	12	2018	Year-2	1	0	Winter
12/17/2018	FP	13A	12	2018	Year-2	1	0	Winter
12/18/2018	FP	1M	12	2018	Year-2	1	0	Winter
12/18/2018	FP	2M	12	2018	Year-2		1	Winter
12/18/2018	FP	3M	12	2018	Year-2	0	0	Winter
12/18/2018	FP	4M	12	2018	Year-2		1	Winter
12/18/2018	FP	5M	12	2018	Year-2		1	Winter
12/18/2018	FP	6M	12	2018	Year-2		1	Winter
12/18/2018	FP	7M	12	2018	Year-2	1	0	Winter
12/18/2018	FP	10M	12	2018	Year-2		1	Winter
12/18/2018	FP	12M	12	2018	Year-2		1	Winter
12/18/2018	FP	14M	12	2018	Year-2	0	0	Winter
12/18/2018	FP	8A	12	2018	Year-2		1	Winter
12/18/2018	FP	9A	12	2018	Year-2	1	0	Winter
12/18/2018	FP	11A	12	2018	Year-2	1	0	Winter
12/18/2018	FP	13A	12	2018	Year-2	1	0	Winter
12/19/2018	FP	1M	12	2018	Year-2	1	0	Winter
12/19/2018	FP	2M	12	2018	Year-2		1	Winter
12/19/2018	FP	3M	12	2018	Year-2	1	0	Winter
12/19/2018	FP	4M	12	2018	Year-2		1	Winter
12/19/2018	FP	5M	12	2018	Year-2		1	Winter
12/19/2018	FP	6M	12	2018	Year-2		1	Winter
12/19/2018	FP	7M	12	2018	Year-2	1	0	Winter
12/19/2018	FP	10M	12	2018	Year-2		1	Winter
12/19/2018	FP	12M	12	2018	Year-2		1	Winter
12/19/2018	FP	14M	12	2018	Year-2	0	0	Winter
12/19/2018	FP	8A	12	2018	Year-2		1	Winter
12/19/2018	FP	9A	12	2018	Year-2	1	0	Winter
12/19/2018	FP	11A	12	2018	Year-2	1	0	Winter
12/19/2018	FP	13A	12	2018	Year-2	1	0	Winter
12/20/2018	FP	1M	12	2018	Year-2	1	0	Winter
12/20/2018	FP	2M	12	2018	Year-2		1	Winter
12/20/2018	FP	3M	12	2018	Year-2	1	0	Winter
12/20/2018	FP	4M	12	2018	Year-2		1	Winter
12/20/2018	FP	5M	12	2018	Year-2		1	Winter
12/20/2018	FP	6M	12	2018	Year-2		1	Winter
12/20/2018	FP	7M	12	2018	Year-2	1	0	Winter
12/20/2018	FP	10M	12	2018	Year-2		1	Winter
12/20/2018	FP	12M	12	2018	Year-2		1	Winter

Date	Species	Site	Month	Year	Survey Year	Presence	Data Gap	Season
12/20/2018	FP	14M	12	2018	Year-2		1	Winter
12/20/2018	FP	8A	12	2018	Year-2		1	Winter
12/20/2018	FP	9A	12	2018	Year-2	1	0	Winter
12/20/2018	FP	11A	12	2018	Year-2	1	0	Winter
12/20/2018	FP	13A	12	2018	Year-2	1	0	Winter
12/21/2018	FP	1M	12	2018	Year-2	1	0	Winter
12/21/2018	FP	2M	12	2018	Year-2		1	Winter
12/21/2018	FP	3M	12	2018	Year-2	1	0	Winter
12/21/2018	FP	4M	12	2018	Year-2		1	Winter
12/21/2018	FP	5M	12	2018	Year-2		1	Winter
12/21/2018	FP	6M	12	2018	Year-2		1	Winter
12/21/2018	FP	7M	12	2018	Year-2	1	0	Winter
12/21/2018	FP	10M	12	2018	Year-2		1	Winter
12/21/2018	FP	12M	12	2018	Year-2		1	Winter
12/21/2018	FP	14M	12	2018	Year-2		1	Winter
12/21/2018	FP	8A	12	2018	Year-2		1	Winter
12/21/2018	FP	9A	12	2018	Year-2	1	0	Winter
12/21/2018	FP	11A	12	2018	Year-2	1	0	Winter
12/21/2018	FP	13A	12	2018	Year-2	1	0	Winter
12/22/2018	FP	1M	12	2018	Year-2	1	0	Winter
12/22/2018	FP	2M	12	2018	Year-2		1	Winter
12/22/2018	FP	3M	12	2018	Year-2	1	0	Winter
12/22/2018	FP	4M	12	2018	Year-2		1	Winter
12/22/2018	FP	5M	12	2018	Year-2		1	Winter
12/22/2018	FP	6M	12	2018	Year-2		1	Winter
12/22/2018	FP	7M	12	2018	Year-2	1	0	Winter
12/22/2018	FP	10M	12	2018	Year-2		1	Winter
12/22/2018	FP	12M	12	2018	Year-2		1	Winter
12/22/2018	FP	14M	12	2018	Year-2		1	Winter
12/22/2018	FP	8A	12	2018	Year-2		1	Winter
12/22/2018	FP	9A	12	2018	Year-2	1	0	Winter
12/22/2018	FP	11A	12	2018	Year-2	1	0	Winter
12/22/2018	FP	13A	12	2018	Year-2	1	0	Winter
12/23/2018	FP	1M	12	2018	Year-2	1	0	Winter
12/23/2018	FP	2M	12	2018	Year-2		1	Winter
12/23/2018	FP	3M	12	2018	Year-2		1	Winter
12/23/2018	FP	4M	12	2018	Year-2		1	Winter
12/23/2018	FP	5M	12	2018	Year-2		1	Winter
12/23/2018	FP	6M	12	2018	Year-2		1	Winter
12/23/2018	FP	7M	12	2018	Year-2	1	0	Winter
12/23/2018	FP	10M	12	2018	Year-2		1	Winter
12/23/2018	FP	12M	12	2018	Year-2		1	Winter
12/23/2018	FP	14M	12	2018	Year-2		1	Winter
12/23/2018	FP	8A	12	2018	Year-2		1	Winter

Date	Species	Site	Month	Year	Survey Year	Presence	Data Gap	Season
12/23/2018	FP	9A	12	2018	Year-2	1	0	Winter
12/23/2018	FP	11A	12	2018	Year-2	1	0	Winter
12/23/2018	FP	13A	12	2018	Year-2	1	0	Winter
12/24/2018	FP	1M	12	2018	Year-2		1	Winter
12/24/2018	FP	2M	12	2018	Year-2		1	Winter
12/24/2018	FP	3M	12	2018	Year-2		1	Winter
12/24/2018	FP	4M	12	2018	Year-2		1	Winter
12/24/2018	FP	5M	12	2018	Year-2		1	Winter
12/24/2018	FP	6M	12	2018	Year-2		1	Winter
12/24/2018	FP	7M	12	2018	Year-2		1	Winter
12/24/2018	FP	10M	12	2018	Year-2		1	Winter
12/24/2018	FP	12M	12	2018	Year-2		1	Winter
12/24/2018	FP	14M	12	2018	Year-2		1	Winter
12/24/2018	FP	8A	12	2018	Year-2		1	Winter
12/24/2018	FP	9A	12	2018	Year-2	1	0	Winter
12/24/2018	FP	11A	12	2018	Year-2	1	0	Winter
12/24/2018	FP	13A	12	2018	Year-2	1	0	Winter
12/25/2018	FP	1M	12	2018	Year-2		1	Winter
12/25/2018	FP	2M	12	2018	Year-2		1	Winter
12/25/2018	FP	3M	12	2018	Year-2		1	Winter
12/25/2018	FP	4M	12	2018	Year-2		1	Winter
12/25/2018	FP	5M	12	2018	Year-2		1	Winter
12/25/2018	FP	6M	12	2018	Year-2		1	Winter
12/25/2018	FP	7M	12	2018	Year-2		1	Winter
12/25/2018	FP	10M	12	2018	Year-2		1	Winter
12/25/2018	FP	12M	12	2018	Year-2		1	Winter
12/25/2018	FP	14M	12	2018	Year-2		1	Winter
12/25/2018	FP	8A	12	2018	Year-2		1	Winter
12/25/2018	FP	9A	12	2018	Year-2	1	0	Winter
12/25/2018	FP	11A	12	2018	Year-2	1	0	Winter
12/25/2018	FP	13A	12	2018	Year-2	1	0	Winter
12/26/2018	FP	1M	12	2018	Year-2		1	Winter
12/26/2018	FP	2M	12	2018	Year-2		1	Winter
12/26/2018	FP	3M	12	2018	Year-2		1	Winter
12/26/2018	FP	4M	12	2018	Year-2		1	Winter
12/26/2018	FP	5M	12	2018	Year-2		1	Winter
12/26/2018	FP	6M	12	2018	Year-2		1	Winter
12/26/2018	FP	7M	12	2018	Year-2		1	Winter
12/26/2018	FP	10M	12	2018	Year-2		1	Winter
12/26/2018	FP	12M	12	2018	Year-2		1	Winter
12/26/2018	FP	14M	12	2018	Year-2		1	Winter
12/26/2018	FP	8A	12	2018	Year-2		1	Winter
12/26/2018	FP	9A	12	2018	Year-2	1	0	Winter
12/26/2018	FP	11A	12	2018	Year-2	1	0	Winter

Date	Species	Site	Month	Year	Survey Year	Presence	Data Gap	Season
12/26/2018	FP	13A	12	2018	Year-2	1	0	Winter
12/27/2018	FP	1M	12	2018	Year-2		1	Winter
12/27/2018	FP	2M	12	2018	Year-2		1	Winter
12/27/2018	FP	3M	12	2018	Year-2		1	Winter
12/27/2018	FP	4M	12	2018	Year-2		1	Winter
12/27/2018	FP	5M	12	2018	Year-2		1	Winter
12/27/2018	FP	6M	12	2018	Year-2		1	Winter
12/27/2018	FP	7M	12	2018	Year-2		1	Winter
12/27/2018	FP	10M	12	2018	Year-2		1	Winter
12/27/2018	FP	12M	12	2018	Year-2		1	Winter
12/27/2018	FP	14M	12	2018	Year-2		1	Winter
12/27/2018	FP	8A	12	2018	Year-2		1	Winter
12/27/2018	FP	9A	12	2018	Year-2	1	0	Winter
12/27/2018	FP	11A	12	2018	Year-2	1	0	Winter
12/27/2018	FP	13A	12	2018	Year-2	1	0	Winter
12/28/2018	FP	1M	12	2018	Year-2		1	Winter
12/28/2018	FP	2M	12	2018	Year-2		1	Winter
12/28/2018	FP	3M	12	2018	Year-2		1	Winter
12/28/2018	FP	4M	12	2018	Year-2		1	Winter
12/28/2018	FP	5M	12	2018	Year-2		1	Winter
12/28/2018	FP	6M	12	2018	Year-2		1	Winter
12/28/2018	FP	7M	12	2018	Year-2		1	Winter
12/28/2018	FP	10M	12	2018	Year-2		1	Winter
12/28/2018	FP	12M	12	2018	Year-2		1	Winter
12/28/2018	FP	14M	12	2018	Year-2		1	Winter
12/28/2018	FP	8A	12	2018	Year-2		1	Winter
12/28/2018	FP	9A	12	2018	Year-2	0	0	Winter
12/28/2018	FP	11A	12	2018	Year-2	1	0	Winter
12/28/2018	FP	13A	12	2018	Year-2	1	0	Winter
12/29/2018	FP	1M	12	2018	Year-2		1	Winter
12/29/2018	FP	2M	12	2018	Year-2		1	Winter
12/29/2018	FP	3M	12	2018	Year-2		1	Winter
12/29/2018	FP	4M	12	2018	Year-2		1	Winter
12/29/2018	FP	5M	12	2018	Year-2		1	Winter
12/29/2018	FP	6M	12	2018	Year-2		1	Winter
12/29/2018	FP	7M	12	2018	Year-2		1	Winter
12/29/2018	FP	10M	12	2018	Year-2		1	Winter
12/29/2018	FP	12M	12	2018	Year-2		1	Winter
12/29/2018	FP	14M	12	2018	Year-2		1	Winter
12/29/2018	FP	8A	12	2018	Year-2		1	Winter
12/29/2018	FP	9A	12	2018	Year-2	1	0	Winter
12/29/2018	FP	11A	12	2018	Year-2	1	0	Winter
12/29/2018	FP	13A	12	2018	Year-2	1	0	Winter
12/30/2018	FP	1M	12	2018	Year-2		1	Winter

Date	Species	Site	Month	Year	Survey Year	Presence	Data Gap	Season
12/30/2018	FP	2M	12	2018	Year-2		1	Winter
12/30/2018	FP	3M	12	2018	Year-2		1	Winter
12/30/2018	FP	4M	12	2018	Year-2		1	Winter
12/30/2018	FP	5M	12	2018	Year-2		1	Winter
12/30/2018	FP	6M	12	2018	Year-2		1	Winter
12/30/2018	FP	7M	12	2018	Year-2		1	Winter
12/30/2018	FP	10M	12	2018	Year-2		1	Winter
12/30/2018	FP	12M	12	2018	Year-2		1	Winter
12/30/2018	FP	14M	12	2018	Year-2		1	Winter
12/30/2018	FP	8A	12	2018	Year-2		1	Winter
12/30/2018	FP	9A	12	2018	Year-2	1	0	Winter
12/30/2018	FP	11A	12	2018	Year-2	1	0	Winter
12/30/2018	FP	13A	12	2018	Year-2	1	0	Winter
12/31/2018	FP	1M	12	2018	Year-2		1	Winter
12/31/2018	FP	2M	12	2018	Year-2		1	Winter
12/31/2018	FP	3M	12	2018	Year-2		1	Winter
12/31/2018	FP	4M	12	2018	Year-2		1	Winter
12/31/2018	FP	5M	12	2018	Year-2		1	Winter
12/31/2018	FP	6M	12	2018	Year-2		1	Winter
12/31/2018	FP	7M	12	2018	Year-2		1	Winter
12/31/2018	FP	10M	12	2018	Year-2		1	Winter
12/31/2018	FP	12M	12	2018	Year-2		1	Winter
12/31/2018	FP	14M	12	2018	Year-2		1	Winter
12/31/2018	FP	8A	12	2018	Year-2		1	Winter
12/31/2018	FP	9A	12	2018	Year-2	1	0	Winter
12/31/2018	FP	11A	12	2018	Year-2	1	0	Winter
12/31/2018	FP	13A	12	2018	Year-2	1	0	Winter
1/1/2019	FP	1M	1	2019	Year-2		1	Winter
1/1/2019	FP	2M	1	2019	Year-2		1	Winter
1/1/2019	FP	3M	1	2019	Year-2		1	Winter
1/1/2019	FP	4M	1	2019	Year-2		1	Winter
1/1/2019	FP	5M	1	2019	Year-2		1	Winter
1/1/2019	FP	6M	1	2019	Year-2		1	Winter
1/1/2019	FP	7M	1	2019	Year-2		1	Winter
1/1/2019	FP	10M	1	2019	Year-2		1	Winter
1/1/2019	FP	12M	1	2019	Year-2		1	Winter
1/1/2019	FP	14M	1	2019	Year-2		1	Winter
1/1/2019	FP	8A	1	2019	Year-2		1	Winter
1/1/2019	FP	9A	1	2019	Year-2	1	0	Winter
1/1/2019	FP	11A	1	2019	Year-2	1	0	Winter
1/1/2019	FP	13A	1	2019	Year-2	1	0	Winter
1/2/2019	FP	1M	1	2019	Year-2		1	Winter
1/2/2019	FP	2M	1	2019	Year-2		1	Winter
1/2/2019	FP	3M	1	2019	Year-2		1	Winter

Date	Species	Site	Month	Year	Survey Year	Presence	Data Gap	Season
1/2/2019	FP	4M	1	2019	Year-2		1	Winter
1/2/2019	FP	5M	1	2019	Year-2		1	Winter
1/2/2019	FP	6M	1	2019	Year-2		1	Winter
1/2/2019	FP	7M	1	2019	Year-2		1	Winter
1/2/2019	FP	10M	1	2019	Year-2		1	Winter
1/2/2019	FP	12M	1	2019	Year-2		1	Winter
1/2/2019	FP	14M	1	2019	Year-2		1	Winter
1/2/2019	FP	8A	1	2019	Year-2		1	Winter
1/2/2019	FP	9A	1	2019	Year-2	1	0	Winter
1/2/2019	FP	11A	1	2019	Year-2	1	0	Winter
1/2/2019	FP	13A	1	2019	Year-2	1	0	Winter
1/3/2019	FP	1M	1	2019	Year-2		1	Winter
1/3/2019	FP	2M	1	2019	Year-2		1	Winter
1/3/2019	FP	3M	1	2019	Year-2		1	Winter
1/3/2019	FP	4M	1	2019	Year-2		1	Winter
1/3/2019	FP	5M	1	2019	Year-2		1	Winter
1/3/2019	FP	6M	1	2019	Year-2		1	Winter
1/3/2019	FP	7M	1	2019	Year-2		1	Winter
1/3/2019	FP	10M	1	2019	Year-2		1	Winter
1/3/2019	FP	12M	1	2019	Year-2		1	Winter
1/3/2019	FP	14M	1	2019	Year-2		1	Winter
1/3/2019	FP	8A	1	2019	Year-2		1	Winter
1/3/2019	FP	9A	1	2019	Year-2	0	0	Winter
1/3/2019	FP	11A	1	2019	Year-2	0	0	Winter
1/3/2019	FP	13A	1	2019	Year-2	1	0	Winter
1/4/2019	FP	1M	1	2019	Year-2		1	Winter
1/4/2019	FP	2M	1	2019	Year-2		1	Winter
1/4/2019	FP	3M	1	2019	Year-2		1	Winter
1/4/2019	FP	4M	1	2019	Year-2		1	Winter
1/4/2019	FP	5M	1	2019	Year-2		1	Winter
1/4/2019	FP	6M	1	2019	Year-2		1	Winter
1/4/2019	FP	7M	1	2019	Year-2		1	Winter
1/4/2019	FP	10M	1	2019	Year-2		1	Winter
1/4/2019	FP	12M	1	2019	Year-2		1	Winter
1/4/2019	FP	14M	1	2019	Year-2		1	Winter
1/4/2019	FP	8A	1	2019	Year-2		1	Winter
1/4/2019	FP	9A	1	2019	Year-2	1	0	Winter
1/4/2019	FP	11A	1	2019	Year-2	1	0	Winter
1/4/2019	FP	13A	1	2019	Year-2	1	0	Winter
1/5/2019	FP	1M	1	2019	Year-2		1	Winter
1/5/2019	FP	2M	1	2019	Year-2		1	Winter
1/5/2019	FP	3M	1	2019	Year-2		1	Winter
1/5/2019	FP	4M	1	2019	Year-2		1	Winter
1/5/2019	FP	5M	1	2019	Year-2		1	Winter

Date	Species	Site	Month	Year	Survey Year	Presence	Data Gap	Season
1/5/2019	FP	6M	1	2019	Year-2		1	Winter
1/5/2019	FP	7M	1	2019	Year-2		1	Winter
1/5/2019	FP	10M	1	2019	Year-2		1	Winter
1/5/2019	FP	12M	1	2019	Year-2		1	Winter
1/5/2019	FP	14M	1	2019	Year-2		1	Winter
1/5/2019	FP	8A	1	2019	Year-2		1	Winter
1/5/2019	FP	9A	1	2019	Year-2	1	0	Winter
1/5/2019	FP	11A	1	2019	Year-2	1	0	Winter
1/5/2019	FP	13A	1	2019	Year-2	1	0	Winter
1/6/2019	FP	1M	1	2019	Year-2		1	Winter
1/6/2019	FP	2M	1	2019	Year-2		1	Winter
1/6/2019	FP	3M	1	2019	Year-2		1	Winter
1/6/2019	FP	4M	1	2019	Year-2		1	Winter
1/6/2019	FP	5M	1	2019	Year-2		1	Winter
1/6/2019	FP	6M	1	2019	Year-2		1	Winter
1/6/2019	FP	7M	1	2019	Year-2		1	Winter
1/6/2019	FP	10M	1	2019	Year-2		1	Winter
1/6/2019	FP	12M	1	2019	Year-2		1	Winter
1/6/2019	FP	14M	1	2019	Year-2		1	Winter
1/6/2019	FP	8A	1	2019	Year-2		1	Winter
1/6/2019	FP	9A	1	2019	Year-2	1	0	Winter
1/6/2019	FP	11A	1	2019	Year-2	1	0	Winter
1/6/2019	FP	13A	1	2019	Year-2	1	0	Winter
1/7/2019	FP	1M	1	2019	Year-2		1	Winter
1/7/2019	FP	2M	1	2019	Year-2		1	Winter
1/7/2019	FP	3M	1	2019	Year-2		1	Winter
1/7/2019	FP	4M	1	2019	Year-2		1	Winter
1/7/2019	FP	5M	1	2019	Year-2		1	Winter
1/7/2019	FP	6M	1	2019	Year-2		1	Winter
1/7/2019	FP	7M	1	2019	Year-2		1	Winter
1/7/2019	FP	10M	1	2019	Year-2		1	Winter
1/7/2019	FP	12M	1	2019	Year-2		1	Winter
1/7/2019	FP	14M	1	2019	Year-2		1	Winter
1/7/2019	FP	8A	1	2019	Year-2		1	Winter
1/7/2019	FP	9A	1	2019	Year-2	1	0	Winter
1/7/2019	FP	11A	1	2019	Year-2	1	0	Winter
1/7/2019	FP	13A	1	2019	Year-2	1	0	Winter
1/8/2019	FP	1M	1	2019	Year-2		1	Winter
1/8/2019	FP	2M	1	2019	Year-2		1	Winter
1/8/2019	FP	3M	1	2019	Year-2		1	Winter
1/8/2019	FP	4M	1	2019	Year-2		1	Winter
1/8/2019	FP	5M	1	2019	Year-2		1	Winter
1/8/2019	FP	6M	1	2019	Year-2		1	Winter
1/8/2019	FP	7M	1	2019	Year-2		1	Winter

Date	Species	Site	Month	Year	Survey Year	Presence	Data Gap	Season
1/8/2019	FP	10M	1	2019	Year-2		1	Winter
1/8/2019	FP	12M	1	2019	Year-2		1	Winter
1/8/2019	FP	14M	1	2019	Year-2		1	Winter
1/8/2019	FP	8A	1	2019	Year-2		1	Winter
1/8/2019	FP	9A	1	2019	Year-2	1	0	Winter
1/8/2019	FP	11A	1	2019	Year-2	1	0	Winter
1/8/2019	FP	13A	1	2019	Year-2	1	0	Winter
1/9/2019	FP	1M	1	2019	Year-2		1	Winter
1/9/2019	FP	2M	1	2019	Year-2		1	Winter
1/9/2019	FP	3M	1	2019	Year-2		1	Winter
1/9/2019	FP	4M	1	2019	Year-2		1	Winter
1/9/2019	FP	5M	1	2019	Year-2		1	Winter
1/9/2019	FP	6M	1	2019	Year-2		1	Winter
1/9/2019	FP	7M	1	2019	Year-2		1	Winter
1/9/2019	FP	10M	1	2019	Year-2		1	Winter
1/9/2019	FP	12M	1	2019	Year-2		1	Winter
1/9/2019	FP	14M	1	2019	Year-2		1	Winter
1/9/2019	FP	8A	1	2019	Year-2		1	Winter
1/9/2019	FP	9A	1	2019	Year-2	1	0	Winter
1/9/2019	FP	11A	1	2019	Year-2	1	0	Winter
1/9/2019	FP	13A	1	2019	Year-2	1	0	Winter
1/10/2019	FP	1M	1	2019	Year-2		1	Winter
1/10/2019	FP	2M	1	2019	Year-2		1	Winter
1/10/2019	FP	3M	1	2019	Year-2		1	Winter
1/10/2019	FP	4M	1	2019	Year-2		1	Winter
1/10/2019	FP	5M	1	2019	Year-2		1	Winter
1/10/2019	FP	6M	1	2019	Year-2		1	Winter
1/10/2019	FP	7M	1	2019	Year-2		1	Winter
1/10/2019	FP	10M	1	2019	Year-2		1	Winter
1/10/2019	FP	12M	1	2019	Year-2		1	Winter
1/10/2019	FP	14M	1	2019	Year-2		1	Winter
1/10/2019	FP	8A	1	2019	Year-2		1	Winter
1/10/2019	FP	9A	1	2019	Year-2	1	0	Winter
1/10/2019	FP	11A	1	2019	Year-2	1	0	Winter
1/10/2019	FP	13A	1	2019	Year-2	1	0	Winter
1/11/2019	FP	1M	1	2019	Year-2		1	Winter
1/11/2019	FP	2M	1	2019	Year-2		1	Winter
1/11/2019	FP	3M	1	2019	Year-2		1	Winter
1/11/2019	FP	4M	1	2019	Year-2		1	Winter
1/11/2019	FP	5M	1	2019	Year-2		1	Winter
1/11/2019	FP	6M	1	2019	Year-2		1	Winter
1/11/2019	FP	7M	1	2019	Year-2		1	Winter
1/11/2019	FP	10M	1	2019	Year-2		1	Winter
1/11/2019	FP	12M	1	2019	Year-2		1	Winter

Date	Species	Site	Month	Year	Survey Year	Presence	Data Gap	Season
1/11/2019	FP	14M	1	2019	Year-2		1	Winter
1/11/2019	FP	8A	1	2019	Year-2		1	Winter
1/11/2019	FP	9A	1	2019	Year-2	1	0	Winter
1/11/2019	FP	11A	1	2019	Year-2	0	0	Winter
1/11/2019	FP	13A	1	2019	Year-2	1	0	Winter
1/12/2019	FP	1M	1	2019	Year-2		1	Winter
1/12/2019	FP	2M	1	2019	Year-2		1	Winter
1/12/2019	FP	3M	1	2019	Year-2		1	Winter
1/12/2019	FP	4M	1	2019	Year-2		1	Winter
1/12/2019	FP	5M	1	2019	Year-2		1	Winter
1/12/2019	FP	6M	1	2019	Year-2		1	Winter
1/12/2019	FP	7M	1	2019	Year-2		1	Winter
1/12/2019	FP	10M	1	2019	Year-2		1	Winter
1/12/2019	FP	12M	1	2019	Year-2		1	Winter
1/12/2019	FP	14M	1	2019	Year-2		1	Winter
1/12/2019	FP	8A	1	2019	Year-2		1	Winter
1/12/2019	FP	9A	1	2019	Year-2	1	0	Winter
1/12/2019	FP	11A	1	2019	Year-2	0	0	Winter
1/12/2019	FP	13A	1	2019	Year-2	1	0	Winter
1/13/2019	FP	1M	1	2019	Year-2	0	0	Winter
1/13/2019	FP	2M	1	2019	Year-2		1	Winter
1/13/2019	FP	3M	1	2019	Year-2		1	Winter
1/13/2019	FP	4M	1	2019	Year-2		1	Winter
1/13/2019	FP	5M	1	2019	Year-2		1	Winter
1/13/2019	FP	6M	1	2019	Year-2		1	Winter
1/13/2019	FP	7M	1	2019	Year-2		1	Winter
1/13/2019	FP	10M	1	2019	Year-2		1	Winter
1/13/2019	FP	12M	1	2019	Year-2		1	Winter
1/13/2019	FP	14M	1	2019	Year-2		1	Winter
1/13/2019	FP	8A	1	2019	Year-2		1	Winter
1/13/2019	FP	9A	1	2019	Year-2	1	0	Winter
1/13/2019	FP	11A	1	2019	Year-2	1	0	Winter
1/13/2019	FP	13A	1	2019	Year-2	1	0	Winter
1/14/2019	FP	1M	1	2019	Year-2	1	0	Winter
1/14/2019	FP	2M	1	2019	Year-2	1	0	Winter
1/14/2019	FP	3M	1	2019	Year-2	1	0	Winter
1/14/2019	FP	4M	1	2019	Year-2	0	0	Winter
1/14/2019	FP	5M	1	2019	Year-2	1	0	Winter
1/14/2019	FP	6M	1	2019	Year-2	1	0	Winter
1/14/2019	FP	7M	1	2019	Year-2	0	0	Winter
1/14/2019	FP	10M	1	2019	Year-2		1	Winter
1/14/2019	FP	12M	1	2019	Year-2		1	Winter
1/14/2019	FP	14M	1	2019	Year-2		1	Winter
1/14/2019	FP	8A	1	2019	Year-2		1	Winter

Date	Species	Site	Month	Year	Survey Year	Presence	Data Gap	Season
1/14/2019	FP	9A	1	2019	Year-2	1	0	Winter
1/14/2019	FP	11A	1	2019	Year-2	1	0	Winter
1/14/2019	FP	13A	1	2019	Year-2	1	0	Winter
1/15/2019	FP	1M	1	2019	Year-2	1	0	Winter
1/15/2019	FP	2M	1	2019	Year-2	1	0	Winter
1/15/2019	FP	3M	1	2019	Year-2	1	0	Winter
1/15/2019	FP	4M	1	2019	Year-2	1	0	Winter
1/15/2019	FP	5M	1	2019	Year-2	1	0	Winter
1/15/2019	FP	6M	1	2019	Year-2	1	0	Winter
1/15/2019	FP	7M	1	2019	Year-2	0	0	Winter
1/15/2019	FP	10M	1	2019	Year-2		1	Winter
1/15/2019	FP	12M	1	2019	Year-2		1	Winter
1/15/2019	FP	14M	1	2019	Year-2		1	Winter
1/15/2019	FP	8A	1	2019	Year-2		1	Winter
1/15/2019	FP	9A	1	2019	Year-2	0	0	Winter
1/15/2019	FP	11A	1	2019	Year-2	1	0	Winter
1/15/2019	FP	13A	1	2019	Year-2	1	0	Winter
1/16/2019	FP	1M	1	2019	Year-2	1	0	Winter
1/16/2019	FP	2M	1	2019	Year-2	1	0	Winter
1/16/2019	FP	3M	1	2019	Year-2	0	0	Winter
1/16/2019	FP	4M	1	2019	Year-2	1	0	Winter
1/16/2019	FP	5M	1	2019	Year-2	1	0	Winter
1/16/2019	FP	6M	1	2019	Year-2	0	0	Winter
1/16/2019	FP	7M	1	2019	Year-2	1	0	Winter
1/16/2019	FP	10M	1	2019	Year-2		1	Winter
1/16/2019	FP	12M	1	2019	Year-2		1	Winter
1/16/2019	FP	14M	1	2019	Year-2		1	Winter
1/16/2019	FP	8A	1	2019	Year-2		1	Winter
1/16/2019	FP	9A	1	2019	Year-2	1	0	Winter
1/16/2019	FP	11A	1	2019	Year-2	1	0	Winter
1/16/2019	FP	13A	1	2019	Year-2	1	0	Winter
1/17/2019	FP	1M	1	2019	Year-2	1	0	Winter
1/17/2019	FP	2M	1	2019	Year-2	1	0	Winter
1/17/2019	FP	3M	1	2019	Year-2	1	0	Winter
1/17/2019	FP	4M	1	2019	Year-2	1	0	Winter
1/17/2019	FP	5M	1	2019	Year-2	0	0	Winter
1/17/2019	FP	6M	1	2019	Year-2	0	0	Winter
1/17/2019	FP	7M	1	2019	Year-2	0	0	Winter
1/17/2019	FP	10M	1	2019	Year-2		1	Winter
1/17/2019	FP	12M	1	2019	Year-2		1	Winter
1/17/2019	FP	14M	1	2019	Year-2		1	Winter
1/17/2019	FP	8A	1	2019	Year-2		1	Winter
1/17/2019	FP	9A	1	2019	Year-2	0	0	Winter
1/17/2019	FP	11A	1	2019	Year-2	1	0	Winter

Date	Species	Site	Month	Year	Survey Year	Presence	Data Gap	Season
1/17/2019	FP	13A	1	2019	Year-2	1	0	Winter
1/18/2019	FP	1M	1	2019	Year-2	1	0	Winter
1/18/2019	FP	2M	1	2019	Year-2	1	0	Winter
1/18/2019	FP	3M	1	2019	Year-2	1	0	Winter
1/18/2019	FP	4M	1	2019	Year-2	0	0	Winter
1/18/2019	FP	5M	1	2019	Year-2	0	0	Winter
1/18/2019	FP	6M	1	2019	Year-2	0	0	Winter
1/18/2019	FP	7M	1	2019	Year-2	0	0	Winter
1/18/2019	FP	10M	1	2019	Year-2		1	Winter
1/18/2019	FP	12M	1	2019	Year-2		1	Winter
1/18/2019	FP	14M	1	2019	Year-2	0	0	Winter
1/18/2019	FP	8A	1	2019	Year-2		1	Winter
1/18/2019	FP	9A	1	2019	Year-2	0	0	Winter
1/18/2019	FP	11A	1	2019	Year-2	1	0	Winter
1/18/2019	FP	13A	1	2019	Year-2	0	0	Winter
1/19/2019	FP	1M	1	2019	Year-2	1	0	Winter
1/19/2019	FP	2M	1	2019	Year-2	1	0	Winter
1/19/2019	FP	3M	1	2019	Year-2	1	0	Winter
1/19/2019	FP	4M	1	2019	Year-2	1	0	Winter
1/19/2019	FP	5M	1	2019	Year-2	1	0	Winter
1/19/2019	FP	6M	1	2019	Year-2	0	0	Winter
1/19/2019	FP	7M	1	2019	Year-2	0	0	Winter
1/19/2019	FP	10M	1	2019	Year-2	0	0	Winter
1/19/2019	FP	12M	1	2019	Year-2	0	0	Winter
1/19/2019	FP	14M	1	2019	Year-2	0	0	Winter
1/19/2019	FP	8A	1	2019	Year-2		1	Winter
1/19/2019	FP	9A	1	2019	Year-2	1	0	Winter
1/19/2019	FP	11A	1	2019	Year-2	1	0	Winter
1/19/2019	FP	13A	1	2019	Year-2	1	0	Winter
1/20/2019	FP	1M	1	2019	Year-2	1	0	Winter
1/20/2019	FP	2M	1	2019	Year-2	1	0	Winter
1/20/2019	FP	3M	1	2019	Year-2	1	0	Winter
1/20/2019	FP	4M	1	2019	Year-2	1	0	Winter
1/20/2019	FP	5M	1	2019	Year-2	1	0	Winter
1/20/2019	FP	6M	1	2019	Year-2	1	0	Winter
1/20/2019	FP	7M	1	2019	Year-2	1	0	Winter
1/20/2019	FP	10M	1	2019	Year-2	1	0	Winter
1/20/2019	FP	12M	1	2019	Year-2	1	0	Winter
1/20/2019	FP	14M	1	2019	Year-2	1	0	Winter
1/20/2019	FP	8A	1	2019	Year-2		1	Winter
1/20/2019	FP	9A	1	2019	Year-2	1	0	Winter
1/20/2019	FP	11A	1	2019	Year-2	1	0	Winter
1/20/2019	FP	13A	1	2019	Year-2	1	0	Winter
1/21/2019	FP	1M	1	2019	Year-2	0	0	Winter

Date	Species	Site	Month	Year	Survey Year	Presence	Data Gap	Season
1/21/2019	FP	2M	1	2019	Year-2	0	0	Winter
1/21/2019	FP	3M	1	2019	Year-2	1	0	Winter
1/21/2019	FP	4M	1	2019	Year-2	1	0	Winter
1/21/2019	FP	5M	1	2019	Year-2	1	0	Winter
1/21/2019	FP	6M	1	2019	Year-2	1	0	Winter
1/21/2019	FP	7M	1	2019	Year-2	1	0	Winter
1/21/2019	FP	10M	1	2019	Year-2	1	0	Winter
1/21/2019	FP	12M	1	2019	Year-2	1	0	Winter
1/21/2019	FP	14M	1	2019	Year-2	1	0	Winter
1/21/2019	FP	8A	1	2019	Year-2		1	Winter
1/21/2019	FP	9A	1	2019	Year-2	1	0	Winter
1/21/2019	FP	11A	1	2019	Year-2	1	0	Winter
1/21/2019	FP	13A	1	2019	Year-2	1	0	Winter
1/22/2019	FP	1M	1	2019	Year-2	1	0	Winter
1/22/2019	FP	2M	1	2019	Year-2	1	0	Winter
1/22/2019	FP	3M	1	2019	Year-2	1	0	Winter
1/22/2019	FP	4M	1	2019	Year-2	1	0	Winter
1/22/2019	FP	5M	1	2019	Year-2	0	0	Winter
1/22/2019	FP	6M	1	2019	Year-2	0	0	Winter
1/22/2019	FP	7M	1	2019	Year-2	0	0	Winter
1/22/2019	FP	10M	1	2019	Year-2	0	0	Winter
1/22/2019	FP	12M	1	2019	Year-2	0	0	Winter
1/22/2019	FP	14M	1	2019	Year-2	0	0	Winter
1/22/2019	FP	8A	1	2019	Year-2		1	Winter
1/22/2019	FP	9A	1	2019	Year-2	1	0	Winter
1/22/2019	FP	11A	1	2019	Year-2	1	0	Winter
1/22/2019	FP	13A	1	2019	Year-2	1	0	Winter
1/23/2019	FP	1M	1	2019	Year-2	1	0	Winter
1/23/2019	FP	2M	1	2019	Year-2	1	0	Winter
1/23/2019	FP	3M	1	2019	Year-2	1	0	Winter
1/23/2019	FP	4M	1	2019	Year-2	1	0	Winter
1/23/2019	FP	5M	1	2019	Year-2	1	0	Winter
1/23/2019	FP	6M	1	2019	Year-2	1	0	Winter
1/23/2019	FP	7M	1	2019	Year-2	1	0	Winter
1/23/2019	FP	10M	1	2019	Year-2	1	0	Winter
1/23/2019	FP	12M	1	2019	Year-2	1	0	Winter
1/23/2019	FP	14M	1	2019	Year-2	1	0	Winter
1/23/2019	FP	8A	1	2019	Year-2		1	Winter
1/23/2019	FP	9A	1	2019	Year-2	1	0	Winter
1/23/2019	FP	11A	1	2019	Year-2	1	0	Winter
1/23/2019	FP	13A	1	2019	Year-2	1	0	Winter
1/24/2019	FP	1M	1	2019	Year-2	1	0	Winter
1/24/2019	FP	2M	1	2019	Year-2	1	0	Winter
1/24/2019	FP	3M	1	2019	Year-2	1	0	Winter

Date	Species	Site	Month	Year	Survey Year	Presence	Data Gap	Season
1/24/2019	FP	4M	1	2019	Year-2	1	0	Winter
1/24/2019	FP	5M	1	2019	Year-2	1	0	Winter
1/24/2019	FP	6M	1	2019	Year-2	1	0	Winter
1/24/2019	FP	7M	1	2019	Year-2	0	0	Winter
1/24/2019	FP	10M	1	2019	Year-2	1	0	Winter
1/24/2019	FP	12M	1	2019	Year-2	1	0	Winter
1/24/2019	FP	14M	1	2019	Year-2	1	0	Winter
1/24/2019	FP	8A	1	2019	Year-2		1	Winter
1/24/2019	FP	9A	1	2019	Year-2	1	0	Winter
1/24/2019	FP	11A	1	2019	Year-2	1	0	Winter
1/24/2019	FP	13A	1	2019	Year-2	1	0	Winter
1/25/2019	FP	1M	1	2019	Year-2	1	0	Winter
1/25/2019	FP	2M	1	2019	Year-2	1	0	Winter
1/25/2019	FP	3M	1	2019	Year-2	1	0	Winter
1/25/2019	FP	4M	1	2019	Year-2	1	0	Winter
1/25/2019	FP	5M	1	2019	Year-2	1	0	Winter
1/25/2019	FP	6M	1	2019	Year-2	1	0	Winter
1/25/2019	FP	7M	1	2019	Year-2	1	0	Winter
1/25/2019	FP	10M	1	2019	Year-2	1	0	Winter
1/25/2019	FP	12M	1	2019	Year-2	1	0	Winter
1/25/2019	FP	14M	1	2019	Year-2	1	0	Winter
1/25/2019	FP	8A	1	2019	Year-2		1	Winter
1/25/2019	FP	9A	1	2019	Year-2	1	0	Winter
1/25/2019	FP	11A	1	2019	Year-2	1	0	Winter
1/25/2019	FP	13A	1	2019	Year-2	1	0	Winter
1/26/2019	FP	1M	1	2019	Year-2	1	0	Winter
1/26/2019	FP	2M	1	2019	Year-2	1	0	Winter
1/26/2019	FP	3M	1	2019	Year-2	1	0	Winter
1/26/2019	FP	4M	1	2019	Year-2	1	0	Winter
1/26/2019	FP	5M	1	2019	Year-2	1	0	Winter
1/26/2019	FP	6M	1	2019	Year-2	1	0	Winter
1/26/2019	FP	7M	1	2019	Year-2	1	0	Winter
1/26/2019	FP	10M	1	2019	Year-2	1	0	Winter
1/26/2019	FP	12M	1	2019	Year-2	1	0	Winter
1/26/2019	FP	14M	1	2019	Year-2	1	0	Winter
1/26/2019	FP	8A	1	2019	Year-2		1	Winter
1/26/2019	FP	9A	1	2019	Year-2	1	0	Winter
1/26/2019	FP	11A	1	2019	Year-2	1	0	Winter
1/26/2019	FP	13A	1	2019	Year-2	1	0	Winter
1/27/2019	FP	1M	1	2019	Year-2	1	0	Winter
1/27/2019	FP	2M	1	2019	Year-2	1	0	Winter
1/27/2019	FP	3M	1	2019	Year-2	1	0	Winter
1/27/2019	FP	4M	1	2019	Year-2	0	0	Winter
1/27/2019	FP	5M	1	2019	Year-2	1	0	Winter

Date	Species	Site	Month	Year	Survey Year	Presence	Data Gap	Season
1/27/2019	FP	6M	1	2019	Year-2	1	0	Winter
1/27/2019	FP	7M	1	2019	Year-2	1	0	Winter
1/27/2019	FP	10M	1	2019	Year-2	1	0	Winter
1/27/2019	FP	12M	1	2019	Year-2	1	0	Winter
1/27/2019	FP	14M	1	2019	Year-2	1	0	Winter
1/27/2019	FP	8A	1	2019	Year-2		1	Winter
1/27/2019	FP	9A	1	2019	Year-2	1	0	Winter
1/27/2019	FP	11A	1	2019	Year-2	1	0	Winter
1/27/2019	FP	13A	1	2019	Year-2	1	0	Winter
1/28/2019	FP	1M	1	2019	Year-2	1	0	Winter
1/28/2019	FP	2M	1	2019	Year-2	1	0	Winter
1/28/2019	FP	3M	1	2019	Year-2	1	0	Winter
1/28/2019	FP	4M	1	2019	Year-2	1	0	Winter
1/28/2019	FP	5M	1	2019	Year-2	1	0	Winter
1/28/2019	FP	6M	1	2019	Year-2	1	0	Winter
1/28/2019	FP	7M	1	2019	Year-2	1	0	Winter
1/28/2019	FP	10M	1	2019	Year-2	1	0	Winter
1/28/2019	FP	12M	1	2019	Year-2	1	0	Winter
1/28/2019	FP	14M	1	2019	Year-2	1	0	Winter
1/28/2019	FP	8A	1	2019	Year-2		1	Winter
1/28/2019	FP	9A	1	2019	Year-2	1	0	Winter
1/28/2019	FP	11A	1	2019	Year-2	1	0	Winter
1/28/2019	FP	13A	1	2019	Year-2	1	0	Winter
1/29/2019	FP	1M	1	2019	Year-2	0	0	Winter
1/29/2019	FP	2M	1	2019	Year-2	1	0	Winter
1/29/2019	FP	3M	1	2019	Year-2	1	0	Winter
1/29/2019	FP	4M	1	2019	Year-2	1	0	Winter
1/29/2019	FP	5M	1	2019	Year-2	1	0	Winter
1/29/2019	FP	6M	1	2019	Year-2	1	0	Winter
1/29/2019	FP	7M	1	2019	Year-2	1	0	Winter
1/29/2019	FP	10M	1	2019	Year-2	1	0	Winter
1/29/2019	FP	12M	1	2019	Year-2	1	0	Winter
1/29/2019	FP	14M	1	2019	Year-2	1	0	Winter
1/29/2019	FP	8A	1	2019	Year-2		1	Winter
1/29/2019	FP	9A	1	2019	Year-2	1	0	Winter
1/29/2019	FP	11A	1	2019	Year-2	1	0	Winter
1/29/2019	FP	13A	1	2019	Year-2	1	0	Winter
1/30/2019	FP	1M	1	2019	Year-2	1	0	Winter
1/30/2019	FP	2M	1	2019	Year-2	1	0	Winter
1/30/2019	FP	3M	1	2019	Year-2	1	0	Winter
1/30/2019	FP	4M	1	2019	Year-2	1	0	Winter
1/30/2019	FP	5M	1	2019	Year-2	1	0	Winter
1/30/2019	FP	6M	1	2019	Year-2	1	0	Winter
1/30/2019	FP	7M	1	2019	Year-2	1	0	Winter

Date	Species	Site	Month	Year	Survey Year	Presence	Data Gap	Season
1/30/2019	FP	10M	1	2019	Year-2	1	0	Winter
1/30/2019	FP	12M	1	2019	Year-2	1	0	Winter
1/30/2019	FP	14M	1	2019	Year-2	1	0	Winter
1/30/2019	FP	8A	1	2019	Year-2		1	Winter
1/30/2019	FP	9A	1	2019	Year-2	1	0	Winter
1/30/2019	FP	11A	1	2019	Year-2	1	0	Winter
1/30/2019	FP	13A	1	2019	Year-2	1	0	Winter
1/31/2019	FP	1M	1	2019	Year-2	1	0	Winter
1/31/2019	FP	2M	1	2019	Year-2	1	0	Winter
1/31/2019	FP	3M	1	2019	Year-2	1	0	Winter
1/31/2019	FP	4M	1	2019	Year-2	1	0	Winter
1/31/2019	FP	5M	1	2019	Year-2	1	0	Winter
1/31/2019	FP	6M	1	2019	Year-2	1	0	Winter
1/31/2019	FP	7M	1	2019	Year-2	1	0	Winter
1/31/2019	FP	10M	1	2019	Year-2	1	0	Winter
1/31/2019	FP	12M	1	2019	Year-2	1	0	Winter
1/31/2019	FP	14M	1	2019	Year-2	1	0	Winter
1/31/2019	FP	8A	1	2019	Year-2		1	Winter
1/31/2019	FP	9A	1	2019	Year-2	1	0	Winter
1/31/2019	FP	11A	1	2019	Year-2	1	0	Winter
1/31/2019	FP	13A	1	2019	Year-2	1	0	Winter
2/1/2019	FP	1M	2	2019	Year-2	1	0	Winter
2/1/2019	FP	2M	2	2019	Year-2	1	0	Winter
2/1/2019	FP	3M	2	2019	Year-2	1	0	Winter
2/1/2019	FP	4M	2	2019	Year-2	1	0	Winter
2/1/2019	FP	5M	2	2019	Year-2	1	0	Winter
2/1/2019	FP	6M	2	2019	Year-2	1	0	Winter
2/1/2019	FP	7M	2	2019	Year-2	1	0	Winter
2/1/2019	FP	10M	2	2019	Year-2	1	0	Winter
2/1/2019	FP	12M	2	2019	Year-2	1	0	Winter
2/1/2019	FP	14M	2	2019	Year-2	1	0	Winter
2/1/2019	FP	8A	2	2019	Year-2		1	Winter
2/1/2019	FP	9A	2	2019	Year-2	1	0	Winter
2/1/2019	FP	11A	2	2019	Year-2	1	0	Winter
2/1/2019	FP	13A	2	2019	Year-2	1	0	Winter
2/2/2019	FP	1M	2	2019	Year-2	1	0	Winter
2/2/2019	FP	2M	2	2019	Year-2	1	0	Winter
2/2/2019	FP	3M	2	2019	Year-2	1	0	Winter
2/2/2019	FP	4M	2	2019	Year-2	1	0	Winter
2/2/2019	FP	5M	2	2019	Year-2	1	0	Winter
2/2/2019	FP	6M	2	2019	Year-2	1	0	Winter
2/2/2019	FP	7M	2	2019	Year-2	1	0	Winter
2/2/2019	FP	10M	2	2019	Year-2	1	0	Winter
2/2/2019	FP	12M	2	2019	Year-2	1	0	Winter

Date	Species	Site	Month	Year	Survey Year	Presence	Data Gap	Season
2/2/2019	FP	14M	2	2019	Year-2	1	0	Winter
2/2/2019	FP	8A	2	2019	Year-2		1	Winter
2/2/2019	FP	9A	2	2019	Year-2	1	0	Winter
2/2/2019	FP	11A	2	2019	Year-2	1	0	Winter
2/2/2019	FP	13A	2	2019	Year-2	1	0	Winter
2/3/2019	FP	1M	2	2019	Year-2	1	0	Winter
2/3/2019	FP	2M	2	2019	Year-2	1	0	Winter
2/3/2019	FP	3M	2	2019	Year-2	1	0	Winter
2/3/2019	FP	4M	2	2019	Year-2	1	0	Winter
2/3/2019	FP	5M	2	2019	Year-2	1	0	Winter
2/3/2019	FP	6M	2	2019	Year-2	1	0	Winter
2/3/2019	FP	7M	2	2019	Year-2	1	0	Winter
2/3/2019	FP	10M	2	2019	Year-2	1	0	Winter
2/3/2019	FP	12M	2	2019	Year-2	1	0	Winter
2/3/2019	FP	14M	2	2019	Year-2	1	0	Winter
2/3/2019	FP	8A	2	2019	Year-2		1	Winter
2/3/2019	FP	9A	2	2019	Year-2	1	0	Winter
2/3/2019	FP	11A	2	2019	Year-2	0	0	Winter
2/3/2019	FP	13A	2	2019	Year-2	1	0	Winter
2/4/2019	FP	1M	2	2019	Year-2	1	0	Winter
2/4/2019	FP	2M	2	2019	Year-2	1	0	Winter
2/4/2019	FP	3M	2	2019	Year-2	1	0	Winter
2/4/2019	FP	4M	2	2019	Year-2	1	0	Winter
2/4/2019	FP	5M	2	2019	Year-2	1	0	Winter
2/4/2019	FP	6M	2	2019	Year-2	1	0	Winter
2/4/2019	FP	7M	2	2019	Year-2	1	0	Winter
2/4/2019	FP	10M	2	2019	Year-2	1	0	Winter
2/4/2019	FP	12M	2	2019	Year-2	1	0	Winter
2/4/2019	FP	14M	2	2019	Year-2	1	0	Winter
2/4/2019	FP	8A	2	2019	Year-2		1	Winter
2/4/2019	FP	9A	2	2019	Year-2	1	0	Winter
2/4/2019	FP	11A	2	2019	Year-2	1	0	Winter
2/4/2019	FP	13A	2	2019	Year-2	1	0	Winter
2/5/2019	FP	1M	2	2019	Year-2	1	0	Winter
2/5/2019	FP	2M	2	2019	Year-2	1	0	Winter
2/5/2019	FP	3M	2	2019	Year-2	1	0	Winter
2/5/2019	FP	4M	2	2019	Year-2	1	0	Winter
2/5/2019	FP	5M	2	2019	Year-2	1	0	Winter
2/5/2019	FP	6M	2	2019	Year-2	1	0	Winter
2/5/2019	FP	7M	2	2019	Year-2	1	0	Winter
2/5/2019	FP	10M	2	2019	Year-2	1	0	Winter
2/5/2019	FP	12M	2	2019	Year-2	1	0	Winter
2/5/2019	FP	14M	2	2019	Year-2	1	0	Winter
2/5/2019	FP	8A	2	2019	Year-2		1	Winter

Date	Species	Site	Month	Year	Survey Year	Presence	Data Gap	Season
2/5/2019	FP	9A	2	2019	Year-2	1	0	Winter
2/5/2019	FP	11A	2	2019	Year-2	1	0	Winter
2/5/2019	FP	13A	2	2019	Year-2	1	0	Winter
2/6/2019	FP	1M	2	2019	Year-2	1	0	Winter
2/6/2019	FP	2M	2	2019	Year-2	1	0	Winter
2/6/2019	FP	3M	2	2019	Year-2	1	0	Winter
2/6/2019	FP	4M	2	2019	Year-2	1	0	Winter
2/6/2019	FP	5M	2	2019	Year-2	1	0	Winter
2/6/2019	FP	6M	2	2019	Year-2	1	0	Winter
2/6/2019	FP	7M	2	2019	Year-2	1	0	Winter
2/6/2019	FP	10M	2	2019	Year-2	1	0	Winter
2/6/2019	FP	12M	2	2019	Year-2	1	0	Winter
2/6/2019	FP	14M	2	2019	Year-2	1	0	Winter
2/6/2019	FP	8A	2	2019	Year-2		1	Winter
2/6/2019	FP	9A	2	2019	Year-2	1	0	Winter
2/6/2019	FP	11A	2	2019	Year-2	1	0	Winter
2/6/2019	FP	13A	2	2019	Year-2	1	0	Winter
2/7/2019	FP	1M	2	2019	Year-2	1	0	Winter
2/7/2019	FP	2M	2	2019	Year-2	1	0	Winter
2/7/2019	FP	3M	2	2019	Year-2	1	0	Winter
2/7/2019	FP	4M	2	2019	Year-2	1	0	Winter
2/7/2019	FP	5M	2	2019	Year-2	0	0	Winter
2/7/2019	FP	6M	2	2019	Year-2	0	0	Winter
2/7/2019	FP	7M	2	2019	Year-2	1	0	Winter
2/7/2019	FP	10M	2	2019	Year-2	1	0	Winter
2/7/2019	FP	12M	2	2019	Year-2	1	0	Winter
2/7/2019	FP	14M	2	2019	Year-2	1	0	Winter
2/7/2019	FP	8A	2	2019	Year-2		1	Winter
2/7/2019	FP	9A	2	2019	Year-2	1	0	Winter
2/7/2019	FP	11A	2	2019	Year-2	1	0	Winter
2/7/2019	FP	13A	2	2019	Year-2	1	0	Winter
2/8/2019	FP	1M	2	2019	Year-2	0	0	Winter
2/8/2019	FP	2M	2	2019	Year-2	0	0	Winter
2/8/2019	FP	3M	2	2019	Year-2	0	0	Winter
2/8/2019	FP	4M	2	2019	Year-2	0	0	Winter
2/8/2019	FP	5M	2	2019	Year-2	1	0	Winter
2/8/2019	FP	6M	2	2019	Year-2	1	0	Winter
2/8/2019	FP	7M	2	2019	Year-2	0	0	Winter
2/8/2019	FP	10M	2	2019	Year-2	0	0	Winter
2/8/2019	FP	12M	2	2019	Year-2	0	0	Winter
2/8/2019	FP	14M	2	2019	Year-2	0	0	Winter
2/8/2019	FP	8A	2	2019	Year-2		1	Winter
2/8/2019	FP	9A	2	2019	Year-2	1	0	Winter
2/8/2019	FP	11A	2	2019	Year-2	1	0	Winter

Date	Species	Site	Month	Year	Survey Year	Presence	Data Gap	Season
2/8/2019	FP	13A	2	2019	Year-2	1	0	Winter
2/9/2019	FP	1M	2	2019	Year-2	0	0	Winter
2/9/2019	FP	2M	2	2019	Year-2	1	0	Winter
2/9/2019	FP	3M	2	2019	Year-2	1	0	Winter
2/9/2019	FP	4M	2	2019	Year-2	1	0	Winter
2/9/2019	FP	5M	2	2019	Year-2	1	0	Winter
2/9/2019	FP	6M	2	2019	Year-2	1	0	Winter
2/9/2019	FP	7M	2	2019	Year-2	1	0	Winter
2/9/2019	FP	10M	2	2019	Year-2	1	0	Winter
2/9/2019	FP	12M	2	2019	Year-2	1	0	Winter
2/9/2019	FP	14M	2	2019	Year-2	1	0	Winter
2/9/2019	FP	8A	2	2019	Year-2		1	Winter
2/9/2019	FP	9A	2	2019	Year-2	1	0	Winter
2/9/2019	FP	11A	2	2019	Year-2	1	0	Winter
2/9/2019	FP	13A	2	2019	Year-2	1	0	Winter
2/10/2019	FP	1M	2	2019	Year-2	1	0	Winter
2/10/2019	FP	2M	2	2019	Year-2	0	0	Winter
2/10/2019	FP	3M	2	2019	Year-2	0	0	Winter
2/10/2019	FP	4M	2	2019	Year-2	0	0	Winter
2/10/2019	FP	5M	2	2019	Year-2	0	0	Winter
2/10/2019	FP	6M	2	2019	Year-2	0	0	Winter
2/10/2019	FP	7M	2	2019	Year-2	0	0	Winter
2/10/2019	FP	10M	2	2019	Year-2	0	0	Winter
2/10/2019	FP	12M	2	2019	Year-2	0	0	Winter
2/10/2019	FP	14M	2	2019	Year-2	0	0	Winter
2/10/2019	FP	8A	2	2019	Year-2		1	Winter
2/10/2019	FP	9A	2	2019	Year-2	1	0	Winter
2/10/2019	FP	11A	2	2019	Year-2	1	0	Winter
2/10/2019	FP	13A	2	2019	Year-2	1	0	Winter
2/11/2019	FP	1M	2	2019	Year-2	1	0	Winter
2/11/2019	FP	2M	2	2019	Year-2	1	0	Winter
2/11/2019	FP	3M	2	2019	Year-2	1	0	Winter
2/11/2019	FP	4M	2	2019	Year-2	1	0	Winter
2/11/2019	FP	5M	2	2019	Year-2	0	0	Winter
2/11/2019	FP	6M	2	2019	Year-2	1	0	Winter
2/11/2019	FP	7M	2	2019	Year-2	1	0	Winter
2/11/2019	FP	10M	2	2019	Year-2	1	0	Winter
2/11/2019	FP	12M	2	2019	Year-2	1	0	Winter
2/11/2019	FP	14M	2	2019	Year-2	1	0	Winter
2/11/2019	FP	8A	2	2019	Year-2		1	Winter
2/11/2019	FP	9A	2	2019	Year-2	1	0	Winter
2/11/2019	FP	11A	2	2019	Year-2	1	0	Winter
2/11/2019	FP	13A	2	2019	Year-2	1	0	Winter
2/12/2019	FP	1M	2	2019	Year-2	0	0	Winter

Date	Species	Site	Month	Year	Survey Year	Presence	Data Gap	Season
2/12/2019	FP	2M	2	2019	Year-2	0	0	Winter
2/12/2019	FP	3M	2	2019	Year-2	0	0	Winter
2/12/2019	FP	4M	2	2019	Year-2	0	0	Winter
2/12/2019	FP	5M	2	2019	Year-2	1	0	Winter
2/12/2019	FP	6M	2	2019	Year-2	0	0	Winter
2/12/2019	FP	7M	2	2019	Year-2	0	0	Winter
2/12/2019	FP	10M	2	2019	Year-2	0	0	Winter
2/12/2019	FP	12M	2	2019	Year-2	0	0	Winter
2/12/2019	FP	14M	2	2019	Year-2	0	0	Winter
2/12/2019	FP	8A	2	2019	Year-2		1	Winter
2/12/2019	FP	9A	2	2019	Year-2	1	0	Winter
2/12/2019	FP	11A	2	2019	Year-2	1	0	Winter
2/12/2019	FP	13A	2	2019	Year-2	1	0	Winter
2/13/2019	FP	1M	2	2019	Year-2	1	0	Winter
2/13/2019	FP	2M	2	2019	Year-2	1	0	Winter
2/13/2019	FP	3M	2	2019	Year-2	1	0	Winter
2/13/2019	FP	4M	2	2019	Year-2	1	0	Winter
2/13/2019	FP	5M	2	2019	Year-2	1	0	Winter
2/13/2019	FP	6M	2	2019	Year-2	1	0	Winter
2/13/2019	FP	7M	2	2019	Year-2	1	0	Winter
2/13/2019	FP	10M	2	2019	Year-2	1	0	Winter
2/13/2019	FP	12M	2	2019	Year-2	1	0	Winter
2/13/2019	FP	14M	2	2019	Year-2	1	0	Winter
2/13/2019	FP	8A	2	2019	Year-2		1	Winter
2/13/2019	FP	9A	2	2019	Year-2	1	0	Winter
2/13/2019	FP	11A	2	2019	Year-2	1	0	Winter
2/13/2019	FP	13A	2	2019	Year-2	1	0	Winter
2/14/2019	FP	1M	2	2019	Year-2	0	0	Winter
2/14/2019	FP	2M	2	2019	Year-2	0	0	Winter
2/14/2019	FP	3M	2	2019	Year-2	1	0	Winter
2/14/2019	FP	4M	2	2019	Year-2	0	0	Winter
2/14/2019	FP	5M	2	2019	Year-2	0	0	Winter
2/14/2019	FP	6M	2	2019	Year-2	0	0	Winter
2/14/2019	FP	7M	2	2019	Year-2	0	0	Winter
2/14/2019	FP	10M	2	2019	Year-2	0	0	Winter
2/14/2019	FP	12M	2	2019	Year-2	1	0	Winter
2/14/2019	FP	14M	2	2019	Year-2	0	0	Winter
2/14/2019	FP	8A	2	2019	Year-2		1	Winter
2/14/2019	FP	9A	2	2019	Year-2	1	0	Winter
2/14/2019	FP	11A	2	2019	Year-2	0	0	Winter
2/14/2019	FP	13A	2	2019	Year-2	1	0	Winter
2/15/2019	FP	1M	2	2019	Year-2	0	0	Winter
2/15/2019	FP	2M	2	2019	Year-2	0	0	Winter
2/15/2019	FP	3M	2	2019	Year-2	0	0	Winter

Date	Species	Site	Month	Year	Survey Year	Presence	Data Gap	Season
2/15/2019	FP	4M	2	2019	Year-2	0	0	Winter
2/15/2019	FP	5M	2	2019	Year-2	0	0	Winter
2/15/2019	FP	6M	2	2019	Year-2	0	0	Winter
2/15/2019	FP	7M	2	2019	Year-2	0	0	Winter
2/15/2019	FP	10M	2	2019	Year-2	0	0	Winter
2/15/2019	FP	12M	2	2019	Year-2	0	0	Winter
2/15/2019	FP	14M	2	2019	Year-2	0	0	Winter
2/15/2019	FP	8A	2	2019	Year-2		1	Winter
2/15/2019	FP	9A	2	2019	Year-2	1	0	Winter
2/15/2019	FP	11A	2	2019	Year-2	1	0	Winter
2/15/2019	FP	13A	2	2019	Year-2	1	0	Winter
2/16/2019	FP	1M	2	2019	Year-2	1	0	Winter
2/16/2019	FP	2M	2	2019	Year-2	1	0	Winter
2/16/2019	FP	3M	2	2019	Year-2	1	0	Winter
2/16/2019	FP	4M	2	2019	Year-2	1	0	Winter
2/16/2019	FP	5M	2	2019	Year-2	0	0	Winter
2/16/2019	FP	6M	2	2019	Year-2	0	0	Winter
2/16/2019	FP	7M	2	2019	Year-2	0	0	Winter
2/16/2019	FP	10M	2	2019	Year-2	0	0	Winter
2/16/2019	FP	12M	2	2019	Year-2	0	0	Winter
2/16/2019	FP	14M	2	2019	Year-2	0	0	Winter
2/16/2019	FP	8A	2	2019	Year-2		1	Winter
2/16/2019	FP	9A	2	2019	Year-2	1	0	Winter
2/16/2019	FP	11A	2	2019	Year-2	1	0	Winter
2/16/2019	FP	13A	2	2019	Year-2	1	0	Winter
2/17/2019	FP	1M	2	2019	Year-2	0	0	Winter
2/17/2019	FP	2M	2	2019	Year-2	0	0	Winter
2/17/2019	FP	3M	2	2019	Year-2	0	0	Winter
2/17/2019	FP	4M	2	2019	Year-2	0	0	Winter
2/17/2019	FP	5M	2	2019	Year-2	1	0	Winter
2/17/2019	FP	6M	2	2019	Year-2	1	0	Winter
2/17/2019	FP	7M	2	2019	Year-2	0	0	Winter
2/17/2019	FP	10M	2	2019	Year-2	1	0	Winter
2/17/2019	FP	12M	2	2019	Year-2	0	0	Winter
2/17/2019	FP	14M	2	2019	Year-2	1	0	Winter
2/17/2019	FP	8A	2	2019	Year-2		1	Winter
2/17/2019	FP	9A	2	2019	Year-2	0	0	Winter
2/17/2019	FP	11A	2	2019	Year-2	1	0	Winter
2/17/2019	FP	13A	2	2019	Year-2	1	0	Winter
2/18/2019	FP	1M	2	2019	Year-2	0	0	Winter
2/18/2019	FP	2M	2	2019	Year-2	0	0	Winter
2/18/2019	FP	3M	2	2019	Year-2	0	0	Winter
2/18/2019	FP	4M	2	2019	Year-2	0	0	Winter
2/18/2019	FP	5M	2	2019	Year-2	0	0	Winter

Date	Species	Site	Month	Year	Survey Year	Presence	Data Gap	Season
2/18/2019	FP	6M	2	2019	Year-2	0	0	Winter
2/18/2019	FP	7M	2	2019	Year-2	1	0	Winter
2/18/2019	FP	10M	2	2019	Year-2	0	0	Winter
2/18/2019	FP	12M	2	2019	Year-2	0	0	Winter
2/18/2019	FP	14M	2	2019	Year-2	0	0	Winter
2/18/2019	FP	8A	2	2019	Year-2		1	Winter
2/18/2019	FP	9A	2	2019	Year-2	1	0	Winter
2/18/2019	FP	11A	2	2019	Year-2	1	0	Winter
2/18/2019	FP	13A	2	2019	Year-2	1	0	Winter
2/19/2019	FP	1M	2	2019	Year-2	1	0	Winter
2/19/2019	FP	2M	2	2019	Year-2	1	0	Winter
2/19/2019	FP	3M	2	2019	Year-2	1	0	Winter
2/19/2019	FP	4M	2	2019	Year-2	1	0	Winter
2/19/2019	FP	5M	2	2019	Year-2	1	0	Winter
2/19/2019	FP	6M	2	2019	Year-2	1	0	Winter
2/19/2019	FP	7M	2	2019	Year-2	1	0	Winter
2/19/2019	FP	10M	2	2019	Year-2	1	0	Winter
2/19/2019	FP	12M	2	2019	Year-2	1	0	Winter
2/19/2019	FP	14M	2	2019	Year-2	1	0	Winter
2/19/2019	FP	8A	2	2019	Year-2		1	Winter
2/19/2019	FP	9A	2	2019	Year-2	1	0	Winter
2/19/2019	FP	11A	2	2019	Year-2	1	0	Winter
2/19/2019	FP	13A	2	2019	Year-2	1	0	Winter
2/20/2019	FP	1M	2	2019	Year-2	1	0	Winter
2/20/2019	FP	2M	2	2019	Year-2	1	0	Winter
2/20/2019	FP	3M	2	2019	Year-2	1	0	Winter
2/20/2019	FP	4M	2	2019	Year-2	1	0	Winter
2/20/2019	FP	5M	2	2019	Year-2	1	0	Winter
2/20/2019	FP	6M	2	2019	Year-2	1	0	Winter
2/20/2019	FP	7M	2	2019	Year-2	1	0	Winter
2/20/2019	FP	10M	2	2019	Year-2	0	0	Winter
2/20/2019	FP	12M	2	2019	Year-2	0	0	Winter
2/20/2019	FP	14M	2	2019	Year-2	1	0	Winter
2/20/2019	FP	8A	2	2019	Year-2		1	Winter
2/20/2019	FP	9A	2	2019	Year-2	1	0	Winter
2/20/2019	FP	11A	2	2019	Year-2	0	0	Winter
2/20/2019	FP	13A	2	2019	Year-2	1	0	Winter
2/21/2019	FP	1M	2	2019	Year-2	0	0	Winter
2/21/2019	FP	2M	2	2019	Year-2	0	0	Winter
2/21/2019	FP	3M	2	2019	Year-2	0	0	Winter
2/21/2019	FP	4M	2	2019	Year-2	0	0	Winter
2/21/2019	FP	5M	2	2019	Year-2	0	0	Winter
2/21/2019	FP	6M	2	2019	Year-2	0	0	Winter
2/21/2019	FP	7M	2	2019	Year-2	0	0	Winter

Date	Species	Site	Month	Year	Survey Year	Presence	Data Gap	Season
2/21/2019	FP	10M	2	2019	Year-2	1	0	Winter
2/21/2019	FP	12M	2	2019	Year-2	1	0	Winter
2/21/2019	FP	14M	2	2019	Year-2	0	0	Winter
2/21/2019	FP	8A	2	2019	Year-2		1	Winter
2/21/2019	FP	9A	2	2019	Year-2	1	0	Winter
2/21/2019	FP	11A	2	2019	Year-2	0	0	Winter
2/21/2019	FP	13A	2	2019	Year-2	1	0	Winter
2/22/2019	FP	1M	2	2019	Year-2	1	0	Winter
2/22/2019	FP	2M	2	2019	Year-2	1	0	Winter
2/22/2019	FP	3M	2	2019	Year-2	1	0	Winter
2/22/2019	FP	4M	2	2019	Year-2	1	0	Winter
2/22/2019	FP	5M	2	2019	Year-2	1	0	Winter
2/22/2019	FP	6M	2	2019	Year-2	1	0	Winter
2/22/2019	FP	7M	2	2019	Year-2	0	0	Winter
2/22/2019	FP	10M	2	2019	Year-2	1	0	Winter
2/22/2019	FP	12M	2	2019	Year-2	1	0	Winter
2/22/2019	FP	14M	2	2019	Year-2	1	0	Winter
2/22/2019	FP	8A	2	2019	Year-2		1	Winter
2/22/2019	FP	9A	2	2019	Year-2	1	0	Winter
2/22/2019	FP	11A	2	2019	Year-2	1	0	Winter
2/22/2019	FP	13A	2	2019	Year-2	1	0	Winter
2/23/2019	FP	1M	2	2019	Year-2	1	0	Winter
2/23/2019	FP	2M	2	2019	Year-2	1	0	Winter
2/23/2019	FP	3M	2	2019	Year-2	1	0	Winter
2/23/2019	FP	4M	2	2019	Year-2	1	0	Winter
2/23/2019	FP	5M	2	2019	Year-2	1	0	Winter
2/23/2019	FP	6M	2	2019	Year-2	1	0	Winter
2/23/2019	FP	7M	2	2019	Year-2	0	0	Winter
2/23/2019	FP	10M	2	2019	Year-2	1	0	Winter
2/23/2019	FP	12M	2	2019	Year-2	1	0	Winter
2/23/2019	FP	14M	2	2019	Year-2	1	0	Winter
2/23/2019	FP	8A	2	2019	Year-2		1	Winter
2/23/2019	FP	9A	2	2019	Year-2	0	0	Winter
2/23/2019	FP	11A	2	2019	Year-2	1	0	Winter
2/23/2019	FP	13A	2	2019	Year-2	1	0	Winter
2/24/2019	FP	1M	2	2019	Year-2	0	0	Winter
2/24/2019	FP	2M	2	2019	Year-2	0	0	Winter
2/24/2019	FP	3M	2	2019	Year-2	1	0	Winter
2/24/2019	FP	4M	2	2019	Year-2	0	0	Winter
2/24/2019	FP	5M	2	2019	Year-2	1	0	Winter
2/24/2019	FP	6M	2	2019	Year-2	1	0	Winter
2/24/2019	FP	7M	2	2019	Year-2	1	0	Winter
2/24/2019	FP	10M	2	2019	Year-2	1	0	Winter
2/24/2019	FP	12M	2	2019	Year-2	1	0	Winter

Date	Species	Site	Month	Year	Survey Year	Presence	Data Gap	Season
2/24/2019	FP	14M	2	2019	Year-2	1	0	Winter
2/24/2019	FP	8A	2	2019	Year-2		1	Winter
2/24/2019	FP	9A	2	2019	Year-2	1	0	Winter
2/24/2019	FP	11A	2	2019	Year-2	1	0	Winter
2/24/2019	FP	13A	2	2019	Year-2	1	0	Winter
2/25/2019	FP	1M	2	2019	Year-2	1	0	Winter
2/25/2019	FP	2M	2	2019	Year-2	1	0	Winter
2/25/2019	FP	3M	2	2019	Year-2	0	0	Winter
2/25/2019	FP	4M	2	2019	Year-2	1	0	Winter
2/25/2019	FP	5M	2	2019	Year-2	0	0	Winter
2/25/2019	FP	6M	2	2019	Year-2	0	0	Winter
2/25/2019	FP	7M	2	2019	Year-2	0	0	Winter
2/25/2019	FP	10M	2	2019	Year-2	0	0	Winter
2/25/2019	FP	12M	2	2019	Year-2	0	0	Winter
2/25/2019	FP	14M	2	2019	Year-2	0	0	Winter
2/25/2019	FP	8A	2	2019	Year-2		1	Winter
2/25/2019	FP	9A	2	2019	Year-2	1	0	Winter
2/25/2019	FP	11A	2	2019	Year-2	1	0	Winter
2/25/2019	FP	13A	2	2019	Year-2	1	0	Winter
2/26/2019	FP	1M	2	2019	Year-2	1	0	Winter
2/26/2019	FP	2M	2	2019	Year-2	1	0	Winter
2/26/2019	FP	3M	2	2019	Year-2	1	0	Winter
2/26/2019	FP	4M	2	2019	Year-2	1	0	Winter
2/26/2019	FP	5M	2	2019	Year-2	0	0	Winter
2/26/2019	FP	6M	2	2019	Year-2	1	0	Winter
2/26/2019	FP	7M	2	2019	Year-2	1	0	Winter
2/26/2019	FP	10M	2	2019	Year-2	1	0	Winter
2/26/2019	FP	12M	2	2019	Year-2	1	0	Winter
2/26/2019	FP	14M	2	2019	Year-2	1	0	Winter
2/26/2019	FP	8A	2	2019	Year-2		1	Winter
2/26/2019	FP	9A	2	2019	Year-2	1	0	Winter
2/26/2019	FP	11A	2	2019	Year-2	1	0	Winter
2/26/2019	FP	13A	2	2019	Year-2	1	0	Winter
2/27/2019	FP	1M	2	2019	Year-2	1	0	Winter
2/27/2019	FP	2M	2	2019	Year-2	1	0	Winter
2/27/2019	FP	3M	2	2019	Year-2	1	0	Winter
2/27/2019	FP	4M	2	2019	Year-2	1	0	Winter
2/27/2019	FP	5M	2	2019	Year-2	1	0	Winter
2/27/2019	FP	6M	2	2019	Year-2	1	0	Winter
2/27/2019	FP	7M	2	2019	Year-2	1	0	Winter
2/27/2019	FP	10M	2	2019	Year-2	1	0	Winter
2/27/2019	FP	12M	2	2019	Year-2	1	0	Winter
2/27/2019	FP	14M	2	2019	Year-2	1	0	Winter
2/27/2019	FP	8A	2	2019	Year-2		1	Winter

Date	Species	Site	Month	Year	Survey Year	Presence	Data Gap	Season
2/27/2019	FP	9A	2	2019	Year-2	1	0	Winter
2/27/2019	FP	11A	2	2019	Year-2	1	0	Winter
2/27/2019	FP	13A	2	2019	Year-2	1	0	Winter
2/28/2019	FP	1M	2	2019	Year-2	1	0	Winter
2/28/2019	FP	2M	2	2019	Year-2	1	0	Winter
2/28/2019	FP	3M	2	2019	Year-2	1	0	Winter
2/28/2019	FP	4M	2	2019	Year-2	1	0	Winter
2/28/2019	FP	5M	2	2019	Year-2	1	0	Winter
2/28/2019	FP	6M	2	2019	Year-2	1	0	Winter
2/28/2019	FP	7M	2	2019	Year-2	1	0	Winter
2/28/2019	FP	10M	2	2019	Year-2	1	0	Winter
2/28/2019	FP	12M	2	2019	Year-2	1	0	Winter
2/28/2019	FP	14M	2	2019	Year-2	1	0	Winter
2/28/2019	FP	8A	2	2019	Year-2		1	Winter
2/28/2019	FP	9A	2	2019	Year-2	0	0	Winter
2/28/2019	FP	11A	2	2019	Year-2	1	0	Winter
2/28/2019	FP	13A	2	2019	Year-2	1	0	Winter
3/1/2019	FP	1M	3	2019	Year-2	1	0	Spring
3/1/2019	FP	2M	3	2019	Year-2	1	0	Spring
3/1/2019	FP	3M	3	2019	Year-2	1	0	Spring
3/1/2019	FP	4M	3	2019	Year-2	1	0	Spring
3/1/2019	FP	5M	3	2019	Year-2	1	0	Spring
3/1/2019	FP	6M	3	2019	Year-2	1	0	Spring
3/1/2019	FP	7M	3	2019	Year-2	0	0	Spring
3/1/2019	FP	10M	3	2019	Year-2	1	0	Spring
3/1/2019	FP	12M	3	2019	Year-2	1	0	Spring
3/1/2019	FP	14M	3	2019	Year-2	1	0	Spring
3/1/2019	FP	8A	3	2019	Year-2		1	Spring
3/1/2019	FP	9A	3	2019	Year-2	0	0	Spring
3/1/2019	FP	11A	3	2019	Year-2	1	0	Spring
3/1/2019	FP	13A	3	2019	Year-2	1	0	Spring
3/2/2019	FP	1M	3	2019	Year-2	1	0	Spring
3/2/2019	FP	2M	3	2019	Year-2	1	0	Spring
3/2/2019	FP	3M	3	2019	Year-2	1	0	Spring
3/2/2019	FP	4M	3	2019	Year-2	1	0	Spring
3/2/2019	FP	5M	3	2019	Year-2	1	0	Spring
3/2/2019	FP	6M	3	2019	Year-2	1	0	Spring
3/2/2019	FP	7M	3	2019	Year-2	1	0	Spring
3/2/2019	FP	10M	3	2019	Year-2	1	0	Spring
3/2/2019	FP	12M	3	2019	Year-2	1	0	Spring
3/2/2019	FP	14M	3	2019	Year-2	1	0	Spring
3/2/2019	FP	8A	3	2019	Year-2		1	Spring
3/2/2019	FP	9A	3	2019	Year-2	1	0	Spring
3/2/2019	FP	11A	3	2019	Year-2	0	0	Spring

Date	Species	Site	Month	Year	Survey Year	Presence	Data Gap	Season
3/2/2019	FP	13A	3	2019	Year-2	1	0	Spring
3/3/2019	FP	1M	3	2019	Year-2	1	0	Spring
3/3/2019	FP	2M	3	2019	Year-2	1	0	Spring
3/3/2019	FP	3M	3	2019	Year-2	1	0	Spring
3/3/2019	FP	4M	3	2019	Year-2	1	0	Spring
3/3/2019	FP	5M	3	2019	Year-2	1	0	Spring
3/3/2019	FP	6M	3	2019	Year-2	1	0	Spring
3/3/2019	FP	7M	3	2019	Year-2	1	0	Spring
3/3/2019	FP	10M	3	2019	Year-2	1	0	Spring
3/3/2019	FP	12M	3	2019	Year-2	1	0	Spring
3/3/2019	FP	14M	3	2019	Year-2	1	0	Spring
3/3/2019	FP	8A	3	2019	Year-2		1	Spring
3/3/2019	FP	9A	3	2019	Year-2	1	0	Spring
3/3/2019	FP	11A	3	2019	Year-2	1	0	Spring
3/3/2019	FP	13A	3	2019	Year-2	1	0	Spring
3/4/2019	FP	1M	3	2019	Year-2	1	0	Spring
3/4/2019	FP	2M	3	2019	Year-2	1	0	Spring
3/4/2019	FP	3M	3	2019	Year-2	1	0	Spring
3/4/2019	FP	4M	3	2019	Year-2	1	0	Spring
3/4/2019	FP	5M	3	2019	Year-2	1	0	Spring
3/4/2019	FP	6M	3	2019	Year-2	1	0	Spring
3/4/2019	FP	7M	3	2019	Year-2	1	0	Spring
3/4/2019	FP	10M	3	2019	Year-2	1	0	Spring
3/4/2019	FP	12M	3	2019	Year-2	1	0	Spring
3/4/2019	FP	14M	3	2019	Year-2	1	0	Spring
3/4/2019	FP	8A	3	2019	Year-2		1	Spring
3/4/2019	FP	9A	3	2019	Year-2	1	0	Spring
3/4/2019	FP	11A	3	2019	Year-2	1	0	Spring
3/4/2019	FP	13A	3	2019	Year-2	1	0	Spring
3/5/2019	FP	1M	3	2019	Year-2	1	0	Spring
3/5/2019	FP	2M	3	2019	Year-2	1	0	Spring
3/5/2019	FP	3M	3	2019	Year-2	1	0	Spring
3/5/2019	FP	4M	3	2019	Year-2	1	0	Spring
3/5/2019	FP	5M	3	2019	Year-2	1	0	Spring
3/5/2019	FP	6M	3	2019	Year-2	1	0	Spring
3/5/2019	FP	7M	3	2019	Year-2	0	0	Spring
3/5/2019	FP	10M	3	2019	Year-2	1	0	Spring
3/5/2019	FP	12M	3	2019	Year-2	1	0	Spring
3/5/2019	FP	14M	3	2019	Year-2	1	0	Spring
3/5/2019	FP	8A	3	2019	Year-2		1	Spring
3/5/2019	FP	9A	3	2019	Year-2	1	0	Spring
3/5/2019	FP	11A	3	2019	Year-2	1	0	Spring
3/5/2019	FP	13A	3	2019	Year-2	1	0	Spring
3/6/2019	FP	1M	3	2019	Year-2	0	0	Spring

Date	Species	Site	Month	Year	Survey Year	Presence	Data Gap	Season
3/6/2019	FP	2M	3	2019	Year-2	0	0	Spring
3/6/2019	FP	3M	3	2019	Year-2	0	0	Spring
3/6/2019	FP	4M	3	2019	Year-2	0	0	Spring
3/6/2019	FP	5M	3	2019	Year-2	0	0	Spring
3/6/2019	FP	6M	3	2019	Year-2	0	0	Spring
3/6/2019	FP	7M	3	2019	Year-2	1	0	Spring
3/6/2019	FP	10M	3	2019	Year-2	0	0	Spring
3/6/2019	FP	12M	3	2019	Year-2	0	0	Spring
3/6/2019	FP	14M	3	2019	Year-2	0	0	Spring
3/6/2019	FP	8A	3	2019	Year-2		1	Spring
3/6/2019	FP	9A	3	2019	Year-2	1	0	Spring
3/6/2019	FP	11A	3	2019	Year-2	1	0	Spring
3/6/2019	FP	13A	3	2019	Year-2	1	0	Spring
3/7/2019	FP	1M	3	2019	Year-2	1	0	Spring
3/7/2019	FP	2M	3	2019	Year-2	1	0	Spring
3/7/2019	FP	3M	3	2019	Year-2	1	0	Spring
3/7/2019	FP	4M	3	2019	Year-2	1	0	Spring
3/7/2019	FP	5M	3	2019	Year-2	1	0	Spring
3/7/2019	FP	6M	3	2019	Year-2	1	0	Spring
3/7/2019	FP	7M	3	2019	Year-2	0	0	Spring
3/7/2019	FP	10M	3	2019	Year-2	1	0	Spring
3/7/2019	FP	12M	3	2019	Year-2	1	0	Spring
3/7/2019	FP	14M	3	2019	Year-2	1	0	Spring
3/7/2019	FP	8A	3	2019	Year-2		1	Spring
3/7/2019	FP	9A	3	2019	Year-2	1	0	Spring
3/7/2019	FP	11A	3	2019	Year-2	1	0	Spring
3/7/2019	FP	13A	3	2019	Year-2	1	0	Spring
3/8/2019	FP	1M	3	2019	Year-2	1	0	Spring
3/8/2019	FP	2M	3	2019	Year-2	1	0	Spring
3/8/2019	FP	3M	3	2019	Year-2	1	0	Spring
3/8/2019	FP	4M	3	2019	Year-2	1	0	Spring
3/8/2019	FP	5M	3	2019	Year-2	1	0	Spring
3/8/2019	FP	6M	3	2019	Year-2	1	0	Spring
3/8/2019	FP	7M	3	2019	Year-2	1	0	Spring
3/8/2019	FP	10M	3	2019	Year-2	1	0	Spring
3/8/2019	FP	12M	3	2019	Year-2	1	0	Spring
3/8/2019	FP	14M	3	2019	Year-2	1	0	Spring
3/8/2019	FP	8A	3	2019	Year-2		1	Spring
3/8/2019	FP	9A	3	2019	Year-2	1	0	Spring
3/8/2019	FP	11A	3	2019	Year-2	1	0	Spring
3/8/2019	FP	13A	3	2019	Year-2	1	0	Spring
3/9/2019	FP	1M	3	2019	Year-2	1	0	Spring
3/9/2019	FP	2M	3	2019	Year-2	1	0	Spring
3/9/2019	FP	3M	3	2019	Year-2	1	0	Spring

Date	Species	Site	Month	Year	Survey Year	Presence	Data Gap	Season
3/9/2019	FP	4M	3	2019	Year-2	1	0	Spring
3/9/2019	FP	5M	3	2019	Year-2	1	0	Spring
3/9/2019	FP	6M	3	2019	Year-2	1	0	Spring
3/9/2019	FP	7M	3	2019	Year-2	0	0	Spring
3/9/2019	FP	10M	3	2019	Year-2	1	0	Spring
3/9/2019	FP	12M	3	2019	Year-2	1	0	Spring
3/9/2019	FP	14M	3	2019	Year-2	1	0	Spring
3/9/2019	FP	8A	3	2019	Year-2		1	Spring
3/9/2019	FP	9A	3	2019	Year-2	1	0	Spring
3/9/2019	FP	11A	3	2019	Year-2	1	0	Spring
3/9/2019	FP	13A	3	2019	Year-2	0	0	Spring
3/10/2019	FP	1M	3	2019	Year-2	1	0	Spring
3/10/2019	FP	2M	3	2019	Year-2	0	0	Spring
3/10/2019	FP	3M	3	2019	Year-2	1	0	Spring
3/10/2019	FP	4M	3	2019	Year-2	1	0	Spring
3/10/2019	FP	5M	3	2019	Year-2	1	0	Spring
3/10/2019	FP	6M	3	2019	Year-2	1	0	Spring
3/10/2019	FP	7M	3	2019	Year-2	1	0	Spring
3/10/2019	FP	10M	3	2019	Year-2	1	0	Spring
3/10/2019	FP	12M	3	2019	Year-2	1	0	Spring
3/10/2019	FP	14M	3	2019	Year-2	1	0	Spring
3/10/2019	FP	8A	3	2019	Year-2		1	Spring
3/10/2019	FP	9A	3	2019	Year-2	1	0	Spring
3/10/2019	FP	11A	3	2019	Year-2	1	0	Spring
3/10/2019	FP	13A	3	2019	Year-2	1	0	Spring
3/11/2019	FP	1M	3	2019	Year-2	1	0	Spring
3/11/2019	FP	2M	3	2019	Year-2	1	0	Spring
3/11/2019	FP	3M	3	2019	Year-2	1	0	Spring
3/11/2019	FP	4M	3	2019	Year-2	1	0	Spring
3/11/2019	FP	5M	3	2019	Year-2	1	0	Spring
3/11/2019	FP	6M	3	2019	Year-2	1	0	Spring
3/11/2019	FP	7M	3	2019	Year-2	1	0	Spring
3/11/2019	FP	10M	3	2019	Year-2	1	0	Spring
3/11/2019	FP	12M	3	2019	Year-2	1	0	Spring
3/11/2019	FP	14M	3	2019	Year-2	1	0	Spring
3/11/2019	FP	8A	3	2019	Year-2		1	Spring
3/11/2019	FP	9A	3	2019	Year-2	1	0	Spring
3/11/2019	FP	11A	3	2019	Year-2	1	0	Spring
3/11/2019	FP	13A	3	2019	Year-2	1	0	Spring
3/12/2019	FP	1M	3	2019	Year-2	1	0	Spring
3/12/2019	FP	2M	3	2019	Year-2	1	0	Spring
3/12/2019	FP	3M	3	2019	Year-2	1	0	Spring
3/12/2019	FP	4M	3	2019	Year-2	0	0	Spring
3/12/2019	FP	5M	3	2019	Year-2	0	0	Spring

Date	Species	Site	Month	Year	Survey Year	Presence	Data Gap	Season
3/12/2019	FP	6M	3	2019	Year-2	1	0	Spring
3/12/2019	FP	7M	3	2019	Year-2	1	0	Spring
3/12/2019	FP	10M	3	2019	Year-2	1	0	Spring
3/12/2019	FP	12M	3	2019	Year-2	1	0	Spring
3/12/2019	FP	14M	3	2019	Year-2	1	0	Spring
3/12/2019	FP	8A	3	2019	Year-2		1	Spring
3/12/2019	FP	9A	3	2019	Year-2	1	0	Spring
3/12/2019	FP	11A	3	2019	Year-2	1	0	Spring
3/12/2019	FP	13A	3	2019	Year-2	1	0	Spring
3/13/2019	FP	1M	3	2019	Year-2	1	0	Spring
3/13/2019	FP	2M	3	2019	Year-2	1	0	Spring
3/13/2019	FP	3M	3	2019	Year-2	1	0	Spring
3/13/2019	FP	4M	3	2019	Year-2	1	0	Spring
3/13/2019	FP	5M	3	2019	Year-2	0	0	Spring
3/13/2019	FP	6M	3	2019	Year-2	1	0	Spring
3/13/2019	FP	7M	3	2019	Year-2	1	0	Spring
3/13/2019	FP	10M	3	2019	Year-2	1	0	Spring
3/13/2019	FP	12M	3	2019	Year-2	1	0	Spring
3/13/2019	FP	14M	3	2019	Year-2	1	0	Spring
3/13/2019	FP	8A	3	2019	Year-2		1	Spring
3/13/2019	FP	9A	3	2019	Year-2	1	0	Spring
3/13/2019	FP	11A	3	2019	Year-2	1	0	Spring
3/13/2019	FP	13A	3	2019	Year-2	1	0	Spring
3/14/2019	FP	1M	3	2019	Year-2	1	0	Spring
3/14/2019	FP	2M	3	2019	Year-2	1	0	Spring
3/14/2019	FP	3M	3	2019	Year-2	1	0	Spring
3/14/2019	FP	4M	3	2019	Year-2	1	0	Spring
3/14/2019	FP	5M	3	2019	Year-2	1	0	Spring
3/14/2019	FP	6M	3	2019	Year-2	1	0	Spring
3/14/2019	FP	7M	3	2019	Year-2	0	0	Spring
3/14/2019	FP	10M	3	2019	Year-2	1	0	Spring
3/14/2019	FP	12M	3	2019	Year-2	1	0	Spring
3/14/2019	FP	14M	3	2019	Year-2	1	0	Spring
3/14/2019	FP	8A	3	2019	Year-2	1	0	Spring
3/14/2019	FP	9A	3	2019	Year-2	1	0	Spring
3/14/2019	FP	11A	3	2019	Year-2		1	Spring
3/14/2019	FP	13A	3	2019	Year-2		1	Spring
3/15/2019	FP	1M	3	2019	Year-2	1	0	Spring
3/15/2019	FP	2M	3	2019	Year-2	1	0	Spring
3/15/2019	FP	3M	3	2019	Year-2	1	0	Spring
3/15/2019	FP	4M	3	2019	Year-2	1	0	Spring
3/15/2019	FP	5M	3	2019	Year-2	1	0	Spring
3/15/2019	FP	6M	3	2019	Year-2	1	0	Spring
3/15/2019	FP	7M	3	2019	Year-2	1	0	Spring

Date	Species	Site	Month	Year	Survey Year	Presence	Data Gap	Season
3/15/2019	FP	10M	3	2019	Year-2	1	0	Spring
3/15/2019	FP	12M	3	2019	Year-2	1	0	Spring
3/15/2019	FP	14M	3	2019	Year-2	0	0	Spring
3/15/2019	FP	8A	3	2019	Year-2	1	0	Spring
3/15/2019	FP	9A	3	2019	Year-2	1	0	Spring
3/15/2019	FP	11A	3	2019	Year-2	1	0	Spring
3/15/2019	FP	13A	3	2019	Year-2	1	0	Spring
3/16/2019	FP	1M	3	2019	Year-2	1	0	Spring
3/16/2019	FP	2M	3	2019	Year-2	1	0	Spring
3/16/2019	FP	3M	3	2019	Year-2	1	0	Spring
3/16/2019	FP	4M	3	2019	Year-2	1	0	Spring
3/16/2019	FP	5M	3	2019	Year-2	1	0	Spring
3/16/2019	FP	6M	3	2019	Year-2	1	0	Spring
3/16/2019	FP	7M	3	2019	Year-2	0	0	Spring
3/16/2019	FP	10M	3	2019	Year-2	1	0	Spring
3/16/2019	FP	12M	3	2019	Year-2	1	0	Spring
3/16/2019	FP	14M	3	2019	Year-2	1	0	Spring
3/16/2019	FP	8A	3	2019	Year-2	1	0	Spring
3/16/2019	FP	9A	3	2019	Year-2	0	0	Spring
3/16/2019	FP	11A	3	2019	Year-2	1	0	Spring
3/16/2019	FP	13A	3	2019	Year-2	1	0	Spring
3/17/2019	FP	1M	3	2019	Year-2	1	0	Spring
3/17/2019	FP	2M	3	2019	Year-2	1	0	Spring
3/17/2019	FP	3M	3	2019	Year-2	1	0	Spring
3/17/2019	FP	4M	3	2019	Year-2	1	0	Spring
3/17/2019	FP	5M	3	2019	Year-2	1	0	Spring
3/17/2019	FP	6M	3	2019	Year-2	1	0	Spring
3/17/2019	FP	7M	3	2019	Year-2	1	0	Spring
3/17/2019	FP	10M	3	2019	Year-2	1	0	Spring
3/17/2019	FP	12M	3	2019	Year-2	1	0	Spring
3/17/2019	FP	14M	3	2019	Year-2	1	0	Spring
3/17/2019	FP	8A	3	2019	Year-2	0	0	Spring
3/17/2019	FP	9A	3	2019	Year-2	1	0	Spring
3/17/2019	FP	11A	3	2019	Year-2	1	0	Spring
3/17/2019	FP	13A	3	2019	Year-2	1	0	Spring
3/18/2019	FP	1M	3	2019	Year-2	1	0	Spring
3/18/2019	FP	2M	3	2019	Year-2	1	0	Spring
3/18/2019	FP	3M	3	2019	Year-2	1	0	Spring
3/18/2019	FP	4M	3	2019	Year-2	1	0	Spring
3/18/2019	FP	5M	3	2019	Year-2	1	0	Spring
3/18/2019	FP	6M	3	2019	Year-2	1	0	Spring
3/18/2019	FP	7M	3	2019	Year-2	1	0	Spring
3/18/2019	FP	10M	3	2019	Year-2	1	0	Spring
3/18/2019	FP	12M	3	2019	Year-2	1	0	Spring

Date	Species	Site	Month	Year	Survey Year	Presence	Data Gap	Season
3/18/2019	FP	14M	3	2019	Year-2	1	0	Spring
3/18/2019	FP	8A	3	2019	Year-2	0	0	Spring
3/18/2019	FP	9A	3	2019	Year-2	0	0	Spring
3/18/2019	FP	11A	3	2019	Year-2	1	0	Spring
3/18/2019	FP	13A	3	2019	Year-2	1	0	Spring
3/19/2019	FP	1M	3	2019	Year-2	1	0	Spring
3/19/2019	FP	2M	3	2019	Year-2	1	0	Spring
3/19/2019	FP	3M	3	2019	Year-2	1	0	Spring
3/19/2019	FP	4M	3	2019	Year-2	1	0	Spring
3/19/2019	FP	5M	3	2019	Year-2	0	0	Spring
3/19/2019	FP	6M	3	2019	Year-2	0	0	Spring
3/19/2019	FP	7M	3	2019	Year-2	1	0	Spring
3/19/2019	FP	10M	3	2019	Year-2	1	0	Spring
3/19/2019	FP	12M	3	2019	Year-2	1	0	Spring
3/19/2019	FP	14M	3	2019	Year-2	1	0	Spring
3/19/2019	FP	8A	3	2019	Year-2	0	0	Spring
3/19/2019	FP	9A	3	2019	Year-2	1	0	Spring
3/19/2019	FP	11A	3	2019	Year-2	1	0	Spring
3/19/2019	FP	13A	3	2019	Year-2	1	0	Spring
3/20/2019	FP	1M	3	2019	Year-2	1	0	Spring
3/20/2019	FP	2M	3	2019	Year-2	1	0	Spring
3/20/2019	FP	3M	3	2019	Year-2	1	0	Spring
3/20/2019	FP	4M	3	2019	Year-2	1	0	Spring
3/20/2019	FP	5M	3	2019	Year-2	1	0	Spring
3/20/2019	FP	6M	3	2019	Year-2	1	0	Spring
3/20/2019	FP	7M	3	2019	Year-2	0	0	Spring
3/20/2019	FP	10M	3	2019	Year-2	1	0	Spring
3/20/2019	FP	12M	3	2019	Year-2	1	0	Spring
3/20/2019	FP	14M	3	2019	Year-2	1	0	Spring
3/20/2019	FP	8A	3	2019	Year-2	1	0	Spring
3/20/2019	FP	9A	3	2019	Year-2	0	0	Spring
3/20/2019	FP	11A	3	2019	Year-2	1	0	Spring
3/20/2019	FP	13A	3	2019	Year-2	1	0	Spring
3/21/2019	FP	1M	3	2019	Year-2	1	0	Spring
3/21/2019	FP	2M	3	2019	Year-2	1	0	Spring
3/21/2019	FP	3M	3	2019	Year-2	1	0	Spring
3/21/2019	FP	4M	3	2019	Year-2	1	0	Spring
3/21/2019	FP	5M	3	2019	Year-2	0	0	Spring
3/21/2019	FP	6M	3	2019	Year-2	1	0	Spring
3/21/2019	FP	7M	3	2019	Year-2	0	0	Spring
3/21/2019	FP	10M	3	2019	Year-2	1	0	Spring
3/21/2019	FP	12M	3	2019	Year-2	1	0	Spring
3/21/2019	FP	14M	3	2019	Year-2	1	0	Spring
3/21/2019	FP	8A	3	2019	Year-2	0	0	Spring

Date	Species	Site	Month	Year	Survey Year	Presence	Data Gap	Season
3/21/2019	FP	9A	3	2019	Year-2	0	0	Spring
3/21/2019	FP	11A	3	2019	Year-2	1	0	Spring
3/21/2019	FP	13A	3	2019	Year-2	1	0	Spring
3/22/2019	FP	1M	3	2019	Year-2	1	0	Spring
3/22/2019	FP	2M	3	2019	Year-2	1	0	Spring
3/22/2019	FP	3M	3	2019	Year-2	1	0	Spring
3/22/2019	FP	4M	3	2019	Year-2	1	0	Spring
3/22/2019	FP	5M	3	2019	Year-2	1	0	Spring
3/22/2019	FP	6M	3	2019	Year-2	0	0	Spring
3/22/2019	FP	7M	3	2019	Year-2	0	0	Spring
3/22/2019	FP	10M	3	2019	Year-2	1	0	Spring
3/22/2019	FP	12M	3	2019	Year-2	1	0	Spring
3/22/2019	FP	14M	3	2019	Year-2	1	0	Spring
3/22/2019	FP	8A	3	2019	Year-2	0	0	Spring
3/22/2019	FP	9A	3	2019	Year-2	0	0	Spring
3/22/2019	FP	11A	3	2019	Year-2	1	0	Spring
3/22/2019	FP	13A	3	2019	Year-2	1	0	Spring
3/23/2019	FP	1M	3	2019	Year-2	1	0	Spring
3/23/2019	FP	2M	3	2019	Year-2	1	0	Spring
3/23/2019	FP	3M	3	2019	Year-2	1	0	Spring
3/23/2019	FP	4M	3	2019	Year-2	1	0	Spring
3/23/2019	FP	5M	3	2019	Year-2	1	0	Spring
3/23/2019	FP	6M	3	2019	Year-2	1	0	Spring
3/23/2019	FP	7M	3	2019	Year-2	0	0	Spring
3/23/2019	FP	10M	3	2019	Year-2	1	0	Spring
3/23/2019	FP	12M	3	2019	Year-2	1	0	Spring
3/23/2019	FP	14M	3	2019	Year-2	1	0	Spring
3/23/2019	FP	8A	3	2019	Year-2	0	0	Spring
3/23/2019	FP	9A	3	2019	Year-2	0	0	Spring
3/23/2019	FP	11A	3	2019	Year-2	1	0	Spring
3/23/2019	FP	13A	3	2019	Year-2	1	0	Spring
3/24/2019	FP	1M	3	2019	Year-2	1	0	Spring
3/24/2019	FP	2M	3	2019	Year-2	1	0	Spring
3/24/2019	FP	3M	3	2019	Year-2	1	0	Spring
3/24/2019	FP	4M	3	2019	Year-2	1	0	Spring
3/24/2019	FP	5M	3	2019	Year-2	1	0	Spring
3/24/2019	FP	6M	3	2019	Year-2	1	0	Spring
3/24/2019	FP	7M	3	2019	Year-2	0	0	Spring
3/24/2019	FP	10M	3	2019	Year-2	1	0	Spring
3/24/2019	FP	12M	3	2019	Year-2	1	0	Spring
3/24/2019	FP	14M	3	2019	Year-2	1	0	Spring
3/24/2019	FP	8A	3	2019	Year-2	0	0	Spring
3/24/2019	FP	9A	3	2019	Year-2	0	0	Spring
3/24/2019	FP	11A	3	2019	Year-2	1	0	Spring

Date	Species	Site	Month	Year	Survey Year	Presence	Data Gap	Season
3/24/2019	FP	13A	3	2019	Year-2	1	0	Spring
3/25/2019	FP	1M	3	2019	Year-2	1	0	Spring
3/25/2019	FP	2M	3	2019	Year-2	1	0	Spring
3/25/2019	FP	3M	3	2019	Year-2	1	0	Spring
3/25/2019	FP	4M	3	2019	Year-2	1	0	Spring
3/25/2019	FP	5M	3	2019	Year-2	1	0	Spring
3/25/2019	FP	6M	3	2019	Year-2	0	0	Spring
3/25/2019	FP	7M	3	2019	Year-2	1	0	Spring
3/25/2019	FP	10M	3	2019	Year-2	1	0	Spring
3/25/2019	FP	12M	3	2019	Year-2	1	0	Spring
3/25/2019	FP	14M	3	2019	Year-2	1	0	Spring
3/25/2019	FP	8A	3	2019	Year-2	1	0	Spring
3/25/2019	FP	9A	3	2019	Year-2	0	0	Spring
3/25/2019	FP	11A	3	2019	Year-2	1	0	Spring
3/25/2019	FP	13A	3	2019	Year-2	1	0	Spring
3/26/2019	FP	1M	3	2019	Year-2	1	0	Spring
3/26/2019	FP	2M	3	2019	Year-2	1	0	Spring
3/26/2019	FP	3M	3	2019	Year-2	1	0	Spring
3/26/2019	FP	4M	3	2019	Year-2	1	0	Spring
3/26/2019	FP	5M	3	2019	Year-2	0	0	Spring
3/26/2019	FP	6M	3	2019	Year-2	1	0	Spring
3/26/2019	FP	7M	3	2019	Year-2	1	0	Spring
3/26/2019	FP	10M	3	2019	Year-2	1	0	Spring
3/26/2019	FP	12M	3	2019	Year-2	1	0	Spring
3/26/2019	FP	14M	3	2019	Year-2	1	0	Spring
3/26/2019	FP	8A	3	2019	Year-2	0	0	Spring
3/26/2019	FP	9A	3	2019	Year-2	1	0	Spring
3/26/2019	FP	11A	3	2019	Year-2	1	0	Spring
3/26/2019	FP	13A	3	2019	Year-2	1	0	Spring
3/27/2019	FP	1M	3	2019	Year-2	1	0	Spring
3/27/2019	FP	2M	3	2019	Year-2	1	0	Spring
3/27/2019	FP	3M	3	2019	Year-2	1	0	Spring
3/27/2019	FP	4M	3	2019	Year-2	1	0	Spring
3/27/2019	FP	5M	3	2019	Year-2	0	0	Spring
3/27/2019	FP	6M	3	2019	Year-2	1	0	Spring
3/27/2019	FP	7M	3	2019	Year-2	0	0	Spring
3/27/2019	FP	10M	3	2019	Year-2	1	0	Spring
3/27/2019	FP	12M	3	2019	Year-2	1	0	Spring
3/27/2019	FP	14M	3	2019	Year-2	1	0	Spring
3/27/2019	FP	8A	3	2019	Year-2	0	0	Spring
3/27/2019	FP	9A	3	2019	Year-2	0	0	Spring
3/27/2019	FP	11A	3	2019	Year-2	1	0	Spring
3/27/2019	FP	13A	3	2019	Year-2	1	0	Spring
3/28/2019	FP	1M	3	2019	Year-2	1	0	Spring

Date	Species	Site	Month	Year	Survey Year	Presence	Data Gap	Season
3/28/2019	FP	2M	3	2019	Year-2	1	0	Spring
3/28/2019	FP	3M	3	2019	Year-2	1	0	Spring
3/28/2019	FP	4M	3	2019	Year-2	0	0	Spring
3/28/2019	FP	5M	3	2019	Year-2	1	0	Spring
3/28/2019	FP	6M	3	2019	Year-2	1	0	Spring
3/28/2019	FP	7M	3	2019	Year-2	0	0	Spring
3/28/2019	FP	10M	3	2019	Year-2	1	0	Spring
3/28/2019	FP	12M	3	2019	Year-2	1	0	Spring
3/28/2019	FP	14M	3	2019	Year-2	1	0	Spring
3/28/2019	FP	8A	3	2019	Year-2	0	0	Spring
3/28/2019	FP	9A	3	2019	Year-2	0	0	Spring
3/28/2019	FP	11A	3	2019	Year-2	1	0	Spring
3/28/2019	FP	13A	3	2019	Year-2	1	0	Spring
3/29/2019	FP	1M	3	2019	Year-2	1	0	Spring
3/29/2019	FP	2M	3	2019	Year-2	1	0	Spring
3/29/2019	FP	3M	3	2019	Year-2	1	0	Spring
3/29/2019	FP	4M	3	2019	Year-2	1	0	Spring
3/29/2019	FP	5M	3	2019	Year-2	1	0	Spring
3/29/2019	FP	6M	3	2019	Year-2	1	0	Spring
3/29/2019	FP	7M	3	2019	Year-2	0	0	Spring
3/29/2019	FP	10M	3	2019	Year-2	1	0	Spring
3/29/2019	FP	12M	3	2019	Year-2	1	0	Spring
3/29/2019	FP	14M	3	2019	Year-2	1	0	Spring
3/29/2019	FP	8A	3	2019	Year-2	0	0	Spring
3/29/2019	FP	9A	3	2019	Year-2	0	0	Spring
3/29/2019	FP	11A	3	2019	Year-2	1	0	Spring
3/29/2019	FP	13A	3	2019	Year-2	1	0	Spring
3/30/2019	FP	1M	3	2019	Year-2	1	0	Spring
3/30/2019	FP	2M	3	2019	Year-2	1	0	Spring
3/30/2019	FP	3M	3	2019	Year-2	1	0	Spring
3/30/2019	FP	4M	3	2019	Year-2	1	0	Spring
3/30/2019	FP	5M	3	2019	Year-2	1	0	Spring
3/30/2019	FP	6M	3	2019	Year-2	0	0	Spring
3/30/2019	FP	7M	3	2019	Year-2	0	0	Spring
3/30/2019	FP	10M	3	2019	Year-2	1	0	Spring
3/30/2019	FP	12M	3	2019	Year-2	1	0	Spring
3/30/2019	FP	14M	3	2019	Year-2	1	0	Spring
3/30/2019	FP	8A	3	2019	Year-2	0	0	Spring
3/30/2019	FP	9A	3	2019	Year-2	0	0	Spring
3/30/2019	FP	11A	3	2019	Year-2	1	0	Spring
3/30/2019	FP	13A	3	2019	Year-2	1	0	Spring
3/31/2019	FP	1M	3	2019	Year-2	0	0	Spring
3/31/2019	FP	2M	3	2019	Year-2	0	0	Spring
3/31/2019	FP	3M	3	2019	Year-2	0	0	Spring

Date	Species	Site	Month	Year	Survey Year	Presence	Data Gap	Season
3/31/2019	FP	4M	3	2019	Year-2	0	0	Spring
3/31/2019	FP	5M	3	2019	Year-2	1	0	Spring
3/31/2019	FP	6M	3	2019	Year-2	0	0	Spring
3/31/2019	FP	7M	3	2019	Year-2	1	0	Spring
3/31/2019	FP	10M	3	2019	Year-2	1	0	Spring
3/31/2019	FP	12M	3	2019	Year-2	0	0	Spring
3/31/2019	FP	14M	3	2019	Year-2	0	0	Spring
3/31/2019	FP	8A	3	2019	Year-2	1	0	Spring
3/31/2019	FP	9A	3	2019	Year-2	0	0	Spring
3/31/2019	FP	11A	3	2019	Year-2	1	0	Spring
3/31/2019	FP	13A	3	2019	Year-2	1	0	Spring
4/1/2019	FP	1M	4	2019	Year-2	1	0	Spring
4/1/2019	FP	2M	4	2019	Year-2	1	0	Spring
4/1/2019	FP	3M	4	2019	Year-2	1	0	Spring
4/1/2019	FP	4M	4	2019	Year-2	1	0	Spring
4/1/2019	FP	5M	4	2019	Year-2	0	0	Spring
4/1/2019	FP	6M	4	2019	Year-2	0	0	Spring
4/1/2019	FP	7M	4	2019	Year-2	1	0	Spring
4/1/2019	FP	10M	4	2019	Year-2	1	0	Spring
4/1/2019	FP	12M	4	2019	Year-2	1	0	Spring
4/1/2019	FP	14M	4	2019	Year-2	1	0	Spring
4/1/2019	FP	8A	4	2019	Year-2	0	0	Spring
4/1/2019	FP	9A	4	2019	Year-2	0	0	Spring
4/1/2019	FP	11A	4	2019	Year-2	1	0	Spring
4/1/2019	FP	13A	4	2019	Year-2	1	0	Spring
4/2/2019	FP	1M	4	2019	Year-2	0	0	Spring
4/2/2019	FP	2M	4	2019	Year-2	0	0	Spring
4/2/2019	FP	3M	4	2019	Year-2	0	0	Spring
4/2/2019	FP	4M	4	2019	Year-2	0	0	Spring
4/2/2019	FP	5M	4	2019	Year-2	0	0	Spring
4/2/2019	FP	6M	4	2019	Year-2	1	0	Spring
4/2/2019	FP	7M	4	2019	Year-2	0	0	Spring
4/2/2019	FP	10M	4	2019	Year-2	0	0	Spring
4/2/2019	FP	12M	4	2019	Year-2	0	0	Spring
4/2/2019	FP	14M	4	2019	Year-2	0	0	Spring
4/2/2019	FP	8A	4	2019	Year-2	0	0	Spring
4/2/2019	FP	9A	4	2019	Year-2	0	0	Spring
4/2/2019	FP	11A	4	2019	Year-2	1	0	Spring
4/2/2019	FP	13A	4	2019	Year-2	1	0	Spring
4/3/2019	FP	1M	4	2019	Year-2	0	0	Spring
4/3/2019	FP	2M	4	2019	Year-2	1	0	Spring
4/3/2019	FP	3M	4	2019	Year-2	1	0	Spring
4/3/2019	FP	4M	4	2019	Year-2	1	0	Spring
4/3/2019	FP	5M	4	2019	Year-2	1	0	Spring

Date	Species	Site	Month	Year	Survey Year	Presence	Data Gap	Season
4/3/2019	FP	6M	4	2019	Year-2	1	0	Spring
4/3/2019	FP	7M	4	2019	Year-2	0	0	Spring
4/3/2019	FP	10M	4	2019	Year-2	1	0	Spring
4/3/2019	FP	12M	4	2019	Year-2	0	0	Spring
4/3/2019	FP	14M	4	2019	Year-2	0	0	Spring
4/3/2019	FP	8A	4	2019	Year-2	0	0	Spring
4/3/2019	FP	9A	4	2019	Year-2	0	0	Spring
4/3/2019	FP	11A	4	2019	Year-2	1	0	Spring
4/3/2019	FP	13A	4	2019	Year-2	1	0	Spring
4/4/2019	FP	1M	4	2019	Year-2	1	0	Spring
4/4/2019	FP	2M	4	2019	Year-2	1	0	Spring
4/4/2019	FP	3M	4	2019	Year-2	1	0	Spring
4/4/2019	FP	4M	4	2019	Year-2	1	0	Spring
4/4/2019	FP	5M	4	2019	Year-2	0	0	Spring
4/4/2019	FP	6M	4	2019	Year-2	0	0	Spring
4/4/2019	FP	7M	4	2019	Year-2	0	0	Spring
4/4/2019	FP	10M	4	2019	Year-2	0	0	Spring
4/4/2019	FP	12M	4	2019	Year-2	0	0	Spring
4/4/2019	FP	14M	4	2019	Year-2	1	0	Spring
4/4/2019	FP	8A	4	2019	Year-2	1	0	Spring
4/4/2019	FP	9A	4	2019	Year-2	0	0	Spring
4/4/2019	FP	11A	4	2019	Year-2	1	0	Spring
4/4/2019	FP	13A	4	2019	Year-2	1	0	Spring
4/5/2019	FP	1M	4	2019	Year-2	1	0	Spring
4/5/2019	FP	2M	4	2019	Year-2	1	0	Spring
4/5/2019	FP	3M	4	2019	Year-2	1	0	Spring
4/5/2019	FP	4M	4	2019	Year-2	1	0	Spring
4/5/2019	FP	5M	4	2019	Year-2	0	0	Spring
4/5/2019	FP	6M	4	2019	Year-2	0	0	Spring
4/5/2019	FP	7M	4	2019	Year-2	0	0	Spring
4/5/2019	FP	10M	4	2019	Year-2	1	0	Spring
4/5/2019	FP	12M	4	2019	Year-2	0	0	Spring
4/5/2019	FP	14M	4	2019	Year-2	1	0	Spring
4/5/2019	FP	8A	4	2019	Year-2	1	0	Spring
4/5/2019	FP	9A	4	2019	Year-2	1	0	Spring
4/5/2019	FP	11A	4	2019	Year-2	0	0	Spring
4/5/2019	FP	13A	4	2019	Year-2	1	0	Spring
4/6/2019	FP	1M	4	2019	Year-2	1	0	Spring
4/6/2019	FP	2M	4	2019	Year-2	1	0	Spring
4/6/2019	FP	3M	4	2019	Year-2	0	0	Spring
4/6/2019	FP	4M	4	2019	Year-2	0	0	Spring
4/6/2019	FP	5M	4	2019	Year-2	1	0	Spring
4/6/2019	FP	6M	4	2019	Year-2	0	0	Spring
4/6/2019	FP	7M	4	2019	Year-2	0	0	Spring

Date	Species	Site	Month	Year	Survey Year	Presence	Data Gap	Season
4/6/2019	FP	10M	4	2019	Year-2	1	0	Spring
4/6/2019	FP	12M	4	2019	Year-2	0	0	Spring
4/6/2019	FP	14M	4	2019	Year-2	1	0	Spring
4/6/2019	FP	8A	4	2019	Year-2	1	0	Spring
4/6/2019	FP	9A	4	2019	Year-2	1	0	Spring
4/6/2019	FP	11A	4	2019	Year-2	0	0	Spring
4/6/2019	FP	13A	4	2019	Year-2	1	0	Spring
4/7/2019	FP	1M	4	2019	Year-2	1	0	Spring
4/7/2019	FP	2M	4	2019	Year-2	1	0	Spring
4/7/2019	FP	3M	4	2019	Year-2	1	0	Spring
4/7/2019	FP	4M	4	2019	Year-2	1	0	Spring
4/7/2019	FP	5M	4	2019	Year-2	0	0	Spring
4/7/2019	FP	6M	4	2019	Year-2	0	0	Spring
4/7/2019	FP	7M	4	2019	Year-2	1	0	Spring
4/7/2019	FP	10M	4	2019	Year-2	0	0	Spring
4/7/2019	FP	12M	4	2019	Year-2	0	0	Spring
4/7/2019	FP	14M	4	2019	Year-2	1	0	Spring
4/7/2019	FP	8A	4	2019	Year-2	1	0	Spring
4/7/2019	FP	9A	4	2019	Year-2	0	0	Spring
4/7/2019	FP	11A	4	2019	Year-2	0	0	Spring
4/7/2019	FP	13A	4	2019	Year-2	1	0	Spring
4/8/2019	FP	1M	4	2019	Year-2	1	0	Spring
4/8/2019	FP	2M	4	2019	Year-2	1	0	Spring
4/8/2019	FP	3M	4	2019	Year-2	1	0	Spring
4/8/2019	FP	4M	4	2019	Year-2	1	0	Spring
4/8/2019	FP	5M	4	2019	Year-2	1	0	Spring
4/8/2019	FP	6M	4	2019	Year-2	0	0	Spring
4/8/2019	FP	7M	4	2019	Year-2	1	0	Spring
4/8/2019	FP	10M	4	2019	Year-2	0	0	Spring
4/8/2019	FP	12M	4	2019	Year-2	0	0	Spring
4/8/2019	FP	14M	4	2019	Year-2	1	0	Spring
4/8/2019	FP	8A	4	2019	Year-2	0	0	Spring
4/8/2019	FP	9A	4	2019	Year-2	1	0	Spring
4/8/2019	FP	11A	4	2019	Year-2	0	0	Spring
4/8/2019	FP	13A	4	2019	Year-2	1	0	Spring
4/9/2019	FP	1M	4	2019	Year-2	1	0	Spring
4/9/2019	FP	2M	4	2019	Year-2	1	0	Spring
4/9/2019	FP	3M	4	2019	Year-2	1	0	Spring
4/9/2019	FP	4M	4	2019	Year-2	1	0	Spring
4/9/2019	FP	5M	4	2019	Year-2	1	0	Spring
4/9/2019	FP	6M	4	2019	Year-2	1	0	Spring
4/9/2019	FP	7M	4	2019	Year-2	0	0	Spring
4/9/2019	FP	10M	4	2019	Year-2	1	0	Spring
4/9/2019	FP	12M	4	2019	Year-2	0	0	Spring

Date	Species	Site	Month	Year	Survey Year	Presence	Data Gap	Season
4/9/2019	FP	14M	4	2019	Year-2	1	0	Spring
4/9/2019	FP	8A	4	2019	Year-2	1	0	Spring
4/9/2019	FP	9A	4	2019	Year-2	1	0	Spring
4/9/2019	FP	11A	4	2019	Year-2	1	0	Spring
4/9/2019	FP	13A	4	2019	Year-2	1	0	Spring
4/10/2019	FP	1M	4	2019	Year-2	1	0	Spring
4/10/2019	FP	2M	4	2019	Year-2	1	0	Spring
4/10/2019	FP	3M	4	2019	Year-2	1	0	Spring
4/10/2019	FP	4M	4	2019	Year-2	1	0	Spring
4/10/2019	FP	5M	4	2019	Year-2	0	0	Spring
4/10/2019	FP	6M	4	2019	Year-2		1	Spring
4/10/2019	FP	7M	4	2019	Year-2		1	Spring
4/10/2019	FP	10M	4	2019	Year-2	1	0	Spring
4/10/2019	FP	12M	4	2019	Year-2	1	0	Spring
4/10/2019	FP	14M	4	2019	Year-2	1	0	Spring
4/10/2019	FP	8A	4	2019	Year-2	1	0	Spring
4/10/2019	FP	9A	4	2019	Year-2	1	0	Spring
4/10/2019	FP	11A	4	2019	Year-2	1	0	Spring
4/10/2019	FP	13A	4	2019	Year-2	1	0	Spring
4/11/2019	FP	1M	4	2019	Year-2		1	Spring
4/11/2019	FP	2M	4	2019	Year-2		1	Spring
4/11/2019	FP	3M	4	2019	Year-2		1	Spring
4/11/2019	FP	4M	4	2019	Year-2	0	0	Spring
4/11/2019	FP	5M	4	2019	Year-2	0	0	Spring
4/11/2019	FP	6M	4	2019	Year-2		1	Spring
4/11/2019	FP	7M	4	2019	Year-2	0	0	Spring
4/11/2019	FP	10M	4	2019	Year-2	1	0	Spring
4/11/2019	FP	12M	4	2019	Year-2	1	0	Spring
4/11/2019	FP	14M	4	2019	Year-2	1	0	Spring
4/11/2019	FP	8A	4	2019	Year-2	1	0	Spring
4/11/2019	FP	9A	4	2019	Year-2	1	0	Spring
4/11/2019	FP	11A	4	2019	Year-2	1	0	Spring
4/11/2019	FP	13A	4	2019	Year-2	1	0	Spring
4/12/2019	FP	1M	4	2019	Year-2	1	0	Spring
4/12/2019	FP	2M	4	2019	Year-2	0	0	Spring
4/12/2019	FP	3M	4	2019	Year-2	0	0	Spring
4/12/2019	FP	4M	4	2019	Year-2	1	0	Spring
4/12/2019	FP	5M	4	2019	Year-2	0	0	Spring
4/12/2019	FP	6M	4	2019	Year-2		1	Spring
4/12/2019	FP	7M	4	2019	Year-2	0	0	Spring
4/12/2019	FP	10M	4	2019	Year-2	1	0	Spring
4/12/2019	FP	12M	4	2019	Year-2	1	0	Spring
4/12/2019	FP	14M	4	2019	Year-2	1	0	Spring
4/12/2019	FP	8A	4	2019	Year-2	1	0	Spring

Date	Species	Site	Month	Year	Survey Year	Presence	Data Gap	Season
4/12/2019	FP	9A	4	2019	Year-2	1	0	Spring
4/12/2019	FP	11A	4	2019	Year-2	1	0	Spring
4/12/2019	FP	13A	4	2019	Year-2	1	0	Spring
4/13/2019	FP	1M	4	2019	Year-2	1	0	Spring
4/13/2019	FP	2M	4	2019	Year-2	1	0	Spring
4/13/2019	FP	3M	4	2019	Year-2	1	0	Spring
4/13/2019	FP	4M	4	2019	Year-2	1	0	Spring
4/13/2019	FP	5M	4	2019	Year-2	1	0	Spring
4/13/2019	FP	6M	4	2019	Year-2		1	Spring
4/13/2019	FP	7M	4	2019	Year-2	0	0	Spring
4/13/2019	FP	10M	4	2019	Year-2	1	0	Spring
4/13/2019	FP	12M	4	2019	Year-2	1	0	Spring
4/13/2019	FP	14M	4	2019	Year-2	1	0	Spring
4/13/2019	FP	8A	4	2019	Year-2	1	0	Spring
4/13/2019	FP	9A	4	2019	Year-2	1	0	Spring
4/13/2019	FP	11A	4	2019	Year-2	1	0	Spring
4/13/2019	FP	13A	4	2019	Year-2	1	0	Spring
4/14/2019	FP	1M	4	2019	Year-2	1	0	Spring
4/14/2019	FP	2M	4	2019	Year-2	1	0	Spring
4/14/2019	FP	3M	4	2019	Year-2	1	0	Spring
4/14/2019	FP	4M	4	2019	Year-2	1	0	Spring
4/14/2019	FP	5M	4	2019	Year-2	0	0	Spring
4/14/2019	FP	6M	4	2019	Year-2		1	Spring
4/14/2019	FP	7M	4	2019	Year-2	1	0	Spring
4/14/2019	FP	10M	4	2019	Year-2	1	0	Spring
4/14/2019	FP	12M	4	2019	Year-2	1	0	Spring
4/14/2019	FP	14M	4	2019	Year-2	1	0	Spring
4/14/2019	FP	8A	4	2019	Year-2	1	0	Spring
4/14/2019	FP	9A	4	2019	Year-2	0	0	Spring
4/14/2019	FP	11A	4	2019	Year-2	1	0	Spring
4/14/2019	FP	13A	4	2019	Year-2	1	0	Spring
4/15/2019	FP	1M	4	2019	Year-2	1	0	Spring
4/15/2019	FP	2M	4	2019	Year-2	1	0	Spring
4/15/2019	FP	3M	4	2019	Year-2	1	0	Spring
4/15/2019	FP	4M	4	2019	Year-2	1	0	Spring
4/15/2019	FP	5M	4	2019	Year-2	0	0	Spring
4/15/2019	FP	6M	4	2019	Year-2		1	Spring
4/15/2019	FP	7M	4	2019	Year-2	0	0	Spring
4/15/2019	FP	10M	4	2019	Year-2	1	0	Spring
4/15/2019	FP	12M	4	2019	Year-2	1	0	Spring
4/15/2019	FP	14M	4	2019	Year-2	1	0	Spring
4/15/2019	FP	8A	4	2019	Year-2	1	0	Spring
4/15/2019	FP	9A	4	2019	Year-2	0	0	Spring
4/15/2019	FP	11A	4	2019	Year-2	1	0	Spring

Date	Species	Site	Month	Year	Survey Year	Presence	Data Gap	Season
4/15/2019	FP	13A	4	2019	Year-2	1	0	Spring
4/16/2019	FP	1M	4	2019	Year-2	1	0	Spring
4/16/2019	FP	2M	4	2019	Year-2	1	0	Spring
4/16/2019	FP	3M	4	2019	Year-2	0	0	Spring
4/16/2019	FP	4M	4	2019	Year-2	1	0	Spring
4/16/2019	FP	5M	4	2019	Year-2	1	0	Spring
4/16/2019	FP	6M	4	2019	Year-2		1	Spring
4/16/2019	FP	7M	4	2019	Year-2	1	0	Spring
4/16/2019	FP	10M	4	2019	Year-2	1	0	Spring
4/16/2019	FP	12M	4	2019	Year-2	1	0	Spring
4/16/2019	FP	14M	4	2019	Year-2	1	0	Spring
4/16/2019	FP	8A	4	2019	Year-2	0	0	Spring
4/16/2019	FP	9A	4	2019	Year-2	0	0	Spring
4/16/2019	FP	11A	4	2019	Year-2	1	0	Spring
4/16/2019	FP	13A	4	2019	Year-2	1	0	Spring
4/17/2019	FP	1M	4	2019	Year-2	1	0	Spring
4/17/2019	FP	2M	4	2019	Year-2	1	0	Spring
4/17/2019	FP	3M	4	2019	Year-2	1	0	Spring
4/17/2019	FP	4M	4	2019	Year-2	1	0	Spring
4/17/2019	FP	5M	4	2019	Year-2	0	0	Spring
4/17/2019	FP	6M	4	2019	Year-2		1	Spring
4/17/2019	FP	7M	4	2019	Year-2	0	0	Spring
4/17/2019	FP	10M	4	2019	Year-2	1	0	Spring
4/17/2019	FP	12M	4	2019	Year-2	1	0	Spring
4/17/2019	FP	14M	4	2019	Year-2	1	0	Spring
4/17/2019	FP	8A	4	2019	Year-2	0	0	Spring
4/17/2019	FP	9A	4	2019	Year-2	0	0	Spring
4/17/2019	FP	11A	4	2019	Year-2	1	0	Spring
4/17/2019	FP	13A	4	2019	Year-2	1	0	Spring
4/18/2019	FP	1M	4	2019	Year-2	1	0	Spring
4/18/2019	FP	2M	4	2019	Year-2	1	0	Spring
4/18/2019	FP	3M	4	2019	Year-2	1	0	Spring
4/18/2019	FP	4M	4	2019	Year-2	1	0	Spring
4/18/2019	FP	5M	4	2019	Year-2	1	0	Spring
4/18/2019	FP	6M	4	2019	Year-2		1	Spring
4/18/2019	FP	7M	4	2019	Year-2	0	0	Spring
4/18/2019	FP	10M	4	2019	Year-2	1	0	Spring
4/18/2019	FP	12M	4	2019	Year-2	1	0	Spring
4/18/2019	FP	14M	4	2019	Year-2	1	0	Spring
4/18/2019	FP	8A	4	2019	Year-2	1	0	Spring
4/18/2019	FP	9A	4	2019	Year-2	0	0	Spring
4/18/2019	FP	11A	4	2019	Year-2	1	0	Spring
4/18/2019	FP	13A	4	2019	Year-2	1	0	Spring
4/19/2019	FP	1M	4	2019	Year-2	0	0	Spring

Date	Species	Site	Month	Year	Survey Year	Presence	Data Gap	Season
4/19/2019	FP	2M	4	2019	Year-2	1	0	Spring
4/19/2019	FP	3M	4	2019	Year-2	1	0	Spring
4/19/2019	FP	4M	4	2019	Year-2	1	0	Spring
4/19/2019	FP	5M	4	2019	Year-2	0	0	Spring
4/19/2019	FP	6M	4	2019	Year-2		1	Spring
4/19/2019	FP	7M	4	2019	Year-2	1	0	Spring
4/19/2019	FP	10M	4	2019	Year-2	1	0	Spring
4/19/2019	FP	12M	4	2019	Year-2	1	0	Spring
4/19/2019	FP	14M	4	2019	Year-2	1	0	Spring
4/19/2019	FP	8A	4	2019	Year-2	1	0	Spring
4/19/2019	FP	9A	4	2019	Year-2	1	0	Spring
4/19/2019	FP	11A	4	2019	Year-2	1	0	Spring
4/19/2019	FP	13A	4	2019	Year-2	1	0	Spring
4/20/2019	FP	1M	4	2019	Year-2	1	0	Spring
4/20/2019	FP	2M	4	2019	Year-2	1	0	Spring
4/20/2019	FP	3M	4	2019	Year-2	1	0	Spring
4/20/2019	FP	4M	4	2019	Year-2	1	0	Spring
4/20/2019	FP	5M	4	2019	Year-2	0	0	Spring
4/20/2019	FP	6M	4	2019	Year-2		1	Spring
4/20/2019	FP	7M	4	2019	Year-2	0	0	Spring
4/20/2019	FP	10M	4	2019	Year-2	1	0	Spring
4/20/2019	FP	12M	4	2019	Year-2	1	0	Spring
4/20/2019	FP	14M	4	2019	Year-2	1	0	Spring
4/20/2019	FP	8A	4	2019	Year-2	0	0	Spring
4/20/2019	FP	9A	4	2019	Year-2	0	0	Spring
4/20/2019	FP	11A	4	2019	Year-2	1	0	Spring
4/20/2019	FP	13A	4	2019	Year-2	1	0	Spring
4/21/2019	FP	1M	4	2019	Year-2	1	0	Spring
4/21/2019	FP	2M	4	2019	Year-2	1	0	Spring
4/21/2019	FP	3M	4	2019	Year-2	1	0	Spring
4/21/2019	FP	4M	4	2019	Year-2	0	0	Spring
4/21/2019	FP	5M	4	2019	Year-2	1	0	Spring
4/21/2019	FP	6M	4	2019	Year-2		1	Spring
4/21/2019	FP	7M	4	2019	Year-2	0	0	Spring
4/21/2019	FP	10M	4	2019	Year-2	0	0	Spring
4/21/2019	FP	12M	4	2019	Year-2	1	0	Spring
4/21/2019	FP	14M	4	2019	Year-2	0	0	Spring
4/21/2019	FP	8A	4	2019	Year-2	0	0	Spring
4/21/2019	FP	9A	4	2019	Year-2	0	0	Spring
4/21/2019	FP	11A	4	2019	Year-2	1	0	Spring
4/21/2019	FP	13A	4	2019	Year-2	1	0	Spring
4/22/2019	FP	1M	4	2019	Year-2	1	0	Spring
4/22/2019	FP	2M	4	2019	Year-2	1	0	Spring
4/22/2019	FP	3M	4	2019	Year-2	1	0	Spring

Date	Species	Site	Month	Year	Survey Year	Presence	Data Gap	Season
4/22/2019	FP	4M	4	2019	Year-2	0	0	Spring
4/22/2019	FP	5M	4	2019	Year-2	1	0	Spring
4/22/2019	FP	6M	4	2019	Year-2		1	Spring
4/22/2019	FP	7M	4	2019	Year-2	0	0	Spring
4/22/2019	FP	10M	4	2019	Year-2	0	0	Spring
4/22/2019	FP	12M	4	2019	Year-2	1	0	Spring
4/22/2019	FP	14M	4	2019	Year-2	1	0	Spring
4/22/2019	FP	8A	4	2019	Year-2	0	0	Spring
4/22/2019	FP	9A	4	2019	Year-2	0	0	Spring
4/22/2019	FP	11A	4	2019	Year-2	1	0	Spring
4/22/2019	FP	13A	4	2019	Year-2	1	0	Spring
4/23/2019	FP	1M	4	2019	Year-2	0	0	Spring
4/23/2019	FP	2M	4	2019	Year-2	0	0	Spring
4/23/2019	FP	3M	4	2019	Year-2	0	0	Spring
4/23/2019	FP	4M	4	2019	Year-2	0	0	Spring
4/23/2019	FP	5M	4	2019	Year-2	1	0	Spring
4/23/2019	FP	6M	4	2019	Year-2		1	Spring
4/23/2019	FP	7M	4	2019	Year-2	0	0	Spring
4/23/2019	FP	10M	4	2019	Year-2	0	0	Spring
4/23/2019	FP	12M	4	2019	Year-2	1	0	Spring
4/23/2019	FP	14M	4	2019	Year-2	1	0	Spring
4/23/2019	FP	8A	4	2019	Year-2	0	0	Spring
4/23/2019	FP	9A	4	2019	Year-2	0	0	Spring
4/23/2019	FP	11A	4	2019	Year-2	1	0	Spring
4/23/2019	FP	13A	4	2019	Year-2	1	0	Spring
4/24/2019	FP	1M	4	2019	Year-2	0	0	Spring
4/24/2019	FP	2M	4	2019	Year-2	1	0	Spring
4/24/2019	FP	3M	4	2019	Year-2	1	0	Spring
4/24/2019	FP	4M	4	2019	Year-2	1	0	Spring
4/24/2019	FP	5M	4	2019	Year-2	1	0	Spring
4/24/2019	FP	6M	4	2019	Year-2		1	Spring
4/24/2019	FP	7M	4	2019	Year-2	0	0	Spring
4/24/2019	FP	10M	4	2019	Year-2	0	0	Spring
4/24/2019	FP	12M	4	2019	Year-2	1	0	Spring
4/24/2019	FP	14M	4	2019	Year-2	1	0	Spring
4/24/2019	FP	8A	4	2019	Year-2	1	0	Spring
4/24/2019	FP	9A	4	2019	Year-2	0	0	Spring
4/24/2019	FP	11A	4	2019	Year-2	1	0	Spring
4/24/2019	FP	13A	4	2019	Year-2	1	0	Spring
4/25/2019	FP	1M	4	2019	Year-2	0	0	Spring
4/25/2019	FP	2M	4	2019	Year-2	1	0	Spring
4/25/2019	FP	3M	4	2019	Year-2	1	0	Spring
4/25/2019	FP	4M	4	2019	Year-2	1	0	Spring
4/25/2019	FP	5M	4	2019	Year-2	1	0	Spring

Date	Species	Site	Month	Year	Survey Year	Presence	Data Gap	Season
4/25/2019	FP	6M	4	2019	Year-2		1	Spring
4/25/2019	FP	7M	4	2019	Year-2	0	0	Spring
4/25/2019	FP	10M	4	2019	Year-2		1	Spring
4/25/2019	FP	12M	4	2019	Year-2		1	Spring
4/25/2019	FP	14M	4	2019	Year-2		1	Spring
4/25/2019	FP	8A	4	2019	Year-2	1	0	Spring
4/25/2019	FP	9A	4	2019	Year-2	1	0	Spring
4/25/2019	FP	11A	4	2019	Year-2	1	0	Spring
4/25/2019	FP	13A	4	2019	Year-2	1	0	Spring
4/26/2019	FP	1M	4	2019	Year-2	1	0	Spring
4/26/2019	FP	2M	4	2019	Year-2	1	0	Spring
4/26/2019	FP	3M	4	2019	Year-2	1	0	Spring
4/26/2019	FP	4M	4	2019	Year-2	1	0	Spring
4/26/2019	FP	5M	4	2019	Year-2	1	0	Spring
4/26/2019	FP	6M	4	2019	Year-2		1	Spring
4/26/2019	FP	7M	4	2019	Year-2	0	0	Spring
4/26/2019	FP	10M	4	2019	Year-2	1	0	Spring
4/26/2019	FP	12M	4	2019	Year-2	1	0	Spring
4/26/2019	FP	14M	4	2019	Year-2	1	0	Spring
4/26/2019	FP	8A	4	2019	Year-2	1	0	Spring
4/26/2019	FP	9A	4	2019	Year-2	0	0	Spring
4/26/2019	FP	11A	4	2019	Year-2	1	0	Spring
4/26/2019	FP	13A	4	2019	Year-2	1	0	Spring
4/27/2019	FP	1M	4	2019	Year-2	1	0	Spring
4/27/2019	FP	2M	4	2019	Year-2	1	0	Spring
4/27/2019	FP	3M	4	2019	Year-2	1	0	Spring
4/27/2019	FP	4M	4	2019	Year-2	1	0	Spring
4/27/2019	FP	5M	4	2019	Year-2	1	0	Spring
4/27/2019	FP	6M	4	2019	Year-2		1	Spring
4/27/2019	FP	7M	4	2019	Year-2	0	0	Spring
4/27/2019	FP	10M	4	2019	Year-2	1	0	Spring
4/27/2019	FP	12M	4	2019	Year-2	1	0	Spring
4/27/2019	FP	14M	4	2019	Year-2	1	0	Spring
4/27/2019	FP	8A	4	2019	Year-2	1	0	Spring
4/27/2019	FP	9A	4	2019	Year-2	0	0	Spring
4/27/2019	FP	11A	4	2019	Year-2	1	0	Spring
4/27/2019	FP	13A	4	2019	Year-2	1	0	Spring
4/28/2019	FP	1M	4	2019	Year-2	1	0	Spring
4/28/2019	FP	2M	4	2019	Year-2	1	0	Spring
4/28/2019	FP	3M	4	2019	Year-2	1	0	Spring
4/28/2019	FP	4M	4	2019	Year-2	1	0	Spring
4/28/2019	FP	5M	4	2019	Year-2	1	0	Spring
4/28/2019	FP	6M	4	2019	Year-2		1	Spring
4/28/2019	FP	7M	4	2019	Year-2	0	0	Spring

Date	Species	Site	Month	Year	Survey Year	Presence	Data Gap	Season
4/28/2019	FP	10M	4	2019	Year-2	1	0	Spring
4/28/2019	FP	12M	4	2019	Year-2	1	0	Spring
4/28/2019	FP	14M	4	2019	Year-2	1	0	Spring
4/28/2019	FP	8A	4	2019	Year-2	0	0	Spring
4/28/2019	FP	9A	4	2019	Year-2	1	0	Spring
4/28/2019	FP	11A	4	2019	Year-2	1	0	Spring
4/28/2019	FP	13A	4	2019	Year-2	1	0	Spring
4/29/2019	FP	1M	4	2019	Year-2	1	0	Spring
4/29/2019	FP	2M	4	2019	Year-2	1	0	Spring
4/29/2019	FP	3M	4	2019	Year-2	1	0	Spring
4/29/2019	FP	4M	4	2019	Year-2	1	0	Spring
4/29/2019	FP	5M	4	2019	Year-2	1	0	Spring
4/29/2019	FP	6M	4	2019	Year-2		1	Spring
4/29/2019	FP	7M	4	2019	Year-2	0	0	Spring
4/29/2019	FP	10M	4	2019	Year-2	1	0	Spring
4/29/2019	FP	12M	4	2019	Year-2	1	0	Spring
4/29/2019	FP	14M	4	2019	Year-2	1	0	Spring
4/29/2019	FP	8A	4	2019	Year-2	1	0	Spring
4/29/2019	FP	9A	4	2019	Year-2	0	0	Spring
4/29/2019	FP	11A	4	2019	Year-2	1	0	Spring
4/29/2019	FP	13A	4	2019	Year-2	1	0	Spring
4/30/2019	FP	1M	4	2019	Year-2	1	0	Spring
4/30/2019	FP	2M	4	2019	Year-2	1	0	Spring
4/30/2019	FP	3M	4	2019	Year-2	1	0	Spring
4/30/2019	FP	4M	4	2019	Year-2	1	0	Spring
4/30/2019	FP	5M	4	2019	Year-2	1	0	Spring
4/30/2019	FP	6M	4	2019	Year-2		1	Spring
4/30/2019	FP	7M	4	2019	Year-2	0	0	Spring
4/30/2019	FP	10M	4	2019	Year-2	1	0	Spring
4/30/2019	FP	12M	4	2019	Year-2	1	0	Spring
4/30/2019	FP	14M	4	2019	Year-2	1	0	Spring
4/30/2019	FP	8A	4	2019	Year-2	1	0	Spring
4/30/2019	FP	9A	4	2019	Year-2	0	0	Spring
4/30/2019	FP	11A	4	2019	Year-2	1	0	Spring
4/30/2019	FP	13A	4	2019	Year-2	1	0	Spring
5/1/2019	FP	1M	5	2019	Year-2	1	0	Spring
5/1/2019	FP	2M	5	2019	Year-2	1	0	Spring
5/1/2019	FP	3M	5	2019	Year-2	1	0	Spring
5/1/2019	FP	4M	5	2019	Year-2	1	0	Spring
5/1/2019	FP	5M	5	2019	Year-2	0	0	Spring
5/1/2019	FP	6M	5	2019	Year-2		1	Spring
5/1/2019	FP	7M	5	2019	Year-2	0	0	Spring
5/1/2019	FP	10M	5	2019	Year-2	0	0	Spring
5/1/2019	FP	12M	5	2019	Year-2	1	0	Spring

Date	Species	Site	Month	Year	Survey Year	Presence	Data Gap	Season
5/1/2019	FP	14M	5	2019	Year-2	1	0	Spring
5/1/2019	FP	8A	5	2019	Year-2	1	0	Spring
5/1/2019	FP	9A	5	2019	Year-2	0	0	Spring
5/1/2019	FP	11A	5	2019	Year-2	0	0	Spring
5/1/2019	FP	13A	5	2019	Year-2	1	0	Spring
5/2/2019	FP	1M	5	2019	Year-2	1	0	Spring
5/2/2019	FP	2M	5	2019	Year-2	1	0	Spring
5/2/2019	FP	3M	5	2019	Year-2	1	0	Spring
5/2/2019	FP	4M	5	2019	Year-2	1	0	Spring
5/2/2019	FP	5M	5	2019	Year-2	0	0	Spring
5/2/2019	FP	6M	5	2019	Year-2		1	Spring
5/2/2019	FP	7M	5	2019	Year-2	0	0	Spring
5/2/2019	FP	10M	5	2019	Year-2	0	0	Spring
5/2/2019	FP	12M	5	2019	Year-2	1	0	Spring
5/2/2019	FP	14M	5	2019	Year-2	1	0	Spring
5/2/2019	FP	8A	5	2019	Year-2	1	0	Spring
5/2/2019	FP	9A	5	2019	Year-2	0	0	Spring
5/2/2019	FP	11A	5	2019	Year-2	0	0	Spring
5/2/2019	FP	13A	5	2019	Year-2	1	0	Spring
5/3/2019	FP	1M	5	2019	Year-2	1	0	Spring
5/3/2019	FP	2M	5	2019	Year-2	1	0	Spring
5/3/2019	FP	3M	5	2019	Year-2	1	0	Spring
5/3/2019	FP	4M	5	2019	Year-2	1	0	Spring
5/3/2019	FP	5M	5	2019	Year-2	1	0	Spring
5/3/2019	FP	6M	5	2019	Year-2		1	Spring
5/3/2019	FP	7M	5	2019	Year-2	0	0	Spring
5/3/2019	FP	10M	5	2019	Year-2	1	0	Spring
5/3/2019	FP	12M	5	2019	Year-2	1	0	Spring
5/3/2019	FP	14M	5	2019	Year-2	1	0	Spring
5/3/2019	FP	8A	5	2019	Year-2	1	0	Spring
5/3/2019	FP	9A	5	2019	Year-2	1	0	Spring
5/3/2019	FP	11A	5	2019	Year-2	1	0	Spring
5/3/2019	FP	13A	5	2019	Year-2	1	0	Spring
5/4/2019	FP	1M	5	2019	Year-2	1	0	Spring
5/4/2019	FP	2M	5	2019	Year-2	1	0	Spring
5/4/2019	FP	3M	5	2019	Year-2	1	0	Spring
5/4/2019	FP	4M	5	2019	Year-2	1	0	Spring
5/4/2019	FP	5M	5	2019	Year-2	0	0	Spring
5/4/2019	FP	6M	5	2019	Year-2		1	Spring
5/4/2019	FP	7M	5	2019	Year-2	0	0	Spring
5/4/2019	FP	10M	5	2019	Year-2	0	0	Spring
5/4/2019	FP	12M	5	2019	Year-2	1	0	Spring
5/4/2019	FP	14M	5	2019	Year-2	1	0	Spring
5/4/2019	FP	8A	5	2019	Year-2	1	0	Spring

Date	Species	Site	Month	Year	Survey Year	Presence	Data Gap	Season
5/4/2019	FP	9A	5	2019	Year-2	0	0	Spring
5/4/2019	FP	11A	5	2019	Year-2	0	0	Spring
5/4/2019	FP	13A	5	2019	Year-2	1	0	Spring
5/5/2019	FP	1M	5	2019	Year-2	1	0	Spring
5/5/2019	FP	2M	5	2019	Year-2	1	0	Spring
5/5/2019	FP	3M	5	2019	Year-2	1	0	Spring
5/5/2019	FP	4M	5	2019	Year-2	1	0	Spring
5/5/2019	FP	5M	5	2019	Year-2	0	0	Spring
5/5/2019	FP	6M	5	2019	Year-2		1	Spring
5/5/2019	FP	7M	5	2019	Year-2	0	0	Spring
5/5/2019	FP	10M	5	2019	Year-2	0	0	Spring
5/5/2019	FP	12M	5	2019	Year-2	1	0	Spring
5/5/2019	FP	14M	5	2019	Year-2	1	0	Spring
5/5/2019	FP	8A	5	2019	Year-2	1	0	Spring
5/5/2019	FP	9A	5	2019	Year-2	1	0	Spring
5/5/2019	FP	11A	5	2019	Year-2	0	0	Spring
5/5/2019	FP	13A	5	2019	Year-2	1	0	Spring
5/6/2019	FP	1M	5	2019	Year-2	0	0	Spring
5/6/2019	FP	2M	5	2019	Year-2	1	0	Spring
5/6/2019	FP	3M	5	2019	Year-2	1	0	Spring
5/6/2019	FP	4M	5	2019	Year-2	0	0	Spring
5/6/2019	FP	5M	5	2019	Year-2	0	0	Spring
5/6/2019	FP	6M	5	2019	Year-2		1	Spring
5/6/2019	FP	7M	5	2019	Year-2	0	0	Spring
5/6/2019	FP	10M	5	2019	Year-2	0	0	Spring
5/6/2019	FP	12M	5	2019	Year-2	1	0	Spring
5/6/2019	FP	14M	5	2019	Year-2	1	0	Spring
5/6/2019	FP	8A	5	2019	Year-2	0	0	Spring
5/6/2019	FP	9A	5	2019	Year-2	0	0	Spring
5/6/2019	FP	11A	5	2019	Year-2	0	0	Spring
5/6/2019	FP	13A	5	2019	Year-2	1	0	Spring
5/7/2019	FP	1M	5	2019	Year-2	1	0	Spring
5/7/2019	FP	2M	5	2019	Year-2	1	0	Spring
5/7/2019	FP	3M	5	2019	Year-2	1	0	Spring
5/7/2019	FP	4M	5	2019	Year-2	0	0	Spring
5/7/2019	FP	5M	5	2019	Year-2	0	0	Spring
5/7/2019	FP	6M	5	2019	Year-2		1	Spring
5/7/2019	FP	7M	5	2019	Year-2	0	0	Spring
5/7/2019	FP	10M	5	2019	Year-2	0	0	Spring
5/7/2019	FP	12M	5	2019	Year-2	1	0	Spring
5/7/2019	FP	14M	5	2019	Year-2	1	0	Spring
5/7/2019	FP	8A	5	2019	Year-2	1	0	Spring
5/7/2019	FP	9A	5	2019	Year-2	0	0	Spring
5/7/2019	FP	11A	5	2019	Year-2	0	0	Spring

Date	Species	Site	Month	Year	Survey Year	Presence	Data Gap	Season
5/7/2019	FP	13A	5	2019	Year-2	1	0	Spring
5/8/2019	FP	1M	5	2019	Year-2	1	0	Spring
5/8/2019	FP	2M	5	2019	Year-2	1	0	Spring
5/8/2019	FP	3M	5	2019	Year-2	1	0	Spring
5/8/2019	FP	4M	5	2019	Year-2	1	0	Spring
5/8/2019	FP	5M	5	2019	Year-2	0	0	Spring
5/8/2019	FP	6M	5	2019	Year-2		1	Spring
5/8/2019	FP	7M	5	2019	Year-2	0	0	Spring
5/8/2019	FP	10M	5	2019	Year-2	1	0	Spring
5/8/2019	FP	12M	5	2019	Year-2	0	0	Spring
5/8/2019	FP	14M	5	2019	Year-2	1	0	Spring
5/8/2019	FP	8A	5	2019	Year-2	0	0	Spring
5/8/2019	FP	9A	5	2019	Year-2	0	0	Spring
5/8/2019	FP	11A	5	2019	Year-2	0	0	Spring
5/8/2019	FP	13A	5	2019	Year-2	1	0	Spring
5/9/2019	FP	1M	5	2019	Year-2	1	0	Spring
5/9/2019	FP	2M	5	2019	Year-2	1	0	Spring
5/9/2019	FP	3M	5	2019	Year-2	1	0	Spring
5/9/2019	FP	4M	5	2019	Year-2	1	0	Spring
5/9/2019	FP	5M	5	2019	Year-2	0	0	Spring
5/9/2019	FP	6M	5	2019	Year-2		1	Spring
5/9/2019	FP	7M	5	2019	Year-2	0	0	Spring
5/9/2019	FP	10M	5	2019	Year-2	0	0	Spring
5/9/2019	FP	12M	5	2019	Year-2	0	0	Spring
5/9/2019	FP	14M	5	2019	Year-2	1	0	Spring
5/9/2019	FP	8A	5	2019	Year-2	1	0	Spring
5/9/2019	FP	9A	5	2019	Year-2	0	0	Spring
5/9/2019	FP	11A	5	2019	Year-2	0	0	Spring
5/9/2019	FP	13A	5	2019	Year-2	1	0	Spring
5/10/2019	FP	1M	5	2019	Year-2	1	0	Spring
5/10/2019	FP	2M	5	2019	Year-2	1	0	Spring
5/10/2019	FP	3M	5	2019	Year-2	1	0	Spring
5/10/2019	FP	4M	5	2019	Year-2	1	0	Spring
5/10/2019	FP	5M	5	2019	Year-2	0	0	Spring
5/10/2019	FP	6M	5	2019	Year-2		1	Spring
5/10/2019	FP	7M	5	2019	Year-2	0	0	Spring
5/10/2019	FP	10M	5	2019	Year-2	0	0	Spring
5/10/2019	FP	12M	5	2019	Year-2	1	0	Spring
5/10/2019	FP	14M	5	2019	Year-2	1	0	Spring
5/10/2019	FP	8A	5	2019	Year-2	1	0	Spring
5/10/2019	FP	9A	5	2019	Year-2	1	0	Spring
5/10/2019	FP	11A	5	2019	Year-2	0	0	Spring
5/10/2019	FP	13A	5	2019	Year-2	1	0	Spring
5/11/2019	FP	1M	5	2019	Year-2	1	0	Spring

Date	Species	Site	Month	Year	Survey Year	Presence	Data Gap	Season
5/11/2019	FP	2M	5	2019	Year-2	1	0	Spring
5/11/2019	FP	3M	5	2019	Year-2	1	0	Spring
5/11/2019	FP	4M	5	2019	Year-2	0	0	Spring
5/11/2019	FP	5M	5	2019	Year-2	0	0	Spring
5/11/2019	FP	6M	5	2019	Year-2		1	Spring
5/11/2019	FP	7M	5	2019	Year-2	0	0	Spring
5/11/2019	FP	10M	5	2019	Year-2	0	0	Spring
5/11/2019	FP	12M	5	2019	Year-2	1	0	Spring
5/11/2019	FP	14M	5	2019	Year-2	1	0	Spring
5/11/2019	FP	8A	5	2019	Year-2	0	0	Spring
5/11/2019	FP	9A	5	2019	Year-2	0	0	Spring
5/11/2019	FP	11A	5	2019	Year-2	0	0	Spring
5/11/2019	FP	13A	5	2019	Year-2	1	0	Spring
5/12/2019	FP	1M	5	2019	Year-2	1	0	Spring
5/12/2019	FP	2M	5	2019	Year-2	1	0	Spring
5/12/2019	FP	3M	5	2019	Year-2	1	0	Spring
5/12/2019	FP	4M	5	2019	Year-2	1	0	Spring
5/12/2019	FP	5M	5	2019	Year-2	0	0	Spring
5/12/2019	FP	6M	5	2019	Year-2		1	Spring
5/12/2019	FP	7M	5	2019	Year-2	0	0	Spring
5/12/2019	FP	10M	5	2019	Year-2	0	0	Spring
5/12/2019	FP	12M	5	2019	Year-2	1	0	Spring
5/12/2019	FP	14M	5	2019	Year-2	1	0	Spring
5/12/2019	FP	8A	5	2019	Year-2	0	0	Spring
5/12/2019	FP	9A	5	2019	Year-2	0	0	Spring
5/12/2019	FP	11A	5	2019	Year-2	0	0	Spring
5/12/2019	FP	13A	5	2019	Year-2	1	0	Spring
5/13/2019	FP	1M	5	2019	Year-2	1	0	Spring
5/13/2019	FP	2M	5	2019	Year-2	1	0	Spring
5/13/2019	FP	3M	5	2019	Year-2	1	0	Spring
5/13/2019	FP	4M	5	2019	Year-2	1	0	Spring
5/13/2019	FP	5M	5	2019	Year-2	0	0	Spring
5/13/2019	FP	6M	5	2019	Year-2		1	Spring
5/13/2019	FP	7M	5	2019	Year-2	0	0	Spring
5/13/2019	FP	10M	5	2019	Year-2	0	0	Spring
5/13/2019	FP	12M	5	2019	Year-2	0	0	Spring
5/13/2019	FP	14M	5	2019	Year-2	1	0	Spring
5/13/2019	FP	8A	5	2019	Year-2	0	0	Spring
5/13/2019	FP	9A	5	2019	Year-2	1	0	Spring
5/13/2019	FP	11A	5	2019	Year-2	0	0	Spring
5/13/2019	FP	13A	5	2019	Year-2	1	0	Spring
5/14/2019	FP	1M	5	2019	Year-2	1	0	Spring
5/14/2019	FP	2M	5	2019	Year-2	1	0	Spring
5/14/2019	FP	3M	5	2019	Year-2	1	0	Spring

Date	Species	Site	Month	Year	Survey Year	Presence	Data Gap	Season
5/14/2019	FP	4M	5	2019	Year-2	1	0	Spring
5/14/2019	FP	5M	5	2019	Year-2	1	0	Spring
5/14/2019	FP	6M	5	2019	Year-2		1	Spring
5/14/2019	FP	7M	5	2019	Year-2	0	0	Spring
5/14/2019	FP	10M	5	2019	Year-2	1	0	Spring
5/14/2019	FP	12M	5	2019	Year-2	0	0	Spring
5/14/2019	FP	14M	5	2019	Year-2	1	0	Spring
5/14/2019	FP	8A	5	2019	Year-2	1	0	Spring
5/14/2019	FP	9A	5	2019	Year-2	1	0	Spring
5/14/2019	FP	11A	5	2019	Year-2	1	0	Spring
5/14/2019	FP	13A	5	2019	Year-2	1	0	Spring
5/15/2019	FP	1M	5	2019	Year-2	1	0	Spring
5/15/2019	FP	2M	5	2019	Year-2	1	0	Spring
5/15/2019	FP	3M	5	2019	Year-2	1	0	Spring
5/15/2019	FP	4M	5	2019	Year-2	1	0	Spring
5/15/2019	FP	5M	5	2019	Year-2	0	0	Spring
5/15/2019	FP	6M	5	2019	Year-2		1	Spring
5/15/2019	FP	7M	5	2019	Year-2	0	0	Spring
5/15/2019	FP	10M	5	2019	Year-2	0	0	Spring
5/15/2019	FP	12M	5	2019	Year-2	1	0	Spring
5/15/2019	FP	14M	5	2019	Year-2	1	0	Spring
5/15/2019	FP	8A	5	2019	Year-2	1	0	Spring
5/15/2019	FP	9A	5	2019	Year-2	1	0	Spring
5/15/2019	FP	11A	5	2019	Year-2	0	0	Spring
5/15/2019	FP	13A	5	2019	Year-2	1	0	Spring
5/16/2019	FP	1M	5	2019	Year-2	1	0	Spring
5/16/2019	FP	2M	5	2019	Year-2	1	0	Spring
5/16/2019	FP	3M	5	2019	Year-2	1	0	Spring
5/16/2019	FP	4M	5	2019	Year-2	1	0	Spring
5/16/2019	FP	5M	5	2019	Year-2	0	0	Spring
5/16/2019	FP	6M	5	2019	Year-2		1	Spring
5/16/2019	FP	7M	5	2019	Year-2	0	0	Spring
5/16/2019	FP	10M	5	2019	Year-2	0	0	Spring
5/16/2019	FP	12M	5	2019	Year-2	1	0	Spring
5/16/2019	FP	14M	5	2019	Year-2	1	0	Spring
5/16/2019	FP	8A	5	2019	Year-2	0	0	Spring
5/16/2019	FP	9A	5	2019	Year-2	0	0	Spring
5/16/2019	FP	11A	5	2019	Year-2		1	Spring
5/16/2019	FP	13A	5	2019	Year-2	1	0	Spring
5/17/2019	FP	1M	5	2019	Year-2	1	0	Spring
5/17/2019	FP	2M	5	2019	Year-2	1	0	Spring
5/17/2019	FP	3M	5	2019	Year-2	1	0	Spring
5/17/2019	FP	4M	5	2019	Year-2	1	0	Spring
5/17/2019	FP	5M	5	2019	Year-2	0	0	Spring

Date	Species	Site	Month	Year	Survey Year	Presence	Data Gap	Season
5/17/2019	FP	6M	5	2019	Year-2		1	Spring
5/17/2019	FP	7M	5	2019	Year-2	0	0	Spring
5/17/2019	FP	10M	5	2019	Year-2	0	0	Spring
5/17/2019	FP	12M	5	2019	Year-2	0	0	Spring
5/17/2019	FP	14M	5	2019	Year-2	1	0	Spring
5/17/2019	FP	8A	5	2019	Year-2	0	0	Spring
5/17/2019	FP	9A	5	2019	Year-2	0	0	Spring
5/17/2019	FP	11A	5	2019	Year-2	0	0	Spring
5/17/2019	FP	13A	5	2019	Year-2	1	0	Spring
5/18/2019	FP	1M	5	2019	Year-2	0	0	Spring
5/18/2019	FP	2M	5	2019	Year-2	1	0	Spring
5/18/2019	FP	3M	5	2019	Year-2	1	0	Spring
5/18/2019	FP	4M	5	2019	Year-2	0	0	Spring
5/18/2019	FP	5M	5	2019	Year-2	0	0	Spring
5/18/2019	FP	6M	5	2019	Year-2		1	Spring
5/18/2019	FP	7M	5	2019	Year-2	0	0	Spring
5/18/2019	FP	10M	5	2019	Year-2	1	0	Spring
5/18/2019	FP	12M	5	2019	Year-2	1	0	Spring
5/18/2019	FP	14M	5	2019	Year-2	1	0	Spring
5/18/2019	FP	8A	5	2019	Year-2	0	0	Spring
5/18/2019	FP	9A	5	2019	Year-2	0	0	Spring
5/18/2019	FP	11A	5	2019	Year-2	0	0	Spring
5/18/2019	FP	13A	5	2019	Year-2	1	0	Spring
5/19/2019	FP	1M	5	2019	Year-2	0	0	Spring
5/19/2019	FP	2M	5	2019	Year-2	0	0	Spring
5/19/2019	FP	3M	5	2019	Year-2	0	0	Spring
5/19/2019	FP	4M	5	2019	Year-2	0	0	Spring
5/19/2019	FP	5M	5	2019	Year-2	0	0	Spring
5/19/2019	FP	6M	5	2019	Year-2		1	Spring
5/19/2019	FP	7M	5	2019	Year-2	0	0	Spring
5/19/2019	FP	10M	5	2019	Year-2	0	0	Spring
5/19/2019	FP	12M	5	2019	Year-2	1	0	Spring
5/19/2019	FP	14M	5	2019	Year-2	1	0	Spring
5/19/2019	FP	8A	5	2019	Year-2	1	0	Spring
5/19/2019	FP	9A	5	2019	Year-2	0	0	Spring
5/19/2019	FP	11A	5	2019	Year-2	0	0	Spring
5/19/2019	FP	13A	5	2019	Year-2	1	0	Spring
5/20/2019	FP	1M	5	2019	Year-2	0	0	Spring
5/20/2019	FP	2M	5	2019	Year-2	0	0	Spring
5/20/2019	FP	3M	5	2019	Year-2	1	0	Spring
5/20/2019	FP	4M	5	2019	Year-2	0	0	Spring
5/20/2019	FP	5M	5	2019	Year-2	0	0	Spring
5/20/2019	FP	6M	5	2019	Year-2		1	Spring
5/20/2019	FP	7M	5	2019	Year-2	0	0	Spring

Date	Species	Site	Month	Year	Survey Year	Presence	Data Gap	Season
5/20/2019	FP	10M	5	2019	Year-2	0	0	Spring
5/20/2019	FP	12M	5	2019	Year-2	1	0	Spring
5/20/2019	FP	14M	5	2019	Year-2	1	0	Spring
5/20/2019	FP	8A	5	2019	Year-2	1	0	Spring
5/20/2019	FP	9A	5	2019	Year-2	0	0	Spring
5/20/2019	FP	11A	5	2019	Year-2	0	0	Spring
5/20/2019	FP	13A	5	2019	Year-2	1	0	Spring
5/21/2019	FP	1M	5	2019	Year-2	0	0	Spring
5/21/2019	FP	2M	5	2019	Year-2	1	0	Spring
5/21/2019	FP	3M	5	2019	Year-2	1	0	Spring
5/21/2019	FP	4M	5	2019	Year-2	1	0	Spring
5/21/2019	FP	5M	5	2019	Year-2	0	0	Spring
5/21/2019	FP	6M	5	2019	Year-2		1	Spring
5/21/2019	FP	7M	5	2019	Year-2	0	0	Spring
5/21/2019	FP	10M	5	2019	Year-2	0	0	Spring
5/21/2019	FP	12M	5	2019	Year-2	1	0	Spring
5/21/2019	FP	14M	5	2019	Year-2	1	0	Spring
5/21/2019	FP	8A	5	2019	Year-2	1	0	Spring
5/21/2019	FP	9A	5	2019	Year-2	0	0	Spring
5/21/2019	FP	11A	5	2019	Year-2	1	0	Spring
5/21/2019	FP	13A	5	2019	Year-2	1	0	Spring
5/22/2019	FP	1M	5	2019	Year-2	1	0	Spring
5/22/2019	FP	2M	5	2019	Year-2	1	0	Spring
5/22/2019	FP	3M	5	2019	Year-2	1	0	Spring
5/22/2019	FP	4M	5	2019	Year-2	0	0	Spring
5/22/2019	FP	5M	5	2019	Year-2	0	0	Spring
5/22/2019	FP	6M	5	2019	Year-2		1	Spring
5/22/2019	FP	7M	5	2019	Year-2	0	0	Spring
5/22/2019	FP	10M	5	2019	Year-2	0	0	Spring
5/22/2019	FP	12M	5	2019	Year-2	1	0	Spring
5/22/2019	FP	14M	5	2019	Year-2	1	0	Spring
5/22/2019	FP	8A	5	2019	Year-2	1	0	Spring
5/22/2019	FP	9A	5	2019	Year-2	1	0	Spring
5/22/2019	FP	11A	5	2019	Year-2	1	0	Spring
5/22/2019	FP	13A	5	2019	Year-2	1	0	Spring
5/23/2019	FP	1M	5	2019	Year-2	1	0	Spring
5/23/2019	FP	2M	5	2019	Year-2	1	0	Spring
5/23/2019	FP	3M	5	2019	Year-2	1	0	Spring
5/23/2019	FP	4M	5	2019	Year-2	1	0	Spring
5/23/2019	FP	5M	5	2019	Year-2	0	0	Spring
5/23/2019	FP	6M	5	2019	Year-2		1	Spring
5/23/2019	FP	7M	5	2019	Year-2	0	0	Spring
5/23/2019	FP	10M	5	2019	Year-2	0	0	Spring
5/23/2019	FP	12M	5	2019	Year-2	1	0	Spring

Date	Species	Site	Month	Year	Survey Year	Presence	Data Gap	Season
5/23/2019	FP	14M	5	2019	Year-2	1	0	Spring
5/23/2019	FP	8A	5	2019	Year-2	1	0	Spring
5/23/2019	FP	9A	5	2019	Year-2	0	0	Spring
5/23/2019	FP	11A	5	2019	Year-2	1	0	Spring
5/23/2019	FP	13A	5	2019	Year-2	1	0	Spring
5/24/2019	FP	1M	5	2019	Year-2	1	0	Spring
5/24/2019	FP	2M	5	2019	Year-2	1	0	Spring
5/24/2019	FP	3M	5	2019	Year-2	1	0	Spring
5/24/2019	FP	4M	5	2019	Year-2	1	0	Spring
5/24/2019	FP	5M	5	2019	Year-2	0	0	Spring
5/24/2019	FP	6M	5	2019	Year-2		1	Spring
5/24/2019	FP	7M	5	2019	Year-2	0	0	Spring
5/24/2019	FP	10M	5	2019	Year-2	0	0	Spring
5/24/2019	FP	12M	5	2019	Year-2	1	0	Spring
5/24/2019	FP	14M	5	2019	Year-2	1	0	Spring
5/24/2019	FP	8A	5	2019	Year-2	0	0	Spring
5/24/2019	FP	9A	5	2019	Year-2	0	0	Spring
5/24/2019	FP	11A	5	2019	Year-2	1	0	Spring
5/24/2019	FP	13A	5	2019	Year-2	1	0	Spring
5/25/2019	FP	1M	5	2019	Year-2	1	0	Spring
5/25/2019	FP	2M	5	2019	Year-2	1	0	Spring
5/25/2019	FP	3M	5	2019	Year-2	1	0	Spring
5/25/2019	FP	4M	5	2019	Year-2	0	0	Spring
5/25/2019	FP	5M	5	2019	Year-2	0	0	Spring
5/25/2019	FP	6M	5	2019	Year-2		1	Spring
5/25/2019	FP	7M	5	2019	Year-2	0	0	Spring
5/25/2019	FP	10M	5	2019	Year-2	0	0	Spring
5/25/2019	FP	12M	5	2019	Year-2	1	0	Spring
5/25/2019	FP	14M	5	2019	Year-2	1	0	Spring
5/25/2019	FP	8A	5	2019	Year-2	1	0	Spring
5/25/2019	FP	9A	5	2019	Year-2	0	0	Spring
5/25/2019	FP	11A	5	2019	Year-2	1	0	Spring
5/25/2019	FP	13A	5	2019	Year-2	1	0	Spring
5/26/2019	FP	1M	5	2019	Year-2	1	0	Spring
5/26/2019	FP	2M	5	2019	Year-2	1	0	Spring
5/26/2019	FP	3M	5	2019	Year-2	1	0	Spring
5/26/2019	FP	4M	5	2019	Year-2	1	0	Spring
5/26/2019	FP	5M	5	2019	Year-2	0	0	Spring
5/26/2019	FP	6M	5	2019	Year-2		1	Spring
5/26/2019	FP	7M	5	2019	Year-2	0	0	Spring
5/26/2019	FP	10M	5	2019	Year-2	0	0	Spring
5/26/2019	FP	12M	5	2019	Year-2	1	0	Spring
5/26/2019	FP	14M	5	2019	Year-2	1	0	Spring
5/26/2019	FP	8A	5	2019	Year-2	1	0	Spring

Date	Species	Site	Month	Year	Survey Year	Presence	Data Gap	Season
5/26/2019	FP	9A	5	2019	Year-2	0	0	Spring
5/26/2019	FP	11A	5	2019	Year-2	0	0	Spring
5/26/2019	FP	13A	5	2019	Year-2	1	0	Spring
5/27/2019	FP	1M	5	2019	Year-2	1	0	Spring
5/27/2019	FP	2M	5	2019	Year-2	1	0	Spring
5/27/2019	FP	3M	5	2019	Year-2	1	0	Spring
5/27/2019	FP	4M	5	2019	Year-2	1	0	Spring
5/27/2019	FP	5M	5	2019	Year-2	0	0	Spring
5/27/2019	FP	6M	5	2019	Year-2		1	Spring
5/27/2019	FP	7M	5	2019	Year-2	0	0	Spring
5/27/2019	FP	10M	5	2019	Year-2	0	0	Spring
5/27/2019	FP	12M	5	2019	Year-2	1	0	Spring
5/27/2019	FP	14M	5	2019	Year-2	1	0	Spring
5/27/2019	FP	8A	5	2019	Year-2	1	0	Spring
5/27/2019	FP	9A	5	2019	Year-2	1	0	Spring
5/27/2019	FP	11A	5	2019	Year-2	1	0	Spring
5/27/2019	FP	13A	5	2019	Year-2	1	0	Spring
5/28/2019	FP	1M	5	2019	Year-2	1	0	Spring
5/28/2019	FP	2M	5	2019	Year-2	1	0	Spring
5/28/2019	FP	3M	5	2019	Year-2	1	0	Spring
5/28/2019	FP	4M	5	2019	Year-2	1	0	Spring
5/28/2019	FP	5M	5	2019	Year-2	1	0	Spring
5/28/2019	FP	6M	5	2019	Year-2		1	Spring
5/28/2019	FP	7M	5	2019	Year-2	0	0	Spring
5/28/2019	FP	10M	5	2019	Year-2	0	0	Spring
5/28/2019	FP	12M	5	2019	Year-2	1	0	Spring
5/28/2019	FP	14M	5	2019	Year-2	1	0	Spring
5/28/2019	FP	8A	5	2019	Year-2	1	0	Spring
5/28/2019	FP	9A	5	2019	Year-2	0	0	Spring
5/28/2019	FP	11A	5	2019	Year-2	0	0	Spring
5/28/2019	FP	13A	5	2019	Year-2	1	0	Spring
5/29/2019	FP	1M	5	2019	Year-2	1	0	Spring
5/29/2019	FP	2M	5	2019	Year-2	1	0	Spring
5/29/2019	FP	3M	5	2019	Year-2	1	0	Spring
5/29/2019	FP	4M	5	2019	Year-2	1	0	Spring
5/29/2019	FP	5M	5	2019	Year-2	0	0	Spring
5/29/2019	FP	6M	5	2019	Year-2		1	Spring
5/29/2019	FP	7M	5	2019	Year-2	0	0	Spring
5/29/2019	FP	10M	5	2019	Year-2	1	0	Spring
5/29/2019	FP	12M	5	2019	Year-2	1	0	Spring
5/29/2019	FP	14M	5	2019	Year-2	1	0	Spring
5/29/2019	FP	8A	5	2019	Year-2	0	0	Spring
5/29/2019	FP	9A	5	2019	Year-2	0	0	Spring
5/29/2019	FP	11A	5	2019	Year-2	1	0	Spring

Date	Species	Site	Month	Year	Survey Year	Presence	Data Gap	Season
5/29/2019	FP	13A	5	2019	Year-2	1	0	Spring
5/30/2019	FP	1M	5	2019	Year-2	1	0	Spring
5/30/2019	FP	2M	5	2019	Year-2	1	0	Spring
5/30/2019	FP	3M	5	2019	Year-2	1	0	Spring
5/30/2019	FP	4M	5	2019	Year-2	1	0	Spring
5/30/2019	FP	5M	5	2019	Year-2	0	0	Spring
5/30/2019	FP	6M	5	2019	Year-2		1	Spring
5/30/2019	FP	7M	5	2019	Year-2	0	0	Spring
5/30/2019	FP	10M	5	2019	Year-2	1	0	Spring
5/30/2019	FP	12M	5	2019	Year-2	1	0	Spring
5/30/2019	FP	14M	5	2019	Year-2	1	0	Spring
5/30/2019	FP	8A	5	2019	Year-2	0	0	Spring
5/30/2019	FP	9A	5	2019	Year-2	0	0	Spring
5/30/2019	FP	11A	5	2019	Year-2	1	0	Spring
5/30/2019	FP	13A	5	2019	Year-2	1	0	Spring
5/31/2019	FP	1M	5	2019	Year-2	0	0	Spring
5/31/2019	FP	2M	5	2019	Year-2	0	0	Spring
5/31/2019	FP	3M	5	2019	Year-2	1	0	Spring
5/31/2019	FP	4M	5	2019	Year-2	1	0	Spring
5/31/2019	FP	5M	5	2019	Year-2	1	0	Spring
5/31/2019	FP	6M	5	2019	Year-2		1	Spring
5/31/2019	FP	7M	5	2019	Year-2	0	0	Spring
5/31/2019	FP	10M	5	2019	Year-2	1	0	Spring
5/31/2019	FP	12M	5	2019	Year-2	1	0	Spring
5/31/2019	FP	14M	5	2019	Year-2	1	0	Spring
5/31/2019	FP	8A	5	2019	Year-2	0	0	Spring
5/31/2019	FP	9A	5	2019	Year-2	0	0	Spring
5/31/2019	FP	11A	5	2019	Year-2	1	0	Spring
5/31/2019	FP	13A	5	2019	Year-2	1	0	Spring
6/1/2019	FP	1M	6	2019	Year-2	0	0	Summer
6/1/2019	FP	2M	6	2019	Year-2	0	0	Summer
6/1/2019	FP	3M	6	2019	Year-2	1	0	Summer
6/1/2019	FP	4M	6	2019	Year-2	1	0	Summer
6/1/2019	FP	5M	6	2019	Year-2	0	0	Summer
6/1/2019	FP	6M	6	2019	Year-2		1	Summer
6/1/2019	FP	7M	6	2019	Year-2	0	0	Summer
6/1/2019	FP	10M	6	2019	Year-2	0	0	Summer
6/1/2019	FP	12M	6	2019	Year-2	1	0	Summer
6/1/2019	FP	14M	6	2019	Year-2	1	0	Summer
6/1/2019	FP	8A	6	2019	Year-2	0	0	Summer
6/1/2019	FP	9A	6	2019	Year-2	0	0	Summer
6/1/2019	FP	11A	6	2019	Year-2	1	0	Summer
6/1/2019	FP	13A	6	2019	Year-2	1	0	Summer
6/2/2019	FP	1M	6	2019	Year-2	1	0	Summer

Date	Species	Site	Month	Year	Survey Year	Presence	Data Gap	Season
6/2/2019	FP	2M	6	2019	Year-2	1	0	Summer
6/2/2019	FP	3M	6	2019	Year-2	1	0	Summer
6/2/2019	FP	4M	6	2019	Year-2	1	0	Summer
6/2/2019	FP	5M	6	2019	Year-2	1	0	Summer
6/2/2019	FP	6M	6	2019	Year-2		1	Summer
6/2/2019	FP	7M	6	2019	Year-2	0	0	Summer
6/2/2019	FP	10M	6	2019	Year-2	1	0	Summer
6/2/2019	FP	12M	6	2019	Year-2	1	0	Summer
6/2/2019	FP	14M	6	2019	Year-2	1	0	Summer
6/2/2019	FP	8A	6	2019	Year-2	1	0	Summer
6/2/2019	FP	9A	6	2019	Year-2	0	0	Summer
6/2/2019	FP	11A	6	2019	Year-2	1	0	Summer
6/2/2019	FP	13A	6	2019	Year-2	0	0	Summer
6/3/2019	FP	1M	6	2019	Year-2	1	0	Summer
6/3/2019	FP	2M	6	2019	Year-2	1	0	Summer
6/3/2019	FP	3M	6	2019	Year-2	1	0	Summer
6/3/2019	FP	4M	6	2019	Year-2	1	0	Summer
6/3/2019	FP	5M	6	2019	Year-2	0	0	Summer
6/3/2019	FP	6M	6	2019	Year-2		1	Summer
6/3/2019	FP	7M	6	2019	Year-2	0	0	Summer
6/3/2019	FP	10M	6	2019	Year-2	0	0	Summer
6/3/2019	FP	12M	6	2019	Year-2	1	0	Summer
6/3/2019	FP	14M	6	2019	Year-2	1	0	Summer
6/3/2019	FP	8A	6	2019	Year-2	1	0	Summer
6/3/2019	FP	9A	6	2019	Year-2	0	0	Summer
6/3/2019	FP	11A	6	2019	Year-2	1	0	Summer
6/3/2019	FP	13A	6	2019	Year-2	0	0	Summer
6/4/2019	FP	1M	6	2019	Year-2	0	0	Summer
6/4/2019	FP	2M	6	2019	Year-2	0	0	Summer
6/4/2019	FP	3M	6	2019	Year-2	1	0	Summer
6/4/2019	FP	4M	6	2019	Year-2	1	0	Summer
6/4/2019	FP	5M	6	2019	Year-2	0	0	Summer
6/4/2019	FP	6M	6	2019	Year-2		1	Summer
6/4/2019	FP	7M	6	2019	Year-2	0	0	Summer
6/4/2019	FP	10M	6	2019	Year-2	0	0	Summer
6/4/2019	FP	12M	6	2019	Year-2	1	0	Summer
6/4/2019	FP	14M	6	2019	Year-2	1	0	Summer
6/4/2019	FP	8A	6	2019	Year-2	0	0	Summer
6/4/2019	FP	9A	6	2019	Year-2	0	0	Summer
6/4/2019	FP	11A	6	2019	Year-2	1	0	Summer
6/4/2019	FP	13A	6	2019	Year-2	1	0	Summer
6/5/2019	FP	1M	6	2019	Year-2	0	0	Summer
6/5/2019	FP	2M	6	2019	Year-2	0	0	Summer
6/5/2019	FP	3M	6	2019	Year-2	1	0	Summer

Date	Species	Site	Month	Year	Survey Year	Presence	Data Gap	Season
6/5/2019	FP	4M	6	2019	Year-2	1	0	Summer
6/5/2019	FP	5M	6	2019	Year-2	1	0	Summer
6/5/2019	FP	6M	6	2019	Year-2		1	Summer
6/5/2019	FP	7M	6	2019	Year-2	0	0	Summer
6/5/2019	FP	10M	6	2019	Year-2	1	0	Summer
6/5/2019	FP	12M	6	2019	Year-2	1	0	Summer
6/5/2019	FP	14M	6	2019	Year-2	1	0	Summer
6/5/2019	FP	8A	6	2019	Year-2	0	0	Summer
6/5/2019	FP	9A	6	2019	Year-2	0	0	Summer
6/5/2019	FP	11A	6	2019	Year-2	1	0	Summer
6/5/2019	FP	13A	6	2019	Year-2	1	0	Summer
6/6/2019	FP	1M	6	2019	Year-2	0	0	Summer
6/6/2019	FP	2M	6	2019	Year-2	0	0	Summer
6/6/2019	FP	3M	6	2019	Year-2	0	0	Summer
6/6/2019	FP	4M	6	2019	Year-2	1	0	Summer
6/6/2019	FP	5M	6	2019	Year-2	1	0	Summer
6/6/2019	FP	6M	6	2019	Year-2		1	Summer
6/6/2019	FP	7M	6	2019	Year-2	0	0	Summer
6/6/2019	FP	10M	6	2019	Year-2	1	0	Summer
6/6/2019	FP	12M	6	2019	Year-2	1	0	Summer
6/6/2019	FP	14M	6	2019	Year-2	1	0	Summer
6/6/2019	FP	8A	6	2019	Year-2	0	0	Summer
6/6/2019	FP	9A	6	2019	Year-2	0	0	Summer
6/6/2019	FP	11A	6	2019	Year-2	1	0	Summer
6/6/2019	FP	13A	6	2019	Year-2	0	0	Summer
6/7/2019	FP	1M	6	2019	Year-2	0	0	Summer
6/7/2019	FP	2M	6	2019	Year-2	1	0	Summer
6/7/2019	FP	3M	6	2019	Year-2	1	0	Summer
6/7/2019	FP	4M	6	2019	Year-2	1	0	Summer
6/7/2019	FP	5M	6	2019	Year-2	1	0	Summer
6/7/2019	FP	6M	6	2019	Year-2		1	Summer
6/7/2019	FP	7M	6	2019	Year-2	0	0	Summer
6/7/2019	FP	10M	6	2019	Year-2	0	0	Summer
6/7/2019	FP	12M	6	2019	Year-2	1	0	Summer
6/7/2019	FP	14M	6	2019	Year-2	0	0	Summer
6/7/2019	FP	8A	6	2019	Year-2	1	0	Summer
6/7/2019	FP	9A	6	2019	Year-2	0	0	Summer
6/7/2019	FP	11A	6	2019	Year-2	1	0	Summer
6/7/2019	FP	13A	6	2019	Year-2	0	0	Summer
6/8/2019	FP	1M	6	2019	Year-2	1	0	Summer
6/8/2019	FP	2M	6	2019	Year-2	1	0	Summer
6/8/2019	FP	3M	6	2019	Year-2	1	0	Summer
6/8/2019	FP	4M	6	2019	Year-2	1	0	Summer
6/8/2019	FP	5M	6	2019	Year-2	1	0	Summer

Date	Species	Site	Month	Year	Survey Year	Presence	Data Gap	Season
6/8/2019	FP	6M	6	2019	Year-2		1	Summer
6/8/2019	FP	7M	6	2019	Year-2	0	0	Summer
6/8/2019	FP	10M	6	2019	Year-2	1	0	Summer
6/8/2019	FP	12M	6	2019	Year-2	1	0	Summer
6/8/2019	FP	14M	6	2019	Year-2	0	0	Summer
6/8/2019	FP	8A	6	2019	Year-2	0	0	Summer
6/8/2019	FP	9A	6	2019	Year-2	0	0	Summer
6/8/2019	FP	11A	6	2019	Year-2	1	0	Summer
6/8/2019	FP	13A	6	2019	Year-2	1	0	Summer
6/9/2019	FP	1M	6	2019	Year-2	1	0	Summer
6/9/2019	FP	2M	6	2019	Year-2	1	0	Summer
6/9/2019	FP	3M	6	2019	Year-2	1	0	Summer
6/9/2019	FP	4M	6	2019	Year-2	1	0	Summer
6/9/2019	FP	5M	6	2019	Year-2	1	0	Summer
6/9/2019	FP	6M	6	2019	Year-2		1	Summer
6/9/2019	FP	7M	6	2019	Year-2	0	0	Summer
6/9/2019	FP	10M	6	2019	Year-2	0	0	Summer
6/9/2019	FP	12M	6	2019	Year-2	1	0	Summer
6/9/2019	FP	14M	6	2019	Year-2	1	0	Summer
6/9/2019	FP	8A	6	2019	Year-2	0	0	Summer
6/9/2019	FP	9A	6	2019	Year-2	1	0	Summer
6/9/2019	FP	11A	6	2019	Year-2	1	0	Summer
6/9/2019	FP	13A	6	2019	Year-2	1	0	Summer
6/10/2019	FP	1M	6	2019	Year-2	0	0	Summer
6/10/2019	FP	2M	6	2019	Year-2	1	0	Summer
6/10/2019	FP	3M	6	2019	Year-2	1	0	Summer
6/10/2019	FP	4M	6	2019	Year-2	1	0	Summer
6/10/2019	FP	5M	6	2019	Year-2	0	0	Summer
6/10/2019	FP	6M	6	2019	Year-2		1	Summer
6/10/2019	FP	7M	6	2019	Year-2	0	0	Summer
6/10/2019	FP	10M	6	2019	Year-2	1	0	Summer
6/10/2019	FP	12M	6	2019	Year-2	1	0	Summer
6/10/2019	FP	14M	6	2019	Year-2	1	0	Summer
6/10/2019	FP	8A	6	2019	Year-2	1	0	Summer
6/10/2019	FP	9A	6	2019	Year-2	0	0	Summer
6/10/2019	FP	11A	6	2019	Year-2	1	0	Summer
6/10/2019	FP	13A	6	2019	Year-2	1	0	Summer
6/11/2019	FP	1M	6	2019	Year-2	1	0	Summer
6/11/2019	FP	2M	6	2019	Year-2	1	0	Summer
6/11/2019	FP	3M	6	2019	Year-2	1	0	Summer
6/11/2019	FP	4M	6	2019	Year-2	1	0	Summer
6/11/2019	FP	5M	6	2019	Year-2	0	0	Summer
6/11/2019	FP	6M	6	2019	Year-2		1	Summer
6/11/2019	FP	7M	6	2019	Year-2	0	0	Summer

Date	Species	Site	Month	Year	Survey Year	Presence	Data Gap	Season
6/11/2019	FP	10M	6	2019	Year-2	1	0	Summer
6/11/2019	FP	12M	6	2019	Year-2	1	0	Summer
6/11/2019	FP	14M	6	2019	Year-2	1	0	Summer
6/11/2019	FP	8A	6	2019	Year-2	0	0	Summer
6/11/2019	FP	9A	6	2019	Year-2	0	0	Summer
6/11/2019	FP	11A	6	2019	Year-2	1	0	Summer
6/11/2019	FP	13A	6	2019	Year-2	1	0	Summer
6/12/2019	FP	1M	6	2019	Year-2	1	0	Summer
6/12/2019	FP	2M	6	2019	Year-2	1	0	Summer
6/12/2019	FP	3M	6	2019	Year-2	1	0	Summer
6/12/2019	FP	4M	6	2019	Year-2	1	0	Summer
6/12/2019	FP	5M	6	2019	Year-2	0	0	Summer
6/12/2019	FP	6M	6	2019	Year-2		1	Summer
6/12/2019	FP	7M	6	2019	Year-2	0	0	Summer
6/12/2019	FP	10M	6	2019	Year-2	1	0	Summer
6/12/2019	FP	12M	6	2019	Year-2	1	0	Summer
6/12/2019	FP	14M	6	2019	Year-2	1	0	Summer
6/12/2019	FP	8A	6	2019	Year-2	0	0	Summer
6/12/2019	FP	9A	6	2019	Year-2	1	0	Summer
6/12/2019	FP	11A	6	2019	Year-2	1	0	Summer
6/12/2019	FP	13A	6	2019	Year-2	1	0	Summer
6/13/2019	FP	1M	6	2019	Year-2	1	0	Summer
6/13/2019	FP	2M	6	2019	Year-2	1	0	Summer
6/13/2019	FP	3M	6	2019	Year-2	1	0	Summer
6/13/2019	FP	4M	6	2019	Year-2	1	0	Summer
6/13/2019	FP	5M	6	2019	Year-2	0	0	Summer
6/13/2019	FP	6M	6	2019	Year-2		1	Summer
6/13/2019	FP	7M	6	2019	Year-2	0	0	Summer
6/13/2019	FP	10M	6	2019	Year-2	1	0	Summer
6/13/2019	FP	12M	6	2019	Year-2	1	0	Summer
6/13/2019	FP	14M	6	2019	Year-2	1	0	Summer
6/13/2019	FP	8A	6	2019	Year-2	0	0	Summer
6/13/2019	FP	9A	6	2019	Year-2	0	0	Summer
6/13/2019	FP	11A	6	2019	Year-2	1	0	Summer
6/13/2019	FP	13A	6	2019	Year-2	1	0	Summer
6/14/2019	FP	1M	6	2019	Year-2	0	0	Summer
6/14/2019	FP	2M	6	2019	Year-2	1	0	Summer
6/14/2019	FP	3M	6	2019	Year-2	1	0	Summer
6/14/2019	FP	4M	6	2019	Year-2	1	0	Summer
6/14/2019	FP	5M	6	2019	Year-2	0	0	Summer
6/14/2019	FP	6M	6	2019	Year-2		1	Summer
6/14/2019	FP	7M	6	2019	Year-2	0	0	Summer
6/14/2019	FP	10M	6	2019	Year-2	1	0	Summer
6/14/2019	FP	12M	6	2019	Year-2	1	0	Summer

Date	Species	Site	Month	Year	Survey Year	Presence	Data Gap	Season
6/14/2019	FP	14M	6	2019	Year-2	0	0	Summer
6/14/2019	FP	8A	6	2019	Year-2	0	0	Summer
6/14/2019	FP	9A	6	2019	Year-2	1	0	Summer
6/14/2019	FP	11A	6	2019	Year-2	1	0	Summer
6/14/2019	FP	13A	6	2019	Year-2	0	0	Summer
6/15/2019	FP	1M	6	2019	Year-2	1	0	Summer
6/15/2019	FP	2M	6	2019	Year-2	1	0	Summer
6/15/2019	FP	3M	6	2019	Year-2	1	0	Summer
6/15/2019	FP	4M	6	2019	Year-2	1	0	Summer
6/15/2019	FP	5M	6	2019	Year-2	0	0	Summer
6/15/2019	FP	6M	6	2019	Year-2		1	Summer
6/15/2019	FP	7M	6	2019	Year-2	0	0	Summer
6/15/2019	FP	10M	6	2019	Year-2	1	0	Summer
6/15/2019	FP	12M	6	2019	Year-2	1	0	Summer
6/15/2019	FP	14M	6	2019	Year-2	0	0	Summer
6/15/2019	FP	8A	6	2019	Year-2	0	0	Summer
6/15/2019	FP	9A	6	2019	Year-2	0	0	Summer
6/15/2019	FP	11A	6	2019	Year-2	1	0	Summer
6/15/2019	FP	13A	6	2019	Year-2	0	0	Summer
6/16/2019	FP	1M	6	2019	Year-2	0	0	Summer
6/16/2019	FP	2M	6	2019	Year-2	1	0	Summer
6/16/2019	FP	3M	6	2019	Year-2	1	0	Summer
6/16/2019	FP	4M	6	2019	Year-2	1	0	Summer
6/16/2019	FP	5M	6	2019	Year-2	0	0	Summer
6/16/2019	FP	6M	6	2019	Year-2		1	Summer
6/16/2019	FP	7M	6	2019	Year-2	0	0	Summer
6/16/2019	FP	10M	6	2019	Year-2	1	0	Summer
6/16/2019	FP	12M	6	2019	Year-2	1	0	Summer
6/16/2019	FP	14M	6	2019	Year-2	0	0	Summer
6/16/2019	FP	8A	6	2019	Year-2	0	0	Summer
6/16/2019	FP	9A	6	2019	Year-2	0	0	Summer
6/16/2019	FP	11A	6	2019	Year-2	1	0	Summer
6/16/2019	FP	13A	6	2019	Year-2	0	0	Summer
6/17/2019	FP	1M	6	2019	Year-2	0	0	Summer
6/17/2019	FP	2M	6	2019	Year-2	0	0	Summer
6/17/2019	FP	3M	6	2019	Year-2	1	0	Summer
6/17/2019	FP	4M	6	2019	Year-2	1	0	Summer
6/17/2019	FP	5M	6	2019	Year-2	1	0	Summer
6/17/2019	FP	6M	6	2019	Year-2		1	Summer
6/17/2019	FP	7M	6	2019	Year-2	0	0	Summer
6/17/2019	FP	10M	6	2019	Year-2	1	0	Summer
6/17/2019	FP	12M	6	2019	Year-2	1	0	Summer
6/17/2019	FP	14M	6	2019	Year-2	0	0	Summer
6/17/2019	FP	8A	6	2019	Year-2	0	0	Summer

Date	Species	Site	Month	Year	Survey Year	Presence	Data Gap	Season
6/17/2019	FP	9A	6	2019	Year-2	1	0	Summer
6/17/2019	FP	11A	6	2019	Year-2	1	0	Summer
6/17/2019	FP	13A	6	2019	Year-2	1	0	Summer
6/18/2019	FP	1M	6	2019	Year-2	0	0	Summer
6/18/2019	FP	2M	6	2019	Year-2	0	0	Summer
6/18/2019	FP	3M	6	2019	Year-2	1	0	Summer
6/18/2019	FP	4M	6	2019	Year-2	1	0	Summer
6/18/2019	FP	5M	6	2019	Year-2	1	0	Summer
6/18/2019	FP	6M	6	2019	Year-2		1	Summer
6/18/2019	FP	7M	6	2019	Year-2	0	0	Summer
6/18/2019	FP	10M	6	2019	Year-2	1	0	Summer
6/18/2019	FP	12M	6	2019	Year-2	1	0	Summer
6/18/2019	FP	14M	6	2019	Year-2	1	0	Summer
6/18/2019	FP	8A	6	2019	Year-2	0	0	Summer
6/18/2019	FP	9A	6	2019	Year-2	0	0	Summer
6/18/2019	FP	11A	6	2019	Year-2	1	0	Summer
6/18/2019	FP	13A	6	2019	Year-2	1	0	Summer
6/19/2019	FP	1M	6	2019	Year-2	1	0	Summer
6/19/2019	FP	2M	6	2019	Year-2	1	0	Summer
6/19/2019	FP	3M	6	2019	Year-2	1	0	Summer
6/19/2019	FP	4M	6	2019	Year-2	1	0	Summer
6/19/2019	FP	5M	6	2019	Year-2	1	0	Summer
6/19/2019	FP	6M	6	2019	Year-2		1	Summer
6/19/2019	FP	7M	6	2019	Year-2	0	0	Summer
6/19/2019	FP	10M	6	2019	Year-2	0	0	Summer
6/19/2019	FP	12M	6	2019	Year-2	1	0	Summer
6/19/2019	FP	14M	6	2019	Year-2	1	0	Summer
6/19/2019	FP	8A	6	2019	Year-2	1	0	Summer
6/19/2019	FP	9A	6	2019	Year-2	1	0	Summer
6/19/2019	FP	11A	6	2019	Year-2	1	0	Summer
6/19/2019	FP	13A	6	2019	Year-2	1	0	Summer
6/20/2019	FP	1M	6	2019	Year-2	0	0	Summer
6/20/2019	FP	2M	6	2019	Year-2	1	0	Summer
6/20/2019	FP	3M	6	2019	Year-2	1	0	Summer
6/20/2019	FP	4M	6	2019	Year-2	1	0	Summer
6/20/2019	FP	5M	6	2019	Year-2	0	0	Summer
6/20/2019	FP	6M	6	2019	Year-2		1	Summer
6/20/2019	FP	7M	6	2019	Year-2	0	0	Summer
6/20/2019	FP	10M	6	2019	Year-2	1	0	Summer
6/20/2019	FP	12M	6	2019	Year-2	1	0	Summer
6/20/2019	FP	14M	6	2019	Year-2	1	0	Summer
6/20/2019	FP	8A	6	2019	Year-2	0	0	Summer
6/20/2019	FP	9A	6	2019	Year-2	0	0	Summer
6/20/2019	FP	11A	6	2019	Year-2	1	0	Summer

Date	Species	Site	Month	Year	Survey Year	Presence	Data Gap	Season
6/20/2019	FP	13A	6	2019	Year-2	1	0	Summer
6/21/2019	FP	1M	6	2019	Year-2	0	0	Summer
6/21/2019	FP	2M	6	2019	Year-2	0	0	Summer
6/21/2019	FP	3M	6	2019	Year-2	1	0	Summer
6/21/2019	FP	4M	6	2019	Year-2	1	0	Summer
6/21/2019	FP	5M	6	2019	Year-2	1	0	Summer
6/21/2019	FP	6M	6	2019	Year-2		1	Summer
6/21/2019	FP	7M	6	2019	Year-2	0	0	Summer
6/21/2019	FP	10M	6	2019	Year-2	1	0	Summer
6/21/2019	FP	12M	6	2019	Year-2	1	0	Summer
6/21/2019	FP	14M	6	2019	Year-2	0	0	Summer
6/21/2019	FP	8A	6	2019	Year-2	0	0	Summer
6/21/2019	FP	9A	6	2019	Year-2	0	0	Summer
6/21/2019	FP	11A	6	2019	Year-2	1	0	Summer
6/21/2019	FP	13A	6	2019	Year-2	1	0	Summer
6/22/2019	FP	1M	6	2019	Year-2	0	0	Summer
6/22/2019	FP	2M	6	2019	Year-2	1	0	Summer
6/22/2019	FP	3M	6	2019	Year-2	1	0	Summer
6/22/2019	FP	4M	6	2019	Year-2	1	0	Summer
6/22/2019	FP	5M	6	2019	Year-2	0	0	Summer
6/22/2019	FP	6M	6	2019	Year-2		1	Summer
6/22/2019	FP	7M	6	2019	Year-2	0	0	Summer
6/22/2019	FP	10M	6	2019	Year-2	1	0	Summer
6/22/2019	FP	12M	6	2019	Year-2	0	0	Summer
6/22/2019	FP	14M	6	2019	Year-2	1	0	Summer
6/22/2019	FP	8A	6	2019	Year-2	0	0	Summer
6/22/2019	FP	9A	6	2019	Year-2	0	0	Summer
6/22/2019	FP	11A	6	2019	Year-2	1	0	Summer
6/22/2019	FP	13A	6	2019	Year-2	1	0	Summer
6/23/2019	FP	1M	6	2019	Year-2	0	0	Summer
6/23/2019	FP	2M	6	2019	Year-2	1	0	Summer
6/23/2019	FP	3M	6	2019	Year-2	1	0	Summer
6/23/2019	FP	4M	6	2019	Year-2	1	0	Summer
6/23/2019	FP	5M	6	2019	Year-2	0	0	Summer
6/23/2019	FP	6M	6	2019	Year-2		1	Summer
6/23/2019	FP	7M	6	2019	Year-2	0	0	Summer
6/23/2019	FP	10M	6	2019	Year-2	0	0	Summer
6/23/2019	FP	12M	6	2019	Year-2	0	0	Summer
6/23/2019	FP	14M	6	2019	Year-2	0	0	Summer
6/23/2019	FP	8A	6	2019	Year-2	0	0	Summer
6/23/2019	FP	9A	6	2019	Year-2	0	0	Summer
6/23/2019	FP	11A	6	2019	Year-2	0	0	Summer
6/23/2019	FP	13A	6	2019	Year-2	0	0	Summer
6/24/2019	FP	1M	6	2019	Year-2	0	0	Summer

Date	Species	Site	Month	Year	Survey Year	Presence	Data Gap	Season
6/24/2019	FP	2M	6	2019	Year-2	1	0	Summer
6/24/2019	FP	3M	6	2019	Year-2	1	0	Summer
6/24/2019	FP	4M	6	2019	Year-2	1	0	Summer
6/24/2019	FP	5M	6	2019	Year-2	0	0	Summer
6/24/2019	FP	6M	6	2019	Year-2		1	Summer
6/24/2019	FP	7M	6	2019	Year-2	0	0	Summer
6/24/2019	FP	10M	6	2019	Year-2	1	0	Summer
6/24/2019	FP	12M	6	2019	Year-2	1	0	Summer
6/24/2019	FP	14M	6	2019	Year-2	1	0	Summer
6/24/2019	FP	8A	6	2019	Year-2	0	0	Summer
6/24/2019	FP	9A	6	2019	Year-2	0	0	Summer
6/24/2019	FP	11A	6	2019	Year-2	0	0	Summer
6/24/2019	FP	13A	6	2019	Year-2	0	0	Summer
6/25/2019	FP	1M	6	2019	Year-2	1	0	Summer
6/25/2019	FP	2M	6	2019	Year-2	1	0	Summer
6/25/2019	FP	3M	6	2019	Year-2	1	0	Summer
6/25/2019	FP	4M	6	2019	Year-2	1	0	Summer
6/25/2019	FP	5M	6	2019	Year-2	1	0	Summer
6/25/2019	FP	6M	6	2019	Year-2		1	Summer
6/25/2019	FP	7M	6	2019	Year-2	0	0	Summer
6/25/2019	FP	10M	6	2019	Year-2	1	0	Summer
6/25/2019	FP	12M	6	2019	Year-2	1	0	Summer
6/25/2019	FP	14M	6	2019	Year-2	1	0	Summer
6/25/2019	FP	8A	6	2019	Year-2	0	0	Summer
6/25/2019	FP	9A	6	2019	Year-2	0	0	Summer
6/25/2019	FP	11A	6	2019	Year-2	1	0	Summer
6/25/2019	FP	13A	6	2019	Year-2	1	0	Summer
6/26/2019	FP	1M	6	2019	Year-2	0	0	Summer
6/26/2019	FP	2M	6	2019	Year-2	0	0	Summer
6/26/2019	FP	3M	6	2019	Year-2	1	0	Summer
6/26/2019	FP	4M	6	2019	Year-2	1	0	Summer
6/26/2019	FP	5M	6	2019	Year-2	1	0	Summer
6/26/2019	FP	6M	6	2019	Year-2		1	Summer
6/26/2019	FP	7M	6	2019	Year-2	0	0	Summer
6/26/2019	FP	10M	6	2019	Year-2	1	0	Summer
6/26/2019	FP	12M	6	2019	Year-2	1	0	Summer
6/26/2019	FP	14M	6	2019	Year-2	1	0	Summer
6/26/2019	FP	8A	6	2019	Year-2	0	0	Summer
6/26/2019	FP	9A	6	2019	Year-2	0	0	Summer
6/26/2019	FP	11A	6	2019	Year-2	1	0	Summer
6/26/2019	FP	13A	6	2019	Year-2	0	0	Summer
6/27/2019	FP	1M	6	2019	Year-2	0	0	Summer
6/27/2019	FP	2M	6	2019	Year-2	0	0	Summer
6/27/2019	FP	3M	6	2019	Year-2	1	0	Summer

Date	Species	Site	Month	Year	Survey Year	Presence	Data Gap	Season
6/27/2019	FP	4M	6	2019	Year-2	1	0	Summer
6/27/2019	FP	5M	6	2019	Year-2	0	0	Summer
6/27/2019	FP	6M	6	2019	Year-2		1	Summer
6/27/2019	FP	7M	6	2019	Year-2	0	0	Summer
6/27/2019	FP	10M	6	2019	Year-2	0	0	Summer
6/27/2019	FP	12M	6	2019	Year-2	1	0	Summer
6/27/2019	FP	14M	6	2019	Year-2	1	0	Summer
6/27/2019	FP	8A	6	2019	Year-2	0	0	Summer
6/27/2019	FP	9A	6	2019	Year-2	0	0	Summer
6/27/2019	FP	11A	6	2019	Year-2	0	0	Summer
6/27/2019	FP	13A	6	2019	Year-2	0	0	Summer
6/28/2019	FP	1M	6	2019	Year-2	0	0	Summer
6/28/2019	FP	2M	6	2019	Year-2	1	0	Summer
6/28/2019	FP	3M	6	2019	Year-2	1	0	Summer
6/28/2019	FP	4M	6	2019	Year-2	1	0	Summer
6/28/2019	FP	5M	6	2019	Year-2	1	0	Summer
6/28/2019	FP	6M	6	2019	Year-2		1	Summer
6/28/2019	FP	7M	6	2019	Year-2	0	0	Summer
6/28/2019	FP	10M	6	2019	Year-2	1	0	Summer
6/28/2019	FP	12M	6	2019	Year-2	1	0	Summer
6/28/2019	FP	14M	6	2019	Year-2	1	0	Summer
6/28/2019	FP	8A	6	2019	Year-2	0	0	Summer
6/28/2019	FP	9A	6	2019	Year-2	0	0	Summer
6/28/2019	FP	11A	6	2019	Year-2	1	0	Summer
6/28/2019	FP	13A	6	2019	Year-2	0	0	Summer
6/29/2019	FP	1M	6	2019	Year-2	1	0	Summer
6/29/2019	FP	2M	6	2019	Year-2	1	0	Summer
6/29/2019	FP	3M	6	2019	Year-2	1	0	Summer
6/29/2019	FP	4M	6	2019	Year-2	1	0	Summer
6/29/2019	FP	5M	6	2019	Year-2	1	0	Summer
6/29/2019	FP	6M	6	2019	Year-2		1	Summer
6/29/2019	FP	7M	6	2019	Year-2	0	0	Summer
6/29/2019	FP	10M	6	2019	Year-2	1	0	Summer
6/29/2019	FP	12M	6	2019	Year-2	1	0	Summer
6/29/2019	FP	14M	6	2019	Year-2	1	0	Summer
6/29/2019	FP	8A	6	2019	Year-2	0	0	Summer
6/29/2019	FP	9A	6	2019	Year-2	0	0	Summer
6/29/2019	FP	11A	6	2019	Year-2	1	0	Summer
6/29/2019	FP	13A	6	2019	Year-2	1	0	Summer
6/30/2019	FP	1M	6	2019	Year-2	1	0	Summer
6/30/2019	FP	2M	6	2019	Year-2	1	0	Summer
6/30/2019	FP	3M	6	2019	Year-2	1	0	Summer
6/30/2019	FP	4M	6	2019	Year-2	1	0	Summer
6/30/2019	FP	5M	6	2019	Year-2	1	0	Summer

Date	Species	Site	Month	Year	Survey Year	Presence	Data Gap	Season
6/30/2019	FP	6M	6	2019	Year-2		1	Summer
6/30/2019	FP	7M	6	2019	Year-2	0	0	Summer
6/30/2019	FP	10M	6	2019	Year-2	1	0	Summer
6/30/2019	FP	12M	6	2019	Year-2	1	0	Summer
6/30/2019	FP	14M	6	2019	Year-2	1	0	Summer
6/30/2019	FP	8A	6	2019	Year-2	0	0	Summer
6/30/2019	FP	9A	6	2019	Year-2	0	0	Summer
6/30/2019	FP	11A	6	2019	Year-2	1	0	Summer
6/30/2019	FP	13A	6	2019	Year-2	1	0	Summer
7/1/2019	FP	1M	7	2019	Year-2	0	0	Summer
7/1/2019	FP	2M	7	2019	Year-2	1	0	Summer
7/1/2019	FP	3M	7	2019	Year-2	1	0	Summer
7/1/2019	FP	4M	7	2019	Year-2	1	0	Summer
7/1/2019	FP	5M	7	2019	Year-2	1	0	Summer
7/1/2019	FP	6M	7	2019	Year-2		1	Summer
7/1/2019	FP	7M	7	2019	Year-2	0	0	Summer
7/1/2019	FP	10M	7	2019	Year-2	1	0	Summer
7/1/2019	FP	12M	7	2019	Year-2	1	0	Summer
7/1/2019	FP	14M	7	2019	Year-2	1	0	Summer
7/1/2019	FP	8A	7	2019	Year-2	0	0	Summer
7/1/2019	FP	9A	7	2019	Year-2	0	0	Summer
7/1/2019	FP	11A	7	2019	Year-2	0	0	Summer
7/1/2019	FP	13A	7	2019	Year-2	1	0	Summer
7/2/2019	FP	1M	7	2019	Year-2	0	0	Summer
7/2/2019	FP	2M	7	2019	Year-2	1	0	Summer
7/2/2019	FP	3M	7	2019	Year-2	1	0	Summer
7/2/2019	FP	4M	7	2019	Year-2	1	0	Summer
7/2/2019	FP	5M	7	2019	Year-2	0	0	Summer
7/2/2019	FP	6M	7	2019	Year-2		1	Summer
7/2/2019	FP	7M	7	2019	Year-2	0	0	Summer
7/2/2019	FP	10M	7	2019	Year-2	1	0	Summer
7/2/2019	FP	12M	7	2019	Year-2	1	0	Summer
7/2/2019	FP	14M	7	2019	Year-2	1	0	Summer
7/2/2019	FP	8A	7	2019	Year-2	0	0	Summer
7/2/2019	FP	9A	7	2019	Year-2	1	0	Summer
7/2/2019	FP	11A	7	2019	Year-2	1	0	Summer
7/2/2019	FP	13A	7	2019	Year-2	1	0	Summer
7/3/2019	FP	1M	7	2019	Year-2	1	0	Summer
7/3/2019	FP	2M	7	2019	Year-2	1	0	Summer
7/3/2019	FP	3M	7	2019	Year-2	1	0	Summer
7/3/2019	FP	4M	7	2019	Year-2	1	0	Summer
7/3/2019	FP	5M	7	2019	Year-2	1	0	Summer
7/3/2019	FP	6M	7	2019	Year-2		1	Summer
7/3/2019	FP	7M	7	2019	Year-2	0	0	Summer

Date	Species	Site	Month	Year	Survey Year	Presence	Data Gap	Season
7/3/2019	FP	10M	7	2019	Year-2	0	0	Summer
7/3/2019	FP	12M	7	2019	Year-2	1	0	Summer
7/3/2019	FP	14M	7	2019	Year-2	1	0	Summer
7/3/2019	FP	8A	7	2019	Year-2	0	0	Summer
7/3/2019	FP	9A	7	2019	Year-2	0	0	Summer
7/3/2019	FP	11A	7	2019	Year-2	1	0	Summer
7/3/2019	FP	13A	7	2019	Year-2	1	0	Summer
7/4/2019	FP	1M	7	2019	Year-2	1	0	Summer
7/4/2019	FP	2M	7	2019	Year-2	1	0	Summer
7/4/2019	FP	3M	7	2019	Year-2	1	0	Summer
7/4/2019	FP	4M	7	2019	Year-2	1	0	Summer
7/4/2019	FP	5M	7	2019	Year-2	0	0	Summer
7/4/2019	FP	6M	7	2019	Year-2		1	Summer
7/4/2019	FP	7M	7	2019	Year-2	0	0	Summer
7/4/2019	FP	10M	7	2019	Year-2	0	0	Summer
7/4/2019	FP	12M	7	2019	Year-2	1	0	Summer
7/4/2019	FP	14M	7	2019	Year-2	1	0	Summer
7/4/2019	FP	8A	7	2019	Year-2	0	0	Summer
7/4/2019	FP	9A	7	2019	Year-2	0	0	Summer
7/4/2019	FP	11A	7	2019	Year-2	0	0	Summer
7/4/2019	FP	13A	7	2019	Year-2	1	0	Summer
7/5/2019	FP	1M	7	2019	Year-2	1	0	Summer
7/5/2019	FP	2M	7	2019	Year-2	1	0	Summer
7/5/2019	FP	3M	7	2019	Year-2	1	0	Summer
7/5/2019	FP	4M	7	2019	Year-2	1	0	Summer
7/5/2019	FP	5M	7	2019	Year-2	1	0	Summer
7/5/2019	FP	6M	7	2019	Year-2		1	Summer
7/5/2019	FP	7M	7	2019	Year-2	0	0	Summer
7/5/2019	FP	10M	7	2019	Year-2	1	0	Summer
7/5/2019	FP	12M	7	2019	Year-2	1	0	Summer
7/5/2019	FP	14M	7	2019	Year-2	1	0	Summer
7/5/2019	FP	8A	7	2019	Year-2	0	0	Summer
7/5/2019	FP	9A	7	2019	Year-2	0	0	Summer
7/5/2019	FP	11A	7	2019	Year-2	1	0	Summer
7/5/2019	FP	13A	7	2019	Year-2	1	0	Summer
7/6/2019	FP	1M	7	2019	Year-2	1	0	Summer
7/6/2019	FP	2M	7	2019	Year-2	1	0	Summer
7/6/2019	FP	3M	7	2019	Year-2	1	0	Summer
7/6/2019	FP	4M	7	2019	Year-2	1	0	Summer
7/6/2019	FP	5M	7	2019	Year-2	0	0	Summer
7/6/2019	FP	6M	7	2019	Year-2		1	Summer
7/6/2019	FP	7M	7	2019	Year-2	0	0	Summer
7/6/2019	FP	10M	7	2019	Year-2	1	0	Summer
7/6/2019	FP	12M	7	2019	Year-2	1	0	Summer

Date	Species	Site	Month	Year	Survey Year	Presence	Data Gap	Season
7/6/2019	FP	14M	7	2019	Year-2	1	0	Summer
7/6/2019	FP	8A	7	2019	Year-2	0	0	Summer
7/6/2019	FP	9A	7	2019	Year-2	0	0	Summer
7/6/2019	FP	11A	7	2019	Year-2	1	0	Summer
7/6/2019	FP	13A	7	2019	Year-2	1	0	Summer
7/7/2019	FP	1M	7	2019	Year-2	1	0	Summer
7/7/2019	FP	2M	7	2019	Year-2	1	0	Summer
7/7/2019	FP	3M	7	2019	Year-2	1	0	Summer
7/7/2019	FP	4M	7	2019	Year-2	1	0	Summer
7/7/2019	FP	5M	7	2019	Year-2	1	0	Summer
7/7/2019	FP	6M	7	2019	Year-2		1	Summer
7/7/2019	FP	7M	7	2019	Year-2	0	0	Summer
7/7/2019	FP	10M	7	2019	Year-2	1	0	Summer
7/7/2019	FP	12M	7	2019	Year-2	1	0	Summer
7/7/2019	FP	14M	7	2019	Year-2	1	0	Summer
7/7/2019	FP	8A	7	2019	Year-2	0	0	Summer
7/7/2019	FP	9A	7	2019	Year-2	0	0	Summer
7/7/2019	FP	11A	7	2019	Year-2	1	0	Summer
7/7/2019	FP	13A	7	2019	Year-2	1	0	Summer
7/8/2019	FP	1M	7	2019	Year-2	0	0	Summer
7/8/2019	FP	2M	7	2019	Year-2	0	0	Summer
7/8/2019	FP	3M	7	2019	Year-2	0	0	Summer
7/8/2019	FP	4M	7	2019	Year-2	1	0	Summer
7/8/2019	FP	5M	7	2019	Year-2	1	0	Summer
7/8/2019	FP	6M	7	2019	Year-2		1	Summer
7/8/2019	FP	7M	7	2019	Year-2	0	0	Summer
7/8/2019	FP	10M	7	2019	Year-2	1	0	Summer
7/8/2019	FP	12M	7	2019	Year-2	1	0	Summer
7/8/2019	FP	14M	7	2019	Year-2	0	0	Summer
7/8/2019	FP	8A	7	2019	Year-2	0	0	Summer
7/8/2019	FP	9A	7	2019	Year-2	0	0	Summer
7/8/2019	FP	11A	7	2019	Year-2	1	0	Summer
7/8/2019	FP	13A	7	2019	Year-2	1	0	Summer
7/9/2019	FP	1M	7	2019	Year-2	0	0	Summer
7/9/2019	FP	2M	7	2019	Year-2	0	0	Summer
7/9/2019	FP	3M	7	2019	Year-2	1	0	Summer
7/9/2019	FP	4M	7	2019	Year-2	1	0	Summer
7/9/2019	FP	5M	7	2019	Year-2	0	0	Summer
7/9/2019	FP	6M	7	2019	Year-2		1	Summer
7/9/2019	FP	7M	7	2019	Year-2	0	0	Summer
7/9/2019	FP	10M	7	2019	Year-2	1	0	Summer
7/9/2019	FP	12M	7	2019	Year-2	1	0	Summer
7/9/2019	FP	14M	7	2019	Year-2	1	0	Summer
7/9/2019	FP	8A	7	2019	Year-2	0	0	Summer

Date	Species	Site	Month	Year	Survey Year	Presence	Data Gap	Season
7/9/2019	FP	9A	7	2019	Year-2	0	0	Summer
7/9/2019	FP	11A	7	2019	Year-2	1	0	Summer
7/9/2019	FP	13A	7	2019	Year-2	1	0	Summer
7/10/2019	FP	1M	7	2019	Year-2	1	0	Summer
7/10/2019	FP	2M	7	2019	Year-2	1	0	Summer
7/10/2019	FP	3M	7	2019	Year-2	1	0	Summer
7/10/2019	FP	4M	7	2019	Year-2	1	0	Summer
7/10/2019	FP	5M	7	2019	Year-2	1	0	Summer
7/10/2019	FP	6M	7	2019	Year-2		1	Summer
7/10/2019	FP	7M	7	2019	Year-2	0	0	Summer
7/10/2019	FP	10M	7	2019	Year-2	1	0	Summer
7/10/2019	FP	12M	7	2019	Year-2	1	0	Summer
7/10/2019	FP	14M	7	2019	Year-2	0	0	Summer
7/10/2019	FP	8A	7	2019	Year-2	0	0	Summer
7/10/2019	FP	9A	7	2019	Year-2	0	0	Summer
7/10/2019	FP	11A	7	2019	Year-2	1	0	Summer
7/10/2019	FP	13A	7	2019	Year-2	0	0	Summer
7/11/2019	FP	1M	7	2019	Year-2	1	0	Summer
7/11/2019	FP	2M	7	2019	Year-2	1	0	Summer
7/11/2019	FP	3M	7	2019	Year-2	1	0	Summer
7/11/2019	FP	4M	7	2019	Year-2	1	0	Summer
7/11/2019	FP	5M	7	2019	Year-2	0	0	Summer
7/11/2019	FP	6M	7	2019	Year-2		1	Summer
7/11/2019	FP	7M	7	2019	Year-2	0	0	Summer
7/11/2019	FP	10M	7	2019	Year-2	0	0	Summer
7/11/2019	FP	12M	7	2019	Year-2	1	0	Summer
7/11/2019	FP	14M	7	2019	Year-2	1	0	Summer
7/11/2019	FP	8A	7	2019	Year-2	0	0	Summer
7/11/2019	FP	9A	7	2019	Year-2	0	0	Summer
7/11/2019	FP	11A	7	2019	Year-2	1	0	Summer
7/11/2019	FP	13A	7	2019	Year-2	0	0	Summer
7/12/2019	FP	1M	7	2019	Year-2	1	0	Summer
7/12/2019	FP	2M	7	2019	Year-2	1	0	Summer
7/12/2019	FP	3M	7	2019	Year-2	1	0	Summer
7/12/2019	FP	4M	7	2019	Year-2	1	0	Summer
7/12/2019	FP	5M	7	2019	Year-2	0	0	Summer
7/12/2019	FP	6M	7	2019	Year-2		1	Summer
7/12/2019	FP	7M	7	2019	Year-2	0	0	Summer
7/12/2019	FP	10M	7	2019	Year-2	0	0	Summer
7/12/2019	FP	12M	7	2019	Year-2	1	0	Summer
7/12/2019	FP	14M	7	2019	Year-2	1	0	Summer
7/12/2019	FP	8A	7	2019	Year-2	0	0	Summer
7/12/2019	FP	9A	7	2019	Year-2	0	0	Summer
7/12/2019	FP	11A	7	2019	Year-2	0	0	Summer

Date	Species	Site	Month	Year	Survey Year	Presence	Data Gap	Season
7/12/2019	FP	13A	7	2019	Year-2	1	0	Summer
7/13/2019	FP	1M	7	2019	Year-2	1	0	Summer
7/13/2019	FP	2M	7	2019	Year-2	1	0	Summer
7/13/2019	FP	3M	7	2019	Year-2	1	0	Summer
7/13/2019	FP	4M	7	2019	Year-2	1	0	Summer
7/13/2019	FP	5M	7	2019	Year-2	1	0	Summer
7/13/2019	FP	6M	7	2019	Year-2		1	Summer
7/13/2019	FP	7M	7	2019	Year-2	0	0	Summer
7/13/2019	FP	10M	7	2019	Year-2	1	0	Summer
7/13/2019	FP	12M	7	2019	Year-2	1	0	Summer
7/13/2019	FP	14M	7	2019	Year-2	1	0	Summer
7/13/2019	FP	8A	7	2019	Year-2	0	0	Summer
7/13/2019	FP	9A	7	2019	Year-2	0	0	Summer
7/13/2019	FP	11A	7	2019	Year-2	1	0	Summer
7/13/2019	FP	13A	7	2019	Year-2	1	0	Summer
7/14/2019	FP	1M	7	2019	Year-2	1	0	Summer
7/14/2019	FP	2M	7	2019	Year-2	1	0	Summer
7/14/2019	FP	3M	7	2019	Year-2	1	0	Summer
7/14/2019	FP	4M	7	2019	Year-2	1	0	Summer
7/14/2019	FP	5M	7	2019	Year-2	0	0	Summer
7/14/2019	FP	6M	7	2019	Year-2		1	Summer
7/14/2019	FP	7M	7	2019	Year-2	0	0	Summer
7/14/2019	FP	10M	7	2019	Year-2	0	0	Summer
7/14/2019	FP	12M	7	2019	Year-2	1	0	Summer
7/14/2019	FP	14M	7	2019	Year-2	1	0	Summer
7/14/2019	FP	8A	7	2019	Year-2	0	0	Summer
7/14/2019	FP	9A	7	2019	Year-2	0	0	Summer
7/14/2019	FP	11A	7	2019	Year-2	1	0	Summer
7/14/2019	FP	13A	7	2019	Year-2	1	0	Summer
7/15/2019	FP	1M	7	2019	Year-2	1	0	Summer
7/15/2019	FP	2M	7	2019	Year-2	1	0	Summer
7/15/2019	FP	3M	7	2019	Year-2	1	0	Summer
7/15/2019	FP	4M	7	2019	Year-2	1	0	Summer
7/15/2019	FP	5M	7	2019	Year-2	1	0	Summer
7/15/2019	FP	6M	7	2019	Year-2		1	Summer
7/15/2019	FP	7M	7	2019	Year-2	0	0	Summer
7/15/2019	FP	10M	7	2019	Year-2	0	0	Summer
7/15/2019	FP	12M	7	2019	Year-2	1	0	Summer
7/15/2019	FP	14M	7	2019	Year-2	1	0	Summer
7/15/2019	FP	8A	7	2019	Year-2	0	0	Summer
7/15/2019	FP	9A	7	2019	Year-2	0	0	Summer
7/15/2019	FP	11A	7	2019	Year-2	1	0	Summer
7/15/2019	FP	13A	7	2019	Year-2	1	0	Summer
7/16/2019	FP	1M	7	2019	Year-2	1	0	Summer

Date	Species	Site	Month	Year	Survey Year	Presence	Data Gap	Season
7/16/2019	FP	2M	7	2019	Year-2	1	0	Summer
7/16/2019	FP	3M	7	2019	Year-2	1	0	Summer
7/16/2019	FP	4M	7	2019	Year-2	1	0	Summer
7/16/2019	FP	5M	7	2019	Year-2	0	0	Summer
7/16/2019	FP	6M	7	2019	Year-2		1	Summer
7/16/2019	FP	7M	7	2019	Year-2	0	0	Summer
7/16/2019	FP	10M	7	2019	Year-2	0	0	Summer
7/16/2019	FP	12M	7	2019	Year-2	1	0	Summer
7/16/2019	FP	14M	7	2019	Year-2	1	0	Summer
7/16/2019	FP	8A	7	2019	Year-2	0	0	Summer
7/16/2019	FP	9A	7	2019	Year-2	0	0	Summer
7/16/2019	FP	11A	7	2019	Year-2	0	0	Summer
7/16/2019	FP	13A	7	2019	Year-2	1	0	Summer
7/17/2019	FP	1M	7	2019	Year-2	0	0	Summer
7/17/2019	FP	2M	7	2019	Year-2	1	0	Summer
7/17/2019	FP	3M	7	2019	Year-2	1	0	Summer
7/17/2019	FP	4M	7	2019	Year-2	1	0	Summer
7/17/2019	FP	5M	7	2019	Year-2	0	0	Summer
7/17/2019	FP	6M	7	2019	Year-2		1	Summer
7/17/2019	FP	7M	7	2019	Year-2	0	0	Summer
7/17/2019	FP	10M	7	2019	Year-2	0	0	Summer
7/17/2019	FP	12M	7	2019	Year-2	1	0	Summer
7/17/2019	FP	14M	7	2019	Year-2	1	0	Summer
7/17/2019	FP	8A	7	2019	Year-2	0	0	Summer
7/17/2019	FP	9A	7	2019	Year-2	0	0	Summer
7/17/2019	FP	11A	7	2019	Year-2	1	0	Summer
7/17/2019	FP	13A	7	2019	Year-2	1	0	Summer
7/18/2019	FP	1M	7	2019	Year-2	1	0	Summer
7/18/2019	FP	2M	7	2019	Year-2	1	0	Summer
7/18/2019	FP	3M	7	2019	Year-2	1	0	Summer
7/18/2019	FP	4M	7	2019	Year-2	1	0	Summer
7/18/2019	FP	5M	7	2019	Year-2	0	0	Summer
7/18/2019	FP	6M	7	2019	Year-2		1	Summer
7/18/2019	FP	7M	7	2019	Year-2	0	0	Summer
7/18/2019	FP	10M	7	2019	Year-2	1	0	Summer
7/18/2019	FP	12M	7	2019	Year-2	1	0	Summer
7/18/2019	FP	14M	7	2019	Year-2	1	0	Summer
7/18/2019	FP	8A	7	2019	Year-2	0	0	Summer
7/18/2019	FP	9A	7	2019	Year-2	0	0	Summer
7/18/2019	FP	11A	7	2019	Year-2	1	0	Summer
7/18/2019	FP	13A	7	2019	Year-2	1	0	Summer
7/19/2019	FP	1M	7	2019	Year-2	1	0	Summer
7/19/2019	FP	2M	7	2019	Year-2	1	0	Summer
7/19/2019	FP	3M	7	2019	Year-2	1	0	Summer

Date	Species	Site	Month	Year	Survey Year	Presence	Data Gap	Season
7/19/2019	FP	4M	7	2019	Year-2	1	0	Summer
7/19/2019	FP	5M	7	2019	Year-2	0	0	Summer
7/19/2019	FP	6M	7	2019	Year-2		1	Summer
7/19/2019	FP	7M	7	2019	Year-2	0	0	Summer
7/19/2019	FP	10M	7	2019	Year-2	0	0	Summer
7/19/2019	FP	12M	7	2019	Year-2	0	0	Summer
7/19/2019	FP	14M	7	2019	Year-2	1	0	Summer
7/19/2019	FP	8A	7	2019	Year-2	0	0	Summer
7/19/2019	FP	9A	7	2019	Year-2	0	0	Summer
7/19/2019	FP	11A	7	2019	Year-2	0	0	Summer
7/19/2019	FP	13A	7	2019	Year-2	0	0	Summer
7/20/2019	FP	1M	7	2019	Year-2	0	0	Summer
7/20/2019	FP	2M	7	2019	Year-2	1	0	Summer
7/20/2019	FP	3M	7	2019	Year-2	1	0	Summer
7/20/2019	FP	4M	7	2019	Year-2	1	0	Summer
7/20/2019	FP	5M	7	2019	Year-2	0	0	Summer
7/20/2019	FP	6M	7	2019	Year-2		1	Summer
7/20/2019	FP	7M	7	2019	Year-2	0	0	Summer
7/20/2019	FP	10M	7	2019	Year-2	1	0	Summer
7/20/2019	FP	12M	7	2019	Year-2	1	0	Summer
7/20/2019	FP	14M	7	2019	Year-2	1	0	Summer
7/20/2019	FP	8A	7	2019	Year-2	0	0	Summer
7/20/2019	FP	9A	7	2019	Year-2	0	0	Summer
7/20/2019	FP	11A	7	2019	Year-2	1	0	Summer
7/20/2019	FP	13A	7	2019	Year-2	0	0	Summer
7/21/2019	FP	1M	7	2019	Year-2	0	0	Summer
7/21/2019	FP	2M	7	2019	Year-2	0	0	Summer
7/21/2019	FP	3M	7	2019	Year-2	1	0	Summer
7/21/2019	FP	4M	7	2019	Year-2	1	0	Summer
7/21/2019	FP	5M	7	2019	Year-2	1	0	Summer
7/21/2019	FP	6M	7	2019	Year-2		1	Summer
7/21/2019	FP	7M	7	2019	Year-2	0	0	Summer
7/21/2019	FP	10M	7	2019	Year-2	0	0	Summer
7/21/2019	FP	12M	7	2019	Year-2	1	0	Summer
7/21/2019	FP	14M	7	2019	Year-2	1	0	Summer
7/21/2019	FP	8A	7	2019	Year-2	0	0	Summer
7/21/2019	FP	9A	7	2019	Year-2	0	0	Summer
7/21/2019	FP	11A	7	2019	Year-2	0	0	Summer
7/21/2019	FP	13A	7	2019	Year-2	0	0	Summer
7/22/2019	FP	1M	7	2019	Year-2	1	0	Summer
7/22/2019	FP	2M	7	2019	Year-2	1	0	Summer
7/22/2019	FP	3M	7	2019	Year-2	1	0	Summer
7/22/2019	FP	4M	7	2019	Year-2	1	0	Summer
7/22/2019	FP	5M	7	2019	Year-2	1	0	Summer

Date	Species	Site	Month	Year	Survey Year	Presence	Data Gap	Season
7/22/2019	FP	6M	7	2019	Year-2		1	Summer
7/22/2019	FP	7M	7	2019	Year-2	0	0	Summer
7/22/2019	FP	10M	7	2019	Year-2	0	0	Summer
7/22/2019	FP	12M	7	2019	Year-2	1	0	Summer
7/22/2019	FP	14M	7	2019	Year-2	1	0	Summer
7/22/2019	FP	8A	7	2019	Year-2	0	0	Summer
7/22/2019	FP	9A	7	2019	Year-2	1	0	Summer
7/22/2019	FP	11A	7	2019	Year-2	1	0	Summer
7/22/2019	FP	13A	7	2019	Year-2	0	0	Summer
7/23/2019	FP	1M	7	2019	Year-2	0	0	Summer
7/23/2019	FP	2M	7	2019	Year-2	0	0	Summer
7/23/2019	FP	3M	7	2019	Year-2	1	0	Summer
7/23/2019	FP	4M	7	2019	Year-2	1	0	Summer
7/23/2019	FP	5M	7	2019	Year-2	1	0	Summer
7/23/2019	FP	6M	7	2019	Year-2		1	Summer
7/23/2019	FP	7M	7	2019	Year-2	0	0	Summer
7/23/2019	FP	10M	7	2019	Year-2	1	0	Summer
7/23/2019	FP	12M	7	2019	Year-2	1	0	Summer
7/23/2019	FP	14M	7	2019	Year-2	1	0	Summer
7/23/2019	FP	8A	7	2019	Year-2	0	0	Summer
7/23/2019	FP	9A	7	2019	Year-2	0	0	Summer
7/23/2019	FP	11A	7	2019	Year-2	1	0	Summer
7/23/2019	FP	13A	7	2019	Year-2	1	0	Summer
7/24/2019	FP	1M	7	2019	Year-2	0	0	Summer
7/24/2019	FP	2M	7	2019	Year-2	1	0	Summer
7/24/2019	FP	3M	7	2019	Year-2	1	0	Summer
7/24/2019	FP	4M	7	2019	Year-2	1	0	Summer
7/24/2019	FP	5M	7	2019	Year-2	0	0	Summer
7/24/2019	FP	6M	7	2019	Year-2		1	Summer
7/24/2019	FP	7M	7	2019	Year-2	0	0	Summer
7/24/2019	FP	10M	7	2019	Year-2	0	0	Summer
7/24/2019	FP	12M	7	2019	Year-2	1	0	Summer
7/24/2019	FP	14M	7	2019	Year-2	1	0	Summer
7/24/2019	FP	8A	7	2019	Year-2	0	0	Summer
7/24/2019	FP	9A	7	2019	Year-2	0	0	Summer
7/24/2019	FP	11A	7	2019	Year-2	1	0	Summer
7/24/2019	FP	13A	7	2019	Year-2	0	0	Summer
7/25/2019	FP	1M	7	2019	Year-2	0	0	Summer
7/25/2019	FP	2M	7	2019	Year-2	0	0	Summer
7/25/2019	FP	3M	7	2019	Year-2	0	0	Summer
7/25/2019	FP	4M	7	2019	Year-2	1	0	Summer
7/25/2019	FP	5M	7	2019	Year-2	0	0	Summer
7/25/2019	FP	6M	7	2019	Year-2		1	Summer
7/25/2019	FP	7M	7	2019	Year-2	0	0	Summer

Date	Species	Site	Month	Year	Survey Year	Presence	Data Gap	Season
7/25/2019	FP	10M	7	2019	Year-2	0	0	Summer
7/25/2019	FP	12M	7	2019	Year-2	1	0	Summer
7/25/2019	FP	14M	7	2019	Year-2	1	0	Summer
7/25/2019	FP	8A	7	2019	Year-2	0	0	Summer
7/25/2019	FP	9A	7	2019	Year-2	1	0	Summer
7/25/2019	FP	11A	7	2019	Year-2	1	0	Summer
7/25/2019	FP	13A	7	2019	Year-2	0	0	Summer
7/26/2019	FP	1M	7	2019	Year-2	0	0	Summer
7/26/2019	FP	2M	7	2019	Year-2	1	0	Summer
7/26/2019	FP	3M	7	2019	Year-2	1	0	Summer
7/26/2019	FP	4M	7	2019	Year-2	1	0	Summer
7/26/2019	FP	5M	7	2019	Year-2	0	0	Summer
7/26/2019	FP	6M	7	2019	Year-2		1	Summer
7/26/2019	FP	7M	7	2019	Year-2	0	0	Summer
7/26/2019	FP	10M	7	2019	Year-2	1	0	Summer
7/26/2019	FP	12M	7	2019	Year-2	1	0	Summer
7/26/2019	FP	14M	7	2019	Year-2	1	0	Summer
7/26/2019	FP	8A	7	2019	Year-2	0	0	Summer
7/26/2019	FP	9A	7	2019	Year-2	0	0	Summer
7/26/2019	FP	11A	7	2019	Year-2	1	0	Summer
7/26/2019	FP	13A	7	2019	Year-2	0	0	Summer
7/27/2019	FP	1M	7	2019	Year-2	0	0	Summer
7/27/2019	FP	2M	7	2019	Year-2	1	0	Summer
7/27/2019	FP	3M	7	2019	Year-2	1	0	Summer
7/27/2019	FP	4M	7	2019	Year-2	1	0	Summer
7/27/2019	FP	5M	7	2019	Year-2	1	0	Summer
7/27/2019	FP	6M	7	2019	Year-2		1	Summer
7/27/2019	FP	7M	7	2019	Year-2	0	0	Summer
7/27/2019	FP	10M	7	2019	Year-2	0	0	Summer
7/27/2019	FP	12M	7	2019	Year-2	1	0	Summer
7/27/2019	FP	14M	7	2019	Year-2	0	0	Summer
7/27/2019	FP	8A	7	2019	Year-2	0	0	Summer
7/27/2019	FP	9A	7	2019	Year-2	0	0	Summer
7/27/2019	FP	11A	7	2019	Year-2	1	0	Summer
7/27/2019	FP	13A	7	2019	Year-2	0	0	Summer
7/28/2019	FP	1M	7	2019	Year-2	0	0	Summer
7/28/2019	FP	2M	7	2019	Year-2	0	0	Summer
7/28/2019	FP	3M	7	2019	Year-2	1	0	Summer
7/28/2019	FP	4M	7	2019	Year-2	1	0	Summer
7/28/2019	FP	5M	7	2019	Year-2	1	0	Summer
7/28/2019	FP	6M	7	2019	Year-2		1	Summer
7/28/2019	FP	7M	7	2019	Year-2	0	0	Summer
7/28/2019	FP	10M	7	2019	Year-2	1	0	Summer
7/28/2019	FP	12M	7	2019	Year-2	1	0	Summer

Date	Species	Site	Month	Year	Survey Year	Presence	Data Gap	Season
7/28/2019	FP	14M	7	2019	Year-2	1	0	Summer
7/28/2019	FP	8A	7	2019	Year-2	0	0	Summer
7/28/2019	FP	9A	7	2019	Year-2	0	0	Summer
7/28/2019	FP	11A	7	2019	Year-2	1	0	Summer
7/28/2019	FP	13A	7	2019	Year-2	1	0	Summer
7/29/2019	FP	1M	7	2019	Year-2	1	0	Summer
7/29/2019	FP	2M	7	2019	Year-2	1	0	Summer
7/29/2019	FP	3M	7	2019	Year-2	1	0	Summer
7/29/2019	FP	4M	7	2019	Year-2	1	0	Summer
7/29/2019	FP	5M	7	2019	Year-2	1	0	Summer
7/29/2019	FP	6M	7	2019	Year-2		1	Summer
7/29/2019	FP	7M	7	2019	Year-2	0	0	Summer
7/29/2019	FP	10M	7	2019	Year-2	1	0	Summer
7/29/2019	FP	12M	7	2019	Year-2	1	0	Summer
7/29/2019	FP	14M	7	2019	Year-2	1	0	Summer
7/29/2019	FP	8A	7	2019	Year-2	0	0	Summer
7/29/2019	FP	9A	7	2019	Year-2	1	0	Summer
7/29/2019	FP	11A	7	2019	Year-2	1	0	Summer
7/29/2019	FP	13A	7	2019	Year-2	1	0	Summer
7/30/2019	FP	1M	7	2019	Year-2	1	0	Summer
7/30/2019	FP	2M	7	2019	Year-2	1	0	Summer
7/30/2019	FP	3M	7	2019	Year-2	1	0	Summer
7/30/2019	FP	4M	7	2019	Year-2	1	0	Summer
7/30/2019	FP	5M	7	2019	Year-2	1	0	Summer
7/30/2019	FP	6M	7	2019	Year-2		1	Summer
7/30/2019	FP	7M	7	2019	Year-2	0	0	Summer
7/30/2019	FP	10M	7	2019	Year-2	1	0	Summer
7/30/2019	FP	12M	7	2019	Year-2	1	0	Summer
7/30/2019	FP	14M	7	2019	Year-2	0	0	Summer
7/30/2019	FP	8A	7	2019	Year-2	0	0	Summer
7/30/2019	FP	9A	7	2019	Year-2	0	0	Summer
7/30/2019	FP	11A	7	2019	Year-2	1	0	Summer
7/30/2019	FP	13A	7	2019	Year-2	1	0	Summer
7/31/2019	FP	1M	7	2019	Year-2	0	0	Summer
7/31/2019	FP	2M	7	2019	Year-2	1	0	Summer
7/31/2019	FP	3M	7	2019	Year-2	1	0	Summer
7/31/2019	FP	4M	7	2019	Year-2	1	0	Summer
7/31/2019	FP	5M	7	2019	Year-2	1	0	Summer
7/31/2019	FP	6M	7	2019	Year-2		1	Summer
7/31/2019	FP	7M	7	2019	Year-2	0	0	Summer
7/31/2019	FP	10M	7	2019	Year-2	1	0	Summer
7/31/2019	FP	12M	7	2019	Year-2	1	0	Summer
7/31/2019	FP	14M	7	2019	Year-2	1	0	Summer
7/31/2019	FP	8A	7	2019	Year-2	0	0	Summer

Date	Species	Site	Month	Year	Survey Year	Presence	Data Gap	Season
7/31/2019	FP	9A	7	2019	Year-2	0	0	Summer
7/31/2019	FP	11A	7	2019	Year-2	1	0	Summer
7/31/2019	FP	13A	7	2019	Year-2	1	0	Summer
8/1/2019	FP	1M	8	2019	Year-2	0	0	Summer
8/1/2019	FP	2M	8	2019	Year-2	0	0	Summer
8/1/2019	FP	3M	8	2019	Year-2	1	0	Summer
8/1/2019	FP	4M	8	2019	Year-2	1	0	Summer
8/1/2019	FP	5M	8	2019	Year-2	1	0	Summer
8/1/2019	FP	6M	8	2019	Year-2		1	Summer
8/1/2019	FP	7M	8	2019	Year-2	0	0	Summer
8/1/2019	FP	10M	8	2019	Year-2	1	0	Summer
8/1/2019	FP	12M	8	2019	Year-2	1	0	Summer
8/1/2019	FP	14M	8	2019	Year-2	1	0	Summer
8/1/2019	FP	8A	8	2019	Year-2	0	0	Summer
8/1/2019	FP	9A	8	2019	Year-2	0	0	Summer
8/1/2019	FP	11A	8	2019	Year-2	1	0	Summer
8/1/2019	FP	13A	8	2019	Year-2	1	0	Summer
8/2/2019	FP	1M	8	2019	Year-2	0	0	Summer
8/2/2019	FP	2M	8	2019	Year-2	0	0	Summer
8/2/2019	FP	3M	8	2019	Year-2	0	0	Summer
8/2/2019	FP	4M	8	2019	Year-2	0	0	Summer
8/2/2019	FP	5M	8	2019	Year-2	0	0	Summer
8/2/2019	FP	6M	8	2019	Year-2		1	Summer
8/2/2019	FP	7M	8	2019	Year-2	0	0	Summer
8/2/2019	FP	10M	8	2019	Year-2		1	Summer
8/2/2019	FP	12M	8	2019	Year-2		1	Summer
8/2/2019	FP	14M	8	2019	Year-2		1	Summer
8/2/2019	FP	8A	8	2019	Year-2	0	0	Summer
8/2/2019	FP	9A	8	2019	Year-2	0	0	Summer
8/2/2019	FP	11A	8	2019	Year-2	1	0	Summer
8/2/2019	FP	13A	8	2019	Year-2	1	0	Summer
8/3/2019	FP	1M	8	2019	Year-2	0	0	Summer
8/3/2019	FP	2M	8	2019	Year-2	0	0	Summer
8/3/2019	FP	3M	8	2019	Year-2	0	0	Summer
8/3/2019	FP	4M	8	2019	Year-2	0	0	Summer
8/3/2019	FP	5M	8	2019	Year-2		1	Summer
8/3/2019	FP	6M	8	2019	Year-2		1	Summer
8/3/2019	FP	7M	8	2019	Year-2		1	Summer
8/3/2019	FP	10M	8	2019	Year-2	0	0	Summer
8/3/2019	FP	12M	8	2019	Year-2	0	0	Summer
8/3/2019	FP	14M	8	2019	Year-2		1	Summer
8/3/2019	FP	8A	8	2019	Year-2	0	0	Summer
8/3/2019	FP	9A	8	2019	Year-2	0	0	Summer
8/3/2019	FP	11A	8	2019	Year-2	1	0	Summer

Date	Species	Site	Month	Year	Survey Year	Presence	Data Gap	Season
8/3/2019	FP	13A	8	2019	Year-2	1	0	Summer
8/4/2019	FP	1M	8	2019	Year-2		1	Summer
8/4/2019	FP	2M	8	2019	Year-2		1	Summer
8/4/2019	FP	3M	8	2019	Year-2		1	Summer
8/4/2019	FP	4M	8	2019	Year-2		1	Summer
8/4/2019	FP	5M	8	2019	Year-2	0	0	Summer
8/4/2019	FP	6M	8	2019	Year-2	0	0	Summer
8/4/2019	FP	7M	8	2019	Year-2	0	0	Summer
8/4/2019	FP	10M	8	2019	Year-2	0	0	Summer
8/4/2019	FP	12M	8	2019	Year-2	0	0	Summer
8/4/2019	FP	14M	8	2019	Year-2		1	Summer
8/4/2019	FP	8A	8	2019	Year-2	0	0	Summer
8/4/2019	FP	9A	8	2019	Year-2	0	0	Summer
8/4/2019	FP	11A	8	2019	Year-2	1	0	Summer
8/4/2019	FP	13A	8	2019	Year-2	1	0	Summer
8/5/2019	FP	1M	8	2019	Year-2	1	0	Summer
8/5/2019	FP	2M	8	2019	Year-2	1	0	Summer
8/5/2019	FP	3M	8	2019	Year-2	1	0	Summer
8/5/2019	FP	4M	8	2019	Year-2	1	0	Summer
8/5/2019	FP	5M	8	2019	Year-2	1	0	Summer
8/5/2019	FP	6M	8	2019	Year-2	1	0	Summer
8/5/2019	FP	7M	8	2019	Year-2	0	0	Summer
8/5/2019	FP	10M	8	2019	Year-2	1	0	Summer
8/5/2019	FP	12M	8	2019	Year-2	1	0	Summer
8/5/2019	FP	14M	8	2019	Year-2		1	Summer
8/5/2019	FP	8A	8	2019	Year-2	0	0	Summer
8/5/2019	FP	9A	8	2019	Year-2	0	0	Summer
8/5/2019	FP	11A	8	2019	Year-2	1	0	Summer
8/5/2019	FP	13A	8	2019	Year-2	1	0	Summer
8/6/2019	FP	1M	8	2019	Year-2	1	0	Summer
8/6/2019	FP	2M	8	2019	Year-2	1	0	Summer
8/6/2019	FP	3M	8	2019	Year-2	1	0	Summer
8/6/2019	FP	4M	8	2019	Year-2	1	0	Summer
8/6/2019	FP	5M	8	2019	Year-2	1	0	Summer
8/6/2019	FP	6M	8	2019	Year-2	1	0	Summer
8/6/2019	FP	7M	8	2019	Year-2	0	0	Summer
8/6/2019	FP	10M	8	2019	Year-2	1	0	Summer
8/6/2019	FP	12M	8	2019	Year-2	1	0	Summer
8/6/2019	FP	14M	8	2019	Year-2		1	Summer
8/6/2019	FP	8A	8	2019	Year-2	0	0	Summer
8/6/2019	FP	9A	8	2019	Year-2	0	0	Summer
8/6/2019	FP	11A	8	2019	Year-2	1	0	Summer
8/6/2019	FP	13A	8	2019	Year-2	1	0	Summer
8/7/2019	FP	1M	8	2019	Year-2	1	0	Summer

Date	Species	Site	Month	Year	Survey Year	Presence	Data Gap	Season
8/7/2019	FP	2M	8	2019	Year-2	1	0	Summer
8/7/2019	FP	3M	8	2019	Year-2	1	0	Summer
8/7/2019	FP	4M	8	2019	Year-2	1	0	Summer
8/7/2019	FP	5M	8	2019	Year-2	1	0	Summer
8/7/2019	FP	6M	8	2019	Year-2	1	0	Summer
8/7/2019	FP	7M	8	2019	Year-2	0	0	Summer
8/7/2019	FP	10M	8	2019	Year-2	1	0	Summer
8/7/2019	FP	12M	8	2019	Year-2	1	0	Summer
8/7/2019	FP	14M	8	2019	Year-2		1	Summer
8/7/2019	FP	8A	8	2019	Year-2	0	0	Summer
8/7/2019	FP	9A	8	2019	Year-2	0	0	Summer
8/7/2019	FP	11A	8	2019	Year-2	1	0	Summer
8/7/2019	FP	13A	8	2019	Year-2	1	0	Summer
8/8/2019	FP	1M	8	2019	Year-2	1	0	Summer
8/8/2019	FP	2M	8	2019	Year-2	1	0	Summer
8/8/2019	FP	3M	8	2019	Year-2	1	0	Summer
8/8/2019	FP	4M	8	2019	Year-2	1	0	Summer
8/8/2019	FP	5M	8	2019	Year-2	1	0	Summer
8/8/2019	FP	6M	8	2019	Year-2	1	0	Summer
8/8/2019	FP	7M	8	2019	Year-2	0	0	Summer
8/8/2019	FP	10M	8	2019	Year-2	1	0	Summer
8/8/2019	FP	12M	8	2019	Year-2	1	0	Summer
8/8/2019	FP	14M	8	2019	Year-2		1	Summer
8/8/2019	FP	8A	8	2019	Year-2	0	0	Summer
8/8/2019	FP	9A	8	2019	Year-2	0	0	Summer
8/8/2019	FP	11A	8	2019	Year-2	1	0	Summer
8/8/2019	FP	13A	8	2019	Year-2	1	0	Summer
8/9/2019	FP	1M	8	2019	Year-2	1	0	Summer
8/9/2019	FP	2M	8	2019	Year-2	1	0	Summer
8/9/2019	FP	3M	8	2019	Year-2	1	0	Summer
8/9/2019	FP	4M	8	2019	Year-2	1	0	Summer
8/9/2019	FP	5M	8	2019	Year-2	1	0	Summer
8/9/2019	FP	6M	8	2019	Year-2	1	0	Summer
8/9/2019	FP	7M	8	2019	Year-2	0	0	Summer
8/9/2019	FP	10M	8	2019	Year-2	1	0	Summer
8/9/2019	FP	12M	8	2019	Year-2	1	0	Summer
8/9/2019	FP	14M	8	2019	Year-2		1	Summer
8/9/2019	FP	8A	8	2019	Year-2	0	0	Summer
8/9/2019	FP	9A	8	2019	Year-2	0	0	Summer
8/9/2019	FP	11A	8	2019	Year-2	1	0	Summer
8/9/2019	FP	13A	8	2019	Year-2	1	0	Summer
8/10/2019	FP	1M	8	2019	Year-2	1	0	Summer
8/10/2019	FP	2M	8	2019	Year-2	1	0	Summer
8/10/2019	FP	3M	8	2019	Year-2	1	0	Summer

Date	Species	Site	Month	Year	Survey Year	Presence	Data Gap	Season
8/10/2019	FP	4M	8	2019	Year-2	1	0	Summer
8/10/2019	FP	5M	8	2019	Year-2	1	0	Summer
8/10/2019	FP	6M	8	2019	Year-2	1	0	Summer
8/10/2019	FP	7M	8	2019	Year-2	0	0	Summer
8/10/2019	FP	10M	8	2019	Year-2	1	0	Summer
8/10/2019	FP	12M	8	2019	Year-2	1	0	Summer
8/10/2019	FP	14M	8	2019	Year-2		1	Summer
8/10/2019	FP	8A	8	2019	Year-2	0	0	Summer
8/10/2019	FP	9A	8	2019	Year-2	0	0	Summer
8/10/2019	FP	11A	8	2019	Year-2	1	0	Summer
8/10/2019	FP	13A	8	2019	Year-2	1	0	Summer
8/11/2019	FP	1M	8	2019	Year-2	0	0	Summer
8/11/2019	FP	2M	8	2019	Year-2	1	0	Summer
8/11/2019	FP	3M	8	2019	Year-2	1	0	Summer
8/11/2019	FP	4M	8	2019	Year-2	1	0	Summer
8/11/2019	FP	5M	8	2019	Year-2	1	0	Summer
8/11/2019	FP	6M	8	2019	Year-2	1	0	Summer
8/11/2019	FP	7M	8	2019	Year-2	0	0	Summer
8/11/2019	FP	10M	8	2019	Year-2	1	0	Summer
8/11/2019	FP	12M	8	2019	Year-2	1	0	Summer
8/11/2019	FP	14M	8	2019	Year-2		1	Summer
8/11/2019	FP	8A	8	2019	Year-2	0	0	Summer
8/11/2019	FP	9A	8	2019	Year-2	0	0	Summer
8/11/2019	FP	11A	8	2019	Year-2	1	0	Summer
8/11/2019	FP	13A	8	2019	Year-2	1	0	Summer
8/12/2019	FP	1M	8	2019	Year-2	1	0	Summer
8/12/2019	FP	2M	8	2019	Year-2	1	0	Summer
8/12/2019	FP	3M	8	2019	Year-2	1	0	Summer
8/12/2019	FP	4M	8	2019	Year-2	1	0	Summer
8/12/2019	FP	5M	8	2019	Year-2	1	0	Summer
8/12/2019	FP	6M	8	2019	Year-2	1	0	Summer
8/12/2019	FP	7M	8	2019	Year-2	0	0	Summer
8/12/2019	FP	10M	8	2019	Year-2	1	0	Summer
8/12/2019	FP	12M	8	2019	Year-2	1	0	Summer
8/12/2019	FP	14M	8	2019	Year-2		1	Summer
8/12/2019	FP	8A	8	2019	Year-2	0	0	Summer
8/12/2019	FP	9A	8	2019	Year-2	0	0	Summer
8/12/2019	FP	11A	8	2019	Year-2	1	0	Summer
8/12/2019	FP	13A	8	2019	Year-2	1	0	Summer
8/13/2019	FP	1M	8	2019	Year-2	1	0	Summer
8/13/2019	FP	2M	8	2019	Year-2	1	0	Summer
8/13/2019	FP	3M	8	2019	Year-2	1	0	Summer
8/13/2019	FP	4M	8	2019	Year-2	1	0	Summer
8/13/2019	FP	5M	8	2019	Year-2	1	0	Summer

Date	Species	Site	Month	Year	Survey Year	Presence	Data Gap	Season
8/13/2019	FP	6M	8	2019	Year-2	1	0	Summer
8/13/2019	FP	7M	8	2019	Year-2	1	0	Summer
8/13/2019	FP	10M	8	2019	Year-2	1	0	Summer
8/13/2019	FP	12M	8	2019	Year-2	1	0	Summer
8/13/2019	FP	14M	8	2019	Year-2		1	Summer
8/13/2019	FP	8A	8	2019	Year-2	0	0	Summer
8/13/2019	FP	9A	8	2019	Year-2	1	0	Summer
8/13/2019	FP	11A	8	2019	Year-2	1	0	Summer
8/13/2019	FP	13A	8	2019	Year-2	1	0	Summer
8/14/2019	FP	1M	8	2019	Year-2	1	0	Summer
8/14/2019	FP	2M	8	2019	Year-2	1	0	Summer
8/14/2019	FP	3M	8	2019	Year-2	1	0	Summer
8/14/2019	FP	4M	8	2019	Year-2	1	0	Summer
8/14/2019	FP	5M	8	2019	Year-2	1	0	Summer
8/14/2019	FP	6M	8	2019	Year-2	1	0	Summer
8/14/2019	FP	7M	8	2019	Year-2	0	0	Summer
8/14/2019	FP	10M	8	2019	Year-2	1	0	Summer
8/14/2019	FP	12M	8	2019	Year-2	1	0	Summer
8/14/2019	FP	14M	8	2019	Year-2		1	Summer
8/14/2019	FP	8A	8	2019	Year-2	0	0	Summer
8/14/2019	FP	9A	8	2019	Year-2	0	0	Summer
8/14/2019	FP	11A	8	2019	Year-2	1	0	Summer
8/14/2019	FP	13A	8	2019	Year-2	1	0	Summer
8/15/2019	FP	1M	8	2019	Year-2	1	0	Summer
8/15/2019	FP	2M	8	2019	Year-2	1	0	Summer
8/15/2019	FP	3M	8	2019	Year-2	1	0	Summer
8/15/2019	FP	4M	8	2019	Year-2	1	0	Summer
8/15/2019	FP	5M	8	2019	Year-2	1	0	Summer
8/15/2019	FP	6M	8	2019	Year-2	1	0	Summer
8/15/2019	FP	7M	8	2019	Year-2	0	0	Summer
8/15/2019	FP	10M	8	2019	Year-2	1	0	Summer
8/15/2019	FP	12M	8	2019	Year-2	1	0	Summer
8/15/2019	FP	14M	8	2019	Year-2		1	Summer
8/15/2019	FP	8A	8	2019	Year-2	0	0	Summer
8/15/2019	FP	9A	8	2019	Year-2	1	0	Summer
8/15/2019	FP	11A	8	2019	Year-2	1	0	Summer
8/15/2019	FP	13A	8	2019	Year-2	1	0	Summer
8/16/2019	FP	1M	8	2019	Year-2	1	0	Summer
8/16/2019	FP	2M	8	2019	Year-2	1	0	Summer
8/16/2019	FP	3M	8	2019	Year-2	1	0	Summer
8/16/2019	FP	4M	8	2019	Year-2	1	0	Summer
8/16/2019	FP	5M	8	2019	Year-2	1	0	Summer
8/16/2019	FP	6M	8	2019	Year-2	1	0	Summer
8/16/2019	FP	7M	8	2019	Year-2	1	0	Summer

Date	Species	Site	Month	Year	Survey Year	Presence	Data Gap	Season
8/16/2019	FP	10M	8	2019	Year-2	1	0	Summer
8/16/2019	FP	12M	8	2019	Year-2	1	0	Summer
8/16/2019	FP	14M	8	2019	Year-2		1	Summer
8/16/2019	FP	8A	8	2019	Year-2	0	0	Summer
8/16/2019	FP	9A	8	2019	Year-2	0	0	Summer
8/16/2019	FP	11A	8	2019	Year-2	1	0	Summer
8/16/2019	FP	13A	8	2019	Year-2	1	0	Summer
8/17/2019	FP	1M	8	2019	Year-2	1	0	Summer
8/17/2019	FP	2M	8	2019	Year-2	1	0	Summer
8/17/2019	FP	3M	8	2019	Year-2	1	0	Summer
8/17/2019	FP	4M	8	2019	Year-2	1	0	Summer
8/17/2019	FP	5M	8	2019	Year-2	1	0	Summer
8/17/2019	FP	6M	8	2019	Year-2	1	0	Summer
8/17/2019	FP	7M	8	2019	Year-2	0	0	Summer
8/17/2019	FP	10M	8	2019	Year-2	1	0	Summer
8/17/2019	FP	12M	8	2019	Year-2	1	0	Summer
8/17/2019	FP	14M	8	2019	Year-2		1	Summer
8/17/2019	FP	8A	8	2019	Year-2	0	0	Summer
8/17/2019	FP	9A	8	2019	Year-2	0	0	Summer
8/17/2019	FP	11A	8	2019	Year-2	1	0	Summer
8/17/2019	FP	13A	8	2019	Year-2	1	0	Summer
8/18/2019	FP	1M	8	2019	Year-2	1	0	Summer
8/18/2019	FP	2M	8	2019	Year-2	1	0	Summer
8/18/2019	FP	3M	8	2019	Year-2	1	0	Summer
8/18/2019	FP	4M	8	2019	Year-2	1	0	Summer
8/18/2019	FP	5M	8	2019	Year-2	1	0	Summer
8/18/2019	FP	6M	8	2019	Year-2	1	0	Summer
8/18/2019	FP	7M	8	2019	Year-2	0	0	Summer
8/18/2019	FP	10M	8	2019	Year-2	1	0	Summer
8/18/2019	FP	12M	8	2019	Year-2	1	0	Summer
8/18/2019	FP	14M	8	2019	Year-2		1	Summer
8/18/2019	FP	8A	8	2019	Year-2	0	0	Summer
8/18/2019	FP	9A	8	2019	Year-2	1	0	Summer
8/18/2019	FP	11A	8	2019	Year-2	1	0	Summer
8/18/2019	FP	13A	8	2019	Year-2	1	0	Summer
8/19/2019	FP	1M	8	2019	Year-2	1	0	Summer
8/19/2019	FP	2M	8	2019	Year-2	1	0	Summer
8/19/2019	FP	3M	8	2019	Year-2	1	0	Summer
8/19/2019	FP	4M	8	2019	Year-2	1	0	Summer
8/19/2019	FP	5M	8	2019	Year-2	1	0	Summer
8/19/2019	FP	6M	8	2019	Year-2	1	0	Summer
8/19/2019	FP	7M	8	2019	Year-2	0	0	Summer
8/19/2019	FP	10M	8	2019	Year-2	1	0	Summer
8/19/2019	FP	12M	8	2019	Year-2	1	0	Summer

Date	Species	Site	Month	Year	Survey Year	Presence	Data Gap	Season
8/19/2019	FP	14M	8	2019	Year-2		1	Summer
8/19/2019	FP	8A	8	2019	Year-2	0	0	Summer
8/19/2019	FP	9A	8	2019	Year-2	1	0	Summer
8/19/2019	FP	11A	8	2019	Year-2	1	0	Summer
8/19/2019	FP	13A	8	2019	Year-2	1	0	Summer
8/20/2019	FP	1M	8	2019	Year-2	1	0	Summer
8/20/2019	FP	2M	8	2019	Year-2	1	0	Summer
8/20/2019	FP	3M	8	2019	Year-2	1	0	Summer
8/20/2019	FP	4M	8	2019	Year-2	1	0	Summer
8/20/2019	FP	5M	8	2019	Year-2	1	0	Summer
8/20/2019	FP	6M	8	2019	Year-2	1	0	Summer
8/20/2019	FP	7M	8	2019	Year-2	0	0	Summer
8/20/2019	FP	10M	8	2019	Year-2	1	0	Summer
8/20/2019	FP	12M	8	2019	Year-2	1	0	Summer
8/20/2019	FP	14M	8	2019	Year-2		1	Summer
8/20/2019	FP	8A	8	2019	Year-2	0	0	Summer
8/20/2019	FP	9A	8	2019	Year-2	0	0	Summer
8/20/2019	FP	11A	8	2019	Year-2	1	0	Summer
8/20/2019	FP	13A	8	2019	Year-2	1	0	Summer
8/21/2019	FP	1M	8	2019	Year-2	1	0	Summer
8/21/2019	FP	2M	8	2019	Year-2	1	0	Summer
8/21/2019	FP	3M	8	2019	Year-2	1	0	Summer
8/21/2019	FP	4M	8	2019	Year-2	1	0	Summer
8/21/2019	FP	5M	8	2019	Year-2	1	0	Summer
8/21/2019	FP	6M	8	2019	Year-2	1	0	Summer
8/21/2019	FP	7M	8	2019	Year-2	1	0	Summer
8/21/2019	FP	10M	8	2019	Year-2	1	0	Summer
8/21/2019	FP	12M	8	2019	Year-2	1	0	Summer
8/21/2019	FP	14M	8	2019	Year-2		1	Summer
8/21/2019	FP	8A	8	2019	Year-2	0	0	Summer
8/21/2019	FP	9A	8	2019	Year-2	0	0	Summer
8/21/2019	FP	11A	8	2019	Year-2	1	0	Summer
8/21/2019	FP	13A	8	2019	Year-2	1	0	Summer
8/22/2019	FP	1M	8	2019	Year-2	1	0	Summer
8/22/2019	FP	2M	8	2019	Year-2	1	0	Summer
8/22/2019	FP	3M	8	2019	Year-2	1	0	Summer
8/22/2019	FP	4M	8	2019	Year-2	1	0	Summer
8/22/2019	FP	5M	8	2019	Year-2	1	0	Summer
8/22/2019	FP	6M	8	2019	Year-2	1	0	Summer
8/22/2019	FP	7M	8	2019	Year-2	0	0	Summer
8/22/2019	FP	10M	8	2019	Year-2	1	0	Summer
8/22/2019	FP	12M	8	2019	Year-2	1	0	Summer
8/22/2019	FP	14M	8	2019	Year-2		1	Summer
8/22/2019	FP	8A	8	2019	Year-2	0	0	Summer

Date	Species	Site	Month	Year	Survey Year	Presence	Data Gap	Season
8/22/2019	FP	9A	8	2019	Year-2	1	0	Summer
8/22/2019	FP	11A	8	2019	Year-2	1	0	Summer
8/22/2019	FP	13A	8	2019	Year-2	1	0	Summer
8/23/2019	FP	1M	8	2019	Year-2	1	0	Summer
8/23/2019	FP	2M	8	2019	Year-2	1	0	Summer
8/23/2019	FP	3M	8	2019	Year-2	1	0	Summer
8/23/2019	FP	4M	8	2019	Year-2	1	0	Summer
8/23/2019	FP	5M	8	2019	Year-2	1	0	Summer
8/23/2019	FP	6M	8	2019	Year-2	1	0	Summer
8/23/2019	FP	7M	8	2019	Year-2	0	0	Summer
8/23/2019	FP	10M	8	2019	Year-2	1	0	Summer
8/23/2019	FP	12M	8	2019	Year-2	1	0	Summer
8/23/2019	FP	14M	8	2019	Year-2		1	Summer
8/23/2019	FP	8A	8	2019	Year-2	0	0	Summer
8/23/2019	FP	9A	8	2019	Year-2	0	0	Summer
8/23/2019	FP	11A	8	2019	Year-2	1	0	Summer
8/23/2019	FP	13A	8	2019	Year-2	1	0	Summer
8/24/2019	FP	1M	8	2019	Year-2	1	0	Summer
8/24/2019	FP	2M	8	2019	Year-2	1	0	Summer
8/24/2019	FP	3M	8	2019	Year-2	1	0	Summer
8/24/2019	FP	4M	8	2019	Year-2	1	0	Summer
8/24/2019	FP	5M	8	2019	Year-2	1	0	Summer
8/24/2019	FP	6M	8	2019	Year-2	1	0	Summer
8/24/2019	FP	7M	8	2019	Year-2	0	0	Summer
8/24/2019	FP	10M	8	2019	Year-2	1	0	Summer
8/24/2019	FP	12M	8	2019	Year-2	1	0	Summer
8/24/2019	FP	14M	8	2019	Year-2		1	Summer
8/24/2019	FP	8A	8	2019	Year-2	0	0	Summer
8/24/2019	FP	9A	8	2019	Year-2	0	0	Summer
8/24/2019	FP	11A	8	2019	Year-2	1	0	Summer
8/24/2019	FP	13A	8	2019	Year-2	1	0	Summer
8/25/2019	FP	1M	8	2019	Year-2	1	0	Summer
8/25/2019	FP	2M	8	2019	Year-2	1	0	Summer
8/25/2019	FP	3M	8	2019	Year-2	1	0	Summer
8/25/2019	FP	4M	8	2019	Year-2	1	0	Summer
8/25/2019	FP	5M	8	2019	Year-2	1	0	Summer
8/25/2019	FP	6M	8	2019	Year-2	1	0	Summer
8/25/2019	FP	7M	8	2019	Year-2	0	0	Summer
8/25/2019	FP	10M	8	2019	Year-2	1	0	Summer
8/25/2019	FP	12M	8	2019	Year-2	1	0	Summer
8/25/2019	FP	14M	8	2019	Year-2		1	Summer
8/25/2019	FP	8A	8	2019	Year-2	0	0	Summer
8/25/2019	FP	9A	8	2019	Year-2	0	0	Summer
8/25/2019	FP	11A	8	2019	Year-2	1	0	Summer

Date	Species	Site	Month	Year	Survey Year	Presence	Data Gap	Season
8/25/2019	FP	13A	8	2019	Year-2	1	0	Summer
8/26/2019	FP	1M	8	2019	Year-2	1	0	Summer
8/26/2019	FP	2M	8	2019	Year-2	1	0	Summer
8/26/2019	FP	3M	8	2019	Year-2	1	0	Summer
8/26/2019	FP	4M	8	2019	Year-2	1	0	Summer
8/26/2019	FP	5M	8	2019	Year-2	1	0	Summer
8/26/2019	FP	6M	8	2019	Year-2	1	0	Summer
8/26/2019	FP	7M	8	2019	Year-2	0	0	Summer
8/26/2019	FP	10M	8	2019	Year-2	1	0	Summer
8/26/2019	FP	12M	8	2019	Year-2	1	0	Summer
8/26/2019	FP	14M	8	2019	Year-2		1	Summer
8/26/2019	FP	8A	8	2019	Year-2	0	0	Summer
8/26/2019	FP	9A	8	2019	Year-2	0	0	Summer
8/26/2019	FP	11A	8	2019	Year-2	1	0	Summer
8/26/2019	FP	13A	8	2019	Year-2	1	0	Summer
8/27/2019	FP	1M	8	2019	Year-2	1	0	Summer
8/27/2019	FP	2M	8	2019	Year-2	1	0	Summer
8/27/2019	FP	3M	8	2019	Year-2	1	0	Summer
8/27/2019	FP	4M	8	2019	Year-2	1	0	Summer
8/27/2019	FP	5M	8	2019	Year-2	1	0	Summer
8/27/2019	FP	6M	8	2019	Year-2	1	0	Summer
8/27/2019	FP	7M	8	2019	Year-2	0	0	Summer
8/27/2019	FP	10M	8	2019	Year-2	1	0	Summer
8/27/2019	FP	12M	8	2019	Year-2	1	0	Summer
8/27/2019	FP	14M	8	2019	Year-2		1	Summer
8/27/2019	FP	8A	8	2019	Year-2	0	0	Summer
8/27/2019	FP	9A	8	2019	Year-2	0	0	Summer
8/27/2019	FP	11A	8	2019	Year-2	1	0	Summer
8/27/2019	FP	13A	8	2019	Year-2	1	0	Summer
8/28/2019	FP	1M	8	2019	Year-2	1	0	Summer
8/28/2019	FP	2M	8	2019	Year-2	1	0	Summer
8/28/2019	FP	3M	8	2019	Year-2	1	0	Summer
8/28/2019	FP	4M	8	2019	Year-2	1	0	Summer
8/28/2019	FP	5M	8	2019	Year-2	1	0	Summer
8/28/2019	FP	6M	8	2019	Year-2	1	0	Summer
8/28/2019	FP	7M	8	2019	Year-2	1	0	Summer
8/28/2019	FP	10M	8	2019	Year-2	1	0	Summer
8/28/2019	FP	12M	8	2019	Year-2	1	0	Summer
8/28/2019	FP	14M	8	2019	Year-2		1	Summer
8/28/2019	FP	8A	8	2019	Year-2	0	0	Summer
8/28/2019	FP	9A	8	2019	Year-2	0	0	Summer
8/28/2019	FP	11A	8	2019	Year-2	1	0	Summer
8/28/2019	FP	13A	8	2019	Year-2	1	0	Summer
8/29/2019	FP	1M	8	2019	Year-2	1	0	Summer

Date	Species	Site	Month	Year	Survey Year	Presence	Data Gap	Season
8/29/2019	FP	2M	8	2019	Year-2	1	0	Summer
8/29/2019	FP	3M	8	2019	Year-2	1	0	Summer
8/29/2019	FP	4M	8	2019	Year-2	1	0	Summer
8/29/2019	FP	5M	8	2019	Year-2	1	0	Summer
8/29/2019	FP	6M	8	2019	Year-2	1	0	Summer
8/29/2019	FP	7M	8	2019	Year-2	0	0	Summer
8/29/2019	FP	10M	8	2019	Year-2	1	0	Summer
8/29/2019	FP	12M	8	2019	Year-2	1	0	Summer
8/29/2019	FP	14M	8	2019	Year-2		1	Summer
8/29/2019	FP	8A	8	2019	Year-2	0	0	Summer
8/29/2019	FP	9A	8	2019	Year-2	0	0	Summer
8/29/2019	FP	11A	8	2019	Year-2	1	0	Summer
8/29/2019	FP	13A	8	2019	Year-2	1	0	Summer
8/30/2019	FP	1M	8	2019	Year-2	1	0	Summer
8/30/2019	FP	2M	8	2019	Year-2	1	0	Summer
8/30/2019	FP	3M	8	2019	Year-2	1	0	Summer
8/30/2019	FP	4M	8	2019	Year-2	1	0	Summer
8/30/2019	FP	5M	8	2019	Year-2	1	0	Summer
8/30/2019	FP	6M	8	2019	Year-2	1	0	Summer
8/30/2019	FP	7M	8	2019	Year-2	0	0	Summer
8/30/2019	FP	10M	8	2019	Year-2	1	0	Summer
8/30/2019	FP	12M	8	2019	Year-2	1	0	Summer
8/30/2019	FP	14M	8	2019	Year-2		1	Summer
8/30/2019	FP	8A	8	2019	Year-2	0	0	Summer
8/30/2019	FP	9A	8	2019	Year-2	0	0	Summer
8/30/2019	FP	11A	8	2019	Year-2	1	0	Summer
8/30/2019	FP	13A	8	2019	Year-2	1	0	Summer
8/31/2019	FP	1M	8	2019	Year-2	1	0	Summer
8/31/2019	FP	2M	8	2019	Year-2	1	0	Summer
8/31/2019	FP	3M	8	2019	Year-2	1	0	Summer
8/31/2019	FP	4M	8	2019	Year-2	1	0	Summer
8/31/2019	FP	5M	8	2019	Year-2	1	0	Summer
8/31/2019	FP	6M	8	2019	Year-2	1	0	Summer
8/31/2019	FP	7M	8	2019	Year-2	1	0	Summer
8/31/2019	FP	10M	8	2019	Year-2	1	0	Summer
8/31/2019	FP	12M	8	2019	Year-2	1	0	Summer
8/31/2019	FP	14M	8	2019	Year-2		1	Summer
8/31/2019	FP	8A	8	2019	Year-2	0	0	Summer
8/31/2019	FP	9A	8	2019	Year-2	0	0	Summer
8/31/2019	FP	11A	8	2019	Year-2	1	0	Summer
8/31/2019	FP	13A	8	2019	Year-2	1	0	Summer
9/1/2019	FP	1M	9	2019	Year-2	1	0	Fall
9/1/2019	FP	2M	9	2019	Year-2	1	0	Fall
9/1/2019	FP	3M	9	2019	Year-2	1	0	Fall

Date	Species	Site	Month	Year	Survey Year	Presence	Data Gap	Season
9/1/2019	FP	4M	9	2019	Year-2	1	0	Fall
9/1/2019	FP	5M	9	2019	Year-2	1	0	Fall
9/1/2019	FP	6M	9	2019	Year-2	1	0	Fall
9/1/2019	FP	7M	9	2019	Year-2	0	0	Fall
9/1/2019	FP	10M	9	2019	Year-2	1	0	Fall
9/1/2019	FP	12M	9	2019	Year-2	1	0	Fall
9/1/2019	FP	14M	9	2019	Year-2		1	Fall
9/1/2019	FP	8A	9	2019	Year-2	1	0	Fall
9/1/2019	FP	9A	9	2019	Year-2	1	0	Fall
9/1/2019	FP	11A	9	2019	Year-2	1	0	Fall
9/1/2019	FP	13A	9	2019	Year-2	1	0	Fall
9/2/2019	FP	1M	9	2019	Year-2	1	0	Fall
9/2/2019	FP	2M	9	2019	Year-2	1	0	Fall
9/2/2019	FP	3M	9	2019	Year-2	1	0	Fall
9/2/2019	FP	4M	9	2019	Year-2	1	0	Fall
9/2/2019	FP	5M	9	2019	Year-2	1	0	Fall
9/2/2019	FP	6M	9	2019	Year-2	1	0	Fall
9/2/2019	FP	7M	9	2019	Year-2	0	0	Fall
9/2/2019	FP	10M	9	2019	Year-2	1	0	Fall
9/2/2019	FP	12M	9	2019	Year-2	1	0	Fall
9/2/2019	FP	14M	9	2019	Year-2		1	Fall
9/2/2019	FP	8A	9	2019	Year-2	0	0	Fall
9/2/2019	FP	9A	9	2019	Year-2	0	0	Fall
9/2/2019	FP	11A	9	2019	Year-2	1	0	Fall
9/2/2019	FP	13A	9	2019	Year-2	1	0	Fall
9/3/2019	FP	1M	9	2019	Year-2	1	0	Fall
9/3/2019	FP	2M	9	2019	Year-2	1	0	Fall
9/3/2019	FP	3M	9	2019	Year-2	1	0	Fall
9/3/2019	FP	4M	9	2019	Year-2	1	0	Fall
9/3/2019	FP	5M	9	2019	Year-2	1	0	Fall
9/3/2019	FP	6M	9	2019	Year-2	1	0	Fall
9/3/2019	FP	7M	9	2019	Year-2	1	0	Fall
9/3/2019	FP	10M	9	2019	Year-2	1	0	Fall
9/3/2019	FP	12M	9	2019	Year-2	1	0	Fall
9/3/2019	FP	14M	9	2019	Year-2		1	Fall
9/3/2019	FP	8A	9	2019	Year-2	0	0	Fall
9/3/2019	FP	9A	9	2019	Year-2	0	0	Fall
9/3/2019	FP	11A	9	2019	Year-2	1	0	Fall
9/3/2019	FP	13A	9	2019	Year-2	1	0	Fall
9/4/2019	FP	1M	9	2019	Year-2	1	0	Fall
9/4/2019	FP	2M	9	2019	Year-2	1	0	Fall
9/4/2019	FP	3M	9	2019	Year-2	1	0	Fall
9/4/2019	FP	4M	9	2019	Year-2	1	0	Fall
9/4/2019	FP	5M	9	2019	Year-2	1	0	Fall

Date	Species	Site	Month	Year	Survey Year	Presence	Data Gap	Season
9/4/2019	FP	6M	9	2019	Year-2	1	0	Fall
9/4/2019	FP	7M	9	2019	Year-2	0	0	Fall
9/4/2019	FP	10M	9	2019	Year-2	1	0	Fall
9/4/2019	FP	12M	9	2019	Year-2	1	0	Fall
9/4/2019	FP	14M	9	2019	Year-2		1	Fall
9/4/2019	FP	8A	9	2019	Year-2	0	0	Fall
9/4/2019	FP	9A	9	2019	Year-2	0	0	Fall
9/4/2019	FP	11A	9	2019	Year-2	1	0	Fall
9/4/2019	FP	13A	9	2019	Year-2	1	0	Fall
9/5/2019	FP	1M	9	2019	Year-2	1	0	Fall
9/5/2019	FP	2M	9	2019	Year-2	1	0	Fall
9/5/2019	FP	3M	9	2019	Year-2	1	0	Fall
9/5/2019	FP	4M	9	2019	Year-2	1	0	Fall
9/5/2019	FP	5M	9	2019	Year-2	1	0	Fall
9/5/2019	FP	6M	9	2019	Year-2	1	0	Fall
9/5/2019	FP	7M	9	2019	Year-2	0	0	Fall
9/5/2019	FP	10M	9	2019	Year-2	1	0	Fall
9/5/2019	FP	12M	9	2019	Year-2	1	0	Fall
9/5/2019	FP	14M	9	2019	Year-2		1	Fall
9/5/2019	FP	8A	9	2019	Year-2	0	0	Fall
9/5/2019	FP	9A	9	2019	Year-2	0	0	Fall
9/5/2019	FP	11A	9	2019	Year-2	1	0	Fall
9/5/2019	FP	13A	9	2019	Year-2	1	0	Fall
9/6/2019	FP	1M	9	2019	Year-2	1	0	Fall
9/6/2019	FP	2M	9	2019	Year-2	1	0	Fall
9/6/2019	FP	3M	9	2019	Year-2	1	0	Fall
9/6/2019	FP	4M	9	2019	Year-2	1	0	Fall
9/6/2019	FP	5M	9	2019	Year-2	1	0	Fall
9/6/2019	FP	6M	9	2019	Year-2	1	0	Fall
9/6/2019	FP	7M	9	2019	Year-2	0	0	Fall
9/6/2019	FP	10M	9	2019	Year-2	1	0	Fall
9/6/2019	FP	12M	9	2019	Year-2	1	0	Fall
9/6/2019	FP	14M	9	2019	Year-2		1	Fall
9/6/2019	FP	8A	9	2019	Year-2	1	0	Fall
9/6/2019	FP	9A	9	2019	Year-2	0	0	Fall
9/6/2019	FP	11A	9	2019	Year-2	1	0	Fall
9/6/2019	FP	13A	9	2019	Year-2	1	0	Fall
9/7/2019	FP	1M	9	2019	Year-2	1	0	Fall
9/7/2019	FP	2M	9	2019	Year-2	1	0	Fall
9/7/2019	FP	3M	9	2019	Year-2	1	0	Fall
9/7/2019	FP	4M	9	2019	Year-2	1	0	Fall
9/7/2019	FP	5M	9	2019	Year-2	1	0	Fall
9/7/2019	FP	6M	9	2019	Year-2	1	0	Fall
9/7/2019	FP	7M	9	2019	Year-2	0	0	Fall

Date	Species	Site	Month	Year	Survey Year	Presence	Data Gap	Season
9/7/2019	FP	10M	9	2019	Year-2	1	0	Fall
9/7/2019	FP	12M	9	2019	Year-2	1	0	Fall
9/7/2019	FP	14M	9	2019	Year-2		1	Fall
9/7/2019	FP	8A	9	2019	Year-2	1	0	Fall
9/7/2019	FP	9A	9	2019	Year-2	0	0	Fall
9/7/2019	FP	11A	9	2019	Year-2	1	0	Fall
9/7/2019	FP	13A	9	2019	Year-2	1	0	Fall
9/8/2019	FP	1M	9	2019	Year-2	1	0	Fall
9/8/2019	FP	2M	9	2019	Year-2	1	0	Fall
9/8/2019	FP	3M	9	2019	Year-2	1	0	Fall
9/8/2019	FP	4M	9	2019	Year-2	1	0	Fall
9/8/2019	FP	5M	9	2019	Year-2	1	0	Fall
9/8/2019	FP	6M	9	2019	Year-2	1	0	Fall
9/8/2019	FP	7M	9	2019	Year-2	0	0	Fall
9/8/2019	FP	10M	9	2019	Year-2	1	0	Fall
9/8/2019	FP	12M	9	2019	Year-2	1	0	Fall
9/8/2019	FP	14M	9	2019	Year-2		1	Fall
9/8/2019	FP	8A	9	2019	Year-2	0	0	Fall
9/8/2019	FP	9A	9	2019	Year-2	1	0	Fall
9/8/2019	FP	11A	9	2019	Year-2	1	0	Fall
9/8/2019	FP	13A	9	2019	Year-2	1	0	Fall
9/9/2019	FP	1M	9	2019	Year-2	1	0	Fall
9/9/2019	FP	2M	9	2019	Year-2	1	0	Fall
9/9/2019	FP	3M	9	2019	Year-2	1	0	Fall
9/9/2019	FP	4M	9	2019	Year-2	1	0	Fall
9/9/2019	FP	5M	9	2019	Year-2	1	0	Fall
9/9/2019	FP	6M	9	2019	Year-2	1	0	Fall
9/9/2019	FP	7M	9	2019	Year-2	1	0	Fall
9/9/2019	FP	10M	9	2019	Year-2	1	0	Fall
9/9/2019	FP	12M	9	2019	Year-2	1	0	Fall
9/9/2019	FP	14M	9	2019	Year-2		1	Fall
9/9/2019	FP	8A	9	2019	Year-2	0	0	Fall
9/9/2019	FP	9A	9	2019	Year-2	1	0	Fall
9/9/2019	FP	11A	9	2019	Year-2	1	0	Fall
9/9/2019	FP	13A	9	2019	Year-2	1	0	Fall
9/10/2019	FP	1M	9	2019	Year-2	1	0	Fall
9/10/2019	FP	2M	9	2019	Year-2	1	0	Fall
9/10/2019	FP	3M	9	2019	Year-2	1	0	Fall
9/10/2019	FP	4M	9	2019	Year-2	1	0	Fall
9/10/2019	FP	5M	9	2019	Year-2	1	0	Fall
9/10/2019	FP	6M	9	2019	Year-2	1	0	Fall
9/10/2019	FP	7M	9	2019	Year-2	0	0	Fall
9/10/2019	FP	10M	9	2019	Year-2	1	0	Fall
9/10/2019	FP	12M	9	2019	Year-2	1	0	Fall

Date	Species	Site	Month	Year	Survey Year	Presence	Data Gap	Season
9/10/2019	FP	14M	9	2019	Year-2		1	Fall
9/10/2019	FP	8A	9	2019	Year-2	0	0	Fall
9/10/2019	FP	9A	9	2019	Year-2	0	0	Fall
9/10/2019	FP	11A	9	2019	Year-2	1	0	Fall
9/10/2019	FP	13A	9	2019	Year-2	1	0	Fall
9/11/2019	FP	1M	9	2019	Year-2	1	0	Fall
9/11/2019	FP	2M	9	2019	Year-2	1	0	Fall
9/11/2019	FP	3M	9	2019	Year-2	1	0	Fall
9/11/2019	FP	4M	9	2019	Year-2	1	0	Fall
9/11/2019	FP	5M	9	2019	Year-2	1	0	Fall
9/11/2019	FP	6M	9	2019	Year-2	1	0	Fall
9/11/2019	FP	7M	9	2019	Year-2	0	0	Fall
9/11/2019	FP	10M	9	2019	Year-2	1	0	Fall
9/11/2019	FP	12M	9	2019	Year-2	1	0	Fall
9/11/2019	FP	14M	9	2019	Year-2		1	Fall
9/11/2019	FP	8A	9	2019	Year-2	0	0	Fall
9/11/2019	FP	9A	9	2019	Year-2	0	0	Fall
9/11/2019	FP	11A	9	2019	Year-2	1	0	Fall
9/11/2019	FP	13A	9	2019	Year-2	1	0	Fall
9/12/2019	FP	1M	9	2019	Year-2	1	0	Fall
9/12/2019	FP	2M	9	2019	Year-2	1	0	Fall
9/12/2019	FP	3M	9	2019	Year-2	1	0	Fall
9/12/2019	FP	4M	9	2019	Year-2	1	0	Fall
9/12/2019	FP	5M	9	2019	Year-2	1	0	Fall
9/12/2019	FP	6M	9	2019	Year-2	1	0	Fall
9/12/2019	FP	7M	9	2019	Year-2	0	0	Fall
9/12/2019	FP	10M	9	2019	Year-2	1	0	Fall
9/12/2019	FP	12M	9	2019	Year-2	1	0	Fall
9/12/2019	FP	14M	9	2019	Year-2		1	Fall
9/12/2019	FP	8A	9	2019	Year-2	1	0	Fall
9/12/2019	FP	9A	9	2019	Year-2	0	0	Fall
9/12/2019	FP	11A	9	2019	Year-2	1	0	Fall
9/12/2019	FP	13A	9	2019	Year-2	1	0	Fall
9/13/2019	FP	1M	9	2019	Year-2	1	0	Fall
9/13/2019	FP	2M	9	2019	Year-2	1	0	Fall
9/13/2019	FP	3M	9	2019	Year-2	1	0	Fall
9/13/2019	FP	4M	9	2019	Year-2	1	0	Fall
9/13/2019	FP	5M	9	2019	Year-2	1	0	Fall
9/13/2019	FP	6M	9	2019	Year-2	1	0	Fall
9/13/2019	FP	7M	9	2019	Year-2	0	0	Fall
9/13/2019	FP	10M	9	2019	Year-2	1	0	Fall
9/13/2019	FP	12M	9	2019	Year-2	1	0	Fall
9/13/2019	FP	14M	9	2019	Year-2		1	Fall
9/13/2019	FP	8A	9	2019	Year-2	0	0	Fall

Date	Species	Site	Month	Year	Survey Year	Presence	Data Gap	Season
9/13/2019	FP	9A	9	2019	Year-2	1	0	Fall
9/13/2019	FP	11A	9	2019	Year-2	1	0	Fall
9/13/2019	FP	13A	9	2019	Year-2	1	0	Fall
9/14/2019	FP	1M	9	2019	Year-2	1	0	Fall
9/14/2019	FP	2M	9	2019	Year-2	1	0	Fall
9/14/2019	FP	3M	9	2019	Year-2	1	0	Fall
9/14/2019	FP	4M	9	2019	Year-2	1	0	Fall
9/14/2019	FP	5M	9	2019	Year-2	1	0	Fall
9/14/2019	FP	6M	9	2019	Year-2	1	0	Fall
9/14/2019	FP	7M	9	2019	Year-2	0	0	Fall
9/14/2019	FP	10M	9	2019	Year-2	1	0	Fall
9/14/2019	FP	12M	9	2019	Year-2	1	0	Fall
9/14/2019	FP	14M	9	2019	Year-2		1	Fall
9/14/2019	FP	8A	9	2019	Year-2	0	0	Fall
9/14/2019	FP	9A	9	2019	Year-2	1	0	Fall
9/14/2019	FP	11A	9	2019	Year-2	1	0	Fall
9/14/2019	FP	13A	9	2019	Year-2	1	0	Fall
9/15/2019	FP	1M	9	2019	Year-2	1	0	Fall
9/15/2019	FP	2M	9	2019	Year-2	1	0	Fall
9/15/2019	FP	3M	9	2019	Year-2	1	0	Fall
9/15/2019	FP	4M	9	2019	Year-2	1	0	Fall
9/15/2019	FP	5M	9	2019	Year-2	1	0	Fall
9/15/2019	FP	6M	9	2019	Year-2	1	0	Fall
9/15/2019	FP	7M	9	2019	Year-2	0	0	Fall
9/15/2019	FP	10M	9	2019	Year-2	1	0	Fall
9/15/2019	FP	12M	9	2019	Year-2	1	0	Fall
9/15/2019	FP	14M	9	2019	Year-2		1	Fall
9/15/2019	FP	8A	9	2019	Year-2	0	0	Fall
9/15/2019	FP	9A	9	2019	Year-2	0	0	Fall
9/15/2019	FP	11A	9	2019	Year-2	1	0	Fall
9/15/2019	FP	13A	9	2019	Year-2	1	0	Fall
9/16/2019	FP	1M	9	2019	Year-2	1	0	Fall
9/16/2019	FP	2M	9	2019	Year-2	1	0	Fall
9/16/2019	FP	3M	9	2019	Year-2	1	0	Fall
9/16/2019	FP	4M	9	2019	Year-2	1	0	Fall
9/16/2019	FP	5M	9	2019	Year-2	1	0	Fall
9/16/2019	FP	6M	9	2019	Year-2	1	0	Fall
9/16/2019	FP	7M	9	2019	Year-2	0	0	Fall
9/16/2019	FP	10M	9	2019	Year-2	1	0	Fall
9/16/2019	FP	12M	9	2019	Year-2	1	0	Fall
9/16/2019	FP	14M	9	2019	Year-2		1	Fall
9/16/2019	FP	8A	9	2019	Year-2	0	0	Fall
9/16/2019	FP	9A	9	2019	Year-2	0	0	Fall
9/16/2019	FP	11A	9	2019	Year-2	1	0	Fall

Date	Species	Site	Month	Year	Survey Year	Presence	Data Gap	Season
9/16/2019	FP	13A	9	2019	Year-2	1	0	Fall
9/17/2019	FP	1M	9	2019	Year-2	1	0	Fall
9/17/2019	FP	2M	9	2019	Year-2	1	0	Fall
9/17/2019	FP	3M	9	2019	Year-2	1	0	Fall
9/17/2019	FP	4M	9	2019	Year-2	1	0	Fall
9/17/2019	FP	5M	9	2019	Year-2	1	0	Fall
9/17/2019	FP	6M	9	2019	Year-2	1	0	Fall
9/17/2019	FP	7M	9	2019	Year-2	1	0	Fall
9/17/2019	FP	10M	9	2019	Year-2	1	0	Fall
9/17/2019	FP	12M	9	2019	Year-2	1	0	Fall
9/17/2019	FP	14M	9	2019	Year-2		1	Fall
9/17/2019	FP	8A	9	2019	Year-2	0	0	Fall
9/17/2019	FP	9A	9	2019	Year-2	0	0	Fall
9/17/2019	FP	11A	9	2019	Year-2	1	0	Fall
9/17/2019	FP	13A	9	2019	Year-2	1	0	Fall
9/18/2019	FP	1M	9	2019	Year-2	1	0	Fall
9/18/2019	FP	2M	9	2019	Year-2	1	0	Fall
9/18/2019	FP	3M	9	2019	Year-2	1	0	Fall
9/18/2019	FP	4M	9	2019	Year-2	1	0	Fall
9/18/2019	FP	5M	9	2019	Year-2	1	0	Fall
9/18/2019	FP	6M	9	2019	Year-2	1	0	Fall
9/18/2019	FP	7M	9	2019	Year-2	1	0	Fall
9/18/2019	FP	10M	9	2019	Year-2	1	0	Fall
9/18/2019	FP	12M	9	2019	Year-2	1	0	Fall
9/18/2019	FP	14M	9	2019	Year-2		1	Fall
9/18/2019	FP	8A	9	2019	Year-2	0	0	Fall
9/18/2019	FP	9A	9	2019	Year-2	1	0	Fall
9/18/2019	FP	11A	9	2019	Year-2	1	0	Fall
9/18/2019	FP	13A	9	2019	Year-2	1	0	Fall
9/19/2019	FP	1M	9	2019	Year-2	1	0	Fall
9/19/2019	FP	2M	9	2019	Year-2	1	0	Fall
9/19/2019	FP	3M	9	2019	Year-2	1	0	Fall
9/19/2019	FP	4M	9	2019	Year-2	1	0	Fall
9/19/2019	FP	5M	9	2019	Year-2	1	0	Fall
9/19/2019	FP	6M	9	2019	Year-2	1	0	Fall
9/19/2019	FP	7M	9	2019	Year-2	0	0	Fall
9/19/2019	FP	10M	9	2019	Year-2	1	0	Fall
9/19/2019	FP	12M	9	2019	Year-2	1	0	Fall
9/19/2019	FP	14M	9	2019	Year-2		1	Fall
9/19/2019	FP	8A	9	2019	Year-2	0	0	Fall
9/19/2019	FP	9A	9	2019	Year-2	0	0	Fall
9/19/2019	FP	11A	9	2019	Year-2	1	0	Fall
9/19/2019	FP	13A	9	2019	Year-2	1	0	Fall
9/20/2019	FP	1M	9	2019	Year-2	1	0	Fall

Date	Species	Site	Month	Year	Survey Year	Presence	Data Gap	Season
9/20/2019	FP	2M	9	2019	Year-2	1	0	Fall
9/20/2019	FP	3M	9	2019	Year-2	1	0	Fall
9/20/2019	FP	4M	9	2019	Year-2	1	0	Fall
9/20/2019	FP	5M	9	2019	Year-2	1	0	Fall
9/20/2019	FP	6M	9	2019	Year-2	1	0	Fall
9/20/2019	FP	7M	9	2019	Year-2	0	0	Fall
9/20/2019	FP	10M	9	2019	Year-2	1	0	Fall
9/20/2019	FP	12M	9	2019	Year-2	1	0	Fall
9/20/2019	FP	14M	9	2019	Year-2		1	Fall
9/20/2019	FP	8A	9	2019	Year-2	0	0	Fall
9/20/2019	FP	9A	9	2019	Year-2	1	0	Fall
9/20/2019	FP	11A	9	2019	Year-2	1	0	Fall
9/20/2019	FP	13A	9	2019	Year-2	1	0	Fall
9/21/2019	FP	1M	9	2019	Year-2	1	0	Fall
9/21/2019	FP	2M	9	2019	Year-2	1	0	Fall
9/21/2019	FP	3M	9	2019	Year-2	1	0	Fall
9/21/2019	FP	4M	9	2019	Year-2	1	0	Fall
9/21/2019	FP	5M	9	2019	Year-2	1	0	Fall
9/21/2019	FP	6M	9	2019	Year-2	1	0	Fall
9/21/2019	FP	7M	9	2019	Year-2	0	0	Fall
9/21/2019	FP	10M	9	2019	Year-2	1	0	Fall
9/21/2019	FP	12M	9	2019	Year-2	1	0	Fall
9/21/2019	FP	14M	9	2019	Year-2		1	Fall
9/21/2019	FP	8A	9	2019	Year-2	0	0	Fall
9/21/2019	FP	9A	9	2019	Year-2	0	0	Fall
9/21/2019	FP	11A	9	2019	Year-2	1	0	Fall
9/21/2019	FP	13A	9	2019	Year-2	1	0	Fall
9/22/2019	FP	1M	9	2019	Year-2	1	0	Fall
9/22/2019	FP	2M	9	2019	Year-2	1	0	Fall
9/22/2019	FP	3M	9	2019	Year-2	1	0	Fall
9/22/2019	FP	4M	9	2019	Year-2	1	0	Fall
9/22/2019	FP	5M	9	2019	Year-2	1	0	Fall
9/22/2019	FP	6M	9	2019	Year-2	1	0	Fall
9/22/2019	FP	7M	9	2019	Year-2	0	0	Fall
9/22/2019	FP	10M	9	2019	Year-2	1	0	Fall
9/22/2019	FP	12M	9	2019	Year-2	1	0	Fall
9/22/2019	FP	14M	9	2019	Year-2		1	Fall
9/22/2019	FP	8A	9	2019	Year-2	0	0	Fall
9/22/2019	FP	9A	9	2019	Year-2	1	0	Fall
9/22/2019	FP	11A	9	2019	Year-2	1	0	Fall
9/22/2019	FP	13A	9	2019	Year-2	1	0	Fall
9/23/2019	FP	1M	9	2019	Year-2	1	0	Fall
9/23/2019	FP	2M	9	2019	Year-2	1	0	Fall
9/23/2019	FP	3M	9	2019	Year-2	1	0	Fall

Date	Species	Site	Month	Year	Survey Year	Presence	Data Gap	Season
9/23/2019	FP	4M	9	2019	Year-2	1	0	Fall
9/23/2019	FP	5M	9	2019	Year-2	1	0	Fall
9/23/2019	FP	6M	9	2019	Year-2	1	0	Fall
9/23/2019	FP	7M	9	2019	Year-2	0	0	Fall
9/23/2019	FP	10M	9	2019	Year-2	1	0	Fall
9/23/2019	FP	12M	9	2019	Year-2	1	0	Fall
9/23/2019	FP	14M	9	2019	Year-2		1	Fall
9/23/2019	FP	8A	9	2019	Year-2	0	0	Fall
9/23/2019	FP	9A	9	2019	Year-2	0	0	Fall
9/23/2019	FP	11A	9	2019	Year-2	1	0	Fall
9/23/2019	FP	13A	9	2019	Year-2	1	0	Fall
9/24/2019	FP	1M	9	2019	Year-2	1	0	Fall
9/24/2019	FP	2M	9	2019	Year-2	1	0	Fall
9/24/2019	FP	3M	9	2019	Year-2	1	0	Fall
9/24/2019	FP	4M	9	2019	Year-2	1	0	Fall
9/24/2019	FP	5M	9	2019	Year-2	1	0	Fall
9/24/2019	FP	6M	9	2019	Year-2	1	0	Fall
9/24/2019	FP	7M	9	2019	Year-2	0	0	Fall
9/24/2019	FP	10M	9	2019	Year-2	1	0	Fall
9/24/2019	FP	12M	9	2019	Year-2	1	0	Fall
9/24/2019	FP	14M	9	2019	Year-2		1	Fall
9/24/2019	FP	8A	9	2019	Year-2	0	0	Fall
9/24/2019	FP	9A	9	2019	Year-2	0	0	Fall
9/24/2019	FP	11A	9	2019	Year-2	1	0	Fall
9/24/2019	FP	13A	9	2019	Year-2	1	0	Fall
9/25/2019	FP	1M	9	2019	Year-2	1	0	Fall
9/25/2019	FP	2M	9	2019	Year-2	1	0	Fall
9/25/2019	FP	3M	9	2019	Year-2	1	0	Fall
9/25/2019	FP	4M	9	2019	Year-2	1	0	Fall
9/25/2019	FP	5M	9	2019	Year-2	1	0	Fall
9/25/2019	FP	6M	9	2019	Year-2	1	0	Fall
9/25/2019	FP	7M	9	2019	Year-2	0	0	Fall
9/25/2019	FP	10M	9	2019	Year-2	1	0	Fall
9/25/2019	FP	12M	9	2019	Year-2	1	0	Fall
9/25/2019	FP	14M	9	2019	Year-2		1	Fall
9/25/2019	FP	8A	9	2019	Year-2	0	0	Fall
9/25/2019	FP	9A	9	2019	Year-2	0	0	Fall
9/25/2019	FP	11A	9	2019	Year-2	1	0	Fall
9/25/2019	FP	13A	9	2019	Year-2	1	0	Fall
9/26/2019	FP	1M	9	2019	Year-2	1	0	Fall
9/26/2019	FP	2M	9	2019	Year-2	1	0	Fall
9/26/2019	FP	3M	9	2019	Year-2	1	0	Fall
9/26/2019	FP	4M	9	2019	Year-2	1	0	Fall
9/26/2019	FP	5M	9	2019	Year-2	1	0	Fall

Date	Species	Site	Month	Year	Survey Year	Presence	Data Gap	Season
9/26/2019	FP	6M	9	2019	Year-2	1	0	Fall
9/26/2019	FP	7M	9	2019	Year-2	0	0	Fall
9/26/2019	FP	10M	9	2019	Year-2	1	0	Fall
9/26/2019	FP	12M	9	2019	Year-2	1	0	Fall
9/26/2019	FP	14M	9	2019	Year-2		1	Fall
9/26/2019	FP	8A	9	2019	Year-2	0	0	Fall
9/26/2019	FP	9A	9	2019	Year-2	0	0	Fall
9/26/2019	FP	11A	9	2019	Year-2	1	0	Fall
9/26/2019	FP	13A	9	2019	Year-2	1	0	Fall
9/27/2019	FP	1M	9	2019	Year-2	1	0	Fall
9/27/2019	FP	2M	9	2019	Year-2	1	0	Fall
9/27/2019	FP	3M	9	2019	Year-2	1	0	Fall
9/27/2019	FP	4M	9	2019	Year-2	1	0	Fall
9/27/2019	FP	5M	9	2019	Year-2	1	0	Fall
9/27/2019	FP	6M	9	2019	Year-2	1	0	Fall
9/27/2019	FP	7M	9	2019	Year-2	0	0	Fall
9/27/2019	FP	10M	9	2019	Year-2	1	0	Fall
9/27/2019	FP	12M	9	2019	Year-2	1	0	Fall
9/27/2019	FP	14M	9	2019	Year-2		1	Fall
9/27/2019	FP	8A	9	2019	Year-2	0	0	Fall
9/27/2019	FP	9A	9	2019	Year-2	0	0	Fall
9/27/2019	FP	11A	9	2019	Year-2	1	0	Fall
9/27/2019	FP	13A	9	2019	Year-2	1	0	Fall
9/28/2019	FP	1M	9	2019	Year-2	1	0	Fall
9/28/2019	FP	2M	9	2019	Year-2	1	0	Fall
9/28/2019	FP	3M	9	2019	Year-2	1	0	Fall
9/28/2019	FP	4M	9	2019	Year-2	1	0	Fall
9/28/2019	FP	5M	9	2019	Year-2	1	0	Fall
9/28/2019	FP	6M	9	2019	Year-2	1	0	Fall
9/28/2019	FP	7M	9	2019	Year-2	0	0	Fall
9/28/2019	FP	10M	9	2019	Year-2	1	0	Fall
9/28/2019	FP	12M	9	2019	Year-2	1	0	Fall
9/28/2019	FP	14M	9	2019	Year-2		1	Fall
9/28/2019	FP	8A	9	2019	Year-2	0	0	Fall
9/28/2019	FP	9A	9	2019	Year-2	0	0	Fall
9/28/2019	FP	11A	9	2019	Year-2	1	0	Fall
9/28/2019	FP	13A	9	2019	Year-2	1	0	Fall
9/29/2019	FP	1M	9	2019	Year-2	1	0	Fall
9/29/2019	FP	2M	9	2019	Year-2	1	0	Fall
9/29/2019	FP	3M	9	2019	Year-2	1	0	Fall
9/29/2019	FP	4M	9	2019	Year-2	1	0	Fall
9/29/2019	FP	5M	9	2019	Year-2	1	0	Fall
9/29/2019	FP	6M	9	2019	Year-2	1	0	Fall
9/29/2019	FP	7M	9	2019	Year-2	0	0	Fall

Date	Species	Site	Month	Year	Survey Year	Presence	Data Gap	Season
9/29/2019	FP	10M	9	2019	Year-2	1	0	Fall
9/29/2019	FP	12M	9	2019	Year-2	1	0	Fall
9/29/2019	FP	14M	9	2019	Year-2		1	Fall
9/29/2019	FP	8A	9	2019	Year-2	0	0	Fall
9/29/2019	FP	9A	9	2019	Year-2	1	0	Fall
9/29/2019	FP	11A	9	2019	Year-2	1	0	Fall
9/29/2019	FP	13A	9	2019	Year-2	1	0	Fall
9/30/2019	FP	1M	9	2019	Year-2	1	0	Fall
9/30/2019	FP	2M	9	2019	Year-2	1	0	Fall
9/30/2019	FP	3M	9	2019	Year-2	1	0	Fall
9/30/2019	FP	4M	9	2019	Year-2	1	0	Fall
9/30/2019	FP	5M	9	2019	Year-2	1	0	Fall
9/30/2019	FP	6M	9	2019	Year-2	1	0	Fall
9/30/2019	FP	7M	9	2019	Year-2	0	0	Fall
9/30/2019	FP	10M	9	2019	Year-2	1	0	Fall
9/30/2019	FP	12M	9	2019	Year-2	1	0	Fall
9/30/2019	FP	14M	9	2019	Year-2		1	Fall
9/30/2019	FP	8A	9	2019	Year-2	0	0	Fall
9/30/2019	FP	9A	9	2019	Year-2	0	0	Fall
9/30/2019	FP	11A	9	2019	Year-2	1	0	Fall
9/30/2019	FP	13A	9	2019	Year-2	1	0	Fall
10/1/2019	FP	1M	10	2019	Year-2	1	0	Fall
10/1/2019	FP	2M	10	2019	Year-2	1	0	Fall
10/1/2019	FP	3M	10	2019	Year-2	1	0	Fall
10/1/2019	FP	4M	10	2019	Year-2	1	0	Fall
10/1/2019	FP	5M	10	2019	Year-2	1	0	Fall
10/1/2019	FP	6M	10	2019	Year-2	1	0	Fall
10/1/2019	FP	7M	10	2019	Year-2	0	0	Fall
10/1/2019	FP	10M	10	2019	Year-2	1	0	Fall
10/1/2019	FP	12M	10	2019	Year-2	1	0	Fall
10/1/2019	FP	14M	10	2019	Year-2		1	Fall
10/1/2019	FP	8A	10	2019	Year-2	0	0	Fall
10/1/2019	FP	9A	10	2019	Year-2	0	0	Fall
10/1/2019	FP	11A	10	2019	Year-2	1	0	Fall
10/1/2019	FP	13A	10	2019	Year-2	1	0	Fall
10/2/2019	FP	1M	10	2019	Year-2	1	0	Fall
10/2/2019	FP	2M	10	2019	Year-2	1	0	Fall
10/2/2019	FP	3M	10	2019	Year-2	1	0	Fall
10/2/2019	FP	4M	10	2019	Year-2	1	0	Fall
10/2/2019	FP	5M	10	2019	Year-2	1	0	Fall
10/2/2019	FP	6M	10	2019	Year-2	1	0	Fall
10/2/2019	FP	7M	10	2019	Year-2	0	0	Fall
10/2/2019	FP	10M	10	2019	Year-2	1	0	Fall
10/2/2019	FP	12M	10	2019	Year-2	1	0	Fall

Date	Species	Site	Month	Year	Survey Year	Presence	Data Gap	Season
10/2/2019	FP	14M	10	2019	Year-2		1	Fall
10/2/2019	FP	8A	10	2019	Year-2	0	0	Fall
10/2/2019	FP	9A	10	2019	Year-2	0	0	Fall
10/2/2019	FP	11A	10	2019	Year-2	1	0	Fall
10/2/2019	FP	13A	10	2019	Year-2	1	0	Fall
10/3/2019	FP	1M	10	2019	Year-2	1	0	Fall
10/3/2019	FP	2M	10	2019	Year-2	1	0	Fall
10/3/2019	FP	3M	10	2019	Year-2	1	0	Fall
10/3/2019	FP	4M	10	2019	Year-2	1	0	Fall
10/3/2019	FP	5M	10	2019	Year-2	1	0	Fall
10/3/2019	FP	6M	10	2019	Year-2	1	0	Fall
10/3/2019	FP	7M	10	2019	Year-2	0	0	Fall
10/3/2019	FP	10M	10	2019	Year-2	1	0	Fall
10/3/2019	FP	12M	10	2019	Year-2	1	0	Fall
10/3/2019	FP	14M	10	2019	Year-2		1	Fall
10/3/2019	FP	8A	10	2019	Year-2	0	0	Fall
10/3/2019	FP	9A	10	2019	Year-2	0	0	Fall
10/3/2019	FP	11A	10	2019	Year-2	1	0	Fall
10/3/2019	FP	13A	10	2019	Year-2	1	0	Fall
10/4/2019	FP	1M	10	2019	Year-2	1	0	Fall
10/4/2019	FP	2M	10	2019	Year-2	1	0	Fall
10/4/2019	FP	3M	10	2019	Year-2	1	0	Fall
10/4/2019	FP	4M	10	2019	Year-2	1	0	Fall
10/4/2019	FP	5M	10	2019	Year-2	1	0	Fall
10/4/2019	FP	6M	10	2019	Year-2	1	0	Fall
10/4/2019	FP	7M	10	2019	Year-2	0	0	Fall
10/4/2019	FP	10M	10	2019	Year-2	1	0	Fall
10/4/2019	FP	12M	10	2019	Year-2	1	0	Fall
10/4/2019	FP	14M	10	2019	Year-2		1	Fall
10/4/2019	FP	8A	10	2019	Year-2	0	0	Fall
10/4/2019	FP	9A	10	2019	Year-2	0	0	Fall
10/4/2019	FP	11A	10	2019	Year-2	1	0	Fall
10/4/2019	FP	13A	10	2019	Year-2	1	0	Fall
10/5/2019	FP	1M	10	2019	Year-2	1	0	Fall
10/5/2019	FP	2M	10	2019	Year-2	1	0	Fall
10/5/2019	FP	3M	10	2019	Year-2	1	0	Fall
10/5/2019	FP	4M	10	2019	Year-2	1	0	Fall
10/5/2019	FP	5M	10	2019	Year-2	1	0	Fall
10/5/2019	FP	6M	10	2019	Year-2	1	0	Fall
10/5/2019	FP	7M	10	2019	Year-2	0	0	Fall
10/5/2019	FP	10M	10	2019	Year-2	1	0	Fall
10/5/2019	FP	12M	10	2019	Year-2	1	0	Fall
10/5/2019	FP	14M	10	2019	Year-2		1	Fall
10/5/2019	FP	8A	10	2019	Year-2	0	0	Fall

Date	Species	Site	Month	Year	Survey Year	Presence	Data Gap	Season
10/5/2019	FP	9A	10	2019	Year-2	1	0	Fall
10/5/2019	FP	11A	10	2019	Year-2	1	0	Fall
10/5/2019	FP	13A	10	2019	Year-2	1	0	Fall
10/6/2019	FP	1M	10	2019	Year-2	1	0	Fall
10/6/2019	FP	2M	10	2019	Year-2	1	0	Fall
10/6/2019	FP	3M	10	2019	Year-2	1	0	Fall
10/6/2019	FP	4M	10	2019	Year-2	1	0	Fall
10/6/2019	FP	5M	10	2019	Year-2	1	0	Fall
10/6/2019	FP	6M	10	2019	Year-2	1	0	Fall
10/6/2019	FP	7M	10	2019	Year-2	0	0	Fall
10/6/2019	FP	10M	10	2019	Year-2	1	0	Fall
10/6/2019	FP	12M	10	2019	Year-2	1	0	Fall
10/6/2019	FP	14M	10	2019	Year-2		1	Fall
10/6/2019	FP	8A	10	2019	Year-2	0	0	Fall
10/6/2019	FP	9A	10	2019	Year-2	0	0	Fall
10/6/2019	FP	11A	10	2019	Year-2	1	0	Fall
10/6/2019	FP	13A	10	2019	Year-2	1	0	Fall
10/7/2019	FP	1M	10	2019	Year-2	1	0	Fall
10/7/2019	FP	2M	10	2019	Year-2	1	0	Fall
10/7/2019	FP	3M	10	2019	Year-2	1	0	Fall
10/7/2019	FP	4M	10	2019	Year-2	1	0	Fall
10/7/2019	FP	5M	10	2019	Year-2	1	0	Fall
10/7/2019	FP	6M	10	2019	Year-2	1	0	Fall
10/7/2019	FP	7M	10	2019	Year-2	0	0	Fall
10/7/2019	FP	10M	10	2019	Year-2	1	0	Fall
10/7/2019	FP	12M	10	2019	Year-2	1	0	Fall
10/7/2019	FP	14M	10	2019	Year-2		1	Fall
10/7/2019	FP	8A	10	2019	Year-2	0	0	Fall
10/7/2019	FP	9A	10	2019	Year-2	1	0	Fall
10/7/2019	FP	11A	10	2019	Year-2	1	0	Fall
10/7/2019	FP	13A	10	2019	Year-2	1	0	Fall
10/8/2019	FP	1M	10	2019	Year-2	1	0	Fall
10/8/2019	FP	2M	10	2019	Year-2	1	0	Fall
10/8/2019	FP	3M	10	2019	Year-2	1	0	Fall
10/8/2019	FP	4M	10	2019	Year-2	1	0	Fall
10/8/2019	FP	5M	10	2019	Year-2	1	0	Fall
10/8/2019	FP	6M	10	2019	Year-2	1	0	Fall
10/8/2019	FP	7M	10	2019	Year-2	0	0	Fall
10/8/2019	FP	10M	10	2019	Year-2	1	0	Fall
10/8/2019	FP	12M	10	2019	Year-2	1	0	Fall
10/8/2019	FP	14M	10	2019	Year-2		1	Fall
10/8/2019	FP	8A	10	2019	Year-2	0	0	Fall
10/8/2019	FP	9A	10	2019	Year-2	0	0	Fall
10/8/2019	FP	11A	10	2019	Year-2	1	0	Fall

Date	Species	Site	Month	Year	Survey Year	Presence	Data Gap	Season
10/8/2019	FP	13A	10	2019	Year-2	1	0	Fall
10/9/2019	FP	1M	10	2019	Year-2	1	0	Fall
10/9/2019	FP	2M	10	2019	Year-2	1	0	Fall
10/9/2019	FP	3M	10	2019	Year-2	1	0	Fall
10/9/2019	FP	4M	10	2019	Year-2	1	0	Fall
10/9/2019	FP	5M	10	2019	Year-2	1	0	Fall
10/9/2019	FP	6M	10	2019	Year-2	1	0	Fall
10/9/2019	FP	7M	10	2019	Year-2	0	0	Fall
10/9/2019	FP	10M	10	2019	Year-2	1	0	Fall
10/9/2019	FP	12M	10	2019	Year-2	1	0	Fall
10/9/2019	FP	14M	10	2019	Year-2		1	Fall
10/9/2019	FP	8A	10	2019	Year-2	0	0	Fall
10/9/2019	FP	9A	10	2019	Year-2	0	0	Fall
10/9/2019	FP	11A	10	2019	Year-2	1	0	Fall
10/9/2019	FP	13A	10	2019	Year-2	1	0	Fall
10/10/2019	FP	1M	10	2019	Year-2	0	0	Fall
10/10/2019	FP	2M	10	2019	Year-2	1	0	Fall
10/10/2019	FP	3M	10	2019	Year-2	0	0	Fall
10/10/2019	FP	4M	10	2019	Year-2	1	0	Fall
10/10/2019	FP	5M	10	2019	Year-2	1	0	Fall
10/10/2019	FP	6M	10	2019	Year-2	0	0	Fall
10/10/2019	FP	7M	10	2019	Year-2	0	0	Fall
10/10/2019	FP	10M	10	2019	Year-2	1	0	Fall
10/10/2019	FP	12M	10	2019	Year-2	1	0	Fall
10/10/2019	FP	14M	10	2019	Year-2		1	Fall
10/10/2019	FP	8A	10	2019	Year-2	0	0	Fall
10/10/2019	FP	9A	10	2019	Year-2	0	0	Fall
10/10/2019	FP	11A	10	2019	Year-2	1	0	Fall
10/10/2019	FP	13A	10	2019	Year-2	1	0	Fall
10/11/2019	FP	1M	10	2019	Year-2	1	0	Fall
10/11/2019	FP	2M	10	2019	Year-2	0	0	Fall
10/11/2019	FP	3M	10	2019	Year-2	0	0	Fall
10/11/2019	FP	4M	10	2019	Year-2	0	0	Fall
10/11/2019	FP	5M	10	2019	Year-2	1	0	Fall
10/11/2019	FP	6M	10	2019	Year-2	1	0	Fall
10/11/2019	FP	7M	10	2019	Year-2	0	0	Fall
10/11/2019	FP	10M	10	2019	Year-2	1	0	Fall
10/11/2019	FP	12M	10	2019	Year-2	1	0	Fall
10/11/2019	FP	14M	10	2019	Year-2		1	Fall
10/11/2019	FP	8A	10	2019	Year-2	0	0	Fall
10/11/2019	FP	9A	10	2019	Year-2	0	0	Fall
10/11/2019	FP	11A	10	2019	Year-2	1	0	Fall
10/11/2019	FP	13A	10	2019	Year-2	0	0	Fall
10/12/2019	FP	1M	10	2019	Year-2	1	0	Fall

Date	Species	Site	Month	Year	Survey Year	Presence	Data Gap	Season
10/12/2019	FP	2M	10	2019	Year-2	1	0	Fall
10/12/2019	FP	3M	10	2019	Year-2	1	0	Fall
10/12/2019	FP	4M	10	2019	Year-2	0	0	Fall
10/12/2019	FP	5M	10	2019	Year-2	0	0	Fall
10/12/2019	FP	6M	10	2019	Year-2	0	0	Fall
10/12/2019	FP	7M	10	2019	Year-2	0	0	Fall
10/12/2019	FP	10M	10	2019	Year-2	1	0	Fall
10/12/2019	FP	12M	10	2019	Year-2	1	0	Fall
10/12/2019	FP	14M	10	2019	Year-2		1	Fall
10/12/2019	FP	8A	10	2019	Year-2	0	0	Fall
10/12/2019	FP	9A	10	2019	Year-2	0	0	Fall
10/12/2019	FP	11A	10	2019	Year-2	1	0	Fall
10/12/2019	FP	13A	10	2019	Year-2	1	0	Fall
10/13/2019	FP	1M	10	2019	Year-2	1	0	Fall
10/13/2019	FP	2M	10	2019	Year-2	1	0	Fall
10/13/2019	FP	3M	10	2019	Year-2	1	0	Fall
10/13/2019	FP	4M	10	2019	Year-2	1	0	Fall
10/13/2019	FP	5M	10	2019	Year-2	0	0	Fall
10/13/2019	FP	6M	10	2019	Year-2	1	0	Fall
10/13/2019	FP	7M	10	2019	Year-2	0	0	Fall
10/13/2019	FP	10M	10	2019	Year-2	1	0	Fall
10/13/2019	FP	12M	10	2019	Year-2	1	0	Fall
10/13/2019	FP	14M	10	2019	Year-2		1	Fall
10/13/2019	FP	8A	10	2019	Year-2	0	0	Fall
10/13/2019	FP	9A	10	2019	Year-2	1	0	Fall
10/13/2019	FP	11A	10	2019	Year-2	1	0	Fall
10/13/2019	FP	13A	10	2019	Year-2	1	0	Fall
10/14/2019	FP	1M	10	2019	Year-2	1	0	Fall
10/14/2019	FP	2M	10	2019	Year-2	1	0	Fall
10/14/2019	FP	3M	10	2019	Year-2	1	0	Fall
10/14/2019	FP	4M	10	2019	Year-2	1	0	Fall
10/14/2019	FP	5M	10	2019	Year-2	0	0	Fall
10/14/2019	FP	6M	10	2019	Year-2	1	0	Fall
10/14/2019	FP	7M	10	2019	Year-2	0	0	Fall
10/14/2019	FP	10M	10	2019	Year-2	1	0	Fall
10/14/2019	FP	12M	10	2019	Year-2	1	0	Fall
10/14/2019	FP	14M	10	2019	Year-2		1	Fall
10/14/2019	FP	8A	10	2019	Year-2	1	0	Fall
10/14/2019	FP	9A	10	2019	Year-2	1	0	Fall
10/14/2019	FP	11A	10	2019	Year-2	1	0	Fall
10/14/2019	FP	13A	10	2019	Year-2	1	0	Fall
10/15/2019	FP	1M	10	2019	Year-2	1	0	Fall
10/15/2019	FP	2M	10	2019	Year-2	1	0	Fall
10/15/2019	FP	3M	10	2019	Year-2	1	0	Fall

Date	Species	Site	Month	Year	Survey Year	Presence	Data Gap	Season
10/15/2019	FP	4M	10	2019	Year-2	1	0	Fall
10/15/2019	FP	5M	10	2019	Year-2	1	0	Fall
10/15/2019	FP	6M	10	2019	Year-2	1	0	Fall
10/15/2019	FP	7M	10	2019	Year-2	0	0	Fall
10/15/2019	FP	10M	10	2019	Year-2	1	0	Fall
10/15/2019	FP	12M	10	2019	Year-2	1	0	Fall
10/15/2019	FP	14M	10	2019	Year-2		1	Fall
10/15/2019	FP	8A	10	2019	Year-2	1	0	Fall
10/15/2019	FP	9A	10	2019	Year-2	1	0	Fall
10/15/2019	FP	11A	10	2019	Year-2	1	0	Fall
10/15/2019	FP	13A	10	2019	Year-2	1	0	Fall
10/16/2019	FP	1M	10	2019	Year-3	1	0	Fall
10/16/2019	FP	2M	10	2019	Year-3	1	0	Fall
10/16/2019	FP	3M	10	2019	Year-3	1	0	Fall
10/16/2019	FP	4M	10	2019	Year-3	1	0	Fall
10/16/2019	FP	5M	10	2019	Year-3	1	0	Fall
10/16/2019	FP	6M	10	2019	Year-3	1	0	Fall
10/16/2019	FP	7M	10	2019	Year-3	1	0	Fall
10/16/2019	FP	10M	10	2019	Year-3	1	0	Fall
10/16/2019	FP	12M	10	2019	Year-3	1	0	Fall
10/16/2019	FP	14M	10	2019	Year-3		1	Fall
10/16/2019	FP	8A	10	2019	Year-3	1	0	Fall
10/16/2019	FP	9A	10	2019	Year-3	1	0	Fall
10/16/2019	FP	11A	10	2019	Year-3	1	0	Fall
10/16/2019	FP	13A	10	2019	Year-3	1	0	Fall
10/17/2019	FP	1M	10	2019	Year-3	1	0	Fall
10/17/2019	FP	2M	10	2019	Year-3	1	0	Fall
10/17/2019	FP	3M	10	2019	Year-3	1	0	Fall
10/17/2019	FP	4M	10	2019	Year-3	1	0	Fall
10/17/2019	FP	5M	10	2019	Year-3	1	0	Fall
10/17/2019	FP	6M	10	2019	Year-3	1	0	Fall
10/17/2019	FP	7M	10	2019	Year-3	1	0	Fall
10/17/2019	FP	10M	10	2019	Year-3	1	0	Fall
10/17/2019	FP	12M	10	2019	Year-3	1	0	Fall
10/17/2019	FP	14M	10	2019	Year-3		1	Fall
10/17/2019	FP	8A	10	2019	Year-3	0	0	Fall
10/17/2019	FP	9A	10	2019	Year-3	1	0	Fall
10/17/2019	FP	11A	10	2019	Year-3	1	0	Fall
10/17/2019	FP	13A	10	2019	Year-3	1	0	Fall
10/18/2019	FP	1M	10	2019	Year-3	1	0	Fall
10/18/2019	FP	2M	10	2019	Year-3	1	0	Fall
10/18/2019	FP	3M	10	2019	Year-3	1	0	Fall
10/18/2019	FP	4M	10	2019	Year-3	1	0	Fall
10/18/2019	FP	5M	10	2019	Year-3	1	0	Fall

Date	Species	Site	Month	Year	Survey Year	Presence	Data Gap	Season
10/18/2019	FP	6M	10	2019	Year-3	1	0	Fall
10/18/2019	FP	7M	10	2019	Year-3	0	0	Fall
10/18/2019	FP	10M	10	2019	Year-3	1	0	Fall
10/18/2019	FP	12M	10	2019	Year-3	1	0	Fall
10/18/2019	FP	14M	10	2019	Year-3		1	Fall
10/18/2019	FP	8A	10	2019	Year-3	0	0	Fall
10/18/2019	FP	9A	10	2019	Year-3	1	0	Fall
10/18/2019	FP	11A	10	2019	Year-3	1	0	Fall
10/18/2019	FP	13A	10	2019	Year-3	1	0	Fall
10/19/2019	FP	1M	10	2019	Year-3	0	0	Fall
10/19/2019	FP	2M	10	2019	Year-3	0	0	Fall
10/19/2019	FP	3M	10	2019	Year-3	0	0	Fall
10/19/2019	FP	4M	10	2019	Year-3	0	0	Fall
10/19/2019	FP	5M	10	2019	Year-3	0	0	Fall
10/19/2019	FP	6M	10	2019	Year-3	0	0	Fall
10/19/2019	FP	7M	10	2019	Year-3	0	0	Fall
10/19/2019	FP	10M	10	2019	Year-3	0	0	Fall
10/19/2019	FP	12M	10	2019	Year-3	0	0	Fall
10/19/2019	FP	14M	10	2019	Year-3		1	Fall
10/19/2019	FP	8A	10	2019	Year-3	0	0	Fall
10/19/2019	FP	9A	10	2019	Year-3	1	0	Fall
10/19/2019	FP	11A	10	2019	Year-3	1	0	Fall
10/19/2019	FP	13A	10	2019	Year-3	1	0	Fall
10/20/2019	FP	1M	10	2019	Year-3	0	0	Fall
10/20/2019	FP	2M	10	2019	Year-3	0	0	Fall
10/20/2019	FP	3M	10	2019	Year-3	0	0	Fall
10/20/2019	FP	4M	10	2019	Year-3	0	0	Fall
10/20/2019	FP	5M	10	2019	Year-3	0	0	Fall
10/20/2019	FP	6M	10	2019	Year-3	0	0	Fall
10/20/2019	FP	7M	10	2019	Year-3	0	0	Fall
10/20/2019	FP	10M	10	2019	Year-3		1	Fall
10/20/2019	FP	12M	10	2019	Year-3		1	Fall
10/20/2019	FP	14M	10	2019	Year-3		1	Fall
10/20/2019	FP	8A	10	2019	Year-3		1	Fall
10/20/2019	FP	9A	10	2019	Year-3		1	Fall
10/20/2019	FP	11A	10	2019	Year-3	1	0	Fall
10/20/2019	FP	13A	10	2019	Year-3		1	Fall
10/21/2019	FP	1M	10	2019	Year-3	0	0	Fall
10/21/2019	FP	2M	10	2019	Year-3	0	0	Fall
10/21/2019	FP	3M	10	2019	Year-3	0	0	Fall
10/21/2019	FP	4M	10	2019	Year-3	0	0	Fall
10/21/2019	FP	5M	10	2019	Year-3	0	0	Fall
10/21/2019	FP	6M	10	2019	Year-3	0	0	Fall
10/21/2019	FP	7M	10	2019	Year-3	0	0	Fall

Date	Species	Site	Month	Year	Survey Year	Presence	Data Gap	Season
10/21/2019	FP	10M	10	2019	Year-3	0	0	Fall
10/21/2019	FP	12M	10	2019	Year-3	0	0	Fall
10/21/2019	FP	14M	10	2019	Year-3	0	0	Fall
10/21/2019	FP	8A	10	2019	Year-3		1	Fall
10/21/2019	FP	9A	10	2019	Year-3		1	Fall
10/21/2019	FP	11A	10	2019	Year-3	1	0	Fall
10/21/2019	FP	13A	10	2019	Year-3		1	Fall
10/22/2019	FP	1M	10	2019	Year-3	0	0	Fall
10/22/2019	FP	2M	10	2019	Year-3	0	0	Fall
10/22/2019	FP	3M	10	2019	Year-3	0	0	Fall
10/22/2019	FP	4M	10	2019	Year-3	0	0	Fall
10/22/2019	FP	5M	10	2019	Year-3	0	0	Fall
10/22/2019	FP	6M	10	2019	Year-3	0	0	Fall
10/22/2019	FP	7M	10	2019	Year-3	0	0	Fall
10/22/2019	FP	10M	10	2019	Year-3	0	0	Fall
10/22/2019	FP	12M	10	2019	Year-3	0	0	Fall
10/22/2019	FP	14M	10	2019	Year-3	0	0	Fall
10/22/2019	FP	8A	10	2019	Year-3		1	Fall
10/22/2019	FP	9A	10	2019	Year-3		1	Fall
10/22/2019	FP	11A	10	2019	Year-3	1	0	Fall
10/22/2019	FP	13A	10	2019	Year-3		1	Fall
10/23/2019	FP	1M	10	2019	Year-3	0	0	Fall
10/23/2019	FP	2M	10	2019	Year-3	0	0	Fall
10/23/2019	FP	3M	10	2019	Year-3	0	0	Fall
10/23/2019	FP	4M	10	2019	Year-3	0	0	Fall
10/23/2019	FP	5M	10	2019	Year-3	0	0	Fall
10/23/2019	FP	6M	10	2019	Year-3	0	0	Fall
10/23/2019	FP	7M	10	2019	Year-3	0	0	Fall
10/23/2019	FP	10M	10	2019	Year-3	0	0	Fall
10/23/2019	FP	12M	10	2019	Year-3	0	0	Fall
10/23/2019	FP	14M	10	2019	Year-3	0	0	Fall
10/23/2019	FP	8A	10	2019	Year-3		1	Fall
10/23/2019	FP	9A	10	2019	Year-3		1	Fall
10/23/2019	FP	11A	10	2019	Year-3	1	0	Fall
10/23/2019	FP	13A	10	2019	Year-3		1	Fall
10/24/2019	FP	1M	10	2019	Year-3	0	0	Fall
10/24/2019	FP	2M	10	2019	Year-3	0	0	Fall
10/24/2019	FP	3M	10	2019	Year-3		1	Fall
10/24/2019	FP	4M	10	2019	Year-3		1	Fall
10/24/2019	FP	5M	10	2019	Year-3		1	Fall
10/24/2019	FP	6M	10	2019	Year-3		1	Fall
10/24/2019	FP	7M	10	2019	Year-3		1	Fall
10/24/2019	FP	10M	10	2019	Year-3	0	0	Fall
10/24/2019	FP	12M	10	2019	Year-3	0	0	Fall

Date	Species	Site	Month	Year	Survey Year	Presence	Data Gap	Season
10/24/2019	FP	14M	10	2019	Year-3	0	0	Fall
10/24/2019	FP	8A	10	2019	Year-3		1	Fall
10/24/2019	FP	9A	10	2019	Year-3		1	Fall
10/24/2019	FP	11A	10	2019	Year-3	1	0	Fall
10/24/2019	FP	13A	10	2019	Year-3		1	Fall
10/25/2019	FP	1M	10	2019	Year-3		1	Fall
10/25/2019	FP	2M	10	2019	Year-3		1	Fall
10/25/2019	FP	3M	10	2019	Year-3		1	Fall
10/25/2019	FP	4M	10	2019	Year-3	0	0	Fall
10/25/2019	FP	5M	10	2019	Year-3	0	0	Fall
10/25/2019	FP	6M	10	2019	Year-3	0	0	Fall
10/25/2019	FP	7M	10	2019	Year-3	0	0	Fall
10/25/2019	FP	10M	10	2019	Year-3	0	0	Fall
10/25/2019	FP	12M	10	2019	Year-3	0	0	Fall
10/25/2019	FP	14M	10	2019	Year-3	0	0	Fall
10/25/2019	FP	8A	10	2019	Year-3		1	Fall
10/25/2019	FP	9A	10	2019	Year-3		1	Fall
10/25/2019	FP	11A	10	2019	Year-3	1	0	Fall
10/25/2019	FP	13A	10	2019	Year-3		1	Fall
10/26/2019	FP	1M	10	2019	Year-3	1	0	Fall
10/26/2019	FP	2M	10	2019	Year-3	1	0	Fall
10/26/2019	FP	3M	10	2019	Year-3		1	Fall
10/26/2019	FP	4M	10	2019	Year-3	1	0	Fall
10/26/2019	FP	5M	10	2019	Year-3	1	0	Fall
10/26/2019	FP	6M	10	2019	Year-3	1	0	Fall
10/26/2019	FP	7M	10	2019	Year-3	0	0	Fall
10/26/2019	FP	10M	10	2019	Year-3	1	0	Fall
10/26/2019	FP	12M	10	2019	Year-3	1	0	Fall
10/26/2019	FP	14M	10	2019	Year-3	1	0	Fall
10/26/2019	FP	8A	10	2019	Year-3		1	Fall
10/26/2019	FP	9A	10	2019	Year-3		1	Fall
10/26/2019	FP	11A	10	2019	Year-3	1	0	Fall
10/26/2019	FP	13A	10	2019	Year-3		1	Fall
10/27/2019	FP	1M	10	2019	Year-3	1	0	Fall
10/27/2019	FP	2M	10	2019	Year-3	0	0	Fall
10/27/2019	FP	3M	10	2019	Year-3		1	Fall
10/27/2019	FP	4M	10	2019	Year-3	0	0	Fall
10/27/2019	FP	5M	10	2019	Year-3	1	0	Fall
10/27/2019	FP	6M	10	2019	Year-3	1	0	Fall
10/27/2019	FP	7M	10	2019	Year-3	1	0	Fall
10/27/2019	FP	10M	10	2019	Year-3	1	0	Fall
10/27/2019	FP	12M	10	2019	Year-3	1	0	Fall
10/27/2019	FP	14M	10	2019	Year-3	1	0	Fall
10/27/2019	FP	8A	10	2019	Year-3		1	Fall

Date	Species	Site	Month	Year	Survey Year	Presence	Data Gap	Season
10/27/2019	FP	9A	10	2019	Year-3		1	Fall
10/27/2019	FP	11A	10	2019	Year-3	1	0	Fall
10/27/2019	FP	13A	10	2019	Year-3		1	Fall
10/28/2019	FP	1M	10	2019	Year-3	1	0	Fall
10/28/2019	FP	2M	10	2019	Year-3	1	0	Fall
10/28/2019	FP	3M	10	2019	Year-3		1	Fall
10/28/2019	FP	4M	10	2019	Year-3	1	0	Fall
10/28/2019	FP	5M	10	2019	Year-3	0	0	Fall
10/28/2019	FP	6M	10	2019	Year-3	1	0	Fall
10/28/2019	FP	7M	10	2019	Year-3	1	0	Fall
10/28/2019	FP	10M	10	2019	Year-3	1	0	Fall
10/28/2019	FP	12M	10	2019	Year-3	1	0	Fall
10/28/2019	FP	14M	10	2019	Year-3	1	0	Fall
10/28/2019	FP	8A	10	2019	Year-3		1	Fall
10/28/2019	FP	9A	10	2019	Year-3		1	Fall
10/28/2019	FP	11A	10	2019	Year-3	1	0	Fall
10/28/2019	FP	13A	10	2019	Year-3		1	Fall
10/29/2019	FP	1M	10	2019	Year-3	0	0	Fall
10/29/2019	FP	2M	10	2019	Year-3	1	0	Fall
10/29/2019	FP	3M	10	2019	Year-3		1	Fall
10/29/2019	FP	4M	10	2019	Year-3	1	0	Fall
10/29/2019	FP	5M	10	2019	Year-3	1	0	Fall
10/29/2019	FP	6M	10	2019	Year-3	1	0	Fall
10/29/2019	FP	7M	10	2019	Year-3	0	0	Fall
10/29/2019	FP	10M	10	2019	Year-3	1	0	Fall
10/29/2019	FP	12M	10	2019	Year-3	1	0	Fall
10/29/2019	FP	14M	10	2019	Year-3	0	0	Fall
10/29/2019	FP	8A	10	2019	Year-3		1	Fall
10/29/2019	FP	9A	10	2019	Year-3		1	Fall
10/29/2019	FP	11A	10	2019	Year-3	1	0	Fall
10/29/2019	FP	13A	10	2019	Year-3		1	Fall
10/30/2019	FP	1M	10	2019	Year-3	1	0	Fall
10/30/2019	FP	2M	10	2019	Year-3	1	0	Fall
10/30/2019	FP	3M	10	2019	Year-3		1	Fall
10/30/2019	FP	4M	10	2019	Year-3	1	0	Fall
10/30/2019	FP	5M	10	2019	Year-3	1	0	Fall
10/30/2019	FP	6M	10	2019	Year-3	1	0	Fall
10/30/2019	FP	7M	10	2019	Year-3	0	0	Fall
10/30/2019	FP	10M	10	2019	Year-3	1	0	Fall
10/30/2019	FP	12M	10	2019	Year-3	1	0	Fall
10/30/2019	FP	14M	10	2019	Year-3	0	0	Fall
10/30/2019	FP	8A	10	2019	Year-3		1	Fall
10/30/2019	FP	9A	10	2019	Year-3		1	Fall
10/30/2019	FP	11A	10	2019	Year-3	1	0	Fall

Date	Species	Site	Month	Year	Survey Year	Presence	Data Gap	Season
10/30/2019	FP	13A	10	2019	Year-3		1	Fall
10/31/2019	FP	1M	10	2019	Year-3	1	0	Fall
10/31/2019	FP	2M	10	2019	Year-3	1	0	Fall
10/31/2019	FP	3M	10	2019	Year-3		1	Fall
10/31/2019	FP	4M	10	2019	Year-3	1	0	Fall
10/31/2019	FP	5M	10	2019	Year-3	1	0	Fall
10/31/2019	FP	6M	10	2019	Year-3	1	0	Fall
10/31/2019	FP	7M	10	2019	Year-3	0	0	Fall
10/31/2019	FP	10M	10	2019	Year-3	1	0	Fall
10/31/2019	FP	12M	10	2019	Year-3	0	0	Fall
10/31/2019	FP	14M	10	2019	Year-3	1	0	Fall
10/31/2019	FP	8A	10	2019	Year-3		1	Fall
10/31/2019	FP	9A	10	2019	Year-3		1	Fall
10/31/2019	FP	11A	10	2019	Year-3	1	0	Fall
10/31/2019	FP	13A	10	2019	Year-3		1	Fall
11/1/2019	FP	1M	11	2019	Year-3	1	0	Fall
11/1/2019	FP	2M	11	2019	Year-3	1	0	Fall
11/1/2019	FP	3M	11	2019	Year-3		1	Fall
11/1/2019	FP	4M	11	2019	Year-3	0	0	Fall
11/1/2019	FP	5M	11	2019	Year-3	0	0	Fall
11/1/2019	FP	6M	11	2019	Year-3	1	0	Fall
11/1/2019	FP	7M	11	2019	Year-3	0	0	Fall
11/1/2019	FP	10M	11	2019	Year-3	0	0	Fall
11/1/2019	FP	12M	11	2019	Year-3	1	0	Fall
11/1/2019	FP	14M	11	2019	Year-3	1	0	Fall
11/1/2019	FP	8A	11	2019	Year-3		1	Fall
11/1/2019	FP	9A	11	2019	Year-3		1	Fall
11/1/2019	FP	11A	11	2019	Year-3	1	0	Fall
11/1/2019	FP	13A	11	2019	Year-3		1	Fall
11/2/2019	FP	1M	11	2019	Year-3	1	0	Fall
11/2/2019	FP	2M	11	2019	Year-3	1	0	Fall
11/2/2019	FP	3M	11	2019	Year-3		1	Fall
11/2/2019	FP	4M	11	2019	Year-3	1	0	Fall
11/2/2019	FP	5M	11	2019	Year-3	1	0	Fall
11/2/2019	FP	6M	11	2019	Year-3	0	0	Fall
11/2/2019	FP	7M	11	2019	Year-3	0	0	Fall
11/2/2019	FP	10M	11	2019	Year-3	1	0	Fall
11/2/2019	FP	12M	11	2019	Year-3	1	0	Fall
11/2/2019	FP	14M	11	2019	Year-3	1	0	Fall
11/2/2019	FP	8A	11	2019	Year-3		1	Fall
11/2/2019	FP	9A	11	2019	Year-3		1	Fall
11/2/2019	FP	11A	11	2019	Year-3	1	0	Fall
11/2/2019	FP	13A	11	2019	Year-3		1	Fall
11/3/2019	FP	1M	11	2019	Year-3	1	0	Fall

Date	Species	Site	Month	Year	Survey Year	Presence	Data Gap	Season
11/3/2019	FP	2M	11	2019	Year-3	1	0	Fall
11/3/2019	FP	3M	11	2019	Year-3		1	Fall
11/3/2019	FP	4M	11	2019	Year-3	1	0	Fall
11/3/2019	FP	5M	11	2019	Year-3	1	0	Fall
11/3/2019	FP	6M	11	2019	Year-3	1	0	Fall
11/3/2019	FP	7M	11	2019	Year-3	1	0	Fall
11/3/2019	FP	10M	11	2019	Year-3	1	0	Fall
11/3/2019	FP	12M	11	2019	Year-3	1	0	Fall
11/3/2019	FP	14M	11	2019	Year-3	1	0	Fall
11/3/2019	FP	8A	11	2019	Year-3		1	Fall
11/3/2019	FP	9A	11	2019	Year-3		1	Fall
11/3/2019	FP	11A	11	2019	Year-3	1	0	Fall
11/3/2019	FP	13A	11	2019	Year-3		1	Fall
11/4/2019	FP	1M	11	2019	Year-3	1	0	Fall
11/4/2019	FP	2M	11	2019	Year-3	1	0	Fall
11/4/2019	FP	3M	11	2019	Year-3		1	Fall
11/4/2019	FP	4M	11	2019	Year-3	1	0	Fall
11/4/2019	FP	5M	11	2019	Year-3	1	0	Fall
11/4/2019	FP	6M	11	2019	Year-3	1	0	Fall
11/4/2019	FP	7M	11	2019	Year-3	0	0	Fall
11/4/2019	FP	10M	11	2019	Year-3	1	0	Fall
11/4/2019	FP	12M	11	2019	Year-3	1	0	Fall
11/4/2019	FP	14M	11	2019	Year-3	1	0	Fall
11/4/2019	FP	8A	11	2019	Year-3		1	Fall
11/4/2019	FP	9A	11	2019	Year-3		1	Fall
11/4/2019	FP	11A	11	2019	Year-3	1	0	Fall
11/4/2019	FP	13A	11	2019	Year-3		1	Fall
11/5/2019	FP	1M	11	2019	Year-3	1	0	Fall
11/5/2019	FP	2M	11	2019	Year-3	1	0	Fall
11/5/2019	FP	3M	11	2019	Year-3		1	Fall
11/5/2019	FP	4M	11	2019	Year-3	1	0	Fall
11/5/2019	FP	5M	11	2019	Year-3	1	0	Fall
11/5/2019	FP	6M	11	2019	Year-3	1	0	Fall
11/5/2019	FP	7M	11	2019	Year-3	0	0	Fall
11/5/2019	FP	10M	11	2019	Year-3	1	0	Fall
11/5/2019	FP	12M	11	2019	Year-3	1	0	Fall
11/5/2019	FP	14M	11	2019	Year-3	1	0	Fall
11/5/2019	FP	8A	11	2019	Year-3		1	Fall
11/5/2019	FP	9A	11	2019	Year-3		1	Fall
11/5/2019	FP	11A	11	2019	Year-3	1	0	Fall
11/5/2019	FP	13A	11	2019	Year-3		1	Fall
11/6/2019	FP	1M	11	2019	Year-3	1	0	Fall
11/6/2019	FP	2M	11	2019	Year-3	1	0	Fall
11/6/2019	FP	3M	11	2019	Year-3		1	Fall

Date	Species	Site	Month	Year	Survey Year	Presence	Data Gap	Season
11/6/2019	FP	4M	11	2019	Year-3	1	0	Fall
11/6/2019	FP	5M	11	2019	Year-3	1	0	Fall
11/6/2019	FP	6M	11	2019	Year-3	1	0	Fall
11/6/2019	FP	7M	11	2019	Year-3	1	0	Fall
11/6/2019	FP	10M	11	2019	Year-3	1	0	Fall
11/6/2019	FP	12M	11	2019	Year-3	1	0	Fall
11/6/2019	FP	14M	11	2019	Year-3	1	0	Fall
11/6/2019	FP	8A	11	2019	Year-3		1	Fall
11/6/2019	FP	9A	11	2019	Year-3		1	Fall
11/6/2019	FP	11A	11	2019	Year-3	1	0	Fall
11/6/2019	FP	13A	11	2019	Year-3		1	Fall
11/7/2019	FP	1M	11	2019	Year-3	1	0	Fall
11/7/2019	FP	2M	11	2019	Year-3	1	0	Fall
11/7/2019	FP	3M	11	2019	Year-3		1	Fall
11/7/2019	FP	4M	11	2019	Year-3	1	0	Fall
11/7/2019	FP	5M	11	2019	Year-3	1	0	Fall
11/7/2019	FP	6M	11	2019	Year-3	1	0	Fall
11/7/2019	FP	7M	11	2019	Year-3	0	0	Fall
11/7/2019	FP	10M	11	2019	Year-3	1	0	Fall
11/7/2019	FP	12M	11	2019	Year-3	1	0	Fall
11/7/2019	FP	14M	11	2019	Year-3	1	0	Fall
11/7/2019	FP	8A	11	2019	Year-3		1	Fall
11/7/2019	FP	9A	11	2019	Year-3		1	Fall
11/7/2019	FP	11A	11	2019	Year-3	1	0	Fall
11/7/2019	FP	13A	11	2019	Year-3		1	Fall
11/8/2019	FP	1M	11	2019	Year-3	1	0	Fall
11/8/2019	FP	2M	11	2019	Year-3	1	0	Fall
11/8/2019	FP	3M	11	2019	Year-3		1	Fall
11/8/2019	FP	4M	11	2019	Year-3	1	0	Fall
11/8/2019	FP	5M	11	2019	Year-3	1	0	Fall
11/8/2019	FP	6M	11	2019	Year-3	1	0	Fall
11/8/2019	FP	7M	11	2019	Year-3	0	0	Fall
11/8/2019	FP	10M	11	2019	Year-3	1	0	Fall
11/8/2019	FP	12M	11	2019	Year-3	1	0	Fall
11/8/2019	FP	14M	11	2019	Year-3	1	0	Fall
11/8/2019	FP	8A	11	2019	Year-3		1	Fall
11/8/2019	FP	9A	11	2019	Year-3		1	Fall
11/8/2019	FP	11A	11	2019	Year-3	1	0	Fall
11/8/2019	FP	13A	11	2019	Year-3		1	Fall
11/9/2019	FP	1M	11	2019	Year-3	1	0	Fall
11/9/2019	FP	2M	11	2019	Year-3	1	0	Fall
11/9/2019	FP	3M	11	2019	Year-3		1	Fall
11/9/2019	FP	4M	11	2019	Year-3	1	0	Fall
11/9/2019	FP	5M	11	2019	Year-3	1	0	Fall

Date	Species	Site	Month	Year	Survey Year	Presence	Data Gap	Season
11/9/2019	FP	6M	11	2019	Year-3	1	0	Fall
11/9/2019	FP	7M	11	2019	Year-3	1	0	Fall
11/9/2019	FP	10M	11	2019	Year-3	1	0	Fall
11/9/2019	FP	12M	11	2019	Year-3	0	0	Fall
11/9/2019	FP	14M	11	2019	Year-3	1	0	Fall
11/9/2019	FP	8A	11	2019	Year-3		1	Fall
11/9/2019	FP	9A	11	2019	Year-3		1	Fall
11/9/2019	FP	11A	11	2019	Year-3	1	0	Fall
11/9/2019	FP	13A	11	2019	Year-3		1	Fall
11/10/2019	FP	1M	11	2019	Year-3	1	0	Fall
11/10/2019	FP	2M	11	2019	Year-3	0	0	Fall
11/10/2019	FP	3M	11	2019	Year-3		1	Fall
11/10/2019	FP	4M	11	2019	Year-3	1	0	Fall
11/10/2019	FP	5M	11	2019	Year-3	1	0	Fall
11/10/2019	FP	6M	11	2019	Year-3	1	0	Fall
11/10/2019	FP	7M	11	2019	Year-3	1	0	Fall
11/10/2019	FP	10M	11	2019	Year-3	1	0	Fall
11/10/2019	FP	12M	11	2019	Year-3	1	0	Fall
11/10/2019	FP	14M	11	2019	Year-3	1	0	Fall
11/10/2019	FP	8A	11	2019	Year-3		1	Fall
11/10/2019	FP	9A	11	2019	Year-3		1	Fall
11/10/2019	FP	11A	11	2019	Year-3	1	0	Fall
11/10/2019	FP	13A	11	2019	Year-3		1	Fall
11/11/2019	FP	1M	11	2019	Year-3	1	0	Fall
11/11/2019	FP	2M	11	2019	Year-3	1	0	Fall
11/11/2019	FP	3M	11	2019	Year-3		1	Fall
11/11/2019	FP	4M	11	2019	Year-3	1	0	Fall
11/11/2019	FP	5M	11	2019	Year-3	1	0	Fall
11/11/2019	FP	6M	11	2019	Year-3	1	0	Fall
11/11/2019	FP	7M	11	2019	Year-3	0	0	Fall
11/11/2019	FP	10M	11	2019	Year-3	0	0	Fall
11/11/2019	FP	12M	11	2019	Year-3	1	0	Fall
11/11/2019	FP	14M	11	2019	Year-3	1	0	Fall
11/11/2019	FP	8A	11	2019	Year-3		1	Fall
11/11/2019	FP	9A	11	2019	Year-3		1	Fall
11/11/2019	FP	11A	11	2019	Year-3	1	0	Fall
11/11/2019	FP	13A	11	2019	Year-3		1	Fall
11/12/2019	FP	1M	11	2019	Year-3	1	0	Fall
11/12/2019	FP	2M	11	2019	Year-3	1	0	Fall
11/12/2019	FP	3M	11	2019	Year-3		1	Fall
11/12/2019	FP	4M	11	2019	Year-3	1	0	Fall
11/12/2019	FP	5M	11	2019	Year-3	1	0	Fall
11/12/2019	FP	6M	11	2019	Year-3	1	0	Fall
11/12/2019	FP	7M	11	2019	Year-3	0	0	Fall

Date	Species	Site	Month	Year	Survey Year	Presence	Data Gap	Season
11/12/2019	FP	10M	11	2019	Year-3	0	0	Fall
11/12/2019	FP	12M	11	2019	Year-3	1	0	Fall
11/12/2019	FP	14M	11	2019	Year-3	1	0	Fall
11/12/2019	FP	8A	11	2019	Year-3		1	Fall
11/12/2019	FP	9A	11	2019	Year-3		1	Fall
11/12/2019	FP	11A	11	2019	Year-3	0	0	Fall
11/12/2019	FP	13A	11	2019	Year-3		1	Fall
11/13/2019	FP	1M	11	2019	Year-3	1	0	Fall
11/13/2019	FP	2M	11	2019	Year-3	1	0	Fall
11/13/2019	FP	3M	11	2019	Year-3		1	Fall
11/13/2019	FP	4M	11	2019	Year-3	1	0	Fall
11/13/2019	FP	5M	11	2019	Year-3	0	0	Fall
11/13/2019	FP	6M	11	2019	Year-3	1	0	Fall
11/13/2019	FP	7M	11	2019	Year-3	1	0	Fall
11/13/2019	FP	10M	11	2019	Year-3	0	0	Fall
11/13/2019	FP	12M	11	2019	Year-3	0	0	Fall
11/13/2019	FP	14M	11	2019	Year-3	1	0	Fall
11/13/2019	FP	8A	11	2019	Year-3		1	Fall
11/13/2019	FP	9A	11	2019	Year-3		1	Fall
11/13/2019	FP	11A	11	2019	Year-3	0	0	Fall
11/13/2019	FP	13A	11	2019	Year-3		1	Fall
11/14/2019	FP	1M	11	2019	Year-3	1	0	Fall
11/14/2019	FP	2M	11	2019	Year-3	1	0	Fall
11/14/2019	FP	3M	11	2019	Year-3		1	Fall
11/14/2019	FP	4M	11	2019	Year-3	1	0	Fall
11/14/2019	FP	5M	11	2019	Year-3	1	0	Fall
11/14/2019	FP	6M	11	2019	Year-3	1	0	Fall
11/14/2019	FP	7M	11	2019	Year-3	1	0	Fall
11/14/2019	FP	10M	11	2019	Year-3	1	0	Fall
11/14/2019	FP	12M	11	2019	Year-3	1	0	Fall
11/14/2019	FP	14M	11	2019	Year-3	1	0	Fall
11/14/2019	FP	8A	11	2019	Year-3		1	Fall
11/14/2019	FP	9A	11	2019	Year-3		1	Fall
11/14/2019	FP	11A	11	2019	Year-3	1	0	Fall
11/14/2019	FP	13A	11	2019	Year-3		1	Fall
11/15/2019	FP	1M	11	2019	Year-3	1	0	Fall
11/15/2019	FP	2M	11	2019	Year-3	1	0	Fall
11/15/2019	FP	3M	11	2019	Year-3		1	Fall
11/15/2019	FP	4M	11	2019	Year-3	1	0	Fall
11/15/2019	FP	5M	11	2019	Year-3	1	0	Fall
11/15/2019	FP	6M	11	2019	Year-3	1	0	Fall
11/15/2019	FP	7M	11	2019	Year-3	1	0	Fall
11/15/2019	FP	10M	11	2019	Year-3	1	0	Fall
11/15/2019	FP	12M	11	2019	Year-3	0	0	Fall

Date	Species	Site	Month	Year	Survey Year	Presence	Data Gap	Season
11/15/2019	FP	14M	11	2019	Year-3	1	0	Fall
11/15/2019	FP	8A	11	2019	Year-3		1	Fall
11/15/2019	FP	9A	11	2019	Year-3		1	Fall
11/15/2019	FP	11A	11	2019	Year-3	1	0	Fall
11/15/2019	FP	13A	11	2019	Year-3		1	Fall
11/16/2019	FP	1M	11	2019	Year-3	1	0	Fall
11/16/2019	FP	2M	11	2019	Year-3	1	0	Fall
11/16/2019	FP	3M	11	2019	Year-3		1	Fall
11/16/2019	FP	4M	11	2019	Year-3	0	0	Fall
11/16/2019	FP	5M	11	2019	Year-3	0	0	Fall
11/16/2019	FP	6M	11	2019	Year-3	1	0	Fall
11/16/2019	FP	7M	11	2019	Year-3	1	0	Fall
11/16/2019	FP	10M	11	2019	Year-3	1	0	Fall
11/16/2019	FP	12M	11	2019	Year-3	1	0	Fall
11/16/2019	FP	14M	11	2019	Year-3	1	0	Fall
11/16/2019	FP	8A	11	2019	Year-3		1	Fall
11/16/2019	FP	9A	11	2019	Year-3		1	Fall
11/16/2019	FP	11A	11	2019	Year-3	1	0	Fall
11/16/2019	FP	13A	11	2019	Year-3		1	Fall
11/17/2019	FP	1M	11	2019	Year-3	1	0	Fall
11/17/2019	FP	2M	11	2019	Year-3	1	0	Fall
11/17/2019	FP	3M	11	2019	Year-3		1	Fall
11/17/2019	FP	4M	11	2019	Year-3	0	0	Fall
11/17/2019	FP	5M	11	2019	Year-3	1	0	Fall
11/17/2019	FP	6M	11	2019	Year-3	1	0	Fall
11/17/2019	FP	7M	11	2019	Year-3	1	0	Fall
11/17/2019	FP	10M	11	2019	Year-3	1	0	Fall
11/17/2019	FP	12M	11	2019	Year-3	1	0	Fall
11/17/2019	FP	14M	11	2019	Year-3	1	0	Fall
11/17/2019	FP	8A	11	2019	Year-3		1	Fall
11/17/2019	FP	9A	11	2019	Year-3		1	Fall
11/17/2019	FP	11A	11	2019	Year-3	1	0	Fall
11/17/2019	FP	13A	11	2019	Year-3		1	Fall
11/18/2019	FP	1M	11	2019	Year-3	1	0	Fall
11/18/2019	FP	2M	11	2019	Year-3	1	0	Fall
11/18/2019	FP	3M	11	2019	Year-3		1	Fall
11/18/2019	FP	4M	11	2019	Year-3	1	0	Fall
11/18/2019	FP	5M	11	2019	Year-3	1	0	Fall
11/18/2019	FP	6M	11	2019	Year-3	0	0	Fall
11/18/2019	FP	7M	11	2019	Year-3	1	0	Fall
11/18/2019	FP	10M	11	2019	Year-3	1	0	Fall
11/18/2019	FP	12M	11	2019	Year-3	1	0	Fall
11/18/2019	FP	14M	11	2019	Year-3	1	0	Fall
11/18/2019	FP	8A	11	2019	Year-3		1	Fall

Date	Species	Site	Month	Year	Survey Year	Presence	Data Gap	Season
11/18/2019	FP	9A	11	2019	Year-3		1	Fall
11/18/2019	FP	11A	11	2019	Year-3	1	0	Fall
11/18/2019	FP	13A	11	2019	Year-3		1	Fall
11/19/2019	FP	1M	11	2019	Year-3	1	0	Fall
11/19/2019	FP	2M	11	2019	Year-3	1	0	Fall
11/19/2019	FP	3M	11	2019	Year-3		1	Fall
11/19/2019	FP	4M	11	2019	Year-3	1	0	Fall
11/19/2019	FP	5M	11	2019	Year-3	1	0	Fall
11/19/2019	FP	6M	11	2019	Year-3	1	0	Fall
11/19/2019	FP	7M	11	2019	Year-3	1	0	Fall
11/19/2019	FP	10M	11	2019	Year-3	1	0	Fall
11/19/2019	FP	12M	11	2019	Year-3	1	0	Fall
11/19/2019	FP	14M	11	2019	Year-3	1	0	Fall
11/19/2019	FP	8A	11	2019	Year-3		1	Fall
11/19/2019	FP	9A	11	2019	Year-3		1	Fall
11/19/2019	FP	11A	11	2019	Year-3	1	0	Fall
11/19/2019	FP	13A	11	2019	Year-3		1	Fall
11/20/2019	FP	1M	11	2019	Year-3	1	0	Fall
11/20/2019	FP	2M	11	2019	Year-3	1	0	Fall
11/20/2019	FP	3M	11	2019	Year-3		1	Fall
11/20/2019	FP	4M	11	2019	Year-3	1	0	Fall
11/20/2019	FP	5M	11	2019	Year-3	1	0	Fall
11/20/2019	FP	6M	11	2019	Year-3	1	0	Fall
11/20/2019	FP	7M	11	2019	Year-3	1	0	Fall
11/20/2019	FP	10M	11	2019	Year-3	1	0	Fall
11/20/2019	FP	12M	11	2019	Year-3	1	0	Fall
11/20/2019	FP	14M	11	2019	Year-3	1	0	Fall
11/20/2019	FP	8A	11	2019	Year-3		1	Fall
11/20/2019	FP	9A	11	2019	Year-3		1	Fall
11/20/2019	FP	11A	11	2019	Year-3	1	0	Fall
11/20/2019	FP	13A	11	2019	Year-3		1	Fall
11/21/2019	FP	1M	11	2019	Year-3	1	0	Fall
11/21/2019	FP	2M	11	2019	Year-3	1	0	Fall
11/21/2019	FP	3M	11	2019	Year-3		1	Fall
11/21/2019	FP	4M	11	2019	Year-3	1	0	Fall
11/21/2019	FP	5M	11	2019	Year-3	0	0	Fall
11/21/2019	FP	6M	11	2019	Year-3	1	0	Fall
11/21/2019	FP	7M	11	2019	Year-3	1	0	Fall
11/21/2019	FP	10M	11	2019	Year-3	1	0	Fall
11/21/2019	FP	12M	11	2019	Year-3	1	0	Fall
11/21/2019	FP	14M	11	2019	Year-3	1	0	Fall
11/21/2019	FP	8A	11	2019	Year-3		1	Fall
11/21/2019	FP	9A	11	2019	Year-3		1	Fall
11/21/2019	FP	11A	11	2019	Year-3	1	0	Fall

Date	Species	Site	Month	Year	Survey Year	Presence	Data Gap	Season
11/21/2019	FP	13A	11	2019	Year-3		1	Fall
11/22/2019	FP	1M	11	2019	Year-3	1	0	Fall
11/22/2019	FP	2M	11	2019	Year-3	1	0	Fall
11/22/2019	FP	3M	11	2019	Year-3		1	Fall
11/22/2019	FP	4M	11	2019	Year-3	1	0	Fall
11/22/2019	FP	5M	11	2019	Year-3	1	0	Fall
11/22/2019	FP	6M	11	2019	Year-3	1	0	Fall
11/22/2019	FP	7M	11	2019	Year-3	1	0	Fall
11/22/2019	FP	10M	11	2019	Year-3	1	0	Fall
11/22/2019	FP	12M	11	2019	Year-3	0	0	Fall
11/22/2019	FP	14M	11	2019	Year-3	1	0	Fall
11/22/2019	FP	8A	11	2019	Year-3		1	Fall
11/22/2019	FP	9A	11	2019	Year-3		1	Fall
11/22/2019	FP	11A	11	2019	Year-3	1	0	Fall
11/22/2019	FP	13A	11	2019	Year-3		1	Fall
11/23/2019	FP	1M	11	2019	Year-3	1	0	Fall
11/23/2019	FP	2M	11	2019	Year-3	1	0	Fall
11/23/2019	FP	3M	11	2019	Year-3		1	Fall
11/23/2019	FP	4M	11	2019	Year-3	1	0	Fall
11/23/2019	FP	5M	11	2019	Year-3	1	0	Fall
11/23/2019	FP	6M	11	2019	Year-3	1	0	Fall
11/23/2019	FP	7M	11	2019	Year-3	1	0	Fall
11/23/2019	FP	10M	11	2019	Year-3	1	0	Fall
11/23/2019	FP	12M	11	2019	Year-3	1	0	Fall
11/23/2019	FP	14M	11	2019	Year-3	1	0	Fall
11/23/2019	FP	8A	11	2019	Year-3		1	Fall
11/23/2019	FP	9A	11	2019	Year-3		1	Fall
11/23/2019	FP	11A	11	2019	Year-3	1	0	Fall
11/23/2019	FP	13A	11	2019	Year-3		1	Fall
11/24/2019	FP	1M	11	2019	Year-3	1	0	Fall
11/24/2019	FP	2M	11	2019	Year-3	1	0	Fall
11/24/2019	FP	3M	11	2019	Year-3		1	Fall
11/24/2019	FP	4M	11	2019	Year-3	1	0	Fall
11/24/2019	FP	5M	11	2019	Year-3	1	0	Fall
11/24/2019	FP	6M	11	2019	Year-3	1	0	Fall
11/24/2019	FP	7M	11	2019	Year-3	1	0	Fall
11/24/2019	FP	10M	11	2019	Year-3	1	0	Fall
11/24/2019	FP	12M	11	2019	Year-3	0	0	Fall
11/24/2019	FP	14M	11	2019	Year-3	1	0	Fall
11/24/2019	FP	8A	11	2019	Year-3		1	Fall
11/24/2019	FP	9A	11	2019	Year-3		1	Fall
11/24/2019	FP	11A	11	2019	Year-3	1	0	Fall
11/24/2019	FP	13A	11	2019	Year-3		1	Fall
11/25/2019	FP	1M	11	2019	Year-3	1	0	Fall

Date	Species	Site	Month	Year	Survey Year	Presence	Data Gap	Season
11/25/2019	FP	2M	11	2019	Year-3	1	0	Fall
11/25/2019	FP	3M	11	2019	Year-3		1	Fall
11/25/2019	FP	4M	11	2019	Year-3	1	0	Fall
11/25/2019	FP	5M	11	2019	Year-3	1	0	Fall
11/25/2019	FP	6M	11	2019	Year-3	1	0	Fall
11/25/2019	FP	7M	11	2019	Year-3	1	0	Fall
11/25/2019	FP	10M	11	2019	Year-3	1	0	Fall
11/25/2019	FP	12M	11	2019	Year-3	1	0	Fall
11/25/2019	FP	14M	11	2019	Year-3	1	0	Fall
11/25/2019	FP	8A	11	2019	Year-3		1	Fall
11/25/2019	FP	9A	11	2019	Year-3		1	Fall
11/25/2019	FP	11A	11	2019	Year-3	1	0	Fall
11/25/2019	FP	13A	11	2019	Year-3		1	Fall
11/26/2019	FP	1M	11	2019	Year-3	1	0	Fall
11/26/2019	FP	2M	11	2019	Year-3	1	0	Fall
11/26/2019	FP	3M	11	2019	Year-3		1	Fall
11/26/2019	FP	4M	11	2019	Year-3	1	0	Fall
11/26/2019	FP	5M	11	2019	Year-3	1	0	Fall
11/26/2019	FP	6M	11	2019	Year-3	1	0	Fall
11/26/2019	FP	7M	11	2019	Year-3	1	0	Fall
11/26/2019	FP	10M	11	2019	Year-3	1	0	Fall
11/26/2019	FP	12M	11	2019	Year-3	1	0	Fall
11/26/2019	FP	14M	11	2019	Year-3	1	0	Fall
11/26/2019	FP	8A	11	2019	Year-3		1	Fall
11/26/2019	FP	9A	11	2019	Year-3		1	Fall
11/26/2019	FP	11A	11	2019	Year-3	1	0	Fall
11/26/2019	FP	13A	11	2019	Year-3		1	Fall
11/27/2019	FP	1M	11	2019	Year-3	1	0	Fall
11/27/2019	FP	2M	11	2019	Year-3	1	0	Fall
11/27/2019	FP	3M	11	2019	Year-3		1	Fall
11/27/2019	FP	4M	11	2019	Year-3	1	0	Fall
11/27/2019	FP	5M	11	2019	Year-3	1	0	Fall
11/27/2019	FP	6M	11	2019	Year-3	0	0	Fall
11/27/2019	FP	7M	11	2019	Year-3	0	0	Fall
11/27/2019	FP	10M	11	2019	Year-3	1	0	Fall
11/27/2019	FP	12M	11	2019	Year-3	1	0	Fall
11/27/2019	FP	14M	11	2019	Year-3	1	0	Fall
11/27/2019	FP	8A	11	2019	Year-3		1	Fall
11/27/2019	FP	9A	11	2019	Year-3		1	Fall
11/27/2019	FP	11A	11	2019	Year-3	0	0	Fall
11/27/2019	FP	13A	11	2019	Year-3		1	Fall
11/28/2019	FP	1M	11	2019	Year-3	1	0	Fall
11/28/2019	FP	2M	11	2019	Year-3	1	0	Fall
11/28/2019	FP	3M	11	2019	Year-3		1	Fall

Date	Species	Site	Month	Year	Survey Year	Presence	Data Gap	Season
11/28/2019	FP	4M	11	2019	Year-3	1	0	Fall
11/28/2019	FP	5M	11	2019	Year-3	0	0	Fall
11/28/2019	FP	6M	11	2019	Year-3	0	0	Fall
11/28/2019	FP	7M	11	2019	Year-3	0	0	Fall
11/28/2019	FP	10M	11	2019	Year-3	1	0	Fall
11/28/2019	FP	12M	11	2019	Year-3	0	0	Fall
11/28/2019	FP	14M	11	2019	Year-3	1	0	Fall
11/28/2019	FP	8A	11	2019	Year-3		1	Fall
11/28/2019	FP	9A	11	2019	Year-3		1	Fall
11/28/2019	FP	11A	11	2019	Year-3	1	0	Fall
11/28/2019	FP	13A	11	2019	Year-3		1	Fall
11/29/2019	FP	1M	11	2019	Year-3	1	0	Fall
11/29/2019	FP	2M	11	2019	Year-3	1	0	Fall
11/29/2019	FP	3M	11	2019	Year-3		1	Fall
11/29/2019	FP	4M	11	2019	Year-3	1	0	Fall
11/29/2019	FP	5M	11	2019	Year-3	0	0	Fall
11/29/2019	FP	6M	11	2019	Year-3	0	0	Fall
11/29/2019	FP	7M	11	2019	Year-3	0	0	Fall
11/29/2019	FP	10M	11	2019	Year-3	0	0	Fall
11/29/2019	FP	12M	11	2019	Year-3	0	0	Fall
11/29/2019	FP	14M	11	2019	Year-3	1	0	Fall
11/29/2019	FP	8A	11	2019	Year-3		1	Fall
11/29/2019	FP	9A	11	2019	Year-3		1	Fall
11/29/2019	FP	11A	11	2019	Year-3	1	0	Fall
11/29/2019	FP	13A	11	2019	Year-3		1	Fall
11/30/2019	FP	1M	11	2019	Year-3	1	0	Fall
11/30/2019	FP	2M	11	2019	Year-3	1	0	Fall
11/30/2019	FP	3M	11	2019	Year-3		1	Fall
11/30/2019	FP	4M	11	2019	Year-3	1	0	Fall
11/30/2019	FP	5M	11	2019	Year-3	1	0	Fall
11/30/2019	FP	6M	11	2019	Year-3	0	0	Fall
11/30/2019	FP	7M	11	2019	Year-3	0	0	Fall
11/30/2019	FP	10M	11	2019	Year-3	0	0	Fall
11/30/2019	FP	12M	11	2019	Year-3	0	0	Fall
11/30/2019	FP	14M	11	2019	Year-3	1	0	Fall
11/30/2019	FP	8A	11	2019	Year-3		1	Fall
11/30/2019	FP	9A	11	2019	Year-3		1	Fall
11/30/2019	FP	11A	11	2019	Year-3	0	0	Fall
11/30/2019	FP	13A	11	2019	Year-3		1	Fall
12/1/2019	FP	1M	12	2019	Year-3	1	0	Winter
12/1/2019	FP	2M	12	2019	Year-3	1	0	Winter
12/1/2019	FP	3M	12	2019	Year-3		1	Winter
12/1/2019	FP	4M	12	2019	Year-3	1	0	Winter
12/1/2019	FP	5M	12	2019	Year-3	1	0	Winter

Date	Species	Site	Month	Year	Survey Year	Presence	Data Gap	Season
12/1/2019	FP	6M	12	2019	Year-3	1	0	Winter
12/1/2019	FP	7M	12	2019	Year-3	1	0	Winter
12/1/2019	FP	10M	12	2019	Year-3	1	0	Winter
12/1/2019	FP	12M	12	2019	Year-3	0	0	Winter
12/1/2019	FP	14M	12	2019	Year-3	1	0	Winter
12/1/2019	FP	8A	12	2019	Year-3		1	Winter
12/1/2019	FP	9A	12	2019	Year-3		1	Winter
12/1/2019	FP	11A	12	2019	Year-3	1	0	Winter
12/1/2019	FP	13A	12	2019	Year-3		1	Winter
12/2/2019	FP	1M	12	2019	Year-3	1	0	Winter
12/2/2019	FP	2M	12	2019	Year-3	1	0	Winter
12/2/2019	FP	3M	12	2019	Year-3		1	Winter
12/2/2019	FP	4M	12	2019	Year-3	0	0	Winter
12/2/2019	FP	5M	12	2019	Year-3	0	0	Winter
12/2/2019	FP	6M	12	2019	Year-3	0	0	Winter
12/2/2019	FP	7M	12	2019	Year-3	1	0	Winter
12/2/2019	FP	10M	12	2019	Year-3	1	0	Winter
12/2/2019	FP	12M	12	2019	Year-3	1	0	Winter
12/2/2019	FP	14M	12	2019	Year-3	1	0	Winter
12/2/2019	FP	8A	12	2019	Year-3		1	Winter
12/2/2019	FP	9A	12	2019	Year-3		1	Winter
12/2/2019	FP	11A	12	2019	Year-3	0	0	Winter
12/2/2019	FP	13A	12	2019	Year-3		1	Winter
12/3/2019	FP	1M	12	2019	Year-3	1	0	Winter
12/3/2019	FP	2M	12	2019	Year-3	1	0	Winter
12/3/2019	FP	3M	12	2019	Year-3		1	Winter
12/3/2019	FP	4M	12	2019	Year-3	1	0	Winter
12/3/2019	FP	5M	12	2019	Year-3	0	0	Winter
12/3/2019	FP	6M	12	2019	Year-3	0	0	Winter
12/3/2019	FP	7M	12	2019	Year-3	1	0	Winter
12/3/2019	FP	10M	12	2019	Year-3	0	0	Winter
12/3/2019	FP	12M	12	2019	Year-3	1	0	Winter
12/3/2019	FP	14M	12	2019	Year-3	1	0	Winter
12/3/2019	FP	8A	12	2019	Year-3		1	Winter
12/3/2019	FP	9A	12	2019	Year-3		1	Winter
12/3/2019	FP	11A	12	2019	Year-3	1	0	Winter
12/3/2019	FP	13A	12	2019	Year-3		1	Winter
12/4/2019	FP	1M	12	2019	Year-3	1	0	Winter
12/4/2019	FP	2M	12	2019	Year-3	1	0	Winter
12/4/2019	FP	3M	12	2019	Year-3		1	Winter
12/4/2019	FP	4M	12	2019	Year-3	1	0	Winter
12/4/2019	FP	5M	12	2019	Year-3	0	0	Winter
12/4/2019	FP	6M	12	2019	Year-3	0	0	Winter
12/4/2019	FP	7M	12	2019	Year-3	0	0	Winter

Date	Species	Site	Month	Year	Survey Year	Presence	Data Gap	Season
12/4/2019	FP	10M	12	2019	Year-3	0	0	Winter
12/4/2019	FP	12M	12	2019	Year-3	0	0	Winter
12/4/2019	FP	14M	12	2019	Year-3	1	0	Winter
12/4/2019	FP	8A	12	2019	Year-3		1	Winter
12/4/2019	FP	9A	12	2019	Year-3		1	Winter
12/4/2019	FP	11A	12	2019	Year-3	0	0	Winter
12/4/2019	FP	13A	12	2019	Year-3		1	Winter
12/5/2019	FP	1M	12	2019	Year-3	1	0	Winter
12/5/2019	FP	2M	12	2019	Year-3	1	0	Winter
12/5/2019	FP	3M	12	2019	Year-3		1	Winter
12/5/2019	FP	4M	12	2019	Year-3	0	0	Winter
12/5/2019	FP	5M	12	2019	Year-3	0	0	Winter
12/5/2019	FP	6M	12	2019	Year-3	0	0	Winter
12/5/2019	FP	7M	12	2019	Year-3	0	0	Winter
12/5/2019	FP	10M	12	2019	Year-3	0	0	Winter
12/5/2019	FP	12M	12	2019	Year-3	0	0	Winter
12/5/2019	FP	14M	12	2019	Year-3	1	0	Winter
12/5/2019	FP	8A	12	2019	Year-3		1	Winter
12/5/2019	FP	9A	12	2019	Year-3		1	Winter
12/5/2019	FP	11A	12	2019	Year-3	0	0	Winter
12/5/2019	FP	13A	12	2019	Year-3		1	Winter
12/6/2019	FP	1M	12	2019	Year-3	1	0	Winter
12/6/2019	FP	2M	12	2019	Year-3	1	0	Winter
12/6/2019	FP	3M	12	2019	Year-3		1	Winter
12/6/2019	FP	4M	12	2019	Year-3	1	0	Winter
12/6/2019	FP	5M	12	2019	Year-3	0	0	Winter
12/6/2019	FP	6M	12	2019	Year-3	0	0	Winter
12/6/2019	FP	7M	12	2019	Year-3	1	0	Winter
12/6/2019	FP	10M	12	2019	Year-3	0	0	Winter
12/6/2019	FP	12M	12	2019	Year-3	1	0	Winter
12/6/2019	FP	14M	12	2019	Year-3	1	0	Winter
12/6/2019	FP	8A	12	2019	Year-3		1	Winter
12/6/2019	FP	9A	12	2019	Year-3		1	Winter
12/6/2019	FP	11A	12	2019	Year-3	0	0	Winter
12/6/2019	FP	13A	12	2019	Year-3		1	Winter
12/7/2019	FP	1M	12	2019	Year-3	1	0	Winter
12/7/2019	FP	2M	12	2019	Year-3	1	0	Winter
12/7/2019	FP	3M	12	2019	Year-3		1	Winter
12/7/2019	FP	4M	12	2019	Year-3	1	0	Winter
12/7/2019	FP	5M	12	2019	Year-3	1	0	Winter
12/7/2019	FP	6M	12	2019	Year-3	1	0	Winter
12/7/2019	FP	7M	12	2019	Year-3	1	0	Winter
12/7/2019	FP	10M	12	2019	Year-3	1	0	Winter
12/7/2019	FP	12M	12	2019	Year-3	1	0	Winter

Date	Species	Site	Month	Year	Survey Year	Presence	Data Gap	Season
12/7/2019	FP	14M	12	2019	Year-3	1	0	Winter
12/7/2019	FP	8A	12	2019	Year-3		1	Winter
12/7/2019	FP	9A	12	2019	Year-3		1	Winter
12/7/2019	FP	11A	12	2019	Year-3	1	0	Winter
12/7/2019	FP	13A	12	2019	Year-3		1	Winter
12/8/2019	FP	1M	12	2019	Year-3	1	0	Winter
12/8/2019	FP	2M	12	2019	Year-3	1	0	Winter
12/8/2019	FP	3M	12	2019	Year-3		1	Winter
12/8/2019	FP	4M	12	2019	Year-3	1	0	Winter
12/8/2019	FP	5M	12	2019	Year-3	0	0	Winter
12/8/2019	FP	6M	12	2019	Year-3	1	0	Winter
12/8/2019	FP	7M	12	2019	Year-3	1	0	Winter
12/8/2019	FP	10M	12	2019	Year-3	1	0	Winter
12/8/2019	FP	12M	12	2019	Year-3	1	0	Winter
12/8/2019	FP	14M	12	2019	Year-3	1	0	Winter
12/8/2019	FP	8A	12	2019	Year-3		1	Winter
12/8/2019	FP	9A	12	2019	Year-3		1	Winter
12/8/2019	FP	11A	12	2019	Year-3	1	0	Winter
12/8/2019	FP	13A	12	2019	Year-3		1	Winter
12/9/2019	FP	1M	12	2019	Year-3	1	0	Winter
12/9/2019	FP	2M	12	2019	Year-3	1	0	Winter
12/9/2019	FP	3M	12	2019	Year-3		1	Winter
12/9/2019	FP	4M	12	2019	Year-3	1	0	Winter
12/9/2019	FP	5M	12	2019	Year-3	1	0	Winter
12/9/2019	FP	6M	12	2019	Year-3	0	0	Winter
12/9/2019	FP	7M	12	2019	Year-3	1	0	Winter
12/9/2019	FP	10M	12	2019	Year-3	1	0	Winter
12/9/2019	FP	12M	12	2019	Year-3	0	0	Winter
12/9/2019	FP	14M	12	2019	Year-3	1	0	Winter
12/9/2019	FP	8A	12	2019	Year-3		1	Winter
12/9/2019	FP	9A	12	2019	Year-3		1	Winter
12/9/2019	FP	11A	12	2019	Year-3	1	0	Winter
12/9/2019	FP	13A	12	2019	Year-3		1	Winter
12/10/2019	FP	1M	12	2019	Year-3	1	0	Winter
12/10/2019	FP	2M	12	2019	Year-3	1	0	Winter
12/10/2019	FP	3M	12	2019	Year-3		1	Winter
12/10/2019	FP	4M	12	2019	Year-3	0	0	Winter
12/10/2019	FP	5M	12	2019	Year-3	1	0	Winter
12/10/2019	FP	6M	12	2019	Year-3	1	0	Winter
12/10/2019	FP	7M	12	2019	Year-3	1	0	Winter
12/10/2019	FP	10M	12	2019	Year-3	1	0	Winter
12/10/2019	FP	12M	12	2019	Year-3	1	0	Winter
12/10/2019	FP	14M	12	2019	Year-3	1	0	Winter
12/10/2019	FP	8A	12	2019	Year-3		1	Winter

Date	Species	Site	Month	Year	Survey Year	Presence	Data Gap	Season
12/10/2019	FP	9A	12	2019	Year-3		1	Winter
12/10/2019	FP	11A	12	2019	Year-3	1	0	Winter
12/10/2019	FP	13A	12	2019	Year-3		1	Winter
12/11/2019	FP	1M	12	2019	Year-3	1	0	Winter
12/11/2019	FP	2M	12	2019	Year-3	1	0	Winter
12/11/2019	FP	3M	12	2019	Year-3		1	Winter
12/11/2019	FP	4M	12	2019	Year-3	1	0	Winter
12/11/2019	FP	5M	12	2019	Year-3	1	0	Winter
12/11/2019	FP	6M	12	2019	Year-3	1	0	Winter
12/11/2019	FP	7M	12	2019	Year-3	1	0	Winter
12/11/2019	FP	10M	12	2019	Year-3	1	0	Winter
12/11/2019	FP	12M	12	2019	Year-3	1	0	Winter
12/11/2019	FP	14M	12	2019	Year-3	1	0	Winter
12/11/2019	FP	8A	12	2019	Year-3		1	Winter
12/11/2019	FP	9A	12	2019	Year-3		1	Winter
12/11/2019	FP	11A	12	2019	Year-3	1	0	Winter
12/11/2019	FP	13A	12	2019	Year-3		1	Winter
12/12/2019	FP	1M	12	2019	Year-3	1	0	Winter
12/12/2019	FP	2M	12	2019	Year-3	1	0	Winter
12/12/2019	FP	3M	12	2019	Year-3		1	Winter
12/12/2019	FP	4M	12	2019	Year-3	1	0	Winter
12/12/2019	FP	5M	12	2019	Year-3	1	0	Winter
12/12/2019	FP	6M	12	2019	Year-3	1	0	Winter
12/12/2019	FP	7M	12	2019	Year-3	1	0	Winter
12/12/2019	FP	10M	12	2019	Year-3	1	0	Winter
12/12/2019	FP	12M	12	2019	Year-3	0	0	Winter
12/12/2019	FP	14M	12	2019	Year-3	1	0	Winter
12/12/2019	FP	8A	12	2019	Year-3		1	Winter
12/12/2019	FP	9A	12	2019	Year-3		1	Winter
12/12/2019	FP	11A	12	2019	Year-3	1	0	Winter
12/12/2019	FP	13A	12	2019	Year-3		1	Winter
12/13/2019	FP	1M	12	2019	Year-3	1	0	Winter
12/13/2019	FP	2M	12	2019	Year-3	1	0	Winter
12/13/2019	FP	3M	12	2019	Year-3		1	Winter
12/13/2019	FP	4M	12	2019	Year-3	1	0	Winter
12/13/2019	FP	5M	12	2019	Year-3	1	0	Winter
12/13/2019	FP	6M	12	2019	Year-3	1	0	Winter
12/13/2019	FP	7M	12	2019	Year-3	1	0	Winter
12/13/2019	FP	10M	12	2019	Year-3	0	0	Winter
12/13/2019	FP	12M	12	2019	Year-3	1	0	Winter
12/13/2019	FP	14M	12	2019	Year-3	1	0	Winter
12/13/2019	FP	8A	12	2019	Year-3		1	Winter
12/13/2019	FP	9A	12	2019	Year-3		1	Winter
12/13/2019	FP	11A	12	2019	Year-3	1	0	Winter

Date	Species	Site	Month	Year	Survey Year	Presence	Data Gap	Season
12/13/2019	FP	13A	12	2019	Year-3		1	Winter
12/14/2019	FP	1M	12	2019	Year-3	1	0	Winter
12/14/2019	FP	2M	12	2019	Year-3	1	0	Winter
12/14/2019	FP	3M	12	2019	Year-3		1	Winter
12/14/2019	FP	4M	12	2019	Year-3	1	0	Winter
12/14/2019	FP	5M	12	2019	Year-3	1	0	Winter
12/14/2019	FP	6M	12	2019	Year-3	1	0	Winter
12/14/2019	FP	7M	12	2019	Year-3	1	0	Winter
12/14/2019	FP	10M	12	2019	Year-3	1	0	Winter
12/14/2019	FP	12M	12	2019	Year-3	1	0	Winter
12/14/2019	FP	14M	12	2019	Year-3	1	0	Winter
12/14/2019	FP	8A	12	2019	Year-3		1	Winter
12/14/2019	FP	9A	12	2019	Year-3		1	Winter
12/14/2019	FP	11A	12	2019	Year-3	1	0	Winter
12/14/2019	FP	13A	12	2019	Year-3		1	Winter
12/15/2019	FP	1M	12	2019	Year-3	1	0	Winter
12/15/2019	FP	2M	12	2019	Year-3	1	0	Winter
12/15/2019	FP	3M	12	2019	Year-3		1	Winter
12/15/2019	FP	4M	12	2019	Year-3	0	0	Winter
12/15/2019	FP	5M	12	2019	Year-3	1	0	Winter
12/15/2019	FP	6M	12	2019	Year-3	1	0	Winter
12/15/2019	FP	7M	12	2019	Year-3	1	0	Winter
12/15/2019	FP	10M	12	2019	Year-3	1	0	Winter
12/15/2019	FP	12M	12	2019	Year-3	1	0	Winter
12/15/2019	FP	14M	12	2019	Year-3	1	0	Winter
12/15/2019	FP	8A	12	2019	Year-3		1	Winter
12/15/2019	FP	9A	12	2019	Year-3		1	Winter
12/15/2019	FP	11A	12	2019	Year-3	1	0	Winter
12/15/2019	FP	13A	12	2019	Year-3		1	Winter
12/16/2019	FP	1M	12	2019	Year-3	1	0	Winter
12/16/2019	FP	2M	12	2019	Year-3	1	0	Winter
12/16/2019	FP	3M	12	2019	Year-3		1	Winter
12/16/2019	FP	4M	12	2019	Year-3	1	0	Winter
12/16/2019	FP	5M	12	2019	Year-3	1	0	Winter
12/16/2019	FP	6M	12	2019	Year-3	1	0	Winter
12/16/2019	FP	7M	12	2019	Year-3	1	0	Winter
12/16/2019	FP	10M	12	2019	Year-3	0	0	Winter
12/16/2019	FP	12M	12	2019	Year-3	1	0	Winter
12/16/2019	FP	14M	12	2019	Year-3	1	0	Winter
12/16/2019	FP	8A	12	2019	Year-3		1	Winter
12/16/2019	FP	9A	12	2019	Year-3		1	Winter
12/16/2019	FP	11A	12	2019	Year-3	1	0	Winter
12/16/2019	FP	13A	12	2019	Year-3		1	Winter
12/17/2019	FP	1M	12	2019	Year-3	1	0	Winter

Date	Species	Site	Month	Year	Survey Year	Presence	Data Gap	Season
12/17/2019	FP	2M	12	2019	Year-3	1	0	Winter
12/17/2019	FP	3M	12	2019	Year-3		1	Winter
12/17/2019	FP	4M	12	2019	Year-3	1	0	Winter
12/17/2019	FP	5M	12	2019	Year-3	1	0	Winter
12/17/2019	FP	6M	12	2019	Year-3	1	0	Winter
12/17/2019	FP	7M	12	2019	Year-3	1	0	Winter
12/17/2019	FP	10M	12	2019	Year-3	1	0	Winter
12/17/2019	FP	12M	12	2019	Year-3	1	0	Winter
12/17/2019	FP	14M	12	2019	Year-3	1	0	Winter
12/17/2019	FP	8A	12	2019	Year-3		1	Winter
12/17/2019	FP	9A	12	2019	Year-3		1	Winter
12/17/2019	FP	11A	12	2019	Year-3	1	0	Winter
12/17/2019	FP	13A	12	2019	Year-3		1	Winter
12/18/2019	FP	1M	12	2019	Year-3	1	0	Winter
12/18/2019	FP	2M	12	2019	Year-3	1	0	Winter
12/18/2019	FP	3M	12	2019	Year-3		1	Winter
12/18/2019	FP	4M	12	2019	Year-3	1	0	Winter
12/18/2019	FP	5M	12	2019	Year-3	1	0	Winter
12/18/2019	FP	6M	12	2019	Year-3	1	0	Winter
12/18/2019	FP	7M	12	2019	Year-3	1	0	Winter
12/18/2019	FP	10M	12	2019	Year-3	1	0	Winter
12/18/2019	FP	12M	12	2019	Year-3	1	0	Winter
12/18/2019	FP	14M	12	2019	Year-3	1	0	Winter
12/18/2019	FP	8A	12	2019	Year-3		1	Winter
12/18/2019	FP	9A	12	2019	Year-3		1	Winter
12/18/2019	FP	11A	12	2019	Year-3	1	0	Winter
12/18/2019	FP	13A	12	2019	Year-3		1	Winter
12/19/2019	FP	1M	12	2019	Year-3	1	0	Winter
12/19/2019	FP	2M	12	2019	Year-3	1	0	Winter
12/19/2019	FP	3M	12	2019	Year-3		1	Winter
12/19/2019	FP	4M	12	2019	Year-3	1	0	Winter
12/19/2019	FP	5M	12	2019	Year-3	1	0	Winter
12/19/2019	FP	6M	12	2019	Year-3	1	0	Winter
12/19/2019	FP	7M	12	2019	Year-3	1	0	Winter
12/19/2019	FP	10M	12	2019	Year-3	1	0	Winter
12/19/2019	FP	12M	12	2019	Year-3	1	0	Winter
12/19/2019	FP	14M	12	2019	Year-3	1	0	Winter
12/19/2019	FP	8A	12	2019	Year-3		1	Winter
12/19/2019	FP	9A	12	2019	Year-3		1	Winter
12/19/2019	FP	11A	12	2019	Year-3	1	0	Winter
12/19/2019	FP	13A	12	2019	Year-3		1	Winter
12/20/2019	FP	1M	12	2019	Year-3	1	0	Winter
12/20/2019	FP	2M	12	2019	Year-3	1	0	Winter
12/20/2019	FP	3M	12	2019	Year-3		1	Winter

Date	Species	Site	Month	Year	Survey Year	Presence	Data Gap	Season
12/20/2019	FP	4M	12	2019	Year-3	1	0	Winter
12/20/2019	FP	5M	12	2019	Year-3	0	0	Winter
12/20/2019	FP	6M	12	2019	Year-3	1	0	Winter
12/20/2019	FP	7M	12	2019	Year-3	1	0	Winter
12/20/2019	FP	10M	12	2019	Year-3	1	0	Winter
12/20/2019	FP	12M	12	2019	Year-3	1	0	Winter
12/20/2019	FP	14M	12	2019	Year-3	1	0	Winter
12/20/2019	FP	8A	12	2019	Year-3		1	Winter
12/20/2019	FP	9A	12	2019	Year-3		1	Winter
12/20/2019	FP	11A	12	2019	Year-3	1	0	Winter
12/20/2019	FP	13A	12	2019	Year-3		1	Winter
12/21/2019	FP	1M	12	2019	Year-3	1	0	Winter
12/21/2019	FP	2M	12	2019	Year-3	1	0	Winter
12/21/2019	FP	3M	12	2019	Year-3		1	Winter
12/21/2019	FP	4M	12	2019	Year-3	1	0	Winter
12/21/2019	FP	5M	12	2019	Year-3	1	0	Winter
12/21/2019	FP	6M	12	2019	Year-3	1	0	Winter
12/21/2019	FP	7M	12	2019	Year-3	1	0	Winter
12/21/2019	FP	10M	12	2019	Year-3	1	0	Winter
12/21/2019	FP	12M	12	2019	Year-3	1	0	Winter
12/21/2019	FP	14M	12	2019	Year-3	1	0	Winter
12/21/2019	FP	8A	12	2019	Year-3		1	Winter
12/21/2019	FP	9A	12	2019	Year-3		1	Winter
12/21/2019	FP	11A	12	2019	Year-3	1	0	Winter
12/21/2019	FP	13A	12	2019	Year-3		1	Winter
12/22/2019	FP	1M	12	2019	Year-3	1	0	Winter
12/22/2019	FP	2M	12	2019	Year-3	1	0	Winter
12/22/2019	FP	3M	12	2019	Year-3		1	Winter
12/22/2019	FP	4M	12	2019	Year-3	1	0	Winter
12/22/2019	FP	5M	12	2019	Year-3	1	0	Winter
12/22/2019	FP	6M	12	2019	Year-3	1	0	Winter
12/22/2019	FP	7M	12	2019	Year-3	1	0	Winter
12/22/2019	FP	10M	12	2019	Year-3	1	0	Winter
12/22/2019	FP	12M	12	2019	Year-3	1	0	Winter
12/22/2019	FP	14M	12	2019	Year-3	1	0	Winter
12/22/2019	FP	8A	12	2019	Year-3		1	Winter
12/22/2019	FP	9A	12	2019	Year-3		1	Winter
12/22/2019	FP	11A	12	2019	Year-3	1	0	Winter
12/22/2019	FP	13A	12	2019	Year-3		1	Winter
12/23/2019	FP	1M	12	2019	Year-3	1	0	Winter
12/23/2019	FP	2M	12	2019	Year-3	1	0	Winter
12/23/2019	FP	3M	12	2019	Year-3		1	Winter
12/23/2019	FP	4M	12	2019	Year-3	1	0	Winter
12/23/2019	FP	5M	12	2019	Year-3	1	0	Winter

Date	Species	Site	Month	Year	Survey Year	Presence	Data Gap	Season
12/23/2019	FP	6M	12	2019	Year-3	1	0	Winter
12/23/2019	FP	7M	12	2019	Year-3	1	0	Winter
12/23/2019	FP	10M	12	2019	Year-3	1	0	Winter
12/23/2019	FP	12M	12	2019	Year-3	1	0	Winter
12/23/2019	FP	14M	12	2019	Year-3	1	0	Winter
12/23/2019	FP	8A	12	2019	Year-3		1	Winter
12/23/2019	FP	9A	12	2019	Year-3		1	Winter
12/23/2019	FP	11A	12	2019	Year-3	1	0	Winter
12/23/2019	FP	13A	12	2019	Year-3		1	Winter
12/24/2019	FP	1M	12	2019	Year-3	1	0	Winter
12/24/2019	FP	2M	12	2019	Year-3	1	0	Winter
12/24/2019	FP	3M	12	2019	Year-3		1	Winter
12/24/2019	FP	4M	12	2019	Year-3	1	0	Winter
12/24/2019	FP	5M	12	2019	Year-3	1	0	Winter
12/24/2019	FP	6M	12	2019	Year-3	1	0	Winter
12/24/2019	FP	7M	12	2019	Year-3	1	0	Winter
12/24/2019	FP	10M	12	2019	Year-3	0	0	Winter
12/24/2019	FP	12M	12	2019	Year-3	1	0	Winter
12/24/2019	FP	14M	12	2019	Year-3	1	0	Winter
12/24/2019	FP	8A	12	2019	Year-3		1	Winter
12/24/2019	FP	9A	12	2019	Year-3		1	Winter
12/24/2019	FP	11A	12	2019	Year-3	1	0	Winter
12/24/2019	FP	13A	12	2019	Year-3		1	Winter
12/25/2019	FP	1M	12	2019	Year-3	1	0	Winter
12/25/2019	FP	2M	12	2019	Year-3	1	0	Winter
12/25/2019	FP	3M	12	2019	Year-3		1	Winter
12/25/2019	FP	4M	12	2019	Year-3	1	0	Winter
12/25/2019	FP	5M	12	2019	Year-3	0	0	Winter
12/25/2019	FP	6M	12	2019	Year-3	1	0	Winter
12/25/2019	FP	7M	12	2019	Year-3	1	0	Winter
12/25/2019	FP	10M	12	2019	Year-3	1	0	Winter
12/25/2019	FP	12M	12	2019	Year-3	1	0	Winter
12/25/2019	FP	14M	12	2019	Year-3	1	0	Winter
12/25/2019	FP	8A	12	2019	Year-3		1	Winter
12/25/2019	FP	9A	12	2019	Year-3		1	Winter
12/25/2019	FP	11A	12	2019	Year-3	1	0	Winter
12/25/2019	FP	13A	12	2019	Year-3		1	Winter
12/26/2019	FP	1M	12	2019	Year-3	1	0	Winter
12/26/2019	FP	2M	12	2019	Year-3	1	0	Winter
12/26/2019	FP	3M	12	2019	Year-3		1	Winter
12/26/2019	FP	4M	12	2019	Year-3	1	0	Winter
12/26/2019	FP	5M	12	2019	Year-3	0	0	Winter
12/26/2019	FP	6M	12	2019	Year-3	0	0	Winter
12/26/2019	FP	7M	12	2019	Year-3	1	0	Winter

Date	Species	Site	Month	Year	Survey Year	Presence	Data Gap	Season
12/26/2019	FP	10M	12	2019	Year-3	1	0	Winter
12/26/2019	FP	12M	12	2019	Year-3	1	0	Winter
12/26/2019	FP	14M	12	2019	Year-3	1	0	Winter
12/26/2019	FP	8A	12	2019	Year-3		1	Winter
12/26/2019	FP	9A	12	2019	Year-3		1	Winter
12/26/2019	FP	11A	12	2019	Year-3	1	0	Winter
12/26/2019	FP	13A	12	2019	Year-3		1	Winter
12/27/2019	FP	1M	12	2019	Year-3	1	0	Winter
12/27/2019	FP	2M	12	2019	Year-3	1	0	Winter
12/27/2019	FP	3M	12	2019	Year-3		1	Winter
12/27/2019	FP	4M	12	2019	Year-3	1	0	Winter
12/27/2019	FP	5M	12	2019	Year-3	1	0	Winter
12/27/2019	FP	6M	12	2019	Year-3	1	0	Winter
12/27/2019	FP	7M	12	2019	Year-3	1	0	Winter
12/27/2019	FP	10M	12	2019	Year-3	1	0	Winter
12/27/2019	FP	12M	12	2019	Year-3	1	0	Winter
12/27/2019	FP	14M	12	2019	Year-3	0	0	Winter
12/27/2019	FP	8A	12	2019	Year-3		1	Winter
12/27/2019	FP	9A	12	2019	Year-3		1	Winter
12/27/2019	FP	11A	12	2019	Year-3	1	0	Winter
12/27/2019	FP	13A	12	2019	Year-3		1	Winter
12/28/2019	FP	1M	12	2019	Year-3	1	0	Winter
12/28/2019	FP	2M	12	2019	Year-3	1	0	Winter
12/28/2019	FP	3M	12	2019	Year-3		1	Winter
12/28/2019	FP	4M	12	2019	Year-3	1	0	Winter
12/28/2019	FP	5M	12	2019	Year-3	1	0	Winter
12/28/2019	FP	6M	12	2019	Year-3	1	0	Winter
12/28/2019	FP	7M	12	2019	Year-3	0	0	Winter
12/28/2019	FP	10M	12	2019	Year-3	1	0	Winter
12/28/2019	FP	12M	12	2019	Year-3	0	0	Winter
12/28/2019	FP	14M	12	2019	Year-3	1	0	Winter
12/28/2019	FP	8A	12	2019	Year-3		1	Winter
12/28/2019	FP	9A	12	2019	Year-3		1	Winter
12/28/2019	FP	11A	12	2019	Year-3	0	0	Winter
12/28/2019	FP	13A	12	2019	Year-3		1	Winter
12/29/2019	FP	1M	12	2019	Year-3	1	0	Winter
12/29/2019	FP	2M	12	2019	Year-3	1	0	Winter
12/29/2019	FP	3M	12	2019	Year-3		1	Winter
12/29/2019	FP	4M	12	2019	Year-3	1	0	Winter
12/29/2019	FP	5M	12	2019	Year-3	0	0	Winter
12/29/2019	FP	6M	12	2019	Year-3	1	0	Winter
12/29/2019	FP	7M	12	2019	Year-3	1	0	Winter
12/29/2019	FP	10M	12	2019	Year-3	1	0	Winter
12/29/2019	FP	12M	12	2019	Year-3	1	0	Winter

Date	Species	Site	Month	Year	Survey Year	Presence	Data Gap	Season
12/29/2019	FP	14M	12	2019	Year-3	1	0	Winter
12/29/2019	FP	8A	12	2019	Year-3		1	Winter
12/29/2019	FP	9A	12	2019	Year-3		1	Winter
12/29/2019	FP	11A	12	2019	Year-3	1	0	Winter
12/29/2019	FP	13A	12	2019	Year-3		1	Winter
12/30/2019	FP	1M	12	2019	Year-3	1	0	Winter
12/30/2019	FP	2M	12	2019	Year-3	1	0	Winter
12/30/2019	FP	3M	12	2019	Year-3		1	Winter
12/30/2019	FP	4M	12	2019	Year-3	1	0	Winter
12/30/2019	FP	5M	12	2019	Year-3	1	0	Winter
12/30/2019	FP	6M	12	2019	Year-3	1	0	Winter
12/30/2019	FP	7M	12	2019	Year-3	1	0	Winter
12/30/2019	FP	10M	12	2019	Year-3	1	0	Winter
12/30/2019	FP	12M	12	2019	Year-3	1	0	Winter
12/30/2019	FP	14M	12	2019	Year-3	0	0	Winter
12/30/2019	FP	8A	12	2019	Year-3		1	Winter
12/30/2019	FP	9A	12	2019	Year-3		1	Winter
12/30/2019	FP	11A	12	2019	Year-3	1	0	Winter
12/30/2019	FP	13A	12	2019	Year-3		1	Winter
12/31/2019	FP	1M	12	2019	Year-3	1	0	Winter
12/31/2019	FP	2M	12	2019	Year-3	1	0	Winter
12/31/2019	FP	3M	12	2019	Year-3		1	Winter
12/31/2019	FP	4M	12	2019	Year-3	1	0	Winter
12/31/2019	FP	5M	12	2019	Year-3	1	0	Winter
12/31/2019	FP	6M	12	2019	Year-3	1	0	Winter
12/31/2019	FP	7M	12	2019	Year-3	1	0	Winter
12/31/2019	FP	10M	12	2019	Year-3	1	0	Winter
12/31/2019	FP	12M	12	2019	Year-3	1	0	Winter
12/31/2019	FP	14M	12	2019	Year-3	0	0	Winter
12/31/2019	FP	8A	12	2019	Year-3		1	Winter
12/31/2019	FP	9A	12	2019	Year-3		1	Winter
12/31/2019	FP	11A	12	2019	Year-3	1	0	Winter
12/31/2019	FP	13A	12	2019	Year-3		1	Winter
1/1/2020	FP	1M	1	2020	Year-3	1	0	Winter
1/1/2020	FP	2M	1	2020	Year-3	1	0	Winter
1/1/2020	FP	3M	1	2020	Year-3		1	Winter
1/1/2020	FP	4M	1	2020	Year-3	1	0	Winter
1/1/2020	FP	5M	1	2020	Year-3	1	0	Winter
1/1/2020	FP	6M	1	2020	Year-3	1	0	Winter
1/1/2020	FP	7M	1	2020	Year-3	1	0	Winter
1/1/2020	FP	10M	1	2020	Year-3	1	0	Winter
1/1/2020	FP	12M	1	2020	Year-3	1	0	Winter
1/1/2020	FP	14M	1	2020	Year-3	1	0	Winter
1/1/2020	FP	8A	1	2020	Year-3		1	Winter

Date	Species	Site	Month	Year	Survey Year	Presence	Data Gap	Season
1/1/2020	FP	9A	1	2020	Year-3		1	Winter
1/1/2020	FP	11A	1	2020	Year-3	0	0	Winter
1/1/2020	FP	13A	1	2020	Year-3		1	Winter
1/2/2020	FP	1M	1	2020	Year-3	1	0	Winter
1/2/2020	FP	2M	1	2020	Year-3	1	0	Winter
1/2/2020	FP	3M	1	2020	Year-3		1	Winter
1/2/2020	FP	4M	1	2020	Year-3	1	0	Winter
1/2/2020	FP	5M	1	2020	Year-3	1	0	Winter
1/2/2020	FP	6M	1	2020	Year-3	1	0	Winter
1/2/2020	FP	7M	1	2020	Year-3	1	0	Winter
1/2/2020	FP	10M	1	2020	Year-3	1	0	Winter
1/2/2020	FP	12M	1	2020	Year-3	1	0	Winter
1/2/2020	FP	14M	1	2020	Year-3	1	0	Winter
1/2/2020	FP	8A	1	2020	Year-3		1	Winter
1/2/2020	FP	9A	1	2020	Year-3		1	Winter
1/2/2020	FP	11A	1	2020	Year-3	1	0	Winter
1/2/2020	FP	13A	1	2020	Year-3		1	Winter
1/3/2020	FP	1M	1	2020	Year-3	1	0	Winter
1/3/2020	FP	2M	1	2020	Year-3	1	0	Winter
1/3/2020	FP	3M	1	2020	Year-3		1	Winter
1/3/2020	FP	4M	1	2020	Year-3	1	0	Winter
1/3/2020	FP	5M	1	2020	Year-3	1	0	Winter
1/3/2020	FP	6M	1	2020	Year-3	1	0	Winter
1/3/2020	FP	7M	1	2020	Year-3	0	0	Winter
1/3/2020	FP	10M	1	2020	Year-3	1	0	Winter
1/3/2020	FP	12M	1	2020	Year-3	1	0	Winter
1/3/2020	FP	14M	1	2020	Year-3	1	0	Winter
1/3/2020	FP	8A	1	2020	Year-3		1	Winter
1/3/2020	FP	9A	1	2020	Year-3		1	Winter
1/3/2020	FP	11A	1	2020	Year-3	1	0	Winter
1/3/2020	FP	13A	1	2020	Year-3		1	Winter
1/4/2020	FP	1M	1	2020	Year-3	1	0	Winter
1/4/2020	FP	2M	1	2020	Year-3	1	0	Winter
1/4/2020	FP	3M	1	2020	Year-3		1	Winter
1/4/2020	FP	4M	1	2020	Year-3	1	0	Winter
1/4/2020	FP	5M	1	2020	Year-3	1	0	Winter
1/4/2020	FP	6M	1	2020	Year-3	1	0	Winter
1/4/2020	FP	7M	1	2020	Year-3	1	0	Winter
1/4/2020	FP	10M	1	2020	Year-3	1	0	Winter
1/4/2020	FP	12M	1	2020	Year-3	1	0	Winter
1/4/2020	FP	14M	1	2020	Year-3	1	0	Winter
1/4/2020	FP	8A	1	2020	Year-3		1	Winter
1/4/2020	FP	9A	1	2020	Year-3		1	Winter
1/4/2020	FP	11A	1	2020	Year-3	1	0	Winter

Date	Species	Site	Month	Year	Survey Year	Presence	Data Gap	Season
1/4/2020	FP	13A	1	2020	Year-3		1	Winter
1/5/2020	FP	1M	1	2020	Year-3	1	0	Winter
1/5/2020	FP	2M	1	2020	Year-3	1	0	Winter
1/5/2020	FP	3M	1	2020	Year-3		1	Winter
1/5/2020	FP	4M	1	2020	Year-3	1	0	Winter
1/5/2020	FP	5M	1	2020	Year-3	1	0	Winter
1/5/2020	FP	6M	1	2020	Year-3	1	0	Winter
1/5/2020	FP	7M	1	2020	Year-3	1	0	Winter
1/5/2020	FP	10M	1	2020	Year-3	1	0	Winter
1/5/2020	FP	12M	1	2020	Year-3	1	0	Winter
1/5/2020	FP	14M	1	2020	Year-3	1	0	Winter
1/5/2020	FP	8A	1	2020	Year-3		1	Winter
1/5/2020	FP	9A	1	2020	Year-3		1	Winter
1/5/2020	FP	11A	1	2020	Year-3	1	0	Winter
1/5/2020	FP	13A	1	2020	Year-3		1	Winter
1/6/2020	FP	1M	1	2020	Year-3	1	0	Winter
1/6/2020	FP	2M	1	2020	Year-3	1	0	Winter
1/6/2020	FP	3M	1	2020	Year-3		1	Winter
1/6/2020	FP	4M	1	2020	Year-3	1	0	Winter
1/6/2020	FP	5M	1	2020	Year-3	1	0	Winter
1/6/2020	FP	6M	1	2020	Year-3	1	0	Winter
1/6/2020	FP	7M	1	2020	Year-3	1	0	Winter
1/6/2020	FP	10M	1	2020	Year-3	1	0	Winter
1/6/2020	FP	12M	1	2020	Year-3	1	0	Winter
1/6/2020	FP	14M	1	2020	Year-3	1	0	Winter
1/6/2020	FP	8A	1	2020	Year-3		1	Winter
1/6/2020	FP	9A	1	2020	Year-3		1	Winter
1/6/2020	FP	11A	1	2020	Year-3	1	0	Winter
1/6/2020	FP	13A	1	2020	Year-3		1	Winter
1/7/2020	FP	1M	1	2020	Year-3	1	0	Winter
1/7/2020	FP	2M	1	2020	Year-3	1	0	Winter
1/7/2020	FP	3M	1	2020	Year-3		1	Winter
1/7/2020	FP	4M	1	2020	Year-3	1	0	Winter
1/7/2020	FP	5M	1	2020	Year-3	1	0	Winter
1/7/2020	FP	6M	1	2020	Year-3	1	0	Winter
1/7/2020	FP	7M	1	2020	Year-3	1	0	Winter
1/7/2020	FP	10M	1	2020	Year-3	1	0	Winter
1/7/2020	FP	12M	1	2020	Year-3	1	0	Winter
1/7/2020	FP	14M	1	2020	Year-3	1	0	Winter
1/7/2020	FP	8A	1	2020	Year-3		1	Winter
1/7/2020	FP	9A	1	2020	Year-3		1	Winter
1/7/2020	FP	11A	1	2020	Year-3	1	0	Winter
1/7/2020	FP	13A	1	2020	Year-3		1	Winter
1/8/2020	FP	1M	1	2020	Year-3	1	0	Winter

Date	Species	Site	Month	Year	Survey Year	Presence	Data Gap	Season
1/8/2020	FP	2M	1	2020	Year-3	1	0	Winter
1/8/2020	FP	3M	1	2020	Year-3		1	Winter
1/8/2020	FP	4M	1	2020	Year-3	1	0	Winter
1/8/2020	FP	5M	1	2020	Year-3	1	0	Winter
1/8/2020	FP	6M	1	2020	Year-3	1	0	Winter
1/8/2020	FP	7M	1	2020	Year-3	0	0	Winter
1/8/2020	FP	10M	1	2020	Year-3	1	0	Winter
1/8/2020	FP	12M	1	2020	Year-3	1	0	Winter
1/8/2020	FP	14M	1	2020	Year-3	1	0	Winter
1/8/2020	FP	8A	1	2020	Year-3		1	Winter
1/8/2020	FP	9A	1	2020	Year-3		1	Winter
1/8/2020	FP	11A	1	2020	Year-3	1	0	Winter
1/8/2020	FP	13A	1	2020	Year-3		1	Winter
1/9/2020	FP	1M	1	2020	Year-3	1	0	Winter
1/9/2020	FP	2M	1	2020	Year-3	1	0	Winter
1/9/2020	FP	3M	1	2020	Year-3		1	Winter
1/9/2020	FP	4M	1	2020	Year-3	1	0	Winter
1/9/2020	FP	5M	1	2020	Year-3	1	0	Winter
1/9/2020	FP	6M	1	2020	Year-3	1	0	Winter
1/9/2020	FP	7M	1	2020	Year-3	0	0	Winter
1/9/2020	FP	10M	1	2020	Year-3	1	0	Winter
1/9/2020	FP	12M	1	2020	Year-3	1	0	Winter
1/9/2020	FP	14M	1	2020	Year-3	1	0	Winter
1/9/2020	FP	8A	1	2020	Year-3		1	Winter
1/9/2020	FP	9A	1	2020	Year-3		1	Winter
1/9/2020	FP	11A	1	2020	Year-3	1	0	Winter
1/9/2020	FP	13A	1	2020	Year-3		1	Winter
1/10/2020	FP	1M	1	2020	Year-3	1	0	Winter
1/10/2020	FP	2M	1	2020	Year-3	1	0	Winter
1/10/2020	FP	3M	1	2020	Year-3		1	Winter
1/10/2020	FP	4M	1	2020	Year-3	1	0	Winter
1/10/2020	FP	5M	1	2020	Year-3	1	0	Winter
1/10/2020	FP	6M	1	2020	Year-3	1	0	Winter
1/10/2020	FP	7M	1	2020	Year-3	1	0	Winter
1/10/2020	FP	10M	1	2020	Year-3	1	0	Winter
1/10/2020	FP	12M	1	2020	Year-3	1	0	Winter
1/10/2020	FP	14M	1	2020	Year-3	1	0	Winter
1/10/2020	FP	8A	1	2020	Year-3		1	Winter
1/10/2020	FP	9A	1	2020	Year-3		1	Winter
1/10/2020	FP	11A	1	2020	Year-3	1	0	Winter
1/10/2020	FP	13A	1	2020	Year-3		1	Winter
1/11/2020	FP	1M	1	2020	Year-3	1	0	Winter
1/11/2020	FP	2M	1	2020	Year-3	1	0	Winter
1/11/2020	FP	3M	1	2020	Year-3		1	Winter

Date	Species	Site	Month	Year	Survey Year	Presence	Data Gap	Season
1/11/2020	FP	4M	1	2020	Year-3	1	0	Winter
1/11/2020	FP	5M	1	2020	Year-3	1	0	Winter
1/11/2020	FP	6M	1	2020	Year-3	1	0	Winter
1/11/2020	FP	7M	1	2020	Year-3	1	0	Winter
1/11/2020	FP	10M	1	2020	Year-3	1	0	Winter
1/11/2020	FP	12M	1	2020	Year-3	1	0	Winter
1/11/2020	FP	14M	1	2020	Year-3	1	0	Winter
1/11/2020	FP	8A	1	2020	Year-3		1	Winter
1/11/2020	FP	9A	1	2020	Year-3		1	Winter
1/11/2020	FP	11A	1	2020	Year-3		1	Winter
1/11/2020	FP	13A	1	2020	Year-3		1	Winter
1/12/2020	FP	1M	1	2020	Year-3	1	0	Winter
1/12/2020	FP	2M	1	2020	Year-3	1	0	Winter
1/12/2020	FP	3M	1	2020	Year-3		1	Winter
1/12/2020	FP	4M	1	2020	Year-3	1	0	Winter
1/12/2020	FP	5M	1	2020	Year-3	1	0	Winter
1/12/2020	FP	6M	1	2020	Year-3	1	0	Winter
1/12/2020	FP	7M	1	2020	Year-3	1	0	Winter
1/12/2020	FP	10M	1	2020	Year-3	1	0	Winter
1/12/2020	FP	12M	1	2020	Year-3	1	0	Winter
1/12/2020	FP	14M	1	2020	Year-3	1	0	Winter
1/12/2020	FP	8A	1	2020	Year-3		1	Winter
1/12/2020	FP	9A	1	2020	Year-3		1	Winter
1/12/2020	FP	11A	1	2020	Year-3		1	Winter
1/12/2020	FP	13A	1	2020	Year-3		1	Winter
1/13/2020	FP	1M	1	2020	Year-3	0	0	Winter
1/13/2020	FP	2M	1	2020	Year-3	1	0	Winter
1/13/2020	FP	3M	1	2020	Year-3		1	Winter
1/13/2020	FP	4M	1	2020	Year-3	1	0	Winter
1/13/2020	FP	5M	1	2020	Year-3	1	0	Winter
1/13/2020	FP	6M	1	2020	Year-3	1	0	Winter
1/13/2020	FP	7M	1	2020	Year-3	1	0	Winter
1/13/2020	FP	10M	1	2020	Year-3	1	0	Winter
1/13/2020	FP	12M	1	2020	Year-3	1	0	Winter
1/13/2020	FP	14M	1	2020	Year-3	1	0	Winter
1/13/2020	FP	8A	1	2020	Year-3		1	Winter
1/13/2020	FP	9A	1	2020	Year-3		1	Winter
1/13/2020	FP	11A	1	2020	Year-3		1	Winter
1/13/2020	FP	13A	1	2020	Year-3		1	Winter
1/14/2020	FP	1M	1	2020	Year-3	1	0	Winter
1/14/2020	FP	2M	1	2020	Year-3	1	0	Winter
1/14/2020	FP	3M	1	2020	Year-3		1	Winter
1/14/2020	FP	4M	1	2020	Year-3	0	0	Winter
1/14/2020	FP	5M	1	2020	Year-3	1	0	Winter

Date	Species	Site	Month	Year	Survey Year	Presence	Data Gap	Season
1/14/2020	FP	6M	1	2020	Year-3	0	0	Winter
1/14/2020	FP	7M	1	2020	Year-3	0	0	Winter
1/14/2020	FP	10M	1	2020	Year-3	1	0	Winter
1/14/2020	FP	12M	1	2020	Year-3	1	0	Winter
1/14/2020	FP	14M	1	2020	Year-3	1	0	Winter
1/14/2020	FP	8A	1	2020	Year-3		1	Winter
1/14/2020	FP	9A	1	2020	Year-3		1	Winter
1/14/2020	FP	11A	1	2020	Year-3		1	Winter
1/14/2020	FP	13A	1	2020	Year-3		1	Winter
1/15/2020	FP	1M	1	2020	Year-3	1	0	Winter
1/15/2020	FP	2M	1	2020	Year-3	1	0	Winter
1/15/2020	FP	3M	1	2020	Year-3		1	Winter
1/15/2020	FP	4M	1	2020	Year-3	1	0	Winter
1/15/2020	FP	5M	1	2020	Year-3	1	0	Winter
1/15/2020	FP	6M	1	2020	Year-3	1	0	Winter
1/15/2020	FP	7M	1	2020	Year-3	1	0	Winter
1/15/2020	FP	10M	1	2020	Year-3	1	0	Winter
1/15/2020	FP	12M	1	2020	Year-3	1	0	Winter
1/15/2020	FP	14M	1	2020	Year-3	1	0	Winter
1/15/2020	FP	8A	1	2020	Year-3		1	Winter
1/15/2020	FP	9A	1	2020	Year-3		1	Winter
1/15/2020	FP	11A	1	2020	Year-3		1	Winter
1/15/2020	FP	13A	1	2020	Year-3		1	Winter
1/16/2020	FP	1M	1	2020	Year-3	1	0	Winter
1/16/2020	FP	2M	1	2020	Year-3	1	0	Winter
1/16/2020	FP	3M	1	2020	Year-3		1	Winter
1/16/2020	FP	4M	1	2020	Year-3	1	0	Winter
1/16/2020	FP	5M	1	2020	Year-3	1	0	Winter
1/16/2020	FP	6M	1	2020	Year-3	0	0	Winter
1/16/2020	FP	7M	1	2020	Year-3	1	0	Winter
1/16/2020	FP	10M	1	2020	Year-3	1	0	Winter
1/16/2020	FP	12M	1	2020	Year-3	1	0	Winter
1/16/2020	FP	14M	1	2020	Year-3	1	0	Winter
1/16/2020	FP	8A	1	2020	Year-3		1	Winter
1/16/2020	FP	9A	1	2020	Year-3		1	Winter
1/16/2020	FP	11A	1	2020	Year-3		1	Winter
1/16/2020	FP	13A	1	2020	Year-3		1	Winter
1/17/2020	FP	1M	1	2020	Year-3	1	0	Winter
1/17/2020	FP	2M	1	2020	Year-3	1	0	Winter
1/17/2020	FP	3M	1	2020	Year-3		1	Winter
1/17/2020	FP	4M	1	2020	Year-3	1	0	Winter
1/17/2020	FP	5M	1	2020	Year-3	1	0	Winter
1/17/2020	FP	6M	1	2020	Year-3	1	0	Winter
1/17/2020	FP	7M	1	2020	Year-3	1	0	Winter

Date	Species	Site	Month	Year	Survey Year	Presence	Data Gap	Season
1/17/2020	FP	10M	1	2020	Year-3	1	0	Winter
1/17/2020	FP	12M	1	2020	Year-3	1	0	Winter
1/17/2020	FP	14M	1	2020	Year-3	1	0	Winter
1/17/2020	FP	8A	1	2020	Year-3		1	Winter
1/17/2020	FP	9A	1	2020	Year-3		1	Winter
1/17/2020	FP	11A	1	2020	Year-3		1	Winter
1/17/2020	FP	13A	1	2020	Year-3		1	Winter
1/18/2020	FP	1M	1	2020	Year-3	1	0	Winter
1/18/2020	FP	2M	1	2020	Year-3	1	0	Winter
1/18/2020	FP	3M	1	2020	Year-3		1	Winter
1/18/2020	FP	4M	1	2020	Year-3	1	0	Winter
1/18/2020	FP	5M	1	2020	Year-3	1	0	Winter
1/18/2020	FP	6M	1	2020	Year-3	1	0	Winter
1/18/2020	FP	7M	1	2020	Year-3	1	0	Winter
1/18/2020	FP	10M	1	2020	Year-3	1	0	Winter
1/18/2020	FP	12M	1	2020	Year-3	1	0	Winter
1/18/2020	FP	14M	1	2020	Year-3	1	0	Winter
1/18/2020	FP	8A	1	2020	Year-3		1	Winter
1/18/2020	FP	9A	1	2020	Year-3		1	Winter
1/18/2020	FP	11A	1	2020	Year-3		1	Winter
1/18/2020	FP	13A	1	2020	Year-3		1	Winter
1/19/2020	FP	1M	1	2020	Year-3	1	0	Winter
1/19/2020	FP	2M	1	2020	Year-3	1	0	Winter
1/19/2020	FP	3M	1	2020	Year-3		1	Winter
1/19/2020	FP	4M	1	2020	Year-3	1	0	Winter
1/19/2020	FP	5M	1	2020	Year-3	1	0	Winter
1/19/2020	FP	6M	1	2020	Year-3	1	0	Winter
1/19/2020	FP	7M	1	2020	Year-3	1	0	Winter
1/19/2020	FP	10M	1	2020	Year-3	1	0	Winter
1/19/2020	FP	12M	1	2020	Year-3	1	0	Winter
1/19/2020	FP	14M	1	2020	Year-3	1	0	Winter
1/19/2020	FP	8A	1	2020	Year-3		1	Winter
1/19/2020	FP	9A	1	2020	Year-3		1	Winter
1/19/2020	FP	11A	1	2020	Year-3		1	Winter
1/19/2020	FP	13A	1	2020	Year-3		1	Winter
1/20/2020	FP	1M	1	2020	Year-3	0	0	Winter
1/20/2020	FP	2M	1	2020	Year-3	1	0	Winter
1/20/2020	FP	3M	1	2020	Year-3		1	Winter
1/20/2020	FP	4M	1	2020	Year-3	0	0	Winter
1/20/2020	FP	5M	1	2020	Year-3	1	0	Winter
1/20/2020	FP	6M	1	2020	Year-3	0	0	Winter
1/20/2020	FP	7M	1	2020	Year-3	0	0	Winter
1/20/2020	FP	10M	1	2020	Year-3	1	0	Winter
1/20/2020	FP	12M	1	2020	Year-3	0	0	Winter

Date	Species	Site	Month	Year	Survey Year	Presence	Data Gap	Season
1/20/2020	FP	14M	1	2020	Year-3	0	0	Winter
1/20/2020	FP	8A	1	2020	Year-3		1	Winter
1/20/2020	FP	9A	1	2020	Year-3		1	Winter
1/20/2020	FP	11A	1	2020	Year-3		1	Winter
1/20/2020	FP	13A	1	2020	Year-3		1	Winter
1/21/2020	FP	1M	1	2020	Year-3	0	0	Winter
1/21/2020	FP	2M	1	2020	Year-3	0	0	Winter
1/21/2020	FP	3M	1	2020	Year-3		1	Winter
1/21/2020	FP	4M	1	2020	Year-3	0	0	Winter
1/21/2020	FP	5M	1	2020	Year-3	0	0	Winter
1/21/2020	FP	6M	1	2020	Year-3	0	0	Winter
1/21/2020	FP	7M	1	2020	Year-3	0	0	Winter
1/21/2020	FP	10M	1	2020	Year-3		1	Winter
1/21/2020	FP	12M	1	2020	Year-3	0	0	Winter
1/21/2020	FP	14M	1	2020	Year-3	0	0	Winter
1/21/2020	FP	8A	1	2020	Year-3		1	Winter
1/21/2020	FP	9A	1	2020	Year-3		1	Winter
1/21/2020	FP	11A	1	2020	Year-3		1	Winter
1/21/2020	FP	13A	1	2020	Year-3		1	Winter
1/22/2020	FP	1M	1	2020	Year-3	0	0	Winter
1/22/2020	FP	2M	1	2020	Year-3	0	0	Winter
1/22/2020	FP	3M	1	2020	Year-3		1	Winter
1/22/2020	FP	4M	1	2020	Year-3		1	Winter
1/22/2020	FP	5M	1	2020	Year-3		1	Winter
1/22/2020	FP	6M	1	2020	Year-3		1	Winter
1/22/2020	FP	7M	1	2020	Year-3		1	Winter
1/22/2020	FP	10M	1	2020	Year-3	0	0	Winter
1/22/2020	FP	12M	1	2020	Year-3		1	Winter
1/22/2020	FP	14M	1	2020	Year-3		1	Winter
1/22/2020	FP	8A	1	2020	Year-3		1	Winter
1/22/2020	FP	9A	1	2020	Year-3		1	Winter
1/22/2020	FP	11A	1	2020	Year-3		1	Winter
1/22/2020	FP	13A	1	2020	Year-3		1	Winter
1/23/2020	FP	1M	1	2020	Year-3		1	Winter
1/23/2020	FP	2M	1	2020	Year-3		1	Winter
1/23/2020	FP	3M	1	2020	Year-3	0	0	Winter
1/23/2020	FP	4M	1	2020	Year-3	0	0	Winter
1/23/2020	FP	5M	1	2020	Year-3	0	0	Winter
1/23/2020	FP	6M	1	2020	Year-3	0	0	Winter
1/23/2020	FP	7M	1	2020	Year-3		1	Winter
1/23/2020	FP	10M	1	2020	Year-3	0	0	Winter
1/23/2020	FP	12M	1	2020	Year-3	0	0	Winter
1/23/2020	FP	14M	1	2020	Year-3	0	0	Winter
1/23/2020	FP	8A	1	2020	Year-3		1	Winter

Date	Species	Site	Month	Year	Survey Year	Presence	Data Gap	Season
1/23/2020	FP	9A	1	2020	Year-3		1	Winter
1/23/2020	FP	11A	1	2020	Year-3		1	Winter
1/23/2020	FP	13A	1	2020	Year-3		1	Winter
1/24/2020	FP	1M	1	2020	Year-3	1	0	Winter
1/24/2020	FP	2M	1	2020	Year-3	1	0	Winter
1/24/2020	FP	3M	1	2020	Year-3	1	0	Winter
1/24/2020	FP	4M	1	2020	Year-3	1	0	Winter
1/24/2020	FP	5M	1	2020	Year-3	1	0	Winter
1/24/2020	FP	6M	1	2020	Year-3	1	0	Winter
1/24/2020	FP	7M	1	2020	Year-3		1	Winter
1/24/2020	FP	10M	1	2020	Year-3	1	0	Winter
1/24/2020	FP	12M	1	2020	Year-3	1	0	Winter
1/24/2020	FP	14M	1	2020	Year-3	1	0	Winter
1/24/2020	FP	8A	1	2020	Year-3		1	Winter
1/24/2020	FP	9A	1	2020	Year-3		1	Winter
1/24/2020	FP	11A	1	2020	Year-3		1	Winter
1/24/2020	FP	13A	1	2020	Year-3		1	Winter
1/25/2020	FP	1M	1	2020	Year-3	1	0	Winter
1/25/2020	FP	2M	1	2020	Year-3	1	0	Winter
1/25/2020	FP	3M	1	2020	Year-3	1	0	Winter
1/25/2020	FP	4M	1	2020	Year-3	1	0	Winter
1/25/2020	FP	5M	1	2020	Year-3	1	0	Winter
1/25/2020	FP	6M	1	2020	Year-3	1	0	Winter
1/25/2020	FP	7M	1	2020	Year-3		1	Winter
1/25/2020	FP	10M	1	2020	Year-3	1	0	Winter
1/25/2020	FP	12M	1	2020	Year-3	1	0	Winter
1/25/2020	FP	14M	1	2020	Year-3	1	0	Winter
1/25/2020	FP	8A	1	2020	Year-3		1	Winter
1/25/2020	FP	9A	1	2020	Year-3		1	Winter
1/25/2020	FP	11A	1	2020	Year-3		1	Winter
1/25/2020	FP	13A	1	2020	Year-3		1	Winter
1/26/2020	FP	1M	1	2020	Year-3	1	0	Winter
1/26/2020	FP	2M	1	2020	Year-3	1	0	Winter
1/26/2020	FP	3M	1	2020	Year-3	1	0	Winter
1/26/2020	FP	4M	1	2020	Year-3	1	0	Winter
1/26/2020	FP	5M	1	2020	Year-3	1	0	Winter
1/26/2020	FP	6M	1	2020	Year-3	1	0	Winter
1/26/2020	FP	7M	1	2020	Year-3		1	Winter
1/26/2020	FP	10M	1	2020	Year-3	1	0	Winter
1/26/2020	FP	12M	1	2020	Year-3	1	0	Winter
1/26/2020	FP	14M	1	2020	Year-3	1	0	Winter
1/26/2020	FP	8A	1	2020	Year-3		1	Winter
1/26/2020	FP	9A	1	2020	Year-3		1	Winter
1/26/2020	FP	11A	1	2020	Year-3		1	Winter

Date	Species	Site	Month	Year	Survey Year	Presence	Data Gap	Season
1/26/2020	FP	13A	1	2020	Year-3		1	Winter
1/27/2020	FP	1M	1	2020	Year-3	1	0	Winter
1/27/2020	FP	2M	1	2020	Year-3	1	0	Winter
1/27/2020	FP	3M	1	2020	Year-3	1	0	Winter
1/27/2020	FP	4M	1	2020	Year-3	1	0	Winter
1/27/2020	FP	5M	1	2020	Year-3	1	0	Winter
1/27/2020	FP	6M	1	2020	Year-3	1	0	Winter
1/27/2020	FP	7M	1	2020	Year-3		1	Winter
1/27/2020	FP	10M	1	2020	Year-3	1	0	Winter
1/27/2020	FP	12M	1	2020	Year-3	1	0	Winter
1/27/2020	FP	14M	1	2020	Year-3	1	0	Winter
1/27/2020	FP	8A	1	2020	Year-3		1	Winter
1/27/2020	FP	9A	1	2020	Year-3		1	Winter
1/27/2020	FP	11A	1	2020	Year-3		1	Winter
1/27/2020	FP	13A	1	2020	Year-3		1	Winter
1/28/2020	FP	1M	1	2020	Year-3	1	0	Winter
1/28/2020	FP	2M	1	2020	Year-3	1	0	Winter
1/28/2020	FP	3M	1	2020	Year-3	1	0	Winter
1/28/2020	FP	4M	1	2020	Year-3	1	0	Winter
1/28/2020	FP	5M	1	2020	Year-3	1	0	Winter
1/28/2020	FP	6M	1	2020	Year-3	1	0	Winter
1/28/2020	FP	7M	1	2020	Year-3		1	Winter
1/28/2020	FP	10M	1	2020	Year-3	1	0	Winter
1/28/2020	FP	12M	1	2020	Year-3	1	0	Winter
1/28/2020	FP	14M	1	2020	Year-3	1	0	Winter
1/28/2020	FP	8A	1	2020	Year-3		1	Winter
1/28/2020	FP	9A	1	2020	Year-3		1	Winter
1/28/2020	FP	11A	1	2020	Year-3		1	Winter
1/28/2020	FP	13A	1	2020	Year-3		1	Winter
1/29/2020	FP	1M	1	2020	Year-3	1	0	Winter
1/29/2020	FP	2M	1	2020	Year-3	1	0	Winter
1/29/2020	FP	3M	1	2020	Year-3	1	0	Winter
1/29/2020	FP	4M	1	2020	Year-3	1	0	Winter
1/29/2020	FP	5M	1	2020	Year-3	1	0	Winter
1/29/2020	FP	6M	1	2020	Year-3	1	0	Winter
1/29/2020	FP	7M	1	2020	Year-3		1	Winter
1/29/2020	FP	10M	1	2020	Year-3	1	0	Winter
1/29/2020	FP	12M	1	2020	Year-3	1	0	Winter
1/29/2020	FP	14M	1	2020	Year-3	1	0	Winter
1/29/2020	FP	8A	1	2020	Year-3		1	Winter
1/29/2020	FP	9A	1	2020	Year-3		1	Winter
1/29/2020	FP	11A	1	2020	Year-3		1	Winter
1/29/2020	FP	13A	1	2020	Year-3		1	Winter
1/30/2020	FP	1M	1	2020	Year-3	1	0	Winter

Date	Species	Site	Month	Year	Survey Year	Presence	Data Gap	Season
1/30/2020	FP	2M	1	2020	Year-3	1	0	Winter
1/30/2020	FP	3M	1	2020	Year-3	1	0	Winter
1/30/2020	FP	4M	1	2020	Year-3	1	0	Winter
1/30/2020	FP	5M	1	2020	Year-3	1	0	Winter
1/30/2020	FP	6M	1	2020	Year-3	1	0	Winter
1/30/2020	FP	7M	1	2020	Year-3		1	Winter
1/30/2020	FP	10M	1	2020	Year-3	1	0	Winter
1/30/2020	FP	12M	1	2020	Year-3	1	0	Winter
1/30/2020	FP	14M	1	2020	Year-3	1	0	Winter
1/30/2020	FP	8A	1	2020	Year-3		1	Winter
1/30/2020	FP	9A	1	2020	Year-3		1	Winter
1/30/2020	FP	11A	1	2020	Year-3		1	Winter
1/30/2020	FP	13A	1	2020	Year-3		1	Winter
1/31/2020	FP	1M	1	2020	Year-3	1	0	Winter
1/31/2020	FP	2M	1	2020	Year-3	1	0	Winter
1/31/2020	FP	3M	1	2020	Year-3	1	0	Winter
1/31/2020	FP	4M	1	2020	Year-3	1	0	Winter
1/31/2020	FP	5M	1	2020	Year-3	1	0	Winter
1/31/2020	FP	6M	1	2020	Year-3	1	0	Winter
1/31/2020	FP	7M	1	2020	Year-3		1	Winter
1/31/2020	FP	10M	1	2020	Year-3	1	0	Winter
1/31/2020	FP	12M	1	2020	Year-3	1	0	Winter
1/31/2020	FP	14M	1	2020	Year-3	1	0	Winter
1/31/2020	FP	8A	1	2020	Year-3		1	Winter
1/31/2020	FP	9A	1	2020	Year-3		1	Winter
1/31/2020	FP	11A	1	2020	Year-3		1	Winter
1/31/2020	FP	13A	1	2020	Year-3		1	Winter
2/1/2020	FP	1M	2	2020	Year-3	1	0	Winter
2/1/2020	FP	2M	2	2020	Year-3	1	0	Winter
2/1/2020	FP	3M	2	2020	Year-3	1	0	Winter
2/1/2020	FP	4M	2	2020	Year-3	1	0	Winter
2/1/2020	FP	5M	2	2020	Year-3	1	0	Winter
2/1/2020	FP	6M	2	2020	Year-3	1	0	Winter
2/1/2020	FP	7M	2	2020	Year-3		1	Winter
2/1/2020	FP	10M	2	2020	Year-3	1	0	Winter
2/1/2020	FP	12M	2	2020	Year-3	1	0	Winter
2/1/2020	FP	14M	2	2020	Year-3	1	0	Winter
2/1/2020	FP	8A	2	2020	Year-3		1	Winter
2/1/2020	FP	9A	2	2020	Year-3		1	Winter
2/1/2020	FP	11A	2	2020	Year-3		1	Winter
2/1/2020	FP	13A	2	2020	Year-3		1	Winter
2/2/2020	FP	1M	2	2020	Year-3	1	0	Winter
2/2/2020	FP	2M	2	2020	Year-3	1	0	Winter
2/2/2020	FP	3M	2	2020	Year-3	1	0	Winter

Date	Species	Site	Month	Year	Survey Year	Presence	Data Gap	Season
2/2/2020	FP	4M	2	2020	Year-3	1	0	Winter
2/2/2020	FP	5M	2	2020	Year-3	1	0	Winter
2/2/2020	FP	6M	2	2020	Year-3	1	0	Winter
2/2/2020	FP	7M	2	2020	Year-3		1	Winter
2/2/2020	FP	10M	2	2020	Year-3	1	0	Winter
2/2/2020	FP	12M	2	2020	Year-3	1	0	Winter
2/2/2020	FP	14M	2	2020	Year-3	1	0	Winter
2/2/2020	FP	8A	2	2020	Year-3		1	Winter
2/2/2020	FP	9A	2	2020	Year-3		1	Winter
2/2/2020	FP	11A	2	2020	Year-3		1	Winter
2/2/2020	FP	13A	2	2020	Year-3		1	Winter
2/3/2020	FP	1M	2	2020	Year-3	1	0	Winter
2/3/2020	FP	2M	2	2020	Year-3	1	0	Winter
2/3/2020	FP	3M	2	2020	Year-3	1	0	Winter
2/3/2020	FP	4M	2	2020	Year-3	1	0	Winter
2/3/2020	FP	5M	2	2020	Year-3	1	0	Winter
2/3/2020	FP	6M	2	2020	Year-3	1	0	Winter
2/3/2020	FP	7M	2	2020	Year-3		1	Winter
2/3/2020	FP	10M	2	2020	Year-3	1	0	Winter
2/3/2020	FP	12M	2	2020	Year-3	1	0	Winter
2/3/2020	FP	14M	2	2020	Year-3	1	0	Winter
2/3/2020	FP	8A	2	2020	Year-3		1	Winter
2/3/2020	FP	9A	2	2020	Year-3		1	Winter
2/3/2020	FP	11A	2	2020	Year-3		1	Winter
2/3/2020	FP	13A	2	2020	Year-3		1	Winter
2/4/2020	FP	1M	2	2020	Year-3	1	0	Winter
2/4/2020	FP	2M	2	2020	Year-3	1	0	Winter
2/4/2020	FP	3M	2	2020	Year-3	1	0	Winter
2/4/2020	FP	4M	2	2020	Year-3	1	0	Winter
2/4/2020	FP	5M	2	2020	Year-3	1	0	Winter
2/4/2020	FP	6M	2	2020	Year-3	1	0	Winter
2/4/2020	FP	7M	2	2020	Year-3		1	Winter
2/4/2020	FP	10M	2	2020	Year-3	1	0	Winter
2/4/2020	FP	12M	2	2020	Year-3	1	0	Winter
2/4/2020	FP	14M	2	2020	Year-3	1	0	Winter
2/4/2020	FP	8A	2	2020	Year-3		1	Winter
2/4/2020	FP	9A	2	2020	Year-3		1	Winter
2/4/2020	FP	11A	2	2020	Year-3		1	Winter
2/4/2020	FP	13A	2	2020	Year-3		1	Winter
2/5/2020	FP	1M	2	2020	Year-3	1	0	Winter
2/5/2020	FP	2M	2	2020	Year-3	1	0	Winter
2/5/2020	FP	3M	2	2020	Year-3	1	0	Winter
2/5/2020	FP	4M	2	2020	Year-3	1	0	Winter
2/5/2020	FP	5M	2	2020	Year-3	1	0	Winter

Date	Species	Site	Month	Year	Survey Year	Presence	Data Gap	Season
2/5/2020	FP	6M	2	2020	Year-3	1	0	Winter
2/5/2020	FP	7M	2	2020	Year-3		1	Winter
2/5/2020	FP	10M	2	2020	Year-3	1	0	Winter
2/5/2020	FP	12M	2	2020	Year-3	1	0	Winter
2/5/2020	FP	14M	2	2020	Year-3	1	0	Winter
2/5/2020	FP	8A	2	2020	Year-3		1	Winter
2/5/2020	FP	9A	2	2020	Year-3		1	Winter
2/5/2020	FP	11A	2	2020	Year-3		1	Winter
2/5/2020	FP	13A	2	2020	Year-3		1	Winter
2/6/2020	FP	1M	2	2020	Year-3	1	0	Winter
2/6/2020	FP	2M	2	2020	Year-3	1	0	Winter
2/6/2020	FP	3M	2	2020	Year-3	1	0	Winter
2/6/2020	FP	4M	2	2020	Year-3	1	0	Winter
2/6/2020	FP	5M	2	2020	Year-3	1	0	Winter
2/6/2020	FP	6M	2	2020	Year-3	1	0	Winter
2/6/2020	FP	7M	2	2020	Year-3		1	Winter
2/6/2020	FP	10M	2	2020	Year-3	1	0	Winter
2/6/2020	FP	12M	2	2020	Year-3	1	0	Winter
2/6/2020	FP	14M	2	2020	Year-3	1	0	Winter
2/6/2020	FP	8A	2	2020	Year-3		1	Winter
2/6/2020	FP	9A	2	2020	Year-3		1	Winter
2/6/2020	FP	11A	2	2020	Year-3		1	Winter
2/6/2020	FP	13A	2	2020	Year-3		1	Winter
2/7/2020	FP	1M	2	2020	Year-3	1	0	Winter
2/7/2020	FP	2M	2	2020	Year-3	1	0	Winter
2/7/2020	FP	3M	2	2020	Year-3	1	0	Winter
2/7/2020	FP	4M	2	2020	Year-3	1	0	Winter
2/7/2020	FP	5M	2	2020	Year-3	1	0	Winter
2/7/2020	FP	6M	2	2020	Year-3	1	0	Winter
2/7/2020	FP	7M	2	2020	Year-3		1	Winter
2/7/2020	FP	10M	2	2020	Year-3	1	0	Winter
2/7/2020	FP	12M	2	2020	Year-3	1	0	Winter
2/7/2020	FP	14M	2	2020	Year-3	1	0	Winter
2/7/2020	FP	8A	2	2020	Year-3		1	Winter
2/7/2020	FP	9A	2	2020	Year-3		1	Winter
2/7/2020	FP	11A	2	2020	Year-3		1	Winter
2/7/2020	FP	13A	2	2020	Year-3		1	Winter
2/8/2020	FP	1M	2	2020	Year-3	1	0	Winter
2/8/2020	FP	2M	2	2020	Year-3	1	0	Winter
2/8/2020	FP	3M	2	2020	Year-3	1	0	Winter
2/8/2020	FP	4M	2	2020	Year-3	1	0	Winter
2/8/2020	FP	5M	2	2020	Year-3	1	0	Winter
2/8/2020	FP	6M	2	2020	Year-3	1	0	Winter
2/8/2020	FP	7M	2	2020	Year-3		1	Winter

Date	Species	Site	Month	Year	Survey Year	Presence	Data Gap	Season
2/8/2020	FP	10M	2	2020	Year-3	1	0	Winter
2/8/2020	FP	12M	2	2020	Year-3	1	0	Winter
2/8/2020	FP	14M	2	2020	Year-3	1	0	Winter
2/8/2020	FP	8A	2	2020	Year-3		1	Winter
2/8/2020	FP	9A	2	2020	Year-3		1	Winter
2/8/2020	FP	11A	2	2020	Year-3		1	Winter
2/8/2020	FP	13A	2	2020	Year-3		1	Winter
2/9/2020	FP	1M	2	2020	Year-3	1	0	Winter
2/9/2020	FP	2M	2	2020	Year-3	1	0	Winter
2/9/2020	FP	3M	2	2020	Year-3	1	0	Winter
2/9/2020	FP	4M	2	2020	Year-3	1	0	Winter
2/9/2020	FP	5M	2	2020	Year-3	1	0	Winter
2/9/2020	FP	6M	2	2020	Year-3	1	0	Winter
2/9/2020	FP	7M	2	2020	Year-3		1	Winter
2/9/2020	FP	10M	2	2020	Year-3	1	0	Winter
2/9/2020	FP	12M	2	2020	Year-3	1	0	Winter
2/9/2020	FP	14M	2	2020	Year-3	1	0	Winter
2/9/2020	FP	8A	2	2020	Year-3		1	Winter
2/9/2020	FP	9A	2	2020	Year-3		1	Winter
2/9/2020	FP	11A	2	2020	Year-3		1	Winter
2/9/2020	FP	13A	2	2020	Year-3		1	Winter
2/10/2020	FP	1M	2	2020	Year-3	1	0	Winter
2/10/2020	FP	2M	2	2020	Year-3	1	0	Winter
2/10/2020	FP	3M	2	2020	Year-3	1	0	Winter
2/10/2020	FP	4M	2	2020	Year-3	1	0	Winter
2/10/2020	FP	5M	2	2020	Year-3	1	0	Winter
2/10/2020	FP	6M	2	2020	Year-3	0	0	Winter
2/10/2020	FP	7M	2	2020	Year-3		1	Winter
2/10/2020	FP	10M	2	2020	Year-3	1	0	Winter
2/10/2020	FP	12M	2	2020	Year-3	1	0	Winter
2/10/2020	FP	14M	2	2020	Year-3	1	0	Winter
2/10/2020	FP	8A	2	2020	Year-3		1	Winter
2/10/2020	FP	9A	2	2020	Year-3		1	Winter
2/10/2020	FP	11A	2	2020	Year-3		1	Winter
2/10/2020	FP	13A	2	2020	Year-3		1	Winter
2/11/2020	FP	1M	2	2020	Year-3	1	0	Winter
2/11/2020	FP	2M	2	2020	Year-3	1	0	Winter
2/11/2020	FP	3M	2	2020	Year-3	1	0	Winter
2/11/2020	FP	4M	2	2020	Year-3	1	0	Winter
2/11/2020	FP	5M	2	2020	Year-3	1	0	Winter
2/11/2020	FP	6M	2	2020	Year-3	1	0	Winter
2/11/2020	FP	7M	2	2020	Year-3		1	Winter
2/11/2020	FP	10M	2	2020	Year-3	1	0	Winter
2/11/2020	FP	12M	2	2020	Year-3	1	0	Winter

Date	Species	Site	Month	Year	Survey Year	Presence	Data Gap	Season
2/11/2020	FP	14M	2	2020	Year-3	1	0	Winter
2/11/2020	FP	8A	2	2020	Year-3		1	Winter
2/11/2020	FP	9A	2	2020	Year-3		1	Winter
2/11/2020	FP	11A	2	2020	Year-3		1	Winter
2/11/2020	FP	13A	2	2020	Year-3		1	Winter
2/12/2020	FP	1M	2	2020	Year-3	1	0	Winter
2/12/2020	FP	2M	2	2020	Year-3	1	0	Winter
2/12/2020	FP	3M	2	2020	Year-3	1	0	Winter
2/12/2020	FP	4M	2	2020	Year-3	1	0	Winter
2/12/2020	FP	5M	2	2020	Year-3	1	0	Winter
2/12/2020	FP	6M	2	2020	Year-3	1	0	Winter
2/12/2020	FP	7M	2	2020	Year-3		1	Winter
2/12/2020	FP	10M	2	2020	Year-3	1	0	Winter
2/12/2020	FP	12M	2	2020	Year-3	1	0	Winter
2/12/2020	FP	14M	2	2020	Year-3	1	0	Winter
2/12/2020	FP	8A	2	2020	Year-3		1	Winter
2/12/2020	FP	9A	2	2020	Year-3		1	Winter
2/12/2020	FP	11A	2	2020	Year-3		1	Winter
2/12/2020	FP	13A	2	2020	Year-3		1	Winter
2/13/2020	FP	1M	2	2020	Year-3	1	0	Winter
2/13/2020	FP	2M	2	2020	Year-3	1	0	Winter
2/13/2020	FP	3M	2	2020	Year-3	1	0	Winter
2/13/2020	FP	4M	2	2020	Year-3	1	0	Winter
2/13/2020	FP	5M	2	2020	Year-3	1	0	Winter
2/13/2020	FP	6M	2	2020	Year-3	1	0	Winter
2/13/2020	FP	7M	2	2020	Year-3		1	Winter
2/13/2020	FP	10M	2	2020	Year-3	1	0	Winter
2/13/2020	FP	12M	2	2020	Year-3	1	0	Winter
2/13/2020	FP	14M	2	2020	Year-3	1	0	Winter
2/13/2020	FP	8A	2	2020	Year-3		1	Winter
2/13/2020	FP	9A	2	2020	Year-3		1	Winter
2/13/2020	FP	11A	2	2020	Year-3		1	Winter
2/13/2020	FP	13A	2	2020	Year-3		1	Winter
2/14/2020	FP	1M	2	2020	Year-3	1	0	Winter
2/14/2020	FP	2M	2	2020	Year-3	1	0	Winter
2/14/2020	FP	3M	2	2020	Year-3	1	0	Winter
2/14/2020	FP	4M	2	2020	Year-3	1	0	Winter
2/14/2020	FP	5M	2	2020	Year-3	1	0	Winter
2/14/2020	FP	6M	2	2020	Year-3	1	0	Winter
2/14/2020	FP	7M	2	2020	Year-3		1	Winter
2/14/2020	FP	10M	2	2020	Year-3	1	0	Winter
2/14/2020	FP	12M	2	2020	Year-3	1	0	Winter
2/14/2020	FP	14M	2	2020	Year-3	1	0	Winter
2/14/2020	FP	8A	2	2020	Year-3		1	Winter

Date	Species	Site	Month	Year	Survey Year	Presence	Data Gap	Season
2/14/2020	FP	9A	2	2020	Year-3		1	Winter
2/14/2020	FP	11A	2	2020	Year-3		1	Winter
2/14/2020	FP	13A	2	2020	Year-3		1	Winter
2/15/2020	FP	1M	2	2020	Year-3	1	0	Winter
2/15/2020	FP	2M	2	2020	Year-3	1	0	Winter
2/15/2020	FP	3M	2	2020	Year-3	1	0	Winter
2/15/2020	FP	4M	2	2020	Year-3	1	0	Winter
2/15/2020	FP	5M	2	2020	Year-3	1	0	Winter
2/15/2020	FP	6M	2	2020	Year-3	1	0	Winter
2/15/2020	FP	7M	2	2020	Year-3		1	Winter
2/15/2020	FP	10M	2	2020	Year-3	1	0	Winter
2/15/2020	FP	12M	2	2020	Year-3	1	0	Winter
2/15/2020	FP	14M	2	2020	Year-3	1	0	Winter
2/15/2020	FP	8A	2	2020	Year-3		1	Winter
2/15/2020	FP	9A	2	2020	Year-3		1	Winter
2/15/2020	FP	11A	2	2020	Year-3		1	Winter
2/15/2020	FP	13A	2	2020	Year-3		1	Winter
2/16/2020	FP	1M	2	2020	Year-3	1	0	Winter
2/16/2020	FP	2M	2	2020	Year-3	1	0	Winter
2/16/2020	FP	3M	2	2020	Year-3	1	0	Winter
2/16/2020	FP	4M	2	2020	Year-3	1	0	Winter
2/16/2020	FP	5M	2	2020	Year-3	1	0	Winter
2/16/2020	FP	6M	2	2020	Year-3	1	0	Winter
2/16/2020	FP	7M	2	2020	Year-3		1	Winter
2/16/2020	FP	10M	2	2020	Year-3	1	0	Winter
2/16/2020	FP	12M	2	2020	Year-3	1	0	Winter
2/16/2020	FP	14M	2	2020	Year-3	1	0	Winter
2/16/2020	FP	8A	2	2020	Year-3		1	Winter
2/16/2020	FP	9A	2	2020	Year-3		1	Winter
2/16/2020	FP	11A	2	2020	Year-3		1	Winter
2/16/2020	FP	13A	2	2020	Year-3		1	Winter
2/17/2020	FP	1M	2	2020	Year-3	1	0	Winter
2/17/2020	FP	2M	2	2020	Year-3	1	0	Winter
2/17/2020	FP	3M	2	2020	Year-3	1	0	Winter
2/17/2020	FP	4M	2	2020	Year-3	1	0	Winter
2/17/2020	FP	5M	2	2020	Year-3	1	0	Winter
2/17/2020	FP	6M	2	2020	Year-3	1	0	Winter
2/17/2020	FP	7M	2	2020	Year-3		1	Winter
2/17/2020	FP	10M	2	2020	Year-3	1	0	Winter
2/17/2020	FP	12M	2	2020	Year-3	1	0	Winter
2/17/2020	FP	14M	2	2020	Year-3	1	0	Winter
2/17/2020	FP	8A	2	2020	Year-3		1	Winter
2/17/2020	FP	9A	2	2020	Year-3		1	Winter
2/17/2020	FP	11A	2	2020	Year-3		1	Winter

Date	Species	Site	Month	Year	Survey Year	Presence	Data Gap	Season
2/17/2020	FP	13A	2	2020	Year-3		1	Winter
2/18/2020	FP	1M	2	2020	Year-3	1	0	Winter
2/18/2020	FP	2M	2	2020	Year-3	1	0	Winter
2/18/2020	FP	3M	2	2020	Year-3	1	0	Winter
2/18/2020	FP	4M	2	2020	Year-3	1	0	Winter
2/18/2020	FP	5M	2	2020	Year-3	1	0	Winter
2/18/2020	FP	6M	2	2020	Year-3	1	0	Winter
2/18/2020	FP	7M	2	2020	Year-3		1	Winter
2/18/2020	FP	10M	2	2020	Year-3	1	0	Winter
2/18/2020	FP	12M	2	2020	Year-3	1	0	Winter
2/18/2020	FP	14M	2	2020	Year-3	1	0	Winter
2/18/2020	FP	8A	2	2020	Year-3		1	Winter
2/18/2020	FP	9A	2	2020	Year-3		1	Winter
2/18/2020	FP	11A	2	2020	Year-3		1	Winter
2/18/2020	FP	13A	2	2020	Year-3		1	Winter
2/19/2020	FP	1M	2	2020	Year-3	1	0	Winter
2/19/2020	FP	2M	2	2020	Year-3	1	0	Winter
2/19/2020	FP	3M	2	2020	Year-3	1	0	Winter
2/19/2020	FP	4M	2	2020	Year-3	1	0	Winter
2/19/2020	FP	5M	2	2020	Year-3	1	0	Winter
2/19/2020	FP	6M	2	2020	Year-3	1	0	Winter
2/19/2020	FP	7M	2	2020	Year-3		1	Winter
2/19/2020	FP	10M	2	2020	Year-3	1	0	Winter
2/19/2020	FP	12M	2	2020	Year-3	1	0	Winter
2/19/2020	FP	14M	2	2020	Year-3	1	0	Winter
2/19/2020	FP	8A	2	2020	Year-3		1	Winter
2/19/2020	FP	9A	2	2020	Year-3		1	Winter
2/19/2020	FP	11A	2	2020	Year-3		1	Winter
2/19/2020	FP	13A	2	2020	Year-3		1	Winter
2/20/2020	FP	1M	2	2020	Year-3	1	0	Winter
2/20/2020	FP	2M	2	2020	Year-3	1	0	Winter
2/20/2020	FP	3M	2	2020	Year-3	1	0	Winter
2/20/2020	FP	4M	2	2020	Year-3	1	0	Winter
2/20/2020	FP	5M	2	2020	Year-3	1	0	Winter
2/20/2020	FP	6M	2	2020	Year-3	1	0	Winter
2/20/2020	FP	7M	2	2020	Year-3		1	Winter
2/20/2020	FP	10M	2	2020	Year-3	1	0	Winter
2/20/2020	FP	12M	2	2020	Year-3	1	0	Winter
2/20/2020	FP	14M	2	2020	Year-3	1	0	Winter
2/20/2020	FP	8A	2	2020	Year-3		1	Winter
2/20/2020	FP	9A	2	2020	Year-3		1	Winter
2/20/2020	FP	11A	2	2020	Year-3		1	Winter
2/20/2020	FP	13A	2	2020	Year-3		1	Winter
2/21/2020	FP	1M	2	2020	Year-3	1	0	Winter

Date	Species	Site	Month	Year	Survey Year	Presence	Data Gap	Season
2/21/2020	FP	2M	2	2020	Year-3	1	0	Winter
2/21/2020	FP	3M	2	2020	Year-3	1	0	Winter
2/21/2020	FP	4M	2	2020	Year-3	1	0	Winter
2/21/2020	FP	5M	2	2020	Year-3	1	0	Winter
2/21/2020	FP	6M	2	2020	Year-3	1	0	Winter
2/21/2020	FP	7M	2	2020	Year-3		1	Winter
2/21/2020	FP	10M	2	2020	Year-3	1	0	Winter
2/21/2020	FP	12M	2	2020	Year-3	1	0	Winter
2/21/2020	FP	14M	2	2020	Year-3	1	0	Winter
2/21/2020	FP	8A	2	2020	Year-3		1	Winter
2/21/2020	FP	9A	2	2020	Year-3		1	Winter
2/21/2020	FP	11A	2	2020	Year-3		1	Winter
2/21/2020	FP	13A	2	2020	Year-3		1	Winter
2/22/2020	FP	1M	2	2020	Year-3	1	0	Winter
2/22/2020	FP	2M	2	2020	Year-3	1	0	Winter
2/22/2020	FP	3M	2	2020	Year-3	1	0	Winter
2/22/2020	FP	4M	2	2020	Year-3	1	0	Winter
2/22/2020	FP	5M	2	2020	Year-3	1	0	Winter
2/22/2020	FP	6M	2	2020	Year-3	1	0	Winter
2/22/2020	FP	7M	2	2020	Year-3		1	Winter
2/22/2020	FP	10M	2	2020	Year-3	1	0	Winter
2/22/2020	FP	12M	2	2020	Year-3	1	0	Winter
2/22/2020	FP	14M	2	2020	Year-3	1	0	Winter
2/22/2020	FP	8A	2	2020	Year-3		1	Winter
2/22/2020	FP	9A	2	2020	Year-3		1	Winter
2/22/2020	FP	11A	2	2020	Year-3		1	Winter
2/22/2020	FP	13A	2	2020	Year-3		1	Winter
2/23/2020	FP	1M	2	2020	Year-3	1	0	Winter
2/23/2020	FP	2M	2	2020	Year-3	1	0	Winter
2/23/2020	FP	3M	2	2020	Year-3	1	0	Winter
2/23/2020	FP	4M	2	2020	Year-3	1	0	Winter
2/23/2020	FP	5M	2	2020	Year-3	1	0	Winter
2/23/2020	FP	6M	2	2020	Year-3	1	0	Winter
2/23/2020	FP	7M	2	2020	Year-3		1	Winter
2/23/2020	FP	10M	2	2020	Year-3	1	0	Winter
2/23/2020	FP	12M	2	2020	Year-3	1	0	Winter
2/23/2020	FP	14M	2	2020	Year-3	1	0	Winter
2/23/2020	FP	8A	2	2020	Year-3		1	Winter
2/23/2020	FP	9A	2	2020	Year-3		1	Winter
2/23/2020	FP	11A	2	2020	Year-3		1	Winter
2/23/2020	FP	13A	2	2020	Year-3		1	Winter
2/24/2020	FP	1M	2	2020	Year-3	1	0	Winter
2/24/2020	FP	2M	2	2020	Year-3	1	0	Winter
2/24/2020	FP	3M	2	2020	Year-3	1	0	Winter

Date	Species	Site	Month	Year	Survey Year	Presence	Data Gap	Season
2/24/2020	FP	4M	2	2020	Year-3	1	0	Winter
2/24/2020	FP	5M	2	2020	Year-3	1	0	Winter
2/24/2020	FP	6M	2	2020	Year-3	1	0	Winter
2/24/2020	FP	7M	2	2020	Year-3		1	Winter
2/24/2020	FP	10M	2	2020	Year-3	1	0	Winter
2/24/2020	FP	12M	2	2020	Year-3	1	0	Winter
2/24/2020	FP	14M	2	2020	Year-3	1	0	Winter
2/24/2020	FP	8A	2	2020	Year-3		1	Winter
2/24/2020	FP	9A	2	2020	Year-3		1	Winter
2/24/2020	FP	11A	2	2020	Year-3		1	Winter
2/24/2020	FP	13A	2	2020	Year-3		1	Winter
2/25/2020	FP	1M	2	2020	Year-3	1	0	Winter
2/25/2020	FP	2M	2	2020	Year-3	1	0	Winter
2/25/2020	FP	3M	2	2020	Year-3	1	0	Winter
2/25/2020	FP	4M	2	2020	Year-3	1	0	Winter
2/25/2020	FP	5M	2	2020	Year-3	1	0	Winter
2/25/2020	FP	6M	2	2020	Year-3	1	0	Winter
2/25/2020	FP	7M	2	2020	Year-3		1	Winter
2/25/2020	FP	10M	2	2020	Year-3	1	0	Winter
2/25/2020	FP	12M	2	2020	Year-3	1	0	Winter
2/25/2020	FP	14M	2	2020	Year-3	1	0	Winter
2/25/2020	FP	8A	2	2020	Year-3		1	Winter
2/25/2020	FP	9A	2	2020	Year-3		1	Winter
2/25/2020	FP	11A	2	2020	Year-3		1	Winter
2/25/2020	FP	13A	2	2020	Year-3		1	Winter
2/26/2020	FP	1M	2	2020	Year-3	1	0	Winter
2/26/2020	FP	2M	2	2020	Year-3	1	0	Winter
2/26/2020	FP	3M	2	2020	Year-3	1	0	Winter
2/26/2020	FP	4M	2	2020	Year-3	1	0	Winter
2/26/2020	FP	5M	2	2020	Year-3	1	0	Winter
2/26/2020	FP	6M	2	2020	Year-3	1	0	Winter
2/26/2020	FP	7M	2	2020	Year-3		1	Winter
2/26/2020	FP	10M	2	2020	Year-3	1	0	Winter
2/26/2020	FP	12M	2	2020	Year-3	1	0	Winter
2/26/2020	FP	14M	2	2020	Year-3	1	0	Winter
2/26/2020	FP	8A	2	2020	Year-3		1	Winter
2/26/2020	FP	9A	2	2020	Year-3		1	Winter
2/26/2020	FP	11A	2	2020	Year-3		1	Winter
2/26/2020	FP	13A	2	2020	Year-3		1	Winter
2/27/2020	FP	1M	2	2020	Year-3	1	0	Winter
2/27/2020	FP	2M	2	2020	Year-3	1	0	Winter
2/27/2020	FP	3M	2	2020	Year-3	1	0	Winter
2/27/2020	FP	4M	2	2020	Year-3	1	0	Winter
2/27/2020	FP	5M	2	2020	Year-3	1	0	Winter

Date	Species	Site	Month	Year	Survey Year	Presence	Data Gap	Season
2/27/2020	FP	6M	2	2020	Year-3	1	0	Winter
2/27/2020	FP	7M	2	2020	Year-3		1	Winter
2/27/2020	FP	10M	2	2020	Year-3	1	0	Winter
2/27/2020	FP	12M	2	2020	Year-3	1	0	Winter
2/27/2020	FP	14M	2	2020	Year-3	1	0	Winter
2/27/2020	FP	8A	2	2020	Year-3		1	Winter
2/27/2020	FP	9A	2	2020	Year-3		1	Winter
2/27/2020	FP	11A	2	2020	Year-3		1	Winter
2/27/2020	FP	13A	2	2020	Year-3		1	Winter
2/28/2020	FP	1M	2	2020	Year-3	1	0	Winter
2/28/2020	FP	2M	2	2020	Year-3	1	0	Winter
2/28/2020	FP	3M	2	2020	Year-3	1	0	Winter
2/28/2020	FP	4M	2	2020	Year-3	1	0	Winter
2/28/2020	FP	5M	2	2020	Year-3	1	0	Winter
2/28/2020	FP	6M	2	2020	Year-3	0	0	Winter
2/28/2020	FP	7M	2	2020	Year-3		1	Winter
2/28/2020	FP	10M	2	2020	Year-3	1	0	Winter
2/28/2020	FP	12M	2	2020	Year-3	1	0	Winter
2/28/2020	FP	14M	2	2020	Year-3	1	0	Winter
2/28/2020	FP	8A	2	2020	Year-3		1	Winter
2/28/2020	FP	9A	2	2020	Year-3		1	Winter
2/28/2020	FP	11A	2	2020	Year-3		1	Winter
2/28/2020	FP	13A	2	2020	Year-3		1	Winter
2/29/2020	FP	1M	2	2020	Year-3	1	0	Winter
2/29/2020	FP	2M	2	2020	Year-3	1	0	Winter
2/29/2020	FP	3M	2	2020	Year-3	1	0	Winter
2/29/2020	FP	4M	2	2020	Year-3	1	0	Winter
2/29/2020	FP	5M	2	2020	Year-3	1	0	Winter
2/29/2020	FP	6M	2	2020	Year-3	1	0	Winter
2/29/2020	FP	7M	2	2020	Year-3		1	Winter
2/29/2020	FP	10M	2	2020	Year-3	1	0	Winter
2/29/2020	FP	12M	2	2020	Year-3	1	0	Winter
2/29/2020	FP	14M	2	2020	Year-3	1	0	Winter
2/29/2020	FP	8A	2	2020	Year-3		1	Winter
2/29/2020	FP	9A	2	2020	Year-3		1	Winter
2/29/2020	FP	11A	2	2020	Year-3		1	Winter
2/29/2020	FP	13A	2	2020	Year-3		1	Winter
3/1/2020	FP	1M	3	2020	Year-3	1	0	Spring
3/1/2020	FP	2M	3	2020	Year-3	1	0	Spring
3/1/2020	FP	3M	3	2020	Year-3	1	0	Spring
3/1/2020	FP	4M	3	2020	Year-3	1	0	Spring
3/1/2020	FP	5M	3	2020	Year-3	1	0	Spring
3/1/2020	FP	6M	3	2020	Year-3	0	0	Spring
3/1/2020	FP	7M	3	2020	Year-3		1	Spring

Date	Species	Site	Month	Year	Survey Year	Presence	Data Gap	Season
3/1/2020	FP	10M	3	2020	Year-3	1	0	Spring
3/1/2020	FP	12M	3	2020	Year-3	1	0	Spring
3/1/2020	FP	14M	3	2020	Year-3	1	0	Spring
3/1/2020	FP	8A	3	2020	Year-3		1	Spring
3/1/2020	FP	9A	3	2020	Year-3		1	Spring
3/1/2020	FP	11A	3	2020	Year-3		1	Spring
3/1/2020	FP	13A	3	2020	Year-3		1	Spring
3/2/2020	FP	1M	3	2020	Year-3	1	0	Spring
3/2/2020	FP	2M	3	2020	Year-3	1	0	Spring
3/2/2020	FP	3M	3	2020	Year-3	1	0	Spring
3/2/2020	FP	4M	3	2020	Year-3	1	0	Spring
3/2/2020	FP	5M	3	2020	Year-3	1	0	Spring
3/2/2020	FP	6M	3	2020	Year-3	1	0	Spring
3/2/2020	FP	7M	3	2020	Year-3		1	Spring
3/2/2020	FP	10M	3	2020	Year-3	1	0	Spring
3/2/2020	FP	12M	3	2020	Year-3	1	0	Spring
3/2/2020	FP	14M	3	2020	Year-3	1	0	Spring
3/2/2020	FP	8A	3	2020	Year-3		1	Spring
3/2/2020	FP	9A	3	2020	Year-3		1	Spring
3/2/2020	FP	11A	3	2020	Year-3		1	Spring
3/2/2020	FP	13A	3	2020	Year-3		1	Spring
3/3/2020	FP	1M	3	2020	Year-3	1	0	Spring
3/3/2020	FP	2M	3	2020	Year-3	1	0	Spring
3/3/2020	FP	3M	3	2020	Year-3	1	0	Spring
3/3/2020	FP	4M	3	2020	Year-3	1	0	Spring
3/3/2020	FP	5M	3	2020	Year-3	0	0	Spring
3/3/2020	FP	6M	3	2020	Year-3	1	0	Spring
3/3/2020	FP	7M	3	2020	Year-3		1	Spring
3/3/2020	FP	10M	3	2020	Year-3	1	0	Spring
3/3/2020	FP	12M	3	2020	Year-3	1	0	Spring
3/3/2020	FP	14M	3	2020	Year-3	1	0	Spring
3/3/2020	FP	8A	3	2020	Year-3		1	Spring
3/3/2020	FP	9A	3	2020	Year-3		1	Spring
3/3/2020	FP	11A	3	2020	Year-3		1	Spring
3/3/2020	FP	13A	3	2020	Year-3		1	Spring
3/4/2020	FP	1M	3	2020	Year-3	1	0	Spring
3/4/2020	FP	2M	3	2020	Year-3	1	0	Spring
3/4/2020	FP	3M	3	2020	Year-3	1	0	Spring
3/4/2020	FP	4M	3	2020	Year-3	1	0	Spring
3/4/2020	FP	5M	3	2020	Year-3	0	0	Spring
3/4/2020	FP	6M	3	2020	Year-3	0	0	Spring
3/4/2020	FP	7M	3	2020	Year-3		1	Spring
3/4/2020	FP	10M	3	2020	Year-3	1	0	Spring
3/4/2020	FP	12M	3	2020	Year-3	0	0	Spring

Date	Species	Site	Month	Year	Survey Year	Presence	Data Gap	Season
3/4/2020	FP	14M	3	2020	Year-3	1	0	Spring
3/4/2020	FP	8A	3	2020	Year-3		1	Spring
3/4/2020	FP	9A	3	2020	Year-3		1	Spring
3/4/2020	FP	11A	3	2020	Year-3		1	Spring
3/4/2020	FP	13A	3	2020	Year-3		1	Spring
3/5/2020	FP	1M	3	2020	Year-3	1	0	Spring
3/5/2020	FP	2M	3	2020	Year-3	1	0	Spring
3/5/2020	FP	3M	3	2020	Year-3	1	0	Spring
3/5/2020	FP	4M	3	2020	Year-3	1	0	Spring
3/5/2020	FP	5M	3	2020	Year-3	0	0	Spring
3/5/2020	FP	6M	3	2020	Year-3	0	0	Spring
3/5/2020	FP	7M	3	2020	Year-3		1	Spring
3/5/2020	FP	10M	3	2020	Year-3	1	0	Spring
3/5/2020	FP	12M	3	2020	Year-3	1	0	Spring
3/5/2020	FP	14M	3	2020	Year-3	1	0	Spring
3/5/2020	FP	8A	3	2020	Year-3		1	Spring
3/5/2020	FP	9A	3	2020	Year-3		1	Spring
3/5/2020	FP	11A	3	2020	Year-3		1	Spring
3/5/2020	FP	13A	3	2020	Year-3		1	Spring
3/6/2020	FP	1M	3	2020	Year-3	1	0	Spring
3/6/2020	FP	2M	3	2020	Year-3	1	0	Spring
3/6/2020	FP	3M	3	2020	Year-3	1	0	Spring
3/6/2020	FP	4M	3	2020	Year-3	1	0	Spring
3/6/2020	FP	5M	3	2020	Year-3	0	0	Spring
3/6/2020	FP	6M	3	2020	Year-3	1	0	Spring
3/6/2020	FP	7M	3	2020	Year-3		1	Spring
3/6/2020	FP	10M	3	2020	Year-3	1	0	Spring
3/6/2020	FP	12M	3	2020	Year-3	1	0	Spring
3/6/2020	FP	14M	3	2020	Year-3	1	0	Spring
3/6/2020	FP	8A	3	2020	Year-3		1	Spring
3/6/2020	FP	9A	3	2020	Year-3		1	Spring
3/6/2020	FP	11A	3	2020	Year-3		1	Spring
3/6/2020	FP	13A	3	2020	Year-3		1	Spring
3/7/2020	FP	1M	3	2020	Year-3	1	0	Spring
3/7/2020	FP	2M	3	2020	Year-3	1	0	Spring
3/7/2020	FP	3M	3	2020	Year-3	1	0	Spring
3/7/2020	FP	4M	3	2020	Year-3	0	0	Spring
3/7/2020	FP	5M	3	2020	Year-3	1	0	Spring
3/7/2020	FP	6M	3	2020	Year-3	1	0	Spring
3/7/2020	FP	7M	3	2020	Year-3		1	Spring
3/7/2020	FP	10M	3	2020	Year-3	1	0	Spring
3/7/2020	FP	12M	3	2020	Year-3	1	0	Spring
3/7/2020	FP	14M	3	2020	Year-3	1	0	Spring
3/7/2020	FP	8A	3	2020	Year-3		1	Spring

Date	Species	Site	Month	Year	Survey Year	Presence	Data Gap	Season
3/7/2020	FP	9A	3	2020	Year-3		1	Spring
3/7/2020	FP	11A	3	2020	Year-3		1	Spring
3/7/2020	FP	13A	3	2020	Year-3		1	Spring
3/8/2020	FP	1M	3	2020	Year-3	1	0	Spring
3/8/2020	FP	2M	3	2020	Year-3	1	0	Spring
3/8/2020	FP	3M	3	2020	Year-3	1	0	Spring
3/8/2020	FP	4M	3	2020	Year-3	1	0	Spring
3/8/2020	FP	5M	3	2020	Year-3	1	0	Spring
3/8/2020	FP	6M	3	2020	Year-3	1	0	Spring
3/8/2020	FP	7M	3	2020	Year-3		1	Spring
3/8/2020	FP	10M	3	2020	Year-3	1	0	Spring
3/8/2020	FP	12M	3	2020	Year-3	1	0	Spring
3/8/2020	FP	14M	3	2020	Year-3	1	0	Spring
3/8/2020	FP	8A	3	2020	Year-3		1	Spring
3/8/2020	FP	9A	3	2020	Year-3		1	Spring
3/8/2020	FP	11A	3	2020	Year-3		1	Spring
3/8/2020	FP	13A	3	2020	Year-3		1	Spring
3/9/2020	FP	1M	3	2020	Year-3	1	0	Spring
3/9/2020	FP	2M	3	2020	Year-3	1	0	Spring
3/9/2020	FP	3M	3	2020	Year-3	1	0	Spring
3/9/2020	FP	4M	3	2020	Year-3	1	0	Spring
3/9/2020	FP	5M	3	2020	Year-3	0	0	Spring
3/9/2020	FP	6M	3	2020	Year-3	1	0	Spring
3/9/2020	FP	7M	3	2020	Year-3		1	Spring
3/9/2020	FP	10M	3	2020	Year-3	1	0	Spring
3/9/2020	FP	12M	3	2020	Year-3	1	0	Spring
3/9/2020	FP	14M	3	2020	Year-3	1	0	Spring
3/9/2020	FP	8A	3	2020	Year-3		1	Spring
3/9/2020	FP	9A	3	2020	Year-3		1	Spring
3/9/2020	FP	11A	3	2020	Year-3		1	Spring
3/9/2020	FP	13A	3	2020	Year-3		1	Spring
3/10/2020	FP	1M	3	2020	Year-3	1	0	Spring
3/10/2020	FP	2M	3	2020	Year-3	1	0	Spring
3/10/2020	FP	3M	3	2020	Year-3	1	0	Spring
3/10/2020	FP	4M	3	2020	Year-3	1	0	Spring
3/10/2020	FP	5M	3	2020	Year-3	1	0	Spring
3/10/2020	FP	6M	3	2020	Year-3	1	0	Spring
3/10/2020	FP	7M	3	2020	Year-3		1	Spring
3/10/2020	FP	10M	3	2020	Year-3	1	0	Spring
3/10/2020	FP	12M	3	2020	Year-3	1	0	Spring
3/10/2020	FP	14M	3	2020	Year-3	1	0	Spring
3/10/2020	FP	8A	3	2020	Year-3		1	Spring
3/10/2020	FP	9A	3	2020	Year-3		1	Spring
3/10/2020	FP	11A	3	2020	Year-3		1	Spring

Date	Species	Site	Month	Year	Survey Year	Presence	Data Gap	Season
3/10/2020	FP	13A	3	2020	Year-3		1	Spring
3/11/2020	FP	1M	3	2020	Year-3	1	0	Spring
3/11/2020	FP	2M	3	2020	Year-3	1	0	Spring
3/11/2020	FP	3M	3	2020	Year-3	1	0	Spring
3/11/2020	FP	4M	3	2020	Year-3	1	0	Spring
3/11/2020	FP	5M	3	2020	Year-3	1	0	Spring
3/11/2020	FP	6M	3	2020	Year-3	1	0	Spring
3/11/2020	FP	7M	3	2020	Year-3		1	Spring
3/11/2020	FP	10M	3	2020	Year-3	1	0	Spring
3/11/2020	FP	12M	3	2020	Year-3	1	0	Spring
3/11/2020	FP	14M	3	2020	Year-3	1	0	Spring
3/11/2020	FP	8A	3	2020	Year-3		1	Spring
3/11/2020	FP	9A	3	2020	Year-3		1	Spring
3/11/2020	FP	11A	3	2020	Year-3		1	Spring
3/11/2020	FP	13A	3	2020	Year-3		1	Spring
3/12/2020	FP	1M	3	2020	Year-3	1	0	Spring
3/12/2020	FP	2M	3	2020	Year-3	1	0	Spring
3/12/2020	FP	3M	3	2020	Year-3	1	0	Spring
3/12/2020	FP	4M	3	2020	Year-3	1	0	Spring
3/12/2020	FP	5M	3	2020	Year-3	1	0	Spring
3/12/2020	FP	6M	3	2020	Year-3	1	0	Spring
3/12/2020	FP	7M	3	2020	Year-3		1	Spring
3/12/2020	FP	10M	3	2020	Year-3	1	0	Spring
3/12/2020	FP	12M	3	2020	Year-3	1	0	Spring
3/12/2020	FP	14M	3	2020	Year-3	1	0	Spring
3/12/2020	FP	8A	3	2020	Year-3		1	Spring
3/12/2020	FP	9A	3	2020	Year-3		1	Spring
3/12/2020	FP	11A	3	2020	Year-3		1	Spring
3/12/2020	FP	13A	3	2020	Year-3		1	Spring
3/13/2020	FP	1M	3	2020	Year-3	1	0	Spring
3/13/2020	FP	2M	3	2020	Year-3	1	0	Spring
3/13/2020	FP	3M	3	2020	Year-3	1	0	Spring
3/13/2020	FP	4M	3	2020	Year-3	1	0	Spring
3/13/2020	FP	5M	3	2020	Year-3	0	0	Spring
3/13/2020	FP	6M	3	2020	Year-3	0	0	Spring
3/13/2020	FP	7M	3	2020	Year-3		1	Spring
3/13/2020	FP	10M	3	2020	Year-3	0	0	Spring
3/13/2020	FP	12M	3	2020	Year-3	1	0	Spring
3/13/2020	FP	14M	3	2020	Year-3	1	0	Spring
3/13/2020	FP	8A	3	2020	Year-3		1	Spring
3/13/2020	FP	9A	3	2020	Year-3		1	Spring
3/13/2020	FP	11A	3	2020	Year-3		1	Spring
3/13/2020	FP	13A	3	2020	Year-3		1	Spring
3/14/2020	FP	1M	3	2020	Year-3	1	0	Spring

Date	Species	Site	Month	Year	Survey Year	Presence	Data Gap	Season
3/14/2020	FP	2M	3	2020	Year-3	1	0	Spring
3/14/2020	FP	3M	3	2020	Year-3	1	0	Spring
3/14/2020	FP	4M	3	2020	Year-3	1	0	Spring
3/14/2020	FP	5M	3	2020	Year-3	1	0	Spring
3/14/2020	FP	6M	3	2020	Year-3	1	0	Spring
3/14/2020	FP	7M	3	2020	Year-3		1	Spring
3/14/2020	FP	10M	3	2020	Year-3	1	0	Spring
3/14/2020	FP	12M	3	2020	Year-3	1	0	Spring
3/14/2020	FP	14M	3	2020	Year-3	1	0	Spring
3/14/2020	FP	8A	3	2020	Year-3		1	Spring
3/14/2020	FP	9A	3	2020	Year-3		1	Spring
3/14/2020	FP	11A	3	2020	Year-3		1	Spring
3/14/2020	FP	13A	3	2020	Year-3		1	Spring
3/15/2020	FP	1M	3	2020	Year-3	1	0	Spring
3/15/2020	FP	2M	3	2020	Year-3	1	0	Spring
3/15/2020	FP	3M	3	2020	Year-3	1	0	Spring
3/15/2020	FP	4M	3	2020	Year-3	1	0	Spring
3/15/2020	FP	5M	3	2020	Year-3	1	0	Spring
3/15/2020	FP	6M	3	2020	Year-3	1	0	Spring
3/15/2020	FP	7M	3	2020	Year-3		1	Spring
3/15/2020	FP	10M	3	2020	Year-3	1	0	Spring
3/15/2020	FP	12M	3	2020	Year-3	1	0	Spring
3/15/2020	FP	14M	3	2020	Year-3	1	0	Spring
3/15/2020	FP	8A	3	2020	Year-3		1	Spring
3/15/2020	FP	9A	3	2020	Year-3		1	Spring
3/15/2020	FP	11A	3	2020	Year-3		1	Spring
3/15/2020	FP	13A	3	2020	Year-3		1	Spring
3/16/2020	FP	1M	3	2020	Year-3	1	0	Spring
3/16/2020	FP	2M	3	2020	Year-3	1	0	Spring
3/16/2020	FP	3M	3	2020	Year-3	1	0	Spring
3/16/2020	FP	4M	3	2020	Year-3	1	0	Spring
3/16/2020	FP	5M	3	2020	Year-3	1	0	Spring
3/16/2020	FP	6M	3	2020	Year-3	1	0	Spring
3/16/2020	FP	7M	3	2020	Year-3		1	Spring
3/16/2020	FP	10M	3	2020	Year-3	1	0	Spring
3/16/2020	FP	12M	3	2020	Year-3	1	0	Spring
3/16/2020	FP	14M	3	2020	Year-3	1	0	Spring
3/16/2020	FP	8A	3	2020	Year-3		1	Spring
3/16/2020	FP	9A	3	2020	Year-3		1	Spring
3/16/2020	FP	11A	3	2020	Year-3		1	Spring
3/16/2020	FP	13A	3	2020	Year-3		1	Spring
3/17/2020	FP	1M	3	2020	Year-3	1	0	Spring
3/17/2020	FP	2M	3	2020	Year-3	1	0	Spring
3/17/2020	FP	3M	3	2020	Year-3	1	0	Spring

Date	Species	Site	Month	Year	Survey Year	Presence	Data Gap	Season
3/17/2020	FP	4M	3	2020	Year-3	1	0	Spring
3/17/2020	FP	5M	3	2020	Year-3	1	0	Spring
3/17/2020	FP	6M	3	2020	Year-3	1	0	Spring
3/17/2020	FP	7M	3	2020	Year-3		1	Spring
3/17/2020	FP	10M	3	2020	Year-3	1	0	Spring
3/17/2020	FP	12M	3	2020	Year-3	1	0	Spring
3/17/2020	FP	14M	3	2020	Year-3	1	0	Spring
3/17/2020	FP	8A	3	2020	Year-3		1	Spring
3/17/2020	FP	9A	3	2020	Year-3		1	Spring
3/17/2020	FP	11A	3	2020	Year-3		1	Spring
3/17/2020	FP	13A	3	2020	Year-3		1	Spring
3/18/2020	FP	1M	3	2020	Year-3	1	0	Spring
3/18/2020	FP	2M	3	2020	Year-3	1	0	Spring
3/18/2020	FP	3M	3	2020	Year-3	1	0	Spring
3/18/2020	FP	4M	3	2020	Year-3	1	0	Spring
3/18/2020	FP	5M	3	2020	Year-3	0	0	Spring
3/18/2020	FP	6M	3	2020	Year-3	1	0	Spring
3/18/2020	FP	7M	3	2020	Year-3		1	Spring
3/18/2020	FP	10M	3	2020	Year-3	1	0	Spring
3/18/2020	FP	12M	3	2020	Year-3	1	0	Spring
3/18/2020	FP	14M	3	2020	Year-3	1	0	Spring
3/18/2020	FP	8A	3	2020	Year-3		1	Spring
3/18/2020	FP	9A	3	2020	Year-3		1	Spring
3/18/2020	FP	11A	3	2020	Year-3		1	Spring
3/18/2020	FP	13A	3	2020	Year-3		1	Spring
3/19/2020	FP	1M	3	2020	Year-3	1	0	Spring
3/19/2020	FP	2M	3	2020	Year-3	1	0	Spring
3/19/2020	FP	3M	3	2020	Year-3	1	0	Spring
3/19/2020	FP	4M	3	2020	Year-3	1	0	Spring
3/19/2020	FP	5M	3	2020	Year-3	0	0	Spring
3/19/2020	FP	6M	3	2020	Year-3	1	0	Spring
3/19/2020	FP	7M	3	2020	Year-3		1	Spring
3/19/2020	FP	10M	3	2020	Year-3	1	0	Spring
3/19/2020	FP	12M	3	2020	Year-3	1	0	Spring
3/19/2020	FP	14M	3	2020	Year-3	1	0	Spring
3/19/2020	FP	8A	3	2020	Year-3		1	Spring
3/19/2020	FP	9A	3	2020	Year-3		1	Spring
3/19/2020	FP	11A	3	2020	Year-3		1	Spring
3/19/2020	FP	13A	3	2020	Year-3		1	Spring
3/20/2020	FP	1M	3	2020	Year-3	1	0	Spring
3/20/2020	FP	2M	3	2020	Year-3	1	0	Spring
3/20/2020	FP	3M	3	2020	Year-3	1	0	Spring
3/20/2020	FP	4M	3	2020	Year-3	1	0	Spring
3/20/2020	FP	5M	3	2020	Year-3	0	0	Spring

Date	Species	Site	Month	Year	Survey Year	Presence	Data Gap	Season
3/20/2020	FP	6M	3	2020	Year-3	1	0	Spring
3/20/2020	FP	7M	3	2020	Year-3		1	Spring
3/20/2020	FP	10M	3	2020	Year-3	1	0	Spring
3/20/2020	FP	12M	3	2020	Year-3	1	0	Spring
3/20/2020	FP	14M	3	2020	Year-3	1	0	Spring
3/20/2020	FP	8A	3	2020	Year-3		1	Spring
3/20/2020	FP	9A	3	2020	Year-3		1	Spring
3/20/2020	FP	11A	3	2020	Year-3		1	Spring
3/20/2020	FP	13A	3	2020	Year-3		1	Spring
3/21/2020	FP	1M	3	2020	Year-3	1	0	Spring
3/21/2020	FP	2M	3	2020	Year-3	1	0	Spring
3/21/2020	FP	3M	3	2020	Year-3	1	0	Spring
3/21/2020	FP	4M	3	2020	Year-3	1	0	Spring
3/21/2020	FP	5M	3	2020	Year-3	1	0	Spring
3/21/2020	FP	6M	3	2020	Year-3	1	0	Spring
3/21/2020	FP	7M	3	2020	Year-3		1	Spring
3/21/2020	FP	10M	3	2020	Year-3	0	0	Spring
3/21/2020	FP	12M	3	2020	Year-3	1	0	Spring
3/21/2020	FP	14M	3	2020	Year-3	1	0	Spring
3/21/2020	FP	8A	3	2020	Year-3		1	Spring
3/21/2020	FP	9A	3	2020	Year-3		1	Spring
3/21/2020	FP	11A	3	2020	Year-3		1	Spring
3/21/2020	FP	13A	3	2020	Year-3		1	Spring
3/22/2020	FP	1M	3	2020	Year-3	1	0	Spring
3/22/2020	FP	2M	3	2020	Year-3	1	0	Spring
3/22/2020	FP	3M	3	2020	Year-3	1	0	Spring
3/22/2020	FP	4M	3	2020	Year-3	1	0	Spring
3/22/2020	FP	5M	3	2020	Year-3	1	0	Spring
3/22/2020	FP	6M	3	2020	Year-3	1	0	Spring
3/22/2020	FP	7M	3	2020	Year-3		1	Spring
3/22/2020	FP	10M	3	2020	Year-3	0	0	Spring
3/22/2020	FP	12M	3	2020	Year-3	1	0	Spring
3/22/2020	FP	14M	3	2020	Year-3	1	0	Spring
3/22/2020	FP	8A	3	2020	Year-3		1	Spring
3/22/2020	FP	9A	3	2020	Year-3		1	Spring
3/22/2020	FP	11A	3	2020	Year-3		1	Spring
3/22/2020	FP	13A	3	2020	Year-3		1	Spring
3/23/2020	FP	1M	3	2020	Year-3	1	0	Spring
3/23/2020	FP	2M	3	2020	Year-3	1	0	Spring
3/23/2020	FP	3M	3	2020	Year-3	1	0	Spring
3/23/2020	FP	4M	3	2020	Year-3	1	0	Spring
3/23/2020	FP	5M	3	2020	Year-3	0	0	Spring
3/23/2020	FP	6M	3	2020	Year-3	1	0	Spring
3/23/2020	FP	7M	3	2020	Year-3		1	Spring

Date	Species	Site	Month	Year	Survey Year	Presence	Data Gap	Season
3/23/2020	FP	10M	3	2020	Year-3	0	0	Spring
3/23/2020	FP	12M	3	2020	Year-3	1	0	Spring
3/23/2020	FP	14M	3	2020	Year-3	1	0	Spring
3/23/2020	FP	8A	3	2020	Year-3		1	Spring
3/23/2020	FP	9A	3	2020	Year-3		1	Spring
3/23/2020	FP	11A	3	2020	Year-3		1	Spring
3/23/2020	FP	13A	3	2020	Year-3		1	Spring
3/24/2020	FP	1M	3	2020	Year-3	1	0	Spring
3/24/2020	FP	2M	3	2020	Year-3	1	0	Spring
3/24/2020	FP	3M	3	2020	Year-3	1	0	Spring
3/24/2020	FP	4M	3	2020	Year-3	1	0	Spring
3/24/2020	FP	5M	3	2020	Year-3	1	0	Spring
3/24/2020	FP	6M	3	2020	Year-3	1	0	Spring
3/24/2020	FP	7M	3	2020	Year-3		1	Spring
3/24/2020	FP	10M	3	2020	Year-3	1	0	Spring
3/24/2020	FP	12M	3	2020	Year-3	1	0	Spring
3/24/2020	FP	14M	3	2020	Year-3	1	0	Spring
3/24/2020	FP	8A	3	2020	Year-3		1	Spring
3/24/2020	FP	9A	3	2020	Year-3		1	Spring
3/24/2020	FP	11A	3	2020	Year-3		1	Spring
3/24/2020	FP	13A	3	2020	Year-3		1	Spring
3/25/2020	FP	1M	3	2020	Year-3	1	0	Spring
3/25/2020	FP	2M	3	2020	Year-3	1	0	Spring
3/25/2020	FP	3M	3	2020	Year-3	1	0	Spring
3/25/2020	FP	4M	3	2020	Year-3	1	0	Spring
3/25/2020	FP	5M	3	2020	Year-3	1	0	Spring
3/25/2020	FP	6M	3	2020	Year-3	1	0	Spring
3/25/2020	FP	7M	3	2020	Year-3		1	Spring
3/25/2020	FP	10M	3	2020	Year-3	0	0	Spring
3/25/2020	FP	12M	3	2020	Year-3	1	0	Spring
3/25/2020	FP	14M	3	2020	Year-3	1	0	Spring
3/25/2020	FP	8A	3	2020	Year-3		1	Spring
3/25/2020	FP	9A	3	2020	Year-3		1	Spring
3/25/2020	FP	11A	3	2020	Year-3		1	Spring
3/25/2020	FP	13A	3	2020	Year-3		1	Spring
3/26/2020	FP	1M	3	2020	Year-3	1	0	Spring
3/26/2020	FP	2M	3	2020	Year-3	1	0	Spring
3/26/2020	FP	3M	3	2020	Year-3	1	0	Spring
3/26/2020	FP	4M	3	2020	Year-3	1	0	Spring
3/26/2020	FP	5M	3	2020	Year-3	1	0	Spring
3/26/2020	FP	6M	3	2020	Year-3	1	0	Spring
3/26/2020	FP	7M	3	2020	Year-3		1	Spring
3/26/2020	FP	10M	3	2020	Year-3	0	0	Spring
3/26/2020	FP	12M	3	2020	Year-3	1	0	Spring

Date	Species	Site	Month	Year	Survey Year	Presence	Data Gap	Season
3/26/2020	FP	14M	3	2020	Year-3	1	0	Spring
3/26/2020	FP	8A	3	2020	Year-3		1	Spring
3/26/2020	FP	9A	3	2020	Year-3		1	Spring
3/26/2020	FP	11A	3	2020	Year-3		1	Spring
3/26/2020	FP	13A	3	2020	Year-3		1	Spring
3/27/2020	FP	1M	3	2020	Year-3	1	0	Spring
3/27/2020	FP	2M	3	2020	Year-3	1	0	Spring
3/27/2020	FP	3M	3	2020	Year-3	1	0	Spring
3/27/2020	FP	4M	3	2020	Year-3	1	0	Spring
3/27/2020	FP	5M	3	2020	Year-3	1	0	Spring
3/27/2020	FP	6M	3	2020	Year-3	1	0	Spring
3/27/2020	FP	7M	3	2020	Year-3		1	Spring
3/27/2020	FP	10M	3	2020	Year-3	1	0	Spring
3/27/2020	FP	12M	3	2020	Year-3	1	0	Spring
3/27/2020	FP	14M	3	2020	Year-3	1	0	Spring
3/27/2020	FP	8A	3	2020	Year-3		1	Spring
3/27/2020	FP	9A	3	2020	Year-3		1	Spring
3/27/2020	FP	11A	3	2020	Year-3		1	Spring
3/27/2020	FP	13A	3	2020	Year-3		1	Spring
3/28/2020	FP	1M	3	2020	Year-3	1	0	Spring
3/28/2020	FP	2M	3	2020	Year-3	1	0	Spring
3/28/2020	FP	3M	3	2020	Year-3	1	0	Spring
3/28/2020	FP	4M	3	2020	Year-3	1	0	Spring
3/28/2020	FP	5M	3	2020	Year-3	1	0	Spring
3/28/2020	FP	6M	3	2020	Year-3	1	0	Spring
3/28/2020	FP	7M	3	2020	Year-3		1	Spring
3/28/2020	FP	10M	3	2020	Year-3	1	0	Spring
3/28/2020	FP	12M	3	2020	Year-3	0	0	Spring
3/28/2020	FP	14M	3	2020	Year-3	1	0	Spring
3/28/2020	FP	8A	3	2020	Year-3		1	Spring
3/28/2020	FP	9A	3	2020	Year-3		1	Spring
3/28/2020	FP	11A	3	2020	Year-3		1	Spring
3/28/2020	FP	13A	3	2020	Year-3		1	Spring
3/29/2020	FP	1M	3	2020	Year-3	0	0	Spring
3/29/2020	FP	2M	3	2020	Year-3	1	0	Spring
3/29/2020	FP	3M	3	2020	Year-3	1	0	Spring
3/29/2020	FP	4M	3	2020	Year-3	1	0	Spring
3/29/2020	FP	5M	3	2020	Year-3	1	0	Spring
3/29/2020	FP	6M	3	2020	Year-3	1	0	Spring
3/29/2020	FP	7M	3	2020	Year-3		1	Spring
3/29/2020	FP	10M	3	2020	Year-3	0	0	Spring
3/29/2020	FP	12M	3	2020	Year-3	1	0	Spring
3/29/2020	FP	14M	3	2020	Year-3	1	0	Spring
3/29/2020	FP	8A	3	2020	Year-3		1	Spring

Date	Species	Site	Month	Year	Survey Year	Presence	Data Gap	Season
3/29/2020	FP	9A	3	2020	Year-3		1	Spring
3/29/2020	FP	11A	3	2020	Year-3		1	Spring
3/29/2020	FP	13A	3	2020	Year-3		1	Spring
3/30/2020	FP	1M	3	2020	Year-3	1	0	Spring
3/30/2020	FP	2M	3	2020	Year-3	1	0	Spring
3/30/2020	FP	3M	3	2020	Year-3	1	0	Spring
3/30/2020	FP	4M	3	2020	Year-3	0	0	Spring
3/30/2020	FP	5M	3	2020	Year-3	0	0	Spring
3/30/2020	FP	6M	3	2020	Year-3	0	0	Spring
3/30/2020	FP	7M	3	2020	Year-3		1	Spring
3/30/2020	FP	10M	3	2020	Year-3	0	0	Spring
3/30/2020	FP	12M	3	2020	Year-3	1	0	Spring
3/30/2020	FP	14M	3	2020	Year-3	1	0	Spring
3/30/2020	FP	8A	3	2020	Year-3		1	Spring
3/30/2020	FP	9A	3	2020	Year-3		1	Spring
3/30/2020	FP	11A	3	2020	Year-3		1	Spring
3/30/2020	FP	13A	3	2020	Year-3		1	Spring
3/31/2020	FP	1M	3	2020	Year-3	1	0	Spring
3/31/2020	FP	2M	3	2020	Year-3	1	0	Spring
3/31/2020	FP	3M	3	2020	Year-3	1	0	Spring
3/31/2020	FP	4M	3	2020	Year-3	0	0	Spring
3/31/2020	FP	5M	3	2020	Year-3	0	0	Spring
3/31/2020	FP	6M	3	2020	Year-3	1	0	Spring
3/31/2020	FP	7M	3	2020	Year-3		1	Spring
3/31/2020	FP	10M	3	2020	Year-3	1	0	Spring
3/31/2020	FP	12M	3	2020	Year-3	1	0	Spring
3/31/2020	FP	14M	3	2020	Year-3	1	0	Spring
3/31/2020	FP	8A	3	2020	Year-3		1	Spring
3/31/2020	FP	9A	3	2020	Year-3		1	Spring
3/31/2020	FP	11A	3	2020	Year-3		1	Spring
3/31/2020	FP	13A	3	2020	Year-3		1	Spring
4/1/2020	FP	1M	4	2020	Year-3	1	0	Spring
4/1/2020	FP	2M	4	2020	Year-3	1	0	Spring
4/1/2020	FP	3M	4	2020	Year-3	1	0	Spring
4/1/2020	FP	4M	4	2020	Year-3	1	0	Spring
4/1/2020	FP	5M	4	2020	Year-3	0	0	Spring
4/1/2020	FP	6M	4	2020	Year-3	1	0	Spring
4/1/2020	FP	7M	4	2020	Year-3		1	Spring
4/1/2020	FP	10M	4	2020	Year-3	1	0	Spring
4/1/2020	FP	12M	4	2020	Year-3	1	0	Spring
4/1/2020	FP	14M	4	2020	Year-3	1	0	Spring
4/1/2020	FP	8A	4	2020	Year-3		1	Spring
4/1/2020	FP	9A	4	2020	Year-3		1	Spring
4/1/2020	FP	11A	4	2020	Year-3		1	Spring

Date	Species	Site	Month	Year	Survey Year	Presence	Data Gap	Season
4/1/2020	FP	13A	4	2020	Year-3		1	Spring
4/2/2020	FP	1M	4	2020	Year-3	1	0	Spring
4/2/2020	FP	2M	4	2020	Year-3	1	0	Spring
4/2/2020	FP	3M	4	2020	Year-3	1	0	Spring
4/2/2020	FP	4M	4	2020	Year-3	1	0	Spring
4/2/2020	FP	5M	4	2020	Year-3	1	0	Spring
4/2/2020	FP	6M	4	2020	Year-3	0	0	Spring
4/2/2020	FP	7M	4	2020	Year-3		1	Spring
4/2/2020	FP	10M	4	2020	Year-3	1	0	Spring
4/2/2020	FP	12M	4	2020	Year-3	1	0	Spring
4/2/2020	FP	14M	4	2020	Year-3	1	0	Spring
4/2/2020	FP	8A	4	2020	Year-3		1	Spring
4/2/2020	FP	9A	4	2020	Year-3		1	Spring
4/2/2020	FP	11A	4	2020	Year-3		1	Spring
4/2/2020	FP	13A	4	2020	Year-3		1	Spring
4/3/2020	FP	1M	4	2020	Year-3	1	0	Spring
4/3/2020	FP	2M	4	2020	Year-3	1	0	Spring
4/3/2020	FP	3M	4	2020	Year-3	0	0	Spring
4/3/2020	FP	4M	4	2020	Year-3	1	0	Spring
4/3/2020	FP	5M	4	2020	Year-3	1	0	Spring
4/3/2020	FP	6M	4	2020	Year-3	1	0	Spring
4/3/2020	FP	7M	4	2020	Year-3		1	Spring
4/3/2020	FP	10M	4	2020	Year-3	1	0	Spring
4/3/2020	FP	12M	4	2020	Year-3	1	0	Spring
4/3/2020	FP	14M	4	2020	Year-3	1	0	Spring
4/3/2020	FP	8A	4	2020	Year-3		1	Spring
4/3/2020	FP	9A	4	2020	Year-3		1	Spring
4/3/2020	FP	11A	4	2020	Year-3		1	Spring
4/3/2020	FP	13A	4	2020	Year-3		1	Spring
4/4/2020	FP	1M	4	2020	Year-3	0	0	Spring
4/4/2020	FP	2M	4	2020	Year-3	1	0	Spring
4/4/2020	FP	3M	4	2020	Year-3	0	0	Spring
4/4/2020	FP	4M	4	2020	Year-3	0	0	Spring
4/4/2020	FP	5M	4	2020	Year-3	0	0	Spring
4/4/2020	FP	6M	4	2020	Year-3	0	0	Spring
4/4/2020	FP	7M	4	2020	Year-3		1	Spring
4/4/2020	FP	10M	4	2020	Year-3	0	0	Spring
4/4/2020	FP	12M	4	2020	Year-3	0	0	Spring
4/4/2020	FP	14M	4	2020	Year-3	0	0	Spring
4/4/2020	FP	8A	4	2020	Year-3		1	Spring
4/4/2020	FP	9A	4	2020	Year-3		1	Spring
4/4/2020	FP	11A	4	2020	Year-3		1	Spring
4/4/2020	FP	13A	4	2020	Year-3		1	Spring
4/5/2020	FP	1M	4	2020	Year-3	0	0	Spring

Date	Species	Site	Month	Year	Survey Year	Presence	Data Gap	Season
4/5/2020	FP	2M	4	2020	Year-3	0	0	Spring
4/5/2020	FP	3M	4	2020	Year-3	0	0	Spring
4/5/2020	FP	4M	4	2020	Year-3	0	0	Spring
4/5/2020	FP	5M	4	2020	Year-3	0	0	Spring
4/5/2020	FP	6M	4	2020	Year-3	1	0	Spring
4/5/2020	FP	7M	4	2020	Year-3		1	Spring
4/5/2020	FP	10M	4	2020	Year-3	0	0	Spring
4/5/2020	FP	12M	4	2020	Year-3	0	0	Spring
4/5/2020	FP	14M	4	2020	Year-3	0	0	Spring
4/5/2020	FP	8A	4	2020	Year-3		1	Spring
4/5/2020	FP	9A	4	2020	Year-3		1	Spring
4/5/2020	FP	11A	4	2020	Year-3		1	Spring
4/5/2020	FP	13A	4	2020	Year-3		1	Spring
4/6/2020	FP	1M	4	2020	Year-3	1	0	Spring
4/6/2020	FP	2M	4	2020	Year-3	1	0	Spring
4/6/2020	FP	3M	4	2020	Year-3	1	0	Spring
4/6/2020	FP	4M	4	2020	Year-3	1	0	Spring
4/6/2020	FP	5M	4	2020	Year-3	0	0	Spring
4/6/2020	FP	6M	4	2020	Year-3	0	0	Spring
4/6/2020	FP	7M	4	2020	Year-3		1	Spring
4/6/2020	FP	10M	4	2020	Year-3	0	0	Spring
4/6/2020	FP	12M	4	2020	Year-3	0	0	Spring
4/6/2020	FP	14M	4	2020	Year-3	1	0	Spring
4/6/2020	FP	8A	4	2020	Year-3		1	Spring
4/6/2020	FP	9A	4	2020	Year-3		1	Spring
4/6/2020	FP	11A	4	2020	Year-3		1	Spring
4/6/2020	FP	13A	4	2020	Year-3		1	Spring
4/7/2020	FP	1M	4	2020	Year-3	0	0	Spring
4/7/2020	FP	2M	4	2020	Year-3	1	0	Spring
4/7/2020	FP	3M	4	2020	Year-3	1	0	Spring
4/7/2020	FP	4M	4	2020	Year-3	0	0	Spring
4/7/2020	FP	5M	4	2020	Year-3	0	0	Spring
4/7/2020	FP	6M	4	2020	Year-3	0	0	Spring
4/7/2020	FP	7M	4	2020	Year-3		1	Spring
4/7/2020	FP	10M	4	2020	Year-3	0	0	Spring
4/7/2020	FP	12M	4	2020	Year-3	0	0	Spring
4/7/2020	FP	14M	4	2020	Year-3	1	0	Spring
4/7/2020	FP	8A	4	2020	Year-3		1	Spring
4/7/2020	FP	9A	4	2020	Year-3		1	Spring
4/7/2020	FP	11A	4	2020	Year-3		1	Spring
4/7/2020	FP	13A	4	2020	Year-3		1	Spring
4/8/2020	FP	1M	4	2020	Year-3	0	0	Spring
4/8/2020	FP	2M	4	2020	Year-3	0	0	Spring
4/8/2020	FP	3M	4	2020	Year-3	0	0	Spring

Date	Species	Site	Month	Year	Survey Year	Presence	Data Gap	Season
4/8/2020	FP	4M	4	2020	Year-3	0	0	Spring
4/8/2020	FP	5M	4	2020	Year-3	1	0	Spring
4/8/2020	FP	6M	4	2020	Year-3	0	0	Spring
4/8/2020	FP	7M	4	2020	Year-3		1	Spring
4/8/2020	FP	10M	4	2020	Year-3	0	0	Spring
4/8/2020	FP	12M	4	2020	Year-3	1	0	Spring
4/8/2020	FP	14M	4	2020	Year-3	1	0	Spring
4/8/2020	FP	8A	4	2020	Year-3		1	Spring
4/8/2020	FP	9A	4	2020	Year-3		1	Spring
4/8/2020	FP	11A	4	2020	Year-3		1	Spring
4/8/2020	FP	13A	4	2020	Year-3		1	Spring
4/9/2020	FP	1M	4	2020	Year-3	1	0	Spring
4/9/2020	FP	2M	4	2020	Year-3	1	0	Spring
4/9/2020	FP	3M	4	2020	Year-3	1	0	Spring
4/9/2020	FP	4M	4	2020	Year-3	0	0	Spring
4/9/2020	FP	5M	4	2020	Year-3	0	0	Spring
4/9/2020	FP	6M	4	2020	Year-3	0	0	Spring
4/9/2020	FP	7M	4	2020	Year-3		1	Spring
4/9/2020	FP	10M	4	2020	Year-3	0	0	Spring
4/9/2020	FP	12M	4	2020	Year-3	1	0	Spring
4/9/2020	FP	14M	4	2020	Year-3	1	0	Spring
4/9/2020	FP	8A	4	2020	Year-3		1	Spring
4/9/2020	FP	9A	4	2020	Year-3		1	Spring
4/9/2020	FP	11A	4	2020	Year-3		1	Spring
4/9/2020	FP	13A	4	2020	Year-3		1	Spring
4/10/2020	FP	1M	4	2020	Year-3	1	0	Spring
4/10/2020	FP	2M	4	2020	Year-3	1	0	Spring
4/10/2020	FP	3M	4	2020	Year-3	1	0	Spring
4/10/2020	FP	4M	4	2020	Year-3	0	0	Spring
4/10/2020	FP	5M	4	2020	Year-3	1	0	Spring
4/10/2020	FP	6M	4	2020	Year-3	0	0	Spring
4/10/2020	FP	7M	4	2020	Year-3		1	Spring
4/10/2020	FP	10M	4	2020	Year-3	0	0	Spring
4/10/2020	FP	12M	4	2020	Year-3	1	0	Spring
4/10/2020	FP	14M	4	2020	Year-3	1	0	Spring
4/10/2020	FP	8A	4	2020	Year-3		1	Spring
4/10/2020	FP	9A	4	2020	Year-3		1	Spring
4/10/2020	FP	11A	4	2020	Year-3		1	Spring
4/10/2020	FP	13A	4	2020	Year-3		1	Spring
4/11/2020	FP	1M	4	2020	Year-3	1	0	Spring
4/11/2020	FP	2M	4	2020	Year-3	1	0	Spring
4/11/2020	FP	3M	4	2020	Year-3	1	0	Spring
4/11/2020	FP	4M	4	2020	Year-3	1	0	Spring
4/11/2020	FP	5M	4	2020	Year-3	0	0	Spring

Date	Species	Site	Month	Year	Survey Year	Presence	Data Gap	Season
4/11/2020	FP	6M	4	2020	Year-3	0	0	Spring
4/11/2020	FP	7M	4	2020	Year-3		1	Spring
4/11/2020	FP	10M	4	2020	Year-3	0	0	Spring
4/11/2020	FP	12M	4	2020	Year-3	1	0	Spring
4/11/2020	FP	14M	4	2020	Year-3	1	0	Spring
4/11/2020	FP	8A	4	2020	Year-3		1	Spring
4/11/2020	FP	9A	4	2020	Year-3		1	Spring
4/11/2020	FP	11A	4	2020	Year-3		1	Spring
4/11/2020	FP	13A	4	2020	Year-3		1	Spring
4/12/2020	FP	1M	4	2020	Year-3	1	0	Spring
4/12/2020	FP	2M	4	2020	Year-3	1	0	Spring
4/12/2020	FP	3M	4	2020	Year-3	1	0	Spring
4/12/2020	FP	4M	4	2020	Year-3	1	0	Spring
4/12/2020	FP	5M	4	2020	Year-3	0	0	Spring
4/12/2020	FP	6M	4	2020	Year-3	0	0	Spring
4/12/2020	FP	7M	4	2020	Year-3		1	Spring
4/12/2020	FP	10M	4	2020	Year-3	1	0	Spring
4/12/2020	FP	12M	4	2020	Year-3	1	0	Spring
4/12/2020	FP	14M	4	2020	Year-3	1	0	Spring
4/12/2020	FP	8A	4	2020	Year-3		1	Spring
4/12/2020	FP	9A	4	2020	Year-3		1	Spring
4/12/2020	FP	11A	4	2020	Year-3		1	Spring
4/12/2020	FP	13A	4	2020	Year-3		1	Spring
4/13/2020	FP	1M	4	2020	Year-3	1	0	Spring
4/13/2020	FP	2M	4	2020	Year-3	1	0	Spring
4/13/2020	FP	3M	4	2020	Year-3	1	0	Spring
4/13/2020	FP	4M	4	2020	Year-3	1	0	Spring
4/13/2020	FP	5M	4	2020	Year-3	0	0	Spring
4/13/2020	FP	6M	4	2020	Year-3	0	0	Spring
4/13/2020	FP	7M	4	2020	Year-3		1	Spring
4/13/2020	FP	10M	4	2020	Year-3	1	0	Spring
4/13/2020	FP	12M	4	2020	Year-3	0	0	Spring
4/13/2020	FP	14M	4	2020	Year-3	1	0	Spring
4/13/2020	FP	8A	4	2020	Year-3		1	Spring
4/13/2020	FP	9A	4	2020	Year-3		1	Spring
4/13/2020	FP	11A	4	2020	Year-3		1	Spring
4/13/2020	FP	13A	4	2020	Year-3		1	Spring
4/14/2020	FP	1M	4	2020	Year-3	1	0	Spring
4/14/2020	FP	2M	4	2020	Year-3	1	0	Spring
4/14/2020	FP	3M	4	2020	Year-3	1	0	Spring
4/14/2020	FP	4M	4	2020	Year-3	1	0	Spring
4/14/2020	FP	5M	4	2020	Year-3	1	0	Spring
4/14/2020	FP	6M	4	2020	Year-3	0	0	Spring
4/14/2020	FP	7M	4	2020	Year-3		1	Spring

Date	Species	Site	Month	Year	Survey Year	Presence	Data Gap	Season
4/14/2020	FP	10M	4	2020	Year-3	1	0	Spring
4/14/2020	FP	12M	4	2020	Year-3	1	0	Spring
4/14/2020	FP	14M	4	2020	Year-3	1	0	Spring
4/14/2020	FP	8A	4	2020	Year-3		1	Spring
4/14/2020	FP	9A	4	2020	Year-3		1	Spring
4/14/2020	FP	11A	4	2020	Year-3		1	Spring
4/14/2020	FP	13A	4	2020	Year-3		1	Spring
4/15/2020	FP	1M	4	2020	Year-3	1	0	Spring
4/15/2020	FP	2M	4	2020	Year-3	1	0	Spring
4/15/2020	FP	3M	4	2020	Year-3	1	0	Spring
4/15/2020	FP	4M	4	2020	Year-3	1	0	Spring
4/15/2020	FP	5M	4	2020	Year-3	0	0	Spring
4/15/2020	FP	6M	4	2020	Year-3	1	0	Spring
4/15/2020	FP	7M	4	2020	Year-3		1	Spring
4/15/2020	FP	10M	4	2020	Year-3	1	0	Spring
4/15/2020	FP	12M	4	2020	Year-3	0	0	Spring
4/15/2020	FP	14M	4	2020	Year-3	1	0	Spring
4/15/2020	FP	8A	4	2020	Year-3		1	Spring
4/15/2020	FP	9A	4	2020	Year-3		1	Spring
4/15/2020	FP	11A	4	2020	Year-3		1	Spring
4/15/2020	FP	13A	4	2020	Year-3		1	Spring
4/16/2020	FP	1M	4	2020	Year-3	1	0	Spring
4/16/2020	FP	2M	4	2020	Year-3	1	0	Spring
4/16/2020	FP	3M	4	2020	Year-3	1	0	Spring
4/16/2020	FP	4M	4	2020	Year-3	1	0	Spring
4/16/2020	FP	5M	4	2020	Year-3	0	0	Spring
4/16/2020	FP	6M	4	2020	Year-3	0	0	Spring
4/16/2020	FP	7M	4	2020	Year-3		1	Spring
4/16/2020	FP	10M	4	2020	Year-3	1	0	Spring
4/16/2020	FP	12M	4	2020	Year-3	1	0	Spring
4/16/2020	FP	14M	4	2020	Year-3	1	0	Spring
4/16/2020	FP	8A	4	2020	Year-3		1	Spring
4/16/2020	FP	9A	4	2020	Year-3		1	Spring
4/16/2020	FP	11A	4	2020	Year-3		1	Spring
4/16/2020	FP	13A	4	2020	Year-3		1	Spring
4/17/2020	FP	1M	4	2020	Year-3	1	0	Spring
4/17/2020	FP	2M	4	2020	Year-3	1	0	Spring
4/17/2020	FP	3M	4	2020	Year-3	1	0	Spring
4/17/2020	FP	4M	4	2020	Year-3	1	0	Spring
4/17/2020	FP	5M	4	2020	Year-3	0	0	Spring
4/17/2020	FP	6M	4	2020	Year-3	0	0	Spring
4/17/2020	FP	7M	4	2020	Year-3		1	Spring
4/17/2020	FP	10M	4	2020	Year-3	0	0	Spring
4/17/2020	FP	12M	4	2020	Year-3	1	0	Spring

Date	Species	Site	Month	Year	Survey Year	Presence	Data Gap	Season
4/17/2020	FP	14M	4	2020	Year-3	1	0	Spring
4/17/2020	FP	8A	4	2020	Year-3		1	Spring
4/17/2020	FP	9A	4	2020	Year-3		1	Spring
4/17/2020	FP	11A	4	2020	Year-3		1	Spring
4/17/2020	FP	13A	4	2020	Year-3		1	Spring
4/18/2020	FP	1M	4	2020	Year-3	1	0	Spring
4/18/2020	FP	2M	4	2020	Year-3	1	0	Spring
4/18/2020	FP	3M	4	2020	Year-3	1	0	Spring
4/18/2020	FP	4M	4	2020	Year-3	1	0	Spring
4/18/2020	FP	5M	4	2020	Year-3	1	0	Spring
4/18/2020	FP	6M	4	2020	Year-3	1	0	Spring
4/18/2020	FP	7M	4	2020	Year-3		1	Spring
4/18/2020	FP	10M	4	2020	Year-3	1	0	Spring
4/18/2020	FP	12M	4	2020	Year-3	1	0	Spring
4/18/2020	FP	14M	4	2020	Year-3	1	0	Spring
4/18/2020	FP	8A	4	2020	Year-3		1	Spring
4/18/2020	FP	9A	4	2020	Year-3		1	Spring
4/18/2020	FP	11A	4	2020	Year-3		1	Spring
4/18/2020	FP	13A	4	2020	Year-3		1	Spring
4/19/2020	FP	1M	4	2020	Year-3	1	0	Spring
4/19/2020	FP	2M	4	2020	Year-3	1	0	Spring
4/19/2020	FP	3M	4	2020	Year-3	1	0	Spring
4/19/2020	FP	4M	4	2020	Year-3	0	0	Spring
4/19/2020	FP	5M	4	2020	Year-3	0	0	Spring
4/19/2020	FP	6M	4	2020	Year-3	0	0	Spring
4/19/2020	FP	7M	4	2020	Year-3		1	Spring
4/19/2020	FP	10M	4	2020	Year-3	1	0	Spring
4/19/2020	FP	12M	4	2020	Year-3	1	0	Spring
4/19/2020	FP	14M	4	2020	Year-3	1	0	Spring
4/19/2020	FP	8A	4	2020	Year-3		1	Spring
4/19/2020	FP	9A	4	2020	Year-3		1	Spring
4/19/2020	FP	11A	4	2020	Year-3		1	Spring
4/19/2020	FP	13A	4	2020	Year-3		1	Spring
4/20/2020	FP	1M	4	2020	Year-3	1	0	Spring
4/20/2020	FP	2M	4	2020	Year-3	1	0	Spring
4/20/2020	FP	3M	4	2020	Year-3	1	0	Spring
4/20/2020	FP	4M	4	2020	Year-3	1	0	Spring
4/20/2020	FP	5M	4	2020	Year-3	0	0	Spring
4/20/2020	FP	6M	4	2020	Year-3	0	0	Spring
4/20/2020	FP	7M	4	2020	Year-3		1	Spring
4/20/2020	FP	10M	4	2020	Year-3	0	0	Spring
4/20/2020	FP	12M	4	2020	Year-3	0	0	Spring
4/20/2020	FP	14M	4	2020	Year-3	1	0	Spring
4/20/2020	FP	8A	4	2020	Year-3		1	Spring

Date	Species	Site	Month	Year	Survey Year	Presence	Data Gap	Season
4/20/2020	FP	9A	4	2020	Year-3		1	Spring
4/20/2020	FP	11A	4	2020	Year-3		1	Spring
4/20/2020	FP	13A	4	2020	Year-3		1	Spring
4/21/2020	FP	1M	4	2020	Year-3	1	0	Spring
4/21/2020	FP	2M	4	2020	Year-3	1	0	Spring
4/21/2020	FP	3M	4	2020	Year-3	1	0	Spring
4/21/2020	FP	4M	4	2020	Year-3	1	0	Spring
4/21/2020	FP	5M	4	2020	Year-3	0	0	Spring
4/21/2020	FP	6M	4	2020	Year-3	0	0	Spring
4/21/2020	FP	7M	4	2020	Year-3		1	Spring
4/21/2020	FP	10M	4	2020	Year-3	0	0	Spring
4/21/2020	FP	12M	4	2020	Year-3	1	0	Spring
4/21/2020	FP	14M	4	2020	Year-3	1	0	Spring
4/21/2020	FP	8A	4	2020	Year-3		1	Spring
4/21/2020	FP	9A	4	2020	Year-3		1	Spring
4/21/2020	FP	11A	4	2020	Year-3		1	Spring
4/21/2020	FP	13A	4	2020	Year-3		1	Spring
4/22/2020	FP	1M	4	2020	Year-3	1	0	Spring
4/22/2020	FP	2M	4	2020	Year-3	1	0	Spring
4/22/2020	FP	3M	4	2020	Year-3	1	0	Spring
4/22/2020	FP	4M	4	2020	Year-3	0	0	Spring
4/22/2020	FP	5M	4	2020	Year-3	0	0	Spring
4/22/2020	FP	6M	4	2020	Year-3	0	0	Spring
4/22/2020	FP	7M	4	2020	Year-3		1	Spring
4/22/2020	FP	10M	4	2020	Year-3	1	0	Spring
4/22/2020	FP	12M	4	2020	Year-3	1	0	Spring
4/22/2020	FP	14M	4	2020	Year-3	1	0	Spring
4/22/2020	FP	8A	4	2020	Year-3		1	Spring
4/22/2020	FP	9A	4	2020	Year-3		1	Spring
4/22/2020	FP	11A	4	2020	Year-3		1	Spring
4/22/2020	FP	13A	4	2020	Year-3		1	Spring
4/23/2020	FP	1M	4	2020	Year-3	1	0	Spring
4/23/2020	FP	2M	4	2020	Year-3	1	0	Spring
4/23/2020	FP	3M	4	2020	Year-3	1	0	Spring
4/23/2020	FP	4M	4	2020	Year-3	1	0	Spring
4/23/2020	FP	5M	4	2020	Year-3	0	0	Spring
4/23/2020	FP	6M	4	2020	Year-3	0	0	Spring
4/23/2020	FP	7M	4	2020	Year-3		1	Spring
4/23/2020	FP	10M	4	2020	Year-3	0	0	Spring
4/23/2020	FP	12M	4	2020	Year-3	1	0	Spring
4/23/2020	FP	14M	4	2020	Year-3	1	0	Spring
4/23/2020	FP	8A	4	2020	Year-3		1	Spring
4/23/2020	FP	9A	4	2020	Year-3		1	Spring
4/23/2020	FP	11A	4	2020	Year-3		1	Spring

Date	Species	Site	Month	Year	Survey Year	Presence	Data Gap	Season
4/23/2020	FP	13A	4	2020	Year-3		1	Spring
4/24/2020	FP	1M	4	2020	Year-3	1	0	Spring
4/24/2020	FP	2M	4	2020	Year-3	1	0	Spring
4/24/2020	FP	3M	4	2020	Year-3	1	0	Spring
4/24/2020	FP	4M	4	2020	Year-3	1	0	Spring
4/24/2020	FP	5M	4	2020	Year-3	0	0	Spring
4/24/2020	FP	6M	4	2020	Year-3	0	0	Spring
4/24/2020	FP	7M	4	2020	Year-3		1	Spring
4/24/2020	FP	10M	4	2020	Year-3	0	0	Spring
4/24/2020	FP	12M	4	2020	Year-3	1	0	Spring
4/24/2020	FP	14M	4	2020	Year-3	1	0	Spring
4/24/2020	FP	8A	4	2020	Year-3		1	Spring
4/24/2020	FP	9A	4	2020	Year-3		1	Spring
4/24/2020	FP	11A	4	2020	Year-3		1	Spring
4/24/2020	FP	13A	4	2020	Year-3		1	Spring
4/25/2020	FP	1M	4	2020	Year-3	1	0	Spring
4/25/2020	FP	2M	4	2020	Year-3	1	0	Spring
4/25/2020	FP	3M	4	2020	Year-3	1	0	Spring
4/25/2020	FP	4M	4	2020	Year-3	1	0	Spring
4/25/2020	FP	5M	4	2020	Year-3	0	0	Spring
4/25/2020	FP	6M	4	2020	Year-3	0	0	Spring
4/25/2020	FP	7M	4	2020	Year-3		1	Spring
4/25/2020	FP	10M	4	2020	Year-3	0	0	Spring
4/25/2020	FP	12M	4	2020	Year-3	1	0	Spring
4/25/2020	FP	14M	4	2020	Year-3	1	0	Spring
4/25/2020	FP	8A	4	2020	Year-3		1	Spring
4/25/2020	FP	9A	4	2020	Year-3		1	Spring
4/25/2020	FP	11A	4	2020	Year-3		1	Spring
4/25/2020	FP	13A	4	2020	Year-3		1	Spring
4/26/2020	FP	1M	4	2020	Year-3	1	0	Spring
4/26/2020	FP	2M	4	2020	Year-3	1	0	Spring
4/26/2020	FP	3M	4	2020	Year-3	1	0	Spring
4/26/2020	FP	4M	4	2020	Year-3	1	0	Spring
4/26/2020	FP	5M	4	2020	Year-3	0	0	Spring
4/26/2020	FP	6M	4	2020	Year-3	0	0	Spring
4/26/2020	FP	7M	4	2020	Year-3		1	Spring
4/26/2020	FP	10M	4	2020	Year-3	0	0	Spring
4/26/2020	FP	12M	4	2020	Year-3	0	0	Spring
4/26/2020	FP	14M	4	2020	Year-3	1	0	Spring
4/26/2020	FP	8A	4	2020	Year-3		1	Spring
4/26/2020	FP	9A	4	2020	Year-3		1	Spring
4/26/2020	FP	11A	4	2020	Year-3		1	Spring
4/26/2020	FP	13A	4	2020	Year-3		1	Spring
4/27/2020	FP	1M	4	2020	Year-3	1	0	Spring

Date	Species	Site	Month	Year	Survey Year	Presence	Data Gap	Season
4/27/2020	FP	2M	4	2020	Year-3	1	0	Spring
4/27/2020	FP	3M	4	2020	Year-3	1	0	Spring
4/27/2020	FP	4M	4	2020	Year-3	1	0	Spring
4/27/2020	FP	5M	4	2020	Year-3	0	0	Spring
4/27/2020	FP	6M	4	2020	Year-3	0	0	Spring
4/27/2020	FP	7M	4	2020	Year-3		1	Spring
4/27/2020	FP	10M	4	2020	Year-3	0	0	Spring
4/27/2020	FP	12M	4	2020	Year-3	0	0	Spring
4/27/2020	FP	14M	4	2020	Year-3	1	0	Spring
4/27/2020	FP	8A	4	2020	Year-3		1	Spring
4/27/2020	FP	9A	4	2020	Year-3		1	Spring
4/27/2020	FP	11A	4	2020	Year-3		1	Spring
4/27/2020	FP	13A	4	2020	Year-3		1	Spring
4/28/2020	FP	1M	4	2020	Year-3	1	0	Spring
4/28/2020	FP	2M	4	2020	Year-3	1	0	Spring
4/28/2020	FP	3M	4	2020	Year-3	1	0	Spring
4/28/2020	FP	4M	4	2020	Year-3	1	0	Spring
4/28/2020	FP	5M	4	2020	Year-3	0	0	Spring
4/28/2020	FP	6M	4	2020	Year-3	0	0	Spring
4/28/2020	FP	7M	4	2020	Year-3		1	Spring
4/28/2020	FP	10M	4	2020	Year-3	0	0	Spring
4/28/2020	FP	12M	4	2020	Year-3	0	0	Spring
4/28/2020	FP	14M	4	2020	Year-3	1	0	Spring
4/28/2020	FP	8A	4	2020	Year-3		1	Spring
4/28/2020	FP	9A	4	2020	Year-3		1	Spring
4/28/2020	FP	11A	4	2020	Year-3		1	Spring
4/28/2020	FP	13A	4	2020	Year-3		1	Spring
4/29/2020	FP	1M	4	2020	Year-3	1	0	Spring
4/29/2020	FP	2M	4	2020	Year-3	1	0	Spring
4/29/2020	FP	3M	4	2020	Year-3	1	0	Spring
4/29/2020	FP	4M	4	2020	Year-3	1	0	Spring
4/29/2020	FP	5M	4	2020	Year-3	0	0	Spring
4/29/2020	FP	6M	4	2020	Year-3	0	0	Spring
4/29/2020	FP	7M	4	2020	Year-3		1	Spring
4/29/2020	FP	10M	4	2020	Year-3	0	0	Spring
4/29/2020	FP	12M	4	2020	Year-3	1	0	Spring
4/29/2020	FP	14M	4	2020	Year-3	1	0	Spring
4/29/2020	FP	8A	4	2020	Year-3		1	Spring
4/29/2020	FP	9A	4	2020	Year-3		1	Spring
4/29/2020	FP	11A	4	2020	Year-3		1	Spring
4/29/2020	FP	13A	4	2020	Year-3		1	Spring
4/30/2020	FP	1M	4	2020	Year-3	1	0	Spring
4/30/2020	FP	2M	4	2020	Year-3	1	0	Spring
4/30/2020	FP	3M	4	2020	Year-3	1	0	Spring

Date	Species	Site	Month	Year	Survey Year	Presence	Data Gap	Season
4/30/2020	FP	4M	4	2020	Year-3	1	0	Spring
4/30/2020	FP	5M	4	2020	Year-3	0	0	Spring
4/30/2020	FP	6M	4	2020	Year-3	0	0	Spring
4/30/2020	FP	7M	4	2020	Year-3		1	Spring
4/30/2020	FP	10M	4	2020	Year-3	0	0	Spring
4/30/2020	FP	12M	4	2020	Year-3	0	0	Spring
4/30/2020	FP	14M	4	2020	Year-3	1	0	Spring
4/30/2020	FP	8A	4	2020	Year-3		1	Spring
4/30/2020	FP	9A	4	2020	Year-3		1	Spring
4/30/2020	FP	11A	4	2020	Year-3		1	Spring
4/30/2020	FP	13A	4	2020	Year-3		1	Spring
5/1/2020	FP	1M	5	2020	Year-3	1	0	Spring
5/1/2020	FP	2M	5	2020	Year-3	1	0	Spring
5/1/2020	FP	3M	5	2020	Year-3	1	0	Spring
5/1/2020	FP	4M	5	2020	Year-3	0	0	Spring
5/1/2020	FP	5M	5	2020	Year-3	0	0	Spring
5/1/2020	FP	6M	5	2020	Year-3	0	0	Spring
5/1/2020	FP	7M	5	2020	Year-3		1	Spring
5/1/2020	FP	10M	5	2020	Year-3	0	0	Spring
5/1/2020	FP	12M	5	2020	Year-3	0	0	Spring
5/1/2020	FP	14M	5	2020	Year-3	1	0	Spring
5/1/2020	FP	8A	5	2020	Year-3		1	Spring
5/1/2020	FP	9A	5	2020	Year-3		1	Spring
5/1/2020	FP	11A	5	2020	Year-3		1	Spring
5/1/2020	FP	13A	5	2020	Year-3		1	Spring
5/2/2020	FP	1M	5	2020	Year-3	1	0	Spring
5/2/2020	FP	2M	5	2020	Year-3	1	0	Spring
5/2/2020	FP	3M	5	2020	Year-3	1	0	Spring
5/2/2020	FP	4M	5	2020	Year-3	1	0	Spring
5/2/2020	FP	5M	5	2020	Year-3	0	0	Spring
5/2/2020	FP	6M	5	2020	Year-3	0	0	Spring
5/2/2020	FP	7M	5	2020	Year-3		1	Spring
5/2/2020	FP	10M	5	2020	Year-3	0	0	Spring
5/2/2020	FP	12M	5	2020	Year-3	0	0	Spring
5/2/2020	FP	14M	5	2020	Year-3	1	0	Spring
5/2/2020	FP	8A	5	2020	Year-3		1	Spring
5/2/2020	FP	9A	5	2020	Year-3		1	Spring
5/2/2020	FP	11A	5	2020	Year-3		1	Spring
5/2/2020	FP	13A	5	2020	Year-3		1	Spring
5/3/2020	FP	1M	5	2020	Year-3	1	0	Spring
5/3/2020	FP	2M	5	2020	Year-3	1	0	Spring
5/3/2020	FP	3M	5	2020	Year-3	1	0	Spring
5/3/2020	FP	4M	5	2020	Year-3	1	0	Spring
5/3/2020	FP	5M	5	2020	Year-3	0	0	Spring

Date	Species	Site	Month	Year	Survey Year	Presence	Data Gap	Season
5/3/2020	FP	6M	5	2020	Year-3	0	0	Spring
5/3/2020	FP	7M	5	2020	Year-3		1	Spring
5/3/2020	FP	10M	5	2020	Year-3	0	0	Spring
5/3/2020	FP	12M	5	2020	Year-3	0	0	Spring
5/3/2020	FP	14M	5	2020	Year-3	0	0	Spring
5/3/2020	FP	8A	5	2020	Year-3		1	Spring
5/3/2020	FP	9A	5	2020	Year-3		1	Spring
5/3/2020	FP	11A	5	2020	Year-3		1	Spring
5/3/2020	FP	13A	5	2020	Year-3		1	Spring
5/4/2020	FP	1M	5	2020	Year-3	1	0	Spring
5/4/2020	FP	2M	5	2020	Year-3	1	0	Spring
5/4/2020	FP	3M	5	2020	Year-3	1	0	Spring
5/4/2020	FP	4M	5	2020	Year-3	0	0	Spring
5/4/2020	FP	5M	5	2020	Year-3	0	0	Spring
5/4/2020	FP	6M	5	2020	Year-3	0	0	Spring
5/4/2020	FP	7M	5	2020	Year-3		1	Spring
5/4/2020	FP	10M	5	2020	Year-3	0	0	Spring
5/4/2020	FP	12M	5	2020	Year-3	0	0	Spring
5/4/2020	FP	14M	5	2020	Year-3	0	0	Spring
5/4/2020	FP	8A	5	2020	Year-3		1	Spring
5/4/2020	FP	9A	5	2020	Year-3		1	Spring
5/4/2020	FP	11A	5	2020	Year-3		1	Spring
5/4/2020	FP	13A	5	2020	Year-3		1	Spring
5/5/2020	FP	1M	5	2020	Year-3	1	0	Spring
5/5/2020	FP	2M	5	2020	Year-3	1	0	Spring
5/5/2020	FP	3M	5	2020	Year-3	1	0	Spring
5/5/2020	FP	4M	5	2020	Year-3	1	0	Spring
5/5/2020	FP	5M	5	2020	Year-3	0	0	Spring
5/5/2020	FP	6M	5	2020	Year-3	0	0	Spring
5/5/2020	FP	7M	5	2020	Year-3		1	Spring
5/5/2020	FP	10M	5	2020	Year-3	0	0	Spring
5/5/2020	FP	12M	5	2020	Year-3	0	0	Spring
5/5/2020	FP	14M	5	2020	Year-3	0	0	Spring
5/5/2020	FP	8A	5	2020	Year-3		1	Spring
5/5/2020	FP	9A	5	2020	Year-3		1	Spring
5/5/2020	FP	11A	5	2020	Year-3		1	Spring
5/5/2020	FP	13A	5	2020	Year-3		1	Spring
5/6/2020	FP	1M	5	2020	Year-3	1	0	Spring
5/6/2020	FP	2M	5	2020	Year-3	1	0	Spring
5/6/2020	FP	3M	5	2020	Year-3	1	0	Spring
5/6/2020	FP	4M	5	2020	Year-3	1	0	Spring
5/6/2020	FP	5M	5	2020	Year-3	0	0	Spring
5/6/2020	FP	6M	5	2020	Year-3	0	0	Spring
5/6/2020	FP	7M	5	2020	Year-3		1	Spring

Date	Species	Site	Month	Year	Survey Year	Presence	Data Gap	Season
5/6/2020	FP	10M	5	2020	Year-3	0	0	Spring
5/6/2020	FP	12M	5	2020	Year-3	0	0	Spring
5/6/2020	FP	14M	5	2020	Year-3	1	0	Spring
5/6/2020	FP	8A	5	2020	Year-3		1	Spring
5/6/2020	FP	9A	5	2020	Year-3		1	Spring
5/6/2020	FP	11A	5	2020	Year-3		1	Spring
5/6/2020	FP	13A	5	2020	Year-3		1	Spring
5/7/2020	FP	1M	5	2020	Year-3	1	0	Spring
5/7/2020	FP	2M	5	2020	Year-3	1	0	Spring
5/7/2020	FP	3M	5	2020	Year-3	1	0	Spring
5/7/2020	FP	4M	5	2020	Year-3	0	0	Spring
5/7/2020	FP	5M	5	2020	Year-3	0	0	Spring
5/7/2020	FP	6M	5	2020	Year-3	0	0	Spring
5/7/2020	FP	7M	5	2020	Year-3		1	Spring
5/7/2020	FP	10M	5	2020	Year-3	0	0	Spring
5/7/2020	FP	12M	5	2020	Year-3	0	0	Spring
5/7/2020	FP	14M	5	2020	Year-3	1	0	Spring
5/7/2020	FP	8A	5	2020	Year-3		1	Spring
5/7/2020	FP	9A	5	2020	Year-3		1	Spring
5/7/2020	FP	11A	5	2020	Year-3		1	Spring
5/7/2020	FP	13A	5	2020	Year-3		1	Spring
5/8/2020	FP	1M	5	2020	Year-3	1	0	Spring
5/8/2020	FP	2M	5	2020	Year-3	1	0	Spring
5/8/2020	FP	3M	5	2020	Year-3	1	0	Spring
5/8/2020	FP	4M	5	2020	Year-3	0	0	Spring
5/8/2020	FP	5M	5	2020	Year-3	0	0	Spring
5/8/2020	FP	6M	5	2020	Year-3	0	0	Spring
5/8/2020	FP	7M	5	2020	Year-3		1	Spring
5/8/2020	FP	10M	5	2020	Year-3	0	0	Spring
5/8/2020	FP	12M	5	2020	Year-3	0	0	Spring
5/8/2020	FP	14M	5	2020	Year-3	1	0	Spring
5/8/2020	FP	8A	5	2020	Year-3		1	Spring
5/8/2020	FP	9A	5	2020	Year-3		1	Spring
5/8/2020	FP	11A	5	2020	Year-3		1	Spring
5/8/2020	FP	13A	5	2020	Year-3		1	Spring
5/9/2020	FP	1M	5	2020	Year-3	1	0	Spring
5/9/2020	FP	2M	5	2020	Year-3	1	0	Spring
5/9/2020	FP	3M	5	2020	Year-3	0	0	Spring
5/9/2020	FP	4M	5	2020	Year-3	0	0	Spring
5/9/2020	FP	5M	5	2020	Year-3	0	0	Spring
5/9/2020	FP	6M	5	2020	Year-3	0	0	Spring
5/9/2020	FP	7M	5	2020	Year-3		1	Spring
5/9/2020	FP	10M	5	2020	Year-3	0	0	Spring
5/9/2020	FP	12M	5	2020	Year-3	0	0	Spring

Date	Species	Site	Month	Year	Survey Year	Presence	Data Gap	Season
5/9/2020	FP	14M	5	2020	Year-3	1	0	Spring
5/9/2020	FP	8A	5	2020	Year-3		1	Spring
5/9/2020	FP	9A	5	2020	Year-3		1	Spring
5/9/2020	FP	11A	5	2020	Year-3		1	Spring
5/9/2020	FP	13A	5	2020	Year-3		1	Spring
5/10/2020	FP	1M	5	2020	Year-3	0	0	Spring
5/10/2020	FP	2M	5	2020	Year-3	0	0	Spring
5/10/2020	FP	3M	5	2020	Year-3	1	0	Spring
5/10/2020	FP	4M	5	2020	Year-3	0	0	Spring
5/10/2020	FP	5M	5	2020	Year-3	0	0	Spring
5/10/2020	FP	6M	5	2020	Year-3	0	0	Spring
5/10/2020	FP	7M	5	2020	Year-3		1	Spring
5/10/2020	FP	10M	5	2020	Year-3	0	0	Spring
5/10/2020	FP	12M	5	2020	Year-3	0	0	Spring
5/10/2020	FP	14M	5	2020	Year-3	1	0	Spring
5/10/2020	FP	8A	5	2020	Year-3		1	Spring
5/10/2020	FP	9A	5	2020	Year-3		1	Spring
5/10/2020	FP	11A	5	2020	Year-3		1	Spring
5/10/2020	FP	13A	5	2020	Year-3		1	Spring
5/11/2020	FP	1M	5	2020	Year-3	1	0	Spring
5/11/2020	FP	2M	5	2020	Year-3	1	0	Spring
5/11/2020	FP	3M	5	2020	Year-3	1	0	Spring
5/11/2020	FP	4M	5	2020	Year-3	1	0	Spring
5/11/2020	FP	5M	5	2020	Year-3	0	0	Spring
5/11/2020	FP	6M	5	2020	Year-3	0	0	Spring
5/11/2020	FP	7M	5	2020	Year-3		1	Spring
5/11/2020	FP	10M	5	2020	Year-3	0	0	Spring
5/11/2020	FP	12M	5	2020	Year-3	0	0	Spring
5/11/2020	FP	14M	5	2020	Year-3	1	0	Spring
5/11/2020	FP	8A	5	2020	Year-3		1	Spring
5/11/2020	FP	9A	5	2020	Year-3		1	Spring
5/11/2020	FP	11A	5	2020	Year-3		1	Spring
5/11/2020	FP	13A	5	2020	Year-3		1	Spring
5/12/2020	FP	1M	5	2020	Year-3	1	0	Spring
5/12/2020	FP	2M	5	2020	Year-3	1	0	Spring
5/12/2020	FP	3M	5	2020	Year-3	0	0	Spring
5/12/2020	FP	4M	5	2020	Year-3	0	0	Spring
5/12/2020	FP	5M	5	2020	Year-3	0	0	Spring
5/12/2020	FP	6M	5	2020	Year-3	0	0	Spring
5/12/2020	FP	7M	5	2020	Year-3		1	Spring
5/12/2020	FP	10M	5	2020	Year-3	0	0	Spring
5/12/2020	FP	12M	5	2020	Year-3	0	0	Spring
5/12/2020	FP	14M	5	2020	Year-3	1	0	Spring
5/12/2020	FP	8A	5	2020	Year-3		1	Spring

Date	Species	Site	Month	Year	Survey Year	Presence	Data Gap	Season
5/12/2020	FP	9A	5	2020	Year-3		1	Spring
5/12/2020	FP	11A	5	2020	Year-3		1	Spring
5/12/2020	FP	13A	5	2020	Year-3		1	Spring
5/13/2020	FP	1M	5	2020	Year-3	1	0	Spring
5/13/2020	FP	2M	5	2020	Year-3	1	0	Spring
5/13/2020	FP	3M	5	2020	Year-3	1	0	Spring
5/13/2020	FP	4M	5	2020	Year-3	1	0	Spring
5/13/2020	FP	5M	5	2020	Year-3	0	0	Spring
5/13/2020	FP	6M	5	2020	Year-3	0	0	Spring
5/13/2020	FP	7M	5	2020	Year-3		1	Spring
5/13/2020	FP	10M	5	2020	Year-3	0	0	Spring
5/13/2020	FP	12M	5	2020	Year-3	1	0	Spring
5/13/2020	FP	14M	5	2020	Year-3	1	0	Spring
5/13/2020	FP	8A	5	2020	Year-3		1	Spring
5/13/2020	FP	9A	5	2020	Year-3		1	Spring
5/13/2020	FP	11A	5	2020	Year-3		1	Spring
5/13/2020	FP	13A	5	2020	Year-3		1	Spring
5/14/2020	FP	1M	5	2020	Year-3	1	0	Spring
5/14/2020	FP	2M	5	2020	Year-3	1	0	Spring
5/14/2020	FP	3M	5	2020	Year-3	1	0	Spring
5/14/2020	FP	4M	5	2020	Year-3		1	Spring
5/14/2020	FP	5M	5	2020	Year-3	0	0	Spring
5/14/2020	FP	6M	5	2020	Year-3	0	0	Spring
5/14/2020	FP	7M	5	2020	Year-3		1	Spring
5/14/2020	FP	10M	5	2020	Year-3	0	0	Spring
5/14/2020	FP	12M	5	2020	Year-3	0	0	Spring
5/14/2020	FP	14M	5	2020	Year-3	1	0	Spring
5/14/2020	FP	8A	5	2020	Year-3		1	Spring
5/14/2020	FP	9A	5	2020	Year-3		1	Spring
5/14/2020	FP	11A	5	2020	Year-3		1	Spring
5/14/2020	FP	13A	5	2020	Year-3		1	Spring
5/15/2020	FP	1M	5	2020	Year-3	1	0	Spring
5/15/2020	FP	2M	5	2020	Year-3	1	0	Spring
5/15/2020	FP	3M	5	2020	Year-3	1	0	Spring
5/15/2020	FP	4M	5	2020	Year-3		1	Spring
5/15/2020	FP	5M	5	2020	Year-3	0	0	Spring
5/15/2020	FP	6M	5	2020	Year-3	0	0	Spring
5/15/2020	FP	7M	5	2020	Year-3		1	Spring
5/15/2020	FP	10M	5	2020	Year-3	0	0	Spring
5/15/2020	FP	12M	5	2020	Year-3	0	0	Spring
5/15/2020	FP	14M	5	2020	Year-3	1	0	Spring
5/15/2020	FP	8A	5	2020	Year-3		1	Spring
5/15/2020	FP	9A	5	2020	Year-3		1	Spring
5/15/2020	FP	11A	5	2020	Year-3		1	Spring

Date	Species	Site	Month	Year	Survey Year	Presence	Data Gap	Season
5/15/2020	FP	13A	5	2020	Year-3		1	Spring
5/16/2020	FP	1M	5	2020	Year-3	1	0	Spring
5/16/2020	FP	2M	5	2020	Year-3	1	0	Spring
5/16/2020	FP	3M	5	2020	Year-3	1	0	Spring
5/16/2020	FP	4M	5	2020	Year-3		1	Spring
5/16/2020	FP	5M	5	2020	Year-3	0	0	Spring
5/16/2020	FP	6M	5	2020	Year-3	0	0	Spring
5/16/2020	FP	7M	5	2020	Year-3		1	Spring
5/16/2020	FP	10M	5	2020	Year-3	0	0	Spring
5/16/2020	FP	12M	5	2020	Year-3	0	0	Spring
5/16/2020	FP	14M	5	2020	Year-3	1	0	Spring
5/16/2020	FP	8A	5	2020	Year-3		1	Spring
5/16/2020	FP	9A	5	2020	Year-3		1	Spring
5/16/2020	FP	11A	5	2020	Year-3		1	Spring
5/16/2020	FP	13A	5	2020	Year-3		1	Spring
5/17/2020	FP	1M	5	2020	Year-3	1	0	Spring
5/17/2020	FP	2M	5	2020	Year-3	1	0	Spring
5/17/2020	FP	3M	5	2020	Year-3	0	0	Spring
5/17/2020	FP	4M	5	2020	Year-3		1	Spring
5/17/2020	FP	5M	5	2020	Year-3	0	0	Spring
5/17/2020	FP	6M	5	2020	Year-3	0	0	Spring
5/17/2020	FP	7M	5	2020	Year-3		1	Spring
5/17/2020	FP	10M	5	2020	Year-3	0	0	Spring
5/17/2020	FP	12M	5	2020	Year-3	0	0	Spring
5/17/2020	FP	14M	5	2020	Year-3	1	0	Spring
5/17/2020	FP	8A	5	2020	Year-3		1	Spring
5/17/2020	FP	9A	5	2020	Year-3		1	Spring
5/17/2020	FP	11A	5	2020	Year-3		1	Spring
5/17/2020	FP	13A	5	2020	Year-3		1	Spring
5/18/2020	FP	1M	5	2020	Year-3	1	0	Spring
5/18/2020	FP	2M	5	2020	Year-3	1	0	Spring
5/18/2020	FP	3M	5	2020	Year-3	0	0	Spring
5/18/2020	FP	4M	5	2020	Year-3		1	Spring
5/18/2020	FP	5M	5	2020	Year-3	0	0	Spring
5/18/2020	FP	6M	5	2020	Year-3	0	0	Spring
5/18/2020	FP	7M	5	2020	Year-3		1	Spring
5/18/2020	FP	10M	5	2020	Year-3	0	0	Spring
5/18/2020	FP	12M	5	2020	Year-3	1	0	Spring
5/18/2020	FP	14M	5	2020	Year-3	1	0	Spring
5/18/2020	FP	8A	5	2020	Year-3		1	Spring
5/18/2020	FP	9A	5	2020	Year-3		1	Spring
5/18/2020	FP	11A	5	2020	Year-3		1	Spring
5/18/2020	FP	13A	5	2020	Year-3		1	Spring
5/19/2020	FP	1M	5	2020	Year-3	1	0	Spring

Date	Species	Site	Month	Year	Survey Year	Presence	Data Gap	Season
5/19/2020	FP	2M	5	2020	Year-3	1	0	Spring
5/19/2020	FP	3M	5	2020	Year-3	1	0	Spring
5/19/2020	FP	4M	5	2020	Year-3		1	Spring
5/19/2020	FP	5M	5	2020	Year-3	0	0	Spring
5/19/2020	FP	6M	5	2020	Year-3	0	0	Spring
5/19/2020	FP	7M	5	2020	Year-3		1	Spring
5/19/2020	FP	10M	5	2020	Year-3	0	0	Spring
5/19/2020	FP	12M	5	2020	Year-3	1	0	Spring
5/19/2020	FP	14M	5	2020	Year-3	1	0	Spring
5/19/2020	FP	8A	5	2020	Year-3		1	Spring
5/19/2020	FP	9A	5	2020	Year-3		1	Spring
5/19/2020	FP	11A	5	2020	Year-3		1	Spring
5/19/2020	FP	13A	5	2020	Year-3		1	Spring
5/20/2020	FP	1M	5	2020	Year-3	0	0	Spring
5/20/2020	FP	2M	5	2020	Year-3	1	0	Spring
5/20/2020	FP	3M	5	2020	Year-3	0	0	Spring
5/20/2020	FP	4M	5	2020	Year-3		1	Spring
5/20/2020	FP	5M	5	2020	Year-3	0	0	Spring
5/20/2020	FP	6M	5	2020	Year-3	0	0	Spring
5/20/2020	FP	7M	5	2020	Year-3		1	Spring
5/20/2020	FP	10M	5	2020	Year-3	0	0	Spring
5/20/2020	FP	12M	5	2020	Year-3	0	0	Spring
5/20/2020	FP	14M	5	2020	Year-3	1	0	Spring
5/20/2020	FP	8A	5	2020	Year-3		1	Spring
5/20/2020	FP	9A	5	2020	Year-3		1	Spring
5/20/2020	FP	11A	5	2020	Year-3		1	Spring
5/20/2020	FP	13A	5	2020	Year-3		1	Spring
5/21/2020	FP	1M	5	2020	Year-3	1	0	Spring
5/21/2020	FP	2M	5	2020	Year-3	1	0	Spring
5/21/2020	FP	3M	5	2020	Year-3	1	0	Spring
5/21/2020	FP	4M	5	2020	Year-3		1	Spring
5/21/2020	FP	5M	5	2020	Year-3	0	0	Spring
5/21/2020	FP	6M	5	2020	Year-3	0	0	Spring
5/21/2020	FP	7M	5	2020	Year-3		1	Spring
5/21/2020	FP	10M	5	2020	Year-3	0	0	Spring
5/21/2020	FP	12M	5	2020	Year-3	0	0	Spring
5/21/2020	FP	14M	5	2020	Year-3	1	0	Spring
5/21/2020	FP	8A	5	2020	Year-3		1	Spring
5/21/2020	FP	9A	5	2020	Year-3		1	Spring
5/21/2020	FP	11A	5	2020	Year-3		1	Spring
5/21/2020	FP	13A	5	2020	Year-3		1	Spring
5/22/2020	FP	1M	5	2020	Year-3	0	0	Spring
5/22/2020	FP	2M	5	2020	Year-3	1	0	Spring
5/22/2020	FP	3M	5	2020	Year-3	1	0	Spring

Date	Species	Site	Month	Year	Survey Year	Presence	Data Gap	Season
5/22/2020	FP	4M	5	2020	Year-3		1	Spring
5/22/2020	FP	5M	5	2020	Year-3	0	0	Spring
5/22/2020	FP	6M	5	2020	Year-3	0	0	Spring
5/22/2020	FP	7M	5	2020	Year-3		1	Spring
5/22/2020	FP	10M	5	2020	Year-3	0	0	Spring
5/22/2020	FP	12M	5	2020	Year-3	1	0	Spring
5/22/2020	FP	14M	5	2020	Year-3	1	0	Spring
5/22/2020	FP	8A	5	2020	Year-3		1	Spring
5/22/2020	FP	9A	5	2020	Year-3		1	Spring
5/22/2020	FP	11A	5	2020	Year-3		1	Spring
5/22/2020	FP	13A	5	2020	Year-3		1	Spring
5/23/2020	FP	1M	5	2020	Year-3	1	0	Spring
5/23/2020	FP	2M	5	2020	Year-3	0	0	Spring
5/23/2020	FP	3M	5	2020	Year-3	1	0	Spring
5/23/2020	FP	4M	5	2020	Year-3		1	Spring
5/23/2020	FP	5M	5	2020	Year-3	0	0	Spring
5/23/2020	FP	6M	5	2020	Year-3	0	0	Spring
5/23/2020	FP	7M	5	2020	Year-3		1	Spring
5/23/2020	FP	10M	5	2020	Year-3	0	0	Spring
5/23/2020	FP	12M	5	2020	Year-3	1	0	Spring
5/23/2020	FP	14M	5	2020	Year-3	1	0	Spring
5/23/2020	FP	8A	5	2020	Year-3		1	Spring
5/23/2020	FP	9A	5	2020	Year-3		1	Spring
5/23/2020	FP	11A	5	2020	Year-3		1	Spring
5/23/2020	FP	13A	5	2020	Year-3		1	Spring
5/24/2020	FP	1M	5	2020	Year-3	1	0	Spring
5/24/2020	FP	2M	5	2020	Year-3	1	0	Spring
5/24/2020	FP	3M	5	2020	Year-3	1	0	Spring
5/24/2020	FP	4M	5	2020	Year-3		1	Spring
5/24/2020	FP	5M	5	2020	Year-3	0	0	Spring
5/24/2020	FP	6M	5	2020	Year-3	0	0	Spring
5/24/2020	FP	7M	5	2020	Year-3		1	Spring
5/24/2020	FP	10M	5	2020	Year-3	0	0	Spring
5/24/2020	FP	12M	5	2020	Year-3	1	0	Spring
5/24/2020	FP	14M	5	2020	Year-3	1	0	Spring
5/24/2020	FP	8A	5	2020	Year-3		1	Spring
5/24/2020	FP	9A	5	2020	Year-3		1	Spring
5/24/2020	FP	11A	5	2020	Year-3		1	Spring
5/24/2020	FP	13A	5	2020	Year-3		1	Spring
5/25/2020	FP	1M	5	2020	Year-3	1	0	Spring
5/25/2020	FP	2M	5	2020	Year-3	1	0	Spring
5/25/2020	FP	3M	5	2020	Year-3	0	0	Spring
5/25/2020	FP	4M	5	2020	Year-3		1	Spring
5/25/2020	FP	5M	5	2020	Year-3	0	0	Spring

Date	Species	Site	Month	Year	Survey Year	Presence	Data Gap	Season
5/25/2020	FP	6M	5	2020	Year-3	0	0	Spring
5/25/2020	FP	7M	5	2020	Year-3		1	Spring
5/25/2020	FP	10M	5	2020	Year-3	0	0	Spring
5/25/2020	FP	12M	5	2020	Year-3	0	0	Spring
5/25/2020	FP	14M	5	2020	Year-3	1	0	Spring
5/25/2020	FP	8A	5	2020	Year-3		1	Spring
5/25/2020	FP	9A	5	2020	Year-3		1	Spring
5/25/2020	FP	11A	5	2020	Year-3		1	Spring
5/25/2020	FP	13A	5	2020	Year-3		1	Spring
5/26/2020	FP	1M	5	2020	Year-3	1	0	Spring
5/26/2020	FP	2M	5	2020	Year-3	1	0	Spring
5/26/2020	FP	3M	5	2020	Year-3	1	0	Spring
5/26/2020	FP	4M	5	2020	Year-3		1	Spring
5/26/2020	FP	5M	5	2020	Year-3	0	0	Spring
5/26/2020	FP	6M	5	2020	Year-3	0	0	Spring
5/26/2020	FP	7M	5	2020	Year-3		1	Spring
5/26/2020	FP	10M	5	2020	Year-3	0	0	Spring
5/26/2020	FP	12M	5	2020	Year-3	0	0	Spring
5/26/2020	FP	14M	5	2020	Year-3	1	0	Spring
5/26/2020	FP	8A	5	2020	Year-3		1	Spring
5/26/2020	FP	9A	5	2020	Year-3		1	Spring
5/26/2020	FP	11A	5	2020	Year-3		1	Spring
5/26/2020	FP	13A	5	2020	Year-3		1	Spring
5/27/2020	FP	1M	5	2020	Year-3	1	0	Spring
5/27/2020	FP	2M	5	2020	Year-3	1	0	Spring
5/27/2020	FP	3M	5	2020	Year-3	1	0	Spring
5/27/2020	FP	4M	5	2020	Year-3		1	Spring
5/27/2020	FP	5M	5	2020	Year-3	0	0	Spring
5/27/2020	FP	6M	5	2020	Year-3	0	0	Spring
5/27/2020	FP	7M	5	2020	Year-3		1	Spring
5/27/2020	FP	10M	5	2020	Year-3	0	0	Spring
5/27/2020	FP	12M	5	2020	Year-3	0	0	Spring
5/27/2020	FP	14M	5	2020	Year-3	1	0	Spring
5/27/2020	FP	8A	5	2020	Year-3		1	Spring
5/27/2020	FP	9A	5	2020	Year-3		1	Spring
5/27/2020	FP	11A	5	2020	Year-3		1	Spring
5/27/2020	FP	13A	5	2020	Year-3		1	Spring
5/28/2020	FP	1M	5	2020	Year-3	1	0	Spring
5/28/2020	FP	2M	5	2020	Year-3	1	0	Spring
5/28/2020	FP	3M	5	2020	Year-3	1	0	Spring
5/28/2020	FP	4M	5	2020	Year-3		1	Spring
5/28/2020	FP	5M	5	2020	Year-3	0	0	Spring
5/28/2020	FP	6M	5	2020	Year-3	0	0	Spring
5/28/2020	FP	7M	5	2020	Year-3		1	Spring

Date	Species	Site	Month	Year	Survey Year	Presence	Data Gap	Season
5/28/2020	FP	10M	5	2020	Year-3	0	0	Spring
5/28/2020	FP	12M	5	2020	Year-3	0	0	Spring
5/28/2020	FP	14M	5	2020	Year-3	1	0	Spring
5/28/2020	FP	8A	5	2020	Year-3		1	Spring
5/28/2020	FP	9A	5	2020	Year-3		1	Spring
5/28/2020	FP	11A	5	2020	Year-3		1	Spring
5/28/2020	FP	13A	5	2020	Year-3		1	Spring
5/29/2020	FP	1M	5	2020	Year-3	1	0	Spring
5/29/2020	FP	2M	5	2020	Year-3	1	0	Spring
5/29/2020	FP	3M	5	2020	Year-3	1	0	Spring
5/29/2020	FP	4M	5	2020	Year-3		1	Spring
5/29/2020	FP	5M	5	2020	Year-3	0	0	Spring
5/29/2020	FP	6M	5	2020	Year-3	0	0	Spring
5/29/2020	FP	7M	5	2020	Year-3		1	Spring
5/29/2020	FP	10M	5	2020	Year-3	0	0	Spring
5/29/2020	FP	12M	5	2020	Year-3	0	0	Spring
5/29/2020	FP	14M	5	2020	Year-3	1	0	Spring
5/29/2020	FP	8A	5	2020	Year-3		1	Spring
5/29/2020	FP	9A	5	2020	Year-3		1	Spring
5/29/2020	FP	11A	5	2020	Year-3		1	Spring
5/29/2020	FP	13A	5	2020	Year-3		1	Spring
5/30/2020	FP	1M	5	2020	Year-3	1	0	Spring
5/30/2020	FP	2M	5	2020	Year-3	1	0	Spring
5/30/2020	FP	3M	5	2020	Year-3	1	0	Spring
5/30/2020	FP	4M	5	2020	Year-3		1	Spring
5/30/2020	FP	5M	5	2020	Year-3	0	0	Spring
5/30/2020	FP	6M	5	2020	Year-3	0	0	Spring
5/30/2020	FP	7M	5	2020	Year-3		1	Spring
5/30/2020	FP	10M	5	2020	Year-3	1	0	Spring
5/30/2020	FP	12M	5	2020	Year-3	0	0	Spring
5/30/2020	FP	14M	5	2020	Year-3	1	0	Spring
5/30/2020	FP	8A	5	2020	Year-3		1	Spring
5/30/2020	FP	9A	5	2020	Year-3		1	Spring
5/30/2020	FP	11A	5	2020	Year-3		1	Spring
5/30/2020	FP	13A	5	2020	Year-3		1	Spring
5/31/2020	FP	1M	5	2020	Year-3	1	0	Spring
5/31/2020	FP	2M	5	2020	Year-3	1	0	Spring
5/31/2020	FP	3M	5	2020	Year-3	1	0	Spring
5/31/2020	FP	4M	5	2020	Year-3		1	Spring
5/31/2020	FP	5M	5	2020	Year-3	0	0	Spring
5/31/2020	FP	6M	5	2020	Year-3	0	0	Spring
5/31/2020	FP	7M	5	2020	Year-3		1	Spring
5/31/2020	FP	10M	5	2020	Year-3	0	0	Spring
5/31/2020	FP	12M	5	2020	Year-3	0	0	Spring

Date	Species	Site	Month	Year	Survey Year	Presence	Data Gap	Season
5/31/2020	FP	14M	5	2020	Year-3	1	0	Spring
5/31/2020	FP	8A	5	2020	Year-3		1	Spring
5/31/2020	FP	9A	5	2020	Year-3		1	Spring
5/31/2020	FP	11A	5	2020	Year-3		1	Spring
5/31/2020	FP	13A	5	2020	Year-3		1	Spring
6/1/2020	FP	1M	6	2020	Year-3	1	0	Summer
6/1/2020	FP	2M	6	2020	Year-3	1	0	Summer
6/1/2020	FP	3M	6	2020	Year-3	1	0	Summer
6/1/2020	FP	4M	6	2020	Year-3		1	Summer
6/1/2020	FP	5M	6	2020	Year-3	0	0	Summer
6/1/2020	FP	6M	6	2020	Year-3	0	0	Summer
6/1/2020	FP	7M	6	2020	Year-3		1	Summer
6/1/2020	FP	10M	6	2020	Year-3	1	0	Summer
6/1/2020	FP	12M	6	2020	Year-3	0	0	Summer
6/1/2020	FP	14M	6	2020	Year-3	1	0	Summer
6/1/2020	FP	8A	6	2020	Year-3		1	Summer
6/1/2020	FP	9A	6	2020	Year-3		1	Summer
6/1/2020	FP	11A	6	2020	Year-3		1	Summer
6/1/2020	FP	13A	6	2020	Year-3		1	Summer
6/2/2020	FP	1M	6	2020	Year-3	1	0	Summer
6/2/2020	FP	2M	6	2020	Year-3	1	0	Summer
6/2/2020	FP	3M	6	2020	Year-3	0	0	Summer
6/2/2020	FP	4M	6	2020	Year-3		1	Summer
6/2/2020	FP	5M	6	2020	Year-3	0	0	Summer
6/2/2020	FP	6M	6	2020	Year-3	0	0	Summer
6/2/2020	FP	7M	6	2020	Year-3		1	Summer
6/2/2020	FP	10M	6	2020	Year-3	0	0	Summer
6/2/2020	FP	12M	6	2020	Year-3	0	0	Summer
6/2/2020	FP	14M	6	2020	Year-3	0	0	Summer
6/2/2020	FP	8A	6	2020	Year-3		1	Summer
6/2/2020	FP	9A	6	2020	Year-3		1	Summer
6/2/2020	FP	11A	6	2020	Year-3		1	Summer
6/2/2020	FP	13A	6	2020	Year-3		1	Summer
6/3/2020	FP	1M	6	2020	Year-3	1	0	Summer
6/3/2020	FP	2M	6	2020	Year-3	1	0	Summer
6/3/2020	FP	3M	6	2020	Year-3	0	0	Summer
6/3/2020	FP	4M	6	2020	Year-3		1	Summer
6/3/2020	FP	5M	6	2020	Year-3	0	0	Summer
6/3/2020	FP	6M	6	2020	Year-3	0	0	Summer
6/3/2020	FP	7M	6	2020	Year-3		1	Summer
6/3/2020	FP	10M	6	2020	Year-3	0	0	Summer
6/3/2020	FP	12M	6	2020	Year-3	0	0	Summer
6/3/2020	FP	14M	6	2020	Year-3	1	0	Summer
6/3/2020	FP	8A	6	2020	Year-3		1	Summer

Date	Species	Site	Month	Year	Survey Year	Presence	Data Gap	Season
6/3/2020	FP	9A	6	2020	Year-3		1	Summer
6/3/2020	FP	11A	6	2020	Year-3		1	Summer
6/3/2020	FP	13A	6	2020	Year-3		1	Summer
6/4/2020	FP	1M	6	2020	Year-3	1	0	Summer
6/4/2020	FP	2M	6	2020	Year-3	1	0	Summer
6/4/2020	FP	3M	6	2020	Year-3	1	0	Summer
6/4/2020	FP	4M	6	2020	Year-3		1	Summer
6/4/2020	FP	5M	6	2020	Year-3	0	0	Summer
6/4/2020	FP	6M	6	2020	Year-3	0	0	Summer
6/4/2020	FP	7M	6	2020	Year-3		1	Summer
6/4/2020	FP	10M	6	2020	Year-3	0	0	Summer
6/4/2020	FP	12M	6	2020	Year-3	0	0	Summer
6/4/2020	FP	14M	6	2020	Year-3	1	0	Summer
6/4/2020	FP	8A	6	2020	Year-3		1	Summer
6/4/2020	FP	9A	6	2020	Year-3		1	Summer
6/4/2020	FP	11A	6	2020	Year-3		1	Summer
6/4/2020	FP	13A	6	2020	Year-3		1	Summer
6/5/2020	FP	1M	6	2020	Year-3	0	0	Summer
6/5/2020	FP	2M	6	2020	Year-3	0	0	Summer
6/5/2020	FP	3M	6	2020	Year-3	1	0	Summer
6/5/2020	FP	4M	6	2020	Year-3		1	Summer
6/5/2020	FP	5M	6	2020	Year-3	0	0	Summer
6/5/2020	FP	6M	6	2020	Year-3	0	0	Summer
6/5/2020	FP	7M	6	2020	Year-3		1	Summer
6/5/2020	FP	10M	6	2020	Year-3	0	0	Summer
6/5/2020	FP	12M	6	2020	Year-3	0	0	Summer
6/5/2020	FP	14M	6	2020	Year-3	1	0	Summer
6/5/2020	FP	8A	6	2020	Year-3		1	Summer
6/5/2020	FP	9A	6	2020	Year-3		1	Summer
6/5/2020	FP	11A	6	2020	Year-3		1	Summer
6/5/2020	FP	13A	6	2020	Year-3		1	Summer
6/6/2020	FP	1M	6	2020	Year-3	1	0	Summer
6/6/2020	FP	2M	6	2020	Year-3	1	0	Summer
6/6/2020	FP	3M	6	2020	Year-3	1	0	Summer
6/6/2020	FP	4M	6	2020	Year-3		1	Summer
6/6/2020	FP	5M	6	2020	Year-3	0	0	Summer
6/6/2020	FP	6M	6	2020	Year-3	0	0	Summer
6/6/2020	FP	7M	6	2020	Year-3		1	Summer
6/6/2020	FP	10M	6	2020	Year-3	0	0	Summer
6/6/2020	FP	12M	6	2020	Year-3	0	0	Summer
6/6/2020	FP	14M	6	2020	Year-3	1	0	Summer
6/6/2020	FP	8A	6	2020	Year-3		1	Summer
6/6/2020	FP	9A	6	2020	Year-3		1	Summer
6/6/2020	FP	11A	6	2020	Year-3		1	Summer

Date	Species	Site	Month	Year	Survey Year	Presence	Data Gap	Season
6/6/2020	FP	13A	6	2020	Year-3		1	Summer
6/7/2020	FP	1M	6	2020	Year-3	0	0	Summer
6/7/2020	FP	2M	6	2020	Year-3	0	0	Summer
6/7/2020	FP	3M	6	2020	Year-3	1	0	Summer
6/7/2020	FP	4M	6	2020	Year-3		1	Summer
6/7/2020	FP	5M	6	2020	Year-3	0	0	Summer
6/7/2020	FP	6M	6	2020	Year-3	0	0	Summer
6/7/2020	FP	7M	6	2020	Year-3		1	Summer
6/7/2020	FP	10M	6	2020	Year-3	0	0	Summer
6/7/2020	FP	12M	6	2020	Year-3	0	0	Summer
6/7/2020	FP	14M	6	2020	Year-3	1	0	Summer
6/7/2020	FP	8A	6	2020	Year-3		1	Summer
6/7/2020	FP	9A	6	2020	Year-3		1	Summer
6/7/2020	FP	11A	6	2020	Year-3		1	Summer
6/7/2020	FP	13A	6	2020	Year-3		1	Summer
6/8/2020	FP	1M	6	2020	Year-3	0	0	Summer
6/8/2020	FP	2M	6	2020	Year-3	0	0	Summer
6/8/2020	FP	3M	6	2020	Year-3	0	0	Summer
6/8/2020	FP	4M	6	2020	Year-3		1	Summer
6/8/2020	FP	5M	6	2020	Year-3	0	0	Summer
6/8/2020	FP	6M	6	2020	Year-3	0	0	Summer
6/8/2020	FP	7M	6	2020	Year-3		1	Summer
6/8/2020	FP	10M	6	2020	Year-3	0	0	Summer
6/8/2020	FP	12M	6	2020	Year-3	0	0	Summer
6/8/2020	FP	14M	6	2020	Year-3	1	0	Summer
6/8/2020	FP	8A	6	2020	Year-3		1	Summer
6/8/2020	FP	9A	6	2020	Year-3		1	Summer
6/8/2020	FP	11A	6	2020	Year-3		1	Summer
6/8/2020	FP	13A	6	2020	Year-3		1	Summer
6/9/2020	FP	1M	6	2020	Year-3	1	0	Summer
6/9/2020	FP	2M	6	2020	Year-3	0	0	Summer
6/9/2020	FP	3M	6	2020	Year-3	1	0	Summer
6/9/2020	FP	4M	6	2020	Year-3		1	Summer
6/9/2020	FP	5M	6	2020	Year-3	0	0	Summer
6/9/2020	FP	6M	6	2020	Year-3	0	0	Summer
6/9/2020	FP	7M	6	2020	Year-3		1	Summer
6/9/2020	FP	10M	6	2020	Year-3	0	0	Summer
6/9/2020	FP	12M	6	2020	Year-3	0	0	Summer
6/9/2020	FP	14M	6	2020	Year-3	0	0	Summer
6/9/2020	FP	8A	6	2020	Year-3		1	Summer
6/9/2020	FP	9A	6	2020	Year-3		1	Summer
6/9/2020	FP	11A	6	2020	Year-3		1	Summer
6/9/2020	FP	13A	6	2020	Year-3		1	Summer
6/10/2020	FP	1M	6	2020	Year-3	1	0	Summer

Date	Species	Site	Month	Year	Survey Year	Presence	Data Gap	Season
6/10/2020	FP	2M	6	2020	Year-3	1	0	Summer
6/10/2020	FP	3M	6	2020	Year-3	1	0	Summer
6/10/2020	FP	4M	6	2020	Year-3		1	Summer
6/10/2020	FP	5M	6	2020	Year-3	0	0	Summer
6/10/2020	FP	6M	6	2020	Year-3	0	0	Summer
6/10/2020	FP	7M	6	2020	Year-3		1	Summer
6/10/2020	FP	10M	6	2020	Year-3	0	0	Summer
6/10/2020	FP	12M	6	2020	Year-3	0	0	Summer
6/10/2020	FP	14M	6	2020	Year-3	1	0	Summer
6/10/2020	FP	8A	6	2020	Year-3		1	Summer
6/10/2020	FP	9A	6	2020	Year-3		1	Summer
6/10/2020	FP	11A	6	2020	Year-3		1	Summer
6/10/2020	FP	13A	6	2020	Year-3		1	Summer
6/11/2020	FP	1M	6	2020	Year-3	1	0	Summer
6/11/2020	FP	2M	6	2020	Year-3	1	0	Summer
6/11/2020	FP	3M	6	2020	Year-3	1	0	Summer
6/11/2020	FP	4M	6	2020	Year-3		1	Summer
6/11/2020	FP	5M	6	2020	Year-3	0	0	Summer
6/11/2020	FP	6M	6	2020	Year-3	0	0	Summer
6/11/2020	FP	7M	6	2020	Year-3		1	Summer
6/11/2020	FP	10M	6	2020	Year-3	0	0	Summer
6/11/2020	FP	12M	6	2020	Year-3	0	0	Summer
6/11/2020	FP	14M	6	2020	Year-3	1	0	Summer
6/11/2020	FP	8A	6	2020	Year-3		1	Summer
6/11/2020	FP	9A	6	2020	Year-3		1	Summer
6/11/2020	FP	11A	6	2020	Year-3		1	Summer
6/11/2020	FP	13A	6	2020	Year-3		1	Summer
6/12/2020	FP	1M	6	2020	Year-3	0	0	Summer
6/12/2020	FP	2M	6	2020	Year-3	1	0	Summer
6/12/2020	FP	3M	6	2020	Year-3	1	0	Summer
6/12/2020	FP	4M	6	2020	Year-3		1	Summer
6/12/2020	FP	5M	6	2020	Year-3	0	0	Summer
6/12/2020	FP	6M	6	2020	Year-3	0	0	Summer
6/12/2020	FP	7M	6	2020	Year-3		1	Summer
6/12/2020	FP	10M	6	2020	Year-3	0	0	Summer
6/12/2020	FP	12M	6	2020	Year-3	0	0	Summer
6/12/2020	FP	14M	6	2020	Year-3	1	0	Summer
6/12/2020	FP	8A	6	2020	Year-3		1	Summer
6/12/2020	FP	9A	6	2020	Year-3		1	Summer
6/12/2020	FP	11A	6	2020	Year-3		1	Summer
6/12/2020	FP	13A	6	2020	Year-3		1	Summer
6/13/2020	FP	1M	6	2020	Year-3	1	0	Summer
6/13/2020	FP	2M	6	2020	Year-3	1	0	Summer
6/13/2020	FP	3M	6	2020	Year-3	1	0	Summer

Date	Species	Site	Month	Year	Survey Year	Presence	Data Gap	Season
6/13/2020	FP	4M	6	2020	Year-3		1	Summer
6/13/2020	FP	5M	6	2020	Year-3	1	0	Summer
6/13/2020	FP	6M	6	2020	Year-3	0	0	Summer
6/13/2020	FP	7M	6	2020	Year-3		1	Summer
6/13/2020	FP	10M	6	2020	Year-3	0	0	Summer
6/13/2020	FP	12M	6	2020	Year-3	1	0	Summer
6/13/2020	FP	14M	6	2020	Year-3	1	0	Summer
6/13/2020	FP	8A	6	2020	Year-3		1	Summer
6/13/2020	FP	9A	6	2020	Year-3		1	Summer
6/13/2020	FP	11A	6	2020	Year-3		1	Summer
6/13/2020	FP	13A	6	2020	Year-3		1	Summer
6/14/2020	FP	1M	6	2020	Year-3	1	0	Summer
6/14/2020	FP	2M	6	2020	Year-3	0	0	Summer
6/14/2020	FP	3M	6	2020	Year-3	1	0	Summer
6/14/2020	FP	4M	6	2020	Year-3		1	Summer
6/14/2020	FP	5M	6	2020	Year-3	0	0	Summer
6/14/2020	FP	6M	6	2020	Year-3	0	0	Summer
6/14/2020	FP	7M	6	2020	Year-3		1	Summer
6/14/2020	FP	10M	6	2020	Year-3	0	0	Summer
6/14/2020	FP	12M	6	2020	Year-3	1	0	Summer
6/14/2020	FP	14M	6	2020	Year-3	1	0	Summer
6/14/2020	FP	8A	6	2020	Year-3		1	Summer
6/14/2020	FP	9A	6	2020	Year-3		1	Summer
6/14/2020	FP	11A	6	2020	Year-3		1	Summer
6/14/2020	FP	13A	6	2020	Year-3		1	Summer
6/15/2020	FP	1M	6	2020	Year-3	1	0	Summer
6/15/2020	FP	2M	6	2020	Year-3	1	0	Summer
6/15/2020	FP	3M	6	2020	Year-3	1	0	Summer
6/15/2020	FP	4M	6	2020	Year-3		1	Summer
6/15/2020	FP	5M	6	2020	Year-3	0	0	Summer
6/15/2020	FP	6M	6	2020	Year-3	0	0	Summer
6/15/2020	FP	7M	6	2020	Year-3		1	Summer
6/15/2020	FP	10M	6	2020	Year-3	1	0	Summer
6/15/2020	FP	12M	6	2020	Year-3	0	0	Summer
6/15/2020	FP	14M	6	2020	Year-3	0	0	Summer
6/15/2020	FP	8A	6	2020	Year-3		1	Summer
6/15/2020	FP	9A	6	2020	Year-3		1	Summer
6/15/2020	FP	11A	6	2020	Year-3		1	Summer
6/15/2020	FP	13A	6	2020	Year-3		1	Summer
6/16/2020	FP	1M	6	2020	Year-3	0	0	Summer
6/16/2020	FP	2M	6	2020	Year-3	1	0	Summer
6/16/2020	FP	3M	6	2020	Year-3	1	0	Summer
6/16/2020	FP	4M	6	2020	Year-3		1	Summer
6/16/2020	FP	5M	6	2020	Year-3	0	0	Summer

Date	Species	Site	Month	Year	Survey Year	Presence	Data Gap	Season
6/16/2020	FP	6M	6	2020	Year-3	0	0	Summer
6/16/2020	FP	7M	6	2020	Year-3		1	Summer
6/16/2020	FP	10M	6	2020	Year-3	0	0	Summer
6/16/2020	FP	12M	6	2020	Year-3	0	0	Summer
6/16/2020	FP	14M	6	2020	Year-3	0	0	Summer
6/16/2020	FP	8A	6	2020	Year-3		1	Summer
6/16/2020	FP	9A	6	2020	Year-3		1	Summer
6/16/2020	FP	11A	6	2020	Year-3		1	Summer
6/16/2020	FP	13A	6	2020	Year-3		1	Summer
6/17/2020	FP	1M	6	2020	Year-3	0	0	Summer
6/17/2020	FP	2M	6	2020	Year-3	1	0	Summer
6/17/2020	FP	3M	6	2020	Year-3	1	0	Summer
6/17/2020	FP	4M	6	2020	Year-3		1	Summer
6/17/2020	FP	5M	6	2020	Year-3	0	0	Summer
6/17/2020	FP	6M	6	2020	Year-3	1	0	Summer
6/17/2020	FP	7M	6	2020	Year-3		1	Summer
6/17/2020	FP	10M	6	2020	Year-3	0	0	Summer
6/17/2020	FP	12M	6	2020	Year-3	0	0	Summer
6/17/2020	FP	14M	6	2020	Year-3	0	0	Summer
6/17/2020	FP	8A	6	2020	Year-3		1	Summer
6/17/2020	FP	9A	6	2020	Year-3		1	Summer
6/17/2020	FP	11A	6	2020	Year-3		1	Summer
6/17/2020	FP	13A	6	2020	Year-3		1	Summer
6/18/2020	FP	1M	6	2020	Year-3	0	0	Summer
6/18/2020	FP	2M	6	2020	Year-3	1	0	Summer
6/18/2020	FP	3M	6	2020	Year-3	1	0	Summer
6/18/2020	FP	4M	6	2020	Year-3		1	Summer
6/18/2020	FP	5M	6	2020	Year-3	0	0	Summer
6/18/2020	FP	6M	6	2020	Year-3	0	0	Summer
6/18/2020	FP	7M	6	2020	Year-3		1	Summer
6/18/2020	FP	10M	6	2020	Year-3	0	0	Summer
6/18/2020	FP	12M	6	2020	Year-3	1	0	Summer
6/18/2020	FP	14M	6	2020	Year-3	1	0	Summer
6/18/2020	FP	8A	6	2020	Year-3		1	Summer
6/18/2020	FP	9A	6	2020	Year-3		1	Summer
6/18/2020	FP	11A	6	2020	Year-3		1	Summer
6/18/2020	FP	13A	6	2020	Year-3		1	Summer
6/19/2020	FP	1M	6	2020	Year-3	0	0	Summer
6/19/2020	FP	2M	6	2020	Year-3	0	0	Summer
6/19/2020	FP	3M	6	2020	Year-3	1	0	Summer
6/19/2020	FP	4M	6	2020	Year-3		1	Summer
6/19/2020	FP	5M	6	2020	Year-3	0	0	Summer
6/19/2020	FP	6M	6	2020	Year-3	0	0	Summer
6/19/2020	FP	7M	6	2020	Year-3		1	Summer

Date	Species	Site	Month	Year	Survey Year	Presence	Data Gap	Season
6/19/2020	FP	10M	6	2020	Year-3	0	0	Summer
6/19/2020	FP	12M	6	2020	Year-3	0	0	Summer
6/19/2020	FP	14M	6	2020	Year-3	1	0	Summer
6/19/2020	FP	8A	6	2020	Year-3		1	Summer
6/19/2020	FP	9A	6	2020	Year-3		1	Summer
6/19/2020	FP	11A	6	2020	Year-3		1	Summer
6/19/2020	FP	13A	6	2020	Year-3		1	Summer
6/20/2020	FP	1M	6	2020	Year-3	0	0	Summer
6/20/2020	FP	2M	6	2020	Year-3	1	0	Summer
6/20/2020	FP	3M	6	2020	Year-3	1	0	Summer
6/20/2020	FP	4M	6	2020	Year-3		1	Summer
6/20/2020	FP	5M	6	2020	Year-3	0	0	Summer
6/20/2020	FP	6M	6	2020	Year-3	0	0	Summer
6/20/2020	FP	7M	6	2020	Year-3		1	Summer
6/20/2020	FP	10M	6	2020	Year-3		1	Summer
6/20/2020	FP	12M	6	2020	Year-3		1	Summer
6/20/2020	FP	14M	6	2020	Year-3		1	Summer
6/20/2020	FP	8A	6	2020	Year-3		1	Summer
6/20/2020	FP	9A	6	2020	Year-3		1	Summer
6/20/2020	FP	11A	6	2020	Year-3		1	Summer
6/20/2020	FP	13A	6	2020	Year-3		1	Summer
6/21/2020	FP	1M	6	2020	Year-3	0	0	Summer
6/21/2020	FP	2M	6	2020	Year-3	0	0	Summer
6/21/2020	FP	3M	6	2020	Year-3	1	0	Summer
6/21/2020	FP	4M	6	2020	Year-3		1	Summer
6/21/2020	FP	5M	6	2020	Year-3		1	Summer
6/21/2020	FP	6M	6	2020	Year-3		1	Summer
6/21/2020	FP	7M	6	2020	Year-3		1	Summer
6/21/2020	FP	10M	6	2020	Year-3	0	0	Summer
6/21/2020	FP	12M	6	2020	Year-3	1	0	Summer
6/21/2020	FP	14M	6	2020	Year-3	1	0	Summer
6/21/2020	FP	8A	6	2020	Year-3		1	Summer
6/21/2020	FP	9A	6	2020	Year-3		1	Summer
6/21/2020	FP	11A	6	2020	Year-3		1	Summer
6/21/2020	FP	13A	6	2020	Year-3		1	Summer
6/22/2020	FP	1M	6	2020	Year-3	1	0	Summer
6/22/2020	FP	2M	6	2020	Year-3	1	0	Summer
6/22/2020	FP	3M	6	2020	Year-3	1	0	Summer
6/22/2020	FP	4M	6	2020	Year-3		1	Summer
6/22/2020	FP	5M	6	2020	Year-3		1	Summer
6/22/2020	FP	6M	6	2020	Year-3		1	Summer
6/22/2020	FP	7M	6	2020	Year-3		1	Summer
6/22/2020	FP	10M	6	2020	Year-3	0	0	Summer
6/22/2020	FP	12M	6	2020	Year-3	0	0	Summer

Date	Species	Site	Month	Year	Survey Year	Presence	Data Gap	Season
6/22/2020	FP	14M	6	2020	Year-3	1	0	Summer
6/22/2020	FP	8A	6	2020	Year-3		1	Summer
6/22/2020	FP	9A	6	2020	Year-3		1	Summer
6/22/2020	FP	11A	6	2020	Year-3		1	Summer
6/22/2020	FP	13A	6	2020	Year-3		1	Summer
6/23/2020	FP	1M	6	2020	Year-3	1	0	Summer
6/23/2020	FP	2M	6	2020	Year-3	1	0	Summer
6/23/2020	FP	3M	6	2020	Year-3	1	0	Summer
6/23/2020	FP	4M	6	2020	Year-3		1	Summer
6/23/2020	FP	5M	6	2020	Year-3		1	Summer
6/23/2020	FP	6M	6	2020	Year-3		1	Summer
6/23/2020	FP	7M	6	2020	Year-3		1	Summer
6/23/2020	FP	10M	6	2020	Year-3	0	0	Summer
6/23/2020	FP	12M	6	2020	Year-3	0	0	Summer
6/23/2020	FP	14M	6	2020	Year-3	1	0	Summer
6/23/2020	FP	8A	6	2020	Year-3		1	Summer
6/23/2020	FP	9A	6	2020	Year-3		1	Summer
6/23/2020	FP	11A	6	2020	Year-3		1	Summer
6/23/2020	FP	13A	6	2020	Year-3		1	Summer
6/24/2020	FP	1M	6	2020	Year-3	1	0	Summer
6/24/2020	FP	2M	6	2020	Year-3	0	0	Summer
6/24/2020	FP	3M	6	2020	Year-3	0	0	Summer
6/24/2020	FP	4M	6	2020	Year-3		1	Summer
6/24/2020	FP	5M	6	2020	Year-3		1	Summer
6/24/2020	FP	6M	6	2020	Year-3		1	Summer
6/24/2020	FP	7M	6	2020	Year-3		1	Summer
6/24/2020	FP	10M	6	2020	Year-3	1	0	Summer
6/24/2020	FP	12M	6	2020	Year-3	1	0	Summer
6/24/2020	FP	14M	6	2020	Year-3	1	0	Summer
6/24/2020	FP	8A	6	2020	Year-3		1	Summer
6/24/2020	FP	9A	6	2020	Year-3		1	Summer
6/24/2020	FP	11A	6	2020	Year-3		1	Summer
6/24/2020	FP	13A	6	2020	Year-3		1	Summer
6/25/2020	FP	1M	6	2020	Year-3	0	0	Summer
6/25/2020	FP	2M	6	2020	Year-3	0	0	Summer
6/25/2020	FP	3M	6	2020	Year-3	1	0	Summer
6/25/2020	FP	4M	6	2020	Year-3		1	Summer
6/25/2020	FP	5M	6	2020	Year-3		1	Summer
6/25/2020	FP	6M	6	2020	Year-3		1	Summer
6/25/2020	FP	7M	6	2020	Year-3		1	Summer
6/25/2020	FP	10M	6	2020	Year-3	0	0	Summer
6/25/2020	FP	12M	6	2020	Year-3	0	0	Summer
6/25/2020	FP	14M	6	2020	Year-3	1	0	Summer
6/25/2020	FP	8A	6	2020	Year-3		1	Summer

Date	Species	Site	Month	Year	Survey Year	Presence	Data Gap	Season
6/25/2020	FP	9A	6	2020	Year-3		1	Summer
6/25/2020	FP	11A	6	2020	Year-3		1	Summer
6/25/2020	FP	13A	6	2020	Year-3		1	Summer
6/26/2020	FP	1M	6	2020	Year-3	0	0	Summer
6/26/2020	FP	2M	6	2020	Year-3		1	Summer
6/26/2020	FP	3M	6	2020	Year-3	1	0	Summer
6/26/2020	FP	4M	6	2020	Year-3		1	Summer
6/26/2020	FP	5M	6	2020	Year-3		1	Summer
6/26/2020	FP	6M	6	2020	Year-3		1	Summer
6/26/2020	FP	7M	6	2020	Year-3		1	Summer
6/26/2020	FP	10M	6	2020	Year-3	0	0	Summer
6/26/2020	FP	12M	6	2020	Year-3	0	0	Summer
6/26/2020	FP	14M	6	2020	Year-3	1	0	Summer
6/26/2020	FP	8A	6	2020	Year-3		1	Summer
6/26/2020	FP	9A	6	2020	Year-3		1	Summer
6/26/2020	FP	11A	6	2020	Year-3		1	Summer
6/26/2020	FP	13A	6	2020	Year-3		1	Summer
6/27/2020	FP	1M	6	2020	Year-3	0	0	Summer
6/27/2020	FP	2M	6	2020	Year-3		1	Summer
6/27/2020	FP	3M	6	2020	Year-3	1	0	Summer
6/27/2020	FP	4M	6	2020	Year-3		1	Summer
6/27/2020	FP	5M	6	2020	Year-3		1	Summer
6/27/2020	FP	6M	6	2020	Year-3		1	Summer
6/27/2020	FP	7M	6	2020	Year-3		1	Summer
6/27/2020	FP	10M	6	2020	Year-3	0	0	Summer
6/27/2020	FP	12M	6	2020	Year-3	0	0	Summer
6/27/2020	FP	14M	6	2020	Year-3	1	0	Summer
6/27/2020	FP	8A	6	2020	Year-3		1	Summer
6/27/2020	FP	9A	6	2020	Year-3		1	Summer
6/27/2020	FP	11A	6	2020	Year-3		1	Summer
6/27/2020	FP	13A	6	2020	Year-3		1	Summer
6/28/2020	FP	1M	6	2020	Year-3		1	Summer
6/28/2020	FP	2M	6	2020	Year-3		1	Summer
6/28/2020	FP	3M	6	2020	Year-3	1	0	Summer
6/28/2020	FP	4M	6	2020	Year-3		1	Summer
6/28/2020	FP	5M	6	2020	Year-3		1	Summer
6/28/2020	FP	6M	6	2020	Year-3		1	Summer
6/28/2020	FP	7M	6	2020	Year-3		1	Summer
6/28/2020	FP	10M	6	2020	Year-3	0	0	Summer
6/28/2020	FP	12M	6	2020	Year-3	1	0	Summer
6/28/2020	FP	14M	6	2020	Year-3	1	0	Summer
6/28/2020	FP	8A	6	2020	Year-3		1	Summer
6/28/2020	FP	9A	6	2020	Year-3		1	Summer
6/28/2020	FP	11A	6	2020	Year-3		1	Summer

Date	Species	Site	Month	Year	Survey Year	Presence	Data Gap	Season
6/28/2020	FP	13A	6	2020	Year-3		1	Summer
6/29/2020	FP	1M	6	2020	Year-3		1	Summer
6/29/2020	FP	2M	6	2020	Year-3		1	Summer
6/29/2020	FP	3M	6	2020	Year-3		1	Summer
6/29/2020	FP	4M	6	2020	Year-3		1	Summer
6/29/2020	FP	5M	6	2020	Year-3		1	Summer
6/29/2020	FP	6M	6	2020	Year-3		1	Summer
6/29/2020	FP	7M	6	2020	Year-3		1	Summer
6/29/2020	FP	10M	6	2020	Year-3	0	0	Summer
6/29/2020	FP	12M	6	2020	Year-3	0	0	Summer
6/29/2020	FP	14M	6	2020	Year-3	0	0	Summer
6/29/2020	FP	8A	6	2020	Year-3		1	Summer
6/29/2020	FP	9A	6	2020	Year-3		1	Summer
6/29/2020	FP	11A	6	2020	Year-3		1	Summer
6/29/2020	FP	13A	6	2020	Year-3		1	Summer
6/30/2020	FP	1M	6	2020	Year-3		1	Summer
6/30/2020	FP	2M	6	2020	Year-3		1	Summer
6/30/2020	FP	3M	6	2020	Year-3		1	Summer
6/30/2020	FP	4M	6	2020	Year-3		1	Summer
6/30/2020	FP	5M	6	2020	Year-3		1	Summer
6/30/2020	FP	6M	6	2020	Year-3		1	Summer
6/30/2020	FP	7M	6	2020	Year-3		1	Summer
6/30/2020	FP	10M	6	2020	Year-3	0	0	Summer
6/30/2020	FP	12M	6	2020	Year-3	0	0	Summer
6/30/2020	FP	14M	6	2020	Year-3	0	0	Summer
6/30/2020	FP	8A	6	2020	Year-3		1	Summer
6/30/2020	FP	9A	6	2020	Year-3		1	Summer
6/30/2020	FP	11A	6	2020	Year-3		1	Summer
6/30/2020	FP	13A	6	2020	Year-3		1	Summer
7/1/2020	FP	1M	7	2020	Year-3		1	Summer
7/1/2020	FP	2M	7	2020	Year-3		1	Summer
7/1/2020	FP	3M	7	2020	Year-3		1	Summer
7/1/2020	FP	4M	7	2020	Year-3		1	Summer
7/1/2020	FP	5M	7	2020	Year-3		1	Summer
7/1/2020	FP	6M	7	2020	Year-3		1	Summer
7/1/2020	FP	7M	7	2020	Year-3		1	Summer
7/1/2020	FP	10M	7	2020	Year-3	0	0	Summer
7/1/2020	FP	12M	7	2020	Year-3	1	0	Summer
7/1/2020	FP	14M	7	2020	Year-3	0	0	Summer
7/1/2020	FP	8A	7	2020	Year-3		1	Summer
7/1/2020	FP	9A	7	2020	Year-3		1	Summer
7/1/2020	FP	11A	7	2020	Year-3		1	Summer
7/1/2020	FP	13A	7	2020	Year-3		1	Summer
7/2/2020	FP	1M	7	2020	Year-3		1	Summer

Date	Species	Site	Month	Year	Survey Year	Presence	Data Gap	Season
7/2/2020	FP	2M	7	2020	Year-3		1	Summer
7/2/2020	FP	3M	7	2020	Year-3		1	Summer
7/2/2020	FP	4M	7	2020	Year-3		1	Summer
7/2/2020	FP	5M	7	2020	Year-3		1	Summer
7/2/2020	FP	6M	7	2020	Year-3		1	Summer
7/2/2020	FP	7M	7	2020	Year-3		1	Summer
7/2/2020	FP	10M	7	2020	Year-3	0	0	Summer
7/2/2020	FP	12M	7	2020	Year-3	0	0	Summer
7/2/2020	FP	14M	7	2020	Year-3	1	0	Summer
7/2/2020	FP	8A	7	2020	Year-3		1	Summer
7/2/2020	FP	9A	7	2020	Year-3		1	Summer
7/2/2020	FP	11A	7	2020	Year-3		1	Summer
7/2/2020	FP	13A	7	2020	Year-3		1	Summer
7/3/2020	FP	1M	7	2020	Year-3		1	Summer
7/3/2020	FP	2M	7	2020	Year-3		1	Summer
7/3/2020	FP	3M	7	2020	Year-3		1	Summer
7/3/2020	FP	4M	7	2020	Year-3		1	Summer
7/3/2020	FP	5M	7	2020	Year-3		1	Summer
7/3/2020	FP	6M	7	2020	Year-3		1	Summer
7/3/2020	FP	7M	7	2020	Year-3		1	Summer
7/3/2020	FP	10M	7	2020	Year-3	0	0	Summer
7/3/2020	FP	12M	7	2020	Year-3	0	0	Summer
7/3/2020	FP	14M	7	2020	Year-3	1	0	Summer
7/3/2020	FP	8A	7	2020	Year-3		1	Summer
7/3/2020	FP	9A	7	2020	Year-3		1	Summer
7/3/2020	FP	11A	7	2020	Year-3		1	Summer
7/3/2020	FP	13A	7	2020	Year-3		1	Summer
7/4/2020	FP	1M	7	2020	Year-3		1	Summer
7/4/2020	FP	2M	7	2020	Year-3		1	Summer
7/4/2020	FP	3M	7	2020	Year-3		1	Summer
7/4/2020	FP	4M	7	2020	Year-3		1	Summer
7/4/2020	FP	5M	7	2020	Year-3		1	Summer
7/4/2020	FP	6M	7	2020	Year-3		1	Summer
7/4/2020	FP	7M	7	2020	Year-3		1	Summer
7/4/2020	FP	10M	7	2020	Year-3	1	0	Summer
7/4/2020	FP	12M	7	2020	Year-3	1	0	Summer
7/4/2020	FP	14M	7	2020	Year-3	1	0	Summer
7/4/2020	FP	8A	7	2020	Year-3		1	Summer
7/4/2020	FP	9A	7	2020	Year-3		1	Summer
7/4/2020	FP	11A	7	2020	Year-3		1	Summer
7/4/2020	FP	13A	7	2020	Year-3		1	Summer
7/5/2020	FP	1M	7	2020	Year-3		1	Summer
7/5/2020	FP	2M	7	2020	Year-3		1	Summer
7/5/2020	FP	3M	7	2020	Year-3		1	Summer

Date	Species	Site	Month	Year	Survey Year	Presence	Data Gap	Season
7/5/2020	FP	4M	7	2020	Year-3		1	Summer
7/5/2020	FP	5M	7	2020	Year-3		1	Summer
7/5/2020	FP	6M	7	2020	Year-3		1	Summer
7/5/2020	FP	7M	7	2020	Year-3		1	Summer
7/5/2020	FP	10M	7	2020	Year-3	0	0	Summer
7/5/2020	FP	12M	7	2020	Year-3	1	0	Summer
7/5/2020	FP	14M	7	2020	Year-3	1	0	Summer
7/5/2020	FP	8A	7	2020	Year-3		1	Summer
7/5/2020	FP	9A	7	2020	Year-3		1	Summer
7/5/2020	FP	11A	7	2020	Year-3		1	Summer
7/5/2020	FP	13A	7	2020	Year-3		1	Summer
7/6/2020	FP	1M	7	2020	Year-3		1	Summer
7/6/2020	FP	2M	7	2020	Year-3		1	Summer
7/6/2020	FP	3M	7	2020	Year-3		1	Summer
7/6/2020	FP	4M	7	2020	Year-3		1	Summer
7/6/2020	FP	5M	7	2020	Year-3		1	Summer
7/6/2020	FP	6M	7	2020	Year-3		1	Summer
7/6/2020	FP	7M	7	2020	Year-3		1	Summer
7/6/2020	FP	10M	7	2020	Year-3	0	0	Summer
7/6/2020	FP	12M	7	2020	Year-3	1	0	Summer
7/6/2020	FP	14M	7	2020	Year-3	1	0	Summer
7/6/2020	FP	8A	7	2020	Year-3		1	Summer
7/6/2020	FP	9A	7	2020	Year-3		1	Summer
7/6/2020	FP	11A	7	2020	Year-3		1	Summer
7/6/2020	FP	13A	7	2020	Year-3		1	Summer
7/7/2020	FP	1M	7	2020	Year-3		1	Summer
7/7/2020	FP	2M	7	2020	Year-3		1	Summer
7/7/2020	FP	3M	7	2020	Year-3		1	Summer
7/7/2020	FP	4M	7	2020	Year-3		1	Summer
7/7/2020	FP	5M	7	2020	Year-3		1	Summer
7/7/2020	FP	6M	7	2020	Year-3		1	Summer
7/7/2020	FP	7M	7	2020	Year-3		1	Summer
7/7/2020	FP	10M	7	2020	Year-3	0	0	Summer
7/7/2020	FP	12M	7	2020	Year-3	0	0	Summer
7/7/2020	FP	14M	7	2020	Year-3	1	0	Summer
7/7/2020	FP	8A	7	2020	Year-3		1	Summer
7/7/2020	FP	9A	7	2020	Year-3		1	Summer
7/7/2020	FP	11A	7	2020	Year-3		1	Summer
7/7/2020	FP	13A	7	2020	Year-3		1	Summer
7/8/2020	FP	1M	7	2020	Year-3		1	Summer
7/8/2020	FP	2M	7	2020	Year-3		1	Summer
7/8/2020	FP	3M	7	2020	Year-3		1	Summer
7/8/2020	FP	4M	7	2020	Year-3		1	Summer
7/8/2020	FP	5M	7	2020	Year-3		1	Summer

Date	Species	Site	Month	Year	Survey Year	Presence	Data Gap	Season
7/8/2020	FP	6M	7	2020	Year-3		1	Summer
7/8/2020	FP	7M	7	2020	Year-3		1	Summer
7/8/2020	FP	10M	7	2020	Year-3	1	0	Summer
7/8/2020	FP	12M	7	2020	Year-3	1	0	Summer
7/8/2020	FP	14M	7	2020	Year-3	1	0	Summer
7/8/2020	FP	8A	7	2020	Year-3		1	Summer
7/8/2020	FP	9A	7	2020	Year-3		1	Summer
7/8/2020	FP	11A	7	2020	Year-3		1	Summer
7/8/2020	FP	13A	7	2020	Year-3		1	Summer
7/9/2020	FP	1M	7	2020	Year-3		1	Summer
7/9/2020	FP	2M	7	2020	Year-3	1	0	Summer
7/9/2020	FP	3M	7	2020	Year-3	1	0	Summer
7/9/2020	FP	4M	7	2020	Year-3	1	0	Summer
7/9/2020	FP	5M	7	2020	Year-3		1	Summer
7/9/2020	FP	6M	7	2020	Year-3		1	Summer
7/9/2020	FP	7M	7	2020	Year-3		1	Summer
7/9/2020	FP	10M	7	2020	Year-3	1	0	Summer
7/9/2020	FP	12M	7	2020	Year-3	1	0	Summer
7/9/2020	FP	14M	7	2020	Year-3	1	0	Summer
7/9/2020	FP	8A	7	2020	Year-3		1	Summer
7/9/2020	FP	9A	7	2020	Year-3		1	Summer
7/9/2020	FP	11A	7	2020	Year-3		1	Summer
7/9/2020	FP	13A	7	2020	Year-3		1	Summer
7/10/2020	FP	1M	7	2020	Year-3	0	0	Summer
7/10/2020	FP	2M	7	2020	Year-3		1	Summer
7/10/2020	FP	3M	7	2020	Year-3	1	0	Summer
7/10/2020	FP	4M	7	2020	Year-3	1	0	Summer
7/10/2020	FP	5M	7	2020	Year-3		1	Summer
7/10/2020	FP	6M	7	2020	Year-3		1	Summer
7/10/2020	FP	7M	7	2020	Year-3		1	Summer
7/10/2020	FP	10M	7	2020	Year-3	0	0	Summer
7/10/2020	FP	12M	7	2020	Year-3	1	0	Summer
7/10/2020	FP	14M	7	2020	Year-3	1	0	Summer
7/10/2020	FP	8A	7	2020	Year-3		1	Summer
7/10/2020	FP	9A	7	2020	Year-3		1	Summer
7/10/2020	FP	11A	7	2020	Year-3		1	Summer
7/10/2020	FP	13A	7	2020	Year-3		1	Summer
7/11/2020	FP	1M	7	2020	Year-3	0	0	Summer
7/11/2020	FP	2M	7	2020	Year-3		1	Summer
7/11/2020	FP	3M	7	2020	Year-3	1	0	Summer
7/11/2020	FP	4M	7	2020	Year-3	1	0	Summer
7/11/2020	FP	5M	7	2020	Year-3		1	Summer
7/11/2020	FP	6M	7	2020	Year-3		1	Summer
7/11/2020	FP	7M	7	2020	Year-3		1	Summer

Date	Species	Site	Month	Year	Survey Year	Presence	Data Gap	Season
7/11/2020	FP	10M	7	2020	Year-3	0	0	Summer
7/11/2020	FP	12M	7	2020	Year-3	1	0	Summer
7/11/2020	FP	14M	7	2020	Year-3	1	0	Summer
7/11/2020	FP	8A	7	2020	Year-3		1	Summer
7/11/2020	FP	9A	7	2020	Year-3		1	Summer
7/11/2020	FP	11A	7	2020	Year-3		1	Summer
7/11/2020	FP	13A	7	2020	Year-3		1	Summer
7/12/2020	FP	1M	7	2020	Year-3	1	0	Summer
7/12/2020	FP	2M	7	2020	Year-3		1	Summer
7/12/2020	FP	3M	7	2020	Year-3	1	0	Summer
7/12/2020	FP	4M	7	2020	Year-3	1	0	Summer
7/12/2020	FP	5M	7	2020	Year-3		1	Summer
7/12/2020	FP	6M	7	2020	Year-3		1	Summer
7/12/2020	FP	7M	7	2020	Year-3		1	Summer
7/12/2020	FP	10M	7	2020	Year-3	1	0	Summer
7/12/2020	FP	12M	7	2020	Year-3	1	0	Summer
7/12/2020	FP	14M	7	2020	Year-3	1	0	Summer
7/12/2020	FP	8A	7	2020	Year-3		1	Summer
7/12/2020	FP	9A	7	2020	Year-3		1	Summer
7/12/2020	FP	11A	7	2020	Year-3		1	Summer
7/12/2020	FP	13A	7	2020	Year-3		1	Summer
7/13/2020	FP	1M	7	2020	Year-3	1	0	Summer
7/13/2020	FP	2M	7	2020	Year-3		1	Summer
7/13/2020	FP	3M	7	2020	Year-3	1	0	Summer
7/13/2020	FP	4M	7	2020	Year-3	1	0	Summer
7/13/2020	FP	5M	7	2020	Year-3		1	Summer
7/13/2020	FP	6M	7	2020	Year-3		1	Summer
7/13/2020	FP	7M	7	2020	Year-3		1	Summer
7/13/2020	FP	10M	7	2020	Year-3	1	0	Summer
7/13/2020	FP	12M	7	2020	Year-3	1	0	Summer
7/13/2020	FP	14M	7	2020	Year-3	1	0	Summer
7/13/2020	FP	8A	7	2020	Year-3		1	Summer
7/13/2020	FP	9A	7	2020	Year-3		1	Summer
7/13/2020	FP	11A	7	2020	Year-3		1	Summer
7/13/2020	FP	13A	7	2020	Year-3		1	Summer
7/14/2020	FP	1M	7	2020	Year-3	0	0	Summer
7/14/2020	FP	2M	7	2020	Year-3		1	Summer
7/14/2020	FP	3M	7	2020	Year-3	1	0	Summer
7/14/2020	FP	4M	7	2020	Year-3	1	0	Summer
7/14/2020	FP	5M	7	2020	Year-3		1	Summer
7/14/2020	FP	6M	7	2020	Year-3		1	Summer
7/14/2020	FP	7M	7	2020	Year-3		1	Summer
7/14/2020	FP	10M	7	2020	Year-3	0	0	Summer
7/14/2020	FP	12M	7	2020	Year-3	1	0	Summer

Date	Species	Site	Month	Year	Survey Year	Presence	Data Gap	Season
7/14/2020	FP	14M	7	2020	Year-3	1	0	Summer
7/14/2020	FP	8A	7	2020	Year-3		1	Summer
7/14/2020	FP	9A	7	2020	Year-3		1	Summer
7/14/2020	FP	11A	7	2020	Year-3		1	Summer
7/14/2020	FP	13A	7	2020	Year-3		1	Summer
7/15/2020	FP	1M	7	2020	Year-3	0	0	Summer
7/15/2020	FP	2M	7	2020	Year-3		1	Summer
7/15/2020	FP	3M	7	2020	Year-3	1	0	Summer
7/15/2020	FP	4M	7	2020	Year-3	1	0	Summer
7/15/2020	FP	5M	7	2020	Year-3		1	Summer
7/15/2020	FP	6M	7	2020	Year-3		1	Summer
7/15/2020	FP	7M	7	2020	Year-3		1	Summer
7/15/2020	FP	10M	7	2020	Year-3	0	0	Summer
7/15/2020	FP	12M	7	2020	Year-3	1	0	Summer
7/15/2020	FP	14M	7	2020	Year-3	1	0	Summer
7/15/2020	FP	8A	7	2020	Year-3		1	Summer
7/15/2020	FP	9A	7	2020	Year-3		1	Summer
7/15/2020	FP	11A	7	2020	Year-3		1	Summer
7/15/2020	FP	13A	7	2020	Year-3		1	Summer
7/16/2020	FP	1M	7	2020	Year-3	1	0	Summer
7/16/2020	FP	2M	7	2020	Year-3		1	Summer
7/16/2020	FP	3M	7	2020	Year-3	1	0	Summer
7/16/2020	FP	4M	7	2020	Year-3	1	0	Summer
7/16/2020	FP	5M	7	2020	Year-3		1	Summer
7/16/2020	FP	6M	7	2020	Year-3		1	Summer
7/16/2020	FP	7M	7	2020	Year-3		1	Summer
7/16/2020	FP	10M	7	2020	Year-3	0	0	Summer
7/16/2020	FP	12M	7	2020	Year-3	0	0	Summer
7/16/2020	FP	14M	7	2020	Year-3	1	0	Summer
7/16/2020	FP	8A	7	2020	Year-3		1	Summer
7/16/2020	FP	9A	7	2020	Year-3		1	Summer
7/16/2020	FP	11A	7	2020	Year-3		1	Summer
7/16/2020	FP	13A	7	2020	Year-3		1	Summer
7/17/2020	FP	1M	7	2020	Year-3	1	0	Summer
7/17/2020	FP	2M	7	2020	Year-3		1	Summer
7/17/2020	FP	3M	7	2020	Year-3	1	0	Summer
7/17/2020	FP	4M	7	2020	Year-3	1	0	Summer
7/17/2020	FP	5M	7	2020	Year-3		1	Summer
7/17/2020	FP	6M	7	2020	Year-3		1	Summer
7/17/2020	FP	7M	7	2020	Year-3		1	Summer
7/17/2020	FP	10M	7	2020	Year-3	0	0	Summer
7/17/2020	FP	12M	7	2020	Year-3	1	0	Summer
7/17/2020	FP	14M	7	2020	Year-3	1	0	Summer
7/17/2020	FP	8A	7	2020	Year-3		1	Summer

Date	Species	Site	Month	Year	Survey Year	Presence	Data Gap	Season
7/17/2020	FP	9A	7	2020	Year-3		1	Summer
7/17/2020	FP	11A	7	2020	Year-3		1	Summer
7/17/2020	FP	13A	7	2020	Year-3		1	Summer
7/18/2020	FP	1M	7	2020	Year-3	1	0	Summer
7/18/2020	FP	2M	7	2020	Year-3		1	Summer
7/18/2020	FP	3M	7	2020	Year-3	1	0	Summer
7/18/2020	FP	4M	7	2020	Year-3	1	0	Summer
7/18/2020	FP	5M	7	2020	Year-3		1	Summer
7/18/2020	FP	6M	7	2020	Year-3		1	Summer
7/18/2020	FP	7M	7	2020	Year-3		1	Summer
7/18/2020	FP	10M	7	2020	Year-3	1	0	Summer
7/18/2020	FP	12M	7	2020	Year-3	1	0	Summer
7/18/2020	FP	14M	7	2020	Year-3	1	0	Summer
7/18/2020	FP	8A	7	2020	Year-3		1	Summer
7/18/2020	FP	9A	7	2020	Year-3		1	Summer
7/18/2020	FP	11A	7	2020	Year-3		1	Summer
7/18/2020	FP	13A	7	2020	Year-3		1	Summer
7/19/2020	FP	1M	7	2020	Year-3	1	0	Summer
7/19/2020	FP	2M	7	2020	Year-3		1	Summer
7/19/2020	FP	3M	7	2020	Year-3	1	0	Summer
7/19/2020	FP	4M	7	2020	Year-3	1	0	Summer
7/19/2020	FP	5M	7	2020	Year-3		1	Summer
7/19/2020	FP	6M	7	2020	Year-3		1	Summer
7/19/2020	FP	7M	7	2020	Year-3		1	Summer
7/19/2020	FP	10M	7	2020	Year-3	0	0	Summer
7/19/2020	FP	12M	7	2020	Year-3	1	0	Summer
7/19/2020	FP	14M	7	2020	Year-3	1	0	Summer
7/19/2020	FP	8A	7	2020	Year-3		1	Summer
7/19/2020	FP	9A	7	2020	Year-3		1	Summer
7/19/2020	FP	11A	7	2020	Year-3		1	Summer
7/19/2020	FP	13A	7	2020	Year-3		1	Summer
7/20/2020	FP	1M	7	2020	Year-3	1	0	Summer
7/20/2020	FP	2M	7	2020	Year-3		1	Summer
7/20/2020	FP	3M	7	2020	Year-3	1	0	Summer
7/20/2020	FP	4M	7	2020	Year-3	1	0	Summer
7/20/2020	FP	5M	7	2020	Year-3		1	Summer
7/20/2020	FP	6M	7	2020	Year-3		1	Summer
7/20/2020	FP	7M	7	2020	Year-3		1	Summer
7/20/2020	FP	10M	7	2020	Year-3	1	0	Summer
7/20/2020	FP	12M	7	2020	Year-3	1	0	Summer
7/20/2020	FP	14M	7	2020	Year-3	1	0	Summer
7/20/2020	FP	8A	7	2020	Year-3		1	Summer
7/20/2020	FP	9A	7	2020	Year-3		1	Summer
7/20/2020	FP	11A	7	2020	Year-3		1	Summer

Date	Species	Site	Month	Year	Survey Year	Presence	Data Gap	Season
7/20/2020	FP	13A	7	2020	Year-3		1	Summer
7/21/2020	FP	1M	7	2020	Year-3	0	0	Summer
7/21/2020	FP	2M	7	2020	Year-3		1	Summer
7/21/2020	FP	3M	7	2020	Year-3	1	0	Summer
7/21/2020	FP	4M	7	2020	Year-3	1	0	Summer
7/21/2020	FP	5M	7	2020	Year-3		1	Summer
7/21/2020	FP	6M	7	2020	Year-3		1	Summer
7/21/2020	FP	7M	7	2020	Year-3		1	Summer
7/21/2020	FP	10M	7	2020	Year-3	1	0	Summer
7/21/2020	FP	12M	7	2020	Year-3	1	0	Summer
7/21/2020	FP	14M	7	2020	Year-3	1	0	Summer
7/21/2020	FP	8A	7	2020	Year-3		1	Summer
7/21/2020	FP	9A	7	2020	Year-3		1	Summer
7/21/2020	FP	11A	7	2020	Year-3		1	Summer
7/21/2020	FP	13A	7	2020	Year-3		1	Summer
7/22/2020	FP	1M	7	2020	Year-3	1	0	Summer
7/22/2020	FP	2M	7	2020	Year-3		1	Summer
7/22/2020	FP	3M	7	2020	Year-3	1	0	Summer
7/22/2020	FP	4M	7	2020	Year-3	1	0	Summer
7/22/2020	FP	5M	7	2020	Year-3		1	Summer
7/22/2020	FP	6M	7	2020	Year-3		1	Summer
7/22/2020	FP	7M	7	2020	Year-3		1	Summer
7/22/2020	FP	10M	7	2020	Year-3	1	0	Summer
7/22/2020	FP	12M	7	2020	Year-3	1	0	Summer
7/22/2020	FP	14M	7	2020	Year-3	1	0	Summer
7/22/2020	FP	8A	7	2020	Year-3		1	Summer
7/22/2020	FP	9A	7	2020	Year-3		1	Summer
7/22/2020	FP	11A	7	2020	Year-3		1	Summer
7/22/2020	FP	13A	7	2020	Year-3		1	Summer
7/23/2020	FP	1M	7	2020	Year-3	1	0	Summer
7/23/2020	FP	2M	7	2020	Year-3		1	Summer
7/23/2020	FP	3M	7	2020	Year-3	1	0	Summer
7/23/2020	FP	4M	7	2020	Year-3	1	0	Summer
7/23/2020	FP	5M	7	2020	Year-3		1	Summer
7/23/2020	FP	6M	7	2020	Year-3		1	Summer
7/23/2020	FP	7M	7	2020	Year-3		1	Summer
7/23/2020	FP	10M	7	2020	Year-3	1	0	Summer
7/23/2020	FP	12M	7	2020	Year-3	1	0	Summer
7/23/2020	FP	14M	7	2020	Year-3	1	0	Summer
7/23/2020	FP	8A	7	2020	Year-3		1	Summer
7/23/2020	FP	9A	7	2020	Year-3		1	Summer
7/23/2020	FP	11A	7	2020	Year-3		1	Summer
7/23/2020	FP	13A	7	2020	Year-3		1	Summer
7/24/2020	FP	1M	7	2020	Year-3	1	0	Summer

Date	Species	Site	Month	Year	Survey Year	Presence	Data Gap	Season
7/24/2020	FP	2M	7	2020	Year-3		1	Summer
7/24/2020	FP	3M	7	2020	Year-3	1	0	Summer
7/24/2020	FP	4M	7	2020	Year-3	1	0	Summer
7/24/2020	FP	5M	7	2020	Year-3		1	Summer
7/24/2020	FP	6M	7	2020	Year-3		1	Summer
7/24/2020	FP	7M	7	2020	Year-3		1	Summer
7/24/2020	FP	10M	7	2020	Year-3	1	0	Summer
7/24/2020	FP	12M	7	2020	Year-3	1	0	Summer
7/24/2020	FP	14M	7	2020	Year-3	1	0	Summer
7/24/2020	FP	8A	7	2020	Year-3		1	Summer
7/24/2020	FP	9A	7	2020	Year-3		1	Summer
7/24/2020	FP	11A	7	2020	Year-3		1	Summer
7/24/2020	FP	13A	7	2020	Year-3		1	Summer
7/25/2020	FP	1M	7	2020	Year-3	0	0	Summer
7/25/2020	FP	2M	7	2020	Year-3		1	Summer
7/25/2020	FP	3M	7	2020	Year-3	1	0	Summer
7/25/2020	FP	4M	7	2020	Year-3	1	0	Summer
7/25/2020	FP	5M	7	2020	Year-3		1	Summer
7/25/2020	FP	6M	7	2020	Year-3		1	Summer
7/25/2020	FP	7M	7	2020	Year-3		1	Summer
7/25/2020	FP	10M	7	2020	Year-3	0	0	Summer
7/25/2020	FP	12M	7	2020	Year-3	1	0	Summer
7/25/2020	FP	14M	7	2020	Year-3	1	0	Summer
7/25/2020	FP	8A	7	2020	Year-3		1	Summer
7/25/2020	FP	9A	7	2020	Year-3		1	Summer
7/25/2020	FP	11A	7	2020	Year-3		1	Summer
7/25/2020	FP	13A	7	2020	Year-3		1	Summer
7/26/2020	FP	1M	7	2020	Year-3	0	0	Summer
7/26/2020	FP	2M	7	2020	Year-3		1	Summer
7/26/2020	FP	3M	7	2020	Year-3	1	0	Summer
7/26/2020	FP	4M	7	2020	Year-3	1	0	Summer
7/26/2020	FP	5M	7	2020	Year-3		1	Summer
7/26/2020	FP	6M	7	2020	Year-3		1	Summer
7/26/2020	FP	7M	7	2020	Year-3		1	Summer
7/26/2020	FP	10M	7	2020	Year-3	0	0	Summer
7/26/2020	FP	12M	7	2020	Year-3	1	0	Summer
7/26/2020	FP	14M	7	2020	Year-3	1	0	Summer
7/26/2020	FP	8A	7	2020	Year-3		1	Summer
7/26/2020	FP	9A	7	2020	Year-3		1	Summer
7/26/2020	FP	11A	7	2020	Year-3		1	Summer
7/26/2020	FP	13A	7	2020	Year-3		1	Summer
7/27/2020	FP	1M	7	2020	Year-3	1	0	Summer
7/27/2020	FP	2M	7	2020	Year-3		1	Summer
7/27/2020	FP	3M	7	2020	Year-3	1	0	Summer

Date	Species	Site	Month	Year	Survey Year	Presence	Data Gap	Season
7/27/2020	FP	4M	7	2020	Year-3	1	0	Summer
7/27/2020	FP	5M	7	2020	Year-3		1	Summer
7/27/2020	FP	6M	7	2020	Year-3		1	Summer
7/27/2020	FP	7M	7	2020	Year-3		1	Summer
7/27/2020	FP	10M	7	2020	Year-3	1	0	Summer
7/27/2020	FP	12M	7	2020	Year-3	1	0	Summer
7/27/2020	FP	14M	7	2020	Year-3	1	0	Summer
7/27/2020	FP	8A	7	2020	Year-3		1	Summer
7/27/2020	FP	9A	7	2020	Year-3		1	Summer
7/27/2020	FP	11A	7	2020	Year-3		1	Summer
7/27/2020	FP	13A	7	2020	Year-3		1	Summer
7/28/2020	FP	1M	7	2020	Year-3	1	0	Summer
7/28/2020	FP	2M	7	2020	Year-3		1	Summer
7/28/2020	FP	3M	7	2020	Year-3	1	0	Summer
7/28/2020	FP	4M	7	2020	Year-3	1	0	Summer
7/28/2020	FP	5M	7	2020	Year-3		1	Summer
7/28/2020	FP	6M	7	2020	Year-3		1	Summer
7/28/2020	FP	7M	7	2020	Year-3		1	Summer
7/28/2020	FP	10M	7	2020	Year-3	1	0	Summer
7/28/2020	FP	12M	7	2020	Year-3	1	0	Summer
7/28/2020	FP	14M	7	2020	Year-3	1	0	Summer
7/28/2020	FP	8A	7	2020	Year-3		1	Summer
7/28/2020	FP	9A	7	2020	Year-3		1	Summer
7/28/2020	FP	11A	7	2020	Year-3		1	Summer
7/28/2020	FP	13A	7	2020	Year-3		1	Summer
7/29/2020	FP	1M	7	2020	Year-3	1	0	Summer
7/29/2020	FP	2M	7	2020	Year-3		1	Summer
7/29/2020	FP	3M	7	2020	Year-3	1	0	Summer
7/29/2020	FP	4M	7	2020	Year-3	1	0	Summer
7/29/2020	FP	5M	7	2020	Year-3		1	Summer
7/29/2020	FP	6M	7	2020	Year-3		1	Summer
7/29/2020	FP	7M	7	2020	Year-3		1	Summer
7/29/2020	FP	10M	7	2020	Year-3	0	0	Summer
7/29/2020	FP	12M	7	2020	Year-3	1	0	Summer
7/29/2020	FP	14M	7	2020	Year-3	1	0	Summer
7/29/2020	FP	8A	7	2020	Year-3		1	Summer
7/29/2020	FP	9A	7	2020	Year-3		1	Summer
7/29/2020	FP	11A	7	2020	Year-3		1	Summer
7/29/2020	FP	13A	7	2020	Year-3		1	Summer
7/30/2020	FP	1M	7	2020	Year-3	1	0	Summer
7/30/2020	FP	2M	7	2020	Year-3		1	Summer
7/30/2020	FP	3M	7	2020	Year-3	1	0	Summer
7/30/2020	FP	4M	7	2020	Year-3	1	0	Summer
7/30/2020	FP	5M	7	2020	Year-3		1	Summer

Date	Species	Site	Month	Year	Survey Year	Presence	Data Gap	Season
7/30/2020	FP	6M	7	2020	Year-3		1	Summer
7/30/2020	FP	7M	7	2020	Year-3		1	Summer
7/30/2020	FP	10M	7	2020	Year-3	1	0	Summer
7/30/2020	FP	12M	7	2020	Year-3	1	0	Summer
7/30/2020	FP	14M	7	2020	Year-3	1	0	Summer
7/30/2020	FP	8A	7	2020	Year-3		1	Summer
7/30/2020	FP	9A	7	2020	Year-3		1	Summer
7/30/2020	FP	11A	7	2020	Year-3		1	Summer
7/30/2020	FP	13A	7	2020	Year-3		1	Summer
7/31/2020	FP	1M	7	2020	Year-3	1	0	Summer
7/31/2020	FP	2M	7	2020	Year-3		1	Summer
7/31/2020	FP	3M	7	2020	Year-3	1	0	Summer
7/31/2020	FP	4M	7	2020	Year-3	1	0	Summer
7/31/2020	FP	5M	7	2020	Year-3		1	Summer
7/31/2020	FP	6M	7	2020	Year-3		1	Summer
7/31/2020	FP	7M	7	2020	Year-3		1	Summer
7/31/2020	FP	10M	7	2020	Year-3	1	0	Summer
7/31/2020	FP	12M	7	2020	Year-3	1	0	Summer
7/31/2020	FP	14M	7	2020	Year-3	1	0	Summer
7/31/2020	FP	8A	7	2020	Year-3		1	Summer
7/31/2020	FP	9A	7	2020	Year-3		1	Summer
7/31/2020	FP	11A	7	2020	Year-3		1	Summer
7/31/2020	FP	13A	7	2020	Year-3		1	Summer
8/1/2020	FP	1M	8	2020	Year-3	1	0	Summer
8/1/2020	FP	2M	8	2020	Year-3		1	Summer
8/1/2020	FP	3M	8	2020	Year-3	1	0	Summer
8/1/2020	FP	4M	8	2020	Year-3	1	0	Summer
8/1/2020	FP	5M	8	2020	Year-3		1	Summer
8/1/2020	FP	6M	8	2020	Year-3		1	Summer
8/1/2020	FP	7M	8	2020	Year-3		1	Summer
8/1/2020	FP	10M	8	2020	Year-3	1	0	Summer
8/1/2020	FP	12M	8	2020	Year-3	1	0	Summer
8/1/2020	FP	14M	8	2020	Year-3	1	0	Summer
8/1/2020	FP	8A	8	2020	Year-3		1	Summer
8/1/2020	FP	9A	8	2020	Year-3		1	Summer
8/1/2020	FP	11A	8	2020	Year-3		1	Summer
8/1/2020	FP	13A	8	2020	Year-3		1	Summer
8/2/2020	FP	1M	8	2020	Year-3	1	0	Summer
8/2/2020	FP	2M	8	2020	Year-3		1	Summer
8/2/2020	FP	3M	8	2020	Year-3	1	0	Summer
8/2/2020	FP	4M	8	2020	Year-3	1	0	Summer
8/2/2020	FP	5M	8	2020	Year-3		1	Summer
8/2/2020	FP	6M	8	2020	Year-3		1	Summer
8/2/2020	FP	7M	8	2020	Year-3		1	Summer

Date	Species	Site	Month	Year	Survey Year	Presence	Data Gap	Season
8/2/2020	FP	10M	8	2020	Year-3	1	0	Summer
8/2/2020	FP	12M	8	2020	Year-3	1	0	Summer
8/2/2020	FP	14M	8	2020	Year-3	1	0	Summer
8/2/2020	FP	8A	8	2020	Year-3		1	Summer
8/2/2020	FP	9A	8	2020	Year-3		1	Summer
8/2/2020	FP	11A	8	2020	Year-3		1	Summer
8/2/2020	FP	13A	8	2020	Year-3		1	Summer
8/3/2020	FP	1M	8	2020	Year-3	0	0	Summer
8/3/2020	FP	2M	8	2020	Year-3		1	Summer
8/3/2020	FP	3M	8	2020	Year-3	1	0	Summer
8/3/2020	FP	4M	8	2020	Year-3	1	0	Summer
8/3/2020	FP	5M	8	2020	Year-3		1	Summer
8/3/2020	FP	6M	8	2020	Year-3		1	Summer
8/3/2020	FP	7M	8	2020	Year-3		1	Summer
8/3/2020	FP	10M	8	2020	Year-3	0	0	Summer
8/3/2020	FP	12M	8	2020	Year-3	1	0	Summer
8/3/2020	FP	14M	8	2020	Year-3	1	0	Summer
8/3/2020	FP	8A	8	2020	Year-3		1	Summer
8/3/2020	FP	9A	8	2020	Year-3		1	Summer
8/3/2020	FP	11A	8	2020	Year-3		1	Summer
8/3/2020	FP	13A	8	2020	Year-3		1	Summer
8/4/2020	FP	1M	8	2020	Year-3	1	0	Summer
8/4/2020	FP	2M	8	2020	Year-3		1	Summer
8/4/2020	FP	3M	8	2020	Year-3	1	0	Summer
8/4/2020	FP	4M	8	2020	Year-3	1	0	Summer
8/4/2020	FP	5M	8	2020	Year-3		1	Summer
8/4/2020	FP	6M	8	2020	Year-3		1	Summer
8/4/2020	FP	7M	8	2020	Year-3		1	Summer
8/4/2020	FP	10M	8	2020	Year-3	0	0	Summer
8/4/2020	FP	12M	8	2020	Year-3	1	0	Summer
8/4/2020	FP	14M	8	2020	Year-3	1	0	Summer
8/4/2020	FP	8A	8	2020	Year-3		1	Summer
8/4/2020	FP	9A	8	2020	Year-3		1	Summer
8/4/2020	FP	11A	8	2020	Year-3		1	Summer
8/4/2020	FP	13A	8	2020	Year-3		1	Summer
8/5/2020	FP	1M	8	2020	Year-3	1	0	Summer
8/5/2020	FP	2M	8	2020	Year-3		1	Summer
8/5/2020	FP	3M	8	2020	Year-3	1	0	Summer
8/5/2020	FP	4M	8	2020	Year-3	1	0	Summer
8/5/2020	FP	5M	8	2020	Year-3		1	Summer
8/5/2020	FP	6M	8	2020	Year-3		1	Summer
8/5/2020	FP	7M	8	2020	Year-3		1	Summer
8/5/2020	FP	10M	8	2020	Year-3	0	0	Summer
8/5/2020	FP	12M	8	2020	Year-3	1	0	Summer

Date	Species	Site	Month	Year	Survey Year	Presence	Data Gap	Season
8/5/2020	FP	14M	8	2020	Year-3	1	0	Summer
8/5/2020	FP	8A	8	2020	Year-3		1	Summer
8/5/2020	FP	9A	8	2020	Year-3		1	Summer
8/5/2020	FP	11A	8	2020	Year-3		1	Summer
8/5/2020	FP	13A	8	2020	Year-3		1	Summer
8/6/2020	FP	1M	8	2020	Year-3	1	0	Summer
8/6/2020	FP	2M	8	2020	Year-3		1	Summer
8/6/2020	FP	3M	8	2020	Year-3	1	0	Summer
8/6/2020	FP	4M	8	2020	Year-3	1	0	Summer
8/6/2020	FP	5M	8	2020	Year-3		1	Summer
8/6/2020	FP	6M	8	2020	Year-3		1	Summer
8/6/2020	FP	7M	8	2020	Year-3		1	Summer
8/6/2020	FP	10M	8	2020	Year-3	0	0	Summer
8/6/2020	FP	12M	8	2020	Year-3	1	0	Summer
8/6/2020	FP	14M	8	2020	Year-3	1	0	Summer
8/6/2020	FP	8A	8	2020	Year-3		1	Summer
8/6/2020	FP	9A	8	2020	Year-3		1	Summer
8/6/2020	FP	11A	8	2020	Year-3		1	Summer
8/6/2020	FP	13A	8	2020	Year-3		1	Summer
8/7/2020	FP	1M	8	2020	Year-3	1	0	Summer
8/7/2020	FP	2M	8	2020	Year-3		1	Summer
8/7/2020	FP	3M	8	2020	Year-3	1	0	Summer
8/7/2020	FP	4M	8	2020	Year-3	1	0	Summer
8/7/2020	FP	5M	8	2020	Year-3		1	Summer
8/7/2020	FP	6M	8	2020	Year-3		1	Summer
8/7/2020	FP	7M	8	2020	Year-3		1	Summer
8/7/2020	FP	10M	8	2020	Year-3	0	0	Summer
8/7/2020	FP	12M	8	2020	Year-3	1	0	Summer
8/7/2020	FP	14M	8	2020	Year-3	1	0	Summer
8/7/2020	FP	8A	8	2020	Year-3		1	Summer
8/7/2020	FP	9A	8	2020	Year-3		1	Summer
8/7/2020	FP	11A	8	2020	Year-3		1	Summer
8/7/2020	FP	13A	8	2020	Year-3		1	Summer
8/8/2020	FP	1M	8	2020	Year-3	1	0	Summer
8/8/2020	FP	2M	8	2020	Year-3		1	Summer
8/8/2020	FP	3M	8	2020	Year-3	1	0	Summer
8/8/2020	FP	4M	8	2020	Year-3	1	0	Summer
8/8/2020	FP	5M	8	2020	Year-3		1	Summer
8/8/2020	FP	6M	8	2020	Year-3		1	Summer
8/8/2020	FP	7M	8	2020	Year-3		1	Summer
8/8/2020	FP	10M	8	2020	Year-3	0	0	Summer
8/8/2020	FP	12M	8	2020	Year-3	1	0	Summer
8/8/2020	FP	14M	8	2020	Year-3	1	0	Summer
8/8/2020	FP	8A	8	2020	Year-3		1	Summer

Date	Species	Site	Month	Year	Survey Year	Presence	Data Gap	Season
8/8/2020	FP	9A	8	2020	Year-3		1	Summer
8/8/2020	FP	11A	8	2020	Year-3		1	Summer
8/8/2020	FP	13A	8	2020	Year-3		1	Summer
8/9/2020	FP	1M	8	2020	Year-3	1	0	Summer
8/9/2020	FP	2M	8	2020	Year-3		1	Summer
8/9/2020	FP	3M	8	2020	Year-3	1	0	Summer
8/9/2020	FP	4M	8	2020	Year-3	1	0	Summer
8/9/2020	FP	5M	8	2020	Year-3		1	Summer
8/9/2020	FP	6M	8	2020	Year-3		1	Summer
8/9/2020	FP	7M	8	2020	Year-3		1	Summer
8/9/2020	FP	10M	8	2020	Year-3	0	0	Summer
8/9/2020	FP	12M	8	2020	Year-3	1	0	Summer
8/9/2020	FP	14M	8	2020	Year-3	1	0	Summer
8/9/2020	FP	8A	8	2020	Year-3		1	Summer
8/9/2020	FP	9A	8	2020	Year-3		1	Summer
8/9/2020	FP	11A	8	2020	Year-3		1	Summer
8/9/2020	FP	13A	8	2020	Year-3		1	Summer
8/10/2020	FP	1M	8	2020	Year-3	1	0	Summer
8/10/2020	FP	2M	8	2020	Year-3		1	Summer
8/10/2020	FP	3M	8	2020	Year-3	1	0	Summer
8/10/2020	FP	4M	8	2020	Year-3	1	0	Summer
8/10/2020	FP	5M	8	2020	Year-3		1	Summer
8/10/2020	FP	6M	8	2020	Year-3		1	Summer
8/10/2020	FP	7M	8	2020	Year-3		1	Summer
8/10/2020	FP	10M	8	2020	Year-3	1	0	Summer
8/10/2020	FP	12M	8	2020	Year-3	1	0	Summer
8/10/2020	FP	14M	8	2020	Year-3	1	0	Summer
8/10/2020	FP	8A	8	2020	Year-3		1	Summer
8/10/2020	FP	9A	8	2020	Year-3		1	Summer
8/10/2020	FP	11A	8	2020	Year-3		1	Summer
8/10/2020	FP	13A	8	2020	Year-3		1	Summer
8/11/2020	FP	1M	8	2020	Year-3	1	0	Summer
8/11/2020	FP	2M	8	2020	Year-3		1	Summer
8/11/2020	FP	3M	8	2020	Year-3	1	0	Summer
8/11/2020	FP	4M	8	2020	Year-3	1	0	Summer
8/11/2020	FP	5M	8	2020	Year-3		1	Summer
8/11/2020	FP	6M	8	2020	Year-3		1	Summer
8/11/2020	FP	7M	8	2020	Year-3		1	Summer
8/11/2020	FP	10M	8	2020	Year-3	0	0	Summer
8/11/2020	FP	12M	8	2020	Year-3	1	0	Summer
8/11/2020	FP	14M	8	2020	Year-3	1	0	Summer
8/11/2020	FP	8A	8	2020	Year-3		1	Summer
8/11/2020	FP	9A	8	2020	Year-3		1	Summer
8/11/2020	FP	11A	8	2020	Year-3		1	Summer

Date	Species	Site	Month	Year	Survey Year	Presence	Data Gap	Season
8/11/2020	FP	13A	8	2020	Year-3		1	Summer
8/12/2020	FP	1M	8	2020	Year-3	1	0	Summer
8/12/2020	FP	2M	8	2020	Year-3		1	Summer
8/12/2020	FP	3M	8	2020	Year-3	1	0	Summer
8/12/2020	FP	4M	8	2020	Year-3	1	0	Summer
8/12/2020	FP	5M	8	2020	Year-3		1	Summer
8/12/2020	FP	6M	8	2020	Year-3		1	Summer
8/12/2020	FP	7M	8	2020	Year-3		1	Summer
8/12/2020	FP	10M	8	2020	Year-3	1	0	Summer
8/12/2020	FP	12M	8	2020	Year-3	1	0	Summer
8/12/2020	FP	14M	8	2020	Year-3	1	0	Summer
8/12/2020	FP	8A	8	2020	Year-3		1	Summer
8/12/2020	FP	9A	8	2020	Year-3		1	Summer
8/12/2020	FP	11A	8	2020	Year-3		1	Summer
8/12/2020	FP	13A	8	2020	Year-3		1	Summer
8/13/2020	FP	1M	8	2020	Year-3	1	0	Summer
8/13/2020	FP	2M	8	2020	Year-3		1	Summer
8/13/2020	FP	3M	8	2020	Year-3	1	0	Summer
8/13/2020	FP	4M	8	2020	Year-3	1	0	Summer
8/13/2020	FP	5M	8	2020	Year-3		1	Summer
8/13/2020	FP	6M	8	2020	Year-3		1	Summer
8/13/2020	FP	7M	8	2020	Year-3		1	Summer
8/13/2020	FP	10M	8	2020	Year-3	1	0	Summer
8/13/2020	FP	12M	8	2020	Year-3	1	0	Summer
8/13/2020	FP	14M	8	2020	Year-3	1	0	Summer
8/13/2020	FP	8A	8	2020	Year-3		1	Summer
8/13/2020	FP	9A	8	2020	Year-3		1	Summer
8/13/2020	FP	11A	8	2020	Year-3		1	Summer
8/13/2020	FP	13A	8	2020	Year-3		1	Summer
8/14/2020	FP	1M	8	2020	Year-3	1	0	Summer
8/14/2020	FP	2M	8	2020	Year-3		1	Summer
8/14/2020	FP	3M	8	2020	Year-3	1	0	Summer
8/14/2020	FP	4M	8	2020	Year-3	1	0	Summer
8/14/2020	FP	5M	8	2020	Year-3		1	Summer
8/14/2020	FP	6M	8	2020	Year-3		1	Summer
8/14/2020	FP	7M	8	2020	Year-3		1	Summer
8/14/2020	FP	10M	8	2020	Year-3	1	0	Summer
8/14/2020	FP	12M	8	2020	Year-3	1	0	Summer
8/14/2020	FP	14M	8	2020	Year-3	1	0	Summer
8/14/2020	FP	8A	8	2020	Year-3		1	Summer
8/14/2020	FP	9A	8	2020	Year-3		1	Summer
8/14/2020	FP	11A	8	2020	Year-3		1	Summer
8/14/2020	FP	13A	8	2020	Year-3		1	Summer
8/15/2020	FP	1M	8	2020	Year-3	1	0	Summer

Date	Species	Site	Month	Year	Survey Year	Presence	Data Gap	Season
8/15/2020	FP	2M	8	2020	Year-3		1	Summer
8/15/2020	FP	3M	8	2020	Year-3	1	0	Summer
8/15/2020	FP	4M	8	2020	Year-3	1	0	Summer
8/15/2020	FP	5M	8	2020	Year-3		1	Summer
8/15/2020	FP	6M	8	2020	Year-3		1	Summer
8/15/2020	FP	7M	8	2020	Year-3		1	Summer
8/15/2020	FP	10M	8	2020	Year-3	0	0	Summer
8/15/2020	FP	12M	8	2020	Year-3	1	0	Summer
8/15/2020	FP	14M	8	2020	Year-3	1	0	Summer
8/15/2020	FP	8A	8	2020	Year-3		1	Summer
8/15/2020	FP	9A	8	2020	Year-3		1	Summer
8/15/2020	FP	11A	8	2020	Year-3		1	Summer
8/15/2020	FP	13A	8	2020	Year-3		1	Summer
8/16/2020	FP	1M	8	2020	Year-3	1	0	Summer
8/16/2020	FP	2M	8	2020	Year-3		1	Summer
8/16/2020	FP	3M	8	2020	Year-3	1	0	Summer
8/16/2020	FP	4M	8	2020	Year-3	1	0	Summer
8/16/2020	FP	5M	8	2020	Year-3		1	Summer
8/16/2020	FP	6M	8	2020	Year-3		1	Summer
8/16/2020	FP	7M	8	2020	Year-3		1	Summer
8/16/2020	FP	10M	8	2020	Year-3	1	0	Summer
8/16/2020	FP	12M	8	2020	Year-3	1	0	Summer
8/16/2020	FP	14M	8	2020	Year-3	1	0	Summer
8/16/2020	FP	8A	8	2020	Year-3		1	Summer
8/16/2020	FP	9A	8	2020	Year-3		1	Summer
8/16/2020	FP	11A	8	2020	Year-3		1	Summer
8/16/2020	FP	13A	8	2020	Year-3		1	Summer
8/17/2020	FP	1M	8	2020	Year-3	1	0	Summer
8/17/2020	FP	2M	8	2020	Year-3		1	Summer
8/17/2020	FP	3M	8	2020	Year-3	1	0	Summer
8/17/2020	FP	4M	8	2020	Year-3	1	0	Summer
8/17/2020	FP	5M	8	2020	Year-3		1	Summer
8/17/2020	FP	6M	8	2020	Year-3		1	Summer
8/17/2020	FP	7M	8	2020	Year-3		1	Summer
8/17/2020	FP	10M	8	2020	Year-3	1	0	Summer
8/17/2020	FP	12M	8	2020	Year-3	1	0	Summer
8/17/2020	FP	14M	8	2020	Year-3	1	0	Summer
8/17/2020	FP	8A	8	2020	Year-3		1	Summer
8/17/2020	FP	9A	8	2020	Year-3		1	Summer
8/17/2020	FP	11A	8	2020	Year-3		1	Summer
8/17/2020	FP	13A	8	2020	Year-3		1	Summer
8/18/2020	FP	1M	8	2020	Year-3	1	0	Summer
8/18/2020	FP	2M	8	2020	Year-3		1	Summer
8/18/2020	FP	3M	8	2020	Year-3	1	0	Summer

Date	Species	Site	Month	Year	Survey Year	Presence	Data Gap	Season
8/18/2020	FP	4M	8	2020	Year-3	1	0	Summer
8/18/2020	FP	5M	8	2020	Year-3		1	Summer
8/18/2020	FP	6M	8	2020	Year-3		1	Summer
8/18/2020	FP	7M	8	2020	Year-3		1	Summer
8/18/2020	FP	10M	8	2020	Year-3	0	0	Summer
8/18/2020	FP	12M	8	2020	Year-3	1	0	Summer
8/18/2020	FP	14M	8	2020	Year-3	1	0	Summer
8/18/2020	FP	8A	8	2020	Year-3		1	Summer
8/18/2020	FP	9A	8	2020	Year-3		1	Summer
8/18/2020	FP	11A	8	2020	Year-3		1	Summer
8/18/2020	FP	13A	8	2020	Year-3		1	Summer
8/19/2020	FP	1M	8	2020	Year-3	1	0	Summer
8/19/2020	FP	2M	8	2020	Year-3		1	Summer
8/19/2020	FP	3M	8	2020	Year-3	1	0	Summer
8/19/2020	FP	4M	8	2020	Year-3	1	0	Summer
8/19/2020	FP	5M	8	2020	Year-3		1	Summer
8/19/2020	FP	6M	8	2020	Year-3		1	Summer
8/19/2020	FP	7M	8	2020	Year-3		1	Summer
8/19/2020	FP	10M	8	2020	Year-3	0	0	Summer
8/19/2020	FP	12M	8	2020	Year-3	1	0	Summer
8/19/2020	FP	14M	8	2020	Year-3	1	0	Summer
8/19/2020	FP	8A	8	2020	Year-3		1	Summer
8/19/2020	FP	9A	8	2020	Year-3		1	Summer
8/19/2020	FP	11A	8	2020	Year-3		1	Summer
8/19/2020	FP	13A	8	2020	Year-3		1	Summer
8/20/2020	FP	1M	8	2020	Year-3	1	0	Summer
8/20/2020	FP	2M	8	2020	Year-3		1	Summer
8/20/2020	FP	3M	8	2020	Year-3	1	0	Summer
8/20/2020	FP	4M	8	2020	Year-3	1	0	Summer
8/20/2020	FP	5M	8	2020	Year-3		1	Summer
8/20/2020	FP	6M	8	2020	Year-3		1	Summer
8/20/2020	FP	7M	8	2020	Year-3		1	Summer
8/20/2020	FP	10M	8	2020	Year-3	1	0	Summer
8/20/2020	FP	12M	8	2020	Year-3	1	0	Summer
8/20/2020	FP	14M	8	2020	Year-3	1	0	Summer
8/20/2020	FP	8A	8	2020	Year-3		1	Summer
8/20/2020	FP	9A	8	2020	Year-3		1	Summer
8/20/2020	FP	11A	8	2020	Year-3		1	Summer
8/20/2020	FP	13A	8	2020	Year-3		1	Summer
8/21/2020	FP	1M	8	2020	Year-3	1	0	Summer
8/21/2020	FP	2M	8	2020	Year-3		1	Summer
8/21/2020	FP	3M	8	2020	Year-3	1	0	Summer
8/21/2020	FP	4M	8	2020	Year-3	1	0	Summer
8/21/2020	FP	5M	8	2020	Year-3		1	Summer

Date	Species	Site	Month	Year	Survey Year	Presence	Data Gap	Season
8/21/2020	FP	6M	8	2020	Year-3		1	Summer
8/21/2020	FP	7M	8	2020	Year-3		1	Summer
8/21/2020	FP	10M	8	2020	Year-3	0	0	Summer
8/21/2020	FP	12M	8	2020	Year-3	1	0	Summer
8/21/2020	FP	14M	8	2020	Year-3	1	0	Summer
8/21/2020	FP	8A	8	2020	Year-3		1	Summer
8/21/2020	FP	9A	8	2020	Year-3		1	Summer
8/21/2020	FP	11A	8	2020	Year-3		1	Summer
8/21/2020	FP	13A	8	2020	Year-3		1	Summer
8/22/2020	FP	1M	8	2020	Year-3	1	0	Summer
8/22/2020	FP	2M	8	2020	Year-3		1	Summer
8/22/2020	FP	3M	8	2020	Year-3	1	0	Summer
8/22/2020	FP	4M	8	2020	Year-3	1	0	Summer
8/22/2020	FP	5M	8	2020	Year-3		1	Summer
8/22/2020	FP	6M	8	2020	Year-3		1	Summer
8/22/2020	FP	7M	8	2020	Year-3		1	Summer
8/22/2020	FP	10M	8	2020	Year-3	1	0	Summer
8/22/2020	FP	12M	8	2020	Year-3	1	0	Summer
8/22/2020	FP	14M	8	2020	Year-3	1	0	Summer
8/22/2020	FP	8A	8	2020	Year-3		1	Summer
8/22/2020	FP	9A	8	2020	Year-3		1	Summer
8/22/2020	FP	11A	8	2020	Year-3		1	Summer
8/22/2020	FP	13A	8	2020	Year-3		1	Summer
8/23/2020	FP	1M	8	2020	Year-3	1	0	Summer
8/23/2020	FP	2M	8	2020	Year-3		1	Summer
8/23/2020	FP	3M	8	2020	Year-3	1	0	Summer
8/23/2020	FP	4M	8	2020	Year-3	1	0	Summer
8/23/2020	FP	5M	8	2020	Year-3		1	Summer
8/23/2020	FP	6M	8	2020	Year-3		1	Summer
8/23/2020	FP	7M	8	2020	Year-3		1	Summer
8/23/2020	FP	10M	8	2020	Year-3	1	0	Summer
8/23/2020	FP	12M	8	2020	Year-3	1	0	Summer
8/23/2020	FP	14M	8	2020	Year-3	1	0	Summer
8/23/2020	FP	8A	8	2020	Year-3		1	Summer
8/23/2020	FP	9A	8	2020	Year-3		1	Summer
8/23/2020	FP	11A	8	2020	Year-3		1	Summer
8/23/2020	FP	13A	8	2020	Year-3		1	Summer
8/24/2020	FP	1M	8	2020	Year-3	1	0	Summer
8/24/2020	FP	2M	8	2020	Year-3		1	Summer
8/24/2020	FP	3M	8	2020	Year-3	1	0	Summer
8/24/2020	FP	4M	8	2020	Year-3	1	0	Summer
8/24/2020	FP	5M	8	2020	Year-3		1	Summer
8/24/2020	FP	6M	8	2020	Year-3		1	Summer
8/24/2020	FP	7M	8	2020	Year-3		1	Summer

Date	Species	Site	Month	Year	Survey Year	Presence	Data Gap	Season
8/24/2020	FP	10M	8	2020	Year-3	1	0	Summer
8/24/2020	FP	12M	8	2020	Year-3	1	0	Summer
8/24/2020	FP	14M	8	2020	Year-3	1	0	Summer
8/24/2020	FP	8A	8	2020	Year-3		1	Summer
8/24/2020	FP	9A	8	2020	Year-3		1	Summer
8/24/2020	FP	11A	8	2020	Year-3		1	Summer
8/24/2020	FP	13A	8	2020	Year-3		1	Summer
8/25/2020	FP	1M	8	2020	Year-3	1	0	Summer
8/25/2020	FP	2M	8	2020	Year-3		1	Summer
8/25/2020	FP	3M	8	2020	Year-3	1	0	Summer
8/25/2020	FP	4M	8	2020	Year-3	1	0	Summer
8/25/2020	FP	5M	8	2020	Year-3		1	Summer
8/25/2020	FP	6M	8	2020	Year-3		1	Summer
8/25/2020	FP	7M	8	2020	Year-3		1	Summer
8/25/2020	FP	10M	8	2020	Year-3	1	0	Summer
8/25/2020	FP	12M	8	2020	Year-3	1	0	Summer
8/25/2020	FP	14M	8	2020	Year-3	1	0	Summer
8/25/2020	FP	8A	8	2020	Year-3		1	Summer
8/25/2020	FP	9A	8	2020	Year-3		1	Summer
8/25/2020	FP	11A	8	2020	Year-3		1	Summer
8/25/2020	FP	13A	8	2020	Year-3		1	Summer
8/26/2020	FP	1M	8	2020	Year-3	1	0	Summer
8/26/2020	FP	2M	8	2020	Year-3		1	Summer
8/26/2020	FP	3M	8	2020	Year-3	1	0	Summer
8/26/2020	FP	4M	8	2020	Year-3	1	0	Summer
8/26/2020	FP	5M	8	2020	Year-3		1	Summer
8/26/2020	FP	6M	8	2020	Year-3		1	Summer
8/26/2020	FP	7M	8	2020	Year-3		1	Summer
8/26/2020	FP	10M	8	2020	Year-3	0	0	Summer
8/26/2020	FP	12M	8	2020	Year-3	1	0	Summer
8/26/2020	FP	14M	8	2020	Year-3	1	0	Summer
8/26/2020	FP	8A	8	2020	Year-3		1	Summer
8/26/2020	FP	9A	8	2020	Year-3		1	Summer
8/26/2020	FP	11A	8	2020	Year-3		1	Summer
8/26/2020	FP	13A	8	2020	Year-3		1	Summer
8/27/2020	FP	1M	8	2020	Year-3	1	0	Summer
8/27/2020	FP	2M	8	2020	Year-3		1	Summer
8/27/2020	FP	3M	8	2020	Year-3	1	0	Summer
8/27/2020	FP	4M	8	2020	Year-3	1	0	Summer
8/27/2020	FP	5M	8	2020	Year-3		1	Summer
8/27/2020	FP	6M	8	2020	Year-3		1	Summer
8/27/2020	FP	7M	8	2020	Year-3		1	Summer
8/27/2020	FP	10M	8	2020	Year-3	1	0	Summer
8/27/2020	FP	12M	8	2020	Year-3	1	0	Summer

Date	Species	Site	Month	Year	Survey Year	Presence	Data Gap	Season
8/27/2020	FP	14M	8	2020	Year-3	1	0	Summer
8/27/2020	FP	8A	8	2020	Year-3		1	Summer
8/27/2020	FP	9A	8	2020	Year-3		1	Summer
8/27/2020	FP	11A	8	2020	Year-3		1	Summer
8/27/2020	FP	13A	8	2020	Year-3		1	Summer
8/28/2020	FP	1M	8	2020	Year-3	1	0	Summer
8/28/2020	FP	2M	8	2020	Year-3		1	Summer
8/28/2020	FP	3M	8	2020	Year-3	1	0	Summer
8/28/2020	FP	4M	8	2020	Year-3	1	0	Summer
8/28/2020	FP	5M	8	2020	Year-3		1	Summer
8/28/2020	FP	6M	8	2020	Year-3		1	Summer
8/28/2020	FP	7M	8	2020	Year-3		1	Summer
8/28/2020	FP	10M	8	2020	Year-3	1	0	Summer
8/28/2020	FP	12M	8	2020	Year-3	1	0	Summer
8/28/2020	FP	14M	8	2020	Year-3	1	0	Summer
8/28/2020	FP	8A	8	2020	Year-3		1	Summer
8/28/2020	FP	9A	8	2020	Year-3		1	Summer
8/28/2020	FP	11A	8	2020	Year-3		1	Summer
8/28/2020	FP	13A	8	2020	Year-3		1	Summer
8/29/2020	FP	1M	8	2020	Year-3	1	0	Summer
8/29/2020	FP	2M	8	2020	Year-3		1	Summer
8/29/2020	FP	3M	8	2020	Year-3	1	0	Summer
8/29/2020	FP	4M	8	2020	Year-3	1	0	Summer
8/29/2020	FP	5M	8	2020	Year-3		1	Summer
8/29/2020	FP	6M	8	2020	Year-3		1	Summer
8/29/2020	FP	7M	8	2020	Year-3		1	Summer
8/29/2020	FP	10M	8	2020	Year-3	0	0	Summer
8/29/2020	FP	12M	8	2020	Year-3	1	0	Summer
8/29/2020	FP	14M	8	2020	Year-3	1	0	Summer
8/29/2020	FP	8A	8	2020	Year-3		1	Summer
8/29/2020	FP	9A	8	2020	Year-3		1	Summer
8/29/2020	FP	11A	8	2020	Year-3		1	Summer
8/29/2020	FP	13A	8	2020	Year-3		1	Summer
8/30/2020	FP	1M	8	2020	Year-3	1	0	Summer
8/30/2020	FP	2M	8	2020	Year-3		1	Summer
8/30/2020	FP	3M	8	2020	Year-3	1	0	Summer
8/30/2020	FP	4M	8	2020	Year-3	1	0	Summer
8/30/2020	FP	5M	8	2020	Year-3		1	Summer
8/30/2020	FP	6M	8	2020	Year-3		1	Summer
8/30/2020	FP	7M	8	2020	Year-3		1	Summer
8/30/2020	FP	10M	8	2020	Year-3	1	0	Summer
8/30/2020	FP	12M	8	2020	Year-3	1	0	Summer
8/30/2020	FP	14M	8	2020	Year-3	1	0	Summer
8/30/2020	FP	8A	8	2020	Year-3		1	Summer

Date	Species	Site	Month	Year	Survey Year	Presence	Data Gap	Season
8/30/2020	FP	9A	8	2020	Year-3		1	Summer
8/30/2020	FP	11A	8	2020	Year-3		1	Summer
8/30/2020	FP	13A	8	2020	Year-3		1	Summer
8/31/2020	FP	1M	8	2020	Year-3	1	0	Summer
8/31/2020	FP	2M	8	2020	Year-3		1	Summer
8/31/2020	FP	3M	8	2020	Year-3	1	0	Summer
8/31/2020	FP	4M	8	2020	Year-3	1	0	Summer
8/31/2020	FP	5M	8	2020	Year-3		1	Summer
8/31/2020	FP	6M	8	2020	Year-3		1	Summer
8/31/2020	FP	7M	8	2020	Year-3		1	Summer
8/31/2020	FP	10M	8	2020	Year-3	0	0	Summer
8/31/2020	FP	12M	8	2020	Year-3	1	0	Summer
8/31/2020	FP	14M	8	2020	Year-3	1	0	Summer
8/31/2020	FP	8A	8	2020	Year-3		1	Summer
8/31/2020	FP	9A	8	2020	Year-3		1	Summer
8/31/2020	FP	11A	8	2020	Year-3		1	Summer
8/31/2020	FP	13A	8	2020	Year-3		1	Summer
9/1/2020	FP	1M	9	2020	Year-3	1	0	Fall
9/1/2020	FP	2M	9	2020	Year-3		1	Fall
9/1/2020	FP	3M	9	2020	Year-3	1	0	Fall
9/1/2020	FP	4M	9	2020	Year-3	1	0	Fall
9/1/2020	FP	5M	9	2020	Year-3		1	Fall
9/1/2020	FP	6M	9	2020	Year-3		1	Fall
9/1/2020	FP	7M	9	2020	Year-3		1	Fall
9/1/2020	FP	10M	9	2020	Year-3	1	0	Fall
9/1/2020	FP	12M	9	2020	Year-3	1	0	Fall
9/1/2020	FP	14M	9	2020	Year-3	1	0	Fall
9/1/2020	FP	8A	9	2020	Year-3		1	Fall
9/1/2020	FP	9A	9	2020	Year-3		1	Fall
9/1/2020	FP	11A	9	2020	Year-3		1	Fall
9/1/2020	FP	13A	9	2020	Year-3		1	Fall
9/2/2020	FP	1M	9	2020	Year-3	1	0	Fall
9/2/2020	FP	2M	9	2020	Year-3		1	Fall
9/2/2020	FP	3M	9	2020	Year-3	1	0	Fall
9/2/2020	FP	4M	9	2020	Year-3	1	0	Fall
9/2/2020	FP	5M	9	2020	Year-3		1	Fall
9/2/2020	FP	6M	9	2020	Year-3		1	Fall
9/2/2020	FP	7M	9	2020	Year-3		1	Fall
9/2/2020	FP	10M	9	2020	Year-3	1	0	Fall
9/2/2020	FP	12M	9	2020	Year-3	1	0	Fall
9/2/2020	FP	14M	9	2020	Year-3	1	0	Fall
9/2/2020	FP	8A	9	2020	Year-3		1	Fall
9/2/2020	FP	9A	9	2020	Year-3		1	Fall
9/2/2020	FP	11A	9	2020	Year-3		1	Fall

Date	Species	Site	Month	Year	Survey Year	Presence	Data Gap	Season
9/2/2020	FP	13A	9	2020	Year-3		1	Fall
9/3/2020	FP	1M	9	2020	Year-3	1	0	Fall
9/3/2020	FP	2M	9	2020	Year-3		1	Fall
9/3/2020	FP	3M	9	2020	Year-3	1	0	Fall
9/3/2020	FP	4M	9	2020	Year-3	1	0	Fall
9/3/2020	FP	5M	9	2020	Year-3		1	Fall
9/3/2020	FP	6M	9	2020	Year-3		1	Fall
9/3/2020	FP	7M	9	2020	Year-3		1	Fall
9/3/2020	FP	10M	9	2020	Year-3	1	0	Fall
9/3/2020	FP	12M	9	2020	Year-3	1	0	Fall
9/3/2020	FP	14M	9	2020	Year-3	1	0	Fall
9/3/2020	FP	8A	9	2020	Year-3		1	Fall
9/3/2020	FP	9A	9	2020	Year-3		1	Fall
9/3/2020	FP	11A	9	2020	Year-3		1	Fall
9/3/2020	FP	13A	9	2020	Year-3		1	Fall
9/4/2020	FP	1M	9	2020	Year-3	1	0	Fall
9/4/2020	FP	2M	9	2020	Year-3		1	Fall
9/4/2020	FP	3M	9	2020	Year-3	1	0	Fall
9/4/2020	FP	4M	9	2020	Year-3	1	0	Fall
9/4/2020	FP	5M	9	2020	Year-3		1	Fall
9/4/2020	FP	6M	9	2020	Year-3		1	Fall
9/4/2020	FP	7M	9	2020	Year-3		1	Fall
9/4/2020	FP	10M	9	2020	Year-3	1	0	Fall
9/4/2020	FP	12M	9	2020	Year-3	1	0	Fall
9/4/2020	FP	14M	9	2020	Year-3	1	0	Fall
9/4/2020	FP	8A	9	2020	Year-3		1	Fall
9/4/2020	FP	9A	9	2020	Year-3		1	Fall
9/4/2020	FP	11A	9	2020	Year-3		1	Fall
9/4/2020	FP	13A	9	2020	Year-3		1	Fall
9/5/2020	FP	1M	9	2020	Year-3	1	0	Fall
9/5/2020	FP	2M	9	2020	Year-3		1	Fall
9/5/2020	FP	3M	9	2020	Year-3	1	0	Fall
9/5/2020	FP	4M	9	2020	Year-3	1	0	Fall
9/5/2020	FP	5M	9	2020	Year-3		1	Fall
9/5/2020	FP	6M	9	2020	Year-3		1	Fall
9/5/2020	FP	7M	9	2020	Year-3		1	Fall
9/5/2020	FP	10M	9	2020	Year-3	0	0	Fall
9/5/2020	FP	12M	9	2020	Year-3	1	0	Fall
9/5/2020	FP	14M	9	2020	Year-3	1	0	Fall
9/5/2020	FP	8A	9	2020	Year-3		1	Fall
9/5/2020	FP	9A	9	2020	Year-3		1	Fall
9/5/2020	FP	11A	9	2020	Year-3		1	Fall
9/5/2020	FP	13A	9	2020	Year-3		1	Fall
9/6/2020	FP	1M	9	2020	Year-3	1	0	Fall

Date	Species	Site	Month	Year	Survey Year	Presence	Data Gap	Season
9/6/2020	FP	2M	9	2020	Year-3		1	Fall
9/6/2020	FP	3M	9	2020	Year-3	1	0	Fall
9/6/2020	FP	4M	9	2020	Year-3	1	0	Fall
9/6/2020	FP	5M	9	2020	Year-3		1	Fall
9/6/2020	FP	6M	9	2020	Year-3		1	Fall
9/6/2020	FP	7M	9	2020	Year-3		1	Fall
9/6/2020	FP	10M	9	2020	Year-3	1	0	Fall
9/6/2020	FP	12M	9	2020	Year-3	1	0	Fall
9/6/2020	FP	14M	9	2020	Year-3	1	0	Fall
9/6/2020	FP	8A	9	2020	Year-3		1	Fall
9/6/2020	FP	9A	9	2020	Year-3		1	Fall
9/6/2020	FP	11A	9	2020	Year-3		1	Fall
9/6/2020	FP	13A	9	2020	Year-3		1	Fall
9/7/2020	FP	1M	9	2020	Year-3	1	0	Fall
9/7/2020	FP	2M	9	2020	Year-3		1	Fall
9/7/2020	FP	3M	9	2020	Year-3	1	0	Fall
9/7/2020	FP	4M	9	2020	Year-3	1	0	Fall
9/7/2020	FP	5M	9	2020	Year-3		1	Fall
9/7/2020	FP	6M	9	2020	Year-3		1	Fall
9/7/2020	FP	7M	9	2020	Year-3		1	Fall
9/7/2020	FP	10M	9	2020	Year-3	1	0	Fall
9/7/2020	FP	12M	9	2020	Year-3	1	0	Fall
9/7/2020	FP	14M	9	2020	Year-3	1	0	Fall
9/7/2020	FP	8A	9	2020	Year-3		1	Fall
9/7/2020	FP	9A	9	2020	Year-3		1	Fall
9/7/2020	FP	11A	9	2020	Year-3		1	Fall
9/7/2020	FP	13A	9	2020	Year-3		1	Fall
9/8/2020	FP	1M	9	2020	Year-3	1	0	Fall
9/8/2020	FP	2M	9	2020	Year-3		1	Fall
9/8/2020	FP	3M	9	2020	Year-3	1	0	Fall
9/8/2020	FP	4M	9	2020	Year-3	1	0	Fall
9/8/2020	FP	5M	9	2020	Year-3		1	Fall
9/8/2020	FP	6M	9	2020	Year-3		1	Fall
9/8/2020	FP	7M	9	2020	Year-3		1	Fall
9/8/2020	FP	10M	9	2020	Year-3	1	0	Fall
9/8/2020	FP	12M	9	2020	Year-3	1	0	Fall
9/8/2020	FP	14M	9	2020	Year-3	1	0	Fall
9/8/2020	FP	8A	9	2020	Year-3		1	Fall
9/8/2020	FP	9A	9	2020	Year-3		1	Fall
9/8/2020	FP	11A	9	2020	Year-3		1	Fall
9/8/2020	FP	13A	9	2020	Year-3		1	Fall
9/9/2020	FP	1M	9	2020	Year-3	1	0	Fall
9/9/2020	FP	2M	9	2020	Year-3		1	Fall
9/9/2020	FP	3M	9	2020	Year-3	1	0	Fall

Date	Species	Site	Month	Year	Survey Year	Presence	Data Gap	Season
9/9/2020	FP	4M	9	2020	Year-3	1	0	Fall
9/9/2020	FP	5M	9	2020	Year-3		1	Fall
9/9/2020	FP	6M	9	2020	Year-3		1	Fall
9/9/2020	FP	7M	9	2020	Year-3		1	Fall
9/9/2020	FP	10M	9	2020	Year-3	1	0	Fall
9/9/2020	FP	12M	9	2020	Year-3	1	0	Fall
9/9/2020	FP	14M	9	2020	Year-3	1	0	Fall
9/9/2020	FP	8A	9	2020	Year-3		1	Fall
9/9/2020	FP	9A	9	2020	Year-3		1	Fall
9/9/2020	FP	11A	9	2020	Year-3		1	Fall
9/9/2020	FP	13A	9	2020	Year-3		1	Fall
9/10/2020	FP	1M	9	2020	Year-3	1	0	Fall
9/10/2020	FP	2M	9	2020	Year-3		1	Fall
9/10/2020	FP	3M	9	2020	Year-3	1	0	Fall
9/10/2020	FP	4M	9	2020	Year-3	1	0	Fall
9/10/2020	FP	5M	9	2020	Year-3		1	Fall
9/10/2020	FP	6M	9	2020	Year-3		1	Fall
9/10/2020	FP	7M	9	2020	Year-3		1	Fall
9/10/2020	FP	10M	9	2020	Year-3	1	0	Fall
9/10/2020	FP	12M	9	2020	Year-3	1	0	Fall
9/10/2020	FP	14M	9	2020	Year-3	1	0	Fall
9/10/2020	FP	8A	9	2020	Year-3		1	Fall
9/10/2020	FP	9A	9	2020	Year-3		1	Fall
9/10/2020	FP	11A	9	2020	Year-3		1	Fall
9/10/2020	FP	13A	9	2020	Year-3		1	Fall
9/11/2020	FP	1M	9	2020	Year-3	1	0	Fall
9/11/2020	FP	2M	9	2020	Year-3		1	Fall
9/11/2020	FP	3M	9	2020	Year-3	1	0	Fall
9/11/2020	FP	4M	9	2020	Year-3	1	0	Fall
9/11/2020	FP	5M	9	2020	Year-3		1	Fall
9/11/2020	FP	6M	9	2020	Year-3		1	Fall
9/11/2020	FP	7M	9	2020	Year-3		1	Fall
9/11/2020	FP	10M	9	2020	Year-3	1	0	Fall
9/11/2020	FP	12M	9	2020	Year-3	1	0	Fall
9/11/2020	FP	14M	9	2020	Year-3	1	0	Fall
9/11/2020	FP	8A	9	2020	Year-3		1	Fall
9/11/2020	FP	9A	9	2020	Year-3		1	Fall
9/11/2020	FP	11A	9	2020	Year-3		1	Fall
9/11/2020	FP	13A	9	2020	Year-3		1	Fall
9/12/2020	FP	1M	9	2020	Year-3	1	0	Fall
9/12/2020	FP	2M	9	2020	Year-3		1	Fall
9/12/2020	FP	3M	9	2020	Year-3	1	0	Fall
9/12/2020	FP	4M	9	2020	Year-3	1	0	Fall
9/12/2020	FP	5M	9	2020	Year-3		1	Fall

Date	Species	Site	Month	Year	Survey Year	Presence	Data Gap	Season
9/12/2020	FP	6M	9	2020	Year-3		1	Fall
9/12/2020	FP	7M	9	2020	Year-3		1	Fall
9/12/2020	FP	10M	9	2020	Year-3	0	0	Fall
9/12/2020	FP	12M	9	2020	Year-3	1	0	Fall
9/12/2020	FP	14M	9	2020	Year-3	1	0	Fall
9/12/2020	FP	8A	9	2020	Year-3		1	Fall
9/12/2020	FP	9A	9	2020	Year-3		1	Fall
9/12/2020	FP	11A	9	2020	Year-3		1	Fall
9/12/2020	FP	13A	9	2020	Year-3		1	Fall
9/13/2020	FP	1M	9	2020	Year-3	1	0	Fall
9/13/2020	FP	2M	9	2020	Year-3		1	Fall
9/13/2020	FP	3M	9	2020	Year-3	1	0	Fall
9/13/2020	FP	4M	9	2020	Year-3	1	0	Fall
9/13/2020	FP	5M	9	2020	Year-3		1	Fall
9/13/2020	FP	6M	9	2020	Year-3		1	Fall
9/13/2020	FP	7M	9	2020	Year-3		1	Fall
9/13/2020	FP	10M	9	2020	Year-3	1	0	Fall
9/13/2020	FP	12M	9	2020	Year-3	1	0	Fall
9/13/2020	FP	14M	9	2020	Year-3	1	0	Fall
9/13/2020	FP	8A	9	2020	Year-3		1	Fall
9/13/2020	FP	9A	9	2020	Year-3		1	Fall
9/13/2020	FP	11A	9	2020	Year-3		1	Fall
9/13/2020	FP	13A	9	2020	Year-3		1	Fall
9/14/2020	FP	1M	9	2020	Year-3	1	0	Fall
9/14/2020	FP	2M	9	2020	Year-3		1	Fall
9/14/2020	FP	3M	9	2020	Year-3	1	0	Fall
9/14/2020	FP	4M	9	2020	Year-3	1	0	Fall
9/14/2020	FP	5M	9	2020	Year-3		1	Fall
9/14/2020	FP	6M	9	2020	Year-3		1	Fall
9/14/2020	FP	7M	9	2020	Year-3		1	Fall
9/14/2020	FP	10M	9	2020	Year-3	1	0	Fall
9/14/2020	FP	12M	9	2020	Year-3	1	0	Fall
9/14/2020	FP	14M	9	2020	Year-3	1	0	Fall
9/14/2020	FP	8A	9	2020	Year-3		1	Fall
9/14/2020	FP	9A	9	2020	Year-3		1	Fall
9/14/2020	FP	11A	9	2020	Year-3		1	Fall
9/14/2020	FP	13A	9	2020	Year-3		1	Fall
9/15/2020	FP	1M	9	2020	Year-3	1	0	Fall
9/15/2020	FP	2M	9	2020	Year-3		1	Fall
9/15/2020	FP	3M	9	2020	Year-3	1	0	Fall
9/15/2020	FP	4M	9	2020	Year-3	1	0	Fall
9/15/2020	FP	5M	9	2020	Year-3		1	Fall
9/15/2020	FP	6M	9	2020	Year-3		1	Fall
9/15/2020	FP	7M	9	2020	Year-3		1	Fall

Date	Species	Site	Month	Year	Survey Year	Presence	Data Gap	Season
9/15/2020	FP	10M	9	2020	Year-3	1	0	Fall
9/15/2020	FP	12M	9	2020	Year-3	1	0	Fall
9/15/2020	FP	14M	9	2020	Year-3	1	0	Fall
9/15/2020	FP	8A	9	2020	Year-3		1	Fall
9/15/2020	FP	9A	9	2020	Year-3		1	Fall
9/15/2020	FP	11A	9	2020	Year-3		1	Fall
9/15/2020	FP	13A	9	2020	Year-3		1	Fall
9/16/2020	FP	1M	9	2020	Year-3	1	0	Fall
9/16/2020	FP	2M	9	2020	Year-3		1	Fall
9/16/2020	FP	3M	9	2020	Year-3	1	0	Fall
9/16/2020	FP	4M	9	2020	Year-3	1	0	Fall
9/16/2020	FP	5M	9	2020	Year-3		1	Fall
9/16/2020	FP	6M	9	2020	Year-3		1	Fall
9/16/2020	FP	7M	9	2020	Year-3		1	Fall
9/16/2020	FP	10M	9	2020	Year-3	1	0	Fall
9/16/2020	FP	12M	9	2020	Year-3	1	0	Fall
9/16/2020	FP	14M	9	2020	Year-3	1	0	Fall
9/16/2020	FP	8A	9	2020	Year-3		1	Fall
9/16/2020	FP	9A	9	2020	Year-3		1	Fall
9/16/2020	FP	11A	9	2020	Year-3		1	Fall
9/16/2020	FP	13A	9	2020	Year-3		1	Fall
9/17/2020	FP	1M	9	2020	Year-3	1	0	Fall
9/17/2020	FP	2M	9	2020	Year-3		1	Fall
9/17/2020	FP	3M	9	2020	Year-3	1	0	Fall
9/17/2020	FP	4M	9	2020	Year-3	1	0	Fall
9/17/2020	FP	5M	9	2020	Year-3		1	Fall
9/17/2020	FP	6M	9	2020	Year-3		1	Fall
9/17/2020	FP	7M	9	2020	Year-3		1	Fall
9/17/2020	FP	10M	9	2020	Year-3	1	0	Fall
9/17/2020	FP	12M	9	2020	Year-3	1	0	Fall
9/17/2020	FP	14M	9	2020	Year-3	1	0	Fall
9/17/2020	FP	8A	9	2020	Year-3		1	Fall
9/17/2020	FP	9A	9	2020	Year-3		1	Fall
9/17/2020	FP	11A	9	2020	Year-3		1	Fall
9/17/2020	FP	13A	9	2020	Year-3		1	Fall
9/18/2020	FP	1M	9	2020	Year-3	1	0	Fall
9/18/2020	FP	2M	9	2020	Year-3		1	Fall
9/18/2020	FP	3M	9	2020	Year-3	1	0	Fall
9/18/2020	FP	4M	9	2020	Year-3	1	0	Fall
9/18/2020	FP	5M	9	2020	Year-3		1	Fall
9/18/2020	FP	6M	9	2020	Year-3		1	Fall
9/18/2020	FP	7M	9	2020	Year-3		1	Fall
9/18/2020	FP	10M	9	2020	Year-3	1	0	Fall
9/18/2020	FP	12M	9	2020	Year-3	1	0	Fall

Date	Species	Site	Month	Year	Survey Year	Presence	Data Gap	Season
9/18/2020	FP	14M	9	2020	Year-3	1	0	Fall
9/18/2020	FP	8A	9	2020	Year-3		1	Fall
9/18/2020	FP	9A	9	2020	Year-3		1	Fall
9/18/2020	FP	11A	9	2020	Year-3		1	Fall
9/18/2020	FP	13A	9	2020	Year-3		1	Fall
9/19/2020	FP	1M	9	2020	Year-3	1	0	Fall
9/19/2020	FP	2M	9	2020	Year-3		1	Fall
9/19/2020	FP	3M	9	2020	Year-3	1	0	Fall
9/19/2020	FP	4M	9	2020	Year-3	1	0	Fall
9/19/2020	FP	5M	9	2020	Year-3		1	Fall
9/19/2020	FP	6M	9	2020	Year-3		1	Fall
9/19/2020	FP	7M	9	2020	Year-3		1	Fall
9/19/2020	FP	10M	9	2020	Year-3	1	0	Fall
9/19/2020	FP	12M	9	2020	Year-3	1	0	Fall
9/19/2020	FP	14M	9	2020	Year-3	1	0	Fall
9/19/2020	FP	8A	9	2020	Year-3		1	Fall
9/19/2020	FP	9A	9	2020	Year-3		1	Fall
9/19/2020	FP	11A	9	2020	Year-3		1	Fall
9/19/2020	FP	13A	9	2020	Year-3		1	Fall
9/20/2020	FP	1M	9	2020	Year-3	1	0	Fall
9/20/2020	FP	2M	9	2020	Year-3		1	Fall
9/20/2020	FP	3M	9	2020	Year-3	1	0	Fall
9/20/2020	FP	4M	9	2020	Year-3	1	0	Fall
9/20/2020	FP	5M	9	2020	Year-3		1	Fall
9/20/2020	FP	6M	9	2020	Year-3		1	Fall
9/20/2020	FP	7M	9	2020	Year-3		1	Fall
9/20/2020	FP	10M	9	2020	Year-3	1	0	Fall
9/20/2020	FP	12M	9	2020	Year-3	1	0	Fall
9/20/2020	FP	14M	9	2020	Year-3	1	0	Fall
9/20/2020	FP	8A	9	2020	Year-3		1	Fall
9/20/2020	FP	9A	9	2020	Year-3		1	Fall
9/20/2020	FP	11A	9	2020	Year-3		1	Fall
9/20/2020	FP	13A	9	2020	Year-3		1	Fall
9/21/2020	FP	1M	9	2020	Year-3	1	0	Fall
9/21/2020	FP	2M	9	2020	Year-3		1	Fall
9/21/2020	FP	3M	9	2020	Year-3	1	0	Fall
9/21/2020	FP	4M	9	2020	Year-3	1	0	Fall
9/21/2020	FP	5M	9	2020	Year-3		1	Fall
9/21/2020	FP	6M	9	2020	Year-3		1	Fall
9/21/2020	FP	7M	9	2020	Year-3		1	Fall
9/21/2020	FP	10M	9	2020	Year-3	0	0	Fall
9/21/2020	FP	12M	9	2020	Year-3	1	0	Fall
9/21/2020	FP	14M	9	2020	Year-3	1	0	Fall
9/21/2020	FP	8A	9	2020	Year-3		1	Fall

Date	Species	Site	Month	Year	Survey Year	Presence	Data Gap	Season
9/21/2020	FP	9A	9	2020	Year-3		1	Fall
9/21/2020	FP	11A	9	2020	Year-3		1	Fall
9/21/2020	FP	13A	9	2020	Year-3		1	Fall
9/22/2020	FP	1M	9	2020	Year-3	1	0	Fall
9/22/2020	FP	2M	9	2020	Year-3		1	Fall
9/22/2020	FP	3M	9	2020	Year-3	1	0	Fall
9/22/2020	FP	4M	9	2020	Year-3	1	0	Fall
9/22/2020	FP	5M	9	2020	Year-3		1	Fall
9/22/2020	FP	6M	9	2020	Year-3		1	Fall
9/22/2020	FP	7M	9	2020	Year-3		1	Fall
9/22/2020	FP	10M	9	2020	Year-3	1	0	Fall
9/22/2020	FP	12M	9	2020	Year-3	1	0	Fall
9/22/2020	FP	14M	9	2020	Year-3	1	0	Fall
9/22/2020	FP	8A	9	2020	Year-3		1	Fall
9/22/2020	FP	9A	9	2020	Year-3		1	Fall
9/22/2020	FP	11A	9	2020	Year-3		1	Fall
9/22/2020	FP	13A	9	2020	Year-3		1	Fall
9/23/2020	FP	1M	9	2020	Year-3	1	0	Fall
9/23/2020	FP	2M	9	2020	Year-3		1	Fall
9/23/2020	FP	3M	9	2020	Year-3	1	0	Fall
9/23/2020	FP	4M	9	2020	Year-3	1	0	Fall
9/23/2020	FP	5M	9	2020	Year-3		1	Fall
9/23/2020	FP	6M	9	2020	Year-3		1	Fall
9/23/2020	FP	7M	9	2020	Year-3		1	Fall
9/23/2020	FP	10M	9	2020	Year-3	1	0	Fall
9/23/2020	FP	12M	9	2020	Year-3	1	0	Fall
9/23/2020	FP	14M	9	2020	Year-3	1	0	Fall
9/23/2020	FP	8A	9	2020	Year-3		1	Fall
9/23/2020	FP	9A	9	2020	Year-3		1	Fall
9/23/2020	FP	11A	9	2020	Year-3		1	Fall
9/23/2020	FP	13A	9	2020	Year-3		1	Fall
9/24/2020	FP	1M	9	2020	Year-3	1	0	Fall
9/24/2020	FP	2M	9	2020	Year-3		1	Fall
9/24/2020	FP	3M	9	2020	Year-3	1	0	Fall
9/24/2020	FP	4M	9	2020	Year-3	1	0	Fall
9/24/2020	FP	5M	9	2020	Year-3		1	Fall
9/24/2020	FP	6M	9	2020	Year-3		1	Fall
9/24/2020	FP	7M	9	2020	Year-3		1	Fall
9/24/2020	FP	10M	9	2020	Year-3	1	0	Fall
9/24/2020	FP	12M	9	2020	Year-3	1	0	Fall
9/24/2020	FP	14M	9	2020	Year-3	1	0	Fall
9/24/2020	FP	8A	9	2020	Year-3		1	Fall
9/24/2020	FP	9A	9	2020	Year-3		1	Fall
9/24/2020	FP	11A	9	2020	Year-3		1	Fall

Date	Species	Site	Month	Year	Survey Year	Presence	Data Gap	Season
9/24/2020	FP	13A	9	2020	Year-3		1	Fall
9/25/2020	FP	1M	9	2020	Year-3	1	0	Fall
9/25/2020	FP	2M	9	2020	Year-3		1	Fall
9/25/2020	FP	3M	9	2020	Year-3	1	0	Fall
9/25/2020	FP	4M	9	2020	Year-3	1	0	Fall
9/25/2020	FP	5M	9	2020	Year-3		1	Fall
9/25/2020	FP	6M	9	2020	Year-3		1	Fall
9/25/2020	FP	7M	9	2020	Year-3		1	Fall
9/25/2020	FP	10M	9	2020	Year-3	1	0	Fall
9/25/2020	FP	12M	9	2020	Year-3	1	0	Fall
9/25/2020	FP	14M	9	2020	Year-3	1	0	Fall
9/25/2020	FP	8A	9	2020	Year-3		1	Fall
9/25/2020	FP	9A	9	2020	Year-3		1	Fall
9/25/2020	FP	11A	9	2020	Year-3		1	Fall
9/25/2020	FP	13A	9	2020	Year-3		1	Fall
9/26/2020	FP	1M	9	2020	Year-3	1	0	Fall
9/26/2020	FP	2M	9	2020	Year-3		1	Fall
9/26/2020	FP	3M	9	2020	Year-3	1	0	Fall
9/26/2020	FP	4M	9	2020	Year-3	1	0	Fall
9/26/2020	FP	5M	9	2020	Year-3		1	Fall
9/26/2020	FP	6M	9	2020	Year-3		1	Fall
9/26/2020	FP	7M	9	2020	Year-3		1	Fall
9/26/2020	FP	10M	9	2020	Year-3	1	0	Fall
9/26/2020	FP	12M	9	2020	Year-3	1	0	Fall
9/26/2020	FP	14M	9	2020	Year-3	1	0	Fall
9/26/2020	FP	8A	9	2020	Year-3		1	Fall
9/26/2020	FP	9A	9	2020	Year-3		1	Fall
9/26/2020	FP	11A	9	2020	Year-3		1	Fall
9/26/2020	FP	13A	9	2020	Year-3		1	Fall
9/27/2020	FP	1M	9	2020	Year-3	1	0	Fall
9/27/2020	FP	2M	9	2020	Year-3		1	Fall
9/27/2020	FP	3M	9	2020	Year-3	1	0	Fall
9/27/2020	FP	4M	9	2020	Year-3	1	0	Fall
9/27/2020	FP	5M	9	2020	Year-3		1	Fall
9/27/2020	FP	6M	9	2020	Year-3		1	Fall
9/27/2020	FP	7M	9	2020	Year-3		1	Fall
9/27/2020	FP	10M	9	2020	Year-3	1	0	Fall
9/27/2020	FP	12M	9	2020	Year-3	1	0	Fall
9/27/2020	FP	14M	9	2020	Year-3	1	0	Fall
9/27/2020	FP	8A	9	2020	Year-3		1	Fall
9/27/2020	FP	9A	9	2020	Year-3		1	Fall
9/27/2020	FP	11A	9	2020	Year-3		1	Fall
9/27/2020	FP	13A	9	2020	Year-3		1	Fall
9/28/2020	FP	1M	9	2020	Year-3	1	0	Fall

Date	Species	Site	Month	Year	Survey Year	Presence	Data Gap	Season
9/28/2020	FP	2M	9	2020	Year-3		1	Fall
9/28/2020	FP	3M	9	2020	Year-3	1	0	Fall
9/28/2020	FP	4M	9	2020	Year-3	1	0	Fall
9/28/2020	FP	5M	9	2020	Year-3		1	Fall
9/28/2020	FP	6M	9	2020	Year-3		1	Fall
9/28/2020	FP	7M	9	2020	Year-3		1	Fall
9/28/2020	FP	10M	9	2020	Year-3	1	0	Fall
9/28/2020	FP	12M	9	2020	Year-3	1	0	Fall
9/28/2020	FP	14M	9	2020	Year-3	1	0	Fall
9/28/2020	FP	8A	9	2020	Year-3		1	Fall
9/28/2020	FP	9A	9	2020	Year-3		1	Fall
9/28/2020	FP	11A	9	2020	Year-3		1	Fall
9/28/2020	FP	13A	9	2020	Year-3		1	Fall
9/29/2020	FP	1M	9	2020	Year-3	1	0	Fall
9/29/2020	FP	2M	9	2020	Year-3		1	Fall
9/29/2020	FP	3M	9	2020	Year-3	1	0	Fall
9/29/2020	FP	4M	9	2020	Year-3	1	0	Fall
9/29/2020	FP	5M	9	2020	Year-3		1	Fall
9/29/2020	FP	6M	9	2020	Year-3		1	Fall
9/29/2020	FP	7M	9	2020	Year-3		1	Fall
9/29/2020	FP	10M	9	2020	Year-3	1	0	Fall
9/29/2020	FP	12M	9	2020	Year-3	1	0	Fall
9/29/2020	FP	14M	9	2020	Year-3	1	0	Fall
9/29/2020	FP	8A	9	2020	Year-3		1	Fall
9/29/2020	FP	9A	9	2020	Year-3		1	Fall
9/29/2020	FP	11A	9	2020	Year-3		1	Fall
9/29/2020	FP	13A	9	2020	Year-3		1	Fall
9/30/2020	FP	1M	9	2020	Year-3	1	0	Fall
9/30/2020	FP	2M	9	2020	Year-3		1	Fall
9/30/2020	FP	3M	9	2020	Year-3	1	0	Fall
9/30/2020	FP	4M	9	2020	Year-3	1	0	Fall
9/30/2020	FP	5M	9	2020	Year-3		1	Fall
9/30/2020	FP	6M	9	2020	Year-3		1	Fall
9/30/2020	FP	7M	9	2020	Year-3		1	Fall
9/30/2020	FP	10M	9	2020	Year-3	1	0	Fall
9/30/2020	FP	12M	9	2020	Year-3	1	0	Fall
9/30/2020	FP	14M	9	2020	Year-3	1	0	Fall
9/30/2020	FP	8A	9	2020	Year-3		1	Fall
9/30/2020	FP	9A	9	2020	Year-3		1	Fall
9/30/2020	FP	11A	9	2020	Year-3		1	Fall
9/30/2020	FP	13A	9	2020	Year-3		1	Fall
10/1/2020	FP	1M	10	2020	Year-3	1	0	Fall
10/1/2020	FP	2M	10	2020	Year-3		1	Fall
10/1/2020	FP	3M	10	2020	Year-3	1	0	Fall

Date	Species	Site	Month	Year	Survey Year	Presence	Data Gap	Season
10/1/2020	FP	4M	10	2020	Year-3	1	0	Fall
10/1/2020	FP	5M	10	2020	Year-3		1	Fall
10/1/2020	FP	6M	10	2020	Year-3		1	Fall
10/1/2020	FP	7M	10	2020	Year-3		1	Fall
10/1/2020	FP	10M	10	2020	Year-3	1	0	Fall
10/1/2020	FP	12M	10	2020	Year-3	1	0	Fall
10/1/2020	FP	14M	10	2020	Year-3	1	0	Fall
10/1/2020	FP	8A	10	2020	Year-3		1	Fall
10/1/2020	FP	9A	10	2020	Year-3		1	Fall
10/1/2020	FP	11A	10	2020	Year-3		1	Fall
10/1/2020	FP	13A	10	2020	Year-3		1	Fall
10/2/2020	FP	1M	10	2020	Year-3	1	0	Fall
10/2/2020	FP	2M	10	2020	Year-3		1	Fall
10/2/2020	FP	3M	10	2020	Year-3	1	0	Fall
10/2/2020	FP	4M	10	2020	Year-3	1	0	Fall
10/2/2020	FP	5M	10	2020	Year-3		1	Fall
10/2/2020	FP	6M	10	2020	Year-3		1	Fall
10/2/2020	FP	7M	10	2020	Year-3		1	Fall
10/2/2020	FP	10M	10	2020	Year-3	1	0	Fall
10/2/2020	FP	12M	10	2020	Year-3	1	0	Fall
10/2/2020	FP	14M	10	2020	Year-3	1	0	Fall
10/2/2020	FP	8A	10	2020	Year-3		1	Fall
10/2/2020	FP	9A	10	2020	Year-3		1	Fall
10/2/2020	FP	11A	10	2020	Year-3		1	Fall
10/2/2020	FP	13A	10	2020	Year-3		1	Fall
10/3/2020	FP	1M	10	2020	Year-3	1	0	Fall
10/3/2020	FP	2M	10	2020	Year-3		1	Fall
10/3/2020	FP	3M	10	2020	Year-3	1	0	Fall
10/3/2020	FP	4M	10	2020	Year-3	1	0	Fall
10/3/2020	FP	5M	10	2020	Year-3		1	Fall
10/3/2020	FP	6M	10	2020	Year-3		1	Fall
10/3/2020	FP	7M	10	2020	Year-3		1	Fall
10/3/2020	FP	10M	10	2020	Year-3	1	0	Fall
10/3/2020	FP	12M	10	2020	Year-3	1	0	Fall
10/3/2020	FP	14M	10	2020	Year-3	1	0	Fall
10/3/2020	FP	8A	10	2020	Year-3		1	Fall
10/3/2020	FP	9A	10	2020	Year-3		1	Fall
10/3/2020	FP	11A	10	2020	Year-3		1	Fall
10/3/2020	FP	13A	10	2020	Year-3		1	Fall
10/4/2020	FP	1M	10	2020	Year-3	1	0	Fall
10/4/2020	FP	2M	10	2020	Year-3		1	Fall
10/4/2020	FP	3M	10	2020	Year-3	1	0	Fall
10/4/2020	FP	4M	10	2020	Year-3	1	0	Fall
10/4/2020	FP	5M	10	2020	Year-3		1	Fall

Date	Species	Site	Month	Year	Survey Year	Presence	Data Gap	Season
10/4/2020	FP	6M	10	2020	Year-3		1	Fall
10/4/2020	FP	7M	10	2020	Year-3		1	Fall
10/4/2020	FP	10M	10	2020	Year-3	1	0	Fall
10/4/2020	FP	12M	10	2020	Year-3	1	0	Fall
10/4/2020	FP	14M	10	2020	Year-3	1	0	Fall
10/4/2020	FP	8A	10	2020	Year-3		1	Fall
10/4/2020	FP	9A	10	2020	Year-3		1	Fall
10/4/2020	FP	11A	10	2020	Year-3		1	Fall
10/4/2020	FP	13A	10	2020	Year-3		1	Fall
10/5/2020	FP	1M	10	2020	Year-3	1	0	Fall
10/5/2020	FP	2M	10	2020	Year-3		1	Fall
10/5/2020	FP	3M	10	2020	Year-3	1	0	Fall
10/5/2020	FP	4M	10	2020	Year-3	1	0	Fall
10/5/2020	FP	5M	10	2020	Year-3		1	Fall
10/5/2020	FP	6M	10	2020	Year-3		1	Fall
10/5/2020	FP	7M	10	2020	Year-3		1	Fall
10/5/2020	FP	10M	10	2020	Year-3	1	0	Fall
10/5/2020	FP	12M	10	2020	Year-3	1	0	Fall
10/5/2020	FP	14M	10	2020	Year-3	1	0	Fall
10/5/2020	FP	8A	10	2020	Year-3		1	Fall
10/5/2020	FP	9A	10	2020	Year-3		1	Fall
10/5/2020	FP	11A	10	2020	Year-3		1	Fall
10/5/2020	FP	13A	10	2020	Year-3		1	Fall
10/6/2020	FP	1M	10	2020	Year-3	1	0	Fall
10/6/2020	FP	2M	10	2020	Year-3		1	Fall
10/6/2020	FP	3M	10	2020	Year-3	1	0	Fall
10/6/2020	FP	4M	10	2020	Year-3		1	Fall
10/6/2020	FP	5M	10	2020	Year-3		1	Fall
10/6/2020	FP	6M	10	2020	Year-3		1	Fall
10/6/2020	FP	7M	10	2020	Year-3		1	Fall
10/6/2020	FP	10M	10	2020	Year-3	1	0	Fall
10/6/2020	FP	12M	10	2020	Year-3	1	0	Fall
10/6/2020	FP	14M	10	2020	Year-3	1	0	Fall
10/6/2020	FP	8A	10	2020	Year-3		1	Fall
10/6/2020	FP	9A	10	2020	Year-3		1	Fall
10/6/2020	FP	11A	10	2020	Year-3		1	Fall
10/6/2020	FP	13A	10	2020	Year-3		1	Fall
10/7/2020	FP	1M	10	2020	Year-3	1	0	Fall
10/7/2020	FP	2M	10	2020	Year-3		1	Fall
10/7/2020	FP	3M	10	2020	Year-3	1	0	Fall
10/7/2020	FP	4M	10	2020	Year-3		1	Fall
10/7/2020	FP	5M	10	2020	Year-3		1	Fall
10/7/2020	FP	6M	10	2020	Year-3		1	Fall
10/7/2020	FP	7M	10	2020	Year-3		1	Fall

Date	Species	Site	Month	Year	Survey Year	Presence	Data Gap	Season
10/7/2020	FP	10M	10	2020	Year-3	1	0	Fall
10/7/2020	FP	12M	10	2020	Year-3	1	0	Fall
10/7/2020	FP	14M	10	2020	Year-3	1	0	Fall
10/7/2020	FP	8A	10	2020	Year-3		1	Fall
10/7/2020	FP	9A	10	2020	Year-3		1	Fall
10/7/2020	FP	11A	10	2020	Year-3		1	Fall
10/7/2020	FP	13A	10	2020	Year-3		1	Fall
10/8/2020	FP	1M	10	2020	Year-3	1	0	Fall
10/8/2020	FP	2M	10	2020	Year-3		1	Fall
10/8/2020	FP	3M	10	2020	Year-3	1	0	Fall
10/8/2020	FP	4M	10	2020	Year-3		1	Fall
10/8/2020	FP	5M	10	2020	Year-3		1	Fall
10/8/2020	FP	6M	10	2020	Year-3		1	Fall
10/8/2020	FP	7M	10	2020	Year-3		1	Fall
10/8/2020	FP	10M	10	2020	Year-3	1	0	Fall
10/8/2020	FP	12M	10	2020	Year-3	1	0	Fall
10/8/2020	FP	14M	10	2020	Year-3	1	0	Fall
10/8/2020	FP	8A	10	2020	Year-3		1	Fall
10/8/2020	FP	9A	10	2020	Year-3		1	Fall
10/8/2020	FP	11A	10	2020	Year-3		1	Fall
10/8/2020	FP	13A	10	2020	Year-3		1	Fall
10/9/2020	FP	1M	10	2020	Year-3	1	0	Fall
10/9/2020	FP	2M	10	2020	Year-3		1	Fall
10/9/2020	FP	3M	10	2020	Year-3	1	0	Fall
10/9/2020	FP	4M	10	2020	Year-3		1	Fall
10/9/2020	FP	5M	10	2020	Year-3		1	Fall
10/9/2020	FP	6M	10	2020	Year-3		1	Fall
10/9/2020	FP	7M	10	2020	Year-3		1	Fall
10/9/2020	FP	10M	10	2020	Year-3	1	0	Fall
10/9/2020	FP	12M	10	2020	Year-3	1	0	Fall
10/9/2020	FP	14M	10	2020	Year-3	1	0	Fall
10/9/2020	FP	8A	10	2020	Year-3		1	Fall
10/9/2020	FP	9A	10	2020	Year-3		1	Fall
10/9/2020	FP	11A	10	2020	Year-3		1	Fall
10/9/2020	FP	13A	10	2020	Year-3		1	Fall
10/10/2020	FP	1M	10	2020	Year-3	1	0	Fall
10/10/2020	FP	2M	10	2020	Year-3		1	Fall
10/10/2020	FP	3M	10	2020	Year-3	1	0	Fall
10/10/2020	FP	4M	10	2020	Year-3		1	Fall
10/10/2020	FP	5M	10	2020	Year-3		1	Fall
10/10/2020	FP	6M	10	2020	Year-3		1	Fall
10/10/2020	FP	7M	10	2020	Year-3		1	Fall
10/10/2020	FP	10M	10	2020	Year-3	1	0	Fall
10/10/2020	FP	12M	10	2020	Year-3	1	0	Fall

Date	Species	Site	Month	Year	Survey Year	Presence	Data Gap	Season
10/10/2020	FP	14M	10	2020	Year-3	1	0	Fall
10/10/2020	FP	8A	10	2020	Year-3		1	Fall
10/10/2020	FP	9A	10	2020	Year-3		1	Fall
10/10/2020	FP	11A	10	2020	Year-3		1	Fall
10/10/2020	FP	13A	10	2020	Year-3		1	Fall
10/11/2020	FP	1M	10	2020	Year-3	1	0	Fall
10/11/2020	FP	2M	10	2020	Year-3		1	Fall
10/11/2020	FP	3M	10	2020	Year-3	1	0	Fall
10/11/2020	FP	4M	10	2020	Year-3		1	Fall
10/11/2020	FP	5M	10	2020	Year-3		1	Fall
10/11/2020	FP	6M	10	2020	Year-3		1	Fall
10/11/2020	FP	7M	10	2020	Year-3		1	Fall
10/11/2020	FP	10M	10	2020	Year-3	1	0	Fall
10/11/2020	FP	12M	10	2020	Year-3	1	0	Fall
10/11/2020	FP	14M	10	2020	Year-3	1	0	Fall
10/11/2020	FP	8A	10	2020	Year-3		1	Fall
10/11/2020	FP	9A	10	2020	Year-3		1	Fall
10/11/2020	FP	11A	10	2020	Year-3		1	Fall
10/11/2020	FP	13A	10	2020	Year-3		1	Fall
10/12/2020	FP	1M	10	2020	Year-3	1	0	Fall
10/12/2020	FP	2M	10	2020	Year-3		1	Fall
10/12/2020	FP	3M	10	2020	Year-3	1	0	Fall
10/12/2020	FP	4M	10	2020	Year-3		1	Fall
10/12/2020	FP	5M	10	2020	Year-3		1	Fall
10/12/2020	FP	6M	10	2020	Year-3		1	Fall
10/12/2020	FP	7M	10	2020	Year-3		1	Fall
10/12/2020	FP	10M	10	2020	Year-3	1	0	Fall
10/12/2020	FP	12M	10	2020	Year-3	1	0	Fall
10/12/2020	FP	14M	10	2020	Year-3	1	0	Fall
10/12/2020	FP	8A	10	2020	Year-3		1	Fall
10/12/2020	FP	9A	10	2020	Year-3		1	Fall
10/12/2020	FP	11A	10	2020	Year-3		1	Fall
10/12/2020	FP	13A	10	2020	Year-3		1	Fall
10/13/2020	FP	1M	10	2020	Year-3	1	0	Fall
10/13/2020	FP	2M	10	2020	Year-3		1	Fall
10/13/2020	FP	3M	10	2020	Year-3	1	0	Fall
10/13/2020	FP	4M	10	2020	Year-3		1	Fall
10/13/2020	FP	5M	10	2020	Year-3		1	Fall
10/13/2020	FP	6M	10	2020	Year-3		1	Fall
10/13/2020	FP	7M	10	2020	Year-3		1	Fall
10/13/2020	FP	10M	10	2020	Year-3	0	0	Fall
10/13/2020	FP	12M	10	2020	Year-3	1	0	Fall
10/13/2020	FP	14M	10	2020	Year-3	1	0	Fall
10/13/2020	FP	8A	10	2020	Year-3		1	Fall

Date	Species	Site	Month	Year	Survey Year	Presence	Data Gap	Season
10/13/2020	FP	9A	10	2020	Year-3		1	Fall
10/13/2020	FP	11A	10	2020	Year-3		1	Fall
10/13/2020	FP	13A	10	2020	Year-3		1	Fall
10/14/2020	FP	1M	10	2020	Year-3	1	0	Fall
10/14/2020	FP	2M	10	2020	Year-3		1	Fall
10/14/2020	FP	3M	10	2020	Year-3	1	0	Fall
10/14/2020	FP	4M	10	2020	Year-3		1	Fall
10/14/2020	FP	5M	10	2020	Year-3		1	Fall
10/14/2020	FP	6M	10	2020	Year-3		1	Fall
10/14/2020	FP	7M	10	2020	Year-3		1	Fall
10/14/2020	FP	10M	10	2020	Year-3	1	0	Fall
10/14/2020	FP	12M	10	2020	Year-3	1	0	Fall
10/14/2020	FP	14M	10	2020	Year-3	1	0	Fall
10/14/2020	FP	8A	10	2020	Year-3		1	Fall
10/14/2020	FP	9A	10	2020	Year-3		1	Fall
10/14/2020	FP	11A	10	2020	Year-3		1	Fall
10/14/2020	FP	13A	10	2020	Year-3		1	Fall
10/15/2020	FP	1M	10	2020	Year-3	1	0	Fall
10/15/2020	FP	2M	10	2020	Year-3		1	Fall
10/15/2020	FP	3M	10	2020	Year-3	1	0	Fall
10/15/2020	FP	4M	10	2020	Year-3		1	Fall
10/15/2020	FP	5M	10	2020	Year-3		1	Fall
10/15/2020	FP	6M	10	2020	Year-3		1	Fall
10/15/2020	FP	7M	10	2020	Year-3		1	Fall
10/15/2020	FP	10M	10	2020	Year-3	1	0	Fall
10/15/2020	FP	12M	10	2020	Year-3	1	0	Fall
10/15/2020	FP	14M	10	2020	Year-3	1	0	Fall
10/15/2020	FP	8A	10	2020	Year-3		1	Fall
10/15/2020	FP	9A	10	2020	Year-3		1	Fall
10/15/2020	FP	11A	10	2020	Year-3		1	Fall
10/15/2020	FP	13A	10	2020	Year-3		1	Fall
10/16/2020	FP	1M	10	2020	Year-3	1	0	Fall
10/16/2020	FP	2M	10	2020	Year-3		1	Fall
10/16/2020	FP	3M	10	2020	Year-3	1	0	Fall
10/16/2020	FP	4M	10	2020	Year-3		1	Fall
10/16/2020	FP	5M	10	2020	Year-3		1	Fall
10/16/2020	FP	6M	10	2020	Year-3		1	Fall
10/16/2020	FP	7M	10	2020	Year-3		1	Fall
10/16/2020	FP	10M	10	2020	Year-3	1	0	Fall
10/16/2020	FP	12M	10	2020	Year-3	1	0	Fall
10/16/2020	FP	14M	10	2020	Year-3	1	0	Fall
10/16/2020	FP	8A	10	2020	Year-3		1	Fall
10/16/2020	FP	9A	10	2020	Year-3		1	Fall
10/16/2020	FP	11A	10	2020	Year-3		1	Fall

Date	Species	Site	Month	Year	Survey Year	Presence	Data Gap	Season
10/16/2020	FP	13A	10	2020	Year-3		1	Fall
10/17/2020	FP	1M	10	2020	Year-3	1	0	Fall
10/17/2020	FP	2M	10	2020	Year-3		1	Fall
10/17/2020	FP	3M	10	2020	Year-3	1	0	Fall
10/17/2020	FP	4M	10	2020	Year-3		1	Fall
10/17/2020	FP	5M	10	2020	Year-3		1	Fall
10/17/2020	FP	6M	10	2020	Year-3		1	Fall
10/17/2020	FP	7M	10	2020	Year-3		1	Fall
10/17/2020	FP	10M	10	2020	Year-3	1	0	Fall
10/17/2020	FP	12M	10	2020	Year-3	1	0	Fall
10/17/2020	FP	14M	10	2020	Year-3	1	0	Fall
10/17/2020	FP	8A	10	2020	Year-3		1	Fall
10/17/2020	FP	9A	10	2020	Year-3		1	Fall
10/17/2020	FP	11A	10	2020	Year-3		1	Fall
10/17/2020	FP	13A	10	2020	Year-3		1	Fall
10/18/2020	FP	1M	10	2020	Year-3	1	0	Fall
10/18/2020	FP	2M	10	2020	Year-3		1	Fall
10/18/2020	FP	3M	10	2020	Year-3	1	0	Fall
10/18/2020	FP	4M	10	2020	Year-3		1	Fall
10/18/2020	FP	5M	10	2020	Year-3		1	Fall
10/18/2020	FP	6M	10	2020	Year-3		1	Fall
10/18/2020	FP	7M	10	2020	Year-3		1	Fall
10/18/2020	FP	10M	10	2020	Year-3	1	0	Fall
10/18/2020	FP	12M	10	2020	Year-3	1	0	Fall
10/18/2020	FP	14M	10	2020	Year-3	1	0	Fall
10/18/2020	FP	8A	10	2020	Year-3		1	Fall
10/18/2020	FP	9A	10	2020	Year-3		1	Fall
10/18/2020	FP	11A	10	2020	Year-3		1	Fall
10/18/2020	FP	13A	10	2020	Year-3		1	Fall
10/19/2020	FP	1M	10	2020	Year-3	1	0	Fall
10/19/2020	FP	2M	10	2020	Year-3		1	Fall
10/19/2020	FP	3M	10	2020	Year-3	1	0	Fall
10/19/2020	FP	4M	10	2020	Year-3		1	Fall
10/19/2020	FP	5M	10	2020	Year-3		1	Fall
10/19/2020	FP	6M	10	2020	Year-3		1	Fall
10/19/2020	FP	7M	10	2020	Year-3		1	Fall
10/19/2020	FP	10M	10	2020	Year-3	1	0	Fall
10/19/2020	FP	12M	10	2020	Year-3	1	0	Fall
10/19/2020	FP	14M	10	2020	Year-3	1	0	Fall
10/19/2020	FP	8A	10	2020	Year-3		1	Fall
10/19/2020	FP	9A	10	2020	Year-3		1	Fall
10/19/2020	FP	11A	10	2020	Year-3		1	Fall
10/19/2020	FP	13A	10	2020	Year-3		1	Fall
10/20/2020	FP	1M	10	2020	Year-3	1	0	Fall

Date	Species	Site	Month	Year	Survey Year	Presence	Data Gap	Season
10/20/2020	FP	2M	10	2020	Year-3		1	Fall
10/20/2020	FP	3M	10	2020	Year-3	1	0	Fall
10/20/2020	FP	4M	10	2020	Year-3		1	Fall
10/20/2020	FP	5M	10	2020	Year-3		1	Fall
10/20/2020	FP	6M	10	2020	Year-3		1	Fall
10/20/2020	FP	7M	10	2020	Year-3		1	Fall
10/20/2020	FP	10M	10	2020	Year-3	1	0	Fall
10/20/2020	FP	12M	10	2020	Year-3	1	0	Fall
10/20/2020	FP	14M	10	2020	Year-3	1	0	Fall
10/20/2020	FP	8A	10	2020	Year-3		1	Fall
10/20/2020	FP	9A	10	2020	Year-3		1	Fall
10/20/2020	FP	11A	10	2020	Year-3		1	Fall
10/20/2020	FP	13A	10	2020	Year-3		1	Fall
10/21/2020	FP	1M	10	2020	Year-3	1	0	Fall
10/21/2020	FP	2M	10	2020	Year-3		1	Fall
10/21/2020	FP	3M	10	2020	Year-3	1	0	Fall
10/21/2020	FP	4M	10	2020	Year-3		1	Fall
10/21/2020	FP	5M	10	2020	Year-3		1	Fall
10/21/2020	FP	6M	10	2020	Year-3		1	Fall
10/21/2020	FP	7M	10	2020	Year-3		1	Fall
10/21/2020	FP	10M	10	2020	Year-3	1	0	Fall
10/21/2020	FP	12M	10	2020	Year-3	1	0	Fall
10/21/2020	FP	14M	10	2020	Year-3	1	0	Fall
10/21/2020	FP	8A	10	2020	Year-3		1	Fall
10/21/2020	FP	9A	10	2020	Year-3		1	Fall
10/21/2020	FP	11A	10	2020	Year-3		1	Fall
10/21/2020	FP	13A	10	2020	Year-3		1	Fall
10/22/2020	FP	1M	10	2020	Year-3	1	0	Fall
10/22/2020	FP	2M	10	2020	Year-3		1	Fall
10/22/2020	FP	3M	10	2020	Year-3	1	0	Fall
10/22/2020	FP	4M	10	2020	Year-3		1	Fall
10/22/2020	FP	5M	10	2020	Year-3		1	Fall
10/22/2020	FP	6M	10	2020	Year-3		1	Fall
10/22/2020	FP	7M	10	2020	Year-3		1	Fall
10/22/2020	FP	10M	10	2020	Year-3	1	0	Fall
10/22/2020	FP	12M	10	2020	Year-3	1	0	Fall
10/22/2020	FP	14M	10	2020	Year-3	1	0	Fall
10/22/2020	FP	8A	10	2020	Year-3		1	Fall
10/22/2020	FP	9A	10	2020	Year-3		1	Fall
10/22/2020	FP	11A	10	2020	Year-3		1	Fall
10/22/2020	FP	13A	10	2020	Year-3		1	Fall
10/23/2020	FP	1M	10	2020	Year-3	1	0	Fall
10/23/2020	FP	2M	10	2020	Year-3		1	Fall
10/23/2020	FP	3M	10	2020	Year-3	1	0	Fall

Date	Species	Site	Month	Year	Survey Year	Presence	Data Gap	Season
10/23/2020	FP	4M	10	2020	Year-3		1	Fall
10/23/2020	FP	5M	10	2020	Year-3		1	Fall
10/23/2020	FP	6M	10	2020	Year-3		1	Fall
10/23/2020	FP	7M	10	2020	Year-3		1	Fall
10/23/2020	FP	10M	10	2020	Year-3	1	0	Fall
10/23/2020	FP	12M	10	2020	Year-3	1	0	Fall
10/23/2020	FP	14M	10	2020	Year-3	1	0	Fall
10/23/2020	FP	8A	10	2020	Year-3		1	Fall
10/23/2020	FP	9A	10	2020	Year-3		1	Fall
10/23/2020	FP	11A	10	2020	Year-3		1	Fall
10/23/2020	FP	13A	10	2020	Year-3		1	Fall
10/24/2020	FP	1M	10	2020	Year-3	1	0	Fall
10/24/2020	FP	2M	10	2020	Year-3		1	Fall
10/24/2020	FP	3M	10	2020	Year-3	1	0	Fall
10/24/2020	FP	4M	10	2020	Year-3		1	Fall
10/24/2020	FP	5M	10	2020	Year-3		1	Fall
10/24/2020	FP	6M	10	2020	Year-3		1	Fall
10/24/2020	FP	7M	10	2020	Year-3		1	Fall
10/24/2020	FP	10M	10	2020	Year-3	1	0	Fall
10/24/2020	FP	12M	10	2020	Year-3	1	0	Fall
10/24/2020	FP	14M	10	2020	Year-3	1	0	Fall
10/24/2020	FP	8A	10	2020	Year-3		1	Fall
10/24/2020	FP	9A	10	2020	Year-3		1	Fall
10/24/2020	FP	11A	10	2020	Year-3		1	Fall
10/24/2020	FP	13A	10	2020	Year-3		1	Fall
10/25/2020	FP	1M	10	2020	Year-3	1	0	Fall
10/25/2020	FP	2M	10	2020	Year-3		1	Fall
10/25/2020	FP	3M	10	2020	Year-3	1	0	Fall
10/25/2020	FP	4M	10	2020	Year-3		1	Fall
10/25/2020	FP	5M	10	2020	Year-3		1	Fall
10/25/2020	FP	6M	10	2020	Year-3		1	Fall
10/25/2020	FP	7M	10	2020	Year-3		1	Fall
10/25/2020	FP	10M	10	2020	Year-3	1	0	Fall
10/25/2020	FP	12M	10	2020	Year-3	1	0	Fall
10/25/2020	FP	14M	10	2020	Year-3	1	0	Fall
10/25/2020	FP	8A	10	2020	Year-3		1	Fall
10/25/2020	FP	9A	10	2020	Year-3		1	Fall
10/25/2020	FP	11A	10	2020	Year-3		1	Fall
10/25/2020	FP	13A	10	2020	Year-3		1	Fall
10/26/2020	FP	1M	10	2020	Year-3	1	0	Fall
10/26/2020	FP	2M	10	2020	Year-3		1	Fall
10/26/2020	FP	3M	10	2020	Year-3	1	0	Fall
10/26/2020	FP	4M	10	2020	Year-3		1	Fall
10/26/2020	FP	5M	10	2020	Year-3		1	Fall

Date	Species	Site	Month	Year	Survey Year	Presence	Data Gap	Season
10/26/2020	FP	6M	10	2020	Year-3		1	Fall
10/26/2020	FP	7M	10	2020	Year-3		1	Fall
10/26/2020	FP	10M	10	2020	Year-3	1	0	Fall
10/26/2020	FP	12M	10	2020	Year-3	1	0	Fall
10/26/2020	FP	14M	10	2020	Year-3	1	0	Fall
10/26/2020	FP	8A	10	2020	Year-3		1	Fall
10/26/2020	FP	9A	10	2020	Year-3		1	Fall
10/26/2020	FP	11A	10	2020	Year-3		1	Fall
10/26/2020	FP	13A	10	2020	Year-3		1	Fall
10/27/2020	FP	1M	10	2020	Year-3	1	0	Fall
10/27/2020	FP	2M	10	2020	Year-3		1	Fall
10/27/2020	FP	3M	10	2020	Year-3	1	0	Fall
10/27/2020	FP	4M	10	2020	Year-3		1	Fall
10/27/2020	FP	5M	10	2020	Year-3		1	Fall
10/27/2020	FP	6M	10	2020	Year-3		1	Fall
10/27/2020	FP	7M	10	2020	Year-3		1	Fall
10/27/2020	FP	10M	10	2020	Year-3	1	0	Fall
10/27/2020	FP	12M	10	2020	Year-3	1	0	Fall
10/27/2020	FP	14M	10	2020	Year-3	1	0	Fall
10/27/2020	FP	8A	10	2020	Year-3		1	Fall
10/27/2020	FP	9A	10	2020	Year-3		1	Fall
10/27/2020	FP	11A	10	2020	Year-3		1	Fall
10/27/2020	FP	13A	10	2020	Year-3		1	Fall
10/28/2020	FP	1M	10	2020	Year-3	1	0	Fall
10/28/2020	FP	2M	10	2020	Year-3		1	Fall
10/28/2020	FP	3M	10	2020	Year-3	1	0	Fall
10/28/2020	FP	4M	10	2020	Year-3		1	Fall
10/28/2020	FP	5M	10	2020	Year-3		1	Fall
10/28/2020	FP	6M	10	2020	Year-3		1	Fall
10/28/2020	FP	7M	10	2020	Year-3		1	Fall
10/28/2020	FP	10M	10	2020	Year-3	1	0	Fall
10/28/2020	FP	12M	10	2020	Year-3	1	0	Fall
10/28/2020	FP	14M	10	2020	Year-3	1	0	Fall
10/28/2020	FP	8A	10	2020	Year-3		1	Fall
10/28/2020	FP	9A	10	2020	Year-3		1	Fall
10/28/2020	FP	11A	10	2020	Year-3		1	Fall
10/28/2020	FP	13A	10	2020	Year-3		1	Fall
10/29/2020	FP	1M	10	2020	Year-3	1	0	Fall
10/29/2020	FP	2M	10	2020	Year-3		1	Fall
10/29/2020	FP	3M	10	2020	Year-3	1	0	Fall
10/29/2020	FP	4M	10	2020	Year-3		1	Fall
10/29/2020	FP	5M	10	2020	Year-3		1	Fall
10/29/2020	FP	6M	10	2020	Year-3		1	Fall
10/29/2020	FP	7M	10	2020	Year-3		1	Fall

Date	Species	Site	Month	Year	Survey Year	Presence	Data Gap	Season
10/29/2020	FP	10M	10	2020	Year-3	1	0	Fall
10/29/2020	FP	12M	10	2020	Year-3	1	0	Fall
10/29/2020	FP	14M	10	2020	Year-3	1	0	Fall
10/29/2020	FP	8A	10	2020	Year-3		1	Fall
10/29/2020	FP	9A	10	2020	Year-3		1	Fall
10/29/2020	FP	11A	10	2020	Year-3		1	Fall
10/29/2020	FP	13A	10	2020	Year-3		1	Fall
10/30/2020	FP	1M	10	2020	Year-3	1	0	Fall
10/30/2020	FP	2M	10	2020	Year-3		1	Fall
10/30/2020	FP	3M	10	2020	Year-3	1	0	Fall
10/30/2020	FP	4M	10	2020	Year-3		1	Fall
10/30/2020	FP	5M	10	2020	Year-3		1	Fall
10/30/2020	FP	6M	10	2020	Year-3		1	Fall
10/30/2020	FP	7M	10	2020	Year-3		1	Fall
10/30/2020	FP	10M	10	2020	Year-3	1	0	Fall
10/30/2020	FP	12M	10	2020	Year-3	1	0	Fall
10/30/2020	FP	14M	10	2020	Year-3	1	0	Fall
10/30/2020	FP	8A	10	2020	Year-3		1	Fall
10/30/2020	FP	9A	10	2020	Year-3		1	Fall
10/30/2020	FP	11A	10	2020	Year-3		1	Fall
10/30/2020	FP	13A	10	2020	Year-3		1	Fall
10/31/2020	FP	1M	10	2020	Year-3	1	0	Fall
10/31/2020	FP	2M	10	2020	Year-3		1	Fall
10/31/2020	FP	3M	10	2020	Year-3	1	0	Fall
10/31/2020	FP	4M	10	2020	Year-3		1	Fall
10/31/2020	FP	5M	10	2020	Year-3		1	Fall
10/31/2020	FP	6M	10	2020	Year-3		1	Fall
10/31/2020	FP	7M	10	2020	Year-3		1	Fall
10/31/2020	FP	10M	10	2020	Year-3	1	0	Fall
10/31/2020	FP	12M	10	2020	Year-3	1	0	Fall
10/31/2020	FP	14M	10	2020	Year-3	1	0	Fall
10/31/2020	FP	8A	10	2020	Year-3		1	Fall
10/31/2020	FP	9A	10	2020	Year-3		1	Fall
10/31/2020	FP	11A	10	2020	Year-3		1	Fall
10/31/2020	FP	13A	10	2020	Year-3		1	Fall
10/16/2017	HB	1M	10	2017	Year-1	0	0	Fall
10/16/2017	HB	2M	10	2017	Year-1	0	0	Fall
10/16/2017	HB	3M	10	2017	Year-1	0	0	Fall
10/16/2017	HB	4M	10	2017	Year-1	0	0	Fall
10/16/2017	HB	5M	10	2017	Year-1	0	0	Fall
10/16/2017	HB	6M	10	2017	Year-1	0	0	Fall
10/16/2017	HB	7M	10	2017	Year-1	0	0	Fall
10/16/2017	HB	10M	10	2017	Year-1	0	0	Fall
10/16/2017	HB	12M	10	2017	Year-1	0	0	Fall

Date	Species	Site	Month	Year	Survey Year	Presence	Data Gap	Season
10/16/2017	HB	14M	10	2017	Year-1		1	Fall
10/16/2017	HB	8A	10	2017	Year-1	0	0	Fall
10/16/2017	HB	9A	10	2017	Year-1	0	0	Fall
10/16/2017	HB	11A	10	2017	Year-1	0	0	Fall
10/16/2017	HB	13A	10	2017	Year-1	0	0	Fall
10/17/2017	HB	1M	10	2017	Year-1	0	0	Fall
10/17/2017	HB	2M	10	2017	Year-1	0	0	Fall
10/17/2017	HB	3M	10	2017	Year-1	0	0	Fall
10/17/2017	HB	4M	10	2017	Year-1	0	0	Fall
10/17/2017	HB	5M	10	2017	Year-1	0	0	Fall
10/17/2017	HB	6M	10	2017	Year-1	0	0	Fall
10/17/2017	HB	7M	10	2017	Year-1	0	0	Fall
10/17/2017	HB	10M	10	2017	Year-1	0	0	Fall
10/17/2017	HB	12M	10	2017	Year-1	0	0	Fall
10/17/2017	HB	14M	10	2017	Year-1		1	Fall
10/17/2017	HB	8A	10	2017	Year-1	0	0	Fall
10/17/2017	HB	9A	10	2017	Year-1	0	0	Fall
10/17/2017	HB	11A	10	2017	Year-1	0	0	Fall
10/17/2017	HB	13A	10	2017	Year-1	0	0	Fall
10/18/2017	HB	1M	10	2017	Year-1	0	0	Fall
10/18/2017	HB	2M	10	2017	Year-1	0	0	Fall
10/18/2017	HB	3M	10	2017	Year-1	0	0	Fall
10/18/2017	HB	4M	10	2017	Year-1	0	0	Fall
10/18/2017	HB	5M	10	2017	Year-1	0	0	Fall
10/18/2017	HB	6M	10	2017	Year-1	0	0	Fall
10/18/2017	HB	7M	10	2017	Year-1	0	0	Fall
10/18/2017	HB	10M	10	2017	Year-1	0	0	Fall
10/18/2017	HB	12M	10	2017	Year-1	0	0	Fall
10/18/2017	HB	14M	10	2017	Year-1		1	Fall
10/18/2017	HB	8A	10	2017	Year-1	0	0	Fall
10/18/2017	HB	9A	10	2017	Year-1	0	0	Fall
10/18/2017	HB	11A	10	2017	Year-1	0	0	Fall
10/18/2017	HB	13A	10	2017	Year-1	0	0	Fall
10/19/2017	HB	1M	10	2017	Year-1	0	0	Fall
10/19/2017	HB	2M	10	2017	Year-1	0	0	Fall
10/19/2017	HB	3M	10	2017	Year-1	0	0	Fall
10/19/2017	HB	4M	10	2017	Year-1	0	0	Fall
10/19/2017	HB	5M	10	2017	Year-1	0	0	Fall
10/19/2017	HB	6M	10	2017	Year-1	0	0	Fall
10/19/2017	HB	7M	10	2017	Year-1	0	0	Fall
10/19/2017	HB	10M	10	2017	Year-1	0	0	Fall
10/19/2017	HB	12M	10	2017	Year-1	0	0	Fall
10/19/2017	HB	14M	10	2017	Year-1		1	Fall
10/19/2017	HB	8A	10	2017	Year-1	0	0	Fall

Date	Species	Site	Month	Year	Survey Year	Presence	Data Gap	Season
10/19/2017	HB	9A	10	2017	Year-1	0	0	Fall
10/19/2017	HB	11A	10	2017	Year-1	0	0	Fall
10/19/2017	HB	13A	10	2017	Year-1	0	0	Fall
10/20/2017	HB	1M	10	2017	Year-1	0	0	Fall
10/20/2017	HB	2M	10	2017	Year-1	0	0	Fall
10/20/2017	HB	3M	10	2017	Year-1	0	0	Fall
10/20/2017	HB	4M	10	2017	Year-1	0	0	Fall
10/20/2017	HB	5M	10	2017	Year-1	0	0	Fall
10/20/2017	HB	6M	10	2017	Year-1	0	0	Fall
10/20/2017	HB	7M	10	2017	Year-1	0	0	Fall
10/20/2017	HB	10M	10	2017	Year-1	0	0	Fall
10/20/2017	HB	12M	10	2017	Year-1	0	0	Fall
10/20/2017	HB	14M	10	2017	Year-1		1	Fall
10/20/2017	HB	8A	10	2017	Year-1	0	0	Fall
10/20/2017	HB	9A	10	2017	Year-1	0	0	Fall
10/20/2017	HB	11A	10	2017	Year-1	0	0	Fall
10/20/2017	HB	13A	10	2017	Year-1	0	0	Fall
10/21/2017	HB	1M	10	2017	Year-1	0	0	Fall
10/21/2017	HB	2M	10	2017	Year-1	0	0	Fall
10/21/2017	HB	3M	10	2017	Year-1	0	0	Fall
10/21/2017	HB	4M	10	2017	Year-1	0	0	Fall
10/21/2017	HB	5M	10	2017	Year-1	0	0	Fall
10/21/2017	HB	6M	10	2017	Year-1	0	0	Fall
10/21/2017	HB	7M	10	2017	Year-1	0	0	Fall
10/21/2017	HB	10M	10	2017	Year-1	0	0	Fall
10/21/2017	HB	12M	10	2017	Year-1	0	0	Fall
10/21/2017	HB	14M	10	2017	Year-1		1	Fall
10/21/2017	HB	8A	10	2017	Year-1	0	0	Fall
10/21/2017	HB	9A	10	2017	Year-1	0	0	Fall
10/21/2017	HB	11A	10	2017	Year-1	0	0	Fall
10/21/2017	HB	13A	10	2017	Year-1	0	0	Fall
10/22/2017	HB	1M	10	2017	Year-1	0	0	Fall
10/22/2017	HB	2M	10	2017	Year-1	0	0	Fall
10/22/2017	HB	3M	10	2017	Year-1	0	0	Fall
10/22/2017	HB	4M	10	2017	Year-1	0	0	Fall
10/22/2017	HB	5M	10	2017	Year-1	0	0	Fall
10/22/2017	HB	6M	10	2017	Year-1	0	0	Fall
10/22/2017	HB	7M	10	2017	Year-1	0	0	Fall
10/22/2017	HB	10M	10	2017	Year-1	0	0	Fall
10/22/2017	HB	12M	10	2017	Year-1	0	0	Fall
10/22/2017	HB	14M	10	2017	Year-1		1	Fall
10/22/2017	HB	8A	10	2017	Year-1	0	0	Fall
10/22/2017	HB	9A	10	2017	Year-1	0	0	Fall
10/22/2017	HB	11A	10	2017	Year-1	0	0	Fall

Date	Species	Site	Month	Year	Survey Year	Presence	Data Gap	Season
10/22/2017	HB	13A	10	2017	Year-1	0	0	Fall
10/23/2017	HB	1M	10	2017	Year-1	0	0	Fall
10/23/2017	HB	2M	10	2017	Year-1	0	0	Fall
10/23/2017	HB	3M	10	2017	Year-1	0	0	Fall
10/23/2017	HB	4M	10	2017	Year-1	0	0	Fall
10/23/2017	HB	5M	10	2017	Year-1	0	0	Fall
10/23/2017	HB	6M	10	2017	Year-1	0	0	Fall
10/23/2017	HB	7M	10	2017	Year-1	0	0	Fall
10/23/2017	HB	10M	10	2017	Year-1	0	0	Fall
10/23/2017	HB	12M	10	2017	Year-1	0	0	Fall
10/23/2017	HB	14M	10	2017	Year-1		1	Fall
10/23/2017	HB	8A	10	2017	Year-1	0	0	Fall
10/23/2017	HB	9A	10	2017	Year-1	0	0	Fall
10/23/2017	HB	11A	10	2017	Year-1	0	0	Fall
10/23/2017	HB	13A	10	2017	Year-1	0	0	Fall
10/24/2017	HB	1M	10	2017	Year-1	0	0	Fall
10/24/2017	HB	2M	10	2017	Year-1	0	0	Fall
10/24/2017	HB	3M	10	2017	Year-1	0	0	Fall
10/24/2017	HB	4M	10	2017	Year-1	0	0	Fall
10/24/2017	HB	5M	10	2017	Year-1	0	0	Fall
10/24/2017	HB	6M	10	2017	Year-1	0	0	Fall
10/24/2017	HB	7M	10	2017	Year-1	0	0	Fall
10/24/2017	HB	10M	10	2017	Year-1	0	0	Fall
10/24/2017	HB	12M	10	2017	Year-1	0	0	Fall
10/24/2017	HB	14M	10	2017	Year-1		1	Fall
10/24/2017	HB	8A	10	2017	Year-1	0	0	Fall
10/24/2017	HB	9A	10	2017	Year-1	0	0	Fall
10/24/2017	HB	11A	10	2017	Year-1	0	0	Fall
10/24/2017	HB	13A	10	2017	Year-1	0	0	Fall
10/25/2017	HB	1M	10	2017	Year-1	0	0	Fall
10/25/2017	HB	2M	10	2017	Year-1	0	0	Fall
10/25/2017	HB	3M	10	2017	Year-1	0	0	Fall
10/25/2017	HB	4M	10	2017	Year-1	0	0	Fall
10/25/2017	HB	5M	10	2017	Year-1	0	0	Fall
10/25/2017	HB	6M	10	2017	Year-1	0	0	Fall
10/25/2017	HB	7M	10	2017	Year-1	0	0	Fall
10/25/2017	HB	10M	10	2017	Year-1	0	0	Fall
10/25/2017	HB	12M	10	2017	Year-1	0	0	Fall
10/25/2017	HB	14M	10	2017	Year-1		1	Fall
10/25/2017	HB	8A	10	2017	Year-1	0	0	Fall
10/25/2017	HB	9A	10	2017	Year-1	0	0	Fall
10/25/2017	HB	11A	10	2017	Year-1	0	0	Fall
10/25/2017	HB	13A	10	2017	Year-1	0	0	Fall
10/26/2017	HB	1M	10	2017	Year-1	0	0	Fall

Date	Species	Site	Month	Year	Survey Year	Presence	Data Gap	Season
10/26/2017	HB	2M	10	2017	Year-1	0	0	Fall
10/26/2017	HB	3M	10	2017	Year-1	0	0	Fall
10/26/2017	HB	4M	10	2017	Year-1	0	0	Fall
10/26/2017	HB	5M	10	2017	Year-1	0	0	Fall
10/26/2017	HB	6M	10	2017	Year-1	0	0	Fall
10/26/2017	HB	7M	10	2017	Year-1	0	0	Fall
10/26/2017	HB	10M	10	2017	Year-1	0	0	Fall
10/26/2017	HB	12M	10	2017	Year-1	1	0	Fall
10/26/2017	HB	14M	10	2017	Year-1		1	Fall
10/26/2017	HB	8A	10	2017	Year-1	0	0	Fall
10/26/2017	HB	9A	10	2017	Year-1	0	0	Fall
10/26/2017	HB	11A	10	2017	Year-1	0	0	Fall
10/26/2017	HB	13A	10	2017	Year-1	0	0	Fall
10/27/2017	HB	1M	10	2017	Year-1	0	0	Fall
10/27/2017	HB	2M	10	2017	Year-1	0	0	Fall
10/27/2017	HB	3M	10	2017	Year-1	0	0	Fall
10/27/2017	HB	4M	10	2017	Year-1	0	0	Fall
10/27/2017	HB	5M	10	2017	Year-1	0	0	Fall
10/27/2017	HB	6M	10	2017	Year-1	0	0	Fall
10/27/2017	HB	7M	10	2017	Year-1	0	0	Fall
10/27/2017	HB	10M	10	2017	Year-1	0	0	Fall
10/27/2017	HB	12M	10	2017	Year-1	0	0	Fall
10/27/2017	HB	14M	10	2017	Year-1		1	Fall
10/27/2017	HB	8A	10	2017	Year-1	0	0	Fall
10/27/2017	HB	9A	10	2017	Year-1	0	0	Fall
10/27/2017	HB	11A	10	2017	Year-1	0	0	Fall
10/27/2017	HB	13A	10	2017	Year-1	0	0	Fall
10/28/2017	HB	1M	10	2017	Year-1	0	0	Fall
10/28/2017	HB	2M	10	2017	Year-1	0	0	Fall
10/28/2017	HB	3M	10	2017	Year-1	0	0	Fall
10/28/2017	HB	4M	10	2017	Year-1	0	0	Fall
10/28/2017	HB	5M	10	2017	Year-1	0	0	Fall
10/28/2017	HB	6M	10	2017	Year-1	0	0	Fall
10/28/2017	HB	7M	10	2017	Year-1	0	0	Fall
10/28/2017	HB	10M	10	2017	Year-1	0	0	Fall
10/28/2017	HB	12M	10	2017	Year-1	0	0	Fall
10/28/2017	HB	14M	10	2017	Year-1		1	Fall
10/28/2017	HB	8A	10	2017	Year-1	0	0	Fall
10/28/2017	HB	9A	10	2017	Year-1	0	0	Fall
10/28/2017	HB	11A	10	2017	Year-1	0	0	Fall
10/28/2017	HB	13A	10	2017	Year-1	0	0	Fall
10/29/2017	HB	1M	10	2017	Year-1	0	0	Fall
10/29/2017	HB	2M	10	2017	Year-1	1	0	Fall
10/29/2017	HB	3M	10	2017	Year-1	0	0	Fall

Date	Species	Site	Month	Year	Survey Year	Presence	Data Gap	Season
10/29/2017	HB	4M	10	2017	Year-1	0	0	Fall
10/29/2017	HB	5M	10	2017	Year-1	0	0	Fall
10/29/2017	HB	6M	10	2017	Year-1	0	0	Fall
10/29/2017	HB	7M	10	2017	Year-1	0	0	Fall
10/29/2017	HB	10M	10	2017	Year-1	0	0	Fall
10/29/2017	HB	12M	10	2017	Year-1	0	0	Fall
10/29/2017	HB	14M	10	2017	Year-1		1	Fall
10/29/2017	HB	8A	10	2017	Year-1	0	0	Fall
10/29/2017	HB	9A	10	2017	Year-1	0	0	Fall
10/29/2017	HB	11A	10	2017	Year-1	0	0	Fall
10/29/2017	HB	13A	10	2017	Year-1	0	0	Fall
10/30/2017	HB	1M	10	2017	Year-1	0	0	Fall
10/30/2017	HB	2M	10	2017	Year-1	0	0	Fall
10/30/2017	HB	3M	10	2017	Year-1	0	0	Fall
10/30/2017	HB	4M	10	2017	Year-1	0	0	Fall
10/30/2017	HB	5M	10	2017	Year-1	0	0	Fall
10/30/2017	HB	6M	10	2017	Year-1	0	0	Fall
10/30/2017	HB	7M	10	2017	Year-1	0	0	Fall
10/30/2017	HB	10M	10	2017	Year-1	0	0	Fall
10/30/2017	HB	12M	10	2017	Year-1	0	0	Fall
10/30/2017	HB	14M	10	2017	Year-1		1	Fall
10/30/2017	HB	8A	10	2017	Year-1	0	0	Fall
10/30/2017	HB	9A	10	2017	Year-1	0	0	Fall
10/30/2017	HB	11A	10	2017	Year-1	0	0	Fall
10/30/2017	HB	13A	10	2017	Year-1	1	0	Fall
10/31/2017	HB	1M	10	2017	Year-1	0	0	Fall
10/31/2017	HB	2M	10	2017	Year-1	0	0	Fall
10/31/2017	HB	3M	10	2017	Year-1	0	0	Fall
10/31/2017	HB	4M	10	2017	Year-1	0	0	Fall
10/31/2017	HB	5M	10	2017	Year-1	0	0	Fall
10/31/2017	HB	6M	10	2017	Year-1	0	0	Fall
10/31/2017	HB	7M	10	2017	Year-1	0	0	Fall
10/31/2017	HB	10M	10	2017	Year-1	0	0	Fall
10/31/2017	HB	12M	10	2017	Year-1	0	0	Fall
10/31/2017	HB	14M	10	2017	Year-1		1	Fall
10/31/2017	HB	8A	10	2017	Year-1	0	0	Fall
10/31/2017	HB	9A	10	2017	Year-1	0	0	Fall
10/31/2017	HB	11A	10	2017	Year-1	0	0	Fall
10/31/2017	HB	13A	10	2017	Year-1	0	0	Fall
11/1/2017	HB	1M	11	2017	Year-1	0	0	Fall
11/1/2017	HB	2M	11	2017	Year-1	0	0	Fall
11/1/2017	HB	3M	11	2017	Year-1	0	0	Fall
11/1/2017	HB	4M	11	2017	Year-1	0	0	Fall
11/1/2017	HB	5M	11	2017	Year-1	0	0	Fall

Date	Species	Site	Month	Year	Survey Year	Presence	Data Gap	Season
11/1/2017	HB	6M	11	2017	Year-1	0	0	Fall
11/1/2017	HB	7M	11	2017	Year-1	1	0	Fall
11/1/2017	HB	10M	11	2017	Year-1	0	0	Fall
11/1/2017	HB	12M	11	2017	Year-1	0	0	Fall
11/1/2017	HB	14M	11	2017	Year-1		1	Fall
11/1/2017	HB	8A	11	2017	Year-1	0	0	Fall
11/1/2017	HB	9A	11	2017	Year-1	0	0	Fall
11/1/2017	HB	11A	11	2017	Year-1	0	0	Fall
11/1/2017	HB	13A	11	2017	Year-1	0	0	Fall
11/2/2017	HB	1M	11	2017	Year-1	0	0	Fall
11/2/2017	HB	2M	11	2017	Year-1	0	0	Fall
11/2/2017	HB	3M	11	2017	Year-1	0	0	Fall
11/2/2017	HB	4M	11	2017	Year-1	0	0	Fall
11/2/2017	HB	5M	11	2017	Year-1	0	0	Fall
11/2/2017	HB	6M	11	2017	Year-1	0	0	Fall
11/2/2017	HB	7M	11	2017	Year-1	0	0	Fall
11/2/2017	HB	10M	11	2017	Year-1	0	0	Fall
11/2/2017	HB	12M	11	2017	Year-1	0	0	Fall
11/2/2017	HB	14M	11	2017	Year-1		1	Fall
11/2/2017	HB	8A	11	2017	Year-1	0	0	Fall
11/2/2017	HB	9A	11	2017	Year-1	0	0	Fall
11/2/2017	HB	11A	11	2017	Year-1	0	0	Fall
11/2/2017	HB	13A	11	2017	Year-1	0	0	Fall
11/3/2017	HB	1M	11	2017	Year-1	0	0	Fall
11/3/2017	HB	2M	11	2017	Year-1	0	0	Fall
11/3/2017	HB	3M	11	2017	Year-1	1	0	Fall
11/3/2017	HB	4M	11	2017	Year-1	0	0	Fall
11/3/2017	HB	5M	11	2017	Year-1	0	0	Fall
11/3/2017	HB	6M	11	2017	Year-1	0	0	Fall
11/3/2017	HB	7M	11	2017	Year-1	0	0	Fall
11/3/2017	HB	10M	11	2017	Year-1	0	0	Fall
11/3/2017	HB	12M	11	2017	Year-1	0	0	Fall
11/3/2017	HB	14M	11	2017	Year-1		1	Fall
11/3/2017	HB	8A	11	2017	Year-1	0	0	Fall
11/3/2017	HB	9A	11	2017	Year-1	0	0	Fall
11/3/2017	HB	11A	11	2017	Year-1	0	0	Fall
11/3/2017	HB	13A	11	2017	Year-1	0	0	Fall
11/4/2017	HB	1M	11	2017	Year-1	0	0	Fall
11/4/2017	HB	2M	11	2017	Year-1	0	0	Fall
11/4/2017	HB	3M	11	2017	Year-1	0	0	Fall
11/4/2017	HB	4M	11	2017	Year-1	0	0	Fall
11/4/2017	HB	5M	11	2017	Year-1	0	0	Fall
11/4/2017	HB	6M	11	2017	Year-1	0	0	Fall
11/4/2017	HB	7M	11	2017	Year-1	1	0	Fall

Date	Species	Site	Month	Year	Survey Year	Presence	Data Gap	Season
11/4/2017	HB	10M	11	2017	Year-1	0	0	Fall
11/4/2017	HB	12M	11	2017	Year-1	0	0	Fall
11/4/2017	HB	14M	11	2017	Year-1		1	Fall
11/4/2017	HB	8A	11	2017	Year-1	0	0	Fall
11/4/2017	HB	9A	11	2017	Year-1	0	0	Fall
11/4/2017	HB	11A	11	2017	Year-1	0	0	Fall
11/4/2017	HB	13A	11	2017	Year-1	0	0	Fall
11/5/2017	HB	1M	11	2017	Year-1	0	0	Fall
11/5/2017	HB	2M	11	2017	Year-1	0	0	Fall
11/5/2017	HB	3M	11	2017	Year-1	0	0	Fall
11/5/2017	HB	4M	11	2017	Year-1	0	0	Fall
11/5/2017	HB	5M	11	2017	Year-1	0	0	Fall
11/5/2017	HB	6M	11	2017	Year-1	0	0	Fall
11/5/2017	HB	7M	11	2017	Year-1	0	0	Fall
11/5/2017	HB	10M	11	2017	Year-1	1	0	Fall
11/5/2017	HB	12M	11	2017	Year-1	0	0	Fall
11/5/2017	HB	14M	11	2017	Year-1		1	Fall
11/5/2017	HB	8A	11	2017	Year-1	0	0	Fall
11/5/2017	HB	9A	11	2017	Year-1	0	0	Fall
11/5/2017	HB	11A	11	2017	Year-1	0	0	Fall
11/5/2017	HB	13A	11	2017	Year-1	0	0	Fall
11/6/2017	HB	1M	11	2017	Year-1	0	0	Fall
11/6/2017	HB	2M	11	2017	Year-1	0	0	Fall
11/6/2017	HB	3M	11	2017	Year-1	0	0	Fall
11/6/2017	HB	4M	11	2017	Year-1	0	0	Fall
11/6/2017	HB	5M	11	2017	Year-1	0	0	Fall
11/6/2017	HB	6M	11	2017	Year-1	0	0	Fall
11/6/2017	HB	7M	11	2017	Year-1	0	0	Fall
11/6/2017	HB	10M	11	2017	Year-1	0	0	Fall
11/6/2017	HB	12M	11	2017	Year-1	0	0	Fall
11/6/2017	HB	14M	11	2017	Year-1		1	Fall
11/6/2017	HB	8A	11	2017	Year-1	0	0	Fall
11/6/2017	HB	9A	11	2017	Year-1	0	0	Fall
11/6/2017	HB	11A	11	2017	Year-1	0	0	Fall
11/6/2017	HB	13A	11	2017	Year-1	0	0	Fall
11/7/2017	HB	1M	11	2017	Year-1	0	0	Fall
11/7/2017	HB	2M	11	2017	Year-1	0	0	Fall
11/7/2017	HB	3M	11	2017	Year-1	0	0	Fall
11/7/2017	HB	4M	11	2017	Year-1	0	0	Fall
11/7/2017	HB	5M	11	2017	Year-1	0	0	Fall
11/7/2017	HB	6M	11	2017	Year-1	0	0	Fall
11/7/2017	HB	7M	11	2017	Year-1	0	0	Fall
11/7/2017	HB	10M	11	2017	Year-1	0	0	Fall
11/7/2017	HB	12M	11	2017	Year-1	0	0	Fall

Date	Species	Site	Month	Year	Survey Year	Presence	Data Gap	Season
11/7/2017	HB	14M	11	2017	Year-1		1	Fall
11/7/2017	HB	8A	11	2017	Year-1	0	0	Fall
11/7/2017	HB	9A	11	2017	Year-1	0	0	Fall
11/7/2017	HB	11A	11	2017	Year-1	0	0	Fall
11/7/2017	HB	13A	11	2017	Year-1	0	0	Fall
11/8/2017	HB	1M	11	2017	Year-1	0	0	Fall
11/8/2017	HB	2M	11	2017	Year-1	0	0	Fall
11/8/2017	HB	3M	11	2017	Year-1	0	0	Fall
11/8/2017	HB	4M	11	2017	Year-1	0	0	Fall
11/8/2017	HB	5M	11	2017	Year-1	0	0	Fall
11/8/2017	HB	6M	11	2017	Year-1	0	0	Fall
11/8/2017	HB	7M	11	2017	Year-1	0	0	Fall
11/8/2017	HB	10M	11	2017	Year-1	0	0	Fall
11/8/2017	HB	12M	11	2017	Year-1	0	0	Fall
11/8/2017	HB	14M	11	2017	Year-1		1	Fall
11/8/2017	HB	8A	11	2017	Year-1	0	0	Fall
11/8/2017	HB	9A	11	2017	Year-1	0	0	Fall
11/8/2017	HB	11A	11	2017	Year-1	0	0	Fall
11/8/2017	HB	13A	11	2017	Year-1	0	0	Fall
11/9/2017	HB	1M	11	2017	Year-1	0	0	Fall
11/9/2017	HB	2M	11	2017	Year-1	0	0	Fall
11/9/2017	HB	3M	11	2017	Year-1	0	0	Fall
11/9/2017	HB	4M	11	2017	Year-1	0	0	Fall
11/9/2017	HB	5M	11	2017	Year-1	0	0	Fall
11/9/2017	HB	6M	11	2017	Year-1	0	0	Fall
11/9/2017	HB	7M	11	2017	Year-1	0	0	Fall
11/9/2017	HB	10M	11	2017	Year-1	0	0	Fall
11/9/2017	HB	12M	11	2017	Year-1	0	0	Fall
11/9/2017	HB	14M	11	2017	Year-1		1	Fall
11/9/2017	HB	8A	11	2017	Year-1	0	0	Fall
11/9/2017	HB	9A	11	2017	Year-1	0	0	Fall
11/9/2017	HB	11A	11	2017	Year-1	0	0	Fall
11/9/2017	HB	13A	11	2017	Year-1	0	0	Fall
11/10/2017	HB	1M	11	2017	Year-1	0	0	Fall
11/10/2017	HB	2M	11	2017	Year-1	0	0	Fall
11/10/2017	HB	3M	11	2017	Year-1	0	0	Fall
11/10/2017	HB	4M	11	2017	Year-1	0	0	Fall
11/10/2017	HB	5M	11	2017	Year-1	0	0	Fall
11/10/2017	HB	6M	11	2017	Year-1	0	0	Fall
11/10/2017	HB	7M	11	2017	Year-1	0	0	Fall
11/10/2017	HB	10M	11	2017	Year-1	0	0	Fall
11/10/2017	HB	12M	11	2017	Year-1	0	0	Fall
11/10/2017	HB	14M	11	2017	Year-1		1	Fall
11/10/2017	HB	8A	11	2017	Year-1	0	0	Fall

Date	Species	Site	Month	Year	Survey Year	Presence	Data Gap	Season
11/10/2017	HB	9A	11	2017	Year-1	0	0	Fall
11/10/2017	HB	11A	11	2017	Year-1	0	0	Fall
11/10/2017	HB	13A	11	2017	Year-1	0	0	Fall
11/11/2017	HB	1M	11	2017	Year-1	0	0	Fall
11/11/2017	HB	2M	11	2017	Year-1	0	0	Fall
11/11/2017	HB	3M	11	2017	Year-1	0	0	Fall
11/11/2017	HB	4M	11	2017	Year-1	0	0	Fall
11/11/2017	HB	5M	11	2017	Year-1	0	0	Fall
11/11/2017	HB	6M	11	2017	Year-1	0	0	Fall
11/11/2017	HB	7M	11	2017	Year-1	0	0	Fall
11/11/2017	HB	10M	11	2017	Year-1	0	0	Fall
11/11/2017	HB	12M	11	2017	Year-1	0	0	Fall
11/11/2017	HB	14M	11	2017	Year-1		1	Fall
11/11/2017	HB	8A	11	2017	Year-1	0	0	Fall
11/11/2017	HB	9A	11	2017	Year-1	0	0	Fall
11/11/2017	HB	11A	11	2017	Year-1	0	0	Fall
11/11/2017	HB	13A	11	2017	Year-1	0	0	Fall
11/12/2017	HB	1M	11	2017	Year-1	1	0	Fall
11/12/2017	HB	2M	11	2017	Year-1	1	0	Fall
11/12/2017	HB	3M	11	2017	Year-1	0	0	Fall
11/12/2017	HB	4M	11	2017	Year-1	0	0	Fall
11/12/2017	HB	5M	11	2017	Year-1	0	0	Fall
11/12/2017	HB	6M	11	2017	Year-1	0	0	Fall
11/12/2017	HB	7M	11	2017	Year-1	0	0	Fall
11/12/2017	HB	10M	11	2017	Year-1	0	0	Fall
11/12/2017	HB	12M	11	2017	Year-1	0	0	Fall
11/12/2017	HB	14M	11	2017	Year-1		1	Fall
11/12/2017	HB	8A	11	2017	Year-1	0	0	Fall
11/12/2017	HB	9A	11	2017	Year-1	0	0	Fall
11/12/2017	HB	11A	11	2017	Year-1	0	0	Fall
11/12/2017	HB	13A	11	2017	Year-1	0	0	Fall
11/13/2017	HB	1M	11	2017	Year-1	0	0	Fall
11/13/2017	HB	2M	11	2017	Year-1	0	0	Fall
11/13/2017	HB	3M	11	2017	Year-1	0	0	Fall
11/13/2017	HB	4M	11	2017	Year-1	0	0	Fall
11/13/2017	HB	5M	11	2017	Year-1	0	0	Fall
11/13/2017	HB	6M	11	2017	Year-1	0	0	Fall
11/13/2017	HB	7M	11	2017	Year-1	0	0	Fall
11/13/2017	HB	10M	11	2017	Year-1	0	0	Fall
11/13/2017	HB	12M	11	2017	Year-1	0	0	Fall
11/13/2017	HB	14M	11	2017	Year-1		1	Fall
11/13/2017	HB	8A	11	2017	Year-1	0	0	Fall
11/13/2017	HB	9A	11	2017	Year-1	0	0	Fall
11/13/2017	HB	11A	11	2017	Year-1	0	0	Fall

Date	Species	Site	Month	Year	Survey Year	Presence	Data Gap	Season
11/13/2017	HB	13A	11	2017	Year-1	0	0	Fall
11/14/2017	HB	1M	11	2017	Year-1	0	0	Fall
11/14/2017	HB	2M	11	2017	Year-1	0	0	Fall
11/14/2017	HB	3M	11	2017	Year-1	0	0	Fall
11/14/2017	HB	4M	11	2017	Year-1	0	0	Fall
11/14/2017	HB	5M	11	2017	Year-1	0	0	Fall
11/14/2017	HB	6M	11	2017	Year-1	0	0	Fall
11/14/2017	HB	7M	11	2017	Year-1	0	0	Fall
11/14/2017	HB	10M	11	2017	Year-1	0	0	Fall
11/14/2017	HB	12M	11	2017	Year-1	0	0	Fall
11/14/2017	HB	14M	11	2017	Year-1		1	Fall
11/14/2017	HB	8A	11	2017	Year-1	0	0	Fall
11/14/2017	HB	9A	11	2017	Year-1	0	0	Fall
11/14/2017	HB	11A	11	2017	Year-1	0	0	Fall
11/14/2017	HB	13A	11	2017	Year-1	0	0	Fall
11/15/2017	HB	1M	11	2017	Year-1	0	0	Fall
11/15/2017	HB	2M	11	2017	Year-1	0	0	Fall
11/15/2017	HB	3M	11	2017	Year-1	0	0	Fall
11/15/2017	HB	4M	11	2017	Year-1	0	0	Fall
11/15/2017	HB	5M	11	2017	Year-1	0	0	Fall
11/15/2017	HB	6M	11	2017	Year-1	0	0	Fall
11/15/2017	HB	7M	11	2017	Year-1	0	0	Fall
11/15/2017	HB	10M	11	2017	Year-1	0	0	Fall
11/15/2017	HB	12M	11	2017	Year-1	0	0	Fall
11/15/2017	HB	14M	11	2017	Year-1		1	Fall
11/15/2017	HB	8A	11	2017	Year-1	0	0	Fall
11/15/2017	HB	9A	11	2017	Year-1	0	0	Fall
[truncated: 565,429 more chars]
