# Supplementary material for: Passive acoustic monitoring of baleen whale seasonal presence across the New York Bight
Source: PLoS One. 2025 Feb 13;20(2):e0314857. doi: 10.1371/journal.pone.0314857 (PMC11825016; doi:10.1371/journal.pone.0314857)
Supplement: Attachment — Submitted filename: Edited_PLOS_ONE.docx [file pone.0314857.s018.docx]

PLOS ONE

Passive acoustic monitoring of baleen whale seasonal presence across the New York Bight

--Manuscript Draft--

| Manuscript Number: | PONE-D-24-13777 |
| --- | --- |
| Article Type: | Research Article |
| Full Title: | Passive acoustic monitoring of baleen whale seasonal presence across the New York  Bight |
| Short Title: | Seasonal acoustic presence of baleen whales in NY Bight |
| Corresponding Author: | Susan E. Parks, Ph.D.  Syracuse University  Syracuse, NY UNITED STATES |
| Keywords: | cetacean; protected species; baleen whale; seasonality; passive acoustic monitoring; management |
| Abstract: | The New York Bight is an ecologically and economically important marine region along the U.S. Atlantic Coast. Extensive assessments have characterized the habitats and biota in this ecosystem; however, most have focused on fishes, benthic habitats, and human impacts. To investigate the spatial and temporal occurrence of whales in this region, we conducted a three-year passive acoustic monitoring survey that  documented the acoustic presence of five baleen whale species that occur within the New York Bight and are of conservation concern: North Atlantic right whales  (<i>Eubalaena glacialis</i>), humpback whales (<i>Megaptera novaeangliae</i>), fin whales (<i>Balaenoptera physalus</i>), sei whales (<i>Balaenoptera borealis</i>), and blue whales (<i>Balaenoptera musculus</i>). Data were recorded with 14 bottommounted acoustic sensors across the continental shelf between 2017 and 2020. Right whales were detected across all seasons, with most detections in the autumn closer to New York Harbor and spring detections at sites closer to the continental shelf edge. Humpbacks were detected during all months of the year with varying distribution of detections across the shelf. Fin whales were detected at all sites on most days. Sei whales were detected primarily during the spring at offshore sites. Blue whales were detected in the winter at sites closer to the continental shelf edge, but were rare. These data improve our understanding of baleen whale seasonal occurrences in the New York Bight and can inform monitoring and mitigation efforts associated with the management and conservation of these species. |
| Order of Authors: | Bobbi J. Estabrook |
|  | Lisa A. Bonacci-Sullivan |
|  | Danielle V. Harris |
|  | Kristin B. Hodge |
|  | Ashakur Rahaman |
|  | Meghan E. Rickard |
|  | Daniel P. Salisbury |
|  | Matthew D. Schlesinger |
|  | Julia M. Zeh |
|  | Susan E. Parks |
|  | Aaron N. Rice |
| Additional Information: |  |
| Question | Response |
| Financial Disclosure | Yes |

| Enter a financial disclosure statement that describes the sources of funding for the work included in this submission. Review the [submission guidelines](http://journals.plos.org/plosone/s/submission-guidelines#loc-financial-disclosure-statement) for detailed requirements. View published research articles from [PLOS ONE](https://journals.plos.org/plosone/) for specific examples.  This statement is required for submission and will appear in the published article if the submission is accepted. Please make sure it is accurate. |  |
| --- | --- |
| Funded studies Enter a statement with the following details: - Initials of the authors who received each award - Grant numbers awarded to each author - The full name of each funder - URL of each funder website - Did the sponsors or funders play any role in the study design, data collection and analysis, decision to publish, or preparation of the manuscript? |  |
| Did you receive funding for this work? |  |
| Please add funding details.   as follow-up to "Financial Disclosure  Enter a financial disclosure statement that describes the sources of funding for the work included in this submission. Review the [submission guidelines](http://journals.plos.org/plosone/s/submission-guidelines#loc-financial-disclosure-statement) for detailed requirements. View published research articles from [PLOS ONE](https://journals.plos.org/plosone/) for specific examples.  This statement is required for submission and will appear in the published article if the submission is accepted. Please make sure it is accurate. | Funding for this study was provided by a contract from the New York State Department of Environmental Conservation (https://dec.ny.gov/) to ANR and SEP (Contract C009925). Scientists from NYSDEC (LAB-S, MER) and the NY State Natural Heritage Program (MDS) were involved in survey design and defining project goals, but did not have any influence over the interpretation or presentation of results. |

| Funded studies Enter a statement with the following details: - Initials of the authors who received each award - Grant numbers awarded to each author - The full name of each funder - URL of each funder website - Did the sponsors or funders play any role in the study design, data collection and analysis, decision to publish, or preparation of the manuscript? |  |
| --- | --- |
| Did you receive funding for this work?" |  |
| Please select the country of your main research funder (please select carefully as in some cases this is used in fee calculation).   as follow-up to "Financial Disclosure  Enter a financial disclosure statement that describes the sources of funding for the work included in this submission. Review the [submission guidelines](http://journals.plos.org/plosone/s/submission-guidelines#loc-financial-disclosure-statement) for detailed requirements. View published research articles from [PLOS ONE](https://journals.plos.org/plosone/) for specific examples.  This statement is required for submission and will appear in the published article if the submission is accepted. Please make sure it is accurate. | UNITED STATES - US |
| Funded studies Enter a statement with the following details: - Initials of the authors who received each award - Grant numbers awarded to each author - The full name of each funder - URL of each funder website - Did the sponsors or funders play any role in the study design, data collection and analysis, decision to publish, or preparation of the manuscript? |  |
| Did you receive funding for this work?" |  |
| Competing Interests | The authors have declared that no competing interests exist. |

| Use the instructions below to enter a competing interest statement for this submission. On behalf of all authors, disclose any [competing interests](http://journals.plos.org/plosone/s/competing-interests) that could be perceived to bias this work—acknowledging all financial support and any other relevant financial or nonfinancial competing interests.  This statement is required for submission and will appear in the published article if the submission is accepted. Please make sure it is accurate and that any funding sources listed in your Funding Information later in the submission form are also declared in your Financial Disclosure statement.  View published research articles from [PLOS ONE](http://journals.plos.org/plosone/) for specific examples. |  |
| --- | --- |
| NO authors have competing interests Enter: The authors have declared that no competing interests exist. Authors with competing interests Enter competing interest details beginning with this statement: I have read the journal's policy and the authors of this manuscript have the following competing interests: [insert competing interests here] |  |
| * typeset |  |
| Ethics Statement  Enter an ethics statement for this submission. This statement is required if the study involved:   - Human participants - Human specimens or tissue - Vertebrate animals or cephalopods - Vertebrate embryos or tissues | N/A |

| • Field research  Write "N/A" if the submission does not require an ethics statement.  General guidance is provided below. Consult the [submission guidelines](http://journals.plos.org/plosone/s/submission-guidelines#loc-guidelines-for-specific-study-types) for detailed instructions. Make sure that all information entered here is included in the Methods section of the manuscript. |  |
| --- | --- |

Format for specific study types

Human Subject Research (involving human

participants and/or tissue)

Give the name of the institutional review

board or ethics committee that approved the

study

•

Include the approval number and/or a

statement indicating approval of this

research

•

Indicate the form of consent obtained

(

written/oral) or the reason that consent was

not obtained (e.g. the data were analyzed

anonymously)

•

Animal Research (involving vertebrate

animals, embryos or tissues)

Provide the name of the Institutional Animal

Care and Use Committee (IACUC) or other

relevant ethics board that reviewed the

study protocol, and indicate whether they

approved this research or granted a formal

waiver of ethical approval

•

Include an approval number if one was

obtained

•

If the study involved

non-human primates

,

add

additional details

about animal welfare

and steps taken to ameliorate suffering

•

If anesthesia, euthanasia, or any kind of

animal sacrifice is part of the study, include

briefly which substances and/or methods

were applied

•

Field Research

Include the following details if this study

involves the collection of plant, animal, or

other materials from a natural setting:

Field permit number

•

Name of the institution or relevant body that

granted permission

•

Data Availability

Authors are required to make all data

underlying the findings described fully

available, without restriction, and from the

time of publication. PLOS allows rare

exceptions to address legal and ethical

concerns. See the

[PLOS Data Polic](http://journals.plos.org/plosone/s/data-availability)

[y](http://journals.plos.org/plosone/s/data-availability)

and

[FA](http://journals.plos.org/plosone/s/data-availability#loc-faqs-for-data-policy)

[Q](http://journals.plos.org/plosone/s/data-availability#loc-faqs-for-data-policy)

for detailed information.

No - some restrictions will apply

| A Data Availability Statement describing where the data can be found is required at submission. Your answers to this question constitute the Data Availability Statement and will be published in the article, if accepted. |  |
| --- | --- |
| Important: Stating ‘data available on request from the author’ is not sufficient. If your data are only available upon request, select ‘No’ for the first question and explain your exceptional situation in the text box. |  |
| Do the authors confirm that all data underlying the findings described in their manuscript are fully available without restriction? |  |
| Describe where the data may be found in full sentences. If you are copying our sample text, replace any instances of XXX with the appropriate details.   - If the data are held or will be held in a public repository, include URLs, accession numbers or DOIs. If this information will only be available after acceptance, indicate this by ticking the box below. For example: All XXX files   are available from the XXX database (accession number(s) XXX, XXX.).   - If the data are all contained within the manuscript and/or Supporting Information files, enter the following: All relevant data are within the manuscript and its Supporting Information files. - If neither of these applies but you are able to provide details of access elsewhere, with or without limitations, please do so. For example:   Data cannot be shared publicly because of [XXX]. Data are available from the XXX Institutional Data Access / Ethics Committee (contact via XXX) for researchers who meet the criteria for access to confidential data.  The data underlying the results presented in the study are available from (include the name of the third party | All relevant data products are included within the manuscript and/or Supporting Information files. Raw audio data (~30 TB) are in the process of being transferred to a public data repository. Raw audio data are also available upon request from NYS DEC. |

| and contact information or URL).  • This text is appropriate if the data are owned by a third party and authors do not have permission to share the data.  * typeset |  |
| --- | --- |
| Additional data availability information: |  |

Manuscript	[Click here to](https://www.editorialmanager.com/pone/download.aspx?id=35995281&guid=26d68b0c-42e6-4190-92fa-a0f0418604c9&scheme=1)

[access/download;Manuscript;EstabrookBJ_et_al_2024_PLOSO](https://www.editorialmanager.com/pone/download.aspx?id=35995281&guid=26d68b0c-42e6-4190-92fa-a0f0418604c9&scheme=1)

1 Passive acoustic monitoring of baleen whale seasonal presence across
2 the New York Bight

3

4 Bobbi J. Estabrook^1,*^, Lisa A. Bonacci-Sullivan^2^, Danielle V. Harris^1,3^, Kristin B. Hodge^1,4^,
5 Ashakur Rahaman^1^, Meghan E. Rickard^5^, Daniel P. Salisbury^1^, Matthew D. Schlesinger^6^, Julia 6	M. Zeh^7^, Susan E. Parks^7*^, Aaron N. Rice^1,8*^

7

8	^1^K. Lisa Yang Center for Conservation Bioacoustics, Cornell Lab of Ornithology, Cornell 9	University, Ithaca, New York, United States of America

10	^2^New York State Department of Environmental Conservation, Kings Park, New York, United 11	States of America

12 ^3^Present Address: Centre for Research into Ecological and Environmental Modelling, The
13 Observatory, University of St. Andrews, Buchanan Gardens, St. Andrews, Fife, United Kingdom
14 ^4^Present Address: Nicholas School of the Environment, Duke University Marine Laboratory, 15	Beaufort, North Carolina, United States of America

16	^5^New York Natural Heritage Program, College of Environmental Science and Forestry, State 17	University of New York, Kings Park, New York, United States of America

18	^6^New York Natural Heritage Program, College of Environmental Science and Forestry, State 19	University of New York, Albany, New York, United States of America

20 ^7^Department of Biology, Syracuse University, Syracuse, New York, United States of America
21 ^8^Department of Public and Ecosystem Health, College of Veterinary Medicine, Cornell 22	University, Ithaca, New York, United States of America
23 *** Corresponding authors:**
24 E-mail: sparks@syr.edu (SEP), arice@cornell.edu (ANR), bobbi.estabrook@cornell.edu (BJE)

25

26 **Keywords:** cetacean, protected species, baleen whale, seasonality, passive acoustic monitoring,
27 management
28 **Short title:** Seasonal presence of Baleen whales in the New York Bight

# Abstract

The New York Bight is an ecologically and economically important marine region along the U.S. Atlantic Coast. Extensive assessments have characterized the habitats and biota in this ecosystem; however, most have focused on fishes, benthic habitats, and human impacts. To investigate the spatial and temporal occurrence of whales in this region, we conducted a three year passive acoustic monitoring survey that documented the acoustic presence of five baleen whale species that occur within the New York Bight and are of conservation concern: North Atlantic right whales (*Eubalaena glacialis*), humpback whales (*Megaptera novaeangliae*), fin whales (*Balaenoptera physalus*), sei whales (*Balaenoptera borealis*), and blue whales (*Balaenoptera musculus*). Data were recorded with 14 bottom-mounted acoustic sensors across the continental shelf between 2017 and 2020. Right whales were detected across all seasons, with most detections in the autumn closer to New York Harbor and spring detections at sites closer to the continental shelf edge. Humpbacks were detected during all months of the year with varying distribution of detections across the shelf. Fin whales were detected at all sites on most days. Sei whales were detected primarily during the spring at offshore sites. Blue whales were detected in the winter at sites closer to the continental shelf edge but were rare. These data improve our understanding of baleen whale seasonal occurrences in the New York Bight and can inform monitoring and mitigation efforts associated with the management and conservation of these species.

# 1. Introduction

The New York Bight (NY Bight) is an ecologically important marine region within the mid-Atlantic of the U.S. Atlantic Exclusive Economic Zone and has significant environmental and economic value to New York State (NYS) and the United States more broadly. The NY

Bight extends along the U.S. East Coast from Long Island and New York City in the north to Delaware Bay in the south, and east to the continental shelf edge. The NY Bight is approximately 12,650 nm^2^ and includes many habitats and taxa. In the 1960s and 1970s, several ecological surveys and assessments were conducted to characterize the habitats and biota (1-7). However, despite this focused effort, there have been comparatively few systematic surveys for marine mammals in the NY Bight (1, 2, 5, 7).

Both the National Oceanic and Atmospheric Administration (NOAA) and the Department of Interior (DOI) recognized the potential importance of the NY Bight to marine mammals and conducted systematic aerial and ship-based visual survey efforts (8-11). These surveys included the Cetacean and Turtle Assessment Program (CETAP) that took place between 1979-1980 and had “fair” coverage of the NY Bight (11). The first systematic passive acoustic monitoring (PAM) surveys of this region were in 2008-2009, when a three-season PAM survey sought to characterize baleen whale occurrence (12). Later, a 2010-2011 PAM survey documented sustained occurrence of endangered North Atlantic right whales (*Eubalaena glacialis*; hereafter referred to as right whales) along the New Jersey Coast (13). More recently there have been several cetacean survey efforts, including environmental DNA (eDNA) detection (14) and the aggregation of opportunistic visual sightings (15). A PAM buoy deployed in the Empire Wind

Lease Area at the junction of two shipping lanes had a high number of right whale detections in November through March, with occasional detections over the summer, between 2016-2020

(16).

Due to planned expansion of anthropogenic activity of the NY Bight in the near future (NYSDEC and NYSDOS 17), several remote sensing surveys were initiated to characterize the occurrence of marine protected species in the NY Bight. New York State agencies commissioned aerial surveys in 2015 (NYSERDA) and later in 2017 aerial and PAM (NYSDEC) surveys across the NY Bight to systematically characterize cetacean occurrence. The aerial surveys identified spatial and temporal occurrence patterns of large whale species across the NY Bight; fin whales (*Balaenoptera physalus*) and humpback whales (*Megaptera novaeangliae*) were most frequently observed, right whales were sighted in all seasons except summer, and blue whales

(*Balaenoptera musculus*) and sei whales (*Balaenoptera borealis*) were rarely seen (18, 19). Data presented here presents passive acoustic survey results of vocalizing baleen whale occurrence across the NY Bight.

PAM is a particularly useful sensing tool for long-term, continuous monitoring of visually cryptic but highly vocal species, such as baleen whales (20-22). The primary research output from PAM establishes the spatial and temporal occurrence of focal vocal species, and, depending on the survey design, PAM can also provide more refined estimates of behavior, space use, or density and abundance (e.g., 23, 24-26). Due to the long-term deployment capabilities of modern recording instrumentation, data can be continuously collected over broad spatial scales, even when other surveys are not possible, including overnight and in inclement weather (e.g., poor visibility, high sea state). PAM requires that an animal is vocalizing, meaning that this survey type can only provide detection and non-detection data rather than true absence data. However, the long-term, continuous nature of PAM data allows for an analysis of temporal trends in species detections as well as other ambient noise conditions. Three recent studies aggregated PAM data for baleen whales across the U.S. Atlantic coast and synthesized the current scientific understanding of baleen whale occurrence, migration, and ecology across the Atlantic (see 26, 27, 28). Davis et al. (26, 27) identiying changes in the distribution of baleen whales before and after 2010, but these meta-analyses only included archival acoustic data through 2014.

As part of the NYSDEC New York Bight Whale Monitoring Program

(https://www.dec.ny.gov/lands/113647.html), we conducted a PAM survey from October 2017 to October 2020 in the NY Bight to investigate temporal and spatial trends in acoustic detections of five baleen whale species: North Atlantic right whales (*Eubalaena glacialis*), humpback whales (*Megaptera novaeangliae*), fin whales (*Balaenoptera physalus*), sei whales (*Balaenoptera borealis*), and blue whales (*Balaenoptera musculus musculus*). These data can inform a larger scale monitoring plan to mitigate the effects of expanded anthropogenic activities across the NY Bight on marine protected species and can provide insights into broader temporal presence trends in the NY Bight.

# 2. Materials and methods

## 2.1 Data collection

Passive acoustic data were collected using two different bottom-moored archival digital acoustic recording devices: Marine Autonomous Recording Units (MARUs; 29), and

Autonomous Multichannel Acoustic Recorders (AMARs, [http://www.jasco.com/amar)](http://www.jasco.com/amar). The

MARUs were suspended 2 m from the seafloor and recorded continuously with a sample rate of

5 kHz at 12-bit resolution, a high-pass filter at 10 Hz, and a low-pass filter at 2 kHz. The

effective recording bandwidth (10 - 2000 Hz) had a sensitivity of -168 dB ± 3.0 dB (re: 1V/µPa) with a flat frequency response between 15 and 585 Hz. Sites for which MARUs were used are named with “M” in their site-specific names. AMARs were suspended 1 m from the seafloor and recorded continuously at 8 kHz with a 24-bit resolution. The AMAR hydrophones were calibrated with a sensitivity of -164 dB (re: 1V/µPa at 1 kHz). Sites for which AMARs were used contain “A” in the site name.

Fourteen sites were configured across two transects along the Nantucket-Ambrose and Ambrose-Hudson Traffic Separation Schemes (Fig 1) to capture the sounds of cetaceans moving across the continental shelf near the primary NY Harbor shipping lanes. Data were collected from 16 October 2017 through 15 October 2020, with occasional gaps in the recording periods when units were trawled (i.e., dragged up by fishing vessels), lost (not recovered), or malfunctioned (Table 1, Fig 2, S1 Table).

**Table 1. Deployment location, depth, start date of first deployment, end date of last deployment, and total recorded days of passive acoustic surveys at each site in the New**

**York Bight, 2017-2020.**

| **Site** | **Latitude (°N)** | **Longitude (°W)** | **Depth (m)** | **Start Date** | **End Date** | **Total**  **Recording Days** |
| --- | --- | --- | --- | --- | --- | --- |
| **1M** | 40.348 | -71.224 | 90 | 2017-10-16 | 2020-10-15 | 1031 |
| **2M** | 40.342 | -71.606 | 84 | 2017-10-16 | 2020-07-09 | 679 |
| **3M** | 40.334 | -71.999 | 65 | 2017-10-16 | 2020-10-15 | 848 |
| **4M** | 40.327 | -72.408 | 54 | 2017-10-16 | 2020-10-05 | 693 |
| **5M** | 40.320 | -72.782 | 50 | 2017-10-16 | 2020-06-20 | 655 |
| **6M** | 40.284 | -73.152 | 40 | 2017-10-16 | 2020-06-20 | 727 |
| **7M** | 40.347 | -73.484 | 30 | 2017-10-16 | 2020-01-21 | 757 |
| **8A** | 40.414 | -73.762 | 28 | 2017-10-16 | 2019-10-15 | 492 |
| **9A** | 40.200 | -73.628 | 38 | 2017-10-16 | 2019-10-15 | 733 |
| **10M** | 40.033 | -73.500 | 49 | 2017-10-16 | 2020-10-15 | 856 |
| **11A** | 39.883 | -73.292 | 49 | 2017-10-16 | 2020-01-10 | 814 |
| **12M** | 39.734 | -73.106 | 51 | 2017-10-16 | 2020-10-15 | 896 |
| **13A** | 39.589 | -72.922 | 63 | 2017-10-16 | 2019-10-19 | 732 |
| **14M** | 39.440 | -72.730 | 80 | 2018-04-12 | 2020-10-15 | 801 |

**Fig 1. Map of the acoustic recording locations within the New York Bight, with sensor location numbers.**

“M” denotes MARUs (red), and “A” denotes AMARs (yellow). Inset shows the New York Bight at a larger spatial scale for geographical context. Shipping Areas to be Avoided represent shipping lanes and turning basins for vessel traffic in the NY Bight (data from marinecadastre.gov).

**Fig 2. Recording effort for each MARU and AMAR site during the three survey years (16 October 2017 - 15 October 2020).**

The multi-colored horizontal bars represent each site and indicate the time periods in which acoustic data were recorded. The white gaps along the site lines indicate times when acoustic data were not recorded (i.e. data gaps).

## 2.2 Acoustic detection of focal whale species

We used a combination of automated detection algorithms with human-aided visual and aural detection to determine the acoustic presence of right, humpback, fin, sei, and blue whales in the NY Bight. Here, “presence” refers to instances of acoustic detections of a focal species in the audio files. A lack of acoustic detection does not imply absence, rather that focal species vocalizations were not detected at that time. Automated detector algorithms were used to identify right, fin, and sei whale signals, and each detection was reviewed and validated by an analyst to confirm true positive detections for each day and site. Manual detection was used to identify humpback and blue whale presence, for which spectrograms of audio files were reviewed for each day and site. Audio files and detections were viewed as spectrograms using Raven Pro 2.0 Sound Analysis Software (30). All automatically or manually detected signals were subjected to a second verification process to improve data accuracy by eliminating false detections.

To evaluate daily acoustic presence of right whales at each site, we focused on right whale upcalls. Upcalls are one of the most common sounds produced by North Atlantic right whales (31-34), and are frequently used to determine right whale acoustic presence (e.g., 12, 35, 36, 37). Upcalls are produced by males and females of all age classes and appear to be produced in social contexts (38-40). They are characterized as a frequency-modulated upsweep with a typical duration between 0.3 and 1.5 s within a frequency band of approximately 71-224 Hz (41), with a bandwidth of 100 ± 37 Hz SD (42). A custom MATLAB-based (MathWorks, Natick, MA) automated detection algorithm was used to detect upcalls (43). Spectrogram settings for reviewing detections included a 60-s page duration, a frequency range of 10–450 Hz, and a fast Fourier transform (FFT) window size of 512 points for the 5 kHz MARU audio files and 2048 points for the 8 kHz AMAR files. Automated detection of right whale upcalls sometimes falsely detects humpback whale signals with similar acoustic properties (39, 44). To prevent these types of false positives, upcall detections with concurrent humpback whale signals were not included in the right whale presence analysis if the detection could not be distinguished from humpback sounds.

Humpback whale songs and social calls (non-song vocalizations) were used to determine humpback whale acoustic presence. Humpback whale songs have been recorded only from males, whereas social calls are produced by both sexes and all age classes in various contexts (e.g., 45, 46). Thus, song and social call detections account for male and female presence (e.g., 26, 47, 48-50). We manually reviewed 1 of every 4 h of audio for humpback signals. When tested using a subset of 50 days, the 25% subsample for humpback whale daily presence per site yielded the same results as manually reviewing 100% of the audio files. Spectrogram settings included a 5-min page duration, frequency range of 10–600 Hz, and a FFT window size of 512 points for MARU audio files and 1024 points for AMAR files.

Fin whales produce a song composed of long sequences of short individual notes centered around 20 Hz, and song bouts can last up to 32 h (51-53). Songs are hypothesized to be produced only by males in feeding and mating contexts (54, 55). Since the 20-Hz notes have been recorded in parts of the western North Atlantic throughout most of the year (26, 56) and over long durations, they are likely to be recorded while male fin whales are present in the NY Bight, and are, therefore, good indicators of male fin whale presence. We used a MATLABbased matched-filter data-template detector algorithm (57) to automatically detect 20-Hz notes in the audio files (58). Down-sampled 400-Hz audio files were used for this analysis, with spectrogram settings of a 90-s page duration, 0-100 Hz frequency band, and an FFT window size of 512 points.

Daily presence of sei whales was determined by using the template detector in Raven Pro 2.0 (30) to detect sei whale downsweeps, which are low-frequency (34-82 Hz) signals of approximately 1.4 s (59), and can occur singularly, in doublets, or triplets (60). It is not known if downsweeps are sex-specific or which behavioral context they are associated with. Six exemplars of sei whale downsweeps were selected from our dataset and used as templates for cross-correlation against continuous sound data to find instances of sei whale downsweeps.

Spectrogram settings comprised a 60-s page duration, 0–200 Hz frequency band, and an FFT window size of 1024 points for MARU audio files and 2048 points for AMAR audio files.

Blue whale daily presence was determined by manual review of down-sampled 400-Hz audio files for characteristic patterns of blue whale 14–22 Hz sounds. Blue whales in the North Atlantic produce song that is characterized by a sequence of phrases, most commonly composed of part A, a tonal signal, and part B, a descending tonal signal (referred to as the A-B phrase), between 15 and 20 Hz (61). Only male blue whales have been confirmed as singers (62). Spectrogram settings included a page length of 45 min and a frequency range of 10–25 Hz, with an FFT window size set to 1025 points.

## 2.3 Detector performance evaluation

Detector performance was evaluated using a subset of data spanning the 3-year survey period. Beginning with a random date (24 October 2017) through the end of the survey, we manually searched audio files for each instance of the target signals on every 20^th^ day, where between 1 and 3 sites were checked for every selected day, yielding a ‘ground-truthed’ dataset with 58 calendar days. Right whale acoustic presence was sparse in the ground-truthed dataset, so we reviewed every 10^th^ day during months when right whales were commonly detected in the NY Bight (November–May), yielding 80 calendar days (171 site-days) for the right whale ground-truthed dataset. The manually annotated ground-truthed datasets were compared against the detector output to determine true positive rates (TPR) on a daily scale. Days in which at least one automated detection overlapped in time and frequency with a ground-truthed signal were considered true positive (TP) days. Days in which there was at least one ground-truthed signal but no overlapping detections were defined as false negative (FN) days. The true positive rate (TPR) was the sum of TP days divided by the sum of the TP days and FN days for the corresponding target signal.

The TPR of the right whale upcall detector was 0.69, where 20 of the 29 days with presence had at least one TP detection and 9 days had FN. The fin whale detector had a TPR of 0.99, with 85 TP days and 1 FN day. The sei whale detector had a TPR of 0.33, with 7 TP days and 12 FN days. We observed many distorted, mode-dispersed sei whale downsweeps (S1 Fig) that might be due to signal propagation in the shallow water of the NY Bight (63-65). The detector had a lower TPR for downsweeps with multi-modal dispersion than for downsweeps that did not exhibit significant modal dispersion, likely because mode-dispersed downsweeps were not included in the template set. This suggests that downsweeps that originated near an acoustic sensor were more likely to be detected, as those signals are less susceptible to multimodal dispersion. The effect of the distorted sei whale signals on the downsweep template detector performance is described further in the Supporting Information.

## 2.4 Statistical analysis of whale acoustic presence

To describe seasonal acoustic presence of each focal species, we used generalized additive models (GAM). A GAM allows for non-linear relationships between the response and predictor variables by using smoothed functions, providing more flexibility when modeling temporal trends. With these models, we tested if there was monthly variation in species presence and if species presence differed between survey years. Models were fitted using the ‘bam’ function of the ‘mgcv’ package (66; https://cran.r-project.org/web/packages/mgcv/index.html) in

R (67), and were structured with daily detection per site as a Bernoulli response variable

(detection/no detection) using a binomial distribution with a logit link function. Predictor variables included month as an integer with a cyclic smooth function and survey year as a factor. We defined survey years as follows: Year 1 (October 16, 2017 – October 15, 2018), Year 2 (October 16, 2018- October 15, 2019), and Year 3 (October 16, 2019 – October 15, 2020). Site as a factor was included as a random effect. To address temporal autocorrelation from daily samples, a continuous-time first-order autoregressive correlation structure (AR1) was included in the model. Models were fitted separately for each species using the same parameters between models. To compare the goodness of fit and model complexity of the GAM with the AR1 and the GAM without the AR1, we compared the Akaike information criterion (AIC), where the model with the lowest AIC was selected as the better fitting model. Model checking was conducted using the ‘DHARMa’ package (68) in R to confirm that model assumptions (e.g., homoscedasticity, independence, normality, dispersion, and zero-inflation) were not violated, following Zuur et al. 2009 (69). To make the results of the GAM more interpretable, model estimates were back-transformed and the estimated marginal means (EMM) of the response variable on the response scale are presented, which represent the proportion of time with presence per month and year using the ‘emmeans’ package in R (70). A statistical pair-wise comparison of the estimated marginal means between each survey year was conducted using the tukey method on the log odds ratio scale.

## 2.5 Detection range estimation

Each of the focal whale species’ signals has a different detection distance, which can be affected by habitat features and noise conditions in the environment. To better understand the potential area of coverage for this survey, we estimated approximate feasible detection ranges of each species in the NY Bight using an assumed source level of each target signal (Table 2), the ambient noise levels in the target signal frequency band, and physical and oceanographic factors in the study area that can influence sound propagation of the signal.

**Table 2. Referenced signal type, frequency band, source levels and source depth of the target species used for ambient noise calculations and detection range estimation.**

| **Species** | **Target Signal** | **Frequency Band** | **1/3^rd^ Octave Frequency Band** | **Source Level (dB re 1μPa @ 1m**  **RMS) ± SD** | **Source Depth** |
| --- | --- | --- | --- | --- | --- |
| **North Atlantic Right Whale** | Upcall | 71 – 224 Hz (41) | 70.8 – 224 Hz | 172 ± 6.6 (32, 71) | 2 m (40) |
| **Humpback Whale** | Song | 29 – 2480 Hz  (Dunlop et al.  2007) | 17.8 – 708 Hz | 169 ± 3 (72) | 50 m  (73) |
| **Fin Whale** | 20-Hz pulse | 17 – 25 Hz (53) | 17.8 – 28.2 Hz | 189 ± 4 (74, 75) | 20 m  (76) |
| **Sei Whale** | Downsweep | 34 – 82 Hz (59) | 28.2 – 89.2 Hz | 173.5 ± 3.2 (77) | 20 m  (63) |
| **Blue Whale** | Song | 15 – 19 Hz (61) | 14.1 – 22.4 Hz | 189 ± 3 (78) | 30 m  (79) |

Ambient noise levels were quantified as equivalent continuous sound pressure level, L_eq_ (dB_rms_ re: 1 μPa) (e.g., see 80), and measured from the audio data using SEDNA (81) in

MATLAB, with a Hann window with zero overlap, FFT with a resolution of 1 s and 1 Hz. The L_eq_ represents the average unweighted sound level of a continuous time-varying pressure signal over intervals of 10 min, a temporal resolution that captures changes in noise levels from a passing vessel (71), a dominant sound source in the NY Bight. Species-specific frequency bands were used to represent the frequency range in which each species’ hearing is believed to be most sensitive (Table 2), and the bandwidth in which each whale species’ target signals occur (28, 45, 71, 74). We included a “full band” (8.9 – 2239.6 Hz) to describe broadband noise conditions during the survey period.

Detection range was estimated using the following transmission loss (TL) model:

 (1)

where *H* is the depth of the source (considered to be the transition range from spherical to intermediate spherical/cylindrical spreading), and *R* represents the range of the source to the receiver. The calculated TL is then used to estimate the received level (RL) of the sound when it reaches the receiver using the passive sonar equation (e.g., 82): RL = SL – TL. The estimated detection range is the distance from the source at which the RL equals the ambient noise level. The intermediate (relative to cylindrical and spherical spreading) 17log_10_R spreading loss model was used in a custom MATLAB package to estimate sound propagation. The model incorporates bathymetry in its calculation of propagation by using spherical spreading at the approximate water depth and intermediate spreading beyond that calculated threshold (e.g., 83, 84). We estimated the detection range of each whale species at specific range steps for eight different bearings relative to the sensor (0°, 45°, 90°, 135°, 180°, 225°, 270°, 315°) at each site location to account for varying bathymetry around each sensor, during high (95^th^ percentile), median (50^th^ percentile), and low (5^th^ percentile) noise conditions. Depths of the sources and source levels used in the models are presented in Table 2. The detection range estimates presented here do not illustrate temporal variation as a function of noise levels on the seasonal or annual scale, but rather overall noise levels during the survey. Therefore, we present seasonal and annual L_eq_ statistics in the Supporting Information to reflect potential temporal variation in relative detection range estimates.

# 3. Results

## 3.1 Acoustic presence of focal whale species

During the survey period (16 October 2017 - 15 October 2020), 1095 calendar days of audio data were collected, during which all 5 baleen whale species were acoustically detected with varying seasonal and spatial distributions across the NY Bight (Figs 3 and 4, Table 3). For each species, the best fitting GAM was the model that incorporated the AR1 structure, and the model assumptions were met.

**Table 3. Daily acoustic detections per species by season and year in the New York Bight during Fall: September – November, Winter: December – February, Spring: March – May, and Summer: June - August. ‘Total Days Recorded’ is the total calendar days recorded, ‘# Days Detected’ refers to the number of calendar days with detections. ‘% Days Detected’ is the percentage of recording days with a detection of the species.**

| **Season** |  | **Right** | | **Humpback** | | **Fin** | | **Sei** | | **Blue** | |
| --- | --- | --- | --- | --- | --- | --- | --- | --- | --- | --- | --- |
|  | **Total Days** | **# Days**  **Detected** | **% Days Detected** | **# Days**  **Detected** | **% Days Detected** | **# Days**  **Detected** | **% Days Detected** | **# Days**  **Detected** | **% Days Detected** | **# Days**  **Detected** | **% Days Detected** |
| **Fall 2017** | 46 | 27 | 59% | 14 | 30% | 46 | 100% | 17 | 37% | 3 | 7% |
| **Winter 2017-2018** | 90 | 66 | 73% | 82 | 91% | 90 | 100% | 22 | 24% | 23 | 26% |
| **Spring 2018** | 92 | 55 | 60% | 79 | 86% | 90 | 98% | 81 | 88% | 0 | 0% |
| **Summer 2018** | 91 | 14 | 15% | 88 | 96% | 91 | 99% | 15 | 16% | 0 | 0% |
| **Fall 2018** | 91 | 26 | 29% | 85 | 92% | 91 | 100% | 8 | 12% | 0 | 0% |
| **Winter 2018-2019** | 90 | 68 | 76% | 72 | 80% | 90 | 100% | 2 | 2% | 7 | 8% |
| **Spring 2019** | 92 | 77 | 84% | 88 | 96% | 92 | 100% | 88 | 96% | 0 | 0% |
| **Summer 2019** | 92 | 32 | 35% | 86 | 93% | 92 | 100% | 24 | 26% | 0 | 0% |
| **Fall 2019** | 91 | 17 | 19% | 78 | 86% | 91 | 100% | 7 | 8% | 0 | 0% |
| **Winter 2019-2020** | 91 | 33 | 36% | 64 | 70% | 88 | 97% | 31 | 34% | 17 | 19% |
| **Spring 2020** | 92 | 23 | 25% | 36 | 39% | 92 | 100% | 82 | 89% | 0 | 0% |
| **Summer 2020** | 92 | 15 | 16% | 50 | 54% | 90 | 98% | 16 | 17% | 0 | 0% |
| **Fall 2020** | 45 | 5 | 11% | 43 | 96% | 45 | 100% | 0 | 0% | 0 | 0% |
| **Total** | 1095 | 458 | 42% | 865 | 79% | 1088 | 99% | 393 | 36% | 50 | 5% |

16

### 3.1.1 North Atlantic right whale presence

North Atlantic right whale upcalls were detected on 42% (458) of the days (Table 3), during each month of the year, with a peak in detections during winter months (November – January), a small secondary peak in spring months (March – May), and the fewest detections in July through October (Fig 3a, Table 3, S2 Table). Right whales were detected at all recording sites (Fig 4a), with the fewest detections at site 8A (3% of days) and most detections at sites 9A and 11A (14% and 15% of days) (S3 Table). Right whales demonstrated strong seasonal spatial patterns, with more detections at sites closer to the continental shelf edge from March to May, and at sites nearer to NY Harbor from November to February (S2 Fig). There was a significant effect of survey year on daily detections (GAM: *X*^2^ = 16.72, d.f. = 2, *p* <.001), where the probability of upcall detections occurring during Year 1 (EMM = 0.102, SE = 0.017, d.f. =

10688) and Year 2 (EMM = 0.106, SE = 0.015, d.f. = 10688) were significantly different (*p* <.005) from Year 3 (EMM = 0.038, SE = 0.009, d.f.= 10688), while Year 1 and Year 2 were not significantly different from each other (Tukey adjustment *p* value = 0.970) (Fig 3b). During Year

1 and Year 2, right whales were detected most at sites 9A, 11A, and 13A, comprising approximately 27% and 31% of the daily upcall detections at those sites during each year, respectively. However, the sensors at those sites experienced substantial data loss during Year 3 (Fig 2), limiting our interpretation of these interannual differences in presence.

### 3.1.2 Humpback whale presence

Humpback whale vocalizations were detected on 79% (865) of the recording days during the survey period, and in all months of the year, with variable presence across seasons (Fig 3c,

Table 3, S2 Table). Humpback whales were detected across all sites, with the fewest detections

at site 8A (9% of recorded days), while daily detections at other sites ranged between 21% (site 2M) and 53% (site 13A) of the recording days (Fig 4b, S3 Table). Humpback whales demonstrated seasonal spatial patterns, where more detections occurred at sites nearer to NY

Harbor between November and March, and at sites nearer to the shelf edge between July and

September (S3 Fig). Survey year had a significant effect on daily humpback whale detections (GAM: *X*^2^ = 34.84, d.f. = 2, p <001), and each survey year was significantly different (Tukey adjusted p value < .001) from the others, where Year 1 (EEM = 0.522, SE = 0.047, d.f. = 10687) had the highest probability of detections, followed by Year 2 (EEM = 0.252, SE = 0.034, d.f. = 10687), and Year 3 (EEM = 0.090, SE = 0.018, d.f. = 10687) (Fig 3d). However, the sites missing from the Year 3 survey (9A, 11A, and 13A) accounted for 12% and 10% of all daily detections during Year 1 and Year 2.

### 3.1.3 Fin whale presence

Fin whale 20-Hz pulses were the most frequently detected among baleen whale signals, with detections on 99% (1088 days) of the recording days (Table 3, S2 Table). While prominent throughout the survey, fin whales exhibited slight seasonal trends in presence, with the fewest detections between April and June (Fig 3e and S4 Fig). Fin whales were detected >50% of the days at most sites, with the fewest daily detections at sites near NY Harbor and more detections at sites closer to the continental shelf edge (Fig 4c and S4 Fig), demonstrating some spatial variability of fin whale detections throughout the NY Bight. Survey year was a significant predictor of daily detections at all sites (*X*^2^ = 44.21, d.f. = 2, p < 0.001), where the probability of daily detections during Year 1 (EMM = 0.546, SE = 0.084, d.f. = 10687) was significantly lower (p < 0.001) than Year 2 (EMM = 0.776, SE = 0.058, d.f. = 10687) and Year 3 (EMM = 0.798, SE = 0.056, d.f. = 10687), indicating interannual differences in presence (Fig 3f).

### 3.1.4 Sei whale presence

Sei whale downsweeps were detected during all months of the year and on 36% (393 days) of the recorded days. Sei whale detections exhibited strong seasonality where a single peak in daily detections occurred during spring months, between March and May (Fig 3g, Table 3, S5 Fig, S2 Table) in all three survey years. Sei whales were detected most at sites near the continental shelf edge (Sites 2M: 26% days; 14M: 23%; 1M: 19%) and the least at sites closer to NY Harbor (Figs 4d and S5), with daily detections ranging between 1% (sites 7M and 8A) and 5% (sites 5M, 6M, and 9A) of the recording days. The probability of daily sei whale detections in the NY Bight were not significantly different (p > 0.05) between Year 1 (EMM = 0.286, SE = 0.082, d.f. = 10687), Year 2 (EMM = 0.353, SE = 0.090, d.f. = 10687), and Year 3 (EMM = 0.330, SE = 0.870, d.f. = 10687), indicating low interannual variability in sei whale detections in the NY Bight during this survey (Fig 3h).

### 3.1.5 Blue whale presence

Blue whales were the least frequently detected species (Table 3, S2 Table), with acoustic detections on 5% (50) of the recorded days. Blue whales were detected between November and

February only, and only at sites near the continental shelf edge (Fig 4e and S6 Fig). Sites 1M and 2M recorded the most days with blue whale detections, where 4% (42 days) and 5% (36 days) of the recorded days had detections, respectively, while detections at the remaining sites occurred on 0% – 2% of the recorded days. Since there were very few days with detected blue whale song, it was not appropriate to test for temporal trends statistically. Daily detections varied little between Year 1 (26 days, 7%), Year 2 (7 days, 2%), and Year 3 (18 days, 5%).

**Fig 3. GAM plots of daily acoustic presence of right whales, humpback whales, fin whales and sei whales** (panels a, c, g, e)**,** with the estimated marginal means of the probability of presence on the y-axis by month and survey year (panels b, d, f, h). For panels a, c, g, e, Survey Year 1 (October 2017-2018) is indicated by a red line, Year 2 (October 2018-2019) is indicated by a blue line, and Year 3 (October 2019–2020) is indicated by a yellow line.

**Fig 4. Detection area and cumulative spatial distribution of focal whale species detected between 16 October 2017 and 15 October 2020.** The size of the circles around each site represents the estimated detection area based on the 5th, 50th, and 95th noise percentiles (the 5th percentile detection areas were excluded from the fin whale and blue whale plots because they encompassed the entire map area). The colormap is scaled to the proportion of days with detections for each species over total days recorded, where white represents 0 presence and deep red represents the highest presence for each species.

## 3.2 Detection range estimation

### 3.2.1 Ambient noise measurements

Noise levels varied across the NY Bight (Fig 5), where site 8A recorded the highest median L_eq_ within the right whale, humpback whale, and sei whale frequency bands, limiting the range of detectability for those species’ target signals. In all frequency bands, median L_eq_ were generally lower at sites 4M – 7M, 9A, and 10M, while sites nearer to the shelf edge recorded slightly higher L_eq_ values. The full and humpback whale frequency bands, the broadest frequency bands measured here, recorded the overall highest L_eq_, followed by the sei and right whale frequency bands (Fig 5). Overall, noise levels were lowest during summer (June - August) and highest during fall (September - November) and winter (December - February) (S7-S12 Figs) for all frequency bands.

**Fig 5. Boxplots of 10-minute averaged Leq (dB re 1μPa) per site for each frequency band between 16 October 2017 and 15 October 2020.**

### 3.2.2 Detection range by species

During median noise conditions, right whale upcalls were estimated to have an average detection range of 7 km (SE = 0.38) across all sites and bearings for a source level of 172 dB. Median humpback whale song detection range estimates were 3 km (SE = 0.14) for a source level of 169 dB. Humpback whale social calls, which were also detected in this study, are produced at variable source levels. They have lower source levels than song (164 dB for social songs vs. 170 dB for song; described in 85) and would therefore have a smaller detection range. Sei whale detection range estimates were approximately 11 km (SE = 0.63) for 173.5 dB source level. Fin and blue whale detection ranges were longest, with estimated ranges exceeding 160 km for both species, based on median noise levels within their respective frequency bands (SE = 16 and 12 km, respectively). Detection range estimates for each noise percentile and source level per species’ frequency band can be found in S5 Table.

# 4. Discussion

The results represent a comprehensive, multi-year continuous PAM survey of right, humpback, fin, sei, and blue whale presence in the NY Bight. Right, fin whales and humpback whales were detected in the NY Bight throughout most of the year, strengthening the evidence (19, 86) that this region serves as more than just the migratory corridor that was previously suggested (87-90). Baleen whale detections were distributed broadly across the NY Bight. Fin, sei, and blue whales were detected primarily at sites closer to the shelf edge, while right and humpback whales were detected more broadly across the shelf (Fig 4).

The year-round temporal occurrence of baleen whale species in the NY Bight raises several interesting questions about how the region fits into the life history of these species and has direct implications for management. The year-round occurrence of right whales and humpback whales in the NY Bight challenges previous hypotheses that this region is primarily a stopover location as part of their migration paths. Additionally, it is unclear how most baleen whales are using this habitat. The consistent presence of many baleen whale species suggests that seasonal management actions, such as Seasonal Management Areas near ports that restrict vessel speed or seasonal exclusions associated with offshore construction activity that limit the loudest actions to certain months, may be insufficient to mitigate impacts to these protected species.

## 4.1 Baleen whale ecological patterns

Right whales were most frequently acoustically detected in the NY Bight from fall through spring, with presence >5 days/week for most of that period across years. Right whales were detected least frequently during summer months. The U.S. Mid-Atlantic and the NY Bight are considered part of the right whale migratory corridor (87, 88), and while is it unclear exactly how right whales use the NY Bight, their extended acoustic presence in the NY Bight during all months of the year suggests that they may not be solely migrating through this region. Similar patterns of extended seasonal occurrence of right whales outside of migration periods have been observed in other areas along their migration corridor (27, 35-37, 91). Right whales have been observed feeding as far south as Nantucket Shoals (92), and Zoidis et al. (19) observed one instance of a feeding right whale in the NY Bight during the NYSDEC aerial surveys that were contemporaneous with our passive acoustic survey. Right whales were detected predominantly near the shelf edge during spring months, likely during their northward migration to feeding grounds in the Gulf of Maine, and closer to shore during fall months when pregnant females transit southward to calving grounds in southeastern US waters (87, 88). These spatial patterns corroborate those from the visual surveys conducted by Zoidis et al. (19). However, right whales were not visually observed during summer months, while they were acoustically detected during June and July in these data and by Murray et al. (16), illustrating the important data gap PAM can fill. Muirhead et al. (12) also described a similar spatial pattern in seasonal detections in the NY Bight in 2008. Detection range estimates indicate right whale presence within and around the high-use Nantucket-Ambrose and Ambrose-Hudson Traffic Separation Schemes.

Humpback whales were among the more frequently detected baleen whale species throughout the NY Bight, with year-round acoustic presence that increased in winter and spring. Both humpback whale song and non-song calls were detected in the NY Bight, indicating a range of humpback behaviors occurring in this region, including advertisement and foraging (86, 93). Humpback whale songs have regularly been detected in feeding grounds in the Western North Atlantic Ocean (50, 73, 94, 95), and it is thought that singing in feeding grounds may precede and follow migratory movements (96).

Fin whales were acoustically detected on nearly every day in the NY Bight, which is consistent with previous observations in the mid-Atlantic and Gulf of Maine (12, 26, 56), and in the NY Bight in 2008 – 2009 (12). The 2008-2009 passive acoustic data show the fewest acoustic detections of fin whales in the fall and highest in winter in the NY Bight, while summer months were not surveyed (12). Our survey had fewer detections in spring and more in fall and winter. Fin whales had higher detection rates at offshore sites, which may be in part due to the long-range propagation distance of fin whale song (75, 97, 98) originating off the continental shelf. However, the spatial pattern in fin whale acoustic detections observed in this survey suggests that fin whales were within the NY Bight, supported by visual observations during concurrent aerial surveys (19).

The NY Bight likely represents the southerly extent of the range for most sei whales (99), though a low incidence of acoustic detection has been found farther south (26). Our data present one of the most extensive time-series records of sei whale acoustic occurrence collected to date. All three years of survey data show that March through mid-June is the peak detection period for sei whales in the NY Bight, though sei whales were detected during each season. Sei whales have only recently been the focus of PAM surveys (26, 59, 60, 100), and data on their occurrence in the Gulf of Maine and mid-Atlantic region have only recently been analyzed (26). Davis et al. (26) reported similar seasonal occurrence patterns of sei whales in 2008-2009. Sei whales were detected mostly at sites near the shelf edge, consistent with concurrent visual observations (19). The TPR of the sei whale detector (0.33) suggests that more than half of the downsweeps were not detected; however, sei whales were detected on more days throughout the year during the acoustic survey than in visual surveys (19), illustrating that PAM methods are beneficial for long-term monitoring of sei whale presence in the NY Bight. For comparison, visual sightings of sei whales were rare between 2017 and 2020 (2 sightings) and occurred only in April, while sei whales were acoustically detected during every season, with a peak in spring months. Considering the estimated short detection ranges for sei whale downsweeps and the superior detector performance on downsweeps that were not mode dispersed (i.e., the source was close to the site), detected downsweeps were most likely produced by sei whales near the recording sites. We do not suspect that the rate of missed downsweeps by the detector was biased by time, in which case these data represent broad temporal trends of vocalizing sei whale occurrence in the NY Bight, which align with trends observed in the 2008-2009 acoustic data

(26).

Blue whales were detected least frequently among the focal species, consistent with the 2008-2009 survey (12, 26) and visual surveys (19). Future studies should investigate how far into the NY Offshore Planning area blue whales are travelling. Given the long detection range of blue whale song and spatial pattern of acoustic detections, it is likely that most of the detected blue whales in this survey were off the shelf or along the shelf edge. During concurrent aerial surveys, the only blue whale observations were on the continental shelf and at the shelf edge, suggesting that blue whales may not utilize the NY Bight waters. A single blue whale was acoustically tracked by Muirhead et al. (12) on the shelf, which may indicate that individuals transit through the planning area. Blue whales elsewhere in the western North Atlantic have been acoustically detected more off the continental shelf or near the shelf edge than in waters closer to shore (26).

## 4.2 Ambient noise and detection range estimates

At the northeast convergence of the two shipping lanes, site 8A consistently recorded the highest noise levels. Sites adjacent to 8A, where large vessels reduce speed or anchor, recorded lower noise levels. Considering the placement of the recording sites along two major shipping lanes (Fig 1), vessel noise is likely the most dominant contributor to frequencies between 50 and 500 Hz throughout the recording area. Elevated ambient noise levels near shipping lanes can limit the acoustic detection range of low frequency (< 1 kHz) baleen whale signals to less than 10 km and potentially restrict their conspecific communication space through increased acoustic masking (41, 71, 85). We found that noise levels varied by season, which may affect communication space between seasons, but did not differ greatly between survey years.

While our detection range estimates merely provide approximate areas of acoustic coverage for each target species, they were similar to estimates in nearby regions of the western North Atlantic continental shelf (20, 59, 85), with the exception of fin whale detection range estimates. Fin whale detection ranges in the NY Bight were estimated to exceed 150 km from the source, while in Massachusetts Bay, fin whale detection ranges were estimated to be < 50 km (85). We note that our estimated detection ranges for these species are based on noise conditions along the two major shipping lanes of the NY Bight at the time of this survey and are not representative of other regions within the NY Bight that are farther from the noise of the shipping lanes.

## 4.3 Implications

With the recent intensive, multi-modal, marine mammal survey efforts in the NY Bight, this region has gone from being a significant data gap to now one of the most intensively studied regions along the U.S. Atlantic EEZ. Each of these survey methods – systematic or opportunistic visual surveys, eDNA, and passive acoustics – has advantages and limitations, but when combined, they can yield a more comprehensive understanding of the ecology of cryptic taxa. Visual surveys have long been the most common survey tool for monitoring the presence and density of marine mammals at sea. Visual surveys (18, 19, 101) provide data including species identification, the number of different individuals present, geographic location, and potentially behavioral information, yet are difficult to conduct at night or in other low visibility conditions. Opportunistic survey data (15) can be useful for establishing the presence of protected species, but cannot reliably demonstrate absence of animals in this area due to the lack of systematic effort. eDNA is an emerging survey method with tremendous promise of single or multi-species detection (e.g., 102, 103, 104), including marine mammals (14, 105), though questions of detection probability remain (106, 107). Passive acoustic monitoring is a relatively low-cost way to increase the spatial and temporal survey coverage for marine mammals that generate sounds; it allows for continuous surveillance of the species of interest, providing coverage at night and in poor weather conditions, when visual surveys from aerial or shipboard platforms are not feasible (22), and allows for detection of animals below the surface. Extensive aerial and now passive acoustic survey efforts for monitoring whale occurrence in the NY Bight provides new insights into their seasonal and spatial presence and can help inform more effective conservation and management strategies to minimize impacts to these protected species, yet allow for sustainable marine spatial planning and human use of this region. Integrated analysis of different survey methods (e.g., 20, 108) may provide further opportunities to optimize large scale sustained monitoring efforts for protected species to guide sustainable marine spatial planning in areas of high human use and biological importance.

**Conflict of interest**

None

# Author contributions

Conceptualization and analysis design: L.A.B., M.E.R., M.D.S., B.J.E., S.E.P., A.N.R.

Data collection and curation: B.J.E., K.B.H., A.R., D.P.S., A.N.R.

Formal analysis: B.J.E., D.V.H., K.B.H., A.R., D.P.S., J.M.Z., S.E.P., A.N.R.

Wrote the paper: B.J.E., D.V.H., J.M.Z., S.E.P., A.N.R.

# Acknowledgements

This work could not have been completed without major contributions from several key individuals. Special thanks to Captain Fred Channel and Derek Jaskula for deploying and recovering recording devices in a wide range of seafaring conditions, to Deborah Cipolla-Dennis, Linda Harris, Edward Moore, Tish Klein, and Holger Klinck for invaluable administrative support, to Christopher Tessaglia-Hymes and Raymond Mack for engineering support, to Peter Dugan for optimizing and running automated detection algorithms, to Michael Pitzrick for software support, to Dimitri Ponirakis for ambient noise and detection range software development and support, and to Christopher Pelkie for assisting with data management. We thank JASCO Applied Sciences for AMAR and AURAL support.

# References

1. National Oceanic and Atmospheric Administration (NOAA). Marine EcoSystems

Analysis Program: Bibliography of the New York Bight. Rockville, MD: National Oceanic and Atmospheric Administration, Office of Coastal Management, Environmental Science Information Center; 1974.

2. National Oceanic and Atmospheric Administration (NOAA). Marine EcoSystems

Analysis (MESA) Program: The New York Bight Project - 1975; Stony Brook, Long Island, New York. Boulder, CO: NOAA Special Report. National Oceanic and Atmospheric Administration, Environmental Research Laboratories; 1976.

3. Grosslein MD, Azarovitz TR. Fish Distribution. Albany, NY: Marine Ecosystems Analysis (MESA) Program, MESA New York Bight Atlas Monograph 15. New York Sea Grant Institute; 1981.
4. Pearce JB, Radosh DJ, Caracciolo JV, Steimle FW. Benthic Fauna. Albany, NY: Marine Ecosystems Analysis (MESA) Program, MESA New York Bight Atlas Monograph 14. New York Sea Grant Institute; 1981.
5. Mayer GF, editor. Ecological Stress and the New York Bight: Science and Management. Columbia, SC: Estuarine Research Federation; 1982.
6. Menza C, Kinlan BP, Dorfman DS, Poti M, Caldow C, editors. A Biogeographic

Assessment of Seabirds, Deep Sea Corals and Ocean Habitats of the New York Bight: Science to Support Offshore Spatial Planning. Silver Spring, MD: NOAA Technical Memorandum NOS NCCOS 141, National Oceanic and Atmospheric Administration; 2012.

7. U.S. Army Corps of Engineers (USACE). New York Bight Study: An Annotated

Biblography of the New York Bight: Emphasis on Biological Studies. Vicksburg, MS: Technical Report EL-94-11. U.S. Army Corps of Engineers, Waterways Experiment Station; 1994.

8. Palka DL, Chavez-Rosales S, Josephson E, Cholewiak D, Haas HL, Garrison L, et al.

Atlantic Marine Assessment Program for Protected Species: 2010-2014. Washington, DC: U.S. Department of the Interior, Bureau of Ocean Energy Management, Atlantic OCS Region. OCS Study BOEM 2017-071; 2017.

9. Cetacean and Turtle Assessment Program (CETAP). A Characterization of Marine

Mammals and Turtles in the Mid- and North-Atlantic Areas of the U.S. Outer Continental Shelf, Annual Report for 1979. Contract AA551-CT8-48. Washington, DC: Bureau of Land Management. NTIS# PB-81-243289; 1981.

10. Cetacean and Turtle Assessment Program (CETAP). A Characterization of Marine

Mammals and Turtles in the Mid- and North-Atlantic Areas of the U.S. Outer Continental Shelf, Annual Report for 1980. Contract AA551-CT8-48. Washington, DC: Bureau of Land Management. NTIS# PB-83-149906; 1982.

11. Cetacean and Turtle Assessment Program (CETAP). A Characterization of Marine

Mammals and Turtles in the Mid- and North-Atlantic Areas of the U.S. Outer Continental Shelf, Final Report. Contract AA551-CT8-48. Washington, DC: Bureau of Land Management. NTIS# PB-83-149906; 1982.

12. Muirhead CA, Warde AM, Biedron IS, Mihnovets AN, Clark CW, Rice AN. Seasonal acoustic occurrence of blue, fin, and North Atlantic right whales in the New York Bight. Aquatic Conservation: Marine and Freshwater Ecosystems. 2018;28(3):744-53.
13. Whitt AD, Dudzinski K, Laliberte JR. North Atlantic right whale distribution and seasonal occurrence in nearshore waters off New Jersey, USA, and implications for management. Endangered Species Research. 2013;20(1):59-69.
14. Alter SE, King CD, Chou E, Chin SC, Rekdahl M, Rosenbaum HC. Using environmental DNA to detect whales and dolphins in the New York Bight. Frontiers in Conservation Science. 2022;3:820377.
15. Chou E, Rekdahl ML, Kopelman AH, Brown DM, Sieswerda PL, DiGiovanni RA, et al. Occurrence of baleen whales in the New York Bight, 1998–2017: insights from opportunistic data. Journal of the Marine Biological Association of the United Kingdom. 2022;102(6):438-44.
16. Murray A, Rekdahl ML, Baumgartner MF, Rosenbaum HC. Acoustic presence and vocal activity of North Atlantic right whales in the New York Bight: Implications for protecting a critically endangered species in a human-dominated environment. Conservation Science and Practice. 2022;4(11):e12798.
17. New York State Department of Environmental Conservation (NYSDEC), New York

State Department of State (NYSDOS). New York Ocean Action Plan; 2015 - 2025. Albany, NY: New York State Department of Environmental Conservation; 2015. Contract No.: Report.

18. Tetra Tech, LGL. Final Comprehensive Report for New York Bight Whale Monitoring Aerial Surveys, March 2017 – February 2020 East Setauket, NY. Available at: [https://www.dec.ny.gov/docs/fish_marine_pdf/mmaeran3.pdf:](https://www.dec.ny.gov/docs/fish_marine_pdf/mmaeran3.pdf) Technical report prepared by Tetra Tech, Inc. and LGL Ecological Research Associates, Inc. Prepared for New York State Department of Environmental Conservation, Division of Marine Resources; 2020.
19. Zoidis AM, Lomac-MacNair KS, Ireland DS, Rickard ME, McKown KA, Schlesinger MD. Distribution and density of six large whale species in the New York Bight from monthly aerial surveys 2017 to 2020. Cont Shelf Res. 2021;230:104572.
20. Clark CW, Brown MW, Corkeron P. Visual and acoustic surveys for North Atlantic right whales, *Eubalaena glacialis*, in Cape Cod Bay, Massachusetts, 2001-2005: Management implications. Marine Mammal Science. 2010;26(4):837-54.
21. Mellinger DK, Stafford KM, Moore SE, Dziak RP, Matsumoto H. An overview of fixed passive acoustic observation methods for cetaceans. Oceanography. 2007;20(4):36-45.
22. Van Parijs SM, Clark CW, Sousa-Lima RS, Parks SE, Rankin S, Risch D, et al. Management and research applications of real-time and archival passive acoustic sensors over varying temporal and spatial scales. Marine Ecology Progress Series. 2009;395:21-36.
23. Fleishman E, Cholewiak D, Gillespie D, Helble T, Klinck H, Nosal E-M, et al.

Ecological inferences about marine mammals from passive acoustic data. Biological Reviews. 2023;98(5):1633-47.

24. Marques TA, Thomas L, Martin SW, Mellinger DK, Ward JA, Moretti DJ, et al. Estimating animal population density using passive acoustics. Biological Reviews. 2013;88(2):287-309.
25. Varghese HK, Lowell K, Miksis-Olds J. Global-local-comparison approach: Understanding marine mammal spatial behavior by applying spatial statistics and hypothesis testing to passive acoustic data. Frontiers in Marine Science. 2021;8:625322.
26. Davis GE, Baumgartner M, Corkeron P, Bell J, Berchok C, Bonnell J, et al. Exploring movement patterns and changing distribution of baleen whales in the western North Atlantic using a decade of passive acoustic data. Global Change Biology. 2020;26:4812-40.
27. Davis GE, Baumgartner MF, Gurnee J, Bell J, Berchok C, Bort Thornton J, et al. Longterm passive acoustic recordings track the changing distribution of North Atlantic right whales (*Eubalaena glacialis*) from 2004 to 2014. Scientific Reports. 2017;7:13460.
28. Risch D, Castellote M, Clark CW, Davis GE, Dugan PJ, Hodge LE, et al. Seasonal migrations of North Atlantic minke whales: novel insights from large-scale passive acoustic monitoring networks. Movement Ecology. 2014;2(1):24.
29. Calupca TA, Fristrup KM, Clark CW. A compact digital recording system for autonomous bioacoustic monitoring. Journal of the Acoustical Society of America. 2000;108(5):2582.
30. K. Lisa Yang Center for Conservation Bioacoustics (KLY-CCB). Raven Pro 2.0: Interactive Sound Analysis Software. Ithaca, NY: Cornell Lab of Ornithology. Available at: [http://www.birds.cornell.edu/brp/raven/RavenOverview.html;](http://www.birds.cornell.edu/brp/raven/RavenOverview.html) 2022.
31. Parks SE, Clark CW. Acoustic communication: Social sounds and the potential impacts of noise. In: Kraus SD, Rolland RM, editors. The Urban Whale: North Atlantic Right Whales at the Crossroads. Cambridge, MA: Harvard University Press; 2007. p. 310-32.
32. Parks SE, Tyack PL. Sound production by North Atlantic right whales (*Eubalaena glacialis*) in surface active groups. Journal of the Acoustical Society of America. 2005;117(5):3297-306.
33. Urazghildiiev IR, Clark CW, Krein TP, Parks SE. Detection and recognition of North Atlantic right whale contact calls in the presence of ambient noise. IEEE Journal of Oceanic Engineering. 2009;34(3):358-68.
34. Matthews LP, Parks SE. An overview of North Atlantic right whale acoustic behavior, hearing capabilities, and responses to sound. Marine Pollution Bulletin. 2021;173:113043.
35. Morano JL, Rice AN, Tielens JT, Estabrook BJ, Murray A, Roberts B, et al. Acoustically detected year-round presence of right whales in an urbanized migration corridor. Conservation Biology. 2012;26(4):698-707.
36. Salisbury DP, Clark CW, Rice AN. Right whale occurrence in the coastal waters of Virginia, U.S.A.: implications of endangered species presence in a rapidly developing energy market. Marine Mammal Science. 2016;32(2):509-19.
37. Hodge KB, Muirhead CA, Morano JL, Clark CW, Rice AN. North Atlantic right whale occurrence in two wind planning areas along the mid-Atlantic U.S. coast: Implications for management. Endangered Species Research. 2015;28:225-34.
38. McCordic JA, Root-Gutteridge H, Cusano DA, Denes SL, Parks SE. Calls of North Atlantic right whales *Eubalaena glacialis* contain information on individual identity and age class. Endangered Species Research. 2016;30:157-69.
39. Mussoline SE, Risch D, Hatch LT, Weinrich MT, Wiley DN, Thompson MA, et al.

Seasonal and diel variation in North Atlantic right whale up-calls: implications for management and conservation in the northwestern Atlantic Ocean. Endangered Species Research. 2012;17(1):17-26.

40. Parks SE, Searby A, Celerier A, Johnson MP, Nowacek DP, Tyack PL. Sound production behavior of individual North Atlantic right whales: implications for passive acoustic monitoring. Endangered Species Research. 2011;15(1):63-76.
41. Clark CW, Ellison WT, Southall BL, Hatch L, Van Parijs SM, Frankel A, et al. Acoustic masking in marine ecosystems: intuitions, analysis, and implication. Marine Ecology Progress Series. 2009;395:201-22.
42. Parks SE, Clark CW, Tyack PL. Short- and long-term changes in right whale calling behavior: The potential effects of noise on acoustic communication. Journal of the Acoustical Society of America. 2007;122(6):3725-31.
43. Dugan P, Pourhomayoun M, Shiu Y, Paradis R, Rice A, Clark C. Using high performance computing to explore large complex bioacoustic soundscapes: case study for right whale acoustics. Procedia Computer Science. 2013;20:156-62.
44. Mellinger DK, Nieukirk SL, Klinck K, Klinck H, Dziak RP, Clapham PJ, et al. Confirmation of right whales near a nineteenth-century whaling ground east of southern Greenland. Biology Letters. 2011;7(3):411-3.
45. Dunlop RA, Noad MJ, Cato DH, Stokes D. The social vocalization repertoire of east Australian migrating humpback whales (*Megaptera novaeangliae*). Journal of the Acoustical Society of America. 2007;122(5):2893-905.
46. Payne RS, McVay S. Songs of humpback whales. Science. 1971;173(3997):585-97.
47. Stimpert AK, Au WWL, Parks SE, Hurst T, Wiley DN. Common humpback whale (*Megaptera novaeangliae*) sound types for passive acoustic monitoring. Journal of the Acoustical Society of America. 2011;129(1):476-82.
48. Fournet MEH, Jacobsen L, Gabriele CM, Mellinger DK, Klinck H. More of the same:

allopatric humpback whale populations share acoustic repertoire. PeerJ. 2018;6:e5365.

49. Murray A, Rice AN, Clark CW. Extended seasonal occurrence of humpback whales in Massachusetts Bay. Journal of the Marine Biological Association of the UK. 2014;94(6):111725.
50. Vu ET, Risch D, Clark CW, Gaylord S, Hatch LT, Thompson MA, et al. Humpback whale song occurs extensively on feeding grounds in the western North Atlantic Ocean. Aquatic Biology. 2012;14(2):175-83.
51. Clark CW, Borsani JF, Notarbartolo-di-Sciara G. Vocal activity of fin whales, *Balaenoptera physalus*, in the Ligurian Sea. Marine Mammal Science. 2002;18(1):286-95.
52. McDonald MA, Hildebrand JA, Webb SC. Blue and fin whales observed on a seafloor array in the Northeast Pacific. Journal of the Acoustical Society of America. 1995;98(2):712-21.
53. Watkins WA, Tyack P, Moore KE, Bird JE. The 20-Hz signals of finback whales (*Balaenoptera physalus*). Journal of the Acoustical Society of America. 1987;82(6):1901-12.
54. Croll DA, Clark CW, Acevedo A, Tershy B, Flores S, Gedamke J, et al. Only male fin whales sing loud songs. Nature. 2002;417(6891):809.
55. Clark CW, Gagnon GJ, Frankel AS. Fin whale singing decreases with increased swimming speed. Royal Society Open Science. 2019;6(6):180525.
56. Morano JL, Salisbury DP, Rice AN, Conklin KL, Falk KL, Clark CW. Seasonal changes in fin whale song in the Western North Atlantic Ocean. Journal of the Acoustical Society of America. 2012;132(2):1207-12.
57. Mellinger DK, Clark CW. Recognizing transient low-frequency whale sounds by spectrogram correlation. Journal of the Acoustical Society of America. 2000;107(6):3518-29.
58. Dugan PJ, Klinck H, Roch MA, Helble TA. RAVEN X: High performance data mining toolbox for bioacoustic data analysis. arxiv. 2016:1610.03772.
59. Baumgartner MF, Van Parijs SM, Wenzel FW, Tremblay CJ, Esch HC, Warde AM. Low frequency vocalizations attributed to sei whales (*Balaenoptera borealis*). Journal of the Acoustical Society of America. 2008;124(2):1339-49.
60. Tremblay CJ, Parijs SMV, Cholewiak D. 50 to 30-Hz triplet and singlet down sweep vocalizations produced by sei whales (*Balaenoptera borealis*) in the western North Atlantic Ocean. Journal of the Acoustical Society of America. 2019;145(6):3351-8.
61. Mellinger DK, Clark CW. Blue whale (*Balaenoptera musculus*) sounds from the North Atlantic. Journal of the Acoustical Society of America. 2003;114(2):1108-19.
62. McDonald MA, Calambokidis J, Teranishi AM, Hildebrand JA. The acoustic calls of blue whales off California with gender data. Journal of the Acoustical Society of America. 2001;109(4):1728-35.
63. Newhall AE, Lin Y-T, Lynch JF, Baumgartner MF, Gawarkiewicz GG. Long distance passive localization of vocalizing sei whales using an acoustic normal mode approach. Journal of the Acoustical Society of America. 2012;131:1814-25.
64. Munger LM, Wiggins SM, Hildebrand JA. North Pacific right whale up-call source levels and propagation distance on the southeastern Bering Sea shelf. Journal of the Acoustical Society of America. 2011;129(6):4047-54.
65. Wiggins SM, McDonald MA, Munger LM, Moore SE, Hildebrand JA. Waveguide propagation allows range estimates for North Pacific right whales in the Bering Sea. Canadian Acoustics. 2004;32(2):146-54.
66. Wood S. Fast stable restricted maximum likelihood and marginal likelihood estimation of semiparametric generalized linear models. Journal of the Royal Statistical Society B. 2011;73(1):3-36.
67. R Core Team. R: A Language and Environment for Statistical Computing. Vienna, Austria: R Foundation for Statistical Computing, [https://www.R-project.org/;](https://www.r-project.org/) 2021.
68. Hartig F. DHARMa: Residual Diagnostics for Hierarchical (Multi-Level / Mixed) Regression Models. 0.4.6 ed2022.
69. Zuur AF, Ieno EN, Elphick CS. A protocol for data exploration to avoid common statistical problems. Methods in Ecology and Evolution. 2010;1(1):3-14.
70. Lenth RV. emmeans: Estimated Marginal Means, aka Least-Squares Means. R version

1.7.0: [https://CRAN.R-project.org/package=emmeans;](https://cran.r-project.org/package=emmeans) 2021.

71. Hatch LT, Clark CW, Van Parijs SM, Frankel AS, Ponirakis DW. Quantifying loss of acoustic communication space for right whales in and around a U.S. National Marine Sanctuary. Conservation Biology. 2012;26(5):983-94.
72. Au WWL, Pack AA, Lammers MO, Herman LM, Deakos MH, Andrews K. Acoustic properties of humpback whale songs. Journal of the Acoustical Society of America. 2006;120(2):1103-10.
73. Kowarski K, Evers C, Moors-Murphy H, Martin B, Denes SL. Singing through winter nights: Seasonal and diel occurrence of humpback whale (Megaptera novaeangliae) calls in and around the Gully MPA, offshore eastern Canada. Marine Mammal Science. 2018;34(1):169-89.
74. Weirathmueller MJ, Wilcock WSD, Soule DC. Source levels of fin whale 20 Hz pulses measured in the Northeast Pacific Ocean. Journal of the Acoustical Society of America. 2013;133(2):741-9.
75. Širović A, Hildebrand JA, Wiggins SM. Blue and fin whale call source levels and propagation range in the Southern Ocean. Journal of the Acoustical Society of America. 2007;122(2):1208-15.
76. Stimpert AK, DeRuiter SL, Falcone EA, Joseph J, Douglas AB, Moretti DJ, et al. Sound production and associated behavior of tagged fin whales (*Balaenoptera physalus*) in the Southern California Bight. Animal Biotelemetry. 2015;3(1):23.
77. Wang D, Huang W, Garcia H, Ratilal P. Vocalization source level distributions and pulse compression gains of diverse baleen whale species in the Gulf of Maine. Remote Sensing. 2016;8(11):881.
78. McDonald MA, Hildebrand JA, Mesnick S. Worldwide decline in tonal frequencies of blue whale songs. Endangered Species Research. 2009;9(1):13-21.
79. Thode AM, D'Spain GL, Kuperman WA. Matched-field processing, geoacoustic inversion, and source signature recovery of blue whale vocalizations. Journal of the Acoustical Society of America. 2000;107(3):1286-300.
80. Morfey CL. Dictionary of Acoustics. San Diego: Academic Press; 2001.
81. Dugan PJ, Ponirakis DW, Zollweg JA, Pitzrick MS, Morano JL, Warde AM, et al. SEDNA - Bioacoustic Analysis Toolbox. IEEE OCEANS 2011. 2011;2011:1-10.
82. Urick RJ. Principles of Underwater Sound, 3rd edition. Los Altos, CA: Peninsula Publishing; 1983.
83. Siderius M, Porter MB. Modeling broadband ocean acoustic transmissions with timevarying sea surfaces. Journal of the Acoustical Society of America. 2008;124(1):137-50.
84. Porter MB. Beam tracing for two- and three-dimensional problems in ocean acoustics. Journal of the Acoustical Society of America. 2019;146(3):2016-29.
85. Cholewiak D, Clark CW, Ponirakis D, Frankel A, Hatch LT, Risch D, et al.

Communicating amidst the noise: Modeling the aggregate influence of ambient and vessel noise on baleen whale communication space in a national marine sanctuary. Endangered Species Research. 2018;36:59-75.

86. Lomac-MacNair KS, Zoidis AM, Ireland DS, Rickard ME, McKown KA. Fin, humpback, and minke whale foraging events in the New York Bight as observed from aerial surveys, 2017-2020. Aquatic Mammals. 2022;48(2):142-58.
87. Kraus SD, Prescottt JH, Knowlton AR, Stone GS. Migration and calving of right whales (*Eubalaena glacialis*) in the Western North Atlantic. Reports of the International Whaling Commission. 1986;Special Issue 10:139-44.
88. Kraus SD, Rolland RM. Right whales in the urban ocean. In: Kraus SD, Rolland RM, editors. The Urban Whale: North Atlantic Right Whales at the Crossroads. Cambridge, MA: Harvard University Press; 2007. p. 1-38.
89. Hayes SA, Josephson E, Maze-Foley K, Rosel PE, Turek J, editors. U.S. Atlantic and

Gulf of Mexico Marine Mammal Stock Assessments - 2020. Woods Hole, MA: NOAA

Technical Memorandum NMFS-NE-271. U.S. Department of Commerce, National Oceanic and Atmospheric Administration, National Marine Fisheries Service Northeast Fisheries Science Center; 2021.

90. Heenehan H, Stanistreet JE, Corkeron PJ, Bouveret L, Chalifour J, Davis GE, et al. Caribbean sea soundscapes: Monitoring humpback whales, biological sounds, geological events, and anthropogenic impacts of vessel noise. Frontiers in Marine Science. 2019;6:347.
91. Charif RA, Shiu Y, Muirhead CA, Clark CW, Parks SE, Rice AN. Phenological changes in North Atlantic right whale habitat use in Massachusetts Bay. Global Change Biology. 2020;26(2):734-45.
92. Leiter SM, Stone KM, Thompson JL, Accardo CM, Wikgren BC, Zani MA, et al. North Atlantic right whale Eubalaena glacialis occurrence in offshore wind energy areas near Massachusetts and Rhode Island, USA. Endangered Species Research. 2017;34:45-59.
93. Zeh JM, Rekdahl ML, Rice AN, Clark CW, Rosenbaum HC. Detections of humpback whale (*Megaptera novaeangliae*) vocalizations on an acoustic sensor in the New York Bight. Marine Mammal Science. 2021;37(2):751-60.
94. Clark CW, Clapham PJ. Acoustic monitoring on a humpback whale (*Megaptera novaeangliae*) feeding ground shows continual singing into late spring Proceedings of the Royal Society of London B: Biological Sciences. 2004;271(1543):1051-7.
95. Stanistreet JE, Risch D, Van Parijs SM. Passive acoustic tracking of singing humpback whales (*Megaptera novaeangliae*) on a Northwest Atlantic feeding ground. PLoS ONE. 2013;8(4):e61263.
96. Kowarski K, Cerchio S, Whitehead H, Moors-Murphy H. Where, when, and why do western North Atlantic humpback whales begin to sing? Bioacoustics. 2022;31(4):450-69.
97. Stafford KM, Mellinger DK, Moore SE, Fox CG. Seasonal variability and detection range modeling of baleen whale calls in the Gulf of Alaska, 1999--2002. Journal of the Acoustical Society of America. 2007;122(6):3378-90.
98. Payne R, Webb D. Orientation by means of long range acoustic signaling in baleen whales. Annals of the New York Academy of Sciences. 1971;188(1):110-41.
99. Hayes SA, Josephson E, Maze-Foley K, Rosel PE, editors. U.S. Atlantic and Gulf of

Mexico Marine Mammal Stock Assessments - 2018. . Silver Spring, MD: NOAA Technical Memorandum NMFS-NE-258. U.S. Department of Commerce, National Oceanic and Atmospheric Administration; 2019.

100. Nieukirk SL, Mellinger DK, Dziak RP, Matsumoto H, Klinck H. Multi-year occurrence of sei whale calls in North Atlantic polar waters. Journal of the Acoustical Society of America. 2020;147(3):1842-50.
101. Normandeau Associates Inc., APEM Ltd. Digital Aerial Baseline Survey of Marine Wildlife in Support of Offshore Wind Energy. First Annual Report: 2016-2017. Albany, NY: NYSERDA; 2018.
102. Beng KC, Corlett RT. Applications of environmental DNA (eDNA) in ecology and conservation: opportunities, challenges and prospects. Biodivers Conserv. 2020;29(7):2089-121.
103. Huang SQ, Yoshitake K, Watabe S, Asakawa S. Environmental DNA study on aquatic ecosystem monitoring and management: Recent advances and prospects. J Environ Manage. 2022;323:116310.
104. Veilleux HD, Misutka MD, Glover CN. Environmental DNA and environmental RNA: Current and prospective applications for biological monitoring. Sci Total Environ. 2021;782:146891.
105. Suarez-Bregua P, Alvarez-Gonzalez M, Parsons KM, Rotllant J, Pierce GJ, Saavedra C.

Environmental DNA (eDNA) for monitoring marine mammals: Challenges and opportunities. Frontiers in Marine Science. 2022;9:987774.

106. Guillera-Arroita G, Lahoz-Monfort JJ, van Rooyen AR, Weeks AR, Tingley R. Dealing with false-positive and false-negative errors about species occurrence at multiple levels. Methods in Ecology and Evolution. 2017;9(9):1081-91.
107. Joseph C, Faiq ME, Li ZY, Chen G. Persistence and degradation dynamics of eDNA affected by environmental factors in aquatic ecosystems. Hydrobiologia. 2022;849:4119–33.
108. Frouin-Mouy H, Mouy X, Pilkington J, Küsel E, Nichol L, Doniol-Valcroze T, et al. Acoustic and visual cetacean surveys reveal year-round spatial and temporal distributions for multiple species in northern British Columbia, Canada. Scientific Reports. 2022;12(1):19272.

[Click here to access/download;Figure;Fig1.tif](https://www.editorialmanager.com/pone/download.aspx?id=35986596&guid=d1ad5fcd-9d2e-4ad1-a6b1-b4237739fd5e&scheme=1)

[Click here to access/download;Figure;Fig2.tif](https://www.editorialmanager.com/pone/download.aspx?id=35986597&guid=271ef92b-1d4a-4ea9-bc84-bda63c50a91f&scheme=1)

[Click here to access/download;Figure;Fig3.tif](https://www.editorialmanager.com/pone/download.aspx?id=35986598&guid=47b2a6b4-7a3f-4dcc-9012-208944c4f5c0&scheme=1)

[Click here to access/download;Figure;Fig4.tif](https://www.editorialmanager.com/pone/download.aspx?id=35986660&guid=f4730fc7-381e-4487-aa0d-3870f4857b7f&scheme=1)

[Click here to access/download;Figure;Fig5.tif](https://www.editorialmanager.com/pone/download.aspx?id=35986661&guid=be07f322-6af3-4a3c-9ad2-70cca659fcc0&scheme=1)

Supporting Information

[Click here to access/download](https://www.editorialmanager.com/pone/download.aspx?id=35986671&guid=fd20a3f0-91b2-4bb8-9122-193039c0a9f7&scheme=1)

[Supporting Information](https://www.editorialmanager.com/pone/download.aspx?id=35986671&guid=fd20a3f0-91b2-4bb8-9122-193039c0a9f7&scheme=1)

[EstabrookBJ_et_al_2024_PLOSONE_SupportingInforma tion.docx](https://www.editorialmanager.com/pone/download.aspx?id=35986671&guid=fd20a3f0-91b2-4bb8-9122-193039c0a9f7&scheme=1)

Supporting Information

[Click here to access/download](https://www.editorialmanager.com/pone/download.aspx?id=35986672&guid=d40e787f-16f7-4d2a-984f-aba3e096aba6&scheme=1)

[Supporting Information](https://www.editorialmanager.com/pone/download.aspx?id=35986672&guid=d40e787f-16f7-4d2a-984f-aba3e096aba6&scheme=1)

[EstabrookBJ_et_al_2024_NYBight_Baleen_Daily_20172020.txt](https://www.editorialmanager.com/pone/download.aspx?id=35986672&guid=d40e787f-16f7-4d2a-984f-aba3e096aba6&scheme=1)
