# Supplementary material for: Passive acoustic monitoring of baleen whale seasonal presence across the New York Bight
Source: PLoS One. 2025 Feb 13;20(2):e0314857. doi: 10.1371/journal.pone.0314857 (PMC11825016; doi:10.1371/journal.pone.0314857)
Supplement: Attachment — Submitted filename: PONE-D-24-13777 Rebuttal.docx [file pone.0314857.s019.docx]

**Response to Reviewers** (reviewer comments in plain text, and our replies are in italics)

**Reviewer #1**

It would be helpful if the introduction described the New York Bight within the context of the target species’ distributions, seasonal movements, and other activities. These topics are addressed in the discussion, but not the introduction. For example, the introduction does not explain hypotheses related to migration versus residence of some species.

*We appreciate the suggestion. We added text as suggested to the introduction.*

lines 54, 56. Habitat is a species-specific construct that is not synonymous with ecosystem type. Habitat contains the elements necessary for a given species to survive and reproduce. In this case, “ecosystems” likely would be the correct word.

*Thanks for suggesting this. We changed the language accordingly.*

line 60. What is a “survey effort”? Why not simply “surveys”?

*The reviewer is correct, this should be “surveys”. We changed the text.*

line 62. Why is fair in quotation marks? I’m assuming it’s a quotation from the citation, but the meaning is ambiguous. Can you explain in a manner that doesn’t require quotation marks?

*We replaced “fair” with “intermittent” to make the sentence less ambiguous.*

line 62. What is a “monitoring survey”? I know what monitoring is, and I know what a survey is, but not what a monitoring survey is.

*“Passive acoustic monitoring” refers to the method of data collection (also referred to as PAM in the manuscript). These data are the result of a PAM survey. Identical terminology has been used for several other passive acoustic monitoring surveys by other research groups.*

line 77. Unless these aerial surveys accounted for detection probability, it may not be entirely accurate to say that they identified occurrence patterns.

*We respectfully disagree, since* oc*currence pattern” can be a series of positive detections or observations in space or time, with no inference for population size, number of animals, or density.*

line 92. PAM is not necessarily synonymous with long-term or continuous data collection.

*Fair point. We clarified this in the text.*

line 102. Why these five species?

*These baleen whales were identified as “species of conservation need” by New York State, and are the likely baleen whale species to be found on the continental shelf within the New York Bight. We added this text and relevant citations to the intro.*

line 105. Neither a monitoring plan nor monitoring can mitigate effects of human activities. Data from monitoring might inform development of actions to mitigate those effects.

*We appreciate the important clarification. We modified the text accordingly. Thank you for flagging this.*

It doesn’t seem that there is a research question. That’s okay—surveys were conducted to document species presence—but I’m uncertain that reporting on presence warrants publication in the journal.

*This is particularly helpful feedback. The text was reframed to demonstrate the research question and the overarching conservation needs.*

lines 111-112. Why these two particular devices? To what extent can the data collected by these devices be integrated, and are there any trade-offs to doing so?

*We clarified this in the text at the end of the paragraph. Thank you for highlighting this.*

line 134. Why is it necessary or sensible to avoid these areas? Are vessels likely to damage the sensors, for example?

“*Areas to be avoided” is a U.S. Coast Guard designation for particular waterways for commercial vessels to avoid due to compromised navigation or potential for ship collisions (see* <https://www.noaa.gov/gc-international-section/marine-protected-areas-mpas-areas-to-be-avoided>*). We reworded this sentence to make it clearer.*

line 155. What was that second verification process?

*The second verification process was a review by a second expert human analyst to reduce the potential for single-observer bias. We clarified this in the text.*

line 177. It seems unlikely that 25% and 100% yielded identical results. Please elaborate the meaning of “the same results.”

*We reworded this sentence for clarification.*

line 190. There is a reference to daily presence here (sei whales) and at line 156 (North Atlantic right whales). What was the temporal resolution of detections of the other species?

*Thanks for catching this, and it was an accidental omission. Temporal analysis resolution was daily for all species, and we clarified this for all species.*

line 194. I’m not entirely following. Do you mean that you sought sei whale downsweeps in your data, selected six, used those downsweeps to develop a classifier, and then applied the classifier to the complete recordings?

*Yes. We reworded the sentence to make this clearer.*

line 198. Please define down-sampled.

*We reworded this sentence and added a definition.*

line 209. Why is groundtruthed in quotation marks?

*The reviewer is correct that quotation marks are not necessary, and we removed them accordingly.*

line 225. The multi-modal dispersion question should be described above in the section on sei whales.

*Good suggestion. We moved this sentence accordingly*

line 239. Is “daily detection per site” an average across sites, or was site treated as the sample unit? line 244 appears to imply the latter, but I’m not sure.

*Thanks for pointing this out. Day was the sample unit, and we clarified the phrasing to make the text in line 244 to make this clearer*

line 249. Did you set a threshold AIC difference (e.g., 2 or 4) for considering the strength of evidence for two models to be different?

*We set a threshold for the difference in AIC to 2. We added that information to this line.*

line 252. This sentence is constructed poorly. Presence is not using R.

*We reworded the sentence*

line 277. Why is full band in quotation marks?

*This sentence was modified to make it a bit clearer and rephrased such that the quotations are not necessary*

The results are quantitative, but largely descriptive. What is one to infer biologically from the extent to which the probability of detection of a given species varied among years?

*Inferring detection probability of mysticetes through PAM has a number of confounding factors not accounted for here (i.e. vocalization rate), and thus we tried to simplify the summary of our results through describing spatial and temporal patterns.*

The discussion seems a bit ad hoc. I felt that some of the material on each species could be moved to the introduction to better establish biological questions that the data potentially could answer. Similarly, questions about detection ranges largely were examined in the discussion without context earlier in the manuscript.

*Thanks for the feedback. We modified the discussion accordingly.*

**Reviewer #2:**

This is a good paper, I just have a few comments that can be addressed relatively easily. The two larger points is that I think it would be good to include more context on the limitations of PAM

*We added a bit more context on limitations of PAM, though we also included citations to references that address PAM limitations in greater detail than we have space to do here.*

and also the findings relating to the interactions between whales, you focus on each as a separate entity without any discussion of hose the presence of one group impacts the presence of others and there is nothing on overlap and interactions.

*Excellent suggestion. We added a bit of language to this point in the discussion. However, in terms of regulatory context, each of these species is managed individually without any context of possible interaction, so we didn’t want to get too much into mysticete community composition based on some of the inference limitations from our data.*

I also think you need to spend more time in the discussion talking about policy implications and future plans to protect these species.

*Helpful suggestion. We added language to the discussion on these topics.*

You don’t need to include keywords if they are in your title

*Good point. We removed “baleen whale” from the keywords.*

In what sense the ecological and economic importance needs to be unpacked.

*We added more details about this in the introduction.*

This stands in opposition to what you say above and also below – this is a very well surveyed area

*We clarified our statement earlier to reiterate that there have been relatively few cetacean surveys, compared to other systematic surveys in the New York Bight. In this section, we are not stating that cetaceans have not been studied in the New York Bight, rather there have not been many surveys conducted in this region.*

Please elaborate on the plans

*We added context about the planned offshore wind development in the region*

It would be good if you discussed the difficulty with this

*Describing density and abundance estimation and their limitations goes beyond the scope of this paper since we do not estimate density or abundance with these data.*

Please cite <https://onlinelibrary.wiley.com/journal/17487692> & <https://onlinelibrary.wiley.com/doi/abs/10.1111/brv.12969>

*We included the second one, but the first link is just the landing page for Marine Mammal Science. We are happy to include more*

or will?

*Yes, we agree. We changed “can” to “will”.*

Why 2m and 1m suspension?

*The length of the suspended unit from the seafloor varied by recording device, based on specific equipment configuration of the device.*

Please explain more here about which software you used and when visual or aural detection was used

*The software used for visual and aural detection is described in the text below this sentence in the paragraph.*

This seems like an important finding that should be in the abstract.

*We agree. We added some text in the abstract to convey this finding.*
