# Supplementary material for: Passive acoustic monitoring of baleen whale seasonal presence across the New York Bight
Source: PLoS One. 2025 Feb 13;20(2):e0314857. doi: 10.1371/journal.pone.0314857 (PMC11825016; doi:10.1371/journal.pone.0314857)
Supplement: Attachment — Submitted filename: PONE-D-24-13777 R2 Rebuttal.docx [file pone.0314857.s020.docx]

**Response to Reviewers** (reviewer comments in plain text, and our replies are in italics)

**Reviewer #3**

Line 116: “part of the limits of their geographic range extent”
Text in bold is a little confusing. Perhaps “at the edge of”?

*We agree with this suggestion, and changed that part of the sentence to “at the edge of”.*

Line 164: Typo, space needed in “eventsof”

*We corrected the mistake*

Line 166: Wouldn’t one usually say “automated detector” or “automated detection algorithm”?
Please provide a reference be provided for this algorithm.

*Yes, that was a mistake – we changed “automated detector algorithm” to “automated detector”.*

*We do not have references for these algorithms. We specified that they are custom algorithms in line 161. We also provide detector performance metrics in the supplementary materials.*

Line 192: Extra space between presence and .

*We removed the space*

Line 295: Why is the species' hearing relevant? Isn't the point of this to quantify your acoustic detection range, not to evaluate their ability to hear?

*We agree with the reviewer’s point, we removed this portion of the sentence.*

Line 301/Equation 1
I don't have Urick (84) on hand, but I don't recall seeing this formulation in it or anywhere else previously. Is H the depth of the source or the depth of the water column?
If it's depth of the source, that seems odd to me, wouldn't this give very different answers for the different source depths given in Table 2?

*Thank you for pointing this out. The formula that we from Urick (1983, page 22) is the passive sonar equation. We simplified RL from the original formula, which is derived from Noise Level (NL) – Receiving Directivity Index (DI) + Detection Threshold (DT). We modified the text to include an additional reference (not additional to this manuscript, however) from which these methods followed.*

*The Equation 1 references a transmission loss model that we used, which is plugged into the passive sonar question as TL. As stated in the paragraph, H is the depth of the source. Each species has a different estimated detection range value, and therefore, yes, different answers for different source depths. Our detection range estimates are based on the typical depths and frequencies of each target species.*

The decision to assume cylindrical spreading usually depends on bottom depth, not source depth.

*Our decision for cylindrical spreading was due to bottom depth, not source depth. We removed the misleading text from the sentence on line 301.*

I recognize that different source depths do affect transmission loss, but that also depends on wavelength, which is not part of this equation.

*However, frequency is the reciprocal of wavelength, and frequency is include in our TL model.*

The R/H part of this equation does not make sense to me. I think a reference or a more detailed explanation is needed, I'm not seeing how you get this from the two propagation references (85 & 86). If H is actually the depth of the water column, a bit more explanation would still be helpful.

This approach is based on modeling approaches used in Frankel et al. 2002, and Clark et al. 2009, and we added the citations to the text.

I am also surprised that you're finding something close to spherical spreading (17log10(R)) in such shallow water over a flat shelf. How is 17 picked instead of the usual 10log10(R) for cylindrical spreading? I could see this making sense for sensors near the shelf break with sources beyond the shelf break in deep water, but it doesn’t seem to make sense for the sensors close to the coast where detection ranges are estimated to be much less than the shelf width.

*Thank you for the comment. Based on findings from empirical playback experiment in similar coastal habitats along the northern North Atlantic continental shelf, we used 17logR in the transmission loss model. We added these two references to support the use of this value to the manuscript:*

*Estabrook BJ, Tielens JT, Rahaman A, Ponirakis DW, Clark CW, Rice AN. Dynamic spatiotemporal acoustic occurrence of North Atlantic right whales in the offshore Rhode Island and Massachusetts Wind Energy Areas. Endangered Species Research. 2022;49:115-33.*

*Hatch LT, Clark CW, Van Parijs SM, Frankel AS, Ponirakis DW. Quantifying Loss of Acoustic Communication Space for Right Whales in and around a U.S. National Marine Sanctuary. Conservation Biology. 2012;26(6):983-94.*

Figure 4. I appreciate figures with lots of info packed in, but these maps are really difficult to interpret, particularly for the blue and fin whales.

Showing 5th, 50th, and 95th noise percentiles is too much for one plot once they start overlapping. I suggest showing 50th, and putting 5th and 95th in the supplementary materials.

*The 5^th^ percentile ranges were already excluded from the figure for fin and blue whales for exactly this reason. However, we feel that the 3 percentiles for right whales, humpback whales, and sei whales are not confusing. Excluding the 95^th^ percentile ranges from the fin and blue whale plots will not eliminate the significant overlap of the 50^th^ percentiles, and we therefore do not agree with the reviewer that excluding the 95^th^ percentile will benefit the ease of interpretation for this figure.*

Site names need to be larger on all panels, or omit the names and put a dot at each site location. Scale bar text is also too small.

*We agree that the site labels and scale bar fronts were too small. To help clean up the figure, we removed the site labels completely. We feel that the center point of each circle is enough to denote the recorder locations, which are also presented in Fig 1. We increased the font size of the scale bars.*

Line 435: It would be helpful to remind the reader what kind of dBs we’re talking about (dB re 1 uPa).

*We added the reference pressure as a reminder*

Line 522: The detector is biased against distant calls, right, since it is struggling with the dispersed ones? So does that mean that your effective detection range may be less than you estimate?

*We had stated in this section that “detected downsweeps were most likely produced by sei whales near the recording sites”, which we feel makes clear that the reported sei whale detections are likely closer to the recording device than what the recording device would be capable of recording, given the detection range model.*

Line 536: More discussion of the limitations of this detection range estimation approach is needed. It is overly simplistic to use a variant of the sonar equation to estimate detection ranges when using a detector based on what appears to be some kind of computer vision or spectral cross-correlation. The sensitivity of these methods varies considerably, and does not always seem to be clearly related to SNR. Detectability may also depend on signal bandwidth and duration, which differs between species.
Here you're assuming if the RL = ambient noise level, then the call is detectable. Is that a fair assumption? SNR must be quite low in such cases.
If a blue whale is beyond the shelf break, does that signal propagate well up onto the shelf? How much do seasonal changes in oceanography have the potential to influence detectability? If right whales are vocalizing at 2m depth, are they sometimes above the thermocline, and sometimes below, and could that make a difference at these ranges?

*We added an additional source for the methods we used to estimate the detection range for each species, which describes the model in greater detail, including how signal frequency bands are incorporated in the model. We clarified in the methods and discussion that this model is based on variables whose values can vary greatly, and that this model does not contain all variables possible to more accurately estimate detection ranges. We specified that we present our range estimates as approximate, plausible ranges within which each PAM sensor may be capable of detecting each species to illustrate the potential area of coverage in this survey. We also clarified that these estimates are specific to this study only, for these specific site locations.*

*Our assumption for this model is that a target signal can be detectable if the received level exceeds the ambient noise levels, given noise and source level, as well as bathymetry. We state this assumption in the methods section with an added note that variables are variable and these are approximate estimates for the purposes of illustrating potential acoustic coverage around each site location. We acknowledge the assumptions made with this model and specify that seasonal variation is not considered.*

Line 548: What is your explanation for this discrepancy? (150km vs < 50km).

*These differences are likely due to differences in slope along the continental shelf, shadowing, depth-related attenuation, but it is also possible that more refined modeling approaches were used in more recent work.*

Supplementary docx:
The ambient noise box plots are hard to read, the text is overlapping, and the y axes seem to cover and unnecessarily broad range. There don’t seem to be values lower than about 80 or above about 130, so perhaps the range could be reduced to make the plots easier to read.

*There are values below 80 dB in the boxplots (e.g., see S9 Fig, site 09A Year-1), therefore, changing the minimum would result in cutting off the plot. Similarly, some boxplots exceed 140 dB (e.g., see S10 Fig, site 08A, Year-1), and would result in cutting out data if we reduced it as suggested. We fixed the overlapping text along the y-axis.*
